# Burden of *Streptococcus pneumoniae* and *Haemophilus influenzae* type b disease in children in the era of conjugate vaccines: global, regional, and national estimates for 2000–15

**DOI:** 10.1016/S2214-109X(18)30247-X

**Published:** 2018-06-13

**Authors:** Brian Wahl, Katherine L O'Brien, Adena Greenbaum, Anwesha Majumder, Li Liu, Yue Chu, Ivana Lukšić, Harish Nair, David A McAllister, Harry Campbell, Igor Rudan, Robert Black, Maria Deloria Knoll

**Affiliations:** a International Vaccine Access Center, Johns Hopkins Bloomberg School of Public Health, Baltimore, MD, USA; b Institute for International Programs, Johns Hopkins Bloomberg School of Public Health, Baltimore, MD, USA; c Department of International Health and Department of Population, Family and Reproductive Health, Johns Hopkins Bloomberg School of Public Health, Baltimore, MD, USA; d Department of Microbiology, Dr Andrija Štampar Institute of Public Health, Zagreb, Croatia; e Usher Institute of Population Health Sciences and Informatics, Medical School, University of Edinburgh, Edinburgh, UK; f Public Health Foundation of India, New Delhi, India; g Institute of Health and Wellbeing, University of Glasgow, Glasgow, UK

## Abstract

**Background:**

Pneumococcal conjugate vaccine (PCV) and *Haemophilus influenzae* type b (Hib) vaccine are now used in most countries. To monitor global and regional progress towards improving child health and to inform national policies for disease prevention and treatment, we prepared global, regional, and national disease burden estimates for these pathogens in children from 2000 to 2015.

**Methods:**

Using WHO and Maternal and Child Epidemiology Estimation collaboration country-specific estimates of pneumonia and meningitis mortality and pneumonia morbidity from 2000 to 2015, we applied pneumococcal and Hib cause-specific proportions to estimate pathogen-specific deaths and cases. Summary estimates of the proportion of pneumonia deaths and cases attributable to these pathogens were derived from four Hib vaccine and six PCV efficacy and effectiveness study values. The proportion of meningitis deaths due to each pathogen was derived from bacterial meningitis aetiology and adjusted pathogen-specific meningitis case–fatality data. Pneumococcal and Hib meningitis cases were inferred from modelled pathogen-specific meningitis deaths and literature-derived case–fatality estimates. Cases of pneumococcal and Hib syndromes other than pneumonia and meningitis were estimated using the ratio of pathogen-specific non-pneumonia, non-meningitis cases to pathogen-specific meningitis cases from the literature. We accounted for annual HIV infection prevalence, access to care, and vaccine use.

**Findings:**

We estimated that there were 294 000 pneumococcal deaths (uncertainty range [UR] 192 000–366 000) and 29 500 Hib deaths (18 400–40 700) in HIV-uninfected children aged 1–59 months in 2015. An additional 23 300 deaths (15 300–28 700) associated with pneumococcus and fewer than 1000 deaths associated Hib were estimated to have occurred in children infected with HIV. We estimate that pneumococcal deaths declined by 51% (7–74) and Hib deaths by 90% (78–96) from 2000 to 2015. Most children who died of pneumococcus (81%) and Hib (76%) presented with pneumonia. Less conservative assumptions result in pneumococcccal death estimates that could be as high as 515 000 deaths (302 000–609 000) in 2015. Approximately 50% of all pneumococcal deaths in 2015 occurred in four countries in Africa and Asia: India (68 700 deaths, UR 44 600–86 100), Nigeria (49 000 deaths, 32 400–59 000), the Democratic Republic of the Congo (14 500 deaths, 9300–18 700), and Pakistan (14 400 deaths, 9700–17 000]). India (15 600 deaths, 9800–21 500), Nigeria (3600 deaths, 2200–5100), China (3400 deaths, 2300–4600), and South Sudan (1000 deaths, 600–1400) had the greatest number of Hib deaths in 2015. We estimated 3·7 million episodes (UR 2·7 million–4·3 million) of severe pneumococcus and 340 000 episodes (196 000–669 000) of severe Hib globally in children in 2015.

**Interpretation:**

The widespread use of Hib vaccine and the recent introduction of PCV in countries with high child mortality is associated with reductions in Hib and pneumococcal cases and deaths. Uncertainties in the burden of pneumococcal disease are largely driven by the fraction of pneumonia deaths attributable to pneumococcus. Progress towards further reducing the global burden of Hib and pneumococcal disease burden will depend on the efforts of a few large countries in Africa and Asia.

**Funding:**

Bill & Melinda Gates Foundation.

## Introduction

*Streptococcus pneumoniae* (pneumococcus) and *Haemophilus influenzae* type b (Hib) are common causes of pneumonia, meningitis, and other serious infections in children. Pneumococcus was estimated to be responsible for 735 000 deaths in HIV-uninfected children and 14·5 million total illnesses in children in 2000.^1^ Hib was estimated to have caused 363 000 deaths in HIV-uninfected children and 8·1 million total episodes in children in the same year.^2^ These estimates represent the most recent, publicly available WHO national-level disease burden measures for these pathogens. Updated regional mortality estimates were published by WHO for 2008, estimating 541 000 pneumococcal deaths and 203 000 Hib deaths that year.^3^

**Research in context**

**Evidence before this study**

We previously conducted a systematic review of invasive disease caused by *Streptococcus pneumoniae* (pneumococcus) and *Haemophilus influenzae* type b (Hib). On the basis of this review and using data from other sources, pneumococcus was estimated to be responsible for 735 000 deaths and Hib for 363 000 deaths in HIV-uninfected children in 2000. Updated estimates later published online for the year 2008 estimated that pneumococcus accounted for 541 000 deaths and Hib accounted for 203 000 deaths that year. The Institute for Health Metrics and Evaluation estimated that pneumococcus was responsible for 393 000 pneumonia deaths and Hib for 59 000 pneumonia deaths in children younger than 5 years in 2015.

**Added value of this study**

We present updated national estimates of pneumococcal and Hib mortality and morbidity due to all syndromes associated with these pathogens (ie, pneumonia, meningitis, and other invasive diseases) from 2000 to 2015. These estimates incorporate new data from 67 studies published between 2006 and 2014 that report on pathogen-specific meningitis case fatality and the distribution of meningitis cases by aetiology. Methods for estimating pneumococcal and Hib meningitis morbidity and mortality have been updated. To account for substantial within-country disease risk disparities, we developed India subnational pathogen-specific disease burden models for all syndromes associated with these pathogens for the first time.

**Implications of all the available evidence**

To our knowledge, the results presented here are the most up-to-date estimates available of pneumococcal and Hib mortality and morbidity attributable to all syndromes. These estimates highlight global progress towards reducing the morbidity and mortality associated with these two pathogens and indicate countries and regions where intensified prevention and treatment efforts are needed. They can be used to inform national child health intervention strategies.

Several important vaccine developments have occurred since 2000. Protein-polysaccharide conjugate Hib vaccines are now included in the routine immunisation schedule of almost every country worldwide. By the end of 2015, 129 countries, including 54 low-income and middle-income countries eligible for vaccine funding support from Gavi, the Vaccine Alliance, had introduced pneumococcal conjugate vaccine (PCV); this number increased to 141 countries by September, 2017.^4^ Where Hib vaccine is used routinely with high coverage, virtual elimination of invasive Hib disease in children has occurred.^5^, ^6^, ^7^ Similarly, PCV has substantially reduced vaccine-type invasive pneumococcal disease among children.^8^, ^9^, ^10^

To measure progress towards reducing the burden of pneumococcal and Hib disease and inform the introduction and sustained use of prevention and treatment policies in the era of conjugate vaccines, we prepared annual modelled country-specific estimates of pneumococcal and Hib morbidity and mortality from 2000 to 2015 in children aged 1–59 months using country-specific data inputs whenever possible.

## Methods

### Conceptual models and inputs

**Figure 1:**
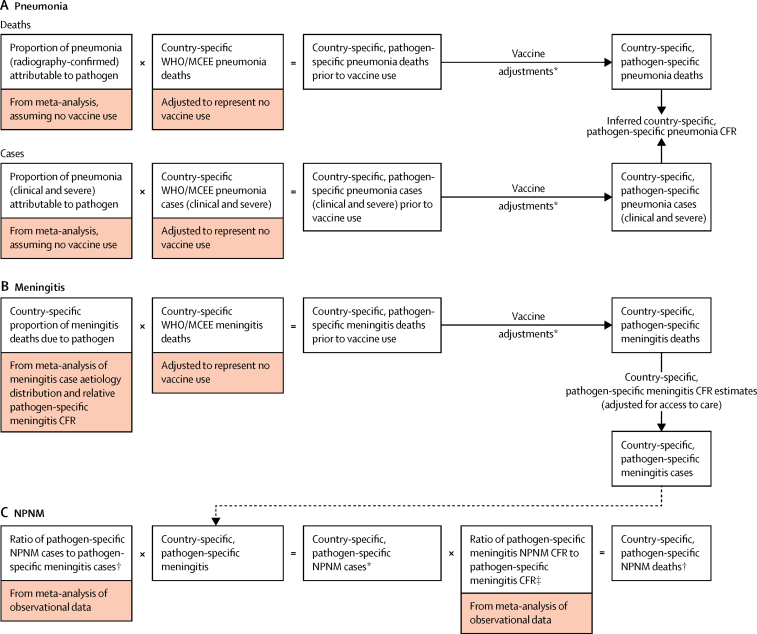
Pathogen-specific pneumonia, meningitis, and NPNM morbidity and mortality conceptual models MCEE=Maternal and Child Epidemiology Estimation. NPNM=non-pneumonia, non-meningitis. CFR=case–fatality ratio. *See Methods for description of vaccine adjustments. †Pneumococcal NPNM cases are stratified by severe and non-severe cases. ‡We assumed non-severe pneumococcal NPNM cases to have a 0% CFR; all pneumococcal NPNM deaths are assumed to be severe.

We estimated annual pneumococcal and Hib deaths, cases, mortality rates, and incidence rates for each country from 2000 to 2015 for each of the three primary syndromes associated with these pathogens: pneumonia, meningitis, and invasive non-pneumonia, non-meningitis (NPNM). We developed conceptual models for each syndrome (figure 1). For the pneumonia models, we identified PCV and Hib vaccine trials and effectiveness studies from a systematic review of the literature (see appendix pp 4–8 for search strategy) and a Cochrane review of PCV efficacy.^11^ We also updated a systematic review^12^ of pneumococcal and Hib invasive disease from 1980 to 2005 with published and unpublished data up to and including 2014 for the pathogen-specific meningitis and NPNM models. We searched six global databases (ie, PubMed, Embase, Biosis, Cochrane, Global Health, and Pascal) and five regional databases (ie, IMEMR, IMSEAR, LILACS, WHOLIS, and WPRIM) and followed the same quality assessment criteria described in the previously published literature review.^12^ We also used unpublished data from the Hib Rapid Assessment Tool and WHO Invasive Bacterial Disease surveillance network.^13^ Other model parameter values were obtained from publicly available sources or personal communication. We used state-specific input values for India whenever possible and developed subnational models to account for wide disparities across states in the country.

An independent expert committee and the Immunization and Vaccines Related Implementation Research Advisory Committee at WHO reviewed the conceptual models and inputs (appendix pp 33–34). A country consultation was undertaken by WHO to verify with country representatives the methodology and country-specific model inputs. Where evidence was unavailable or contradictory, we selected the most conservative approach that would be likely to underestimate disease. Our rationale was to minimise the likelihood that policy decisions would use overestimated burden estimates.

We report modelled pathogen-specific cases and deaths for each syndrome. Country-level results are available in the appendix (pp 36–145). Aetiology-specific deaths and cases, including country-specific estimates presented in the appendix, are modelled and not based on direct national reporting. We used world population estimates to calculate mortality and incidence rates reported as cases or deaths per 100 000 children aged 1–59 months. Population numbers were obtained from the United Nations Population Division.^14^ Global and regional results are the sum of country-specific estimates. Results for deaths and cases were rounded and reported with three significant digits or fewer; precision was not provided for fewer than 100 cases or deaths. Incidence and mortality rates were rounded to the nearest integer.

All analyses were done using Stata 14 (College Station, TX, USA). This analysis is compliant with the Guidelines for Accurate and Transparent Health Estimates Reporting (appendix p 35).^15^

### Pathogen-specific pneumonia

To estimate aetiology-specific pneumonia deaths and cases, we applied aetiological fractions to annual, country-specific all-cause pneumonia deaths and cases prepared by WHO and the Maternal and Child Epidemiology Estimation (MCEE) collaboration (figure 1A).^16^ All-cause pneumonia estimates published by the WHO/MCEE collaboration already take into account immunisation with PCV and Hib vaccine. To avoid overestimating the vaccine effect, we adjusted all-cause pneumonia deaths and cases to represent the burden of pneumonia that would have occurred had PCV and the Hib vaccine not been introduced. We assumed Hib pneumonia deaths and cases occurred only in children aged 1–23 months as described in the appendix (p 30).

We determined the proportion of all-cause pneumonia mortality and morbidity attributable to pneumococcus and Hib using the probe approach we previously developed and described.^1^, ^2^ Briefly, we meta-analysed efficacy and effectiveness results from six PCV and four Hib vaccine randomised controlled trials and case-control studies with random or systematic allocation of treatment with PCV^17^, ^18^, ^19^, ^20^, ^21^, ^22^, ^23^, ^24^, ^25^ and Hib vaccine^26^, ^27^, ^28^, ^29^, ^30^ and all-cause pneumonia case definitions.^31^, ^32^, ^33^ Because vaccine trials and studies did not assess efficacy against pneumonia mortality, we used values of efficacy and effectiveness against radiography-confirmed, primary endpoint pneumonia (ie, consolidation),^33^ with relevant adjustments, to approximate the proportion of pneumonia deaths caused by each pathogen. We used values of PCV and Hib vaccine efficacy and effectiveness against WHO-defined clinical and severe pneumonia^30^, ^32^ to estimate the contributions of each pathogen to these case definitions. Efficacy and effectiveness values were adjusted to account for incomplete efficacy against vaccine-type pneumonia. Because true efficacy values for vaccine-type pneumonia in children are not known, we used the observed efficacy against vaccine-type invasive disease for each trial as a proxy. For pneumococcus, we estimated the degree of bias this proxy measure introduced by using two sensitivity analyses described in detail in the appendix (pp 27–29): (1) vaccine-type acute otitis media (AOM) efficacy and (2) the ratio of the vaccine efficacy against vaccine-type invasive pneumococcal disease to that of vaccine-type non-bacteraemic pneumococcal pneumonia detected by a novel serotype-specific urinary antigen assay in a PCV trial^34^ done in Dutch adults older than 65 years. PCV efficacy values were also adjusted by the proportion of disease that was vaccine-type using invasive pneumococcal disease serotype distribution in the control group and by the proportion of pneumonia cases caused by Hib, because all PCV trials were done in the context of Hib vaccine.

### Pathogen-specific meningitis

The conceptual model used to estimate pathogen-specific meningitis deaths was the same as for pathogen-specific pneumonia deaths (figure 1B). To calculate pathogen-specific meningitis cases, we divided pathogen-specific meningitis deaths by country-specific and pathogen-specific meningitis case–fatality ratio (CFR) estimates.

No studies were identified that reported the distribution of meningitis deaths by pathogen. We therefore used studies reporting the distribution of meningitis cases and adjusted these estimates by the relative pathogen-specific meningitis CFR to calculate the proportion of meningitis deaths due to pneumococcus and Hib, as described in the appendix (p 31). To estimate the proportion of meningitis cases due to each pathogen, we used data from observational studies because the aetiological diagnostic yield from investigations of meningitis cases is high when done with appropriate clinical and microbiological methods. Data from 98 studies reporting aetiologically confirmed bacterial meningitis cases attributable to common causes of bacterial meningitis were combined in random effects meta-analyses stratified by region.

We estimated pathogen-specific meningitis CFR using data from the systematic literature review (53 estimates for pneumococcus and 61 estimates for Hib), stratified by child mortality setting as previously defined: low (<30 deaths per 1000 livebirths), medium (30 to <75 deaths), high (75 to <150 deaths), and very high (≥150 deaths).^1^, ^2^ Because reported CFR values reflect mortality in children who access care at a study site or health facility, we adjusted CFR values to account for the higher CFR (ie, 90% for all pathogens, as recommended by the independent expert committee) assumed for those who did not access care. There is no measure of care-seeking for children with meningitis in standardised surveys; therefore, we used the proportion of children seeking care for pneumonia symptoms from Multiple Indicator Cluster Surveys and Demographic and Health Surveys as a proxy. We assumed 100% access to care in countries with low child mortality. We assumed a linear relationship between access to care over time to estimate for years missing data. In the three countries without access-to-care data or the 24 countries with only 1 year of access-to-care data from these datasets, we developed a generalised linear model to explain the relationship between child mortality and access to care so that we could extrapolate from countries with access-to-care data.

### Pathogen-specific invasive NPNM

Pathogen-specific cases due to other invasive syndromes (eg, sepsis) were estimated using random effects meta-analyses of the ratio of pathogen-specific NPNM cases to pathogen-specific meningitis cases reported from 46 studies for pneumococcus and 28 studies for Hib studies (figure 1C). Analyses were stratified by child mortality setting and, for pneumococcus, by severe and non-severe cases. We estimated NPNM deaths by multiplying NPNM cases by NPNM CFR. The latter was derived for each pathogen by estimating the ratio of NPNM CFR to meningitis CFR by child mortality setting and applying the ratio to meningitis CFR estimates.

### HIV prevalence

Children infected with HIV who die from any cause, including pneumococcus or Hib, are accounted for in HIV death estimates.^16^ We therefore estimated pneumococcal and Hib deaths for each syndrome in HIV-infected children using annual estimates of HIV prevalence in children younger than 59 months of age provided by UNAIDS (personal communication with BW) and estimates of relative risk for invasive pneumococcal disease and invasive Hib disease in HIV-infected children from the supplemental material for previously published estimates.^1^, ^2^ We assumed children on highly active antiretroviral therapy (HAART) had the same risk of pneumococcal and Hib disease as did HIV-uninfected children. UNAIDS and the US President's Emergency Plan for AIDS Relief provided the number of children on HAART therapy for 35 countries in 2015 (personal communication with BW). We assumed no HAART coverage in other countries.

### Immunisation

We accounted for vaccine impact by subtracting the number of deaths and cases in children estimated to have been directly and indirectly protected by vaccination from the total number of pathogen-specific deaths and cases. Country-specific WHO and UNICEF Hib vaccine coverage estimates^35^ were adjusted to represent the annual proportion of children aged 1–23 months at risk of disease on the basis of the number of years after vaccine introduction and assuming no catch-up campaigns. For PCV, we stratified pneumococcal disease estimates by age on the basis of a review of its age distribution^36^ and applied vaccine coverage for each birth cohort to the corresponding estimate of pneumococcal disease for that age. Vaccine coverage estimates were combined with vaccine efficacy estimates and, in the case of PCV, regional estimates of disease serotype coverage by vaccine formulation prior to vaccine introduction^4^, ^37^ to estimate the proportion of children directly protected by vaccination. We used vaccine-type invasive pneumococcal disease efficacy and serotype coverage as proxies for vaccine-type pneumococcal pneumonia efficacy and the distribution of serotypes causing pneumococcal pneumonia, respectively.^11^, ^37^

To estimate the herd protection associated with Hib vaccine in children, we used data from vaccine studies measuring the impact of Hib vaccine on invasive Hib disease and modelled the proportion of children protected on the basis of WHO and UNICEF coverage estimates using the same methods previously reported with updated model inputs.^1^, ^2^ Although we were unable to make assumptions about herd immunity in the years following the introduction of PCV in the same way owing to too few datapoints, we assumed 95% of children were protected from vaccine-type pneumococcal disease 5 years or more after the introduction of PCV if reported vaccine coverage exceeded 65%.^38^ We also accounted for an increase in disease caused by non-vaccine serotypes in countries where the seven-valent PCV product (PCV7) was used (ie, serotype replacement). For this, we used data from a systematic review^38^ reporting the relative risk for non-vaccine-type invasive pneumococcal disease each year following the introduction of PCV7. Because serotype replacement in invasive pneumococcal disease might underestimate the extent of replacement observed for pneumococcal pneumonia, we did a sensitivity analysis assuming full replacement by non-PCV7 serotypes for pneumococcal pneumonia. Summary estimates are not yet available for higher valency PCV products and so we did not account for serotype replacement when and where these vaccines were used.

### Uncertainty

We estimated the uncertainty around our estimates using methods similar to previous analyses.^1^, ^2^ For pathogen-specific pneumonia estimates, uncertainty intervals were based on a jack-knife, leave-one-study-out approach for the upper and lower bounds of the pneumococcal and Hib pneumonia fractions. For meningitis aetiology estimates, we determined the most conservative (ie, wide) uncertainty estimates for aetiological proportions on the basis of a jack-knife leave-one-study-out analysis and reported confidence intervals from meta-analyses. Pathogen-specific NPNM morbidity and mortality uncertainty ranges were based on uncertainty from pathogen-specific meningitis models. We did not account for uncertainty in other model parameter values (ie, vaccine coverage or access to care) or all-cause mortality estimates.

### Role of the funding source

The sponsor of this study had no role in the study design, data collection, data analysis, data interpretation, writing of the report, or the decision to submit this report for publication. All authors had full access to all the data used in the study and the corresponding author had final responsibility for the decision to submit for publication.

## Results

**Figure 2:**
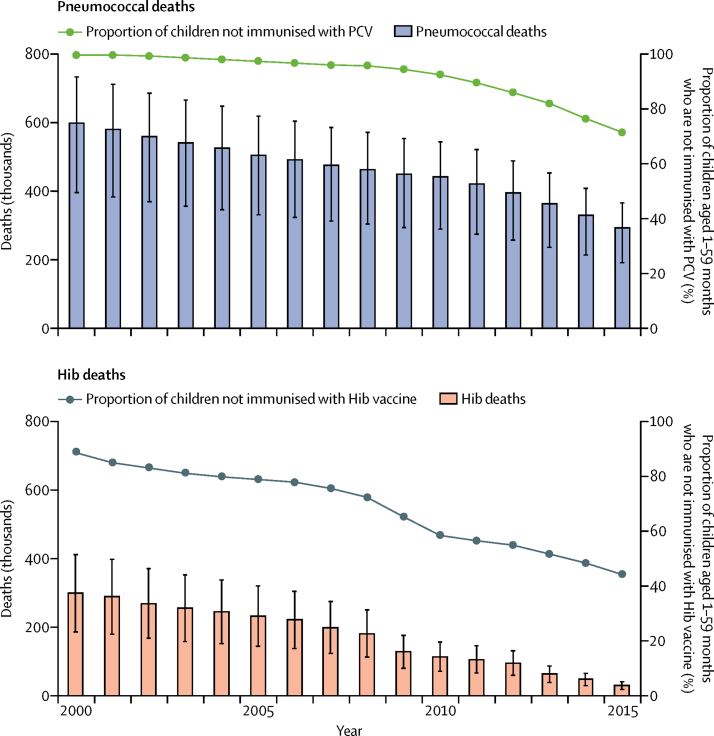
Deaths due to pneumococcus and Hib in children aged 1–59 months Pneumococcal and Hib deaths in children aged 1–59 months from 2000 to 2015 are HIV-negative deaths only. WHO/UNICEF vaccine coverage estimates have been adjusted to represent the proportion of children aged 1–59 months that received PCV and Hib vaccine. Vertical bars denote uncertainty intervals. Pneumococcus=*Streptococcus pneumoniae*. Hib=*Haemophilus influenzae* type b.

We estimated that pneumococcus was responsible for 294 000 deaths (uncertainty range [UR] 192 000–366 000) in HIV-uninfected children in 2015 (table 1). An additional 23 300 (15 300–28 700) estimated pneumococcal deaths occurred in children with HIV/AIDS in that year. Pneumococcal deaths in 2015 reflect a 51% (7–74) decline compared with 600 000 (396 000–733 000) pneumococcal deaths estimated to have occurred in HIV-uninfected children aged 1–59 months in 2000 (figure 2). Pneumococcal deaths in children infected with HIV declined by 75% from 2000 when there were approximately 95 200 deaths (61 200–114 000). Of all pneumococcal deaths in HIV-uninfected children in 2015, pneumonia accounted for 81%, meningitis accounted for 12%, and NPNM accounted for 7% (table 1). The global mortality rate for pneumococcus in 2015 was 45 deaths (29–56) per 100 000 children aged 1–59 months.

**Table 1:** Pneumococcus morbidity and mortality in 2015, by syndrome and WHO region

			**Global**	**Africa**	**Americas**	**Eastern Mediterranean**	**Europe**	**Southeast Asia**	**Western Pacific**
**Population input parameters**									
Children aged 1–59 months*			657 127 399	157 167 486	73 551 167	78 313 066	55 681 027	175 795 291	116 619 363
	Deaths†		3 260 731	1 835 315	106 231	428 791	60 540	664 465	165 389
		Pneumonia deaths‡	761 193	395 431	19 560	111 895	10 453	188 229	36 243
		Meningitis deaths‡	115 173	65 735	2756	13 801	1515	26 955	4388
**Total pneumococcal burden**									
Incidence rate			1419 (1197–1737)	1603 (1337–1997)	358 (301–441)	1261 (1066–1542)	207 (170–261)	2509 (2132–3048)	881 (745–1067)
	Severe		559 (411–658)	619 (452–739)	142 (104–169)	491 (362–576)	129 (94–154)	986 (729–1154)	347 (256–404)
Cases			9 180 000 (7 870 000–11 400 000)	2 440 000 (2 100 000–3 140 000)	259 000 (221 000–325 000)	968 000 (835 000–1 210 000)	111 000 (94 800–145 000)	4 400 000 (3 750 000–5 360 000)	1 010 000 (868 000–1 240 000)
	Severe		3 670 000 (2 700 000–4 320 000)	973 000 (710 000–1 160 000)	104 000 (76 300–124 000)	385 000 (284 000–451 000)	72 100 (52 600–85 800)	1730 000 (1 280 000–2 030 000)	404 000 (298 000–472 000)
Mortality rate			45 (29–56)	92 (60–114)	8 (5–10)	47 (31–57)	9 (6–11)	50 (32–62)	13 (9–16)
Deaths			318 000 (207 000–395 000)	166 000 (109 000–205 000)	5700 (3600–7800)	37 100 (24 300–45 300)	4900 (3100–6100)	88 500 (57 200–111 000)	15 300 (10 100–18 700)
	HIV uninfected		294 000 (192 000–366 000)	145 000 (94 500–179 000)	5700 (3500–7700)	36 700 (24 100–44 800)	4800 (3100–6100)	87 200 (56 400–110 000)	15 200 (10 000–18 600)
	HIV infected		23 300 (15 300–28 700)	21 400 (14 100–26 300)	<100	400 (200–500)	<100	1300 (800–1600)	<100
**Pneumococcal pneumonia**									
Incidence rate			1356 (1170–1612)	1504 (1298–1788)	342 (295–406)	1214 (1047–1443)	187 (161–222)	2432 (2098–2891)	831 (717–987)
	Severe		535 (401–609)	585 (438–667)	135 (101–154)	475 (356–541)	121 (91–138)	954 (715–1089)	326 (244–372)
Cases			8 910 000 (7 690 000–10 600 000)	2 360 000 (2 040 000–2 810 000)	251 000 (217 000–299 000)	951 000 (820 000–1 130 000)	104 000 (89 600–123 000)	4 280 000 (3 690 000–5 080 000)	969 000 (836 000–1 150 000)
	Severe		3 520 700 (2 640 000–4 010 000)	919 000 (689 000–1 050 000)	99 400 (74 500–113 000)	372 000 (278 000–424 000)	67 300 (50 400–76 800)	1 680 000 (1 260 000–1 910 000)	380 000 (285 000–433 000)
CFR			3% (2–3)	6% (4–6)	2% (1–2)	3% (2–3)	4% (3–4)	2% (1–2)	1% (1–1)
	Severe		7% (5–7)	15% (11–15)	5% (3–5)	8% (6–9)	6% (4–6)	4% (3–4)	3% (2–3)
Mortality rate			36 (26–38)	76 (54–79)	6 (4–6)	39 (28–41)	7 (5–7)	39 (28–40)	10 (7–10)
Deaths			257 000 (182 000–268 000)	137 000 (96 900–142 000)	4600 (3200–4800)	31 000 (22 000–32 300)	3800 (2700–4000)	69 200 (49 100–72 100)	11 600 (8200–12 100)
	HIV uninfected		238 000 (169 000–248 000)	119 000 (84 300–124 000)	4500 (3200–4700)	30 700 (21 800–32 000)	3800 (2700–4000)	68 200 (48 400–71 100)	11 600 (8200–12 000)
	HIV infected		19 300 (13 700–20 100)	17 800 (12 600–18 600)	<100	300 (200–300)	<100	1000 (700–1000)	<100
**Pneumococcal meningitis**									
Incidence rate			13 (5–26)	21 (9–45)	3 (1–7)	10 (4–21)	4 (2–8)	15 (6–31)	10 (5–15)
Cases			83 900 (36 100–169 000)	29 400 (11 800–62 300)	2300 (900–5000)	7900 (3200–16 500)	2200 (1000–4200)	26 100 (11 200–52 900)	11 400 (6300–18 000)
CFR			44%(18–93)	61%(24–100)	27%(8–70)	52%(21–100)	25%(10–50)	39%(16–79)	17%(8–30)
Mortality rate			5 (2–11)	13 (5–28)	1 (0–2)	5 (2–11)	1 (0–2)	6 (2–12)	2 (1–3)
Deaths			37 900 (15 400–79 700)	20 400 (8000–43 700)	600 (200–1600)	4200 (1600–8900)	600 (200–1100)	10 200 (4300–20 800)	1900 (1000–3500)
	HIV-uninfected		35 200 (14 300–73 800)	17 900 (7000–38 300)	600 (200–1600)	4100 (1600–8800)	550 (200–1100)	10 100 (4300–20 500)	1900 (1000–3500)
	HIV-infected		2700 (1100–5900)	2500 (1000–5400)	<100	<100	<100	200 (100–300)	<100
**Pneumococcal NPNM**									
Incidence rate			50 (22–100)	77 (31–164)	13 (5–28)	37 (15–78)	17 (7–32)	62 (27–127)	41 (22–64)
	Severe		11 (5–22)	13 (5–27)	4 (1–8)	6 (2–13)	5 (2–9)	17 (7–35)	11 (6–18)
	Non-severe		39 (17–78)	64 (26–137)	9 (4–21)	31 (12–64)	12 (5–23)	45 (19–92)	30 (16–47)
	Cases		326 000 (142 000–653 000)	122 000 (49 000–257 000)	9500 (3600–21 000)	29 000 (12 000–60 900)	9300 (4200–18 000)	110 000 (47 100–223 000)	47 500 (26 000–75 000)
	Severe		73 400 (32 400–145 000)	20 500 (8400–42 800)	2600 (1000–5700)	4900 (1900–10 400)	2500 (1100–4800)	29 900 (12 800–60 700)	13 000 (7100–20 500)
	Non-severe		253 000 (109 000–510 000)	101 000 (41 000–215 000)	6900 (2600–15 000)	24 000 (9600–50 500)	6700 (3000–13 000)	79 900 (34 300–162 000)	34 500 (19 000–54 500)
CFR§			31%(13–63)	44%(17–94)	21%(6–55)	38%(14–82)	19%(8–39)	30%(13–62)	13%(7–24)
Mortality rate			3 (1–7)	5 (2–11)	1 (0–2)	2 (1–5)	1 (0–2)	5 (2–10)	1 (1–3)
Deaths			22 700 (9400–47 200)	9000 (3600–19 100)	600 (200–1400)	1900 (700–4100)	500 (200–1000)	9100 (3800–18 400)	1700 (900–3100)
	HIV uninfected		21 500 (8800–44 500)	7900 (3100–16 800)	600 (200–1400)	1900 (700–4000)	500 (200–1000)	8900 (3800–18 100)	1700 (900–3100)
	HIV infected		1300 (500–2700)	1100 (400–2300)	<100	<100	<100	100 (100–300)	<100

Data are estimates (uncertainty range). Mortality and incidence rates are per 100 000 children. Morbidity estimates (ie, incidence rates and cases) include children regardless of HIV infection. Mortality estimates (ie, mortality rates, deaths, and CFR) do not include HIV-infected children except where specified. CFR=case–fatality ratio. Pneumococcus=*Streptococcus pneumoniae.* NPNM=non-pneumonia, non-meningitis.

* Derived from UN World Population Prospect 2015.

† Data from UN Interagency Group for Child Mortality Estimation 2015.

‡ From Maternal and Child Epidemiology Estimation/WHO collaboration estimates.^14^

§ Severe NPNM only.

By the end of 2015, 129 countries were using PCV. Pneumococcal deaths declined most sharply between 2010 and 2015, when the average annual reduction was 8%, compared with just 3% from 2000 to 2010. After 2010, 52 Gavi-eligible countries—many of them countries with the highest pneumococcal disease burden—introduced PCV in their national immunisation programmes. We estimated that PCV prevented 250 000 cumulative pneumococcal-related deaths from 2000 to 2015, with more than 95% of them prevented after 2010. A sensitivity analysis suggests that our adjustment for serotype replacement for PCV7 could overestimate averted deaths by 3100 deaths. Nevertheless, total pneumococcal deaths among HIV-uninfected children aged 1–59 months remained substantial in 2015, accounting for at least 11% (UR 7–13) of all mortality in that age group.

The updated proportion of radiography-confirmed pneumonia attributable to pneumococcus before accounting for vaccination, which included data from two additional studies,^24^, ^25^ was estimated to be 34% (UR 24–36). Our sensitivity analysis using vaccine-type efficacy of AOM found the proportion could be as high as 51% (15–87). This assumes that the efficacy of PCV against vaccine-type pneumococcal pneumonia is as low as the efficacy of PCV against vaccine-type pneumococcal AOM. Furthermore, our second sensitivity analysis showed the proportion of radiography-confirmed pneumonia caused by pneumococcus could be as high as 65% (16–86) if the ratio of PCV efficacy against vaccine-type pneumococcal pneumonia to that of vaccine-type invasive pneumococcal disease in children is similar to that observed among the Dutch elderly population.^34^ We provide country-specific pneumococcal pneumonia mortality estimates using these sensitivity parameters in the appendix (pp 146–174).

**Figure 3:**
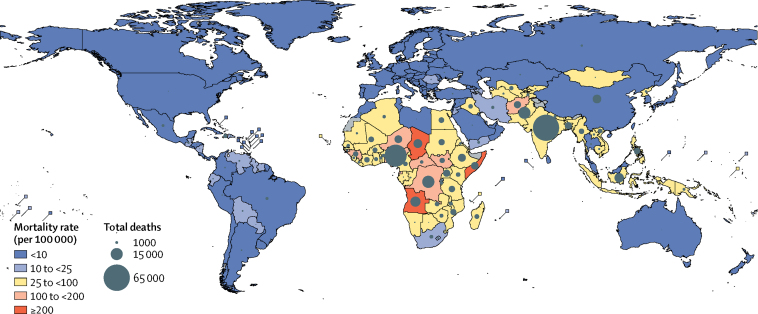
Country-specific mortality rates and deaths attributable to pneumococcus in 2015 Mortality rates and deaths in children aged 1–59 months are HIV-negative deaths only. Mortality rates are deaths per 100 000 children aged 1–59 months. Pneumococcus=*Streptococcus pneumoniae*.

India (68 700 deaths, UR 44 600–86 100), Nigeria (49 000 deaths, 32 400–59 000), the Democratic Republic of the Congo (14 500 deaths, 9300–18 700), and Pakistan (14 400 deaths, 9700–17 000) had the most and accounted for half of all pneumococcal deaths in HIV-uninfected children in 2015 (figure 3), but only 29% of the global population aged 1–59 months. Among low-income and middle-income countries, Rwanda (93%), Peru (90%), El Salvador (89%), and Brazil (88%) had the greatest relative reductions in pneumococcal deaths from 2000 to 2015 (appendix pp 36–71). Rwanda and Peru introduced PCV in 2009 and El Salvador and Brazil introduced PCV in 2010. Chad (296, UR 193–367), Somalia (294, 184–386), and Angola (216, 148–248) had the highest pneumococcal mortality rates per 100 000 children aged 1–59 months.

We estimated 8·9 million cases (UR 7·7 million–10·6 million) of clinical pneumococcal pneumonia, irrespective of HIV status, in 2015—a reduction of 37% from 14·2 million cases (12·3 million–16·9 million) in 2000. We also estimated 3·5 million cases (2·6 million–4·0 million) of severe pneumococcal pneumonia in 2015, a reduction from 5·6 million cases (4·2 million–6·4 million) in 2000. We also estimated 83 900 cases (36 100–169 000) of pneumococcal meningitis in 2015 in children regardless of HIV status, providing a global incidence rate of 13 cases (5–26) per 100 000 children aged 1–59 months. Pneumococcal meningitis cases and deaths estimated for 2000 using our updated models were very similar to the previously published estimates.^1^ Pneumococcal NPNM contributed 326 000 cases (142 000–653 000) in 2015, of which 22% were severe (table 1).

With regard to Hib, we estimated that there were 29 500 (UR 18 400–40 700) Hib deaths in HIV-uninfected children aged 1–59 months in 2015, all of which we estimated to have occurred in children aged 1–23 months. Hib was estimated to contribute to fewer than 1000 additional deaths in children infected with HIV (table 2). Of Hib deaths in HIV-uninfected children in 2015, 76% were due to pneumonia, 24% to meningitis, and less than 1% to NPNM (table 2). Hib deaths declined by 90% (78–96) from 2000, when there were an estimated 299 000 deaths (186 000–412 000) in HIV-uninfected children (figure 2). The substantial decrease in Hib mortality corresponded with an increase in the number of countries using Hib vaccine between 2000 (n=60) and 2015 (n=192). We estimated that Hib vaccine prevented approximately 1·2 million total deaths from 2000 to 2015, not including the Hib deaths prevented in HIV-infected children.

**Table 2:** Hib morbidity and mortality in 2015, by syndrome and WHO region

			**Global**	**Africa**	**Americas**	**Eastern Mediterranean**	**Europe**	**Southeast Asia**	**Western Pacific**
**Population parameters**									
Children aged 1–59 months*			657 127 399	157 167 486	73 551 167	78 313 066	55 681 027	175 795 291	116 619 363
	Deaths†		3 260 731	1835 315	106 231	428 791	60 540	664 465	165 389
		Pneumonia deaths‡	761 193	395 431	19 560	111 895	10 453	188 229	36 243
		Meningitis deaths‡	115 173	65 735	2756	13 801	1515	26 955	4388
**Total Hib burden**									
Incidence rate			148 (132–242)	75 (67–122)	8 (7–12)	41 (37–67)	58 (51–95)	238 (212–388)	317 (283–518)
	Severe		52 (30–102)	16 (9–31)	2 (1–3)	8 (5–16)	20 (12–40)	121 (70–240)	71 (41–138)
Cases			975 000 (870 000–1 590 000)	117 000 (105 000–192 000)	5500 (4900–9100)	32 000 (28 800–52 200)	32 100 (28 400–52 900)	418 000 (372 000–682 000)	370 000 (331 000–605 000)
	Severe		340 000 (196 000–669 000)	24 800 (13 900–48 600)	1200 (700–2400)	6500 (3700–12 900)	11 300 (6500–22 400)	213 000 (124 000–422 000)	82 800 (47 900–161 000)
Mortality rate			4 (3–6)	5 (3–7)	0 (0–0)	1 (1–2)	1 (0–1)	9 (6–13)	3 (2–4)
Deaths			29 800 (18 600–41 100)	8000 (4900–11 000)	200 (100–200)	1000 (600–1500)	300 (200–500)	16 500 (10 300–22 700)	3800 (2500–5200)
	HIV uninfected		29 500 (18 400–40 700)	7700 (4700–10 700)	200 (100–200)	1000 (1000–1500)	300 (200–500)	16 500 (10 300–22 600)	3800 (2500–5200)
	HIV infected		300 (200–400)	200 (100–300)	<100	<100	<100	<100	<100
**Hib pneumonia**									
Incidence rate			142 (130–232)	72 (66–118)	7 (7–12)	40 (37–65)	54 (50–89)	228 (208–372)	302 (276–494)
	Severe		45 (27–92)	13 (8–27)	1 (1–3)	8 (4–15)	17 (10–34)	111 (67–224)	56 (34–114)
Cases			934 000 (852 000–1 530 000)	114 000 (100 000–186 000)	5300 (4900–8700)	31 000 (29 000–51 200)	30 000 (28 000–49 000)	400 000 (366 000–654 000)	353 000 (320 000–576 000)
	Severe		298 000 (179 000–602 000)	21 000 (13 000–42 500)	1000 (600–2000)	5900 (3500–11 900)	9400 (5600–19 000)	195 000 (117 000–395 000)	65 700 (39 000–133 000)
CFR			2% (2–3)	5% (4–7)	2% (2–3)	2% (2–3)	1% (1–1)	3% (2–4)	1% (1–1)
	Severe		8% (5–10)	28% (20–37)	12% (9–16)	13% (9–17)	3% (2–3)	6% (4–8)	5% (3–6)
Mortality rate			3 (2–4)	4 (3–5)	0 (0–0)	1 (1–1)	0 (0–1)	7 (5–9)	3 (2–4)
Deaths			22 600 (15 900–29 700)	6000 (4200–7900)	100 (100–200)	800 (500–1000)	200 (200–300)	12 300 (8700–16 200)	3100 (2200–4100)
	HIV uninfected		22 400 (15 700–29 400)	5800 (4100–7600)	100 (100–200)	800 (500–1000)	200 (200–300)	12 300 (8700–16 200)	3100 (2200–4100)
	HIV infected		200 (100–300)	200 (100–200)	<100	<100	<100	<100	<100
**Hib meningitis**									
Incidence rate			5 (2–8)	2 (1–3)	0 (0–0)	1 (0–1)	3 (1–5)	8 (3–12)	11 (6–18)
Cases			31 400 (13 400–50 800)	3200 (1100–5200)	200 (100–300)	500 (100–800)	1400 (600–2600)	13 200 (5100–20 500)	12 900 (6400–21 500)
CFR			19%(7–29)	61%(20–98)	30%(7–51)	54%(16–89)	5%(2–9)	32%(12–49)	5%(2–8)
Mortality rate			1 (0–2)	1 (0–2)	0 (0–0)	0 (0–1)	0 (0–0)	2 (1–4)	1 (0–1)
Deaths			7200 (2700–11 300)	2000 (600–3100)	<100	300 (100–400)	<100	4200 (1600–6500)	700 (300–1100)
	HIV uninfected		7100 (2700–11 200)	1900 (600–3100)	<100	300 (100–400)	<100	4200 (1600–6400)	700 (300–1100)
	HIV infected		<100	<100	<100	<100	<100	<100	<100
**Hib NPNM**									
Incidence rate^§^			2 (1–2)	0 (0–1)	0 (0–0)	0 (0–0)	1 (0–2)	3 (1–4)	4 (2–6)
Cases			10 000 (4300–16 200)	600 (200–1000)	<100	100 (0–200)	500 (200–900)	4400 (1700–6800)	4300 (2200–7300)
CFR			0% (0–1)	1% (1–2)	0% (0–2)	1% (0–2)	0% (0–0)	1% (0–1)	0% (0–0)
Mortality rate			0 (0–0)	0 (0–0)	0 (0–0)	0 (0–0)	0 (0–0)	0 (0–0)	0 (0–0)
Deaths			<100	<100	<100	<100	<100	<100	<100
	HIV uninfected		<100	<100	<100	<100	<100	<100	<100
	HIV infected		<100	<100	<100	<100	<100	<100	<100

Data are estimates (uncertainty range). Mortality and incidence rates are per 100 000 children. Morbidity estimates (ie, incidence rates and cases) include children regardless of HIV infection. Mortality estimates (ie, mortality rates, deaths, and CFR) include only HIV-infected children unless specified. CFR=case–fatality ratio. NPNM=non-pneumonia, non-meningitis. Hib=*Haemophilus influenzae* type b.

* Derived from UN World Population Prospect 2015.

† Data from UN Interagency Group for Child Mortality Estimation 2015.

‡ From Maternal Child Epidemiology Estimation/WHO collaboration estimates.^14^

**Figure 4:**
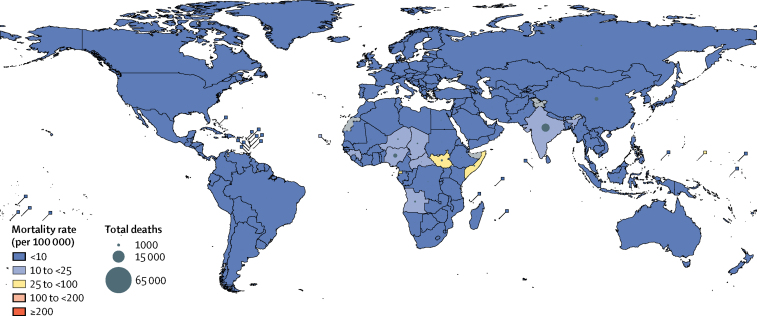
Country-specific mortality rates and deaths attributable to Hib in 2015 Mortality rates and deaths in children aged 1–59 months are HIV-negative deaths only. Mortality rates are deaths per 100 000 children aged 1–59 months. Hib=*Haemophilus influenzae* type b.

Approximately 82% of all Hib deaths in HIV-uninfected children in 2015 (24 200 deaths, UR 15 000–33 400) occurred in the Africa and southeast Asia regions (figure 4), which together accounted for 51% of the child population in that year. The greatest reductions in Hib deaths from 2000 to 2015 were observed in the eastern Mediterranean (97%) and in the Americas (96%; appendix pp 72–107). The countries with the greatest number of total Hib deaths (excluding those in HIV-infected children) in 2015 were India (15 600 deaths, 9800–21 500), Nigeria (3600 deaths, 2200–5100), China (3400 deaths, 2300–4600), and South Sudan (1000 deaths, 600–1400). The highest estimated Hib mortality rates per 100 000 children aged 1–59 months in 2015 were in South Sudan (54, UR 33–75), Equatorial Guinea (43, 28–58), and Somalia (26, 14–37) (figure 4).

There were 0·9 million estimated cases (UR 0·9 million–1·5 million) of clinical Hib pneumonia in 2015, irrespective of HIV status. Of these, 298 000 cases (179 000–602 000) were estimated to also be severe pneumonia. Assuming all Hib pneumonia deaths were among severe cases, the global inferred CFR was 8% (5–10) in 2015 for children not infected with HIV. We estimated 31 400 cases (13 400–50 800) of Hib meningitis in children in 2015, including all children regardless of HIV status, corresponding to a global incidence of 5 cases (2–8) of Hib meningitis per 100 000 children aged 1–59 months. The global Hib meningitis CFR, among HIV-uninfected children only, fell by almost half between 2000 and 2015, from 44% (18–68) in 2000 to 23% (9–36) in 2015. We also estimated 10 000 severe cases (4300–16 200) of Hib NPNM in 2015.

## Discussion

To our knowledge, we present the first comprehensive (ie, pneumonia, meningitis, and NPNM) country-level estimates of pneumococcal and Hib disease burden since the estimates^1^, ^2^ for the year 2000 published in 2009, as well as the first comprehensive regional and global burden estimates for these pathogens since they were updated for 2008.^3^ We estimated that pneumococcal and Hib deaths in children declined substantially in the era of conjugate vaccines.

Most pneumococcal and Hib deaths in 2015 were limited to a few countries in Africa and Asia. We estimated that four countries (India, Nigeria, China, and South Sudan) had more than 1000 Hib deaths in 2015. India and Nigeria introduced the Hib vaccine in 2011 and 2012, respectively; however, estimated national coverage in both countries was relatively low (ie, <50%) thereafter. South Sudan introduced the Hib vaccine in 2014. Hib deaths in these countries will probably decline as Hib vaccine coverage increases. China remained one of three countries that had not introduced Hib vaccine by 2015, along with Thailand and Russia. Likewise, half of global pneumococcal deaths in 2015 were limited to only four countries in Africa and Asia (India, Nigeria, the Democratic Republic of the Congo, and Pakistan). Pakistan and the Democratic Republic of the Congo introduced PCV in 2012. Nigeria began routine immunisation with PCV in 2014 and India started routine use in three states in 2017. Progress towards prevention of pneumococcal deaths in these countries will accelerate the global reduction in pneumococcal deaths.

The syndromic distribution of pneumococcal and Hib deaths was dominated by pneumonia, underscoring that disease burden estimates for these pathogens are highly sensitive to changes in all-cause pneumonia deaths, which are an independent input value in our pathogen-specific models. Given the low sensitivity of diagnostic tests for determining bacterial causes of pneumonia, we continued to use the probe approach to determine the fraction of pneumonia cases caused by pneumococcus and Hib.^1^, ^2^ Our updated estimate of radiography-confirmed pneumonia cases attributable to pneumococcus is similar to values estimated in previous models. Using this value as a proxy for the proportion of pneumonia deaths attributable to each pathogen inherently implies that the pneumonia CFR for radiography-confirmed pneumococcal pneumonia is equivalent to the CFR for radiography-confirmed Hib pneumonia, and that they are equal to the CFR for other causes of radiography-confirmed pneumonia. Globally, most (81%) pneumonia deaths are estimated to occur in the community,^39^ presumably with poor access to effective antibiotics. In such settings, the CFR of bacterial pneumonia is likely to be substantially higher than for non-bacterial pneumonia, revealing that our proxy method contributes to underestimating pneumococcal and Hib pneumonia deaths in settings of low access to care—the same settings that account for the greatest burden of pneumonia deaths.

We used vaccine-type invasive pneumococcal disease efficacy as a proxy for vaccine-type pneumococcal pneumonia efficacy when determining the proportion of pneumonia deaths caused by pneumococcus in the probe approach. This choice probably leads to an underestimate of the contribution of pneumococcus to pneumonia deaths and so we considered several alternatives. Based on the sensitivity analysis using vaccine-type AOM efficacy as the proxy value for vaccine-type pneumococcal pneumonia efficacy, pneumococcal pneumonia deaths could be as much as 51% higher than estimated by the base-case model. However, these results should be approached with caution as the efficacy against vaccine-type AOM is not likely to reflect the efficacy against vaccine-type pneumococcal pneumonia. Using vaccine-type non-bacteraemic pneumococcal pneumonia efficacy in an elderly Dutch population^34^ as the relative proxy, the estimate of pneumococcal pneumonia deaths in children is twice that estimated by our base-case model. This method assumes the relative vaccine efficacy against bacteraemic and non-bacteraemic pneumococcal pneumonia in elderly people reflects the same ratio in children. However, there is little evidence to support this assertion. Furthermore, if the urine antigen test used to identify the non-bacteraemic pneumococcal pneumonia cases in the Dutch trial also detects antigenuria on the basis of vaccine-type pneumococcal nasopharyngeal colonisation, the observed efficacy value in that trial could reflect a contribution of vaccine efficacy against nasopharyngeal colonisation with vaccine-type pneumococcus, which would probably be lower than the true vaccine-type pneumococcal pneumonia efficacy estimate.^34^ The limitations associated with these sensitivity analyses contributed to our decision to use vaccine-type invasive pneumococcal disease efficacy in the base-case model.

Until highly specific diagnostic approaches for vaccine-type non-bacteraemic pneumococcal pneumonia in children are available, the validity of these sensitivity analyses will remain unclear. Nevertheless, they do emphasise that our base-case estimates are very likely to be underestimates, possibly by as much as 100%. Our decision around which base-case analysis to use was also informed by consultation with external experts and a review of PCV impact studies with clinical pneumonia outcomes in several high-income and middle-income settings (appendix pp 22–23). Where parameter selection could not be grounded in clear evidence, we erred on the side of being conservative in our approach (ie, making decisions that would lead to an underestimate of disease burden). As evidence continues to accrue, we will revisit the assumptions in the base-case model. We have provided the results of the country-level pneumococcal pneumonia estimates using the sensitivity analyses in the appendix (pp 146–74) to provide full access to the range of possible disease burden estimates for each country. Recently reported pneumococcal pneumonia disease burden estimates for 2015 from the Institute for Health Metrics and Evaluation (IHME) Global Burden of Disease collaboration use the vaccine-type pneumococcal pneumonia efficacy value in elderly people in their base-case model.^40^ Consequently, pneumococcal pneumonia deaths estimated by the IHME—approximately 393 000 in children aged 1–59 months—are greater in number than those presented here.

We prepared annual estimates of pneumococcal and Hib disease burden from 2000 to 2015, allowing us to make comparisons with previously published estimates. We previously estimated 735 000 pneumococcal deaths^1^ and 363 000 Hib deaths in 2000 in HIV-uninfected children.^2^ By comparison, we estimated 600 000 pneumococcal deaths and 299 000 Hib deaths in 2000 excluding HIV-infected children, using updated methods and new data. We observed similar differences comparing the current mortality estimates with those for the year 2008,^3^ which were generated using the original model with updated input values. Pathogen-specific URs for disease burden estimates for the years 2000 and 2008 from current and previously published methods overlap substantially. In addition, our validation for pneumococcal disease (appendix p 177) suggests that our pneumococcal disease burden estimates are likely to be underestimated but within the confidence bounds. We were unable to validate the plausibility of our Hib cases and deaths because we found no other study that independently quantified the burden accounting for the poor diagnostic sensitivity.

All-cause child mortality has been decreasing in almost all low-income and middle-income countries as a result of vaccine and non-vaccine interventions.^41^, ^42^ As this trend continues, morbidity associated with childhood illnesses, which is less affected by non-vaccine interventions, will play an increasingly important role in programme planning and policy making. We prepared country-specific estimates of severe and non-severe disease associated with pneumococcus and Hib, which can be used for these purposes.

The availability of all-cause meningitis death estimates allowed us to shift from an incidence-based model to a proportion-based model. The motivation for this shift was to ensure that any pathogen-specific meningitis mortality estimates were inherently bounded by the total meningitis mortality. The sum of pathogen-specific death estimates derived using incidence data could theoretically exceed the total number of meningitis deaths. In addition, if the sensitivity of meningitis diagnostic tests is reasonably similar for each pathogen, using a relative distribution of pathogens (ie, our proportion-based method) could be less likely to underestimate the pathogen-specific burden of meningitis. We were unable to account for outbreaks of pneumococcal meningitis, which could lead to an underestimate of meningitis disease burden, particularly countries in sub-Saharan Africa. To provide context to aetiology-specific meningitis death and case estimates derived using the proportion-based method, we also estimated pneumococcal and Hib meningitis mortality and morbidity using the incidence-based model (appendix pp 175–76).

Our pathogen-specific meningitis and NPNM models rely on data from a systematic literature review of studies published between 1990 and 2014. For the proportion-based approach, we used studies that were conducted prior to the introduction of any conjugate vaccine. As most countries (n=192) were using Hib vaccine at the end of 2015, we anticipated very few additional studies would contribute data. Methods that do not rely on data from the era prior to the introduction of Hib vaccine will be needed to assess the burden of pneumococcal and Hib disease in the future.

Our model may have overestimated the impact of vaccination in children, particularly for PCV, due to the limited availability of data. We use country-specific vaccine coverage data from WHO and UNICEF. Reported coverage data have been observed to underestimate and overestimate coverage. The latter is more likely to occur when estimates are based on administrative data.^43^ As a result, our model could have overestimated the impact of vaccination. National vaccine coverage estimates can also mask regional and local coverage disparities. If those at risk of disease, independently of whether they received vaccine, are differentially reached by vaccination programmes, our model will overestimate the effect of immunisation. We also assumed serotype replacement for pneumococcal pneumonia to occur with the same frequency as invasive pneumococcal disease. We were also unable to account for serotype replacement in countries where PCV10 or PCV13 were routinely used. We probably overestimated vaccine impact in both cases. However, the extent to which vaccine impact is overestimated in any of these cases remains unknown. It is important to note that PCV and Hib vaccine probably provide protection to other age groups not included in this analysis.

These estimates should not be interpreted as a primary source of vaccine impact data and cannot be used to validate vaccine impact measured directly in the field as our models rely on assumptions of vaccine efficacy and effectiveness to estimate vaccine impact. However, our results can be used to emphasise the efforts of several organisations working to expand access to these vaccines. Our modelled estimates suggest an accelerated reduction in pneumococcal burden in the years after 2010 when the Advanced Market Commitment for PCV, a financing mechanism for the development of new vaccines, was launched by Gavi.^44^ The availability of funding for PCV in low-income and middle-income countries with the highest disease burden accelerated its adoption and therefore the reduction in pneumococcal deaths.

Pathogen-specific pneumonia and meningitis deaths are constrained by the all-cause pneumonia and meningitis death estimates produced by the WHO/MCEE collaboration.^16^ Given that few deaths occur in countries with adequate vital registration systems, these estimates are based largely on verbal autopsy data. The potential for misclassification and other challenges associated with verbal autopsy studies, particularly for non-specific syndromes such as pneumonia, have been described extensively.^45^, ^46^ Given the dependence of the present pathogen-specific mortality estimates on all-cause pneumonia and meningitis deaths, advances in improving the confidence of cause of death estimates in high child-mortality settings would also positively affect pathogen-specific estimates.

Although we accounted for the increased risk of pneumococcal and Hib disease in children infected with HIV, we only accounted for the use of HAART among HIV-infected children in 35 countries in 2015. Studies have shown a marked reduction in pneumococcal disease in children younger than 18 years after the widespread use of HAART.^47^ Failing to account for HAART use in all countries probably leads to an overestimate of the pneumococcal and Hib disease burden in HIV-infected children. In addition, we were unable to account for HIV-exposed but uninfected children who are at increased risk of morbidity and mortality from invasive pneumococcal disease.^48^ Other than HIV, we were unable to account for other underlying risk factors for invasive bacterial diseases, including malaria infection^49^ and sickle cell anaemia.^50^ We assumed 0% Hib vaccine coverage for China: the vaccine is not used in the national immunisation programme; however, relatively high immunisation coverage through the private sector has been reported in some cities.^51^, ^52^ We could not locate a reliable source of Hib vaccine coverage for the country. Although this would lead to an overestimate of disease, we believe those who would have access to the vaccine through the private sector would be at lower overall risk of disease compared with those without access to the vaccine. Despite these limitations, we believe the net effect of our estimates remains an underestimate of disease burden.

The burden of Hib disease is largely limited to a handful of countries that have not or only recently initiated routine use of Hib vaccine. Pneumococcal disease is beginning to decline substantially around the world, but still causes a large number of deaths and cases. The remaining burden of disease associated with these two pathogens warrants sustained and intensified efforts, including the introduction of vaccine in countries that have not yet introduced the vaccine and dedicated efforts to enhance coverage, particularly among children whose socioeconomic situation puts them at increased risk of disease.
