# Supplementary material for: Burden of Streptococcus pneumoniae and Haemophilus influenzae type b disease in children in the era of conjugate vaccines: global, regional, and national estimates for 2000–15
Source: Lancet Glob Health. 2018 Jun 13;6(7):e744–57. doi: 10.1016/S2214-109X(18)30247-X (PMC6005122; doi:10.1016/S2214-109X(18)30247-X)
Supplement: Supplementary appendix [file mmc1.pdf]

THE LANCET
Global Health

Supplementary appendix

This appendix formed part of the original submission and has been peer reviewed. We post it as supplied by the authors.

Supplement to: Wahl B, O'Brien KL, Greenbaum A, et al. Burden of *Streptococcus pneumoniae* and *Haemophilus influenzae* type b disease in children in the era of conjugate vaccines: global, regional, and national estimates for 2000–15. *Lancet Glob Health* 2018; **6:** e744–57.

Webappendix: Global, regional, and national burden of *Streptococcus pneumoniae* and *Haemophilus influenzae* type b in children in the era of conjugate vaccines: estimates for 2000-2015

Authors: Brian Wahl, MPH (1), Katherine L O'Brien, MD (1), Adena Greenbaum, MD (1), Anwesha Majumder, MHS (1), Li Liu, PhD (2, 3), Yue Chu, MSPH (2), Ivana Lukšić, MD (4), Harish Nair, PhD (5,6), David A McAllister, MD (5), Harry Campbell, MD (5), Igor Rudan, MD (5), Robert Black, MD (2), Maria Deloria Knoll, PhD (1)

- International Vaccine Access Center (IVAC), Department of International Health, Johns Hopkins Bloomberg School of Public Health, 415 N Washington Street, Floor 5, Baltimore, MD 21231, USA
- Institute for International Programs, Department of International Health, Johns Hopkins Bloomberg School of Public Health, 615 N Wolfe St, Baltimore, MD 21205, USA
- Department of Population, Family and Reproductive Health, Johns Hopkins Bloomberg School of Public Health, 615 N Wolfe St, Baltimore, MD 21205, USA
- Department of Microbiology, Dr. Andrija Štampar Institute of Public Health, Mirogojska 16, 10000 Zagreb, Croatia
- Usher Institute of Population Health Sciences and Informatics, Medical School, The University of Edinburgh, Teviot Place, Edinburgh EH8 9AG, Scotland, UK
- Public Health Foundation of India, New Delhi, India

Correspondence: Brian Wahl; International Vaccine Access Center (IVAC), Department of International Health, Johns Hopkins Bloomberg School of Public Health, 415 N Washington Street, Floor 5, Baltimore, MD 21231, USA; bwahl@jhu.edu

Contents

- Webappendix 1: Literature review
- Webappendix 2: Details of disease burden data inputs
- Webappendix 3: Analysis of pneumonia impact studies to inform proportion of pneumonia morbidity and mortality attributable to *Streptococcus pneumoniae* and *Haemophilus influenzae* type b
- Webappendix 4: Proportion of pneumonia morbidity and mortality attributable to *Streptococcus pneumoniae* and *Haemophilus influenzae* type b
- Webappendix 5: Details of sensitivity analysis on proportion of pneumonia deaths attributable to *Streptococcus pneumoniae*
- Webappendix 6: Methods used for stratifying pneumonia and meningitis deaths by age (i.e., 1-23 months and 24-59 months) for estimating the burden of Hib disease
- Webappendix 7: Methods used to estimate the proportion of meningitis deaths attributable to each pathogen.
- Webappendix 8: Equation used to account for increased risk of Hib and pneumococcal disease in children infected with HIV
- Webappendix 9: External review members
- Webappendix 10: GATHER checklist
- Webappendix 11: Country-level *Streptococcus pneumoniae* and *Haemophilus influenzae* type b disease burden estimates by syndrome for 2000-2015 in children 1-59 months
- Webappendix 12: Comparison of *Streptococcus pneumoniae* pneumonia mortality sensitivity analyses for 2000-2015 in children 1-59 months
- Webappendix 13: Comparison of meningitis mortality and morbidity estimates, proportional approach versus incidence approach
- Webappendix 14: Plausibility check for pneumococcal morbidity and mortality

List of tables and figures

1. Systematic review PRISMA flow diagram of studies
2. Studies contributing to estimates of pneumonia morbidity and mortality attributable fractions
3. Studies contributing to estimates of pathogen-specific meningitis case fatality ratio estimates stratified by child mortality setting
4. Studies contributing to the distribution of meningitis cases by bacterial pathogen, stratified by geographic region
5. Other indicators used for modeling Hib and pneumococcal disease burden
6. Flow Diagram of PCV Impact studies for Use in Determining the Proportion of Pneumonia Deaths Attributable to Pneumococcus
7. Proportion of radiography-confirmed pneumonia attributable to pneumococcus
8. Proportion of severe pneumonia attributable to pneumococcus
9. Proportion of clinical pneumonia attributable to pneumococcus
10. Proportion of radiography-confirmed pneumonia attributable to Hib
11. Proportion of severe pneumonia attributable to Hib
12. Proportion of clinical pneumonia attributable to Hib

13. Estimates of radiography-confirmed pneumonia attributable to pneumococcus in the absence of vaccination using different adjustments to account for incomplete efficacy against VT pneumococcal pneumonia in the absence of vaccination with PCV or Hib vaccine
14. Impact of PCV on all-cause pneumonia hospitalizations stratified by age-group
15. Pneumococcal and Hib mortality estimates by syndrome, country, and year
16. Comparison of pneumococcal pneumonia mortality estimates with sensitivity analysis on vaccine-type pneumococcal pneumonia efficacy
17. Pneumococcal meningitis cases by approach for 2000-2015
18. Pneumococcal meningitis deaths by approach for 2000-2015
19. Hib meningitis cases by approach for 2000-2015
20. Hib meningitis deaths by approach for 2000-2015

Webappendix 1: Literature review

Data sources

For the pneumonia models, we identified PCV and Hib vaccine trials and effectiveness studies from a systematic review of the literature and a Cochrane review of PCV efficacy.[1] We also updated a systematic review of pneumococcal and Hib invasive disease from 1980-2005[2] with published and unpublished data through 2014 for the pathogen-specific meningitis and NPNM models. We searched six global databases (i.e., PubMed, Embase, Biosis, Cochrane, Global Health, Pascal) and five regional databases (i.e., IMEMR, IMSEAR, LILACS, WHOLIS, WPRIM) and followed the same quality assessment criteria described in the previously published literature review.[2] We also used unpublished data from the Hib Rapid Assessment Tool and WHO Invasive Bacterial Disease surveillance network.[3] Other model parameter values were obtained from publicly available sources or personal communication. We used state-specific input values for India whenever possible to account for wide disparities across states.

Literature review methods

The existing systematic literature review[2] of pneumococcal and Hib disease data from 1980-2005 was updated to include published and unpublished literature through 2014, including data on pathogen-specific meningitis case fatality ratio (CFR), the etiologic distribution of bacterial meningitis cases, and pathogen-specific meningitis. We also used unpublished data from the Hib Rapid Assessment Tool and WHO Invasive Bacterial Disease surveillance network.[3]

The literature review was conducted in several phases. Published studies were identified through six global databases (i.e., PubMed, Embase, Biosis, Cochrane, Global Health, Pascal) and five regional databases (i.e., IMEMR, IMSEAR, LILACS, WHOLIS, WPRIM). Using an online systematic review platform, DistillerSR (https://www.evidencepartners.com), two reviewers screened each article based on titles and abstracts. Next, the full text of studies meeting the inclusion criteria based on title and abstract were screened again by two reviewers. Finally, two reviewers reviewed studies one additional time, extracted relevant data, and assessed quality for those that met the inclusion criteria. Two lead reviewers adjudicated conflicting reviews. The PRISMA flow diagram of studies is described in Figure 1. In total, 378 studies published between 1990 and 2014 were identified that contributed data to at least one of the pathogen-specific syndromic models – 150 were identified in the updated literature review (i.e., 2010 to 2014).

PubMed Search Strategy

Concept 1: *Streptococcus pneumoniae*
"Streptococcus pneumoniae"[Mesh] OR "streptococcus pneumoniae"[all fields] OR "streptococcal pneumonia"[all fields] OR "diplococcus pneumoniae"[all fields] OR "pneumococcus"[all fields] OR "pneumococcic pneumonia"[all fields] OR "s pneumoniae"[all fields] OR "pneumococci"[all fields] OR "pneumococcal"[all fields] OR "Pneumococcal

Vaccines"[Mesh] OR "prevnar"[all fields] OR "pnu-imune 23"[all fields] OR " 7-valent pncompc vaccine"[all fields] OR "pncrm7"[all fields] OR "mnc-crm197"[all fields] OR "mncc"[all fields] OR "pneumovax"[all fields] OR "pnu-imune vaccine"[all fields] OR “pcv 7"[all fields] OR “pcv 10"[all fields] OR “pcv 13"[all fields] OR "prevenar"[all fields] OR "prevnar"[all fields] OR "streptorix"[all fields] OR "synflorix"[all fields]

Concept 2: *Haemophilus influenzae* type b (Hib)
“Haemophilus influenzae”[Mesh] OR “haemophilus influenzae type b”[Mesh] OR haemophilus influenzae”[Mesh] OR “diphtheria pertussis poliomyelitis tetanus haemophilus influenzae type b hepatitis b vaccine”[all fields] OR “diphtheria pertussis tetanus haemophilus influenzae type b vaccine”[all fields] OR “diphtheria pertussis tetanus haemophilus influenzae type b vaccine”[all fields] OR “haemophilus influenzae type b dtp vaccine”[all fields] OR “haemophilus influenzae type b hepatitis b vaccine”[all fields] OR “haemophilus influenzae type b vaccine”[all fields] OR “hepatitis b haemophilus influenzae type b vaccine”[all fields] OR “hib ompc”[all fields] OR “hib vax”[all fields] OR “h influenzae”[all fields] OR “hib disease”[all fields] OR “hib infection”[all fields] OR “hib immuni”[all fields] OR “hemophilius influ”[all fields] OR “hemophilus influenzae”[all fields]

Concept 3: Disease
“invasive pneumococcal disease”[all fields] OR “pneumococcal invasive disease”[all fields] OR “Streptococcal Infections”[Mesh] OR “Streptococcal Infections”[all fields] OR “Streptococcal Infection”[all fields] OR “streptococcus infection” OR “Meningitis, Pneumococcal”[Mesh] OR “pneumococcal bacteraemia” OR “Meningitis, Bacterial”[Mesh] OR “Bacterial Infections”[Mesh] OR “bacterial infection” OR “bacterial infections” OR “Meningitis”[Mesh] OR “meningitis” OR “meningitides” OR “meningeal” OR “perimeningeal” OR “Respiratory Tract Diseases”[Mesh] OR “respiratory tract disease”[all fields] OR “respiratory tract diseases”[all fields] OR “airway disease”[all fields] OR “airway diseases”[all fields] OR “airway disorder”[all fields] OR “airway disorders”[all fields] OR “respiratory disease”[all fields] OR “respiratory disorder”[all fields] OR “respiratory disorders”[all fields] OR “respiratory illness”[all fields] OR “respiratory tract diseases”[all fields] OR “respiratory tract disease”[all fields] OR “respiratory tract disorder”[all fields] OR “respiratory tract disorders”[all fields] OR “lower respiratory tract infection”[all fields] OR “lower respiratory tract infections”[all fields] OR “lower respiration tract infections”[all fields] OR “lung infection”[all fields] OR “lung infections”[all fields] OR “pulmonary infection”[all fields] OR “pulmonary infections”[all fields] OR “Pneumonia, Bacterial”[Mesh] OR “pneumonia”[mesh] OR “pneumonia”[all fields] OR “pneumonic”[all fields] OR “Pneumonias”[all fields] OR “Pneumonitis”[all fields] OR “Pneumonitides”[all fields] OR “pleuropneumonia”[all fields] OR “pleuropneumonitis”[all fields] OR “Pulmonary Inflammation”[all fields] OR “alri”[all fields] OR “peripneumonia”[all fields] OR “pulmonary inflammation”[all fields] OR “Bacteremia”[Mesh] OR “bacteremia”[all fields] OR “bacteremias”[all fields] OR “septicemia”[all fields] OR “septicaemia”[all fields] OR “Sepsis”[Mesh] OR “sepsis”[all fields] OR “Pyemia”[all fields] OR “Pyemias”[all fields] OR “Pyohemia”[all fields] OR “Pyaemia”[all fields] OR “infectious pneumopath”[all fields]

Concept 4: Age group
"child"[mesh] OR "child"[all fields] OR "children"[all fields] OR youth* OR "young people"[all fields] OR "baby"[all fields] OR "babies"[all fields] OR "infant"[mesh] OR infant* OR "childhood"[all fields] OR toddler* OR "kid"[all fields] OR "kids"[all fields] OR "young patient"[all fields] OR "young patients"[all fields] OR "boy"[all fields] OR "boys"[all fields] OR girl* OR "young age"[all fields] OR "child mortality"[mesh] OR "pediatric"[all fields] OR "Child, Preschool"[Mesh] OR “pre-schooler"[all fields] OR “preschooler"[all fields] OR “under 5"[all fields] OR “under five"[all fields] OR “under fives"[all fields] OR "less than five"[all fields]

Concept 5: Exclusions
NOT "Case Reports" [Publication Type] OR "case report"[all fields] OR "case reports"[all fields] NOT ("animals"[mh] NOT ("animals"[mh] AND "humans"[mh]))

Figure 1: Systematic review PRISMA flow diagram of studies

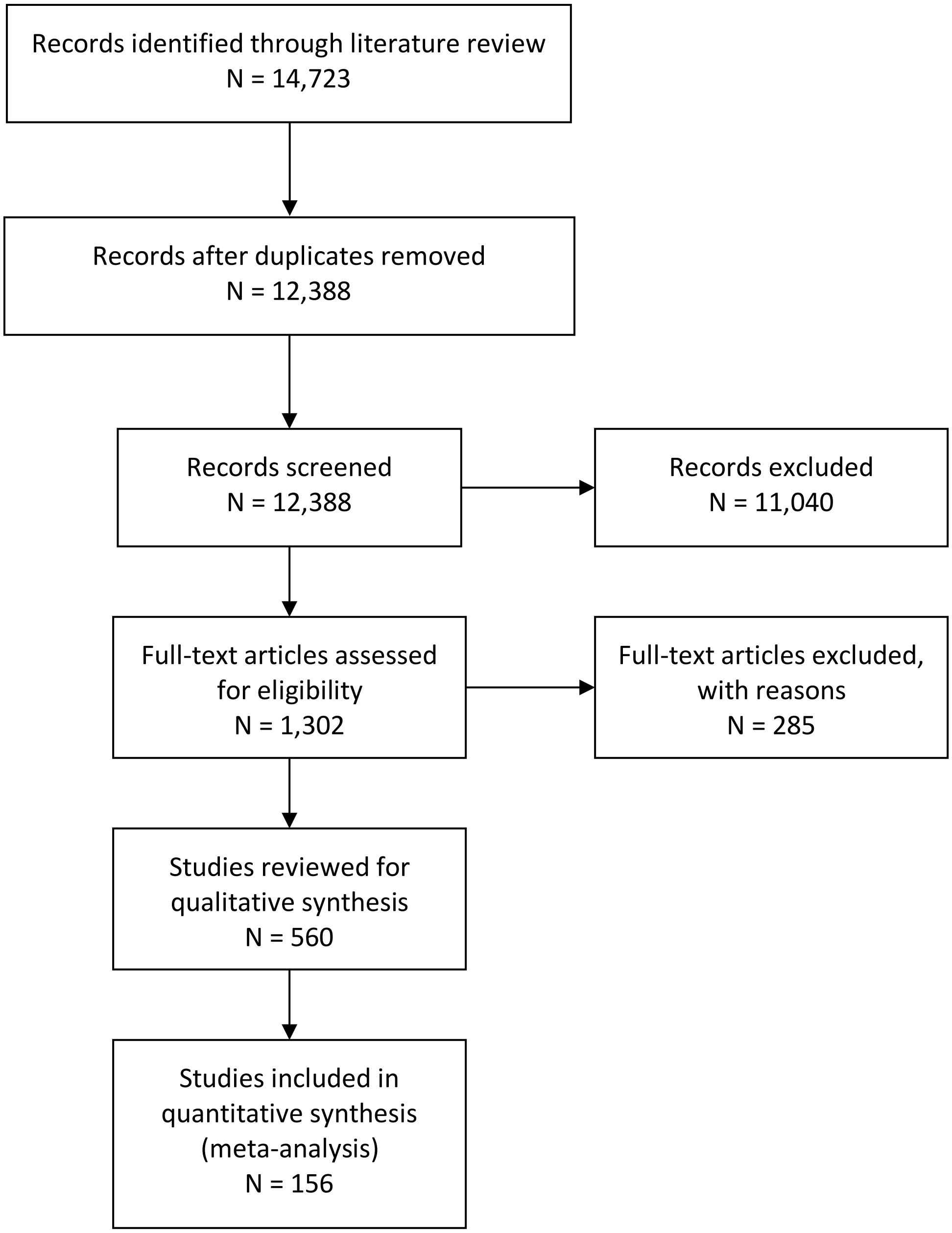

From: Moher D, Liberati A, Tetzlaff J, Altman DG, The PRISMA Group (2009). *P*referred *R*eporting *I*tems for *S*ystematic Reviews and *M*eta-*A*nalyses: The PRISMA Statement. PLoS Med 6(7): e1000097. doi:10.1371/journal.pmed1000097

Quality assessment criteria

Quality assessments for studies reporting incidence, case fatality ratio, and the distribution of bacterial meningitis cases by etiology were based on two subjective criteria: (1) the likelihood of the study missing cases and (2) the reliability of the diagnostic methods for laboratory isolation. These criteria were assessed independently by two data revieewers during the data abstraction phase. Based on these criteria, studies were classified into one of three categories: "A" papers where both reviewers judged both criteria to have been met; "B" papers where only one reviewer judged each criterion to have been met; "C" papers where both reviewers judged that neither criterion had been met or data was not available to allow a judgment. Category “C” studies, as well as all included studies from Asia and Africa, underwent a third quality assessment before a decision was made about inclusion in the final data set for meningitis.

Webappendix 2: Details of disease burden data inputs

Table 1: Studies contributing to estimates of pneumonia morbidity and mortality attributable fractions

a. Streptococcus pneumoniae
United States (California)<br>• Black, S. B., et al. "Effectiveness of heptavalent pneumococcal conjugate vaccine in children younger than five years of age for prevention of pneumonia." *Pediatric Infectious Disease Journal* 21.9 (2002): 810-815.<br>• Hansen, J., et al. "Effectiveness of heptavalent pneumococcal conjugate vaccine in children younger than 5 years of age for prevention of pneumonia: updated analysis using World Health Organization standardized interpretation of chest radiographs." *Pediatric Infectious Disease Journal* 25.9 (2006): 779-781.<br>• Black, S. B., et al. "Efficacy, safety and immunogenicity of heptavalent pneumococcal conjugate vaccine in children." *Pediatric Infectious Disease Journal* 19.3 (2000): 187-195.<br><br>South Africa<br>• Madhi, S. A., et al. "The impact of a 9-valent pneumococcal conjugate vaccine on the public health burden of pneumonia in HIV-infected and-uninfected children." *Clinical Infectious Diseases* 40.10 (2005): 1511-1518.<br>• Klugman, K. P., et al. "A trial of a 9-valent pneumococcal conjugate vaccine in children with and those without HIV infection." *New England Journal of Medicine* 349.14 (2003): 1341-1348.<br><br>The Gambia<br>• Cutts, F. T., et al. "Efficacy of nine-valent pneumococcal conjugate vaccine against pneumonia and invasive pneumococcal disease in The Gambia: randomised, double-blind, placebo-controlled trial." *The Lancet* 365.9465 (2005): 1139-1146.<br><br>The Philippines<br>• Lucero, M. G., et al. "Efficacy of an 11-valent pneumococcal conjugate vaccine against radiologically confirmed pneumonia among children less than 2 years of age in the Philippines: a randomized, double-blind, placebo-controlled trial." *Pediatric Infectious Disease Journal* 28.6 (2009): 455-462.<br>• Lupisan, S. P., et al. "Invasive bacterial infections of children in a rural province in the central Philippines." *American Journal of Tropical Medicine and Hygiene* 62.3 (2000): 341-346.<br><br>South America<br>• Tregnaghi, M. W., et al. "Efficacy of pneumococcal nontypable *Haemophilus influenzae* protein D conjugate vaccine (PHiD-CV) in young Latin American children: a double-blind randomized controlled trial." *PLoS Med* 11.6 (2014): e1001657.<br><br>Finland<br>• Kilpi, T., et al. "Effectiveness of Pneumococcal *Haemophilus influenzae* Protein D Conjugate Vaccine against Radiologically Confirmed Pneumonia." Interscience Conference on Antimicrobial Agents and Chemotherapy (ICAAC) 2014, Washington, D.C.<br>• Palmu, A. A., et al. "Effectiveness of the ten-valent pneumococcal *Haemophilus influenzae* protein D conjugate vaccine (PHiD-CV10) against invasive pneumococcal disease: a cluster randomised trial." *The Lancet*. 381.9862 (2013): 214-222.

***b. Haemophilus influenzae* type b (Hib)**
The Gambia<br>• Mulholland, Kim, et al. "Randomised trial of *Haemophilus influenzae* type-b tetanus protein conjugate for prevention of pneumonia and meningitis in Gambian infants." *The Lancet* 349.9060 (1997): 1191-1197. Ref 2<br><br>Indonesia<br>• Gessner, Bradford D., et al. "Incidences of vaccine-preventable *Haemophilus influenzae* type b pneumonia and meningitis in Indonesian children: hamlet-randomised vaccine-probe trial." *The Lancet* 365.9453 (2005): 43-52.<br><br>Bangladesh<br>• Baqui, Abdullah H., et al. "Effectiveness of *Haemophilus influenzae* type B conjugate vaccine on prevention of pneumonia and meningitis in Bangladeshi children: a case–control study." *Pediatric Infectious Disease Journal* 26.7 (2007): 565-571.<br><br>Chile<br>• Levine, Orin S., et al. "Defining the burden of pneumonia in children preventable by vaccination against *Haemophilus influenzae* type b." *Pediatric Infectious Disease Journal* 18.12 (1999): 1060-1064.<br>• Lagos, Rosanna, et al. "Large scale, postlicensure, selective vaccination of Chilean infants with PRP-T conjugate vaccine: practicality and effectiveness in preventing invasive *Haemophilus influenzae* type b infections." *Pediatric Infectious Disease Journal* 15.3 (1996): 216-222.

Table 2: Studies contributing to estimates of pathogen-specific meningitis case fatality ratio estimates stratified by child mortality setting

a. Streptococcus pneumoniae
Low child mortality settings (<30 deaths per 1000 live births)
• Al Awaidy, et al. "Epidemiology of invasive pneumococcal infection among hospitalized children aged less than 5 years in Oman." *Vaccine* 30 (2012): G7-G10. • Al Ayed, et al. "Retrospective review of invasive pediatric pneumococcal diseases in a military hospital in the southern region of Saudi Arabia." *Annals of Saudi Medicine* 31.5 (2011): 469. • Al Musawi, M., et al. "A retrospective epidemiological study of invasive pneumococcal infections in children aged 0-5 years in Bahrain from 1 January 1999 to 31 December 2003." *Vaccine*. 2012. 30 Suppl 6:G2-6. • Andrade, A. L., et al. "Population-based surveillance for invasive pneumococcal disease and pneumonia in infants and young children in Goiania, Brazil." *Vaccine*. 2012. 30:1901-9 • Batuwanthudawe, R., et al. "Surveillance of invasive pneumococcal disease in Colombo, Sri Lanka." *Clinical Infectious Diseases* 48.Supplement 2 (2009): S136-S140. • Benavides, J. A., et al. "Population-based surveillance for invasive pneumococcal disease and pneumonia in infants and young children in Bogota, Colombia." *Vaccine*. 2012. 30:5886-92 • Biaukula, V. L., et al. "Meningitis in children in Fiji: etiology, epidemiology, and neurological sequelae." *International Journal of Infectious Diseases* 16.4 (2012): e289-e295. • Camou, T., et al. "Invasive pneumococcal diseases in Uruguayan children: comparison between serotype distribution and conjugate vaccine formulations." *Vaccine* 21.17 (2003): 2093-2096. • Chan, Iris Mei-Ching, et al. "Invasive pneumococcal disease in Hong Kong children." J *Pediatr Respirology Critic Care* 9.2 (2013): 4-8. • Chavez, P., et al. "Meningitis bacteriana aguda: experiencia de 10 años." *Rev Chil Infectol*;11(2):92-98, 1994 • Davidson, M., et al. "The Epidemiology of Invasive Pneumococcal Disease in Alaska, 1986-1990 Ethnic Differences and Opportunities for Prevention." *Journal of Infectious Diseases* 170.2 (1994): 368-376. • Dickinson M., et al., "Las meningoencefalitis bacterianas en la población infantil cubana: 1998-2000." *Revista Cubana de Pediatría* 74.2 (2002): 106-114. • Fortnum, H. M. and A. C. Davis. "Epidemiology of bacterial meningitis." *Archives of Disease in Childhood* 68.6 (1993): 763-767. • Grzesiowski, P., et al. "Invasive pneumococcal disease in children up to 5 years of age in Poland." *Eur J Clin Microbiol Infect Dis.* 2008. 27:883-5 • Hanna J. N., et al. "Bacterial meningitis in children under five years of age in Western Australia. *Medical Journal of Australia*." 1991.;155:3,160-164 • Harboe, Z. B., et al. "Pneumococcal serotypes and mortality following invasive pneumococcal disease: a population-based cohort study." *PLoS Med* 6.5 (2009): e1000081. • Ishikawa, T., et al. "Epidemiology of bacterial meningitis in children: Aichi prefecture, Japan, 1984-1993." *Pediatric Neurology*. 1996 Apr;14(3):244-250 • Jansen, A. G., et al. " Invasive pneumococcal disease in the Netherlands: Syndromes, outcome and potential vaccine benefits." *Vaccine*. 2009. 27:2394-401 • Johnson, A. P., et al. "Morbidity and mortality of pneumococcal meningitis and serotypes of causative strains prior to introduction of the 7-valent conjugant pneumococcal vaccine in England." *Journal of Infection* 55.5 (2007): 394-399. • Lagos, R., et al., "Age- and serotype-specific pediatric invasive pneumococcal disease: insights from systematic surveillance in Santiago, Chile, 1994--2007." *J Infect Dis.* 2008. 198:1809-17 • Lagos, Z. R., et al. "Epidemiología de las enfermedades invasoras causadas por *Streptococcus pneumoniae* en niños chilenos: proyecciones clínicas y de salud pública." *Revista chilena de infectología* 18 (2001): 15-21. • Lim, L. H., et al. "Childhood invasive pneumococcal disease: a hospital-based study from Malaysia." *J Paediatr Child Health*. 2007. 43:366-9 • Lucey, J. M., et al. "Pneumococcal meningitis: clinical outcomes in a pre-vaccine era at a Dublin paediatric hospital, 1999–2007." *Irish Journal of Medical Science* 180.1 (2011): 47-50.

- McIntyre, P. B., et al., "A population based study of the impact of corticosteroid therapy and delayed diagnosis on the outcome of childhood pneumococcal meningitis." *Archives of Disease in Childhood*. 2005. 90:391-396
- Memish, Z. A., et al., "Epidemiology of invasive pneumococcal infection in children aged five years and under in Saudi Arabia: a five-year retrospective surveillance study." *Int J Infect Dis*. 2010. 14:e708-12
- Miller, E., et al. "Epidemiology of invasive and other pneumococcal disease in children in England and Wales 1996–1998." *Acta Paediatrica* 89.s435 (2000): 11-16.
- Namani, S. R. K., et al. "Causative pathogens of bacterial meningitis in children and their susceptibility to antibiotics." *The Internet JInfects Dis* 9 (2010): 1-6.
- Rendi-Wagner, P., et al. "Prospective surveillance of incidence, serotypes and antimicrobial susceptibility of invasive *Streptococcus pneumoniae* among hospitalized children in Austria." *Journal of Antimicrobial Chemotherapy*. 2004;53(5):826-831
- Ríos, A. M., et al. "Impacto de la resistencia a antimicrobianos y de serotipos de *Streptococcus pneumoniae* en la mortalidad de niños menores de 5 años con enfermedad invasora." (1999).
- Robinson, K. A., et al. "Epidemiology of invasive *Streptococcus pneumoniae* infections in the United States, 1995-1998: Opportunities for prevention in the conjugate vaccine era." *Jama* 285.13 (2001): 1729-1735.
- Trotman, H., et al. "Pneumococcal meningitis in Jamaican children." *West Indian Medical Journal* 58.6 (2009): 585-588.
- Von Kries, R., et al. "Prediction of the potential benefit of different pneumococcal conjugate vaccines on invasive pneumococcal disease in German children." *Pediatric Infectious Disease Journal* 21.11 (2002): 1017-1023.
- Voss, L., et al. "Invasive pneumococcal disease in a pediatric population, Auckland, New Zealand." *Pediatric Infectious Disease Journal*. 1994;13(10):873-878

Medium child mortality settings (30-<75 deaths per 1000 live births)

- Abucejo-Ladesma, E., et al. "Serious community-acquired paediatric infections in rural Asia (Bohol Island, Philippines): bacterial meningitis in children less than 5 years of age." *Scandinavian Journal of Infectious Diseases* 39.11-12 (2007): 983-989.
- Asturias, E. J., et al. "Meningitis and pneumonia in Guatemalan children: the importance of *Haemophilus influenzae* type b and *Streptococcus pneumoniae*." *Revista Panamericana de Salud Pública* 14.6 (2003): 377-384.
- Berkley, J. A., et al. "Bacteremia among children admitted to a rural hospital in Kenya." New England Journal of Medicine 352.1 (2005): 39-47.
- Capeding, Maria Rosario, et al. "Prospective surveillance study of invasive pneumococcal disease among urban children in the Philippines." Pediatric Infectious Disease Journal 32.10 (2013): e383-e389.
- Chhetri, U. D., et al. "Clinical profile of invasive pneumococcal disease in Patan Hospital, Nepal." *Kathmandu University Medical Journa*l 9.1 (2012): 45-49.
- El Mdaghri, N., et al. "Epidemiological profile of invasive bacterial diseases in children in Casablanca, Morocco: antimicrobial susceptibilities and serotype distribution." *Eastern Mediterranean Health Journal* 18.11 (2012): 1097.
- Lovera, D., et al., "Risk factors for mortality in Paraguayan children with pneumococcal bacterial meningitis." *Tropical Medicine & International Health* 10.12 (2005): 1235-1241.
- Reis, J. N., et al. "Population-based survey of antimicrobial susceptibility and serotype distribution of Streptococcus pneumoniae from meningitis patients in Salvador, Brazil." *Journal of Clinical Microbiology* 40.1 (2002): 275-277.
- Shah, A. S., et al. "Invasive pneumococcal disease in Kanti Children's Hospital, Nepal, as observed by the South Asian Pneumococcal Alliance network." *Clinical Infectious Diseases* 48.Supplement 2 (2009): S123-S128.
- Williams, E. J., et al. "Hospital-based surveillance of invasive pneumococcal disease among young children in urban Nepal." *Clinical Infectious Diseases* 48. Supplement 2 (2009): S114-S122.

High child mortality settings (75-<150 deaths per 1000 live births)

- Al Khorasani, A., et al., "Bacterial profile and clinical outcome of childhood meningitis in rural Yemen: a 2-year hospital-based study." *Journal of Infection* 53.4 (2006): 228-234.
- Ba, O., et al. "Hospital surveillance of childhood bacterial meningitis in Senegal and the introduction *of Haemophilus influenzae* type b conjugate vaccine." *American Journal of Tropical Medicine and Hygiene* 83.6 (2010): 1330-1335.
- Iriso, R., et al. "Bacterial meningitis following introduction of Hib conjugate vaccine in northern Uganda." *Annals of Tropical Paediatrics* 28.3 (2008): 211-216.
- Khowaja, A. R., et al. "Mortality and neurodevelopmental outcomes of acute bacterial meningitis in children aged< 5 years in Pakistan." The Journal of Pediatrics 163.1 (2013): S86-S91.
- Kisakye, A., et al. "Surveillance for *Streptococcus pneumoniae* meningitis in children aged< 5 years: implications for immunization in Uganda." *Clinical Infectious Diseases* 48.Supplement 2 (2009): S153-S161.
- Menezes, Ana Paula de O., et al. "Serotype distribution and antimicrobial resistance of *Streptococcus pneumoniae* prior to introduction of the 10-valent pneumococcal conjugate vaccine in Brazil, 2000–2007." Vaccine 29.6 (2011): 1139-1144.
- Zaidi, A. K. M., et al. "Surveillance of pneumococcal meningitis among children in Sindh, southern Pakistan." *Clinical Infectious Diseases* 48.Supplement 2 (2009): S129-S135.

Very high child mortality settings (>=150 deaths per 1000 live births)

- Bercion, R., et al. "Acute bacterial meningitis at the ‘Complexe Pédiatrique’of Bangui, Central African Republic." *Journal of Tropical Pediatrics* 54.2 (2008): 125-128.
- Bernard-Bonnin, A. C., et al. "Purulent meningitis in children in Yaounde: epidemiological and prognostic aspects." *Annales de la Societe Belge de Medecine Tropicale*. Vol. 65. No. 1. 1984.
- Falade, A. G., et al. "Invasive pneumococcal disease in children aged< 5 years admitted to 3 urban hospitals in Ibadan, Nigeria." *Clinical Infectious Diseases* 48.Supplement 2 (2009): S190-S196.
- Tall, F. R., et al. "*Haemophilus influenzae* meningitis in a hospital in Burkina-Faso." *Medecine et Maladies Infectieuses*. 1992;22(12):1173-1177
- Yaro, S., et al. "The epidemiology of Haemophilus influenzae type b meningitis in Burkina Faso." *Pediatric Infectious Disease Journal* 25.5 (2006): 415-419.

***b. Haemophilus influenzae* type b (Hib)**
Low child mortality settings (<30 deaths per 1000 live births)

- Almuneef, M., et al. "Childhood bacterial meningitis in Saudi Arabia." *Journal of Infection* 36.2 (1998): 157-160.
- Anh, Dang D., et al. "*Haemophilus influenzae* type B meningitis among children in Hanoi, Vietnam: epidemiologic patterns and estimates of H. Influenzae type B disease burden." *American Journal of Tropical Medicine and Hygiene* 74.3 (2006): 509-515.
- Anh, D. D., et al. "Surveillance of pneumococcal-associated disease among hospitalized children in Khanh Hoa Province, Vietnam." *Clinical Infectious Diseases* 48.Supplement 2 (2009): S57-S64.
- Barton-Forbes, M. A., et al., "Epidemiology of *Haemophilus influenzae* invasive disease in Jamaica, 1990-1993." *West Indian Medical Journal* 49.3 (2000): 200-204.
- Batuwanthudawe, R., et al. "Surveillance of invasive pneumococcal disease in Colombo, Sri Lanka." *Clinical Infectious Diseases* 48.Suppl. 2 (2009): S136-S140.
- Batuwanthudawe, R., et al. "Incidence of childhood *Haemophilus influenzae* type b meningitis in Sri Lanka." *International Journal of Infectious Diseases* 14.5 (2010): e372-e376.
- Boehme, C., et al. "Three years of acute bacterial meningitis in the pediatric service at the Temuco Regional Hospital." *Revista Medica de* Chile 121.6 (1993): 633-638.
- Booy, R., et al. "Invasive *Haemophilus influenzae* type b disease in the Oxford region (1985-91)." *Archives of Disease in Childhood* 69.2 (1993): 225-228.
- Bower, C., et al. "Sequelae of *Haemophilus influenzae* type b meningitis in Aboriginal and non-Aboriginal children under 5 years of age." *Journal of Paediatrics and Child Health* 30.5 (1994): 393-397.
- Chavez, P., et al. "Meningitis bacteriana aguda: experiencia de 10 años." *Rev. Chil. Infectol* 11.2 (1994): 92-8.
- Chen, M. K., et al. "Prospective surveillance of children with invasive *Haemophilus influenzae* disease in Taiwan." *Journal of Microbiology, Immunology, and Infection* 32.4 (1999): 257-260.
- Dagan, R., et al. "Epidemiology of pediatric meningitis caused by *Haemophilus influenzae* type b, *Streptococcus pneumoniae*, and Neisseria meningitidis in Israel: a 3-year nationwide prospective study." *Journal of Infectious Diseases* 169.4 (1994): 912-916.
- De Jonghe, M. and G. Glaesener. "Type B *Haemophilus influenzae* infections. Experience at the pediatric hospital of Luxembourg." *Bulletin de la Société des Sciences Médicales du Grand-Duché de Luxembourg* 132.2 (1995): 17.
- Dickinson, M., et al., "Las meningoencefalitis bacterianas en la población infantil cubana: 1998-2000." *Revista Cubana de Pediatría* 74.2 (2002): 106-114.
- Fortnum, H. M. and A. C. Davis. "Epidemiology of bacterial meningitis." Archives of disease in childhood 68.6 (1993): 763-767.
- Gilbert, G. L., et al., "Clinical manifestations and outcome of *Haemophilus influenzae* type b disease." *Journal of Paediatrics and Child Health* 31.2 (1995): 99-104.
- Halfon-yaniv, I., et al., "Epidemiology of invasive *Haemophilus influenzae* type b infections in Bedouins and Jews in Southern Israel." *Pediatric Infectious Disease Journal* 9.5 (1990): 321-325.
- Ishikawa, T., et al. "Epidemiology of bacterial meningitis in children: Aichi Prefecture, Japan, 1984–1993." *Pediatric Neurology* 14.3 (1996): 244-250.
- Kamiya, Hitoshi, et al. "Childhood bacterial meningitis in Japan." *Pediatric Infectious Disease Journal* 17.9 (1998): S183-S185.
- Kristensen, K., et al. "Epidemiology of *Haemophilus influenzae* type b infections among children in Denmark in 1985 and 1986." *Acta Paediatrica* 79.6-7 (1990): 587-592.
- McIntyre, P. B., et al. "Invasive *Haemophilus influenzae* type b disease in Sydney children 1985-1987: a population-based study." *Medical Journal of Australia* 154.12 (1991): 832-837.
- McIntyre, P., et al. "The outcome of childhood *Haemophilus influenzae* meningitis. A population based study." *Medical Journal of Australia* 159.11-12 (1992): 766-772.
- Muangchana, C., et al. "Bacterial meningitis incidence in Thai children estimated by a rapid assessment tool." *Southeast Asian Journal of Tropical Medicine and Public Health* 40.3 (2009): 553.

- Namani, S., et al. "Causative pathogens of bacterial meningitis in children and their susceptibility to antibiotics." *Journal of Infectious Disease* 9 (2010): 1-6.
- Rauter, L., and I. Mutz. "*Haemophilus influenzae* meningitis 1983 to 1992--epidemiology and sequelae of the disease." *Wiener Klinische Wochenschrift* 106.7 (1993): 187-192.
- Reinert, P., et al. "Epidemiology of *Haemophilus influenzae* type b disease in France." *Vaccine* 11 (1993): S38-S42.
- Schoendorf, K. C., et al. "National trends in *Haemophilus influenzae* meningitis mortality and hospitalization among children, 1980 through 1991." *Pediatrics* 93.4 (1994): 663-668.
- Zaki, M., et al. "Childhood bacterial meningitis in Kuwait." *Journal of Tropical Medicine and Hygiene* 93.1 (1990): 7-11.

Medium child mortality settings (30-<75 deaths per 1000 live births)

- Abucejo-Ladesma, E., et al. "Serious community-acquired paediatric infections in rural Asia (Bohol Island, Philippines): bacterial meningitis in children less than 5 years of age." *Scandinavian Journal of Infectious Diseases* 39.11-12 (2007): 983-989.
- Ansari, I., et al. "Culture proven bacterial meningitis in children: agents, clinical profile and outcome." *Kathmandu Univ Med J.* 2011. 9:36-40
- Asturias, Edwin J., et al. "Meningitis and pneumonia in Guatemalan children: the importance of *Haemophilus influenzae* type b and *Streptococcus pneumoniae*." *Revista Panamericana de Salud Pública* 14.6 (2003): 377-384.
- Basualdo, W., et al. "Invasive *Haemophilus influenzae* type b infections in children in Paraguay." *Archives of Medical Research* 35.2 (2004): 126-133.
- El Mdaghri, N., et al. "Epidemiological profile of invasive bacterial diseases in children in Casablanca, Morocco: antimicrobial susceptibilities and serotype distribution." *Eastern Mediterranean Health Journal* 18.11 (2012): 1097.
- Hussey, G., et al. "Epidemiology of invasive *Haemophilus influenzae* infections in Cape Town, South Africa." *Annals of Tropical Paediatrics* 14.2 (1994): 97-103.
- Limcangco, M. R. T., et al. "Epidemiology of *Haemophilus influenzae* type b meningitis in Manila, Philippines, 1994 to 1996." *Pediatric Infectious Disease Journal* 19.1 (2000): 7-11.
- Luby, S. P., et al. "A low-cost approach to measure the burden of vaccine preventable diseases in urban areas." *Vaccine* 28.31 (2010): 4903-4912.
- Mendsaikhan, Jamsran, et al. "Childhood bacterial meningitis in Ulaanbaatar, Mongolia, 2002–2004." *Clinical Infectious Diseases* 48.Supplement 2 (2009): S141-S146.
- Minz, S., et al. "Incidence of *Haemophilus influenzae* type b meningitis in India." *Indian Journal of Medical Research* 128.1 (2008): 57.
- Ribeiro, G. S., et al. "Prevention of *Haemophilus influenzae* type b (Hib) meningitis and emergence of serotype replacement with type a strains after introduction of Hib immunization in Brazil." *Journal of Infectious Diseases* 187.1 (2003): 109-116.
- Saha, S. K., et al. "The increasing burden of disease in Bangladeshi children due to *Haemophilus influenzae* type b meningitis." *Annals of Tropical Paediatrics* 17.1 (1997): 5-8.
- Shah, A. S., et al. "Invasive pneumococcal disease in Kanti Children's Hospital, Nepal, as observed by the South Asian Pneumococcal Alliance network." *Clinical Infectious Diseases* 48.Supplement 2 (2009): S123-S128.
- Simões, L. L. P., et al. "Impact of *Haemophilus influenzae* b (Hib) vaccination on meningitis in Central Brazil." *Revista de Saude Publica* 38.5 (2004): 664-670.
- Torres, A., et al. "Infecciones invasivas por *Haemophilus influenzae* tipo b (Hib) en Tucumán-Argentina." *Arch. Argent. Pediatr* 93.4 (1995): 238-44.
- Weiss, D. P.L., et al. "Epidemiology of bacterial meningitis among children in Brazil, 1997-1998." *Revista de Saude Publica* 35.3 (2001): 249-255.

High child mortality settings (75-<150 deaths per 1000 live births)

- Adegbola, R. A., et al. "*Haemophilus influenzae* type b disease in the western region of The Gambia: background surveillance for a vaccine efficacy trial." *Annals of Tropical Paediatrics* 16.2 (1996): 103-111.
- Invasive Bacterial Infections Surveillance (IBIS) Group of the International Clinical Epidemiology Network. "Are *Haemophilus influenzae* infections a significant problem in India? A prospective study and review." *Clinical Infectious Diseases* 34.7 (2002): 949-957.
- Khowaja, A. R., et al. "Mortality and neurodevelopmental outcomes of acute bacterial meningitis in children aged< 5 years in Pakistan." *Journal of Pediatrics* 163.1 (2013): S86-S91.
- Koko, J., et al. "Bacterial meningitis in children in Libreville, Gabon. Epidemiologic, therapeutic and prognostic features." *Medecine et Maladies Infectieuses* 30.1 (2000): 50-56.
- Lewis, R. F., et al. "Action for child survival: elimination of *Haemophilus influenzae* type b meningitis in Uganda." *Bulletin of the World Health Organization* 86.4 (2008): 292-301.
- Zaidi, A. K. M., et al. "Surveillance of pneumococcal meningitis among children in Sindh, southern Pakistan." *Clinical Infectious Diseases* 48.Supplement 2 (2009): S129-S135.

Very high child mortality settings (>=150 deaths per 1000 live births)

- Bercion, R., et al. "Acute bacterial meningitis at the ‘Complexe Pédiatrique’of Bangui, Central African Republic." *Journal of Tropical Pediatrics* 54.2 (2008): 125-128.
- Bernard-Bonnin, A. C., et al. "Purulent meningitis in children in Yaounde: epidemiological and prognostic aspects." *Annales de la Societe Belge de Medecine Tropicale*. Vol. 65. No. 1. 1984.
- Bijlmer, H. A., et al. "The epidemiology of *Haemophilus influenzae* meningitis in children under five years of age in The Gambia, West Africa." *Journal of Infectious Diseases* 161.6 (1990): 1210-1215.
- Campagne, G., et al. "Epidemiology of bacterial meningitis in Niamey, Niger, 1981-96." *Bulletin of the World Health Organization* 77.6 (1999): 499.
- Lagunju, I. A., et al. "Childhood bacterial meningitis in Ibadan, Nigeria--antibiotic sensitivity pattern of pathogens, prognostic indices and outcome." *African Journal of Medicine and Medical sciences* 37.2 (2008): 185-191.
- Molyneux, E., et al. "Acute bacterial meningitis in children admitted to the Queen Elizabeth Central Hospital, Blantyre, Malawi in 1996–97." *Tropical Medicine & International Health* 3.8 (1998): 610-618.
- Muhe, L., et al. "Pneumococcal and *Haemophilus influenzae* meningitis in a children's hospital in Ethiopia: serotypes and susceptibility patterns." *Tropical Medicine & International Health* 4.6 (1999): 421-427.
- Tall, F. R., et al. "*Haemophilus influenzae* meningitis in a hospital in Burkina-Faso." *Medecine et Maladies Infectieuses*. 1992;22(12):1173-1177
- Yaro, S., et al. "The epidemiology of Haemophilus influenzae type b meningitis in Burkina Faso." *Pediatric Infectious Disease Journal* 25.5 (2006): 415-419.

Table 3: Studies contributing to the distribution of meningitis cases by bacterial pathogen, stratified by geographic region

Distribution of meningitis cases by etiology
Africa
• Afifi, S., et al. "Laboratory-based surveillance of patients with bacterial meningitis in Egypt (1998–2004)." *European Journal of Clinical Microbiology & Infectious Diseases* 26.5 (2007): 331-340.<br>• Akpede, O., et al. "Childhood bacterial meningitis beyond the neonatal period in southern Nigeria: changes in organisms/antibiotic susceptibility." *East African Medical Journal* 71.1 (1994): 14-20.<br>• Ba, O., et al. "Hospital surveillance of childhood bacterial meningitis in Senegal and the introduction of *Haemophilus influenzae* type b conjugate vaccine." *American Journal of Tropical Medicine and Hygiene* 83.6 (2010): 1330-1335.<br>• Bercion, R., et al. "Acute bacterial meningitis at the 'Complexe Pédiatrique'of Bangui, Central African Republic." *Journal of Tropical Pediatrics* 54.2 (2008): 125-128.<br>• Berkley, J. A., et al. "Indicators of acute bacterial meningitis in children at a rural Kenyan district hospital." *Pediatrics* 114.6 (2004): e713-e719.<br>• Bernard-Bonnin, A. C., and T. Ekoe. "Purulent meningitis in children in Yaounde: epidemiological and prognostic aspects." *Annales de la Societe Belge de Medecine Tropicale*. Vol. 65. No. 1. 1984.<br>• Camara, B., et al. "Purulent meningitis in a pediatric hospital (Dakar, Senegal)." *Medecine et Maladies Infectieuses* 33.8 (2003): 422-426.<br>• Cisse, M. F., et al. "Bacterial meningitis in a pediatric hospital in a tropical zone." *Medecine Tropicale: Revue du Corps de Sante Colonial* 49.3 (1988): 265-269.<br>• de Bary, J. B., et al. "Purulent meningitis at a semi-rural hospital in the forest area of the Ivory Coast." *Bulletin de la Société de Pathologie Exotique* (1990) 83.4 (1990): 460.<br>• du Châtelet, I. P., et al. "Bacterial meningitis in Burkina Faso: surveillance using field-based polymerase chain reaction testing." *Clinical Infectious Diseases* 40.1 (2005): 17-25.<br>• Emele, F. E. "Etiologic spectrum and pattern of antimicrobial drug susceptibility in bacterial meningitis in Sokoto, Nigeria." *Acta Paediatrica* 89.8 (2000): 942-946.<br>• Falade, A. G., et al. "Invasive pneumococcal disease in children aged< 5 years admitted to 3 urban hospitals in Ibadan, Nigeria." *Clinical Infectious Diseases* 48.Supplement 2 (2009): S190-S196.<br>• Faye-Ketté, H., et al. "Community acquired bacterial meningitis in childhood: epidemiological trends in Abidjan, Cote d'Ivoire from 1995 to 2000." *Bulletin-Societe de Pathologie* Exotique 96.4 (2003): 313-316.<br>• Fonkoua, M. C., et al., "Bacterial meningitis in Yaounde (Cameroon) in 1999-2000." *Bull Soc Pathol Exot.* 2001 Nov;94(4):d-303<br>• Forsyth, H., et al. "Hearing loss in Malawian children after bacterial meningitis: incidence and risk factors." *Audiological Medicine* 2.2 (2004): 100-107.<br>• Gedlu, E., et al., "Pyogenic meningitis in children in north-western Ethiopia." *Annals of Tropical Paediatrics* 15.3 (1995): 243-247.<br>• Khalifa, A. B., et al. "Acquired bacterial meningitis in Monastir region, Tunisia (1999-2006): bacteriological aspects and susceptibility patterns." *Bulletin de la Societe de Pathologie Exotique* (1990) 104.1 (2011): 42-48.<br>• Koko, J., et al. "Bacterial meningitis in children in Libreville, Gabon. Epidemiologic, therapeutic and prognostic features." *Medecine et Maladies Infectieuses* 30.1 (2000): 50-56.<br>• Lewis, Rosamund F., et al. "Action for child survival: elimination of *Haemophilus influenzae* type b meningitis in Uganda." *Bulletin of the World Health Organization* 86.4 (2008): 292-301.<br>• Lourd, M., et al. "The epidemiology of Neisseria meningitidis meningitis in Togo during 2003–2005." Vaccine 25 (2007): A47-A52.<br>• Maalej, S. M., et al. "Bacteriology of community acquired meningitis in Sfax, Tunisia (1993-2001)." *Medecine et Maladies Infectieuses* 36.2 (2006): 105-110.<br>• Madhi, S. A., et al. "Impact of human immunodeficiency virus type 1 infection on the epidemiology and outcome of bacterial meningitis in South African children." *International Journal of Infectious Diseases* 5.3 (2001): 119-125.

- Mbonda, E., et al. "Sequelles neurologiques des méningites: bactériennes chez le nourrisson et l'enfant a Yaounde." *Médecine d'Afrique Noire* 42.1 (1995): 39-45.
- Mirza, N. B., et al. "Bacterial meningitis in children admitted in hospitals within Nairobi." *East African Medical Journal* 75.2 (1998): 73-76.
- Mullan, P. C., et al. "Etiology of meningitis among patients admitted to a tertiary referral hospital in Botswana." *Pediatric Infectious Disease Journal* 30.7 (2011): 620-622.
- Naguib, H. F., et al., "Meningitis in El-Minia locality."
- O'dempsey, T. J. D., et al. "Importance of enteric bacteria as a cause of pneumonia, meningitis and septicemia among children in a rural community in The Gambia, West Africa." *Pediatric Infectious Disease Journal* 13.2 (1994): 122-127.
- Onipede, Anthony O., et al. "Invasive bacteria isolates from children with severe infections in a Nigerian hospital." *Journal of Infection in Developing Countries* 3.06 (2009): 429-436.
- Onyemelukwe, N. F. "*Haemophilus influenzae* meningitis in parts of eastern Nigeria." *East African Medical Journal* 71.2 (1994): 129-131.
- Orega, M., et al. "Les Méningites Purulentes De L'enfant A Abidjan (A Propos De 521 Cas)." *Médecine D'afrique Noire* 44.4 (1997): 213-9.
- Ouédraogo, S. M., et al. "Acute bacterial meningitis with soluble antigen detected by latex particle agglutination tests at the Sourô-Sanou University Hospital of Bobo-Dioulasso (Burkina Faso)." *Medecine et Sante Tropicales* 22.4 (2011): 412-416.
- Palmer, A., et al. "Brief report. Acute bacterial meningitis in The Gambia: a four-year review of paediatric hospital admissions." *Journal of Tropical Pediatrics* 45.1 (1999): 51-53.
- Roca, A., et al. "Surveillance of acute bacterial meningitis among children admitted to a district hospital in rural Mozambique." *Clinical Infectious Diseases* 48.Supplement 2 (2009): S172-S180.
- Salih, K. E. M. A., et al. "Pattern of bacterial meningitis in Sudanese children, Omdurman, Sudan." *African Journal of Microbiology Research* 4.24 (2010): 2670-2673.
- Salih, M. A. M., et al. "Endemic bacterial meningitis in Sudanese children: aetiology, clinical findings, treatment and short-term outcome." *Annals of Tropical Paediatrics* 10.2 (1990): 203-210.
- Shafie, W. A., et al. "Bacterial meningitis of children in Banha."
- Traore, Yves, et al. "Incidence, seasonality, age distribution, and mortality of pneumococcal meningitis in Burkina Faso and Togo." *Clinical Infectious Diseases* 48.Supplement 2 (2009): S181-S189.

Asia

- Abanamy, A., et al. "Childhood bacterial meningitis in Riyadh." *Annals of Saudi Medicine* 11.6 (1991): 628-632.
- Abomelha A. et al., "Childhood bacterial meningitis"
- Al Khorasani, A., et al., "Bacterial profile and clinical outcome of childhood meningitis in rural Yemen: a 2-year hospital-based study." *Journal of Infection* 53.4 (2006): 228-234.
- al-Jurayyan N. A., et al., "Childhood bacterial meningitis in Al-Baha province, Saudi Arabia"
- Al-Mazrou, Y. Y., et al. "*Haemophilus type* B meningitis in Saudi children under 5 years old." *Journal of Tropical Pediatrics* 50.3 (2004): 131-136.
- Almuneef, M., et al. "Childhood bacterial meningitis in Saudi Arabia." *Journal of Infection* 36.2 (1998): 157-160.
- Ansari, I., et al. "Culture proven bacterial meningitis in children: agents, clinical profile and outcome." *Kathmandu University Medical Journal* 9.1 (2012): 36-40.
- Azubuike J. C., et al., "Childhood bacterial meningitis in Tabuk, Saudi Arabia"
- Batuwanthudawe, R., et al. "Incidence of childhood *Haemophilus influenzae* type b meningitis in Sri Lanka." *International Journal of Infectious Diseases* 14.5 (2010): e372-e376.
- Batuwanthudawe, R., et al. "Surveillance of invasive pneumococcal disease in Colombo, Sri Lanka." *Clinical Infectious Diseases* 48.Supplement 2 (2009): S136-S140.
- Ceyhan, M., "A Prospective Study of Etiology of Childhood Acute Bacterial Meningitis, Turkey-Volume 14, Number 7—July 2008-Emerging Infectious Disease journal-CDC." (2008).

- Chinchankar, Nandita, et al. "Diagnosis and outcome of acute bacterial meningitis in early childhood." Indian pediatrics 39.10 (2002): 914-921.
- Choo, K. E., et al. "Pyogenic meningitis in hospitalized children in Kelantan, Malaysia." *Annals of Tropical Paediatrics* 10.1 (1990): 89-98.
- Dagan, R., et al. "Effectiveness of a nationwide infant immunization program against *Haemophilus influenzae* b." *Vaccine* 17.2 (1999): 134-141.
- Fujii, R., et al. "Trends in childhood bacterial meningitis in Japan (1979-1984).(Part 1). On the causative organisms." Kansenshōgaku zasshi. *Journal of the Japanese Association for Infectious Diseases* 60.6 (1986): 592.
- Gratten, M., et al. "The Bacteriology of Acute Pneumonia and Meningitis in Children in Papua New Guinea: Assumptions, Facts and Technical Strategies." *Papua New Guinea Medical Journal* 48.1/2 (2005): 73.
- Gurley, E. S., et al. "Etiologies of bacterial meningitis in Bangladesh: results from a hospital-based study." *American Journal of Tropical Medicine and Hygiene* 81.3 (2009): 475-483.
- Kim, Kyung-Hyo, et al. "The Causative Organisms of Bacterial Meningitis in Korean Children." *J Korean Med Sci* 13 (1998): 60-4.
- Kim, S. A., et al. "An expanded age range for meningococcal meningitis: molecular diagnostic evidence from population-based surveillance in Asia." *BMC Infectious Diseases* 12.1 (2012): 310.
- Lehmann, D., et al. "Aetiology and clinical signs of bacterial meningitis in children admitted to Goroka Base Hospital, Papua New Guinea, 1989–1992." *Annals of Tropical Paediatrics* 19.1 (1999): 21-32.
- Liu, C. C., et al. "Bacterial meningitis in infants and children in southern Taiwan: emphasis on *Haemophilus influenzae* type B infection." *Journal of the Formosan Medical Association Taiwan* 92.10 (1993): 884-888.
- Mahmood Y. A., et al., "Neonatal and childhood bacterial meningitis at Saddam general hospital in Tikrit." *Medical Journal of Tikrit* University (1997);3:39-45
- Mahmoud, R., et al. "Pattern of meningitis in Al-Ain medical district, United Arab Emirates—a decadal experience (1990–99)." *Journal of Infection* 44.1 (2002): 22-25.
- Mani, R., et al. "Bacteriological profile of community acquired acute bacterial meningitis: a ten-year retrospective study in a tertiary neurocare centre in South India." *Indian Journal of Medical Microbiology* 25.2 (2007): 108.
- Mao, Fei-Fei, et al. "Aetiological spectrum and antibiotic susceptibility pattern of bacterial meningitis in infants and children in Hangzhou, China." *Acta Paediatrica* 94.8 (2005): 1162-1163.
- Mendsaikhan, Jamsran, et al. "Childhood bacterial meningitis in Ulaanbaatar, Mongolia, 2002–2004." *Clinical Infectious Diseases* 48.Supplement 2 (2009): S141-S146.
- Moïsi, J. C., et al. "Enhanced diagnosis of pneumococcal meningitis with use of the Binax NOW immunochromatographic test of *Streptococcus pneumoniae* antigen: a multisite study." *Clinical Infectious Diseases* 48.Supplement 2 (2009): S49-S56.
- Saha, S. K., et al. "The increasing burden of disease in Bangladeshi children due to *Haemophilus influenzae* type b meningitis." *Annals of Tropical Paediatrics* 17.1 (1997): 5-8.
- Sakata, H., et al. "A study of bacterial meningitis in Hokkaido between 1994 and 1998." Kansenshogaku zasshi. *The Journal of the Japanese Association for Infectious Diseases* 74.4 (2000): 339-344.
- Sung, R. Y. T., et al. "Meningitis in Hong Kong children, with special reference to the infrequency of haemophilus and meningococcal infection." *Journal of Paediatrics and Child Health* 33.4 (1997): 296-299.
- Sutinen, J., et al. "Etiology of central nervous system infections in the Philippines and the role of serum C-reactive protein in excluding acute bacterial meningitis." *International Journal of Infectious Diseases* 3.2 (1999): 88-93.
- Yang, Y., et al. "Acute bacterial meningitis in children in Hefei, China 1990-1992." *Chinese Medical Journal* 109.5 (1996): 385-388.
- Zaidi, Anita KM, et al. "Surveillance of pneumococcal meningitis among children in Sindh, southern Pakistan." *Clinical Infectious Diseases* 48.Supplement 2 (2009): S129-S135.

Europe

- Christensen, A. C., et al. "Etiology of bacterial meningitis." Ugeskr Laeger. 1988 Mar 14;150(11):655-657
- Fortnum, H. M., et al. "Epidemiology of bacterial meningitis." Archives of Disease in childhood 68.6 (1993): 763-767.
- Kojouharova, M., et al. "Childhood bacterial meningitis in Bulgaria: a population-based retrospective study in six regions during 1992–1996." International journal of infectious diseases 7.2 (2003): 109-112.
- Kojouharova, M., et al. "Epidemiology of meningitis due to *Haemophilus influenzae* type b in children in Bulgaria: a prospective, population-based surveillance study." *Bulletin of the World Health Organization* 80.9 (2002): 690-695.
- Martínez, L. M., et al. "Bacterial meningitis. Clinical-epidemiological study. Review of 8 years (1988-1995)." *Anales Espanoles de Pediatria* 48.3 (1998): 277-282.
- Namani, S., et al. "Causative pathogens of bacterial meningitis in children and their susceptibility to antibiotics." *JInfects Dis* 9 (2010): 1-6.
- Pizzuti, R., et al. "*Haemophilus influenzae* type b meningitis in the city and district of Naples: a retrospective study on hospitalizations in the period 1987-1995." Annali di igiene: medicina preventiva e di comunità 10.3 (1998): 137.
- Stefanoff, P., et al. "Meningitis and encephalitis in Poland in 2000." *Przeglad Epidemiologiczny* 56.2 (2001): 265-273.
- Stefanoff, P., et al. "Meningitis and encephalitis in Poland in 2004." *Przeglad Epidemiologiczny* 60.3 (2005): 419-428.
- Stefanoff, P., et al. "Meningitis and encephalitis in Poland in 2005." *Przeglad Epidemiologiczny* 61.2 (2006): 225-234.
- Ticca, F., et al. "*Haemophilus influenzae* type b in meningitis: antibiotic resistance in pediatric patients." *Le Infezioni in Medicina: Rivista Periodica di Eziologia, Epidemiologia, Diagnostica, Clinica e Terapia delle Patologie Infettive* 5.2 (1997): 96-99.
- Zabicka, J., et al. "Meningitis and encephalitis in Poland in 1999." *Przeglad Epidemiologiczny* 55.1-2 (2000): 63-73.

Latin America and the Caribbean

- Boehme, C., et al. "Three years of acute bacterial meningitis in the pediatric service at the Temuco Regional Hospital." *Revista Medica de Chile* 121.6 (1993): 633-638.
- Diaz, J. M., et al. "Trends of etiology of acute bacterial meningitis in Chilean children from 1989 to 1998. Impact of the anti-H influenzae type b vaccine." *Revista Medica de Chile* 129.7 (2001): 719-726.
- Dickinson Meneses, Félix Orlando, and Antonio Esteban Pérez Rodríguez. "Meningoencefalitis bacterianas en Cuba." *Revista Cubana de Higiene y Epidemiología* 39.2 (2001): 86-94.
- Dueger, Erica L., Edwin J. Asturias, and Neal A. Halsey. "Culture-and antigen-negative meningitis in Guatemalan children." Revista Panamericana de Salud Pública 24.4 (2008): 248-255.
- Gomez, E., et al. "Population-based surveillance for bacterial meningitis in the Dominican Republic: implications for control by vaccination." Epidemiology and infection 125.03 (2000): 549-554.
- Lucena, R., et al. "Características clínicas e laboratoriais de meningites bacterianas em crianças." (1996).
- Miranzi, S., et al. "*Haemophilus influenzae* type b: epidemiological situation in the State of Minas Gerais, Brazil, 1993-1997." Cadernos de Saúde Pública 19.5 (2003): 1267-1275.
- Nascimento-Carvalho CM, et al. "Etiology of bacterial meningitis in a cohort from Salvador, Bahia Arq Neuropsiquiatr." 1998 Mar;56(1):83-87
- Patiño, R. O., et al. "Meningitis bacteriana aguda en niños: estudio clínico y bacteriológico en el Hospital Infantil de Medellín." *Latreia* 1.2 (1988): 69-76.
- Ribeiro, G. S., et al. "*Haemophilus influenzae* meningitis 5 years after introduction of the *Haemophilus influenzae* type b conjugate vaccine in Brazil." *Vaccine* 25.22 (2007): 4420-4428.

Northern America

- Coant, P. N., et al. "Blood culture results as determinants in the organism identification of bacterial meningitis." *Pediatric Emergency Care* 8.4 (1992): 200-205.
- Walling, A. D., et al. "The epidemiology of bacterial meningitis." *The Journal of the American Board of Family Practice* 4.5 (1991): 307-311.

Oceania
• Hanna, J. N., et al. "Bacterial meningitis in children under five years of age in Western Australia." *Medical Journal of Australia* 155.3 (1991): 160-164.

Table 4: Other indicators used for modeling Hib and pneumococcal disease burden

**Values for**	
**Indicator**	**Source**
Population less than 5 years	United Nations Department of Economic and Social Affairs Population Division. World Population Prospects: The 2015 Revision, DVD Edition. New York: United Nations, 2015.
Child mortality rate	United Nations Interagency Group for Child Mortality Estimation (IGME). 2015 Revision. United Nations, 2015.
Access to care	Derived from Demographic and Health Surveys (http://dhsprogram.com/data/) and Multiple Indicator Cluster Surveys (https://data.unicef.org)
% of children <5 years infected with HIV	Personal communication, UNAIDS[1]
% of deaths in children 1-59 months due to pneumonia	Liu, Li, et al. "Global, regional, and national causes of under-5 mortality in 2000–15: an updated systematic analysis with implications for the Sustainable Development Goals." *The Lancet* 388.10063 (2016): 3027-3035.
% of deaths in children 1-59 months due to meningitis	Liu, Li, et al. "Global, regional, and national causes of under-5 mortality in 2000–15: an updated systematic analysis with implications for the Sustainable Development Goals." *The Lancet* 388.10063 (2017): 3027-3035.
Hib vaccine coverage in 2015	WHO and UNICEF estimates of national immunization coverage.
Pneumococcal conjugate vaccine coverage in 2015	WHO and UNICEF estimates of national immunization coverage.

[1] Contact aidsinfo@unaids.org to request data.

Webappendix 3: Analysis of pneumonia impact studies to inform proportion of pneumonia mortality attributable to *Streptococcus pneumoniae* and *Haemophilus influenzae* type b (Hib)

To supplement the data from the efficacy trials, we identified PCV and Hib vaccine studies published between 1990 to 2014 with non-specific, clinical pneumonia outcomes (e.g., radiography-confirmed pneumonia, pneumonia hospitalizations). We aimed to make similar adjustments to those described before combining with adjusted efficacy and effectiveness estimates in a random effects meta-analysis. Because of herd effects, we assumed vaccine-type efficacy and vaccine coverage to be 100%. We excluded case-control studies without randomization or systematic vaccine distribution. Pre-post impact evaluations with less than 36 months of pre-vaccine introduction data, less than 24 months of post-vaccine introduction data, or unstable background rates of disease were also excluded.

Only one PCV impact study, and no Hib studies, met the inclusion criteria; the PCV study reported on pneumonia hospitalizations in young children in the United States.[4] After adjusting for serotype distribution and Hib vaccine use, the proportion of pneumonia hospitalizations attributable to pneumococcus (i.e., >50%) was assessed as being inconsistent with the other base case data. Advances in analytic methods for vaccine impact data from administrative databases suggest that overestimates of impact may occur.[5] As such, we did not include it in the meta-analysis with other PCV efficacy or effectiveness data. We plan to revisit the opportunity to include these and other similar data in the analysis.

Figure 2: Flow Diagram of PCV Impact studies for Use in Determining the Proportion of Pneumonia Deaths Attributable to Pneumococcus

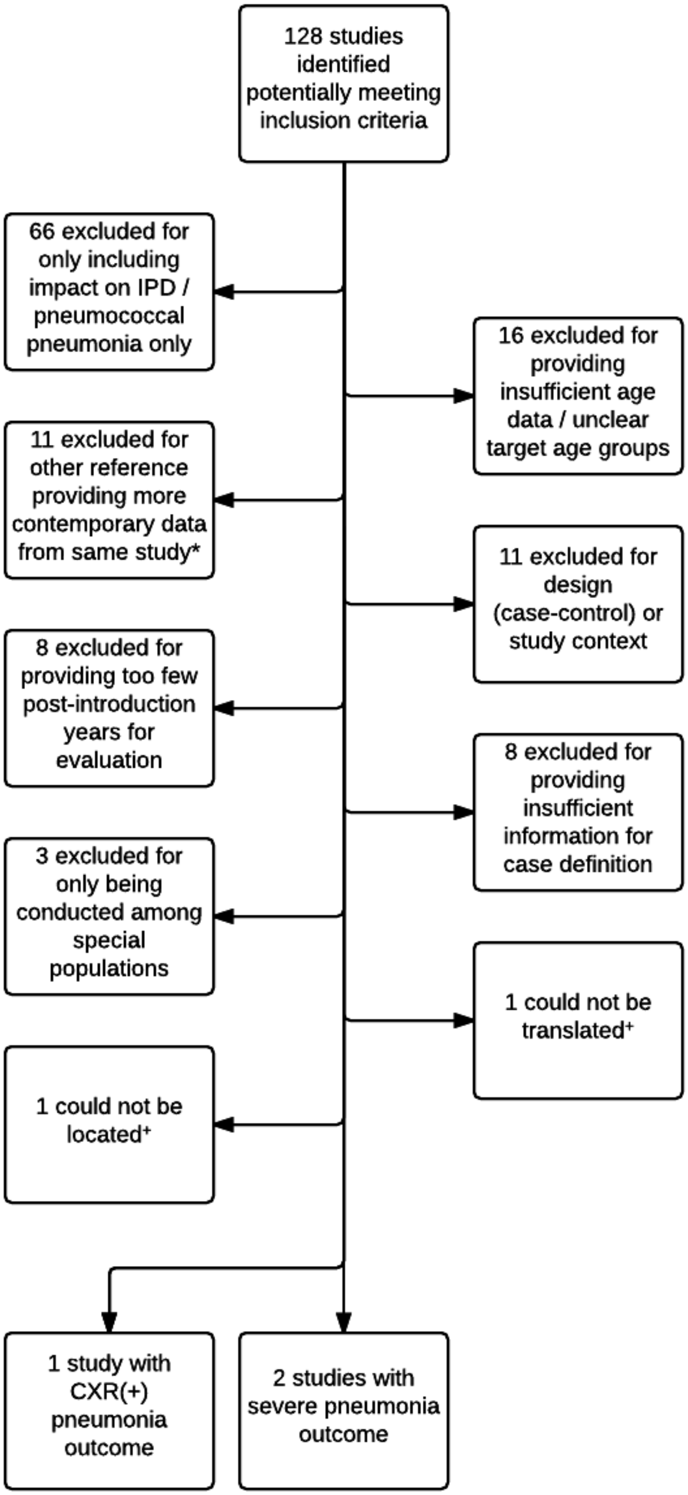

Webappendix 4: Proportion of pneumonia morbidity and mortality attributable to *Streptococcus pneumoniae* and *Haemophilus influenzae* type b

Figure 3: Proportion of radiography-confirmed pneumonia attributable to pneumococcus

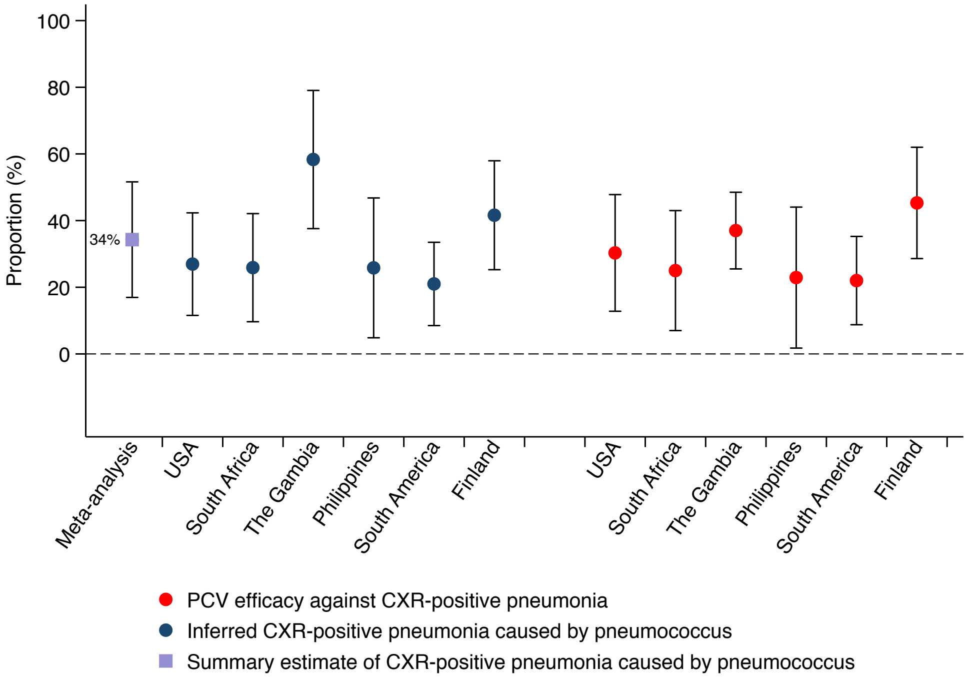

Figure 4: Proportion of severe pneumonia attributable to pneumococcus

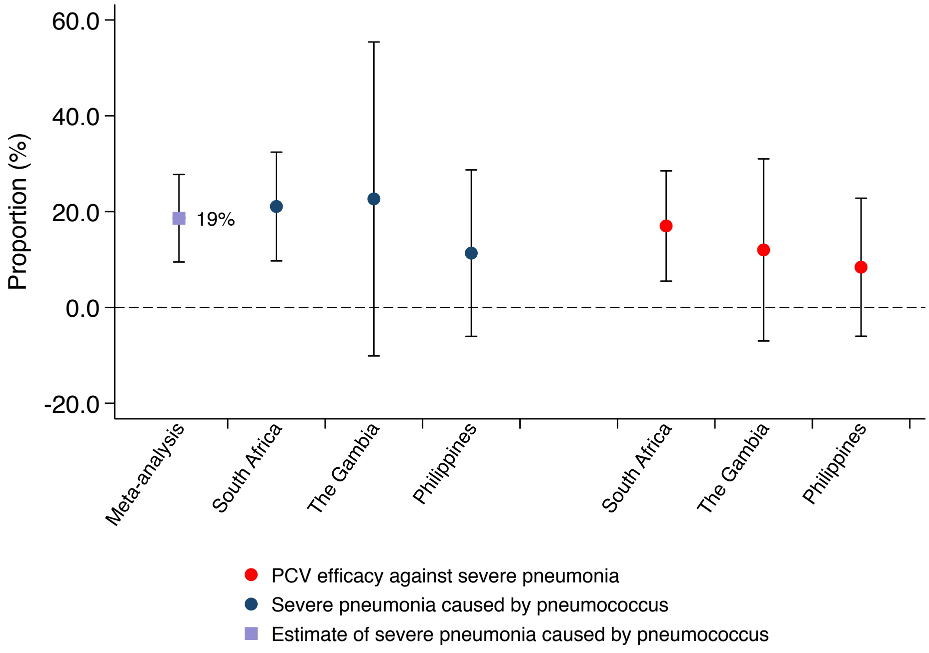

Figure 5: Proportion of clinical pneumonia attributable to pneumococcus

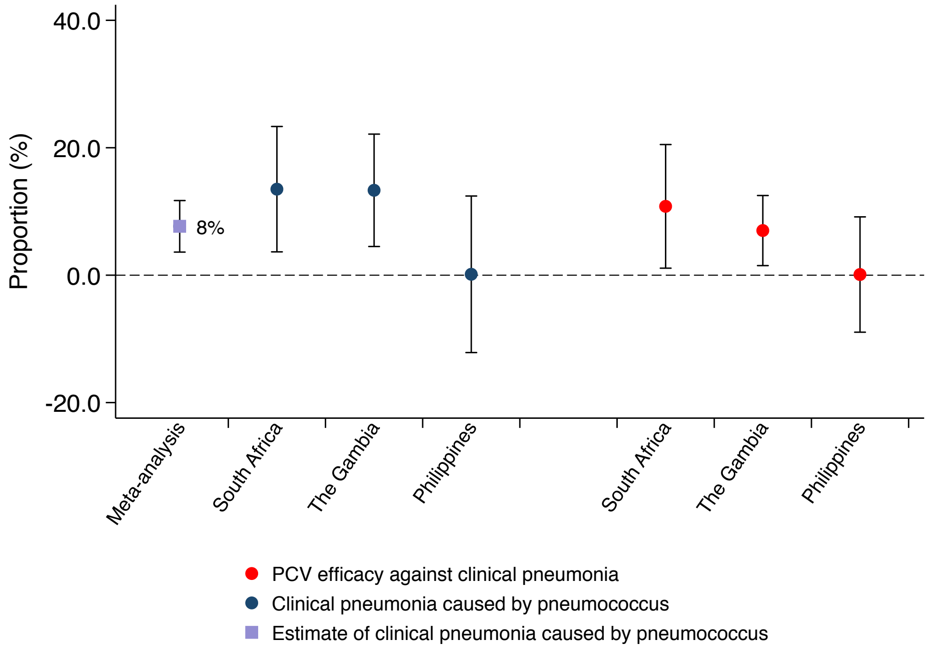

Figure 6: Proportion of radiography-confirmed pneumonia attributable to Hib

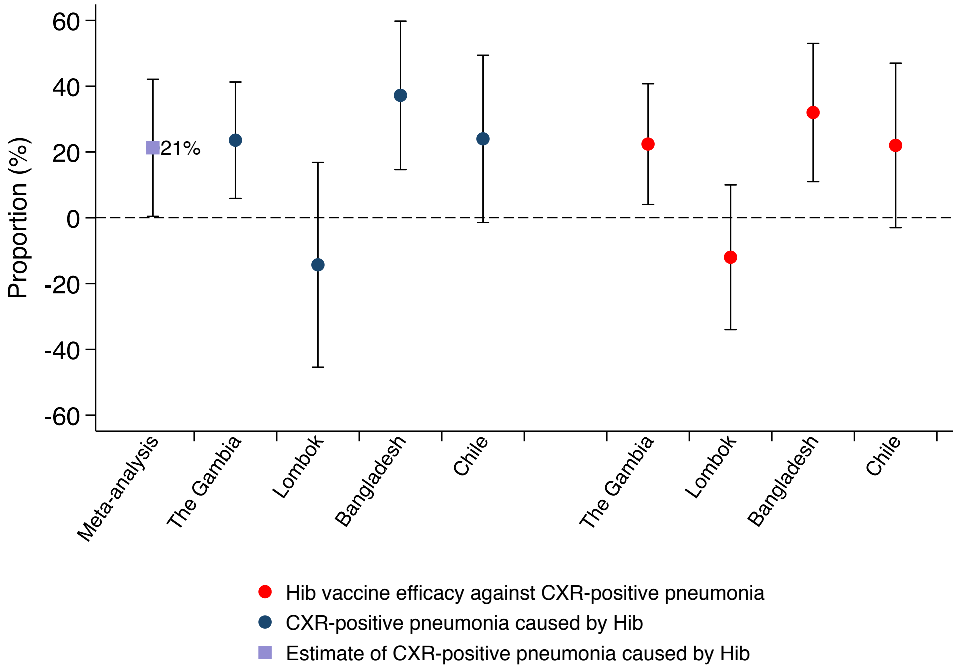

Figure 7: Proportion of severe pneumonia attributable to Hib

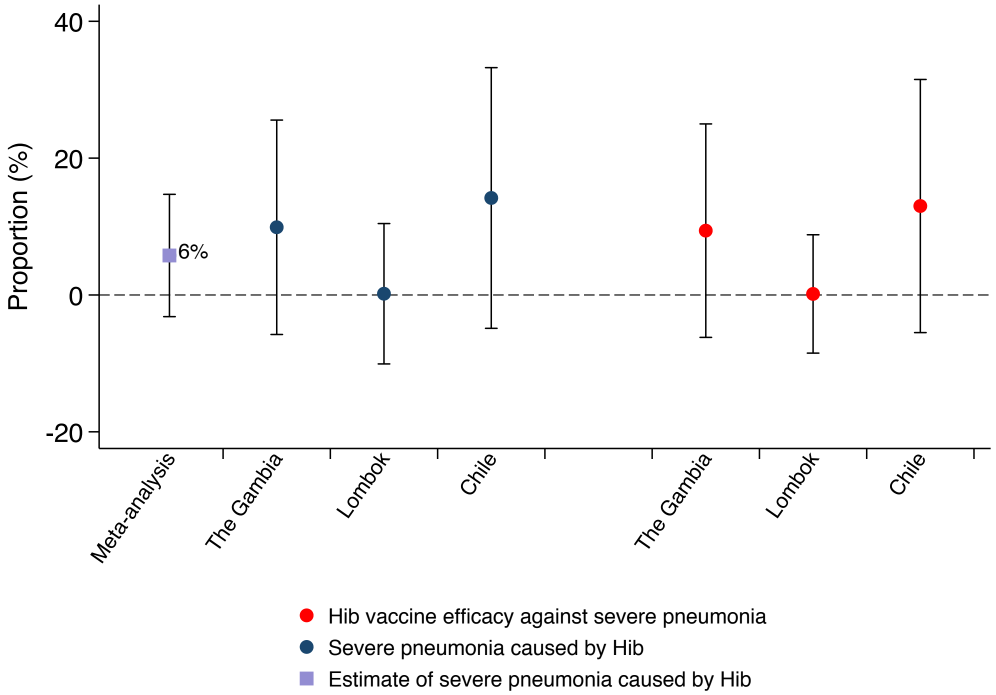

Figure 8: Proportion of clinical pneumonia attributable to Hib

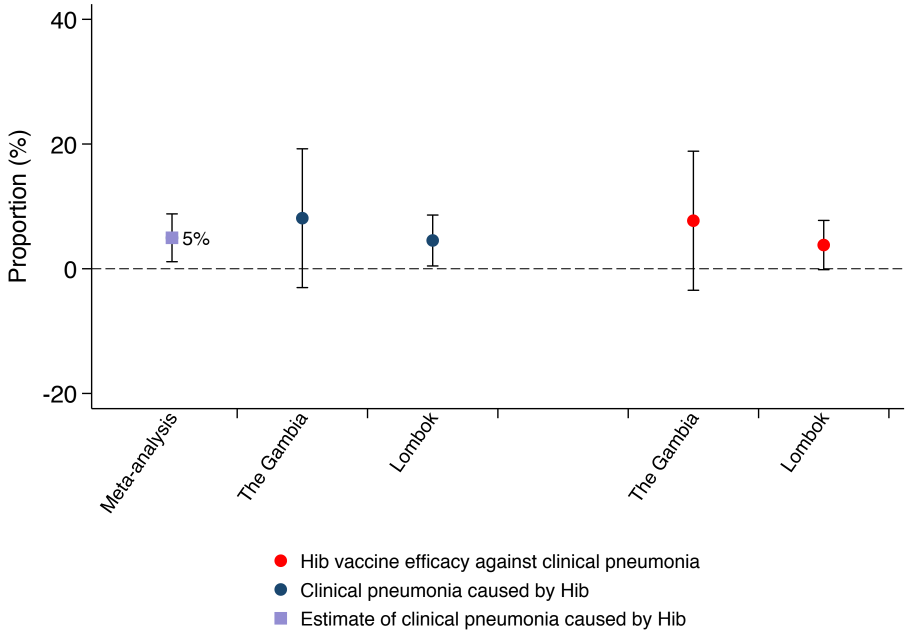

Webappendix 5: Details of sensitivity analysis on proportion of pneumonia deaths attributable to *Streptococcus pneumoniae*

The use of observed efficacy against vaccine-type invasive disease as a proxy for efficacy against vaccine-type pneumonia was a major point of consideration in our updated mortality estimates for pneumococcus. Vaccine-type efficacy against invasive disease almost certainly overestimates vaccine efficacy against vaccine-type pneumonia. As a result, our estimates likely underestimate the burden of pneumococcal pneumonia deaths. We considered three options.

1. A summary measure using observed efficacy against vaccine-type invasive disease to account for incomplete efficacy against vaccine-type pneumonia (i.e., the current base case).
2. A summary measure using observed efficacy against vaccine-type invasive disease, adjusted by the ratio of efficacy against vaccine-type community-acquired pneumococcal pneumonia to efficacy against vaccine-type invasive disease both from a study in elderly Dutch adults that utilizes the urine antigen test, to account for incomplete efficacy against vaccine-type pneumonia
3. A summary estimate using observed efficacy against vaccine-type nasopharyngeal (NP) carriage or vaccine-type acute otitis media (AOM) to account for incomplete efficacy against VT pneumonia

The results of the three adjustments are included in Table 5 below.

Table 5: Estimates of radiography-confirmed pneumonia attributable to pneumococcus in the absence of vaccination using different adjustments to account for incomplete efficacy against VT pneumococcal pneumonia in the absence of vaccination with PCV or Hib vaccine

Approach	Parameters used in adjustment	Adjustment factor for IPD efficacy	Summary estimate
1. Observed efficacy against VT invasive disease to account for incomplete efficacy against VT pneumonia	• N/A	1.000	34% (17 – 52%)
2. Observed efficacy against VT invasive disease, adjusted by ratio of efficacy against VT pneumococcal pneumonia to efficacy against VT invasive disease, to account for incomplete efficacy against VT pneumonia	• 45.6% (21.8-62.5%) for VT pneumococcal pneumonia[6]<br>• 75.0% (41.4-90.8%) for VT IPD[6]	0.608	66% (-7 – 100%)
3. Observed efficacy against VT invasive disease, adjusted by ratio of VT acute otitis media (AOM) efficacy to VT invasive disease efficacy, to account for incomplete efficacy against VT pneumonia	• 57% (50-63%) for VT AOM[7]<br>• 80% (58-90%) for VT IPD[1]	0.713	51% (15 – 87%)
4. Observed efficacy against VT invasive disease, adjusted by ratio of VT nasopharyngeal (NP) carriage efficacy to VT invasive disease efficacy, to account for incomplete efficacy against VT pneumonia	• 57% (50-65%) for VT NP[8]<br>• 80% (58-90%) for VT IPD[1]	0.7125	51% (14 – 89%)

PCV impact studies can provide a unique insight into which adjustment might be more appropriate. This is because through assumed herd effects, there is no need to adjust for imperfect vaccine efficacy—the source of substantial uncertainty in the current estimates. We conducted a review of PCV impact studies with non-specific, clinical pneumonia outcomes in children less than 5 years published between 2000 and 2016. We identified 64 studies with relevant outcomes. Of these, we evaluated those with more than 2 years of post-vaccine introduction data. We used the most contemporary estimates when publications overlapped in time and geography. Results were stratified by age and case definition.

Assuming complete elimination of Hib and complete vaccine-type efficacy stemming from elimination of carried vaccine serotypes, adjustments for serotype distribution were applied to the vaccine impact data. For the distribution of serotypes causing disease, we used estimates of serotypes causing invasive disease. To provide an absolute upper limit, we also adjusted by the distribution of serotypes colonizing the NP from an unpublished meta-analysis. Limiting the analysis to impact studies with all-cause pneumonia hospitalization endpoints in children 0-23 months (i.e., an age group similar to the efficacy and effectiveness studies), the median estimate of pneumonia hospitalizations caused by pneumococcus in the absence of immunization is 38% (range: 13-52%) using the adjustment by the distribution of serotypes causing IPD. The upper limit of pneumonia hospitalizations caused by pneumococcus in the absence of immunization, using an NP adjustment, is 49% (range: 21-62%).

Figure 9: Impact of PCV on all-cause pneumonia hospitalizations stratified by age-group

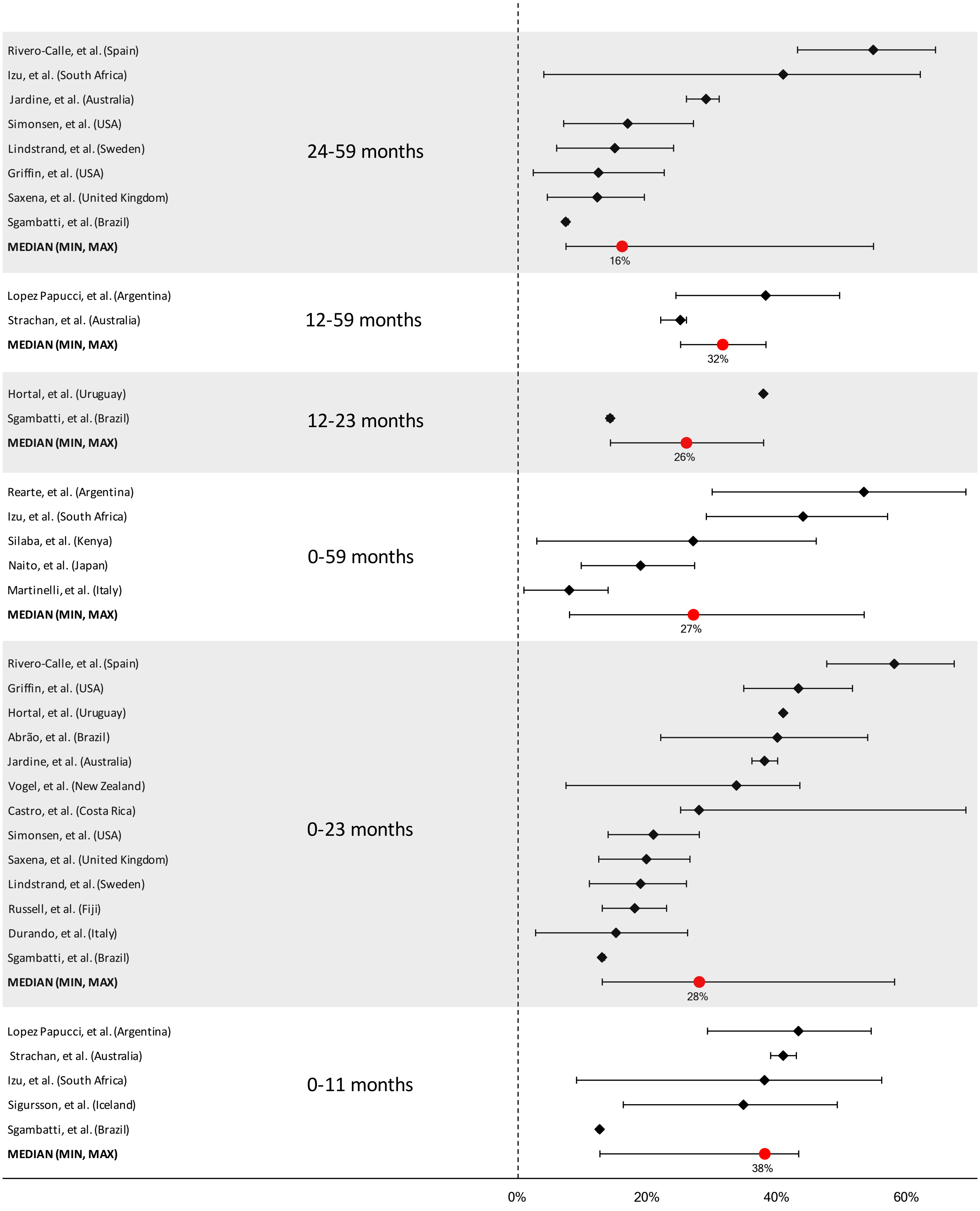

Webappendix 6: Methods used for stratifying pneumonia and meningitis deaths by age (i.e., 1-23 months and 24-59 months) for estimating the burden of Hib disease

We stratified all-cause pneumonia and meningitis deaths by age group (i.e., 1-23 months and 24-59 months) using estimates of total child deaths from IGME for each country.[9] IGME estimates are already stratified by age (i.e., <1 month, <12 months, <59 months). We assumed that half of all deaths in children 12-59 months occurred in children 12-23 months of age. From this estimate, we used the ratio of all deaths in children 1-23 months to all deaths in children 24-59 months and assumed the same distribution for pneumonia and meningitis deaths. To estimate Hib deaths due to pneumonia and meningitis, we applied estimates of the proportion of pneumonia and meningitis due to Hib to pneumonia and meningitis deaths in children 1-23 months.

Webappendix 7: Methods used to estimate the proportion of meningitis deaths attributable to each pathogen.

No observational studies were identified that reported on the proportion of meningitis deaths due to each pathogen. We therefore used data from studies reporting on the distribution of meningitis cases and adjusted these estimates by the relative pathogen-specific meningitis CFR to calculate the proportion of meningitis deaths due to pneumococcus and Hib. We constrained the total contribution of the most common causes of bacterial meningitis (i.e., pneumococcus, Hib, and *Neisseria meningitidis*) to all-cause meningitis cases and deaths to 83% based on an analysis of studies reporting on multiple meningitis etiologies using diagnostic techniques with equivalent sensitivity for all pathogens. To derive the proportion of all-cause meningitis deaths attributable to pathogen x, we used the following equation

$$\%\, Meningitis\ deaths_x = \frac{Meningitis\ CFR_x \times \%\, Meningitis\ cases_x}{\sum_i^z (Meningitis\ CFR_i \times \%\, Meningitis\ cases_i)}$$

where $Meningitis\ CFR_x$ is the case fatality ratio (CFR) for pathogen x, $\%\, Meningitis\ cases_x$ is the proportion of meningitis cases attributable to pathogen x, and the denominator on the right-hand side of the equation is the sum of the products for each pathogen.

Webappendix 8: Equation used to account for increased risk of Hib and pneumococcal disease in children infected with HIV

We used the following equation to prepare annual mortality and morbidity estimates due to each syndrome (i.e., pneumonia, meningitis, and NPNM) in HIV-infected children for each pathogen

$$HIV^{+}\ deaths_{pathogen,syndrome}$$

$$= \frac{HIV\ \%\ \times\ Population_{1-59\ mos.}\ \times\ HIV^{-}\ deaths_{pathogen,syndrome}\ \times\ ID\ Relative\ Risk_{pathogen}}{(1 - HIV\ \%)\ \times\ Population_{1-59\ mos.}}$$

Where $HIV\ \%$ represents the proportion of children infected with HIV and not on HAART, $Population_{1-59\ mos.}$ is the total population of children 1-59 months, $HIV^{-}\ deaths_{pathogen,syndrome}$ is the estimated number of deaths due to the pathogen by syndrome, and $ID\ Relative\ Risk_{pathogen}$ is the relative risk of invasive disease for pneumococcus (40.96) or Hib (7.39) in children infected with HIV.

Webappendix 9: External review members

<u>World Health Organization (WHO) Immunization and Vaccines related Implementation Research Advisory Committee (IVIR-AC) members</u>

- Mary Amuyunzu-Nyamongo, Executive Director and co-founder, African Institute for Health and Development (AIHD), Nairobi, Kenya
- Philippe Beutels, Associate Professor, Health Economics, Health Economics and Modeling Infectious Diseases Unit, University of Antwerp, Belgium
- Robert F. Breiman (Chair), Director, Emory Global Health Institute, Emory University, Atlanta, USA
- Marc Brisson, Associate Professor, Department of social and preventive medicine, Faculty of Medicine, Laval University, Canada
- Donald Burke, Dean of the Graduate School of Public Health and UPMC Jonas Salk Chair of Global Health, University of Pittsburgh, USA
- Rachel Feilden, FBA Health System Analysts, Bath Somerset, UK
- Brad Gessner, Scientific Director, Association pour la Médicine Préventive (AMP), Paris, France
- Gagandeep Kang, Head, Department of Gastrointestinal Sciences, Christian Medical College, Vellore, India
- Peter McIntyre, Director, National Centre for Immunization Research & Surveillance, University of Sydney, Australia
- Wilfred Ndifon, Chair, Career Development Research, African Institute for Mathematical, Sciences, Cape Town, South Africa
- Yot Teerawattananon, Founding Leader of Health Intervention and Technology Assessment Program & Senior Researcher Scholar of Thailand's Research Fund, Ministry of Public Health, Thailand
- Mitchell Weiss, Professor & Head, Swiss Tropical & Public Health Institute, Basel, Switzerland

<u>External Expert Review Committee</u>

- Philippe Beutels, Associate Professor, Health Economics, Health Economics and Modeling Infectious Diseases Unit, University of Antwerp, Belgium
- Robert F. Breiman, Director, Emory Global Health Institute, Emory University, Atlanta, USA
- Claire Broome, Consultant, USA
- Harry Campbell, Professor, Usher Institute of Population Health Sciences and Informatics, Medical School, The University of Edinburgh, Scotland, UK
- Thomas Cherian, Coordinator, Expanded Programme on Immunization, Department of Immunization, Vaccines and Biologicals, World Health Organization, Geneva, Switzerland
- Brad Gessner, Scientific Director, Association pour la Médicine Préventive (AMP), Paris, France

- Gagandeep Kang, Head, Department of Gastrointestinal Sciences, Christian Medical College, Vellore, India
- Keith Klugman, Director, Pneumonia, Bill & Melinda Gates Foundation, Seattle, USA
- Shabir Madhi, Executive Director, National Institute for Communicable Diseases, National Health Laboratory Service, Johannesburg, South Africa
- Kim Mulholland, Professor, Murdoch Childrens Research Institute, Victoria, Australia
- Samir Saha, Senior Consultant, Department of Microbiology, Dhaka Shishu Hospital, Dhaka, Bangladesh
- Cynthia Whitney, Chief of the Respiratory Diseases Branch, National Center for Immunization and Respiratory Diseases, Centers for Disease Control and Prevention, Atlanta, USA

Webappendix 10: GATHER checklist

Table 6: GATHER Checklist

GATHER checklist item	Section(s) or sources providing information
**Objectives and funding**	
1. Define the indicators, populations, and time periods for which estimates were made.	Indicators, populations, and time periods provided in methods.
2. List the funding sources for the work.	Funding source provided in summary.
**Data inputs**	
3. Describe how the data were identified and how the data were accessed.	Data identification and collection approaches provided in "data sources" section of methods and webappendix.
4. Specify the inclusion and exclusion criteria. Identify all ad-hoc exclusions.	Inclusion and exclusion criteria provided in webappendix and previous publications.[2,10]
5. Provide information about all included data sources and their main characteristics. For each data source used, report reference information or contact name/institution, population represented, data collection method, years of data collection, sex and age range, diagnostic criteria or measurement method, and sample size, as relevant.	Data sources and characteristics provided in online open access database.
6. Identify and describe any categories of input data that have potentially important biases (e.g., based on characteristics listed in item 5).	Potentially important biases of input data provided in discussion and webappendix.
7. Describe and give sources for any other data inputs.	Other sources of data described in methods and webappendix and provided in online open access database when appropriate.
8. Provide all data inputs in a file format from which data can be efficiently extracted, including all relevant meta-data listed in item 5. For any data inputs that cannot be shared because of ethical or legal reasons, such as third-party ownership, provide a contact name or the name of the institution that retains the right to the data.	Data inputs available in Excel spreadsheet in online open access database when appropriate. Data inputs that cannot be shared are described in webappendix with institutional contacts for potentially obtaining these data.
**Data analysis**	
9. Provide a conceptual overview of the data analysis method. A diagram may be helpful.	Conceptual model provided in Figure 1 of manuscript.
10. Provide a detailed description of all steps of the analysis, including mathematical formulae. This description should cover, as relevant, data cleaning, data pre-processing, data adjustments and weighting of data sources, and mathematical or statistical models.	Detailed description of analysis provided in methods, appendix, and previous publications.[10-12]
11. Describe how candidate models were evaluated and how the final models were selected.	Discussion of candidate model considerations provided in methods and webappendix.
12. Provide the results of an evaluation of model performance, if done, as well as the results of any relevant sensitivity analysis.	Results of model performance at country-level provided in results and webappendix.
13. Describe methods of calculating uncertainty of the estimates. State which sources of uncertainty were, and were not, accounted for in the uncertainty analysis.	Uncertainty methods and sources provided in methods.
14. State how analytic or statistical source code used to generate estimates can be accessed.	Access to source code provided in online open access database.
**Results and discussion**	
15. Provide published estimates in a file format from which data can be efficiently extracted.	Results in Excel spreadsheet provided in online open access database.
16. Report a quantitative measure of the uncertainty of the estimates.	Uncertainty intervals provided with all results in manuscript, webappendix, and online open access database.
17. Interpret results in light of existing evidence. If updating a previous set of estimates, describe the reasons for changes in estimates.	Discussion of results relevant to existing research provided in research in context, results, and discussion.
18. Discuss limitations of the estimates. Include a discussion of any modelling assumptions or data limitations that affect interpretation of the estimates.	Limitations of data and models provided in discussion.

Table 7: *Streptococcus pneumoniae* mortality by syndrome, country, and year

		Pneumonia deaths				Meningitis deaths				NPNM deaths			
Year	Country	Spn pneumonia deaths (UR)	Spn pneumonia deaths, HIV (UR)	Spn pneumonia mortality rate (UR)	Spn pneumonia mortality rate, HIV (UR)	Spn meningitis deaths (UR)	Spn meningitis deaths, HIV (UR)	Spn meningitis mortality rate (UR)	Spn meningitis mortality rate, HIV (UR)	Spn NPNM deaths (UR)	Spn NPNM deaths, HIV (UR)	Spn NPNM mortality rate (UR)	Spn NPNM mortality rate, HIV (UR)
2000	Afghanistan	8257 (5855-8605)		213 (151-222)		1588 (638-3366)		41 (16-87)		499 (200-1058)		13 (5-27)	
2001	Afghanistan	8415 (5967-8769)		209 (148-218)		1617 (652-3421)		40 (16-85)		508 (205-1076)		13 (5-27)	
2002	Afghanistan	8520 (6041-8879)		203 (144-212)		1622 (658-3425)		39 (16-82)		510 (207-1077)		12 (5-26)	
2003	Afghanistan	8598 (6097-8960)		198 (140-206)		1623 (661-3418)		37 (15-79)		510 (208-1074)		12 (5-25)	
2004	Afghanistan	8603 (6101-8966)		192 (136-200)		1609 (659-3378)		36 (15-75)		506 (207-1062)		11 (5-24)	
2005	Afghanistan	8534 (6051-8894)		186 (132-194)		1579 (650-3307)		34 (14-72)		496 (204-1040)		11 (4-23)	
2006	Afghanistan	8493 (6022-8851)		181 (128-188)		1553 (642-3244)		33 (14-69)		488 (202-1020)		10 (4-22)	
2007	Afghanistan	8394 (5952-8747)		175 (124-183)		1511 (628-3147)		32 (13-66)		475 (197-989)		10 (4-21)	
2008	Afghanistan	8267 (5862-8616)		171 (121-178)		1462 (610-3039)		30 (13-63)		460 (192-955)		9 (4-20)	
2009	Afghanistan	8113 (5753-8455)		166 (118-173)		1407 (590-2916)		29 (12-60)		442 (185-917)		9 (4-19)	
2010	Afghanistan	7920 (5616-8254)		161 (114-167)		1348 (568-2789)		27 (12-57)		424 (178-877)		9 (4-18)	
2011	Afghanistan	7663 (5434-7986)		155 (110-162)		1286 (541-2658)		26 (11-54)		404 (170-836)		8 (3-17)	
2012	Afghanistan	7369 (5225-7679)		149 (106-156)		1223 (517-2522)		25 (10-51)		385 (163-793)		8 (3-16)	
2013	Afghanistan	7046 (4996-7343)		143 (102-150)		1164 (495-2395)		24 (10-49)		366 (156-753)		7 (3-15)	
2014	Afghanistan	5496 (3897-5728)		113 (80-117)		880 (376-1806)		18 (8-37)		277 (118-568)		6 (2-12)	
2015	Afghanistan	4199 (2978-4376)	7 (5-8)	86 (61-90)	0 (0-0)	667 (286-1366)	1 (1-2)	14 (6-28)	0 (0-0)	210 (90-429)	0 (0-1)	4 (2-9)	0 (0-0)
2000	Albania	72 (51-75)		26 (19-27)		5 (2-19)		2 (1-7)		5 (2-17)		2 (1-6)	
2001	Albania	62 (44-65)		24 (17-25)		4 (1-17)		2 (0-7)		4 (1-16)		1 (0-6)	
2002	Albania	53 (38-55)		21 (15-22)		4 (1-16)		1 (0-6)		3 (1-14)		1 (0-6)	
2003	Albania	46 (33-48)		19 (14-20)		3 (1-14)		1 (0-6)		3 (1-13)		1 (0-5)	
2004	Albania	40 (28-42)		18 (13-19)		3 (0-13)		1 (0-6)		2 (0-11)		1 (0-5)	
2005	Albania	35 (25-36)		17 (12-17)		2 (0-11)		1 (0-5)		2 (0-10)		1 (0-5)	
2006	Albania	30 (21-31)		15 (11-16)		2 (0-10)		1 (0-5)		2 (0-9)		1 (0-4)	
2007	Albania	26 (19-28)		14 (10-15)		2 (0-8)		1 (0-4)		2 (0-7)		1 (0-4)	
2008	Albania	23 (16-24)		14 (10-14)		2 (0-7)		1 (0-4)		1 (0-6)		1 (0-4)	
2009	Albania	23 (17-24)		15 (10-15)		3 (1-11)		2 (0-7)		2 (1-10)		1 (0-6)	
2010	Albania	23 (16-24)		15 (10-15)		2 (1-11)		2 (0-7)		2 (1-9)		1 (0-6)	
2011	Albania	14 (10-15)		9 (6-10)		1 (0-6)		1 (0-4)		1 (0-5)		1 (0-3)	
2012	Albania	12 (8-12)		7 (5-8)		1 (0-5)		1 (0-3)		1 (0-4)		1 (0-3)	
2013	Albania	11 (8-11)		6 (5-7)		1 (0-4)		1 (0-3)		1 (0-4)		1 (0-2)	
2014	Albania	11 (8-12)		6 (4-7)		1 (0-4)		1 (0-2)		1 (0-4)		1 (0-2)	
2015	Albania	9 (6-9)	0 (0-0)	5 (3-5)	0 (0-0)	1 (0-3)	0 (0-0)	0 (0-2)	0 (0-0)	1 (0-3)	0 (0-0)	0 (0-2)	0 (0-0)
2000	Algeria	1054 (747-1098)		35 (25-36)		107 (38-233)		4 (1-8)		96 (34-208)		3 (1-7)	
2001	Algeria	1007 (714-1049)		35 (25-36)		102 (36-221)		3 (1-8)		91 (32-197)		3 (1-7)	
2002	Algeria	964 (684-1005)		34 (24-35)		97 (35-209)		3 (1-7)		86 (31-186)		3 (1-7)	
2003	Algeria	932 (661-972)		33 (23-34)		92 (33-200)		3 (1-7)		82 (30-178)		3 (1-6)	
2004	Algeria	911 (646-949)		32 (23-33)		90 (32-194)		3 (1-7)		80 (29-172)		3 (1-6)	
2005	Algeria	894 (634-932)		31 (22-32)		88 (32-189)		3 (1-6)		78 (28-168)		3 (1-6)	
2006	Algeria	901 (639-939)		30 (21-31)		88 (32-190)		3 (1-6)		79 (28-169)		3 (1-6)	
2007	Algeria	908 (644-947)		28 (20-30)		89 (33-190)		3 (1-6)		79 (29-170)		2 (1-5)	
2008	Algeria	930 (659-969)		28 (20-29)		91 (25-231)		3 (1-7)		81 (22-205)		2 (1-6)	
2009	Algeria	941 (667-980)		26 (19-27)		93 (26-232)		3 (1-6)		83 (23-207)		2 (1-6)	
2010	Algeria	950 (674-990)		25 (18-26)		94 (27-233)		2 (1-6)		84 (24-208)		2 (1-5)	
2011	Algeria	956 (678-997)		24 (17-25)		95 (29-234)		2 (1-6)		85 (25-208)		2 (1-5)	
2012	Algeria	969 (687-1010)		23 (16-24)		97 (30-235)		2 (1-6)		86 (27-209)		2 (1-5)	
2013	Algeria	978 (694-1019)		22 (16-23)		98 (31-237)		2 (1-5)		87 (27-211)		2 (1-5)	
2014	Algeria	974 (691-1015)		22 (16-23)		97 (30-235)		2 (1-5)		87 (27-210)		2 (1-5)	
2015	Algeria	972 (689-1013)	1 (1-1)	22 (15-22)	0 (0-0)	97 (30-234)	0 (0-0)	2 (1-5)	0 (0-0)	87 (27-209)	0 (0-0)	2 (1-5)	0 (0-0)
2000	Andorra	0 (0-0)		1 (1-1)		0 (0-0)		0 (0-1)		0 (0-0)		0 (0-0)	
2001	Andorra	0 (0-0)		2 (1-2)		0 (0-0)		1 (0-1)		0 (0-0)		1 (0-1)	
2002	Andorra	0 (0-0)		2 (1-2)		0 (0-0)		1 (0-1)		0 (0-0)		1 (0-1)	
2003	Andorra	0 (0-0)		2 (1-2)		0 (0-0)		1 (0-1)		0 (0-0)		1 (0-1)	
2004	Andorra	0 (0-0)		2 (1-2)		0 (0-0)		1 (0-1)		0 (0-0)		1 (0-1)	
2005	Andorra	0 (0-0)		2 (1-2)		0 (0-0)		1 (0-1)		0 (0-0)		0 (0-1)	
2006	Andorra	0 (0-0)		1 (1-1)		0 (0-0)		0 (0-0)		0 (0-0)		0 (0-0)	
2007	Andorra	0 (0-0)		1 (1-1)		0 (0-0)		0 (0-0)		0 (0-0)		0 (0-0)	
2008	Andorra	0 (0-0)		0 (0-0)		0 (0-0)		0 (0-0)		0 (0-0)		0 (0-0)	
2009	Andorra	0 (0-0)		0 (0-0)		0 (0-0)		0 (0-0)		0 (0-0)		0 (0-0)	
2010	Andorra	0 (0-0)		0 (0-0)		0 (0-0)		0 (0-0)		0 (0-0)		0 (0-0)	
2011	Andorra	0 (0-0)		0 (0-0)		0 (0-0)		0 (0-0)		0 (0-0)		0 (0-0)	
2012	Andorra	0 (0-0)		0 (0-0)		0 (0-0)		0 (0-0)		0 (0-0)		0 (0-0)	
2013	Andorra	0 (0-0)		0 (0-0)		0 (0-0)		0 (0-0)		0 (0-0)		0 (0-0)	
2014	Andorra	0 (0-0)		0 (0-0)		0 (0-0)		0 (0-0)		0 (0-0)		0 (0-0)	
2015	Andorra	0 (0-0)	0 (0-0)	0 (0-0)	0 (0-0)	0 (0-0)	0 (0-0)	0 (0-0)	0 (0-0)	0 (0-0)	0 (0-0)	0 (0-0)	0 (0-0)
2000	Angola	9355 (6633-9749)		319 (226-332)		1305 (688-2497)		44 (23-85)		410 (216-785)		14 (7-27)	
2001	Angola	9584 (6796-9988)		314 (223-328)		1322 (697-2528)		43 (23-83)		416 (219-795)		14 (7-26)	
2002	Angola	10074 (7143-10499)		319 (226-332)		1369 (723-2616)		43 (23-83)		430 (227-822)		14 (7-26)	
2003	Angola	10368 (7352-10806)		317 (225-331)		1381 (730-2637)		42 (22-81)		434 (229-829)		13 (7-25)	
2004	Angola	10743 (7618-11196)		318 (225-331)		1393 (737-2657)		41 (22-79)		438 (232-835)		13 (7-25)	
2005	Angola	11195 (7938-11667)		320 (227-333)		1418 (751-2703)		41 (21-77)		446 (236-850)		13 (7-24)	
2006	Angola	11687 (8287-12179)		322 (228-335)		1444 (766-2750)		40 (21-76)		454 (241-865)		13 (7-24)	
2007	Angola	12096 (8577-12606)		322 (229-336)		1464 (778-2783)		39 (21-74)		460 (245-875)		12 (7-23)	
2008	Angola	12432 (8815-12956)		322 (228-335)		1484 (790-2816)		38 (20-73)		467 (248-885)		12 (6-23)	
2009	Angola	12606 (8939-13137)		317 (225-330)		1487 (793-2817)		37 (20-71)		467 (249-886)		12 (6-22)	
2010	Angola	12681 (8992-13216)		310 (220-323)		1491 (797-2819)		36 (19-69)		469 (250-886)		11 (6-22)	
2011	Angola	12611 (8943-13143)		299 (212-312)		1484 (795-2801)		35 (19-66)		467 (250-880)		11 (6-21)	
2012	Angola	12462 (8837-12988)		288 (204-300)		1467 (788-2763)		34 (18-64)		461 (248-869)		11 (6-20)	
2013	Angola	11867 (8415-12368)		268 (190-279)		1387 (747-2608)		31 (17-59)		436 (235-820)		10 (5-19)	
2014	Angola	10033 (7115-10456)		221 (157-231)		1145 (618-2149)		25 (14-47)		360 (194-676)		8 (4-15)	
2015	Angola	8704 (6172-9071)	1161 (824-1210)	188 (133-196)	25 (18-26)	984 (533-1844)	131 (71-246)	21 (11-40)	3 (2-5)	310 (168-580)	41 (22-77)	7 (4-12)	1 (0-2)
2000	Antigua and Barbuda	0 (0-0)		0 (0-0)		0 (0-0)		2 (1-4)		0 (0-0)		2 (1-3)	
2001	Antigua and Barbuda	0 (0-0)		3 (2-3)		0 (0-1)		6 (3-10)		0 (0-1)		5 (3-9)	
2002	Antigua and Barbuda	0 (0-0)		3 (2-4)		1 (0-1)		7 (3-11)		0 (0-1)		6 (3-9)	
2003	Antigua and Barbuda	0 (0-0)		5 (4-5)		0 (0-1)		4 (2-7)		0 (0-0)		4 (2-6)	
2004	Antigua and Barbuda	0 (0-0)		5 (3-5)		0 (0-0)		3 (2-5)		0 (0-0)		3 (1-5)	
2005	Antigua and Barbuda	0 (0-0)		5 (3-5)		0 (0-0)		4 (2-6)		0 (0-0)		3 (2-5)	
2006	Antigua and Barbuda	0 (0-0)		4 (3-4)		0 (0-0)		3 (2-5)		0 (0-0)		3 (1-5)	

Spn = *Streptococcus pneumoniae*; npnm = non-pneumonia, non-meningitis; HIV = deaths only in HIV-infected children; Uncertainty range provided in parentheses.

Year	Country	Spn pneumonia deaths (UR)	Spn pneumonia deaths, HIV (UR)	Spn pneumonia mortality rate (UR)	Spn pneumonia mortality rate, HIV (UR)	Spn meningitis deaths (UR)	Spn meningitis deaths, HIV (UR)	Spn meningitis mortality rate (UR)	Spn meningitis mortality rate, HIV (UR)	Spn NPNM deaths (UR)	Spn NPNM deaths, HIV (UR)	Spn NPNM mortality rate (UR)	Spn NPNM mortality rate, HIV (UR)
2007	Antigua and Barbuda	0 (0-0)		4 (3-4)		0 (0-0)		0 (0-0)		0 (0-0)		0 (0-0)	
2008	Antigua and Barbuda	0 (0-0)		4 (3-4)		0 (0-0)		0 (0-0)		0 (0-0)		0 (0-0)	
2009	Antigua and Barbuda	0 (0-0)		4 (3-4)		0 (0-0)		0 (0-0)		0 (0-0)		0 (0-0)	
2010	Antigua and Barbuda	0 (0-0)		3 (2-3)		0 (0-0)		0 (0-0)		0 (0-0)		0 (0-0)	
2011	Antigua and Barbuda	0 (0-0)		2 (2-2)		0 (0-0)		0 (0-0)		0 (0-0)		0 (0-0)	
2012	Antigua and Barbuda	0 (0-0)		2 (2-2)		0 (0-0)		0 (0-0)		0 (0-0)		0 (0-0)	
2013	Antigua and Barbuda	0 (0-0)		2 (1-2)		0 (0-0)		0 (0-0)		0 (0-0)		0 (0-0)	
2014	Antigua and Barbuda	0 (0-0)		2 (2-2)		0 (0-0)		0 (0-0)		0 (0-0)		0 (0-0)	
2015	Antigua and Barbuda	0 (0-0)	0 (0-0)	2 (2-2)	0 (0-0)	0 (0-0)	0 (0-0)	0 (0-0)	0 (0-0)	0 (0-0)	0 (0-0)	0 (0-0)	0 (0-0)
2000	Argentina	350 (248-365)		10 (7-11)		98 (44-201)		3 (1-6)		87 (39-179)		3 (1-5)	
2001	Argentina	363 (258-378)		11 (8-11)		88 (40-181)		3 (1-5)		79 (35-161)		2 (1-5)	
2002	Argentina	349 (248-364)		10 (7-11)		96 (43-195)		3 (1-6)		85 (38-174)		2 (1-5)	
2003	Argentina	549 (390-572)		16 (11-17)		113 (51-229)		3 (1-7)		100 (45-204)		3 (1-6)	
2004	Argentina	398 (282-414)		11 (8-12)		114 (52-231)		3 (1-7)		101 (46-205)		3 (1-6)	
2005	Argentina	320 (227-334)		9 (6-9)		102 (47-206)		3 (1-6)		91 (42-184)		3 (1-5)	
2006	Argentina	340 (241-355)		10 (7-10)		98 (45-197)		3 (1-5)		87 (40-175)		2 (1-5)	
2007	Argentina	398 (283-415)		11 (8-12)		82 (38-164)		2 (1-5)		73 (33-146)		2 (1-4)	
2008	Argentina	342 (243-356)		10 (7-10)		84 (39-167)		2 (1-5)		74 (34-149)		2 (1-4)	
2009	Argentina	418 (296-435)		12 (8-12)		81 (37-161)		2 (1-5)		72 (33-144)		2 (1-4)	
2010	Argentina	309 (219-322)		9 (6-9)		90 (42-178)		3 (1-5)		80 (37-159)		2 (1-4)	
2011	Argentina	314 (223-328)		9 (6-9)		66 (31-131)		2 (1-4)		59 (27-116)		2 (1-3)	
2012	Argentina	238 (168-248)		7 (5-7)		62 (29-123)		2 (1-3)		55 (26-109)		2 (1-3)	
2013	Argentina	190 (135-198)		5 (4-5)		42 (19-82)		1 (1-2)		37 (17-73)		1 (0-2)	
2014	Argentina	163 (115-169)		4 (3-5)		37 (18-74)		1 (0-2)		33 (16-66)		1 (0-2)	
2015	Argentina	154 (109-161)	1 (1-1)	4 (3-4)	0 (0-0)	36 (17-72)	0 (0-0)	1 (0-2)	0 (0-0)	32 (15-64)	0 (0-0)	1 (0-2)	0 (0-0)
2000	Armenia	52 (37-54)		27 (19-28)		7 (2-16)		3 (1-8)		6 (2-14)		3 (1-7)	
2001	Armenia	49 (35-51)		26 (19-28)		6 (1-18)		3 (1-10)		6 (1-16)		3 (1-9)	
2002	Armenia	46 (33-48)		25 (18-26)		6 (1-17)		3 (1-9)		5 (1-15)		3 (1-8)	
2003	Armenia	45 (32-46)		24 (17-25)		6 (1-17)		3 (1-9)		5 (1-15)		3 (1-8)	
2004	Armenia	43 (30-44)		22 (16-23)		6 (1-16)		3 (1-8)		5 (1-14)		3 (0-8)	
2005	Armenia	40 (28-42)		20 (15-21)		6 (1-15)		3 (1-8)		5 (1-14)		3 (0-7)	
2006	Armenia	37 (26-39)		18 (13-19)		5 (1-15)		3 (1-7)		5 (1-13)		2 (0-6)	
2007	Armenia	35 (25-36)		17 (12-17)		5 (1-14)		2 (0-7)		4 (1-12)		2 (0-6)	
2008	Armenia	32 (22-33)		15 (11-16)		5 (1-12)		2 (0-6)		4 (1-11)		2 (0-5)	
2009	Armenia	29 (21-30)		14 (10-14)		4 (1-11)		2 (0-5)		4 (1-10)		2 (0-5)	
2010	Armenia	28 (20-29)		13 (9-14)		6 (1-15)		3 (1-7)		5 (1-14)		2 (1-6)	
2011	Armenia	27 (19-29)		13 (9-14)		7 (2-19)		3 (1-9)		7 (2-17)		3 (1-8)	
2012	Armenia	25 (18-26)		12 (8-12)		7 (2-17)		3 (1-8)		6 (2-15)		3 (1-7)	
2013	Armenia	22 (16-23)		11 (8-11)		6 (2-15)		3 (1-7)		5 (2-13)		3 (1-6)	
2014	Armenia	20 (14-21)		10 (7-10)		5 (2-13)		3 (1-6)		5 (2-12)		2 (1-6)	
2015	Armenia	16 (12-17)	0 (0-0)	8 (6-8)	0 (0-0)	4 (2-10)	0 (0-0)	2 (1-5)	0 (0-0)	4 (1-9)	0 (0-0)	2 (1-4)	0 (0-0)
2000	Australia	15 (10-15)		1 (1-1)		12 (6-22)		1 (0-2)		11 (5-20)		1 (0-2)	
2001	Australia	21 (15-21)		2 (1-2)		18 (8-32)		1 (1-3)		16 (7-29)		1 (1-2)	
2002	Australia	16 (11-16)		1 (1-1)		6 (3-11)		1 (0-1)		6 (3-10)		0 (0-1)	
2003	Australia	22 (16-23)		2 (1-2)		13 (6-23)		1 (0-2)		11 (5-20)		1 (0-2)	
2004	Australia	28 (20-29)		2 (2-2)		14 (6-24)		1 (1-2)		12 (6-22)		1 (0-2)	
2005	Australia	18 (12-18)		1 (1-1)		9 (4-17)		1 (0-1)		8 (4-15)		1 (0-1)	
2006	Australia	14 (10-14)		1 (1-1)		8 (4-14)		1 (0-1)		7 (3-12)		1 (0-1)	
2007	Australia	13 (9-14)		1 (1-1)		4 (2-7)		0 (0-1)		4 (2-6)		0 (0-0)	
2008	Australia	10 (7-10)		1 (1-1)		5 (2-8)		0 (0-1)		4 (2-8)		0 (0-1)	
2009	Australia	11 (8-11)		1 (1-1)		2 (1-4)		0 (0-0)		2 (1-4)		0 (0-0)	
2010	Australia	17 (12-18)		1 (1-1)		7 (3-13)		1 (0-1)		6 (3-11)		0 (0-1)	
2011	Australia	9 (6-9)		1 (0-1)		4 (2-7)		0 (0-0)		4 (2-6)		0 (0-0)	
2012	Australia	5 (4-5)		0 (0-0)		2 (1-4)		0 (0-0)		2 (1-4)		0 (0-0)	
2013	Australia	5 (3-5)		0 (0-0)		2 (1-3)		0 (0-0)		2 (1-3)		0 (0-0)	
2014	Australia	4 (3-5)		0 (0-0)		2 (1-3)		0 (0-0)		1 (1-3)		0 (0-0)	
2015	Australia	4 (3-5)	0 (0-0)	0 (0-0)	0 (0-0)	2 (1-3)	0 (0-0)	0 (0-0)	0 (0-0)	1 (1-2)	0 (0-0)	0 (0-0)	0 (0-0)
2000	Austria	2 (2-2)		1 (0-1)		2 (1-4)		1 (0-1)		2 (1-4)		0 (0-1)	
2001	Austria	4 (3-4)		1 (1-1)		2 (1-4)		1 (0-1)		2 (1-4)		1 (0-1)	
2002	Austria	5 (3-5)		1 (1-1)		2 (1-3)		0 (0-1)		1 (1-2)		0 (0-1)	
2003	Austria	2 (1-2)		0 (0-0)		3 (1-5)		1 (0-1)		2 (1-4)		1 (0-1)	
2004	Austria	5 (3-5)		1 (1-1)		2 (1-3)		0 (0-1)		2 (1-3)		0 (0-1)	
2005	Austria	1 (0-1)		0 (0-0)		2 (1-4)		1 (0-1)		2 (1-4)		1 (0-1)	
2006	Austria	2 (1-2)		0 (0-0)		2 (1-4)		1 (0-1)		2 (1-3)		0 (0-1)	
2007	Austria	2 (1-2)		0 (0-1)		1 (0-1)		0 (0-0)		0 (0-1)		0 (0-0)	
2008	Austria	2 (1-2)		1 (0-1)		3 (1-5)		1 (0-1)		3 (1-5)		1 (0-1)	
2009	Austria	2 (2-3)		1 (0-1)		3 (2-6)		1 (0-2)		3 (1-5)		1 (0-1)	
2010	Austria	2 (1-2)		1 (0-1)		3 (1-5)		1 (0-1)		3 (1-5)		1 (0-1)	
2011	Austria	4 (3-4)		1 (1-1)		1 (1-2)		0 (0-1)		1 (1-2)		0 (0-1)	
2012	Austria	3 (2-3)		1 (1-1)		3 (1-5)		1 (0-1)		2 (1-4)		1 (0-1)	
2013	Austria	1 (1-1)		0 (0-0)		3 (1-5)		1 (0-1)		3 (1-5)		1 (0-1)	
2014	Austria	2 (1-2)		1 (0-1)		1 (0-2)		0 (0-0)		1 (0-1)		0 (0-0)	
2015	Austria	2 (1-2)	0 (0-0)	0 (0-0)	0 (0-0)	2 (1-4)	0 (0-0)	0 (0-1)	0 (0-0)	2 (1-3)	0 (0-0)	0 (0-1)	0 (0-0)
2000	Azerbaijan	559 (397-583)		81 (58-85)		67 (19-159)		10 (3-23)		60 (17-142)		9 (2-21)	
2001	Azerbaijan	485 (344-506)		75 (53-78)		57 (16-135)		9 (3-21)		51 (15-120)		8 (2-19)	
2002	Azerbaijan	429 (305-448)		69 (49-72)		49 (14-116)		8 (2-19)		44 (13-103)		7 (2-17)	
2003	Azerbaijan	388 (275-405)		64 (45-66)		43 (13-102)		7 (2-17)		38 (11-90)		6 (2-15)	
2004	Azerbaijan	361 (256-376)		60 (42-62)		39 (12-91)		6 (2-15)		34 (10-81)		6 (2-13)	
2005	Azerbaijan	337 (239-351)		56 (40-58)		35 (10-81)		6 (2-13)		31 (9-72)		5 (2-12)	
2006	Azerbaijan	321 (228-335)		52 (37-55)		32 (10-73)		5 (2-12)		28 (9-65)		5 (1-11)	
2007	Azerbaijan	305 (217-318)		49 (35-51)		29 (9-67)		5 (1-11)		26 (8-59)		4 (1-10)	
2008	Azerbaijan	295 (209-307)		46 (33-48)		27 (8-62)		4 (1-10)		24 (7-55)		4 (1-9)	
2009	Azerbaijan	288 (204-300)		43 (31-45)		26 (8-59)		4 (1-9)		23 (7-53)		3 (1-8)	
2010	Azerbaijan	288 (204-300)		41 (29-43)		25 (8-58)		4 (1-8)		23 (7-52)		3 (1-7)	
2011	Azerbaijan	294 (209-307)		40 (28-42)		26 (8-59)		4 (1-8)		23 (7-53)		3 (1-7)	
2012	Azerbaijan	303 (215-316)		39 (27-40)		27 (8-61)		3 (1-8)		24 (8-54)		3 (1-7)	
2013	Azerbaijan	313 (222-326)		37 (26-39)		27 (9-62)		3 (1-7)		24 (8-56)		3 (1-7)	
2014	Azerbaijan	230 (163-240)		26 (18-27)		19 (6-44)		2 (1-5)		17 (6-39)		2 (1-4)	

Spn = *Streptococcus pneumoniae*; npnm = non-pneumonia, non-meningitis; HIV = deaths only in HIV-infected children; Uncertainty range provided in parentheses.

Year	Country	Spn pneumonia deaths (UR)	Spn pneumonia deaths, HIV (UR)	Spn pneumonia mortality rate (UR)	Spn pneumonia mortality rate, HIV (UR)	Spn meningitis deaths (UR)	Spn meningitis deaths, HIV (UR)	Spn meningitis mortality rate (UR)	Spn meningitis mortality rate, HIV (UR)	Spn NPNM deaths (UR)	Spn NPNM deaths, HIV (UR)	Spn NPNM mortality rate (UR)	Spn NPNM mortality rate, HIV (UR)
2015	Azerbaijan	163 (116-170)	1 (0-1)	18 (13-19)	0 (0-0)	14 (4-31)	0 (0-0)	1 (0-3)	0 (0-0)	12 (4-27)	0 (0-0)	1 (0-3)	0 (0-0)
2000	Bahamas	4 (3-4)		15 (11-16)		0 (0-1)		1 (1-2)		0 (0-1)		1 (1-2)	
2001	Bahamas	3 (2-4)		13 (9-14)		1 (0-1)		2 (1-3)		0 (0-1)		2 (1-3)	
2002	Bahamas	3 (2-3)		11 (8-11)		1 (0-1)		2 (1-3)		0 (0-1)		2 (1-3)	
2003	Bahamas	2 (1-2)		6 (4-6)		0 (0-0)		1 (1-2)		0 (0-0)		1 (0-1)	
2004	Bahamas	1 (1-1)		5 (4-6)		0 (0-0)		1 (0-1)		0 (0-0)		1 (0-1)	
2005	Bahamas	2 (1-2)		6 (4-7)		0 (0-0)		1 (0-1)		0 (0-0)		1 (0-1)	
2006	Bahamas	2 (1-2)		7 (5-8)		0 (0-0)		1 (0-1)		0 (0-0)		1 (0-1)	
2007	Bahamas	3 (2-3)		11 (8-11)		0 (0-0)		0 (0-0)		0 (0-0)		0 (0-0)	
2008	Bahamas	4 (3-4)		14 (10-15)		0 (0-0)		0 (0-0)		0 (0-0)		0 (0-0)	
2009	Bahamas	4 (3-4)		15 (11-16)		0 (0-0)		1 (1-2)		0 (0-0)		1 (0-2)	
2010	Bahamas	3 (2-3)		12 (9-13)		0 (0-0)		1 (1-2)		0 (0-0)		1 (1-2)	
2011	Bahamas	3 (2-3)		11 (8-12)		0 (0-0)		1 (1-2)		0 (0-0)		1 (0-1)	
2012	Bahamas	3 (2-3)		10 (7-10)		0 (0-0)		0 (0-0)		0 (0-0)		0 (0-0)	
2013	Bahamas	2 (1-2)		6 (5-7)		0 (0-0)		0 (0-1)		0 (0-0)		0 (0-0)	
2014	Bahamas	1 (1-1)		4 (3-4)		0 (0-0)		0 (0-1)		0 (0-0)		0 (0-1)	
2015	Bahamas	1 (1-1)	0 (0-0)	3 (2-3)	0 (0-0)	0 (0-0)	0 (0-0)	0 (0-1)	0 (0-0)	0 (0-0)	0 (0-0)	0 (0-0)	0 (0-0)
2000	Bahrain	1 (1-1)		2 (1-2)		0 (0-1)		1 (0-1)		0 (0-1)		1 (0-1)	
2001	Bahrain	3 (2-3)		4 (3-4)		0 (0-0)		0 (0-1)		0 (0-0)		0 (0-1)	
2002	Bahrain	3 (2-3)		4 (3-4)		1 (1-1)		1 (1-2)		1 (1-1)		1 (1-2)	
2003	Bahrain	3 (2-3)		5 (3-5)		1 (0-1)		1 (0-1)		1 (0-1)		1 (1-1)	
2004	Bahrain	2 (2-3)		3 (2-3)		1 (1-2)		2 (1-2)		1 (1-2)		2 (1-2)	
2005	Bahrain	2 (2-3)		3 (2-3)		1 (1-2)		2 (1-2)		1 (1-2)		1 (1-2)	
2006	Bahrain	3 (2-3)		3 (2-4)		3 (2-4)		4 (2-5)		3 (2-4)		3 (2-5)	
2007	Bahrain	3 (2-3)		4 (3-4)		3 (2-4)		3 (2-5)		2 (1-3)		3 (2-4)	
2008	Bahrain	3 (2-3)		4 (3-4)		2 (1-3)		2 (1-3)		2 (1-2)		2 (1-3)	
2009	Bahrain	3 (2-3)		3 (2-3)		1 (0-1)		1 (0-1)		1 (0-1)		1 (0-1)	
2010	Bahrain	2 (1-2)		2 (1-2)		0 (0-0)		0 (0-0)		0 (0-0)		0 (0-0)	
2011	Bahrain	1 (1-1)		1 (1-2)		1 (1-1)		1 (1-1)		1 (1-1)		1 (1-1)	
2012	Bahrain	1 (0-1)		1 (0-1)		1 (0-1)		1 (0-1)		1 (0-1)		1 (0-1)	
2013	Bahrain	0 (0-1)		0 (0-0)		1 (0-1)		1 (0-1)		1 (0-1)		1 (0-1)	
2014	Bahrain	1 (0-1)		1 (0-1)		0 (0-0)		0 (0-0)		0 (0-0)		0 (0-0)	
2015	Bahrain	1 (0-1)	0 (0-0)	0 (0-0)	0 (0-0)	0 (0-1)	0 (0-0)	0 (0-1)	0 (0-0)	0 (0-1)	0 (0-0)	0 (0-1)	0 (0-0)
2000	Bangladesh	16671 (11822-17374)		101 (72-105)		2652 (860-6267)		16 (5-38)		834 (270-1970)		5 (2-12)	
2001	Bangladesh	15795 (11200-16461)		95 (67-99)		2484 (795-5897)		15 (5-36)		781 (250-1854)		5 (2-11)	
2002	Bangladesh	14261 (10113-14863)		86 (61-89)		2216 (700-5283)		13 (4-32)		697 (220-1661)		4 (1-10)	
2003	Bangladesh	13286 (9421-13846)		80 (57-83)		2040 (516-5033)		12 (3-30)		1816 (459-4481)		11 (3-27)	
2004	Bangladesh	12289 (8714-12807)		74 (53-78)		1867 (465-4617)		11 (3-28)		1662 (414-4110)		10 (3-25)	
2005	Bangladesh	11650 (8261-12141)		71 (50-74)		1754 (456-4296)		11 (3-26)		1561 (406-3825)		10 (2-23)	
2006	Bangladesh	10654 (7555-11103)		65 (46-68)		1588 (432-3848)		10 (3-24)		1414 (384-3425)		9 (2-21)	
2007	Bangladesh	9724 (6895-10134)		60 (43-63)		1436 (417-3427)		9 (3-21)		1279 (372-3051)		8 (2-19)	
2008	Bangladesh	8777 (6224-9147)		55 (39-57)		1282 (370-3053)		8 (2-19)		1141 (329-2718)		7 (2-17)	
2009	Bangladesh	7935 (5627-8270)		51 (36-53)		1145 (328-2725)		7 (2-17)		1019 (292-2426)		6 (2-15)	
2010	Bangladesh	7258 (5147-7564)		47 (33-49)		1032 (295-2458)		7 (2-16)		919 (262-2188)		6 (2-14)	
2011	Bangladesh	6629 (4700-6908)		43 (31-45)		931 (265-2219)		6 (2-14)		829 (236-1976)		5 (2-13)	
2012	Bangladesh	6123 (4342-6381)		40 (29-42)		853 (243-2027)		6 (2-13)		759 (217-1805)		5 (1-12)	
2013	Bangladesh	5655 (4010-5893)		37 (26-39)		784 (223-1860)		5 (1-12)		698 (199-1656)		5 (1-11)	
2014	Bangladesh	5273 (3739-5495)		35 (25-36)		729 (222-1709)		5 (1-11)		649 (197-1521)		4 (1-10)	
2015	Bangladesh	3886 (2755-4049)	2 (1-2)	26 (18-27)	0 (0-0)	518 (159-1211)	0 (0-1)	3 (1-8)	0 (0-0)	462 (141-1078)	0 (0-1)	3 (1-7)	0 (0-0)
2000	Barbados	0 (0-0)		0 (0-0)		1 (0-1)		4 (2-6)		1 (0-1)		4 (2-6)	
2001	Barbados	0 (0-0)		2 (1-2)		1 (0-1)		3 (1-4)		0 (0-1)		3 (1-4)	
2002	Barbados	1 (0-1)		4 (3-4)		0 (0-1)		2 (1-4)		0 (0-1)		2 (1-3)	
2003	Barbados	1 (0-1)		4 (3-4)		0 (0-0)		0 (0-0)		0 (0-0)		0 (0-0)	
2004	Barbados	1 (1-1)		5 (3-5)		0 (0-0)		0 (0-0)		0 (0-0)		0 (0-0)	
2005	Barbados	1 (1-1)		6 (4-6)		0 (0-0)		0 (0-0)		0 (0-0)		0 (0-0)	
2006	Barbados	2 (1-2)		9 (7-10)		0 (0-0)		0 (0-0)		0 (0-0)		0 (0-0)	
2007	Barbados	1 (1-1)		8 (5-8)		0 (0-0)		0 (0-0)		0 (0-0)		0 (0-0)	
2008	Barbados	1 (1-1)		5 (4-5)		0 (0-0)		0 (0-0)		0 (0-0)		0 (0-0)	
2009	Barbados	0 (0-0)		2 (1-2)		0 (0-0)		0 (0-0)		0 (0-0)		0 (0-0)	
2010	Barbados	0 (0-0)		3 (2-3)		0 (0-0)		0 (0-0)		0 (0-0)		0 (0-0)	
2011	Barbados	0 (0-0)		2 (2-2)		0 (0-0)		0 (0-0)		0 (0-0)		0 (0-0)	
2012	Barbados	0 (0-0)		3 (2-3)		0 (0-0)		0 (0-0)		0 (0-0)		0 (0-0)	
2013	Barbados	0 (0-0)		1 (1-1)		0 (0-0)		0 (0-0)		0 (0-0)		0 (0-0)	
2014	Barbados	0 (0-0)		1 (1-1)		0 (0-0)		0 (0-0)		0 (0-0)		0 (0-0)	
2015	Barbados	0 (0-0)	0 (0-0)	1 (1-1)	0 (0-0)	0 (0-0)	0 (0-0)	0 (0-0)	0 (0-0)	0 (0-0)	0 (0-0)	0 (0-0)	0 (0-0)
2000	Belarus	45 (32-47)		10 (7-10)		4 (2-13)		1 (0-3)		4 (1-12)		1 (0-3)	
2001	Belarus	39 (28-41)		9 (6-9)		4 (1-12)		1 (0-3)		3 (1-10)		1 (0-2)	
2002	Belarus	35 (25-36)		8 (6-8)		3 (1-10)		1 (0-2)		3 (1-9)		1 (0-2)	
2003	Belarus	31 (22-32)		7 (5-7)		3 (1-9)		1 (0-2)		3 (1-8)		1 (0-2)	
2004	Belarus	29 (21-30)		7 (5-7)		3 (1-9)		1 (0-2)		3 (1-8)		1 (0-2)	
2005	Belarus	26 (18-27)		6 (4-6)		3 (1-8)		1 (0-2)		2 (1-7)		1 (0-2)	
2006	Belarus	24 (17-25)		5 (4-5)		3 (1-7)		1 (0-2)		2 (1-6)		1 (0-1)	
2007	Belarus	21 (15-22)		5 (3-5)		2 (1-7)		1 (0-1)		2 (1-6)		0 (0-1)	
2008	Belarus	18 (13-19)		4 (3-4)		2 (1-7)		1 (0-1)		2 (1-6)		0 (0-1)	
2009	Belarus	17 (12-18)		4 (3-4)		3 (1-7)		1 (0-1)		2 (1-6)		0 (0-1)	
2010	Belarus	15 (11-16)		3 (2-3)		2 (1-5)		0 (0-1)		2 (1-4)		0 (0-1)	
2011	Belarus	13 (9-13)		2 (2-3)		2 (1-5)		0 (0-1)		2 (1-5)		0 (0-1)	
2012	Belarus	13 (9-13)		2 (2-2)		2 (1-5)		0 (0-1)		2 (1-5)		0 (0-1)	
2013	Belarus	12 (9-13)		2 (2-2)		2 (1-5)		0 (0-1)		2 (1-4)		0 (0-1)	
2014	Belarus	11 (8-11)		2 (1-2)		2 (1-4)		0 (0-1)		2 (1-4)		0 (0-1)	
2015	Belarus	10 (7-11)	0 (0-0)	2 (1-2)	0 (0-0)	2 (1-4)	0 (0-0)	0 (0-1)	0 (0-0)	1 (1-3)	0 (0-0)	0 (0-1)	0 (0-0)
2000	Belgium	3 (2-3)		0 (0-0)		10 (4-17)		2 (1-3)		9 (4-16)		2 (1-3)	
2001	Belgium	2 (2-2)		0 (0-0)		12 (5-22)		2 (1-4)		11 (5-19)		2 (1-3)	
2002	Belgium	8 (5-8)		1 (1-1)		8 (4-15)		1 (1-3)		7 (3-13)		1 (1-2)	
2003	Belgium	4 (3-4)		1 (1-1)		8 (4-15)		1 (1-3)		7 (3-13)		1 (1-2)	
2004	Belgium	2 (2-3)		0 (0-0)		11 (5-19)		2 (1-3)		9 (4-17)		2 (1-3)	
2005	Belgium	8 (5-8)		1 (1-1)		9 (4-16)		2 (1-3)		8 (3-14)		1 (1-2)	
2006	Belgium	3 (2-3)		1 (0-1)		10 (4-18)		2 (1-3)		9 (4-16)		1 (1-3)	

Spn = *Streptococcus pneumoniae*; npnm = non-pneumonia, non-meningitis; HIV = deaths only in HIV-infected children; Uncertainty range provided in parentheses.

Year	Country	Spn pneumonia deaths (UR)	Spn pneumonia deaths, HIV (UR)	Spn pneumonia mortality rate (UR)	Spn pneumonia mortality rate, HIV (UR)	Spn meningitis deaths (UR)	Spn meningitis deaths, HIV (UR)	Spn meningitis mortality rate (UR)	Spn meningitis mortality rate, HIV (UR)	Spn NPNM deaths (UR)	Spn NPNM deaths, HIV (UR)	Spn NPNM mortality rate (UR)	Spn NPNM mortality rate, HIV (UR)
2007	Belgium	7 (5-8)		1 (1-1)		9 (4-16)		1 (1-3)		8 (3-14)		1 (1-2)	
2008	Belgium	4 (3-4)		1 (0-1)		6 (3-11)		1 (0-2)		5 (2-10)		1 (0-2)	
2009	Belgium	6 (4-6)		1 (1-1)		6 (3-11)		1 (0-2)		5 (2-10)		1 (0-2)	
2010	Belgium	6 (4-6)		1 (1-1)		3 (1-5)		0 (0-1)		2 (1-4)		0 (0-1)	
2011	Belgium	4 (3-4)		1 (0-1)		4 (2-6)		1 (0-1)		3 (1-6)		0 (0-1)	
2012	Belgium	0 (0-0)		0 (0-0)		3 (1-6)		0 (0-1)		3 (1-5)		0 (0-1)	
2013	Belgium	2 (1-2)		0 (0-0)		2 (1-3)		0 (0-1)		2 (1-3)		0 (0-0)	
2014	Belgium	2 (1-2)		0 (0-0)		2 (1-3)		0 (0-0)		1 (1-3)		0 (0-0)	
2015	Belgium	1 (1-1)	0 (0-0)	0 (0-0)	0 (0-0)	1 (1-3)	0 (0-0)	0 (0-0)	0 (0-0)	1 (1-2)	0 (0-0)	0 (0-0)	0 (0-0)
2000	Belize	8 (6-8)		22 (15-22)		1 (0-5)		4 (1-13)		1 (0-4)		4 (1-11)	
2001	Belize	7 (5-7)		19 (13-20)		2 (0-8)		7 (1-21)		2 (0-7)		6 (1-18)	
2002	Belize	11 (8-12)		30 (22-32)		4 (1-11)		10 (2-30)		3 (1-10)		9 (2-27)	
2003	Belize	9 (6-9)		24 (17-25)		1 (0-3)		2 (0-7)		1 (0-2)		2 (0-6)	
2004	Belize	9 (6-9)		24 (17-25)		2 (0-6)		5 (1-16)		2 (0-5)		5 (1-14)	
2005	Belize	5 (3-5)		13 (9-13)		0 (0-0)		0 (0-0)		0 (0-0)		0 (0-0)	
2006	Belize	9 (7-10)		25 (18-26)		0 (0-1)		1 (0-4)		0 (0-1)		1 (0-3)	
2007	Belize	2 (1-2)		5 (3-5)		1 (0-3)		3 (1-9)		1 (0-3)		3 (1-8)	
2008	Belize	3 (2-3)		7 (5-7)		0 (0-0)		0 (0-0)		0 (0-0)		0 (0-0)	
2009	Belize	3 (2-3)		8 (6-8)		0 (0-0)		0 (0-0)		0 (0-0)		0 (0-0)	
2010	Belize	4 (3-4)		11 (7-11)		1 (0-2)		2 (1-5)		1 (0-2)		2 (1-5)	
2011	Belize	1 (1-1)		2 (1-2)		0 (0-0)		0 (0-0)		0 (0-0)		0 (0-0)	
2012	Belize	8 (6-9)		22 (16-23)		1 (0-2)		2 (1-6)		1 (0-2)		2 (1-5)	
2013	Belize	3 (2-3)		8 (5-8)		2 (1-4)		5 (2-12)		2 (1-4)		4 (2-10)	
2014	Belize	4 (3-4)		10 (7-11)		1 (0-2)		3 (1-6)		1 (0-2)		2 (1-5)	
2015	Belize	4 (3-4)	0 (0-0)	10 (7-11)	0 (0-0)	1 (0-2)	0 (0-0)	3 (1-6)	0 (0-0)	1 (0-2)	0 (0-0)	2 (1-5)	0 (0-0)
2000	Benin	2211 (1568-2304)		181 (128-188)		381 (139-846)		31 (11-69)		120 (44-266)		10 (4-22)	
2001	Benin	2164 (1535-2256)		172 (122-179)		371 (136-823)		30 (11-65)		117 (43-259)		9 (3-21)	
2002	Benin	2148 (1523-2239)		166 (118-173)		367 (135-813)		28 (10-63)		115 (42-256)		9 (3-20)	
2003	Benin	2122 (1505-2211)		160 (113-167)		361 (133-800)		27 (10-60)		114 (42-252)		9 (3-19)	
2004	Benin	2086 (1479-2174)		153 (109-160)		355 (131-785)		26 (10-58)		111 (41-247)		8 (3-18)	
2005	Benin	2057 (1459-2144)		148 (105-154)		349 (129-773)		25 (9-55)		110 (41-243)		8 (3-17)	
2006	Benin	2107 (1494-2196)		147 (104-153)		357 (132-790)		25 (9-55)		112 (42-248)		8 (3-17)	
2007	Benin	2136 (1515-2226)		146 (103-152)		361 (133-802)		25 (9-55)		114 (42-252)		8 (3-17)	
2008	Benin	2188 (1552-2280)		147 (104-153)		369 (134-821)		25 (9-55)		116 (42-258)		8 (3-17)	
2009	Benin	2245 (1592-2340)		148 (105-154)		378 (137-843)		25 (9-55)		119 (43-265)		8 (3-17)	
2010	Benin	2294 (1627-2390)		148 (105-155)		385 (138-861)		25 (9-56)		121 (43-271)		8 (3-17)	
2011	Benin	2014 (1428-2099)		128 (91-133)		330 (117-739)		21 (7-47)		104 (37-232)		7 (2-15)	
2012	Benin	1539 (1091-1604)		96 (68-100)		242 (86-544)		15 (5-34)		76 (27-171)		5 (2-11)	
2013	Benin	1464 (1038-1525)		90 (64-94)		235 (84-527)		14 (5-32)		74 (26-166)		5 (2-10)	
2014	Benin	1369 (971-1427)		83 (59-86)		224 (80-501)		14 (5-30)		70 (25-157)		4 (2-10)	
2015	Benin	898 (637-936)	47 (33-49)	53 (38-56)	3 (2-3)	138 (49-308)	7 (3-16)	8 (3-18)	0 (0-1)	43 (16-97)	2 (1-5)	3 (1-6)	0 (0-0)
2000	Bhutan	78 (55-81)		105 (74-109)		11 (5-22)		14 (6-29)		3 (1-7)		5 (2-9)	
2001	Bhutan	71 (50-74)		96 (68-100)		10 (4-19)		13 (6-26)		3 (1-6)		4 (2-8)	
2002	Bhutan	64 (46-67)		89 (63-92)		9 (3-18)		12 (5-25)		8 (3-16)		11 (4-22)	
2003	Bhutan	58 (41-60)		81 (57-84)		8 (3-16)		11 (4-22)		7 (3-14)		10 (4-20)	
2004	Bhutan	52 (37-54)		73 (52-76)		7 (3-14)		10 (4-20)		6 (2-13)		9 (3-18)	
2005	Bhutan	47 (34-49)		67 (48-70)		6 (3-13)		9 (4-18)		5 (2-11)		8 (3-16)	
2006	Bhutan	43 (31-45)		62 (44-64)		6 (2-11)		8 (3-16)		5 (2-10)		7 (3-14)	
2007	Bhutan	40 (28-42)		57 (40-59)		5 (2-10)		7 (3-15)		5 (2-9)		6 (3-13)	
2008	Bhutan	37 (26-38)		52 (37-54)		5 (2-9)		7 (3-13)		4 (2-8)		6 (2-12)	
2009	Bhutan	34 (24-35)		47 (34-49)		4 (2-8)		6 (3-12)		4 (2-7)		5 (2-10)	
2010	Bhutan	31 (22-32)		43 (31-45)		4 (2-7)		5 (2-11)		3 (1-7)		5 (2-9)	
2011	Bhutan	28 (20-29)		39 (28-41)		3 (1-7)		5 (2-9)		3 (1-6)		4 (2-8)	
2012	Bhutan	25 (18-26)		36 (25-37)		3 (1-6)		4 (2-8)		3 (1-5)		4 (2-8)	
2013	Bhutan	22 (16-23)		33 (23-34)		3 (1-5)		4 (2-8)		2 (1-5)		4 (2-7)	
2014	Bhutan	20 (14-21)		31 (22-32)		2 (1-5)		4 (2-7)		2 (1-4)		3 (1-6)	
2015	Bhutan	19 (13-20)	1 (0-1)	29 (21-30)	1 (1-1)	2 (1-4)	0 (0-0)	3 (2-7)	0 (0-0)	2 (1-4)	0 (0-0)	3 (1-6)	0 (0-0)
2000	Bolivia (Plurinational State of)	1187 (841-1237)		104 (74-108)		123 (40-317)		11 (4-28)		39 (13-100)		3 (1-9)	
2001	Bolivia (Plurinational State of)	1116 (791-1163)		97 (69-101)		116 (38-300)		10 (3-26)		37 (12-94)		3 (1-8)	
2002	Bolivia (Plurinational State of)	1036 (734-1079)		89 (63-93)		108 (29-289)		9 (2-25)		96 (26-257)		8 (2-22)	
2003	Bolivia (Plurinational State of)	955 (677-995)		82 (58-86)		99 (26-267)		9 (2-23)		88 (24-237)		8 (2-20)	
2004	Bolivia (Plurinational State of)	872 (618-908)		75 (53-78)		90 (24-242)		8 (2-21)		80 (21-216)		7 (2-19)	
2005	Bolivia (Plurinational State of)	801 (568-835)		69 (49-72)		82 (22-221)		7 (2-19)		73 (19-197)		6 (2-17)	
2006	Bolivia (Plurinational State of)	741 (525-772)		64 (45-66)		75 (20-202)		6 (2-17)		67 (18-180)		6 (2-15)	
2007	Bolivia (Plurinational State of)	688 (488-717)		59 (42-61)		69 (18-187)		6 (2-16)		62 (16-166)		5 (1-14)	
2008	Bolivia (Plurinational State of)	650 (461-677)		56 (39-58)		65 (17-175)		6 (1-15)		58 (15-156)		5 (1-13)	
2009	Bolivia (Plurinational State of)	613 (434-638)		52 (37-55)		62 (20-158)		5 (2-13)		55 (18-140)		5 (2-12)	
2010	Bolivia (Plurinational State of)	577 (410-602)		49 (35-51)		58 (18-149)		5 (2-13)		52 (16-132)		4 (1-11)	
2011	Bolivia (Plurinational State of)	542 (384-565)		46 (33-48)		54 (16-140)		5 (1-12)		48 (14-124)		4 (1-11)	
2012	Bolivia (Plurinational State of)	513 (364-534)		44 (31-46)		51 (15-132)		4 (1-11)		45 (13-118)		4 (1-10)	
2013	Bolivia (Plurinational State of)	481 (341-501)		41 (29-43)		47 (13-124)		4 (1-11)		42 (12-110)		4 (1-9)	
2014	Bolivia (Plurinational State of)	351 (249-366)		30 (21-31)		33 (9-89)		3 (1-8)		30 (8-79)		3 (1-7)	
2015	Bolivia (Plurinational State of)	245 (174-255)	0 (0-0)	21 (15-22)	0 (0-0)	23 (6-62)	0 (0-0)	2 (1-5)	0 (0-0)	20 (5-55)	0 (0-0)	2 (0-5)	0 (0-0)
2000	Bosnia and Herzegovina	6 (4-6)		3 (2-3)		1 (0-2)		0 (0-1)		0 (0-2)		0 (0-1)	
2001	Bosnia and Herzegovina	5 (4-5)		2 (2-3)		0 (0-2)		0 (0-1)		0 (0-1)		0 (0-1)	
2002	Bosnia and Herzegovina	5 (3-5)		2 (2-2)		0 (0-1)		0 (0-1)		0 (0-1)		0 (0-1)	
2003	Bosnia and Herzegovina	4 (3-5)		2 (2-2)		0 (0-1)		0 (0-1)		0 (0-1)		0 (0-1)	
2004	Bosnia and Herzegovina	4 (3-4)		2 (2-3)		1 (0-2)		0 (0-1)		0 (0-1)		0 (0-1)	
2005	Bosnia and Herzegovina	5 (3-5)		3 (2-3)		1 (0-2)		0 (0-1)		1 (0-2)		0 (0-1)	
2006	Bosnia and Herzegovina	4 (3-4)		3 (2-3)		1 (0-2)		0 (0-1)		1 (0-2)		0 (0-1)	
2007	Bosnia and Herzegovina	5 (3-5)		3 (2-3)		1 (0-2)		0 (0-1)		1 (0-2)		0 (0-1)	
2008	Bosnia and Herzegovina	5 (3-5)		3 (2-3)		1 (0-2)		0 (0-1)		1 (0-2)		0 (0-1)	
2009	Bosnia and Herzegovina	5 (3-5)		3 (2-3)		1 (0-2)		0 (0-1)		1 (0-2)		0 (0-1)	
2010	Bosnia and Herzegovina	4 (3-4)		2 (2-2)		1 (0-2)		0 (0-1)		0 (0-2)		0 (0-1)	
2011	Bosnia and Herzegovina	3 (2-3)		2 (1-2)		0 (0-1)		0 (0-1)		0 (0-1)		0 (0-1)	
2012	Bosnia and Herzegovina	3 (2-3)		2 (1-2)		0 (0-1)		0 (0-1)		0 (0-1)		0 (0-1)	
2013	Bosnia and Herzegovina	2 (2-2)		1 (1-1)		0 (0-1)		0 (0-1)		0 (0-1)		0 (0-0)	
2014	Bosnia and Herzegovina	2 (1-2)		1 (1-1)		0 (0-1)		0 (0-0)		0 (0-1)		0 (0-0)	

Spn = *Streptococcus pneumoniae*; npnm = non-pneumonia, non-meningitis; HIV = deaths only in HIV-infected children; Uncertainty range provided in parentheses.

Year	Country	Spn pneumonia deaths (UR)	Spn pneumonia deaths, HIV (UR)	Spn pneumonia mortality rate (UR)	Spn pneumonia mortality rate, HIV (UR)	Spn meningitis deaths (UR)	Spn meningitis deaths, HIV (UR)	Spn meningitis mortality rate (UR)	Spn meningitis mortality rate, HIV (UR)	Spn NPNM deaths (UR)	Spn NPNM deaths, HIV (UR)	Spn NPNM mortality rate (UR)	Spn NPNM mortality rate, HIV (UR)
2015	Bosnia and Herzegovina	2 (1-2)	0 (0-0)	1 (1-1)	0 (0-0)	0 (0-1)	0 (0-0)	0 (0-0)	0 (0-0)	0 (0-1)	0 (0-0)	0 (0-0)	0 (0-0)
2000	Botswana	86 (61-89)		39 (28-41)		9 (3-20)		4 (1-9)		3 (1-6)		1 (0-3)	
2001	Botswana	88 (62-92)		40 (28-42)		9 (3-21)		4 (1-9)		3 (1-6)		1 (0-3)	
2002	Botswana	91 (64-95)		41 (29-43)		9 (3-21)		4 (1-10)		3 (1-7)		1 (0-3)	
2003	Botswana	94 (67-98)		43 (31-45)		9 (3-22)		4 (1-10)		3 (1-7)		1 (0-3)	
2004	Botswana	99 (70-103)		45 (32-47)		10 (3-23)		4 (1-10)		3 (1-7)		1 (0-3)	
2005	Botswana	103 (73-108)		47 (33-49)		10 (3-24)		5 (1-11)		9 (2-21)		4 (1-10)	
2006	Botswana	106 (76-111)		49 (34-51)		10 (3-24)		5 (1-11)		9 (2-22)		4 (1-10)	
2007	Botswana	110 (78-115)		50 (35-52)		10 (3-25)		5 (1-11)		9 (2-22)		4 (1-10)	
2008	Botswana	114 (81-118)		51 (36-53)		11 (3-25)		5 (1-11)		9 (2-23)		4 (1-10)	
2009	Botswana	115 (82-120)		50 (36-52)		11 (3-25)		5 (1-11)		9 (2-23)		4 (1-10)	
2010	Botswana	115 (82-120)		49 (35-51)		10 (3-25)		4 (1-11)		9 (2-22)		4 (1-10)	
2011	Botswana	117 (83-122)		49 (35-51)		11 (3-25)		4 (1-11)		9 (2-23)		4 (1-9)	
2012	Botswana	95 (68-99)		39 (27-40)		8 (2-20)		3 (1-8)		7 (2-18)		3 (1-7)	
2013	Botswana	82 (58-85)		32 (23-34)		7 (2-17)		3 (1-7)		6 (2-15)		2 (1-6)	
2014	Botswana	68 (48-71)		26 (19-28)		6 (2-14)		2 (1-5)		5 (1-12)		2 (1-5)	
2015	Botswana	61 (44-64)	14 (10-15)	23 (17-24)	5 (4-6)	5 (1-13)	1 (0-3)	2 (1-5)	0 (0-1)	5 (1-11)	1 (0-3)	2 (0-4)	0 (0-1)
2000	Brazil	5337 (3784-5562)		31 (22-32)		1139 (280-3197)		7 (2-18)		1014 (249-2846)		6 (1-16)	
2001	Brazil	4715 (3344-4914)		27 (19-28)		1044 (170-3499)		6 (1-20)		929 (152-3115)		5 (1-18)	
2002	Brazil	4095 (2904-4268)		23 (16-24)		815 (135-2727)		5 (1-15)		726 (120-2427)		4 (1-14)	
2003	Brazil	3735 (2648-3892)		21 (15-22)		682 (115-2275)		4 (1-13)		607 (102-2025)		3 (1-11)	
2004	Brazil	3412 (2419-3555)		20 (14-20)		641 (109-2130)		4 (1-12)		570 (97-1897)		3 (1-11)	
2005	Brazil	2877 (2040-2999)		17 (12-17)		604 (105-2000)		4 (1-12)		537 (93-1781)		3 (1-10)	
2006	Brazil	2612 (1852-2722)		15 (11-16)		570 (100-1883)		3 (1-11)		508 (89-1677)		3 (1-10)	
2007	Brazil	2193 (1555-2286)		13 (9-14)		466 (83-1534)		3 (1-9)		415 (74-1366)		3 (0-8)	
2008	Brazil	1791 (1270-1866)		11 (8-12)		402 (72-1320)		3 (0-8)		358 (64-1175)		2 (0-7)	
2009	Brazil	1587 (1125-1654)		10 (7-11)		330 (60-1080)		2 (0-7)		293 (53-961)		2 (0-6)	
2010	Brazil	1146 (813-1195)		8 (5-8)		259 (47-846)		2 (0-6)		230 (42-753)		2 (0-5)	
2011	Brazil	900 (638-938)		6 (4-6)		131 (24-429)		1 (0-3)		117 (21-382)		1 (0-3)	
2012	Brazil	752 (533-784)		5 (4-5)		101 (19-331)		1 (0-2)		90 (17-295)		1 (0-2)	
2013	Brazil	753 (534-784)		5 (4-5)		87 (16-283)		1 (0-2)		77 (14-252)		1 (0-2)	
2014	Brazil	678 (481-706)		5 (3-5)		88 (16-287)		1 (0-2)		78 (14-256)		1 (0-2)	
2015	Brazil	701 (497-730)	1 (1-1)	5 (3-5)	0 (0-0)	89 (16-291)	0 (0-0)	1 (0-2)	0 (0-0)	79 (15-259)	0 (0-0)	1 (0-2)	0 (0-0)
2000	Brunei Darussalam	1 (1-1)		3 (2-3)		0 (0-0)		0 (0-0)		0 (0-0)		0 (0-0)	
2001	Brunei Darussalam	1 (1-1)		3 (2-3)		0 (0-0)		0 (0-0)		0 (0-0)		0 (0-0)	
2002	Brunei Darussalam	1 (1-1)		3 (2-4)		0 (0-0)		0 (0-0)		0 (0-0)		0 (0-0)	
2003	Brunei Darussalam	1 (1-1)		3 (2-4)		0 (0-0)		0 (0-0)		0 (0-0)		0 (0-0)	
2004	Brunei Darussalam	1 (1-1)		3 (2-4)		0 (0-0)		0 (0-0)		0 (0-0)		0 (0-0)	
2005	Brunei Darussalam	1 (1-1)		3 (2-3)		0 (0-0)		0 (0-0)		0 (0-0)		0 (0-0)	
2006	Brunei Darussalam	1 (1-1)		3 (2-3)		0 (0-0)		0 (0-0)		0 (0-0)		0 (0-0)	
2007	Brunei Darussalam	1 (1-1)		3 (2-3)		0 (0-0)		0 (0-0)		0 (0-0)		0 (0-0)	
2008	Brunei Darussalam	1 (1-1)		4 (3-4)		0 (0-0)		0 (0-0)		0 (0-0)		0 (0-0)	
2009	Brunei Darussalam	1 (1-1)		3 (2-3)		0 (0-0)		0 (0-0)		0 (0-0)		0 (0-0)	
2010	Brunei Darussalam	1 (1-1)		4 (3-4)		0 (0-0)		0 (0-0)		0 (0-0)		0 (0-0)	
2011	Brunei Darussalam	1 (1-1)		3 (2-3)		0 (0-0)		0 (0-0)		0 (0-0)		0 (0-0)	
2012	Brunei Darussalam	1 (1-1)		3 (2-4)		0 (0-0)		1 (0-1)		0 (0-0)		1 (0-1)	
2013	Brunei Darussalam	1 (1-1)		3 (2-3)		0 (0-0)		1 (0-1)		0 (0-0)		1 (0-1)	
2014	Brunei Darussalam	1 (1-1)		2 (2-3)		0 (0-0)		1 (1-1)		0 (0-0)		1 (1-1)	
2015	Brunei Darussalam	1 (1-1)	0 (0-0)	2 (2-2)	0 (0-0)	0 (0-0)	0 (0-0)	0 (0-1)	0 (0-0)	0 (0-0)	0 (0-0)	0 (0-0)	0 (0-0)
2000	Bulgaria	84 (60-88)		26 (18-27)		6 (3-10)		2 (1-3)		5 (2-9)		2 (1-3)	
2001	Bulgaria	78 (56-82)		24 (17-25)		5 (2-9)		1 (1-3)		4 (2-8)		1 (1-2)	
2002	Bulgaria	63 (45-65)		20 (14-21)		3 (1-5)		1 (0-1)		2 (1-4)		1 (0-1)	
2003	Bulgaria	73 (52-77)		24 (17-25)		7 (3-12)		2 (1-4)		6 (3-11)		2 (1-3)	
2004	Bulgaria	52 (37-54)		17 (12-17)		6 (3-10)		2 (1-3)		5 (2-9)		2 (1-3)	
2005	Bulgaria	58 (41-61)		19 (13-19)		5 (2-9)		2 (1-3)		4 (2-8)		1 (1-3)	
2006	Bulgaria	56 (40-58)		18 (12-18)		6 (3-11)		2 (1-4)		6 (3-10)		2 (1-3)	
2007	Bulgaria	51 (36-53)		16 (11-16)		5 (2-9)		2 (1-3)		5 (2-8)		1 (1-3)	
2008	Bulgaria	45 (32-47)		13 (9-14)		7 (3-13)		2 (1-4)		6 (3-11)		2 (1-3)	
2009	Bulgaria	67 (47-69)		19 (14-20)		5 (2-9)		1 (1-2)		4 (2-8)		1 (1-2)	
2010	Bulgaria	48 (34-50)		14 (10-14)		4 (2-8)		1 (1-2)		4 (2-7)		1 (1-2)	
2011	Bulgaria	29 (21-31)		8 (6-8)		3 (1-5)		1 (0-1)		2 (1-4)		1 (0-1)	
2012	Bulgaria	25 (17-26)		7 (5-7)		2 (1-4)		1 (0-1)		2 (1-4)		1 (0-1)	
2013	Bulgaria	23 (16-24)		7 (5-7)		2 (1-4)		1 (0-1)		2 (1-3)		1 (0-1)	
2014	Bulgaria	15 (11-16)		4 (3-5)		1 (1-2)		0 (0-1)		1 (1-2)		0 (0-1)	
2015	Bulgaria	13 (9-13)	0 (0-0)	4 (3-4)	0 (0-0)	1 (1-2)	0 (0-0)	0 (0-1)	0 (0-0)	1 (0-2)	0 (0-0)	0 (0-1)	0 (0-0)
2000	Burkina Faso	2822 (2001-2941)		132 (94-138)		543 (287-1035)		25 (13-48)		171 (90-325)		8 (4-15)	
2001	Burkina Faso	2991 (2121-3117)		136 (96-141)		575 (305-1092)		26 (14-50)		181 (96-343)		8 (4-16)	
2002	Burkina Faso	3173 (2250-3307)		140 (99-146)		609 (325-1154)		27 (14-51)		192 (102-363)		8 (5-16)	
2003	Burkina Faso	3363 (2385-3505)		145 (103-151)		644 (345-1217)		28 (15-52)		202 (108-383)		9 (5-16)	
2004	Burkina Faso	3525 (2499-3673)		148 (105-154)		671 (362-1268)		28 (15-53)		211 (114-399)		9 (5-17)	
2005	Burkina Faso	3569 (2531-3719)		146 (104-152)		676 (367-1277)		28 (15-52)		212 (115-401)		9 (5-16)	
2006	Burkina Faso	3454 (2449-3600)		137 (97-143)		650 (242-1404)		26 (10-56)		204 (76-441)		8 (3-17)	
2007	Burkina Faso	3186 (2259-3320)		123 (87-128)		599 (232-1282)		23 (9-49)		188 (73-403)		7 (3-15)	
2008	Burkina Faso	2878 (2041-2999)		108 (76-112)		541 (218-1143)		20 (8-43)		170 (68-359)		6 (3-13)	
2009	Burkina Faso	2719 (1928-2834)		99 (70-103)		511 (213-1064)		19 (8-39)		161 (67-334)		6 (2-12)	
2010	Burkina Faso	2707 (1919-2821)		96 (68-100)		509 (220-1039)		18 (8-37)		160 (69-327)		6 (2-12)	
2011	Burkina Faso	2877 (2040-2998)		100 (71-104)		540 (242-1081)		19 (8-38)		170 (76-340)		6 (3-12)	
2012	Burkina Faso	3054 (2165-3182)		104 (74-108)		574 (267-1122)		20 (9-38)		180 (84-353)		6 (3-12)	
2013	Burkina Faso	3253 (2307-3390)		109 (77-113)		612 (297-1170)		20 (10-39)		192 (93-368)		6 (3-12)	
2014	Burkina Faso	2241 (1589-2335)		74 (52-77)		395 (200-739)		13 (7-24)		124 (63-232)		4 (2-8)	
2015	Burkina Faso	1882 (1335-1961)	71 (51-74)	61 (43-63)	2 (2-2)	332 (177-608)	13 (7-23)	11 (6-20)	0 (0-1)	104 (56-191)	4 (2-7)	3 (2-6)	0 (0-0)
2000	Burundi	2274 (1613-2370)		190 (135-198)		497 (275-940)		41 (23-78)		156 (86-296)		13 (7-25)	
2001	Burundi	2264 (1605-2359)		187 (132-195)		495 (187-1074)		41 (15-89)		156 (59-338)		13 (5-28)	
2002	Burundi	2224 (1577-2318)		180 (128-188)		488 (184-1062)		39 (15-86)		153 (58-334)		12 (5-27)	
2003	Burundi	2358 (1672-2457)		186 (132-194)		519 (195-1134)		41 (15-90)		163 (61-356)		13 (5-28)	
2004	Burundi	2445 (1734-2548)		187 (133-195)		541 (203-1183)		41 (16-91)		170 (64-372)		13 (5-28)	
2005	Burundi	2433 (1725-2535)		179 (127-187)		541 (202-1183)		40 (15-87)		170 (64-372)		13 (5-27)	
2006	Burundi	2565 (1819-2673)		181 (128-189)		573 (220-1241)		40 (16-88)		180 (69-390)		13 (5-28)	

Spn = *Streptococcus pneumoniae*; npnm = non-pneumonia, non-meningitis; HIV = deaths only in HIV-infected children; Uncertainty range provided in parentheses.

Year	Country	Spn pneumonia deaths (UR)	Spn pneumonia deaths, HIV (UR)	Spn pneumonia mortality rate (UR)	Spn pneumonia mortality rate, HIV (UR)	Spn meningitis deaths (UR)	Spn meningitis deaths, HIV (UR)	Spn meningitis mortality rate (UR)	Spn meningitis mortality rate, HIV (UR)	Spn NPNM deaths (UR)	Spn NPNM deaths, HIV (UR)	Spn NPNM mortality rate (UR)	Spn NPNM mortality rate, HIV (UR)
2007	Burundi	2578 (1828-2686)		174 (123-181)		578 (228-1237)		39 (15-83)		182 (72-389)		12 (5-26)	
2008	Burundi	2574 (1826-2683)		166 (118-173)		579 (234-1225)		37 (15-79)		182 (74-385)		12 (5-25)	
2009	Burundi	2582 (1831-2691)		159 (113-166)		585 (243-1219)		36 (15-75)		184 (76-383)		11 (5-24)	
2010	Burundi	2574 (1826-2683)		152 (108-158)		588 (251-1207)		35 (15-71)		185 (79-380)		11 (5-22)	
2011	Burundi	2459 (1744-2563)		138 (98-144)		562 (247-1137)		32 (14-64)		177 (78-358)		10 (4-20)	
2012	Burundi	1623 (1151-1691)		88 (62-92)		349 (158-695)		19 (9-38)		110 (50-218)		6 (3-12)	
2013	Burundi	1338 (949-1394)		70 (50-73)		291 (136-570)		15 (7-30)		92 (43-179)		5 (2-9)	
2014	Burundi	1199 (850-1249)		61 (43-64)		277 (134-533)		14 (7-27)		87 (42-168)		4 (2-9)	
2015	Burundi	879 (623-916)	38 (27-40)	43 (31-45)	2 (1-2)	199 (100-378)	9 (4-16)	10 (5-19)	0 (0-1)	63 (31-119)	3 (1-5)	3 (2-6)	0 (0-0)
2000	Cambodia	2341 (1660-2440)		151 (107-157)		404 (140-918)		26 (9-59)		127 (44-289)		8 (3-19)	
2001	Cambodia	2053 (1456-2139)		135 (96-141)		348 (123-788)		23 (8-52)		110 (39-248)		7 (3-16)	
2002	Cambodia	1783 (1264-1858)		119 (84-124)		298 (107-670)		20 (7-45)		94 (34-211)		6 (2-14)	
2003	Cambodia	1557 (1104-1622)		104 (74-108)		256 (94-573)		17 (6-38)		80 (30-180)		5 (2-12)	
2004	Cambodia	1400 (993-1459)		93 (66-97)		227 (71-519)		15 (5-35)		202 (63-462)		13 (4-31)	
2005	Cambodia	1286 (912-1340)		85 (60-89)		204 (65-465)		14 (4-31)		182 (58-414)		12 (4-27)	
2006	Cambodia	1199 (851-1250)		78 (55-81)		188 (62-423)		12 (4-27)		167 (55-376)		11 (4-24)	
2007	Cambodia	1117 (792-1164)		71 (50-74)		173 (59-385)		11 (4-24)		154 (53-342)		10 (3-22)	
2008	Cambodia	1037 (735-1080)		65 (46-68)		160 (57-351)		10 (4-22)		143 (51-312)		9 (3-20)	
2009	Cambodia	946 (671-986)		58 (41-61)		146 (54-316)		9 (3-19)		130 (48-281)		8 (3-17)	
2010	Cambodia	841 (596-876)		51 (36-53)		130 (50-276)		8 (3-17)		115 (44-245)		7 (3-15)	
2011	Cambodia	752 (533-784)		44 (31-46)		115 (45-243)		7 (3-14)		103 (40-216)		6 (2-13)	
2012	Cambodia	657 (466-685)		38 (27-40)		100 (39-209)		6 (2-12)		89 (35-186)		5 (2-11)	
2013	Cambodia	594 (422-620)		34 (24-36)		90 (36-186)		5 (2-11)		80 (32-166)		5 (2-10)	
2014	Cambodia	548 (388-571)		32 (22-33)		82 (33-169)		5 (2-10)		73 (30-151)		4 (2-9)	
2015	Cambodia	349 (247-364)	6 (4-6)	20 (14-21)	0 (0-0)	49 (15-121)	1 (0-2)	3 (1-7)	0 (0-0)	44 (14-108)	1 (0-2)	3 (1-6)	0 (0-0)
2000	Cameroon	4934 (3499-5142)		182 (129-190)		745 (384-1465)		27 (14-54)		234 (121-461)		9 (4-17)	
2001	Cameroon	4771 (3383-4973)		172 (122-179)		718 (250-1612)		26 (9-58)		226 (79-507)		8 (3-18)	
2002	Cameroon	4738 (3360-4938)		167 (118-174)		711 (255-1586)		25 (9-56)		224 (80-499)		8 (3-18)	
2003	Cameroon	4600 (3262-4794)		158 (112-165)		690 (256-1525)		24 (9-52)		217 (80-479)		7 (3-16)	
2004	Cameroon	4488 (3182-4677)		151 (107-157)		672 (257-1474)		23 (9-49)		211 (81-463)		7 (3-16)	
2005	Cameroon	4464 (3165-4652)		146 (104-153)		668 (250-1471)		22 (8-48)		210 (79-463)		7 (3-15)	
2006	Cameroon	4553 (3228-4745)		145 (103-151)		680 (249-1507)		22 (8-48)		214 (78-474)		7 (3-15)	
2007	Cameroon	4603 (3264-4797)		144 (102-150)		687 (250-1527)		21 (8-48)		216 (79-480)		7 (2-15)	
2008	Cameroon	4770 (3383-4971)		146 (104-152)		710 (257-1585)		22 (8-49)		223 (81-498)		7 (2-15)	
2009	Cameroon	5010 (3553-5221)		151 (107-157)		743 (267-1665)		22 (8-50)		234 (84-523)		7 (3-16)	
2010	Cameroon	5066 (3593-5280)		150 (106-156)		750 (267-1683)		22 (8-50)		236 (84-529)		7 (2-16)	
2011	Cameroon	4654 (3300-4850)		135 (96-141)		677 (239-1526)		20 (7-44)		213 (75-480)		6 (2-14)	
2012	Cameroon	3341 (2369-3482)		95 (68-99)		462 (162-1043)		13 (5-30)		145 (51-328)		4 (1-9)	
2013	Cameroon	2771 (1965-2888)		78 (55-81)		384 (134-870)		11 (4-24)		121 (42-274)		3 (1-8)	
2014	Cameroon	2492 (1767-2597)		69 (49-72)		358 (124-812)		10 (3-22)		112 (39-255)		3 (1-7)	
2015	Cameroon	1739 (1233-1812)	335 (237-349)	47 (34-49)	9 (6-9)	241 (83-548)	46 (16-105)	7 (2-15)	1 (0-3)	76 (26-172)	15 (5-33)	2 (1-5)	0 (0-1)
2000	Canada	18 (13-18)		1 (1-1)		17 (5-30)		1 (0-2)		15 (5-27)		1 (0-2)	
2001	Canada	18 (12-18)		1 (1-1)		13 (4-23)		1 (0-1)		11 (4-21)		1 (0-1)	
2002	Canada	14 (10-14)		1 (1-1)		12 (4-21)		1 (0-1)		11 (3-19)		1 (0-1)	
2003	Canada	15 (10-15)		1 (1-1)		12 (4-22)		1 (0-1)		11 (3-19)		1 (0-1)	
2004	Canada	12 (8-12)		1 (0-1)		10 (3-19)		1 (0-1)		9 (3-17)		1 (0-1)	
2005	Canada	12 (8-12)		1 (0-1)		10 (3-18)		1 (0-1)		9 (3-16)		1 (0-1)	
2006	Canada	14 (10-14)		1 (1-1)		12 (4-21)		1 (0-1)		10 (3-19)		1 (0-1)	
2007	Canada	18 (13-19)		1 (1-1)		11 (3-19)		1 (0-1)		10 (3-17)		1 (0-1)	
2008	Canada	9 (6-9)		0 (0-1)		4 (1-8)		0 (0-0)		4 (1-7)		0 (0-0)	
2009	Canada	14 (10-15)		1 (1-1)		12 (4-22)		1 (0-1)		11 (3-19)		1 (0-1)	
2010	Canada	10 (7-11)		1 (0-1)		10 (3-18)		1 (0-1)		9 (3-16)		0 (0-1)	
2011	Canada	6 (4-6)		0 (0-0)		4 (1-7)		0 (0-0)		4 (1-7)		0 (0-0)	
2012	Canada	5 (3-5)		0 (0-0)		4 (1-7)		0 (0-0)		3 (1-6)		0 (0-0)	
2013	Canada	4 (3-4)		0 (0-0)		3 (1-6)		0 (0-0)		3 (1-5)		0 (0-0)	
2014	Canada	3 (2-3)		0 (0-0)		3 (1-5)		0 (0-0)		3 (1-5)		0 (0-0)	
2015	Canada	3 (2-3)	0 (0-0)	0 (0-0)	0 (0-0)	3 (1-5)	0 (0-0)	0 (0-0)	0 (0-0)	2 (1-4)	0 (0-0)	0 (0-0)	0 (0-0)
2000	Cape Verde	22 (16-23)		36 (26-38)		2 (1-3)		4 (2-5)		2 (1-3)		3 (2-5)	
2001	Cape Verde	19 (14-20)		32 (22-33)		2 (1-3)		3 (2-5)		2 (1-3)		3 (1-4)	
2002	Cape Verde	17 (12-18)		28 (20-30)		2 (1-3)		3 (2-5)		2 (1-3)		3 (2-4)	
2003	Cape Verde	15 (11-16)		26 (19-28)		2 (1-3)		3 (2-4)		2 (1-2)		3 (2-4)	
2004	Cape Verde	14 (10-15)		26 (18-27)		2 (1-2)		3 (2-4)		2 (1-2)		3 (2-4)	
2005	Cape Verde	14 (10-15)		26 (18-27)		2 (1-2)		3 (2-4)		2 (1-2)		3 (2-4)	
2006	Cape Verde	14 (10-15)		27 (19-28)		2 (1-2)		3 (2-5)		2 (1-2)		3 (2-4)	
2007	Cape Verde	14 (10-15)		28 (20-29)		2 (1-2)		3 (2-5)		2 (1-2)		3 (2-4)	
2008	Cape Verde	14 (10-15)		28 (20-29)		2 (1-2)		4 (2-5)		2 (1-2)		3 (2-4)	
2009	Cape Verde	14 (10-15)		27 (19-29)		2 (1-2)		4 (2-5)		2 (1-2)		3 (2-4)	
2010	Cape Verde	14 (10-14)		27 (19-28)		2 (1-2)		3 (2-5)		2 (1-2)		3 (2-4)	
2011	Cape Verde	15 (10-15)		28 (20-29)		3 (2-4)		5 (3-7)		2 (2-3)		5 (3-6)	
2012	Cape Verde	14 (10-15)		27 (19-28)		3 (2-4)		5 (3-7)		2 (1-3)		4 (3-6)	
2013	Cape Verde	14 (10-14)		26 (19-28)		3 (2-3)		5 (3-7)		2 (1-3)		4 (3-6)	
2014	Cape Verde	13 (9-14)		25 (18-26)		2 (2-3)		5 (3-6)		2 (1-3)		4 (3-5)	
2015	Cape Verde	13 (9-13)	0 (0-1)	24 (17-25)	1 (1-1)	2 (1-3)	0 (0-0)	4 (3-6)	0 (0-0)	2 (1-3)	0 (0-0)	4 (2-5)	0 (0-0)
2000	Central African Republic	891 (632-929)		150 (106-156)		192 (101-367)		32 (17-62)		60 (32-115)		10 (5-19)	
2001	Central African Republic	899 (638-937)		147 (105-154)		193 (102-371)		32 (17-61)		61 (32-116)		10 (5-19)	
2002	Central African Republic	911 (646-950)		147 (104-154)		196 (104-376)		32 (17-61)		62 (33-118)		10 (5-19)	
2003	Central African Republic	891 (632-929)		143 (102-149)		193 (102-369)		31 (16-59)		61 (32-116)		10 (5-19)	
2004	Central African Republic	868 (616-905)		139 (98-145)		188 (99-359)		30 (16-57)		59 (31-113)		9 (5-18)	
2005	Central African Republic	843 (598-879)		134 (95-140)		182 (96-348)		29 (15-55)		57 (30-109)		9 (5-17)	
2006	Central African Republic	879 (623-916)		139 (98-145)		188 (99-360)		30 (16-57)		59 (31-113)		9 (5-18)	
2007	Central African Republic	919 (652-958)		145 (103-151)		194 (102-372)		31 (16-59)		61 (32-117)		10 (5-18)	
2008	Central African Republic	950 (674-990)		149 (106-156)		197 (104-380)		31 (16-60)		62 (33-119)		10 (5-19)	
2009	Central African Republic	1029 (730-1072)		161 (114-168)		210 (110-406)		33 (17-64)		66 (35-128)		10 (5-20)	
2010	Central African Republic	1081 (767-1127)		168 (119-175)		219 (115-426)		34 (18-66)		69 (36-134)		11 (6-21)	
2011	Central African Republic	1091 (773-1137)		167 (119-174)		220 (77-493)		34 (12-76)		69 (24-155)		11 (4-24)	
2012	Central African Republic	916 (650-955)		139 (98-144)		182 (64-411)		28 (10-62)		57 (20-129)		9 (3-20)	
2013	Central African Republic	995 (706-1037)		148 (105-154)		207 (72-467)		31 (11-69)		65 (23-147)		10 (3-22)	
2014	Central African Republic	893 (633-930)		130 (92-136)		189 (66-428)		28 (10-62)		59 (21-134)		9 (3-20)	

Spn = *Streptococcus pneumoniae*; npnm = non-pneumonia, non-meningitis; HIV = deaths only in HIV-infected children; Uncertainty range provided in parentheses.

Year	Country	Spn pneumonia deaths (UR)	Spn pneumonia deaths, HIV (UR)	Spn pneumonia mortality rate (UR)	Spn pneumonia mortality rate, HIV (UR)	Spn meningitis deaths (UR)	Spn meningitis deaths, HIV (UR)	Spn meningitis mortality rate (UR)	Spn meningitis mortality rate, HIV (UR)	Spn NPNM deaths (UR)	Spn NPNM deaths, HIV (UR)	Spn NPNM mortality rate (UR)	Spn NPNM mortality rate, HIV (UR)
2015	Central African Republic	856 (607-892)	213 (151-222)	123 (87-128)	31 (22-32)	183 (64-416)	46 (16-103)	26 (9-60)	7 (2-15)	58 (20-131)	14 (5-33)	8 (3-19)	2 (1-5)
2000	Chad	4851 (3440-5055)		292 (207-305)		906 (462-1799)		55 (28-108)		285 (145-566)		17 (9-34)	
2001	Chad	4956 (3515-5165)		287 (204-299)		911 (459-1819)		53 (27-105)		286 (144-572)		17 (8-33)	
2002	Chad	5366 (3805-5592)		300 (212-312)		961 (478-1928)		54 (27-108)		302 (150-606)		17 (8-34)	
2003	Chad	5402 (3831-5630)		291 (206-303)		937 (461-1889)		51 (25-102)		295 (145-594)		16 (8-32)	
2004	Chad	5598 (3970-5834)		292 (207-304)		940 (457-1903)		49 (24-99)		296 (144-598)		15 (7-31)	
2005	Chad	5788 (4104-6032)		292 (207-305)		951 (468-1919)		48 (24-97)		299 (147-603)		15 (7-30)	
2006	Chad	6167 (4373-6427)		301 (214-314)		1003 (498-2012)		49 (24-98)		315 (157-633)		15 (8-31)	
2007	Chad	6115 (4336-6373)		290 (206-303)		990 (496-1973)		47 (24-94)		311 (156-620)		15 (7-29)	
2008	Chad	6402 (4539-6671)		296 (210-309)		1036 (524-2051)		48 (24-95)		326 (165-645)		15 (8-30)	
2009	Chad	6268 (4444-6532)		283 (201-295)		1014 (518-1994)		46 (23-90)		319 (163-627)		14 (7-28)	
2010	Chad	6331 (4490-6598)		278 (197-290)		1022 (527-1997)		45 (23-88)		321 (166-628)		14 (7-28)	
2011	Chad	6376 (4521-6644)		272 (193-284)		1027 (536-1996)		44 (23-85)		323 (168-628)		14 (7-27)	
2012	Chad	6389 (4530-6658)		266 (188-277)		1023 (541-1982)		43 (22-82)		322 (170-623)		13 (7-26)	
2013	Chad	6426 (4557-6697)		261 (185-272)		1022 (367-2263)		41 (15-92)		321 (115-711)		13 (5-29)	
2014	Chad	6391 (4532-6661)		253 (179-264)		1010 (371-2229)		40 (15-88)		317 (116-701)		13 (5-28)	
2015	Chad	6347 (4500-6614)	1020 (723-1063)	245 (174-256)	39 (28-41)	997 (374-2192)	160 (60-352)	38 (14-85)	6 (2-14)	313 (118-689)	50 (19-111)	12 (5-27)	2 (1-4)
2000	Chile	102 (72-106)		8 (6-8)		30 (15-47)		2 (1-4)		27 (13-42)		2 (1-3)	
2001	Chile	113 (80-118)		9 (6-9)		42 (20-64)		3 (2-5)		37 (18-57)		3 (1-5)	
2002	Chile	55 (39-57)		4 (3-5)		30 (15-46)		2 (1-4)		27 (13-41)		2 (1-3)	
2003	Chile	54 (38-56)		4 (3-5)		31 (15-48)		3 (1-4)		28 (14-43)		2 (1-3)	
2004	Chile	52 (37-54)		4 (3-4)		26 (13-40)		2 (1-3)		23 (11-35)		2 (1-3)	
2005	Chile	45 (32-47)		4 (3-4)		28 (14-43)		2 (1-4)		25 (12-38)		2 (1-3)	
2006	Chile	34 (24-35)		3 (2-3)		17 (8-26)		1 (1-2)		15 (7-24)		1 (1-2)	
2007	Chile	41 (29-42)		3 (2-4)		14 (7-21)		1 (1-2)		12 (6-19)		1 (1-2)	
2008	Chile	46 (32-47)		4 (3-4)		15 (7-23)		1 (1-2)		13 (7-21)		1 (1-2)	
2009	Chile	44 (31-46)		4 (3-4)		11 (6-18)		1 (0-1)		10 (5-16)		1 (0-1)	
2010	Chile	33 (24-35)		3 (2-3)		19 (9-30)		2 (1-3)		17 (8-26)		1 (1-2)	
2011	Chile	18 (13-18)		2 (1-2)		10 (5-15)		1 (0-1)		8 (4-13)		1 (0-1)	
2012	Chile	15 (10-15)		1 (1-1)		10 (5-15)		1 (0-1)		8 (4-13)		1 (0-1)	
2013	Chile	16 (11-17)		1 (1-1)		5 (2-7)		0 (0-1)		4 (2-6)		0 (0-1)	
2014	Chile	12 (9-13)		1 (1-1)		6 (3-10)		1 (0-1)		6 (3-9)		0 (0-1)	
2015	Chile	9 (7-10)	0 (0-0)	1 (1-1)	0 (0-0)	5 (2-7)	0 (0-0)	0 (0-1)	0 (0-0)	4 (2-6)	0 (0-0)	0 (0-1)	0 (0-0)
2000	China	22713 (16106-23670)		29 (21-30)		2882 (1348-4374)		4 (2-6)		2566 (1201-3894)		3 (2-5)	
2001	China	19466 (13803-20286)		25 (18-26)		2497 (1147-3720)		3 (1-5)		2223 (1021-3312)		3 (1-4)	
2002	China	16759 (11884-17465)		22 (16-23)		2197 (980-3179)		3 (1-4)		1956 (873-2830)		3 (1-4)	
2003	China	14281 (10126-14883)		19 (14-20)		1990 (1369-3146)		3 (2-4)		1772 (1219-2801)		2 (2-4)	
2004	China	12283 (8710-12801)		17 (12-17)		1809 (1176-2701)		2 (2-4)		1610 (1047-2405)		2 (1-3)	
2005	China	10736 (7613-11189)		14 (10-15)		1725 (1067-2452)		2 (1-3)		1536 (950-2183)		2 (1-3)	
2006	China	9824 (6966-10238)		13 (9-13)		1732 (1040-2389)		2 (1-3)		1542 (926-2127)		2 (1-3)	
2007	China	9131 (6475-9516)		12 (8-12)		1701 (1021-2346)		2 (1-3)		1515 (909-2089)		2 (1-3)	
2008	China	8604 (6101-8966)		11 (8-12)		1743 (1046-2404)		2 (1-3)		1552 (931-2140)		2 (1-3)	
2009	China	8067 (5720-8407)		10 (7-11)		1710 (1027-2359)		2 (1-3)		1523 (914-2100)		2 (1-3)	
2010	China	7614 (5399-7934)		10 (7-10)		1681 (1009-2318)		2 (1-3)		1496 (898-2064)		2 (1-3)	
2011	China	7019 (4977-7315)		9 (6-9)		1579 (947-2177)		2 (1-3)		1405 (844-1938)		2 (1-2)	
2012	China	6490 (4602-6763)		8 (6-9)		1427 (856-1968)		2 (1-2)		1270 (763-1752)		2 (1-2)	
2013	China	6012 (4263-6265)		7 (5-8)		1306 (784-1802)		2 (1-2)		1163 (698-1604)		1 (1-2)	
2014	China	5545 (3932-5779)		7 (5-7)		1176 (706-1621)		1 (1-2)		1047 (628-1443)		1 (1-2)	
2015	China	5257 (3728-5479)	6 (4-6)	6 (5-7)	0 (0-0)	1114 (669-1537)	1 (1-2)	1 (1-2)	0 (0-0)	992 (595-1368)	1 (1-2)	1 (1-2)	0 (0-0)
2000	Colombia	906 (643-945)		22 (16-23)		145 (26-473)		4 (1-12)		129 (23-421)		3 (1-10)	
2001	Colombia	785 (556-818)		19 (14-20)		133 (25-433)		3 (1-11)		119 (22-385)		3 (1-10)	
2002	Colombia	623 (442-649)		15 (11-16)		137 (26-441)		3 (1-11)		122 (23-393)		3 (1-10)	
2003	Colombia	691 (490-720)		17 (12-18)		145 (29-464)		4 (1-11)		129 (26-413)		3 (1-10)	
2004	Colombia	700 (496-729)		17 (12-18)		135 (28-425)		3 (1-11)		120 (25-379)		3 (1-9)	
2005	Colombia	681 (483-709)		17 (12-18)		149 (32-468)		4 (1-12)		133 (28-417)		3 (1-10)	
2006	Colombia	711 (504-741)		18 (12-18)		142 (31-444)		4 (1-11)		127 (27-395)		3 (1-10)	
2007	Colombia	659 (468-687)		16 (12-17)		104 (23-324)		3 (1-8)		93 (20-289)		2 (0-7)	
2008	Colombia	568 (403-592)		14 (10-15)		96 (21-299)		2 (1-8)		86 (19-266)		2 (0-7)	
2009	Colombia	534 (379-557)		14 (10-14)		96 (21-297)		2 (1-8)		86 (19-265)		2 (0-7)	
2010	Colombia	456 (323-475)		12 (8-12)		73 (16-225)		2 (0-6)		65 (14-200)		2 (0-5)	
2011	Colombia	424 (301-442)		11 (8-12)		58 (13-179)		2 (0-5)		52 (12-160)		1 (0-4)	
2012	Colombia	325 (231-339)		9 (6-9)		35 (8-107)		1 (0-3)		31 (7-95)		1 (0-3)	
2013	Colombia	278 (197-289)		7 (5-8)		37 (8-112)		1 (0-3)		33 (7-100)		1 (0-3)	
2014	Colombia	200 (142-208)		5 (4-6)		26 (6-79)		1 (0-2)		23 (5-70)		1 (0-2)	
2015	Colombia	166 (118-173)	0 (0-0)	5 (3-5)	0 (0-0)	22 (5-66)	0 (0-0)	1 (0-2)	0 (0-0)	19 (4-58)	0 (0-0)	1 (0-2)	0 (0-0)
2000	Comoros	89 (63-93)		101 (71-105)		16 (7-33)		18 (8-38)		5 (2-10)		6 (3-12)	
2001	Comoros	90 (64-94)		100 (71-105)		16 (7-34)		18 (8-38)		5 (2-11)		6 (2-12)	
2002	Comoros	93 (66-97)		101 (72-105)		17 (7-35)		18 (8-38)		5 (2-11)		6 (2-12)	
2003	Comoros	94 (67-98)		101 (71-105)		17 (7-35)		18 (8-38)		5 (2-11)		6 (2-12)	
2004	Comoros	95 (67-99)		100 (71-104)		17 (7-36)		18 (7-38)		5 (2-11)		6 (2-12)	
2005	Comoros	94 (67-98)		97 (69-102)		17 (7-36)		18 (7-37)		5 (2-11)		6 (2-12)	
2006	Comoros	93 (66-97)		95 (67-99)		17 (7-36)		17 (7-36)		5 (2-11)		5 (2-11)	
2007	Comoros	92 (66-96)		92 (65-96)		17 (7-35)		16 (7-35)		5 (2-11)		5 (2-11)	
2008	Comoros	92 (65-96)		89 (63-93)		16 (6-35)		16 (6-34)		5 (2-11)		5 (2-11)	
2009	Comoros	90 (64-94)		86 (61-89)		16 (6-35)		15 (6-33)		5 (2-11)		5 (2-10)	
2010	Comoros	88 (62-92)		82 (58-86)		16 (6-34)		15 (6-32)		5 (2-11)		5 (2-10)	
2011	Comoros	86 (61-89)		79 (56-82)		15 (6-34)		14 (5-31)		5 (2-11)		4 (2-10)	
2012	Comoros	83 (59-87)		75 (53-78)		15 (6-33)		13 (5-29)		5 (2-10)		4 (2-9)	
2013	Comoros	81 (57-84)		71 (51-74)		15 (5-32)		13 (5-28)		5 (2-10)		4 (1-9)	
2014	Comoros	79 (56-83)		69 (49-71)		14 (5-31)		12 (5-27)		4 (2-10)		4 (1-9)	
2015	Comoros	77 (55-80)	0 (0-0)	66 (47-68)	0 (0-0)	14 (4-32)	0 (0-0)	12 (4-27)	0 (0-0)	12 (4-28)	0 (0-0)	10 (3-24)	0 (0-0)
2000	Congo	650 (461-677)		131 (93-136)		83 (32-178)		17 (6-36)		26 (10-56)		5 (2-11)	
2001	Congo	673 (477-702)		131 (93-137)		86 (34-186)		17 (6-36)		27 (11-58)		5 (2-11)	
2002	Congo	676 (479-704)		128 (91-134)		87 (34-187)		17 (7-36)		27 (11-59)		5 (2-11)	
2003	Congo	663 (470-691)		123 (87-128)		86 (34-185)		16 (6-34)		27 (11-58)		5 (2-11)	
2004	Congo	646 (458-673)		117 (83-122)		86 (34-182)		16 (6-33)		27 (11-57)		5 (2-10)	
2005	Congo	623 (442-649)		110 (78-114)		84 (34-177)		15 (6-31)		26 (11-56)		5 (2-10)	
2006	Congo	595 (422-620)		102 (72-106)		80 (33-170)		14 (6-29)		25 (10-53)		4 (2-9)	

Spn = *Streptococcus pneumoniae*; npnm = non-pneumonia, non-meningitis; HIV = deaths only in HIV-infected children; Uncertainty range provided in parentheses.

Year	Country	Spn pneumonia deaths (UR)	Spn pneumonia deaths, HIV (UR)	Spn pneumonia mortality rate (UR)	Spn pneumonia mortality rate, HIV (UR)	Spn meningitis deaths (UR)	Spn meningitis deaths, HIV (UR)	Spn meningitis mortality rate (UR)	Spn meningitis mortality rate, HIV (UR)	Spn NPNM deaths (UR)	Spn NPNM deaths, HIV (UR)	Spn NPNM mortality rate (UR)	Spn NPNM mortality rate, HIV (UR)
2007	Congo	567 (402-591)		94 (66-98)		77 (32-162)		13 (5-27)		24 (10-51)		4 (2-8)	
2008	Congo	535 (379-557)		86 (61-89)		72 (25-156)		11 (4-25)		64 (22-139)		10 (4-22)	
2009	Congo	498 (353-519)		77 (55-81)		66 (23-143)		10 (4-22)		59 (21-127)		9 (3-20)	
2010	Congo	462 (328-482)		70 (49-73)		60 (21-131)		9 (3-20)		54 (19-116)		8 (3-18)	
2011	Congo	437 (310-455)		64 (45-67)		56 (20-121)		8 (3-18)		50 (18-108)		7 (3-16)	
2012	Congo	403 (285-420)		57 (41-60)		50 (18-109)		7 (3-15)		45 (16-97)		6 (2-14)	
2013	Congo	270 (192-282)		38 (27-39)		31 (11-68)		4 (2-9)		28 (10-60)		4 (1-8)	
2014	Congo	224 (159-234)		31 (22-32)		26 (9-56)		4 (1-8)		23 (8-50)		3 (1-7)	
2015	Congo	203 (144-212)	43 (31-45)	27 (19-28)	6 (4-6)	24 (9-52)	5 (2-11)	3 (1-7)	1 (0-1)	22 (8-47)	5 (2-10)	3 (1-6)	1 (0-1)
2000	Cook Islands	0 (0-0)		11 (8-12)		0 (0-0)		1 (1-2)		0 (0-0)		1 (1-2)	
2001	Cook Islands	0 (0-0)		12 (8-12)		0 (0-0)		1 (1-2)		0 (0-0)		1 (1-2)	
2002	Cook Islands	0 (0-0)		12 (8-12)		0 (0-0)		1 (1-3)		0 (0-0)		1 (1-2)	
2003	Cook Islands	0 (0-0)		8 (6-8)		0 (0-0)		1 (0-2)		0 (0-0)		1 (0-2)	
2004	Cook Islands	0 (0-0)		8 (6-8)		0 (0-0)		1 (0-2)		0 (0-0)		1 (0-2)	
2005	Cook Islands	0 (0-0)		12 (8-12)		0 (0-0)		1 (1-2)		0 (0-0)		1 (1-2)	
2006	Cook Islands	0 (0-0)		7 (5-8)		0 (0-0)		1 (0-2)		0 (0-0)		1 (0-1)	
2007	Cook Islands	0 (0-0)		7 (5-7)		0 (0-0)		1 (0-1)		0 (0-0)		1 (0-1)	
2008	Cook Islands	0 (0-0)		6 (5-7)		0 (0-0)		1 (0-1)		0 (0-0)		1 (0-1)	
2009	Cook Islands	0 (0-0)		7 (5-7)		0 (0-0)		1 (1-3)		0 (0-0)		1 (1-2)	
2010	Cook Islands	0 (0-0)		6 (5-7)		0 (0-0)		2 (1-3)		0 (0-0)		1 (1-2)	
2011	Cook Islands	0 (0-0)		6 (4-6)		0 (0-0)		1 (1-3)		0 (0-0)		1 (1-2)	
2012	Cook Islands	0 (0-0)		3 (2-3)		0 (0-0)		1 (0-1)		0 (0-0)		1 (0-1)	
2013	Cook Islands	0 (0-0)		3 (2-3)		0 (0-0)		1 (0-1)		0 (0-0)		1 (0-1)	
2014	Cook Islands	0 (0-0)		3 (2-3)		0 (0-0)		1 (0-1)		0 (0-0)		1 (0-1)	
2015	Cook Islands	0 (0-0)	0 (0-0)	3 (2-3)	0 (0-0)	0 (0-0)	0 (0-0)	1 (0-1)	0 (0-0)	0 (0-0)	0 (0-0)	1 (0-1)	0 (0-0)
2000	Costa Rica	24 (17-25)		6 (4-6)		4 (1-10)		1 (0-3)		3 (1-9)		1 (0-2)	
2001	Costa Rica	19 (14-20)		5 (4-5)		3 (1-8)		1 (0-2)		3 (1-8)		1 (0-2)	
2002	Costa Rica	17 (12-18)		4 (3-5)		6 (2-17)		2 (0-5)		5 (2-15)		1 (0-4)	
2003	Costa Rica	15 (10-15)		4 (3-4)		2 (1-6)		1 (0-2)		2 (1-5)		0 (0-1)	
2004	Costa Rica	14 (10-14)		4 (3-4)		1 (0-4)		0 (0-1)		1 (0-3)		0 (0-1)	
2005	Costa Rica	17 (12-18)		5 (3-5)		3 (1-8)		1 (0-2)		3 (1-7)		1 (0-2)	
2006	Costa Rica	10 (7-10)		3 (2-3)		3 (1-7)		1 (0-2)		2 (1-6)		1 (0-2)	
2007	Costa Rica	13 (9-14)		4 (3-4)		3 (1-8)		1 (0-2)		3 (1-7)		1 (0-2)	
2008	Costa Rica	8 (6-9)		2 (2-2)		4 (1-12)		1 (0-3)		4 (1-10)		1 (0-3)	
2009	Costa Rica	8 (5-8)		2 (2-2)		3 (1-8)		1 (0-2)		3 (1-8)		1 (0-2)	
2010	Costa Rica	8 (6-9)		2 (2-2)		3 (1-7)		1 (0-2)		2 (1-6)		1 (0-2)	
2011	Costa Rica	8 (6-8)		2 (2-2)		3 (1-8)		1 (0-2)		2 (1-7)		1 (0-2)	
2012	Costa Rica	5 (3-5)		1 (1-1)		3 (1-7)		1 (0-2)		2 (1-6)		1 (0-2)	
2013	Costa Rica	6 (4-6)		2 (1-2)		1 (0-3)		0 (0-1)		1 (0-2)		0 (0-1)	
2014	Costa Rica	3 (2-4)		1 (1-1)		1 (0-3)		0 (0-1)		1 (0-2)		0 (0-1)	
2015	Costa Rica	3 (2-3)	0 (0-0)	1 (1-1)	0 (0-0)	1 (0-2)	0 (0-0)	0 (0-1)	0 (0-0)	1 (0-2)	0 (0-0)	0 (0-1)	0 (0-0)
2000	Cote d'Ivoire	2369 (1680-2468)		85 (60-88)		335 (126-736)		12 (4-26)		105 (40-231)		4 (1-8)	
2001	Cote d'Ivoire	2338 (1658-2436)		81 (58-85)		333 (125-734)		12 (4-26)		105 (39-231)		4 (1-8)	
2002	Cote d'Ivoire	2286 (1621-2383)		78 (56-82)		329 (122-724)		11 (4-25)		103 (38-228)		4 (1-8)	
2003	Cote d'Ivoire	2237 (1586-2331)		76 (54-79)		324 (120-715)		11 (4-24)		102 (38-225)		3 (1-8)	
2004	Cote d'Ivoire	2179 (1545-2271)		73 (52-76)		317 (117-701)		11 (4-23)		100 (37-220)		3 (1-7)	
2005	Cote d'Ivoire	2200 (1560-2293)		73 (52-76)		321 (118-710)		11 (4-23)		101 (37-223)		3 (1-7)	
2006	Cote d'Ivoire	2260 (1602-2355)		74 (52-77)		329 (120-728)		11 (4-24)		103 (38-229)		3 (1-7)	
2007	Cote d'Ivoire	2392 (1696-2493)		77 (55-81)		347 (128-767)		11 (4-25)		109 (40-241)		4 (1-8)	
2008	Cote d'Ivoire	2506 (1777-2611)		80 (57-83)		363 (134-801)		12 (4-26)		114 (42-252)		4 (1-8)	
2009	Cote d'Ivoire	2734 (1938-2849)		86 (61-89)		396 (147-872)		12 (5-27)		124 (46-274)		4 (1-9)	
2010	Cote d'Ivoire	2940 (2085-3064)		91 (64-94)		425 (159-935)		13 (5-29)		134 (50-294)		4 (2-9)	
2011	Cote d'Ivoire	3131 (2220-3263)		95 (67-99)		452 (170-992)		14 (5-30)		142 (53-312)		4 (2-9)	
2012	Cote d'Ivoire	3241 (2298-3378)		96 (68-100)		467 (175-1023)		14 (5-30)		147 (55-322)		4 (2-10)	
2013	Cote d'Ivoire	3375 (2393-3517)		98 (69-102)		484 (183-1060)		14 (5-31)		152 (57-333)		4 (2-10)	
2014	Cote d'Ivoire	3426 (2429-3571)		97 (69-101)		491 (186-1072)		14 (5-30)		154 (59-337)		4 (2-10)	
2015	Cote d'Ivoire	2525 (1791-2632)	366 (259-381)	70 (50-73)	10 (7-11)	343 (131-748)	50 (19-108)	10 (4-21)	1 (1-3)	108 (41-235)	16 (6-34)	3 (1-7)	0 (0-1)
2000	Croatia	4 (3-4)		2 (1-2)		2 (1-3)		1 (0-1)		1 (1-3)		1 (0-1)	
2001	Croatia	2 (2-3)		1 (1-1)		2 (1-3)		1 (0-1)		2 (1-3)		1 (0-1)	
2002	Croatia	1 (1-2)		1 (0-1)		2 (1-4)		1 (0-2)		2 (1-3)		1 (0-1)	
2003	Croatia	3 (2-3)		1 (1-1)		1 (0-2)		0 (0-1)		1 (0-2)		0 (0-1)	
2004	Croatia	1 (0-1)		0 (0-0)		0 (0-1)		0 (0-0)		0 (0-1)		0 (0-0)	
2005	Croatia	3 (2-4)		2 (1-2)		3 (1-5)		1 (1-3)		3 (1-5)		1 (1-2)	
2006	Croatia	2 (2-2)		1 (1-1)		0 (0-1)		0 (0-0)		0 (0-1)		0 (0-0)	
2007	Croatia	3 (2-3)		2 (1-2)		0 (0-0)		0 (0-0)		0 (0-0)		0 (0-0)	
2008	Croatia	2 (2-2)		1 (1-1)		1 (1-2)		1 (0-1)		1 (1-2)		1 (0-1)	
2009	Croatia	2 (1-2)		1 (1-1)		2 (1-4)		1 (0-2)		2 (1-3)		1 (0-2)	
2010	Croatia	2 (1-2)		1 (1-1)		2 (1-4)		1 (0-2)		2 (1-3)		1 (0-2)	
2011	Croatia	0 (0-0)		0 (0-0)		2 (1-3)		1 (0-1)		2 (1-3)		1 (0-1)	
2012	Croatia	1 (0-1)		0 (0-0)		3 (1-5)		1 (1-2)		3 (1-5)		1 (1-2)	
2013	Croatia	3 (2-3)		1 (1-1)		1 (0-2)		0 (0-1)		1 (0-2)		0 (0-1)	
2014	Croatia	1 (1-1)		1 (0-1)		2 (1-3)		1 (0-1)		1 (1-3)		1 (0-1)	
2015	Croatia	1 (1-1)	0 (0-0)	0 (0-0)	0 (0-0)	2 (1-3)	0 (0-0)	1 (0-1)	0 (0-0)	1 (1-2)	0 (0-0)	1 (0-1)	0 (0-0)
2000	Cuba	26 (19-27)		4 (3-4)		24 (12-45)		3 (2-6)		22 (11-40)		3 (1-5)	
2001	Cuba	26 (19-27)		4 (3-4)		18 (9-33)		2 (1-5)		16 (8-30)		2 (1-4)	
2002	Cuba	22 (16-23)		3 (2-3)		9 (5-17)		1 (1-2)		8 (4-15)		1 (1-2)	
2003	Cuba	20 (14-21)		3 (2-3)		14 (7-26)		2 (1-4)		12 (6-23)		2 (1-3)	
2004	Cuba	18 (13-19)		3 (2-3)		11 (6-20)		2 (1-3)		10 (5-18)		1 (1-3)	
2005	Cuba	23 (17-24)		3 (2-4)		14 (7-25)		2 (1-4)		12 (6-23)		2 (1-3)	
2006	Cuba	25 (17-26)		4 (3-4)		11 (6-21)		2 (1-3)		10 (5-18)		2 (1-3)	
2007	Cuba	21 (15-21)		3 (2-3)		8 (4-15)		1 (1-2)		7 (4-14)		1 (1-2)	
2008	Cuba	17 (12-18)		3 (2-3)		11 (6-20)		2 (1-3)		10 (5-18)		2 (1-3)	
2009	Cuba	29 (20-30)		5 (3-5)		7 (3-13)		1 (1-2)		6 (3-11)		1 (0-2)	
2010	Cuba	20 (14-21)		3 (2-3)		8 (4-14)		1 (1-2)		7 (3-12)		1 (1-2)	
2011	Cuba	22 (15-23)		4 (3-4)		11 (6-20)		2 (1-3)		10 (5-18)		2 (1-3)	
2012	Cuba	24 (17-25)		4 (3-4)		11 (6-22)		2 (1-4)		10 (5-20)		2 (1-3)	
2013	Cuba	24 (17-25)		4 (3-4)		8 (4-15)		1 (1-3)		7 (3-14)		1 (1-2)	
2014	Cuba	22 (16-23)		4 (3-4)		9 (4-19)		2 (1-3)		8 (4-17)		1 (1-3)	

Spn = *Streptococcus pneumoniae*; npnm = non-pneumonia, non-meningitis; HIV = deaths only in HIV-infected children; Uncertainty range provided in parentheses.

Year	Country	Spn pneumonia deaths (UR)	Spn pneumonia deaths, HIV (UR)	Spn pneumonia mortality rate (UR)	Spn pneumonia mortality rate, HIV (UR)	Spn meningitis deaths (UR)	Spn meningitis deaths, HIV (UR)	Spn meningitis mortality rate (UR)	Spn meningitis mortality rate, HIV (UR)	Spn NPNM deaths (UR)	Spn NPNM deaths, HIV (UR)	Spn NPNM mortality rate (UR)	Spn NPNM mortality rate, HIV (UR)
2015	Cuba	22 (16-23)	0 (0-0)	4 (3-4)	0 (0-0)	9 (4-19)	0 (0-0)	2 (1-3)	0 (0-0)	8 (4-17)	0 (0-0)	1 (1-3)	0 (0-0)
2000	Cyprus	1 (1-1)		2 (1-2)		1 (0-1)		1 (1-2)		1 (0-1)		1 (1-1)	
2001	Cyprus	1 (1-1)		2 (1-2)		1 (0-1)		1 (1-1)		1 (0-1)		1 (1-1)	
2002	Cyprus	1 (1-1)		2 (1-2)		1 (0-1)		1 (1-1)		1 (0-1)		1 (1-1)	
2003	Cyprus	1 (1-1)		2 (1-2)		1 (0-1)		1 (1-1)		1 (0-1)		1 (1-1)	
2004	Cyprus	1 (1-1)		1 (1-2)		1 (0-1)		1 (1-1)		0 (0-1)		1 (0-1)	
2005	Cyprus	1 (1-1)		1 (1-1)		0 (0-1)		1 (0-1)		0 (0-1)		1 (0-1)	
2006	Cyprus	1 (1-1)		1 (1-1)		0 (0-1)		1 (0-1)		0 (0-1)		1 (0-1)	
2007	Cyprus	1 (0-1)		1 (1-1)		0 (0-1)		1 (0-1)		0 (0-0)		1 (0-1)	
2008	Cyprus	1 (0-1)		1 (1-1)		0 (0-1)		1 (0-1)		0 (0-0)		1 (0-1)	
2009	Cyprus	1 (0-1)		1 (1-1)		0 (0-0)		1 (0-1)		0 (0-0)		0 (0-1)	
2010	Cyprus	0 (0-0)		1 (1-1)		0 (0-0)		0 (0-1)		0 (0-0)		0 (0-1)	
2011	Cyprus	0 (0-0)		1 (0-1)		0 (0-0)		0 (0-1)		0 (0-0)		0 (0-0)	
2012	Cyprus	0 (0-0)		1 (0-1)		0 (0-0)		0 (0-1)		0 (0-0)		0 (0-0)	
2013	Cyprus	0 (0-0)		1 (0-1)		0 (0-0)		0 (0-0)		0 (0-0)		0 (0-0)	
2014	Cyprus	0 (0-0)		1 (0-1)		0 (0-0)		0 (0-0)		0 (0-0)		0 (0-0)	
2015	Cyprus	0 (0-0)	0 (0-0)	0 (0-0)	0 (0-0)	0 (0-0)	0 (0-0)	0 (0-0)	0 (0-0)	0 (0-0)	0 (0-0)	0 (0-0)	0 (0-0)
2000	Czech Republic	6 (4-6)		1 (1-1)		2 (1-4)		1 (0-1)		2 (1-4)		0 (0-1)	
2001	Czech Republic	7 (5-7)		2 (1-2)		2 (1-4)		1 (0-1)		2 (1-4)		1 (0-1)	
2002	Czech Republic	8 (6-9)		2 (1-2)		2 (1-3)		0 (0-1)		1 (1-3)		0 (0-1)	
2003	Czech Republic	8 (6-8)		2 (1-2)		2 (1-3)		0 (0-1)		2 (1-3)		0 (0-1)	
2004	Czech Republic	5 (4-6)		1 (1-1)		4 (2-8)		1 (0-2)		4 (2-7)		1 (0-2)	
2005	Czech Republic	7 (5-8)		2 (1-2)		3 (1-5)		1 (0-1)		2 (1-4)		1 (0-1)	
2006	Czech Republic	10 (7-11)		2 (1-2)		1 (0-2)		0 (0-0)		1 (0-2)		0 (0-0)	
2007	Czech Republic	9 (7-10)		2 (1-2)		5 (2-9)		1 (0-2)		4 (2-8)		1 (0-1)	
2008	Czech Republic	8 (6-8)		1 (1-2)		2 (1-3)		0 (0-1)		2 (1-3)		0 (0-1)	
2009	Czech Republic	6 (4-6)		1 (1-1)		4 (2-8)		1 (0-1)		4 (2-7)		1 (0-1)	
2010	Czech Republic	11 (8-12)		2 (1-2)		1 (1-3)		0 (0-0)		1 (1-2)		0 (0-0)	
2011	Czech Republic	7 (5-8)		1 (1-1)		3 (1-5)		0 (0-1)		2 (1-4)		0 (0-1)	
2012	Czech Republic	8 (6-8)		1 (1-1)		2 (1-3)		0 (0-1)		1 (1-3)		0 (0-0)	
2013	Czech Republic	10 (7-11)		2 (1-2)		2 (1-4)		0 (0-1)		2 (1-3)		0 (0-1)	
2014	Czech Republic	8 (5-8)		1 (1-1)		2 (1-3)		0 (0-1)		2 (1-3)		0 (0-1)	
2015	Czech Republic	7 (5-8)	0 (0-0)	1 (1-1)	0 (0-0)	2 (1-3)	0 (0-0)	0 (0-1)	0 (0-0)	2 (1-3)	0 (0-0)	0 (0-1)	0 (0-0)
2000	Democratic People's Republic of Korea	1380 (978-1438)		71 (50-74)		310 (148-481)		16 (8-25)		276 (132-429)		14 (7-22)	
2001	Democratic People's Republic of Korea	1126 (799-1174)		59 (42-61)		253 (122-397)		13 (6-21)		225 (109-354)		12 (6-19)	
2002	Democratic People's Republic of Korea	917 (650-956)		49 (34-51)		204 (102-316)		11 (5-17)		181 (91-282)		10 (5-15)	
2003	Democratic People's Republic of Korea	762 (540-794)		40 (29-42)		166 (87-264)		9 (5-14)		148 (77-235)		8 (4-12)	
2004	Democratic People's Republic of Korea	659 (467-687)		35 (25-37)		140 (76-231)		7 (4-12)		125 (68-206)		7 (4-11)	
2005	Democratic People's Republic of Korea	613 (435-639)		33 (23-34)		128 (70-218)		7 (4-12)		114 (62-194)		6 (3-10)	
2006	Democratic People's Republic of Korea	598 (424-623)		33 (23-34)		122 (64-215)		7 (4-12)		109 (57-192)		6 (3-10)	
2007	Democratic People's Republic of Korea	593 (420-618)		33 (23-34)		119 (60-216)		7 (3-12)		106 (54-193)		6 (3-11)	
2008	Democratic People's Republic of Korea	574 (407-598)		33 (23-34)		114 (55-212)		6 (3-12)		101 (49-188)		6 (3-11)	
2009	Democratic People's Republic of Korea	557 (395-581)		32 (23-34)		109 (51-208)		6 (3-12)		97 (46-185)		6 (3-11)	
2010	Democratic People's Republic of Korea	536 (380-559)		32 (22-33)		104 (47-201)		6 (3-12)		92 (42-179)		5 (2-11)	
2011	Democratic People's Republic of Korea	517 (366-538)		31 (22-32)		99 (43-196)		6 (3-12)		88 (38-174)		5 (2-10)	
2012	Democratic People's Republic of Korea	498 (353-519)		30 (21-31)		94 (30-228)		6 (2-14)		84 (27-203)		5 (2-12)	
2013	Democratic People's Republic of Korea	487 (345-508)		29 (21-30)		91 (28-224)		5 (2-13)		81 (25-200)		5 (1-12)	
2014	Democratic People's Republic of Korea	475 (337-495)		28 (20-29)		88 (26-220)		5 (1-13)		78 (23-195)		5 (1-11)	
2015	Democratic People's Republic of Korea	458 (325-477)	0 (0-0)	27 (19-28)	0 (0-0)	84 (23-212)	0 (0-0)	5 (1-12)	0 (0-0)	75 (21-189)	0 (0-0)	4 (1-11)	0 (0-0)
2000	Democratic Republic of the Congo	12202 (8652-12716)		137 (97-143)		2828 (1512-5354)		32 (17-60)		889 (475-1683)		10 (5-19)	
2001	Democratic Republic of the Congo	11857 (8408-12357)		129 (91-134)		2769 (1488-5249)		30 (16-57)		870 (468-1650)		9 (5-18)	
2002	Democratic Republic of the Congo	11727 (8316-12222)		124 (88-129)		2742 (1486-5205)		29 (16-55)		862 (467-1636)		9 (5-17)	
2003	Democratic Republic of the Congo	11524 (8172-12010)		118 (84-123)		2679 (995-5827)		27 (10-60)		842 (313-1832)		9 (3-19)	
2004	Democratic Republic of the Congo	11538 (8181-12024)		114 (81-119)		2666 (1002-5804)		26 (10-58)		838 (315-1825)		8 (3-18)	
2005	Democratic Republic of the Congo	11112 (7879-11580)		107 (76-111)		2551 (969-5553)		24 (9-53)		802 (305-1746)		8 (3-17)	
2006	Democratic Republic of the Congo	11369 (8062-11848)		105 (75-110)		2595 (996-5641)		24 (9-52)		816 (313-1774)		8 (3-16)	
2007	Democratic Republic of the Congo	11434 (8107-11915)		103 (73-107)		2599 (1006-5635)		23 (9-51)		817 (316-1772)		7 (3-16)	
2008	Democratic Republic of the Congo	12091 (8574-12601)		106 (75-110)		2735 (1053-5936)		24 (9-52)		860 (331-1866)		8 (3-16)	
2009	Democratic Republic of the Congo	13436 (9527-14002)		115 (81-119)		3023 (1157-6567)		26 (10-56)		950 (364-2065)		8 (3-18)	
2010	Democratic Republic of the Congo	14760 (10466-15382)		123 (87-128)		3299 (1257-7175)		27 (10-60)		1037 (395-2256)		9 (3-19)	
2011	Democratic Republic of the Congo	16072 (11396-16749)		130 (92-135)		3563 (1363-7743)		29 (11-63)		1120 (428-2434)		9 (3-20)	
2012	Democratic Republic of the Congo	16377 (11613-17068)		129 (92-135)		3572 (1371-7753)		28 (11-61)		1123 (431-2438)		9 (3-19)	
2013	Democratic Republic of the Congo	15357 (10890-16005)		118 (84-123)		3291 (1268-7135)		25 (10-55)		1035 (399-2243)		8 (3-17)	
2014	Democratic Republic of the Congo	13144 (9320-13698)		99 (70-103)		2750 (1059-5960)		21 (8-45)		865 (333-1874)		6 (3-14)	
2015	Democratic Republic of the Congo	11423 (8100-11904)	754 (534-785)	84 (59-87)	6 (4-6)	2374 (920-5138)	157 (61-339)	17 (7-38)	1 (0-2)	747 (289-1615)	49 (19-107)	5 (2-12)	0 (0-1)
2000	Denmark	3 (2-3)		1 (1-1)		2 (1-4)		1 (0-1)		2 (1-4)		1 (0-1)	
2001	Denmark	3 (2-3)		1 (1-1)		3 (1-5)		1 (0-2)		2 (1-5)		1 (0-1)	
2002	Denmark	4 (3-4)		1 (1-1)		2 (1-4)		1 (0-1)		2 (1-4)		1 (0-1)	
2003	Denmark	4 (3-4)		1 (1-1)		2 (1-4)		1 (0-1)		2 (1-4)		1 (0-1)	
2004	Denmark	2 (1-2)		1 (0-1)		3 (1-5)		1 (0-1)		2 (1-4)		1 (0-1)	
2005	Denmark	3 (2-3)		1 (1-1)		1 (1-2)		0 (0-1)		1 (1-2)		0 (0-1)	
2006	Denmark	1 (1-1)		0 (0-0)		1 (0-1)		0 (0-0)		0 (0-1)		0 (0-0)	
2007	Denmark	0 (0-1)		0 (0-0)		1 (1-2)		0 (0-1)		1 (1-2)		0 (0-1)	
2008	Denmark	1 (1-1)		0 (0-0)		1 (0-1)		0 (0-0)		1 (0-1)		0 (0-0)	
2009	Denmark	2 (2-2)		1 (1-1)		0 (0-1)		0 (0-0)		0 (0-1)		0 (0-0)	
2010	Denmark	1 (1-1)		0 (0-0)		0 (0-1)		0 (0-0)		0 (0-1)		0 (0-0)	
2011	Denmark	1 (0-1)		0 (0-0)		0 (0-1)		0 (0-0)		0 (0-1)		0 (0-0)	
2012	Denmark	0 (0-1)		0 (0-0)		0 (0-1)		0 (0-0)		0 (0-0)		0 (0-0)	
2013	Denmark	0 (0-0)		0 (0-0)		0 (0-0)		0 (0-0)		0 (0-0)		0 (0-0)	
2014	Denmark	0 (0-0)		0 (0-0)		0 (0-0)		0 (0-0)		0 (0-0)		0 (0-0)	
2015	Denmark	0 (0-0)	0 (0-0)	0 (0-0)	0 (0-0)	0 (0-0)	0 (0-0)	0 (0-0)	0 (0-0)	0 (0-0)	0 (0-0)	0 (0-0)	0 (0-0)
2000	Djibouti	100 (71-104)		100 (71-105)		16 (7-32)		16 (7-32)		5 (2-10)		5 (2-10)	
2001	Djibouti	97 (69-101)		98 (70-102)		16 (7-32)		16 (7-32)		5 (2-10)		5 (2-10)	
2002	Djibouti	95 (67-99)		96 (68-100)		15 (7-31)		16 (7-31)		5 (2-10)		5 (2-10)	
2003	Djibouti	93 (66-97)		94 (67-98)		15 (7-30)		15 (7-30)		5 (2-9)		5 (2-10)	
2004	Djibouti	90 (64-94)		92 (65-96)		15 (7-29)		15 (7-29)		5 (2-9)		5 (2-9)	
2005	Djibouti	87 (62-91)		89 (63-93)		14 (6-28)		14 (7-28)		4 (2-9)		5 (2-9)	
2006	Djibouti	85 (60-88)		85 (61-89)		14 (6-27)		14 (6-27)		4 (2-8)		4 (2-9)	

Spn = *Streptococcus pneumoniae*; npnm = non-pneumonia, non-meningitis; HIV = deaths only in HIV-infected children; Uncertainty range provided in parentheses.

Year	Country	Spn pneumonia deaths (UR)	Spn pneumonia deaths, HIV (UR)	Spn pneumonia mortality rate (UR)	Spn pneumonia mortality rate, HIV (UR)	Spn meningitis deaths (UR)	Spn meningitis deaths, HIV (UR)	Spn meningitis mortality rate (UR)	Spn meningitis mortality rate, HIV (UR)	Spn NPNM deaths (UR)	Spn NPNM deaths, HIV (UR)	Spn NPNM mortality rate (UR)	Spn NPNM mortality rate, HIV (UR)
2007	Djibouti	81 (58-85)		82 (58-86)		13 (6-25)		13 (6-26)		4 (2-8)		4 (2-8)	
2008	Djibouti	78 (55-81)		79 (56-82)		13 (6-24)		13 (7-24)		4 (2-7)		4 (2-8)	
2009	Djibouti	74 (53-77)		76 (54-79)		12 (7-22)		13 (7-23)		4 (2-7)		4 (2-7)	
2010	Djibouti	71 (51-74)		73 (52-76)		12 (7-21)		12 (7-21)		4 (2-7)		4 (2-7)	
2011	Djibouti	70 (49-73)		71 (51-74)		12 (7-20)		12 (7-21)		11 (6-18)		11 (6-18)	
2012	Djibouti	70 (49-73)		71 (50-74)		12 (7-20)		13 (7-20)		11 (6-17)		11 (6-18)	
2013	Djibouti	47 (33-49)		47 (34-49)		8 (4-13)		8 (4-13)		7 (4-11)		7 (4-11)	
2014	Djibouti	40 (29-42)		40 (29-42)		7 (4-11)		7 (4-11)		6 (3-10)		6 (3-10)	
2015	Djibouti	35 (25-36)	5 (3-5)	35 (25-36)	5 (3-5)	6 (3-10)	1 (0-1)	6 (3-10)	1 (0-1)	6 (3-9)	1 (0-1)	6 (3-9)	1 (0-1)
2000	Dominica	0 (0-0)		3 (2-3)		0 (0-0)		0 (0-0)		0 (0-0)		0 (0-0)	
2001	Dominica	0 (0-0)		3 (2-3)		0 (0-0)		3 (2-5)		0 (0-0)		3 (1-4)	
2002	Dominica	0 (0-0)		1 (1-1)		0 (0-0)		3 (1-4)		0 (0-0)		2 (1-4)	
2003	Dominica	0 (0-0)		0 (0-0)		0 (0-0)		3 (1-5)		0 (0-0)		3 (1-4)	
2004	Dominica	0 (0-0)		3 (2-3)		0 (0-0)		0 (0-0)		0 (0-0)		0 (0-0)	
2005	Dominica	0 (0-0)		5 (4-6)		0 (0-0)		0 (0-0)		0 (0-0)		0 (0-0)	
2006	Dominica	0 (0-0)		6 (4-6)		0 (0-0)		0 (0-0)		0 (0-0)		0 (0-0)	
2007	Dominica	1 (0-1)		10 (7-11)		0 (0-0)		0 (0-0)		0 (0-0)		0 (0-0)	
2008	Dominica	0 (0-0)		8 (6-9)		0 (0-0)		2 (1-3)		0 (0-0)		2 (1-2)	
2009	Dominica	0 (0-0)		6 (5-7)		0 (0-0)		2 (1-2)		0 (0-0)		1 (1-2)	
2010	Dominica	0 (0-0)		1 (1-1)		0 (0-0)		2 (1-3)		0 (0-0)		2 (1-2)	
2011	Dominica	0 (0-0)		3 (2-3)		0 (0-0)		0 (0-0)		0 (0-0)		0 (0-0)	
2012	Dominica	0 (0-0)		6 (4-7)		0 (0-0)		0 (0-0)		0 (0-0)		0 (0-0)	
2013	Dominica	0 (0-0)		8 (6-8)		0 (0-0)		0 (0-0)		0 (0-0)		0 (0-0)	
2014	Dominica	0 (0-0)		7 (5-7)		0 (0-0)		0 (0-0)		0 (0-0)		0 (0-0)	
2015	Dominica	0 (0-0)	0 (0-0)	6 (4-6)	0 (0-0)	0 (0-0)	0 (0-0)	0 (0-0)	0 (0-0)	0 (0-0)	0 (0-0)	0 (0-0)	0 (0-0)
2000	Dominican Republic	286 (203-298)		29 (20-30)		26 (8-65)		3 (1-6)		23 (8-57)		2 (1-6)	
2001	Dominican Republic	265 (188-277)		27 (19-28)		24 (8-60)		2 (1-6)		21 (7-53)		2 (1-5)	
2002	Dominican Republic	252 (178-262)		25 (18-26)		23 (7-57)		2 (1-6)		20 (6-51)		2 (1-5)	
2003	Dominican Republic	243 (173-254)		24 (17-25)		22 (7-54)		2 (1-5)		19 (6-48)		2 (1-5)	
2004	Dominican Republic	236 (167-246)		23 (16-24)		21 (7-52)		2 (1-5)		19 (6-46)		2 (1-5)	
2005	Dominican Republic	225 (159-234)		22 (15-23)		20 (6-49)		2 (1-5)		17 (6-43)		2 (1-4)	
2006	Dominican Republic	216 (153-225)		21 (15-21)		19 (6-46)		2 (1-4)		17 (5-41)		2 (1-4)	
2007	Dominican Republic	205 (146-214)		20 (14-20)		17 (6-43)		2 (1-4)		16 (5-38)		1 (0-4)	
2008	Dominican Republic	199 (141-208)		19 (13-20)		17 (5-41)		2 (1-4)		15 (5-36)		1 (0-3)	
2009	Dominican Republic	192 (136-201)		18 (13-19)		16 (5-39)		2 (0-4)		14 (5-35)		1 (0-3)	
2010	Dominican Republic	191 (136-199)		18 (13-19)		16 (5-39)		1 (0-4)		14 (5-34)		1 (0-3)	
2011	Dominican Republic	190 (135-198)		18 (13-19)		15 (5-38)		1 (0-4)		14 (4-34)		1 (0-3)	
2012	Dominican Republic	189 (134-197)		18 (13-19)		15 (5-38)		1 (0-4)		14 (4-34)		1 (0-3)	
2013	Dominican Republic	191 (135-199)		18 (13-19)		15 (5-38)		1 (0-4)		14 (4-34)		1 (0-3)	
2014	Dominican Republic	167 (119-174)		16 (11-17)		13 (5-32)		1 (0-3)		12 (4-29)		1 (0-3)	
2015	Dominican Republic	176 (125-184)	1 (0-1)	17 (12-18)	0 (0-0)	14 (5-34)	0 (0-0)	1 (0-3)	0 (0-0)	13 (4-30)	0 (0-0)	1 (0-3)	0 (0-0)
2000	Ecuador	447 (317-465)		30 (21-31)		53 (20-94)		4 (1-6)		48 (18-83)		3 (1-6)	
2001	Ecuador	425 (301-443)		28 (20-30)		51 (19-89)		3 (1-6)		45 (17-79)		3 (1-5)	
2002	Ecuador	402 (285-419)		27 (19-28)		48 (18-84)		3 (1-6)		43 (16-75)		3 (1-5)	
2003	Ecuador	399 (283-416)		27 (19-28)		73 (27-125)		5 (2-8)		65 (24-111)		4 (2-7)	
2004	Ecuador	417 (296-435)		28 (20-29)		91 (33-151)		6 (2-10)		81 (29-134)		5 (2-9)	
2005	Ecuador	405 (287-422)		27 (19-28)		91 (52-163)		6 (3-11)		81 (46-145)		5 (3-10)	
2006	Ecuador	396 (281-412)		27 (19-28)		91 (49-153)		6 (3-10)		81 (43-137)		5 (3-9)	
2007	Ecuador	387 (275-404)		26 (18-27)		90 (46-144)		6 (3-10)		80 (41-128)		5 (3-9)	
2008	Ecuador	391 (277-407)		26 (18-27)		92 (45-143)		6 (3-9)		82 (40-127)		5 (3-8)	
2009	Ecuador	382 (271-398)		25 (18-26)		89 (44-138)		6 (3-9)		80 (39-123)		5 (3-8)	
2010	Ecuador	361 (256-377)		23 (17-24)		84 (41-130)		5 (3-8)		75 (37-116)		5 (2-7)	
2011	Ecuador	299 (212-312)		19 (14-20)		69 (34-107)		4 (2-7)		62 (30-96)		4 (2-6)	
2012	Ecuador	222 (157-231)		14 (10-15)		52 (25-80)		3 (2-5)		46 (23-72)		3 (1-5)	
2013	Ecuador	193 (137-201)		12 (9-13)		46 (23-71)		3 (1-5)		41 (20-64)		3 (1-4)	
2014	Ecuador	137 (97-143)		9 (6-9)		33 (16-51)		2 (1-3)		29 (14-46)		2 (1-3)	
2015	Ecuador	113 (80-118)	0 (0-0)	7 (5-7)	0 (0-0)	27 (13-42)	0 (0-0)	2 (1-3)	0 (0-0)	24 (12-37)	0 (0-0)	2 (1-2)	0 (0-0)
2000	Egypt	4163 (2952-4338)		53 (38-55)		207 (86-418)		3 (1-5)		185 (77-372)		2 (1-5)	
2001	Egypt	3844 (2726-4007)		48 (34-50)		192 (82-386)		2 (1-5)		171 (73-344)		2 (1-4)	
2002	Egypt	3547 (2515-3696)		44 (31-46)		178 (77-355)		2 (1-4)		158 (69-316)		2 (1-4)	
2003	Egypt	3269 (2318-3407)		39 (28-41)		164 (73-327)		2 (1-4)		146 (65-291)		2 (1-4)	
2004	Egypt	3053 (2165-3182)		36 (26-38)		153 (64-309)		2 (1-4)		136 (57-275)		2 (1-3)	
2005	Egypt	2828 (2005-2947)		33 (23-34)		142 (57-289)		2 (1-3)		127 (51-258)		1 (1-3)	
2006	Egypt	2646 (1877-2758)		30 (21-31)		134 (56-270)		2 (1-3)		120 (50-240)		1 (1-3)	
2007	Egypt	2488 (1764-2593)		28 (20-29)		128 (56-253)		1 (1-3)		114 (50-226)		1 (1-3)	
2008	Egypt	2329 (1651-2427)		26 (18-27)		121 (56-236)		1 (1-3)		108 (49-210)		1 (1-2)	
2009	Egypt	2221 (1575-2315)		24 (17-25)		116 (52-226)		1 (1-2)		103 (47-202)		1 (1-2)	
2010	Egypt	2161 (1533-2253)		23 (16-24)		113 (39-264)		1 (0-3)		100 (34-235)		1 (0-2)	
2011	Egypt	2145 (1521-2235)		22 (16-23)		111 (37-261)		1 (0-3)		99 (33-232)		1 (0-2)	
2012	Egypt	2174 (1542-2266)		21 (15-22)		111 (37-262)		1 (0-3)		99 (33-233)		1 (0-2)	
2013	Egypt	2216 (1571-2310)		20 (14-21)		111 (36-264)		1 (0-2)		99 (32-235)		1 (0-2)	
2014	Egypt	2696 (1912-2810)		23 (17-24)		167 (53-397)		1 (0-3)		148 (48-354)		1 (0-3)	
2015	Egypt	2715 (1925-2829)	2 (1-2)	23 (16-24)	0 (0-0)	170 (54-407)	0 (0-0)	1 (0-3)	0 (0-0)	151 (48-362)	0 (0-0)	1 (0-3)	0 (0-0)
2000	El Salvador	253 (179-264)		36 (25-37)		18 (5-47)		3 (1-7)		16 (5-42)		2 (1-6)	
2001	El Salvador	221 (156-230)		32 (23-34)		16 (5-41)		2 (1-6)		14 (4-36)		2 (1-5)	
2002	El Salvador	219 (155-228)		33 (23-34)		28 (6-87)		4 (1-13)		25 (5-77)		4 (1-12)	
2003	El Salvador	198 (141-207)		31 (22-32)		27 (6-83)		4 (1-13)		24 (5-74)		4 (1-11)	
2004	El Salvador	178 (126-186)		28 (20-29)		24 (6-74)		4 (1-12)		22 (5-66)		3 (1-10)	
2005	El Salvador	163 (115-170)		27 (19-28)		22 (5-67)		4 (1-11)		20 (5-60)		3 (1-10)	
2006	El Salvador	148 (105-154)		25 (18-26)		20 (5-61)		3 (1-10)		18 (4-55)		3 (1-9)	
2007	El Salvador	138 (98-144)		24 (17-25)		19 (5-59)		3 (1-10)		17 (4-52)		3 (1-9)	
2008	El Salvador	126 (89-131)		22 (16-23)		18 (4-53)		3 (1-9)		16 (4-47)		3 (1-8)	
2009	El Salvador	116 (82-121)		21 (15-22)		16 (4-48)		3 (1-9)		14 (3-43)		3 (1-8)	
2010	El Salvador	95 (67-99)		17 (12-18)		13 (3-38)		2 (1-7)		11 (3-34)		2 (1-6)	
2011	El Salvador	71 (50-74)		13 (9-14)		9 (2-27)		2 (0-5)		8 (2-24)		2 (0-4)	
2012	El Salvador	53 (38-55)		10 (7-10)		7 (2-19)		1 (0-4)		6 (2-17)		1 (0-3)	
2013	El Salvador	45 (32-47)		9 (6-9)		6 (2-17)		1 (0-3)		5 (2-15)		1 (0-3)	
2014	El Salvador	31 (22-32)		6 (4-6)		4 (1-11)		1 (0-2)		3 (1-10)		1 (0-2)	

Spn = *Streptococcus pneumoniae*; npnm = non-pneumonia, non-meningitis; HIV = deaths only in HIV-infected children; Uncertainty range provided in parentheses.

Year	Country	Spn pneumonia deaths (UR)	Spn pneumonia deaths, HIV (UR)	Spn pneumonia mortality rate (UR)	Spn pneumonia mortality rate, HIV (UR)	Spn meningitis deaths (UR)	Spn meningitis deaths, HIV (UR)	Spn meningitis mortality rate (UR)	Spn meningitis mortality rate, HIV (UR)	Spn NPNM deaths (UR)	Spn NPNM deaths, HIV (UR)	Spn NPNM mortality rate (UR)	Spn NPNM mortality rate, HIV (UR)
2015	El Salvador	25 (18-26)	0 (0-0)	5 (3-5)	0 (0-0)	3 (1-8)	0 (0-0)	1 (0-2)	0 (0-0)	3 (1-7)	0 (0-0)	1 (0-1)	0 (0-0)
2000	Equatorial Guinea	143 (101-149)		164 (117-171)		14 (8-25)		16 (9-29)		4 (2-8)		5 (3-9)	
2001	Equatorial Guinea	137 (97-143)		154 (109-160)		12 (5-26)		14 (6-29)		4 (2-8)		4 (2-9)	
2002	Equatorial Guinea	128 (91-133)		141 (100-147)		11 (4-23)		12 (5-26)		3 (1-7)		4 (2-8)	
2003	Equatorial Guinea	117 (83-122)		126 (90-132)		10 (4-20)		10 (4-22)		3 (1-6)		3 (1-7)	
2004	Equatorial Guinea	109 (77-114)		115 (82-120)		9 (4-18)		9 (4-19)		3 (1-6)		3 (1-6)	
2005	Equatorial Guinea	107 (76-111)		111 (78-115)		8 (3-17)		9 (4-18)		3 (1-5)		3 (1-6)	
2006	Equatorial Guinea	113 (80-118)		114 (81-118)		8 (3-18)		8 (4-18)		3 (1-6)		3 (1-6)	
2007	Equatorial Guinea	122 (87-127)		119 (85-124)		9 (4-19)		9 (4-18)		3 (1-6)		3 (1-6)	
2008	Equatorial Guinea	130 (92-135)		123 (87-128)		9 (4-20)		9 (4-19)		3 (1-6)		3 (1-6)	
2009	Equatorial Guinea	133 (94-139)		123 (87-128)		10 (4-20)		9 (4-18)		3 (1-6)		3 (1-6)	
2010	Equatorial Guinea	134 (95-139)		120 (85-125)		10 (4-20)		9 (4-18)		3 (1-6)		3 (1-6)	
2011	Equatorial Guinea	130 (92-136)		113 (80-118)		10 (4-20)		9 (4-18)		3 (1-6)		3 (1-6)	
2012	Equatorial Guinea	125 (88-130)		106 (75-110)		10 (4-20)		8 (3-17)		3 (1-6)		3 (1-5)	
2013	Equatorial Guinea	126 (90-132)		105 (74-109)		10 (4-20)		8 (4-17)		3 (1-6)		3 (1-5)	
2014	Equatorial Guinea	128 (90-133)		103 (73-108)		10 (4-20)		8 (4-17)		3 (1-6)		3 (1-5)	
2015	Equatorial Guinea	125 (88-130)	51 (36-53)	99 (70-103)	40 (28-42)	10 (4-20)	4 (2-8)	8 (3-16)	3 (1-6)	3 (1-6)	1 (1-3)	2 (1-5)	1 (0-2)
2000	Eritrea	682 (484-711)		124 (88-129)		121 (47-260)		22 (9-47)		38 (15-82)		7 (3-15)	
2001	Eritrea	675 (479-704)		119 (85-124)		121 (47-260)		21 (8-46)		38 (15-82)		7 (3-14)	
2002	Eritrea	673 (477-702)		114 (81-119)		122 (48-262)		21 (8-44)		38 (15-82)		7 (3-14)	
2003	Eritrea	673 (477-701)		110 (78-114)		123 (49-265)		20 (8-43)		39 (15-83)		6 (2-14)	
2004	Eritrea	672 (476-700)		105 (74-109)		124 (41-277)		19 (6-43)		111 (37-246)		17 (6-38)	
2005	Eritrea	666 (472-694)		100 (71-105)		125 (41-278)		19 (6-42)		111 (37-247)		17 (6-37)	
2006	Eritrea	662 (470-690)		95 (67-99)		125 (42-280)		18 (6-40)		112 (37-249)		16 (5-36)	
2007	Eritrea	651 (462-679)		90 (64-94)		125 (42-279)		17 (6-39)		111 (37-248)		15 (5-34)	
2008	Eritrea	634 (450-661)		86 (61-89)		123 (41-275)		17 (6-37)		110 (37-245)		15 (5-33)	
2009	Eritrea	614 (436-640)		81 (58-85)		121 (40-269)		16 (5-36)		107 (36-239)		14 (5-32)	
2010	Eritrea	596 (423-621)		78 (55-81)		117 (39-261)		15 (5-34)		104 (35-233)		14 (5-30)	
2011	Eritrea	577 (409-602)		74 (53-77)		113 (38-252)		15 (5-32)		101 (34-225)		13 (4-29)	
2012	Eritrea	556 (394-580)		71 (50-74)		109 (36-242)		14 (5-31)		97 (32-215)		12 (4-27)	
2013	Eritrea	535 (379-557)		68 (48-70)		104 (35-231)		13 (4-29)		92 (31-206)		12 (4-26)	
2014	Eritrea	519 (368-541)		65 (46-68)		100 (34-224)		13 (4-28)		89 (30-199)		11 (4-25)	
2015	Eritrea	500 (355-521)	17 (12-17)	62 (44-65)	2 (1-2)	97 (32-215)	3 (1-7)	12 (4-27)	0 (0-1)	86 (29-192)	3 (1-6)	11 (4-24)	0 (0-1)
2000	Estonia	2 (1-2)		3 (2-3)		0 (0-1)		1 (0-1)		0 (0-1)		0 (0-1)	
2001	Estonia	1 (0-1)		1 (1-1)		1 (0-1)		1 (0-2)		1 (0-1)		1 (0-2)	
2002	Estonia	2 (1-2)		3 (2-3)		1 (0-1)		1 (0-2)		1 (0-1)		1 (0-2)	
2003	Estonia	1 (1-1)		2 (1-2)		0 (0-1)		1 (0-1)		0 (0-1)		0 (0-1)	
2004	Estonia	2 (1-2)		3 (2-3)		0 (0-1)		1 (0-1)		0 (0-1)		0 (0-1)	
2005	Estonia	2 (1-2)		2 (2-3)		1 (0-1)		1 (0-2)		1 (0-1)		1 (0-2)	
2006	Estonia	0 (0-0)		0 (0-0)		0 (0-1)		1 (0-1)		0 (0-1)		0 (0-1)	
2007	Estonia	1 (1-1)		1 (1-1)		0 (0-1)		1 (0-1)		0 (0-1)		1 (0-1)	
2008	Estonia	0 (0-1)		1 (0-1)		1 (1-2)		2 (1-3)		1 (0-2)		1 (1-3)	
2009	Estonia	1 (1-1)		1 (1-1)		1 (0-1)		1 (0-1)		0 (0-1)		1 (0-1)	
2010	Estonia	2 (1-2)		2 (2-2)		1 (0-1)		1 (0-2)		1 (0-1)		1 (0-2)	
2011	Estonia	1 (1-1)		1 (1-1)		1 (0-1)		1 (0-2)		1 (0-1)		1 (0-1)	
2012	Estonia	1 (1-1)		1 (1-1)		0 (0-0)		0 (0-0)		0 (0-0)		0 (0-0)	
2013	Estonia	1 (1-1)		1 (1-1)		0 (0-1)		1 (0-1)		0 (0-1)		0 (0-1)	
2014	Estonia	1 (1-1)		1 (1-1)		0 (0-1)		0 (0-1)		0 (0-1)		0 (0-1)	
2015	Estonia	1 (1-1)	0 (0-0)	1 (1-1)	0 (0-0)	0 (0-1)	0 (0-0)	0 (0-1)	0 (0-0)	0 (0-0)	0 (0-0)	0 (0-1)	0 (0-0)
2000	Ethiopia	24802 (17587-25847)		203 (144-212)		5307 (1707-12412)		44 (14-102)		1669 (537-3902)		14 (4-32)	
2001	Ethiopia	24253 (17198-25275)		193 (137-201)		5169 (1670-12074)		41 (13-96)		1625 (525-3796)		13 (4-30)	
2002	Ethiopia	23217 (16463-24196)		181 (128-189)		4923 (1597-11484)		38 (12-90)		1548 (502-3611)		12 (4-28)	
2003	Ethiopia	21886 (15519-22808)		168 (119-176)		4607 (1500-10731)		35 (12-83)		1448 (472-3374)		11 (4-26)	
2004	Ethiopia	20400 (14465-21260)		156 (110-162)		4240 (1386-9860)		32 (11-75)		1333 (436-3100)		10 (3-24)	
2005	Ethiopia	18908 (13407-19705)		143 (101-149)		3866 (1268-8975)		29 (10-68)		1216 (399-2822)		9 (3-21)	
2006	Ethiopia	18146 (12868-18911)		136 (97-142)		3644 (1206-8431)		27 (9-63)		1146 (379-2651)		9 (3-20)	
2007	Ethiopia	16567 (11748-17266)		124 (88-129)		3264 (1091-7528)		24 (8-56)		1026 (343-2367)		8 (3-18)	
2008	Ethiopia	14618 (10366-15235)		108 (77-113)		2828 (954-6502)		21 (7-48)		889 (300-2044)		7 (2-15)	
2009	Ethiopia	14003 (9929-14593)		103 (73-107)		2662 (906-6101)		20 (7-45)		837 (285-1918)		6 (2-14)	
2010	Ethiopia	13052 (9255-13602)		95 (68-99)		2436 (838-5569)		18 (6-41)		766 (263-1751)		6 (2-13)	
2011	Ethiopia	11671 (8276-12163)		85 (60-88)		2127 (604-4996)		15 (4-36)		1893 (538-4448)		14 (4-32)	
2012	Ethiopia	9846 (6982-10261)		71 (50-74)		1739 (500-4074)		13 (4-29)		1548 (445-3627)		11 (3-26)	
2013	Ethiopia	8042 (5702-8381)		57 (41-60)		1384 (403-3234)		10 (3-23)		1232 (359-2879)		9 (3-20)	
2014	Ethiopia	6856 (4862-7145)		48 (34-50)		1170 (345-2727)		8 (2-19)		1042 (307-2428)		7 (2-17)	
2015	Ethiopia	4622 (3277-4817)	180 (128-188)	32 (23-34)	1 (1-1)	756 (226-1757)	29 (9-68)	5 (2-12)	0 (0-0)	673 (201-1565)	26 (8-61)	5 (1-11)	0 (0-0)
2000	Fiji	18 (13-19)		19 (13-20)		5 (2-8)		5 (2-9)		4 (2-7)		4 (2-8)	
2001	Fiji	17 (12-18)		19 (13-19)		5 (2-8)		5 (2-9)		4 (2-7)		4 (2-8)	
2002	Fiji	17 (12-18)		19 (13-20)		5 (2-8)		5 (2-9)		4 (2-7)		5 (2-8)	
2003	Fiji	17 (12-18)		19 (13-20)		5 (2-8)		5 (2-9)		4 (2-7)		5 (2-8)	
2004	Fiji	17 (12-18)		19 (14-20)		5 (2-8)		5 (2-9)		4 (2-7)		5 (2-8)	
2005	Fiji	18 (13-18)		20 (14-21)		5 (2-8)		5 (2-10)		4 (2-8)		5 (2-9)	
2006	Fiji	19 (13-20)		21 (15-22)		5 (2-9)		6 (3-10)		5 (2-8)		5 (2-9)	
2007	Fiji	20 (14-21)		23 (16-24)		5 (2-10)		6 (3-11)		5 (2-9)		5 (2-10)	
2008	Fiji	21 (15-21)		23 (17-24)		6 (2-10)		6 (3-11)		5 (2-9)		6 (3-10)	
2009	Fiji	21 (15-22)		24 (17-25)		6 (2-10)		6 (3-11)		5 (2-9)		6 (3-10)	
2010	Fiji	20 (14-21)		23 (16-24)		5 (2-10)		6 (3-11)		5 (2-9)		6 (2-10)	
2011	Fiji	20 (14-21)		22 (16-23)		5 (2-9)		6 (3-10)		5 (2-8)		5 (2-9)	
2012	Fiji	19 (14-20)		22 (15-22)		5 (2-9)		6 (3-10)		4 (2-8)		5 (2-9)	
2013	Fiji	10 (7-11)		12 (8-12)		3 (1-6)		4 (2-7)		3 (1-5)		3 (1-6)	
2014	Fiji	9 (6-9)		10 (7-10)		2 (1-4)		3 (1-5)		2 (1-4)		2 (1-4)	
2015	Fiji	8 (6-8)	0 (0-0)	9 (7-10)	0 (0-0)	2 (1-4)	0 (0-0)	2 (1-4)	0 (0-0)	2 (1-3)	0 (0-0)	2 (1-4)	0 (0-0)
2000	Finland	2 (1-2)		1 (0-1)		0 (0-1)		0 (0-0)		0 (0-1)		0 (0-0)	
2001	Finland	3 (2-3)		1 (1-1)		0 (0-1)		0 (0-0)		0 (0-1)		0 (0-0)	
2002	Finland	0 (0-0)		0 (0-0)		0 (0-0)		0 (0-0)		0 (0-0)		0 (0-0)	
2003	Finland	5 (3-5)		2 (1-2)		2 (1-3)		1 (0-1)		2 (1-3)		1 (0-1)	
2004	Finland	1 (1-1)		1 (0-1)		2 (1-3)		1 (0-1)		2 (1-3)		1 (0-1)	
2005	Finland	2 (1-2)		1 (0-1)		1 (0-2)		0 (0-1)		1 (0-2)		0 (0-1)	
2006	Finland	0 (0-0)		0 (0-0)		1 (0-1)		0 (0-0)		0 (0-1)		0 (0-0)	

Spn = *Streptococcus pneumoniae*; npnm = non-pneumonia, non-meningitis; HIV = deaths only in HIV-infected children; Uncertainty range provided in parentheses.

Year	Country	Spn pneumonia deaths (UR)	Spn pneumonia deaths, HIV (UR)	Spn pneumonia mortality rate (UR)	Spn pneumonia mortality rate, HIV (UR)	Spn meningitis deaths (UR)	Spn meningitis deaths, HIV (UR)	Spn meningitis mortality rate (UR)	Spn meningitis mortality rate, HIV (UR)	Spn NPNM deaths (UR)	Spn NPNM deaths, HIV (UR)	Spn NPNM mortality rate (UR)	Spn NPNM mortality rate, HIV (UR)
2007	Finland	2 (1-2)		1 (0-1)		1 (0-2)		0 (0-1)		1 (0-1)		0 (0-0)	
2008	Finland	2 (1-2)		1 (0-1)		1 (0-2)		0 (0-1)		1 (0-2)		0 (0-1)	
2009	Finland	2 (1-2)		1 (0-1)		0 (0-0)		0 (0-0)		0 (0-0)		0 (0-0)	
2010	Finland	1 (1-1)		0 (0-0)		1 (0-2)		0 (0-1)		1 (0-1)		0 (0-0)	
2011	Finland	3 (2-3)		1 (1-1)		0 (0-1)		0 (0-0)		0 (0-1)		0 (0-0)	
2012	Finland	1 (0-1)		0 (0-0)		0 (0-0)		0 (0-0)		0 (0-0)		0 (0-0)	
2013	Finland	1 (1-1)		0 (0-0)		0 (0-1)		0 (0-0)		0 (0-1)		0 (0-0)	
2014	Finland	1 (1-1)		0 (0-0)		0 (0-0)		0 (0-0)		0 (0-0)		0 (0-0)	
2015	Finland	1 (0-1)	0 (0-0)	0 (0-0)	0 (0-0)	0 (0-0)	0 (0-0)	0 (0-0)	0 (0-0)	0 (0-0)	0 (0-0)	0 (0-0)	0 (0-0)
2000	France	23 (16-24)		1 (0-1)		29 (13-52)		1 (0-1)		26 (12-47)		1 (0-1)	
2001	France	20 (14-21)		1 (0-1)		26 (12-48)		1 (0-1)		24 (11-43)		1 (0-1)	
2002	France	20 (14-21)		1 (0-1)		26 (12-47)		1 (0-1)		23 (10-42)		1 (0-1)	
2003	France	24 (17-25)		1 (0-1)		23 (10-42)		1 (0-1)		21 (9-37)		1 (0-1)	
2004	France	14 (10-14)		0 (0-0)		22 (10-39)		1 (0-1)		19 (9-35)		1 (0-1)	
2005	France	25 (18-26)		1 (0-1)		15 (7-28)		0 (0-1)		14 (6-25)		0 (0-1)	
2006	France	13 (9-13)		0 (0-0)		20 (9-36)		1 (0-1)		18 (8-32)		0 (0-1)	
2007	France	20 (14-21)		1 (0-1)		13 (6-23)		0 (0-1)		11 (5-20)		0 (0-1)	
2008	France	12 (8-12)		0 (0-0)		16 (7-28)		0 (0-1)		14 (6-25)		0 (0-1)	
2009	France	14 (10-15)		0 (0-0)		9 (4-17)		0 (0-0)		8 (4-15)		0 (0-0)	
2010	France	16 (11-17)		0 (0-0)		14 (6-25)		0 (0-1)		12 (6-22)		0 (0-1)	
2011	France	9 (6-9)		0 (0-0)		7 (3-13)		0 (0-0)		6 (3-11)		0 (0-0)	
2012	France	7 (5-7)		0 (0-0)		5 (2-9)		0 (0-0)		5 (2-8)		0 (0-0)	
2013	France	6 (4-6)		0 (0-0)		5 (2-9)		0 (0-0)		4 (2-8)		0 (0-0)	
2014	France	6 (4-6)		0 (0-0)		4 (2-8)		0 (0-0)		4 (2-7)		0 (0-0)	
2015	France	6 (4-6)	0 (0-0)	0 (0-0)	0 (0-0)	4 (2-8)	0 (0-0)	0 (0-0)	0 (0-0)	4 (2-7)	0 (0-0)	0 (0-0)	0 (0-0)
2000	Gabon	106 (75-111)		58 (41-61)		9 (4-19)		5 (2-11)		3 (1-6)		2 (1-3)	
2001	Gabon	110 (78-115)		60 (42-62)		10 (4-20)		5 (2-11)		3 (1-6)		2 (1-3)	
2002	Gabon	114 (81-119)		61 (43-64)		10 (4-21)		5 (2-11)		3 (1-7)		2 (1-4)	
2003	Gabon	118 (83-123)		63 (44-65)		11 (4-22)		6 (2-12)		3 (1-7)		2 (1-4)	
2004	Gabon	120 (85-125)		63 (45-66)		11 (5-22)		6 (2-12)		3 (1-7)		2 (1-4)	
2005	Gabon	122 (86-127)		63 (45-66)		11 (5-23)		6 (2-12)		3 (2-7)		2 (1-4)	
2006	Gabon	122 (87-128)		63 (45-65)		11 (4-23)		6 (2-12)		10 (4-21)		5 (2-11)	
2007	Gabon	123 (87-128)		62 (44-65)		11 (4-23)		6 (2-12)		10 (4-21)		5 (2-11)	
2008	Gabon	122 (86-127)		60 (43-63)		11 (4-23)		5 (2-11)		10 (4-21)		5 (2-10)	
2009	Gabon	121 (86-126)		59 (42-61)		11 (4-23)		5 (2-11)		10 (4-20)		5 (2-10)	
2010	Gabon	119 (85-124)		56 (40-59)		11 (4-22)		5 (2-10)		10 (4-20)		5 (2-9)	
2011	Gabon	119 (84-124)		55 (39-57)		11 (5-22)		5 (2-10)		10 (4-20)		4 (2-9)	
2012	Gabon	116 (83-121)		52 (37-54)		11 (5-21)		5 (2-10)		9 (4-19)		4 (2-8)	
2013	Gabon	114 (81-119)		50 (36-52)		10 (5-21)		5 (2-9)		9 (4-18)		4 (2-8)	
2014	Gabon	110 (78-115)		48 (34-50)		10 (4-20)		4 (2-8)		9 (4-18)		4 (2-8)	
2015	Gabon	107 (76-112)	4 (3-4)	46 (32-48)	2 (1-2)	10 (4-19)	0 (0-1)	4 (2-8)	0 (0-0)	9 (4-17)	0 (0-1)	4 (2-7)	0 (0-0)
2000	Gambia	343 (243-357)		151 (107-157)		67 (35-124)		30 (15-54)		21 (11-39)		9 (5-17)	
2001	Gambia	335 (238-349)		142 (101-148)		65 (33-121)		28 (14-51)		21 (10-38)		9 (4-16)	
2002	Gambia	343 (243-358)		141 (100-147)		67 (34-124)		28 (14-51)		21 (11-39)		9 (4-16)	
2003	Gambia	341 (242-355)		136 (96-142)		66 (33-124)		26 (13-50)		21 (10-39)		8 (4-16)	
2004	Gambia	337 (239-352)		131 (93-136)		65 (32-123)		25 (12-48)		21 (10-39)		8 (4-15)	
2005	Gambia	336 (238-350)		126 (89-131)		65 (31-123)		24 (12-46)		20 (10-39)		8 (4-14)	
2006	Gambia	334 (237-348)		121 (86-127)		64 (31-122)		23 (11-44)		20 (10-38)		7 (4-14)	
2007	Gambia	330 (234-344)		116 (83-121)		63 (31-120)		22 (11-42)		20 (10-38)		7 (3-13)	
2008	Gambia	327 (232-340)		112 (79-117)		62 (30-119)		21 (10-41)		20 (9-37)		7 (3-13)	
2009	Gambia	324 (229-337)		108 (76-112)		62 (30-118)		21 (10-39)		19 (9-37)		6 (3-12)	
2010	Gambia	280 (198-292)		90 (64-94)		52 (25-99)		17 (8-32)		16 (8-31)		5 (3-10)	
2011	Gambia	228 (162-238)		71 (51-74)		41 (20-79)		13 (6-25)		13 (6-25)		4 (2-8)	
2012	Gambia	166 (118-173)		50 (36-52)		30 (14-57)		9 (4-17)		9 (4-18)		3 (1-5)	
2013	Gambia	150 (106-156)		44 (31-46)		27 (12-54)		8 (3-16)		24 (10-48)		7 (3-14)	
2014	Gambia	111 (79-116)		32 (22-33)		20 (9-40)		6 (2-11)		18 (8-35)		5 (2-10)	
2015	Gambia	96 (68-100)	11 (8-11)	27 (19-28)	3 (2-3)	17 (8-35)	2 (1-4)	5 (2-10)	1 (0-1)	15 (7-31)	2 (1-3)	4 (2-9)	0 (0-1)
2000	Georgia	69 (49-72)		26 (18-27)		13 (6-20)		5 (2-7)		11 (6-18)		4 (2-7)	
2001	Georgia	59 (42-62)		23 (17-24)		11 (6-17)		4 (2-7)		9 (5-15)		4 (2-6)	
2002	Georgia	52 (37-55)		22 (15-23)		9 (5-16)		4 (2-6)		8 (4-14)		3 (2-6)	
2003	Georgia	47 (33-49)		20 (14-21)		8 (3-17)		3 (1-7)		7 (3-15)		3 (1-7)	
2004	Georgia	43 (30-44)		18 (13-19)		7 (3-16)		3 (1-7)		6 (2-14)		3 (1-6)	
2005	Georgia	39 (28-41)		17 (12-18)		6 (2-15)		3 (1-7)		6 (2-14)		2 (1-6)	
2006	Georgia	36 (26-38)		15 (11-16)		6 (2-14)		2 (1-6)		5 (2-13)		2 (1-5)	
2007	Georgia	33 (23-34)		13 (9-14)		5 (1-13)		2 (1-5)		5 (1-12)		2 (1-5)	
2008	Georgia	30 (21-31)		12 (8-12)		5 (1-12)		2 (0-5)		4 (1-11)		2 (0-4)	
2009	Georgia	27 (19-28)		10 (7-11)		4 (1-11)		2 (0-4)		4 (1-10)		1 (0-4)	
2010	Georgia	26 (18-27)		10 (7-10)		6 (1-17)		2 (1-7)		6 (1-15)		2 (0-6)	
2011	Georgia	25 (18-26)		9 (7-10)		7 (1-19)		3 (1-7)		6 (1-17)		2 (0-6)	
2012	Georgia	22 (15-23)		8 (6-8)		6 (1-16)		2 (0-6)		5 (1-15)		2 (0-5)	
2013	Georgia	19 (13-20)		7 (5-7)		5 (1-14)		2 (0-5)		5 (1-13)		2 (0-5)	
2014	Georgia	17 (12-18)		6 (4-7)		5 (1-13)		2 (0-5)		4 (1-11)		1 (0-4)	
2015	Georgia	15 (11-15)	0 (0-0)	5 (4-6)	0 (0-0)	4 (1-11)	0 (0-0)	1 (0-4)	0 (0-0)	4 (1-10)	0 (0-0)	1 (0-4)	0 (0-0)
2000	Germany	29 (21-30)		1 (1-1)		27 (12-50)		1 (0-1)		24 (11-44)		1 (0-1)	
2001	Germany	43 (31-45)		1 (1-1)		19 (9-35)		0 (0-1)		17 (8-31)		0 (0-1)	
2002	Germany	37 (26-38)		1 (1-1)		28 (12-50)		1 (0-1)		25 (11-44)		1 (0-1)	
2003	Germany	36 (26-38)		1 (1-1)		25 (11-45)		1 (0-1)		22 (10-40)		1 (0-1)	
2004	Germany	34 (24-35)		1 (1-1)		25 (11-45)		1 (0-1)		22 (10-40)		1 (0-1)	
2005	Germany	25 (18-26)		1 (1-1)		18 (8-33)		1 (0-1)		16 (7-29)		0 (0-1)	
2006	Germany	19 (14-20)		1 (0-1)		21 (9-38)		1 (0-1)		18 (8-33)		1 (0-1)	
2007	Germany	19 (13-20)		1 (0-1)		11 (5-20)		0 (0-1)		10 (4-18)		0 (0-1)	
2008	Germany	20 (14-21)		1 (0-1)		9 (4-17)		0 (0-1)		8 (4-15)		0 (0-0)	
2009	Germany	17 (12-18)		1 (0-1)		5 (2-10)		0 (0-0)		5 (2-9)		0 (0-0)	
2010	Germany	5 (4-6)		0 (0-0)		3 (1-5)		0 (0-0)		3 (1-5)		0 (0-0)	
2011	Germany	6 (4-6)		0 (0-0)		2 (1-4)		0 (0-0)		2 (1-3)		0 (0-0)	
2012	Germany	4 (3-4)		0 (0-0)		2 (1-4)		0 (0-0)		2 (1-3)		0 (0-0)	
2013	Germany	4 (3-4)		0 (0-0)		2 (1-3)		0 (0-0)		2 (1-3)		0 (0-0)	
2014	Germany	4 (3-4)		0 (0-0)		2 (1-3)		0 (0-0)		1 (1-3)		0 (0-0)	

Spn = *Streptococcus pneumoniae*; npnm = non-pneumonia, non-meningitis; HIV = deaths only in HIV-infected children; Uncertainty range provided in parentheses.

Year	Country	Spn pneumonia deaths (UR)	Spn pneumonia deaths, HIV (UR)	Spn pneumonia mortality rate (UR)	Spn pneumonia mortality rate, HIV (UR)	Spn meningitis deaths (UR)	Spn meningitis deaths, HIV (UR)	Spn meningitis mortality rate (UR)	Spn meningitis mortality rate, HIV (UR)	Spn NPNM deaths (UR)	Spn NPNM deaths, HIV (UR)	Spn NPNM mortality rate (UR)	Spn NPNM mortality rate, HIV (UR)
2015	Germany	4 (3-4)	0 (0-0)	0 (0-0)	0 (0-0)	2 (1-3)	0 (0-0)	0 (0-0)	0 (0-0)	1 (1-3)	0 (0-0)	0 (0-0)	0 (0-0)
2000	Ghana	2135 (1514-2225)		76 (54-79)		343 (147-712)		12 (5-25)		108 (46-224)		4 (2-8)	
2001	Ghana	2195 (1557-2288)		77 (55-80)		348 (145-732)		12 (5-26)		110 (45-230)		4 (2-8)	
2002	Ghana	2283 (1619-2380)		78 (56-82)		358 (144-761)		12 (5-26)		113 (45-239)		4 (2-8)	
2003	Ghana	2328 (1651-2426)		78 (55-81)		361 (142-775)		12 (5-26)		113 (45-244)		4 (1-8)	
2004	Ghana	2430 (1723-2533)		79 (56-82)		373 (142-809)		12 (5-26)		117 (45-254)		4 (1-8)	
2005	Ghana	2512 (1781-2618)		80 (57-83)		382 (142-835)		12 (4-27)		120 (45-263)		4 (1-8)	
2006	Ghana	2619 (1857-2729)		81 (57-84)		395 (142-868)		12 (4-27)		124 (45-273)		4 (1-8)	
2007	Ghana	2633 (1867-2744)		79 (56-83)		395 (153-853)		12 (5-26)		124 (48-268)		4 (1-8)	
2008	Ghana	2542 (1802-2649)		75 (53-78)		378 (159-801)		11 (5-24)		119 (50-252)		4 (1-7)	
2009	Ghana	2522 (1789-2629)		73 (52-76)		371 (151-791)		11 (4-23)		117 (47-249)		3 (1-7)	
2010	Ghana	2470 (1752-2575)		70 (50-73)		357 (118-791)		10 (3-22)		318 (105-704)		9 (3-20)	
2011	Ghana	2490 (1766-2595)		69 (49-72)		353 (113-789)		10 (3-22)		314 (100-702)		9 (3-19)	
2012	Ghana	2113 (1498-2202)		57 (40-59)		287 (96-637)		8 (3-17)		256 (85-567)		7 (2-15)	
2013	Ghana	1605 (1138-1673)		42 (30-44)		207 (72-454)		5 (2-12)		184 (64-404)		5 (2-11)	
2014	Ghana	1360 (964-1417)		35 (25-36)		177 (64-384)		5 (2-10)		158 (57-342)		4 (1-9)	
2015	Ghana	1257 (891-1310)	72 (51-75)	32 (22-33)	2 (1-2)	170 (64-364)	10 (4-21)	4 (2-9)	0 (0-1)	151 (57-324)	9 (3-19)	4 (1-8)	0 (0-0)
2000	Greece	8 (6-9)		2 (1-2)		3 (2-6)		1 (0-1)		3 (1-5)		1 (0-1)	
2001	Greece	7 (5-8)		1 (1-1)		2 (1-3)		0 (0-1)		2 (1-3)		0 (0-1)	
2002	Greece	6 (4-6)		1 (1-1)		0 (0-1)		0 (0-0)		0 (0-1)		0 (0-0)	
2003	Greece	8 (6-9)		2 (1-2)		2 (1-4)		0 (0-1)		2 (1-3)		0 (0-1)	
2004	Greece	9 (7-10)		2 (1-2)		2 (1-3)		0 (0-1)		1 (1-3)		0 (0-1)	
2005	Greece	11 (8-12)		2 (2-2)		1 (0-1)		0 (0-0)		1 (0-1)		0 (0-0)	
2006	Greece	13 (9-14)		3 (2-3)		1 (1-2)		0 (0-0)		1 (0-2)		0 (0-0)	
2007	Greece	12 (8-12)		2 (2-2)		1 (0-1)		0 (0-0)		1 (0-1)		0 (0-0)	
2008	Greece	12 (8-12)		2 (2-2)		1 (1-2)		0 (0-0)		1 (0-2)		0 (0-0)	
2009	Greece	7 (5-7)		1 (1-1)		2 (1-3)		0 (0-1)		1 (1-3)		0 (0-0)	
2010	Greece	5 (4-6)		1 (1-1)		0 (0-1)		0 (0-0)		0 (0-0)		0 (0-0)	
2011	Greece	5 (4-5)		1 (1-1)		1 (0-1)		0 (0-0)		1 (0-1)		0 (0-0)	
2012	Greece	8 (6-8)		1 (1-1)		0 (0-1)		0 (0-0)		0 (0-1)		0 (0-0)	
2013	Greece	6 (4-6)		1 (1-1)		0 (0-1)		0 (0-0)		0 (0-1)		0 (0-0)	
2014	Greece	3 (2-3)		1 (0-1)		0 (0-0)		0 (0-0)		0 (0-0)		0 (0-0)	
2015	Greece	2 (1-2)	0 (0-0)	0 (0-0)	0 (0-0)	0 (0-0)	0 (0-0)	0 (0-0)	0 (0-0)	0 (0-0)	0 (0-0)	0 (0-0)	0 (0-0)
2000	Grenada	1 (1-1)		11 (8-12)		0 (0-0)		0 (0-0)		0 (0-0)		0 (0-0)	
2001	Grenada	1 (1-1)		10 (7-10)		0 (0-0)		0 (0-0)		0 (0-0)		0 (0-0)	
2002	Grenada	1 (1-1)		7 (5-7)		0 (0-0)		0 (0-0)		0 (0-0)		0 (0-0)	
2003	Grenada	0 (0-0)		3 (2-3)		0 (0-0)		0 (0-0)		0 (0-0)		0 (0-0)	
2004	Grenada	1 (0-1)		7 (5-7)		0 (0-0)		0 (0-0)		0 (0-0)		0 (0-0)	
2005	Grenada	1 (1-1)		11 (8-11)		0 (0-0)		0 (0-0)		0 (0-0)		0 (0-0)	
2006	Grenada	1 (1-2)		16 (12-17)		0 (0-0)		0 (0-0)		0 (0-0)		0 (0-0)	
2007	Grenada	1 (1-1)		12 (8-12)		0 (0-0)		0 (0-0)		0 (0-0)		0 (0-0)	
2008	Grenada	1 (0-1)		7 (5-7)		0 (0-0)		0 (0-0)		0 (0-0)		0 (0-0)	
2009	Grenada	0 (0-0)		1 (1-1)		0 (0-0)		0 (0-0)		0 (0-0)		0 (0-0)	
2010	Grenada	0 (0-0)		2 (1-2)		0 (0-0)		0 (0-0)		0 (0-0)		0 (0-0)	
2011	Grenada	0 (0-0)		2 (1-2)		0 (0-0)		0 (0-0)		0 (0-0)		0 (0-0)	
2012	Grenada	0 (0-0)		4 (3-4)		0 (0-0)		0 (0-0)		0 (0-0)		0 (0-0)	
2013	Grenada	0 (0-0)		3 (2-3)		0 (0-0)		0 (0-0)		0 (0-0)		0 (0-0)	
2014	Grenada	0 (0-0)		4 (3-4)		0 (0-0)		0 (0-0)		0 (0-0)		0 (0-0)	
2015	Grenada	0 (0-0)	0 (0-0)	3 (2-3)	0 (0-0)	0 (0-0)	0 (0-0)	0 (0-0)	0 (0-0)	0 (0-0)	0 (0-0)	0 (0-0)	0 (0-0)
2000	Guatemala	1242 (881-1295)		67 (48-70)		118 (36-301)		6 (2-16)		105 (32-268)		6 (2-15)	
2001	Guatemala	1197 (849-1247)		64 (46-67)		114 (35-289)		6 (2-16)		101 (31-258)		5 (2-14)	
2002	Guatemala	1151 (816-1199)		61 (43-64)		110 (34-278)		6 (2-15)		98 (30-248)		5 (2-13)	
2003	Guatemala	1102 (782-1149)		58 (41-60)		105 (33-266)		6 (2-14)		94 (29-237)		5 (2-12)	
2004	Guatemala	1062 (753-1107)		55 (39-58)		102 (32-256)		5 (2-13)		91 (28-228)		5 (1-12)	
2005	Guatemala	1021 (724-1064)		53 (37-55)		97 (31-244)		5 (2-13)		87 (27-218)		4 (1-11)	
2006	Guatemala	977 (693-1018)		50 (36-52)		93 (29-232)		5 (2-12)		83 (26-207)		4 (1-11)	
2007	Guatemala	933 (662-972)		48 (34-50)		89 (28-220)		5 (1-11)		79 (25-196)		4 (1-10)	
2008	Guatemala	882 (625-919)		45 (32-47)		84 (27-208)		4 (1-11)		75 (24-185)		4 (1-9)	
2009	Guatemala	849 (602-885)		43 (31-45)		81 (26-199)		4 (1-10)		72 (23-178)		4 (1-9)	
2010	Guatemala	824 (584-859)		42 (30-44)		78 (25-193)		4 (1-10)		70 (23-172)		4 (1-9)	
2011	Guatemala	806 (572-840)		41 (29-43)		76 (25-188)		4 (1-10)		68 (22-167)		3 (1-8)	
2012	Guatemala	785 (557-818)		40 (28-41)		74 (24-182)		4 (1-9)		66 (22-162)		3 (1-8)	
2013	Guatemala	698 (495-727)		35 (25-36)		65 (22-160)		3 (1-8)		58 (19-142)		3 (1-7)	
2014	Guatemala	570 (404-594)		28 (20-29)		53 (18-129)		3 (1-6)		47 (16-115)		2 (1-6)	
2015	Guatemala	421 (298-439)	8 (5-8)	20 (15-21)	0 (0-0)	39 (10-116)	1 (0-2)	2 (0-6)	0 (0-0)	35 (9-103)	1 (0-2)	2 (0-5)	0 (0-0)
2000	Guinea	2545 (1805-2652)		168 (119-175)		472 (236-934)		31 (16-62)		148 (74-294)		10 (5-19)	
2001	Guinea	2538 (1800-2645)		165 (117-172)		471 (241-925)		31 (16-60)		148 (76-291)		10 (5-19)	
2002	Guinea	2515 (1783-2621)		161 (114-168)		467 (245-911)		30 (16-58)		147 (77-286)		9 (5-18)	
2003	Guinea	2487 (1764-2592)		158 (112-164)		465 (167-1031)		29 (11-65)		146 (52-324)		9 (3-21)	
2004	Guinea	2480 (1759-2585)		155 (110-161)		466 (174-1024)		29 (11-64)		147 (55-322)		9 (3-20)	
2005	Guinea	2476 (1756-2580)		152 (108-158)		469 (182-1019)		29 (11-63)		147 (57-320)		9 (4-20)	
2006	Guinea	2473 (1754-2577)		149 (105-155)		471 (181-1023)		28 (11-62)		148 (57-322)		9 (3-19)	
2007	Guinea	2465 (1748-2569)		145 (103-152)		470 (180-1023)		28 (11-60)		148 (57-322)		9 (3-19)	
2008	Guinea	2368 (1679-2468)		137 (97-143)		452 (172-985)		26 (10-57)		142 (54-310)		8 (3-18)	
2009	Guinea	2382 (1689-2483)		135 (96-141)		454 (172-992)		26 (10-56)		143 (54-312)		8 (3-18)	
2010	Guinea	2328 (1651-2426)		129 (92-135)		443 (167-970)		25 (9-54)		139 (52-305)		8 (3-17)	
2011	Guinea	2280 (1617-2376)		124 (88-129)		432 (162-948)		23 (9-51)		136 (51-298)		7 (3-16)	
2012	Guinea	2256 (1600-2351)		120 (85-125)		425 (158-935)		23 (8-50)		134 (50-294)		7 (3-16)	
2013	Guinea	2264 (1605-2359)		117 (83-122)		424 (157-936)		22 (8-48)		133 (50-294)		7 (3-15)	
2014	Guinea	2268 (1608-2364)		115 (82-120)		423 (156-935)		21 (8-47)		133 (49-294)		7 (2-15)	
2015	Guinea	2246 (1593-2341)	92 (66-96)	112 (79-116)	5 (3-5)	418 (154-926)	17 (6-38)	21 (8-46)	1 (0-2)	132 (48-291)	5 (2-12)	7 (2-14)	0 (0-1)
2000	Guinea-Bissau	446 (317-465)		206 (146-215)		86 (52-145)		40 (24-67)		27 (16-46)		12 (7-21)	
2001	Guinea-Bissau	461 (327-480)		210 (149-219)		89 (53-151)		41 (24-69)		28 (17-47)		13 (8-22)	
2002	Guinea-Bissau	471 (334-491)		212 (150-221)		91 (54-155)		41 (24-70)		29 (17-49)		13 (8-22)	
2003	Guinea-Bissau	478 (339-498)		212 (150-220)		92 (55-159)		41 (24-70)		29 (17-50)		13 (8-22)	
2004	Guinea-Bissau	477 (338-497)		208 (147-217)		91 (54-159)		40 (24-69)		29 (17-50)		13 (7-22)	
2005	Guinea-Bissau	469 (332-488)		201 (142-209)		89 (39-177)		38 (17-76)		28 (17-56)		12 (5-24)	
2006	Guinea-Bissau	456 (323-475)		191 (135-199)		86 (37-173)		36 (16-73)		27 (12-54)		11 (5-23)	

Spn = *Streptococcus pneumoniae*; npnm = non-pneumonia, non-meningitis; HIV = deaths only in HIV-infected children; Uncertainty range provided in parentheses.

Year	Country	Spn pneumonia deaths (UR)	Spn pneumonia deaths, HIV (UR)	Spn pneumonia mortality rate (UR)	Spn pneumonia mortality rate, HIV (UR)	Spn meningitis deaths (UR)	Spn meningitis deaths, HIV (UR)	Spn meningitis mortality rate (UR)	Spn meningitis mortality rate, HIV (UR)	Spn NPNM deaths (UR)	Spn NPNM deaths, HIV (UR)	Spn NPNM mortality rate (UR)	Spn NPNM mortality rate, HIV (UR)
2007	Guinea-Bissau	439 (311-458)		181 (128-189)		82 (35-167)		34 (14-69)		26 (11-52)		11 (5-22)	
2008	Guinea-Bissau	420 (298-438)		170 (121-178)		77 (33-159)		31 (13-65)		24 (10-50)		10 (4-20)	
2009	Guinea-Bissau	402 (285-419)		160 (114-167)		73 (31-152)		29 (12-61)		23 (10-48)		9 (4-19)	
2010	Guinea-Bissau	386 (274-403)		151 (107-157)		70 (29-146)		27 (11-57)		22 (9-46)		9 (4-18)	
2011	Guinea-Bissau	371 (263-387)		142 (100-147)		67 (27-142)		25 (10-54)		21 (8-45)		8 (3-17)	
2012	Guinea-Bissau	359 (255-374)		134 (95-140)		64 (25-138)		24 (9-52)		20 (8-43)		8 (3-16)	
2013	Guinea-Bissau	350 (248-365)		128 (91-133)		62 (23-136)		23 (9-50)		20 (7-43)		7 (3-16)	
2014	Guinea-Bissau	343 (243-358)		123 (87-128)		61 (22-134)		22 (8-48)		19 (7-42)		7 (2-15)	
2015	Guinea-Bissau	320 (227-333)	96 (68-100)	113 (80-117)	34 (24-35)	56 (20-126)	17 (6-37)	20 (7-44)	6 (2-13)	18 (6-39)	5 (2-12)	6 (2-14)	2 (1-4)
2000	Guyana	21 (15-22)		22 (16-23)		2 (1-4)		2 (1-4)		1 (1-3)		2 (1-4)	
2001	Guyana	19 (13-19)		20 (14-21)		4 (2-10)		5 (2-11)		4 (1-9)		4 (1-10)	
2002	Guyana	26 (18-27)		28 (20-29)		3 (1-7)		3 (1-8)		3 (1-6)		3 (1-7)	
2003	Guyana	18 (13-19)		20 (15-21)		4 (1-9)		4 (1-10)		3 (1-8)		4 (1-9)	
2004	Guyana	21 (15-22)		24 (17-25)		4 (1-9)		4 (1-11)		3 (1-8)		4 (1-10)	
2005	Guyana	17 (12-18)		20 (15-21)		4 (1-10)		5 (1-12)		3 (1-9)		4 (1-10)	
2006	Guyana	17 (12-18)		22 (15-22)		2 (1-6)		3 (1-7)		2 (1-5)		3 (1-6)	
2007	Guyana	15 (10-15)		20 (14-20)		3 (1-8)		4 (1-10)		3 (1-7)		4 (1-9)	
2008	Guyana	12 (9-13)		18 (12-18)		3 (1-7)		4 (1-10)		3 (1-6)		4 (1-9)	
2009	Guyana	13 (9-14)		19 (14-20)		6 (2-14)		9 (3-22)		5 (2-13)		8 (2-19)	
2010	Guyana	12 (8-12)		18 (13-19)		1 (0-4)		2 (1-6)		1 (0-3)		2 (1-5)	
2011	Guyana	10 (7-11)		17 (12-18)		3 (1-7)		5 (2-12)		3 (1-6)		4 (2-11)	
2012	Guyana	8 (5-8)		13 (9-13)		2 (1-5)		4 (1-8)		2 (1-4)		3 (1-7)	
2013	Guyana	7 (5-7)		11 (8-12)		2 (1-5)		3 (1-7)		2 (1-4)		3 (1-7)	
2014	Guyana	7 (5-7)		10 (7-11)		2 (1-4)		3 (1-7)		2 (1-4)		3 (1-6)	
2015	Guyana	5 (4-5)	0 (0-0)	8 (5-8)	0 (0-0)	1 (1-3)	0 (0-0)	2 (1-5)	0 (0-0)	1 (1-3)	0 (0-0)	2 (1-4)	0 (0-0)
2000	Haiti	1883 (1335-1962)		157 (111-164)		238 (61-678)		20 (5-57)		75 (19-213)		6 (2-18)	
2001	Haiti	1806 (1280-1882)		150 (106-156)		229 (59-652)		19 (5-54)		72 (19-205)		6 (2-17)	
2002	Haiti	1726 (1224-1799)		143 (101-149)		220 (57-626)		18 (5-52)		69 (18-197)		6 (1-16)	
2003	Haiti	1648 (1169-1718)		137 (97-142)		212 (56-599)		18 (5-50)		67 (18-188)		6 (1-16)	
2004	Haiti	1583 (1122-1650)		131 (93-137)		204 (54-577)		17 (5-48)		64 (17-182)		5 (1-15)	
2005	Haiti	1542 (1093-1607)		128 (91-133)		200 (53-563)		17 (4-47)		63 (17-177)		5 (1-15)	
2006	Haiti	1514 (1074-1578)		125 (89-131)		197 (53-554)		16 (4-46)		62 (17-174)		5 (1-14)	
2007	Haiti	1492 (1058-1555)		123 (87-128)		195 (52-546)		16 (4-45)		61 (16-172)		5 (1-14)	
2008	Haiti	1469 (1042-1531)		121 (86-126)		193 (52-537)		16 (4-44)		61 (16-169)		5 (1-14)	
2009	Haiti	1442 (1023-1503)		118 (84-123)		191 (52-527)		16 (4-43)		60 (16-166)		5 (1-14)	
2010	Haiti	5516 (3911-5748)		450 (319-469)		732 (309-1743)		60 (25-142)		230 (97-548)		19 (8-45)	
2011	Haiti	1376 (976-1434)		112 (79-116)		182 (51-500)		15 (4-41)		57 (16-157)		5 (1-13)	
2012	Haiti	1344 (953-1401)		109 (77-114)		177 (50-485)		14 (4-39)		56 (16-153)		5 (1-12)	
2013	Haiti	1297 (920-1351)		106 (75-110)		169 (39-481)		14 (3-39)		151 (35-428)		12 (3-35)	
2014	Haiti	1260 (894-1313)		103 (73-108)		163 (38-465)		13 (3-38)		146 (34-414)		12 (3-34)	
2015	Haiti	1217 (863-1269)	41 (29-43)	100 (71-104)	3 (2-4)	157 (37-448)	5 (1-15)	13 (3-37)	0 (0-1)	140 (33-399)	5 (1-14)	12 (3-33)	0 (0-1)
2000	Honduras	417 (296-435)		45 (32-47)		43 (12-116)		5 (1-12)		38 (10-103)		4 (1-11)	
2001	Honduras	388 (275-404)		42 (30-43)		42 (11-112)		4 (1-12)		37 (10-100)		4 (1-11)	
2002	Honduras	350 (248-365)		38 (27-39)		38 (10-101)		4 (1-11)		34 (9-90)		4 (1-10)	
2003	Honduras	320 (227-334)		35 (25-36)		34 (10-92)		4 (1-10)		31 (8-82)		3 (1-9)	
2004	Honduras	296 (210-308)		32 (23-34)		32 (9-85)		3 (1-9)		28 (8-76)		3 (1-8)	
2005	Honduras	280 (198-291)		31 (22-32)		31 (9-83)		3 (1-9)		28 (8-74)		3 (1-8)	
2006	Honduras	258 (183-268)		29 (20-30)		29 (6-92)		3 (1-10)		26 (5-82)		3 (1-9)	
2007	Honduras	239 (169-249)		27 (19-28)		27 (5-85)		3 (1-10)		24 (5-76)		3 (1-9)	
2008	Honduras	223 (158-232)		25 (18-27)		25 (5-79)		3 (1-9)		22 (5-71)		3 (1-8)	
2009	Honduras	209 (148-218)		24 (17-25)		24 (5-74)		3 (1-9)		21 (4-66)		2 (1-8)	
2010	Honduras	196 (139-204)		23 (16-24)		22 (5-69)		3 (1-8)		20 (4-61)		2 (0-7)	
2011	Honduras	137 (97-143)		16 (11-17)		14 (3-45)		2 (0-5)		13 (3-40)		2 (0-5)	
2012	Honduras	102 (72-106)		12 (9-13)		10 (2-32)		1 (0-4)		9 (2-29)		1 (0-3)	
2013	Honduras	86 (61-90)		11 (7-11)		9 (2-28)		1 (0-3)		8 (2-25)		1 (0-3)	
2014	Honduras	78 (55-81)		10 (7-10)		8 (2-25)		1 (0-3)		7 (2-22)		1 (0-3)	
2015	Honduras	53 (38-55)	1 (0-1)	7 (5-7)	0 (0-0)	5 (1-16)	0 (0-0)	1 (0-2)	0 (0-0)	5 (1-14)	0 (0-0)	1 (0-2)	0 (0-0)
2000	Hungary	23 (16-24)		5 (3-5)		4 (2-7)		1 (0-1)		3 (2-6)		1 (0-1)	
2001	Hungary	16 (11-16)		3 (2-4)		6 (3-12)		1 (1-3)		6 (3-10)		1 (1-2)	
2002	Hungary	10 (7-10)		2 (2-2)		4 (2-7)		1 (0-2)		3 (2-6)		1 (0-1)	
2003	Hungary	16 (12-17)		4 (3-4)		3 (1-6)		1 (0-1)		3 (1-5)		1 (0-1)	
2004	Hungary	13 (9-14)		3 (2-3)		6 (3-11)		1 (1-2)		5 (2-10)		1 (1-2)	
2005	Hungary	16 (12-17)		3 (2-4)		7 (3-12)		1 (1-3)		6 (3-11)		1 (1-2)	
2006	Hungary	10 (7-11)		2 (2-2)		5 (2-8)		1 (0-2)		4 (2-7)		1 (0-2)	
2007	Hungary	15 (11-16)		3 (2-3)		4 (2-7)		1 (0-1)		3 (2-6)		1 (0-1)	
2008	Hungary	8 (6-8)		2 (1-2)		4 (2-7)		1 (0-1)		3 (1-6)		1 (0-1)	
2009	Hungary	8 (6-9)		2 (1-2)		3 (2-6)		1 (0-1)		3 (1-6)		1 (0-1)	
2010	Hungary	9 (6-9)		2 (1-2)		3 (1-5)		1 (0-1)		2 (1-4)		1 (0-1)	
2011	Hungary	7 (5-8)		2 (1-2)		2 (1-3)		0 (0-1)		1 (1-3)		0 (0-1)	
2012	Hungary	6 (4-6)		1 (1-1)		1 (0-1)		0 (0-0)		1 (0-1)		0 (0-0)	
2013	Hungary	4 (3-4)		1 (1-1)		2 (1-4)		0 (0-1)		2 (1-3)		0 (0-1)	
2014	Hungary	4 (3-4)		1 (1-1)		1 (1-2)		0 (0-0)		1 (0-2)		0 (0-0)	
2015	Hungary	3 (2-3)	0 (0-0)	1 (0-1)	0 (0-0)	1 (0-1)	0 (0-0)	0 (0-0)	0 (0-0)	1 (0-1)	0 (0-0)	0 (0-0)	0 (0-0)
2000	Iceland	0 (0-0)		0 (0-0)		0 (0-0)		0 (0-0)		0 (0-0)		0 (0-0)	
2001	Iceland	0 (0-0)		0 (0-0)		0 (0-0)		0 (0-0)		0 (0-0)		0 (0-0)	
2002	Iceland	0 (0-0)		0 (0-0)		0 (0-0)		1 (0-2)		0 (0-0)		1 (0-2)	
2003	Iceland	0 (0-0)		1 (1-1)		0 (0-0)		1 (0-2)		0 (0-0)		1 (0-2)	
2004	Iceland	0 (0-0)		1 (1-1)		0 (0-0)		1 (1-2)		0 (0-0)		1 (0-2)	
2005	Iceland	0 (0-0)		1 (0-1)		0 (0-0)		1 (0-1)		0 (0-0)		1 (0-1)	
2006	Iceland	0 (0-0)		0 (0-0)		0 (0-0)		1 (0-1)		0 (0-0)		1 (0-1)	
2007	Iceland	0 (0-0)		0 (0-0)		0 (0-0)		1 (0-1)		0 (0-0)		1 (0-1)	
2008	Iceland	0 (0-0)		0 (0-0)		0 (0-0)		0 (0-0)		0 (0-0)		0 (0-0)	
2009	Iceland	0 (0-0)		0 (0-0)		0 (0-0)		0 (0-0)		0 (0-0)		0 (0-0)	
2010	Iceland	0 (0-0)		0 (0-0)		0 (0-0)		0 (0-0)		0 (0-0)		0 (0-0)	
2011	Iceland	0 (0-0)		0 (0-0)		0 (0-0)		0 (0-0)		0 (0-0)		0 (0-0)	
2012	Iceland	0 (0-0)		0 (0-0)		0 (0-0)		0 (0-0)		0 (0-0)		0 (0-0)	
2013	Iceland	0 (0-0)		0 (0-0)		0 (0-0)		0 (0-0)		0 (0-0)		0 (0-0)	
2014	Iceland	0 (0-0)		0 (0-0)		0 (0-0)		0 (0-0)		0 (0-0)		0 (0-0)	

Spn = *Streptococcus pneumoniae*; npnm = non-pneumonia, non-meningitis; HIV = deaths only in HIV-infected children; Uncertainty range provided in parentheses.

Year	Country	Spn pneumonia deaths (UR)	Spn pneumonia deaths, HIV (UR)	Spn pneumonia mortality rate (UR)	Spn pneumonia mortality rate, HIV (UR)	Spn meningitis deaths (UR)	Spn meningitis deaths, HIV (UR)	Spn meningitis mortality rate (UR)	Spn meningitis mortality rate, HIV (UR)	Spn NPNM deaths (UR)	Spn NPNM deaths, HIV (UR)	Spn NPNM mortality rate (UR)	Spn NPNM mortality rate, HIV (UR)
2015	Iceland	0 (0-0)	0 (0-0)	0 (0-0)	0 (0-0)	0 (0-0)	0 (0-0)	0 (0-0)	0 (0-0)	0 (0-0)	0 (0-0)	0 (0-0)	0 (0-0)
2000	India	135807 (96300-141532)		108 (77-113)		20968 (9949-40003)		17 (8-32)		8753 (4125-16722)		7 (3-13)	
2001	India	130030 (92204-135511)		103 (73-107)		20069 (9462-38367)		16 (7-30)		9079 (4231-17419)		7 (3-14)	
2002	India	124116 (88010-129348)		98 (69-102)		19152 (9030-36622)		15 (7-29)		8629 (4021-16562)		7 (3-13)	
2003	India	118216 (83827-123199)		93 (66-97)		18239 (8551-34937)		14 (7-27)		8667 (3986-16727)		7 (3-13)	
2004	India	112017 (79431-116739)		88 (62-92)		17286 (8086-33121)		14 (6-26)		8397 (3852-16194)		7 (3-13)	
2005	India	105898 (75092-110362)		83 (59-87)		16352 (7719-31246)		13 (6-25)		7913 (3687-15182)		6 (3-12)	
2006	India	100185 (71041-104408)		78 (56-82)		15480 (7289-29587)		12 (6-23)		8588 (4026-16397)		7 (3-13)	
2007	India	94169 (66775-98139)		74 (52-77)		14564 (6914-27763)		11 (5-22)		8259 (3914-15724)		6 (3-12)	
2008	India	88270 (62592-91991)		69 (49-72)		13653 (6478-26027)		11 (5-20)		7731 (3659-14709)		6 (3-12)	
2009	India	82173 (58269-85637)		65 (46-67)		12701 (5972-24286)		10 (5-19)		7731 (3620-14759)		6 (3-12)	
2010	India	76701 (54388-79934)		61 (43-63)		11838 (5565-22678)		9 (4-18)		7195 (3366-13753)		6 (3-11)	
2011	India	71503 (50702-74517)		57 (40-59)		11016 (4985-21407)		9 (4-17)		8762 (3968-16981)		7 (3-14)	
2012	India	65782 (46646-68555)		53 (37-55)		10119 (4562-19827)		8 (4-16)		8012 (3613-15663)		6 (3-13)	
2013	India	61383 (43527-63971)		50 (35-52)		9432 (4197-18554)		8 (3-15)		8036 (3576-15792)		7 (3-13)	
2014	India	56921 (40362-59320)		47 (33-48)		8739 (3892-17234)		7 (3-14)		7421 (3306-14620)		6 (3-12)	
2015	India	53296 (37792-55542)	747 (530-779)	44 (31-46)	1 (0-1)	8179 (3612-16163)	122 (54-241)	7 (3-13)	0 (0-0)	7226 (3191-14277)	108 (48-213)	6 (3-12)	0 (0-0)
2000	Indonesia	12608 (8941-13140)		60 (43-63)		1521 (551-3294)		7 (3-16)		1354 (491-2933)		6 (2-14)	
2001	Indonesia	11924 (8455-12426)		56 (40-59)		1434 (525-3098)		7 (2-15)		1277 (467-2758)		6 (2-13)	
2002	Indonesia	11400 (8084-11881)		54 (38-56)		1358 (501-2925)		6 (2-14)		1209 (446-2604)		6 (2-12)	
2003	Indonesia	10833 (7682-11289)		50 (36-53)		1275 (470-2741)		6 (2-13)		1135 (418-2440)		5 (2-11)	
2004	Indonesia	13236 (9386-13794)		61 (43-64)		1541 (577-3296)		7 (3-15)		1372 (513-2934)		6 (2-14)	
2005	Indonesia	9752 (6915-10163)		45 (32-47)		1124 (427-2389)		5 (2-11)		1001 (380-2127)		5 (2-10)	
2006	Indonesia	9263 (6568-9653)		42 (30-44)		1058 (408-2232)		5 (2-10)		942 (363-1987)		4 (2-9)	
2007	Indonesia	8736 (6195-9104)		40 (28-41)		988 (386-2069)		4 (2-9)		880 (344-1842)		4 (2-8)	
2008	Indonesia	8213 (5823-8559)		37 (26-39)		921 (369-1908)		4 (2-9)		820 (328-1699)		4 (1-8)	
2009	Indonesia	7836 (5556-8166)		35 (25-36)		872 (357-1787)		4 (2-8)		776 (318-1591)		3 (1-7)	
2010	Indonesia	7564 (5363-7883)		33 (24-35)		837 (351-1695)		4 (2-7)		745 (313-1509)		3 (1-7)	
2011	Indonesia	7434 (5272-7748)		33 (23-34)		820 (353-1639)		4 (2-7)		730 (314-1459)		3 (1-6)	
2012	Indonesia	7402 (5249-7714)		32 (23-33)		815 (359-1609)		4 (2-7)		726 (320-1432)		3 (1-6)	
2013	Indonesia	7472 (5299-7787)		32 (22-33)		823 (289-1942)		3 (1-8)		732 (257-1729)		3 (1-7)	
2014	Indonesia	7392 (5242-7704)		31 (22-32)		814 (287-1911)		3 (1-8)		725 (256-1701)		3 (1-7)	
2015	Indonesia	7221 (5120-7525)	159 (113-166)	30 (21-31)	1 (0-1)	796 (283-1858)	18 (6-41)	3 (1-8)	0 (0-0)	708 (252-1655)	16 (6-36)	3 (1-7)	0 (0-0)
2000	Iran (Islamic Republic of)	1943 (1378-2025)		31 (22-32)		225 (123-374)		4 (2-6)		200 (109-333)		3 (2-5)	
2001	Iran (Islamic Republic of)	1750 (1241-1824)		29 (21-30)		200 (110-336)		3 (2-6)		178 (98-299)		3 (2-5)	
2002	Iran (Islamic Republic of)	1584 (1123-1651)		27 (19-28)		179 (95-303)		3 (2-5)		159 (85-270)		3 (1-5)	
2003	Iran (Islamic Republic of)	1414 (1003-1474)		25 (18-26)		157 (75-322)		3 (1-6)		140 (67-287)		2 (1-5)	
2004	Iran (Islamic Republic of)	1294 (918-1348)		24 (17-25)		141 (64-293)		3 (1-5)		125 (57-261)		2 (1-5)	
2005	Iran (Islamic Republic of)	1186 (841-1236)		22 (16-23)		126 (54-268)		2 (1-5)		112 (48-238)		2 (1-4)	
2006	Iran (Islamic Republic of)	1122 (795-1169)		20 (14-21)		116 (48-253)		2 (1-5)		104 (42-225)		2 (1-4)	
2007	Iran (Islamic Republic of)	1070 (759-1115)		19 (13-19)		109 (43-241)		2 (1-4)		97 (38-214)		2 (1-4)	
2008	Iran (Islamic Republic of)	1029 (730-1073)		17 (12-18)		102 (39-231)		2 (1-4)		91 (34-206)		2 (1-3)	
2009	Iran (Islamic Republic of)	995 (705-1037)		16 (12-17)		97 (35-223)		2 (1-4)		87 (31-199)		1 (1-3)	
2010	Iran (Islamic Republic of)	978 (694-1020)		16 (11-16)		95 (33-220)		2 (1-3)		84 (29-196)		1 (0-3)	
2011	Iran (Islamic Republic of)	944 (669-984)		15 (10-15)		91 (32-211)		1 (0-3)		81 (28-188)		1 (0-3)	
2012	Iran (Islamic Republic of)	912 (646-950)		14 (10-14)		87 (31-203)		1 (0-3)		78 (27-181)		1 (0-3)	
2013	Iran (Islamic Republic of)	885 (628-922)		13 (9-14)		85 (30-198)		1 (0-3)		76 (27-176)		1 (0-3)	
2014	Iran (Islamic Republic of)	839 (595-875)		12 (9-13)		81 (29-187)		1 (0-3)		72 (26-167)		1 (0-2)	
2015	Iran (Islamic Republic of)	790 (560-824)	2 (1-2)	12 (8-12)	0 (0-0)	76 (28-176)	0 (0-0)	1 (0-3)	0 (0-0)	68 (25-157)	0 (0-0)	1 (0-2)	0 (0-0)
2000	Iraq	1648 (1169-1717)		44 (31-45)		170 (75-331)		4 (2-9)		151 (67-295)		4 (2-8)	
2001	Iraq	1638 (1161-1707)		42 (30-44)		169 (76-329)		4 (2-8)		151 (68-293)		4 (2-8)	
2002	Iraq	1622 (1150-1691)		41 (29-43)		170 (78-328)		4 (2-8)		151 (69-292)		4 (2-7)	
2003	Iraq	1610 (1142-1678)		40 (28-42)		171 (79-327)		4 (2-8)		152 (71-292)		4 (2-7)	
2004	Iraq	1601 (1136-1669)		39 (28-41)		170 (80-322)		4 (2-8)		151 (71-287)		4 (2-7)	
2005	Iraq	1604 (1137-1672)		38 (27-40)		168 (81-318)		4 (2-8)		150 (72-283)		4 (2-7)	
2006	Iraq	1605 (1138-1672)		38 (27-39)		166 (81-313)		4 (2-7)		148 (72-278)		3 (2-7)	
2007	Iraq	1628 (1155-1697)		37 (26-39)		166 (79-315)		4 (2-7)		148 (70-281)		3 (2-6)	
2008	Iraq	1662 (1178-1732)		37 (26-38)		168 (78-322)		4 (2-7)		149 (69-286)		3 (2-6)	
2009	Iraq	1692 (1200-1763)		36 (26-38)		168 (76-327)		4 (2-7)		150 (68-291)		3 (1-6)	
2010	Iraq	1707 (1211-1779)		35 (25-37)		167 (74-327)		3 (2-7)		148 (66-291)		3 (1-6)	
2011	Iraq	1697 (1203-1769)		34 (24-35)		162 (71-322)		3 (1-6)		144 (63-287)		3 (1-6)	
2012	Iraq	1705 (1209-1776)		33 (23-34)		159 (68-320)		3 (1-6)		142 (61-285)		3 (1-5)	
2013	Iraq	1708 (1211-1781)		32 (23-33)		157 (66-319)		3 (1-6)		140 (59-284)		3 (1-5)	
2014	Iraq	1672 (1186-1743)		30 (22-32)		152 (62-311)		3 (1-6)		136 (56-277)		2 (1-5)	
2015	Iraq	1669 (1183-1739)	0 (0-0)	30 (21-31)	0 (0-0)	151 (60-310)	0 (0-0)	3 (1-6)	0 (0-0)	134 (54-276)	0 (0-0)	2 (1-5)	0 (0-0)
2000	Ireland	5 (3-5)		2 (1-2)		8 (3-14)		3 (1-5)		7 (3-12)		3 (1-5)	
2001	Ireland	6 (4-7)		2 (2-2)		4 (2-8)		2 (1-3)		4 (2-7)		1 (1-3)	
2002	Ireland	1 (1-1)		0 (0-1)		5 (2-9)		2 (1-3)		4 (2-8)		2 (1-3)	
2003	Ireland	5 (4-6)		2 (1-2)		5 (2-8)		2 (1-3)		4 (2-8)		1 (1-3)	
2004	Ireland	2 (2-2)		1 (1-1)		5 (2-8)		2 (1-3)		4 (2-8)		1 (1-3)	
2005	Ireland	5 (3-5)		2 (1-2)		5 (2-10)		2 (1-3)		5 (2-9)		2 (1-3)	
2006	Ireland	4 (3-4)		1 (1-1)		2 (1-4)		1 (0-1)		2 (1-3)		1 (0-1)	
2007	Ireland	0 (0-0)		0 (0-0)		4 (2-7)		1 (1-2)		4 (2-6)		1 (0-2)	
2008	Ireland	1 (1-1)		0 (0-0)		4 (2-7)		1 (0-2)		3 (1-6)		1 (0-2)	
2009	Ireland	1 (1-1)		0 (0-0)		2 (1-4)		1 (0-1)		2 (1-4)		1 (0-1)	
2010	Ireland	4 (3-4)		1 (1-1)		2 (1-3)		0 (0-1)		1 (1-3)		0 (0-1)	
2011	Ireland	1 (1-1)		0 (0-0)		1 (1-2)		0 (0-1)		1 (1-2)		0 (0-1)	
2012	Ireland	1 (1-1)		0 (0-0)		1 (0-2)		0 (0-1)		1 (0-2)		0 (0-0)	
2013	Ireland	1 (1-1)		0 (0-0)		1 (0-2)		0 (0-0)		1 (0-1)		0 (0-0)	
2014	Ireland	0 (0-1)		0 (0-0)		1 (0-1)		0 (0-0)		0 (0-1)		0 (0-0)	
2015	Ireland	0 (0-0)	0 (0-0)	0 (0-0)	0 (0-0)	0 (0-1)	0 (0-0)	0 (0-0)	0 (0-0)	0 (0-1)	0 (0-0)	0 (0-0)	0 (0-0)
2000	Israel	4 (3-4)		1 (0-1)		1 (1-1)		0 (0-0)		1 (1-1)		0 (0-0)	
2001	Israel	5 (4-6)		1 (1-1)		4 (2-6)		1 (0-1)		4 (2-5)		1 (0-1)	
2002	Israel	7 (5-8)		1 (1-1)		10 (6-14)		2 (1-2)		9 (5-12)		1 (1-2)	
2003	Israel	2 (2-2)		0 (0-0)		8 (5-11)		1 (1-2)		7 (4-10)		1 (1-1)	
2004	Israel	10 (7-11)		2 (1-2)		4 (3-6)		1 (0-1)		4 (2-6)		1 (0-1)	
2005	Israel	9 (6-9)		1 (1-1)		3 (2-4)		0 (0-1)		3 (2-4)		0 (0-1)	
2006	Israel	3 (2-3)		0 (0-0)		9 (6-13)		1 (1-2)		8 (5-11)		1 (1-2)	

Spn = *Streptococcus pneumoniae*; npnm = non-pneumonia, non-meningitis; HIV = deaths only in HIV-infected children; Uncertainty range provided in parentheses.

Year	Country	Spn pneumonia deaths (UR)	Spn pneumonia deaths, HIV (UR)	Spn pneumonia mortality rate (UR)	Spn pneumonia mortality rate, HIV (UR)	Spn meningitis deaths (UR)	Spn meningitis deaths, HIV (UR)	Spn meningitis mortality rate (UR)	Spn meningitis mortality rate, HIV (UR)	Spn NPNM deaths (UR)	Spn NPNM deaths, HIV (UR)	Spn NPNM mortality rate (UR)	Spn NPNM mortality rate, HIV (UR)
2007	Israel	5 (3-5)		1 (0-1)		3 (2-5)		1 (0-1)		3 (2-4)		0 (0-1)	
2008	Israel	8 (6-9)		1 (1-1)		6 (4-9)		1 (1-1)		6 (3-8)		1 (0-1)	
2009	Israel	5 (4-6)		1 (1-1)		5 (3-7)		1 (0-1)		5 (3-6)		1 (0-1)	
2010	Israel	4 (3-4)		1 (0-1)		5 (3-7)		1 (0-1)		4 (3-6)		1 (0-1)	
2011	Israel	3 (2-3)		0 (0-0)		4 (2-6)		1 (0-1)		4 (2-5)		0 (0-1)	
2012	Israel	4 (3-4)		1 (0-1)		2 (1-3)		0 (0-0)		2 (1-3)		0 (0-0)	
2013	Israel	3 (2-3)		0 (0-0)		3 (2-4)		0 (0-1)		3 (2-4)		0 (0-0)	
2014	Israel	3 (2-3)		0 (0-0)		3 (2-4)		0 (0-1)		3 (2-4)		0 (0-0)	
2015	Israel	2 (2-2)	0 (0-0)	0 (0-0)	0 (0-0)	2 (1-3)	0 (0-0)	0 (0-0)	0 (0-0)	2 (1-3)	0 (0-0)	0 (0-0)	0 (0-0)
2000	Italy	17 (12-17)		1 (0-1)		5 (2-9)		0 (0-0)		5 (2-8)		0 (0-0)	
2001	Italy	18 (13-19)		1 (1-1)		6 (3-11)		0 (0-0)		6 (3-10)		0 (0-0)	
2002	Italy	24 (17-25)		1 (1-1)		6 (3-12)		0 (0-0)		6 (3-10)		0 (0-0)	
2003	Italy	11 (8-11)		0 (0-0)		10 (5-19)		0 (0-1)		9 (4-17)		0 (0-1)	
2004	Italy	13 (10-14)		1 (0-1)		7 (3-13)		0 (0-0)		6 (3-11)		0 (0-0)	
2005	Italy	14 (10-14)		1 (0-1)		7 (3-13)		0 (0-0)		6 (3-11)		0 (0-0)	
2006	Italy	12 (8-12)		0 (0-0)		9 (4-17)		0 (0-1)		8 (4-15)		0 (0-1)	
2007	Italy	10 (7-10)		0 (0-0)		8 (3-14)		0 (0-1)		7 (3-12)		0 (0-0)	
2008	Italy	11 (8-11)		0 (0-0)		4 (2-8)		0 (0-0)		4 (2-7)		0 (0-0)	
2009	Italy	6 (4-6)		0 (0-0)		6 (3-10)		0 (0-0)		5 (2-9)		0 (0-0)	
2010	Italy	7 (5-7)		0 (0-0)		4 (2-8)		0 (0-0)		4 (2-7)		0 (0-0)	
2011	Italy	8 (6-9)		0 (0-0)		2 (1-4)		0 (0-0)		2 (1-4)		0 (0-0)	
2012	Italy	4 (3-4)		0 (0-0)		1 (1-3)		0 (0-0)		1 (1-2)		0 (0-0)	
2013	Italy	3 (2-3)		0 (0-0)		1 (1-2)		0 (0-0)		1 (0-2)		0 (0-0)	
2014	Italy	2 (2-3)		0 (0-0)		1 (0-2)		0 (0-0)		1 (0-2)		0 (0-0)	
2015	Italy	2 (2-2)	0 (0-0)	0 (0-0)	0 (0-0)	1 (0-2)	0 (0-0)	0 (0-0)	0 (0-0)	1 (0-1)	0 (0-0)	0 (0-0)	0 (0-0)
2000	Jamaica	13 (9-13)		5 (3-5)		1 (0-4)		0 (0-1)		1 (0-3)		0 (0-1)	
2001	Jamaica	12 (8-12)		4 (3-4)		1 (0-4)		0 (0-1)		1 (0-3)		0 (0-1)	
2002	Jamaica	11 (8-12)		4 (3-4)		1 (0-3)		0 (0-1)		1 (0-3)		0 (0-1)	
2003	Jamaica	11 (8-11)		4 (3-4)		1 (0-4)		1 (0-2)		1 (0-4)		0 (0-2)	
2004	Jamaica	12 (8-12)		5 (3-5)		2 (0-6)		1 (0-2)		2 (0-6)		1 (0-2)	
2005	Jamaica	13 (9-14)		5 (4-5)		2 (1-7)		1 (0-3)		2 (1-6)		1 (0-2)	
2006	Jamaica	14 (10-14)		6 (4-6)		3 (1-7)		1 (0-3)		2 (1-6)		1 (0-3)	
2007	Jamaica	15 (10-15)		6 (5-7)		3 (1-7)		1 (0-3)		2 (1-7)		1 (0-3)	
2008	Jamaica	16 (12-17)		7 (5-7)		3 (1-8)		1 (0-4)		3 (1-7)		1 (0-3)	
2009	Jamaica	15 (11-16)		7 (5-7)		3 (1-8)		1 (0-3)		2 (1-7)		1 (0-3)	
2010	Jamaica	14 (10-15)		6 (5-7)		3 (1-7)		1 (0-3)		2 (1-6)		1 (0-3)	
2011	Jamaica	13 (9-14)		6 (4-6)		2 (1-6)		1 (0-3)		2 (1-6)		1 (0-3)	
2012	Jamaica	13 (9-14)		6 (4-6)		2 (1-6)		1 (0-3)		2 (1-5)		1 (0-3)	
2013	Jamaica	12 (8-12)		6 (4-6)		2 (1-5)		1 (0-3)		2 (1-5)		1 (0-2)	
2014	Jamaica	11 (8-11)		5 (4-6)		2 (1-5)		1 (0-2)		2 (1-4)		1 (0-2)	
2015	Jamaica	11 (7-11)	0 (0-0)	5 (4-5)	0 (0-0)	2 (1-5)	0 (0-0)	1 (0-2)	0 (0-0)	2 (1-4)	0 (0-0)	1 (0-2)	0 (0-0)
2000	Japan	113 (80-118)		2 (1-2)		28 (17-39)		0 (0-1)		25 (15-35)		0 (0-1)	
2001	Japan	89 (63-93)		2 (1-2)		26 (15-35)		0 (0-1)		23 (14-31)		0 (0-1)	
2002	Japan	98 (69-102)		2 (1-2)		20 (12-27)		0 (0-0)		17 (10-24)		0 (0-0)	
2003	Japan	99 (70-103)		2 (1-2)		23 (14-32)		0 (0-1)		21 (13-29)		0 (0-1)	
2004	Japan	80 (57-84)		1 (1-1)		18 (11-24)		0 (0-0)		16 (9-22)		0 (0-0)	
2005	Japan	88 (63-92)		2 (1-2)		20 (12-27)		0 (0-0)		17 (10-24)		0 (0-0)	
2006	Japan	76 (54-79)		1 (1-1)		15 (9-20)		0 (0-0)		13 (8-18)		0 (0-0)	
2007	Japan	80 (57-83)		1 (1-2)		19 (12-27)		0 (0-0)		17 (10-24)		0 (0-0)	
2008	Japan	83 (59-87)		2 (1-2)		11 (7-15)		0 (0-0)		10 (6-14)		0 (0-0)	
2009	Japan	63 (44-65)		1 (1-1)		15 (9-21)		0 (0-0)		13 (8-18)		0 (0-0)	
2010	Japan	71 (50-74)		1 (1-1)		20 (12-27)		0 (0-1)		18 (11-24)		0 (0-0)	
2011	Japan	69 (49-72)		1 (1-1)		9 (5-12)		0 (0-0)		8 (5-11)		0 (0-0)	
2012	Japan	65 (46-68)		1 (1-1)		7 (4-9)		0 (0-0)		6 (4-8)		0 (0-0)	
2013	Japan	60 (42-62)		1 (1-1)		7 (4-10)		0 (0-0)		7 (4-9)		0 (0-0)	
2014	Japan	57 (41-60)		1 (1-1)		7 (4-9)		0 (0-0)		6 (4-8)		0 (0-0)	
2015	Japan	46 (32-47)	0 (0-0)	1 (1-1)	0 (0-0)	6 (3-8)	0 (0-0)	0 (0-0)	0 (0-0)	5 (3-7)	0 (0-0)	0 (0-0)	0 (0-0)
2000	Jordan	126 (89-131)		18 (13-19)		9 (3-21)		1 (1-3)		8 (3-18)		1 (0-3)	
2001	Jordan	125 (89-131)		18 (13-19)		14 (5-32)		2 (1-5)		12 (5-28)		2 (1-4)	
2002	Jordan	127 (90-132)		18 (13-19)		16 (6-38)		2 (1-5)		15 (5-34)		2 (1-5)	
2003	Jordan	121 (86-127)		18 (12-18)		16 (6-37)		2 (1-5)		14 (5-32)		2 (1-5)	
2004	Jordan	116 (82-121)		17 (12-17)		15 (5-35)		2 (1-5)		13 (5-31)		2 (1-4)	
2005	Jordan	114 (81-119)		16 (11-17)		15 (5-35)		2 (1-5)		13 (5-31)		2 (1-4)	
2006	Jordan	113 (80-118)		15 (11-16)		15 (5-35)		2 (1-5)		13 (5-31)		2 (1-4)	
2007	Jordan	113 (80-118)		15 (10-15)		15 (5-35)		2 (1-5)		13 (5-31)		2 (1-4)	
2008	Jordan	112 (80-117)		14 (10-15)		15 (5-34)		2 (1-4)		13 (5-31)		2 (1-4)	
2009	Jordan	113 (80-118)		14 (10-14)		15 (5-34)		2 (1-4)		13 (5-30)		2 (1-4)	
2010	Jordan	112 (79-116)		13 (9-14)		14 (5-33)		2 (1-4)		13 (4-29)		1 (1-3)	
2011	Jordan	112 (79-117)		13 (9-13)		14 (5-32)		2 (1-4)		12 (4-29)		1 (1-3)	
2012	Jordan	112 (79-116)		12 (9-13)		14 (5-32)		2 (1-3)		12 (4-28)		1 (0-3)	
2013	Jordan	110 (78-115)		12 (8-12)		13 (5-31)		1 (1-3)		12 (4-27)		1 (0-3)	
2014	Jordan	108 (76-112)		11 (8-12)		13 (5-29)		1 (0-3)		11 (4-26)		1 (0-3)	
2015	Jordan	104 (74-108)	0 (0-0)	11 (8-11)	0 (0-0)	12 (5-28)	0 (0-0)	1 (0-3)	0 (0-0)	11 (4-25)	0 (0-0)	1 (0-3)	0 (0-0)
2000	Kazakhstan	499 (354-520)		45 (32-47)		52 (20-108)		5 (2-10)		46 (18-96)		4 (2-9)	
2001	Kazakhstan	470 (333-490)		44 (31-46)		48 (19-99)		4 (2-9)		42 (17-88)		4 (2-8)	
2002	Kazakhstan	461 (327-480)		43 (30-45)		46 (18-95)		4 (2-9)		41 (16-84)		4 (2-8)	
2003	Kazakhstan	455 (323-474)		41 (29-43)		44 (18-91)		4 (2-8)		39 (16-81)		4 (1-7)	
2004	Kazakhstan	450 (319-469)		39 (27-40)		43 (17-88)		4 (1-8)		38 (16-78)		3 (1-7)	
2005	Kazakhstan	449 (318-468)		37 (26-38)		42 (17-86)		3 (1-7)		38 (15-77)		3 (1-6)	
2006	Kazakhstan	444 (315-463)		34 (24-36)		42 (17-84)		3 (1-6)		37 (15-75)		3 (1-6)	
2007	Kazakhstan	435 (308-453)		32 (23-33)		41 (14-97)		3 (1-7)		36 (12-86)		3 (1-6)	
2008	Kazakhstan	424 (300-442)		30 (21-31)		40 (14-93)		3 (1-6)		35 (12-83)		2 (1-6)	
2009	Kazakhstan	405 (287-422)		27 (19-28)		38 (14-87)		3 (1-6)		34 (13-78)		2 (1-5)	
2010	Kazakhstan	388 (275-404)		25 (17-26)		37 (15-82)		2 (1-5)		33 (13-73)		2 (1-5)	
2011	Kazakhstan	363 (257-378)		22 (15-23)		34 (14-76)		2 (1-5)		30 (12-68)		2 (1-4)	
2012	Kazakhstan	285 (202-297)		16 (11-17)		26 (10-57)		1 (1-3)		23 (9-51)		1 (1-3)	
2013	Kazakhstan	233 (165-243)		13 (9-13)		21 (9-46)		1 (0-3)		19 (8-41)		1 (0-2)	
2014	Kazakhstan	200 (142-209)		11 (8-11)		18 (8-40)		1 (0-2)		16 (7-35)		1 (0-2)	

Spn = *Streptococcus pneumoniae*; npnm = non-pneumonia, non-meningitis; HIV = deaths only in HIV-infected children; Uncertainty range provided in parentheses.

Year	Country	Spn pneumonia deaths (UR)	Spn pneumonia deaths, HIV (UR)	Spn pneumonia mortality rate (UR)	Spn pneumonia mortality rate, HIV (UR)	Spn meningitis deaths (UR)	Spn meningitis deaths, HIV (UR)	Spn meningitis mortality rate (UR)	Spn meningitis mortality rate, HIV (UR)	Spn NPNM deaths (UR)	Spn NPNM deaths, HIV (UR)	Spn NPNM mortality rate (UR)	Spn NPNM mortality rate, HIV (UR)
2015	Kazakhstan	163 (115-170)	0 (0-0)	8 (6-9)	0 (0-0)	15 (6-32)	0 (0-0)	1 (0-2)	0 (0-0)	13 (6-28)	0 (0-0)	1 (0-1)	0 (0-0)
2000	Kenya	6300 (4467-6566)		126 (90-132)		1013 (405-2151)		20 (8-43)		318 (127-676)		6 (3-14)	
2001	Kenya	6245 (4428-6508)		121 (86-126)		1004 (404-2127)		19 (8-41)		316 (127-669)		6 (2-13)	
2002	Kenya	6180 (4382-6440)		116 (82-121)		994 (402-2100)		19 (8-39)		313 (127-660)		6 (2-12)	
2003	Kenya	6061 (4298-6316)		111 (78-115)		973 (396-2047)		18 (7-37)		306 (125-644)		6 (2-12)	
2004	Kenya	5919 (4197-6169)		105 (74-109)		946 (390-1981)		17 (7-35)		298 (123-623)		5 (2-11)	
2005	Kenya	5595 (3967-5831)		97 (68-101)		891 (372-1855)		15 (6-32)		280 (117-583)		5 (2-10)	
2006	Kenya	5451 (3866-5681)		91 (65-95)		866 (366-1796)		14 (6-30)		272 (115-565)		5 (2-9)	
2007	Kenya	5393 (3824-5621)		88 (62-92)		855 (367-1768)		14 (6-29)		269 (115-556)		4 (2-9)	
2008	Kenya	5176 (3670-5394)		82 (58-86)		820 (305-1740)		13 (5-28)		730 (271-1549)		12 (4-25)	
2009	Kenya	4954 (3513-5163)		77 (55-81)		782 (291-1659)		12 (5-26)		696 (259-1477)		11 (4-23)	
2010	Kenya	4713 (3342-4911)		72 (51-75)		741 (282-1561)		11 (4-24)		660 (251-1390)		10 (4-21)	
2011	Kenya	3168 (2246-3302)		48 (34-50)		469 (183-981)		7 (3-15)		418 (163-874)		6 (2-13)	
2012	Kenya	2665 (1890-2777)		39 (28-41)		397 (158-821)		6 (2-12)		353 (141-731)		5 (2-11)	
2013	Kenya	2430 (1723-2532)		35 (25-37)		379 (155-778)		6 (2-11)		338 (138-693)		5 (2-10)	
2014	Kenya	2192 (1554-2284)		32 (22-33)		343 (144-698)		5 (2-10)		306 (128-622)		4 (2-9)	
2015	Kenya	1548 (1098-1613)	216 (153-226)	22 (16-23)	3 (2-3)	234 (98-473)	33 (14-66)	3 (1-7)	0 (0-1)	208 (88-421)	29 (12-59)	3 (1-6)	0 (0-1)
2000	Kiribati	11 (8-11)		93 (66-97)		1 (0-2)		7 (3-19)		1 (0-2)		6 (2-17)	
2001	Kiribati	10 (7-11)		87 (62-91)		1 (0-2)		7 (2-18)		1 (0-2)		6 (2-16)	
2002	Kiribati	9 (7-10)		83 (59-86)		1 (0-2)		6 (2-17)		1 (0-2)		6 (2-15)	
2003	Kiribati	8 (6-9)		77 (55-80)		1 (0-2)		6 (2-15)		1 (0-2)		5 (2-14)	
2004	Kiribati	8 (6-8)		75 (53-78)		1 (0-2)		6 (2-15)		1 (0-1)		5 (2-13)	
2005	Kiribati	8 (6-9)		77 (55-81)		1 (0-2)		6 (2-16)		1 (0-1)		5 (2-14)	
2006	Kiribati	9 (6-9)		84 (59-87)		1 (0-2)		6 (2-17)		1 (0-2)		6 (2-15)	
2007	Kiribati	10 (7-10)		91 (65-95)		1 (0-2)		7 (2-18)		1 (0-2)		6 (2-16)	
2008	Kiribati	11 (8-12)		96 (68-100)		1 (0-2)		7 (3-19)		1 (0-2)		6 (2-17)	
2009	Kiribati	12 (9-13)		96 (68-100)		1 (0-3)		7 (3-20)		1 (0-2)		6 (2-17)	
2010	Kiribati	13 (9-14)		95 (67-99)		1 (0-3)		7 (3-19)		1 (0-2)		6 (2-17)	
2011	Kiribati	13 (9-14)		90 (64-94)		1 (0-3)		7 (3-18)		1 (0-2)		6 (2-16)	
2012	Kiribati	13 (9-13)		85 (60-88)		1 (0-3)		7 (2-17)		1 (0-2)		6 (2-15)	
2013	Kiribati	9 (6-9)		60 (42-62)		1 (0-2)		5 (2-13)		1 (0-2)		4 (2-12)	
2014	Kiribati	7 (5-7)		48 (34-50)		1 (0-2)		4 (2-11)		1 (0-1)		4 (1-10)	
2015	Kiribati	4 (3-4)	0 (0-0)	29 (21-30)	0 (0-0)	0 (0-1)	0 (0-0)	3 (1-7)	0 (0-0)	0 (0-1)	0 (0-0)	2 (1-6)	0 (0-0)
2000	Kuwait	13 (9-14)		6 (5-7)		3 (2-4)		1 (1-2)		2 (1-3)		1 (1-2)	
2001	Kuwait	12 (9-13)		6 (4-6)		5 (3-7)		3 (2-3)		5 (3-6)		2 (1-3)	
2002	Kuwait	12 (9-13)		6 (4-6)		1 (1-1)		0 (0-1)		1 (1-1)		0 (0-1)	
2003	Kuwait	10 (7-11)		5 (4-5)		4 (3-6)		2 (1-3)		4 (2-5)		2 (1-2)	
2004	Kuwait	9 (7-10)		4 (3-5)		3 (2-4)		1 (1-2)		2 (1-3)		1 (1-2)	
2005	Kuwait	9 (7-10)		4 (3-4)		4 (2-6)		2 (1-3)		4 (2-5)		2 (1-2)	
2006	Kuwait	8 (6-8)		4 (3-4)		1 (1-1)		0 (0-1)		1 (0-1)		0 (0-0)	
2007	Kuwait	14 (10-15)		6 (4-6)		2 (1-3)		1 (0-1)		2 (1-2)		1 (0-1)	
2008	Kuwait	9 (6-9)		3 (2-4)		0 (0-0)		0 (0-0)		0 (0-0)		0 (0-0)	
2009	Kuwait	12 (9-13)		4 (3-5)		3 (2-4)		1 (1-1)		2 (1-3)		1 (1-1)	
2010	Kuwait	13 (9-13)		4 (3-4)		2 (1-3)		1 (0-1)		2 (1-3)		1 (0-1)	
2011	Kuwait	16 (11-17)		5 (4-5)		4 (3-6)		1 (1-2)		4 (2-5)		1 (1-2)	
2012	Kuwait	11 (8-12)		3 (2-4)		3 (2-4)		1 (0-1)		2 (1-3)		1 (0-1)	
2013	Kuwait	10 (7-11)		3 (2-3)		2 (1-3)		1 (0-1)		2 (1-3)		1 (0-1)	
2014	Kuwait	10 (7-10)		3 (2-3)		2 (1-3)		1 (0-1)		2 (1-3)		1 (0-1)	
2015	Kuwait	9 (6-10)	0 (0-0)	3 (2-3)	0 (0-0)	2 (1-3)	0 (0-0)	1 (0-1)	0 (0-0)	2 (1-3)	0 (0-0)	1 (0-1)	0 (0-0)
2000	Kyrgyzstan	291 (207-304)		54 (38-57)		49 (19-103)		9 (3-19)		43 (16-92)		8 (3-17)	
2001	Kyrgyzstan	267 (190-279)		51 (36-53)		44 (17-94)		8 (3-18)		39 (15-84)		8 (3-16)	
2002	Kyrgyzstan	246 (174-256)		48 (34-50)		40 (15-86)		8 (3-17)		36 (14-76)		7 (3-15)	
2003	Kyrgyzstan	225 (160-235)		46 (32-48)		36 (14-78)		7 (3-16)		32 (12-69)		7 (2-14)	
2004	Kyrgyzstan	209 (148-218)		43 (31-45)		33 (13-71)		7 (3-15)		30 (11-64)		6 (2-13)	
2005	Kyrgyzstan	199 (141-207)		41 (29-43)		31 (12-67)		7 (2-14)		28 (10-60)		6 (2-12)	
2006	Kyrgyzstan	195 (138-203)		40 (28-41)		30 (11-65)		6 (2-13)		27 (10-58)		5 (2-12)	
2007	Kyrgyzstan	193 (137-201)		38 (27-39)		30 (11-64)		6 (2-13)		27 (10-57)		5 (2-11)	
2008	Kyrgyzstan	190 (135-198)		35 (25-37)		29 (11-62)		5 (2-12)		26 (10-56)		5 (2-10)	
2009	Kyrgyzstan	191 (135-199)		34 (24-35)		29 (11-62)		5 (2-11)		26 (10-56)		5 (2-10)	
2010	Kyrgyzstan	189 (134-197)		32 (22-33)		29 (11-61)		5 (2-10)		25 (9-55)		4 (2-9)	
2011	Kyrgyzstan	188 (134-196)		29 (21-31)		28 (7-73)		4 (1-11)		25 (7-65)		4 (1-10)	
2012	Kyrgyzstan	185 (131-193)		27 (19-28)		27 (7-71)		4 (1-10)		24 (6-63)		4 (1-9)	
2013	Kyrgyzstan	181 (129-189)		25 (18-26)		27 (7-69)		4 (1-10)		24 (6-62)		3 (1-9)	
2014	Kyrgyzstan	175 (124-182)		23 (17-24)		26 (7-67)		3 (1-9)		23 (6-59)		3 (1-8)	
2015	Kyrgyzstan	164 (116-171)	2 (2-2)	21 (15-22)	0 (0-0)	24 (6-62)	0 (0-1)	3 (1-8)	0 (0-0)	21 (5-55)	0 (0-1)	3 (1-7)	0 (0-0)
2000	Lao People's Democratic Republic	1206 (855-1257)		153 (109-160)		176 (61-404)		22 (8-51)		55 (19-127)		7 (2-16)	
2001	Lao People's Democratic Republic	1160 (822-1209)		151 (107-157)		167 (57-384)		22 (7-50)		53 (18-121)		7 (2-16)	
2002	Lao People's Democratic Republic	1118 (792-1165)		148 (105-154)		159 (54-365)		21 (7-48)		50 (17-115)		7 (2-15)	
2003	Lao People's Democratic Republic	1083 (768-1129)		145 (103-151)		151 (51-349)		20 (7-47)		48 (16-110)		6 (2-15)	
2004	Lao People's Democratic Republic	1051 (746-1096)		141 (100-147)		145 (49-334)		19 (7-45)		45 (15-105)		6 (2-14)	
2005	Lao People's Democratic Republic	1023 (725-1066)		137 (97-143)		138 (47-320)		19 (6-43)		44 (15-101)		6 (2-14)	
2006	Lao People's Democratic Republic	1000 (709-1043)		134 (95-139)		134 (45-309)		18 (6-41)		42 (14-97)		6 (2-13)	
2007	Lao People's Democratic Republic	976 (692-1017)		129 (91-134)		129 (45-294)		17 (6-39)		41 (14-92)		5 (2-12)	
2008	Lao People's Democratic Republic	955 (677-996)		124 (88-129)		125 (45-282)		16 (6-37)		39 (14-89)		5 (2-11)	
2009	Lao People's Democratic Republic	928 (658-967)		118 (84-123)		121 (45-268)		15 (6-34)		38 (14-84)		5 (2-11)	
2010	Lao People's Democratic Republic	901 (639-939)		113 (80-118)		117 (45-255)		15 (6-32)		37 (14-80)		5 (2-10)	
2011	Lao People's Democratic Republic	874 (619-910)		107 (76-111)		112 (45-242)		14 (6-30)		35 (14-76)		4 (2-9)	
2012	Lao People's Democratic Republic	835 (592-871)		101 (72-106)		107 (36-236)		13 (4-29)		95 (32-210)		12 (4-25)	
2013	Lao People's Democratic Republic	790 (560-823)		96 (68-100)		100 (35-221)		12 (4-27)		89 (31-197)		11 (4-24)	
2014	Lao People's Democratic Republic	502 (356-523)		61 (43-64)		59 (21-131)		7 (2-16)		53 (18-116)		6 (2-14)	
2015	Lao People's Democratic Republic	403 (286-420)	8 (5-8)	49 (35-51)	1 (1-1)	49 (17-108)	1 (0-2)	6 (2-13)	0 (0-0)	44 (15-96)	1 (0-2)	5 (2-12)	0 (0-0)
2000	Latvia	3 (2-3)		3 (2-3)		2 (1-3)		2 (1-3)		1 (1-3)		2 (1-3)	
2001	Latvia	4 (3-4)		4 (3-4)		2 (1-3)		2 (1-4)		2 (1-3)		2 (1-3)	
2002	Latvia	2 (1-2)		2 (1-2)		0 (0-1)		0 (0-1)		0 (0-1)		0 (0-1)	
2003	Latvia	2 (2-2)		2 (2-2)		2 (1-3)		2 (1-4)		2 (1-3)		2 (1-3)	
2004	Latvia	2 (1-2)		2 (1-2)		1 (1-2)		1 (1-2)		1 (0-2)		1 (0-2)	
2005	Latvia	4 (3-4)		4 (3-4)		1 (1-3)		1 (1-3)		1 (1-2)		1 (1-2)	
2006	Latvia	2 (1-2)		2 (1-2)		1 (1-3)		1 (1-3)		1 (1-2)		1 (1-2)	

Spn = *Streptococcus pneumoniae*; npnm = non-pneumonia, non-meningitis; HIV = deaths only in HIV-infected children; Uncertainty range provided in parentheses.

Year	Country	Spn pneumonia deaths (UR)	Spn pneumonia deaths, HIV (UR)	Spn pneumonia mortality rate (UR)	Spn pneumonia mortality rate, HIV (UR)	Spn meningitis deaths (UR)	Spn meningitis deaths, HIV (UR)	Spn meningitis mortality rate (UR)	Spn meningitis mortality rate, HIV (UR)	Spn NPNM deaths (UR)	Spn NPNM deaths, HIV (UR)	Spn NPNM mortality rate (UR)	Spn NPNM mortality rate, HIV (UR)
2007	Latvia	3 (2-3)		3 (2-3)		1 (0-1)		0 (0-1)		0 (0-1)		0 (0-1)	
2008	Latvia	0 (0-0)		0 (0-0)		1 (1-2)		1 (1-2)		1 (1-2)		1 (0-2)	
2009	Latvia	1 (1-1)		1 (1-1)		1 (0-2)		1 (0-2)		1 (0-1)		1 (0-1)	
2010	Latvia	2 (2-2)		2 (1-2)		0 (0-0)		0 (0-0)		0 (0-0)		0 (0-0)	
2011	Latvia	1 (1-1)		1 (1-1)		1 (1-2)		1 (0-2)		1 (0-2)		1 (0-2)	
2012	Latvia	1 (1-1)		1 (1-1)		0 (0-0)		0 (0-0)		0 (0-0)		0 (0-0)	
2013	Latvia	1 (1-1)		1 (1-1)		0 (0-0)		0 (0-0)		0 (0-0)		0 (0-0)	
2014	Latvia	1 (0-1)		1 (1-1)		0 (0-0)		0 (0-0)		0 (0-0)		0 (0-0)	
2015	Latvia	0 (0-0)	0 (0-0)	0 (0-1)	0 (0-0)	0 (0-0)	0 (0-0)	0 (0-0)	0 (0-0)	0 (0-0)	0 (0-0)	0 (0-0)	0 (0-0)
2000	Lebanon	32 (23-34)		11 (8-11)		3 (1-6)		1 (0-2)		2 (1-5)		1 (0-2)	
2001	Lebanon	29 (20-30)		9 (7-10)		2 (1-5)		1 (0-2)		2 (1-5)		1 (0-2)	
2002	Lebanon	27 (19-28)		9 (6-9)		2 (1-5)		1 (0-2)		2 (1-4)		1 (0-1)	
2003	Lebanon	27 (19-28)		8 (6-9)		3 (1-8)		1 (0-2)		3 (1-7)		1 (0-2)	
2004	Lebanon	25 (18-26)		8 (5-8)		3 (1-8)		1 (0-2)		3 (1-7)		1 (0-2)	
2005	Lebanon	22 (16-23)		7 (5-7)		3 (1-7)		1 (0-2)		3 (1-6)		1 (0-2)	
2006	Lebanon	19 (13-20)		6 (4-6)		2 (1-6)		1 (0-2)		2 (1-5)		1 (0-2)	
2007	Lebanon	16 (11-16)		5 (4-6)		2 (1-5)		1 (0-2)		2 (1-4)		1 (0-1)	
2008	Lebanon	13 (9-14)		5 (3-5)		2 (1-4)		1 (0-1)		2 (1-4)		1 (0-1)	
2009	Lebanon	11 (8-12)		4 (3-5)		2 (1-3)		1 (0-1)		1 (1-3)		1 (0-1)	
2010	Lebanon	11 (8-11)		4 (3-4)		1 (1-3)		1 (0-1)		1 (0-3)		0 (0-1)	
2011	Lebanon	11 (8-12)		4 (3-4)		2 (1-3)		1 (0-1)		1 (1-3)		0 (0-1)	
2012	Lebanon	12 (9-13)		4 (3-4)		2 (1-4)		0 (0-1)		1 (1-3)		0 (0-1)	
2013	Lebanon	14 (10-14)		4 (3-4)		2 (1-4)		0 (0-1)		2 (1-4)		0 (0-1)	
2014	Lebanon	15 (11-15)		3 (2-4)		2 (1-4)		0 (0-1)		2 (1-4)		0 (0-1)	
2015	Lebanon	15 (11-16)	0 (0-0)	3 (2-3)	0 (0-0)	2 (1-4)	0 (0-0)	0 (0-1)	0 (0-0)	2 (1-4)	0 (0-0)	0 (0-1)	0 (0-0)
2000	Lesotho	309 (219-322)		117 (83-121)		49 (20-103)		19 (8-39)		15 (6-32)		6 (2-12)	
2001	Lesotho	317 (225-331)		120 (85-125)		50 (21-103)		19 (8-39)		16 (6-33)		6 (2-12)	
2002	Lesotho	321 (228-335)		123 (87-128)		50 (21-104)		19 (8-39)		16 (7-33)		6 (3-12)	
2003	Lesotho	322 (228-335)		125 (89-130)		50 (22-103)		20 (9-40)		16 (7-32)		6 (3-13)	
2004	Lesotho	320 (227-334)		127 (90-132)		51 (22-102)		20 (9-40)		16 (7-32)		6 (3-13)	
2005	Lesotho	320 (227-334)		129 (91-134)		51 (23-102)		20 (9-41)		16 (7-32)		6 (3-13)	
2006	Lesotho	322 (228-336)		132 (93-137)		52 (23-102)		21 (10-42)		16 (7-32)		7 (3-13)	
2007	Lesotho	323 (229-337)		133 (94-138)		52 (24-102)		21 (10-42)		16 (7-32)		7 (3-13)	
2008	Lesotho	326 (231-340)		133 (95-139)		52 (24-102)		21 (10-42)		16 (8-32)		7 (3-13)	
2009	Lesotho	325 (231-339)		131 (93-137)		52 (24-101)		21 (10-41)		16 (8-32)		7 (3-13)	
2010	Lesotho	325 (230-338)		130 (92-135)		51 (24-100)		20 (10-40)		16 (8-32)		6 (3-13)	
2011	Lesotho	326 (231-340)		128 (91-133)		51 (24-100)		20 (9-39)		16 (7-31)		6 (3-12)	
2012	Lesotho	331 (235-345)		127 (90-132)		51 (24-100)		20 (9-38)		16 (7-31)		6 (3-12)	
2013	Lesotho	334 (237-348)		126 (89-131)		50 (23-99)		19 (9-37)		16 (7-31)		6 (3-12)	
2014	Lesotho	334 (237-348)		124 (88-129)		50 (23-98)		18 (8-36)		16 (7-31)		6 (3-11)	
2015	Lesotho	296 (210-309)	149 (106-155)	108 (77-113)	54 (39-57)	43 (20-85)	22 (10-43)	16 (7-31)	8 (4-16)	14 (6-27)	7 (3-13)	5 (2-10)	2 (1-5)
2000	Liberia	842 (597-878)		172 (122-180)		252 (159-404)		52 (33-83)		79 (50-127)		16 (10-26)	
2001	Liberia	838 (594-873)		163 (116-170)		249 (157-404)		49 (30-79)		78 (49-127)		15 (10-25)	
2002	Liberia	812 (576-846)		153 (109-160)		240 (150-395)		45 (28-75)		75 (47-124)		14 (9-23)	
2003	Liberia	776 (550-809)		145 (103-151)		231 (110-430)		43 (21-80)		73 (35-135)		14 (6-25)	
2004	Liberia	749 (531-781)		138 (98-144)		217 (103-413)		40 (19-76)		68 (32-130)		13 (6-24)	
2005	Liberia	740 (525-772)		133 (95-139)		207 (97-400)		37 (17-72)		65 (31-126)		12 (5-23)	
2006	Liberia	736 (522-767)		129 (91-134)		198 (92-388)		35 (16-68)		62 (29-122)		11 (5-21)	
2007	Liberia	740 (525-771)		125 (89-131)		191 (87-379)		32 (15-64)		60 (27-119)		10 (5-20)	
2008	Liberia	746 (529-778)		122 (87-127)		187 (84-374)		31 (14-61)		59 (26-117)		10 (4-19)	
2009	Liberia	743 (527-775)		118 (84-123)		180 (79-365)		29 (13-58)		57 (25-115)		9 (4-18)	
2010	Liberia	726 (515-757)		113 (80-117)		171 (74-350)		27 (12-54)		54 (23-110)		8 (4-17)	
2011	Liberia	701 (497-731)		107 (76-111)		162 (69-334)		25 (11-51)		51 (22-105)		8 (3-16)	
2012	Liberia	677 (480-706)		102 (72-106)		154 (65-320)		23 (10-48)		48 (20-101)		7 (3-15)	
2013	Liberia	646 (458-674)		96 (68-100)		146 (60-306)		22 (9-45)		46 (19-96)		7 (3-14)	
2014	Liberia	520 (369-542)		76 (54-80)		113 (39-247)		17 (6-36)		101 (35-220)		15 (5-32)	
2015	Liberia	452 (320-471)	25 (18-26)	65 (46-68)	4 (3-4)	97 (33-213)	5 (2-12)	14 (5-31)	1 (0-2)	87 (29-190)	5 (2-11)	13 (4-28)	1 (0-2)
2000	Libya	104 (74-108)		19 (13-20)		14 (9-19)		3 (2-3)		12 (8-16)		2 (1-3)	
2001	Libya	99 (70-103)		18 (13-19)		13 (8-18)		2 (1-3)		12 (7-16)		2 (1-3)	
2002	Libya	95 (67-99)		17 (12-18)		13 (8-17)		2 (1-3)		11 (7-15)		2 (1-3)	
2003	Libya	89 (63-93)		16 (11-16)		12 (7-16)		2 (1-3)		11 (7-14)		2 (1-3)	
2004	Libya	83 (59-87)		15 (10-15)		11 (7-15)		2 (1-3)		10 (6-13)		2 (1-2)	
2005	Libya	77 (55-81)		13 (9-14)		10 (7-14)		2 (1-2)		9 (6-12)		2 (1-2)	
2006	Libya	72 (51-75)		12 (8-12)		10 (6-13)		2 (1-2)		9 (5-12)		1 (1-2)	
2007	Libya	72 (51-75)		12 (8-12)		13 (8-18)		2 (1-3)		12 (7-16)		2 (1-3)	
2008	Libya	73 (51-76)		11 (8-12)		14 (9-19)		2 (1-3)		13 (8-17)		2 (1-3)	
2009	Libya	68 (48-70)		10 (7-11)		13 (8-17)		2 (1-3)		11 (7-15)		2 (1-2)	
2010	Libya	64 (45-67)		10 (7-10)		12 (7-16)		2 (1-2)		11 (7-14)		2 (1-2)	
2011	Libya	62 (44-64)		9 (7-10)		11 (7-15)		2 (1-2)		10 (6-13)		1 (1-2)	
2012	Libya	57 (40-59)		8 (6-9)		10 (6-14)		2 (1-2)		9 (6-12)		1 (1-2)	
2013	Libya	52 (37-54)		8 (6-8)		9 (6-12)		1 (1-2)		8 (5-11)		1 (1-2)	
2014	Libya	43 (30-44)		7 (5-7)		7 (5-10)		1 (1-2)		7 (4-9)		1 (1-1)	
2015	Libya	38 (27-40)	0 (0-0)	6 (4-6)	0 (0-0)	7 (4-9)	0 (0-0)	1 (1-1)	0 (0-0)	6 (4-8)	0 (0-0)	1 (1-1)	0 (0-0)
2000	Lithuania	10 (7-11)		6 (4-6)		3 (1-5)		1 (1-3)		2 (1-4)		1 (1-2)	
2001	Lithuania	7 (5-7)		4 (3-4)		3 (1-5)		2 (1-3)		3 (1-5)		1 (1-3)	
2002	Lithuania	10 (7-10)		6 (4-6)		3 (1-5)		2 (1-3)		3 (1-5)		2 (1-3)	
2003	Lithuania	7 (5-7)		4 (3-5)		3 (2-6)		2 (1-4)		3 (1-6)		2 (1-4)	
2004	Lithuania	8 (6-9)		6 (4-6)		1 (0-2)		1 (0-1)		1 (0-1)		1 (0-1)	
2005	Lithuania	9 (7-10)		6 (5-7)		2 (1-4)		2 (1-3)		2 (1-4)		1 (1-3)	
2006	Lithuania	7 (5-7)		5 (3-5)		3 (1-5)		2 (1-3)		2 (1-4)		2 (1-3)	
2007	Lithuania	4 (3-5)		3 (2-3)		2 (1-3)		1 (1-2)		2 (1-3)		1 (1-2)	
2008	Lithuania	4 (3-4)		3 (2-3)		2 (1-3)		1 (0-2)		1 (1-2)		1 (0-2)	
2009	Lithuania	8 (6-9)		6 (4-6)		1 (0-2)		1 (0-1)		1 (0-1)		1 (0-1)	
2010	Lithuania	2 (1-2)		1 (1-1)		3 (1-6)		2 (1-4)		3 (1-5)		2 (1-4)	
2011	Lithuania	1 (1-1)		1 (1-1)		2 (1-4)		1 (1-3)		2 (1-3)		1 (1-2)	
2012	Lithuania	5 (4-5)		3 (2-4)		2 (1-4)		1 (1-3)		2 (1-4)		1 (1-2)	
2013	Lithuania	3 (2-3)		2 (1-2)		2 (1-4)		1 (1-3)		2 (1-3)		1 (1-2)	
2014	Lithuania	3 (2-3)		2 (1-2)		2 (1-4)		1 (1-3)		2 (1-3)		1 (1-2)	

Spn = *Streptococcus pneumoniae*; npnm = non-pneumonia, non-meningitis; HIV = deaths only in HIV-infected children; Uncertainty range provided in parentheses.

Year	Country	Spn pneumonia deaths (UR)	Spn pneumonia deaths, HIV (UR)	Spn pneumonia mortality rate (UR)	Spn pneumonia mortality rate, HIV (UR)	Spn meningitis deaths (UR)	Spn meningitis deaths, HIV (UR)	Spn meningitis mortality rate (UR)	Spn meningitis mortality rate, HIV (UR)	Spn NPNM deaths (UR)	Spn NPNM deaths, HIV (UR)	Spn NPNM mortality rate (UR)	Spn NPNM mortality rate, HIV (UR)
2015	Lithuania	3 (2-3)	0 (0-0)	2 (1-2)	0 (0-0)	2 (1-4)	0 (0-0)	1 (1-3)	0 (0-0)	2 (1-3)	0 (0-0)	1 (1-2)	0 (0-0)
2000	Luxembourg	0 (0-0)		0 (0-0)		1 (0-1)		2 (1-4)		1 (0-1)		2 (1-4)	
2001	Luxembourg	0 (0-0)		0 (0-0)		0 (0-1)		2 (1-3)		0 (0-1)		1 (1-3)	
2002	Luxembourg	0 (0-0)		0 (0-0)		0 (0-0)		1 (0-1)		0 (0-0)		1 (0-1)	
2003	Luxembourg	0 (0-0)		0 (0-0)		0 (0-0)		0 (0-0)		0 (0-0)		0 (0-0)	
2004	Luxembourg	0 (0-0)		0 (0-0)		0 (0-0)		0 (0-0)		0 (0-0)		0 (0-0)	
2005	Luxembourg	0 (0-0)		0 (0-0)		0 (0-0)		0 (0-0)		0 (0-0)		0 (0-0)	
2006	Luxembourg	0 (0-0)		1 (1-1)		0 (0-0)		0 (0-0)		0 (0-0)		0 (0-0)	
2007	Luxembourg	0 (0-0)		1 (0-1)		0 (0-0)		0 (0-0)		0 (0-0)		0 (0-0)	
2008	Luxembourg	0 (0-0)		1 (0-1)		0 (0-0)		0 (0-1)		0 (0-0)		0 (0-1)	
2009	Luxembourg	0 (0-0)		0 (0-0)		0 (0-0)		1 (0-1)		0 (0-0)		0 (0-1)	
2010	Luxembourg	0 (0-0)		0 (0-0)		0 (0-0)		0 (0-1)		0 (0-0)		0 (0-1)	
2011	Luxembourg	0 (0-0)		0 (0-0)		0 (0-0)		0 (0-0)		0 (0-0)		0 (0-0)	
2012	Luxembourg	0 (0-0)		0 (0-0)		0 (0-0)		0 (0-0)		0 (0-0)		0 (0-0)	
2013	Luxembourg	0 (0-0)		0 (0-0)		0 (0-0)		0 (0-0)		0 (0-0)		0 (0-0)	
2014	Luxembourg	0 (0-0)		0 (0-0)		0 (0-0)		0 (0-0)		0 (0-0)		0 (0-0)	
2015	Luxembourg	0 (0-0)	0 (0-0)	0 (0-0)	0 (0-0)	0 (0-0)	0 (0-0)	0 (0-0)	0 (0-0)	0 (0-0)	0 (0-0)	0 (0-0)	0 (0-0)
2000	Madagascar	4461 (3163-4649)		160 (113-166)		801 (322-1701)		29 (12-61)		252 (101-535)		9 (4-19)	
2001	Madagascar	4297 (3047-4479)		150 (106-156)		775 (312-1644)		27 (11-57)		244 (98-517)		8 (3-18)	
2002	Madagascar	4100 (2907-4273)		140 (99-146)		745 (301-1579)		25 (10-54)		234 (95-496)		8 (3-17)	
2003	Madagascar	3892 (2760-4056)		131 (93-137)		711 (288-1504)		24 (10-51)		223 (91-473)		8 (3-16)	
2004	Madagascar	3675 (2606-3830)		122 (87-127)		671 (272-1423)		22 (9-47)		211 (86-447)		7 (3-15)	
2005	Madagascar	3494 (2477-3641)		114 (81-119)		634 (254-1352)		21 (8-44)		199 (80-425)		7 (3-14)	
2006	Madagascar	3312 (2348-3451)		107 (76-112)		595 (236-1277)		19 (8-41)		187 (74-402)		6 (2-13)	
2007	Madagascar	3167 (2246-3301)		101 (71-105)		563 (185-1255)		18 (6-40)		502 (164-1118)		16 (5-36)	
2008	Madagascar	3049 (2162-3178)		95 (68-99)		538 (174-1208)		17 (5-38)		479 (155-1075)		15 (5-34)	
2009	Madagascar	2964 (2102-3089)		91 (64-95)		523 (170-1175)		16 (5-36)		465 (151-1046)		14 (5-32)	
2010	Madagascar	2875 (2039-2996)		86 (61-90)		508 (164-1145)		15 (5-34)		452 (146-1019)		14 (4-31)	
2011	Madagascar	2813 (1995-2932)		83 (59-86)		499 (161-1127)		15 (5-33)		444 (143-1003)		13 (4-30)	
2012	Madagascar	2754 (1953-2870)		79 (56-83)		491 (157-1109)		14 (5-32)		437 (140-987)		13 (4-28)	
2013	Madagascar	1945 (1379-2027)		55 (39-57)		329 (105-744)		9 (3-21)		293 (94-662)		8 (3-19)	
2014	Madagascar	1725 (1223-1797)		48 (34-50)		294 (95-663)		8 (3-18)		261 (84-590)		7 (2-16)	
2015	Madagascar	1590 (1128-1657)	25 (18-26)	43 (30-45)	1 (0-1)	282 (91-635)	4 (1-10)	8 (2-17)	0 (0-0)	251 (81-566)	4 (1-9)	7 (2-15)	0 (0-0)
2000	Malawi	4149 (2942-4324)		207 (146-215)		948 (491-1847)		47 (24-92)		298 (154-581)		15 (8-29)	
2001	Malawi	3921 (2780-4086)		190 (135-198)		899 (472-1746)		44 (23-85)		283 (148-549)		14 (7-27)	
2002	Malawi	3606 (2557-3758)		171 (121-178)		828 (295-1839)		39 (14-87)		260 (93-578)		12 (4-27)	
2003	Malawi	3291 (2334-3430)		153 (108-159)		759 (276-1675)		35 (13-78)		238 (87-527)		11 (4-24)	
2004	Malawi	3036 (2153-3164)		138 (98-144)		703 (260-1539)		32 (12-70)		221 (82-484)		10 (4-22)	
2005	Malawi	2835 (2010-2954)		126 (89-131)		660 (260-1414)		29 (12-63)		208 (82-445)		9 (4-20)	
2006	Malawi	2759 (1956-2875)		119 (85-124)		646 (271-1349)		28 (12-58)		203 (85-424)		9 (4-18)	
2007	Malawi	2651 (1880-2763)		111 (79-116)		624 (271-1273)		26 (11-53)		196 (85-400)		8 (4-17)	
2008	Malawi	2569 (1822-2678)		104 (74-109)		608 (275-1211)		25 (11-49)		191 (86-381)		8 (4-15)	
2009	Malawi	2552 (1810-2660)		101 (71-105)		606 (286-1182)		24 (11-47)		191 (90-372)		8 (4-15)	
2010	Malawi	2430 (1723-2532)		93 (66-97)		578 (287-1106)		22 (11-42)		182 (90-348)		7 (3-13)	
2011	Malawi	2508 (1778-2613)		93 (66-97)		596 (292-1138)		22 (11-42)		187 (92-358)		7 (3-13)	
2012	Malawi	1556 (1103-1622)		57 (40-59)		339 (165-650)		12 (6-24)		107 (52-204)		4 (2-7)	
2013	Malawi	1327 (941-1383)		47 (34-49)		289 (123-573)		10 (4-20)		258 (110-511)		9 (4-18)	
2014	Malawi	1177 (835-1227)		41 (29-43)		270 (115-536)		9 (4-19)		240 (103-477)		8 (4-17)	
2015	Malawi	1076 (763-1121)	233 (165-243)	37 (26-39)	8 (6-8)	246 (107-490)	53 (23-106)	8 (4-17)	2 (1-4)	219 (95-436)	47 (21-94)	8 (3-15)	2 (1-3)
2000	Malaysia	141 (100-147)		5 (4-5)		39 (23-54)		1 (1-2)		35 (21-48)		1 (1-2)	
2001	Malaysia	122 (86-127)		5 (3-5)		34 (20-47)		1 (1-2)		30 (18-42)		1 (1-2)	
2002	Malaysia	110 (78-114)		4 (3-4)		31 (18-42)		1 (1-2)		27 (16-38)		1 (1-1)	
2003	Malaysia	99 (70-103)		4 (3-4)		28 (17-38)		1 (1-1)		25 (15-34)		1 (1-1)	
2004	Malaysia	99 (70-103)		4 (3-4)		40 (24-55)		2 (1-2)		35 (21-49)		1 (1-2)	
2005	Malaysia	97 (69-101)		4 (3-4)		40 (24-55)		2 (1-2)		36 (21-49)		1 (1-2)	
2006	Malaysia	89 (63-93)		4 (3-4)		37 (22-51)		2 (1-2)		33 (20-46)		1 (1-2)	
2007	Malaysia	80 (57-83)		3 (2-3)		33 (20-46)		1 (1-2)		30 (18-41)		1 (1-2)	
2008	Malaysia	74 (53-78)		3 (2-3)		31 (19-43)		1 (1-2)		28 (17-38)		1 (1-2)	
2009	Malaysia	76 (54-79)		3 (2-3)		31 (19-43)		1 (1-2)		28 (17-39)		1 (1-2)	
2010	Malaysia	75 (53-78)		3 (2-3)		31 (19-43)		1 (1-2)		28 (17-38)		1 (1-2)	
2011	Malaysia	76 (54-79)		3 (2-4)		31 (19-43)		1 (1-2)		28 (17-38)		1 (1-2)	
2012	Malaysia	75 (53-78)		3 (2-3)		30 (18-42)		1 (1-2)		27 (16-37)		1 (1-2)	
2013	Malaysia	73 (52-76)		3 (2-3)		29 (18-40)		1 (1-2)		26 (16-36)		1 (1-2)	
2014	Malaysia	70 (50-73)		3 (2-3)		28 (17-38)		1 (1-2)		25 (15-34)		1 (1-1)	
2015	Malaysia	69 (49-72)	0 (0-0)	3 (2-3)	0 (0-0)	27 (16-37)	0 (0-0)	1 (1-2)	0 (0-0)	24 (14-33)	0 (0-0)	1 (1-1)	0 (0-0)
2000	Maldives	12 (8-12)		37 (26-38)		1 (0-2)		3 (1-7)		1 (0-2)		3 (1-7)	
2001	Maldives	9 (7-10)		30 (22-32)		1 (0-2)		3 (1-6)		1 (0-2)		2 (1-6)	
2002	Maldives	7 (5-8)		24 (17-25)		1 (0-2)		2 (1-5)		1 (0-1)		2 (0-5)	
2003	Maldives	6 (4-6)		21 (15-21)		1 (0-2)		2 (0-5)		0 (0-1)		2 (0-5)	
2004	Maldives	5 (4-5)		17 (12-18)		0 (0-1)		2 (0-4)		0 (0-1)		1 (0-4)	
2005	Maldives	4 (3-4)		14 (10-15)		0 (0-1)		1 (0-4)		0 (0-1)		1 (0-3)	
2006	Maldives	4 (3-4)		12 (9-13)		0 (0-1)		1 (0-3)		0 (0-1)		1 (0-3)	
2007	Maldives	3 (2-3)		11 (7-11)		0 (0-1)		1 (0-3)		0 (0-1)		1 (0-2)	
2008	Maldives	3 (2-3)		9 (7-10)		0 (0-1)		1 (0-2)		0 (0-1)		1 (0-2)	
2009	Maldives	3 (2-3)		8 (6-8)		0 (0-1)		1 (0-2)		0 (0-1)		1 (0-2)	
2010	Maldives	2 (2-2)		7 (5-7)		0 (0-1)		1 (0-2)		0 (0-1)		1 (0-2)	
2011	Maldives	2 (1-2)		6 (4-6)		0 (0-1)		1 (0-2)		0 (0-0)		0 (0-1)	
2012	Maldives	2 (1-2)		5 (4-5)		0 (0-0)		0 (0-1)		0 (0-0)		0 (0-1)	
2013	Maldives	2 (1-2)		5 (3-5)		0 (0-1)		1 (0-2)		0 (0-1)		1 (0-2)	
2014	Maldives	2 (1-2)		5 (3-5)		0 (0-1)		1 (0-2)		0 (0-1)		1 (0-2)	
2015	Maldives	2 (1-2)	0 (0-0)	4 (3-4)	0 (0-0)	0 (0-1)	0 (0-0)	1 (0-2)	0 (0-0)	0 (0-1)	0 (0-0)	1 (0-2)	0 (0-0)
2000	Mali	5390 (3822-5617)		263 (187-274)		996 (532-1882)		49 (26-92)		313 (167-592)		15 (8-29)	
2001	Mali	5389 (3822-5616)		254 (180-265)		987 (528-1863)		47 (25-88)		310 (166-586)		15 (8-28)	
2002	Mali	5294 (3754-5517)		242 (172-252)		963 (516-1816)		44 (24-83)		303 (162-571)		14 (7-26)	
2003	Mali	5040 (3574-5253)		224 (159-233)		912 (490-1718)		40 (22-76)		287 (154-540)		13 (7-24)	
2004	Mali	4922 (3490-5130)		211 (150-220)		888 (478-1670)		38 (21-72)		279 (150-525)		12 (6-23)	
2005	Mali	4900 (3474-5106)		203 (144-212)		880 (475-1655)		37 (20-69)		277 (149-520)		11 (6-22)	
2006	Mali	5019 (3559-5231)		200 (142-208)		895 (485-1685)		36 (19-67)		281 (152-530)		11 (6-21)	

Spn = *Streptococcus pneumoniae*; npnm = non-pneumonia, non-meningitis; HIV = deaths only in HIV-infected children; Uncertainty range provided in parentheses.

Year	Country	Spn pneumonia deaths (UR)	Spn pneumonia deaths, HIV (UR)	Spn pneumonia mortality rate (UR)	Spn pneumonia mortality rate, HIV (UR)	Spn meningitis deaths (UR)	Spn meningitis deaths, HIV (UR)	Spn meningitis mortality rate (UR)	Spn meningitis mortality rate, HIV (UR)	Spn NPNM deaths (UR)	Spn NPNM deaths, HIV (UR)	Spn NPNM mortality rate (UR)	Spn NPNM mortality rate, HIV (UR)
2007	Mali	5314 (3768-5538)		204 (145-213)		940 (513-1772)		36 (20-68)		295 (161-557)		11 (6-21)	
2008	Mali	5316 (3770-5540)		197 (140-206)		932 (351-2012)		35 (13-75)		293 (110-633)		11 (4-23)	
2009	Mali	5468 (3877-5698)		197 (139-205)		953 (363-2061)		34 (13-74)		300 (114-648)		11 (4-23)	
2010	Mali	5360 (3801-5586)		187 (133-195)		932 (359-2015)		33 (13-70)		293 (113-633)		10 (4-22)	
2011	Mali	4115 (2918-4289)		139 (99-145)		690 (268-1489)		23 (9-50)		217 (84-468)		7 (3-16)	
2012	Mali	3274 (2322-3412)		108 (77-112)		545 (213-1173)		18 (7-39)		171 (67-369)		6 (2-12)	
2013	Mali	2819 (1999-2938)		91 (65-95)		480 (189-1030)		16 (6-33)		151 (60-324)		5 (2-10)	
2014	Mali	2463 (1746-2566)		78 (55-81)		423 (168-904)		13 (5-29)		133 (53-284)		4 (2-9)	
2015	Mali	2597 (1841-2706)	220 (156-230)	81 (57-84)	7 (5-7)	459 (184-978)	39 (16-83)	14 (6-30)	1 (0-3)	144 (58-308)	12 (5-26)	4 (2-10)	0 (0-1)
2000	Malta	0 (0-0)		0 (0-0)		1 (0-1)		3 (1-5)		1 (0-1)		3 (1-5)	
2001	Malta	0 (0-0)		0 (0-0)		0 (0-1)		2 (1-4)		0 (0-1)		2 (1-3)	
2002	Malta	0 (0-0)		0 (0-0)		0 (0-0)		0 (0-0)		0 (0-0)		0 (0-0)	
2003	Malta	0 (0-0)		0 (0-0)		0 (0-0)		0 (0-0)		0 (0-0)		0 (0-0)	
2004	Malta	0 (0-0)		2 (1-2)		0 (0-0)		1 (0-1)		0 (0-0)		0 (0-1)	
2005	Malta	0 (0-0)		2 (1-2)		0 (0-0)		1 (0-1)		0 (0-0)		0 (0-1)	
2006	Malta	0 (0-0)		2 (1-2)		0 (0-0)		1 (0-1)		0 (0-0)		1 (0-1)	
2007	Malta	0 (0-0)		0 (0-0)		0 (0-0)		0 (0-0)		0 (0-0)		0 (0-0)	
2008	Malta	0 (0-0)		0 (0-0)		0 (0-0)		1 (0-1)		0 (0-0)		1 (0-1)	
2009	Malta	0 (0-0)		1 (0-1)		0 (0-0)		1 (1-2)		0 (0-0)		1 (0-2)	
2010	Malta	0 (0-0)		0 (0-0)		1 (0-1)		3 (1-5)		0 (0-1)		2 (1-4)	
2011	Malta	0 (0-0)		1 (1-1)		0 (0-1)		2 (1-4)		0 (0-1)		2 (1-3)	
2012	Malta	0 (0-0)		1 (1-1)		0 (0-1)		2 (1-4)		0 (0-1)		2 (1-4)	
2013	Malta	0 (0-0)		2 (1-2)		0 (0-0)		1 (1-3)		0 (0-0)		1 (1-2)	
2014	Malta	0 (0-0)		1 (1-1)		0 (0-1)		2 (1-4)		0 (0-1)		2 (1-3)	
2015	Malta	0 (0-0)	0 (0-0)	1 (1-1)	0 (0-0)	0 (0-1)	0 (0-0)	2 (1-4)	0 (0-0)	0 (0-1)	0 (0-0)	2 (1-3)	0 (0-0)
2000	Marshall Islands	5 (3-5)		44 (31-45)		0 (0-1)		3 (1-9)		0 (0-1)		2 (1-8)	
2001	Marshall Islands	5 (3-5)		44 (31-45)		0 (0-1)		3 (1-9)		0 (0-1)		2 (1-8)	
2002	Marshall Islands	5 (3-5)		45 (32-47)		0 (0-1)		3 (1-9)		0 (0-1)		2 (1-8)	
2003	Marshall Islands	4 (3-5)		44 (31-46)		0 (0-1)		3 (1-9)		0 (0-1)		2 (1-8)	
2004	Marshall Islands	4 (3-5)		45 (32-47)		0 (0-1)		3 (1-9)		0 (0-1)		2 (1-8)	
2005	Marshall Islands	4 (3-4)		45 (32-47)		0 (0-1)		3 (1-9)		0 (0-1)		2 (1-8)	
2006	Marshall Islands	4 (3-4)		46 (33-48)		0 (0-1)		3 (1-9)		0 (0-1)		2 (1-8)	
2007	Marshall Islands	4 (3-4)		47 (33-49)		0 (0-1)		3 (1-9)		0 (0-1)		3 (1-8)	
2008	Marshall Islands	4 (3-4)		47 (33-49)		0 (0-1)		3 (1-9)		0 (0-1)		2 (1-8)	
2009	Marshall Islands	3 (2-3)		38 (27-39)		0 (0-1)		2 (1-8)		0 (0-1)		2 (1-7)	
2010	Marshall Islands	3 (2-3)		41 (29-43)		0 (0-1)		2 (1-8)		0 (0-1)		2 (1-7)	
2011	Marshall Islands	3 (2-3)		37 (26-38)		0 (0-1)		2 (1-7)		0 (0-0)		2 (1-6)	
2012	Marshall Islands	2 (2-3)		33 (24-35)		0 (0-0)		2 (1-7)		0 (0-0)		2 (1-6)	
2013	Marshall Islands	3 (2-3)		36 (25-37)		0 (0-0)		2 (1-7)		0 (0-0)		2 (1-6)	
2014	Marshall Islands	3 (2-3)		37 (26-39)		0 (0-0)		2 (1-7)		0 (0-0)		2 (1-6)	
2015	Marshall Islands	1 (1-1)	0 (0-0)	11 (8-11)	0 (0-0)	0 (0-0)	0 (0-0)	1 (0-3)	0 (0-0)	0 (0-0)	0 (0-0)	1 (0-3)	0 (0-0)
2000	Mauritania	631 (447-657)		144 (102-150)		94 (36-204)		21 (8-47)		30 (11-64)		7 (3-15)	
2001	Mauritania	655 (464-682)		146 (103-152)		98 (37-212)		22 (8-47)		31 (12-67)		7 (3-15)	
2002	Mauritania	679 (481-707)		147 (104-153)		102 (39-220)		22 (8-48)		32 (12-69)		7 (3-15)	
2003	Mauritania	698 (495-728)		148 (105-154)		105 (41-226)		22 (9-48)		33 (13-71)		7 (3-15)	
2004	Mauritania	711 (504-741)		147 (104-153)		106 (41-228)		22 (9-47)		33 (13-72)		7 (3-15)	
2005	Mauritania	719 (510-749)		146 (103-152)		106 (42-228)		22 (8-46)		33 (13-72)		7 (3-15)	
2006	Mauritania	723 (513-754)		143 (101-149)		107 (42-229)		21 (8-45)		33 (13-72)		7 (3-14)	
2007	Mauritania	724 (513-755)		140 (99-146)		106 (42-228)		21 (8-44)		33 (13-72)		6 (3-14)	
2008	Mauritania	720 (510-750)		137 (97-143)		106 (42-227)		20 (8-43)		33 (13-71)		6 (2-14)	
2009	Mauritania	712 (505-742)		133 (94-139)		105 (41-226)		20 (8-42)		33 (13-71)		6 (2-13)	
2010	Mauritania	707 (502-737)		130 (92-135)		105 (41-225)		19 (8-41)		33 (13-71)		6 (2-13)	
2011	Mauritania	704 (500-734)		127 (90-132)		104 (41-224)		19 (7-40)		33 (13-71)		6 (2-13)	
2012	Mauritania	693 (491-722)		123 (87-128)		102 (40-219)		18 (7-39)		32 (13-69)		6 (2-12)	
2013	Mauritania	682 (483-710)		119 (84-124)		100 (39-214)		17 (7-37)		31 (12-67)		5 (2-12)	
2014	Mauritania	494 (351-515)		85 (60-88)		68 (27-146)		12 (5-25)		21 (8-46)		4 (1-8)	
2015	Mauritania	427 (303-445)	21 (15-22)	72 (51-75)	4 (3-4)	58 (23-125)	3 (1-6)	10 (4-21)	0 (0-1)	18 (7-39)	1 (0-2)	3 (1-7)	0 (0-0)
2000	Mauritius	4 (3-4)		4 (3-4)		3 (2-3)		3 (2-4)		2 (1-3)		2 (1-3)	
2001	Mauritius	6 (4-6)		6 (4-6)		3 (2-4)		3 (2-4)		2 (2-3)		3 (2-4)	
2002	Mauritius	4 (3-4)		5 (3-5)		1 (1-1)		1 (1-2)		1 (1-1)		1 (1-1)	
2003	Mauritius	7 (5-7)		7 (5-8)		2 (1-2)		2 (1-2)		1 (1-2)		2 (1-2)	
2004	Mauritius	7 (5-7)		8 (5-8)		2 (1-3)		2 (1-3)		2 (1-2)		2 (1-2)	
2005	Mauritius	5 (3-5)		5 (4-5)		2 (1-3)		2 (1-3)		2 (1-2)		2 (1-2)	
2006	Mauritius	6 (5-7)		7 (5-7)		6 (4-8)		6 (4-8)		5 (3-7)		6 (3-7)	
2007	Mauritius	8 (6-9)		9 (6-9)		1 (1-1)		1 (1-1)		1 (1-1)		1 (1-1)	
2008	Mauritius	8 (5-8)		9 (6-9)		3 (2-4)		3 (2-4)		3 (2-3)		3 (2-4)	
2009	Mauritius	6 (4-6)		7 (5-7)		2 (2-3)		3 (2-4)		2 (1-3)		3 (2-4)	
2010	Mauritius	6 (4-6)		7 (5-7)		2 (1-3)		3 (2-4)		2 (1-3)		3 (2-4)	
2011	Mauritius	6 (5-7)		9 (6-9)		2 (1-2)		2 (1-3)		1 (1-2)		2 (1-2)	
2012	Mauritius	7 (5-7)		9 (6-9)		0 (0-0)		0 (0-0)		0 (0-0)		0 (0-0)	
2013	Mauritius	8 (6-8)		11 (8-11)		1 (1-1)		1 (1-2)		1 (1-1)		1 (1-2)	
2014	Mauritius	6 (5-7)		9 (6-9)		1 (0-1)		1 (1-1)		1 (0-1)		1 (1-1)	
2015	Mauritius	6 (5-7)	0 (0-0)	9 (7-10)	0 (0-0)	1 (0-1)	0 (0-0)	1 (1-1)	0 (0-0)	1 (0-1)	0 (0-0)	1 (1-1)	0 (0-0)
2000	Mexico	3758 (2664-3916)		31 (22-33)		561 (275-869)		5 (2-7)		499 (245-774)		4 (2-6)	
2001	Mexico	3428 (2431-3572)		29 (20-30)		451 (222-699)		4 (2-6)		402 (197-622)		3 (2-5)	
2002	Mexico	3486 (2472-3633)		29 (21-30)		400 (197-620)		3 (2-5)		356 (175-552)		3 (1-5)	
2003	Mexico	2933 (2080-3057)		25 (18-26)		405 (199-628)		3 (2-5)		361 (177-559)		3 (1-5)	
2004	Mexico	2953 (2094-3077)		25 (18-26)		374 (184-580)		3 (2-5)		333 (164-516)		3 (1-4)	
2005	Mexico	2557 (1813-2665)		22 (15-23)		313 (154-486)		3 (1-4)		279 (137-432)		2 (1-4)	
2006	Mexico	2428 (1722-2531)		21 (15-22)		338 (166-524)		3 (1-5)		301 (148-467)		3 (1-4)	
2007	Mexico	2217 (1572-2310)		19 (14-20)		324 (159-502)		3 (1-4)		289 (142-447)		2 (1-4)	
2008	Mexico	2065 (1464-2152)		18 (13-19)		300 (147-465)		3 (1-4)		267 (131-414)		2 (1-4)	
2009	Mexico	1859 (1318-1937)		16 (11-17)		207 (101-320)		2 (1-3)		184 (90-285)		2 (1-2)	
2010	Mexico	1180 (837-1230)		10 (7-11)		164 (80-254)		1 (1-2)		146 (72-226)		1 (1-2)	
2011	Mexico	867 (615-904)		8 (5-8)		119 (58-185)		1 (1-2)		106 (52-164)		1 (0-1)	
2012	Mexico	652 (462-680)		6 (4-6)		87 (43-134)		1 (0-1)		77 (38-120)		1 (0-1)	
2013	Mexico	651 (461-678)		6 (4-6)		87 (43-134)		1 (0-1)		77 (38-119)		1 (0-1)	
2014	Mexico	611 (433-637)		5 (4-6)		81 (40-126)		1 (0-1)		72 (35-112)		1 (0-1)	

Spn = *Streptococcus pneumoniae*; npnm = non-pneumonia, non-meningitis; HIV = deaths only in HIV-infected children; Uncertainty range provided in parentheses.

Year	Country	Spn pneumonia deaths (UR)	Spn pneumonia deaths, HIV (UR)	Spn pneumonia mortality rate (UR)	Spn pneumonia mortality rate, HIV (UR)	Spn meningitis deaths (UR)	Spn meningitis deaths, HIV (UR)	Spn meningitis mortality rate (UR)	Spn meningitis mortality rate, HIV (UR)	Spn NPNM deaths (UR)	Spn NPNM deaths, HIV (UR)	Spn NPNM mortality rate (UR)	Spn NPNM mortality rate, HIV (UR)
2015	Mexico	583 (413-607)	0 (0-0)	5 (4-5)	0 (0-0)	77 (38-120)	0 (0-0)	1 (0-1)	0 (0-0)	69 (34-106)	0 (0-0)	1 (0-1)	0 (0-0)
2000	Micronesia (Federated States of)	9 (7-10)		63 (45-66)		1 (0-2)		4 (1-13)		1 (0-2)		4 (1-12)	
2001	Micronesia (Federated States of)	9 (6-9)		61 (43-64)		1 (0-2)		4 (1-13)		0 (0-2)		3 (1-11)	
2002	Micronesia (Federated States of)	8 (6-9)		58 (41-61)		1 (0-2)		4 (1-12)		0 (0-2)		3 (1-11)	
2003	Micronesia (Federated States of)	8 (6-8)		57 (41-60)		1 (0-2)		4 (1-12)		0 (0-1)		3 (1-11)	
2004	Micronesia (Federated States of)	7 (5-8)		55 (39-57)		0 (0-2)		3 (1-11)		0 (0-1)		3 (1-10)	
2005	Micronesia (Federated States of)	7 (5-7)		53 (38-56)		0 (0-1)		3 (1-11)		0 (0-1)		3 (1-10)	
2006	Micronesia (Federated States of)	7 (5-7)		51 (36-53)		0 (0-1)		3 (1-10)		0 (0-1)		3 (1-9)	
2007	Micronesia (Federated States of)	6 (4-6)		49 (35-51)		0 (0-1)		3 (1-10)		0 (0-1)		3 (1-9)	
2008	Micronesia (Federated States of)	6 (4-6)		48 (34-50)		0 (0-1)		3 (1-10)		0 (0-1)		3 (1-9)	
2009	Micronesia (Federated States of)	6 (4-6)		46 (33-48)		0 (0-1)		3 (1-9)		0 (0-1)		3 (1-8)	
2010	Micronesia (Federated States of)	3 (2-3)		26 (18-27)		0 (0-1)		2 (1-6)		0 (0-1)		2 (0-6)	
2011	Micronesia (Federated States of)	3 (2-3)		26 (18-27)		0 (0-1)		2 (0-6)		0 (0-1)		2 (0-5)	
2012	Micronesia (Federated States of)	3 (2-3)		26 (19-28)		0 (0-1)		2 (0-5)		0 (0-1)		2 (0-5)	
2013	Micronesia (Federated States of)	3 (2-3)		25 (18-26)		0 (0-1)		2 (0-5)		0 (0-1)		1 (0-5)	
2014	Micronesia (Federated States of)	3 (2-3)		23 (17-24)		0 (0-1)		1 (0-5)		0 (0-0)		1 (0-4)	
2015	Micronesia (Federated States of)	1 (1-1)	0 (0-0)	10 (7-10)	0 (0-0)	0 (0-0)	0 (0-0)	1 (0-3)	0 (0-0)	0 (0-0)	0 (0-0)	1 (0-2)	0 (0-0)
2000	Monaco	0 (0-0)		0 (0-0)		0 (0-0)		0 (0-0)		0 (0-0)		0 (0-0)	
2001	Monaco	0 (0-0)		0 (0-0)		0 (0-0)		0 (0-0)		0 (0-0)		0 (0-0)	
2002	Monaco	0 (0-0)		0 (0-0)		0 (0-0)		0 (0-0)		0 (0-0)		0 (0-0)	
2003	Monaco	0 (0-0)		1 (1-1)		0 (0-0)		1 (0-1)		0 (0-0)		1 (0-1)	
2004	Monaco	0 (0-0)		1 (1-1)		0 (0-0)		1 (0-1)		0 (0-0)		0 (0-1)	
2005	Monaco	0 (0-0)		0 (0-0)		0 (0-0)		0 (0-0)		0 (0-0)		0 (0-0)	
2006	Monaco	0 (0-0)		0 (0-0)		0 (0-0)		0 (0-0)		0 (0-0)		0 (0-0)	
2007	Monaco	0 (0-0)		0 (0-0)		0 (0-0)		0 (0-0)		0 (0-0)		0 (0-0)	
2008	Monaco	0 (0-0)		2 (1-2)		0 (0-0)		1 (0-2)		0 (0-0)		1 (0-2)	
2009	Monaco	0 (0-0)		3 (2-3)		0 (0-0)		1 (1-2)		0 (0-0)		1 (1-2)	
2010	Monaco	0 (0-0)		2 (2-3)		0 (0-0)		1 (1-2)		0 (0-0)		1 (0-2)	
2011	Monaco	0 (0-0)		2 (1-2)		0 (0-0)		1 (0-1)		0 (0-0)		1 (0-1)	
2012	Monaco	0 (0-0)		0 (0-0)		0 (0-0)		0 (0-0)		0 (0-0)		0 (0-0)	
2013	Monaco	0 (0-0)		0 (0-0)		0 (0-0)		0 (0-0)		0 (0-0)		0 (0-0)	
2014	Monaco	0 (0-0)		1 (0-1)		0 (0-0)		0 (0-0)		0 (0-0)		0 (0-0)	
2015	Monaco	0 (0-0)	0 (0-0)	0 (0-0)	0 (0-0)	0 (0-0)	0 (0-0)	0 (0-0)	0 (0-0)	0 (0-0)	0 (0-0)	0 (0-0)	0 (0-0)
2000	Mongolia	179 (127-186)		78 (55-81)		25 (12-49)		11 (5-21)		22 (10-44)		10 (5-19)	
2001	Mongolia	160 (113-166)		71 (50-74)		22 (10-44)		10 (4-20)		20 (9-39)		9 (4-17)	
2002	Mongolia	142 (101-148)		64 (45-67)		19 (8-39)		9 (4-18)		17 (7-35)		8 (3-16)	
2003	Mongolia	129 (92-135)		60 (42-62)		17 (7-36)		8 (3-16)		15 (6-32)		7 (3-15)	
2004	Mongolia	118 (84-123)		55 (39-57)		15 (6-32)		7 (3-15)		14 (5-29)		6 (2-13)	
2005	Mongolia	110 (78-115)		51 (36-53)		14 (5-30)		6 (2-14)		13 (5-26)		6 (2-12)	
2006	Mongolia	104 (74-109)		47 (33-49)		13 (5-27)		6 (2-12)		12 (5-24)		5 (2-11)	
2007	Mongolia	101 (72-105)		43 (31-45)		13 (5-25)		5 (2-11)		11 (5-23)		5 (2-10)	
2008	Mongolia	99 (70-103)		40 (28-42)		12 (6-24)		5 (2-10)		11 (5-22)		4 (2-9)	
2009	Mongolia	98 (70-103)		38 (27-39)		13 (6-23)		5 (2-9)		11 (5-21)		4 (2-8)	
2010	Mongolia	96 (68-100)		35 (25-36)		13 (6-22)		5 (2-8)		11 (6-20)		4 (2-7)	
2011	Mongolia	94 (67-98)		32 (23-33)		13 (7-25)		4 (2-8)		11 (6-22)		4 (2-7)	
2012	Mongolia	92 (65-96)		30 (21-31)		13 (9-23)		4 (3-7)		12 (8-20)		4 (2-7)	
2013	Mongolia	88 (63-92)		28 (20-29)		14 (9-21)		4 (3-7)		12 (8-19)		4 (3-6)	
2014	Mongolia	85 (60-88)		26 (18-27)		14 (9-20)		4 (3-6)		12 (8-18)		4 (2-6)	
2015	Mongolia	81 (57-84)	0 (0-0)	24 (17-25)	0 (0-0)	14 (8-19)	0 (0-0)	4 (3-6)	0 (0-0)	12 (7-17)	0 (0-0)	4 (2-5)	0 (0-0)
2000	Montenegro	0 (0-0)		1 (1-1)		0 (0-0)		0 (0-0)		0 (0-0)		0 (0-0)	
2001	Montenegro	0 (0-1)		1 (0-1)		0 (0-0)		0 (0-0)		0 (0-0)		0 (0-0)	
2002	Montenegro	0 (0-0)		1 (0-1)		0 (0-1)		0 (0-1)		0 (0-0)		0 (0-1)	
2003	Montenegro	0 (0-0)		0 (0-0)		0 (0-1)		0 (0-1)		0 (0-0)		0 (0-1)	
2004	Montenegro	0 (0-0)		1 (1-1)		0 (0-1)		1 (0-3)		0 (0-1)		1 (0-2)	
2005	Montenegro	1 (0-1)		2 (1-2)		0 (0-1)		0 (0-1)		0 (0-0)		0 (0-1)	
2006	Montenegro	1 (1-1)		3 (2-3)		0 (0-1)		1 (0-2)		0 (0-1)		1 (0-2)	
2007	Montenegro	1 (1-1)		3 (2-3)		0 (0-0)		0 (0-1)		0 (0-0)		0 (0-1)	
2008	Montenegro	1 (0-1)		2 (1-2)		0 (0-0)		0 (0-1)		0 (0-0)		0 (0-1)	
2009	Montenegro	0 (0-0)		0 (0-0)		0 (0-1)		1 (0-1)		0 (0-1)		0 (0-1)	
2010	Montenegro	0 (0-0)		1 (1-1)		0 (0-0)		0 (0-1)		0 (0-0)		0 (0-1)	
2011	Montenegro	0 (0-0)		1 (1-1)		0 (0-0)		0 (0-1)		0 (0-0)		0 (0-1)	
2012	Montenegro	0 (0-0)		1 (1-1)		0 (0-0)		0 (0-1)		0 (0-0)		0 (0-1)	
2013	Montenegro	0 (0-0)		1 (1-1)		0 (0-0)		0 (0-1)		0 (0-0)		0 (0-1)	
2014	Montenegro	0 (0-0)		1 (1-1)		0 (0-0)		0 (0-1)		0 (0-0)		0 (0-1)	
2015	Montenegro	0 (0-0)	0 (0-0)	1 (1-1)	0 (0-0)	0 (0-0)	0 (0-0)	0 (0-1)	0 (0-0)	0 (0-0)	0 (0-0)	0 (0-1)	0 (0-0)
2000	Morocco	1477 (1047-1539)		49 (34-51)		189 (58-434)		6 (2-14)		169 (52-386)		6 (2-13)	
2001	Morocco	1356 (961-1413)		46 (32-48)		172 (53-395)		6 (2-13)		153 (47-351)		5 (2-12)	
2002	Morocco	1249 (886-1302)		43 (30-45)		157 (49-359)		5 (2-12)		140 (44-320)		5 (1-11)	
2003	Morocco	1164 (826-1213)		40 (29-42)		145 (45-331)		5 (2-11)		129 (40-294)		4 (1-10)	
2004	Morocco	1095 (776-1141)		38 (27-40)		135 (42-307)		5 (1-11)		121 (38-274)		4 (1-10)	
2005	Morocco	1035 (734-1078)		36 (26-38)		127 (42-285)		4 (1-10)		114 (37-254)		4 (1-9)	
2006	Morocco	981 (696-1022)		34 (24-36)		120 (42-264)		4 (1-9)		107 (37-235)		4 (1-8)	
2007	Morocco	940 (667-980)		32 (23-34)		116 (42-248)		4 (1-9)		103 (38-221)		4 (1-8)	
2008	Morocco	902 (640-940)		31 (22-32)		111 (43-233)		4 (1-8)		99 (38-207)		3 (1-7)	
2009	Morocco	885 (628-922)		29 (21-31)		109 (45-224)		4 (2-7)		97 (40-199)		3 (1-7)	
2010	Morocco	862 (611-898)		28 (20-29)		107 (47-213)		3 (2-7)		95 (42-190)		3 (1-6)	
2011	Morocco	805 (571-839)		25 (18-27)		99 (44-196)		3 (1-6)		88 (39-174)		3 (1-6)	
2012	Morocco	609 (432-635)		19 (13-20)		72 (32-142)		2 (1-4)		64 (28-126)		2 (1-4)	
2013	Morocco	515 (365-537)		16 (11-16)		61 (21-143)		2 (1-4)		54 (18-127)		2 (1-4)	
2014	Morocco	340 (241-354)		10 (7-11)		39 (13-91)		1 (0-3)		35 (12-81)		1 (0-2)	
2015	Morocco	268 (190-279)	1 (1-1)	8 (6-8)	0 (0-0)	31 (11-73)	0 (0-0)	1 (0-2)	0 (0-0)	28 (10-65)	0 (0-0)	1 (0-2)	0 (0-0)
2000	Mozambique	5040 (3574-5252)		154 (109-160)		1233 (693-2213)		38 (21-68)		388 (218-696)		12 (7-21)	
2001	Mozambique	4979 (3531-5189)		147 (104-153)		1203 (686-2155)		36 (20-64)		378 (216-677)		11 (6-20)	
2002	Mozambique	4986 (3535-5196)		143 (101-149)		1187 (687-2124)		34 (20-61)		373 (216-668)		11 (6-19)	
2003	Mozambique	5027 (3565-5239)		140 (99-146)		1178 (498-2385)		33 (14-66)		370 (157-750)		10 (4-21)	
2004	Mozambique	5124 (3633-5340)		139 (98-145)		1183 (512-2388)		32 (14-65)		372 (161-751)		10 (4-20)	
2005	Mozambique	5231 (3709-5451)		138 (98-144)		1193 (528-2396)		31 (14-63)		375 (166-753)		10 (4-20)	
2006	Mozambique	5287 (3749-5509)		135 (96-141)		1193 (539-2380)		31 (14-61)		375 (169-748)		10 (4-19)	

Spn = *Streptococcus pneumoniae*; npnm = non-pneumonia, non-meningitis; HIV = deaths only in HIV-infected children; Uncertainty range provided in parentheses.

Year	Country	Spn pneumonia deaths (UR)	Spn pneumonia deaths, HIV (UR)	Spn pneumonia mortality rate (UR)	Spn pneumonia mortality rate, HIV (UR)	Spn meningitis deaths (UR)	Spn meningitis deaths, HIV (UR)	Spn meningitis mortality rate (UR)	Spn meningitis mortality rate, HIV (UR)	Spn NPNM deaths (UR)	Spn NPNM deaths, HIV (UR)	Spn NPNM mortality rate (UR)	Spn NPNM mortality rate, HIV (UR)
2007	Mozambique	5277 (3742-5499)		132 (94-137)		1177 (543-2333)		29 (14-58)		370 (171-733)		9 (4-18)	
2008	Mozambique	5207 (3693-5427)		127 (90-133)		1145 (542-2261)		28 (13-55)		360 (170-711)		9 (4-17)	
2009	Mozambique	5082 (3604-5297)		122 (86-127)		1095 (492-2203)		26 (12-53)		344 (155-693)		8 (4-17)	
2010	Mozambique	4967 (3522-5176)		116 (82-121)		1046 (449-2146)		24 (11-50)		329 (141-675)		8 (3-16)	
2011	Mozambique	4844 (3435-5048)		111 (79-115)		996 (410-2087)		23 (9-48)		313 (129-656)		7 (3-15)	
2012	Mozambique	4722 (3348-4921)		106 (75-110)		950 (376-2030)		21 (8-45)		299 (118-638)		7 (3-14)	
2013	Mozambique	3927 (2784-4092)		86 (61-90)		759 (289-1649)		17 (6-36)		239 (91-519)		5 (2-11)	
2014	Mozambique	3190 (2262-3325)		69 (49-72)		598 (219-1317)		13 (5-28)		188 (69-414)		4 (1-9)	
2015	Mozambique	2793 (1980-2910)	1031 (731-1075)	59 (42-61)	22 (15-23)	526 (185-1174)	194 (68-433)	11 (4-25)	4 (1-9)	165 (58-369)	61 (22-136)	3 (1-8)	1 (0-3)
2000	Myanmar	4718 (3346-4917)		92 (65-96)		964 (360-2084)		19 (7-41)		303 (113-655)		6 (2-13)	
2001	Myanmar	4648 (3296-4844)		89 (63-93)		924 (365-1961)		18 (7-38)		291 (115-617)		6 (2-12)	
2002	Myanmar	4563 (3236-4756)		86 (61-90)		884 (372-1839)		17 (7-35)		278 (117-578)		5 (2-11)	
2003	Myanmar	4436 (3146-4623)		83 (59-87)		837 (376-1706)		16 (7-32)		263 (118-536)		5 (2-10)	
2004	Myanmar	4265 (3024-4445)		80 (57-83)		783 (308-1632)		15 (6-31)		697 (274-1453)		13 (5-27)	
2005	Myanmar	4075 (2890-4247)		76 (54-80)		727 (287-1509)		14 (5-28)		647 (256-1343)		12 (5-25)	
2006	Myanmar	3862 (2738-4025)		72 (51-75)		670 (267-1387)		13 (5-26)		597 (238-1235)		11 (4-23)	
2007	Myanmar	3619 (2566-3771)		69 (49-71)		615 (246-1268)		12 (5-24)		547 (219-1129)		10 (4-21)	
2008	Myanmar	6132 (4348-6390)		118 (84-123)		1028 (472-2053)		20 (9-40)		323 (148-645)		6 (3-12)	
2009	Myanmar	3151 (2234-3284)		62 (44-65)		522 (212-1070)		10 (4-21)		465 (189-952)		9 (4-19)	
2010	Myanmar	2949 (2091-3073)		59 (42-62)		483 (196-989)		10 (4-20)		430 (175-881)		9 (4-18)	
2011	Myanmar	2729 (1935-2844)		57 (40-59)		441 (181-903)		9 (4-19)		393 (161-804)		8 (3-17)	
2012	Myanmar	2553 (1810-2660)		54 (38-57)		407 (168-832)		9 (4-18)		363 (150-740)		8 (3-16)	
2013	Myanmar	2419 (1716-2521)		52 (37-55)		382 (159-779)		8 (3-17)		341 (141-693)		7 (3-15)	
2014	Myanmar	2304 (1634-2401)		51 (36-53)		362 (151-735)		8 (3-16)		322 (135-654)		7 (3-14)	
2015	Myanmar	2189 (1552-2282)	79 (56-82)	49 (35-51)	2 (1-2)	342 (144-693)	12 (5-25)	8 (3-15)	0 (0-1)	304 (128-617)	11 (5-22)	7 (3-14)	0 (0-0)
2000	Namibia	191 (135-199)		70 (50-73)		23 (9-46)		8 (3-17)		7 (3-15)		3 (1-5)	
2001	Namibia	191 (136-199)		70 (50-73)		22 (10-46)		8 (4-17)		7 (3-15)		3 (1-5)	
2002	Namibia	191 (135-199)		70 (50-73)		22 (10-46)		8 (4-17)		7 (3-14)		3 (1-5)	
2003	Namibia	190 (135-198)		70 (50-73)		22 (10-45)		8 (4-17)		7 (3-14)		3 (1-5)	
2004	Namibia	190 (135-198)		70 (49-73)		22 (8-46)		8 (3-17)		19 (7-41)		7 (3-15)	
2005	Namibia	191 (135-199)		70 (49-73)		21 (8-45)		8 (3-16)		19 (7-40)		7 (3-15)	
2006	Namibia	190 (135-198)		69 (49-72)		21 (8-44)		8 (3-16)		19 (7-39)		7 (3-14)	
2007	Namibia	189 (134-197)		69 (49-71)		21 (8-44)		8 (3-16)		19 (7-39)		7 (3-14)	
2008	Namibia	190 (134-197)		68 (48-71)		21 (8-43)		8 (3-16)		19 (7-39)		7 (3-14)	
2009	Namibia	191 (136-199)		67 (48-70)		21 (9-44)		7 (3-15)		19 (8-39)		7 (3-14)	
2010	Namibia	194 (138-202)		67 (47-70)		21 (9-44)		7 (3-15)		19 (8-39)		7 (3-13)	
2011	Namibia	197 (140-205)		66 (47-69)		22 (9-44)		7 (3-15)		19 (8-39)		6 (3-13)	
2012	Namibia	201 (143-210)		66 (47-69)		22 (9-45)		7 (3-15)		20 (8-40)		6 (3-13)	
2013	Namibia	204 (145-213)		65 (46-67)		22 (10-45)		7 (3-14)		20 (9-40)		6 (3-13)	
2014	Namibia	206 (146-214)		63 (45-66)		22 (10-45)		7 (3-14)		20 (9-40)		6 (3-12)	
2015	Namibia	143 (102-149)	35 (25-37)	43 (31-45)	11 (8-11)	15 (7-29)	4 (2-7)	4 (2-9)	1 (0-2)	13 (6-26)	3 (1-6)	4 (2-8)	1 (0-2)
2000	Nauru	0 (0-0)		38 (27-39)		0 (0-0)		2 (1-6)		0 (0-0)		2 (1-5)	
2001	Nauru	0 (0-0)		38 (27-40)		0 (0-0)		2 (1-6)		0 (0-0)		2 (1-5)	
2002	Nauru	0 (0-0)		38 (27-40)		0 (0-0)		2 (1-6)		0 (0-0)		2 (1-5)	
2003	Nauru	0 (0-0)		37 (26-39)		0 (0-0)		2 (1-6)		0 (0-0)		2 (1-6)	
2004	Nauru	0 (0-0)		37 (26-38)		0 (0-0)		2 (1-6)		0 (0-0)		2 (1-6)	
2005	Nauru	0 (0-0)		37 (26-38)		0 (0-0)		2 (1-7)		0 (0-0)		2 (1-6)	
2006	Nauru	0 (0-0)		37 (27-39)		0 (0-0)		2 (1-7)		0 (0-0)		2 (1-6)	
2007	Nauru	0 (0-0)		38 (27-40)		0 (0-0)		2 (1-7)		0 (0-0)		2 (1-6)	
2008	Nauru	0 (0-0)		40 (28-41)		0 (0-0)		2 (1-7)		0 (0-0)		2 (1-7)	
2009	Nauru	0 (0-0)		28 (20-29)		0 (0-0)		2 (0-5)		0 (0-0)		1 (0-4)	
2010	Nauru	0 (0-0)		29 (20-30)		0 (0-0)		2 (1-5)		0 (0-0)		1 (0-5)	
2011	Nauru	0 (0-0)		29 (21-30)		0 (0-0)		2 (1-5)		0 (0-0)		1 (0-5)	
2012	Nauru	0 (0-0)		29 (21-31)		0 (0-0)		2 (1-5)		0 (0-0)		1 (0-5)	
2013	Nauru	0 (0-0)		30 (21-31)		0 (0-0)		2 (1-5)		0 (0-0)		1 (0-5)	
2014	Nauru	0 (0-0)		30 (21-31)		0 (0-0)		2 (1-5)		0 (0-0)		1 (0-5)	
2015	Nauru	0 (0-0)	0 (0-0)	31 (22-32)	0 (0-0)	0 (0-0)	0 (0-0)	2 (1-5)	0 (0-0)	0 (0-0)	0 (0-0)	2 (0-5)	0 (0-0)
2000	Nepal	3031 (2149-3159)		86 (61-90)		490 (153-1166)		14 (4-33)		154 (48-367)		4 (1-10)	
2001	Nepal	2835 (2011-2955)		80 (57-84)		457 (147-1080)		13 (4-31)		144 (46-340)		4 (1-10)	
2002	Nepal	2607 (1849-2717)		74 (53-77)		419 (113-1014)		12 (3-29)		373 (101-903)		11 (3-26)	
2003	Nepal	2390 (1694-2490)		69 (49-72)		384 (107-921)		11 (3-26)		342 (95-820)		10 (3-24)	
2004	Nepal	2171 (1540-2263)		63 (45-66)		349 (100-829)		10 (3-24)		310 (89-738)		9 (3-22)	
2005	Nepal	1984 (1407-2068)		59 (42-61)		318 (94-749)		9 (3-22)		283 (84-667)		8 (2-20)	
2006	Nepal	1802 (1278-1878)		54 (39-57)		289 (88-672)		9 (3-20)		257 (79-599)		8 (2-18)	
2007	Nepal	1653 (1172-1723)		51 (36-53)		264 (82-611)		8 (3-19)		235 (73-544)		7 (2-17)	
2008	Nepal	1509 (1070-1572)		47 (33-49)		240 (75-551)		7 (2-17)		213 (67-491)		7 (2-15)	
2009	Nepal	1390 (985-1448)		44 (31-46)		220 (70-503)		7 (2-16)		195 (62-448)		6 (2-14)	
2010	Nepal	1261 (894-1314)		41 (29-42)		198 (64-451)		6 (2-15)		176 (57-402)		6 (2-13)	
2011	Nepal	1153 (818-1202)		38 (27-40)		180 (59-408)		6 (2-13)		160 (53-364)		5 (2-12)	
2012	Nepal	1032 (732-1076)		35 (25-36)		160 (52-363)		5 (2-12)		142 (47-323)		5 (2-11)	
2013	Nepal	941 (668-981)		33 (23-34)		145 (48-329)		5 (2-11)		129 (42-293)		4 (1-10)	
2014	Nepal	869 (616-906)		31 (22-32)		134 (44-302)		5 (2-11)		119 (39-269)		4 (1-10)	
2015	Nepal	691 (490-721)	6 (4-6)	25 (18-26)	0 (0-0)	104 (34-235)	1 (0-2)	4 (1-9)	0 (0-0)	92 (30-209)	1 (0-2)	3 (1-8)	0 (0-0)
2000	Netherlands	9 (7-10)		1 (1-1)		12 (6-23)		1 (1-2)		11 (5-20)		1 (1-2)	
2001	Netherlands	11 (7-11)		1 (1-1)		12 (6-22)		1 (1-2)		11 (5-20)		1 (1-2)	
2002	Netherlands	8 (5-8)		1 (1-1)		12 (6-22)		1 (1-2)		11 (5-20)		1 (1-2)	
2003	Netherlands	8 (6-8)		1 (1-1)		11 (5-20)		1 (1-2)		10 (4-18)		1 (0-2)	
2004	Netherlands	8 (6-9)		1 (1-1)		4 (2-8)		0 (0-1)		4 (2-7)		0 (0-1)	
2005	Netherlands	10 (7-10)		1 (1-1)		9 (4-16)		1 (0-2)		8 (3-14)		1 (0-1)	
2006	Netherlands	7 (5-7)		1 (0-1)		4 (2-7)		0 (0-1)		3 (2-6)		0 (0-1)	
2007	Netherlands	6 (4-6)		1 (0-1)		6 (3-10)		1 (0-1)		5 (2-9)		1 (0-1)	
2008	Netherlands	6 (4-6)		1 (0-1)		4 (2-7)		0 (0-1)		4 (2-7)		0 (0-1)	
2009	Netherlands	5 (4-5)		1 (0-1)		4 (2-7)		0 (0-1)		4 (2-6)		0 (0-1)	
2010	Netherlands	4 (3-4)		0 (0-0)		3 (2-6)		0 (0-1)		3 (1-6)		0 (0-1)	
2011	Netherlands	3 (2-3)		0 (0-0)		3 (1-5)		0 (0-1)		2 (1-4)		0 (0-0)	
2012	Netherlands	3 (2-3)		0 (0-0)		1 (1-3)		0 (0-0)		1 (1-2)		0 (0-0)	
2013	Netherlands	2 (1-2)		0 (0-0)		0 (0-0)		0 (0-0)		0 (0-0)		0 (0-0)	
2014	Netherlands	2 (1-2)		0 (0-0)		1 (0-2)		0 (0-0)		1 (0-1)		0 (0-0)	

Spn = *Streptococcus pneumoniae*; npnm = non-pneumonia, non-meningitis; HIV = deaths only in HIV-infected children; Uncertainty range provided in parentheses.

Year	Country	Spn pneumonia deaths (UR)	Spn pneumonia deaths, HIV (UR)	Spn pneumonia mortality rate (UR)	Spn pneumonia mortality rate, HIV (UR)	Spn meningitis deaths (UR)	Spn meningitis deaths, HIV (UR)	Spn meningitis mortality rate (UR)	Spn meningitis mortality rate, HIV (UR)	Spn NPNM deaths (UR)	Spn NPNM deaths, HIV (UR)	Spn NPNM mortality rate (UR)	Spn NPNM mortality rate, HIV (UR)
2015	Netherlands	2 (1-2)	0 (0-0)	0 (0-0)	0 (0-0)	1 (0-1)	0 (0-0)	0 (0-0)	0 (0-0)	1 (0-1)	0 (0-0)	0 (0-0)	0 (0-0)
2000	New Zealand	9 (6-9)		3 (2-3)		8 (4-15)		3 (1-5)		7 (3-13)		3 (1-5)	
2001	New Zealand	4 (3-4)		1 (1-1)		10 (5-18)		4 (2-7)		9 (4-16)		3 (2-6)	
2002	New Zealand	5 (4-6)		2 (1-2)		8 (4-14)		3 (1-5)		7 (3-13)		3 (1-5)	
2003	New Zealand	4 (3-5)		2 (1-2)		6 (3-10)		2 (1-4)		5 (2-9)		2 (1-3)	
2004	New Zealand	13 (9-14)		5 (3-5)		6 (3-11)		2 (1-4)		5 (2-10)		2 (1-3)	
2005	New Zealand	8 (6-9)		3 (2-3)		7 (3-13)		3 (1-4)		6 (3-11)		2 (1-4)	
2006	New Zealand	10 (7-10)		3 (2-4)		1 (1-3)		1 (0-1)		1 (1-2)		0 (0-1)	
2007	New Zealand	10 (7-11)		4 (2-4)		4 (2-7)		1 (1-2)		4 (2-6)		1 (1-2)	
2008	New Zealand	8 (6-9)		3 (2-3)		6 (2-10)		2 (1-3)		5 (2-9)		2 (1-3)	
2009	New Zealand	6 (4-6)		2 (1-2)		1 (1-2)		0 (0-1)		1 (1-2)		0 (0-1)	
2010	New Zealand	5 (4-5)		2 (1-2)		3 (1-4)		1 (0-1)		2 (1-4)		1 (0-1)	
2011	New Zealand	4 (3-4)		1 (1-1)		4 (2-7)		1 (1-2)		4 (2-6)		1 (1-2)	
2012	New Zealand	3 (2-4)		1 (1-1)		2 (1-4)		1 (0-1)		2 (1-3)		1 (0-1)	
2013	New Zealand	2 (2-2)		1 (1-1)		1 (1-2)		0 (0-1)		1 (1-2)		0 (0-1)	
2014	New Zealand	2 (2-2)		1 (1-1)		1 (1-2)		0 (0-1)		1 (0-2)		0 (0-1)	
2015	New Zealand	2 (2-2)	0 (0-0)	1 (1-1)	0 (0-0)	1 (1-2)	0 (0-0)	0 (0-1)	0 (0-0)	1 (0-2)	0 (0-0)	0 (0-1)	0 (0-0)
2000	Nicaragua	351 (249-365)		54 (38-56)		37 (10-97)		6 (2-15)		33 (9-86)		5 (1-13)	
2001	Nicaragua	329 (233-343)		51 (36-53)		35 (10-92)		5 (2-14)		31 (9-81)		5 (1-13)	
2002	Nicaragua	300 (213-312)		47 (33-49)		32 (9-83)		5 (1-13)		28 (8-74)		4 (1-12)	
2003	Nicaragua	273 (194-285)		43 (30-45)		29 (9-76)		5 (1-12)		26 (8-68)		4 (1-11)	
2004	Nicaragua	260 (185-271)		41 (29-43)		29 (9-75)		5 (1-12)		26 (8-67)		4 (1-11)	
2005	Nicaragua	246 (175-257)		39 (28-41)		28 (8-72)		4 (1-11)		25 (7-64)		4 (1-10)	
2006	Nicaragua	239 (169-249)		38 (27-40)		27 (8-69)		4 (1-11)		24 (7-62)		4 (1-10)	
2007	Nicaragua	233 (165-243)		38 (27-39)		26 (6-82)		4 (1-13)		23 (5-73)		4 (1-12)	
2008	Nicaragua	228 (162-238)		37 (26-38)		26 (5-79)		4 (1-13)		23 (5-71)		4 (1-11)	
2009	Nicaragua	220 (156-229)		35 (25-37)		24 (5-76)		4 (1-12)		22 (5-68)		4 (1-11)	
2010	Nicaragua	210 (149-218)		34 (24-35)		23 (5-72)		4 (1-12)		21 (4-64)		3 (1-10)	
2011	Nicaragua	162 (115-169)		26 (19-27)		17 (4-52)		3 (1-8)		15 (3-47)		2 (1-8)	
2012	Nicaragua	113 (80-118)		18 (13-19)		11 (3-35)		2 (0-6)		10 (2-31)		2 (0-5)	
2013	Nicaragua	91 (64-95)		15 (11-16)		9 (2-28)		2 (0-5)		8 (2-25)		1 (0-4)	
2014	Nicaragua	78 (55-81)		13 (9-14)		8 (2-25)		1 (0-4)		7 (2-22)		1 (0-4)	
2015	Nicaragua	57 (40-59)	0 (0-0)	10 (7-10)	0 (0-0)	5 (1-17)	0 (0-0)	1 (0-3)	0 (0-0)	5 (1-15)	0 (0-0)	1 (0-3)	0 (0-0)
2000	Niger	8155 (5782-8498)		359 (255-374)		1693 (869-3273)		75 (38-144)		532 (273-1029)		23 (12-45)	
2001	Niger	8344 (5917-8696)		350 (248-365)		1742 (909-3342)		73 (38-140)		548 (286-1051)		23 (12-44)	
2002	Niger	8355 (5924-8707)		337 (239-351)		1757 (931-3340)		71 (38-135)		552 (293-1050)		22 (12-42)	
2003	Niger	8250 (5850-8598)		321 (228-335)		1747 (939-3288)		68 (37-128)		549 (295-1034)		21 (11-40)	
2004	Niger	8081 (5730-8422)		304 (216-317)		1726 (940-3211)		65 (35-121)		543 (296-1010)		20 (11-38)	
2005	Niger	7884 (5590-8216)		286 (203-298)		1693 (937-3120)		61 (34-113)		532 (295-981)		19 (11-36)	
2006	Niger	7689 (5452-8013)		268 (190-279)		1657 (935-3031)		58 (33-106)		521 (294-953)		18 (10-33)	
2007	Niger	7680 (5446-8004)		258 (183-268)		1654 (940-3025)		55 (32-101)		520 (296-951)		17 (10-32)	
2008	Niger	7083 (5023-7382)		229 (163-239)		1519 (613-3162)		49 (20-102)		478 (193-994)		15 (6-32)	
2009	Niger	6775 (4804-7061)		211 (150-220)		1447 (592-3016)		45 (18-94)		455 (186-948)		14 (6-30)	
2010	Niger	6777 (4806-7063)		203 (144-212)		1440 (596-3001)		43 (18-90)		453 (187-943)		14 (6-28)	
2011	Niger	6643 (4710-6923)		191 (135-199)		1406 (588-2923)		40 (17-84)		442 (185-919)		13 (5-26)	
2012	Niger	6570 (4658-6847)		181 (128-189)		1386 (585-2868)		38 (16-79)		436 (184-902)		12 (5-25)	
2013	Niger	6409 (4545-6679)		170 (121-177)		1349 (574-2781)		36 (15-74)		424 (180-874)		11 (5-23)	
2014	Niger	6141 (4355-6400)		157 (111-163)		1282 (550-2631)		33 (14-67)		403 (173-827)		10 (4-21)	
2015	Niger	4571 (3241-4763)	80 (57-83)	112 (80-117)	2 (1-2)	911 (395-1864)	16 (7-33)	22 (10-46)	0 (0-1)	286 (124-586)	5 (2-10)	7 (3-14)	0 (0-0)
2000	Nigeria	42934 (30444-44744)		207 (147-216)		6305 (3225-12265)		30 (16-59)		1982 (1014-3856)		10 (5-19)	
2001	Nigeria	43717 (31000-45560)		204 (145-213)		6368 (3296-12319)		30 (15-58)		2002 (1036-3873)		9 (5-18)	
2002	Nigeria	44229 (31363-46093)		201 (142-209)		6355 (3327-12216)		29 (15-55)		1998 (1046-3841)		9 (5-17)	
2003	Nigeria	43777 (31042-45622)		193 (137-201)		6158 (3258-11751)		27 (14-52)		1936 (1024-3694)		9 (5-16)	
2004	Nigeria	44065 (31247-45923)		189 (134-197)		6043 (3233-11452)		26 (14-49)		1900 (1016-3601)		8 (4-15)	
2005	Nigeria	44093 (31266-45952)		184 (131-192)		5919 (3208-11156)		25 (13-47)		1861 (1009-3507)		8 (4-15)	
2006	Nigeria	45173 (32032-47077)		183 (130-191)		5938 (3268-11156)		24 (13-45)		1867 (1028-3507)		8 (4-14)	
2007	Nigeria	45444 (32224-47360)		179 (127-187)		5869 (2265-12560)		23 (9-50)		1845 (712-3949)		7 (3-16)	
2008	Nigeria	45148 (32014-47051)		174 (123-181)		5752 (2281-12291)		22 (9-47)		1808 (717-3864)		7 (3-15)	
2009	Nigeria	45719 (32419-47646)		172 (122-179)		5743 (2245-12363)		22 (8-47)		1805 (706-3887)		7 (3-15)	
2010	Nigeria	46948 (33291-48927)		172 (122-180)		5804 (2237-12590)		21 (8-46)		1825 (703-3958)		7 (3-15)	
2011	Nigeria	47046 (33360-49029)		168 (119-175)		5737 (2180-12529)		21 (8-45)		1804 (685-3939)		6 (2-14)	
2012	Nigeria	46006 (32623-47946)		161 (114-167)		5546 (2066-12215)		19 (7-43)		1744 (649-3840)		6 (2-13)	
2013	Nigeria	46045 (32651-47986)		157 (111-164)		5518 (2014-12245)		19 (7-42)		1735 (633-3850)		6 (2-13)	
2014	Nigeria	45213 (32060-47118)		151 (107-157)		5404 (1933-12075)		18 (6-40)		1699 (608-3796)		6 (2-13)	
2015	Nigeria	42386 (30055-44172)	8333 (5909-8684)	139 (98-144)	27 (19-28)	5025 (1762-11299)	988 (346-2221)	16 (6-37)	3 (1-7)	1580 (554-3552)	311 (109-698)	5 (2-12)	1 (0-2)
2000	Niue	0 (0-0)		56 (39-58)		0 (0-0)		12 (5-22)		0 (0-0)		11 (5-19)	
2001	Niue	0 (0-0)		57 (41-60)		0 (0-0)		13 (6-23)		0 (0-0)		12 (5-21)	
2002	Niue	0 (0-0)		55 (39-58)		0 (0-0)		13 (6-23)		0 (0-0)		11 (5-20)	
2003	Niue	0 (0-0)		53 (38-55)		0 (0-0)		12 (6-22)		0 (0-0)		11 (5-20)	
2004	Niue	0 (0-0)		0 (0-0)		0 (0-0)		0 (0-0)		0 (0-0)		0 (0-0)	
2005	Niue	0 (0-0)		0 (0-0)		0 (0-0)		0 (0-0)		0 (0-0)		0 (0-0)	
2006	Niue	0 (0-0)		52 (37-54)		0 (0-0)		13 (6-23)		0 (0-0)		12 (5-21)	
2007	Niue	0 (0-0)		53 (38-55)		0 (0-0)		13 (6-23)		0 (0-0)		12 (5-21)	
2008	Niue	0 (0-0)		56 (39-58)		0 (0-0)		14 (6-25)		0 (0-0)		12 (6-22)	
2009	Niue	0 (0-0)		38 (27-40)		0 (0-0)		11 (5-19)		0 (0-0)		10 (4-17)	
2010	Niue	0 (0-0)		39 (27-40)		0 (0-0)		10 (5-18)		0 (0-0)		9 (4-16)	
2011	Niue	0 (0-0)		28 (20-30)		0 (0-0)		8 (4-14)		0 (0-0)		7 (3-13)	
2012	Niue	0 (0-0)		27 (19-28)		0 (0-0)		7 (3-12)		0 (0-0)		6 (3-11)	
2013	Niue	0 (0-0)		18 (13-18)		0 (0-0)		5 (2-9)		0 (0-0)		4 (2-8)	
2014	Niue	0 (0-0)		17 (12-17)		0 (0-0)		4 (2-7)		0 (0-0)		4 (2-7)	
2015	Niue	0 (0-0)	0 (0-0)	16 (11-17)	0 (0-0)	0 (0-0)	0 (0-0)	4 (2-7)	0 (0-0)	0 (0-0)	0 (0-0)	4 (2-6)	0 (0-0)
2000	Norway	3 (2-3)		1 (1-1)		5 (2-9)		2 (1-3)		5 (2-8)		2 (1-3)	
2001	Norway	2 (1-2)		1 (0-1)		0 (0-0)		0 (0-0)		0 (0-0)		0 (0-0)	
2002	Norway	2 (2-2)		1 (1-1)		1 (0-2)		0 (0-1)		1 (0-1)		0 (0-1)	
2003	Norway	2 (2-3)		1 (1-1)		2 (1-4)		1 (0-1)		2 (1-3)		1 (0-1)	
2004	Norway	1 (0-1)		0 (0-0)		1 (0-2)		0 (0-1)		1 (0-2)		0 (0-1)	
2005	Norway	2 (2-3)		1 (1-1)		0 (0-0)		0 (0-0)		0 (0-0)		0 (0-0)	
2006	Norway	0 (0-0)		0 (0-0)		2 (1-3)		1 (0-1)		2 (1-3)		1 (0-1)	

Spn = *Streptococcus pneumoniae*; npnm = non-pneumonia, non-meningitis; HIV = deaths only in HIV-infected children; Uncertainty range provided in parentheses.

Year	Country	Spn pneumonia deaths (UR)	Spn pneumonia deaths, HIV (UR)	Spn pneumonia mortality rate (UR)	Spn pneumonia mortality rate, HIV (UR)	Spn meningitis deaths (UR)	Spn meningitis deaths, HIV (UR)	Spn meningitis mortality rate (UR)	Spn meningitis mortality rate, HIV (UR)	Spn NPNM deaths (UR)	Spn NPNM deaths, HIV (UR)	Spn NPNM mortality rate (UR)	Spn NPNM mortality rate, HIV (UR)
2007	Norway	5 (3-5)		2 (1-2)		1 (1-3)		1 (0-1)		1 (1-2)		0 (0-1)	
2008	Norway	1 (0-1)		0 (0-0)		0 (0-0)		0 (0-0)		0 (0-0)		0 (0-0)	
2009	Norway	3 (2-3)		1 (1-1)		1 (1-2)		0 (0-1)		1 (0-2)		0 (0-1)	
2010	Norway	1 (1-1)		0 (0-0)		0 (0-1)		0 (0-0)		0 (0-1)		0 (0-0)	
2011	Norway	1 (1-1)		0 (0-0)		1 (0-2)		0 (0-0)		1 (0-1)		0 (0-0)	
2012	Norway	1 (1-1)		0 (0-0)		0 (0-0)		0 (0-0)		0 (0-0)		0 (0-0)	
2013	Norway	0 (0-0)		0 (0-0)		0 (0-0)		0 (0-0)		0 (0-0)		0 (0-0)	
2014	Norway	0 (0-0)		0 (0-0)		0 (0-0)		0 (0-0)		0 (0-0)		0 (0-0)	
2015	Norway	0 (0-0)	0 (0-0)	0 (0-0)	0 (0-0)	0 (0-0)	0 (0-0)	0 (0-0)	0 (0-0)	0 (0-0)	0 (0-0)	0 (0-0)	0 (0-0)
2000	Oman	33 (24-35)		12 (9-13)		4 (2-5)		1 (1-2)		3 (2-5)		1 (1-2)	
2001	Oman	29 (20-30)		11 (8-11)		3 (2-5)		1 (1-2)		3 (2-4)		1 (1-1)	
2002	Oman	28 (20-29)		11 (8-11)		5 (3-6)		2 (1-2)		4 (2-6)		2 (1-2)	
2003	Oman	26 (18-27)		10 (7-10)		4 (3-6)		2 (1-2)		4 (2-5)		1 (1-2)	
2004	Oman	24 (17-25)		9 (7-10)		4 (2-5)		2 (1-2)		4 (2-5)		1 (1-2)	
2005	Oman	23 (16-24)		9 (6-9)		4 (2-5)		1 (1-2)		3 (2-5)		1 (1-2)	
2006	Oman	22 (16-23)		9 (6-9)		3 (2-5)		1 (1-2)		3 (2-4)		1 (1-2)	
2007	Oman	22 (16-23)		9 (6-9)		4 (2-5)		1 (1-2)		3 (2-4)		1 (1-2)	
2008	Oman	22 (16-23)		9 (6-9)		4 (2-5)		1 (1-2)		3 (2-4)		1 (1-2)	
2009	Oman	19 (14-20)		7 (5-7)		3 (2-4)		1 (1-2)		3 (2-4)		1 (1-1)	
2010	Oman	16 (12-17)		5 (4-6)		3 (2-4)		1 (1-1)		3 (2-3)		1 (1-1)	
2011	Oman	13 (9-13)		4 (3-4)		2 (1-3)		1 (0-1)		2 (1-3)		1 (0-1)	
2012	Oman	12 (8-12)		4 (3-4)		2 (1-3)		1 (0-1)		2 (1-3)		1 (0-1)	
2013	Oman	10 (7-11)		3 (2-3)		2 (1-3)		1 (0-1)		2 (1-2)		0 (0-1)	
2014	Oman	10 (7-10)		3 (2-3)		2 (1-3)		1 (0-1)		2 (1-2)		0 (0-1)	
2015	Oman	10 (7-10)	0 (0-0)	3 (2-3)	0 (0-0)	2 (1-3)	0 (0-0)	1 (0-1)	0 (0-0)	2 (1-2)	0 (0-0)	0 (0-1)	0 (0-0)
2000	Pakistan	23715 (16817-24715)		120 (85-125)		3333 (1484-6642)		17 (8-34)		1048 (467-2088)		5 (2-11)	
2001	Pakistan	23339 (16550-24323)		117 (83-122)		3267 (1461-6498)		16 (7-33)		1027 (459-2043)		5 (2-10)	
2002	Pakistan	22769 (16146-23729)		115 (81-120)		3165 (1421-6283)		16 (7-32)		995 (447-1975)		5 (2-10)	
2003	Pakistan	22329 (15833-23270)		113 (80-118)		3075 (1385-6093)		16 (7-31)		967 (436-1916)		5 (2-10)	
2004	Pakistan	22049 (15635-22979)		112 (80-117)		3008 (1360-5949)		15 (7-30)		946 (428-1870)		5 (2-10)	
2005	Pakistan	22061 (15643-22991)		112 (79-117)		2981 (1352-5885)		15 (7-30)		937 (425-1850)		5 (2-9)	
2006	Pakistan	22049 (15635-22978)		111 (79-116)		2951 (1344-5820)		15 (7-29)		928 (422-1830)		5 (2-9)	
2007	Pakistan	22201 (15743-23137)		110 (78-114)		2941 (1341-5806)		15 (7-29)		925 (421-1825)		5 (2-9)	
2008	Pakistan	22404 (15887-23349)		108 (76-112)		2943 (1328-5834)		14 (6-28)		925 (417-1834)		4 (2-9)	
2009	Pakistan	22641 (16055-23595)		106 (75-110)		2950 (1319-5877)		14 (6-27)		927 (415-1848)		4 (2-9)	
2010	Pakistan	22655 (16064-23610)		103 (73-107)		2931 (1299-5870)		13 (6-27)		922 (408-1845)		4 (2-8)	
2011	Pakistan	22691 (16090-23647)		99 (71-104)		2915 (1280-5865)		13 (6-26)		917 (402-1844)		4 (2-8)	
2012	Pakistan	22356 (15853-23298)		96 (68-100)		2855 (1241-5766)		12 (5-25)		897 (390-1813)		4 (2-8)	
2013	Pakistan	15577 (11045-16233)		66 (47-69)		1877 (817-3795)		8 (3-16)		590 (257-1193)		2 (1-5)	
2014	Pakistan	13020 (9232-13568)		54 (39-57)		1614 (706-3258)		7 (3-14)		507 (222-1024)		2 (1-4)	
2015	Pakistan	12361 (8765-12882)	40 (28-42)	51 (36-53)	0 (0-0)	1565 (688-3155)	5 (2-10)	6 (3-13)	0 (0-0)	492 (216-992)	2 (1-3)	2 (1-4)	0 (0-0)
2000	Palau	0 (0-0)		17 (12-18)		0 (0-0)		6 (3-11)		0 (0-0)		5 (2-10)	
2001	Palau	0 (0-0)		16 (11-17)		0 (0-0)		6 (3-10)		0 (0-0)		5 (2-9)	
2002	Palau	0 (0-0)		19 (13-20)		0 (0-0)		7 (3-12)		0 (0-0)		6 (3-10)	
2003	Palau	0 (0-0)		12 (9-13)		0 (0-0)		5 (2-8)		0 (0-0)		4 (2-7)	
2004	Palau	0 (0-0)		15 (11-16)		0 (0-0)		5 (2-10)		0 (0-0)		5 (2-9)	
2005	Palau	0 (0-0)		14 (10-15)		0 (0-0)		5 (2-9)		0 (0-0)		5 (2-8)	
2006	Palau	0 (0-0)		14 (10-15)		0 (0-0)		5 (2-9)		0 (0-0)		5 (2-8)	
2007	Palau	0 (0-0)		12 (8-12)		0 (0-0)		4 (2-7)		0 (0-0)		4 (2-6)	
2008	Palau	0 (0-0)		13 (9-13)		0 (0-0)		4 (2-8)		0 (0-0)		4 (2-7)	
2009	Palau	0 (0-0)		9 (6-9)		0 (0-0)		3 (1-5)		0 (0-0)		2 (1-4)	
2010	Palau	0 (0-0)		13 (9-14)		0 (0-0)		4 (2-8)		0 (0-0)		4 (2-7)	
2011	Palau	0 (0-0)		11 (8-12)		0 (0-0)		4 (2-7)		0 (0-0)		3 (2-6)	
2012	Palau	0 (0-0)		5 (4-5)		0 (0-0)		2 (1-4)		0 (0-0)		2 (1-3)	
2013	Palau	0 (0-0)		3 (2-3)		0 (0-0)		1 (0-2)		0 (0-0)		1 (0-2)	
2014	Palau	0 (0-0)		3 (2-3)		0 (0-0)		1 (0-2)		0 (0-0)		1 (0-1)	
2015	Palau	0 (0-0)	0 (0-0)	3 (2-3)	0 (0-0)	0 (0-0)	0 (0-0)	1 (0-1)	0 (0-0)	0 (0-0)	0 (0-0)	1 (0-1)	0 (0-0)
2000	Panama	38 (27-40)		11 (8-12)		7 (2-20)		2 (1-6)		7 (2-18)		2 (1-5)	
2001	Panama	43 (31-45)		13 (9-13)		11 (3-29)		3 (1-9)		10 (3-26)		3 (1-8)	
2002	Panama	40 (28-41)		12 (8-12)		10 (3-28)		3 (1-8)		9 (3-25)		3 (1-7)	
2003	Panama	43 (31-45)		13 (9-13)		5 (1-13)		1 (0-4)		4 (1-11)		1 (0-3)	
2004	Panama	45 (32-46)		13 (9-14)		10 (3-26)		3 (1-8)		9 (3-23)		3 (1-7)	
2005	Panama	55 (39-58)		16 (12-17)		6 (2-17)		2 (1-5)		6 (2-15)		2 (1-5)	
2006	Panama	53 (38-55)		16 (11-17)		8 (2-20)		2 (1-6)		7 (2-18)		2 (1-5)	
2007	Panama	63 (45-66)		19 (13-20)		7 (2-20)		2 (1-6)		7 (2-18)		2 (1-5)	
2008	Panama	68 (48-71)		20 (14-21)		5 (1-13)		1 (0-4)		4 (1-11)		1 (0-3)	
2009	Panama	44 (31-46)		13 (9-13)		10 (3-27)		3 (1-8)		9 (3-24)		3 (1-7)	
2010	Panama	73 (52-76)		21 (15-22)		9 (3-23)		2 (1-6)		8 (2-20)		2 (1-6)	
2011	Panama	59 (42-62)		17 (12-17)		8 (3-21)		2 (1-6)		7 (2-19)		2 (1-5)	
2012	Panama	45 (32-47)		12 (9-13)		5 (1-12)		1 (0-3)		4 (1-11)		1 (0-3)	
2013	Panama	43 (31-45)		12 (9-13)		5 (2-13)		1 (0-4)		4 (1-11)		1 (0-3)	
2014	Panama	28 (20-29)		8 (5-8)		3 (1-8)		1 (0-2)		3 (1-7)		1 (0-2)	
2015	Panama	21 (15-22)	0 (0-0)	6 (4-6)	0 (0-0)	2 (1-6)	0 (0-0)	1 (0-2)	0 (0-0)	2 (1-6)	0 (0-0)	1 (0-2)	0 (0-0)
2000	Papua New Guinea	859 (609-895)		105 (74-109)		64 (21-196)		8 (3-24)		20 (7-62)		2 (1-8)	
2001	Papua New Guinea	875 (621-912)		104 (74-108)		66 (21-201)		8 (3-24)		21 (7-63)		2 (1-8)	
2002	Papua New Guinea	884 (627-921)		103 (73-108)		67 (22-204)		8 (3-24)		21 (7-64)		2 (1-8)	
2003	Papua New Guinea	886 (628-923)		102 (72-106)		67 (22-205)		8 (3-24)		21 (7-65)		2 (1-7)	
2004	Papua New Guinea	889 (631-927)		101 (72-106)		68 (22-206)		8 (3-24)		21 (7-65)		2 (1-7)	
2005	Papua New Guinea	891 (632-929)		100 (71-105)		67 (19-217)		8 (2-24)		60 (17-193)		7 (2-22)	
2006	Papua New Guinea	890 (631-928)		99 (71-104)		67 (19-215)		7 (2-24)		59 (17-192)		7 (2-21)	
2007	Papua New Guinea	891 (631-928)		98 (70-102)		66 (19-213)		7 (2-23)		59 (17-190)		6 (2-21)	
2008	Papua New Guinea	883 (626-920)		96 (68-100)		65 (18-208)		7 (2-23)		58 (16-185)		6 (2-20)	
2009	Papua New Guinea	878 (623-915)		94 (67-98)		64 (18-204)		7 (2-22)		57 (16-182)		6 (2-19)	
2010	Papua New Guinea	864 (613-900)		91 (65-95)		62 (18-198)		7 (2-21)		55 (16-176)		6 (2-19)	
2011	Papua New Guinea	849 (602-885)		89 (63-93)		60 (18-192)		6 (2-20)		54 (16-171)		6 (2-18)	
2012	Papua New Guinea	832 (590-867)		86 (61-90)		58 (17-185)		6 (2-19)		52 (15-165)		5 (2-17)	
2013	Papua New Guinea	817 (579-852)		84 (60-88)		57 (17-181)		6 (2-19)		51 (15-161)		5 (2-17)	
2014	Papua New Guinea	794 (563-828)		82 (58-85)		55 (16-174)		6 (2-18)		49 (15-155)		5 (1-16)	

Spn = *Streptococcus pneumoniae*; npnm = non-pneumonia, non-meningitis; HIV = deaths only in HIV-infected children; Uncertainty range provided in parentheses.

Year	Country	Spn pneumonia deaths (UR)	Spn pneumonia deaths, HIV (UR)	Spn pneumonia mortality rate (UR)	Spn pneumonia mortality rate, HIV (UR)	Spn meningitis deaths (UR)	Spn meningitis deaths, HIV (UR)	Spn meningitis mortality rate (UR)	Spn meningitis mortality rate, HIV (UR)	Spn NPNM deaths (UR)	Spn NPNM deaths, HIV (UR)	Spn NPNM mortality rate (UR)	Spn NPNM mortality rate, HIV (UR)
2015	Papua New Guinea	671 (475-699)	35 (25-37)	68 (49-71)	4 (3-4)	48 (14-152)	3 (1-8)	5 (1-16)	0 (0-1)	43 (13-135)	2 (1-7)	4 (1-14)	0 (0-1)
2000	Paraguay	204 (145-212)		29 (21-31)		21 (8-37)		3 (1-5)		19 (7-33)		3 (1-5)	
2001	Paraguay	195 (138-203)		28 (20-29)		20 (8-35)		3 (1-5)		18 (7-31)		3 (1-4)	
2002	Paraguay	182 (129-190)		27 (19-28)		19 (7-33)		3 (1-5)		17 (6-29)		2 (1-4)	
2003	Paraguay	188 (134-196)		28 (20-29)		34 (12-57)		5 (2-8)		30 (11-50)		5 (2-7)	
2004	Paraguay	182 (129-189)		27 (19-28)		35 (20-62)		5 (3-9)		31 (18-56)		5 (3-8)	
2005	Paraguay	172 (122-179)		26 (19-27)		34 (18-57)		5 (3-9)		30 (16-51)		5 (2-8)	
2006	Paraguay	165 (117-172)		25 (18-26)		33 (17-52)		5 (3-8)		29 (15-47)		4 (2-7)	
2007	Paraguay	158 (112-164)		24 (17-25)		31 (15-49)		5 (2-8)		28 (14-43)		4 (2-7)	
2008	Paraguay	153 (109-160)		23 (17-24)		31 (15-48)		5 (2-7)		27 (13-43)		4 (2-7)	
2009	Paraguay	146 (104-153)		22 (16-23)		29 (14-45)		4 (2-7)		26 (13-40)		4 (2-6)	
2010	Paraguay	141 (100-147)		21 (15-22)		28 (14-43)		4 (2-7)		25 (12-39)		4 (2-6)	
2011	Paraguay	134 (95-140)		20 (14-21)		26 (13-41)		4 (2-6)		24 (12-36)		3 (2-5)	
2012	Paraguay	97 (68-101)		14 (10-15)		19 (9-29)		3 (1-4)		17 (8-26)		3 (1-4)	
2013	Paraguay	76 (54-79)		11 (8-12)		15 (7-23)		2 (1-3)		13 (7-21)		2 (1-3)	
2014	Paraguay	66 (47-69)		10 (7-10)		13 (7-21)		2 (1-3)		12 (6-18)		2 (1-3)	
2015	Paraguay	59 (42-61)	1 (0-1)	9 (6-9)	0 (0-0)	12 (6-18)	0 (0-0)	2 (1-3)	0 (0-0)	11 (5-16)	0 (0-0)	2 (1-2)	0 (0-0)
2000	Peru	1417 (1005-1476)		48 (34-50)		234 (66-608)		8 (2-21)		208 (59-541)		7 (2-18)	
2001	Peru	1227 (870-1279)		42 (30-44)		195 (57-504)		7 (2-17)		174 (51-449)		6 (2-15)	
2002	Peru	1097 (778-1143)		38 (27-39)		178 (54-455)		6 (2-16)		159 (48-405)		5 (2-14)	
2003	Peru	972 (689-1013)		33 (24-35)		157 (49-397)		5 (2-14)		140 (44-353)		5 (2-12)	
2004	Peru	873 (619-910)		30 (21-31)		160 (38-484)		5 (1-17)		142 (34-431)		5 (1-15)	
2005	Peru	786 (557-819)		27 (19-28)		142 (33-430)		5 (1-15)		127 (29-383)		4 (1-13)	
2006	Peru	729 (517-760)		25 (18-26)		131 (30-396)		5 (1-14)		117 (27-352)		4 (1-12)	
2007	Peru	670 (475-698)		23 (16-24)		120 (29-360)		4 (1-12)		107 (26-321)		4 (1-11)	
2008	Peru	615 (436-641)		21 (15-22)		111 (28-330)		4 (1-11)		99 (25-294)		3 (1-10)	
2009	Peru	554 (393-578)		19 (14-20)		100 (26-297)		3 (1-10)		89 (23-264)		3 (1-9)	
2010	Peru	446 (316-465)		15 (11-16)		78 (19-236)		3 (1-8)		70 (17-210)		2 (1-7)	
2011	Peru	373 (265-389)		13 (9-13)		65 (14-201)		2 (0-7)		58 (12-179)		2 (0-6)	
2012	Peru	286 (203-298)		10 (7-10)		49 (10-153)		2 (0-5)		43 (9-136)		1 (0-5)	
2013	Peru	196 (139-205)		7 (5-7)		32 (7-101)		1 (0-3)		29 (6-90)		1 (0-3)	
2014	Peru	157 (112-164)		5 (4-6)		26 (5-81)		1 (0-3)		23 (5-72)		1 (0-2)	
2015	Peru	140 (99-146)	1 (0-1)	5 (3-5)	0 (0-0)	23 (5-72)	0 (0-0)	1 (0-2)	0 (0-0)	20 (4-64)	0 (0-0)	1 (0-2)	0 (0-0)
2000	Philippines	5040 (3574-5253)		48 (34-50)		613 (219-1344)		6 (2-13)		546 (195-1196)		5 (2-11)	
2001	Philippines	4978 (3530-5188)		46 (33-48)		600 (212-1319)		6 (2-12)		534 (189-1175)		5 (2-11)	
2002	Philippines	4899 (3474-5106)		45 (32-47)		585 (204-1291)		5 (2-12)		521 (182-1150)		5 (2-11)	
2003	Philippines	4838 (3431-5042)		44 (31-46)		572 (197-1267)		5 (2-12)		509 (176-1128)		5 (2-10)	
2004	Philippines	4753 (3371-4954)		42 (30-44)		555 (190-1237)		5 (2-11)		495 (169-1101)		4 (2-10)	
2005	Philippines	4528 (3211-4718)		40 (29-42)		522 (177-1168)		5 (2-10)		465 (157-1040)		4 (1-9)	
2006	Philippines	4482 (3178-4671)		40 (29-42)		511 (171-1146)		5 (2-10)		454 (152-1020)		4 (1-9)	
2007	Philippines	4341 (3078-4524)		39 (28-41)		488 (161-1098)		4 (1-10)		434 (144-978)		4 (1-9)	
2008	Philippines	4193 (2973-4370)		39 (27-40)		466 (152-1050)		4 (1-10)		415 (135-935)		4 (1-9)	
2009	Philippines	4043 (2867-4214)		38 (27-40)		445 (150-995)		4 (1-9)		396 (133-886)		4 (1-8)	
2010	Philippines	3917 (2778-4082)		37 (26-39)		429 (149-948)		4 (1-9)		382 (133-844)		4 (1-8)	
2011	Philippines	3848 (2729-4011)		36 (26-38)		422 (151-921)		4 (1-9)		376 (135-820)		4 (1-8)	
2012	Philippines	3771 (2674-3930)		35 (25-37)		415 (154-894)		4 (1-8)		370 (137-796)		3 (1-7)	
2013	Philippines	3743 (2654-3901)		35 (24-36)		415 (116-1056)		4 (1-10)		369 (103-940)		3 (1-9)	
2014	Philippines	3096 (2195-3226)		28 (20-29)		337 (99-847)		3 (1-8)		300 (88-754)		3 (1-7)	
2015	Philippines	3182 (2256-3316)	3 (2-3)	29 (20-30)	0 (0-0)	357 (111-888)	0 (0-1)	3 (1-8)	0 (0-0)	318 (99-790)	0 (0-1)	3 (1-7)	0 (0-0)
2000	Poland	47 (33-49)		2 (2-3)		10 (5-19)		1 (0-1)		9 (4-17)		0 (0-1)	
2001	Poland	39 (27-40)		2 (1-2)		9 (4-17)		1 (0-1)		8 (4-15)		0 (0-1)	
2002	Poland	29 (21-30)		2 (1-2)		7 (3-12)		0 (0-1)		6 (3-11)		0 (0-1)	
2003	Poland	34 (24-35)		2 (1-2)		7 (3-13)		0 (0-1)		7 (3-12)		0 (0-1)	
2004	Poland	35 (25-37)		2 (1-2)		6 (3-11)		0 (0-1)		5 (2-10)		0 (0-1)	
2005	Poland	36 (26-38)		2 (1-2)		6 (3-12)		0 (0-1)		6 (3-10)		0 (0-1)	
2006	Poland	39 (27-40)		2 (2-2)		10 (5-19)		1 (0-1)		9 (4-17)		1 (0-1)	
2007	Poland	43 (31-45)		2 (2-2)		8 (4-15)		0 (0-1)		7 (3-13)		0 (0-1)	
2008	Poland	40 (28-41)		2 (1-2)		10 (4-18)		1 (0-1)		9 (4-16)		0 (0-1)	
2009	Poland	45 (32-47)		2 (2-2)		8 (4-15)		0 (0-1)		7 (3-14)		0 (0-1)	
2010	Poland	38 (27-40)		2 (1-2)		8 (3-14)		0 (0-1)		7 (3-12)		0 (0-1)	
2011	Poland	30 (21-31)		1 (1-2)		7 (3-13)		0 (0-1)		6 (3-11)		0 (0-1)	
2012	Poland	30 (21-32)		1 (1-2)		6 (3-11)		0 (0-1)		6 (3-10)		0 (0-0)	
2013	Poland	40 (28-41)		2 (1-2)		6 (3-10)		0 (0-0)		5 (2-9)		0 (0-0)	
2014	Poland	31 (22-32)		2 (1-2)		6 (3-10)		0 (0-1)		5 (2-9)		0 (0-0)	
2015	Poland	31 (22-32)	0 (0-0)	2 (1-2)	0 (0-0)	6 (3-11)	0 (0-0)	0 (0-1)	0 (0-0)	5 (2-9)	0 (0-0)	0 (0-0)	0 (0-0)
2000	Portugal	10 (7-11)		2 (1-2)		12 (6-22)		2 (1-4)		11 (5-20)		2 (1-4)	
2001	Portugal	10 (7-11)		2 (1-2)		3 (2-6)		1 (0-1)		3 (1-6)		1 (0-1)	
2002	Portugal	11 (7-11)		2 (1-2)		9 (4-17)		2 (1-3)		8 (4-15)		2 (1-3)	
2003	Portugal	9 (7-10)		2 (1-2)		6 (3-11)		1 (1-2)		5 (2-10)		1 (0-2)	
2004	Portugal	6 (4-7)		1 (1-1)		3 (1-6)		1 (0-1)		3 (1-5)		1 (0-1)	
2005	Portugal	5 (4-6)		1 (1-1)		3 (1-5)		1 (0-1)		2 (1-4)		0 (0-1)	
2006	Portugal	5 (4-5)		1 (1-1)		3 (1-5)		0 (0-1)		2 (1-4)		0 (0-1)	
2007	Portugal	3 (2-3)		1 (0-1)		1 (1-2)		0 (0-0)		1 (1-2)		0 (0-0)	
2008	Portugal	4 (3-4)		1 (1-1)		1 (0-2)		0 (0-0)		1 (0-1)		0 (0-0)	
2009	Portugal	7 (5-7)		1 (1-1)		1 (0-2)		0 (0-0)		1 (0-1)		0 (0-0)	
2010	Portugal	1 (1-1)		0 (0-0)		2 (1-3)		0 (0-1)		1 (1-3)		0 (0-1)	
2011	Portugal	3 (2-3)		1 (0-1)		1 (0-2)		0 (0-0)		1 (0-2)		0 (0-0)	
2012	Portugal	1 (0-1)		0 (0-0)		0 (0-1)		0 (0-0)		0 (0-0)		0 (0-0)	
2013	Portugal	1 (1-1)		0 (0-0)		1 (0-1)		0 (0-0)		1 (0-1)		0 (0-0)	
2014	Portugal	1 (1-1)		0 (0-0)		0 (0-1)		0 (0-0)		0 (0-1)		0 (0-0)	
2015	Portugal	0 (0-1)	0 (0-0)	0 (0-0)	0 (0-0)	0 (0-1)	0 (0-0)	0 (0-0)	0 (0-0)	0 (0-0)	0 (0-0)	0 (0-0)	0 (0-0)
2000	Qatar	3 (2-3)		6 (4-6)		1 (0-1)		1 (1-2)		1 (0-1)		1 (1-2)	
2001	Qatar	3 (2-3)		5 (4-5)		1 (0-1)		1 (1-2)		1 (0-1)		1 (1-2)	
2002	Qatar	3 (2-3)		5 (3-5)		1 (0-1)		1 (1-2)		1 (0-1)		1 (1-1)	
2003	Qatar	3 (2-3)		4 (3-5)		1 (0-1)		1 (1-2)		1 (0-1)		1 (1-1)	
2004	Qatar	3 (2-3)		4 (3-4)		1 (0-1)		1 (1-1)		1 (0-1)		1 (1-1)	
2005	Qatar	3 (2-3)		4 (3-4)		1 (0-1)		1 (1-1)		1 (0-1)		1 (1-1)	
2006	Qatar	2 (2-3)		4 (3-4)		1 (0-1)		1 (1-1)		1 (0-1)		1 (0-1)	

Spn = *Streptococcus pneumoniae*; npnm = non-pneumonia, non-meningitis; HIV = deaths only in HIV-infected children; Uncertainty range provided in parentheses.

Year	Country	Spn pneumonia deaths (UR)	Spn pneumonia deaths, HIV (UR)	Spn pneumonia mortality rate (UR)	Spn pneumonia mortality rate, HIV (UR)	Spn meningitis deaths (UR)	Spn meningitis deaths, HIV (UR)	Spn meningitis mortality rate (UR)	Spn meningitis mortality rate, HIV (UR)	Spn NPNM deaths (UR)	Spn NPNM deaths, HIV (UR)	Spn NPNM mortality rate (UR)	Spn NPNM mortality rate, HIV (UR)
2007	Qatar	2 (2-3)		3 (2-4)		1 (0-1)		1 (1-1)		1 (0-1)		1 (0-1)	
2008	Qatar	3 (2-3)		3 (2-3)		1 (0-1)		1 (0-1)		1 (0-1)		1 (0-1)	
2009	Qatar	2 (2-2)		3 (2-3)		1 (0-1)		1 (0-1)		0 (0-1)		1 (0-1)	
2010	Qatar	2 (2-2)		2 (2-3)		1 (0-1)		1 (0-1)		0 (0-1)		1 (0-1)	
2011	Qatar	2 (1-2)		2 (1-2)		0 (0-1)		0 (0-1)		0 (0-1)		0 (0-1)	
2012	Qatar	2 (1-2)		2 (1-2)		0 (0-1)		0 (0-1)		0 (0-1)		0 (0-1)	
2013	Qatar	2 (1-2)		1 (1-1)		0 (0-1)		0 (0-0)		0 (0-0)		0 (0-0)	
2014	Qatar	1 (1-1)		1 (1-1)		0 (0-1)		0 (0-0)		0 (0-0)		0 (0-0)	
2015	Qatar	1 (1-2)	0 (0-0)	1 (1-1)	0 (0-0)	0 (0-1)	0 (0-0)	0 (0-0)	0 (0-0)	0 (0-0)	0 (0-0)	0 (0-0)	0 (0-0)
2000	Republic of Korea	56 (40-58)		2 (1-2)		24 (15-33)		1 (0-1)		22 (13-30)		1 (0-1)	
2001	Republic of Korea	49 (35-51)		2 (1-2)		20 (12-28)		1 (0-1)		18 (11-25)		1 (0-1)	
2002	Republic of Korea	44 (32-46)		2 (1-2)		21 (12-28)		1 (0-1)		18 (11-25)		1 (0-1)	
2003	Republic of Korea	36 (26-37)		1 (1-1)		16 (10-22)		1 (0-1)		14 (9-20)		1 (0-1)	
2004	Republic of Korea	25 (18-27)		1 (1-1)		17 (10-24)		1 (0-1)		15 (9-21)		1 (0-1)	
2005	Republic of Korea	23 (17-24)		1 (1-1)		13 (8-18)		0 (0-1)		11 (7-16)		0 (0-1)	
2006	Republic of Korea	27 (19-28)		1 (1-1)		17 (10-23)		1 (0-1)		15 (9-21)		1 (0-1)	
2007	Republic of Korea	20 (14-21)		1 (1-1)		20 (12-28)		1 (0-1)		18 (11-25)		1 (0-1)	
2008	Republic of Korea	20 (14-21)		1 (1-1)		13 (8-18)		1 (0-1)		12 (7-16)		0 (0-1)	
2009	Republic of Korea	21 (15-21)		1 (1-1)		14 (8-19)		1 (0-1)		12 (7-17)		1 (0-1)	
2010	Republic of Korea	19 (14-20)		1 (1-1)		14 (8-19)		1 (0-1)		13 (8-17)		1 (0-1)	
2011	Republic of Korea	19 (13-20)		1 (1-1)		18 (11-25)		1 (0-1)		16 (10-22)		1 (0-1)	
2012	Republic of Korea	13 (9-13)		1 (0-1)		13 (8-18)		1 (0-1)		12 (7-16)		1 (0-1)	
2013	Republic of Korea	14 (10-15)		1 (0-1)		13 (8-18)		1 (0-1)		11 (7-16)		1 (0-1)	
2014	Republic of Korea	16 (12-17)		1 (1-1)		16 (9-22)		1 (0-1)		14 (8-19)		1 (0-1)	
2015	Republic of Korea	10 (7-11)	0 (0-0)	0 (0-0)	0 (0-0)	10 (6-14)	0 (0-0)	0 (0-1)	0 (0-0)	9 (5-12)	0 (0-0)	0 (0-1)	0 (0-0)
2000	Republic of Moldova	60 (43-63)		25 (18-26)		4 (1-11)		2 (1-5)		3 (1-10)		1 (1-4)	
2001	Republic of Moldova	49 (35-51)		21 (15-22)		1 (0-6)		1 (0-3)		1 (0-5)		1 (0-2)	
2002	Republic of Moldova	36 (25-37)		16 (12-17)		2 (0-8)		1 (0-4)		2 (0-7)		1 (0-3)	
2003	Republic of Moldova	33 (23-34)		15 (11-16)		2 (0-7)		1 (0-3)		1 (0-6)		1 (0-3)	
2004	Republic of Moldova	24 (17-25)		12 (8-12)		2 (0-7)		1 (0-4)		1 (0-6)		1 (0-3)	
2005	Republic of Moldova	22 (16-23)		11 (8-12)		2 (0-10)		1 (0-5)		2 (0-9)		1 (0-4)	
2006	Republic of Moldova	24 (17-25)		12 (8-12)		1 (0-2)		0 (0-1)		0 (0-2)		0 (0-1)	
2007	Republic of Moldova	18 (13-19)		9 (6-9)		1 (0-4)		0 (0-2)		1 (0-4)		0 (0-2)	
2008	Republic of Moldova	19 (13-20)		9 (6-9)		1 (0-5)		1 (0-3)		1 (0-5)		1 (0-2)	
2009	Republic of Moldova	20 (14-20)		9 (7-10)		2 (0-8)		1 (0-4)		2 (0-7)		1 (0-3)	
2010	Republic of Moldova	20 (14-21)		9 (7-10)		2 (1-9)		1 (0-4)		2 (0-8)		1 (0-4)	
2011	Republic of Moldova	20 (14-21)		9 (6-9)		3 (1-13)		1 (0-6)		3 (1-11)		1 (0-5)	
2012	Republic of Moldova	18 (13-18)		8 (6-8)		2 (1-7)		1 (0-3)		2 (0-6)		1 (0-3)	
2013	Republic of Moldova	19 (13-20)		8 (6-9)		1 (0-4)		0 (0-2)		1 (0-3)		0 (0-1)	
2014	Republic of Moldova	17 (12-17)		8 (5-8)		2 (1-6)		1 (0-3)		2 (1-6)		1 (0-3)	
2015	Republic of Moldova	12 (9-13)	0 (0-0)	6 (4-6)	0 (0-0)	1 (1-4)	0 (0-0)	1 (0-2)	0 (0-0)	1 (0-4)	0 (0-0)	1 (0-2)	0 (0-0)
2000	Romania	587 (416-611)		53 (38-55)		42 (19-76)		4 (2-7)		37 (17-68)		3 (2-6)	
2001	Romania	547 (388-570)		48 (34-50)		42 (19-75)		4 (2-7)		37 (17-67)		3 (1-6)	
2002	Romania	486 (345-506)		42 (30-44)		43 (20-79)		4 (2-7)		39 (17-70)		3 (2-6)	
2003	Romania	392 (278-408)		35 (25-37)		28 (13-50)		2 (1-5)		25 (11-45)		2 (1-4)	
2004	Romania	383 (271-399)		36 (25-37)		20 (9-37)		2 (1-3)		18 (8-32)		2 (1-3)	
2005	Romania	318 (226-332)		30 (22-32)		18 (8-33)		2 (1-3)		16 (7-29)		2 (1-3)	
2006	Romania	280 (199-292)		27 (19-28)		16 (7-30)		2 (1-3)		15 (7-27)		1 (1-3)	
2007	Romania	263 (187-274)		26 (18-27)		16 (7-28)		2 (1-3)		14 (6-25)		1 (1-2)	
2008	Romania	257 (182-268)		25 (18-26)		10 (5-19)		1 (0-2)		9 (4-17)		1 (0-2)	
2009	Romania	226 (160-236)		22 (15-23)		9 (4-17)		1 (0-2)		8 (4-15)		1 (0-1)	
2010	Romania	222 (158-231)		21 (15-22)		11 (5-20)		1 (0-2)		10 (4-17)		1 (0-2)	
2011	Romania	239 (170-249)		23 (16-24)		14 (6-25)		1 (1-2)		12 (6-22)		1 (1-2)	
2012	Romania	214 (151-223)		21 (15-22)		7 (3-13)		1 (0-1)		6 (3-11)		1 (0-1)	
2013	Romania	201 (143-210)		21 (15-22)		10 (4-17)		1 (0-2)		8 (4-15)		1 (0-2)	
2014	Romania	183 (130-191)		20 (14-20)		9 (4-16)		1 (0-2)		8 (4-14)		1 (0-1)	
2015	Romania	169 (120-177)	0 (0-0)	19 (13-19)	0 (0-0)	8 (4-15)	0 (0-0)	1 (0-2)	0 (0-0)	7 (3-13)	0 (0-0)	1 (0-1)	0 (0-0)
2000	Russian Federation	1147 (813-1195)		18 (13-19)		193 (88-350)		3 (1-6)		172 (78-312)		3 (1-5)	
2001	Russian Federation	1090 (773-1136)		17 (12-17)		183 (83-331)		3 (1-5)		163 (74-295)		2 (1-4)	
2002	Russian Federation	997 (707-1039)		15 (11-16)		167 (76-304)		3 (1-5)		149 (68-270)		2 (1-4)	
2003	Russian Federation	876 (621-913)		13 (9-14)		148 (67-268)		2 (1-4)		132 (60-239)		2 (1-4)	
2004	Russian Federation	790 (560-824)		12 (8-12)		134 (61-244)		2 (1-4)		120 (54-217)		2 (1-3)	
2005	Russian Federation	706 (501-736)		10 (7-11)		121 (55-220)		2 (1-3)		108 (49-195)		2 (1-3)	
2006	Russian Federation	632 (448-658)		9 (6-9)		109 (49-198)		2 (1-3)		97 (44-176)		1 (1-2)	
2007	Russian Federation	562 (399-586)		8 (5-8)		97 (44-176)		1 (1-2)		87 (39-157)		1 (1-2)	
2008	Russian Federation	509 (361-530)		7 (5-7)		88 (40-160)		1 (1-2)		78 (36-142)		1 (0-2)	
2009	Russian Federation	477 (338-497)		6 (4-7)		82 (37-149)		1 (0-2)		73 (33-132)		1 (0-2)	
2010	Russian Federation	452 (320-471)		6 (4-6)		77 (35-139)		1 (0-2)		68 (31-124)		1 (0-2)	
2011	Russian Federation	425 (302-443)		5 (4-6)		72 (32-130)		1 (0-2)		64 (29-116)		1 (0-1)	
2012	Russian Federation	392 (278-408)		5 (3-5)		79 (36-144)		1 (0-2)		71 (32-128)		1 (0-2)	
2013	Russian Federation	395 (280-412)		5 (3-5)		79 (36-143)		1 (0-2)		70 (32-127)		1 (0-1)	
2014	Russian Federation	382 (271-399)		4 (3-5)		75 (34-136)		1 (0-2)		67 (30-121)		1 (0-1)	
2015	Russian Federation	373 (264-388)	3 (2-3)	4 (3-4)	0 (0-0)	72 (33-131)	1 (0-1)	1 (0-1)	0 (0-0)	64 (29-116)	1 (0-1)	1 (0-1)	0 (0-0)
2000	Rwanda	3935 (2791-4101)		300 (213-313)		819 (409-1635)		62 (31-125)		258 (128-514)		20 (10-39)	
2001	Rwanda	4037 (2863-4207)		284 (201-296)		833 (420-1659)		59 (30-117)		262 (132-522)		18 (9-37)	
2002	Rwanda	3567 (2530-3718)		242 (172-252)		729 (372-1448)		49 (25-98)		229 (117-455)		16 (8-31)	
2003	Rwanda	3222 (2285-3358)		219 (156-229)		653 (220-1488)		44 (15-101)		205 (69-468)		14 (5-32)	
2004	Rwanda	2763 (1959-2879)		190 (135-198)		554 (190-1260)		38 (13-87)		174 (60-396)		12 (4-27)	
2005	Rwanda	2407 (1707-2508)		165 (117-172)		478 (167-1083)		33 (11-74)		150 (52-340)		10 (4-23)	
2006	Rwanda	2121 (1504-2210)		143 (102-149)		416 (145-942)		28 (10-64)		131 (46-296)		9 (3-20)	
2007	Rwanda	1895 (1344-1975)		125 (89-130)		367 (128-830)		24 (8-55)		115 (40-261)		8 (3-17)	
2008	Rwanda	1709 (1212-1781)		109 (77-113)		327 (113-737)		21 (7-47)		103 (36-232)		7 (2-15)	
2009	Rwanda	1548 (1098-1613)		96 (68-100)		294 (93-666)		18 (6-41)		262 (83-593)		16 (5-37)	
2010	Rwanda	1204 (854-1255)		73 (52-76)		223 (79-491)		13 (5-30)		198 (70-437)		12 (4-26)	
2011	Rwanda	880 (624-917)		52 (37-54)		158 (56-345)		9 (3-20)		141 (50-307)		8 (3-18)	
2012	Rwanda	590 (418-615)		35 (25-36)		104 (37-225)		6 (2-13)		92 (33-200)		5 (2-12)	
2013	Rwanda	457 (324-476)		27 (19-28)		82 (30-177)		5 (2-10)		73 (26-157)		4 (2-9)	
2014	Rwanda	304 (215-317)		18 (13-19)		54 (20-116)		3 (1-7)		48 (18-103)		3 (1-6)	

Spn = *Streptococcus pneumoniae*; npnm = non-pneumonia, non-meningitis; HIV = deaths only in HIV-infected children; Uncertainty range provided in parentheses.

Year	Country	Spn pneumonia deaths (UR)	Spn pneumonia deaths, HIV (UR)	Spn pneumonia mortality rate (UR)	Spn pneumonia mortality rate, HIV (UR)	Spn meningitis deaths (UR)	Spn meningitis deaths, HIV (UR)	Spn meningitis mortality rate (UR)	Spn meningitis mortality rate, HIV (UR)	Spn NPNM deaths (UR)	Spn NPNM deaths, HIV (UR)	Spn NPNM mortality rate (UR)	Spn NPNM mortality rate, HIV (UR)
2015	Rwanda	244 (173-254)	4 (3-4)	15 (10-15)	0 (0-0)	44 (16-94)	1 (0-1)	3 (1-6)	0 (0-0)	39 (14-83)	1 (0-1)	2 (1-5)	0 (0-0)
2000	Saint Kitts and Nevis	0 (0-0)		4 (2-4)		0 (0-0)		0 (0-0)		0 (0-0)		0 (0-0)	
2001	Saint Kitts and Nevis	0 (0-0)		6 (4-6)		0 (0-0)		0 (0-0)		0 (0-0)		0 (0-0)	
2002	Saint Kitts and Nevis	0 (0-0)		6 (5-7)		0 (0-0)		0 (0-0)		0 (0-0)		0 (0-0)	
2003	Saint Kitts and Nevis	0 (0-0)		6 (4-6)		0 (0-0)		0 (0-0)		0 (0-0)		0 (0-0)	
2004	Saint Kitts and Nevis	0 (0-0)		4 (3-4)		0 (0-0)		0 (0-0)		0 (0-0)		0 (0-0)	
2005	Saint Kitts and Nevis	0 (0-0)		2 (2-2)		0 (0-0)		0 (0-0)		0 (0-0)		0 (0-0)	
2006	Saint Kitts and Nevis	0 (0-0)		2 (1-2)		0 (0-0)		0 (0-0)		0 (0-0)		0 (0-0)	
2007	Saint Kitts and Nevis	0 (0-0)		2 (1-2)		0 (0-0)		0 (0-0)		0 (0-0)		0 (0-0)	
2008	Saint Kitts and Nevis	0 (0-0)		0 (0-0)		0 (0-0)		0 (0-0)		0 (0-0)		0 (0-0)	
2009	Saint Kitts and Nevis	0 (0-0)		0 (0-0)		0 (0-0)		0 (0-0)		0 (0-0)		0 (0-0)	
2010	Saint Kitts and Nevis	0 (0-0)		0 (0-0)		0 (0-0)		0 (0-0)		0 (0-0)		0 (0-0)	
2011	Saint Kitts and Nevis	0 (0-0)		0 (0-0)		0 (0-0)		5 (2-7)		0 (0-0)		4 (2-6)	
2012	Saint Kitts and Nevis	0 (0-0)		0 (0-0)		0 (0-0)		5 (2-8)		0 (0-0)		5 (2-7)	
2013	Saint Kitts and Nevis	0 (0-0)		0 (0-0)		0 (0-1)		7 (3-11)		0 (0-0)		6 (3-10)	
2014	Saint Kitts and Nevis	0 (0-0)		0 (0-0)		0 (0-0)		4 (2-7)		0 (0-0)		4 (2-6)	
2015	Saint Kitts and Nevis	0 (0-0)	0 (0-0)	0 (0-0)	0 (0-0)	0 (0-0)	0 (0-0)	4 (2-7)	0 (0-0)	0 (0-0)	0 (0-0)	4 (2-6)	0 (0-0)
2000	Saint Lucia	1 (1-1)		5 (3-5)		0 (0-0)		1 (1-2)		0 (0-0)		1 (1-2)	
2001	Saint Lucia	1 (1-1)		6 (4-6)		0 (0-0)		1 (0-1)		0 (0-0)		1 (0-1)	
2002	Saint Lucia	1 (1-1)		6 (4-6)		0 (0-0)		1 (1-2)		0 (0-0)		1 (1-2)	
2003	Saint Lucia	1 (1-1)		6 (4-6)		0 (0-0)		1 (1-2)		0 (0-0)		1 (1-2)	
2004	Saint Lucia	1 (1-1)		7 (5-8)		0 (0-0)		1 (1-2)		0 (0-0)		1 (1-2)	
2005	Saint Lucia	1 (1-1)		9 (7-10)		0 (0-0)		0 (0-0)		0 (0-0)		0 (0-0)	
2006	Saint Lucia	2 (1-2)		12 (9-13)		0 (0-0)		1 (0-1)		0 (0-0)		0 (0-1)	
2007	Saint Lucia	1 (1-2)		11 (8-11)		0 (0-0)		1 (0-1)		0 (0-0)		1 (0-1)	
2008	Saint Lucia	1 (1-1)		8 (5-8)		0 (0-0)		1 (0-1)		0 (0-0)		0 (0-1)	
2009	Saint Lucia	1 (1-1)		9 (6-9)		0 (0-1)		2 (1-4)		0 (0-0)		2 (1-3)	
2010	Saint Lucia	1 (1-1)		8 (5-8)		0 (0-0)		2 (1-3)		0 (0-0)		2 (1-3)	
2011	Saint Lucia	1 (1-1)		5 (4-6)		0 (0-0)		2 (1-4)		0 (0-0)		2 (1-3)	
2012	Saint Lucia	0 (0-0)		3 (2-3)		0 (0-0)		1 (0-1)		0 (0-0)		1 (0-1)	
2013	Saint Lucia	0 (0-0)		3 (2-3)		0 (0-0)		1 (1-2)		0 (0-0)		1 (1-2)	
2014	Saint Lucia	1 (0-1)		5 (4-5)		0 (0-0)		2 (1-3)		0 (0-0)		2 (1-3)	
2015	Saint Lucia	1 (0-1)	0 (0-0)	5 (3-5)	0 (0-0)	0 (0-0)	0 (0-0)	2 (1-3)	0 (0-0)	0 (0-0)	0 (0-0)	2 (1-3)	0 (0-0)
2000	Saint Vincent and the Grenadines	1 (0-1)		5 (4-5)		0 (0-0)		2 (1-3)		0 (0-0)		2 (1-3)	
2001	Saint Vincent and the Grenadines	1 (0-1)		5 (4-5)		0 (0-0)		1 (1-2)		0 (0-0)		1 (1-2)	
2002	Saint Vincent and the Grenadines	0 (0-0)		3 (2-3)		0 (0-0)		0 (0-0)		0 (0-0)		0 (0-0)	
2003	Saint Vincent and the Grenadines	0 (0-0)		2 (1-2)		0 (0-0)		0 (0-0)		0 (0-0)		0 (0-0)	
2004	Saint Vincent and the Grenadines	0 (0-0)		4 (3-4)		0 (0-0)		0 (0-0)		0 (0-0)		0 (0-0)	
2005	Saint Vincent and the Grenadines	0 (0-0)		4 (3-4)		0 (0-0)		0 (0-0)		0 (0-0)		0 (0-0)	
2006	Saint Vincent and the Grenadines	0 (0-0)		5 (4-5)		0 (0-0)		0 (0-0)		0 (0-0)		0 (0-0)	
2007	Saint Vincent and the Grenadines	0 (0-0)		1 (1-1)		0 (0-0)		0 (0-0)		0 (0-0)		0 (0-0)	
2008	Saint Vincent and the Grenadines	0 (0-0)		1 (1-1)		0 (0-0)		0 (0-0)		0 (0-0)		0 (0-0)	
2009	Saint Vincent and the Grenadines	0 (0-0)		5 (3-5)		0 (0-0)		0 (0-0)		0 (0-0)		0 (0-0)	
2010	Saint Vincent and the Grenadines	0 (0-0)		5 (3-5)		0 (0-0)		0 (0-0)		0 (0-0)		0 (0-0)	
2011	Saint Vincent and the Grenadines	1 (1-1)		9 (7-10)		0 (0-0)		0 (0-0)		0 (0-0)		0 (0-0)	
2012	Saint Vincent and the Grenadines	1 (1-1)		8 (6-8)		0 (0-0)		0 (0-0)		0 (0-0)		0 (0-0)	
2013	Saint Vincent and the Grenadines	1 (1-1)		10 (7-10)		0 (0-0)		0 (0-0)		0 (0-0)		0 (0-0)	
2014	Saint Vincent and the Grenadines	1 (0-1)		8 (6-8)		0 (0-0)		0 (0-0)		0 (0-0)		0 (0-0)	
2015	Saint Vincent and the Grenadines	1 (0-1)	0 (0-0)	7 (5-7)	0 (0-0)	0 (0-0)	0 (0-0)	0 (0-0)	0 (0-0)	0 (0-0)	0 (0-0)	0 (0-0)	0 (0-0)
2000	Samoa	4 (3-4)		15 (11-16)		0 (0-1)		2 (1-3)		0 (0-1)		2 (1-3)	
2001	Samoa	4 (3-4)		14 (10-15)		0 (0-1)		2 (1-3)		0 (0-1)		2 (1-3)	
2002	Samoa	3 (2-3)		13 (9-14)		0 (0-1)		2 (1-3)		0 (0-1)		1 (1-3)	
2003	Samoa	3 (2-3)		12 (9-13)		0 (0-1)		2 (1-3)		0 (0-1)		1 (1-2)	
2004	Samoa	3 (2-3)		11 (8-12)		0 (0-1)		1 (1-2)		0 (0-1)		1 (1-2)	
2005	Samoa	3 (2-3)		11 (8-11)		0 (0-1)		1 (1-2)		0 (0-0)		1 (1-2)	
2006	Samoa	2 (2-3)		10 (7-10)		0 (0-1)		1 (1-2)		0 (0-0)		1 (0-2)	
2007	Samoa	2 (2-3)		10 (7-10)		0 (0-1)		1 (1-2)		0 (0-0)		1 (0-2)	
2008	Samoa	2 (2-2)		9 (6-10)		0 (0-1)		1 (1-2)		0 (0-1)		1 (1-2)	
2009	Samoa	3 (2-3)		10 (7-11)		0 (0-1)		2 (1-3)		0 (0-1)		2 (1-3)	
2010	Samoa	3 (2-3)		12 (8-12)		1 (0-1)		2 (1-4)		1 (0-1)		2 (1-4)	
2011	Samoa	3 (2-3)		11 (8-12)		1 (0-1)		2 (1-4)		1 (0-1)		2 (1-4)	
2012	Samoa	3 (2-3)		11 (8-12)		1 (0-1)		2 (1-4)		1 (0-1)		2 (1-4)	
2013	Samoa	3 (2-3)		11 (8-11)		1 (0-1)		2 (1-4)		0 (0-1)		2 (1-4)	
2014	Samoa	3 (2-3)		11 (8-11)		1 (0-1)		2 (1-4)		0 (0-1)		2 (1-3)	
2015	Samoa	2 (2-2)	0 (0-0)	10 (7-10)	0 (0-0)	0 (0-1)	0 (0-0)	2 (1-4)	0 (0-0)	0 (0-1)	0 (0-0)	2 (1-3)	0 (0-0)
2000	San Marino	0 (0-0)		2 (2-2)		0 (0-0)		1 (1-2)		0 (0-0)		1 (0-2)	
2001	San Marino	0 (0-0)		2 (2-2)		0 (0-0)		1 (0-2)		0 (0-0)		1 (0-2)	
2002	San Marino	0 (0-0)		2 (1-2)		0 (0-0)		1 (0-2)		0 (0-0)		1 (0-1)	
2003	San Marino	0 (0-0)		2 (1-2)		0 (0-0)		1 (0-1)		0 (0-0)		1 (0-1)	
2004	San Marino	0 (0-0)		1 (1-1)		0 (0-0)		1 (0-1)		0 (0-0)		1 (0-1)	
2005	San Marino	0 (0-0)		1 (1-1)		0 (0-0)		1 (0-1)		0 (0-0)		0 (0-1)	
2006	San Marino	0 (0-0)		1 (1-1)		0 (0-0)		1 (0-1)		0 (0-0)		0 (0-1)	
2007	San Marino	0 (0-0)		1 (1-1)		0 (0-0)		1 (0-1)		0 (0-0)		0 (0-1)	
2008	San Marino	0 (0-0)		1 (1-1)		0 (0-0)		1 (0-1)		0 (0-0)		0 (0-1)	
2009	San Marino	0 (0-0)		1 (1-1)		0 (0-0)		1 (0-1)		0 (0-0)		0 (0-1)	
2010	San Marino	0 (0-0)		1 (1-1)		0 (0-0)		1 (0-1)		0 (0-0)		0 (0-1)	
2011	San Marino	0 (0-0)		1 (1-1)		0 (0-0)		1 (0-1)		0 (0-0)		0 (0-1)	
2012	San Marino	0 (0-0)		1 (1-1)		0 (0-0)		1 (0-1)		0 (0-0)		0 (0-1)	
2013	San Marino	0 (0-0)		1 (1-1)		0 (0-0)		1 (0-1)		0 (0-0)		0 (0-1)	
2014	San Marino	0 (0-0)		1 (1-1)		0 (0-0)		1 (0-1)		0 (0-0)		0 (0-1)	
2015	San Marino	0 (0-0)	0 (0-0)	1 (1-1)	0 (0-0)	0 (0-0)	0 (0-0)	1 (0-1)	0 (0-0)	0 (0-0)	0 (0-0)	0 (0-1)	0 (0-0)
2000	Sao Tome and Principe	28 (20-29)		127 (90-132)		4 (2-9)		20 (8-43)		1 (1-3)		6 (3-13)	
2001	Sao Tome and Principe	28 (20-29)		120 (85-125)		4 (2-9)		19 (8-40)		1 (1-3)		6 (2-13)	
2002	Sao Tome and Principe	27 (19-28)		114 (81-119)		4 (2-9)		18 (8-38)		1 (1-3)		6 (2-12)	
2003	Sao Tome and Principe	26 (18-27)		107 (76-112)		4 (2-9)		17 (8-35)		1 (1-3)		5 (2-11)	
2004	Sao Tome and Principe	25 (18-26)		100 (71-105)		4 (2-8)		16 (6-34)		4 (1-7)		14 (6-30)	
2005	Sao Tome and Principe	24 (17-25)		95 (67-98)		4 (2-8)		15 (6-31)		3 (1-7)		14 (6-28)	
2006	Sao Tome and Principe	23 (16-24)		89 (63-93)		4 (2-7)		14 (6-29)		3 (1-7)		13 (5-26)	

Spn = *Streptococcus pneumoniae*; npnm = non-pneumonia, non-meningitis; HIV = deaths only in HIV-infected children; Uncertainty range provided in parentheses.

Year	Country	Spn pneumonia deaths (UR)	Spn pneumonia deaths, HIV (UR)	Spn pneumonia mortality rate (UR)	Spn pneumonia mortality rate, HIV (UR)	Spn meningitis deaths (UR)	Spn meningitis deaths, HIV (UR)	Spn meningitis mortality rate (UR)	Spn meningitis mortality rate, HIV (UR)	Spn NPNM deaths (UR)	Spn NPNM deaths, HIV (UR)	Spn NPNM mortality rate (UR)	Spn NPNM mortality rate, HIV (UR)
2007	Sao Tome and Principe	22 (16-23)		84 (59-87)		4 (2-7)		14 (6-27)		3 (1-6)		12 (5-24)	
2008	Sao Tome and Principe	22 (16-23)		83 (59-86)		4 (2-7)		13 (6-26)		3 (1-6)		12 (6-23)	
2009	Sao Tome and Principe	20 (14-21)		76 (54-79)		3 (2-6)		12 (6-24)		3 (1-6)		11 (5-21)	
2010	Sao Tome and Principe	20 (14-21)		74 (52-77)		3 (2-6)		12 (6-23)		3 (1-6)		11 (5-20)	
2011	Sao Tome and Principe	20 (14-20)		71 (51-74)		3 (1-6)		12 (5-22)		3 (1-5)		10 (5-19)	
2012	Sao Tome and Principe	19 (14-20)		69 (49-72)		3 (1-6)		11 (5-21)		3 (1-5)		10 (5-19)	
2013	Sao Tome and Principe	12 (9-13)		43 (31-45)		2 (1-3)		6 (3-12)		2 (1-3)		6 (3-11)	
2014	Sao Tome and Principe	10 (7-10)		35 (25-36)		1 (1-3)		5 (2-10)		1 (1-2)		5 (2-9)	
2015	Sao Tome and Principe	8 (6-9)	0 (0-0)	29 (21-30)	0 (0-0)	1 (1-2)	0 (0-0)	5 (2-9)	0 (0-0)	1 (1-2)	0 (0-0)	4 (2-8)	0 (0-0)
2000	Saudi Arabia	407 (289-424)		15 (10-15)		52 (31-72)		2 (1-3)		46 (28-64)		2 (1-2)	
2001	Saudi Arabia	375 (266-390)		14 (10-14)		48 (29-67)		2 (1-2)		43 (26-59)		2 (1-2)	
2002	Saudi Arabia	380 (269-396)		14 (10-14)		72 (43-99)		3 (2-4)		64 (39-88)		2 (1-3)	
2003	Saudi Arabia	365 (259-380)		13 (9-14)		70 (42-97)		3 (2-4)		62 (37-86)		2 (1-3)	
2004	Saudi Arabia	341 (242-355)		13 (9-13)		66 (40-91)		2 (1-3)		59 (35-81)		2 (1-3)	
2005	Saudi Arabia	325 (231-339)		12 (8-12)		63 (38-87)		2 (1-3)		56 (34-78)		2 (1-3)	
2006	Saudi Arabia	309 (219-322)		11 (8-12)		60 (36-83)		2 (1-3)		54 (32-74)		2 (1-3)	
2007	Saudi Arabia	303 (215-316)		11 (8-11)		60 (36-83)		2 (1-3)		54 (32-74)		2 (1-3)	
2008	Saudi Arabia	287 (204-299)		10 (7-11)		58 (35-80)		2 (1-3)		52 (31-71)		2 (1-3)	
2009	Saudi Arabia	236 (168-246)		8 (6-9)		49 (29-68)		2 (1-2)		44 (26-60)		2 (1-2)	
2010	Saudi Arabia	182 (129-189)		6 (4-6)		39 (24-54)		1 (1-2)		35 (21-48)		1 (1-2)	
2011	Saudi Arabia	135 (96-141)		5 (3-5)		31 (18-42)		1 (1-1)		27 (16-38)		1 (1-1)	
2012	Saudi Arabia	119 (85-125)		4 (3-4)		28 (17-39)		1 (1-1)		25 (15-35)		1 (0-1)	
2013	Saudi Arabia	89 (63-92)		3 (2-3)		21 (13-29)		1 (0-1)		19 (11-26)		1 (0-1)	
2014	Saudi Arabia	78 (56-82)		3 (2-3)		19 (11-26)		1 (0-1)		17 (10-23)		1 (0-1)	
2015	Saudi Arabia	73 (52-76)	0 (0-0)	2 (2-2)	0 (0-0)	18 (11-24)	0 (0-0)	1 (0-1)	0 (0-0)	16 (9-22)	0 (0-0)	1 (0-1)	0 (0-0)
2000	Senegal	2951 (2093-3076)		178 (126-186)		471 (162-1064)		28 (10-64)		148 (51-334)		9 (3-20)	
2001	Senegal	2988 (2119-3114)		176 (125-184)		476 (169-1065)		28 (10-63)		150 (53-335)		9 (3-20)	
2002	Senegal	2904 (2059-3026)		167 (118-174)		463 (169-1025)		27 (10-59)		145 (53-322)		8 (3-19)	
2003	Senegal	2799 (1985-2917)		157 (111-164)		447 (169-978)		25 (9-55)		140 (53-307)		8 (3-17)	
2004	Senegal	2681 (1901-2794)		147 (104-153)		429 (167-927)		23 (9-51)		135 (53-291)		7 (3-16)	
2005	Senegal	2548 (1807-2655)		136 (96-141)		410 (165-874)		22 (9-47)		129 (52-275)		7 (3-15)	
2006	Senegal	2419 (1716-2521)		125 (89-130)		390 (158-829)		20 (8-43)		123 (50-261)		6 (3-13)	
2007	Senegal	2276 (1614-2372)		114 (81-119)		367 (149-778)		18 (8-39)		116 (47-245)		6 (2-12)	
2008	Senegal	2139 (1517-2229)		105 (74-109)		344 (141-729)		17 (7-36)		108 (44-229)		5 (2-11)	
2009	Senegal	2009 (1424-2093)		96 (68-100)		323 (112-704)		15 (5-34)		287 (100-626)		14 (5-30)	
2010	Senegal	1898 (1346-1978)		88 (62-92)		305 (107-664)		14 (5-31)		271 (95-591)		13 (4-27)	
2011	Senegal	1798 (1275-1874)		81 (57-84)		289 (101-630)		13 (5-28)		257 (90-561)		12 (4-25)	
2012	Senegal	1726 (1224-1799)		75 (53-78)		277 (101-601)		12 (4-26)		246 (90-535)		11 (4-23)	
2013	Senegal	1684 (1194-1755)		70 (50-73)		269 (98-586)		11 (4-24)		240 (87-521)		10 (4-22)	
2014	Senegal	1144 (811-1192)		46 (33-48)		172 (55-383)		7 (2-15)		153 (49-341)		6 (2-14)	
2015	Senegal	909 (645-947)	12 (9-13)	36 (25-37)	0 (0-1)	134 (44-300)	2 (1-4)	5 (2-12)	0 (0-0)	120 (39-267)	2 (1-4)	5 (2-10)	0 (0-0)
2000	Serbia	25 (18-26)		4 (3-4)		5 (2-11)		1 (0-2)		4 (2-10)		1 (0-2)	
2001	Serbia	17 (12-18)		3 (2-3)		4 (2-9)		1 (0-2)		4 (2-8)		1 (0-1)	
2002	Serbia	14 (10-14)		2 (2-2)		2 (1-5)		0 (0-1)		2 (1-5)		0 (0-1)	
2003	Serbia	20 (14-21)		4 (3-4)		3 (2-8)		1 (0-1)		3 (1-8)		1 (0-1)	
2004	Serbia	19 (13-20)		3 (2-4)		3 (1-7)		1 (0-1)		2 (1-6)		0 (0-1)	
2005	Serbia	14 (10-15)		3 (2-3)		4 (2-10)		1 (0-2)		3 (2-9)		1 (0-2)	
2006	Serbia	9 (7-10)		2 (1-2)		2 (1-6)		0 (0-1)		2 (1-5)		0 (0-1)	
2007	Serbia	17 (12-17)		3 (2-4)		3 (1-9)		1 (0-2)		3 (1-8)		1 (0-2)	
2008	Serbia	15 (11-16)		3 (2-3)		1 (0-3)		0 (0-1)		1 (0-2)		0 (0-0)	
2009	Serbia	13 (9-14)		3 (2-3)		2 (1-7)		1 (0-2)		2 (1-6)		0 (0-1)	
2010	Serbia	10 (7-11)		2 (2-2)		1 (0-2)		0 (0-0)		1 (0-2)		0 (0-0)	
2011	Serbia	12 (9-13)		3 (2-3)		2 (1-6)		0 (0-1)		2 (1-5)		0 (0-1)	
2012	Serbia	10 (7-11)		2 (2-2)		0 (0-1)		0 (0-0)		0 (0-1)		0 (0-0)	
2013	Serbia	7 (5-7)		2 (1-2)		1 (0-3)		0 (0-1)		1 (0-3)		0 (0-1)	
2014	Serbia	10 (7-10)		2 (2-2)		1 (0-3)		0 (0-1)		1 (0-3)		0 (0-1)	
2015	Serbia	10 (7-10)	0 (0-0)	2 (2-2)	0 (0-0)	1 (0-3)	0 (0-0)	0 (0-1)	0 (0-0)	1 (0-3)	0 (0-0)	0 (0-1)	0 (0-0)
2000	Seychelles	0 (0-0)		5 (4-5)		0 (0-0)		1 (1-2)		0 (0-0)		1 (1-2)	
2001	Seychelles	0 (0-0)		6 (4-6)		0 (0-0)		1 (1-2)		0 (0-0)		1 (1-2)	
2002	Seychelles	0 (0-0)		6 (4-6)		0 (0-0)		1 (1-2)		0 (0-0)		1 (1-2)	
2003	Seychelles	0 (0-0)		5 (4-5)		0 (0-0)		1 (1-2)		0 (0-0)		1 (1-2)	
2004	Seychelles	0 (0-0)		5 (4-5)		0 (0-0)		1 (1-2)		0 (0-0)		1 (1-2)	
2005	Seychelles	0 (0-0)		5 (4-6)		0 (0-0)		1 (1-2)		0 (0-0)		1 (1-2)	
2006	Seychelles	0 (0-0)		5 (4-5)		0 (0-0)		1 (1-2)		0 (0-0)		1 (1-2)	
2007	Seychelles	0 (0-0)		5 (4-5)		0 (0-0)		1 (1-2)		0 (0-0)		1 (1-1)	
2008	Seychelles	0 (0-0)		5 (4-5)		0 (0-0)		1 (1-2)		0 (0-0)		1 (1-1)	
2009	Seychelles	0 (0-0)		5 (4-5)		0 (0-0)		1 (1-2)		0 (0-0)		1 (1-1)	
2010	Seychelles	0 (0-0)		5 (4-6)		0 (0-0)		2 (1-2)		0 (0-0)		2 (1-2)	
2011	Seychelles	0 (0-0)		6 (4-6)		0 (0-0)		2 (1-3)		0 (0-0)		2 (1-2)	
2012	Seychelles	0 (0-0)		5 (4-5)		0 (0-0)		2 (1-2)		0 (0-0)		1 (1-2)	
2013	Seychelles	0 (0-0)		6 (4-6)		0 (0-0)		2 (1-2)		0 (0-0)		2 (1-2)	
2014	Seychelles	0 (0-0)		6 (4-6)		0 (0-0)		2 (1-2)		0 (0-0)		2 (1-2)	
2015	Seychelles	0 (0-0)	0 (0-0)	6 (4-6)	0 (0-0)	0 (0-0)	0 (0-0)	2 (1-2)	0 (0-0)	0 (0-0)	0 (0-0)	2 (1-2)	0 (0-0)
2000	Sierra Leone	1807 (1281-1883)		259 (184-270)		373 (211-668)		54 (30-96)		117 (66-210)		17 (10-30)	
2001	Sierra Leone	1841 (1305-1919)		253 (179-264)		379 (214-680)		52 (29-93)		119 (67-214)		16 (9-29)	
2002	Sierra Leone	1895 (1344-1975)		249 (176-259)		386 (218-694)		51 (29-91)		121 (68-218)		16 (9-29)	
2003	Sierra Leone	1962 (1391-2045)		246 (174-256)		397 (223-714)		50 (28-90)		125 (70-224)		16 (9-28)	
2004	Sierra Leone	2036 (1444-2122)		245 (173-255)		407 (229-734)		49 (27-88)		128 (72-231)		15 (9-28)	
2005	Sierra Leone	2080 (1475-2167)		242 (171-252)		409 (230-740)		48 (27-86)		129 (72-233)		15 (8-27)	
2006	Sierra Leone	2108 (1495-2197)		237 (168-247)		409 (228-739)		46 (26-83)		129 (72-232)		14 (8-26)	
2007	Sierra Leone	2061 (1462-2148)		226 (160-236)		397 (219-713)		44 (24-78)		125 (69-224)		14 (8-25)	
2008	Sierra Leone	1960 (1390-2043)		211 (150-220)		378 (206-673)		41 (22-73)		119 (65-212)		13 (7-23)	
2009	Sierra Leone	1839 (1304-1917)		196 (139-204)		359 (212-617)		38 (23-66)		113 (67-194)		12 (7-21)	
2010	Sierra Leone	1725 (1223-1798)		182 (129-189)		340 (220-565)		36 (23-60)		107 (69-178)		11 (7-19)	
2011	Sierra Leone	1201 (851-1251)		125 (88-130)		224 (143-370)		23 (15-38)		70 (45-116)		7 (5-12)	
2012	Sierra Leone	937 (664-977)		96 (68-100)		169 (83-310)		17 (9-32)		53 (26-97)		5 (3-10)	
2013	Sierra Leone	807 (573-842)		83 (59-86)		148 (73-274)		15 (7-28)		47 (23-86)		5 (2-9)	
2014	Sierra Leone	796 (565-830)		81 (58-85)		147 (72-274)		15 (7-28)		46 (23-86)		5 (2-9)	

Spn = *Streptococcus pneumoniae*; npnm = non-pneumonia, non-meningitis; HIV = deaths only in HIV-infected children; Uncertainty range provided in parentheses.

Year	Country	Spn pneumonia deaths (UR)	Spn pneumonia deaths, HIV (UR)	Spn pneumonia mortality rate (UR)	Spn pneumonia mortality rate, HIV (UR)	Spn meningitis deaths (UR)	Spn meningitis deaths, HIV (UR)	Spn meningitis mortality rate (UR)	Spn meningitis mortality rate, HIV (UR)	Spn NPNM deaths (UR)	Spn NPNM deaths, HIV (UR)	Spn NPNM mortality rate (UR)	Spn NPNM mortality rate, HIV (UR)
2015	Sierra Leone	554 (393-578)	22 (16-23)	56 (40-59)	2 (2-2)	97 (47-182)	4 (2-7)	10 (5-18)	0 (0-1)	30 (15-57)	1 (1-2)	3 (2-6)	0 (0-0)
2000	Singapore	5 (4-5)		2 (1-2)		3 (2-4)		1 (1-2)		3 (2-4)		1 (1-1)	
2001	Singapore	6 (4-7)		3 (2-3)		1 (1-2)		0 (0-1)		1 (1-1)		0 (0-1)	
2002	Singapore	6 (5-7)		3 (2-3)		2 (1-2)		1 (0-1)		2 (1-2)		1 (0-1)	
2003	Singapore	9 (6-10)		4 (3-4)		0 (0-0)		0 (0-0)		0 (0-0)		0 (0-0)	
2004	Singapore	7 (5-8)		3 (2-3)		1 (0-1)		0 (0-0)		1 (0-1)		0 (0-0)	
2005	Singapore	8 (6-8)		3 (2-3)		1 (0-1)		0 (0-0)		1 (0-1)		0 (0-0)	
2006	Singapore	8 (5-8)		3 (2-3)		0 (0-0)		0 (0-0)		0 (0-0)		0 (0-0)	
2007	Singapore	9 (7-10)		4 (3-4)		1 (0-1)		0 (0-0)		1 (0-1)		0 (0-0)	
2008	Singapore	5 (4-5)		2 (1-2)		1 (0-1)		0 (0-0)		1 (0-1)		0 (0-0)	
2009	Singapore	6 (4-6)		2 (2-2)		1 (1-2)		1 (0-1)		1 (1-2)		0 (0-1)	
2010	Singapore	4 (3-4)		1 (1-1)		0 (0-0)		0 (0-0)		0 (0-0)		0 (0-0)	
2011	Singapore	5 (3-5)		2 (1-2)		1 (1-1)		0 (0-1)		1 (1-1)		0 (0-1)	
2012	Singapore	8 (6-8)		3 (2-3)		0 (0-0)		0 (0-0)		0 (0-0)		0 (0-0)	
2013	Singapore	5 (4-5)		2 (1-2)		0 (0-0)		0 (0-0)		0 (0-0)		0 (0-0)	
2014	Singapore	5 (4-6)		2 (1-2)		0 (0-0)		0 (0-0)		0 (0-0)		0 (0-0)	
2015	Singapore	5 (4-5)	0 (0-0)	2 (1-2)	0 (0-0)	0 (0-0)	0 (0-0)	0 (0-0)	0 (0-0)	0 (0-0)	0 (0-0)	0 (0-0)	0 (0-0)
2000	Slovakia	22 (16-23)		8 (6-8)		3 (1-6)		1 (1-2)		3 (1-5)		1 (0-2)	
2001	Slovakia	19 (13-20)		7 (5-7)		5 (2-9)		2 (1-4)		5 (2-8)		2 (1-3)	
2002	Slovakia	19 (13-20)		7 (5-8)		2 (1-4)		1 (0-1)		2 (1-3)		1 (0-1)	
2003	Slovakia	17 (12-17)		7 (5-7)		3 (1-6)		1 (1-2)		3 (1-5)		1 (1-2)	
2004	Slovakia	18 (13-18)		7 (5-7)		2 (1-3)		1 (0-1)		2 (1-3)		1 (0-1)	
2005	Slovakia	19 (14-20)		8 (5-8)		2 (1-4)		1 (0-2)		2 (1-4)		1 (0-1)	
2006	Slovakia	16 (11-16)		6 (4-6)		2 (1-4)		1 (0-1)		2 (1-3)		1 (0-1)	
2007	Slovakia	21 (15-21)		8 (6-8)		1 (1-2)		0 (0-1)		1 (1-2)		0 (0-1)	
2008	Slovakia	21 (15-22)		8 (5-8)		4 (2-7)		1 (1-3)		3 (2-6)		1 (1-2)	
2009	Slovakia	11 (8-12)		4 (3-4)		2 (1-3)		1 (0-1)		2 (1-3)		1 (0-1)	
2010	Slovakia	8 (5-8)		3 (2-3)		1 (0-2)		0 (0-1)		1 (0-2)		0 (0-1)	
2011	Slovakia	7 (5-7)		2 (2-2)		1 (0-2)		0 (0-1)		1 (0-1)		0 (0-0)	
2012	Slovakia	6 (4-6)		2 (1-2)		1 (0-2)		0 (0-1)		1 (0-2)		0 (0-1)	
2013	Slovakia	6 (4-6)		2 (1-2)		0 (0-0)		0 (0-0)		0 (0-0)		0 (0-0)	
2014	Slovakia	5 (4-5)		2 (1-2)		1 (0-1)		0 (0-0)		1 (0-1)		0 (0-0)	
2015	Slovakia	6 (4-6)	0 (0-0)	2 (1-2)	0 (0-0)	1 (0-1)	0 (0-0)	0 (0-0)	0 (0-0)	1 (0-1)	0 (0-0)	0 (0-0)	0 (0-0)
2000	Slovenia	0 (0-0)		0 (0-0)		0 (0-0)		0 (0-0)		0 (0-0)		0 (0-0)	
2001	Slovenia	1 (0-1)		1 (0-1)		0 (0-0)		0 (0-0)		0 (0-0)		0 (0-0)	
2002	Slovenia	1 (1-1)		1 (1-1)		1 (0-2)		1 (0-2)		1 (0-2)		1 (0-2)	
2003	Slovenia	2 (1-2)		2 (1-2)		0 (0-0)		0 (0-0)		0 (0-0)		0 (0-0)	
2004	Slovenia	0 (0-1)		1 (0-1)		0 (0-0)		0 (0-0)		0 (0-0)		0 (0-0)	
2005	Slovenia	0 (0-0)		0 (0-0)		0 (0-1)		0 (0-1)		0 (0-0)		0 (0-1)	
2006	Slovenia	0 (0-0)		0 (0-0)		0 (0-0)		0 (0-0)		0 (0-0)		0 (0-0)	
2007	Slovenia	1 (1-1)		1 (1-1)		0 (0-0)		0 (0-0)		0 (0-0)		0 (0-0)	
2008	Slovenia	0 (0-0)		0 (0-0)		0 (0-0)		0 (0-0)		0 (0-0)		0 (0-0)	
2009	Slovenia	1 (1-1)		1 (1-1)		0 (0-0)		0 (0-0)		0 (0-0)		0 (0-0)	
2010	Slovenia	1 (1-1)		1 (1-1)		0 (0-0)		0 (0-0)		0 (0-0)		0 (0-0)	
2011	Slovenia	1 (0-1)		1 (0-1)		0 (0-0)		0 (0-0)		0 (0-0)		0 (0-0)	
2012	Slovenia	1 (0-1)		1 (0-1)		0 (0-0)		0 (0-0)		0 (0-0)		0 (0-0)	
2013	Slovenia	1 (0-1)		0 (0-0)		0 (0-0)		0 (0-0)		0 (0-0)		0 (0-0)	
2014	Slovenia	0 (0-0)		0 (0-0)		0 (0-0)		0 (0-0)		0 (0-0)		0 (0-0)	
2015	Slovenia	0 (0-0)	0 (0-0)	0 (0-0)	0 (0-0)	0 (0-0)	0 (0-0)	0 (0-0)	0 (0-0)	0 (0-0)	0 (0-0)	0 (0-0)	0 (0-0)
2000	Solomon Islands	22 (16-23)		35 (25-37)		2 (1-6)		3 (1-9)		2 (1-5)		3 (1-8)	
2001	Solomon Islands	23 (16-24)		35 (25-37)		2 (1-6)		3 (1-9)		2 (1-5)		3 (1-8)	
2002	Solomon Islands	24 (17-25)		36 (25-37)		2 (1-6)		3 (1-9)		2 (1-5)		3 (1-8)	
2003	Solomon Islands	24 (17-25)		36 (25-37)		2 (1-6)		3 (1-9)		2 (1-6)		3 (1-8)	
2004	Solomon Islands	25 (18-26)		36 (26-38)		2 (1-6)		3 (1-9)		2 (1-6)		3 (1-8)	
2005	Solomon Islands	26 (18-27)		36 (26-38)		2 (1-7)		3 (1-9)		2 (1-6)		3 (1-8)	
2006	Solomon Islands	27 (19-28)		37 (26-38)		2 (1-7)		3 (1-9)		2 (1-6)		3 (1-8)	
2007	Solomon Islands	27 (19-28)		36 (26-38)		2 (1-7)		3 (1-9)		2 (1-6)		3 (1-8)	
2008	Solomon Islands	27 (19-28)		36 (25-37)		2 (1-7)		3 (1-9)		2 (1-6)		3 (1-8)	
2009	Solomon Islands	27 (19-28)		35 (25-37)		2 (1-7)		3 (1-9)		2 (1-6)		3 (1-8)	
2010	Solomon Islands	27 (19-28)		35 (25-36)		2 (1-7)		3 (1-8)		2 (1-6)		3 (1-8)	
2011	Solomon Islands	27 (19-28)		34 (24-35)		2 (1-7)		3 (1-8)		2 (1-6)		3 (1-7)	
2012	Solomon Islands	26 (19-27)		33 (23-34)		2 (1-6)		3 (1-8)		2 (1-6)		2 (1-7)	
2013	Solomon Islands	26 (18-27)		32 (23-33)		2 (1-9)		3 (1-11)		2 (0-8)		2 (1-10)	
2014	Solomon Islands	25 (17-26)		31 (22-32)		2 (1-9)		3 (1-11)		2 (0-8)		2 (1-9)	
2015	Solomon Islands	18 (12-18)	0 (0-0)	22 (15-23)	0 (0-0)	2 (0-7)	0 (0-0)	2 (1-8)	0 (0-0)	1 (0-6)	0 (0-0)	2 (0-7)	0 (0-0)
2000	Somalia	3620 (2567-3772)		251 (178-261)		749 (367-1520)		52 (25-105)		236 (116-478)		16 (8-33)	
2001	Somalia	3687 (2615-3843)		246 (175-257)		764 (375-1550)		51 (25-103)		240 (118-487)		16 (8-33)	
2002	Somalia	3838 (2722-4000)		250 (178-261)		795 (390-1614)		52 (25-105)		250 (123-507)		16 (8-33)	
2003	Somalia	4111 (2915-4284)		264 (188-276)		852 (418-1729)		55 (27-111)		268 (131-543)		17 (8-35)	
2004	Somalia	4235 (3003-4414)		269 (191-280)		877 (430-1780)		56 (27-113)		276 (135-559)		18 (9-36)	
2005	Somalia	4394 (3116-4579)		274 (195-286)		908 (445-1843)		57 (28-115)		286 (140-579)		18 (9-36)	
2006	Somalia	4554 (3229-4745)		280 (198-292)		940 (461-1906)		58 (28-117)		295 (145-599)		18 (9-37)	
2007	Somalia	4716 (3344-4915)		284 (201-295)		972 (477-1971)		58 (29-119)		306 (150-620)		18 (9-37)	
2008	Somalia	4833 (3427-5037)		283 (201-295)		996 (489-2018)		58 (29-118)		313 (154-635)		18 (9-37)	
2009	Somalia	4906 (3479-5113)		281 (199-292)		1011 (497-2049)		58 (28-117)		318 (156-644)		18 (9-37)	
2010	Somalia	4928 (3494-5135)		276 (196-288)		1016 (500-2057)		57 (28-115)		319 (157-647)		18 (9-36)	
2011	Somalia	4885 (3464-5091)		267 (189-278)		1005 (496-2036)		55 (27-111)		316 (156-640)		17 (9-35)	
2012	Somalia	4789 (3396-4991)		258 (183-268)		982 (486-1991)		53 (26-107)		309 (153-626)		17 (8-34)	
2013	Somalia	4688 (3324-4886)		250 (177-260)		957 (305-2238)		51 (16-119)		301 (96-704)		16 (5-37)	
2014	Somalia	4558 (3232-4750)		240 (170-250)		927 (296-2170)		49 (16-114)		291 (93-682)		15 (5-36)	
2015	Somalia	4493 (3186-4682)	192 (136-200)	232 (164-242)	10 (7-10)	911 (292-2133)	39 (12-91)	47 (15-110)	2 (1-5)	286 (92-671)	12 (4-29)	15 (5-35)	1 (0-1)
2000	South Africa	4235 (3003-4413)		80 (56-83)		459 (216-893)		9 (4-17)		144 (68-281)		3 (1-5)	
2001	South Africa	4220 (2993-4398)		79 (56-82)		455 (213-886)		8 (4-17)		143 (67-278)		3 (1-5)	
2002	South Africa	4045 (2869-4216)		76 (54-79)		433 (202-843)		8 (4-16)		136 (63-265)		3 (1-5)	
2003	South Africa	3789 (2686-3948)		73 (51-76)		401 (187-782)		8 (4-15)		126 (59-246)		2 (1-5)	
2004	South Africa	3550 (2518-3700)		69 (49-72)		372 (175-726)		7 (3-14)		117 (55-228)		2 (1-4)	
2005	South Africa	3443 (2441-3588)		67 (48-70)		357 (168-697)		7 (3-14)		112 (53-219)		2 (1-4)	
2006	South Africa	3420 (2425-3564)		67 (48-70)		351 (146-705)		7 (3-14)		312 (130-628)		6 (3-12)	

Spn = *Streptococcus pneumoniae*; npnm = non-pneumonia, non-meningitis; HIV = deaths only in HIV-infected children; Uncertainty range provided in parentheses.

Year	Country	Spn pneumonia deaths (UR)	Spn pneumonia deaths, HIV (UR)	Spn pneumonia mortality rate (UR)	Spn pneumonia mortality rate, HIV (UR)	Spn meningitis deaths (UR)	Spn meningitis deaths, HIV (UR)	Spn meningitis mortality rate (UR)	Spn meningitis mortality rate, HIV (UR)	Spn NPNM deaths (UR)	Spn NPNM deaths, HIV (UR)	Spn NPNM mortality rate (UR)	Spn NPNM mortality rate, HIV (UR)
2007	South Africa	3518 (2495-3666)		68 (48-71)		358 (150-720)		7 (3-14)		319 (134-641)		6 (3-12)	
2008	South Africa	3696 (2621-3852)		70 (50-73)		375 (159-753)		7 (3-14)		334 (141-670)		6 (3-13)	
2009	South Africa	3751 (2660-3910)		69 (49-72)		381 (163-760)		7 (3-14)		339 (146-677)		6 (3-12)	
2010	South Africa	3392 (2405-3535)		62 (44-64)		342 (151-674)		6 (3-12)		304 (135-600)		6 (2-11)	
2011	South Africa	2943 (2087-3067)		53 (37-55)		294 (132-576)		5 (2-10)		262 (118-513)		5 (2-9)	
2012	South Africa	2182 (1547-2274)		39 (28-41)		213 (96-414)		4 (2-7)		189 (85-369)		3 (2-7)	
2013	South Africa	2016 (1430-2101)		37 (26-38)		202 (93-391)		4 (2-7)		180 (83-348)		3 (2-6)	
2014	South Africa	1786 (1267-1862)		33 (24-35)		182 (84-351)		3 (2-7)		162 (75-312)		3 (1-6)	
2015	South Africa	1118 (793-1165)	580 (411-605)	21 (15-22)	11 (8-11)	106 (49-203)	55 (25-105)	2 (1-4)	1 (0-2)	94 (44-181)	49 (23-94)	2 (1-3)	1 (0-2)
2000	South Sudan	2919 (2070-3042)		249 (176-259)		454 (244-852)		39 (21-73)		143 (77-268)		12 (7-23)	
2001	South Sudan	2928 (2076-3052)		240 (170-251)		452 (244-846)		37 (20-69)		142 (77-266)		12 (6-22)	
2002	South Sudan	2836 (2011-2956)		226 (161-236)		433 (236-812)		35 (19-65)		136 (74-255)		11 (6-20)	
2003	South Sudan	2739 (1942-2855)		214 (152-223)		414 (228-775)		32 (18-61)		130 (72-244)		10 (6-19)	
2004	South Sudan	2651 (1880-2763)		202 (143-211)		394 (151-845)		30 (11-64)		124 (47-266)		9 (4-20)	
2005	South Sudan	2518 (1786-2624)		186 (132-194)		370 (143-794)		27 (11-59)		116 (45-249)		9 (3-18)	
2006	South Sudan	2457 (1743-2561)		176 (125-183)		356 (140-764)		25 (10-55)		112 (44-240)		8 (3-17)	
2007	South Sudan	2410 (1709-2512)		166 (118-173)		346 (137-740)		24 (9-51)		109 (43-233)		8 (3-16)	
2008	South Sudan	2444 (1733-2547)		162 (115-169)		351 (140-749)		23 (9-50)		110 (44-235)		7 (3-16)	
2009	South Sudan	2434 (1726-2537)		155 (110-162)		352 (141-749)		22 (9-48)		111 (44-235)		7 (3-15)	
2010	South Sudan	2437 (1728-2539)		150 (106-156)		359 (145-761)		22 (9-47)		113 (46-239)		7 (3-15)	
2011	South Sudan	2434 (1726-2536)		143 (102-149)		368 (150-778)		22 (9-46)		116 (47-245)		7 (3-14)	
2012	South Sudan	2345 (1663-2444)		133 (94-139)		364 (148-766)		21 (8-43)		114 (47-241)		6 (3-14)	
2013	South Sudan	2367 (1678-2467)		130 (92-135)		374 (154-786)		21 (8-43)		118 (48-247)		6 (3-14)	
2014	South Sudan	2323 (1647-2421)		124 (88-129)		373 (154-782)		20 (8-42)		117 (48-246)		6 (3-13)	
2015	South Sudan	2287 (1622-2384)	430 (305-448)	119 (84-124)	22 (16-23)	370 (154-773)	70 (29-146)	19 (8-40)	4 (2-8)	116 (48-243)	22 (9-46)	6 (3-13)	1 (0-2)
2000	Spain	18 (13-18)		1 (1-1)		25 (11-46)		1 (1-2)		23 (10-41)		1 (1-2)	
2001	Spain	6 (4-6)		0 (0-0)		15 (7-28)		1 (0-1)		14 (6-25)		1 (0-1)	
2002	Spain	16 (12-17)		1 (1-1)		15 (7-27)		1 (0-1)		13 (6-24)		1 (0-1)	
2003	Spain	18 (13-19)		1 (1-1)		20 (9-37)		1 (0-2)		18 (8-33)		1 (0-2)	
2004	Spain	14 (10-15)		1 (0-1)		16 (7-29)		1 (0-1)		14 (6-26)		1 (0-1)	
2005	Spain	13 (9-14)		1 (0-1)		22 (10-40)		1 (0-2)		20 (9-35)		1 (0-2)	
2006	Spain	18 (12-18)		1 (1-1)		18 (8-33)		1 (0-1)		16 (7-29)		1 (0-1)	
2007	Spain	22 (16-23)		1 (1-1)		17 (8-31)		1 (0-1)		15 (7-28)		1 (0-1)	
2008	Spain	13 (9-14)		1 (0-1)		15 (7-27)		1 (0-1)		13 (6-24)		1 (0-1)	
2009	Spain	17 (12-17)		1 (0-1)		17 (8-31)		1 (0-1)		15 (7-28)		1 (0-1)	
2010	Spain	14 (10-15)		1 (0-1)		9 (4-17)		0 (0-1)		8 (4-15)		0 (0-1)	
2011	Spain	12 (9-13)		1 (0-1)		10 (5-18)		0 (0-1)		9 (4-16)		0 (0-1)	
2012	Spain	9 (6-9)		0 (0-0)		7 (3-13)		0 (0-1)		6 (3-12)		0 (0-1)	
2013	Spain	7 (5-7)		0 (0-0)		10 (5-18)		0 (0-1)		9 (4-16)		0 (0-1)	
2014	Spain	8 (6-8)		0 (0-0)		8 (4-15)		0 (0-1)		7 (3-13)		0 (0-1)	
2015	Spain	7 (5-8)	0 (0-0)	0 (0-0)	0 (0-0)	7 (3-13)	0 (0-0)	0 (0-1)	0 (0-0)	6 (3-12)	0 (0-0)	0 (0-1)	0 (0-0)
2000	Sri Lanka	126 (89-131)		8 (6-8)		15 (4-39)		1 (0-2)		13 (3-35)		1 (0-2)	
2001	Sri Lanka	119 (84-124)		7 (5-8)		14 (3-37)		1 (0-2)		12 (3-33)		1 (0-2)	
2002	Sri Lanka	113 (80-117)		7 (5-7)		13 (3-35)		1 (0-2)		12 (3-31)		1 (0-2)	
2003	Sri Lanka	110 (78-115)		7 (5-7)		13 (3-35)		1 (0-2)		12 (3-31)		1 (0-2)	
2004	Sri Lanka	101 (72-105)		6 (4-6)		12 (3-32)		1 (0-2)		11 (2-29)		1 (0-2)	
2005	Sri Lanka	99 (70-103)		6 (4-6)		12 (3-31)		1 (0-2)		11 (3-28)		1 (0-2)	
2006	Sri Lanka	90 (64-94)		5 (4-5)		11 (3-29)		1 (0-2)		10 (2-25)		1 (0-1)	
2007	Sri Lanka	83 (59-87)		5 (3-5)		10 (3-26)		1 (0-1)		9 (2-24)		1 (0-1)	
2008	Sri Lanka	76 (54-79)		4 (3-4)		9 (2-24)		1 (0-1)		8 (2-22)		0 (0-1)	
2009	Sri Lanka	71 (50-74)		4 (3-4)		9 (2-23)		0 (0-1)		8 (2-20)		0 (0-1)	
2010	Sri Lanka	76 (54-80)		4 (3-5)		17 (4-43)		1 (0-2)		15 (4-39)		1 (0-2)	
2011	Sri Lanka	74 (53-77)		4 (3-4)		17 (4-44)		1 (0-3)		15 (4-39)		1 (0-2)	
2012	Sri Lanka	70 (50-73)		4 (3-4)		16 (4-41)		1 (0-2)		14 (4-37)		1 (0-2)	
2013	Sri Lanka	67 (47-69)		4 (3-4)		15 (4-39)		1 (0-2)		13 (4-35)		1 (0-2)	
2014	Sri Lanka	62 (44-65)		4 (3-4)		14 (4-36)		1 (0-2)		12 (3-32)		1 (0-2)	
2015	Sri Lanka	61 (43-63)	0 (0-0)	4 (3-4)	0 (0-0)	14 (4-35)	0 (0-0)	1 (0-2)	0 (0-0)	12 (3-32)	0 (0-0)	1 (0-2)	0 (0-0)
2000	Sudan	7034 (4988-7330)		149 (106-156)		1162 (597-2148)		25 (13-46)		365 (188-675)		8 (4-14)	
2001	Sudan	7230 (5127-7534)		150 (106-157)		1181 (594-2205)		25 (12-46)		371 (187-693)		8 (4-14)	
2002	Sudan	7214 (5116-7519)		147 (104-153)		1164 (574-2194)		24 (12-45)		366 (181-690)		7 (4-14)	
2003	Sudan	7213 (5115-7517)		143 (102-149)		1148 (556-2187)		23 (11-43)		361 (175-687)		7 (3-14)	
2004	Sudan	7168 (5083-7470)		139 (99-145)		1123 (534-2164)		22 (10-42)		353 (168-680)		7 (3-13)	
2005	Sudan	6914 (4903-7206)		132 (94-138)		1066 (499-2077)		20 (10-40)		335 (157-653)		6 (3-12)	
2006	Sudan	6820 (4836-7108)		128 (91-133)		1036 (476-2038)		19 (9-38)		326 (150-641)		6 (3-12)	
2007	Sudan	6692 (4745-6974)		124 (88-129)		1002 (454-1992)		19 (8-37)		315 (143-626)		6 (3-12)	
2008	Sudan	6723 (4768-7007)		123 (87-128)		996 (444-2000)		18 (8-37)		313 (139-629)		6 (3-11)	
2009	Sudan	6541 (4638-6817)		119 (84-124)		961 (421-1947)		17 (8-35)		302 (132-612)		5 (2-11)	
2010	Sudan	6377 (4522-6645)		115 (81-119)		931 (402-1904)		17 (7-34)		293 (126-598)		5 (2-11)	
2011	Sudan	6311 (4475-6577)		113 (80-117)		914 (389-1889)		16 (7-34)		287 (122-594)		5 (2-11)	
2012	Sudan	6103 (4328-6360)		108 (77-112)		878 (369-1833)		16 (7-32)		276 (116-576)		5 (2-10)	
2013	Sudan	5430 (3850-5659)		95 (67-99)		765 (267-1660)		13 (5-29)		681 (238-1478)		12 (4-26)	
2014	Sudan	3637 (2579-3790)		63 (45-66)		480 (165-1050)		8 (3-18)		427 (147-935)		7 (3-16)	
2015	Sudan	3072 (2178-3201)	47 (33-49)	52 (37-55)	1 (1-1)	412 (139-909)	6 (2-14)	7 (2-16)	0 (0-0)	367 (124-810)	6 (2-12)	6 (2-14)	0 (0-0)
2000	Suriname	7 (5-7)		13 (9-13)		2 (1-5)		4 (1-9)		2 (0-4)		3 (1-8)	
2001	Suriname	7 (5-7)		13 (9-13)		1 (0-4)		3 (1-7)		1 (0-3)		2 (1-6)	
2002	Suriname	4 (3-4)		7 (5-7)		3 (1-9)		7 (2-17)		3 (1-8)		6 (2-15)	
2003	Suriname	10 (7-11)		20 (15-21)		1 (0-2)		2 (1-4)		1 (0-2)		1 (0-4)	
2004	Suriname	4 (3-4)		8 (6-8)		1 (0-4)		3 (1-7)		1 (0-3)		3 (1-7)	
2005	Suriname	10 (7-10)		20 (14-21)		1 (0-2)		1 (0-4)		1 (0-2)		1 (0-3)	
2006	Suriname	7 (5-7)		14 (10-14)		3 (1-9)		6 (2-18)		3 (1-8)		5 (1-16)	
2007	Suriname	9 (7-10)		20 (14-20)		1 (0-2)		2 (0-4)		1 (0-2)		1 (0-4)	
2008	Suriname	6 (4-6)		12 (9-13)		2 (1-6)		4 (1-13)		2 (1-5)		4 (1-11)	
2009	Suriname	8 (6-9)		17 (12-18)		2 (1-7)		5 (1-14)		2 (1-6)		4 (1-13)	
2010	Suriname	9 (6-9)		18 (13-19)		4 (1-12)		9 (3-25)		4 (1-11)		8 (2-23)	
2011	Suriname	6 (4-6)		12 (9-13)		2 (1-6)		5 (1-13)		2 (1-6)		4 (1-12)	
2012	Suriname	8 (6-8)		16 (11-17)		0 (0-0)		0 (0-0)		0 (0-0)		0 (0-0)	
2013	Suriname	7 (5-7)		14 (10-15)		2 (1-5)		4 (1-11)		2 (0-5)		4 (1-10)	
2014	Suriname	7 (5-7)		14 (10-15)		2 (1-5)		4 (1-11)		2 (0-5)		4 (1-10)	

Spn = *Streptococcus pneumoniae*; npnm = non-pneumonia, non-meningitis; HIV = deaths only in HIV-infected children; Uncertainty range provided in parentheses.

Year	Country	Spn pneumonia deaths (UR)	Spn pneumonia deaths, HIV (UR)	Spn pneumonia mortality rate (UR)	Spn pneumonia mortality rate, HIV (UR)	Spn meningitis deaths (UR)	Spn meningitis deaths, HIV (UR)	Spn meningitis mortality rate (UR)	Spn meningitis mortality rate, HIV (UR)	Spn NPNM deaths (UR)	Spn NPNM deaths, HIV (UR)	Spn NPNM mortality rate (UR)	Spn NPNM mortality rate, HIV (UR)
2015	Suriname	6 (5-7)	0 (0-0)	14 (10-14)	0 (0-0)	2 (1-5)	0 (0-0)	4 (1-11)	0 (0-0)	2 (0-4)	0 (0-0)	3 (1-9)	0 (0-0)
2000	Swaziland	209 (148-218)		135 (96-141)		26 (12-52)		17 (7-34)		8 (4-16)		5 (2-11)	
2001	Swaziland	215 (152-224)		140 (99-146)		27 (12-53)		17 (8-34)		8 (4-17)		5 (2-11)	
2002	Swaziland	218 (155-228)		143 (101-149)		27 (13-54)		18 (8-35)		9 (4-17)		6 (3-11)	
2003	Swaziland	221 (157-230)		145 (103-151)		28 (13-54)		18 (9-35)		9 (4-17)		6 (3-11)	
2004	Swaziland	221 (156-230)		145 (103-151)		28 (13-53)		18 (9-35)		9 (4-17)		6 (3-11)	
2005	Swaziland	219 (156-229)		144 (102-150)		28 (14-52)		18 (9-34)		9 (4-16)		6 (3-11)	
2006	Swaziland	214 (152-223)		139 (99-145)		27 (14-50)		18 (9-33)		8 (4-16)		6 (3-10)	
2007	Swaziland	209 (148-218)		134 (95-140)		26 (13-49)		17 (9-32)		8 (4-15)		5 (3-10)	
2008	Swaziland	202 (143-211)		129 (91-134)		25 (12-48)		16 (8-31)		8 (4-15)		5 (2-10)	
2009	Swaziland	193 (137-201)		121 (86-126)		24 (11-47)		15 (7-29)		7 (3-15)		5 (2-9)	
2010	Swaziland	182 (129-190)		113 (80-118)		22 (10-45)		14 (6-28)		7 (3-14)		4 (2-9)	
2011	Swaziland	173 (123-180)		105 (74-109)		21 (9-42)		13 (6-26)		7 (3-13)		4 (2-8)	
2012	Swaziland	166 (118-173)		99 (70-104)		20 (8-41)		12 (5-25)		18 (7-37)		11 (4-22)	
2013	Swaziland	159 (113-166)		95 (67-99)		19 (8-39)		11 (5-23)		17 (7-35)		10 (4-21)	
2014	Swaziland	115 (81-120)		68 (48-71)		13 (5-27)		8 (3-16)		12 (5-24)		7 (3-14)	
2015	Swaziland	88 (63-92)	51 (36-53)	52 (37-54)	30 (21-31)	10 (4-20)	6 (2-12)	6 (2-12)	3 (1-7)	9 (4-18)	5 (2-10)	5 (2-11)	3 (1-6)
2000	Sweden	2 (1-2)		0 (0-0)		1 (1-3)		0 (0-1)		1 (1-2)		0 (0-1)	
2001	Sweden	2 (2-2)		1 (0-1)		1 (1-2)		0 (0-1)		1 (1-2)		0 (0-0)	
2002	Sweden	2 (1-2)		0 (0-0)		2 (1-4)		1 (0-1)		2 (1-4)		0 (0-1)	
2003	Sweden	4 (3-4)		1 (1-1)		2 (1-3)		0 (0-1)		2 (1-3)		0 (0-1)	
2004	Sweden	5 (4-5)		1 (1-1)		2 (1-3)		0 (0-1)		1 (1-2)		0 (0-1)	
2005	Sweden	5 (3-5)		1 (1-1)		2 (1-3)		0 (0-1)		2 (1-3)		0 (0-1)	
2006	Sweden	3 (2-3)		1 (0-1)		1 (1-3)		0 (0-0)		1 (1-2)		0 (0-0)	
2007	Sweden	4 (3-4)		1 (1-1)		3 (1-5)		1 (0-1)		2 (1-4)		0 (0-1)	
2008	Sweden	7 (5-7)		1 (1-1)		0 (0-1)		0 (0-0)		0 (0-1)		0 (0-0)	
2009	Sweden	2 (2-2)		0 (0-0)		0 (0-0)		0 (0-0)		0 (0-0)		0 (0-0)	
2010	Sweden	2 (2-3)		0 (0-0)		1 (0-1)		0 (0-0)		1 (0-1)		0 (0-0)	
2011	Sweden	3 (2-3)		0 (0-0)		0 (0-0)		0 (0-0)		0 (0-0)		0 (0-0)	
2012	Sweden	1 (1-1)		0 (0-0)		0 (0-1)		0 (0-0)		0 (0-1)		0 (0-0)	
2013	Sweden	2 (1-2)		0 (0-0)		0 (0-0)		0 (0-0)		0 (0-0)		0 (0-0)	
2014	Sweden	2 (1-2)		0 (0-0)		0 (0-0)		0 (0-0)		0 (0-0)		0 (0-0)	
2015	Sweden	2 (1-2)	0 (0-0)	0 (0-0)	0 (0-0)	0 (0-0)	0 (0-0)	0 (0-0)	0 (0-0)	0 (0-0)	0 (0-0)	0 (0-0)	0 (0-0)
2000	Switzerland	2 (1-2)		0 (0-0)		3 (1-6)		1 (0-1)		3 (1-5)		1 (0-1)	
2001	Switzerland	3 (2-4)		1 (1-1)		4 (2-7)		1 (0-2)		4 (2-7)		1 (0-2)	
2002	Switzerland	3 (2-3)		1 (1-1)		4 (2-8)		1 (1-2)		4 (2-7)		1 (0-2)	
2003	Switzerland	2 (1-2)		1 (0-1)		2 (1-4)		1 (0-1)		2 (1-4)		1 (0-1)	
2004	Switzerland	1 (1-1)		0 (0-0)		0 (0-1)		0 (0-0)		0 (0-1)		0 (0-0)	
2005	Switzerland	3 (2-3)		1 (1-1)		2 (1-4)		1 (0-1)		2 (1-3)		0 (0-1)	
2006	Switzerland	1 (1-1)		0 (0-0)		2 (1-3)		1 (0-1)		2 (1-3)		0 (0-1)	
2007	Switzerland	1 (1-1)		0 (0-0)		1 (0-2)		0 (0-1)		1 (0-2)		0 (0-0)	
2008	Switzerland	2 (2-2)		1 (0-1)		1 (0-1)		0 (0-0)		1 (0-1)		0 (0-0)	
2009	Switzerland	2 (1-2)		0 (0-0)		2 (1-4)		1 (0-1)		2 (1-3)		0 (0-1)	
2010	Switzerland	2 (1-2)		1 (0-1)		1 (0-1)		0 (0-0)		1 (0-1)		0 (0-0)	
2011	Switzerland	1 (1-1)		0 (0-0)		0 (0-1)		0 (0-0)		0 (0-1)		0 (0-0)	
2012	Switzerland	0 (0-0)		0 (0-0)		0 (0-0)		0 (0-0)		0 (0-0)		0 (0-0)	
2013	Switzerland	0 (0-0)		0 (0-0)		0 (0-0)		0 (0-0)		0 (0-0)		0 (0-0)	
2014	Switzerland	0 (0-0)		0 (0-0)		0 (0-0)		0 (0-0)		0 (0-0)		0 (0-0)	
2015	Switzerland	0 (0-0)	0 (0-0)	0 (0-0)	0 (0-0)	0 (0-0)	0 (0-0)	0 (0-0)	0 (0-0)	0 (0-0)	0 (0-0)	0 (0-0)	0 (0-0)
2000	Syrian Arab Republic	476 (338-496)		20 (14-21)		21 (6-55)		1 (0-2)		19 (5-49)		1 (0-2)	
2001	Syrian Arab Republic	479 (340-499)		20 (14-21)		38 (10-97)		2 (0-4)		34 (9-86)		1 (0-4)	
2002	Syrian Arab Republic	443 (314-462)		18 (13-19)		37 (11-93)		2 (0-4)		33 (9-83)		1 (0-3)	
2003	Syrian Arab Republic	396 (281-412)		17 (12-17)		34 (10-84)		1 (0-3)		30 (9-74)		1 (0-3)	
2004	Syrian Arab Republic	358 (254-373)		15 (11-16)		31 (10-76)		1 (0-3)		28 (9-67)		1 (0-3)	
2005	Syrian Arab Republic	331 (235-345)		14 (10-14)		29 (10-69)		1 (0-3)		26 (9-62)		1 (0-3)	
2006	Syrian Arab Republic	318 (225-331)		13 (9-14)		29 (11-68)		1 (0-3)		26 (9-61)		1 (0-3)	
2007	Syrian Arab Republic	310 (220-323)		13 (9-13)		29 (11-65)		1 (0-3)		26 (10-58)		1 (0-2)	
2008	Syrian Arab Republic	299 (212-312)		12 (9-13)		28 (11-61)		1 (0-2)		25 (10-54)		1 (0-2)	
2009	Syrian Arab Republic	291 (206-303)		12 (8-12)		27 (12-57)		1 (0-2)		24 (11-50)		1 (0-2)	
2010	Syrian Arab Republic	262 (186-273)		11 (7-11)		24 (11-49)		1 (0-2)		22 (10-43)		1 (0-2)	
2011	Syrian Arab Republic	231 (164-240)		9 (7-10)		21 (11-40)		1 (0-2)		19 (10-36)		1 (0-1)	
2012	Syrian Arab Republic	184 (130-192)		8 (5-8)		16 (9-28)		1 (0-1)		14 (8-25)		1 (0-1)	
2013	Syrian Arab Republic	148 (105-154)		6 (5-7)		13 (8-21)		1 (0-1)		11 (7-19)		0 (0-1)	
2014	Syrian Arab Republic	127 (90-132)		6 (4-6)		11 (7-17)		1 (0-1)		10 (7-15)		0 (0-1)	
2015	Syrian Arab Republic	123 (87-128)	0 (0-0)	6 (4-6)	0 (0-0)	11 (7-16)	0 (0-0)	1 (0-1)	0 (0-0)	10 (6-14)	0 (0-0)	0 (0-1)	0 (0-0)
2000	Tajikistan	1247 (884-1299)		143 (101-149)		234 (91-505)		27 (10-58)		74 (28-159)		8 (3-18)	
2001	Tajikistan	1128 (800-1176)		130 (92-135)		209 (83-446)		24 (10-51)		66 (26-140)		8 (3-16)	
2002	Tajikistan	1006 (713-1048)		117 (83-122)		183 (74-388)		21 (9-45)		58 (23-122)		7 (3-14)	
2003	Tajikistan	895 (635-933)		105 (75-110)		160 (57-346)		19 (7-41)		142 (51-308)		17 (6-36)	
2004	Tajikistan	813 (577-848)		96 (68-101)		142 (53-306)		17 (6-36)		127 (47-272)		15 (6-32)	
2005	Tajikistan	756 (536-788)		89 (63-93)		130 (50-276)		15 (6-33)		115 (44-246)		14 (5-29)	
2006	Tajikistan	717 (508-747)		84 (60-88)		121 (46-257)		14 (5-30)		108 (41-229)		13 (5-27)	
2007	Tajikistan	700 (496-729)		81 (57-84)		116 (44-247)		13 (5-29)		104 (39-220)		12 (5-25)	
2008	Tajikistan	697 (494-726)		78 (55-81)		114 (44-243)		13 (5-27)		102 (39-217)		11 (4-24)	
2009	Tajikistan	708 (502-738)		76 (54-79)		115 (44-245)		12 (5-26)		103 (39-218)		11 (4-23)	
2010	Tajikistan	717 (509-748)		74 (53-77)		116 (44-246)		12 (5-26)		103 (39-219)		11 (4-23)	
2011	Tajikistan	724 (514-755)		72 (51-75)		116 (44-246)		11 (4-24)		103 (39-219)		10 (4-22)	
2012	Tajikistan	729 (517-760)		69 (49-72)		115 (43-245)		11 (4-23)		102 (39-218)		10 (4-21)	
2013	Tajikistan	727 (516-758)		66 (47-69)		114 (43-243)		10 (4-22)		101 (38-216)		9 (3-20)	
2014	Tajikistan	720 (510-750)		64 (45-67)		112 (42-239)		10 (4-21)		100 (37-212)		9 (3-19)	
2015	Tajikistan	700 (496-729)	13 (9-14)	61 (43-63)	1 (1-1)	108 (41-231)	2 (1-4)	9 (4-20)	0 (0-0)	96 (36-206)	2 (1-4)	8 (3-18)	0 (0-0)
2000	Thailand	635 (450-662)		14 (10-14)		74 (30-162)		2 (1-4)		66 (27-144)		1 (1-3)	
2001	Thailand	582 (413-607)		13 (9-13)		69 (28-150)		2 (1-3)		61 (25-133)		1 (1-3)	
2002	Thailand	540 (383-563)		12 (9-13)		65 (27-140)		1 (1-3)		58 (24-125)		1 (1-3)	
2003	Thailand	504 (357-525)		11 (8-12)		61 (25-132)		1 (1-3)		54 (23-117)		1 (1-3)	
2004	Thailand	465 (330-484)		10 (7-11)		57 (24-122)		1 (1-3)		51 (21-109)		1 (0-2)	
2005	Thailand	423 (300-440)		10 (7-10)		52 (22-112)		1 (1-3)		47 (20-100)		1 (0-2)	
2006	Thailand	387 (274-403)		9 (6-9)		48 (20-103)		1 (0-2)		43 (18-92)		1 (0-2)	

Spn = *Streptococcus pneumoniae*; npnm = non-pneumonia, non-meningitis; HIV = deaths only in HIV-infected children; Uncertainty range provided in parentheses.

Year	Country	Spn pneumonia deaths (UR)	Spn pneumonia deaths, HIV (UR)	Spn pneumonia mortality rate (UR)	Spn pneumonia mortality rate, HIV (UR)	Spn meningitis deaths (UR)	Spn meningitis deaths, HIV (UR)	Spn meningitis mortality rate (UR)	Spn meningitis mortality rate, HIV (UR)	Spn NPNM deaths (UR)	Spn NPNM deaths, HIV (UR)	Spn NPNM mortality rate (UR)	Spn NPNM mortality rate, HIV (UR)
2007	Thailand	355 (251-370)		8 (6-9)		45 (19-95)		1 (0-2)		40 (17-85)		1 (0-2)	
2008	Thailand	322 (228-335)		8 (6-8)		41 (17-87)		1 (0-2)		36 (15-77)		1 (0-2)	
2009	Thailand	302 (214-314)		8 (5-8)		38 (16-82)		1 (0-2)		34 (14-73)		1 (0-2)	
2010	Thailand	285 (202-297)		7 (5-8)		36 (15-77)		1 (0-2)		32 (13-69)		1 (0-2)	
2011	Thailand	268 (190-280)		7 (5-7)		34 (14-72)		1 (0-2)		30 (12-64)		1 (0-2)	
2012	Thailand	252 (179-263)		7 (5-7)		31 (13-68)		1 (0-2)		28 (12-60)		1 (0-2)	
2013	Thailand	239 (170-249)		6 (4-7)		29 (12-63)		1 (0-2)		26 (11-56)		1 (0-1)	
2014	Thailand	225 (159-234)		6 (4-6)		27 (11-59)		1 (0-2)		24 (10-52)		1 (0-1)	
2015	Thailand	219 (155-228)	0 (0-0)	6 (4-6)	0 (0-0)	26 (11-56)	0 (0-0)	1 (0-2)	0 (0-0)	23 (10-50)	0 (0-0)	1 (0-1)	0 (0-0)
2000	The former Yugoslav Republic of Macedonia	11 (8-12)		8 (6-9)		1 (0-2)		1 (0-1)		1 (0-2)		0 (0-1)	
2001	The former Yugoslav Republic of Macedonia	12 (9-13)		9 (7-10)		1 (1-4)		1 (0-3)		1 (1-3)		1 (0-3)	
2002	The former Yugoslav Republic of Macedonia	10 (7-11)		8 (6-8)		1 (1-4)		1 (0-3)		1 (1-3)		1 (0-3)	
2003	The former Yugoslav Republic of Macedonia	9 (7-10)		7 (5-8)		1 (1-3)		1 (0-3)		1 (1-3)		1 (0-2)	
2004	The former Yugoslav Republic of Macedonia	9 (6-9)		7 (5-7)		1 (1-3)		1 (0-3)		1 (1-3)		1 (0-2)	
2005	The former Yugoslav Republic of Macedonia	8 (6-8)		6 (5-7)		0 (0-1)		0 (0-1)		0 (0-1)		0 (0-1)	
2006	The former Yugoslav Republic of Macedonia	7 (5-8)		6 (4-6)		1 (0-2)		1 (0-2)		1 (0-2)		1 (0-1)	
2007	The former Yugoslav Republic of Macedonia	6 (4-6)		5 (3-5)		2 (1-6)		2 (1-5)		2 (1-5)		2 (1-5)	
2008	The former Yugoslav Republic of Macedonia	3 (2-3)		2 (2-3)		1 (0-3)		1 (0-2)		1 (0-2)		1 (0-2)	
2009	The former Yugoslav Republic of Macedonia	7 (5-7)		6 (4-7)		1 (1-3)		1 (0-3)		1 (0-3)		1 (0-2)	
2010	The former Yugoslav Republic of Macedonia	6 (4-6)		5 (4-5)		2 (1-6)		2 (1-5)		2 (1-5)		2 (1-5)	
2011	The former Yugoslav Republic of Macedonia	4 (3-5)		4 (3-4)		1 (1-3)		1 (1-3)		1 (0-3)		1 (1-3)	
2012	The former Yugoslav Republic of Macedonia	4 (3-4)		4 (3-4)		1 (1-3)		1 (1-3)		1 (0-3)		1 (0-2)	
2013	The former Yugoslav Republic of Macedonia	4 (3-4)		3 (2-3)		1 (1-3)		1 (0-3)		1 (0-3)		1 (0-2)	
2014	The former Yugoslav Republic of Macedonia	4 (3-4)		3 (2-3)		1 (1-3)		1 (0-2)		1 (0-2)		1 (0-2)	
2015	The former Yugoslav Republic of Macedonia	3 (2-3)	0 (0-0)	3 (2-3)	0 (0-0)	1 (0-2)	0 (0-0)	1 (0-2)	0 (0-0)	1 (0-2)	0 (0-0)	1 (0-2)	0 (0-0)
2000	Timor-Leste	299 (212-312)		175 (124-182)		42 (17-89)		25 (10-52)		13 (5-28)		8 (3-16)	
2001	Timor-Leste	278 (197-289)		162 (115-168)		41 (17-87)		24 (10-50)		13 (5-27)		8 (3-16)	
2002	Timor-Leste	256 (181-267)		149 (106-156)		39 (16-82)		23 (9-48)		12 (5-26)		7 (3-15)	
2003	Timor-Leste	235 (167-245)		138 (98-144)		36 (15-75)		21 (9-44)		11 (5-24)		7 (3-14)	
2004	Timor-Leste	214 (152-223)		128 (91-133)		32 (14-67)		19 (8-40)		10 (4-21)		6 (3-12)	
2005	Timor-Leste	193 (137-202)		118 (84-123)		28 (12-57)		17 (7-34)		9 (4-18)		5 (2-11)	
2006	Timor-Leste	173 (123-180)		110 (78-115)		23 (10-47)		15 (7-30)		7 (3-15)		5 (2-9)	
2007	Timor-Leste	154 (109-160)		102 (72-106)		20 (8-41)		13 (5-27)		17 (7-36)		12 (5-24)	
2008	Timor-Leste	140 (99-145)		95 (67-99)		17 (7-35)		11 (5-24)		15 (6-31)		10 (4-21)	
2009	Timor-Leste	130 (93-136)		89 (63-93)		15 (6-31)		10 (4-21)		14 (6-28)		9 (4-19)	
2010	Timor-Leste	130 (93-136)		87 (62-91)		15 (6-31)		10 (4-20)		13 (6-27)		9 (4-18)	
2011	Timor-Leste	134 (95-140)		89 (63-93)		15 (6-31)		10 (4-20)		14 (6-28)		9 (4-18)	
2012	Timor-Leste	145 (103-151)		90 (64-94)		17 (7-34)		10 (4-21)		15 (6-30)		9 (4-19)	
2013	Timor-Leste	157 (111-163)		89 (63-93)		18 (8-36)		10 (4-21)		16 (7-32)		9 (4-18)	
2014	Timor-Leste	164 (116-171)		86 (61-90)		19 (8-38)		10 (4-20)		17 (7-34)		9 (4-18)	
2015	Timor-Leste	165 (117-172)	1 (1-1)	83 (59-86)	1 (1-1)	19 (8-39)	0 (0-0)	10 (4-19)	0 (0-0)	17 (7-35)	0 (0-0)	9 (4-17)	0 (0-0)
2000	Togo	791 (561-824)		98 (70-103)		147 (52-333)		18 (7-41)		46 (16-105)		6 (2-13)	
2001	Togo	803 (569-836)		97 (69-101)		151 (53-341)		18 (6-41)		47 (17-107)		6 (2-13)	
2002	Togo	809 (574-843)		95 (68-99)		153 (53-346)		18 (6-41)		48 (17-109)		6 (2-13)	
2003	Togo	808 (573-842)		93 (66-97)		153 (53-348)		18 (6-40)		48 (17-110)		6 (2-13)	
2004	Togo	805 (571-839)		90 (64-94)		153 (52-350)		17 (6-39)		48 (16-110)		5 (2-12)	
2005	Togo	797 (565-831)		87 (61-90)		152 (52-348)		17 (6-38)		48 (16-109)		5 (2-12)	
2006	Togo	791 (561-824)		83 (59-87)		151 (51-346)		16 (5-36)		47 (16-109)		5 (2-11)	
2007	Togo	792 (562-825)		81 (57-84)		151 (52-344)		15 (5-35)		47 (16-108)		5 (2-11)	
2008	Togo	810 (574-844)		81 (57-84)		154 (54-349)		15 (5-35)		48 (17-110)		5 (2-11)	
2009	Togo	843 (598-878)		82 (58-86)		160 (57-361)		16 (6-35)		50 (18-114)		5 (2-11)	
2010	Togo	893 (633-930)		85 (60-89)		170 (61-380)		16 (6-36)		54 (19-120)		5 (2-11)	
2011	Togo	962 (682-1003)		90 (64-94)		184 (69-405)		17 (6-38)		58 (22-127)		5 (2-12)	
2012	Togo	1026 (727-1069)		94 (67-98)		197 (77-425)		18 (7-39)		62 (24-134)		6 (2-12)	
2013	Togo	1059 (751-1103)		96 (68-100)		203 (83-432)		18 (8-39)		64 (26-136)		6 (2-12)	
2014	Togo	950 (674-990)		85 (60-88)		179 (73-379)		16 (6-34)		56 (23-119)		5 (2-11)	
2015	Togo	699 (495-728)	39 (27-40)	61 (43-64)	3 (2-4)	126 (51-265)	7 (3-15)	11 (5-23)	1 (0-1)	40 (16-83)	2 (1-5)	3 (1-7)	0 (0-0)
2000	Tonga	2 (1-2)		12 (9-13)		0 (0-0)		2 (1-3)		0 (0-0)		1 (1-3)	
2001	Tonga	2 (1-2)		12 (9-13)		0 (0-0)		2 (1-3)		0 (0-0)		1 (1-3)	
2002	Tonga	2 (1-2)		12 (9-13)		0 (0-0)		2 (1-3)		0 (0-0)		1 (1-2)	
2003	Tonga	2 (1-2)		12 (8-12)		0 (0-0)		2 (1-3)		0 (0-0)		1 (1-2)	
2004	Tonga	2 (1-2)		12 (8-12)		0 (0-0)		2 (1-3)		0 (0-0)		1 (1-2)	
2005	Tonga	2 (1-2)		13 (9-13)		0 (0-1)		3 (1-5)		0 (0-1)		3 (1-5)	
2006	Tonga	2 (1-2)		13 (9-14)		0 (0-1)		3 (1-6)		0 (0-1)		3 (1-5)	
2007	Tonga	2 (1-2)		13 (9-14)		0 (0-1)		3 (1-6)		0 (0-1)		3 (1-5)	
2008	Tonga	2 (1-2)		13 (9-14)		0 (0-1)		3 (1-6)		0 (0-1)		3 (1-5)	
2009	Tonga	2 (1-2)		14 (10-14)		0 (0-1)		3 (1-6)		0 (0-1)		3 (1-5)	
2010	Tonga	2 (1-2)		14 (10-15)		0 (0-1)		4 (2-6)		0 (0-1)		3 (1-6)	
2011	Tonga	2 (1-2)		14 (10-15)		0 (0-1)		3 (2-6)		0 (0-1)		3 (1-5)	
2012	Tonga	2 (1-2)		13 (9-14)		0 (0-1)		3 (1-6)		0 (0-1)		3 (1-5)	
2013	Tonga	2 (1-2)		13 (9-14)		0 (0-1)		3 (1-6)		0 (0-1)		3 (1-5)	
2014	Tonga	2 (1-2)		13 (9-14)		0 (0-1)		3 (1-5)		0 (0-1)		3 (1-5)	
2015	Tonga	2 (1-2)	0 (0-0)	13 (9-14)	0 (0-0)	0 (0-1)	0 (0-0)	3 (1-5)	0 (0-0)	0 (0-1)	0 (0-0)	3 (1-5)	0 (0-0)
2000	Trinidad and Tobago	4 (3-4)		4 (3-4)		3 (1-8)		3 (1-9)		2 (1-7)		3 (1-8)	
2001	Trinidad and Tobago	5 (4-5)		6 (4-6)		1 (0-3)		1 (0-3)		1 (0-3)		1 (0-3)	
2002	Trinidad and Tobago	7 (5-7)		7 (5-8)		2 (0-5)		2 (0-5)		1 (0-4)		2 (0-5)	
2003	Trinidad and Tobago	14 (10-14)		15 (11-16)		4 (1-11)		4 (1-12)		3 (1-10)		4 (1-11)	
2004	Trinidad and Tobago	11 (8-11)		12 (9-13)		1 (0-3)		1 (0-3)		1 (0-3)		1 (0-3)	
2005	Trinidad and Tobago	10 (7-10)		11 (8-11)		2 (0-5)		2 (1-5)		2 (0-4)		2 (0-5)	
2006	Trinidad and Tobago	7 (5-7)		7 (5-8)		2 (1-6)		2 (1-6)		2 (0-5)		2 (1-5)	
2007	Trinidad and Tobago	10 (7-10)		11 (8-11)		1 (0-3)		1 (0-4)		1 (0-3)		1 (0-3)	
2008	Trinidad and Tobago	6 (4-6)		6 (4-7)		2 (0-5)		2 (1-6)		2 (0-5)		2 (0-5)	
2009	Trinidad and Tobago	6 (5-7)		7 (5-7)		1 (0-4)		2 (0-4)		1 (0-4)		1 (0-4)	
2010	Trinidad and Tobago	7 (5-7)		7 (5-8)		1 (0-4)		1 (0-4)		1 (0-4)		1 (0-4)	
2011	Trinidad and Tobago	7 (5-7)		7 (5-7)		1 (0-4)		1 (0-4)		1 (0-3)		1 (0-4)	
2012	Trinidad and Tobago	6 (4-6)		6 (4-6)		1 (0-3)		1 (0-3)		1 (0-3)		1 (0-3)	
2013	Trinidad and Tobago	5 (3-5)		5 (3-5)		1 (0-2)		1 (0-3)		1 (0-2)		1 (0-2)	
2014	Trinidad and Tobago	4 (3-4)		4 (3-4)		1 (0-2)		1 (0-2)		1 (0-2)		1 (0-2)	

Spn = *Streptococcus pneumoniae*; npnm = non-pneumonia, non-meningitis; HIV = deaths only in HIV-infected children; Uncertainty range provided in parentheses.

Year	Country	Spn pneumonia deaths (UR)	Spn pneumonia deaths, HIV (UR)	Spn pneumonia mortality rate (UR)	Spn pneumonia mortality rate, HIV (UR)	Spn meningitis deaths (UR)	Spn meningitis deaths, HIV (UR)	Spn meningitis mortality rate (UR)	Spn meningitis mortality rate, HIV (UR)	Spn NPNM deaths (UR)	Spn NPNM deaths, HIV (UR)	Spn NPNM mortality rate (UR)	Spn NPNM mortality rate, HIV (UR)
2015	Trinidad and Tobago	3 (2-3)	0 (0-0)	3 (2-4)	0 (0-0)	1 (0-2)	0 (0-0)	1 (0-2)	0 (0-0)	1 (0-2)	0 (0-0)	1 (0-2)	0 (0-0)
2000	Tunisia	205 (146-214)		25 (18-26)		11 (4-25)		1 (0-3)		10 (3-22)		1 (0-3)	
2001	Tunisia	182 (129-190)		22 (16-23)		10 (2-26)		1 (0-3)		9 (2-23)		1 (0-3)	
2002	Tunisia	163 (116-170)		20 (14-21)		9 (2-24)		1 (0-3)		8 (2-21)		1 (0-3)	
2003	Tunisia	159 (113-166)		20 (14-21)		16 (4-40)		2 (0-5)		14 (3-36)		2 (0-5)	
2004	Tunisia	146 (104-152)		19 (13-19)		15 (4-38)		2 (0-5)		13 (3-34)		2 (0-4)	
2005	Tunisia	134 (95-139)		17 (12-18)		14 (4-34)		2 (0-4)		12 (3-31)		2 (0-4)	
2006	Tunisia	110 (78-115)		14 (10-15)		6 (2-16)		1 (0-2)		6 (2-14)		1 (0-2)	
2007	Tunisia	102 (72-106)		13 (9-13)		6 (2-15)		1 (0-2)		5 (1-13)		1 (0-2)	
2008	Tunisia	94 (67-98)		12 (8-12)		6 (2-14)		1 (0-2)		5 (1-12)		1 (0-2)	
2009	Tunisia	89 (63-92)		11 (8-11)		5 (1-13)		1 (0-2)		5 (1-12)		1 (0-1)	
2010	Tunisia	83 (59-87)		10 (7-10)		5 (1-12)		1 (0-1)		4 (1-11)		1 (0-1)	
2011	Tunisia	80 (57-84)		9 (7-10)		7 (2-17)		1 (0-2)		6 (2-15)		1 (0-2)	
2012	Tunisia	83 (59-87)		9 (7-10)		9 (2-22)		1 (0-2)		8 (2-19)		1 (0-2)	
2013	Tunisia	81 (57-84)		9 (6-9)		8 (2-21)		1 (0-2)		7 (2-18)		1 (0-2)	
2014	Tunisia	77 (54-80)		8 (6-8)		8 (2-19)		1 (0-2)		7 (2-17)		1 (0-2)	
2015	Tunisia	72 (51-75)	0 (0-0)	7 (5-8)	0 (0-0)	7 (2-18)	0 (0-0)	1 (0-2)	0 (0-0)	6 (2-16)	0 (0-0)	1 (0-2)	0 (0-0)
2000	Turkey	1345 (954-1402)		21 (15-21)		147 (70-226)		2 (1-3)		131 (62-201)		2 (1-3)	
2001	Turkey	1189 (843-1239)		18 (13-19)		135 (63-206)		2 (1-3)		120 (56-183)		2 (1-3)	
2002	Turkey	1039 (736-1082)		16 (11-16)		124 (57-185)		2 (1-3)		110 (51-165)		2 (1-3)	
2003	Turkey	913 (647-951)		14 (10-15)		115 (52-168)		2 (1-3)		103 (46-149)		2 (1-2)	
2004	Turkey	802 (569-836)		12 (9-13)		110 (76-174)		2 (1-3)		98 (67-155)		2 (1-2)	
2005	Turkey	714 (506-744)		11 (8-12)		107 (69-159)		2 (1-2)		95 (62-142)		1 (1-2)	
2006	Turkey	628 (446-655)		10 (7-10)		102 (63-144)		2 (1-2)		90 (56-128)		1 (1-2)	
2007	Turkey	668 (474-697)		11 (8-11)		122 (73-168)		2 (1-3)		108 (65-150)		2 (1-2)	
2008	Turkey	620 (440-646)		10 (7-10)		118 (71-162)		2 (1-3)		105 (63-145)		2 (1-2)	
2009	Turkey	483 (343-504)		8 (5-8)		95 (57-131)		2 (1-2)		85 (51-117)		1 (1-2)	
2010	Turkey	299 (212-312)		5 (3-5)		81 (49-112)		1 (1-2)		72 (43-99)		1 (1-2)	
2011	Turkey	193 (137-201)		3 (2-3)		53 (32-72)		1 (0-1)		47 (28-65)		1 (0-1)	
2012	Turkey	135 (95-140)		2 (1-2)		57 (34-79)		1 (1-1)		51 (31-71)		1 (0-1)	
2013	Turkey	183 (129-190)		3 (2-3)		46 (28-64)		1 (0-1)		41 (25-57)		1 (0-1)	
2014	Turkey	147 (105-154)		2 (2-2)		46 (28-64)		1 (0-1)		41 (25-57)		1 (0-1)	
2015	Turkey	139 (98-144)	0 (0-0)	2 (1-2)	0 (0-0)	43 (26-60)	0 (0-0)	1 (0-1)	0 (0-0)	39 (23-53)	0 (0-0)	1 (0-1)	0 (0-0)
2000	Turkmenistan	497 (353-518)		103 (73-108)		62 (24-135)		13 (5-28)		20 (8-42)		4 (2-9)	
2001	Turkmenistan	482 (342-503)		104 (74-109)		60 (23-129)		13 (5-28)		19 (7-41)		4 (2-9)	
2002	Turkmenistan	488 (346-509)		106 (75-110)		60 (24-129)		13 (5-28)		19 (7-41)		4 (2-9)	
2003	Turkmenistan	504 (358-525)		106 (75-111)		61 (20-136)		13 (4-29)		54 (18-121)		11 (4-26)	
2004	Turkmenistan	514 (364-535)		105 (75-110)		61 (21-137)		13 (4-28)		55 (18-122)		11 (4-25)	
2005	Turkmenistan	508 (360-530)		102 (73-107)		59 (20-132)		12 (4-27)		53 (18-118)		11 (4-24)	
2006	Turkmenistan	493 (350-514)		96 (68-100)		56 (19-125)		11 (4-24)		50 (17-111)		10 (3-22)	
2007	Turkmenistan	466 (331-486)		90 (64-94)		52 (18-115)		10 (3-22)		46 (16-103)		9 (3-20)	
2008	Turkmenistan	431 (305-449)		85 (60-88)		47 (16-104)		9 (3-20)		42 (14-92)		8 (3-18)	
2009	Turkmenistan	398 (282-414)		80 (56-83)		42 (15-93)		8 (3-19)		38 (13-83)		8 (3-17)	
2010	Turkmenistan	378 (268-394)		76 (54-79)		39 (14-86)		8 (3-17)		35 (12-77)		7 (2-15)	
2011	Turkmenistan	363 (258-379)		74 (52-77)		37 (13-81)		7 (3-16)		33 (12-72)		7 (2-15)	
2012	Turkmenistan	358 (254-373)		72 (51-75)		35 (13-78)		7 (3-16)		31 (11-69)		6 (2-14)	
2013	Turkmenistan	356 (253-371)		71 (50-74)		35 (12-76)		7 (2-15)		31 (11-67)		6 (2-13)	
2014	Turkmenistan	354 (251-369)		69 (49-72)		34 (12-74)		7 (2-14)		30 (11-66)		6 (2-13)	
2015	Turkmenistan	347 (246-362)	0 (0-0)	67 (47-70)	0 (0-0)	33 (12-72)	0 (0-0)	6 (2-14)	0 (0-0)	29 (11-64)	0 (0-0)	6 (2-12)	0 (0-0)
2000	Tuvalu	1 (0-1)		39 (28-41)		0 (0-0)		2 (1-5)		0 (0-0)		2 (1-4)	
2001	Tuvalu	0 (0-0)		32 (23-34)		0 (0-0)		2 (1-4)		0 (0-0)		2 (1-3)	
2002	Tuvalu	0 (0-0)		33 (24-35)		0 (0-0)		2 (1-4)		0 (0-0)		2 (1-4)	
2003	Tuvalu	0 (0-0)		35 (25-37)		0 (0-0)		2 (1-4)		0 (0-0)		2 (1-4)	
2004	Tuvalu	0 (0-0)		28 (20-30)		0 (0-0)		2 (1-3)		0 (0-0)		1 (1-3)	
2005	Tuvalu	0 (0-0)		30 (21-31)		0 (0-0)		2 (1-3)		0 (0-0)		2 (1-3)	
2006	Tuvalu	0 (0-0)		31 (22-32)		0 (0-0)		2 (1-4)		0 (0-0)		2 (1-3)	
2007	Tuvalu	0 (0-0)		21 (15-21)		0 (0-0)		1 (0-2)		0 (0-0)		1 (0-2)	
2008	Tuvalu	0 (0-0)		20 (14-21)		0 (0-0)		1 (0-2)		0 (0-0)		1 (0-2)	
2009	Tuvalu	0 (0-0)		34 (24-35)		0 (0-0)		4 (1-8)		0 (0-0)		4 (1-8)	
2010	Tuvalu	0 (0-0)		23 (16-24)		0 (0-0)		3 (1-6)		0 (0-0)		3 (1-5)	
2011	Tuvalu	0 (0-0)		22 (16-23)		0 (0-0)		3 (1-6)		0 (0-0)		3 (1-5)	
2012	Tuvalu	0 (0-0)		22 (16-23)		0 (0-0)		3 (2-7)		0 (0-0)		3 (2-7)	
2013	Tuvalu	0 (0-0)		22 (15-23)		0 (0-0)		3 (1-6)		0 (0-0)		3 (1-6)	
2014	Tuvalu	0 (0-0)		22 (15-22)		0 (0-0)		3 (2-7)		0 (0-0)		3 (1-6)	
2015	Tuvalu	0 (0-0)	0 (0-0)	21 (15-22)	0 (0-0)	0 (0-0)	0 (0-0)	3 (2-6)	0 (0-0)	0 (0-0)	0 (0-0)	3 (1-5)	0 (0-0)
2000	Uganda	6651 (4716-6931)		143 (101-149)		1443 (685-2775)		31 (15-60)		454 (215-873)		10 (5-19)	
2001	Uganda	6527 (4628-6802)		136 (96-142)		1411 (669-2710)		29 (14-56)		444 (210-852)		9 (4-18)	
2002	Uganda	6439 (4566-6710)		130 (92-135)		1386 (665-2648)		28 (13-53)		436 (209-832)		9 (4-17)	
2003	Uganda	6252 (4433-6516)		122 (86-127)		1340 (651-2543)		26 (13-50)		421 (205-799)		8 (4-16)	
2004	Uganda	6410 (4545-6680)		121 (86-126)		1366 (672-2575)		26 (13-49)		430 (211-810)		8 (4-15)	
2005	Uganda	6391 (4532-6660)		117 (83-121)		1354 (673-2532)		25 (12-46)		426 (212-796)		8 (4-15)	
2006	Uganda	6086 (4315-6342)		107 (76-112)		1280 (644-2375)		23 (11-42)		403 (202-747)		7 (4-13)	
2007	Uganda	5859 (4155-6106)		100 (71-104)		1223 (621-2250)		21 (11-38)		384 (195-707)		7 (3-12)	
2008	Uganda	5743 (4072-5985)		96 (68-100)		1188 (611-2170)		20 (10-36)		373 (192-682)		6 (3-11)	
2009	Uganda	5521 (3915-5754)		89 (63-93)		1134 (590-2058)		18 (10-33)		356 (186-647)		6 (3-10)	
2010	Uganda	5441 (3858-5670)		86 (61-89)		1109 (586-2004)		18 (9-32)		349 (184-630)		6 (3-10)	
2011	Uganda	5401 (3830-5629)		83 (59-87)		1095 (534-2020)		17 (8-31)		975 (476-1798)		15 (7-28)	
2012	Uganda	5371 (3809-5598)		81 (57-84)		1085 (539-1994)		16 (8-30)		966 (480-1775)		14 (7-27)	
2013	Uganda	5268 (3736-5490)		77 (55-80)		1061 (533-1943)		16 (8-28)		945 (475-1730)		14 (7-25)	
2014	Uganda	4256 (3018-4436)		61 (43-63)		833 (423-1520)		12 (6-22)		741 (376-1353)		11 (5-19)	
2015	Uganda	3626 (2571-3779)	757 (537-789)	51 (36-53)	11 (8-11)	701 (358-1276)	146 (75-267)	10 (5-18)	2 (1-4)	624 (319-1136)	130 (67-237)	9 (4-16)	2 (1-3)
2000	Ukraine	235 (167-245)		11 (8-12)		22 (9-62)		1 (0-3)		19 (8-55)		1 (0-3)	
2001	Ukraine	207 (147-216)		10 (7-11)		19 (8-55)		1 (0-3)		17 (7-49)		1 (0-2)	
2002	Ukraine	180 (128-187)		9 (6-10)		17 (7-48)		1 (0-2)		15 (6-42)		1 (0-2)	
2003	Ukraine	160 (114-167)		8 (6-9)		15 (6-43)		1 (0-2)		14 (5-38)		1 (0-2)	
2004	Ukraine	144 (102-150)		7 (5-8)		14 (6-39)		1 (0-2)		12 (5-35)		1 (0-2)	
2005	Ukraine	136 (97-142)		7 (5-7)		13 (5-37)		1 (0-2)		12 (5-33)		1 (0-2)	
2006	Ukraine	131 (93-137)		7 (5-7)		15 (6-43)		1 (0-2)		14 (6-38)		1 (0-2)	

Spn = *Streptococcus pneumoniae*; npnm = non-pneumonia, non-meningitis; HIV = deaths only in HIV-infected children; Uncertainty range provided in parentheses.

Year	Country	Spn pneumonia deaths (UR)	Spn pneumonia deaths, HIV (UR)	Spn pneumonia mortality rate (UR)	Spn pneumonia mortality rate, HIV (UR)	Spn meningitis deaths (UR)	Spn meningitis deaths, HIV (UR)	Spn meningitis mortality rate (UR)	Spn meningitis mortality rate, HIV (UR)	Spn NPNM deaths (UR)	Spn NPNM deaths, HIV (UR)	Spn NPNM mortality rate (UR)	Spn NPNM mortality rate, HIV (UR)
2007	Ukraine	153 (108-159)		7 (5-8)		22 (9-60)		1 (0-3)		19 (8-54)		1 (0-3)	
2008	Ukraine	160 (114-167)		7 (5-8)		24 (10-66)		1 (0-3)		21 (9-59)		1 (0-3)	
2009	Ukraine	152 (108-158)		7 (5-7)		22 (9-60)		1 (0-3)		20 (8-54)		1 (0-2)	
2010	Ukraine	147 (104-153)		6 (4-7)		21 (9-56)		1 (0-2)		19 (8-50)		1 (0-2)	
2011	Ukraine	129 (91-134)		5 (4-5)		16 (7-44)		1 (0-2)		15 (6-39)		1 (0-2)	
2012	Ukraine	129 (92-135)		5 (4-5)		19 (8-51)		1 (0-2)		17 (7-45)		1 (0-2)	
2013	Ukraine	113 (80-118)		5 (3-5)		16 (7-44)		1 (0-2)		15 (6-39)		1 (0-2)	
2014	Ukraine	98 (69-102)		4 (3-4)		14 (6-36)		1 (0-1)		12 (5-32)		0 (0-1)	
2015	Ukraine	85 (60-89)	3 (2-3)	4 (2-4)	0 (0-0)	12 (5-31)	0 (0-1)	0 (0-1)	0 (0-0)	10 (4-27)	0 (0-1)	0 (0-1)	0 (0-0)
2000	United Arab Emirates	12 (9-13)		5 (3-5)		3 (2-4)		1 (1-2)		3 (2-4)		1 (1-1)	
2001	United Arab Emirates	12 (8-12)		5 (3-5)		3 (2-4)		1 (1-2)		2 (1-3)		1 (1-1)	
2002	United Arab Emirates	11 (8-11)		4 (3-5)		3 (2-4)		1 (1-1)		2 (1-3)		1 (1-1)	
2003	United Arab Emirates	10 (7-11)		4 (3-4)		2 (1-3)		1 (1-1)		2 (1-3)		1 (1-1)	
2004	United Arab Emirates	11 (7-11)		4 (3-4)		3 (2-4)		1 (1-1)		2 (1-3)		1 (1-1)	
2005	United Arab Emirates	11 (8-11)		4 (3-4)		3 (2-4)		1 (1-1)		2 (1-3)		1 (1-1)	
2006	United Arab Emirates	11 (8-12)		4 (3-4)		3 (2-4)		1 (1-1)		2 (1-3)		1 (0-1)	
2007	United Arab Emirates	12 (9-13)		4 (3-4)		3 (2-4)		1 (1-1)		3 (2-4)		1 (0-1)	
2008	United Arab Emirates	13 (9-14)		4 (3-4)		3 (2-4)		1 (0-1)		3 (2-4)		1 (0-1)	
2009	United Arab Emirates	13 (10-14)		3 (2-4)		3 (2-4)		1 (0-1)		3 (2-4)		1 (0-1)	
2010	United Arab Emirates	10 (7-11)		2 (2-3)		2 (1-3)		1 (0-1)		2 (1-3)		0 (0-1)	
2011	United Arab Emirates	8 (6-9)		2 (1-2)		2 (1-3)		0 (0-1)		2 (1-2)		0 (0-1)	
2012	United Arab Emirates	7 (5-7)		2 (1-2)		2 (1-2)		0 (0-0)		1 (1-2)		0 (0-0)	
2013	United Arab Emirates	7 (5-7)		1 (1-1)		2 (1-2)		0 (0-0)		1 (1-2)		0 (0-0)	
2014	United Arab Emirates	5 (3-5)		1 (1-1)		1 (1-1)		0 (0-0)		1 (1-1)		0 (0-0)	
2015	United Arab Emirates	4 (3-4)	0 (0-0)	1 (1-1)	0 (0-0)	1 (1-1)	0 (0-0)	0 (0-0)	0 (0-0)	1 (0-1)	0 (0-0)	0 (0-0)	0 (0-0)
2000	United Kingdom	66 (47-69)		2 (1-2)		49 (22-88)		1 (1-3)		43 (20-78)		1 (1-2)	
2001	United Kingdom	65 (46-68)		2 (1-2)		54 (24-98)		2 (1-3)		48 (22-87)		1 (1-3)	
2002	United Kingdom	52 (37-55)		2 (1-2)		43 (19-78)		1 (1-2)		38 (17-69)		1 (1-2)	
2003	United Kingdom	51 (36-53)		2 (1-2)		49 (22-88)		1 (1-3)		43 (20-78)		1 (1-2)	
2004	United Kingdom	53 (38-55)		2 (1-2)		42 (19-76)		1 (1-2)		37 (17-67)		1 (1-2)	
2005	United Kingdom	57 (41-60)		2 (1-2)		42 (19-75)		1 (1-2)		37 (17-67)		1 (0-2)	
2006	United Kingdom	54 (38-56)		2 (1-2)		30 (13-54)		1 (0-2)		26 (12-48)		1 (0-1)	
2007	United Kingdom	43 (31-45)		1 (1-1)		25 (11-45)		1 (0-1)		22 (10-40)		1 (0-1)	
2008	United Kingdom	43 (31-45)		1 (1-1)		20 (9-36)		1 (0-1)		18 (8-32)		0 (0-1)	
2009	United Kingdom	44 (31-45)		1 (1-1)		22 (10-39)		1 (0-1)		19 (9-35)		1 (0-1)	
2010	United Kingdom	31 (22-32)		1 (1-1)		16 (7-29)		0 (0-1)		14 (7-26)		0 (0-1)	
2011	United Kingdom	21 (15-22)		1 (0-1)		8 (4-15)		0 (0-0)		7 (3-13)		0 (0-0)	
2012	United Kingdom	14 (10-14)		0 (0-0)		5 (2-9)		0 (0-0)		5 (2-8)		0 (0-0)	
2013	United Kingdom	9 (7-10)		0 (0-0)		3 (1-5)		0 (0-0)		3 (1-5)		0 (0-0)	
2014	United Kingdom	11 (8-11)		0 (0-0)		4 (2-7)		0 (0-0)		3 (2-6)		0 (0-0)	
2015	United Kingdom	10 (7-11)	0 (0-0)	0 (0-0)	0 (0-0)	4 (2-6)	0 (0-0)	0 (0-0)	0 (0-0)	3 (1-6)	0 (0-0)	0 (0-0)	0 (0-0)
2000	United Republic of Tanzania	9438 (6692-9836)		162 (115-169)		1908 (806-3927)		33 (14-68)		600 (253-1235)		10 (4-21)	
2001	United Republic of Tanzania	9111 (6461-9495)		152 (108-159)		1838 (786-3766)		31 (13-63)		578 (247-1184)		10 (4-20)	
2002	United Republic of Tanzania	8701 (6170-9068)		141 (100-147)		1750 (757-3568)		28 (12-58)		550 (238-1122)		9 (4-18)	
2003	United Republic of Tanzania	8774 (6222-9144)		137 (97-143)		1756 (770-3561)		27 (12-56)		552 (242-1119)		9 (4-17)	
2004	United Republic of Tanzania	8545 (6060-8906)		128 (91-134)		1697 (753-3418)		26 (11-51)		534 (237-1075)		8 (4-16)	
2005	United Republic of Tanzania	8284 (5874-8634)		120 (85-125)		1628 (720-3270)		24 (10-47)		512 (226-1028)		7 (3-15)	
2006	United Republic of Tanzania	7985 (5662-8322)		112 (79-116)		1553 (703-3093)		22 (10-43)		488 (221-972)		7 (3-14)	
2007	United Republic of Tanzania	7516 (5330-7833)		102 (72-106)		1447 (671-2858)		20 (9-39)		455 (211-898)		6 (3-12)	
2008	United Republic of Tanzania	7122 (5050-7422)		94 (67-98)		1359 (565-2736)		18 (7-36)		1210 (503-2436)		16 (7-32)	
2009	United Republic of Tanzania	6813 (4831-7100)		88 (62-91)		1292 (554-2576)		17 (7-33)		1150 (493-2293)		15 (6-29)	
2010	United Republic of Tanzania	6586 (4670-6863)		82 (58-86)		1243 (551-2453)		16 (7-31)		1107 (490-2184)		14 (6-27)	
2011	United Republic of Tanzania	6369 (4516-6637)		77 (55-81)		1198 (538-2348)		15 (7-29)		1067 (479-2091)		13 (6-25)	
2012	United Republic of Tanzania	6297 (4465-6562)		74 (53-77)		1182 (535-2302)		14 (6-27)		1052 (476-2050)		12 (6-24)	
2013	United Republic of Tanzania	4423 (3136-4610)		51 (36-53)		782 (356-1517)		9 (4-17)		696 (317-1350)		8 (4-15)	
2014	United Republic of Tanzania	3466 (2458-3612)		38 (27-40)		601 (276-1160)		7 (3-13)		535 (246-1033)		6 (3-11)	
2015	United Republic of Tanzania	2942 (2086-3066)	285 (202-297)	32 (23-33)	3 (2-3)	530 (246-1021)	51 (24-99)	6 (3-11)	1 (0-1)	472 (219-909)	46 (21-88)	5 (2-10)	0 (0-1)
2000	United States of America	375 (266-391)		2 (1-2)		174 (55-314)		1 (0-2)		155 (49-280)		1 (0-1)	
2001	United States of America	385 (273-401)		2 (1-2)		140 (44-252)		1 (0-1)		124 (40-225)		1 (0-1)	
2002	United States of America	339 (240-353)		2 (1-2)		114 (36-207)		1 (0-1)		102 (32-184)		1 (0-1)	
2003	United States of America	338 (240-352)		2 (1-2)		97 (31-175)		1 (0-1)		86 (27-156)		0 (0-1)	
2004	United States of America	285 (202-297)		1 (1-2)		86 (27-156)		0 (0-1)		77 (24-139)		0 (0-1)	
2005	United States of America	227 (161-237)		1 (1-1)		79 (25-143)		0 (0-1)		70 (22-127)		0 (0-1)	
2006	United States of America	266 (188-277)		1 (1-1)		81 (26-146)		0 (0-1)		72 (23-130)		0 (0-1)	
2007	United States of America	224 (159-233)		1 (1-1)		74 (24-134)		0 (0-1)		66 (21-119)		0 (0-1)	
2008	United States of America	246 (174-256)		1 (1-1)		67 (21-122)		0 (0-1)		60 (19-108)		0 (0-1)	
2009	United States of America	268 (190-279)		1 (1-1)		68 (22-123)		0 (0-1)		61 (19-110)		0 (0-1)	
2010	United States of America	192 (136-200)		1 (1-1)		77 (24-139)		0 (0-1)		68 (22-123)		0 (0-1)	
2011	United States of America	133 (94-139)		1 (0-1)		27 (9-49)		0 (0-0)		24 (8-43)		0 (0-0)	
2012	United States of America	81 (57-84)		0 (0-0)		17 (6-32)		0 (0-0)		16 (5-28)		0 (0-0)	
2013	United States of America	65 (46-67)		0 (0-0)		21 (7-38)		0 (0-0)		19 (6-34)		0 (0-0)	
2014	United States of America	63 (45-66)		0 (0-0)		16 (5-29)		0 (0-0)		14 (5-26)		0 (0-0)	
2015	United States of America	61 (43-64)	0 (0-0)	0 (0-0)	0 (0-0)	15 (5-27)	0 (0-0)	0 (0-0)	0 (0-0)	13 (4-24)	0 (0-0)	0 (0-0)	0 (0-0)
2000	Uruguay	29 (20-30)		11 (8-11)		6 (3-10)		2 (1-4)		6 (3-9)		2 (1-3)	
2001	Uruguay	35 (25-37)		13 (10-14)		12 (6-19)		5 (2-7)		11 (5-17)		4 (2-6)	
2002	Uruguay	32 (23-33)		12 (9-13)		10 (5-15)		4 (2-6)		9 (4-13)		3 (2-5)	
2003	Uruguay	31 (22-32)		12 (9-13)		10 (5-15)		4 (2-6)		9 (4-13)		3 (2-5)	
2004	Uruguay	23 (16-24)		9 (6-9)		9 (4-14)		3 (2-5)		8 (4-12)		3 (2-5)	
2005	Uruguay	29 (20-30)		11 (8-12)		10 (5-15)		4 (2-6)		9 (4-13)		3 (2-5)	
2006	Uruguay	27 (19-28)		11 (8-11)		9 (4-13)		3 (2-5)		8 (4-12)		3 (1-5)	
2007	Uruguay	31 (22-33)		13 (9-13)		13 (6-20)		5 (3-8)		12 (6-18)		5 (2-7)	
2008	Uruguay	28 (20-30)		12 (8-12)		8 (4-12)		3 (2-5)		7 (3-11)		3 (1-4)	
2009	Uruguay	27 (19-28)		11 (8-12)		9 (5-15)		4 (2-6)		8 (4-13)		3 (2-5)	
2010	Uruguay	15 (10-15)		6 (4-6)		5 (2-7)		2 (1-3)		4 (2-7)		2 (1-3)	
2011	Uruguay	10 (7-11)		4 (3-5)		3 (2-5)		1 (1-2)		3 (1-5)		1 (1-2)	
2012	Uruguay	9 (6-9)		4 (3-4)		2 (1-3)		1 (0-1)		2 (1-3)		1 (0-1)	
2013	Uruguay	5 (4-5)		2 (1-2)		2 (1-4)		1 (1-2)		2 (1-3)		1 (0-1)	
2014	Uruguay	5 (3-5)		2 (1-2)		2 (1-2)		1 (0-1)		1 (1-2)		1 (0-1)	

Spn = *Streptococcus pneumoniae*; npnm = non-pneumonia, non-meningitis; HIV = deaths only in HIV-infected children; Uncertainty range provided in parentheses.

Year	Country	Spn pneumonia deaths (UR)	Spn pneumonia deaths, HIV (UR)	Spn pneumonia mortality rate (UR)	Spn pneumonia mortality rate, HIV (UR)	Spn meningitis deaths (UR)	Spn meningitis deaths, HIV (UR)	Spn meningitis mortality rate (UR)	Spn meningitis mortality rate, HIV (UR)	Spn NPNM deaths (UR)	Spn NPNM deaths, HIV (UR)	Spn NPNM mortality rate (UR)	Spn NPNM mortality rate, HIV (UR)
2015	Uruguay	4 (3-4)	0 (0-0)	2 (1-2)	0 (0-0)	1 (1-2)	0 (0-0)	1 (0-1)	0 (0-0)	1 (1-2)	0 (0-0)	0 (0-1)	0 (0-0)
2000	Uzbekistan	1912 (1356-1993)		71 (50-74)		299 (116-629)		11 (4-23)		266 (103-560)		10 (4-21)	
2001	Uzbekistan	1807 (1281-1883)		69 (49-72)		281 (109-589)		11 (4-22)		250 (97-525)		10 (4-20)	
2002	Uzbekistan	1713 (1215-1785)		67 (47-70)		264 (103-552)		10 (4-22)		235 (92-492)		9 (4-19)	
2003	Uzbekistan	1627 (1154-1695)		65 (46-67)		248 (98-517)		10 (4-21)		220 (87-461)		9 (3-18)	
2004	Uzbekistan	1573 (1115-1639)		63 (45-66)		236 (94-491)		9 (4-20)		210 (83-437)		8 (3-18)	
2005	Uzbekistan	1527 (1083-1592)		61 (43-64)		225 (90-467)		9 (4-19)		200 (80-416)		8 (3-17)	
2006	Uzbekistan	1509 (1070-1572)		60 (42-62)		218 (87-451)		9 (3-18)		194 (78-401)		8 (3-16)	
2007	Uzbekistan	1500 (1064-1563)		58 (41-61)		212 (85-438)		8 (3-17)		189 (76-390)		7 (3-15)	
2008	Uzbekistan	1510 (1071-1574)		57 (40-59)		209 (85-431)		8 (3-16)		186 (76-383)		7 (3-14)	
2009	Uzbekistan	1514 (1073-1578)		55 (39-58)		206 (84-423)		8 (3-15)		183 (75-377)		7 (3-14)	
2010	Uzbekistan	1510 (1071-1574)		54 (38-56)		202 (83-415)		7 (3-15)		180 (74-369)		6 (3-13)	
2011	Uzbekistan	1489 (1056-1552)		51 (36-53)		197 (81-402)		7 (3-14)		175 (72-358)		6 (2-12)	
2012	Uzbekistan	1457 (1033-1518)		48 (34-50)		190 (79-388)		6 (3-13)		170 (70-345)		6 (2-11)	
2013	Uzbekistan	1415 (1004-1475)		46 (33-48)		183 (76-372)		6 (2-12)		163 (68-331)		5 (2-11)	
2014	Uzbekistan	1374 (974-1432)		44 (31-46)		177 (74-358)		6 (2-11)		157 (66-319)		5 (2-10)	
2015	Uzbekistan	1309 (928-1364)	6 (4-6)	42 (30-43)	0 (0-0)	168 (71-339)	1 (0-1)	5 (2-11)	0 (0-0)	149 (63-302)	1 (0-1)	5 (2-10)	0 (0-0)
2000	Vanuatu	7 (5-8)		27 (19-28)		0 (0-1)		1 (0-5)		0 (0-1)		1 (0-5)	
2001	Vanuatu	7 (5-8)		26 (19-27)		0 (0-1)		1 (0-5)		0 (0-1)		1 (0-4)	
2002	Vanuatu	6 (4-6)		21 (15-21)		0 (0-1)		1 (0-4)		0 (0-1)		1 (0-3)	
2003	Vanuatu	5 (3-5)		17 (12-18)		0 (0-1)		1 (0-3)		0 (0-1)		1 (0-3)	
2004	Vanuatu	5 (4-5)		18 (13-19)		0 (0-1)		1 (0-3)		0 (0-1)		1 (0-3)	
2005	Vanuatu	7 (5-7)		23 (17-24)		0 (0-1)		1 (0-4)		0 (0-1)		1 (0-4)	
2006	Vanuatu	7 (5-8)		25 (18-26)		0 (0-1)		1 (0-5)		0 (0-1)		1 (0-4)	
2007	Vanuatu	8 (6-8)		26 (18-27)		0 (0-1)		1 (0-5)		0 (0-1)		1 (0-4)	
2008	Vanuatu	8 (6-9)		26 (18-27)		0 (0-2)		1 (0-5)		0 (0-1)		1 (0-4)	
2009	Vanuatu	9 (6-9)		26 (19-27)		0 (0-2)		1 (0-5)		0 (0-1)		1 (0-4)	
2010	Vanuatu	9 (6-9)		26 (19-27)		0 (0-2)		1 (0-5)		0 (0-1)		1 (0-4)	
2011	Vanuatu	9 (7-10)		27 (19-28)		1 (0-3)		2 (1-9)		1 (0-3)		2 (0-8)	
2012	Vanuatu	10 (7-10)		28 (20-29)		1 (0-4)		3 (1-12)		1 (0-4)		2 (1-10)	
2013	Vanuatu	9 (7-10)		27 (19-28)		1 (0-4)		3 (1-11)		1 (0-3)		2 (1-10)	
2014	Vanuatu	9 (6-9)		26 (19-27)		1 (0-4)		2 (1-11)		1 (0-3)		2 (1-9)	
2015	Vanuatu	9 (6-9)	0 (0-0)	25 (18-26)	0 (0-0)	1 (0-3)	0 (0-0)	2 (1-10)	0 (0-0)	1 (0-3)	0 (0-0)	2 (1-9)	0 (0-0)
2000	Venezuela (Bolivarian Republic of)	277 (197-289)		10 (7-10)		59 (15-172)		2 (1-6)		53 (13-153)		2 (0-6)	
2001	Venezuela (Bolivarian Republic of)	362 (256-377)		13 (9-14)		66 (17-193)		2 (1-7)		59 (15-172)		2 (1-6)	
2002	Venezuela (Bolivarian Republic of)	313 (222-327)		11 (8-12)		67 (18-195)		2 (1-7)		60 (16-174)		2 (1-6)	
2003	Venezuela (Bolivarian Republic of)	367 (260-382)		13 (9-14)		47 (12-136)		2 (0-5)		42 (11-121)		1 (0-4)	
2004	Venezuela (Bolivarian Republic of)	382 (271-399)		14 (10-14)		70 (18-202)		2 (1-7)		62 (16-180)		2 (1-6)	
2005	Venezuela (Bolivarian Republic of)	342 (243-357)		12 (9-13)		62 (16-178)		2 (1-6)		55 (15-158)		2 (1-6)	
2006	Venezuela (Bolivarian Republic of)	346 (245-360)		12 (9-13)		56 (15-162)		2 (1-6)		50 (13-144)		2 (0-5)	
2007	Venezuela (Bolivarian Republic of)	317 (225-330)		11 (8-12)		50 (13-143)		2 (0-5)		44 (12-127)		2 (0-4)	
2008	Venezuela (Bolivarian Republic of)	298 (212-311)		10 (7-11)		52 (14-148)		2 (0-5)		46 (12-131)		2 (0-5)	
2009	Venezuela (Bolivarian Republic of)	282 (200-294)		10 (7-10)		47 (13-135)		2 (0-5)		42 (11-120)		1 (0-4)	
2010	Venezuela (Bolivarian Republic of)	272 (193-284)		9 (7-10)		45 (12-128)		2 (0-4)		40 (11-114)		1 (0-4)	
2011	Venezuela (Bolivarian Republic of)	287 (203-299)		10 (7-10)		57 (15-162)		2 (1-6)		51 (14-144)		2 (0-5)	
2012	Venezuela (Bolivarian Republic of)	273 (193-284)		9 (7-10)		51 (14-146)		2 (0-5)		46 (12-130)		2 (0-4)	
2013	Venezuela (Bolivarian Republic of)	265 (188-277)		9 (6-10)		49 (13-138)		2 (0-5)		44 (12-123)		1 (0-4)	
2014	Venezuela (Bolivarian Republic of)	262 (185-273)		9 (6-9)		48 (13-136)		2 (0-5)		43 (12-121)		1 (0-4)	
2015	Venezuela (Bolivarian Republic of)	239 (170-249)	3 (2-3)	8 (6-9)	0 (0-0)	43 (12-123)	1 (0-2)	1 (0-4)	0 (0-0)	39 (11-109)	1 (0-2)	1 (0-4)	0 (0-0)
2000	Viet Nam	2253 (1597-2348)		32 (22-33)		203 (73-434)		3 (1-6)		181 (65-386)		3 (1-5)	
2001	Viet Nam	2090 (1482-2178)		32 (22-33)		192 (74-401)		3 (1-6)		171 (66-357)		3 (1-5)	
2002	Viet Nam	2059 (1460-2146)		32 (23-33)		193 (81-393)		3 (1-6)		172 (72-350)		3 (1-5)	
2003	Viet Nam	2063 (1463-2150)		32 (23-33)		198 (86-393)		3 (1-6)		176 (76-350)		3 (1-5)	
2004	Viet Nam	2033 (1442-2119)		31 (22-32)		200 (72-466)		3 (1-7)		178 (64-415)		3 (1-6)	
2005	Viet Nam	1970 (1397-2053)		30 (21-31)		198 (77-450)		3 (1-7)		177 (69-401)		3 (1-6)	
2006	Viet Nam	1885 (1337-1965)		27 (19-29)		193 (82-429)		3 (1-6)		172 (73-382)		3 (1-6)	
2007	Viet Nam	1768 (1254-1843)		25 (18-26)		182 (72-409)		3 (1-6)		162 (64-364)		2 (1-5)	
2008	Viet Nam	1631 (1157-1700)		23 (16-24)		168 (63-382)		2 (1-5)		150 (56-340)		2 (1-5)	
2009	Viet Nam	1515 (1075-1579)		21 (15-22)		155 (55-359)		2 (1-5)		138 (49-319)		2 (1-5)	
2010	Viet Nam	1528 (1084-1593)		21 (15-22)		242 (83-567)		3 (1-8)		216 (74-505)		3 (1-7)	
2011	Viet Nam	1552 (1101-1618)		21 (15-22)		282 (91-664)		4 (1-9)		251 (81-591)		3 (1-8)	
2012	Viet Nam	1499 (1063-1562)		20 (14-21)		272 (94-633)		4 (1-9)		242 (84-564)		3 (1-8)	
2013	Viet Nam	1469 (1042-1531)		20 (14-20)		250 (93-572)		3 (1-8)		223 (82-509)		3 (1-7)	
2014	Viet Nam	1462 (1037-1524)		19 (14-20)		254 (101-568)		3 (1-8)		226 (90-506)		3 (1-7)	
2015	Viet Nam	1430 (1014-1490)	18 (13-18)	19 (13-20)	0 (0-0)	253 (109-554)	3 (1-7)	3 (1-7)	0 (0-0)	225 (97-493)	3 (1-6)	3 (1-6)	0 (0-0)
2000	Yemen	3924 (2782-4089)		127 (90-133)		514 (199-1117)		17 (6-36)		161 (62-351)		5 (2-11)	
2001	Yemen	3719 (2637-3876)		121 (86-126)		484 (185-1056)		16 (6-34)		152 (58-332)		5 (2-11)	
2002	Yemen	3547 (2515-3697)		115 (81-119)		458 (174-1005)		15 (6-32)		144 (55-316)		5 (2-10)	
2003	Yemen	3412 (2419-3555)		108 (77-113)		438 (165-966)		14 (5-31)		138 (52-304)		4 (2-10)	
2004	Yemen	3277 (2324-3415)		102 (72-106)		418 (156-928)		13 (5-29)		131 (49-292)		4 (2-9)	
2005	Yemen	3136 (2224-3268)		95 (68-99)		397 (123-914)		12 (4-28)		354 (110-814)		11 (3-25)	
2006	Yemen	2986 (2117-3112)		88 (63-92)		375 (115-869)		11 (3-26)		334 (103-773)		10 (3-23)	
2007	Yemen	2835 (2010-2954)		82 (58-86)		354 (107-824)		10 (3-24)		315 (96-733)		9 (3-21)	
2008	Yemen	2667 (1891-2779)		76 (54-79)		332 (99-777)		9 (3-22)		295 (88-691)		8 (3-20)	
2009	Yemen	2511 (1781-2617)		71 (50-74)		313 (92-735)		9 (3-21)		278 (82-655)		8 (2-18)	
2010	Yemen	2349 (1666-2448)		65 (46-68)		294 (86-695)		8 (2-19)		262 (76-618)		7 (2-17)	
2011	Yemen	1780 (1262-1855)		48 (34-50)		218 (63-518)		6 (2-14)		194 (56-461)		5 (2-13)	
2012	Yemen	1345 (954-1401)		36 (26-37)		165 (47-393)		4 (1-11)		147 (42-350)		4 (1-9)	
2013	Yemen	1160 (823-1209)		31 (22-32)		146 (41-350)		4 (1-9)		130 (37-312)		3 (1-8)	
2014	Yemen	1075 (762-1120)		28 (20-29)		137 (38-330)		4 (1-9)		122 (34-294)		3 (1-8)	
2015	Yemen	641 (454-668)	1 (1-1)	17 (12-17)	0 (0-0)	76 (21-182)	0 (0-0)	2 (1-5)	0 (0-0)	67 (18-162)	0 (0-0)	2 (0-4)	0 (0-0)
2000	Zambia	3828 (2714-3989)		203 (144-212)		676 (414-1124)		36 (22-60)		212 (130-353)		11 (7-19)	
2001	Zambia	3720 (2638-3877)		191 (135-199)		648 (403-1082)		33 (21-56)		204 (127-340)		10 (6-17)	
2002	Zambia	3543 (2512-3692)		177 (125-184)		607 (290-1140)		30 (14-57)		191 (91-358)		10 (5-18)	
2003	Zambia	3356 (2380-3498)		163 (115-170)		567 (272-1072)		27 (13-52)		178 (85-337)		9 (4-16)	
2004	Zambia	3209 (2275-3344)		151 (107-158)		535 (257-1017)		25 (12-48)		168 (81-320)		8 (4-15)	
2005	Zambia	3101 (2199-3231)		142 (101-148)		511 (246-976)		23 (11-45)		161 (77-307)		7 (4-14)	
2006	Zambia	3029 (2148-3157)		134 (95-140)		493 (237-943)		22 (11-42)		155 (75-297)		7 (3-13)	

Spn = *Streptococcus pneumoniae*; npnm = non-pneumonia, non-meningitis; HIV = deaths only in HIV-infected children; Uncertainty range provided in parentheses.

Year	Country	Spn pneumonia deaths (UR)	Spn pneumonia deaths, HIV (UR)	Spn pneumonia mortality rate (UR)	Spn pneumonia mortality rate, HIV (UR)	Spn meningitis deaths (UR)	Spn meningitis deaths, HIV (UR)	Spn meningitis mortality rate (UR)	Spn meningitis mortality rate, HIV (UR)	Spn NPNM deaths (UR)	Spn NPNM deaths, HIV (UR)	Spn NPNM mortality rate (UR)	Spn NPNM mortality rate, HIV (UR)
2007	Zambia	2988 (2119-3114)		129 (91-134)		480 (230-919)		21 (10-40)		151 (72-289)		7 (3-12)	
2008	Zambia	2966 (2103-3091)		124 (88-130)		469 (226-897)		20 (9-38)		148 (71-282)		6 (3-12)	
2009	Zambia	2921 (2072-3044)		119 (85-124)		454 (219-866)		19 (9-35)		143 (69-272)		6 (3-11)	
2010	Zambia	2863 (2030-2984)		114 (81-119)		436 (211-833)		17 (8-33)		137 (66-262)		5 (3-10)	
2011	Zambia	2786 (1975-2903)		108 (77-113)		416 (203-797)		16 (8-31)		131 (64-251)		5 (2-10)	
2012	Zambia	2672 (1895-2785)		101 (72-106)		394 (171-777)		15 (6-29)		351 (152-692)		13 (6-26)	
2013	Zambia	2551 (1809-2659)		95 (67-99)		374 (163-738)		14 (6-27)		333 (145-657)		12 (5-24)	
2014	Zambia	1834 (1300-1911)		67 (47-70)		256 (112-506)		9 (4-18)		228 (99-451)		8 (4-16)	
2015	Zambia	1509 (1070-1572)	358 (254-373)	54 (38-56)	13 (9-13)	209 (92-413)	50 (22-98)	7 (3-15)	2 (1-3)	186 (82-368)	44 (19-87)	7 (3-13)	2 (1-3)
2000	Zimbabwe	1604 (1137-1671)		89 (63-92)		240 (82-549)		13 (5-30)		75 (26-173)		4 (1-10)	
2001	Zimbabwe	1655 (1174-1725)		91 (65-95)		251 (85-574)		14 (5-32)		79 (27-180)		4 (1-10)	
2002	Zimbabwe	1731 (1227-1803)		95 (67-98)		267 (91-611)		15 (5-33)		84 (29-192)		5 (2-10)	
2003	Zimbabwe	1821 (1291-1898)		98 (70-103)		288 (98-658)		16 (5-36)		90 (31-207)		5 (2-11)	
2004	Zimbabwe	1927 (1366-2008)		103 (73-107)		312 (106-713)		17 (6-38)		98 (33-224)		5 (2-12)	
2005	Zimbabwe	2045 (1450-2132)		107 (76-112)		340 (116-777)		18 (6-41)		107 (37-244)		6 (2-13)	
2006	Zimbabwe	2143 (1519-2233)		110 (78-115)		365 (124-831)		19 (6-43)		115 (39-261)		6 (2-13)	
2007	Zimbabwe	2234 (1584-2328)		112 (79-117)		389 (138-872)		19 (7-44)		122 (43-274)		6 (2-14)	
2008	Zimbabwe	2308 (1636-2405)		113 (80-117)		410 (152-904)		20 (7-44)		129 (48-284)		6 (2-14)	
2009	Zimbabwe	2364 (1676-2464)		112 (80-117)		424 (165-919)		20 (8-44)		133 (52-289)		6 (2-14)	
2010	Zimbabwe	2392 (1696-2493)		110 (78-115)		433 (176-920)		20 (8-42)		136 (55-289)		6 (3-13)	
2011	Zimbabwe	2409 (1708-2510)		107 (76-112)		436 (177-923)		19 (8-41)		137 (56-290)		6 (2-13)	
2012	Zimbabwe	2179 (1545-2271)		94 (67-98)		389 (162-813)		17 (7-35)		122 (51-256)		5 (2-11)	
2013	Zimbabwe	1467 (1040-1529)		62 (44-65)		244 (90-521)		10 (4-22)		218 (80-464)		9 (3-20)	
2014	Zimbabwe	1269 (900-1323)		52 (37-55)		214 (82-451)		9 (3-19)		190 (73-402)		8 (3-17)	
2015	Zimbabwe	1168 (828-1217)	337 (239-352)	47 (34-49)	14 (10-14)	206 (80-434)	60 (23-125)	8 (3-18)	2 (1-5)	183 (71-386)	53 (21-112)	7 (3-16)	2 (1-5)

Spn = *Streptococcus pneumoniae*; npnm = non-pneumonia, non-meningitis; HIV = deaths only in HIV-infected children; Uncertainty range provided in parentheses.

Table 8: *Haemophilus influenzae* type b mortality by syndrome, country, and year

Year	Country	Pneumonia deaths: Hib pneumonia deaths (UR)	Hib pneumonia deaths, HIV (UR)	Hib pneumonia mortality rate (UR)	Hib pneumonia mortality rate, HIV (UR)	Meningitis deaths: Hib meningitis deaths (UR)	Hib meningitis deaths, HIV (UR)	Hib meningitis mortality rate (UR)	Hib meningitis mortality rate, HIV (UR)	NPNM deaths: Hib NPNM deaths (UR)	Hib NPNM deaths, HIV (UR)	Hib NPNM mortality rate (UR)	Hib NPNM mortality rate, HIV (UR)
2000	Afghanistan	4007 (2818-5265)		103 (73-136)		1861 (673-2912)		48 (17-75)		8 (3-12)		0 (0-0)	
2001	Afghanistan	4075 (2866-5354)		101 (71-133)		1889 (686-2953)		47 (17-73)		8 (3-12)		0 (0-0)	
2002	Afghanistan	4119 (2897-5412)		98 (69-129)		1890 (690-2951)		45 (16-70)		8 (3-12)		0 (0-0)	
2003	Afghanistan	4153 (2921-5457)		96 (67-126)		1886 (692-2942)		43 (16-68)		8 (3-12)		0 (0-0)	
2004	Afghanistan	4150 (2919-5453)		93 (65-122)		1863 (687-2904)		42 (15-65)		8 (3-12)		0 (0-0)	
2005	Afghanistan	4113 (2893-5404)		90 (63-118)		1823 (676-2839)		40 (15-62)		7 (3-12)		0 (0-0)	
2006	Afghanistan	4089 (2876-5373)		87 (61-114)		1788 (666-2782)		38 (14-59)		7 (3-11)		0 (0-0)	
2007	Afghanistan	4041 (2842-5309)		84 (59-111)		1736 (650-2698)		36 (14-56)		7 (3-11)		0 (0-0)	
2008	Afghanistan	3977 (2797-5225)		82 (58-108)		1676 (630-2603)		35 (13-54)		7 (3-11)		0 (0-0)	
2009	Afghanistan	1455 (1024-1912)		30 (21-39)		600 (227-931)		12 (5-19)		2 (1-4)		0 (0-0)	
2010	Afghanistan	464 (327-610)		9 (7-12)		188 (71-291)		4 (1-6)		1 (0-1)		0 (0-0)	
2011	Afghanistan	436 (306-572)		9 (6-12)		173 (66-269)		4 (1-5)		1 (0-1)		0 (0-0)	
2012	Afghanistan	424 (299-558)		9 (6-11)		167 (64-259)		3 (1-5)		1 (0-1)		0 (0-0)	
2013	Afghanistan	388 (273-510)		8 (6-10)		152 (58-235)		3 (1-5)		1 (0-1)		0 (0-0)	
2014	Afghanistan	32 (23-42)		1 (0-1)		13 (5-19)		0 (0-0)		0 (0-0)		0 (0-0)	
2015	Afghanistan	31 (22-40)	0 (0-0)	1 (0-1)	0 (0-0)	12 (5-18)	0 (0-0)	0 (0-0)	0 (0-0)	0 (0-0)	0 (0-0)	0 (0-0)	0 (0-0)
2000	Albania	40 (28-52)		15 (10-19)		10 (2-20)		4 (1-7)		0 (0-0)		0 (0-0)	
2001	Albania	34 (24-45)		13 (9-17)		9 (1-18)		3 (0-7)		0 (0-0)		0 (0-0)	
2002	Albania	29 (21-38)		12 (8-15)		7 (1-16)		3 (0-7)		0 (0-0)		0 (0-0)	
2003	Albania	25 (18-33)		11 (8-14)		6 (1-14)		3 (0-6)		0 (0-0)		0 (0-0)	
2004	Albania	22 (16-29)		10 (7-13)		6 (1-13)		3 (0-6)		0 (0-0)		0 (0-0)	
2005	Albania	19 (13-25)		9 (6-12)		5 (0-11)		2 (0-5)		0 (0-0)		0 (0-0)	
2006	Albania	17 (12-22)		8 (6-11)		4 (0-10)		2 (0-5)		0 (0-0)		0 (0-0)	
2007	Albania	15 (10-19)		8 (6-10)		4 (0-8)		2 (0-5)		0 (0-0)		0 (0-0)	
2008	Albania	13 (9-17)		8 (5-10)		3 (0-7)		2 (0-4)		0 (0-0)		0 (0-0)	
2009	Albania	2 (1-3)		1 (1-2)		1 (0-2)		0 (0-1)		0 (0-0)		0 (0-0)	
2010	Albania	1 (1-1)		0 (0-1)		0 (0-1)		0 (0-0)		0 (0-0)		0 (0-0)	
2011	Albania	1 (1-1)		1 (0-1)		0 (0-1)		0 (0-0)		0 (0-0)		0 (0-0)	
2012	Albania	1 (1-1)		1 (0-1)		0 (0-1)		0 (0-0)		0 (0-0)		0 (0-0)	
2013	Albania	1 (1-1)		1 (0-1)		0 (0-1)		0 (0-0)		0 (0-0)		0 (0-0)	
2014	Albania	0 (0-0)		0 (0-0)		0 (0-0)		0 (0-0)		0 (0-0)		0 (0-0)	
2015	Albania	0 (0-0)	0 (0-0)	0 (0-0)	0 (0-0)	0 (0-0)	0 (0-0)	0 (0-0)	0 (0-0)	0 (0-0)	0 (0-0)	0 (0-0)	0 (0-0)
2000	Algeria	542 (382-713)		18 (13-23)		126 (40-208)		4 (1-7)		1 (0-2)		0 (0-0)	
2001	Algeria	521 (366-684)		18 (13-24)		120 (38-198)		4 (1-7)		1 (0-2)		0 (0-0)	
2002	Algeria	500 (352-658)		18 (12-23)		114 (36-188)		4 (1-7)		1 (0-2)		0 (0-0)	
2003	Algeria	485 (341-637)		17 (12-23)		110 (35-180)		4 (1-6)		1 (0-1)		0 (0-0)	
2004	Algeria	474 (334-623)		17 (12-22)		106 (34-175)		4 (1-6)		1 (0-1)		0 (0-0)	
2005	Algeria	467 (328-613)		16 (11-21)		104 (33-171)		4 (1-6)		1 (0-1)		0 (0-0)	
2006	Algeria	473 (333-622)		16 (11-21)		105 (34-173)		3 (1-6)		1 (0-1)		0 (0-0)	
2007	Algeria	478 (336-628)		15 (11-20)		105 (35-173)		3 (1-5)		1 (0-1)		0 (0-0)	
2008	Algeria	76 (54-100)		2 (2-3)		17 (2-29)		0 (0-1)		0 (0-0)		0 (0-0)	
2009	Algeria	36 (25-47)		1 (1-1)		8 (1-14)		0 (0-0)		0 (0-0)		0 (0-0)	
2010	Algeria	36 (26-48)		1 (1-1)		8 (1-14)		0 (0-0)		0 (0-0)		0 (0-0)	
2011	Algeria	36 (25-47)		1 (1-1)		8 (1-14)		0 (0-0)		0 (0-0)		0 (0-0)	
2012	Algeria	36 (26-48)		1 (1-1)		8 (1-14)		0 (0-0)		0 (0-0)		0 (0-0)	
2013	Algeria	5 (4-7)		0 (0-0)		1 (0-2)		0 (0-0)		0 (0-0)		0 (0-0)	
2014	Algeria	5 (4-7)		0 (0-0)		1 (0-2)		0 (0-0)		0 (0-0)		0 (0-0)	
2015	Algeria	5 (3-7)	0 (0-0)	0 (0-0)	0 (0-0)	1 (0-2)	0 (0-0)	0 (0-0)	0 (0-0)	0 (0-0)	0 (0-0)	0 (0-0)	0 (0-0)
2000	Andorra	0 (0-0)		0 (0-0)		0 (0-0)		0 (0-0)		0 (0-0)		0 (0-0)	
2001	Andorra	0 (0-0)		0 (0-0)		0 (0-0)		0 (0-0)		0 (0-0)		0 (0-0)	
2002	Andorra	0 (0-0)		0 (0-0)		0 (0-0)		0 (0-0)		0 (0-0)		0 (0-0)	
2003	Andorra	0 (0-0)		0 (0-0)		0 (0-0)		0 (0-0)		0 (0-0)		0 (0-0)	
2004	Andorra	0 (0-0)		0 (0-0)		0 (0-0)		0 (0-0)		0 (0-0)		0 (0-0)	
2005	Andorra	0 (0-0)		0 (0-0)		0 (0-0)		0 (0-0)		0 (0-0)		0 (0-0)	
2006	Andorra	0 (0-0)		0 (0-0)		0 (0-0)		0 (0-0)		0 (0-0)		0 (0-0)	
2007	Andorra	0 (0-0)		0 (0-0)		0 (0-0)		0 (0-0)		0 (0-0)		0 (0-0)	
2008	Andorra	0 (0-0)		0 (0-0)		0 (0-0)		0 (0-0)		0 (0-0)		0 (0-0)	
2009	Andorra	0 (0-0)		0 (0-0)		0 (0-0)		0 (0-0)		0 (0-0)		0 (0-0)	
2010	Andorra	0 (0-0)		0 (0-0)		0 (0-0)		0 (0-0)		0 (0-0)		0 (0-0)	
2011	Andorra	0 (0-0)		0 (0-0)		0 (0-0)		0 (0-0)		0 (0-0)		0 (0-0)	
2012	Andorra	0 (0-0)		0 (0-0)		0 (0-0)		0 (0-0)		0 (0-0)		0 (0-0)	
2013	Andorra	0 (0-0)		0 (0-0)		0 (0-0)		0 (0-0)		0 (0-0)		0 (0-0)	
2014	Andorra	0 (0-0)		0 (0-0)		0 (0-0)		0 (0-0)		0 (0-0)		0 (0-0)	
2015	Andorra	0 (0-0)	0 (0-0)	0 (0-0)	0 (0-0)	0 (0-0)	0 (0-0)	0 (0-0)	0 (0-0)	0 (0-0)	0 (0-0)	0 (0-0)	0 (0-0)
2000	Angola	4211 (2961-5533)		143 (101-188)		1355 (621-2021)		46 (21-69)		6 (3-8)		0 (0-0)	
2001	Angola	4304 (3027-5655)		141 (99-185)		1370 (628-2042)		45 (21-67)		6 (3-8)		0 (0-0)	
2002	Angola	4524 (3182-5944)		143 (101-188)		1418 (651-2113)		45 (21-67)		6 (3-9)		0 (0-0)	
2003	Angola	4655 (3274-6116)		142 (100-187)		1429 (656-2128)		44 (20-65)		6 (3-9)		0 (0-0)	
2004	Angola	4827 (3395-6342)		143 (100-188)		1441 (663-2146)		43 (20-63)		6 (3-9)		0 (0-0)	
2005	Angola	5029 (3537-6608)		144 (101-189)		1466 (675-2182)		42 (19-62)		6 (3-9)		0 (0-0)	
2006	Angola	5245 (3689-6892)		144 (102-190)		1490 (687-2218)		41 (19-61)		6 (3-9)		0 (0-0)	
2007	Angola	1365 (960-1793)		36 (26-48)		379 (175-564)		10 (5-15)		2 (1-2)		0 (0-0)	
2008	Angola	653 (459-858)		17 (12-22)		179 (83-266)		5 (2-7)		1 (0-1)		0 (0-0)	
2009	Angola	739 (520-971)		19 (13-24)		200 (93-297)		5 (2-7)		1 (0-1)		0 (0-0)	
2010	Angola	588 (413-772)		14 (10-19)		158 (74-235)		4 (2-6)		1 (0-1)		0 (0-0)	
2011	Angola	632 (444-830)		15 (11-20)		170 (79-252)		4 (2-6)		1 (0-1)		0 (0-0)	
2012	Angola	56 (39-73)		1 (1-2)		15 (7-22)		0 (0-1)		0 (0-0)		0 (0-0)	
2013	Angola	55 (39-72)		1 (1-2)		15 (7-22)		0 (0-0)		0 (0-0)		0 (0-0)	
2014	Angola	684 (481-899)		15 (11-20)		182 (86-270)		4 (2-6)		1 (0-1)		0 (0-0)	
2015	Angola	672 (473-883)	16 (11-21)	14 (10-19)	0 (0-0)	178 (84-264)	4 (2-6)	4 (2-6)	0 (0-0)	1 (0-1)	0 (0-0)	0 (0-0)	0 (0-0)
2000	Antigua and Barbuda	0 (0-0)		0 (0-0)		0 (0-0)		1 (1-3)		0 (0-0)		0 (0-0)	
2001	Antigua and Barbuda	0 (0-0)		0 (0-0)		0 (0-0)		1 (0-1)		0 (0-0)		0 (0-0)	
2002	Antigua and Barbuda	0 (0-0)		0 (0-0)		0 (0-0)		0 (0-0)		0 (0-0)		0 (0-0)	
2003	Antigua and Barbuda	0 (0-0)		0 (0-0)		0 (0-0)		0 (0-0)		0 (0-0)		0 (0-0)	
2004	Antigua and Barbuda	0 (0-0)		0 (0-0)		0 (0-0)		0 (0-0)		0 (0-0)		0 (0-0)	
2005	Antigua and Barbuda	0 (0-0)		0 (0-0)		0 (0-0)		0 (0-0)		0 (0-0)		0 (0-0)	
2006	Antigua and Barbuda	0 (0-0)		0 (0-0)		0 (0-0)		0 (0-0)		0 (0-0)		0 (0-0)	

Hib = *Haemophilus influenzae* type b; npnm = non-pneumonia, non-meningitis; HIV = deaths only in HIV-infected children; Uncertainty range provided in parentheses.

Year	Country	Hib pneumonia deaths (UR)	Hib pneumonia deaths, HIV (UR)	Hib pneumonia mortality rate (UR)	Hib pneumonia mortality rate, HIV (UR)	Hib meningitis deaths (UR)	Hib meningitis deaths, HIV (UR)	Hib meningitis mortality rate (UR)	Hib meningitis mortality rate, HIV (UR)	Hib NPNM deaths (UR)	Hib NPNM deaths, HIV (UR)	Hib NPNM mortality rate (UR)	Hib NPNM mortality rate, HIV (UR)
2007	Antigua and Barbuda	0 (0-0)		0 (0-0)		0 (0-0)		0 (0-0)		0 (0-0)		0 (0-0)	
2008	Antigua and Barbuda	0 (0-0)		0 (0-0)		0 (0-0)		0 (0-0)		0 (0-0)		0 (0-0)	
2009	Antigua and Barbuda	0 (0-0)		0 (0-0)		0 (0-0)		0 (0-0)		0 (0-0)		0 (0-0)	
2010	Antigua and Barbuda	0 (0-0)		0 (0-0)		0 (0-0)		0 (0-0)		0 (0-0)		0 (0-0)	
2011	Antigua and Barbuda	0 (0-0)		0 (0-0)		0 (0-0)		0 (0-0)		0 (0-0)		0 (0-0)	
2012	Antigua and Barbuda	0 (0-0)		0 (0-0)		0 (0-0)		0 (0-0)		0 (0-0)		0 (0-0)	
2013	Antigua and Barbuda	0 (0-0)		0 (0-0)		0 (0-0)		0 (0-0)		0 (0-0)		0 (0-0)	
2014	Antigua and Barbuda	0 (0-0)		0 (0-0)		0 (0-0)		0 (0-0)		0 (0-0)		0 (0-0)	
2015	Antigua and Barbuda	0 (0-0)	0 (0-0)	0 (0-0)	0 (0-0)	0 (0-0)	0 (0-0)	0 (0-0)	0 (0-0)	0 (0-0)	0 (0-0)	0 (0-0)	0 (0-0)
2000	Argentina	18 (12-23)		1 (0-1)		9 (3-16)		0 (0-0)		0 (0-0)		0 (0-0)	
2001	Argentina	18 (13-24)		1 (0-1)		8 (3-14)		0 (0-0)		0 (0-0)		0 (0-0)	
2002	Argentina	14 (10-19)		0 (0-1)		7 (2-12)		0 (0-0)		0 (0-0)		0 (0-0)	
2003	Argentina	3 (2-4)		0 (0-0)		1 (0-2)		0 (0-0)		0 (0-0)		0 (0-0)	
2004	Argentina	2 (2-3)		0 (0-0)		1 (0-2)		0 (0-0)		0 (0-0)		0 (0-0)	
2005	Argentina	2 (1-2)		0 (0-0)		1 (0-2)		0 (0-0)		0 (0-0)		0 (0-0)	
2006	Argentina	2 (1-2)		0 (0-0)		1 (0-2)		0 (0-0)		0 (0-0)		0 (0-0)	
2007	Argentina	2 (2-3)		0 (0-0)		1 (0-1)		0 (0-0)		0 (0-0)		0 (0-0)	
2008	Argentina	2 (1-2)		0 (0-0)		1 (0-1)		0 (0-0)		0 (0-0)		0 (0-0)	
2009	Argentina	2 (2-3)		0 (0-0)		1 (0-1)		0 (0-0)		0 (0-0)		0 (0-0)	
2010	Argentina	2 (1-2)		0 (0-0)		1 (0-1)		0 (0-0)		0 (0-0)		0 (0-0)	
2011	Argentina	2 (1-2)		0 (0-0)		1 (0-1)		0 (0-0)		0 (0-0)		0 (0-0)	
2012	Argentina	2 (1-2)		0 (0-0)		1 (0-1)		0 (0-0)		0 (0-0)		0 (0-0)	
2013	Argentina	2 (1-2)		0 (0-0)		1 (0-1)		0 (0-0)		0 (0-0)		0 (0-0)	
2014	Argentina	2 (1-2)		0 (0-0)		1 (0-1)		0 (0-0)		0 (0-0)		0 (0-0)	
2015	Argentina	2 (1-2)	0 (0-0)	0 (0-0)	0 (0-0)	1 (0-1)	0 (0-0)	0 (0-0)	0 (0-0)	0 (0-0)	0 (0-0)	0 (0-0)	0 (0-0)
2000	Armenia	28 (20-37)		14 (10-19)		9 (2-15)		5 (1-8)		0 (0-0)		0 (0-0)	
2001	Armenia	26 (18-35)		14 (10-19)		9 (1-15)		5 (0-8)		0 (0-0)		0 (0-0)	
2002	Armenia	25 (18-33)		14 (10-18)		9 (1-15)		5 (0-8)		0 (0-0)		0 (0-0)	
2003	Armenia	25 (17-32)		13 (9-17)		8 (1-15)		5 (0-8)		0 (0-0)		0 (0-0)	
2004	Armenia	24 (17-31)		12 (9-16)		8 (1-14)		4 (0-8)		0 (0-0)		0 (0-0)	
2005	Armenia	22 (16-29)		11 (8-15)		8 (1-14)		4 (0-7)		0 (0-0)		0 (0-0)	
2006	Armenia	21 (15-27)		10 (7-13)		7 (1-13)		4 (0-6)		0 (0-0)		0 (0-0)	
2007	Armenia	19 (14-25)		9 (7-12)		7 (1-12)		3 (0-6)		0 (0-0)		0 (0-0)	
2008	Armenia	18 (12-23)		8 (6-11)		6 (1-11)		3 (0-5)		0 (0-0)		0 (0-0)	
2009	Armenia	16 (11-21)		8 (5-10)		6 (1-10)		3 (0-5)		0 (0-0)		0 (0-0)	
2010	Armenia	8 (6-11)		4 (3-5)		4 (1-8)		2 (0-4)		0 (0-0)		0 (0-0)	
2011	Armenia	1 (1-1)		1 (0-1)		1 (0-1)		0 (0-1)		0 (0-0)		0 (0-0)	
2012	Armenia	1 (1-1)		0 (0-1)		1 (0-1)		0 (0-1)		0 (0-0)		0 (0-0)	
2013	Armenia	1 (1-1)		0 (0-1)		1 (0-1)		0 (0-0)		0 (0-0)		0 (0-0)	
2014	Armenia	1 (1-1)		0 (0-1)		1 (0-1)		0 (0-0)		0 (0-0)		0 (0-0)	
2015	Armenia	0 (0-0)	0 (0-0)	0 (0-0)	0 (0-0)	0 (0-0)	0 (0-0)	0 (0-0)	0 (0-0)	0 (0-0)	0 (0-0)	0 (0-0)	0 (0-0)
2000	Australia	1 (0-1)		0 (0-0)		1 (1-2)		0 (0-0)		0 (0-0)		0 (0-0)	
2001	Australia	1 (1-1)		0 (0-0)		2 (1-3)		0 (0-0)		0 (0-0)		0 (0-0)	
2002	Australia	0 (0-0)		0 (0-0)		0 (0-0)		0 (0-0)		0 (0-0)		0 (0-0)	
2003	Australia	0 (0-0)		0 (0-0)		0 (0-0)		0 (0-0)		0 (0-0)		0 (0-0)	
2004	Australia	0 (0-0)		0 (0-0)		0 (0-0)		0 (0-0)		0 (0-0)		0 (0-0)	
2005	Australia	0 (0-0)		0 (0-0)		0 (0-0)		0 (0-0)		0 (0-0)		0 (0-0)	
2006	Australia	0 (0-0)		0 (0-0)		0 (0-0)		0 (0-0)		0 (0-0)		0 (0-0)	
2007	Australia	0 (0-0)		0 (0-0)		0 (0-0)		0 (0-0)		0 (0-0)		0 (0-0)	
2008	Australia	0 (0-0)		0 (0-0)		0 (0-0)		0 (0-0)		0 (0-0)		0 (0-0)	
2009	Australia	0 (0-0)		0 (0-0)		0 (0-0)		0 (0-0)		0 (0-0)		0 (0-0)	
2010	Australia	0 (0-0)		0 (0-0)		0 (0-0)		0 (0-0)		0 (0-0)		0 (0-0)	
2011	Australia	0 (0-0)		0 (0-0)		0 (0-0)		0 (0-0)		0 (0-0)		0 (0-0)	
2012	Australia	0 (0-0)		0 (0-0)		0 (0-0)		0 (0-0)		0 (0-0)		0 (0-0)	
2013	Australia	0 (0-0)		0 (0-0)		0 (0-0)		0 (0-0)		0 (0-0)		0 (0-0)	
2014	Australia	0 (0-0)		0 (0-0)		0 (0-0)		0 (0-0)		0 (0-0)		0 (0-0)	
2015	Australia	0 (0-0)	0 (0-0)	0 (0-0)	0 (0-0)	0 (0-0)	0 (0-0)	0 (0-0)	0 (0-0)	0 (0-0)	0 (0-0)	0 (0-0)	0 (0-0)
2000	Austria	0 (0-0)		0 (0-0)		0 (0-0)		0 (0-0)		0 (0-0)		0 (0-0)	
2001	Austria	0 (0-0)		0 (0-0)		0 (0-0)		0 (0-0)		0 (0-0)		0 (0-0)	
2002	Austria	0 (0-0)		0 (0-0)		0 (0-0)		0 (0-0)		0 (0-0)		0 (0-0)	
2003	Austria	0 (0-0)		0 (0-0)		0 (0-0)		0 (0-0)		0 (0-0)		0 (0-0)	
2004	Austria	0 (0-0)		0 (0-0)		0 (0-0)		0 (0-0)		0 (0-0)		0 (0-0)	
2005	Austria	0 (0-0)		0 (0-0)		0 (0-0)		0 (0-0)		0 (0-0)		0 (0-0)	
2006	Austria	0 (0-0)		0 (0-0)		0 (0-0)		0 (0-0)		0 (0-0)		0 (0-0)	
2007	Austria	0 (0-0)		0 (0-0)		0 (0-0)		0 (0-0)		0 (0-0)		0 (0-0)	
2008	Austria	0 (0-0)		0 (0-0)		0 (0-0)		0 (0-0)		0 (0-0)		0 (0-0)	
2009	Austria	0 (0-0)		0 (0-0)		0 (0-0)		0 (0-0)		0 (0-0)		0 (0-0)	
2010	Austria	0 (0-0)		0 (0-0)		0 (0-0)		0 (0-0)		0 (0-0)		0 (0-0)	
2011	Austria	0 (0-0)		0 (0-0)		0 (0-0)		0 (0-0)		0 (0-0)		0 (0-0)	
2012	Austria	0 (0-0)		0 (0-0)		0 (0-0)		0 (0-0)		0 (0-0)		0 (0-0)	
2013	Austria	0 (0-0)		0 (0-0)		0 (0-0)		0 (0-0)		0 (0-0)		0 (0-0)	
2014	Austria	0 (0-0)		0 (0-0)		0 (0-0)		0 (0-0)		0 (0-0)		0 (0-0)	
2015	Austria	0 (0-0)	0 (0-0)	0 (0-0)	0 (0-0)	0 (0-0)	0 (0-0)	0 (0-0)	0 (0-0)	0 (0-0)	0 (0-0)	0 (0-0)	0 (0-0)
2000	Azerbaijan	281 (198-369)		41 (29-54)		86 (24-143)		13 (3-21)		1 (0-1)		0 (0-0)	
2001	Azerbaijan	246 (173-323)		38 (27-50)		74 (21-122)		11 (3-19)		1 (0-1)		0 (0-0)	
2002	Azerbaijan	219 (154-288)		35 (25-46)		64 (18-106)		10 (3-17)		1 (0-1)		0 (0-0)	
2003	Azerbaijan	200 (141-263)		33 (23-43)		57 (16-94)		9 (3-15)		0 (0-1)		0 (0-0)	
2004	Azerbaijan	188 (132-248)		31 (22-41)		51 (15-85)		9 (2-14)		0 (0-1)		0 (0-0)	
2005	Azerbaijan	177 (125-233)		29 (21-39)		46 (13-76)		8 (2-13)		0 (0-1)		0 (0-0)	
2006	Azerbaijan	170 (119-223)		28 (19-36)		42 (12-69)		7 (2-11)		0 (0-1)		0 (0-0)	
2007	Azerbaijan	162 (114-213)		26 (18-34)		39 (11-63)		6 (2-10)		0 (0-1)		0 (0-0)	
2008	Azerbaijan	157 (110-206)		24 (17-32)		36 (11-59)		6 (2-9)		0 (0-0)		0 (0-0)	
2009	Azerbaijan	154 (108-202)		23 (16-30)		35 (10-57)		5 (2-8)		0 (0-0)		0 (0-0)	
2010	Azerbaijan	155 (109-204)		22 (16-29)		34 (10-56)		5 (1-8)		0 (0-0)		0 (0-0)	
2011	Azerbaijan	112 (79-147)		15 (11-20)		25 (7-40)		3 (1-5)		0 (0-0)		0 (0-0)	
2012	Azerbaijan	14 (10-18)		2 (1-2)		3 (1-5)		0 (0-1)		0 (0-0)		0 (0-0)	
2013	Azerbaijan	13 (9-17)		2 (1-2)		3 (1-5)		0 (0-1)		0 (0-0)		0 (0-0)	
2014	Azerbaijan	13 (9-17)		1 (1-2)		3 (1-5)		0 (0-1)		0 (0-0)		0 (0-0)	

Hib = *Haemophilus influenzae* type b; npnm = non-pneumonia, non-meningitis; HIV = deaths only in HIV-infected children; Uncertainty range provided in parentheses.

Year	Country	Hib pneumonia deaths (UR)	Hib pneumonia deaths, HIV (UR)	Hib pneumonia mortality rate (UR)	Hib pneumonia mortality rate, HIV (UR)	Hib meningitis deaths (UR)	Hib meningitis deaths, HIV (UR)	Hib meningitis mortality rate (UR)	Hib meningitis mortality rate, HIV (UR)	Hib NPNM deaths (UR)	Hib NPNM deaths, HIV (UR)	Hib NPNM mortality rate (UR)	Hib NPNM mortality rate, HIV (UR)
2015	Azerbaijan	12 (9-16)	0 (0-0)	1 (1-2)	0 (0-0)	3 (1-4)	0 (0-0)	0 (0-0)	0 (0-0)	0 (0-0)	0 (0-0)	0 (0-0)	0 (0-0)
2000	Bahamas	0 (0-0)		1 (0-1)		0 (0-0)		0 (0-0)		0 (0-0)		0 (0-0)	
2001	Bahamas	0 (0-0)		0 (0-1)		0 (0-0)		0 (0-0)		0 (0-0)		0 (0-0)	
2002	Bahamas	0 (0-0)		0 (0-1)		0 (0-0)		0 (0-0)		0 (0-0)		0 (0-0)	
2003	Bahamas	0 (0-0)		0 (0-0)		0 (0-0)		0 (0-0)		0 (0-0)		0 (0-0)	
2004	Bahamas	0 (0-0)		0 (0-0)		0 (0-0)		0 (0-0)		0 (0-0)		0 (0-0)	
2005	Bahamas	0 (0-0)		0 (0-0)		0 (0-0)		0 (0-0)		0 (0-0)		0 (0-0)	
2006	Bahamas	0 (0-0)		0 (0-0)		0 (0-0)		0 (0-0)		0 (0-0)		0 (0-0)	
2007	Bahamas	0 (0-0)		0 (0-0)		0 (0-0)		0 (0-0)		0 (0-0)		0 (0-0)	
2008	Bahamas	0 (0-0)		0 (0-0)		0 (0-0)		0 (0-0)		0 (0-0)		0 (0-0)	
2009	Bahamas	0 (0-0)		0 (0-0)		0 (0-0)		0 (0-0)		0 (0-0)		0 (0-0)	
2010	Bahamas	0 (0-0)		0 (0-0)		0 (0-0)		0 (0-0)		0 (0-0)		0 (0-0)	
2011	Bahamas	0 (0-0)		0 (0-0)		0 (0-0)		0 (0-0)		0 (0-0)		0 (0-0)	
2012	Bahamas	0 (0-0)		0 (0-0)		0 (0-0)		0 (0-0)		0 (0-0)		0 (0-0)	
2013	Bahamas	0 (0-0)		0 (0-0)		0 (0-0)		0 (0-0)		0 (0-0)		0 (0-0)	
2014	Bahamas	0 (0-0)		0 (0-0)		0 (0-0)		0 (0-0)		0 (0-0)		0 (0-0)	
2015	Bahamas	0 (0-0)	0 (0-0)	0 (0-0)	0 (0-0)	0 (0-0)	0 (0-0)	0 (0-0)	0 (0-0)	0 (0-0)	0 (0-0)	0 (0-0)	0 (0-0)
2000	Bahrain	0 (0-0)		0 (0-0)		0 (0-0)		0 (0-0)		0 (0-0)		0 (0-0)	
2001	Bahrain	0 (0-0)		0 (0-0)		0 (0-0)		0 (0-0)		0 (0-0)		0 (0-0)	
2002	Bahrain	0 (0-0)		0 (0-0)		0 (0-0)		0 (0-0)		0 (0-0)		0 (0-0)	
2003	Bahrain	0 (0-0)		0 (0-0)		0 (0-0)		0 (0-0)		0 (0-0)		0 (0-0)	
2004	Bahrain	0 (0-0)		0 (0-0)		0 (0-0)		0 (0-0)		0 (0-0)		0 (0-0)	
2005	Bahrain	0 (0-0)		0 (0-0)		0 (0-0)		0 (0-0)		0 (0-0)		0 (0-0)	
2006	Bahrain	0 (0-0)		0 (0-0)		0 (0-0)		0 (0-0)		0 (0-0)		0 (0-0)	
2007	Bahrain	0 (0-0)		0 (0-0)		0 (0-0)		0 (0-0)		0 (0-0)		0 (0-0)	
2008	Bahrain	0 (0-0)		0 (0-0)		0 (0-0)		0 (0-0)		0 (0-0)		0 (0-0)	
2009	Bahrain	0 (0-0)		0 (0-0)		0 (0-0)		0 (0-0)		0 (0-0)		0 (0-0)	
2010	Bahrain	0 (0-0)		0 (0-0)		0 (0-0)		0 (0-0)		0 (0-0)		0 (0-0)	
2011	Bahrain	0 (0-0)		0 (0-0)		0 (0-0)		0 (0-0)		0 (0-0)		0 (0-0)	
2012	Bahrain	0 (0-0)		0 (0-0)		0 (0-0)		0 (0-0)		0 (0-0)		0 (0-0)	
2013	Bahrain	0 (0-0)		0 (0-0)		0 (0-0)		0 (0-0)		0 (0-0)		0 (0-0)	
2014	Bahrain	0 (0-0)		0 (0-0)		0 (0-0)		0 (0-0)		0 (0-0)		0 (0-0)	
2015	Bahrain	0 (0-0)	0 (0-0)	0 (0-0)	0 (0-0)	0 (0-0)	0 (0-0)	0 (0-0)	0 (0-0)	0 (0-0)	0 (0-0)	0 (0-0)	0 (0-0)
2000	Bangladesh	7626 (5363-10020)		46 (33-61)		3156 (914-5108)		19 (6-31)		13 (4-21)		0 (0-0)	
2001	Bangladesh	7218 (5077-9484)		43 (31-57)		2960 (847-4800)		18 (5-29)		12 (3-20)		0 (0-0)	
2002	Bangladesh	6524 (4588-8572)		39 (28-52)		2649 (749-4303)		16 (5-26)		11 (3-18)		0 (0-0)	
2003	Bangladesh	6081 (4277-7990)		37 (26-48)		2444 (605-4099)		15 (4-25)		20 (5-33)		0 (0-0)	
2004	Bangladesh	5644 (3970-7416)		34 (24-45)		2244 (549-3772)		14 (3-23)		18 (4-30)		0 (0-0)	
2005	Bangladesh	5342 (3757-7019)		33 (23-43)		2099 (532-3508)		13 (3-21)		17 (4-28)		0 (0-0)	
2006	Bangladesh	4889 (3438-6424)		30 (21-39)		1893 (498-3147)		12 (3-19)		15 (4-25)		0 (0-0)	
2007	Bangladesh	4452 (3132-5850)		28 (19-36)		1700 (472-2800)		11 (3-17)		14 (4-22)		0 (0-0)	
2008	Bangladesh	4023 (2829-5286)		25 (18-33)		1514 (419-2497)		10 (3-16)		12 (3-20)		0 (0-0)	
2009	Bangladesh	553 (389-727)		4 (2-5)		205 (57-339)		1 (0-2)		2 (0-3)		0 (0-0)	
2010	Bangladesh	248 (174-325)		2 (1-2)		90 (25-150)		1 (0-1)		1 (0-1)		0 (0-0)	
2011	Bangladesh	264 (186-347)		2 (1-2)		95 (26-158)		1 (0-1)		1 (0-1)		0 (0-0)	
2012	Bangladesh	211 (149-278)		1 (1-2)		76 (21-125)		0 (0-1)		1 (0-1)		0 (0-0)	
2013	Bangladesh	195 (137-257)		1 (1-2)		69 (19-115)		0 (0-1)		1 (0-1)		0 (0-0)	
2014	Bangladesh	25 (17-32)		0 (0-0)		9 (3-14)		0 (0-0)		0 (0-0)		0 (0-0)	
2015	Bangladesh	23 (16-30)	0 (0-0)	0 (0-0)	0 (0-0)	8 (2-13)	0 (0-0)	0 (0-0)	0 (0-0)	0 (0-0)	0 (0-0)	0 (0-0)	0 (0-0)
2000	Barbados	0 (0-0)		0 (0-0)		0 (0-1)		3 (1-5)		0 (0-0)		0 (0-0)	
2001	Barbados	0 (0-0)		1 (1-1)		0 (0-1)		2 (1-3)		0 (0-0)		0 (0-0)	
2002	Barbados	0 (0-0)		0 (0-0)		0 (0-0)		0 (0-0)		0 (0-0)		0 (0-0)	
2003	Barbados	0 (0-0)		0 (0-0)		0 (0-0)		0 (0-0)		0 (0-0)		0 (0-0)	
2004	Barbados	0 (0-0)		0 (0-0)		0 (0-0)		0 (0-0)		0 (0-0)		0 (0-0)	
2005	Barbados	0 (0-0)		0 (0-0)		0 (0-0)		0 (0-0)		0 (0-0)		0 (0-0)	
2006	Barbados	0 (0-0)		0 (0-0)		0 (0-0)		0 (0-0)		0 (0-0)		0 (0-0)	
2007	Barbados	0 (0-0)		0 (0-0)		0 (0-0)		0 (0-0)		0 (0-0)		0 (0-0)	
2008	Barbados	0 (0-0)		0 (0-0)		0 (0-0)		0 (0-0)		0 (0-0)		0 (0-0)	
2009	Barbados	0 (0-0)		0 (0-0)		0 (0-0)		0 (0-0)		0 (0-0)		0 (0-0)	
2010	Barbados	0 (0-0)		0 (0-0)		0 (0-0)		0 (0-0)		0 (0-0)		0 (0-0)	
2011	Barbados	0 (0-0)		0 (0-0)		0 (0-0)		0 (0-0)		0 (0-0)		0 (0-0)	
2012	Barbados	0 (0-0)		0 (0-0)		0 (0-0)		0 (0-0)		0 (0-0)		0 (0-0)	
2013	Barbados	0 (0-0)		0 (0-0)		0 (0-0)		0 (0-0)		0 (0-0)		0 (0-0)	
2014	Barbados	0 (0-0)		0 (0-0)		0 (0-0)		0 (0-0)		0 (0-0)		0 (0-0)	
2015	Barbados	0 (0-0)	0 (0-0)	0 (0-0)	0 (0-0)	0 (0-0)	0 (0-0)	0 (0-0)	0 (0-0)	0 (0-0)	0 (0-0)	0 (0-0)	0 (0-0)
2000	Belarus	22 (15-29)		5 (3-6)		6 (1-12)		1 (0-3)		0 (0-0)		0 (0-0)	
2001	Belarus	19 (14-25)		4 (3-6)		5 (1-11)		1 (0-2)		0 (0-0)		0 (0-0)	
2002	Belarus	17 (12-22)		4 (3-5)		5 (1-9)		1 (0-2)		0 (0-0)		0 (0-0)	
2003	Belarus	15 (11-20)		4 (2-5)		4 (1-8)		1 (0-2)		0 (0-0)		0 (0-0)	
2004	Belarus	14 (10-19)		3 (2-4)		4 (1-8)		1 (0-2)		0 (0-0)		0 (0-0)	
2005	Belarus	13 (9-17)		3 (2-4)		4 (1-7)		1 (0-2)		0 (0-0)		0 (0-0)	
2006	Belarus	12 (9-16)		3 (2-4)		4 (1-7)		1 (0-2)		0 (0-0)		0 (0-0)	
2007	Belarus	11 (8-14)		2 (2-3)		3 (1-6)		1 (0-1)		0 (0-0)		0 (0-0)	
2008	Belarus	9 (6-12)		2 (1-3)		3 (1-6)		1 (0-1)		0 (0-0)		0 (0-0)	
2009	Belarus	6 (4-8)		1 (1-2)		2 (1-4)		0 (0-1)		0 (0-0)		0 (0-0)	
2010	Belarus	8 (5-10)		2 (1-2)		2 (1-4)		0 (0-1)		0 (0-0)		0 (0-0)	
2011	Belarus	6 (4-8)		1 (1-1)		2 (1-4)		0 (0-1)		0 (0-0)		0 (0-0)	
2012	Belarus	4 (3-5)		1 (1-1)		2 (0-3)		0 (0-1)		0 (0-0)		0 (0-0)	
2013	Belarus	4 (2-5)		1 (0-1)		1 (0-3)		0 (0-0)		0 (0-0)		0 (0-0)	
2014	Belarus	4 (2-5)		1 (0-1)		1 (0-2)		0 (0-0)		0 (0-0)		0 (0-0)	
2015	Belarus	3 (2-4)	0 (0-0)	1 (0-1)	0 (0-0)	1 (0-2)	0 (0-0)	0 (0-0)	0 (0-0)	0 (0-0)	0 (0-0)	0 (0-0)	0 (0-0)
2000	Belgium	0 (0-0)		0 (0-0)		0 (0-0)		0 (0-0)		0 (0-0)		0 (0-0)	
2001	Belgium	0 (0-0)		0 (0-0)		0 (0-0)		0 (0-0)		0 (0-0)		0 (0-0)	
2002	Belgium	0 (0-0)		0 (0-0)		0 (0-0)		0 (0-0)		0 (0-0)		0 (0-0)	
2003	Belgium	0 (0-0)		0 (0-0)		0 (0-0)		0 (0-0)		0 (0-0)		0 (0-0)	
2004	Belgium	0 (0-0)		0 (0-0)		0 (0-0)		0 (0-0)		0 (0-0)		0 (0-0)	
2005	Belgium	0 (0-0)		0 (0-0)		0 (0-0)		0 (0-0)		0 (0-0)		0 (0-0)	
2006	Belgium	0 (0-0)		0 (0-0)		0 (0-0)		0 (0-0)		0 (0-0)		0 (0-0)	

Hib = *Haemophilus influenzae* type b; npnm = non-pneumonia, non-meningitis; HIV = deaths only in HIV-infected children; Uncertainty range provided in parentheses.

Year	Country	Hib pneumonia deaths (UR)	Hib pneumonia deaths, HIV (UR)	Hib pneumonia mortality rate (UR)	Hib pneumonia mortality rate, HIV (UR)	Hib meningitis deaths (UR)	Hib meningitis deaths, HIV (UR)	Hib meningitis mortality rate (UR)	Hib meningitis mortality rate, HIV (UR)	Hib NPNM deaths (UR)	Hib NPNM deaths, HIV (UR)	Hib NPNM mortality rate (UR)	Hib NPNM mortality rate, HIV (UR)
2007	Belgium	0 (0-0)		0 (0-0)		0 (0-0)		0 (0-0)		0 (0-0)		0 (0-0)	
2008	Belgium	0 (0-0)		0 (0-0)		0 (0-0)		0 (0-0)		0 (0-0)		0 (0-0)	
2009	Belgium	0 (0-0)		0 (0-0)		0 (0-0)		0 (0-0)		0 (0-0)		0 (0-0)	
2010	Belgium	0 (0-0)		0 (0-0)		0 (0-0)		0 (0-0)		0 (0-0)		0 (0-0)	
2011	Belgium	0 (0-0)		0 (0-0)		0 (0-0)		0 (0-0)		0 (0-0)		0 (0-0)	
2012	Belgium	0 (0-0)		0 (0-0)		0 (0-0)		0 (0-0)		0 (0-0)		0 (0-0)	
2013	Belgium	0 (0-0)		0 (0-0)		0 (0-0)		0 (0-0)		0 (0-0)		0 (0-0)	
2014	Belgium	0 (0-0)		0 (0-0)		0 (0-0)		0 (0-0)		0 (0-0)		0 (0-0)	
2015	Belgium	0 (0-0)	0 (0-0)	0 (0-0)	0 (0-0)	0 (0-0)	0 (0-0)	0 (0-0)	0 (0-0)	0 (0-0)	0 (0-0)	0 (0-0)	0 (0-0)
2000	Belize	4 (3-5)		11 (8-15)		2 (0-4)		6 (1-11)		0 (0-0)		0 (0-0)	
2001	Belize	1 (0-1)		2 (1-2)		1 (0-1)		2 (0-3)		0 (0-0)		0 (0-0)	
2002	Belize	0 (0-1)		1 (1-2)		0 (0-1)		1 (0-2)		0 (0-0)		0 (0-0)	
2003	Belize	0 (0-0)		1 (1-1)		0 (0-0)		0 (0-0)		0 (0-0)		0 (0-0)	
2004	Belize	0 (0-0)		1 (1-1)		0 (0-0)		1 (0-1)		0 (0-0)		0 (0-0)	
2005	Belize	0 (0-0)		0 (0-1)		0 (0-0)		0 (0-0)		0 (0-0)		0 (0-0)	
2006	Belize	0 (0-0)		0 (0-0)		0 (0-0)		0 (0-0)		0 (0-0)		0 (0-0)	
2007	Belize	0 (0-0)		0 (0-0)		0 (0-0)		0 (0-0)		0 (0-0)		0 (0-0)	
2008	Belize	0 (0-0)		0 (0-0)		0 (0-0)		0 (0-0)		0 (0-0)		0 (0-0)	
2009	Belize	0 (0-0)		0 (0-0)		0 (0-0)		0 (0-0)		0 (0-0)		0 (0-0)	
2010	Belize	0 (0-0)		0 (0-0)		0 (0-0)		0 (0-0)		0 (0-0)		0 (0-0)	
2011	Belize	0 (0-0)		0 (0-0)		0 (0-0)		0 (0-0)		0 (0-0)		0 (0-0)	
2012	Belize	0 (0-0)		0 (0-0)		0 (0-0)		0 (0-0)		0 (0-0)		0 (0-0)	
2013	Belize	0 (0-0)		0 (0-0)		0 (0-0)		0 (0-0)		0 (0-0)		0 (0-0)	
2014	Belize	0 (0-0)		0 (0-0)		0 (0-0)		0 (0-0)		0 (0-0)		0 (0-0)	
2015	Belize	0 (0-0)	0 (0-0)	0 (0-0)	0 (0-0)	0 (0-0)	0 (0-0)	0 (0-0)	0 (0-0)	0 (0-0)	0 (0-0)	0 (0-0)	0 (0-0)
2000	Benin	1014 (713-1333)		83 (58-109)		410 (126-671)		34 (10-55)		2 (1-3)		0 (0-0)	
2001	Benin	995 (700-1308)		79 (56-104)		400 (123-654)		32 (10-52)		2 (1-3)		0 (0-0)	
2002	Benin	989 (696-1299)		77 (54-101)		396 (122-647)		31 (9-50)		2 (0-3)		0 (0-0)	
2003	Benin	979 (688-1286)		74 (52-97)		391 (120-638)		29 (9-48)		2 (0-3)		0 (0-0)	
2004	Benin	963 (677-1265)		71 (50-93)		384 (118-627)		28 (9-46)		2 (0-3)		0 (0-0)	
2005	Benin	678 (477-890)		49 (34-64)		270 (83-440)		19 (6-32)		1 (0-2)		0 (0-0)	
2006	Benin	106 (74-139)		7 (5-10)		42 (13-68)		3 (1-5)		0 (0-0)		0 (0-0)	
2007	Benin	94 (66-123)		6 (4-8)		37 (11-61)		3 (1-4)		0 (0-0)		0 (0-0)	
2008	Benin	108 (76-142)		7 (5-9)		43 (13-70)		3 (1-5)		0 (0-0)		0 (0-0)	
2009	Benin	103 (73-136)		7 (5-9)		41 (12-67)		3 (1-4)		0 (0-0)		0 (0-0)	
2010	Benin	11 (7-14)		1 (0-1)		4 (1-7)		0 (0-0)		0 (0-0)		0 (0-0)	
2011	Benin	11 (8-14)		1 (0-1)		4 (1-7)		0 (0-0)		0 (0-0)		0 (0-0)	
2012	Benin	10 (7-14)		1 (0-1)		4 (1-7)		0 (0-0)		0 (0-0)		0 (0-0)	
2013	Benin	11 (7-14)		1 (0-1)		4 (1-7)		0 (0-0)		0 (0-0)		0 (0-0)	
2014	Benin	11 (7-14)		1 (0-1)		4 (1-7)		0 (0-0)		0 (0-0)		0 (0-0)	
2015	Benin	10 (7-14)	0 (0-0)	1 (0-1)	0 (0-0)	4 (1-7)	0 (0-0)	0 (0-0)	0 (0-0)	0 (0-0)	0 (0-0)	0 (0-0)	0 (0-0)
2000	Bhutan	37 (26-49)		51 (36-66)		12 (5-19)		16 (6-25)		0 (0-0)		0 (0-0)	
2001	Bhutan	34 (24-45)		46 (33-61)		11 (4-17)		15 (6-23)		0 (0-0)		0 (0-0)	
2002	Bhutan	31 (22-41)		43 (30-56)		10 (4-15)		13 (5-21)		0 (0-0)		0 (0-0)	
2003	Bhutan	28 (20-37)		39 (27-51)		9 (3-14)		12 (5-19)		0 (0-0)		0 (0-0)	
2004	Bhutan	25 (18-33)		36 (25-47)		8 (3-12)		11 (4-17)		0 (0-0)		0 (0-0)	
2005	Bhutan	23 (16-30)		33 (23-43)		7 (3-11)		10 (4-15)		0 (0-0)		0 (0-0)	
2006	Bhutan	21 (15-28)		30 (21-39)		6 (2-10)		9 (3-14)		0 (0-0)		0 (0-0)	
2007	Bhutan	20 (14-26)		28 (19-36)		6 (2-9)		8 (3-13)		0 (0-0)		0 (0-0)	
2008	Bhutan	18 (13-24)		26 (18-34)		5 (2-8)		7 (3-11)		0 (0-0)		0 (0-0)	
2009	Bhutan	17 (12-22)		23 (16-31)		5 (2-7)		7 (3-10)		0 (0-0)		0 (0-0)	
2010	Bhutan	15 (11-20)		21 (15-28)		4 (2-7)		6 (2-9)		0 (0-0)		0 (0-0)	
2011	Bhutan	14 (10-18)		19 (14-25)		4 (2-6)		5 (2-8)		0 (0-0)		0 (0-0)	
2012	Bhutan	2 (1-2)		3 (2-4)		1 (0-1)		1 (0-1)		0 (0-0)		0 (0-0)	
2013	Bhutan	1 (1-1)		1 (1-1)		0 (0-0)		0 (0-0)		0 (0-0)		0 (0-0)	
2014	Bhutan	1 (0-1)		1 (1-1)		0 (0-0)		0 (0-0)		0 (0-0)		0 (0-0)	
2015	Bhutan	1 (0-1)	0 (0-0)	1 (1-1)	0 (0-0)	0 (0-0)	0 (0-0)	0 (0-0)	0 (0-0)	0 (0-0)	0 (0-0)	0 (0-0)	0 (0-0)
2000	Bolivia (Plurinational State of)	118 (83-155)		10 (7-14)		37 (12-61)		3 (1-5)		0 (0-0)		0 (0-0)	
2001	Bolivia (Plurinational State of)	57 (40-74)		5 (3-6)		18 (6-29)		2 (0-3)		0 (0-0)		0 (0-0)	
2002	Bolivia (Plurinational State of)	52 (37-69)		5 (3-6)		17 (5-28)		1 (0-2)		0 (0-0)		0 (0-0)	
2003	Bolivia (Plurinational State of)	46 (32-60)		4 (3-5)		15 (4-24)		1 (0-2)		0 (0-0)		0 (0-0)	
2004	Bolivia (Plurinational State of)	39 (27-51)		3 (2-4)		12 (3-21)		1 (0-2)		0 (0-0)		0 (0-0)	
2005	Bolivia (Plurinational State of)	4 (3-5)		0 (0-0)		1 (0-2)		0 (0-0)		0 (0-0)		0 (0-0)	
2006	Bolivia (Plurinational State of)	4 (3-5)		0 (0-0)		1 (0-2)		0 (0-0)		0 (0-0)		0 (0-0)	
2007	Bolivia (Plurinational State of)	3 (2-4)		0 (0-0)		1 (0-2)		0 (0-0)		0 (0-0)		0 (0-0)	
2008	Bolivia (Plurinational State of)	3 (2-4)		0 (0-0)		1 (0-2)		0 (0-0)		0 (0-0)		0 (0-0)	
2009	Bolivia (Plurinational State of)	3 (2-4)		0 (0-0)		1 (0-1)		0 (0-0)		0 (0-0)		0 (0-0)	
2010	Bolivia (Plurinational State of)	3 (2-4)		0 (0-0)		1 (0-1)		0 (0-0)		0 (0-0)		0 (0-0)	
2011	Bolivia (Plurinational State of)	3 (2-3)		0 (0-0)		1 (0-1)		0 (0-0)		0 (0-0)		0 (0-0)	
2012	Bolivia (Plurinational State of)	3 (2-3)		0 (0-0)		1 (0-1)		0 (0-0)		0 (0-0)		0 (0-0)	
2013	Bolivia (Plurinational State of)	2 (2-3)		0 (0-0)		1 (0-1)		0 (0-0)		0 (0-0)		0 (0-0)	
2014	Bolivia (Plurinational State of)	2 (2-3)		0 (0-0)		1 (0-1)		0 (0-0)		0 (0-0)		0 (0-0)	
2015	Bolivia (Plurinational State of)	2 (2-3)	0 (0-0)	0 (0-0)	0 (0-0)	1 (0-1)	0 (0-0)	0 (0-0)	0 (0-0)	0 (0-0)	0 (0-0)	0 (0-0)	0 (0-0)
2000	Bosnia and Herzegovina	3 (2-4)		1 (1-2)		1 (0-2)		0 (0-1)		0 (0-0)		0 (0-0)	
2001	Bosnia and Herzegovina	3 (2-3)		1 (1-2)		1 (0-1)		0 (0-1)		0 (0-0)		0 (0-0)	
2002	Bosnia and Herzegovina	2 (2-3)		1 (1-1)		1 (0-1)		0 (0-1)		0 (0-0)		0 (0-0)	
2003	Bosnia and Herzegovina	2 (1-3)		1 (1-1)		1 (0-1)		0 (0-1)		0 (0-0)		0 (0-0)	
2004	Bosnia and Herzegovina	1 (1-1)		1 (0-1)		0 (0-1)		0 (0-0)		0 (0-0)		0 (0-0)	
2005	Bosnia and Herzegovina	0 (0-0)		0 (0-0)		0 (0-0)		0 (0-0)		0 (0-0)		0 (0-0)	
2006	Bosnia and Herzegovina	0 (0-0)		0 (0-0)		0 (0-0)		0 (0-0)		0 (0-0)		0 (0-0)	
2007	Bosnia and Herzegovina	0 (0-0)		0 (0-0)		0 (0-0)		0 (0-0)		0 (0-0)		0 (0-0)	
2008	Bosnia and Herzegovina	0 (0-0)		0 (0-0)		0 (0-0)		0 (0-0)		0 (0-0)		0 (0-0)	
2009	Bosnia and Herzegovina	0 (0-0)		0 (0-0)		0 (0-0)		0 (0-0)		0 (0-0)		0 (0-0)	
2010	Bosnia and Herzegovina	0 (0-0)		0 (0-0)		0 (0-0)		0 (0-0)		0 (0-0)		0 (0-0)	
2011	Bosnia and Herzegovina	0 (0-0)		0 (0-0)		0 (0-0)		0 (0-0)		0 (0-0)		0 (0-0)	
2012	Bosnia and Herzegovina	0 (0-0)		0 (0-0)		0 (0-0)		0 (0-0)		0 (0-0)		0 (0-0)	
2013	Bosnia and Herzegovina	0 (0-0)		0 (0-0)		0 (0-0)		0 (0-0)		0 (0-0)		0 (0-0)	
2014	Bosnia and Herzegovina	0 (0-0)		0 (0-0)		0 (0-0)		0 (0-0)		0 (0-0)		0 (0-0)	

Hib = *Haemophilus influenzae* type b; npnm = non-pneumonia, non-meningitis; HIV = deaths only in HIV-infected children; Uncertainty range provided in parentheses.

Year	Country	Hib pneumonia deaths (UR)	Hib pneumonia deaths, HIV (UR)	Hib pneumonia mortality rate (UR)	Hib pneumonia mortality rate, HIV (UR)	Hib meningitis deaths (UR)	Hib meningitis deaths, HIV (UR)	Hib meningitis mortality rate (UR)	Hib meningitis mortality rate, HIV (UR)	Hib NPNM deaths (UR)	Hib NPNM deaths, HIV (UR)	Hib NPNM mortality rate (UR)	Hib NPNM mortality rate, HIV (UR)
2015	Bosnia and Herzegovina	0 (0-0)	0 (0-0)	0 (0-0)	0 (0-0)	0 (0-0)	0 (0-0)	0 (0-0)	0 (0-0)	0 (0-0)	0 (0-0)	0 (0-0)	0 (0-0)
2000	Botswana	39 (28-52)		18 (13-24)		9 (3-16)		4 (1-7)		0 (0-0)		0 (0-0)	
2001	Botswana	40 (28-53)		18 (13-24)		10 (3-16)		4 (1-7)		0 (0-0)		0 (0-0)	
2002	Botswana	41 (29-54)		19 (13-25)		10 (3-17)		5 (1-8)		0 (0-0)		0 (0-0)	
2003	Botswana	43 (30-56)		20 (14-26)		10 (3-17)		5 (1-8)		0 (0-0)		0 (0-0)	
2004	Botswana	45 (32-59)		21 (14-27)		11 (3-18)		5 (1-8)		0 (0-0)		0 (0-0)	
2005	Botswana	46 (32-61)		21 (15-28)		11 (3-19)		5 (1-9)		0 (0-0)		0 (0-0)	
2006	Botswana	49 (34-64)		22 (16-29)		11 (3-20)		5 (1-9)		0 (0-0)		0 (0-0)	
2007	Botswana	53 (37-69)		24 (17-31)		12 (3-21)		5 (1-9)		0 (0-0)		0 (0-0)	
2008	Botswana	55 (39-72)		24 (17-32)		12 (3-21)		6 (1-9)		0 (0-0)		0 (0-0)	
2009	Botswana	55 (39-73)		24 (17-32)		12 (3-21)		5 (1-9)		0 (0-0)		0 (0-0)	
2010	Botswana	55 (39-72)		24 (17-31)		12 (3-21)		5 (1-9)		0 (0-0)		0 (0-0)	
2011	Botswana	9 (6-12)		4 (3-5)		2 (0-3)		1 (0-1)		0 (0-0)		0 (0-0)	
2012	Botswana	4 (3-6)		2 (1-2)		1 (0-2)		0 (0-1)		0 (0-0)		0 (0-0)	
2013	Botswana	4 (3-5)		2 (1-2)		1 (0-2)		0 (0-1)		0 (0-0)		0 (0-0)	
2014	Botswana	4 (3-5)		2 (1-2)		1 (0-2)		0 (0-1)		0 (0-0)		0 (0-0)	
2015	Botswana	4 (3-5)	0 (0-0)	2 (1-2)	0 (0-0)	1 (0-2)	0 (0-0)	0 (0-1)	0 (0-0)	0 (0-0)	0 (0-0)	0 (0-0)	0 (0-0)
2000	Brazil	463 (326-609)		3 (2-4)		310 (79-527)		2 (0-3)		2 (1-4)		0 (0-0)	
2001	Brazil	197 (139-259)		1 (1-1)		137 (12-243)		1 (0-1)		1 (0-2)		0 (0-0)	
2002	Brazil	172 (121-225)		1 (1-1)		107 (10-190)		1 (0-1)		1 (0-2)		0 (0-0)	
2003	Brazil	152 (107-200)		1 (1-1)		87 (8-154)		0 (0-1)		1 (0-1)		0 (0-0)	
2004	Brazil	136 (96-179)		1 (1-1)		79 (7-141)		0 (0-1)		1 (0-1)		0 (0-0)	
2005	Brazil	16 (11-21)		0 (0-0)		10 (1-18)		0 (0-0)		0 (0-0)		0 (0-0)	
2006	Brazil	14 (10-19)		0 (0-0)		10 (1-17)		0 (0-0)		0 (0-0)		0 (0-0)	
2007	Brazil	12 (8-16)		0 (0-0)		8 (1-14)		0 (0-0)		0 (0-0)		0 (0-0)	
2008	Brazil	10 (7-13)		0 (0-0)		7 (1-12)		0 (0-0)		0 (0-0)		0 (0-0)	
2009	Brazil	8 (6-11)		0 (0-0)		5 (1-9)		0 (0-0)		0 (0-0)		0 (0-0)	
2010	Brazil	7 (5-9)		0 (0-0)		5 (0-8)		0 (0-0)		0 (0-0)		0 (0-0)	
2011	Brazil	7 (5-9)		0 (0-0)		3 (0-6)		0 (0-0)		0 (0-0)		0 (0-0)	
2012	Brazil	7 (5-10)		0 (0-0)		3 (0-5)		0 (0-0)		0 (0-0)		0 (0-0)	
2013	Brazil	9 (6-11)		0 (0-0)		3 (0-5)		0 (0-0)		0 (0-0)		0 (0-0)	
2014	Brazil	11 (8-15)		0 (0-0)		4 (0-8)		0 (0-0)		0 (0-0)		0 (0-0)	
2015	Brazil	13 (9-17)	0 (0-0)	0 (0-0)	0 (0-0)	5 (1-9)	0 (0-0)	0 (0-0)	0 (0-0)	0 (0-0)	0 (0-0)	0 (0-0)	0 (0-0)
2000	Brunei Darussalam	1 (0-1)		2 (1-2)		0 (0-0)		0 (0-0)		0 (0-0)		0 (0-0)	
2001	Brunei Darussalam	0 (0-1)		1 (1-2)		0 (0-0)		0 (0-0)		0 (0-0)		0 (0-0)	
2002	Brunei Darussalam	0 (0-0)		0 (0-0)		0 (0-0)		0 (0-0)		0 (0-0)		0 (0-0)	
2003	Brunei Darussalam	0 (0-0)		0 (0-0)		0 (0-0)		0 (0-0)		0 (0-0)		0 (0-0)	
2004	Brunei Darussalam	0 (0-0)		0 (0-0)		0 (0-0)		0 (0-0)		0 (0-0)		0 (0-0)	
2005	Brunei Darussalam	0 (0-0)		0 (0-0)		0 (0-0)		0 (0-0)		0 (0-0)		0 (0-0)	
2006	Brunei Darussalam	0 (0-0)		0 (0-0)		0 (0-0)		0 (0-0)		0 (0-0)		0 (0-0)	
2007	Brunei Darussalam	0 (0-0)		0 (0-0)		0 (0-0)		0 (0-0)		0 (0-0)		0 (0-0)	
2008	Brunei Darussalam	0 (0-0)		0 (0-0)		0 (0-0)		0 (0-0)		0 (0-0)		0 (0-0)	
2009	Brunei Darussalam	0 (0-0)		0 (0-0)		0 (0-0)		0 (0-0)		0 (0-0)		0 (0-0)	
2010	Brunei Darussalam	0 (0-0)		0 (0-0)		0 (0-0)		0 (0-0)		0 (0-0)		0 (0-0)	
2011	Brunei Darussalam	0 (0-0)		0 (0-0)		0 (0-0)		0 (0-0)		0 (0-0)		0 (0-0)	
2012	Brunei Darussalam	0 (0-0)		0 (0-0)		0 (0-0)		0 (0-0)		0 (0-0)		0 (0-0)	
2013	Brunei Darussalam	0 (0-0)		0 (0-0)		0 (0-0)		0 (0-0)		0 (0-0)		0 (0-0)	
2014	Brunei Darussalam	0 (0-0)		0 (0-0)		0 (0-0)		0 (0-0)		0 (0-0)		0 (0-0)	
2015	Brunei Darussalam	0 (0-0)	0 (0-0)	0 (0-0)	0 (0-0)	0 (0-0)	0 (0-0)	0 (0-0)	0 (0-0)	0 (0-0)	0 (0-0)	0 (0-0)	0 (0-0)
2000	Bulgaria	44 (31-57)		13 (9-18)		4 (2-8)		1 (1-3)		0 (0-0)		0 (0-0)	
2001	Bulgaria	41 (29-54)		13 (9-17)		4 (2-7)		1 (1-2)		0 (0-0)		0 (0-0)	
2002	Bulgaria	33 (23-43)		10 (7-14)		2 (1-4)		1 (0-1)		0 (0-0)		0 (0-0)	
2003	Bulgaria	39 (27-51)		12 (9-16)		5 (2-10)		2 (1-3)		0 (0-0)		0 (0-0)	
2004	Bulgaria	27 (19-36)		9 (6-12)		4 (2-8)		1 (1-3)		0 (0-0)		0 (0-0)	
2005	Bulgaria	31 (21-40)		10 (7-13)		4 (2-7)		1 (1-2)		0 (0-0)		0 (0-0)	
2006	Bulgaria	30 (21-39)		9 (7-12)		5 (2-9)		2 (1-3)		0 (0-0)		0 (0-0)	
2007	Bulgaria	27 (19-36)		8 (6-11)		4 (2-8)		1 (1-2)		0 (0-0)		0 (0-0)	
2008	Bulgaria	24 (17-32)		7 (5-9)		6 (2-10)		2 (1-3)		0 (0-0)		0 (0-0)	
2009	Bulgaria	36 (25-47)		10 (7-13)		4 (2-7)		1 (0-2)		0 (0-0)		0 (0-0)	
2010	Bulgaria	6 (4-8)		2 (1-2)		1 (0-2)		0 (0-0)		0 (0-0)		0 (0-0)	
2011	Bulgaria	2 (2-3)		1 (0-1)		0 (0-1)		0 (0-0)		0 (0-0)		0 (0-0)	
2012	Bulgaria	2 (2-3)		1 (0-1)		0 (0-1)		0 (0-0)		0 (0-0)		0 (0-0)	
2013	Bulgaria	2 (2-3)		1 (0-1)		0 (0-1)		0 (0-0)		0 (0-0)		0 (0-0)	
2014	Bulgaria	3 (2-4)		1 (1-1)		0 (0-1)		0 (0-0)		0 (0-0)		0 (0-0)	
2015	Bulgaria	0 (0-0)	0 (0-0)	0 (0-0)	0 (0-0)	0 (0-0)	0 (0-0)	0 (0-0)	0 (0-0)	0 (0-0)	0 (0-0)	0 (0-0)	0 (0-0)
2000	Burkina Faso	1211 (852-1591)		57 (40-74)		535 (246-798)		25 (12-37)		2 (1-3)		0 (0-0)	
2001	Burkina Faso	1284 (903-1688)		58 (41-77)		566 (262-843)		26 (12-38)		2 (1-3)		0 (0-0)	
2002	Burkina Faso	1366 (961-1795)		60 (42-79)		601 (279-892)		27 (12-39)		2 (1-4)		0 (0-0)	
2003	Burkina Faso	1455 (1023-1911)		63 (44-82)		637 (297-945)		27 (13-41)		3 (1-4)		0 (0-0)	
2004	Burkina Faso	1534 (1079-2015)		64 (45-85)		668 (313-990)		28 (13-42)		3 (1-4)		0 (0-0)	
2005	Burkina Faso	1565 (1101-2057)		64 (45-84)		678 (319-1004)		28 (13-41)		3 (1-4)		0 (0-0)	
2006	Burkina Faso	328 (231-431)		13 (9-17)		142 (45-231)		6 (2-9)		1 (0-1)		0 (0-0)	
2007	Burkina Faso	118 (83-155)		5 (3-6)		51 (17-82)		2 (1-3)		0 (0-0)		0 (0-0)	
2008	Burkina Faso	99 (69-129)		4 (3-5)		42 (14-68)		2 (1-3)		0 (0-0)		0 (0-0)	
2009	Burkina Faso	96 (68-126)		4 (2-5)		41 (14-65)		1 (1-2)		0 (0-0)		0 (0-0)	
2010	Burkina Faso	99 (70-130)		4 (2-5)		41 (15-65)		1 (1-2)		0 (0-0)		0 (0-0)	
2011	Burkina Faso	13 (9-18)		0 (0-1)		5 (2-9)		0 (0-0)		0 (0-0)		0 (0-0)	
2012	Burkina Faso	14 (10-19)		0 (0-1)		6 (2-9)		0 (0-0)		0 (0-0)		0 (0-0)	
2013	Burkina Faso	15 (11-20)		1 (0-1)		6 (3-9)		0 (0-0)		0 (0-0)		0 (0-0)	
2014	Burkina Faso	16 (11-21)		1 (0-1)		6 (3-10)		0 (0-0)		0 (0-0)		0 (0-0)	
2015	Burkina Faso	16 (11-21)	0 (0-0)	1 (0-1)	0 (0-0)	6 (3-10)	0 (0-0)	0 (0-0)	0 (0-0)	0 (0-0)	0 (0-0)	0 (0-0)	0 (0-0)
2000	Burundi	1043 (734-1371)		87 (61-114)		524 (249-772)		44 (21-64)		2 (1-3)		0 (0-0)	
2001	Burundi	1040 (732-1367)		86 (60-113)		525 (167-854)		43 (14-70)		2 (1-3)		0 (0-0)	
2002	Burundi	1025 (721-1347)		83 (58-109)		521 (165-847)		42 (13-69)		2 (1-3)		0 (0-0)	
2003	Burundi	1089 (766-1431)		86 (60-113)		557 (176-905)		44 (14-71)		2 (1-4)		0 (0-0)	
2004	Burundi	186 (131-244)		14 (10-19)		96 (30-156)		7 (2-12)		0 (0-1)		0 (0-0)	
2005	Burundi	98 (69-128)		7 (5-9)		50 (16-82)		4 (1-6)		0 (0-0)		0 (0-0)	
2006	Burundi	93 (65-122)		7 (5-9)		48 (15-78)		3 (1-5)		0 (0-0)		0 (0-0)	

Hib = *Haemophilus influenzae* type b; npnm = non-pneumonia, non-meningitis; HIV = deaths only in HIV-infected children; Uncertainty range provided in parentheses.

Year	Country	Hib pneumonia deaths (UR)	Hib pneumonia deaths, HIV (UR)	Hib pneumonia mortality rate (UR)	Hib pneumonia mortality rate, HIV (UR)	Hib meningitis deaths (UR)	Hib meningitis deaths, HIV (UR)	Hib meningitis mortality rate (UR)	Hib meningitis mortality rate, HIV (UR)	Hib NPNM deaths (UR)	Hib NPNM deaths, HIV (UR)	Hib NPNM mortality rate (UR)	Hib NPNM mortality rate, HIV (UR)
2007	Burundi	72 (50-94)		5 (3-6)		37 (12-59)		2 (1-4)		0 (0-0)		0 (0-0)	
2008	Burundi	93 (66-123)		6 (4-8)		48 (16-77)		3 (1-5)		0 (0-0)		0 (0-0)	
2009	Burundi	12 (8-16)		1 (1-1)		6 (2-10)		0 (0-1)		0 (0-0)		0 (0-0)	
2010	Burundi	12 (8-16)		1 (0-1)		6 (2-10)		0 (0-1)		0 (0-0)		0 (0-0)	
2011	Burundi	12 (9-16)		1 (0-1)		6 (2-10)		0 (0-1)		0 (0-0)		0 (0-0)	
2012	Burundi	12 (8-16)		1 (0-1)		6 (2-9)		0 (0-1)		0 (0-0)		0 (0-0)	
2013	Burundi	12 (8-16)		1 (0-1)		6 (2-9)		0 (0-0)		0 (0-0)		0 (0-0)	
2014	Burundi	12 (8-16)		1 (0-1)		6 (2-9)		0 (0-0)		0 (0-0)		0 (0-0)	
2015	Burundi	12 (8-15)	0 (0-0)	1 (0-1)	0 (0-0)	6 (3-9)	0 (0-0)	0 (0-0)	0 (0-0)	0 (0-0)	0 (0-0)	0 (0-0)	0 (0-0)
2000	Cambodia	1147 (807-1508)		74 (52-97)		500 (156-803)		32 (10-52)		2 (1-3)		0 (0-0)	
2001	Cambodia	1021 (718-1341)		67 (47-88)		437 (139-699)		29 (9-46)		2 (1-3)		0 (0-0)	
2002	Cambodia	901 (634-1184)		60 (42-79)		379 (122-604)		25 (8-40)		2 (2-2)		0 (0-0)	
2003	Cambodia	800 (562-1051)		53 (38-70)		330 (108-525)		22 (7-35)		1 (0-2)		0 (0-0)	
2004	Cambodia	730 (513-959)		49 (34-64)		296 (89-483)		20 (6-32)		2 (1-4)		0 (0-0)	
2005	Cambodia	677 (476-890)		45 (32-59)		269 (82-438)		18 (5-29)		2 (1-3)		0 (0-0)	
2006	Cambodia	637 (448-837)		41 (29-54)		247 (78-401)		16 (5-26)		2 (1-3)		0 (0-0)	
2007	Cambodia	597 (420-784)		38 (27-50)		228 (74-367)		14 (5-23)		2 (1-3)		0 (0-0)	
2008	Cambodia	557 (392-732)		35 (24-46)		210 (70-336)		13 (4-21)		2 (1-3)		0 (0-0)	
2009	Cambodia	509 (358-669)		31 (22-41)		190 (66-303)		12 (4-19)		2 (1-2)		0 (0-0)	
2010	Cambodia	74 (52-98)		5 (3-6)		27 (10-43)		2 (1-3)		0 (0-0)		0 (0-0)	
2011	Cambodia	36 (25-48)		2 (1-3)		13 (5-21)		1 (0-1)		0 (0-0)		0 (0-0)	
2012	Cambodia	31 (22-40)		2 (1-2)		11 (4-17)		1 (0-1)		0 (0-0)		0 (0-0)	
2013	Cambodia	29 (21-39)		2 (1-2)		10 (4-16)		1 (0-1)		0 (0-0)		0 (0-0)	
2014	Cambodia	25 (17-32)		1 (1-2)		9 (3-13)		0 (0-1)		0 (0-0)		0 (0-0)	
2015	Cambodia	3 (2-4)	0 (0-0)	0 (0-0)	0 (0-0)	1 (0-2)	0 (0-0)	0 (0-0)	0 (0-0)	0 (0-0)	0 (0-0)	0 (0-0)	0 (0-0)
2000	Cameroon	2295 (1614-3015)		85 (60-111)		818 (363-1230)		30 (13-45)		3 (1-5)		0 (0-0)	
2001	Cameroon	2225 (1565-2924)		80 (56-105)		789 (232-1302)		28 (8-47)		3 (1-5)		0 (0-0)	
2002	Cameroon	2215 (1558-2910)		78 (55-102)		782 (236-1282)		28 (8-45)		3 (1-5)		0 (0-0)	
2003	Cameroon	2154 (1515-2830)		74 (52-97)		757 (235-1235)		26 (8-42)		3 (1-5)		0 (0-0)	
2004	Cameroon	2104 (1480-2765)		71 (50-93)		737 (235-1195)		25 (8-40)		3 (1-5)		0 (0-0)	
2005	Cameroon	2093 (1472-2750)		69 (48-90)		733 (229-1193)		24 (8-39)		3 (1-5)		0 (0-0)	
2006	Cameroon	2131 (1499-2800)		68 (48-89)		746 (229-1220)		24 (7-39)		3 (1-5)		0 (0-0)	
2007	Cameroon	2155 (1516-2832)		67 (47-88)		756 (230-1237)		24 (7-39)		3 (1-5)		0 (0-0)	
2008	Cameroon	2231 (1569-2931)		68 (48-90)		783 (237-1282)		24 (7-39)		3 (1-5)		0 (0-0)	
2009	Cameroon	391 (275-514)		12 (8-15)		137 (41-225)		4 (1-7)		1 (0-1)		0 (0-0)	
2010	Cameroon	216 (152-284)		6 (5-8)		76 (23-124)		2 (1-4)		0 (0-1)		0 (0-0)	
2011	Cameroon	226 (159-297)		7 (5-9)		79 (23-130)		2 (1-4)		0 (0-1)		0 (0-0)	
2012	Cameroon	212 (149-278)		6 (4-8)		74 (22-122)		2 (1-3)		0 (0-0)		0 (0-0)	
2013	Cameroon	192 (135-252)		5 (4-7)		67 (20-110)		2 (1-3)		0 (0-0)		0 (0-0)	
2014	Cameroon	23 (16-30)		1 (0-1)		8 (2-13)		0 (0-0)		0 (0-0)		0 (0-0)	
2015	Cameroon	22 (16-30)	1 (1-1)	1 (0-1)	0 (0-0)	8 (2-13)	0 (0-0)	0 (0-0)	0 (0-0)	0 (0-0)	0 (0-0)	0 (0-0)	0 (0-0)
2000	Canada	1 (1-1)		0 (0-0)		1 (1-2)		0 (0-0)		0 (0-0)		0 (0-0)	
2001	Canada	0 (0-0)		0 (0-0)		0 (0-0)		0 (0-0)		0 (0-0)		0 (0-0)	
2002	Canada	0 (0-0)		0 (0-0)		0 (0-0)		0 (0-0)		0 (0-0)		0 (0-0)	
2003	Canada	0 (0-0)		0 (0-0)		0 (0-0)		0 (0-0)		0 (0-0)		0 (0-0)	
2004	Canada	0 (0-0)		0 (0-0)		0 (0-0)		0 (0-0)		0 (0-0)		0 (0-0)	
2005	Canada	0 (0-0)		0 (0-0)		0 (0-0)		0 (0-0)		0 (0-0)		0 (0-0)	
2006	Canada	0 (0-0)		0 (0-0)		0 (0-0)		0 (0-0)		0 (0-0)		0 (0-0)	
2007	Canada	0 (0-0)		0 (0-0)		0 (0-0)		0 (0-0)		0 (0-0)		0 (0-0)	
2008	Canada	0 (0-0)		0 (0-0)		0 (0-0)		0 (0-0)		0 (0-0)		0 (0-0)	
2009	Canada	0 (0-0)		0 (0-0)		0 (0-0)		0 (0-0)		0 (0-0)		0 (0-0)	
2010	Canada	0 (0-0)		0 (0-0)		0 (0-0)		0 (0-0)		0 (0-0)		0 (0-0)	
2011	Canada	0 (0-0)		0 (0-0)		0 (0-0)		0 (0-0)		0 (0-0)		0 (0-0)	
2012	Canada	0 (0-0)		0 (0-0)		0 (0-0)		0 (0-0)		0 (0-0)		0 (0-0)	
2013	Canada	0 (0-0)		0 (0-0)		0 (0-0)		0 (0-0)		0 (0-0)		0 (0-0)	
2014	Canada	0 (0-0)		0 (0-0)		0 (0-0)		0 (0-0)		0 (0-0)		0 (0-0)	
2015	Canada	0 (0-0)	0 (0-0)	0 (0-0)	0 (0-0)	0 (0-0)	0 (0-0)	0 (0-0)	0 (0-0)	0 (0-0)	0 (0-0)	0 (0-0)	0 (0-0)
2000	Cape Verde	11 (8-15)		19 (13-24)		2 (1-3)		3 (2-5)		0 (0-0)		0 (0-0)	
2001	Cape Verde	10 (7-13)		16 (11-21)		1 (1-2)		2 (1-4)		0 (0-0)		0 (0-0)	
2002	Cape Verde	9 (6-11)		15 (10-19)		1 (0-2)		2 (1-3)		0 (0-0)		0 (0-0)	
2003	Cape Verde	8 (6-10)		14 (10-18)		1 (0-1)		2 (1-2)		0 (0-0)		0 (0-0)	
2004	Cape Verde	7 (5-10)		13 (9-17)		1 (0-1)		2 (1-2)		0 (0-0)		0 (0-0)	
2005	Cape Verde	7 (5-10)		13 (9-18)		1 (0-1)		1 (1-2)		0 (0-0)		0 (0-0)	
2006	Cape Verde	7 (5-10)		14 (10-18)		1 (0-1)		2 (1-2)		0 (0-0)		0 (0-0)	
2007	Cape Verde	8 (5-10)		14 (10-19)		1 (0-1)		2 (1-3)		0 (0-0)		0 (0-0)	
2008	Cape Verde	8 (5-10)		15 (10-19)		1 (0-1)		2 (1-3)		0 (0-0)		0 (0-0)	
2009	Cape Verde	7 (5-10)		14 (10-19)		1 (0-1)		2 (1-3)		0 (0-0)		0 (0-0)	
2010	Cape Verde	7 (5-9)		14 (10-19)		1 (0-1)		2 (1-3)		0 (0-0)		0 (0-0)	
2011	Cape Verde	1 (1-2)		2 (2-3)		0 (0-0)		0 (0-1)		0 (0-0)		0 (0-0)	
2012	Cape Verde	1 (0-1)		1 (1-1)		0 (0-0)		0 (0-0)		0 (0-0)		0 (0-0)	
2013	Cape Verde	1 (0-1)		1 (1-1)		0 (0-0)		0 (0-0)		0 (0-0)		0 (0-0)	
2014	Cape Verde	1 (0-1)		1 (1-1)		0 (0-0)		0 (0-0)		0 (0-0)		0 (0-0)	
2015	Cape Verde	1 (0-1)	0 (0-0)	1 (1-1)	0 (0-0)	0 (0-0)	0 (0-0)	0 (0-0)	0 (0-0)	0 (0-0)	0 (0-0)	0 (0-0)	0 (0-0)
2000	Central African Republic	421 (296-554)		71 (50-93)		209 (96-312)		35 (16-52)		1 (0-1)		0 (0-0)	
2001	Central African Republic	424 (298-557)		69 (49-91)		211 (96-314)		35 (16-51)		1 (0-1)		0 (0-0)	
2002	Central African Republic	429 (302-563)		69 (49-91)		214 (98-318)		35 (16-51)		1 (0-1)		0 (0-0)	
2003	Central African Republic	419 (295-551)		67 (47-88)		209 (96-312)		34 (15-50)		1 (0-1)		0 (0-0)	
2004	Central African Republic	408 (287-536)		65 (46-86)		204 (93-304)		33 (15-49)		1 (0-1)		0 (0-0)	
2005	Central African Republic	396 (279-520)		63 (44-83)		197 (90-294)		31 (14-47)		1 (0-1)		0 (0-0)	
2006	Central African Republic	412 (290-542)		65 (46-86)		204 (93-304)		32 (15-48)		1 (0-1)		0 (0-0)	
2007	Central African Republic	431 (303-567)		68 (48-89)		210 (96-314)		33 (15-49)		1 (0-1)		0 (0-0)	
2008	Central African Republic	447 (315-588)		70 (49-92)		216 (98-322)		34 (15-51)		1 (0-1)		0 (0-0)	
2009	Central African Republic	305 (215-401)		48 (34-63)		145 (66-217)		23 (10-34)		1 (0-1)		0 (0-0)	
2010	Central African Republic	81 (57-107)		13 (9-17)		38 (17-57)		6 (3-9)		0 (0-0)		0 (0-0)	
2011	Central African Republic	83 (58-109)		13 (9-17)		40 (12-65)		6 (2-10)		0 (0-0)		0 (0-0)	
2012	Central African Republic	83 (59-109)		13 (9-17)		40 (12-66)		6 (2-10)		0 (0-0)		0 (0-0)	
2013	Central African Republic	326 (229-428)		48 (34-64)		162 (47-267)		24 (7-40)		1 (0-1)		0 (0-0)	
2014	Central African Republic	88 (62-115)		13 (9-17)		44 (13-73)		6 (2-11)		0 (0-0)		0 (0-0)	

Hib = *Haemophilus influenzae* type b; npnm = non-pneumonia, non-meningitis; HIV = deaths only in HIV-infected children; Uncertainty range provided in parentheses.

Year	Country	Hib pneumonia deaths	Hib pneumonia deaths (HIV)	Hib pneumonia mortality rate	Hib pneumonia mortality rate (HIV)	Hib meningitis deaths	Hib meningitis deaths (HIV)	Hib meningitis mortality rate	Hib meningitis mortality rate (HIV)	Hib NPNM deaths	Hib NPNM deaths (HIV)	Hib NPNM mortality rate	Hib NPNM mortality rate (HIV)
2015	Central African Republic	88 (62-115)	4 (3-5)	13 (9-17)	1 (0-1)	45 (13-75)	2 (1-3)	6 (2-11)	0 (0-0)	0 (0-0)	0 (0-0)	0 (0-0)	0 (0-0)
2000	Chad	2137 (1503-2808)		129 (91-169)		951 (415-1433)		57 (25-86)		4 (2-6)		0 (0-0)	
2001	Chad	2186 (1537-2872)		127 (89-166)		960 (415-1451)		56 (24-84)		4 (2-6)		0 (0-0)	
2002	Chad	2368 (1665-3111)		132 (93-174)		1016 (435-1541)		57 (24-86)		4 (2-6)		0 (0-0)	
2003	Chad	2387 (1678-3136)		129 (90-169)		995 (421-1513)		54 (23-82)		4 (2-6)		0 (0-0)	
2004	Chad	2477 (1742-3254)		129 (91-170)		1001 (420-1528)		52 (22-80)		4 (2-6)		0 (0-0)	
2005	Chad	2565 (1804-3371)		130 (91-170)		1013 (429-1541)		51 (22-78)		4 (2-6)		0 (0-0)	
2006	Chad	2734 (1923-3592)		134 (94-176)		1065 (455-1615)		52 (22-79)		4 (2-7)		0 (0-0)	
2007	Chad	2713 (1908-3565)		129 (91-169)		1047 (452-1585)		50 (21-75)		4 (2-6)		0 (0-0)	
2008	Chad	2616 (1840-3437)		121 (85-159)		1004 (438-1516)		46 (20-70)		4 (2-6)		0 (0-0)	
2009	Chad	1552 (1091-2039)		70 (49-92)		592 (261-892)		27 (12-40)		2 (1-4)		0 (0-0)	
2010	Chad	541 (380-711)		24 (17-31)		205 (91-308)		9 (4-14)		1 (0-1)		0 (0-0)	
2011	Chad	962 (677-1264)		41 (29-54)		363 (163-543)		15 (7-23)		1 (1-2)		0 (0-0)	
2012	Chad	455 (320-597)		19 (13-25)		170 (77-254)		7 (3-11)		1 (0-1)		0 (0-0)	
2013	Chad	444 (312-584)		18 (13-24)		165 (50-271)		7 (2-11)		1 (0-1)		0 (0-0)	
2014	Chad	453 (319-596)		18 (13-24)		167 (52-273)		7 (2-11)		1 (0-1)		0 (0-0)	
2015	Chad	408 (287-535)	12 (8-15)	16 (11-21)	0 (0-1)	150 (47-243)	4 (1-7)	6 (2-9)	0 (0-0)	1 (0-1)	0 (0-0)	0 (0-0)	0 (0-0)
2000	Chile	4 (3-6)		0 (0-0)		1 (1-3)		0 (0-0)		0 (0-0)		0 (0-0)	
2001	Chile	1 (0-1)		0 (0-0)		0 (0-0)		0 (0-0)		0 (0-0)		0 (0-0)	
2002	Chile	0 (0-0)		0 (0-0)		0 (0-0)		0 (0-0)		0 (0-0)		0 (0-0)	
2003	Chile	0 (0-0)		0 (0-0)		0 (0-0)		0 (0-0)		0 (0-0)		0 (0-0)	
2004	Chile	0 (0-0)		0 (0-0)		0 (0-0)		0 (0-0)		0 (0-0)		0 (0-0)	
2005	Chile	0 (0-0)		0 (0-0)		0 (0-0)		0 (0-0)		0 (0-0)		0 (0-0)	
2006	Chile	0 (0-0)		0 (0-0)		0 (0-0)		0 (0-0)		0 (0-0)		0 (0-0)	
2007	Chile	0 (0-0)		0 (0-0)		0 (0-0)		0 (0-0)		0 (0-0)		0 (0-0)	
2008	Chile	0 (0-0)		0 (0-0)		0 (0-0)		0 (0-0)		0 (0-0)		0 (0-0)	
2009	Chile	0 (0-0)		0 (0-0)		0 (0-0)		0 (0-0)		0 (0-0)		0 (0-0)	
2010	Chile	0 (0-0)		0 (0-0)		0 (0-0)		0 (0-0)		0 (0-0)		0 (0-0)	
2011	Chile	0 (0-0)		0 (0-0)		0 (0-0)		0 (0-0)		0 (0-0)		0 (0-0)	
2012	Chile	0 (0-0)		0 (0-0)		0 (0-0)		0 (0-0)		0 (0-0)		0 (0-0)	
2013	Chile	0 (0-0)		0 (0-0)		0 (0-0)		0 (0-0)		0 (0-0)		0 (0-0)	
2014	Chile	0 (0-0)		0 (0-0)		0 (0-0)		0 (0-0)		0 (0-0)		0 (0-0)	
2015	Chile	0 (0-0)	0 (0-0)	0 (0-0)	0 (0-0)	0 (0-0)	0 (0-0)	0 (0-0)	0 (0-0)	0 (0-0)	0 (0-0)	0 (0-0)	0 (0-0)
2000	China	10980 (7722-14427)		14 (10-18)		2315 (1309-3644)		3 (2-5)		18 (10-29)		0 (0-0)	
2001	China	9542 (6711-12537)		12 (9-16)		1954 (1129-3143)		3 (1-4)		16 (9-25)		0 (0-0)	
2002	China	8321 (5852-10933)		11 (8-14)		1635 (977-2720)		2 (1-4)		13 (8-22)		0 (0-0)	
2003	China	7209 (5070-9473)		10 (7-13)		1343 (543-1790)		2 (1-2)		11 (4-14)		0 (0-0)	
2004	China	6293 (4426-8268)		8 (6-11)		1076 (473-1560)		1 (1-2)		9 (4-12)		0 (0-0)	
2005	China	5537 (3894-7275)		7 (5-10)		903 (433-1425)		1 (1-2)		7 (3-11)		0 (0-0)	
2006	China	5111 (3595-6716)		7 (5-9)		839 (425-1401)		1 (1-2)		7 (3-11)		0 (0-0)	
2007	China	4771 (3355-6268)		6 (4-8)		828 (419-1382)		1 (1-2)		7 (3-11)		0 (0-0)	
2008	China	4503 (3167-5917)		6 (4-8)		849 (430-1418)		1 (1-2)		7 (3-11)		0 (0-0)	
2009	China	4256 (2994-5593)		6 (4-7)		840 (426-1403)		1 (1-2)		7 (3-11)		0 (0-0)	
2010	China	4034 (2837-5300)		5 (4-7)		829 (420-1384)		1 (1-2)		7 (3-11)		0 (0-0)	
2011	China	3736 (2628-4909)		5 (3-6)		782 (396-1306)		1 (1-2)		6 (3-10)		0 (0-0)	
2012	China	3444 (2422-4525)		4 (3-6)		705 (357-1177)		1 (0-1)		6 (3-9)		0 (0-0)	
2013	China	3209 (2257-4217)		4 (3-5)		649 (329-1084)		1 (0-1)		5 (3-9)		0 (0-0)	
2014	China	2948 (2073-3873)		4 (3-5)		582 (295-972)		1 (0-1)		5 (2-8)		0 (0-0)	
2015	China	2797 (1967-3675)	1 (0-1)	3 (2-4)	0 (0-0)	552 (280-922)	0 (0-0)	1 (0-1)	0 (0-0)	4 (2-7)	0 (0-0)	0 (0-0)	0 (0-0)
2000	Colombia	58 (41-76)		1 (1-2)		28 (3-50)		1 (0-1)		0 (0-0)		0 (0-0)	
2001	Colombia	57 (40-75)		1 (1-2)		30 (3-52)		1 (0-1)		0 (0-0)		0 (0-0)	
2002	Colombia	38 (27-50)		1 (1-1)		25 (3-44)		1 (0-1)		0 (0-0)		0 (0-0)	
2003	Colombia	4 (3-5)		0 (0-0)		2 (0-4)		0 (0-0)		0 (0-0)		0 (0-0)	
2004	Colombia	4 (3-5)		0 (0-0)		2 (0-4)		0 (0-0)		0 (0-0)		0 (0-0)	
2005	Colombia	4 (3-5)		0 (0-0)		2 (0-4)		0 (0-0)		0 (0-0)		0 (0-0)	
2006	Colombia	4 (3-5)		0 (0-0)		2 (0-4)		0 (0-0)		0 (0-0)		0 (0-0)	
2007	Colombia	3 (2-5)		0 (0-0)		2 (0-3)		0 (0-0)		0 (0-0)		0 (0-0)	
2008	Colombia	3 (2-4)		0 (0-0)		1 (0-3)		0 (0-0)		0 (0-0)		0 (0-0)	
2009	Colombia	3 (2-4)		0 (0-0)		1 (0-3)		0 (0-0)		0 (0-0)		0 (0-0)	
2010	Colombia	3 (2-3)		0 (0-0)		1 (0-2)		0 (0-0)		0 (0-0)		0 (0-0)	
2011	Colombia	3 (2-4)		0 (0-0)		1 (0-2)		0 (0-0)		0 (0-0)		0 (0-0)	
2012	Colombia	3 (2-4)		0 (0-0)		1 (0-2)		0 (0-0)		0 (0-0)		0 (0-0)	
2013	Colombia	3 (2-4)		0 (0-0)		1 (0-2)		0 (0-0)		0 (0-0)		0 (0-0)	
2014	Colombia	3 (2-4)		0 (0-0)		1 (0-2)		0 (0-0)		0 (0-0)		0 (0-0)	
2015	Colombia	3 (2-4)	0 (0-0)	0 (0-0)	0 (0-0)	1 (0-2)	0 (0-0)	0 (0-0)	0 (0-0)	0 (0-0)	0 (0-0)	0 (0-0)	0 (0-0)
2000	Comoros	42 (30-55)		48 (34-63)		17 (6-27)		19 (7-31)		0 (0-0)		0 (0-0)	
2001	Comoros	43 (30-56)		48 (33-62)		17 (6-28)		19 (7-31)		0 (0-0)		0 (0-0)	
2002	Comoros	44 (31-58)		48 (34-63)		18 (6-28)		19 (7-31)		0 (0-0)		0 (0-0)	
2003	Comoros	45 (31-59)		48 (34-63)		18 (6-29)		19 (7-31)		0 (0-0)		0 (0-0)	
2004	Comoros	45 (32-59)		47 (33-62)		18 (6-29)		19 (7-31)		0 (0-0)		0 (0-0)	
2005	Comoros	45 (31-59)		46 (32-61)		18 (6-29)		19 (6-30)		0 (0-0)		0 (0-0)	
2006	Comoros	44 (31-58)		45 (32-59)		18 (6-29)		18 (6-29)		0 (0-0)		0 (0-0)	
2007	Comoros	44 (31-58)		44 (31-57)		18 (6-29)		18 (6-29)		0 (0-0)		0 (0-0)	
2008	Comoros	44 (31-57)		42 (30-56)		18 (6-29)		17 (6-28)		0 (0-0)		0 (0-0)	
2009	Comoros	29 (20-38)		28 (19-36)		12 (4-19)		11 (4-18)		0 (0-0)		0 (0-0)	
2010	Comoros	5 (3-6)		4 (3-6)		2 (1-3)		2 (1-3)		0 (0-0)		0 (0-0)	
2011	Comoros	4 (3-5)		3 (2-5)		2 (1-3)		1 (0-2)		0 (0-0)		0 (0-0)	
2012	Comoros	3 (2-5)		3 (2-4)		1 (0-2)		1 (0-2)		0 (0-0)		0 (0-0)	
2013	Comoros	4 (3-5)		3 (2-4)		2 (0-2)		1 (0-2)		0 (0-0)		0 (0-0)	
2014	Comoros	0 (0-0)		0 (0-0)		0 (0-0)		0 (0-0)		0 (0-0)		0 (0-0)	
2015	Comoros	0 (0-0)	0 (0-0)	0 (0-0)	0 (0-0)	0 (0-0)	0 (0-0)	0 (0-0)	0 (0-0)	0 (0-0)	0 (0-0)	0 (0-0)	0 (0-0)
2000	Congo	304 (214-399)		61 (43-80)		89 (29-144)		18 (6-29)		0 (0-1)		0 (0-0)	
2001	Congo	314 (221-413)		61 (43-80)		93 (30-150)		18 (6-29)		0 (0-1)		0 (0-0)	
2002	Congo	316 (222-415)		60 (42-79)		94 (31-151)		18 (6-29)		0 (0-1)		0 (0-0)	
2003	Congo	310 (218-407)		57 (40-76)		92 (31-149)		17 (6-28)		0 (0-1)		0 (0-0)	
2004	Congo	303 (213-398)		55 (39-72)		92 (31-147)		17 (6-27)		0 (0-1)		0 (0-0)	
2005	Congo	292 (205-384)		51 (36-68)		89 (30-143)		16 (5-25)		0 (0-1)		0 (0-0)	
2006	Congo	280 (197-367)		48 (34-63)		86 (29-138)		15 (5-24)		0 (0-1)		0 (0-0)	

Hib = *Haemophilus influenzae* type b; npnm = non-pneumonia, non-meningitis; HIV = deaths only in HIV-infected children; Uncertainty range provided in parentheses.

Year	Country	Hib pneumonia deaths	Hib pneumonia deaths (HIV)	Hib pneumonia mortality rate	Hib pneumonia mortality rate (HIV)	Hib meningitis deaths	Hib meningitis deaths (HIV)	Hib meningitis mortality rate	Hib meningitis mortality rate (HIV)	Hib NPNM deaths	Hib NPNM deaths (HIV)	Hib NPNM mortality rate	Hib NPNM mortality rate (HIV)
2007	Congo	267 (188-351)		44 (31-58)		82 (28-132)		14 (5-22)		0 (0-1)		0 (0-0)	
2008	Congo	253 (178-332)		41 (29-53)		77 (24-128)		12 (4-20)		1 (0-1)		0 (0-0)	
2009	Congo	62 (44-82)		10 (7-13)		19 (6-31)		3 (1-5)		0 (0-0)		0 (0-0)	
2010	Congo	24 (17-32)		4 (3-5)		7 (2-12)		1 (0-2)		0 (0-0)		0 (0-0)	
2011	Congo	21 (14-27)		3 (2-4)		6 (2-10)		1 (0-1)		0 (0-0)		0 (0-0)	
2012	Congo	20 (14-27)		3 (2-4)		6 (2-9)		1 (0-1)		0 (0-0)		0 (0-0)	
2013	Congo	17 (12-23)		2 (2-3)		5 (2-8)		1 (0-1)		0 (0-0)		0 (0-0)	
2014	Congo	2 (1-2)		0 (0-0)		1 (0-1)		0 (0-0)		0 (0-0)		0 (0-0)	
2015	Congo	2 (1-2)	0 (0-0)	0 (0-0)	0 (0-0)	1 (0-1)	0 (0-0)	0 (0-0)	0 (0-0)	0 (0-0)	0 (0-0)	0 (0-0)	0 (0-0)
2000	Cook Islands	0 (0-0)		6 (4-8)		0 (0-0)		2 (1-3)		0 (0-0)		0 (0-0)	
2001	Cook Islands	0 (0-0)		6 (4-8)		0 (0-0)		2 (1-3)		0 (0-0)		0 (0-0)	
2002	Cook Islands	0 (0-0)		6 (4-8)		0 (0-0)		2 (1-3)		0 (0-0)		0 (0-0)	
2003	Cook Islands	0 (0-0)		4 (3-5)		0 (0-0)		1 (1-2)		0 (0-0)		0 (0-0)	
2004	Cook Islands	0 (0-0)		4 (3-5)		0 (0-0)		1 (1-2)		0 (0-0)		0 (0-0)	
2005	Cook Islands	0 (0-0)		6 (4-8)		0 (0-0)		2 (1-3)		0 (0-0)		0 (0-0)	
2006	Cook Islands	0 (0-0)		5 (3-6)		0 (0-0)		2 (1-2)		0 (0-0)		0 (0-0)	
2007	Cook Islands	0 (0-0)		4 (3-6)		0 (0-0)		1 (1-2)		0 (0-0)		0 (0-0)	
2008	Cook Islands	0 (0-0)		3 (2-4)		0 (0-0)		1 (1-1)		0 (0-0)		0 (0-0)	
2009	Cook Islands	0 (0-0)		1 (0-1)		0 (0-0)		0 (0-0)		0 (0-0)		0 (0-0)	
2010	Cook Islands	0 (0-0)		0 (0-0)		0 (0-0)		0 (0-0)		0 (0-0)		0 (0-0)	
2011	Cook Islands	0 (0-0)		0 (0-0)		0 (0-0)		0 (0-0)		0 (0-0)		0 (0-0)	
2012	Cook Islands	0 (0-0)		0 (0-0)		0 (0-0)		0 (0-0)		0 (0-0)		0 (0-0)	
2013	Cook Islands	0 (0-0)		0 (0-0)		0 (0-0)		0 (0-0)		0 (0-0)		0 (0-0)	
2014	Cook Islands	0 (0-0)		0 (0-0)		0 (0-0)		0 (0-0)		0 (0-0)		0 (0-0)	
2015	Cook Islands	0 (0-0)	0 (0-0)	0 (0-0)	0 (0-0)	0 (0-0)	0 (0-0)	0 (0-0)	0 (0-0)	0 (0-0)	0 (0-0)	0 (0-0)	0 (0-0)
2000	Costa Rica	1 (1-1)		0 (0-0)		0 (0-1)		0 (0-0)		0 (0-0)		0 (0-0)	
2001	Costa Rica	1 (1-1)		0 (0-0)		0 (0-1)		0 (0-0)		0 (0-0)		0 (0-0)	
2002	Costa Rica	1 (0-1)		0 (0-0)		1 (0-1)		0 (0-0)		0 (0-0)		0 (0-0)	
2003	Costa Rica	0 (0-0)		0 (0-0)		0 (0-0)		0 (0-0)		0 (0-0)		0 (0-0)	
2004	Costa Rica	0 (0-0)		0 (0-0)		0 (0-0)		0 (0-0)		0 (0-0)		0 (0-0)	
2005	Costa Rica	0 (0-0)		0 (0-0)		0 (0-0)		0 (0-0)		0 (0-0)		0 (0-0)	
2006	Costa Rica	0 (0-0)		0 (0-0)		0 (0-0)		0 (0-0)		0 (0-0)		0 (0-0)	
2007	Costa Rica	0 (0-0)		0 (0-0)		0 (0-0)		0 (0-0)		0 (0-0)		0 (0-0)	
2008	Costa Rica	0 (0-0)		0 (0-0)		0 (0-0)		0 (0-0)		0 (0-0)		0 (0-0)	
2009	Costa Rica	0 (0-0)		0 (0-0)		0 (0-0)		0 (0-0)		0 (0-0)		0 (0-0)	
2010	Costa Rica	0 (0-0)		0 (0-0)		0 (0-0)		0 (0-0)		0 (0-0)		0 (0-0)	
2011	Costa Rica	0 (0-0)		0 (0-0)		0 (0-0)		0 (0-0)		0 (0-0)		0 (0-0)	
2012	Costa Rica	0 (0-0)		0 (0-0)		0 (0-0)		0 (0-0)		0 (0-0)		0 (0-0)	
2013	Costa Rica	0 (0-0)		0 (0-0)		0 (0-0)		0 (0-0)		0 (0-0)		0 (0-0)	
2014	Costa Rica	0 (0-0)		0 (0-0)		0 (0-0)		0 (0-0)		0 (0-0)		0 (0-0)	
2015	Costa Rica	0 (0-0)	0 (0-0)	0 (0-0)	0 (0-0)	0 (0-0)	0 (0-0)	0 (0-0)	0 (0-0)	0 (0-0)	0 (0-0)	0 (0-0)	0 (0-0)
2000	Cote d'Ivoire	1121 (788-1473)		40 (28-53)		370 (116-602)		13 (4-22)		2 (0-2)		0 (0-0)	
2001	Cote d'Ivoire	1104 (776-1450)		38 (27-51)		368 (115-599)		13 (4-21)		1 (0-2)		0 (0-0)	
2002	Cote d'Ivoire	1077 (757-1415)		37 (26-48)		362 (113-590)		12 (4-20)		1 (0-2)		0 (0-0)	
2003	Cote d'Ivoire	1052 (740-1383)		36 (25-47)		357 (111-582)		12 (4-20)		1 (0-2)		0 (0-0)	
2004	Cote d'Ivoire	1025 (721-1347)		34 (24-45)		349 (108-570)		12 (4-19)		1 (0-2)		0 (0-0)	
2005	Cote d'Ivoire	1034 (727-1359)		34 (24-45)		353 (109-577)		12 (4-19)		1 (0-2)		0 (0-0)	
2006	Cote d'Ivoire	1064 (748-1398)		35 (24-46)		363 (111-593)		12 (4-19)		1 (0-2)		0 (0-0)	
2007	Cote d'Ivoire	1126 (792-1480)		36 (26-48)		382 (118-624)		12 (4-20)		2 (0-3)		0 (0-0)	
2008	Cote d'Ivoire	1180 (830-1551)		38 (27-50)		400 (124-653)		13 (4-21)		2 (1-3)		0 (0-0)	
2009	Cote d'Ivoire	214 (150-281)		7 (5-9)		72 (23-118)		2 (1-4)		0 (0-0)		0 (0-0)	
2010	Cote d'Ivoire	125 (88-164)		4 (3-5)		42 (13-69)		1 (0-2)		0 (0-0)		0 (0-0)	
2011	Cote d'Ivoire	192 (135-252)		6 (4-8)		65 (20-105)		2 (1-3)		0 (0-0)		0 (0-0)	
2012	Cote d'Ivoire	146 (103-192)		4 (3-6)		49 (15-80)		1 (0-2)		0 (0-0)		0 (0-0)	
2013	Cote d'Ivoire	158 (111-207)		5 (3-6)		53 (17-86)		2 (0-2)		0 (0-0)		0 (0-0)	
2014	Cote d'Ivoire	16 (11-21)		0 (0-1)		5 (2-9)		0 (0-0)		0 (0-0)		0 (0-0)	
2015	Cote d'Ivoire	16 (12-22)	0 (0-1)	0 (0-1)	0 (0-0)	5 (2-9)	0 (0-0)	0 (0-0)	0 (0-0)	0 (0-0)	0 (0-0)	0 (0-0)	0 (0-0)
2000	Croatia	2 (1-2)		1 (1-1)		1 (1-2)		1 (0-1)		0 (0-0)		0 (0-0)	
2001	Croatia	1 (1-2)		1 (0-1)		1 (1-3)		1 (1-3)		0 (0-0)		0 (0-0)	
2002	Croatia	0 (0-0)		0 (0-0)		0 (0-1)		0 (0-0)		0 (0-0)		0 (0-0)	
2003	Croatia	0 (0-0)		0 (0-0)		0 (0-0)		0 (0-0)		0 (0-0)		0 (0-0)	
2004	Croatia	0 (0-0)		0 (0-0)		0 (0-0)		0 (0-0)		0 (0-0)		0 (0-0)	
2005	Croatia	0 (0-0)		0 (0-0)		0 (0-0)		0 (0-0)		0 (0-0)		0 (0-0)	
2006	Croatia	0 (0-0)		0 (0-0)		0 (0-0)		0 (0-0)		0 (0-0)		0 (0-0)	
2007	Croatia	0 (0-0)		0 (0-0)		0 (0-0)		0 (0-0)		0 (0-0)		0 (0-0)	
2008	Croatia	0 (0-0)		0 (0-0)		0 (0-0)		0 (0-0)		0 (0-0)		0 (0-0)	
2009	Croatia	0 (0-0)		0 (0-0)		0 (0-0)		0 (0-0)		0 (0-0)		0 (0-0)	
2010	Croatia	0 (0-0)		0 (0-0)		0 (0-0)		0 (0-0)		0 (0-0)		0 (0-0)	
2011	Croatia	0 (0-0)		0 (0-0)		0 (0-0)		0 (0-0)		0 (0-0)		0 (0-0)	
2012	Croatia	0 (0-0)		0 (0-0)		0 (0-0)		0 (0-0)		0 (0-0)		0 (0-0)	
2013	Croatia	0 (0-0)		0 (0-0)		0 (0-0)		0 (0-0)		0 (0-0)		0 (0-0)	
2014	Croatia	0 (0-0)		0 (0-0)		0 (0-0)		0 (0-0)		0 (0-0)		0 (0-0)	
2015	Croatia	0 (0-0)	0 (0-0)	0 (0-0)	0 (0-0)	0 (0-0)	0 (0-0)	0 (0-0)	0 (0-0)	0 (0-0)	0 (0-0)	0 (0-0)	0 (0-0)
2000	Cuba	1 (1-1)		0 (0-0)		1 (0-2)		0 (0-0)		0 (0-0)		0 (0-0)	
2001	Cuba	1 (1-1)		0 (0-0)		1 (0-1)		0 (0-0)		0 (0-0)		0 (0-0)	
2002	Cuba	1 (0-1)		0 (0-0)		0 (0-1)		0 (0-0)		0 (0-0)		0 (0-0)	
2003	Cuba	1 (0-1)		0 (0-0)		1 (0-1)		0 (0-0)		0 (0-0)		0 (0-0)	
2004	Cuba	0 (0-0)		0 (0-0)		0 (0-0)		0 (0-0)		0 (0-0)		0 (0-0)	
2005	Cuba	0 (0-0)		0 (0-0)		0 (0-0)		0 (0-0)		0 (0-0)		0 (0-0)	
2006	Cuba	0 (0-0)		0 (0-0)		0 (0-0)		0 (0-0)		0 (0-0)		0 (0-0)	
2007	Cuba	0 (0-0)		0 (0-0)		0 (0-0)		0 (0-0)		0 (0-0)		0 (0-0)	
2008	Cuba	0 (0-0)		0 (0-0)		0 (0-0)		0 (0-0)		0 (0-0)		0 (0-0)	
2009	Cuba	0 (0-0)		0 (0-0)		0 (0-0)		0 (0-0)		0 (0-0)		0 (0-0)	
2010	Cuba	0 (0-0)		0 (0-0)		0 (0-0)		0 (0-0)		0 (0-0)		0 (0-0)	
2011	Cuba	0 (0-0)		0 (0-0)		0 (0-0)		0 (0-0)		0 (0-0)		0 (0-0)	
2012	Cuba	0 (0-0)		0 (0-0)		0 (0-0)		0 (0-0)		0 (0-0)		0 (0-0)	
2013	Cuba	0 (0-0)		0 (0-0)		0 (0-0)		0 (0-0)		0 (0-0)		0 (0-0)	
2014	Cuba	0 (0-0)		0 (0-0)		0 (0-0)		0 (0-0)		0 (0-0)		0 (0-0)	

Hib = *Haemophilus influenzae* type b; npnm = non-pneumonia, non-meningitis; HIV = deaths only in HIV-infected children; Uncertainty range provided in parentheses.

Year	Country	Hib pneumonia deaths (UR)	Hib pneumonia deaths, HIV (UR)	Hib pneumonia mortality rate (UR)	Hib pneumonia mortality rate, HIV (UR)	Hib meningitis deaths (UR)	Hib meningitis deaths, HIV (UR)	Hib meningitis mortality rate (UR)	Hib meningitis mortality rate, HIV (UR)	Hib NPNM deaths (UR)	Hib NPNM deaths, HIV (UR)	Hib NPNM mortality rate (UR)	Hib NPNM mortality rate, HIV (UR)
2015	Cuba	0 (0-0)	0 (0-0)	0 (0-0)	0 (0-0)	0 (0-0)	0 (0-0)	0 (0-0)	0 (0-0)	0 (0-0)	0 (0-0)	0 (0-0)	0 (0-0)
2000	Cyprus	1 (0-1)		1 (1-1)		0 (0-0)		0 (0-1)		0 (0-0)		0 (0-0)	
2001	Cyprus	0 (0-0)		0 (0-0)		0 (0-0)		0 (0-0)		0 (0-0)		0 (0-0)	
2002	Cyprus	0 (0-0)		0 (0-0)		0 (0-0)		0 (0-0)		0 (0-0)		0 (0-0)	
2003	Cyprus	0 (0-0)		0 (0-0)		0 (0-0)		0 (0-0)		0 (0-0)		0 (0-0)	
2004	Cyprus	0 (0-0)		0 (0-0)		0 (0-0)		0 (0-0)		0 (0-0)		0 (0-0)	
2005	Cyprus	0 (0-0)		0 (0-0)		0 (0-0)		0 (0-0)		0 (0-0)		0 (0-0)	
2006	Cyprus	0 (0-0)		0 (0-0)		0 (0-0)		0 (0-0)		0 (0-0)		0 (0-0)	
2007	Cyprus	0 (0-0)		0 (0-0)		0 (0-0)		0 (0-0)		0 (0-0)		0 (0-0)	
2008	Cyprus	0 (0-0)		0 (0-0)		0 (0-0)		0 (0-0)		0 (0-0)		0 (0-0)	
2009	Cyprus	0 (0-0)		0 (0-0)		0 (0-0)		0 (0-0)		0 (0-0)		0 (0-0)	
2010	Cyprus	0 (0-0)		0 (0-0)		0 (0-0)		0 (0-0)		0 (0-0)		0 (0-0)	
2011	Cyprus	0 (0-0)		0 (0-0)		0 (0-0)		0 (0-0)		0 (0-0)		0 (0-0)	
2012	Cyprus	0 (0-0)		0 (0-0)		0 (0-0)		0 (0-0)		0 (0-0)		0 (0-0)	
2013	Cyprus	0 (0-0)		0 (0-0)		0 (0-0)		0 (0-0)		0 (0-0)		0 (0-0)	
2014	Cyprus	0 (0-0)		0 (0-0)		0 (0-0)		0 (0-0)		0 (0-0)		0 (0-0)	
2015	Cyprus	0 (0-0)	0 (0-0)	0 (0-0)	0 (0-0)	0 (0-0)	0 (0-0)	0 (0-0)	0 (0-0)	0 (0-0)	0 (0-0)	0 (0-0)	0 (0-0)
2000	Czech Republic	3 (2-4)		1 (0-1)		2 (1-3)		0 (0-1)		0 (0-0)		0 (0-0)	
2001	Czech Republic	3 (2-4)		1 (1-1)		2 (1-3)		0 (0-1)		0 (0-0)		0 (0-0)	
2002	Czech Republic	1 (0-1)		0 (0-0)		0 (0-0)		0 (0-0)		0 (0-0)		0 (0-0)	
2003	Czech Republic	0 (0-0)		0 (0-0)		0 (0-0)		0 (0-0)		0 (0-0)		0 (0-0)	
2004	Czech Republic	0 (0-0)		0 (0-0)		0 (0-0)		0 (0-0)		0 (0-0)		0 (0-0)	
2005	Czech Republic	0 (0-0)		0 (0-0)		0 (0-0)		0 (0-0)		0 (0-0)		0 (0-0)	
2006	Czech Republic	0 (0-0)		0 (0-0)		0 (0-0)		0 (0-0)		0 (0-0)		0 (0-0)	
2007	Czech Republic	0 (0-0)		0 (0-0)		0 (0-0)		0 (0-0)		0 (0-0)		0 (0-0)	
2008	Czech Republic	0 (0-0)		0 (0-0)		0 (0-0)		0 (0-0)		0 (0-0)		0 (0-0)	
2009	Czech Republic	0 (0-0)		0 (0-0)		0 (0-0)		0 (0-0)		0 (0-0)		0 (0-0)	
2010	Czech Republic	0 (0-0)		0 (0-0)		0 (0-0)		0 (0-0)		0 (0-0)		0 (0-0)	
2011	Czech Republic	0 (0-0)		0 (0-0)		0 (0-0)		0 (0-0)		0 (0-0)		0 (0-0)	
2012	Czech Republic	0 (0-0)		0 (0-0)		0 (0-0)		0 (0-0)		0 (0-0)		0 (0-0)	
2013	Czech Republic	0 (0-0)		0 (0-0)		0 (0-0)		0 (0-0)		0 (0-0)		0 (0-0)	
2014	Czech Republic	0 (0-0)		0 (0-0)		0 (0-0)		0 (0-0)		0 (0-0)		0 (0-0)	
2015	Czech Republic	0 (0-0)	0 (0-0)	0 (0-0)	0 (0-0)	0 (0-0)	0 (0-0)	0 (0-0)	0 (0-0)	0 (0-0)	0 (0-0)	0 (0-0)	0 (0-0)
2000	Democratic People's Republic of Korea	645 (454-848)		33 (23-43)		251 (139-388)		13 (7-20)		2 (1-3)		0 (0-0)	
2001	Democratic People's Republic of Korea	527 (371-693)		28 (19-36)		209 (115-320)		11 (6-17)		2 (1-3)		0 (0-0)	
2002	Democratic People's Republic of Korea	432 (304-567)		23 (16-30)		173 (99-261)		9 (5-14)		1 (1-2)		0 (0-0)	
2003	Democratic People's Republic of Korea	362 (255-476)		19 (14-25)		147 (82-218)		8 (4-12)		1 (1-2)		0 (0-0)	
2004	Democratic People's Republic of Korea	316 (222-415)		17 (12-22)		130 (69-189)		7 (4-10)		1 (1-2)		0 (0-0)	
2005	Democratic People's Republic of Korea	298 (209-391)		16 (11-21)		124 (63-179)		7 (3-10)		1 (1-1)		0 (0-0)	
2006	Democratic People's Republic of Korea	290 (204-381)		16 (11-21)		122 (59-178)		7 (3-10)		1 (0-1)		0 (0-0)	
2007	Democratic People's Republic of Korea	285 (200-374)		16 (11-21)		121 (56-179)		7 (3-10)		1 (0-1)		0 (0-0)	
2008	Democratic People's Republic of Korea	274 (193-361)		16 (11-21)		117 (53-176)		7 (3-10)		1 (0-1)		0 (0-0)	
2009	Democratic People's Republic of Korea	264 (186-347)		15 (11-20)		113 (49-172)		7 (3-10)		1 (0-1)		0 (0-0)	
2010	Democratic People's Republic of Korea	255 (179-335)		15 (11-20)		109 (46-168)		6 (3-10)		1 (0-1)		0 (0-0)	
2011	Democratic People's Republic of Korea	247 (173-324)		15 (10-19)		106 (43-165)		6 (3-10)		1 (0-1)		0 (0-0)	
2012	Democratic People's Republic of Korea	179 (126-235)		11 (8-14)		76 (13-127)		5 (1-8)		1 (0-1)		0 (0-0)	
2013	Democratic People's Republic of Korea	18 (13-24)		1 (1-1)		8 (1-13)		0 (0-1)		0 (0-0)		0 (0-0)	
2014	Democratic People's Republic of Korea	18 (12-23)		1 (1-1)		7 (1-13)		0 (0-1)		0 (0-0)		0 (0-0)	
2015	Democratic People's Republic of Korea	16 (11-21)	0 (0-0)	1 (1-1)	0 (0-0)	7 (1-11)	0 (0-0)	0 (0-1)	0 (0-0)	0 (0-0)	0 (0-0)	0 (0-0)	0 (0-0)
2000	Democratic Republic of the Congo	5958 (4190-7828)		67 (47-88)		3160 (1471-4693)		35 (16-53)		13 (6-19)		0 (0-0)	
2001	Democratic Republic of the Congo	5800 (4079-7621)		63 (44-83)		3106 (1448-4608)		34 (16-50)		13 (6-19)		0 (0-0)	
2002	Democratic Republic of the Congo	5756 (4048-7563)		61 (43-80)		3094 (1449-4582)		33 (15-48)		13 (6-19)		0 (0-0)	
2003	Democratic Republic of the Congo	5682 (3996-7465)		58 (41-76)		3049 (959-4967)		31 (10-51)		12 (4-20)		0 (0-0)	
2004	Democratic Republic of the Congo	5715 (4020-7510)		57 (40-74)		3056 (968-4970)		30 (10-49)		12 (4-20)		0 (0-0)	
2005	Democratic Republic of the Congo	5528 (3888-7263)		53 (37-70)		2941 (939-4774)		28 (9-46)		12 (4-19)		0 (0-0)	
2006	Democratic Republic of the Congo	5675 (3991-7457)		53 (37-69)		3002 (966-4866)		28 (9-45)		12 (4-20)		0 (0-0)	
2007	Democratic Republic of the Congo	5726 (4027-7523)		51 (36-68)		3014 (976-4875)		27 (9-44)		12 (4-20)		0 (0-0)	
2008	Democratic Republic of the Congo	6079 (4275-7987)		53 (37-70)		3183 (1027-5156)		28 (9-45)		13 (4-21)		0 (0-0)	
2009	Democratic Republic of the Congo	1790 (1259-2352)		15 (11-20)		932 (300-1512)		8 (3-13)		4 (1-6)		0 (0-0)	
2010	Democratic Republic of the Congo	992 (698-1303)		8 (6-11)		513 (164-833)		4 (1-7)		2 (1-3)		0 (0-0)	
2011	Democratic Republic of the Congo	887 (624-1166)		7 (5-9)		456 (146-739)		4 (1-6)		2 (1-3)		0 (0-0)	
2012	Democratic Republic of the Congo	938 (660-1233)		7 (5-10)		477 (154-773)		4 (1-6)		2 (1-3)		0 (0-0)	
2013	Democratic Republic of the Congo	981 (690-1288)		8 (5-10)		494 (160-800)		4 (1-6)		2 (1-3)		0 (0-0)	
2014	Democratic Republic of the Congo	93 (65-122)		1 (0-1)		46 (15-75)		0 (0-1)		0 (0-0)		0 (0-0)	
2015	Democratic Republic of the Congo	93 (65-122)	1 (1-1)	1 (0-1)	0 (0-0)	46 (15-75)	1 (0-1)	0 (0-1)	0 (0-0)	0 (0-0)	0 (0-0)	0 (0-0)	0 (0-0)
2000	Denmark	0 (0-0)		0 (0-0)		0 (0-0)		0 (0-0)		0 (0-0)		0 (0-0)	
2001	Denmark	0 (0-0)		0 (0-0)		0 (0-0)		0 (0-0)		0 (0-0)		0 (0-0)	
2002	Denmark	0 (0-0)		0 (0-0)		0 (0-0)		0 (0-0)		0 (0-0)		0 (0-0)	
2003	Denmark	0 (0-0)		0 (0-0)		0 (0-0)		0 (0-0)		0 (0-0)		0 (0-0)	
2004	Denmark	0 (0-0)		0 (0-0)		0 (0-0)		0 (0-0)		0 (0-0)		0 (0-0)	
2005	Denmark	0 (0-0)		0 (0-0)		0 (0-0)		0 (0-0)		0 (0-0)		0 (0-0)	
2006	Denmark	0 (0-0)		0 (0-0)		0 (0-0)		0 (0-0)		0 (0-0)		0 (0-0)	
2007	Denmark	0 (0-0)		0 (0-0)		0 (0-0)		0 (0-0)		0 (0-0)		0 (0-0)	
2008	Denmark	0 (0-0)		0 (0-0)		0 (0-0)		0 (0-0)		0 (0-0)		0 (0-0)	
2009	Denmark	0 (0-0)		0 (0-0)		0 (0-0)		0 (0-0)		0 (0-0)		0 (0-0)	
2010	Denmark	0 (0-0)		0 (0-0)		0 (0-0)		0 (0-0)		0 (0-0)		0 (0-0)	
2011	Denmark	0 (0-0)		0 (0-0)		0 (0-0)		0 (0-0)		0 (0-0)		0 (0-0)	
2012	Denmark	0 (0-0)		0 (0-0)		0 (0-0)		0 (0-0)		0 (0-0)		0 (0-0)	
2013	Denmark	0 (0-0)		0 (0-0)		0 (0-0)		0 (0-0)		0 (0-0)		0 (0-0)	
2014	Denmark	0 (0-0)		0 (0-0)		0 (0-0)		0 (0-0)		0 (0-0)		0 (0-0)	
2015	Denmark	0 (0-0)	0 (0-0)	0 (0-0)	0 (0-0)	0 (0-0)	0 (0-0)	0 (0-0)	0 (0-0)	0 (0-0)	0 (0-0)	0 (0-0)	0 (0-0)
2000	Djibouti	50 (35-66)		50 (36-66)		18 (7-28)		18 (7-28)		0 (0-0)		0 (0-0)	
2001	Djibouti	49 (34-64)		50 (35-65)		17 (7-27)		18 (7-27)		0 (0-0)		0 (0-0)	
2002	Djibouti	48 (34-63)		49 (34-64)		17 (7-27)		17 (7-27)		0 (0-0)		0 (0-0)	
2003	Djibouti	47 (33-62)		48 (34-63)		17 (6-26)		17 (7-26)		0 (0-0)		0 (0-0)	
2004	Djibouti	46 (32-60)		47 (33-61)		16 (6-25)		16 (6-26)		0 (0-0)		0 (0-0)	
2005	Djibouti	44 (31-58)		45 (32-59)		15 (6-24)		16 (6-25)		0 (0-0)		0 (0-0)	
2006	Djibouti	43 (30-57)		43 (31-57)		15 (6-23)		15 (6-24)		0 (0-0)		0 (0-0)	

Hib = *Haemophilus influenzae* type b; npnm = non-pneumonia, non-meningitis; HIV = deaths only in HIV-infected children; Uncertainty range provided in parentheses.

Year	Country	Hib pneumonia deaths (UR)	Hib pneumonia deaths, HIV (UR)	Hib pneumonia mortality rate (UR)	Hib pneumonia mortality rate, HIV (UR)	Hib meningitis deaths (UR)	Hib meningitis deaths, HIV (UR)	Hib meningitis mortality rate (UR)	Hib meningitis mortality rate, HIV (UR)	Hib NPNM deaths (UR)	Hib NPNM deaths, HIV (UR)	Hib NPNM mortality rate (UR)	Hib NPNM mortality rate, HIV (UR)
2007	Djibouti	34 (24-45)		35 (25-46)		12 (5-18)		12 (5-18)		0 (0-0)		0 (0-0)	
2008	Djibouti	3 (2-4)		3 (2-4)		1 (0-2)		1 (0-2)		0 (0-0)		0 (0-0)	
2009	Djibouti	3 (2-4)		3 (2-4)		1 (0-2)		1 (0-2)		0 (0-0)		0 (0-0)	
2010	Djibouti	3 (2-4)		3 (2-4)		1 (0-1)		1 (1-2)		0 (0-0)		0 (0-0)	
2011	Djibouti	3 (2-4)		3 (2-4)		1 (1-1)		1 (1-1)		0 (0-0)		0 (0-0)	
2012	Djibouti	0 (0-0)		0 (0-0)		0 (0-0)		0 (0-0)		0 (0-0)		0 (0-0)	
2013	Djibouti	0 (0-0)		0 (0-0)		0 (0-0)		0 (0-0)		0 (0-0)		0 (0-0)	
2014	Djibouti	0 (0-0)		0 (0-0)		0 (0-0)		0 (0-0)		0 (0-0)		0 (0-0)	
2015	Djibouti	0 (0-0)	0 (0-0)	0 (0-0)	0 (0-0)	0 (0-0)	0 (0-0)	0 (0-0)	0 (0-0)	0 (0-0)	0 (0-0)	0 (0-0)	0 (0-0)
2000	Dominica	0 (0-0)		2 (1-2)		0 (0-0)		0 (0-0)		0 (0-0)		0 (0-0)	
2001	Dominica	0 (0-0)		2 (1-2)		0 (0-0)		2 (1-3)		0 (0-0)		0 (0-0)	
2002	Dominica	0 (0-0)		1 (0-1)		0 (0-0)		1 (1-3)		0 (0-0)		0 (0-0)	
2003	Dominica	0 (0-0)		0 (0-0)		0 (0-0)		2 (1-3)		0 (0-0)		0 (0-0)	
2004	Dominica	0 (0-0)		1 (1-2)		0 (0-0)		0 (0-0)		0 (0-0)		0 (0-0)	
2005	Dominica	0 (0-0)		2 (2-3)		0 (0-0)		0 (0-0)		0 (0-0)		0 (0-0)	
2006	Dominica	0 (0-0)		3 (2-4)		0 (0-0)		0 (0-0)		0 (0-0)		0 (0-0)	
2007	Dominica	0 (0-0)		0 (0-1)		0 (0-0)		0 (0-0)		0 (0-0)		0 (0-0)	
2008	Dominica	0 (0-0)		0 (0-0)		0 (0-0)		0 (0-0)		0 (0-0)		0 (0-0)	
2009	Dominica	0 (0-0)		0 (0-0)		0 (0-0)		0 (0-0)		0 (0-0)		0 (0-0)	
2010	Dominica	0 (0-0)		0 (0-0)		0 (0-0)		0 (0-0)		0 (0-0)		0 (0-0)	
2011	Dominica	0 (0-0)		0 (0-0)		0 (0-0)		0 (0-0)		0 (0-0)		0 (0-0)	
2012	Dominica	0 (0-0)		0 (0-0)		0 (0-0)		0 (0-0)		0 (0-0)		0 (0-0)	
2013	Dominica	0 (0-0)		0 (0-0)		0 (0-0)		0 (0-0)		0 (0-0)		0 (0-0)	
2014	Dominica	0 (0-0)		0 (0-0)		0 (0-0)		0 (0-0)		0 (0-0)		0 (0-0)	
2015	Dominica	0 (0-0)	0 (0-0)	0 (0-0)	0 (0-0)	0 (0-0)	0 (0-0)	0 (0-0)	0 (0-0)	0 (0-0)	0 (0-0)	0 (0-0)	0 (0-0)
2000	Dominican Republic	136 (96-178)		14 (10-18)		36 (12-59)		4 (1-6)		0 (0-0)		0 (0-0)	
2001	Dominican Republic	112 (79-148)		11 (8-15)		30 (10-49)		3 (1-5)		0 (0-0)		0 (0-0)	
2002	Dominican Republic	19 (14-25)		2 (1-3)		5 (2-8)		1 (0-1)		0 (0-0)		0 (0-0)	
2003	Dominican Republic	12 (9-16)		1 (1-2)		3 (1-5)		0 (0-1)		0 (0-0)		0 (0-0)	
2004	Dominican Republic	11 (8-14)		1 (1-1)		3 (1-5)		0 (0-0)		0 (0-0)		0 (0-0)	
2005	Dominican Republic	9 (6-12)		1 (1-1)		2 (1-4)		0 (0-0)		0 (0-0)		0 (0-0)	
2006	Dominican Republic	1 (1-1)		0 (0-0)		0 (0-0)		0 (0-0)		0 (0-0)		0 (0-0)	
2007	Dominican Republic	1 (1-1)		0 (0-0)		0 (0-0)		0 (0-0)		0 (0-0)		0 (0-0)	
2008	Dominican Republic	1 (1-1)		0 (0-0)		0 (0-0)		0 (0-0)		0 (0-0)		0 (0-0)	
2009	Dominican Republic	1 (1-1)		0 (0-0)		0 (0-0)		0 (0-0)		0 (0-0)		0 (0-0)	
2010	Dominican Republic	1 (1-1)		0 (0-0)		0 (0-0)		0 (0-0)		0 (0-0)		0 (0-0)	
2011	Dominican Republic	1 (1-1)		0 (0-0)		0 (0-0)		0 (0-0)		0 (0-0)		0 (0-0)	
2012	Dominican Republic	1 (1-1)		0 (0-0)		0 (0-0)		0 (0-0)		0 (0-0)		0 (0-0)	
2013	Dominican Republic	1 (1-1)		0 (0-0)		0 (0-0)		0 (0-0)		0 (0-0)		0 (0-0)	
2014	Dominican Republic	1 (1-1)		0 (0-0)		0 (0-0)		0 (0-0)		0 (0-0)		0 (0-0)	
2015	Dominican Republic	1 (1-1)	0 (0-0)	0 (0-0)	0 (0-0)	0 (0-0)	0 (0-0)	0 (0-0)	0 (0-0)	0 (0-0)	0 (0-0)	0 (0-0)	0 (0-0)
2000	Ecuador	229 (161-301)		15 (11-20)		58 (29-92)		4 (2-6)		0 (0-1)		0 (0-0)	
2001	Ecuador	218 (153-286)		15 (10-19)		55 (28-87)		4 (2-6)		0 (0-1)		0 (0-0)	
2002	Ecuador	207 (145-272)		14 (10-18)		52 (26-83)		3 (2-6)		0 (0-1)		0 (0-0)	
2003	Ecuador	99 (70-130)		7 (5-9)		37 (19-59)		2 (1-4)		0 (0-0)		0 (0-0)	
2004	Ecuador	19 (13-25)		1 (1-2)		8 (4-13)		1 (0-1)		0 (0-0)		0 (0-0)	
2005	Ecuador	17 (12-22)		1 (1-1)		6 (2-9)		0 (0-1)		0 (0-0)		0 (0-0)	
2006	Ecuador	15 (11-20)		1 (1-1)		5 (2-8)		0 (0-1)		0 (0-0)		0 (0-0)	
2007	Ecuador	15 (10-19)		1 (1-1)		4 (2-7)		0 (0-0)		0 (0-0)		0 (0-0)	
2008	Ecuador	2 (1-3)		0 (0-0)		1 (0-1)		0 (0-0)		0 (0-0)		0 (0-0)	
2009	Ecuador	2 (1-3)		0 (0-0)		1 (0-1)		0 (0-0)		0 (0-0)		0 (0-0)	
2010	Ecuador	2 (1-3)		0 (0-0)		1 (0-1)		0 (0-0)		0 (0-0)		0 (0-0)	
2011	Ecuador	2 (1-3)		0 (0-0)		1 (0-1)		0 (0-0)		0 (0-0)		0 (0-0)	
2012	Ecuador	2 (2-3)		0 (0-0)		1 (0-1)		0 (0-0)		0 (0-0)		0 (0-0)	
2013	Ecuador	2 (1-3)		0 (0-0)		1 (0-1)		0 (0-0)		0 (0-0)		0 (0-0)	
2014	Ecuador	2 (1-3)		0 (0-0)		1 (0-1)		0 (0-0)		0 (0-0)		0 (0-0)	
2015	Ecuador	2 (1-3)	0 (0-0)	0 (0-0)	0 (0-0)	1 (0-1)	0 (0-0)	0 (0-0)	0 (0-0)	0 (0-0)	0 (0-0)	0 (0-0)	0 (0-0)
2000	Egypt	2090 (1470-2746)		27 (19-35)		225 (83-360)		3 (1-5)		2 (1-3)		0 (0-0)	
2001	Egypt	1938 (1363-2547)		24 (17-32)		209 (78-333)		3 (1-4)		2 (1-3)		0 (0-0)	
2002	Egypt	1794 (1262-2357)		22 (16-29)		194 (74-307)		2 (1-4)		2 (1-2)		0 (0-0)	
2003	Egypt	1661 (1168-2182)		20 (14-26)		180 (69-283)		2 (1-3)		1 (1-2)		0 (0-0)	
2004	Egypt	1554 (1093-2042)		18 (13-24)		169 (62-269)		2 (1-3)		1 (0-2)		0 (0-0)	
2005	Egypt	1436 (1010-1886)		17 (12-22)		156 (56-253)		2 (1-3)		1 (0-2)		0 (0-0)	
2006	Egypt	1341 (943-1762)		15 (11-20)		147 (54-234)		2 (1-3)		1 (0-2)		0 (0-0)	
2007	Egypt	1265 (890-1662)		14 (10-19)		139 (53-220)		2 (1-2)		1 (0-2)		0 (0-0)	
2008	Egypt	1185 (834-1557)		13 (9-17)		130 (52-204)		1 (1-2)		1 (0-2)		0 (0-0)	
2009	Egypt	1131 (796-1486)		12 (9-16)		124 (49-196)		1 (1-2)		1 (0-2)		0 (0-0)	
2010	Egypt	1113 (783-1463)		12 (8-15)		121 (20-206)		1 (0-2)		1 (0-2)		0 (0-0)	
2011	Egypt	1114 (784-1464)		11 (8-15)		120 (19-205)		1 (0-2)		1 (0-2)		0 (0-0)	
2012	Egypt	1143 (804-1501)		11 (8-15)		121 (19-209)		1 (0-2)		1 (0-2)		0 (0-0)	
2013	Egypt	1170 (823-1538)		11 (8-14)		122 (19-212)		1 (0-2)		1 (0-2)		0 (0-0)	
2014	Egypt	221 (155-290)		2 (1-3)		29 (4-50)		0 (0-0)		0 (0-0)		0 (0-0)	
2015	Egypt	109 (77-143)	0 (0-0)	1 (1-1)	0 (0-0)	14 (2-25)	0 (0-0)	0 (0-0)	0 (0-0)	0 (0-0)	0 (0-0)	0 (0-0)	0 (0-0)
2000	El Salvador	132 (93-173)		19 (13-25)		28 (9-47)		4 (1-7)		0 (0-0)		0 (0-0)	
2001	El Salvador	115 (81-152)		17 (12-22)		25 (8-41)		4 (1-6)		0 (0-0)		0 (0-0)	
2002	El Salvador	19 (13-25)		3 (2-4)		7 (1-12)		1 (0-2)		0 (0-0)		0 (0-0)	
2003	El Salvador	8 (5-10)		1 (1-2)		3 (0-5)		0 (0-1)		0 (0-0)		0 (0-0)	
2004	El Salvador	8 (5-10)		1 (1-2)		3 (0-5)		0 (0-1)		0 (0-0)		0 (0-0)	
2005	El Salvador	7 (5-9)		1 (1-2)		3 (0-5)		0 (0-1)		0 (0-0)		0 (0-0)	
2006	El Salvador	6 (4-7)		1 (1-1)		2 (0-4)		0 (0-1)		0 (0-0)		0 (0-0)	
2007	El Salvador	1 (1-1)		0 (0-0)		0 (0-1)		0 (0-0)		0 (0-0)		0 (0-0)	
2008	El Salvador	1 (0-1)		0 (0-0)		0 (0-0)		0 (0-0)		0 (0-0)		0 (0-0)	
2009	El Salvador	1 (0-1)		0 (0-0)		0 (0-0)		0 (0-0)		0 (0-0)		0 (0-0)	
2010	El Salvador	1 (0-1)		0 (0-0)		0 (0-0)		0 (0-0)		0 (0-0)		0 (0-0)	
2011	El Salvador	1 (0-1)		0 (0-0)		0 (0-0)		0 (0-0)		0 (0-0)		0 (0-0)	
2012	El Salvador	1 (0-1)		0 (0-0)		0 (0-0)		0 (0-0)		0 (0-0)		0 (0-0)	
2013	El Salvador	1 (0-1)		0 (0-0)		0 (0-0)		0 (0-0)		0 (0-0)		0 (0-0)	
2014	El Salvador	1 (0-1)		0 (0-0)		0 (0-0)		0 (0-0)		0 (0-0)		0 (0-0)	

Hib = *Haemophilus influenzae* type b; npnm = non-pneumonia, non-meningitis; HIV = deaths only in HIV-infected children; Uncertainty range provided in parentheses.

Year	Country	Hib pneumonia deaths (UR)	Hib pneumonia deaths, HIV (UR)	Hib pneumonia mortality rate (UR)	Hib pneumonia mortality rate, HIV (UR)	Hib meningitis deaths (UR)	Hib meningitis deaths, HIV (UR)	Hib meningitis mortality rate (UR)	Hib meningitis mortality rate, HIV (UR)	Hib NPNM deaths (UR)	Hib NPNM deaths, HIV (UR)	Hib NPNM mortality rate (UR)	Hib NPNM mortality rate, HIV (UR)
2015	El Salvador	1 (0-1)	0 (0-0)	0 (0-0)	0 (0-0)	0 (0-0)	0 (0-0)	0 (0-0)	0 (0-0)	0 (0-0)	0 (0-0)	0 (0-0)	0 (0-0)
2000	Equatorial Guinea	69 (49-91)		80 (56-105)		15 (7-22)		17 (8-25)		0 (0-0)		0 (0-0)	
2001	Equatorial Guinea	66 (47-87)		75 (53-98)		13 (5-22)		15 (5-24)		0 (0-0)		0 (0-0)	
2002	Equatorial Guinea	62 (44-82)		68 (48-90)		12 (4-19)		13 (5-21)		0 (0-0)		0 (0-0)	
2003	Equatorial Guinea	57 (40-75)		61 (43-81)		11 (4-17)		12 (4-19)		0 (0-0)		0 (0-0)	
2004	Equatorial Guinea	53 (37-70)		56 (39-74)		10 (3-15)		10 (3-16)		0 (0-0)		0 (0-0)	
2005	Equatorial Guinea	52 (37-68)		54 (38-71)		9 (3-15)		9 (3-15)		0 (0-0)		0 (0-0)	
2006	Equatorial Guinea	55 (39-72)		55 (39-73)		9 (3-15)		9 (3-15)		0 (0-0)		0 (0-0)	
2007	Equatorial Guinea	60 (42-79)		58 (41-77)		10 (3-16)		10 (3-15)		0 (0-0)		0 (0-0)	
2008	Equatorial Guinea	64 (45-83)		60 (42-79)		10 (4-16)		10 (3-16)		0 (0-0)		0 (0-0)	
2009	Equatorial Guinea	65 (46-86)		60 (42-79)		11 (4-17)		10 (3-16)		0 (0-0)		0 (0-0)	
2010	Equatorial Guinea	66 (46-86)		59 (41-77)		11 (4-17)		10 (3-15)		0 (0-0)		0 (0-0)	
2011	Equatorial Guinea	64 (45-84)		56 (39-73)		11 (4-17)		9 (3-15)		0 (0-0)		0 (0-0)	
2012	Equatorial Guinea	61 (43-80)		52 (36-68)		10 (4-17)		9 (3-14)		0 (0-0)		0 (0-0)	
2013	Equatorial Guinea	62 (44-81)		51 (36-67)		11 (4-17)		9 (3-14)		0 (0-0)		0 (0-0)	
2014	Equatorial Guinea	56 (39-74)		45 (32-60)		10 (4-15)		8 (3-12)		0 (0-0)		0 (0-0)	
2015	Equatorial Guinea	46 (32-60)	3 (2-4)	36 (26-48)	3 (2-3)	8 (3-13)	1 (0-1)	6 (2-10)	0 (0-1)	0 (0-0)	0 (0-0)	0 (0-0)	0 (0-0)
2000	Eritrea	321 (226-422)		58 (41-77)		131 (43-212)		24 (8-38)		1 (0-1)		0 (0-0)	
2001	Eritrea	320 (225-421)		57 (40-74)		132 (43-213)		23 (8-38)		1 (0-1)		0 (0-0)	
2002	Eritrea	320 (225-421)		54 (38-72)		133 (44-215)		23 (7-37)		1 (0-1)		0 (0-0)	
2003	Eritrea	321 (226-422)		52 (37-69)		136 (45-219)		22 (7-36)		1 (0-1)		0 (0-0)	
2004	Eritrea	322 (226-423)		50 (35-66)		138 (41-230)		22 (6-36)		1 (0-2)		0 (0-0)	
2005	Eritrea	320 (225-420)		48 (34-63)		139 (41-231)		21 (6-35)		1 (0-2)		0 (0-0)	
2006	Eritrea	317 (223-417)		45 (32-60)		139 (41-233)		20 (6-33)		1 (0-2)		0 (0-0)	
2007	Eritrea	312 (220-410)		43 (30-57)		139 (41-232)		19 (6-32)		1 (0-2)		0 (0-0)	
2008	Eritrea	46 (32-60)		6 (4-8)		21 (6-34)		3 (1-5)		0 (0-0)		0 (0-0)	
2009	Eritrea	18 (12-23)		2 (2-3)		8 (2-13)		1 (0-2)		0 (0-0)		0 (0-0)	
2010	Eritrea	17 (12-22)		2 (2-3)		8 (2-13)		1 (0-2)		0 (0-0)		0 (0-0)	
2011	Eritrea	16 (12-22)		2 (1-3)		8 (2-12)		1 (0-2)		0 (0-0)		0 (0-0)	
2012	Eritrea	20 (14-26)		3 (2-3)		9 (3-15)		1 (0-2)		0 (0-0)		0 (0-0)	
2013	Eritrea	3 (2-3)		0 (0-0)		1 (0-2)		0 (0-0)		0 (0-0)		0 (0-0)	
2014	Eritrea	2 (2-3)		0 (0-0)		1 (0-2)		0 (0-0)		0 (0-0)		0 (0-0)	
2015	Eritrea	2 (2-3)	0 (0-0)	0 (0-0)	0 (0-0)	1 (0-2)	0 (0-0)	0 (0-0)	0 (0-0)	0 (0-0)	0 (0-0)	0 (0-0)	0 (0-0)
2000	Estonia	1 (1-1)		1 (1-2)		0 (0-0)		0 (0-1)		0 (0-0)		0 (0-0)	
2001	Estonia	0 (0-0)		1 (0-1)		0 (0-1)		1 (0-1)		0 (0-0)		0 (0-0)	
2002	Estonia	1 (1-1)		2 (1-2)		0 (0-1)		1 (0-1)		0 (0-0)		0 (0-0)	
2003	Estonia	1 (0-1)		1 (1-1)		0 (0-0)		0 (0-1)		0 (0-0)		0 (0-0)	
2004	Estonia	1 (0-1)		1 (1-1)		0 (0-0)		0 (0-1)		0 (0-0)		0 (0-0)	
2005	Estonia	0 (0-0)		0 (0-0)		0 (0-0)		0 (0-0)		0 (0-0)		0 (0-0)	
2006	Estonia	0 (0-0)		0 (0-0)		0 (0-0)		0 (0-0)		0 (0-0)		0 (0-0)	
2007	Estonia	0 (0-0)		0 (0-0)		0 (0-0)		0 (0-0)		0 (0-0)		0 (0-0)	
2008	Estonia	0 (0-0)		0 (0-0)		0 (0-0)		0 (0-0)		0 (0-0)		0 (0-0)	
2009	Estonia	0 (0-0)		0 (0-0)		0 (0-0)		0 (0-0)		0 (0-0)		0 (0-0)	
2010	Estonia	0 (0-0)		0 (0-0)		0 (0-0)		0 (0-0)		0 (0-0)		0 (0-0)	
2011	Estonia	0 (0-0)		0 (0-0)		0 (0-0)		0 (0-0)		0 (0-0)		0 (0-0)	
2012	Estonia	0 (0-0)		0 (0-0)		0 (0-0)		0 (0-0)		0 (0-0)		0 (0-0)	
2013	Estonia	0 (0-0)		0 (0-0)		0 (0-0)		0 (0-0)		0 (0-0)		0 (0-0)	
2014	Estonia	0 (0-0)		0 (0-0)		0 (0-0)		0 (0-0)		0 (0-0)		0 (0-0)	
2015	Estonia	0 (0-0)	0 (0-0)	0 (0-0)	0 (0-0)	0 (0-0)	0 (0-0)	0 (0-0)	0 (0-0)	0 (0-0)	0 (0-0)	0 (0-0)	0 (0-0)
2000	Ethiopia	11047 (7770-14515)		91 (64-119)		5734 (1541-9572)		47 (13-78)		23 (6-39)		0 (0-0)	
2001	Ethiopia	10756 (7565-14132)		86 (60-112)		5557 (1499-9272)		44 (12-74)		23 (6-38)		0 (0-0)	
2002	Ethiopia	10261 (7216-13482)		80 (56-105)		5271 (1427-8788)		41 (11-69)		21 (6-36)		0 (0-0)	
2003	Ethiopia	9643 (6782-12670)		74 (52-98)		4914 (1336-8187)		38 (10-63)		20 (5-33)		0 (0-0)	
2004	Ethiopia	8972 (6310-11788)		68 (48-90)		4509 (1231-7508)		34 (9-57)		18 (5-31)		0 (0-0)	
2005	Ethiopia	8294 (5833-10897)		63 (44-82)		4095 (1123-6816)		31 (8-51)		17 (5-28)		0 (0-0)	
2006	Ethiopia	7964 (5601-10464)		60 (42-79)		3855 (1066-6407)		29 (8-48)		16 (4-26)		0 (0-0)	
2007	Ethiopia	4117 (2896-5410)		31 (22-40)		1952 (545-3239)		15 (4-24)		8 (2-13)		0 (0-0)	
2008	Ethiopia	937 (659-1231)		7 (5-9)		435 (123-722)		3 (1-5)		2 (0-3)		0 (0-0)	
2009	Ethiopia	856 (602-1125)		6 (4-8)		390 (111-646)		3 (1-5)		2 (0-3)		0 (0-0)	
2010	Ethiopia	742 (522-975)		5 (4-7)		332 (95-548)		2 (1-4)		1 (0-2)		0 (0-0)	
2011	Ethiopia	666 (468-875)		5 (3-6)		292 (75-498)		2 (1-4)		2 (1-4)		0 (0-0)	
2012	Ethiopia	51 (36-67)		0 (0-0)		22 (6-37)		0 (0-0)		0 (0-0)		0 (0-0)	
2013	Ethiopia	49 (34-64)		0 (0-0)		21 (5-35)		0 (0-0)		0 (0-0)		0 (0-0)	
2014	Ethiopia	48 (34-63)		0 (0-0)		20 (5-34)		0 (0-0)		0 (0-0)		0 (0-0)	
2015	Ethiopia	46 (32-60)	0 (0-0)	0 (0-0)	0 (0-0)	19 (5-32)	0 (0-0)	0 (0-0)	0 (0-0)	0 (0-0)	0 (0-0)	0 (0-0)	0 (0-0)
2000	Fiji	1 (0-1)		1 (0-1)		0 (0-1)		0 (0-1)		0 (0-0)		0 (0-0)	
2001	Fiji	1 (0-1)		1 (0-1)		0 (0-0)		0 (0-1)		0 (0-0)		0 (0-0)	
2002	Fiji	0 (0-0)		0 (0-0)		0 (0-0)		0 (0-0)		0 (0-0)		0 (0-0)	
2003	Fiji	0 (0-0)		0 (0-0)		0 (0-0)		0 (0-0)		0 (0-0)		0 (0-0)	
2004	Fiji	0 (0-0)		0 (0-0)		0 (0-0)		0 (0-0)		0 (0-0)		0 (0-0)	
2005	Fiji	0 (0-0)		0 (0-0)		0 (0-0)		0 (0-0)		0 (0-0)		0 (0-0)	
2006	Fiji	0 (0-0)		0 (0-0)		0 (0-0)		0 (0-0)		0 (0-0)		0 (0-0)	
2007	Fiji	0 (0-0)		0 (0-0)		0 (0-0)		0 (0-0)		0 (0-0)		0 (0-0)	
2008	Fiji	0 (0-0)		0 (0-0)		0 (0-0)		0 (0-0)		0 (0-0)		0 (0-0)	
2009	Fiji	0 (0-0)		0 (0-0)		0 (0-0)		0 (0-0)		0 (0-0)		0 (0-0)	
2010	Fiji	0 (0-0)		0 (0-0)		0 (0-0)		0 (0-0)		0 (0-0)		0 (0-0)	
2011	Fiji	0 (0-0)		0 (0-0)		0 (0-0)		0 (0-0)		0 (0-0)		0 (0-0)	
2012	Fiji	0 (0-0)		0 (0-0)		0 (0-0)		0 (0-0)		0 (0-0)		0 (0-0)	
2013	Fiji	0 (0-0)		0 (0-0)		0 (0-0)		0 (0-0)		0 (0-0)		0 (0-0)	
2014	Fiji	0 (0-0)		0 (0-0)		0 (0-0)		0 (0-0)		0 (0-0)		0 (0-0)	
2015	Fiji	0 (0-0)	0 (0-0)	0 (0-0)	0 (0-0)	0 (0-0)	0 (0-0)	0 (0-0)	0 (0-0)	0 (0-0)	0 (0-0)	0 (0-0)	0 (0-0)
2000	Finland	0 (0-0)		0 (0-0)		0 (0-0)		0 (0-0)		0 (0-0)		0 (0-0)	
2001	Finland	0 (0-0)		0 (0-0)		0 (0-0)		0 (0-0)		0 (0-0)		0 (0-0)	
2002	Finland	0 (0-0)		0 (0-0)		0 (0-0)		0 (0-0)		0 (0-0)		0 (0-0)	
2003	Finland	0 (0-0)		0 (0-0)		0 (0-0)		0 (0-0)		0 (0-0)		0 (0-0)	
2004	Finland	0 (0-0)		0 (0-0)		0 (0-0)		0 (0-0)		0 (0-0)		0 (0-0)	
2005	Finland	0 (0-0)		0 (0-0)		0 (0-0)		0 (0-0)		0 (0-0)		0 (0-0)	
2006	Finland	0 (0-0)		0 (0-0)		0 (0-0)		0 (0-0)		0 (0-0)		0 (0-0)	

Hib = *Haemophilus influenzae* type b; npnm = non-pneumonia, non-meningitis; HIV = deaths only in HIV-infected children; Uncertainty range provided in parentheses.

Year	Country	Hib pneumonia deaths (UR)	Hib pneumonia deaths, HIV (UR)	Hib pneumonia mortality rate (UR)	Hib pneumonia mortality rate, HIV (UR)	Hib meningitis deaths (UR)	Hib meningitis deaths, HIV (UR)	Hib meningitis mortality rate (UR)	Hib meningitis mortality rate, HIV (UR)	Hib NPNM deaths (UR)	Hib NPNM deaths, HIV (UR)	Hib NPNM mortality rate (UR)	Hib NPNM mortality rate, HIV (UR)
2007	Finland	0 (0-0)		0 (0-0)		0 (0-0)		0 (0-0)		0 (0-0)		0 (0-0)	
2008	Finland	0 (0-0)		0 (0-0)		0 (0-0)		0 (0-0)		0 (0-0)		0 (0-0)	
2009	Finland	0 (0-0)		0 (0-0)		0 (0-0)		0 (0-0)		0 (0-0)		0 (0-0)	
2010	Finland	0 (0-0)		0 (0-0)		0 (0-0)		0 (0-0)		0 (0-0)		0 (0-0)	
2011	Finland	0 (0-0)		0 (0-0)		0 (0-0)		0 (0-0)		0 (0-0)		0 (0-0)	
2012	Finland	0 (0-0)		0 (0-0)		0 (0-0)		0 (0-0)		0 (0-0)		0 (0-0)	
2013	Finland	0 (0-0)		0 (0-0)		0 (0-0)		0 (0-0)		0 (0-0)		0 (0-0)	
2014	Finland	0 (0-0)		0 (0-0)		0 (0-0)		0 (0-0)		0 (0-0)		0 (0-0)	
2015	Finland	0 (0-0)	0 (0-0)	0 (0-0)	0 (0-0)	0 (0-0)	0 (0-0)	0 (0-0)	0 (0-0)	0 (0-0)	0 (0-0)	0 (0-0)	0 (0-0)
2000	France	1 (1-1)		0 (0-0)		2 (1-4)		0 (0-0)		0 (0-0)		0 (0-0)	
2001	France	1 (1-1)		0 (0-0)		2 (1-3)		0 (0-0)		0 (0-0)		0 (0-0)	
2002	France	1 (1-1)		0 (0-0)		2 (1-3)		0 (0-0)		0 (0-0)		0 (0-0)	
2003	France	0 (0-0)		0 (0-0)		0 (0-0)		0 (0-0)		0 (0-0)		0 (0-0)	
2004	France	0 (0-0)		0 (0-0)		0 (0-0)		0 (0-0)		0 (0-0)		0 (0-0)	
2005	France	0 (0-0)		0 (0-0)		0 (0-0)		0 (0-0)		0 (0-0)		0 (0-0)	
2006	France	0 (0-0)		0 (0-0)		0 (0-0)		0 (0-0)		0 (0-0)		0 (0-0)	
2007	France	0 (0-0)		0 (0-0)		0 (0-0)		0 (0-0)		0 (0-0)		0 (0-0)	
2008	France	0 (0-0)		0 (0-0)		0 (0-0)		0 (0-0)		0 (0-0)		0 (0-0)	
2009	France	0 (0-0)		0 (0-0)		0 (0-0)		0 (0-0)		0 (0-0)		0 (0-0)	
2010	France	0 (0-0)		0 (0-0)		0 (0-0)		0 (0-0)		0 (0-0)		0 (0-0)	
2011	France	0 (0-0)		0 (0-0)		0 (0-0)		0 (0-0)		0 (0-0)		0 (0-0)	
2012	France	0 (0-0)		0 (0-0)		0 (0-0)		0 (0-0)		0 (0-0)		0 (0-0)	
2013	France	0 (0-0)		0 (0-0)		0 (0-0)		0 (0-0)		0 (0-0)		0 (0-0)	
2014	France	0 (0-0)		0 (0-0)		0 (0-0)		0 (0-0)		0 (0-0)		0 (0-0)	
2015	France	0 (0-0)	0 (0-0)	0 (0-0)	0 (0-0)	0 (0-0)	0 (0-0)	0 (0-0)	0 (0-0)	0 (0-0)	0 (0-0)	0 (0-0)	0 (0-0)
2000	Gabon	48 (34-63)		27 (19-35)		10 (3-15)		5 (2-8)		0 (0-0)		0 (0-0)	
2001	Gabon	50 (35-66)		27 (19-36)		10 (3-16)		5 (2-9)		0 (0-0)		0 (0-0)	
2002	Gabon	52 (36-68)		28 (20-36)		10 (4-16)		6 (2-9)		0 (0-0)		0 (0-0)	
2003	Gabon	53 (37-70)		28 (20-37)		11 (4-17)		6 (2-9)		0 (0-0)		0 (0-0)	
2004	Gabon	54 (38-71)		29 (20-38)		11 (4-17)		6 (2-9)		0 (0-0)		0 (0-0)	
2005	Gabon	55 (39-72)		29 (20-38)		11 (4-18)		6 (2-9)		0 (0-0)		0 (0-0)	
2006	Gabon	55 (39-73)		28 (20-37)		11 (4-18)		6 (2-9)		0 (0-0)		0 (0-0)	
2007	Gabon	56 (39-73)		28 (20-37)		11 (4-18)		6 (2-9)		0 (0-0)		0 (0-0)	
2008	Gabon	55 (39-72)		27 (19-36)		11 (4-18)		5 (2-9)		0 (0-0)		0 (0-0)	
2009	Gabon	55 (39-72)		27 (19-35)		11 (4-18)		5 (2-9)		0 (0-0)		0 (0-0)	
2010	Gabon	18 (12-23)		8 (6-11)		4 (1-6)		2 (1-3)		0 (0-0)		0 (0-0)	
2011	Gabon	6 (4-8)		3 (2-3)		1 (0-2)		1 (0-1)		0 (0-0)		0 (0-0)	
2012	Gabon	5 (4-7)		2 (2-3)		1 (0-2)		0 (0-1)		0 (0-0)		0 (0-0)	
2013	Gabon	5 (4-7)		2 (2-3)		1 (0-2)		0 (0-1)		0 (0-0)		0 (0-0)	
2014	Gabon	6 (4-8)		3 (2-3)		1 (0-2)		0 (0-1)		0 (0-0)		0 (0-0)	
2015	Gabon	0 (0-1)	0 (0-0)	0 (0-0)	0 (0-0)	0 (0-0)	0 (0-0)	0 (0-0)	0 (0-0)	0 (0-0)	0 (0-0)	0 (0-0)	0 (0-0)
2000	Gambia	11 (8-15)		5 (3-6)		4 (2-7)		2 (1-3)		0 (0-0)		0 (0-0)	
2001	Gambia	12 (8-15)		5 (3-7)		5 (2-7)		2 (1-3)		0 (0-0)		0 (0-0)	
2002	Gambia	12 (8-15)		5 (3-6)		5 (2-7)		2 (1-3)		0 (0-0)		0 (0-0)	
2003	Gambia	1 (1-2)		1 (0-1)		1 (0-1)		0 (0-0)		0 (0-0)		0 (0-0)	
2004	Gambia	1 (1-2)		1 (0-1)		1 (0-1)		0 (0-0)		0 (0-0)		0 (0-0)	
2005	Gambia	1 (1-2)		1 (0-1)		1 (0-1)		0 (0-0)		0 (0-0)		0 (0-0)	
2006	Gambia	1 (1-2)		1 (0-1)		1 (0-1)		0 (0-0)		0 (0-0)		0 (0-0)	
2007	Gambia	1 (1-2)		0 (0-1)		1 (0-1)		0 (0-0)		0 (0-0)		0 (0-0)	
2008	Gambia	1 (1-2)		0 (0-1)		1 (0-1)		0 (0-0)		0 (0-0)		0 (0-0)	
2009	Gambia	1 (1-2)		0 (0-1)		1 (0-1)		0 (0-0)		0 (0-0)		0 (0-0)	
2010	Gambia	1 (1-2)		0 (0-1)		1 (0-1)		0 (0-0)		0 (0-0)		0 (0-0)	
2011	Gambia	1 (1-2)		0 (0-1)		1 (0-1)		0 (0-0)		0 (0-0)		0 (0-0)	
2012	Gambia	1 (1-2)		0 (0-1)		1 (0-1)		0 (0-0)		0 (0-0)		0 (0-0)	
2013	Gambia	1 (1-2)		0 (0-1)		1 (0-1)		0 (0-0)		0 (0-0)		0 (0-0)	
2014	Gambia	1 (1-2)		0 (0-1)		1 (0-1)		0 (0-0)		0 (0-0)		0 (0-0)	
2015	Gambia	1 (1-2)	0 (0-0)	0 (0-1)	0 (0-0)	1 (0-1)	0 (0-0)	0 (0-0)	0 (0-0)	0 (0-0)	0 (0-0)	0 (0-0)	0 (0-0)
2000	Georgia	36 (25-47)		13 (9-17)		12 (7-18)		4 (3-7)		0 (0-0)		0 (0-0)	
2001	Georgia	31 (22-41)		12 (9-16)		11 (6-16)		4 (2-6)		0 (0-0)		0 (0-0)	
2002	Georgia	28 (20-36)		11 (8-15)		10 (5-14)		4 (2-6)		0 (0-0)		0 (0-0)	
2003	Georgia	25 (18-33)		11 (8-14)		9 (2-14)		4 (1-6)		0 (0-0)		0 (0-0)	
2004	Georgia	23 (16-30)		10 (7-13)		8 (2-13)		3 (1-6)		0 (0-0)		0 (0-0)	
2005	Georgia	22 (15-28)		9 (7-12)		8 (1-13)		3 (1-6)		0 (0-0)		0 (0-0)	
2006	Georgia	20 (14-26)		8 (6-11)		7 (1-12)		3 (0-5)		0 (0-0)		0 (0-0)	
2007	Georgia	18 (13-24)		7 (5-9)		7 (1-11)		3 (0-5)		0 (0-0)		0 (0-0)	
2008	Georgia	16 (11-21)		6 (5-8)		6 (1-10)		2 (0-4)		0 (0-0)		0 (0-0)	
2009	Georgia	15 (10-19)		6 (4-8)		5 (1-9)		2 (0-4)		0 (0-0)		0 (0-0)	
2010	Georgia	5 (3-6)		2 (1-2)		3 (0-5)		1 (0-2)		0 (0-0)		0 (0-0)	
2011	Georgia	1 (1-1)		0 (0-0)		1 (0-1)		0 (0-0)		0 (0-0)		0 (0-0)	
2012	Georgia	1 (1-1)		0 (0-0)		1 (0-1)		0 (0-0)		0 (0-0)		0 (0-0)	
2013	Georgia	1 (1-1)		0 (0-0)		1 (0-1)		0 (0-0)		0 (0-0)		0 (0-0)	
2014	Georgia	1 (1-1)		0 (0-0)		1 (0-1)		0 (0-0)		0 (0-0)		0 (0-0)	
2015	Georgia	0 (0-0)	0 (0-0)	0 (0-0)	0 (0-0)	0 (0-0)	0 (0-0)	0 (0-0)	0 (0-0)	0 (0-0)	0 (0-0)	0 (0-0)	0 (0-0)
2000	Germany	0 (0-0)		0 (0-0)		0 (0-0)		0 (0-0)		0 (0-0)		0 (0-0)	
2001	Germany	0 (0-0)		0 (0-0)		0 (0-0)		0 (0-0)		0 (0-0)		0 (0-0)	
2002	Germany	0 (0-0)		0 (0-0)		0 (0-0)		0 (0-0)		0 (0-0)		0 (0-0)	
2003	Germany	0 (0-0)		0 (0-0)		0 (0-0)		0 (0-0)		0 (0-0)		0 (0-0)	
2004	Germany	0 (0-0)		0 (0-0)		0 (0-0)		0 (0-0)		0 (0-0)		0 (0-0)	
2005	Germany	0 (0-0)		0 (0-0)		0 (0-0)		0 (0-0)		0 (0-0)		0 (0-0)	
2006	Germany	0 (0-0)		0 (0-0)		0 (0-0)		0 (0-0)		0 (0-0)		0 (0-0)	
2007	Germany	0 (0-0)		0 (0-0)		0 (0-0)		0 (0-0)		0 (0-0)		0 (0-0)	
2008	Germany	0 (0-0)		0 (0-0)		0 (0-0)		0 (0-0)		0 (0-0)		0 (0-0)	
2009	Germany	0 (0-0)		0 (0-0)		0 (0-0)		0 (0-0)		0 (0-0)		0 (0-0)	
2010	Germany	0 (0-0)		0 (0-0)		0 (0-0)		0 (0-0)		0 (0-0)		0 (0-0)	
2011	Germany	0 (0-0)		0 (0-0)		0 (0-0)		0 (0-0)		0 (0-0)		0 (0-0)	
2012	Germany	0 (0-0)		0 (0-0)		0 (0-0)		0 (0-0)		0 (0-0)		0 (0-0)	
2013	Germany	0 (0-0)		0 (0-0)		0 (0-0)		0 (0-0)		0 (0-0)		0 (0-0)	
2014	Germany	0 (0-0)		0 (0-0)		0 (0-0)		0 (0-0)		0 (0-0)		0 (0-0)	

Hib = *Haemophilus influenzae* type b; npnm = non-pneumonia, non-meningitis; HIV = deaths only in HIV-infected children; Uncertainty range provided in parentheses.

Year	Country	Hib pneumonia deaths (UR)	Hib pneumonia deaths, HIV (UR)	Hib pneumonia mortality rate (UR)	Hib pneumonia mortality rate, HIV (UR)	Hib meningitis deaths (UR)	Hib meningitis deaths, HIV (UR)	Hib meningitis mortality rate (UR)	Hib meningitis mortality rate, HIV (UR)	Hib NPNM deaths (UR)	Hib NPNM deaths, HIV (UR)	Hib NPNM mortality rate (UR)	Hib NPNM mortality rate, HIV (UR)
2015	Germany	0 (0-0)	0 (0-0)	0 (0-0)	0 (0-0)	0 (0-0)	0 (0-0)	0 (0-0)	0 (0-0)	0 (0-0)	0 (0-0)	0 (0-0)	0 (0-0)
2000	Ghana	955 (672-1255)		34 (24-45)		346 (124-548)		12 (4-19)		1 (1-2)		0 (0-0)	
2001	Ghana	984 (692-1293)		34 (24-45)		354 (123-565)		12 (4-20)		1 (1-2)		0 (0-0)	
2002	Ghana	171 (120-225)		6 (4-8)		61 (21-98)		2 (1-3)		0 (0-0)		0 (0-0)	
2003	Ghana	103 (72-135)		3 (2-5)		37 (12-59)		1 (0-2)		0 (0-0)		0 (0-0)	
2004	Ghana	108 (76-141)		4 (2-5)		38 (12-62)		1 (0-2)		0 (0-0)		0 (0-0)	
2005	Ghana	103 (73-136)		3 (2-4)		36 (11-59)		1 (0-2)		0 (0-0)		0 (0-0)	
2006	Ghana	107 (76-141)		3 (2-4)		38 (11-62)		1 (0-2)		0 (0-0)		0 (0-0)	
2007	Ghana	12 (8-15)		0 (0-0)		4 (1-7)		0 (0-0)		0 (0-0)		0 (0-0)	
2008	Ghana	11 (8-15)		0 (0-0)		4 (1-6)		0 (0-0)		0 (0-0)		0 (0-0)	
2009	Ghana	11 (8-15)		0 (0-0)		4 (1-6)		0 (0-0)		0 (0-0)		0 (0-0)	
2010	Ghana	11 (8-14)		0 (0-0)		4 (1-6)		0 (0-0)		0 (0-0)		0 (0-0)	
2011	Ghana	11 (8-15)		0 (0-0)		4 (1-6)		0 (0-0)		0 (0-0)		0 (0-0)	
2012	Ghana	11 (8-15)		0 (0-0)		4 (1-6)		0 (0-0)		0 (0-0)		0 (0-0)	
2013	Ghana	12 (8-15)		0 (0-0)		4 (1-6)		0 (0-0)		0 (0-0)		0 (0-0)	
2014	Ghana	12 (8-15)		0 (0-0)		4 (1-6)		0 (0-0)		0 (0-0)		0 (0-0)	
2015	Ghana	12 (8-15)	0 (0-0)	0 (0-0)	0 (0-0)	4 (1-6)	0 (0-0)	0 (0-0)	0 (0-0)	0 (0-0)	0 (0-0)	0 (0-0)	0 (0-0)
2000	Greece	0 (0-0)		0 (0-0)		0 (0-0)		0 (0-0)		0 (0-0)		0 (0-0)	
2001	Greece	0 (0-0)		0 (0-0)		0 (0-0)		0 (0-0)		0 (0-0)		0 (0-0)	
2002	Greece	0 (0-0)		0 (0-0)		0 (0-0)		0 (0-0)		0 (0-0)		0 (0-0)	
2003	Greece	0 (0-0)		0 (0-0)		0 (0-0)		0 (0-0)		0 (0-0)		0 (0-0)	
2004	Greece	0 (0-0)		0 (0-0)		0 (0-0)		0 (0-0)		0 (0-0)		0 (0-0)	
2005	Greece	0 (0-0)		0 (0-0)		0 (0-0)		0 (0-0)		0 (0-0)		0 (0-0)	
2006	Greece	0 (0-0)		0 (0-0)		0 (0-0)		0 (0-0)		0 (0-0)		0 (0-0)	
2007	Greece	0 (0-0)		0 (0-0)		0 (0-0)		0 (0-0)		0 (0-0)		0 (0-0)	
2008	Greece	0 (0-0)		0 (0-0)		0 (0-0)		0 (0-0)		0 (0-0)		0 (0-0)	
2009	Greece	0 (0-0)		0 (0-0)		0 (0-0)		0 (0-0)		0 (0-0)		0 (0-0)	
2010	Greece	0 (0-0)		0 (0-0)		0 (0-0)		0 (0-0)		0 (0-0)		0 (0-0)	
2011	Greece	0 (0-0)		0 (0-0)		0 (0-0)		0 (0-0)		0 (0-0)		0 (0-0)	
2012	Greece	0 (0-0)		0 (0-0)		0 (0-0)		0 (0-0)		0 (0-0)		0 (0-0)	
2013	Greece	0 (0-0)		0 (0-0)		0 (0-0)		0 (0-0)		0 (0-0)		0 (0-0)	
2014	Greece	0 (0-0)		0 (0-0)		0 (0-0)		0 (0-0)		0 (0-0)		0 (0-0)	
2015	Greece	0 (0-0)	0 (0-0)	0 (0-0)	0 (0-0)	0 (0-0)	0 (0-0)	0 (0-0)	0 (0-0)	0 (0-0)	0 (0-0)	0 (0-0)	0 (0-0)
2000	Grenada	0 (0-1)		5 (3-6)		0 (0-0)		0 (0-0)		0 (0-0)		0 (0-0)	
2001	Grenada	0 (0-0)		0 (0-0)		0 (0-0)		0 (0-0)		0 (0-0)		0 (0-0)	
2002	Grenada	0 (0-0)		0 (0-0)		0 (0-0)		0 (0-0)		0 (0-0)		0 (0-0)	
2003	Grenada	0 (0-0)		0 (0-0)		0 (0-0)		0 (0-0)		0 (0-0)		0 (0-0)	
2004	Grenada	0 (0-0)		0 (0-0)		0 (0-0)		0 (0-0)		0 (0-0)		0 (0-0)	
2005	Grenada	0 (0-0)		0 (0-0)		0 (0-0)		0 (0-0)		0 (0-0)		0 (0-0)	
2006	Grenada	0 (0-0)		0 (0-0)		0 (0-0)		0 (0-0)		0 (0-0)		0 (0-0)	
2007	Grenada	0 (0-0)		0 (0-0)		0 (0-0)		0 (0-0)		0 (0-0)		0 (0-0)	
2008	Grenada	0 (0-0)		0 (0-0)		0 (0-0)		0 (0-0)		0 (0-0)		0 (0-0)	
2009	Grenada	0 (0-0)		0 (0-0)		0 (0-0)		0 (0-0)		0 (0-0)		0 (0-0)	
2010	Grenada	0 (0-0)		0 (0-0)		0 (0-0)		0 (0-0)		0 (0-0)		0 (0-0)	
2011	Grenada	0 (0-0)		0 (0-0)		0 (0-0)		0 (0-0)		0 (0-0)		0 (0-0)	
2012	Grenada	0 (0-0)		0 (0-0)		0 (0-0)		0 (0-0)		0 (0-0)		0 (0-0)	
2013	Grenada	0 (0-0)		0 (0-0)		0 (0-0)		0 (0-0)		0 (0-0)		0 (0-0)	
2014	Grenada	0 (0-0)		0 (0-0)		0 (0-0)		0 (0-0)		0 (0-0)		0 (0-0)	
2015	Grenada	0 (0-0)	0 (0-0)	0 (0-0)	0 (0-0)	0 (0-0)	0 (0-0)	0 (0-0)	0 (0-0)	0 (0-0)	0 (0-0)	0 (0-0)	0 (0-0)
2000	Guatemala	635 (446-834)		34 (24-45)		179 (55-295)		10 (3-16)		1 (0-2)		0 (0-0)	
2001	Guatemala	612 (431-805)		33 (23-43)		173 (54-285)		9 (3-15)		1 (0-2)		0 (0-0)	
2002	Guatemala	590 (415-776)		31 (22-41)		167 (53-274)		9 (3-15)		1 (0-2)		0 (0-0)	
2003	Guatemala	567 (399-745)		30 (21-39)		161 (51-263)		8 (3-14)		1 (0-2)		0 (0-0)	
2004	Guatemala	547 (385-718)		28 (20-37)		155 (49-253)		8 (3-13)		1 (0-2)		0 (0-0)	
2005	Guatemala	85 (60-112)		4 (3-6)		24 (8-39)		1 (0-2)		0 (0-0)		0 (0-0)	
2006	Guatemala	42 (29-55)		2 (2-3)		12 (4-19)		1 (0-1)		0 (0-0)		0 (0-0)	
2007	Guatemala	43 (30-57)		2 (2-3)		12 (4-20)		1 (0-1)		0 (0-0)		0 (0-0)	
2008	Guatemala	33 (23-44)		2 (1-2)		9 (3-15)		0 (0-1)		0 (0-0)		0 (0-0)	
2009	Guatemala	34 (24-45)		2 (1-2)		10 (3-15)		0 (0-1)		0 (0-0)		0 (0-0)	
2010	Guatemala	4 (3-6)		0 (0-0)		1 (0-2)		0 (0-0)		0 (0-0)		0 (0-0)	
2011	Guatemala	4 (3-6)		0 (0-0)		1 (0-2)		0 (0-0)		0 (0-0)		0 (0-0)	
2012	Guatemala	4 (3-5)		0 (0-0)		1 (0-2)		0 (0-0)		0 (0-0)		0 (0-0)	
2013	Guatemala	4 (3-5)		0 (0-0)		1 (0-2)		0 (0-0)		0 (0-0)		0 (0-0)	
2014	Guatemala	4 (3-5)		0 (0-0)		1 (0-2)		0 (0-0)		0 (0-0)		0 (0-0)	
2015	Guatemala	4 (3-5)	0 (0-0)	0 (0-0)	0 (0-0)	1 (0-2)	0 (0-0)	0 (0-0)	0 (0-0)	0 (0-0)	0 (0-0)	0 (0-0)	0 (0-0)
2000	Guinea	1148 (808-1509)		76 (53-100)		503 (218-763)		33 (14-50)		2 (1-3)		0 (0-0)	
2001	Guinea	1148 (808-1509)		75 (52-98)		502 (222-756)		33 (14-49)		2 (1-3)		0 (0-0)	
2002	Guinea	1140 (802-1498)		73 (51-96)		497 (224-744)		32 (14-48)		2 (1-3)		0 (0-0)	
2003	Guinea	1129 (794-1483)		72 (50-94)		494 (149-809)		31 (9-51)		2 (1-3)		0 (0-0)	
2004	Guinea	1127 (793-1481)		70 (50-93)		494 (155-805)		31 (10-50)		2 (1-3)		0 (0-0)	
2005	Guinea	1126 (792-1480)		69 (49-91)		496 (160-801)		30 (10-49)		2 (1-3)		0 (0-0)	
2006	Guinea	1127 (792-1480)		68 (48-89)		497 (160-805)		30 (10-48)		2 (1-3)		0 (0-0)	
2007	Guinea	1127 (793-1481)		67 (47-87)		499 (160-808)		29 (9-48)		2 (1-3)		0 (0-0)	
2008	Guinea	1085 (763-1426)		63 (44-83)		481 (153-781)		28 (9-45)		2 (1-3)		0 (0-0)	
2009	Guinea	488 (343-641)		28 (20-36)		216 (69-351)		12 (4-20)		1 (0-1)		0 (0-0)	
2010	Guinea	134 (95-177)		7 (5-10)		59 (19-97)		3 (1-5)		0 (0-0)		0 (0-0)	
2011	Guinea	133 (94-175)		7 (5-10)		59 (19-96)		3 (1-5)		0 (0-0)		0 (0-0)	
2012	Guinea	134 (94-176)		7 (5-9)		59 (18-96)		3 (1-5)		0 (0-0)		0 (0-0)	
2013	Guinea	133 (93-174)		7 (5-9)		58 (18-95)		3 (1-5)		0 (0-0)		0 (0-0)	
2014	Guinea	155 (109-203)		8 (6-10)		67 (21-110)		3 (1-6)		0 (0-0)		0 (0-0)	
2015	Guinea	153 (108-201)	1 (1-1)	8 (5-10)	0 (0-0)	67 (21-109)	0 (0-1)	3 (1-5)	0 (0-0)	0 (0-0)	0 (0-0)	0 (0-0)	0 (0-0)
2000	Guinea-Bissau	196 (138-258)		91 (64-119)		78 (42-112)		36 (20-52)		0 (0-0)		0 (0-0)	
2001	Guinea-Bissau	203 (143-267)		92 (65-121)		82 (44-117)		37 (20-53)		0 (0-0)		0 (0-0)	
2002	Guinea-Bissau	208 (146-273)		93 (66-122)		84 (45-120)		38 (20-54)		0 (0-0)		0 (0-0)	
2003	Guinea-Bissau	211 (148-277)		93 (66-123)		86 (45-123)		38 (20-54)		0 (0-1)		0 (0-0)	
2004	Guinea-Bissau	210 (148-276)		92 (64-120)		86 (45-123)		38 (20-54)		0 (0-1)		0 (0-0)	
2005	Guinea-Bissau	206 (145-270)		88 (62-116)		85 (32-134)		36 (14-57)		0 (0-1)		0 (0-0)	
2006	Guinea-Bissau	199 (140-262)		84 (59-110)		82 (30-130)		34 (13-54)		0 (0-1)		0 (0-0)	

Hib = *Haemophilus influenzae* type b; npnm = non-pneumonia, non-meningitis; HIV = deaths only in HIV-infected children; Uncertainty range provided in parentheses.

Year	Country	Hib pneumonia deaths (UR)	Hib pneumonia deaths, HIV (UR)	Hib pneumonia mortality rate (UR)	Hib pneumonia mortality rate, HIV (UR)	Hib meningitis deaths (UR)	Hib meningitis deaths, HIV (UR)	Hib meningitis mortality rate (UR)	Hib meningitis mortality rate, HIV (UR)	Hib NPNM deaths (UR)	Hib NPNM deaths, HIV (UR)	Hib NPNM mortality rate (UR)	Hib NPNM mortality rate, HIV (UR)
2007	Guinea-Bissau	192 (135-252)		79 (56-104)		79 (29-125)		33 (12-52)		0 (0-1)		0 (0-0)	
2008	Guinea-Bissau	183 (129-241)		74 (52-98)		75 (27-119)		31 (11-48)		0 (0-0)		0 (0-0)	
2009	Guinea-Bissau	29 (21-38)		12 (8-15)		12 (4-19)		5 (2-8)		0 (0-0)		0 (0-0)	
2010	Guinea-Bissau	17 (12-22)		6 (5-8)		7 (2-11)		3 (1-4)		0 (0-0)		0 (0-0)	
2011	Guinea-Bissau	16 (11-21)		6 (4-8)		7 (2-10)		2 (1-4)		0 (0-0)		0 (0-0)	
2012	Guinea-Bissau	15 (11-20)		6 (4-8)		6 (2-10)		2 (1-4)		0 (0-0)		0 (0-0)	
2013	Guinea-Bissau	15 (11-20)		5 (4-7)		6 (2-10)		2 (1-4)		0 (0-0)		0 (0-0)	
2014	Guinea-Bissau	1 (1-2)		1 (0-1)		1 (0-1)		0 (0-0)		0 (0-0)		0 (0-0)	
2015	Guinea-Bissau	1 (1-2)	0 (0-0)	1 (0-1)	0 (0-0)	1 (0-1)	0 (0-0)	0 (0-0)	0 (0-0)	0 (0-0)	0 (0-0)	0 (0-0)	0 (0-0)
2000	Guyana	10 (7-13)		10 (7-14)		2 (1-3)		2 (1-4)		0 (0-0)		0 (0-0)	
2001	Guyana	1 (1-2)		2 (1-2)		1 (0-1)		1 (0-2)		0 (0-0)		0 (0-0)	
2002	Guyana	1 (1-1)		1 (1-1)		0 (0-1)		0 (0-1)		0 (0-0)		0 (0-0)	
2003	Guyana	1 (0-1)		1 (1-1)		0 (0-1)		0 (0-1)		0 (0-0)		0 (0-0)	
2004	Guyana	1 (1-1)		1 (1-1)		0 (0-1)		0 (0-1)		0 (0-0)		0 (0-0)	
2005	Guyana	1 (0-1)		1 (1-1)		0 (0-1)		0 (0-1)		0 (0-0)		0 (0-0)	
2006	Guyana	0 (0-0)		0 (0-0)		0 (0-0)		0 (0-0)		0 (0-0)		0 (0-0)	
2007	Guyana	0 (0-0)		0 (0-0)		0 (0-0)		0 (0-0)		0 (0-0)		0 (0-0)	
2008	Guyana	0 (0-0)		0 (0-0)		0 (0-0)		0 (0-0)		0 (0-0)		0 (0-0)	
2009	Guyana	0 (0-0)		0 (0-0)		0 (0-0)		0 (0-0)		0 (0-0)		0 (0-0)	
2010	Guyana	0 (0-0)		0 (0-0)		0 (0-0)		0 (0-0)		0 (0-0)		0 (0-0)	
2011	Guyana	0 (0-0)		0 (0-0)		0 (0-0)		0 (0-0)		0 (0-0)		0 (0-0)	
2012	Guyana	0 (0-0)		0 (0-0)		0 (0-0)		0 (0-0)		0 (0-0)		0 (0-0)	
2013	Guyana	0 (0-0)		0 (0-0)		0 (0-0)		0 (0-0)		0 (0-0)		0 (0-0)	
2014	Guyana	0 (0-0)		0 (0-0)		0 (0-0)		0 (0-0)		0 (0-0)		0 (0-0)	
2015	Guyana	0 (0-0)	0 (0-0)	0 (0-0)	0 (0-0)	0 (0-0)	0 (0-0)	0 (0-0)	0 (0-0)	0 (0-0)	0 (0-0)	0 (0-0)	0 (0-0)
2000	Haiti	934 (657-1227)		78 (55-102)		380 (94-639)		32 (8-53)		2 (0-3)		0 (0-0)	
2001	Haiti	894 (629-1175)		74 (52-97)		366 (91-614)		30 (8-51)		1 (0-3)		0 (0-0)	
2002	Haiti	853 (600-1121)		71 (50-93)		351 (88-587)		29 (7-49)		1 (0-2)		0 (0-0)	
2003	Haiti	814 (572-1069)		67 (47-89)		336 (85-563)		28 (7-47)		1 (0-2)		0 (0-0)	
2004	Haiti	781 (549-1026)		65 (46-85)		324 (82-542)		27 (7-45)		1 (0-2)		0 (0-0)	
2005	Haiti	760 (535-999)		63 (44-83)		316 (81-529)		26 (7-44)		1 (0-2)		0 (0-0)	
2006	Haiti	748 (526-982)		62 (44-81)		312 (80-521)		26 (7-43)		1 (0-2)		0 (0-0)	
2007	Haiti	738 (519-970)		61 (43-80)		308 (80-514)		25 (7-42)		1 (0-2)		0 (0-0)	
2008	Haiti	728 (512-957)		60 (42-79)		305 (79-508)		25 (7-42)		1 (0-2)		0 (0-0)	
2009	Haiti	717 (504-942)		59 (41-77)		300 (79-499)		25 (6-41)		1 (0-2)		0 (0-0)	
2010	Haiti	2217 (1559-2913)		181 (127-237)		928 (377-1448)		76 (31-118)		4 (2-6)		0 (0-0)	
2011	Haiti	685 (482-900)		56 (39-73)		286 (77-475)		23 (6-39)		1 (0-2)		0 (0-0)	
2012	Haiti	668 (470-878)		54 (38-71)		278 (75-461)		23 (6-37)		1 (0-2)		0 (0-0)	
2013	Haiti	201 (142-265)		16 (12-22)		83 (20-142)		7 (2-12)		1 (0-1)		0 (0-0)	
2014	Haiti	96 (68-126)		8 (6-10)		40 (10-67)		3 (1-6)		0 (0-1)		0 (0-0)	
2015	Haiti	80 (57-106)	0 (0-1)	7 (5-9)	0 (0-0)	33 (8-56)	0 (0-0)	3 (1-5)	0 (0-0)	0 (0-0)	0 (0-0)	0 (0-0)	0 (0-0)
2000	Honduras	33 (23-43)		4 (2-5)		10 (3-18)		1 (0-2)		0 (0-0)		0 (0-0)	
2001	Honduras	14 (10-19)		2 (1-2)		5 (1-8)		1 (0-1)		0 (0-0)		0 (0-0)	
2002	Honduras	13 (9-17)		1 (1-2)		4 (1-7)		0 (0-1)		0 (0-0)		0 (0-0)	
2003	Honduras	13 (9-17)		1 (1-2)		4 (1-7)		0 (0-1)		0 (0-0)		0 (0-0)	
2004	Honduras	11 (8-15)		1 (1-2)		4 (1-6)		0 (0-1)		0 (0-0)		0 (0-0)	
2005	Honduras	1 (1-2)		0 (0-0)		0 (0-1)		0 (0-0)		0 (0-0)		0 (0-0)	
2006	Honduras	1 (1-2)		0 (0-0)		0 (0-1)		0 (0-0)		0 (0-0)		0 (0-0)	
2007	Honduras	1 (1-2)		0 (0-0)		0 (0-1)		0 (0-0)		0 (0-0)		0 (0-0)	
2008	Honduras	1 (1-2)		0 (0-0)		0 (0-1)		0 (0-0)		0 (0-0)		0 (0-0)	
2009	Honduras	1 (1-1)		0 (0-0)		0 (0-1)		0 (0-0)		0 (0-0)		0 (0-0)	
2010	Honduras	1 (1-1)		0 (0-0)		0 (0-1)		0 (0-0)		0 (0-0)		0 (0-0)	
2011	Honduras	1 (1-1)		0 (0-0)		0 (0-1)		0 (0-0)		0 (0-0)		0 (0-0)	
2012	Honduras	1 (1-1)		0 (0-0)		0 (0-1)		0 (0-0)		0 (0-0)		0 (0-0)	
2013	Honduras	1 (1-1)		0 (0-0)		0 (0-1)		0 (0-0)		0 (0-0)		0 (0-0)	
2014	Honduras	1 (1-1)		0 (0-0)		0 (0-0)		0 (0-0)		0 (0-0)		0 (0-0)	
2015	Honduras	1 (1-1)	0 (0-0)	0 (0-0)	0 (0-0)	0 (0-0)	0 (0-0)	0 (0-0)	0 (0-0)	0 (0-0)	0 (0-0)	0 (0-0)	0 (0-0)
2000	Hungary	1 (0-1)		0 (0-0)		0 (0-0)		0 (0-0)		0 (0-0)		0 (0-0)	
2001	Hungary	0 (0-1)		0 (0-0)		0 (0-1)		0 (0-0)		0 (0-0)		0 (0-0)	
2002	Hungary	0 (0-0)		0 (0-0)		0 (0-0)		0 (0-0)		0 (0-0)		0 (0-0)	
2003	Hungary	0 (0-1)		0 (0-0)		0 (0-0)		0 (0-0)		0 (0-0)		0 (0-0)	
2004	Hungary	0 (0-0)		0 (0-0)		0 (0-0)		0 (0-0)		0 (0-0)		0 (0-0)	
2005	Hungary	0 (0-0)		0 (0-0)		0 (0-0)		0 (0-0)		0 (0-0)		0 (0-0)	
2006	Hungary	0 (0-0)		0 (0-0)		0 (0-0)		0 (0-0)		0 (0-0)		0 (0-0)	
2007	Hungary	0 (0-0)		0 (0-0)		0 (0-0)		0 (0-0)		0 (0-0)		0 (0-0)	
2008	Hungary	0 (0-0)		0 (0-0)		0 (0-0)		0 (0-0)		0 (0-0)		0 (0-0)	
2009	Hungary	0 (0-0)		0 (0-0)		0 (0-0)		0 (0-0)		0 (0-0)		0 (0-0)	
2010	Hungary	0 (0-0)		0 (0-0)		0 (0-0)		0 (0-0)		0 (0-0)		0 (0-0)	
2011	Hungary	0 (0-0)		0 (0-0)		0 (0-0)		0 (0-0)		0 (0-0)		0 (0-0)	
2012	Hungary	0 (0-0)		0 (0-0)		0 (0-0)		0 (0-0)		0 (0-0)		0 (0-0)	
2013	Hungary	0 (0-0)		0 (0-0)		0 (0-0)		0 (0-0)		0 (0-0)		0 (0-0)	
2014	Hungary	0 (0-0)		0 (0-0)		0 (0-0)		0 (0-0)		0 (0-0)		0 (0-0)	
2015	Hungary	0 (0-0)	0 (0-0)	0 (0-0)	0 (0-0)	0 (0-0)	0 (0-0)	0 (0-0)	0 (0-0)	0 (0-0)	0 (0-0)	0 (0-0)	0 (0-0)
2000	Iceland	0 (0-0)		0 (0-0)		0 (0-0)		0 (0-0)		0 (0-0)		0 (0-0)	
2001	Iceland	0 (0-0)		0 (0-0)		0 (0-0)		0 (0-0)		0 (0-0)		0 (0-0)	
2002	Iceland	0 (0-0)		0 (0-0)		0 (0-0)		0 (0-0)		0 (0-0)		0 (0-0)	
2003	Iceland	0 (0-0)		0 (0-0)		0 (0-0)		0 (0-0)		0 (0-0)		0 (0-0)	
2004	Iceland	0 (0-0)		0 (0-0)		0 (0-0)		0 (0-0)		0 (0-0)		0 (0-0)	
2005	Iceland	0 (0-0)		0 (0-0)		0 (0-0)		0 (0-0)		0 (0-0)		0 (0-0)	
2006	Iceland	0 (0-0)		0 (0-0)		0 (0-0)		0 (0-0)		0 (0-0)		0 (0-0)	
2007	Iceland	0 (0-0)		0 (0-0)		0 (0-0)		0 (0-0)		0 (0-0)		0 (0-0)	
2008	Iceland	0 (0-0)		0 (0-0)		0 (0-0)		0 (0-0)		0 (0-0)		0 (0-0)	
2009	Iceland	0 (0-0)		0 (0-0)		0 (0-0)		0 (0-0)		0 (0-0)		0 (0-0)	
2010	Iceland	0 (0-0)		0 (0-0)		0 (0-0)		0 (0-0)		0 (0-0)		0 (0-0)	
2011	Iceland	0 (0-0)		0 (0-0)		0 (0-0)		0 (0-0)		0 (0-0)		0 (0-0)	
2012	Iceland	0 (0-0)		0 (0-0)		0 (0-0)		0 (0-0)		0 (0-0)		0 (0-0)	
2013	Iceland	0 (0-0)		0 (0-0)		0 (0-0)		0 (0-0)		0 (0-0)		0 (0-0)	
2014	Iceland	0 (0-0)		0 (0-0)		0 (0-0)		0 (0-0)		0 (0-0)		0 (0-0)	

Hib = *Haemophilus influenzae* type b; npnm = non-pneumonia, non-meningitis; HIV = deaths only in HIV-infected children; Uncertainty range provided in parentheses.

Year	Country	Hib pneumonia deaths (UR)	Hib pneumonia deaths, HIV (UR)	Hib pneumonia mortality rate (UR)	Hib pneumonia mortality rate, HIV (UR)	Hib meningitis deaths (UR)	Hib meningitis deaths, HIV (UR)	Hib meningitis mortality rate (UR)	Hib meningitis mortality rate, HIV (UR)	Hib NPNM deaths (UR)	Hib NPNM deaths, HIV (UR)	Hib NPNM mortality rate (UR)	Hib NPNM mortality rate, HIV (UR)
2015	Iceland	0 (0-0)	0 (0-0)	0 (0-0)	0 (0-0)	0 (0-0)	0 (0-0)	0 (0-0)	0 (0-0)	0 (0-0)	0 (0-0)	0 (0-0)	0 (0-0)
2000	India	61391 (43178-80663)		49 (34-64)		21065 (9122-31840)		17 (7-25)		101 (44-153)		0 (0-0)	
2001	India	58888 (41417-77375)		47 (33-61)		20207 (8707-30591)		16 (7-24)		102 (44-155)		0 (0-0)	
2002	India	56304 (39600-73979)		44 (31-58)		19321 (8324-29251)		15 (7-23)		97 (42-148)		0 (0-0)	
2003	India	53787 (37830-70673)		42 (30-56)		18456 (7919-27985)		15 (6-22)		96 (41-146)		0 (0-0)	
2004	India	51137 (35966-67190)		40 (28-53)		17542 (7517-26619)		14 (6-21)		93 (39-141)		0 (0-0)	
2005	India	48419 (34054-63619)		38 (27-50)		16602 (7163-25129)		13 (6-20)		87 (37-133)		0 (0-0)	
2006	India	45962 (32326-60391)		36 (25-47)		15751 (6785-23841)		12 (5-19)		90 (39-137)		0 (0-0)	
2007	India	43207 (30389-56772)		34 (24-44)		14798 (6417-22348)		12 (5-18)		86 (37-130)		0 (0-0)	
2008	India	40558 (28525-53290)		32 (22-42)		13885 (6021-20980)		11 (5-16)		81 (35-122)		0 (0-0)	
2009	India	37904 (26659-49804)		30 (21-39)		12973 (5588-19652)		10 (4-15)		79 (34-120)		0 (0-0)	
2010	India	35403 (24900-46517)		28 (20-37)		12115 (5213-18364)		10 (4-15)		74 (32-112)		0 (0-0)	
2011	India	32966 (23186-43316)		26 (18-34)		11279 (4684-17251)		9 (4-14)		83 (34-126)		0 (0-0)	
2012	India	29787 (20950-39139)		24 (17-31)		10197 (4182-15605)		8 (3-13)		74 (31-114)		0 (0-0)	
2013	India	25936 (18241-34078)		21 (15-28)		8887 (3593-13673)		7 (3-11)		68 (28-105)		0 (0-0)	
2014	India	23250 (16352-30549)		19 (13-25)		7968 (3197-12263)		7 (3-10)		61 (24-94)		0 (0-0)	
2015	India	11626 (8177-15276)	29 (21-39)	10 (7-13)	0 (0-0)	3977 (1584-6136)	11 (4-16)	3 (1-5)	0 (0-0)	31 (13-48)	0 (0-0)	0 (0-0)	0 (0-0)
2000	Indonesia	6346 (4463-8338)		30 (21-40)		1850 (633-2955)		9 (3-14)		15 (5-24)		0 (0-0)	
2001	Indonesia	6011 (4228-7899)		28 (20-37)		1745 (602-2783)		8 (3-13)		14 (5-22)		0 (0-0)	
2002	Indonesia	5753 (4047-7560)		27 (19-36)		1651 (573-2629)		8 (3-12)		13 (5-21)		0 (0-0)	
2003	Indonesia	5484 (3857-7205)		26 (18-34)		1552 (539-2472)		7 (3-12)		12 (4-20)		0 (0-0)	
2004	Indonesia	6334 (4455-8322)		29 (21-38)		1768 (622-2808)		8 (3-13)		14 (5-22)		0 (0-0)	
2005	Indonesia	4943 (3476-6494)		23 (16-30)		1360 (485-2154)		6 (2-10)		11 (4-17)		0 (0-0)	
2006	Indonesia	4703 (3308-6179)		21 (15-28)		1275 (461-2015)		6 (2-9)		10 (4-16)		0 (0-0)	
2007	Indonesia	4442 (3124-5836)		20 (14-26)		1186 (435-1869)		5 (2-8)		9 (3-15)		0 (0-0)	
2008	Indonesia	4183 (2942-5496)		19 (13-25)		1099 (411-1724)		5 (2-8)		9 (3-14)		0 (0-0)	
2009	Indonesia	3984 (2802-5235)		18 (13-23)		1031 (394-1609)		5 (2-7)		8 (3-13)		0 (0-0)	
2010	Indonesia	3878 (2728-5096)		17 (12-22)		988 (386-1537)		4 (2-7)		8 (3-12)		0 (0-0)	
2011	Indonesia	3845 (2705-5052)		17 (12-22)		964 (387-1496)		4 (2-7)		8 (3-12)		0 (0-0)	
2012	Indonesia	3857 (2713-5068)		17 (12-22)		952 (392-1476)		4 (2-6)		8 (3-12)		0 (0-0)	
2013	Indonesia	3838 (2699-5042)		16 (11-21)		932 (169-1534)		4 (1-6)		7 (1-12)		0 (0-0)	
2014	Indonesia	1594 (1121-2094)		7 (5-9)		383 (71-633)		2 (0-3)		3 (1-5)		0 (0-0)	
2015	Indonesia	368 (259-483)	1 (1-2)	2 (1-2)	0 (0-0)	87 (16-145)	0 (0-1)	0 (0-1)	0 (0-0)	1 (0-1)	0 (0-0)	0 (0-0)	0 (0-0)
2000	Iran (Islamic Republic of)	965 (679-1268)		15 (11-20)		218 (116-317)		3 (2-5)		2 (1-3)		0 (0-0)	
2001	Iran (Islamic Republic of)	874 (615-1149)		14 (10-19)		194 (101-284)		3 (2-5)		2 (1-2)		0 (0-0)	
2002	Iran (Islamic Republic of)	795 (559-1045)		14 (10-18)		173 (89-258)		3 (2-4)		1 (1-2)		0 (0-0)	
2003	Iran (Islamic Republic of)	715 (503-940)		13 (9-17)		152 (40-236)		3 (1-4)		1 (0-2)		0 (0-0)	
2004	Iran (Islamic Republic of)	659 (463-866)		12 (8-16)		138 (34-219)		3 (1-4)		1 (0-2)		0 (0-0)	
2005	Iran (Islamic Republic of)	604 (425-794)		11 (8-15)		125 (30-202)		2 (1-4)		1 (0-2)		0 (0-0)	
2006	Iran (Islamic Republic of)	570 (401-749)		10 (7-13)		117 (26-192)		2 (0-3)		1 (0-2)		0 (0-0)	
2007	Iran (Islamic Republic of)	545 (384-717)		9 (7-12)		112 (24-185)		2 (0-3)		1 (0-1)		0 (0-0)	
2008	Iran (Islamic Republic of)	528 (371-693)		9 (6-12)		109 (22-180)		2 (0-3)		1 (0-1)		0 (0-0)	
2009	Iran (Islamic Republic of)	510 (359-671)		8 (6-11)		105 (20-175)		2 (0-3)		1 (0-1)		0 (0-0)	
2010	Iran (Islamic Republic of)	501 (352-658)		8 (6-10)		103 (19-173)		2 (0-3)		1 (0-1)		0 (0-0)	
2011	Iran (Islamic Republic of)	483 (340-635)		7 (5-10)		99 (18-166)		2 (0-3)		1 (0-1)		0 (0-0)	
2012	Iran (Islamic Republic of)	469 (330-616)		7 (5-9)		96 (18-161)		1 (0-2)		1 (0-1)		0 (0-0)	
2013	Iran (Islamic Republic of)	454 (320-597)		7 (5-9)		93 (18-156)		1 (0-2)		1 (0-1)		0 (0-0)	
2014	Iran (Islamic Republic of)	65 (46-85)		1 (1-1)		13 (3-22)		0 (0-0)		0 (0-0)		0 (0-0)	
2015	Iran (Islamic Republic of)	28 (20-36)	0 (0-0)	0 (0-1)	0 (0-0)	6 (1-9)	0 (0-0)	0 (0-0)	0 (0-0)	0 (0-0)	0 (0-0)	0 (0-0)	0 (0-0)
2000	Iraq	807 (567-1060)		21 (15-28)		186 (77-285)		5 (2-8)		1 (1-2)		0 (0-0)	
2001	Iraq	801 (563-1052)		21 (15-27)		184 (77-282)		5 (2-7)		1 (1-2)		0 (0-0)	
2002	Iraq	791 (556-1039)		20 (14-26)		184 (78-280)		5 (2-7)		1 (1-2)		0 (0-0)	
2003	Iraq	784 (551-1030)		20 (14-26)		184 (79-279)		5 (2-7)		1 (1-2)		0 (0-0)	
2004	Iraq	780 (549-1025)		19 (13-25)		181 (79-274)		4 (2-7)		1 (1-2)		0 (0-0)	
2005	Iraq	785 (552-1032)		19 (13-25)		180 (79-271)		4 (2-7)		1 (1-2)		0 (0-0)	
2006	Iraq	788 (554-1035)		19 (13-24)		178 (79-267)		4 (2-6)		1 (1-2)		0 (0-0)	
2007	Iraq	803 (565-1055)		18 (13-24)		180 (79-271)		4 (2-6)		1 (1-2)		0 (0-0)	
2008	Iraq	822 (578-1080)		18 (13-24)		183 (79-278)		4 (2-6)		1 (1-2)		0 (0-0)	
2009	Iraq	840 (591-1103)		18 (13-23)		186 (78-284)		4 (2-6)		1 (1-2)		0 (0-0)	
2010	Iraq	851 (598-1118)		17 (12-23)		187 (77-287)		4 (2-6)		1 (1-2)		0 (0-0)	
2011	Iraq	842 (592-1107)		17 (12-22)		183 (74-282)		4 (1-6)		1 (1-2)		0 (0-0)	
2012	Iraq	594 (418-781)		11 (8-15)		127 (50-197)		2 (1-4)		1 (0-2)		0 (0-0)	
2013	Iraq	101 (71-132)		2 (1-2)		21 (8-33)		0 (0-1)		0 (0-0)		0 (0-0)	
2014	Iraq	104 (73-137)		2 (1-2)		22 (8-34)		0 (0-1)		0 (0-0)		0 (0-0)	
2015	Iraq	113 (79-148)	0 (0-0)	2 (1-3)	0 (0-0)	24 (9-37)	0 (0-0)	0 (0-1)	0 (0-0)	0 (0-0)	0 (0-0)	0 (0-0)	0 (0-0)
2000	Ireland	0 (0-0)		0 (0-0)		1 (0-1)		0 (0-0)		0 (0-0)		0 (0-0)	
2001	Ireland	0 (0-0)		0 (0-0)		0 (0-1)		0 (0-0)		0 (0-0)		0 (0-0)	
2002	Ireland	0 (0-0)		0 (0-0)		0 (0-0)		0 (0-0)		0 (0-0)		0 (0-0)	
2003	Ireland	0 (0-0)		0 (0-0)		0 (0-0)		0 (0-0)		0 (0-0)		0 (0-0)	
2004	Ireland	0 (0-0)		0 (0-0)		0 (0-0)		0 (0-0)		0 (0-0)		0 (0-0)	
2005	Ireland	0 (0-0)		0 (0-0)		0 (0-0)		0 (0-0)		0 (0-0)		0 (0-0)	
2006	Ireland	0 (0-0)		0 (0-0)		0 (0-0)		0 (0-0)		0 (0-0)		0 (0-0)	
2007	Ireland	0 (0-0)		0 (0-0)		0 (0-0)		0 (0-0)		0 (0-0)		0 (0-0)	
2008	Ireland	0 (0-0)		0 (0-0)		0 (0-0)		0 (0-0)		0 (0-0)		0 (0-0)	
2009	Ireland	0 (0-0)		0 (0-0)		0 (0-0)		0 (0-0)		0 (0-0)		0 (0-0)	
2010	Ireland	0 (0-0)		0 (0-0)		0 (0-0)		0 (0-0)		0 (0-0)		0 (0-0)	
2011	Ireland	0 (0-0)		0 (0-0)		0 (0-0)		0 (0-0)		0 (0-0)		0 (0-0)	
2012	Ireland	0 (0-0)		0 (0-0)		0 (0-0)		0 (0-0)		0 (0-0)		0 (0-0)	
2013	Ireland	0 (0-0)		0 (0-0)		0 (0-0)		0 (0-0)		0 (0-0)		0 (0-0)	
2014	Ireland	0 (0-0)		0 (0-0)		0 (0-0)		0 (0-0)		0 (0-0)		0 (0-0)	
2015	Ireland	0 (0-0)	0 (0-0)	0 (0-0)	0 (0-0)	0 (0-0)	0 (0-0)	0 (0-0)	0 (0-0)	0 (0-0)	0 (0-0)	0 (0-0)	0 (0-0)
2000	Israel	0 (0-0)		0 (0-0)		0 (0-0)		0 (0-0)		0 (0-0)		0 (0-0)	
2001	Israel	0 (0-0)		0 (0-0)		0 (0-0)		0 (0-0)		0 (0-0)		0 (0-0)	
2002	Israel	0 (0-0)		0 (0-0)		0 (0-0)		0 (0-0)		0 (0-0)		0 (0-0)	
2003	Israel	0 (0-0)		0 (0-0)		0 (0-0)		0 (0-0)		0 (0-0)		0 (0-0)	
2004	Israel	0 (0-0)		0 (0-0)		0 (0-0)		0 (0-0)		0 (0-0)		0 (0-0)	
2005	Israel	0 (0-0)		0 (0-0)		0 (0-0)		0 (0-0)		0 (0-0)		0 (0-0)	
2006	Israel	0 (0-0)		0 (0-0)		0 (0-0)		0 (0-0)		0 (0-0)		0 (0-0)	

Hib = *Haemophilus influenzae* type b; npnm = non-pneumonia, non-meningitis; HIV = deaths only in HIV-infected children; Uncertainty range provided in parentheses.

Year	Country	Hib pneumonia deaths (UR)	Hib pneumonia deaths, HIV (UR)	Hib pneumonia mortality rate (UR)	Hib pneumonia mortality rate, HIV (UR)	Hib meningitis deaths (UR)	Hib meningitis deaths, HIV (UR)	Hib meningitis mortality rate (UR)	Hib meningitis mortality rate, HIV (UR)	Hib NPNM deaths (UR)	Hib NPNM deaths, HIV (UR)	Hib NPNM mortality rate (UR)	Hib NPNM mortality rate, HIV (UR)
2007	Israel	0 (0-0)		0 (0-0)		0 (0-0)		0 (0-0)		0 (0-0)		0 (0-0)	
2008	Israel	0 (0-0)		0 (0-0)		0 (0-0)		0 (0-0)		0 (0-0)		0 (0-0)	
2009	Israel	0 (0-0)		0 (0-0)		0 (0-0)		0 (0-0)		0 (0-0)		0 (0-0)	
2010	Israel	0 (0-0)		0 (0-0)		0 (0-0)		0 (0-0)		0 (0-0)		0 (0-0)	
2011	Israel	0 (0-0)		0 (0-0)		0 (0-0)		0 (0-0)		0 (0-0)		0 (0-0)	
2012	Israel	0 (0-0)		0 (0-0)		0 (0-0)		0 (0-0)		0 (0-0)		0 (0-0)	
2013	Israel	0 (0-0)		0 (0-0)		0 (0-0)		0 (0-0)		0 (0-0)		0 (0-0)	
2014	Israel	0 (0-0)		0 (0-0)		0 (0-0)		0 (0-0)		0 (0-0)		0 (0-0)	
2015	Israel	0 (0-0)	0 (0-0)	0 (0-0)	0 (0-0)	0 (0-0)	0 (0-0)	0 (0-0)	0 (0-0)	0 (0-0)	0 (0-0)	0 (0-0)	0 (0-0)
2000	Italy	4 (3-5)		0 (0-0)		2 (1-3)		0 (0-0)		0 (0-0)		0 (0-0)	
2001	Italy	1 (1-1)		0 (0-0)		1 (0-1)		0 (0-0)		0 (0-0)		0 (0-0)	
2002	Italy	1 (1-1)		0 (0-0)		0 (0-1)		0 (0-0)		0 (0-0)		0 (0-0)	
2003	Italy	0 (0-1)		0 (0-0)		1 (0-1)		0 (0-0)		0 (0-0)		0 (0-0)	
2004	Italy	0 (0-1)		0 (0-0)		0 (0-1)		0 (0-0)		0 (0-0)		0 (0-0)	
2005	Italy	0 (0-0)		0 (0-0)		0 (0-0)		0 (0-0)		0 (0-0)		0 (0-0)	
2006	Italy	0 (0-0)		0 (0-0)		0 (0-0)		0 (0-0)		0 (0-0)		0 (0-0)	
2007	Italy	0 (0-0)		0 (0-0)		0 (0-0)		0 (0-0)		0 (0-0)		0 (0-0)	
2008	Italy	0 (0-0)		0 (0-0)		0 (0-0)		0 (0-0)		0 (0-0)		0 (0-0)	
2009	Italy	0 (0-0)		0 (0-0)		0 (0-0)		0 (0-0)		0 (0-0)		0 (0-0)	
2010	Italy	0 (0-0)		0 (0-0)		0 (0-0)		0 (0-0)		0 (0-0)		0 (0-0)	
2011	Italy	0 (0-0)		0 (0-0)		0 (0-0)		0 (0-0)		0 (0-0)		0 (0-0)	
2012	Italy	0 (0-0)		0 (0-0)		0 (0-0)		0 (0-0)		0 (0-0)		0 (0-0)	
2013	Italy	0 (0-0)		0 (0-0)		0 (0-0)		0 (0-0)		0 (0-0)		0 (0-0)	
2014	Italy	0 (0-0)		0 (0-0)		0 (0-0)		0 (0-0)		0 (0-0)		0 (0-0)	
2015	Italy	0 (0-0)	0 (0-0)	0 (0-0)	0 (0-0)	0 (0-0)	0 (0-0)	0 (0-0)	0 (0-0)	0 (0-0)	0 (0-0)	0 (0-0)	0 (0-0)
2000	Jamaica	5 (4-7)		2 (1-2)		2 (0-3)		1 (0-1)		0 (0-0)		0 (0-0)	
2001	Jamaica	5 (3-6)		2 (1-2)		1 (0-2)		0 (0-1)		0 (0-0)		0 (0-0)	
2002	Jamaica	5 (3-6)		2 (1-2)		1 (0-2)		0 (0-1)		0 (0-0)		0 (0-0)	
2003	Jamaica	3 (2-4)		1 (1-2)		1 (0-2)		0 (0-1)		0 (0-0)		0 (0-0)	
2004	Jamaica	0 (0-0)		0 (0-0)		0 (0-0)		0 (0-0)		0 (0-0)		0 (0-0)	
2005	Jamaica	0 (0-0)		0 (0-0)		0 (0-0)		0 (0-0)		0 (0-0)		0 (0-0)	
2006	Jamaica	0 (0-1)		0 (0-0)		0 (0-0)		0 (0-0)		0 (0-0)		0 (0-0)	
2007	Jamaica	1 (0-1)		0 (0-0)		0 (0-0)		0 (0-0)		0 (0-0)		0 (0-0)	
2008	Jamaica	0 (0-0)		0 (0-0)		0 (0-0)		0 (0-0)		0 (0-0)		0 (0-0)	
2009	Jamaica	0 (0-0)		0 (0-0)		0 (0-0)		0 (0-0)		0 (0-0)		0 (0-0)	
2010	Jamaica	0 (0-0)		0 (0-0)		0 (0-0)		0 (0-0)		0 (0-0)		0 (0-0)	
2011	Jamaica	0 (0-0)		0 (0-0)		0 (0-0)		0 (0-0)		0 (0-0)		0 (0-0)	
2012	Jamaica	0 (0-0)		0 (0-0)		0 (0-0)		0 (0-0)		0 (0-0)		0 (0-0)	
2013	Jamaica	0 (0-0)		0 (0-0)		0 (0-0)		0 (0-0)		0 (0-0)		0 (0-0)	
2014	Jamaica	0 (0-0)		0 (0-0)		0 (0-0)		0 (0-0)		0 (0-0)		0 (0-0)	
2015	Jamaica	0 (0-0)	0 (0-0)	0 (0-0)	0 (0-0)	0 (0-0)	0 (0-0)	0 (0-0)	0 (0-0)	0 (0-0)	0 (0-0)	0 (0-0)	0 (0-0)
2000	Japan	55 (39-72)		1 (1-1)		13 (6-21)		0 (0-0)		0 (0-0)		0 (0-0)	
2001	Japan	43 (30-56)		1 (1-1)		11 (6-19)		0 (0-0)		0 (0-0)		0 (0-0)	
2002	Japan	47 (33-61)		1 (1-1)		9 (4-14)		0 (0-0)		0 (0-0)		0 (0-0)	
2003	Japan	47 (33-62)		1 (1-1)		10 (5-17)		0 (0-0)		0 (0-0)		0 (0-0)	
2004	Japan	39 (28-52)		1 (0-1)		8 (4-13)		0 (0-0)		0 (0-0)		0 (0-0)	
2005	Japan	43 (30-56)		1 (1-1)		9 (4-15)		0 (0-0)		0 (0-0)		0 (0-0)	
2006	Japan	38 (26-49)		1 (0-1)		7 (3-11)		0 (0-0)		0 (0-0)		0 (0-0)	
2007	Japan	39 (28-52)		1 (1-1)		9 (4-15)		0 (0-0)		0 (0-0)		0 (0-0)	
2008	Japan	41 (29-54)		1 (1-1)		5 (3-8)		0 (0-0)		0 (0-0)		0 (0-0)	
2009	Japan	31 (21-40)		1 (0-1)		7 (3-11)		0 (0-0)		0 (0-0)		0 (0-0)	
2010	Japan	36 (25-47)		1 (0-1)		9 (5-16)		0 (0-0)		0 (0-0)		0 (0-0)	
2011	Japan	33 (23-43)		1 (0-1)		4 (2-7)		0 (0-0)		0 (0-0)		0 (0-0)	
2012	Japan	32 (23-43)		1 (0-1)		3 (2-5)		0 (0-0)		0 (0-0)		0 (0-0)	
2013	Japan	29 (21-38)		1 (0-1)		3 (2-6)		0 (0-0)		0 (0-0)		0 (0-0)	
2014	Japan	29 (20-38)		1 (0-1)		3 (2-5)		0 (0-0)		0 (0-0)		0 (0-0)	
2015	Japan	6 (4-8)	0 (0-0)	0 (0-0)	0 (0-0)	1 (0-1)	0 (0-0)	0 (0-0)	0 (0-0)	0 (0-0)	0 (0-0)	0 (0-0)	0 (0-0)
2000	Jordan	63 (44-83)		9 (6-12)		10 (2-16)		1 (0-2)		0 (0-0)		0 (0-0)	
2001	Jordan	26 (19-35)		4 (3-5)		6 (1-10)		1 (0-1)		0 (0-0)		0 (0-0)	
2002	Jordan	5 (3-6)		1 (0-1)		1 (0-2)		0 (0-0)		0 (0-0)		0 (0-0)	
2003	Jordan	4 (3-6)		1 (0-1)		1 (0-2)		0 (0-0)		0 (0-0)		0 (0-0)	
2004	Jordan	4 (3-6)		1 (0-1)		1 (0-2)		0 (0-0)		0 (0-0)		0 (0-0)	
2005	Jordan	4 (3-5)		1 (0-1)		1 (0-2)		0 (0-0)		0 (0-0)		0 (0-0)	
2006	Jordan	1 (0-1)		0 (0-0)		0 (0-0)		0 (0-0)		0 (0-0)		0 (0-0)	
2007	Jordan	1 (0-1)		0 (0-0)		0 (0-0)		0 (0-0)		0 (0-0)		0 (0-0)	
2008	Jordan	1 (0-1)		0 (0-0)		0 (0-0)		0 (0-0)		0 (0-0)		0 (0-0)	
2009	Jordan	1 (0-1)		0 (0-0)		0 (0-0)		0 (0-0)		0 (0-0)		0 (0-0)	
2010	Jordan	1 (0-1)		0 (0-0)		0 (0-0)		0 (0-0)		0 (0-0)		0 (0-0)	
2011	Jordan	1 (0-1)		0 (0-0)		0 (0-0)		0 (0-0)		0 (0-0)		0 (0-0)	
2012	Jordan	1 (0-1)		0 (0-0)		0 (0-0)		0 (0-0)		0 (0-0)		0 (0-0)	
2013	Jordan	1 (0-1)		0 (0-0)		0 (0-0)		0 (0-0)		0 (0-0)		0 (0-0)	
2014	Jordan	1 (0-1)		0 (0-0)		0 (0-0)		0 (0-0)		0 (0-0)		0 (0-0)	
2015	Jordan	1 (0-1)	0 (0-0)	0 (0-0)	0 (0-0)	0 (0-0)	0 (0-0)	0 (0-0)	0 (0-0)	0 (0-0)	0 (0-0)	0 (0-0)	0 (0-0)
2000	Kazakhstan	265 (187-349)		24 (17-32)		65 (24-102)		6 (2-9)		1 (0-1)		0 (0-0)	
2001	Kazakhstan	254 (178-333)		24 (17-31)		60 (22-95)		6 (2-9)		0 (0-1)		0 (0-0)	
2002	Kazakhstan	251 (177-330)		23 (16-31)		58 (22-91)		5 (2-8)		0 (0-1)		0 (0-0)	
2003	Kazakhstan	250 (175-328)		22 (16-29)		57 (21-89)		5 (2-8)		0 (0-1)		0 (0-0)	
2004	Kazakhstan	247 (174-325)		21 (15-28)		55 (21-86)		5 (2-7)		0 (0-1)		0 (0-0)	
2005	Kazakhstan	249 (175-327)		20 (14-27)		54 (21-85)		4 (2-7)		0 (0-1)		0 (0-0)	
2006	Kazakhstan	248 (175-326)		19 (13-25)		53 (21-83)		4 (2-6)		0 (0-1)		0 (0-0)	
2007	Kazakhstan	242 (171-319)		18 (12-23)		50 (9-84)		4 (1-6)		0 (0-1)		0 (0-0)	
2008	Kazakhstan	129 (91-170)		9 (6-12)		26 (5-44)		2 (0-3)		0 (0-0)		0 (0-0)	
2009	Kazakhstan	16 (11-21)		1 (1-1)		3 (1-5)		0 (0-0)		0 (0-0)		0 (0-0)	
2010	Kazakhstan	15 (11-20)		1 (1-1)		3 (1-5)		0 (0-0)		0 (0-0)		0 (0-0)	
2011	Kazakhstan	15 (10-19)		1 (1-1)		3 (1-5)		0 (0-0)		0 (0-0)		0 (0-0)	
2012	Kazakhstan	13 (9-17)		1 (1-1)		3 (1-4)		0 (0-0)		0 (0-0)		0 (0-0)	
2013	Kazakhstan	2 (1-2)		0 (0-0)		0 (0-1)		0 (0-0)		0 (0-0)		0 (0-0)	
2014	Kazakhstan	2 (1-2)		0 (0-0)		0 (0-1)		0 (0-0)		0 (0-0)		0 (0-0)	

Hib = *Haemophilus influenzae* type b; npnm = non-pneumonia, non-meningitis; HIV = deaths only in HIV-infected children; Uncertainty range provided in parentheses.

Year	Country	Hib pneumonia deaths (UR)	Hib pneumonia deaths, HIV (UR)	Hib pneumonia mortality rate (UR)	Hib pneumonia mortality rate, HIV (UR)	Hib meningitis deaths (UR)	Hib meningitis deaths, HIV (UR)	Hib meningitis mortality rate (UR)	Hib meningitis mortality rate, HIV (UR)	Hib NPNM deaths (UR)	Hib NPNM deaths, HIV (UR)	Hib NPNM mortality rate (UR)	Hib NPNM mortality rate, HIV (UR)
2015	Kazakhstan	2 (1-2)	0 (0-0)	0 (0-0)	0 (0-0)	0 (0-0)	0 (0-0)	0 (0-0)	0 (0-0)	0 (0-0)	0 (0-0)	0 (0-0)	0 (0-0)
2000	Kenya	2916 (2051-3831)		59 (41-77)		1068 (360-1717)		21 (7-34)		4 (1-7)		0 (0-0)	
2001	Kenya	2882 (2027-3787)		56 (39-73)		1054 (357-1693)		20 (7-33)		4 (1-7)		0 (0-0)	
2002	Kenya	464 (327-610)		9 (6-11)		170 (58-272)		3 (1-5)		1 (0-1)		0 (0-0)	
2003	Kenya	306 (215-403)		6 (4-7)		111 (38-178)		2 (1-3)		0 (0-1)		0 (0-0)	
2004	Kenya	298 (210-392)		5 (4-7)		108 (37-172)		2 (1-3)		0 (0-1)		0 (0-0)	
2005	Kenya	267 (188-351)		5 (3-6)		96 (34-153)		2 (1-3)		0 (0-1)		0 (0-0)	
2006	Kenya	243 (171-319)		4 (3-5)		87 (31-138)		1 (1-2)		0 (0-1)		0 (0-0)	
2007	Kenya	24 (17-32)		0 (0-1)		9 (3-14)		0 (0-0)		0 (0-0)		0 (0-0)	
2008	Kenya	23 (16-31)		0 (0-0)		8 (3-14)		0 (0-0)		0 (0-0)		0 (0-0)	
2009	Kenya	22 (16-29)		0 (0-0)		8 (3-13)		0 (0-0)		0 (0-0)		0 (0-0)	
2010	Kenya	21 (15-28)		0 (0-0)		8 (3-12)		0 (0-0)		0 (0-0)		0 (0-0)	
2011	Kenya	20 (14-27)		0 (0-0)		7 (2-11)		0 (0-0)		0 (0-0)		0 (0-0)	
2012	Kenya	20 (14-26)		0 (0-0)		7 (2-11)		0 (0-0)		0 (0-0)		0 (0-0)	
2013	Kenya	19 (13-25)		0 (0-0)		6 (2-10)		0 (0-0)		0 (0-0)		0 (0-0)	
2014	Kenya	18 (13-24)		0 (0-0)		6 (2-10)		0 (0-0)		0 (0-0)		0 (0-0)	
2015	Kenya	18 (12-23)	0 (0-1)	0 (0-0)	0 (0-0)	6 (2-10)	0 (0-0)	0 (0-0)	0 (0-0)	0 (0-0)	0 (0-0)	0 (0-0)	0 (0-0)
2000	Kiribati	5 (4-7)		46 (32-60)		3 (2-3)		23 (13-30)		0 (0-0)		0 (0-0)	
2001	Kiribati	5 (4-7)		42 (30-56)		3 (1-3)		21 (12-27)		0 (0-0)		0 (0-0)	
2002	Kiribati	5 (3-6)		40 (28-52)		2 (1-3)		20 (11-26)		0 (0-0)		0 (0-0)	
2003	Kiribati	4 (3-5)		36 (26-48)		2 (1-3)		18 (10-23)		0 (0-0)		0 (0-0)	
2004	Kiribati	4 (3-5)		35 (25-46)		2 (1-2)		18 (10-23)		0 (0-0)		0 (0-0)	
2005	Kiribati	4 (3-5)		37 (26-48)		2 (1-3)		18 (10-24)		0 (0-0)		0 (0-0)	
2006	Kiribati	4 (3-6)		41 (29-54)		2 (1-3)		21 (12-26)		0 (0-0)		0 (0-0)	
2007	Kiribati	5 (3-6)		46 (32-60)		2 (1-3)		23 (13-30)		0 (0-0)		0 (0-0)	
2008	Kiribati	1 (1-1)		8 (6-11)		0 (0-1)		4 (2-5)		0 (0-0)		0 (0-0)	
2009	Kiribati	1 (0-1)		4 (3-6)		0 (0-0)		2 (1-3)		0 (0-0)		0 (0-0)	
2010	Kiribati	1 (0-1)		4 (3-5)		0 (0-0)		2 (1-3)		0 (0-0)		0 (0-0)	
2011	Kiribati	0 (0-1)		3 (2-4)		0 (0-0)		2 (1-2)		0 (0-0)		0 (0-0)	
2012	Kiribati	0 (0-1)		3 (2-4)		0 (0-0)		2 (1-2)		0 (0-0)		0 (0-0)	
2013	Kiribati	0 (0-0)		0 (0-1)		0 (0-0)		0 (0-0)		0 (0-0)		0 (0-0)	
2014	Kiribati	0 (0-0)		0 (0-0)		0 (0-0)		0 (0-0)		0 (0-0)		0 (0-0)	
2015	Kiribati	0 (0-0)	0 (0-0)	0 (0-0)	0 (0-0)	0 (0-0)	0 (0-0)	0 (0-0)	0 (0-0)	0 (0-0)	0 (0-0)	0 (0-0)	0 (0-0)
2000	Kuwait	0 (0-1)		0 (0-0)		0 (0-0)		0 (0-0)		0 (0-0)		0 (0-0)	
2001	Kuwait	0 (0-1)		0 (0-0)		0 (0-0)		0 (0-0)		0 (0-0)		0 (0-0)	
2002	Kuwait	0 (0-1)		0 (0-0)		0 (0-0)		0 (0-0)		0 (0-0)		0 (0-0)	
2003	Kuwait	0 (0-0)		0 (0-0)		0 (0-0)		0 (0-0)		0 (0-0)		0 (0-0)	
2004	Kuwait	0 (0-0)		0 (0-0)		0 (0-0)		0 (0-0)		0 (0-0)		0 (0-0)	
2005	Kuwait	0 (0-0)		0 (0-0)		0 (0-0)		0 (0-0)		0 (0-0)		0 (0-0)	
2006	Kuwait	0 (0-0)		0 (0-0)		0 (0-0)		0 (0-0)		0 (0-0)		0 (0-0)	
2007	Kuwait	0 (0-0)		0 (0-0)		0 (0-0)		0 (0-0)		0 (0-0)		0 (0-0)	
2008	Kuwait	0 (0-0)		0 (0-0)		0 (0-0)		0 (0-0)		0 (0-0)		0 (0-0)	
2009	Kuwait	0 (0-0)		0 (0-0)		0 (0-0)		0 (0-0)		0 (0-0)		0 (0-0)	
2010	Kuwait	0 (0-0)		0 (0-0)		0 (0-0)		0 (0-0)		0 (0-0)		0 (0-0)	
2011	Kuwait	0 (0-0)		0 (0-0)		0 (0-0)		0 (0-0)		0 (0-0)		0 (0-0)	
2012	Kuwait	0 (0-0)		0 (0-0)		0 (0-0)		0 (0-0)		0 (0-0)		0 (0-0)	
2013	Kuwait	0 (0-0)		0 (0-0)		0 (0-0)		0 (0-0)		0 (0-0)		0 (0-0)	
2014	Kuwait	0 (0-0)		0 (0-0)		0 (0-0)		0 (0-0)		0 (0-0)		0 (0-0)	
2015	Kuwait	0 (0-0)	0 (0-0)	0 (0-0)	0 (0-0)	0 (0-0)	0 (0-0)	0 (0-0)	0 (0-0)	0 (0-0)	0 (0-0)	0 (0-0)	0 (0-0)
2000	Kyrgyzstan	156 (110-205)		29 (20-38)		62 (22-98)		12 (4-18)		0 (0-1)		0 (0-0)	
2001	Kyrgyzstan	144 (101-189)		27 (19-36)		57 (20-90)		11 (4-17)		0 (0-1)		0 (0-0)	
2002	Kyrgyzstan	132 (93-174)		26 (18-34)		52 (18-82)		10 (4-16)		0 (0-1)		0 (0-0)	
2003	Kyrgyzstan	121 (85-159)		25 (17-32)		47 (17-74)		10 (3-15)		0 (0-1)		0 (0-0)	
2004	Kyrgyzstan	113 (79-148)		23 (16-31)		43 (15-69)		9 (3-14)		0 (0-1)		0 (0-0)	
2005	Kyrgyzstan	108 (76-142)		22 (16-29)		41 (14-65)		8 (3-13)		0 (0-1)		0 (0-0)	
2006	Kyrgyzstan	106 (74-139)		21 (15-28)		40 (14-63)		8 (3-13)		0 (0-1)		0 (0-0)	
2007	Kyrgyzstan	105 (74-138)		21 (14-27)		39 (14-62)		8 (3-12)		0 (0-0)		0 (0-0)	
2008	Kyrgyzstan	104 (73-136)		19 (14-25)		38 (13-61)		7 (2-11)		0 (0-0)		0 (0-0)	
2009	Kyrgyzstan	105 (74-138)		19 (13-24)		38 (13-61)		7 (2-11)		0 (0-0)		0 (0-0)	
2010	Kyrgyzstan	16 (11-21)		3 (2-4)		6 (2-9)		1 (0-2)		0 (0-0)		0 (0-0)	
2011	Kyrgyzstan	7 (5-10)		1 (1-2)		3 (0-5)		0 (0-1)		0 (0-0)		0 (0-0)	
2012	Kyrgyzstan	7 (5-10)		1 (1-1)		3 (0-4)		0 (0-1)		0 (0-0)		0 (0-0)	
2013	Kyrgyzstan	7 (5-9)		1 (1-1)		2 (0-4)		0 (0-1)		0 (0-0)		0 (0-0)	
2014	Kyrgyzstan	7 (5-9)		1 (1-1)		2 (0-4)		0 (0-1)		0 (0-0)		0 (0-0)	
2015	Kyrgyzstan	1 (1-1)	0 (0-0)	0 (0-0)	0 (0-0)	0 (0-1)	0 (0-0)	0 (0-1)	0 (0-0)	0 (0-0)	0 (0-0)	0 (0-0)	0 (0-0)
2000	Lao People's Democratic Republic	563 (396-740)		71 (50-94)		210 (65-336)		27 (8-43)		1 (0-1)		0 (0-0)	
2001	Lao People's Democratic Republic	543 (382-714)		71 (50-93)		200 (61-320)		26 (8-42)		1 (0-1)		0 (0-0)	
2002	Lao People's Democratic Republic	525 (369-689)		70 (49-91)		190 (58-306)		25 (8-41)		1 (0-1)		0 (0-0)	
2003	Lao People's Democratic Republic	511 (359-671)		68 (48-90)		183 (56-294)		24 (7-39)		1 (0-1)		0 (0-0)	
2004	Lao People's Democratic Republic	499 (351-655)		67 (47-88)		175 (53-282)		24 (7-38)		1 (0-1)		0 (0-0)	
2005	Lao People's Democratic Republic	487 (343-641)		65 (46-86)		169 (51-272)		23 (7-37)		1 (0-1)		0 (0-0)	
2006	Lao People's Democratic Republic	480 (337-630)		64 (45-84)		163 (49-264)		22 (7-35)		1 (0-1)		0 (0-0)	
2007	Lao People's Democratic Republic	470 (331-618)		62 (44-81)		158 (49-253)		21 (6-33)		1 (0-1)		0 (0-0)	
2008	Lao People's Democratic Republic	463 (325-608)		60 (42-79)		153 (49-244)		20 (6-32)		1 (0-1)		0 (0-0)	
2009	Lao People's Democratic Republic	451 (317-592)		57 (40-75)		147 (49-232)		19 (6-30)		1 (0-1)		0 (0-0)	
2010	Lao People's Democratic Republic	105 (74-138)		13 (9-17)		34 (12-53)		4 (1-7)		0 (0-0)		0 (0-0)	
2011	Lao People's Democratic Republic	43 (30-57)		5 (4-7)		14 (5-21)		2 (1-3)		0 (0-0)		0 (0-0)	
2012	Lao People's Democratic Republic	40 (28-53)		5 (3-6)		13 (4-20)		2 (0-2)		0 (0-0)		0 (0-0)	
2013	Lao People's Democratic Republic	33 (23-43)		4 (3-5)		10 (3-16)		1 (0-2)		0 (0-0)		0 (0-0)	
2014	Lao People's Democratic Republic	31 (22-40)		4 (3-5)		9 (3-15)		1 (0-2)		0 (0-0)		0 (0-0)	
2015	Lao People's Democratic Republic	3 (2-5)	0 (0-0)	0 (0-1)	0 (0-0)	1 (0-2)	0 (0-0)	0 (0-0)	0 (0-0)	0 (0-0)	0 (0-0)	0 (0-0)	0 (0-0)
2000	Latvia	0 (0-0)		0 (0-0)		0 (0-0)		0 (0-0)		0 (0-0)		0 (0-0)	
2001	Latvia	0 (0-0)		0 (0-0)		0 (0-0)		0 (0-0)		0 (0-0)		0 (0-0)	
2002	Latvia	0 (0-0)		0 (0-0)		0 (0-0)		0 (0-0)		0 (0-0)		0 (0-0)	
2003	Latvia	0 (0-0)		0 (0-0)		0 (0-0)		0 (0-0)		0 (0-0)		0 (0-0)	
2004	Latvia	0 (0-0)		0 (0-0)		0 (0-0)		0 (0-0)		0 (0-0)		0 (0-0)	
2005	Latvia	0 (0-0)		0 (0-0)		0 (0-0)		0 (0-0)		0 (0-0)		0 (0-0)	
2006	Latvia	0 (0-0)		0 (0-0)		0 (0-0)		0 (0-0)		0 (0-0)		0 (0-0)	

Hib = *Haemophilus influenzae* type b; npnm = non-pneumonia, non-meningitis; HIV = deaths only in HIV-infected children; Uncertainty range provided in parentheses.

Year	Country	Hib pneumonia deaths (UR)	Hib pneumonia deaths, HIV (UR)	Hib pneumonia mortality rate (UR)	Hib pneumonia mortality rate, HIV (UR)	Hib meningitis deaths (UR)	Hib meningitis deaths, HIV (UR)	Hib meningitis mortality rate (UR)	Hib meningitis mortality rate, HIV (UR)	Hib NPNM deaths (UR)	Hib NPNM deaths, HIV (UR)	Hib NPNM mortality rate (UR)	Hib NPNM mortality rate, HIV (UR)
2007	Latvia	0 (0-0)		0 (0-0)		0 (0-0)		0 (0-0)		0 (0-0)		0 (0-0)	
2008	Latvia	0 (0-0)		0 (0-0)		0 (0-0)		0 (0-0)		0 (0-0)		0 (0-0)	
2009	Latvia	0 (0-0)		0 (0-0)		0 (0-0)		0 (0-0)		0 (0-0)		0 (0-0)	
2010	Latvia	0 (0-0)		0 (0-0)		0 (0-0)		0 (0-0)		0 (0-0)		0 (0-0)	
2011	Latvia	0 (0-0)		0 (0-0)		0 (0-0)		0 (0-0)		0 (0-0)		0 (0-0)	
2012	Latvia	0 (0-0)		0 (0-0)		0 (0-0)		0 (0-0)		0 (0-0)		0 (0-0)	
2013	Latvia	0 (0-0)		0 (0-0)		0 (0-0)		0 (0-0)		0 (0-0)		0 (0-0)	
2014	Latvia	0 (0-0)		0 (0-0)		0 (0-0)		0 (0-0)		0 (0-0)		0 (0-0)	
2015	Latvia	0 (0-0)	0 (0-0)	0 (0-0)	0 (0-0)	0 (0-0)	0 (0-0)	0 (0-0)	0 (0-0)	0 (0-0)	0 (0-0)	0 (0-0)	0 (0-0)
2000	Lebanon	16 (12-22)		5 (4-7)		3 (0-5)		1 (0-2)		0 (0-0)		0 (0-0)	
2001	Lebanon	15 (10-19)		5 (3-6)		2 (0-4)		1 (0-1)		0 (0-0)		0 (0-0)	
2002	Lebanon	14 (10-18)		4 (3-6)		2 (0-4)		1 (0-1)		0 (0-0)		0 (0-0)	
2003	Lebanon	3 (2-4)		1 (1-1)		1 (0-2)		0 (0-0)		0 (0-0)		0 (0-0)	
2004	Lebanon	1 (1-2)		0 (0-1)		0 (0-1)		0 (0-0)		0 (0-0)		0 (0-0)	
2005	Lebanon	1 (1-2)		0 (0-0)		0 (0-1)		0 (0-0)		0 (0-0)		0 (0-0)	
2006	Lebanon	1 (1-1)		0 (0-0)		0 (0-0)		0 (0-0)		0 (0-0)		0 (0-0)	
2007	Lebanon	1 (1-1)		0 (0-0)		0 (0-0)		0 (0-0)		0 (0-0)		0 (0-0)	
2008	Lebanon	0 (0-0)		0 (0-0)		0 (0-0)		0 (0-0)		0 (0-0)		0 (0-0)	
2009	Lebanon	0 (0-0)		0 (0-0)		0 (0-0)		0 (0-0)		0 (0-0)		0 (0-0)	
2010	Lebanon	0 (0-0)		0 (0-0)		0 (0-0)		0 (0-0)		0 (0-0)		0 (0-0)	
2011	Lebanon	0 (0-0)		0 (0-0)		0 (0-0)		0 (0-0)		0 (0-0)		0 (0-0)	
2012	Lebanon	0 (0-0)		0 (0-0)		0 (0-0)		0 (0-0)		0 (0-0)		0 (0-0)	
2013	Lebanon	0 (0-0)		0 (0-0)		0 (0-0)		0 (0-0)		0 (0-0)		0 (0-0)	
2014	Lebanon	0 (0-0)		0 (0-0)		0 (0-0)		0 (0-0)		0 (0-0)		0 (0-0)	
2015	Lebanon	0 (0-0)	0 (0-0)	0 (0-0)	0 (0-0)	0 (0-0)	0 (0-0)	0 (0-0)	0 (0-0)	0 (0-0)	0 (0-0)	0 (0-0)	0 (0-0)
2000	Lesotho	152 (107-200)		57 (40-75)		54 (19-87)		20 (7-33)		0 (0-0)		0 (0-0)	
2001	Lesotho	156 (109-204)		59 (41-77)		55 (19-87)		21 (7-33)		0 (0-0)		0 (0-0)	
2002	Lesotho	157 (111-207)		60 (42-79)		55 (20-87)		21 (8-33)		0 (0-0)		0 (0-0)	
2003	Lesotho	157 (110-206)		61 (43-80)		55 (20-86)		21 (8-34)		0 (0-0)		0 (0-0)	
2004	Lesotho	156 (110-205)		62 (44-81)		54 (20-85)		22 (8-34)		0 (0-0)		0 (0-0)	
2005	Lesotho	156 (110-205)		63 (44-82)		54 (21-85)		22 (8-34)		0 (0-0)		0 (0-0)	
2006	Lesotho	158 (111-207)		64 (45-85)		55 (21-86)		22 (9-35)		0 (0-0)		0 (0-0)	
2007	Lesotho	158 (111-207)		65 (46-85)		55 (21-85)		22 (9-35)		0 (0-0)		0 (0-0)	
2008	Lesotho	162 (114-213)		66 (47-87)		56 (22-87)		23 (9-36)		0 (0-0)		0 (0-0)	
2009	Lesotho	26 (18-34)		10 (7-14)		9 (4-14)		4 (1-6)		0 (0-0)		0 (0-0)	
2010	Lesotho	12 (9-16)		5 (3-6)		4 (2-6)		2 (1-3)		0 (0-0)		0 (0-0)	
2011	Lesotho	12 (8-15)		5 (3-6)		4 (2-6)		2 (1-2)		0 (0-0)		0 (0-0)	
2012	Lesotho	12 (9-16)		5 (3-6)		4 (2-6)		2 (1-2)		0 (0-0)		0 (0-0)	
2013	Lesotho	13 (9-17)		5 (3-6)		4 (2-7)		2 (1-2)		0 (0-0)		0 (0-0)	
2014	Lesotho	2 (1-2)		1 (0-1)		1 (0-1)		0 (0-0)		0 (0-0)		0 (0-0)	
2015	Lesotho	2 (1-2)	0 (0-0)	1 (0-1)	0 (0-0)	1 (0-1)	0 (0-0)	0 (0-0)	0 (0-0)	0 (0-0)	0 (0-0)	0 (0-0)	0 (0-0)
2000	Liberia	415 (292-546)		85 (60-112)		247 (142-344)		51 (29-70)		1 (1-1)		0 (0-0)	
2001	Liberia	414 (291-544)		81 (57-106)		247 (140-345)		48 (27-67)		1 (1-1)		0 (0-0)	
2002	Liberia	401 (282-527)		76 (53-100)		241 (136-338)		46 (26-64)		1 (1-1)		0 (0-0)	
2003	Liberia	384 (270-504)		72 (50-94)		235 (98-362)		44 (18-67)		1 (0-1)		0 (0-0)	
2004	Liberia	370 (261-487)		68 (48-90)		225 (92-348)		42 (17-64)		1 (0-1)		0 (0-0)	
2005	Liberia	367 (258-482)		66 (46-87)		218 (88-338)		39 (16-61)		1 (0-1)		0 (0-0)	
2006	Liberia	364 (256-479)		64 (45-84)		211 (83-329)		37 (15-57)		1 (0-1)		0 (0-0)	
2007	Liberia	367 (258-483)		62 (44-82)		206 (80-322)		35 (13-55)		1 (0-1)		0 (0-0)	
2008	Liberia	134 (94-176)		22 (15-29)		73 (28-115)		12 (5-19)		0 (0-0)		0 (0-0)	
2009	Liberia	47 (33-61)		7 (5-10)		25 (9-39)		4 (1-6)		0 (0-0)		0 (0-0)	
2010	Liberia	56 (40-74)		9 (6-11)		29 (11-46)		5 (2-7)		0 (0-0)		0 (0-0)	
2011	Liberia	36 (26-48)		6 (4-7)		19 (7-30)		3 (1-5)		0 (0-0)		0 (0-0)	
2012	Liberia	33 (24-44)		5 (4-7)		17 (6-27)		3 (1-4)		0 (0-0)		0 (0-0)	
2013	Liberia	3 (2-4)		0 (0-1)		2 (1-3)		0 (0-0)		0 (0-0)		0 (0-0)	
2014	Liberia	47 (33-62)		7 (5-9)		24 (7-40)		4 (1-6)		0 (0-0)		0 (0-0)	
2015	Liberia	45 (32-59)	0 (0-1)	7 (5-9)	0 (0-0)	23 (7-38)	0 (0-0)	3 (1-6)	0 (0-0)	0 (0-0)	0 (0-0)	0 (0-0)	0 (0-0)
2000	Libya	55 (39-72)		10 (7-13)		6 (3-10)		1 (1-2)		0 (0-0)		0 (0-0)	
2001	Libya	53 (37-69)		9 (7-12)		6 (3-10)		1 (1-2)		0 (0-0)		0 (0-0)	
2002	Libya	50 (35-66)		9 (6-12)		6 (3-9)		1 (1-2)		0 (0-0)		0 (0-0)	
2003	Libya	47 (33-62)		8 (6-11)		5 (3-9)		1 (0-2)		0 (0-0)		0 (0-0)	
2004	Libya	44 (31-58)		8 (5-10)		5 (3-8)		1 (0-1)		0 (0-0)		0 (0-0)	
2005	Libya	41 (29-54)		7 (5-9)		5 (2-8)		1 (0-1)		0 (0-0)		0 (0-0)	
2006	Libya	38 (27-50)		6 (4-8)		4 (2-7)		1 (0-1)		0 (0-0)		0 (0-0)	
2007	Libya	17 (12-23)		3 (2-4)		3 (1-5)		0 (0-1)		0 (0-0)		0 (0-0)	
2008	Libya	3 (2-3)		0 (0-1)		0 (0-1)		0 (0-0)		0 (0-0)		0 (0-0)	
2009	Libya	2 (2-3)		0 (0-0)		0 (0-1)		0 (0-0)		0 (0-0)		0 (0-0)	
2010	Libya	2 (2-3)		0 (0-0)		0 (0-1)		0 (0-0)		0 (0-0)		0 (0-0)	
2011	Libya	2 (1-3)		0 (0-0)		0 (0-1)		0 (0-0)		0 (0-0)		0 (0-0)	
2012	Libya	0 (0-0)		0 (0-0)		0 (0-0)		0 (0-0)		0 (0-0)		0 (0-0)	
2013	Libya	0 (0-0)		0 (0-0)		0 (0-0)		0 (0-0)		0 (0-0)		0 (0-0)	
2014	Libya	0 (0-0)		0 (0-0)		0 (0-0)		0 (0-0)		0 (0-0)		0 (0-0)	
2015	Libya	0 (0-0)	0 (0-0)	0 (0-0)	0 (0-0)	0 (0-0)	0 (0-0)	0 (0-0)	0 (0-0)	0 (0-0)	0 (0-0)	0 (0-0)	0 (0-0)
2000	Lithuania	5 (4-7)		3 (2-4)		2 (1-4)		1 (0-2)		0 (0-0)		0 (0-0)	
2001	Lithuania	3 (2-4)		2 (1-2)		2 (1-4)		1 (1-2)		0 (0-0)		0 (0-0)	
2002	Lithuania	5 (3-6)		3 (2-4)		2 (1-4)		1 (1-2)		0 (0-0)		0 (0-0)	
2003	Lithuania	3 (2-4)		2 (1-3)		2 (1-4)		2 (1-3)		0 (0-0)		0 (0-0)	
2004	Lithuania	1 (1-2)		1 (1-1)		0 (0-0)		0 (0-0)		0 (0-0)		0 (0-0)	
2005	Lithuania	1 (0-1)		0 (0-1)		0 (0-0)		0 (0-0)		0 (0-0)		0 (0-0)	
2006	Lithuania	0 (0-0)		0 (0-0)		0 (0-0)		0 (0-0)		0 (0-0)		0 (0-0)	
2007	Lithuania	0 (0-0)		0 (0-0)		0 (0-0)		0 (0-0)		0 (0-0)		0 (0-0)	
2008	Lithuania	0 (0-0)		0 (0-0)		0 (0-0)		0 (0-0)		0 (0-0)		0 (0-0)	
2009	Lithuania	0 (0-0)		0 (0-0)		0 (0-0)		0 (0-0)		0 (0-0)		0 (0-0)	
2010	Lithuania	0 (0-0)		0 (0-0)		0 (0-0)		0 (0-0)		0 (0-0)		0 (0-0)	
2011	Lithuania	0 (0-0)		0 (0-0)		0 (0-0)		0 (0-0)		0 (0-0)		0 (0-0)	
2012	Lithuania	0 (0-0)		0 (0-0)		0 (0-0)		0 (0-0)		0 (0-0)		0 (0-0)	
2013	Lithuania	0 (0-0)		0 (0-0)		0 (0-0)		0 (0-0)		0 (0-0)		0 (0-0)	
2014	Lithuania	0 (0-0)		0 (0-0)		0 (0-0)		0 (0-0)		0 (0-0)		0 (0-0)	

Hib = *Haemophilus influenzae* type b; npnm = non-pneumonia, non-meningitis; HIV = deaths only in HIV-infected children; Uncertainty range provided in parentheses.

Year	Country	Hib pneumonia deaths (UR)	Hib pneumonia deaths, HIV (UR)	Hib pneumonia mortality rate (UR)	Hib pneumonia mortality rate, HIV (UR)	Hib meningitis deaths (UR)	Hib meningitis deaths, HIV (UR)	Hib meningitis mortality rate (UR)	Hib meningitis mortality rate, HIV (UR)	Hib NPNM deaths (UR)	Hib NPNM deaths, HIV (UR)	Hib NPNM mortality rate (UR)	Hib NPNM mortality rate, HIV (UR)
2015	Lithuania	0 (0-0)	0 (0-0)	0 (0-0)	0 (0-0)	0 (0-0)	0 (0-0)	0 (0-0)	0 (0-0)	0 (0-0)	0 (0-0)	0 (0-0)	0 (0-0)
2000	Luxembourg	0 (0-0)		0 (0-0)		0 (0-0)		0 (0-0)		0 (0-0)		0 (0-0)	
2001	Luxembourg	0 (0-0)		0 (0-0)		0 (0-0)		0 (0-0)		0 (0-0)		0 (0-0)	
2002	Luxembourg	0 (0-0)		0 (0-0)		0 (0-0)		0 (0-0)		0 (0-0)		0 (0-0)	
2003	Luxembourg	0 (0-0)		0 (0-0)		0 (0-0)		0 (0-0)		0 (0-0)		0 (0-0)	
2004	Luxembourg	0 (0-0)		0 (0-0)		0 (0-0)		0 (0-0)		0 (0-0)		0 (0-0)	
2005	Luxembourg	0 (0-0)		0 (0-0)		0 (0-0)		0 (0-0)		0 (0-0)		0 (0-0)	
2006	Luxembourg	0 (0-0)		0 (0-0)		0 (0-0)		0 (0-0)		0 (0-0)		0 (0-0)	
2007	Luxembourg	0 (0-0)		0 (0-0)		0 (0-0)		0 (0-0)		0 (0-0)		0 (0-0)	
2008	Luxembourg	0 (0-0)		0 (0-0)		0 (0-0)		0 (0-0)		0 (0-0)		0 (0-0)	
2009	Luxembourg	0 (0-0)		0 (0-0)		0 (0-0)		0 (0-0)		0 (0-0)		0 (0-0)	
2010	Luxembourg	0 (0-0)		0 (0-0)		0 (0-0)		0 (0-0)		0 (0-0)		0 (0-0)	
2011	Luxembourg	0 (0-0)		0 (0-0)		0 (0-0)		0 (0-0)		0 (0-0)		0 (0-0)	
2012	Luxembourg	0 (0-0)		0 (0-0)		0 (0-0)		0 (0-0)		0 (0-0)		0 (0-0)	
2013	Luxembourg	0 (0-0)		0 (0-0)		0 (0-0)		0 (0-0)		0 (0-0)		0 (0-0)	
2014	Luxembourg	0 (0-0)		0 (0-0)		0 (0-0)		0 (0-0)		0 (0-0)		0 (0-0)	
2015	Luxembourg	0 (0-0)	0 (0-0)	0 (0-0)	0 (0-0)	0 (0-0)	0 (0-0)	0 (0-0)	0 (0-0)	0 (0-0)	0 (0-0)	0 (0-0)	0 (0-0)
2000	Madagascar	2078 (1461-2730)		74 (52-98)		850 (287-1366)		30 (10-49)		3 (1-6)		0 (0-0)	
2001	Madagascar	1998 (1405-2626)		70 (49-92)		821 (278-1318)		29 (10-46)		3 (1-5)		0 (0-0)	
2002	Madagascar	1906 (1341-2504)		65 (46-86)		789 (268-1266)		27 (9-43)		3 (1-5)		0 (0-0)	
2003	Madagascar	1810 (1273-2378)		61 (43-80)		752 (256-1206)		25 (9-41)		3 (1-5)		0 (0-0)	
2004	Madagascar	1709 (1202-2246)		57 (40-75)		712 (242-1141)		24 (8-38)		3 (1-5)		0 (0-0)	
2005	Madagascar	1627 (1144-2137)		53 (37-70)		676 (227-1086)		22 (7-36)		3 (1-4)		0 (0-0)	
2006	Madagascar	1546 (1087-2031)		50 (35-66)		639 (212-1029)		21 (7-33)		3 (1-4)		0 (0-0)	
2007	Madagascar	1482 (1042-1947)		47 (33-62)		610 (180-1020)		19 (6-32)		5 (1-8)		0 (0-0)	
2008	Madagascar	727 (511-955)		23 (16-30)		299 (87-500)		9 (3-16)		2 (1-4)		0 (0-0)	
2009	Madagascar	145 (102-190)		4 (3-6)		60 (17-100)		2 (1-3)		0 (0-1)		0 (0-0)	
2010	Madagascar	155 (109-204)		5 (3-6)		64 (19-107)		2 (1-3)		1 (0-1)		0 (0-0)	
2011	Madagascar	148 (104-194)		4 (3-6)		61 (18-103)		2 (1-3)		0 (0-1)		0 (0-0)	
2012	Madagascar	152 (107-199)		4 (3-6)		63 (18-106)		2 (1-3)		1 (0-1)		0 (0-0)	
2013	Madagascar	13 (9-17)		0 (0-0)		5 (2-9)		0 (0-0)		0 (0-0)		0 (0-0)	
2014	Madagascar	13 (9-17)		0 (0-0)		5 (2-9)		0 (0-0)		0 (0-0)		0 (0-0)	
2015	Madagascar	13 (9-17)	0 (0-0)	0 (0-0)	0 (0-0)	5 (2-9)	0 (0-0)	0 (0-0)	0 (0-0)	0 (0-0)	0 (0-0)	0 (0-0)	0 (0-0)
2000	Malawi	1921 (1351-2525)		96 (67-126)		1028 (460-1543)		51 (23-77)		4 (2-6)		0 (0-0)	
2001	Malawi	1818 (1279-2389)		88 (62-116)		975 (441-1458)		47 (21-71)		4 (2-6)		0 (0-0)	
2002	Malawi	604 (425-793)		29 (20-38)		324 (97-533)		15 (5-25)		1 (0-2)		0 (0-0)	
2003	Malawi	140 (98-183)		6 (5-9)		75 (23-123)		3 (1-6)		0 (0-1)		0 (0-0)	
2004	Malawi	117 (82-153)		5 (4-7)		63 (20-102)		3 (1-5)		0 (0-0)		0 (0-0)	
2005	Malawi	100 (70-131)		4 (3-6)		53 (18-86)		2 (1-4)		0 (0-0)		0 (0-0)	
2006	Malawi	76 (53-100)		3 (2-4)		40 (14-64)		2 (1-3)		0 (0-0)		0 (0-0)	
2007	Malawi	12 (9-16)		1 (0-1)		6 (2-10)		0 (0-0)		0 (0-0)		0 (0-0)	
2008	Malawi	12 (8-16)		0 (0-1)		6 (2-10)		0 (0-0)		0 (0-0)		0 (0-0)	
2009	Malawi	12 (8-16)		0 (0-1)		6 (2-9)		0 (0-0)		0 (0-0)		0 (0-0)	
2010	Malawi	11 (8-15)		0 (0-1)		6 (2-9)		0 (0-0)		0 (0-0)		0 (0-0)	
2011	Malawi	12 (8-15)		0 (0-1)		6 (2-9)		0 (0-0)		0 (0-0)		0 (0-0)	
2012	Malawi	11 (8-15)		0 (0-1)		6 (2-9)		0 (0-0)		0 (0-0)		0 (0-0)	
2013	Malawi	11 (8-15)		0 (0-1)		6 (2-9)		0 (0-0)		0 (0-0)		0 (0-0)	
2014	Malawi	11 (8-14)		0 (0-1)		5 (2-9)		0 (0-0)		0 (0-0)		0 (0-0)	
2015	Malawi	11 (7-14)	0 (0-1)	0 (0-0)	0 (0-0)	5 (2-8)	0 (0-0)	0 (0-0)	0 (0-0)	0 (0-0)	0 (0-0)	0 (0-0)	0 (0-0)
2000	Malaysia	74 (52-97)		3 (2-4)		19 (10-32)		1 (0-1)		0 (0-0)		0 (0-0)	
2001	Malaysia	65 (45-85)		2 (2-3)		17 (8-28)		1 (0-1)		0 (0-0)		0 (0-0)	
2002	Malaysia	58 (41-76)		2 (2-3)		15 (8-25)		1 (0-1)		0 (0-0)		0 (0-0)	
2003	Malaysia	52 (37-68)		2 (1-3)		14 (7-23)		1 (0-1)		0 (0-0)		0 (0-0)	
2004	Malaysia	14 (10-19)		1 (0-1)		5 (3-9)		0 (0-0)		0 (0-0)		0 (0-0)	
2005	Malaysia	5 (3-6)		0 (0-0)		2 (1-3)		0 (0-0)		0 (0-0)		0 (0-0)	
2006	Malaysia	4 (3-5)		0 (0-0)		1 (1-2)		0 (0-0)		0 (0-0)		0 (0-0)	
2007	Malaysia	3 (2-4)		0 (0-0)		1 (1-2)		0 (0-0)		0 (0-0)		0 (0-0)	
2008	Malaysia	3 (2-4)		0 (0-0)		1 (1-2)		0 (0-0)		0 (0-0)		0 (0-0)	
2009	Malaysia	0 (0-1)		0 (0-0)		0 (0-0)		0 (0-0)		0 (0-0)		0 (0-0)	
2010	Malaysia	0 (0-1)		0 (0-0)		0 (0-0)		0 (0-0)		0 (0-0)		0 (0-0)	
2011	Malaysia	0 (0-1)		0 (0-0)		0 (0-0)		0 (0-0)		0 (0-0)		0 (0-0)	
2012	Malaysia	0 (0-1)		0 (0-0)		0 (0-0)		0 (0-0)		0 (0-0)		0 (0-0)	
2013	Malaysia	0 (0-1)		0 (0-0)		0 (0-0)		0 (0-0)		0 (0-0)		0 (0-0)	
2014	Malaysia	0 (0-0)		0 (0-0)		0 (0-0)		0 (0-0)		0 (0-0)		0 (0-0)	
2015	Malaysia	0 (0-0)	0 (0-0)	0 (0-0)	0 (0-0)	0 (0-0)	0 (0-0)	0 (0-0)	0 (0-0)	0 (0-0)	0 (0-0)	0 (0-0)	0 (0-0)
2000	Maldives	6 (4-7)		17 (12-22)		1 (0-2)		4 (1-6)		0 (0-0)		0 (0-0)	
2001	Maldives	4 (3-6)		14 (10-19)		1 (0-2)		3 (1-5)		0 (0-0)		0 (0-0)	
2002	Maldives	4 (2-5)		12 (8-15)		1 (0-1)		3 (1-4)		0 (0-0)		0 (0-0)	
2003	Maldives	3 (2-4)		10 (7-13)		1 (0-1)		2 (0-4)		0 (0-0)		0 (0-0)	
2004	Maldives	2 (2-3)		8 (6-11)		1 (0-1)		2 (0-3)		0 (0-0)		0 (0-0)	
2005	Maldives	2 (2-3)		7 (5-10)		1 (0-1)		2 (0-3)		0 (0-0)		0 (0-0)	
2006	Maldives	2 (1-3)		6 (5-8)		0 (0-1)		2 (0-3)		0 (0-0)		0 (0-0)	
2007	Maldives	2 (1-2)		5 (4-7)		0 (0-1)		1 (0-2)		0 (0-0)		0 (0-0)	
2008	Maldives	1 (1-2)		5 (3-6)		0 (0-1)		1 (0-2)		0 (0-0)		0 (0-0)	
2009	Maldives	1 (1-2)		4 (3-5)		0 (0-1)		1 (0-2)		0 (0-0)		0 (0-0)	
2010	Maldives	1 (1-2)		4 (3-5)		0 (0-0)		1 (0-2)		0 (0-0)		0 (0-0)	
2011	Maldives	1 (1-1)		3 (2-4)		0 (0-0)		1 (0-1)		0 (0-0)		0 (0-0)	
2012	Maldives	1 (1-1)		3 (2-3)		0 (0-0)		1 (0-1)		0 (0-0)		0 (0-0)	
2013	Maldives	0 (0-0)		1 (1-1)		0 (0-0)		0 (0-1)		0 (0-0)		0 (0-0)	
2014	Maldives	0 (0-0)		0 (0-0)		0 (0-0)		0 (0-0)		0 (0-0)		0 (0-0)	
2015	Maldives	0 (0-0)	0 (0-0)	0 (0-0)	0 (0-0)	0 (0-0)	0 (0-0)	0 (0-0)	0 (0-0)	0 (0-0)	0 (0-0)	0 (0-0)	0 (0-0)
2000	Mali	2288 (1609-3006)		112 (79-147)		966 (450-1434)		47 (22-70)		4 (2-6)		0 (0-0)	
2001	Mali	2300 (1618-3022)		109 (76-143)		962 (449-1427)		45 (21-67)		4 (2-6)		0 (0-0)	
2002	Mali	2273 (1599-2986)		104 (73-137)		943 (441-1399)		43 (20-64)		4 (2-6)		0 (0-0)	
2003	Mali	2176 (1530-2859)		96 (68-127)		898 (420-1331)		40 (19-59)		4 (2-5)		0 (0-0)	
2004	Mali	2136 (1502-2807)		92 (65-121)		877 (411-1300)		38 (18-56)		4 (2-5)		0 (0-0)	
2005	Mali	2109 (1483-2771)		88 (62-115)		862 (405-1277)		36 (17-53)		4 (2-5)		0 (0-0)	
2006	Mali	1649 (1160-2167)		66 (46-86)		670 (316-992)		27 (13-40)		3 (1-4)		0 (0-0)	

Hib = *Haemophilus influenzae* type b; npnm = non-pneumonia, non-meningitis; HIV = deaths only in HIV-infected children; Uncertainty range provided in parentheses.

Year	Country	Hib pneumonia deaths (UR)	Hib pneumonia deaths, HIV (UR)	Hib pneumonia mortality rate (UR)	Hib pneumonia mortality rate, HIV (UR)	Hib meningitis deaths (UR)	Hib meningitis deaths, HIV (UR)	Hib meningitis mortality rate (UR)	Hib meningitis mortality rate, HIV (UR)	Hib NPNM deaths (UR)	Hib NPNM deaths, HIV (UR)	Hib NPNM mortality rate (UR)	Hib NPNM mortality rate, HIV (UR)
2007	Mali	375 (263-492)		14 (10-19)		151 (72-224)		6 (3-9)		1 (0-1)		0 (0-0)	
2008	Mali	256 (180-337)		10 (7-12)		103 (33-168)		4 (1-6)		0 (0-1)		0 (0-0)	
2009	Mali	269 (189-353)		10 (7-13)		108 (35-175)		4 (1-6)		0 (0-1)		0 (0-0)	
2010	Mali	24 (17-32)		1 (1-1)		10 (3-16)		0 (0-1)		0 (0-0)		0 (0-0)	
2011	Mali	23 (16-31)		1 (1-1)		9 (3-15)		0 (0-1)		0 (0-0)		0 (0-0)	
2012	Mali	22 (15-28)		1 (1-1)		9 (3-14)		0 (0-0)		0 (0-0)		0 (0-0)	
2013	Mali	21 (15-27)		1 (0-1)		8 (3-13)		0 (0-0)		0 (0-0)		0 (0-0)	
2014	Mali	20 (14-26)		1 (0-1)		8 (3-13)		0 (0-0)		0 (0-0)		0 (0-0)	
2015	Mali	20 (14-26)	0 (0-0)	1 (0-1)	0 (0-0)	8 (3-13)	0 (0-0)	0 (0-0)	0 (0-0)	0 (0-0)	0 (0-0)	0 (0-0)	0 (0-0)
2000	Malta	0 (0-0)		0 (0-0)		0 (0-0)		0 (0-0)		0 (0-0)		0 (0-0)	
2001	Malta	0 (0-0)		0 (0-0)		0 (0-0)		0 (0-0)		0 (0-0)		0 (0-0)	
2002	Malta	0 (0-0)		0 (0-0)		0 (0-0)		0 (0-0)		0 (0-0)		0 (0-0)	
2003	Malta	0 (0-0)		0 (0-0)		0 (0-0)		0 (0-0)		0 (0-0)		0 (0-0)	
2004	Malta	0 (0-0)		0 (0-0)		0 (0-0)		0 (0-0)		0 (0-0)		0 (0-0)	
2005	Malta	0 (0-0)		0 (0-0)		0 (0-0)		0 (0-0)		0 (0-0)		0 (0-0)	
2006	Malta	0 (0-0)		0 (0-0)		0 (0-0)		0 (0-0)		0 (0-0)		0 (0-0)	
2007	Malta	0 (0-0)		0 (0-0)		0 (0-0)		0 (0-0)		0 (0-0)		0 (0-0)	
2008	Malta	0 (0-0)		0 (0-0)		0 (0-0)		0 (0-0)		0 (0-0)		0 (0-0)	
2009	Malta	0 (0-0)		0 (0-0)		0 (0-0)		0 (0-0)		0 (0-0)		0 (0-0)	
2010	Malta	0 (0-0)		0 (0-0)		0 (0-0)		0 (0-0)		0 (0-0)		0 (0-0)	
2011	Malta	0 (0-0)		0 (0-0)		0 (0-0)		0 (0-0)		0 (0-0)		0 (0-0)	
2012	Malta	0 (0-0)		0 (0-0)		0 (0-0)		0 (0-0)		0 (0-0)		0 (0-0)	
2013	Malta	0 (0-0)		0 (0-0)		0 (0-0)		0 (0-0)		0 (0-0)		0 (0-0)	
2014	Malta	0 (0-0)		0 (0-0)		0 (0-0)		0 (0-0)		0 (0-0)		0 (0-0)	
2015	Malta	0 (0-0)	0 (0-0)	0 (0-0)	0 (0-0)	0 (0-0)	0 (0-0)	0 (0-0)	0 (0-0)	0 (0-0)	0 (0-0)	0 (0-0)	0 (0-0)
2000	Marshall Islands	2 (1-2)		14 (10-18)		1 (0-1)		6 (3-8)		0 (0-0)		0 (0-0)	
2001	Marshall Islands	0 (0-1)		4 (3-5)		0 (0-0)		2 (1-2)		0 (0-0)		0 (0-0)	
2002	Marshall Islands	0 (0-0)		2 (2-3)		0 (0-0)		1 (0-1)		0 (0-0)		0 (0-0)	
2003	Marshall Islands	0 (0-0)		3 (2-4)		0 (0-0)		1 (1-2)		0 (0-0)		0 (0-0)	
2004	Marshall Islands	0 (0-0)		4 (3-5)		0 (0-0)		2 (1-2)		0 (0-0)		0 (0-0)	
2005	Marshall Islands	0 (0-0)		0 (0-0)		0 (0-0)		0 (0-0)		0 (0-0)		0 (0-0)	
2006	Marshall Islands	0 (0-0)		3 (2-4)		0 (0-0)		1 (1-2)		0 (0-0)		0 (0-0)	
2007	Marshall Islands	0 (0-0)		0 (0-0)		0 (0-0)		0 (0-0)		0 (0-0)		0 (0-0)	
2008	Marshall Islands	0 (0-0)		0 (0-0)		0 (0-0)		0 (0-0)		0 (0-0)		0 (0-0)	
2009	Marshall Islands	0 (0-0)		0 (0-0)		0 (0-0)		0 (0-0)		0 (0-0)		0 (0-0)	
2010	Marshall Islands	0 (0-0)		0 (0-0)		0 (0-0)		0 (0-0)		0 (0-0)		0 (0-0)	
2011	Marshall Islands	0 (0-0)		0 (0-0)		0 (0-0)		0 (0-0)		0 (0-0)		0 (0-0)	
2012	Marshall Islands	0 (0-0)		0 (0-0)		0 (0-0)		0 (0-0)		0 (0-0)		0 (0-0)	
2013	Marshall Islands	0 (0-0)		0 (0-0)		0 (0-0)		0 (0-0)		0 (0-0)		0 (0-0)	
2014	Marshall Islands	0 (0-0)		3 (2-4)		0 (0-0)		1 (1-2)		0 (0-0)		0 (0-0)	
2015	Marshall Islands	0 (0-0)	0 (0-0)	0 (0-0)	0 (0-0)	0 (0-0)	0 (0-0)	0 (0-0)	0 (0-0)	0 (0-0)	0 (0-0)	0 (0-0)	0 (0-0)
2000	Mauritania	289 (203-380)		66 (46-87)		100 (32-161)		23 (7-37)		0 (0-1)		0 (0-0)	
2001	Mauritania	300 (211-395)		67 (47-88)		104 (33-168)		23 (7-37)		0 (0-1)		0 (0-0)	
2002	Mauritania	312 (219-409)		68 (48-89)		108 (35-175)		23 (8-38)		0 (0-1)		0 (0-0)	
2003	Mauritania	321 (226-421)		68 (48-89)		111 (36-179)		23 (8-38)		0 (0-1)		0 (0-0)	
2004	Mauritania	327 (230-430)		68 (48-89)		112 (37-181)		23 (8-38)		0 (0-1)		0 (0-0)	
2005	Mauritania	332 (233-436)		67 (47-88)		113 (37-182)		23 (8-37)		0 (0-1)		0 (0-0)	
2006	Mauritania	335 (235-440)		66 (47-87)		113 (37-183)		22 (7-36)		0 (0-1)		0 (0-0)	
2007	Mauritania	337 (237-443)		65 (46-86)		114 (38-183)		22 (7-36)		0 (0-1)		0 (0-0)	
2008	Mauritania	337 (237-443)		64 (45-84)		114 (38-184)		22 (7-35)		0 (0-1)		0 (0-0)	
2009	Mauritania	121 (85-159)		23 (16-30)		41 (14-66)		8 (3-12)		0 (0-0)		0 (0-0)	
2010	Mauritania	42 (30-56)		8 (5-10)		14 (5-23)		3 (1-4)		0 (0-0)		0 (0-0)	
2011	Mauritania	36 (25-47)		6 (5-9)		12 (4-20)		2 (1-4)		0 (0-0)		0 (0-0)	
2012	Mauritania	33 (23-43)		6 (4-8)		11 (4-18)		2 (1-3)		0 (0-0)		0 (0-0)	
2013	Mauritania	33 (23-43)		6 (4-8)		11 (4-18)		2 (1-3)		0 (0-0)		0 (0-0)	
2014	Mauritania	3 (2-4)		1 (0-1)		1 (0-2)		0 (0-0)		0 (0-0)		0 (0-0)	
2015	Mauritania	3 (2-4)	0 (0-0)	1 (0-1)	0 (0-0)	1 (0-2)	0 (0-0)	0 (0-0)	0 (0-0)	0 (0-0)	0 (0-0)	0 (0-0)	0 (0-0)
2000	Mauritius	2 (1-3)		2 (1-3)		1 (1-2)		1 (1-2)		0 (0-0)		0 (0-0)	
2001	Mauritius	3 (2-4)		3 (2-4)		1 (1-2)		1 (1-2)		0 (0-0)		0 (0-0)	
2002	Mauritius	2 (2-3)		2 (2-3)		0 (0-1)		1 (0-1)		0 (0-0)		0 (0-0)	
2003	Mauritius	3 (2-5)		4 (3-5)		1 (0-1)		1 (0-1)		0 (0-0)		0 (0-0)	
2004	Mauritius	4 (3-5)		4 (3-5)		1 (0-1)		1 (0-1)		0 (0-0)		0 (0-0)	
2005	Mauritius	3 (2-3)		3 (2-4)		1 (0-1)		1 (0-1)		0 (0-0)		0 (0-0)	
2006	Mauritius	1 (0-1)		1 (0-1)		0 (0-1)		0 (0-1)		0 (0-0)		0 (0-0)	
2007	Mauritius	0 (0-0)		0 (0-0)		0 (0-0)		0 (0-0)		0 (0-0)		0 (0-0)	
2008	Mauritius	0 (0-0)		0 (0-0)		0 (0-0)		0 (0-0)		0 (0-0)		0 (0-0)	
2009	Mauritius	0 (0-0)		0 (0-0)		0 (0-0)		0 (0-0)		0 (0-0)		0 (0-0)	
2010	Mauritius	0 (0-0)		0 (0-0)		0 (0-0)		0 (0-0)		0 (0-0)		0 (0-0)	
2011	Mauritius	0 (0-0)		0 (0-0)		0 (0-0)		0 (0-0)		0 (0-0)		0 (0-0)	
2012	Mauritius	0 (0-0)		0 (0-0)		0 (0-0)		0 (0-0)		0 (0-0)		0 (0-0)	
2013	Mauritius	0 (0-0)		0 (0-0)		0 (0-0)		0 (0-0)		0 (0-0)		0 (0-0)	
2014	Mauritius	0 (0-0)		0 (0-0)		0 (0-0)		0 (0-0)		0 (0-0)		0 (0-0)	
2015	Mauritius	0 (0-0)	0 (0-0)	0 (0-0)	0 (0-0)	0 (0-0)	0 (0-0)	0 (0-0)	0 (0-0)	0 (0-0)	0 (0-0)	0 (0-0)	0 (0-0)
2000	Mexico	309 (217-406)		3 (2-3)		54 (23-95)		0 (0-1)		0 (0-1)		0 (0-0)	
2001	Mexico	129 (91-169)		1 (1-1)		20 (9-35)		0 (0-0)		0 (0-0)		0 (0-0)	
2002	Mexico	132 (93-173)		1 (1-1)		18 (8-31)		0 (0-0)		0 (0-0)		0 (0-0)	
2003	Mexico	110 (78-145)		1 (1-1)		18 (8-31)		0 (0-0)		0 (0-0)		0 (0-0)	
2004	Mexico	111 (78-146)		1 (1-1)		16 (7-29)		0 (0-0)		0 (0-0)		0 (0-0)	
2005	Mexico	14 (10-18)		0 (0-0)		2 (1-4)		0 (0-0)		0 (0-0)		0 (0-0)	
2006	Mexico	13 (9-17)		0 (0-0)		2 (1-4)		0 (0-0)		0 (0-0)		0 (0-0)	
2007	Mexico	12 (9-16)		0 (0-0)		2 (1-4)		0 (0-0)		0 (0-0)		0 (0-0)	
2008	Mexico	12 (8-15)		0 (0-0)		2 (1-3)		0 (0-0)		0 (0-0)		0 (0-0)	
2009	Mexico	12 (8-15)		0 (0-0)		1 (1-3)		0 (0-0)		0 (0-0)		0 (0-0)	
2010	Mexico	11 (8-14)		0 (0-0)		2 (1-3)		0 (0-0)		0 (0-0)		0 (0-0)	
2011	Mexico	10 (7-13)		0 (0-0)		2 (1-3)		0 (0-0)		0 (0-0)		0 (0-0)	
2012	Mexico	9 (6-11)		0 (0-0)		1 (1-2)		0 (0-0)		0 (0-0)		0 (0-0)	
2013	Mexico	8 (6-10)		0 (0-0)		1 (1-2)		0 (0-0)		0 (0-0)		0 (0-0)	
2014	Mexico	8 (6-10)		0 (0-0)		1 (1-2)		0 (0-0)		0 (0-0)		0 (0-0)	

Hib = *Haemophilus influenzae* type b; npnm = non-pneumonia, non-meningitis; HIV = deaths only in HIV-infected children; Uncertainty range provided in parentheses.

Year	Country	Hib pneumonia deaths (UR)	Hib pneumonia deaths, HIV (UR)	Hib pneumonia mortality rate (UR)	Hib pneumonia mortality rate, HIV (UR)	Hib meningitis deaths (UR)	Hib meningitis deaths, HIV (UR)	Hib meningitis mortality rate (UR)	Hib meningitis mortality rate, HIV (UR)	Hib NPNM deaths (UR)	Hib NPNM deaths, HIV (UR)	Hib NPNM mortality rate (UR)	Hib NPNM mortality rate, HIV (UR)
2015	Mexico	8 (5-10)	0 (0-0)	0 (0-0)	0 (0-0)	1 (1-2)	0 (0-0)	0 (0-0)	0 (0-0)	0 (0-0)	0 (0-0)	0 (0-0)	0 (0-0)
2000	Micronesia (Federated States of)	0 (0-1)		3 (2-4)		0 (0-0)		2 (1-2)		0 (0-0)		0 (0-0)	
2001	Micronesia (Federated States of)	0 (0-1)		3 (2-4)		0 (0-0)		2 (1-2)		0 (0-0)		0 (0-0)	
2002	Micronesia (Federated States of)	0 (0-1)		3 (2-4)		0 (0-0)		2 (1-2)		0 (0-0)		0 (0-0)	
2003	Micronesia (Federated States of)	0 (0-1)		3 (2-4)		0 (0-0)		2 (1-2)		0 (0-0)		0 (0-0)	
2004	Micronesia (Federated States of)	0 (0-1)		3 (2-4)		0 (0-0)		2 (1-2)		0 (0-0)		0 (0-0)	
2005	Micronesia (Federated States of)	0 (0-0)		0 (0-0)		0 (0-0)		0 (0-0)		0 (0-0)		0 (0-0)	
2006	Micronesia (Federated States of)	0 (0-1)		3 (2-4)		0 (0-0)		2 (1-2)		0 (0-0)		0 (0-0)	
2007	Micronesia (Federated States of)	0 (0-0)		0 (0-0)		0 (0-0)		0 (0-0)		0 (0-0)		0 (0-0)	
2008	Micronesia (Federated States of)	0 (0-0)		0 (0-0)		0 (0-0)		0 (0-0)		0 (0-0)		0 (0-0)	
2009	Micronesia (Federated States of)	0 (0-0)		0 (0-0)		0 (0-0)		0 (0-0)		0 (0-0)		0 (0-0)	
2010	Micronesia (Federated States of)	0 (0-0)		0 (0-0)		0 (0-0)		0 (0-0)		0 (0-0)		0 (0-0)	
2011	Micronesia (Federated States of)	0 (0-0)		0 (0-0)		0 (0-0)		0 (0-0)		0 (0-0)		0 (0-0)	
2012	Micronesia (Federated States of)	0 (0-0)		0 (0-0)		0 (0-0)		0 (0-0)		0 (0-0)		0 (0-0)	
2013	Micronesia (Federated States of)	0 (0-0)		0 (0-0)		0 (0-0)		0 (0-0)		0 (0-0)		0 (0-0)	
2014	Micronesia (Federated States of)	0 (0-0)		2 (2-3)		0 (0-0)		1 (1-1)		0 (0-0)		0 (0-0)	
2015	Micronesia (Federated States of)	0 (0-0)	0 (0-0)	3 (2-3)	0 (0-0)	0 (0-0)	0 (0-0)	1 (1-2)	0 (0-0)	0 (0-0)	0 (0-0)	0 (0-0)	0 (0-0)
2000	Monaco	0 (0-0)		0 (0-0)		0 (0-0)		0 (0-0)		0 (0-0)		0 (0-0)	
2001	Monaco	0 (0-0)		0 (0-0)		0 (0-0)		0 (0-0)		0 (0-0)		0 (0-0)	
2002	Monaco	0 (0-0)		0 (0-0)		0 (0-0)		0 (0-0)		0 (0-0)		0 (0-0)	
2003	Monaco	0 (0-0)		0 (0-0)		0 (0-0)		0 (0-0)		0 (0-0)		0 (0-0)	
2004	Monaco	0 (0-0)		0 (0-0)		0 (0-0)		0 (0-0)		0 (0-0)		0 (0-0)	
2005	Monaco	0 (0-0)		0 (0-0)		0 (0-0)		0 (0-0)		0 (0-0)		0 (0-0)	
2006	Monaco	0 (0-0)		0 (0-0)		0 (0-0)		0 (0-0)		0 (0-0)		0 (0-0)	
2007	Monaco	0 (0-0)		0 (0-0)		0 (0-0)		0 (0-0)		0 (0-0)		0 (0-0)	
2008	Monaco	0 (0-0)		0 (0-0)		0 (0-0)		0 (0-0)		0 (0-0)		0 (0-0)	
2009	Monaco	0 (0-0)		0 (0-0)		0 (0-0)		0 (0-0)		0 (0-0)		0 (0-0)	
2010	Monaco	0 (0-0)		0 (0-0)		0 (0-0)		0 (0-0)		0 (0-0)		0 (0-0)	
2011	Monaco	0 (0-0)		0 (0-0)		0 (0-0)		0 (0-0)		0 (0-0)		0 (0-0)	
2012	Monaco	0 (0-0)		0 (0-0)		0 (0-0)		0 (0-0)		0 (0-0)		0 (0-0)	
2013	Monaco	0 (0-0)		0 (0-0)		0 (0-0)		0 (0-0)		0 (0-0)		0 (0-0)	
2014	Monaco	0 (0-0)		0 (0-0)		0 (0-0)		0 (0-0)		0 (0-0)		0 (0-0)	
2015	Monaco	0 (0-0)	0 (0-0)	0 (0-0)	0 (0-0)	0 (0-0)	0 (0-0)	0 (0-0)	0 (0-0)	0 (0-0)	0 (0-0)	0 (0-0)	0 (0-0)
2000	Mongolia	89 (62-116)		39 (27-51)		28 (12-43)		12 (5-19)		0 (0-0)		0 (0-0)	
2001	Mongolia	80 (56-105)		35 (25-46)		25 (10-39)		11 (5-17)		0 (0-0)		0 (0-0)	
2002	Mongolia	71 (50-93)		32 (23-42)		22 (9-35)		10 (4-16)		0 (0-0)		0 (0-0)	
2003	Mongolia	65 (46-86)		30 (21-39)		20 (8-32)		9 (4-15)		0 (0-0)		0 (0-0)	
2004	Mongolia	60 (42-79)		28 (20-37)		18 (7-29)		9 (3-13)		0 (0-0)		0 (0-0)	
2005	Mongolia	45 (32-59)		21 (14-27)		13 (5-21)		6 (2-10)		0 (0-0)		0 (0-0)	
2006	Mongolia	7 (5-10)		3 (2-4)		2 (1-3)		1 (0-2)		0 (0-0)		0 (0-0)	
2007	Mongolia	5 (4-7)		2 (2-3)		2 (1-2)		1 (0-1)		0 (0-0)		0 (0-0)	
2008	Mongolia	4 (3-5)		1 (1-2)		1 (0-2)		0 (0-1)		0 (0-0)		0 (0-0)	
2009	Mongolia	4 (3-5)		1 (1-2)		1 (0-1)		0 (0-1)		0 (0-0)		0 (0-0)	
2010	Mongolia	1 (0-1)		0 (0-0)		0 (0-0)		0 (0-0)		0 (0-0)		0 (0-0)	
2011	Mongolia	1 (0-1)		0 (0-0)		0 (0-0)		0 (0-0)		0 (0-0)		0 (0-0)	
2012	Mongolia	0 (0-1)		0 (0-0)		0 (0-0)		0 (0-0)		0 (0-0)		0 (0-0)	
2013	Mongolia	0 (0-1)		0 (0-0)		0 (0-0)		0 (0-0)		0 (0-0)		0 (0-0)	
2014	Mongolia	0 (0-1)		0 (0-0)		0 (0-0)		0 (0-0)		0 (0-0)		0 (0-0)	
2015	Mongolia	0 (0-1)	0 (0-0)	0 (0-0)	0 (0-0)	0 (0-0)	0 (0-0)	0 (0-0)	0 (0-0)	0 (0-0)	0 (0-0)	0 (0-0)	0 (0-0)
2000	Montenegro	0 (0-0)		0 (0-1)		0 (0-0)		0 (0-0)		0 (0-0)		0 (0-0)	
2001	Montenegro	0 (0-0)		0 (0-0)		0 (0-0)		0 (0-0)		0 (0-0)		0 (0-0)	
2002	Montenegro	0 (0-0)		0 (0-0)		0 (0-1)		1 (0-1)		0 (0-0)		0 (0-0)	
2003	Montenegro	0 (0-0)		0 (0-0)		0 (0-1)		1 (0-1)		0 (0-0)		0 (0-0)	
2004	Montenegro	0 (0-0)		0 (0-1)		1 (0-1)		1 (0-3)		0 (0-0)		0 (0-0)	
2005	Montenegro	0 (0-0)		1 (1-1)		0 (0-1)		1 (0-1)		0 (0-0)		0 (0-0)	
2006	Montenegro	0 (0-0)		0 (0-0)		0 (0-0)		0 (0-0)		0 (0-0)		0 (0-0)	
2007	Montenegro	0 (0-0)		0 (0-0)		0 (0-0)		0 (0-0)		0 (0-0)		0 (0-0)	
2008	Montenegro	0 (0-0)		0 (0-0)		0 (0-0)		0 (0-0)		0 (0-0)		0 (0-0)	
2009	Montenegro	0 (0-0)		0 (0-0)		0 (0-0)		0 (0-0)		0 (0-0)		0 (0-0)	
2010	Montenegro	0 (0-0)		0 (0-0)		0 (0-0)		0 (0-0)		0 (0-0)		0 (0-0)	
2011	Montenegro	0 (0-0)		0 (0-0)		0 (0-0)		0 (0-0)		0 (0-0)		0 (0-0)	
2012	Montenegro	0 (0-0)		0 (0-0)		0 (0-0)		0 (0-0)		0 (0-0)		0 (0-0)	
2013	Montenegro	0 (0-0)		0 (0-0)		0 (0-0)		0 (0-0)		0 (0-0)		0 (0-0)	
2014	Montenegro	0 (0-0)		0 (0-0)		0 (0-0)		0 (0-0)		0 (0-0)		0 (0-0)	
2015	Montenegro	0 (0-0)	0 (0-0)	0 (0-0)	0 (0-0)	0 (0-0)	0 (0-0)	0 (0-0)	0 (0-0)	0 (0-0)	0 (0-0)	0 (0-0)	0 (0-0)
2000	Morocco	748 (526-983)		25 (17-32)		226 (63-382)		7 (2-13)		2 (0-3)		0 (0-0)	
2001	Morocco	685 (482-900)		23 (16-30)		205 (57-346)		7 (2-12)		2 (0-3)		0 (0-0)	
2002	Morocco	632 (445-831)		22 (15-28)		188 (52-316)		6 (2-11)		1 (0-3)		0 (0-0)	
2003	Morocco	590 (415-776)		20 (14-27)		173 (49-291)		6 (2-10)		1 (0-2)		0 (0-0)	
2004	Morocco	554 (390-728)		19 (14-25)		160 (45-271)		6 (2-9)		1 (0-2)		0 (0-0)	
2005	Morocco	524 (368-688)		18 (13-24)		150 (44-250)		5 (2-9)		1 (0-2)		0 (0-0)	
2006	Morocco	496 (349-652)		17 (12-23)		140 (43-232)		5 (2-8)		1 (0-2)		0 (0-0)	
2007	Morocco	75 (52-98)		3 (2-3)		21 (7-34)		1 (0-1)		0 (0-0)		0 (0-0)	
2008	Morocco	27 (19-36)		1 (1-1)		7 (3-12)		0 (0-0)		0 (0-0)		0 (0-0)	
2009	Morocco	27 (19-35)		1 (1-1)		7 (3-12)		0 (0-0)		0 (0-0)		0 (0-0)	
2010	Morocco	26 (18-34)		1 (1-1)		7 (3-11)		0 (0-0)		0 (0-0)		0 (0-0)	
2011	Morocco	26 (19-35)		1 (1-1)		7 (3-11)		0 (0-0)		0 (0-0)		0 (0-0)	
2012	Morocco	4 (3-6)		0 (0-0)		1 (0-2)		0 (0-0)		0 (0-0)		0 (0-0)	
2013	Morocco	4 (3-6)		0 (0-0)		1 (0-2)		0 (0-0)		0 (0-0)		0 (0-0)	
2014	Morocco	4 (3-5)		0 (0-0)		1 (0-2)		0 (0-0)		0 (0-0)		0 (0-0)	
2015	Morocco	4 (3-5)	0 (0-0)	0 (0-0)	0 (0-0)	1 (0-2)	0 (0-0)	0 (0-0)	0 (0-0)	0 (0-0)	0 (0-0)	0 (0-0)	0 (0-0)
2000	Mozambique	2454 (1726-3224)		75 (53-98)		1312 (653-1915)		40 (20-58)		5 (3-8)		0 (0-0)	
2001	Mozambique	2422 (1704-3183)		72 (50-94)		1279 (643-1861)		38 (19-55)		5 (3-8)		0 (0-0)	
2002	Mozambique	2427 (1707-3188)		70 (49-91)		1264 (642-1832)		36 (18-52)		5 (3-7)		0 (0-0)	
2003	Mozambique	2448 (1722-3216)		68 (48-90)		1259 (456-1997)		35 (13-56)		5 (2-8)		0 (0-0)	
2004	Mozambique	2496 (1756-3280)		68 (48-89)		1265 (467-1999)		34 (13-54)		5 (2-8)		0 (0-0)	
2005	Mozambique	2548 (1792-3348)		67 (47-88)		1273 (478-2002)		34 (13-53)		5 (2-8)		0 (0-0)	
2006	Mozambique	2573 (1810-3381)		66 (46-87)		1268 (485-1985)		32 (12-51)		5 (2-8)		0 (0-0)	

Hib = *Haemophilus influenzae* type b; npnm = non-pneumonia, non-meningitis; HIV = deaths only in HIV-infected children; Uncertainty range provided in parentheses.

Year	Country	Hib pneumonia deaths (UR)	Hib pneumonia deaths, HIV (UR)	Hib pneumonia mortality rate (UR)	Hib pneumonia mortality rate, HIV (UR)	Hib meningitis deaths (UR)	Hib meningitis deaths, HIV (UR)	Hib meningitis mortality rate (UR)	Hib meningitis mortality rate, HIV (UR)	Hib NPNM deaths (UR)	Hib NPNM deaths, HIV (UR)	Hib NPNM mortality rate (UR)	Hib NPNM mortality rate, HIV (UR)
2007	Mozambique	2561 (1801-3365)		64 (45-84)		1244 (485-1937)		31 (12-48)		5 (2-8)		0 (0-0)	
2008	Mozambique	2537 (1784-3333)		62 (44-81)		1217 (482-1881)		30 (12-46)		5 (2-8)		0 (0-0)	
2009	Mozambique	593 (417-780)		14 (10-19)		281 (107-441)		7 (3-11)		1 (0-2)		0 (0-0)	
2010	Mozambique	264 (186-347)		6 (4-8)		124 (45-196)		3 (1-5)		1 (0-1)		0 (0-0)	
2011	Mozambique	249 (175-327)		6 (4-7)		115 (40-185)		3 (1-4)		0 (0-1)		0 (0-0)	
2012	Mozambique	241 (170-317)		5 (4-7)		111 (37-179)		2 (1-4)		0 (0-1)		0 (0-0)	
2013	Mozambique	231 (163-304)		5 (4-7)		106 (34-172)		2 (1-4)		0 (0-1)		0 (0-0)	
2014	Mozambique	22 (16-29)		0 (0-1)		10 (3-17)		0 (0-0)		0 (0-0)		0 (0-0)	
2015	Mozambique	22 (16-29)	1 (1-2)	0 (0-1)	0 (0-0)	10 (3-17)	1 (0-1)	0 (0-0)	0 (0-0)	0 (0-0)	0 (0-0)	0 (0-0)	0 (0-0)
2000	Myanmar	2236 (1573-2938)		44 (31-57)		1110 (379-1762)		22 (7-34)		5 (2-7)		0 (0-0)	
2001	Myanmar	2204 (1550-2896)		42 (30-56)		1056 (378-1658)		20 (7-32)		4 (2-7)		0 (0-0)	
2002	Myanmar	2169 (1525-2849)		41 (29-54)		1003 (379-1556)		19 (7-29)		4 (2-6)		0 (0-0)	
2003	Myanmar	2108 (1483-2770)		40 (28-52)		943 (375-1440)		18 (7-27)		4 (2-6)		0 (0-0)	
2004	Myanmar	2028 (1426-2665)		38 (27-50)		877 (323-1379)		16 (6-26)		7 (3-11)		0 (0-0)	
2005	Myanmar	1935 (1361-2543)		36 (26-48)		809 (300-1272)		15 (6-24)		6 (2-10)		0 (0-0)	
2006	Myanmar	1829 (1286-2403)		34 (24-45)		742 (277-1165)		14 (5-22)		6 (2-9)		0 (0-0)	
2007	Myanmar	1708 (1201-2244)		32 (23-43)		677 (254-1062)		13 (5-20)		5 (2-8)		0 (0-0)	
2008	Myanmar	2603 (1831-3420)		50 (35-66)		1014 (417-1545)		20 (8-30)		4 (2-6)		0 (0-0)	
2009	Myanmar	1484 (1043-1949)		29 (21-38)		571 (217-893)		11 (4-18)		5 (2-7)		0 (0-0)	
2010	Myanmar	1386 (975-1821)		28 (20-37)		527 (200-824)		11 (4-17)		4 (2-7)		0 (0-0)	
2011	Myanmar	1289 (906-1693)		27 (19-35)		484 (185-755)		10 (4-16)		4 (1-6)		0 (0-0)	
2012	Myanmar	1207 (849-1586)		26 (18-34)		447 (172-696)		9 (4-15)		4 (1-6)		0 (0-0)	
2013	Myanmar	261 (183-343)		6 (4-7)		95 (37-149)		2 (1-3)		1 (0-1)		0 (0-0)	
2014	Myanmar	117 (82-154)		3 (2-3)		43 (17-66)		1 (0-1)		0 (0-1)		0 (0-0)	
2015	Myanmar	112 (79-147)	1 (1-1)	2 (2-3)	0 (0-0)	40 (16-63)	0 (0-0)	1 (0-1)	0 (0-0)	0 (0-0)	0 (0-0)	0 (0-0)	0 (0-0)
2000	Namibia	90 (63-118)		33 (23-44)		24 (8-38)		9 (3-14)		0 (0-0)		0 (0-0)	
2001	Namibia	90 (64-119)		33 (23-44)		24 (9-38)		9 (3-14)		0 (0-0)		0 (0-0)	
2002	Namibia	90 (63-118)		33 (23-43)		23 (8-37)		9 (3-14)		0 (0-0)		0 (0-0)	
2003	Namibia	90 (63-118)		33 (23-43)		23 (8-36)		8 (3-13)		0 (0-0)		0 (0-0)	
2004	Namibia	90 (63-118)		33 (23-43)		23 (8-37)		8 (3-14)		0 (0-0)		0 (0-0)	
2005	Namibia	90 (63-118)		33 (23-43)		22 (8-36)		8 (3-13)		0 (0-0)		0 (0-0)	
2006	Namibia	90 (63-118)		33 (23-43)		22 (8-36)		8 (3-13)		0 (0-0)		0 (0-0)	
2007	Namibia	91 (64-119)		33 (23-43)		22 (8-36)		8 (3-13)		0 (0-0)		0 (0-0)	
2008	Namibia	92 (65-121)		33 (23-43)		23 (8-36)		8 (3-13)		0 (0-0)		0 (0-0)	
2009	Namibia	93 (65-122)		33 (23-43)		23 (8-36)		8 (3-13)		0 (0-0)		0 (0-0)	
2010	Namibia	15 (11-20)		5 (4-7)		4 (1-6)		1 (0-2)		0 (0-0)		0 (0-0)	
2011	Namibia	9 (6-12)		3 (2-4)		2 (1-3)		1 (0-1)		0 (0-0)		0 (0-0)	
2012	Namibia	9 (6-12)		3 (2-4)		2 (1-3)		1 (0-1)		0 (0-0)		0 (0-0)	
2013	Namibia	8 (6-11)		3 (2-3)		2 (1-3)		1 (0-1)		0 (0-0)		0 (0-0)	
2014	Namibia	9 (6-11)		3 (2-3)		2 (1-3)		1 (0-1)		0 (0-0)		0 (0-0)	
2015	Namibia	1 (1-1)	0 (0-0)	0 (0-0)	0 (0-0)	0 (0-0)	0 (0-0)	0 (0-0)	0 (0-0)	0 (0-0)	0 (0-0)	0 (0-0)	0 (0-0)
2000	Nauru	0 (0-0)		19 (14-26)		0 (0-0)		7 (4-9)		0 (0-0)		0 (0-0)	
2001	Nauru	0 (0-0)		20 (14-26)		0 (0-0)		7 (4-10)		0 (0-0)		0 (0-0)	
2002	Nauru	0 (0-0)		16 (11-21)		0 (0-0)		6 (3-8)		0 (0-0)		0 (0-0)	
2003	Nauru	0 (0-0)		19 (14-25)		0 (0-0)		8 (4-10)		0 (0-0)		0 (0-0)	
2004	Nauru	0 (0-0)		19 (13-25)		0 (0-0)		8 (4-10)		0 (0-0)		0 (0-0)	
2005	Nauru	0 (0-0)		19 (13-25)		0 (0-0)		8 (4-10)		0 (0-0)		0 (0-0)	
2006	Nauru	0 (0-0)		19 (14-25)		0 (0-0)		8 (4-11)		0 (0-0)		0 (0-0)	
2007	Nauru	0 (0-0)		16 (11-21)		0 (0-0)		7 (4-9)		0 (0-0)		0 (0-0)	
2008	Nauru	0 (0-0)		16 (12-22)		0 (0-0)		7 (4-9)		0 (0-0)		0 (0-0)	
2009	Nauru	0 (0-0)		2 (1-3)		0 (0-0)		1 (0-1)		0 (0-0)		0 (0-0)	
2010	Nauru	0 (0-0)		1 (1-1)		0 (0-0)		0 (0-0)		0 (0-0)		0 (0-0)	
2011	Nauru	0 (0-0)		1 (1-1)		0 (0-0)		0 (0-0)		0 (0-0)		0 (0-0)	
2012	Nauru	0 (0-0)		1 (1-2)		0 (0-0)		1 (0-1)		0 (0-0)		0 (0-0)	
2013	Nauru	0 (0-0)		1 (1-2)		0 (0-0)		0 (0-1)		0 (0-0)		0 (0-0)	
2014	Nauru	0 (0-0)		0 (0-0)		0 (0-0)		0 (0-0)		0 (0-0)		0 (0-0)	
2015	Nauru	0 (0-0)	0 (0-0)	0 (0-0)	0 (0-0)	0 (0-0)	0 (0-0)	0 (0-0)	0 (0-0)	0 (0-0)	0 (0-0)	0 (0-0)	0 (0-0)
2000	Nepal	1398 (983-1837)		40 (28-52)		588 (166-958)		17 (5-27)		2 (1-4)		0 (0-0)	
2001	Nepal	1308 (920-1718)		37 (26-49)		547 (158-888)		16 (4-25)		2 (1-4)		0 (0-0)	
2002	Nepal	1197 (842-1573)		34 (24-45)		499 (131-830)		14 (4-24)		4 (1-7)		0 (0-0)	
2003	Nepal	1097 (772-1442)		32 (22-41)		455 (122-755)		13 (4-22)		4 (1-6)		0 (0-0)	
2004	Nepal	997 (701-1310)		29 (20-38)		411 (114-679)		12 (3-20)		3 (1-5)		0 (0-0)	
2005	Nepal	910 (640-1195)		27 (19-35)		373 (106-613)		11 (3-18)		3 (1-5)		0 (0-0)	
2006	Nepal	829 (583-1090)		25 (18-33)		337 (99-553)		10 (3-17)		3 (1-4)		0 (0-0)	
2007	Nepal	762 (536-1001)		23 (16-31)		307 (91-502)		9 (3-15)		2 (1-4)		0 (0-0)	
2008	Nepal	696 (490-915)		22 (15-28)		278 (83-454)		9 (3-14)		2 (1-4)		0 (0-0)	
2009	Nepal	643 (452-845)		20 (14-27)		255 (77-415)		8 (2-13)		2 (1-3)		0 (0-0)	
2010	Nepal	585 (411-769)		19 (13-25)		230 (71-374)		7 (2-12)		2 (1-3)		0 (0-0)	
2011	Nepal	84 (59-110)		3 (2-4)		33 (10-53)		1 (0-2)		0 (0-0)		0 (0-0)	
2012	Nepal	39 (27-51)		1 (1-2)		15 (5-24)		1 (0-1)		0 (0-0)		0 (0-0)	
2013	Nepal	34 (24-45)		1 (1-2)		13 (4-21)		0 (0-1)		0 (0-0)		0 (0-0)	
2014	Nepal	31 (22-41)		1 (1-1)		12 (4-20)		0 (0-1)		0 (0-0)		0 (0-0)	
2015	Nepal	30 (21-40)	0 (0-0)	1 (1-1)	0 (0-0)	11 (4-19)	0 (0-0)	0 (0-1)	0 (0-0)	0 (0-0)	0 (0-0)	0 (0-0)	0 (0-0)
2000	Netherlands	0 (0-0)		0 (0-0)		1 (0-1)		0 (0-0)		0 (0-0)		0 (0-0)	
2001	Netherlands	0 (0-0)		0 (0-0)		1 (0-1)		0 (0-0)		0 (0-0)		0 (0-0)	
2002	Netherlands	0 (0-0)		0 (0-0)		0 (0-0)		0 (0-0)		0 (0-0)		0 (0-0)	
2003	Netherlands	0 (0-0)		0 (0-0)		0 (0-0)		0 (0-0)		0 (0-0)		0 (0-0)	
2004	Netherlands	0 (0-0)		0 (0-0)		0 (0-0)		0 (0-0)		0 (0-0)		0 (0-0)	
2005	Netherlands	0 (0-0)		0 (0-0)		0 (0-0)		0 (0-0)		0 (0-0)		0 (0-0)	
2006	Netherlands	0 (0-0)		0 (0-0)		0 (0-0)		0 (0-0)		0 (0-0)		0 (0-0)	
2007	Netherlands	0 (0-0)		0 (0-0)		0 (0-0)		0 (0-0)		0 (0-0)		0 (0-0)	
2008	Netherlands	0 (0-0)		0 (0-0)		0 (0-0)		0 (0-0)		0 (0-0)		0 (0-0)	
2009	Netherlands	0 (0-0)		0 (0-0)		0 (0-0)		0 (0-0)		0 (0-0)		0 (0-0)	
2010	Netherlands	0 (0-0)		0 (0-0)		0 (0-0)		0 (0-0)		0 (0-0)		0 (0-0)	
2011	Netherlands	0 (0-0)		0 (0-0)		0 (0-0)		0 (0-0)		0 (0-0)		0 (0-0)	
2012	Netherlands	0 (0-0)		0 (0-0)		0 (0-0)		0 (0-0)		0 (0-0)		0 (0-0)	
2013	Netherlands	0 (0-0)		0 (0-0)		0 (0-0)		0 (0-0)		0 (0-0)		0 (0-0)	
2014	Netherlands	0 (0-0)		0 (0-0)		0 (0-0)		0 (0-0)		0 (0-0)		0 (0-0)	

Hib = *Haemophilus influenzae* type b; npnm = non-pneumonia, non-meningitis; HIV = deaths only in HIV-infected children; Uncertainty range provided in parentheses.

Year	Country	Hib pneumonia deaths (UR)	Hib pneumonia deaths, HIV (UR)	Hib pneumonia mortality rate (UR)	Hib pneumonia mortality rate, HIV (UR)	Hib meningitis deaths (UR)	Hib meningitis deaths, HIV (UR)	Hib meningitis mortality rate (UR)	Hib meningitis mortality rate, HIV (UR)	Hib NPNM deaths (UR)	Hib NPNM deaths, HIV (UR)	Hib NPNM mortality rate (UR)	Hib NPNM mortality rate, HIV (UR)
2015	Netherlands	0 (0-0)	0 (0-0)	0 (0-0)	0 (0-0)	0 (0-0)	0 (0-0)	0 (0-0)	0 (0-0)	0 (0-0)	0 (0-0)	0 (0-0)	0 (0-0)
2000	New Zealand	0 (0-0)		0 (0-0)		1 (1-1)		0 (0-0)		0 (0-0)		0 (0-0)	
2001	New Zealand	0 (0-0)		0 (0-0)		1 (1-2)		0 (0-1)		0 (0-0)		0 (0-0)	
2002	New Zealand	0 (0-0)		0 (0-0)		0 (0-0)		0 (0-0)		0 (0-0)		0 (0-0)	
2003	New Zealand	0 (0-0)		0 (0-0)		0 (0-0)		0 (0-0)		0 (0-0)		0 (0-0)	
2004	New Zealand	0 (0-0)		0 (0-0)		0 (0-0)		0 (0-0)		0 (0-0)		0 (0-0)	
2005	New Zealand	0 (0-0)		0 (0-0)		0 (0-0)		0 (0-0)		0 (0-0)		0 (0-0)	
2006	New Zealand	0 (0-0)		0 (0-0)		0 (0-0)		0 (0-0)		0 (0-0)		0 (0-0)	
2007	New Zealand	0 (0-0)		0 (0-0)		0 (0-0)		0 (0-0)		0 (0-0)		0 (0-0)	
2008	New Zealand	0 (0-0)		0 (0-0)		0 (0-0)		0 (0-0)		0 (0-0)		0 (0-0)	
2009	New Zealand	0 (0-0)		0 (0-0)		0 (0-0)		0 (0-0)		0 (0-0)		0 (0-0)	
2010	New Zealand	0 (0-0)		0 (0-0)		0 (0-0)		0 (0-0)		0 (0-0)		0 (0-0)	
2011	New Zealand	0 (0-0)		0 (0-0)		0 (0-0)		0 (0-0)		0 (0-0)		0 (0-0)	
2012	New Zealand	0 (0-0)		0 (0-0)		0 (0-0)		0 (0-0)		0 (0-0)		0 (0-0)	
2013	New Zealand	0 (0-0)		0 (0-0)		0 (0-0)		0 (0-0)		0 (0-0)		0 (0-0)	
2014	New Zealand	0 (0-0)		0 (0-0)		0 (0-0)		0 (0-0)		0 (0-0)		0 (0-0)	
2015	New Zealand	0 (0-0)	0 (0-0)	0 (0-0)	0 (0-0)	0 (0-0)	0 (0-0)	0 (0-0)	0 (0-0)	0 (0-0)	0 (0-0)	0 (0-0)	0 (0-0)
2000	Nicaragua	17 (12-22)		3 (2-3)		5 (2-9)		1 (0-1)		0 (0-0)		0 (0-0)	
2001	Nicaragua	15 (10-19)		2 (2-3)		5 (1-8)		1 (0-1)		0 (0-0)		0 (0-0)	
2002	Nicaragua	14 (10-18)		2 (2-3)		4 (1-7)		1 (0-1)		0 (0-0)		0 (0-0)	
2003	Nicaragua	12 (9-16)		2 (1-3)		4 (1-7)		1 (0-1)		0 (0-0)		0 (0-0)	
2004	Nicaragua	1 (1-2)		0 (0-0)		0 (0-1)		0 (0-0)		0 (0-0)		0 (0-0)	
2005	Nicaragua	1 (1-2)		0 (0-0)		0 (0-1)		0 (0-0)		0 (0-0)		0 (0-0)	
2006	Nicaragua	1 (1-2)		0 (0-0)		0 (0-1)		0 (0-0)		0 (0-0)		0 (0-0)	
2007	Nicaragua	1 (1-2)		0 (0-0)		0 (0-1)		0 (0-0)		0 (0-0)		0 (0-0)	
2008	Nicaragua	1 (1-2)		0 (0-0)		0 (0-1)		0 (0-0)		0 (0-0)		0 (0-0)	
2009	Nicaragua	1 (1-2)		0 (0-0)		0 (0-1)		0 (0-0)		0 (0-0)		0 (0-0)	
2010	Nicaragua	1 (1-1)		0 (0-0)		0 (0-1)		0 (0-0)		0 (0-0)		0 (0-0)	
2011	Nicaragua	1 (1-2)		0 (0-0)		0 (0-1)		0 (0-0)		0 (0-0)		0 (0-0)	
2012	Nicaragua	1 (1-2)		0 (0-0)		0 (0-1)		0 (0-0)		0 (0-0)		0 (0-0)	
2013	Nicaragua	1 (1-2)		0 (0-0)		0 (0-1)		0 (0-0)		0 (0-0)		0 (0-0)	
2014	Nicaragua	1 (1-1)		0 (0-0)		0 (0-1)		0 (0-0)		0 (0-0)		0 (0-0)	
2015	Nicaragua	1 (1-1)	0 (0-0)	0 (0-0)	0 (0-0)	0 (0-1)	0 (0-0)	0 (0-0)	0 (0-0)	0 (0-0)	0 (0-0)	0 (0-0)	0 (0-0)
2000	Niger	3355 (2360-4408)		148 (104-194)		1616 (724-2428)		71 (32-107)		7 (3-10)		0 (0-0)	
2001	Niger	3433 (2415-4511)		144 (101-189)		1655 (753-2475)		69 (32-104)		7 (3-10)		0 (0-0)	
2002	Niger	3448 (2425-4530)		139 (98-183)		1664 (768-2477)		67 (31-100)		7 (3-10)		0 (0-0)	
2003	Niger	3418 (2404-4491)		133 (94-175)		1648 (772-2443)		64 (30-95)		7 (3-10)		0 (0-0)	
2004	Niger	3364 (2366-4420)		127 (89-166)		1621 (772-2392)		61 (29-90)		7 (3-10)		0 (0-0)	
2005	Niger	3298 (2319-4333)		120 (84-157)		1587 (768-2330)		58 (28-85)		6 (3-9)		0 (0-0)	
2006	Niger	3231 (2272-4245)		113 (79-148)		1553 (764-2268)		54 (27-79)		6 (3-9)		0 (0-0)	
2007	Niger	3247 (2284-4266)		109 (77-143)		1561 (771-2276)		52 (26-76)		6 (3-9)		0 (0-0)	
2008	Niger	3019 (2123-3967)		98 (69-128)		1450 (498-2323)		47 (16-75)		6 (2-9)		0 (0-0)	
2009	Niger	804 (565-1056)		25 (18-33)		386 (134-617)		12 (4-19)		2 (1-3)		0 (0-0)	
2010	Niger	339 (239-446)		10 (7-13)		162 (57-259)		5 (2-8)		1 (0-1)		0 (0-0)	
2011	Niger	310 (218-408)		9 (6-12)		148 (52-235)		4 (1-7)		1 (0-1)		0 (0-0)	
2012	Niger	330 (232-433)		9 (6-12)		156 (55-248)		4 (2-7)		1 (0-1)		0 (0-0)	
2013	Niger	344 (242-452)		9 (6-12)		162 (58-257)		4 (2-7)		1 (0-1)		0 (0-0)	
2014	Niger	29 (20-38)		1 (1-1)		14 (5-22)		0 (0-1)		0 (0-0)		0 (0-0)	
2015	Niger	361 (254-474)	1 (1-1)	9 (6-12)	0 (0-0)	168 (62-266)	1 (0-1)	4 (2-7)	0 (0-0)	1 (0-1)	0 (0-0)	0 (0-0)	0 (0-0)
2000	Nigeria	19556 (13754-25696)		94 (66-124)		6702 (2982-10081)		32 (14-49)		27 (12-41)		0 (0-0)	
2001	Nigeria	19914 (14006-26165)		93 (65-122)		6747 (3034-10114)		32 (14-47)		28 (12-41)		0 (0-0)	
2002	Nigeria	20165 (14183-26496)		91 (64-120)		6710 (3051-10026)		30 (14-45)		27 (12-41)		0 (0-0)	
2003	Nigeria	19997 (14065-26275)		88 (62-116)		6479 (2979-9650)		29 (13-43)		26 (12-39)		0 (0-0)	
2004	Nigeria	20168 (14185-26500)		87 (61-114)		6338 (2948-9409)		27 (13-40)		26 (12-38)		0 (0-0)	
2005	Nigeria	20206 (14211-26549)		84 (59-111)		6193 (2914-9163)		26 (12-38)		25 (12-37)		0 (0-0)	
2006	Nigeria	20689 (14551-27184)		84 (59-110)		6203 (2953-9143)		25 (12-37)		25 (12-37)		0 (0-0)	
2007	Nigeria	20812 (14637-27345)		82 (58-108)		6145 (2007-9935)		24 (8-39)		25 (8-40)		0 (0-0)	
2008	Nigeria	20680 (14545-27172)		80 (56-105)		6042 (2011-9717)		23 (8-37)		25 (8-40)		0 (0-0)	
2009	Nigeria	20937 (14725-27509)		79 (55-104)		6057 (1987-9777)		23 (7-37)		25 (8-40)		0 (0-0)	
2010	Nigeria	21510 (15129-28263)		79 (56-104)		6155 (1989-9966)		23 (7-37)		25 (8-41)		0 (0-0)	
2011	Nigeria	21551 (15157-28316)		77 (54-101)		6110 (1945-9920)		22 (7-36)		25 (8-40)		0 (0-0)	
2012	Nigeria	20087 (14128-26393)		70 (49-92)		5659 (1766-9222)		20 (6-32)		23 (7-38)		0 (0-0)	
2013	Nigeria	3339 (2349-4388)		11 (8-15)		939 (287-1536)		3 (1-5)		4 (1-6)		0 (0-0)	
2014	Nigeria	3139 (2208-4125)		10 (7-14)		884 (265-1451)		3 (1-5)		4 (1-6)		0 (0-0)	
2015	Nigeria	2837 (1996-3728)	101 (71-132)	9 (7-12)	0 (0-0)	800 (236-1317)	28 (8-47)	3 (1-4)	0 (0-0)	3 (1-5)	0 (0-0)	0 (0-0)	0 (0-0)
2000	Niue	0 (0-0)		5 (4-7)		0 (0-0)		3 (2-5)		0 (0-0)		0 (0-0)	
2001	Niue	0 (0-0)		2 (1-3)		0 (0-0)		1 (1-2)		0 (0-0)		0 (0-0)	
2002	Niue	0 (0-0)		2 (1-3)		0 (0-0)		1 (1-2)		0 (0-0)		0 (0-0)	
2003	Niue	0 (0-0)		2 (1-3)		0 (0-0)		1 (1-2)		0 (0-0)		0 (0-0)	
2004	Niue	0 (0-0)		0 (0-0)		0 (0-0)		0 (0-0)		0 (0-0)		0 (0-0)	
2005	Niue	0 (0-0)		0 (0-0)		0 (0-0)		0 (0-0)		0 (0-0)		0 (0-0)	
2006	Niue	0 (0-0)		0 (0-0)		0 (0-0)		0 (0-0)		0 (0-0)		0 (0-0)	
2007	Niue	0 (0-0)		0 (0-0)		0 (0-0)		0 (0-0)		0 (0-0)		0 (0-0)	
2008	Niue	0 (0-0)		0 (0-0)		0 (0-0)		0 (0-0)		0 (0-0)		0 (0-0)	
2009	Niue	0 (0-0)		0 (0-1)		0 (0-0)		0 (0-0)		0 (0-0)		0 (0-0)	
2010	Niue	0 (0-0)		0 (0-1)		0 (0-0)		0 (0-0)		0 (0-0)		0 (0-0)	
2011	Niue	0 (0-0)		0 (0-1)		0 (0-0)		0 (0-0)		0 (0-0)		0 (0-0)	
2012	Niue	0 (0-0)		0 (0-1)		0 (0-0)		0 (0-0)		0 (0-0)		0 (0-0)	
2013	Niue	0 (0-0)		0 (0-1)		0 (0-0)		0 (0-0)		0 (0-0)		0 (0-0)	
2014	Niue	0 (0-0)		0 (0-1)		0 (0-0)		0 (0-0)		0 (0-0)		0 (0-0)	
2015	Niue	0 (0-0)	0 (0-0)	0 (0-1)	0 (0-0)	0 (0-0)	0 (0-0)	0 (0-0)	0 (0-0)	0 (0-0)	0 (0-0)	0 (0-0)	0 (0-0)
2000	Norway	0 (0-0)		0 (0-0)		0 (0-1)		0 (0-0)		0 (0-0)		0 (0-0)	
2001	Norway	0 (0-0)		0 (0-0)		0 (0-0)		0 (0-0)		0 (0-0)		0 (0-0)	
2002	Norway	0 (0-0)		0 (0-0)		0 (0-0)		0 (0-0)		0 (0-0)		0 (0-0)	
2003	Norway	0 (0-0)		0 (0-0)		0 (0-0)		0 (0-0)		0 (0-0)		0 (0-0)	
2004	Norway	0 (0-0)		0 (0-0)		0 (0-0)		0 (0-0)		0 (0-0)		0 (0-0)	
2005	Norway	0 (0-0)		0 (0-0)		0 (0-0)		0 (0-0)		0 (0-0)		0 (0-0)	
2006	Norway	0 (0-0)		0 (0-0)		0 (0-0)		0 (0-0)		0 (0-0)		0 (0-0)	

Hib = *Haemophilus influenzae* type b; npnm = non-pneumonia, non-meningitis; HIV = deaths only in HIV-infected children; Uncertainty range provided in parentheses.

Year	Country	Hib pneumonia deaths (UR)	Hib pneumonia deaths, HIV (UR)	Hib pneumonia mortality rate (UR)	Hib pneumonia mortality rate, HIV (UR)	Hib meningitis deaths (UR)	Hib meningitis deaths, HIV (UR)	Hib meningitis mortality rate (UR)	Hib meningitis mortality rate, HIV (UR)	Hib NPNM deaths (UR)	Hib NPNM deaths, HIV (UR)	Hib NPNM mortality rate (UR)	Hib NPNM mortality rate, HIV (UR)
2007	Norway	0 (0-0)		0 (0-0)		0 (0-0)		0 (0-0)		0 (0-0)		0 (0-0)	
2008	Norway	0 (0-0)		0 (0-0)		0 (0-0)		0 (0-0)		0 (0-0)		0 (0-0)	
2009	Norway	0 (0-0)		0 (0-0)		0 (0-0)		0 (0-0)		0 (0-0)		0 (0-0)	
2010	Norway	0 (0-0)		0 (0-0)		0 (0-0)		0 (0-0)		0 (0-0)		0 (0-0)	
2011	Norway	0 (0-0)		0 (0-0)		0 (0-0)		0 (0-0)		0 (0-0)		0 (0-0)	
2012	Norway	0 (0-0)		0 (0-0)		0 (0-0)		0 (0-0)		0 (0-0)		0 (0-0)	
2013	Norway	0 (0-0)		0 (0-0)		0 (0-0)		0 (0-0)		0 (0-0)		0 (0-0)	
2014	Norway	0 (0-0)		0 (0-0)		0 (0-0)		0 (0-0)		0 (0-0)		0 (0-0)	
2015	Norway	0 (0-0)	0 (0-0)	0 (0-0)	0 (0-0)	0 (0-0)	0 (0-0)	0 (0-0)	0 (0-0)	0 (0-0)	0 (0-0)	0 (0-0)	0 (0-0)
2000	Oman	18 (13-24)		7 (5-9)		2 (1-3)		1 (0-1)		0 (0-0)		0 (0-0)	
2001	Oman	15 (11-20)		6 (4-8)		2 (1-3)		1 (0-1)		0 (0-0)		0 (0-0)	
2002	Oman	2 (2-3)		1 (1-1)		0 (0-1)		0 (0-0)		0 (0-0)		0 (0-0)	
2003	Oman	1 (1-1)		0 (0-0)		0 (0-0)		0 (0-0)		0 (0-0)		0 (0-0)	
2004	Oman	1 (1-1)		0 (0-0)		0 (0-0)		0 (0-0)		0 (0-0)		0 (0-0)	
2005	Oman	1 (1-1)		0 (0-0)		0 (0-0)		0 (0-0)		0 (0-0)		0 (0-0)	
2006	Oman	1 (1-1)		0 (0-0)		0 (0-0)		0 (0-0)		0 (0-0)		0 (0-0)	
2007	Oman	0 (0-0)		0 (0-0)		0 (0-0)		0 (0-0)		0 (0-0)		0 (0-0)	
2008	Oman	0 (0-0)		0 (0-0)		0 (0-0)		0 (0-0)		0 (0-0)		0 (0-0)	
2009	Oman	0 (0-0)		0 (0-0)		0 (0-0)		0 (0-0)		0 (0-0)		0 (0-0)	
2010	Oman	0 (0-0)		0 (0-0)		0 (0-0)		0 (0-0)		0 (0-0)		0 (0-0)	
2011	Oman	0 (0-0)		0 (0-0)		0 (0-0)		0 (0-0)		0 (0-0)		0 (0-0)	
2012	Oman	0 (0-0)		0 (0-0)		0 (0-0)		0 (0-0)		0 (0-0)		0 (0-0)	
2013	Oman	0 (0-0)		0 (0-0)		0 (0-0)		0 (0-0)		0 (0-0)		0 (0-0)	
2014	Oman	0 (0-0)		0 (0-0)		0 (0-0)		0 (0-0)		0 (0-0)		0 (0-0)	
2015	Oman	0 (0-0)	0 (0-0)	0 (0-0)	0 (0-0)	0 (0-0)	0 (0-0)	0 (0-0)	0 (0-0)	0 (0-0)	0 (0-0)	0 (0-0)	0 (0-0)
2000	Pakistan	11166 (7853-14671)		56 (40-74)		3622 (1463-5551)		18 (7-28)		15 (6-23)		0 (0-0)	
2001	Pakistan	11005 (7740-14460)		55 (39-73)		3552 (1440-5438)		18 (7-27)		14 (6-22)		0 (0-0)	
2002	Pakistan	10741 (7554-14112)		54 (38-71)		3438 (1399-5259)		17 (7-27)		14 (6-21)		0 (0-0)	
2003	Pakistan	10575 (7437-13894)		54 (38-71)		3349 (1368-5119)		17 (7-26)		14 (6-21)		0 (0-0)	
2004	Pakistan	10490 (7378-13784)		53 (38-70)		3287 (1347-5020)		17 (7-26)		13 (5-20)		0 (0-0)	
2005	Pakistan	10561 (7428-13876)		54 (38-70)		3274 (1346-4996)		17 (7-25)		13 (5-20)		0 (0-0)	
2006	Pakistan	10635 (7480-13973)		54 (38-70)		3264 (1346-4977)		16 (7-25)		13 (5-20)		0 (0-0)	
2007	Pakistan	10774 (7578-14157)		53 (37-70)		3278 (1351-4996)		16 (7-25)		13 (6-20)		0 (0-0)	
2008	Pakistan	10924 (7683-14354)		52 (37-69)		3303 (1349-5046)		16 (6-24)		13 (5-21)		0 (0-0)	
2009	Pakistan	2383 (1676-3131)		11 (8-15)		717 (290-1098)		3 (1-5)		3 (1-4)		0 (0-0)	
2010	Pakistan	1053 (740-1383)		5 (3-6)		316 (127-484)		1 (1-2)		1 (1-2)		0 (0-0)	
2011	Pakistan	1203 (846-1580)		5 (4-7)		359 (143-552)		2 (1-2)		1 (1-2)		0 (0-0)	
2012	Pakistan	1220 (858-1603)		5 (4-7)		363 (143-559)		2 (1-2)		1 (1-2)		0 (0-0)	
2013	Pakistan	1195 (840-1570)		5 (4-7)		354 (140-546)		1 (1-2)		1 (1-2)		0 (0-0)	
2014	Pakistan	103 (72-135)		0 (0-1)		30 (12-47)		0 (0-0)		0 (0-0)		0 (0-0)	
2015	Pakistan	100 (71-132)	0 (0-0)	0 (0-1)	0 (0-0)	30 (12-46)	0 (0-0)	0 (0-0)	0 (0-0)	0 (0-0)	0 (0-0)	0 (0-0)	0 (0-0)
2000	Palau	0 (0-0)		1 (0-1)		0 (0-0)		1 (0-1)		0 (0-0)		0 (0-0)	
2001	Palau	0 (0-0)		1 (0-1)		0 (0-0)		1 (0-1)		0 (0-0)		0 (0-0)	
2002	Palau	0 (0-0)		1 (1-1)		0 (0-0)		1 (0-1)		0 (0-0)		0 (0-0)	
2003	Palau	0 (0-0)		0 (0-0)		0 (0-0)		0 (0-0)		0 (0-0)		0 (0-0)	
2004	Palau	0 (0-0)		0 (0-0)		0 (0-0)		0 (0-0)		0 (0-0)		0 (0-0)	
2005	Palau	0 (0-0)		0 (0-0)		0 (0-0)		0 (0-0)		0 (0-0)		0 (0-0)	
2006	Palau	0 (0-0)		0 (0-0)		0 (0-0)		0 (0-0)		0 (0-0)		0 (0-0)	
2007	Palau	0 (0-0)		0 (0-0)		0 (0-0)		0 (0-0)		0 (0-0)		0 (0-0)	
2008	Palau	0 (0-0)		0 (0-0)		0 (0-0)		0 (0-0)		0 (0-0)		0 (0-0)	
2009	Palau	0 (0-0)		1 (0-1)		0 (0-0)		1 (0-1)		0 (0-0)		0 (0-0)	
2010	Palau	0 (0-0)		0 (0-0)		0 (0-0)		0 (0-0)		0 (0-0)		0 (0-0)	
2011	Palau	0 (0-0)		0 (0-0)		0 (0-0)		0 (0-0)		0 (0-0)		0 (0-0)	
2012	Palau	0 (0-0)		0 (0-0)		0 (0-0)		0 (0-0)		0 (0-0)		0 (0-0)	
2013	Palau	0 (0-0)		0 (0-0)		0 (0-0)		0 (0-0)		0 (0-0)		0 (0-0)	
2014	Palau	0 (0-0)		0 (0-0)		0 (0-0)		0 (0-0)		0 (0-0)		0 (0-0)	
2015	Palau	0 (0-0)	0 (0-0)	0 (0-0)	0 (0-0)	0 (0-0)	0 (0-0)	0 (0-0)	0 (0-0)	0 (0-0)	0 (0-0)	0 (0-0)	0 (0-0)
2000	Panama	19 (14-26)		6 (4-8)		10 (2-17)		3 (0-5)		0 (0-0)		0 (0-0)	
2001	Panama	3 (2-4)		1 (1-1)		2 (0-4)		1 (0-1)		0 (0-0)		0 (0-0)	
2002	Panama	1 (1-2)		0 (0-0)		1 (0-1)		0 (0-0)		0 (0-0)		0 (0-0)	
2003	Panama	2 (1-2)		0 (0-1)		0 (0-1)		0 (0-0)		0 (0-0)		0 (0-0)	
2004	Panama	2 (1-2)		0 (0-1)		1 (0-2)		0 (0-0)		0 (0-0)		0 (0-0)	
2005	Panama	2 (2-3)		1 (0-1)		1 (0-1)		0 (0-0)		0 (0-0)		0 (0-0)	
2006	Panama	0 (0-0)		0 (0-0)		0 (0-0)		0 (0-0)		0 (0-0)		0 (0-0)	
2007	Panama	0 (0-0)		0 (0-0)		0 (0-0)		0 (0-0)		0 (0-0)		0 (0-0)	
2008	Panama	0 (0-0)		0 (0-0)		0 (0-0)		0 (0-0)		0 (0-0)		0 (0-0)	
2009	Panama	0 (0-0)		0 (0-0)		0 (0-0)		0 (0-0)		0 (0-0)		0 (0-0)	
2010	Panama	0 (0-1)		0 (0-0)		0 (0-0)		0 (0-0)		0 (0-0)		0 (0-0)	
2011	Panama	0 (0-1)		0 (0-0)		0 (0-0)		0 (0-0)		0 (0-0)		0 (0-0)	
2012	Panama	0 (0-0)		0 (0-0)		0 (0-0)		0 (0-0)		0 (0-0)		0 (0-0)	
2013	Panama	0 (0-0)		0 (0-0)		0 (0-0)		0 (0-0)		0 (0-0)		0 (0-0)	
2014	Panama	0 (0-1)		0 (0-0)		0 (0-0)		0 (0-0)		0 (0-0)		0 (0-0)	
2015	Panama	0 (0-1)	0 (0-0)	0 (0-0)	0 (0-0)	0 (0-0)	0 (0-0)	0 (0-0)	0 (0-0)	0 (0-0)	0 (0-0)	0 (0-0)	0 (0-0)
2000	Papua New Guinea	425 (299-558)		52 (36-68)		232 (118-299)		28 (14-36)		1 (0-1)		0 (0-0)	
2001	Papua New Guinea	432 (304-568)		51 (36-67)		238 (121-307)		28 (14-36)		1 (0-1)		0 (0-0)	
2002	Papua New Guinea	435 (306-571)		51 (36-67)		241 (123-310)		28 (14-36)		1 (1-1)		0 (0-0)	
2003	Papua New Guinea	435 (306-571)		50 (35-66)		242 (124-311)		28 (14-36)		1 (1-1)		0 (0-0)	
2004	Papua New Guinea	436 (307-573)		50 (35-65)		243 (124-313)		28 (14-36)		1 (1-1)		0 (0-0)	
2005	Papua New Guinea	438 (308-575)		49 (35-65)		243 (116-316)		27 (13-36)		2 (1-3)		0 (0-0)	
2006	Papua New Guinea	438 (308-575)		49 (34-64)		241 (116-314)		27 (13-35)		2 (1-3)		0 (0-0)	
2007	Papua New Guinea	439 (309-577)		48 (34-64)		240 (115-312)		26 (13-34)		2 (1-2)		0 (0-0)	
2008	Papua New Guinea	437 (307-574)		47 (33-62)		235 (114-306)		26 (12-33)		2 (1-2)		0 (0-0)	
2009	Papua New Guinea	168 (118-220)		18 (13-24)		89 (43-116)		10 (5-12)		1 (0-1)		0 (0-0)	
2010	Papua New Guinea	60 (42-78)		6 (4-8)		31 (15-41)		3 (2-4)		0 (0-0)		0 (0-0)	
2011	Papua New Guinea	55 (39-73)		6 (4-8)		29 (14-37)		3 (1-4)		0 (0-0)		0 (0-0)	
2012	Papua New Guinea	53 (37-69)		5 (4-7)		27 (13-35)		3 (1-4)		0 (0-0)		0 (0-0)	
2013	Papua New Guinea	48 (34-64)		5 (4-7)		25 (12-32)		3 (1-3)		0 (0-0)		0 (0-0)	
2014	Papua New Guinea	51 (36-67)		5 (4-7)		26 (13-34)		3 (1-3)		0 (0-0)		0 (0-0)	

Hib = *Haemophilus influenzae* type b; npnm = non-pneumonia, non-meningitis; HIV = deaths only in HIV-infected children; Uncertainty range provided in parentheses.

Year	Country	Hib pneumonia deaths (UR)	Hib pneumonia deaths, HIV (UR)	Hib pneumonia mortality rate (UR)	Hib pneumonia mortality rate, HIV (UR)	Hib meningitis deaths (UR)	Hib meningitis deaths, HIV (UR)	Hib meningitis mortality rate (UR)	Hib meningitis mortality rate, HIV (UR)	Hib NPNM deaths (UR)	Hib NPNM deaths, HIV (UR)	Hib NPNM mortality rate (UR)	Hib NPNM mortality rate, HIV (UR)
2015	Papua New Guinea	49 (35-65)	0 (0-1)	5 (4-7)	0 (0-0)	25 (12-32)	0 (0-0)	3 (1-3)	0 (0-0)	0 (0-0)	0 (0-0)	0 (0-0)	0 (0-0)
2000	Paraguay	104 (73-136)		15 (11-20)		23 (11-36)		3 (2-5)		0 (0-0)		0 (0-0)	
2001	Paraguay	99 (69-129)		14 (10-19)		21 (11-34)		3 (2-5)		0 (0-0)		0 (0-0)	
2002	Paraguay	92 (65-121)		13 (9-18)		20 (10-32)		3 (1-5)		0 (0-0)		0 (0-0)	
2003	Paraguay	15 (11-20)		2 (2-3)		5 (3-9)		1 (0-1)		0 (0-0)		0 (0-0)	
2004	Paraguay	7 (5-9)		1 (1-1)		2 (1-3)		0 (0-0)		0 (0-0)		0 (0-0)	
2005	Paraguay	7 (5-9)		1 (1-1)		2 (1-3)		0 (0-0)		0 (0-0)		0 (0-0)	
2006	Paraguay	7 (5-9)		1 (1-1)		2 (1-3)		0 (0-0)		0 (0-0)		0 (0-0)	
2007	Paraguay	7 (5-9)		1 (1-1)		2 (1-3)		0 (0-0)		0 (0-0)		0 (0-0)	
2008	Paraguay	1 (1-1)		0 (0-0)		0 (0-0)		0 (0-0)		0 (0-0)		0 (0-0)	
2009	Paraguay	1 (1-1)		0 (0-0)		0 (0-0)		0 (0-0)		0 (0-0)		0 (0-0)	
2010	Paraguay	1 (1-1)		0 (0-0)		0 (0-0)		0 (0-0)		0 (0-0)		0 (0-0)	
2011	Paraguay	1 (0-1)		0 (0-0)		0 (0-0)		0 (0-0)		0 (0-0)		0 (0-0)	
2012	Paraguay	1 (1-1)		0 (0-0)		0 (0-0)		0 (0-0)		0 (0-0)		0 (0-0)	
2013	Paraguay	1 (1-1)		0 (0-0)		0 (0-0)		0 (0-0)		0 (0-0)		0 (0-0)	
2014	Paraguay	1 (0-1)		0 (0-0)		0 (0-0)		0 (0-0)		0 (0-0)		0 (0-0)	
2015	Paraguay	1 (0-1)	0 (0-0)	0 (0-0)	0 (0-0)	0 (0-0)	0 (0-0)	0 (0-0)	0 (0-0)	0 (0-0)	0 (0-0)	0 (0-0)	0 (0-0)
2000	Peru	114 (80-149)		4 (3-5)		56 (17-94)		2 (1-3)		0 (0-1)		0 (0-0)	
2001	Peru	132 (93-173)		4 (3-6)		63 (19-104)		2 (1-4)		1 (0-1)		0 (0-0)	
2002	Peru	91 (64-119)		3 (2-4)		44 (14-72)		2 (0-2)		0 (0-1)		0 (0-0)	
2003	Peru	80 (56-105)		3 (2-4)		38 (12-63)		1 (0-2)		0 (0-1)		0 (0-0)	
2004	Peru	4 (3-6)		0 (0-0)		2 (0-4)		0 (0-0)		0 (0-0)		0 (0-0)	
2005	Peru	4 (3-5)		0 (0-0)		2 (0-4)		0 (0-0)		0 (0-0)		0 (0-0)	
2006	Peru	4 (3-5)		0 (0-0)		2 (0-3)		0 (0-0)		0 (0-0)		0 (0-0)	
2007	Peru	3 (2-4)		0 (0-0)		2 (0-3)		0 (0-0)		0 (0-0)		0 (0-0)	
2008	Peru	3 (2-4)		0 (0-0)		2 (0-3)		0 (0-0)		0 (0-0)		0 (0-0)	
2009	Peru	3 (2-4)		0 (0-0)		1 (0-2)		0 (0-0)		0 (0-0)		0 (0-0)	
2010	Peru	3 (2-4)		0 (0-0)		1 (0-2)		0 (0-0)		0 (0-0)		0 (0-0)	
2011	Peru	3 (2-3)		0 (0-0)		1 (0-2)		0 (0-0)		0 (0-0)		0 (0-0)	
2012	Peru	3 (2-3)		0 (0-0)		1 (0-2)		0 (0-0)		0 (0-0)		0 (0-0)	
2013	Peru	3 (2-3)		0 (0-0)		1 (0-2)		0 (0-0)		0 (0-0)		0 (0-0)	
2014	Peru	3 (2-3)		0 (0-0)		1 (0-2)		0 (0-0)		0 (0-0)		0 (0-0)	
2015	Peru	2 (2-3)	0 (0-0)	0 (0-0)	0 (0-0)	1 (0-2)	0 (0-0)	0 (0-0)	0 (0-0)	0 (0-0)	0 (0-0)	0 (0-0)	0 (0-0)
2000	Philippines	2475 (1741-3252)		23 (16-31)		735 (247-1176)		7 (2-11)		6 (2-9)		0 (0-0)	
2001	Philippines	2444 (1719-3212)		23 (16-30)		721 (240-1156)		7 (2-11)		6 (2-9)		0 (0-0)	
2002	Philippines	2406 (1692-3161)		22 (16-29)		704 (232-1131)		6 (2-10)		6 (2-9)		0 (0-0)	
2003	Philippines	2378 (1672-3124)		21 (15-28)		690 (226-1112)		6 (2-10)		6 (2-9)		0 (0-0)	
2004	Philippines	2338 (1644-3071)		21 (15-27)		673 (218-1086)		6 (2-10)		5 (2-9)		0 (0-0)	
2005	Philippines	2229 (1567-2928)		20 (14-26)		636 (204-1027)		6 (2-9)		5 (2-8)		0 (0-0)	
2006	Philippines	2213 (1556-2907)		20 (14-26)		624 (198-1010)		6 (2-9)		5 (2-8)		0 (0-0)	
2007	Philippines	2145 (1508-2818)		19 (14-26)		598 (188-969)		5 (2-9)		5 (2-8)		0 (0-0)	
2008	Philippines	2081 (1464-2735)		19 (14-25)		573 (179-931)		5 (2-9)		5 (1-7)		0 (0-0)	
2009	Philippines	2018 (1419-2652)		19 (13-25)		548 (175-886)		5 (2-8)		4 (1-7)		0 (0-0)	
2010	Philippines	1950 (1371-2562)		18 (13-24)		523 (172-842)		5 (2-8)		4 (1-7)		0 (0-0)	
2011	Philippines	1535 (1080-2017)		14 (10-19)		408 (138-655)		4 (1-6)		3 (1-5)		0 (0-0)	
2012	Philippines	1096 (771-1441)		10 (7-13)		289 (101-463)		3 (1-4)		2 (1-4)		0 (0-0)	
2013	Philippines	140 (98-184)		1 (1-2)		37 (5-62)		0 (0-1)		0 (0-0)		0 (0-0)	
2014	Philippines	186 (131-244)		2 (1-2)		48 (7-82)		0 (0-1)		0 (0-1)		0 (0-0)	
2015	Philippines	241 (169-316)	0 (0-0)	2 (2-3)	0 (0-0)	62 (10-104)	0 (0-0)	1 (0-1)	0 (0-0)	0 (0-1)	0 (0-0)	0 (0-0)	0 (0-0)
2000	Poland	24 (17-31)		1 (1-2)		8 (4-15)		0 (0-1)		0 (0-0)		0 (0-0)	
2001	Poland	19 (14-26)		1 (1-1)		7 (3-13)		0 (0-1)		0 (0-0)		0 (0-0)	
2002	Poland	15 (10-19)		1 (1-1)		5 (2-9)		0 (0-1)		0 (0-0)		0 (0-0)	
2003	Poland	17 (12-22)		1 (1-1)		6 (2-10)		0 (0-1)		0 (0-0)		0 (0-0)	
2004	Poland	18 (13-24)		1 (1-1)		5 (2-9)		0 (0-1)		0 (0-0)		0 (0-0)	
2005	Poland	16 (11-21)		1 (1-1)		4 (2-8)		0 (0-0)		0 (0-0)		0 (0-0)	
2006	Poland	8 (5-10)		0 (0-1)		3 (1-6)		0 (0-0)		0 (0-0)		0 (0-0)	
2007	Poland	2 (1-3)		0 (0-0)		1 (0-1)		0 (0-0)		0 (0-0)		0 (0-0)	
2008	Poland	1 (1-2)		0 (0-0)		1 (0-1)		0 (0-0)		0 (0-0)		0 (0-0)	
2009	Poland	1 (1-2)		0 (0-0)		0 (0-1)		0 (0-0)		0 (0-0)		0 (0-0)	
2010	Poland	0 (0-0)		0 (0-0)		0 (0-0)		0 (0-0)		0 (0-0)		0 (0-0)	
2011	Poland	0 (0-0)		0 (0-0)		0 (0-0)		0 (0-0)		0 (0-0)		0 (0-0)	
2012	Poland	0 (0-0)		0 (0-0)		0 (0-0)		0 (0-0)		0 (0-0)		0 (0-0)	
2013	Poland	0 (0-0)		0 (0-0)		0 (0-0)		0 (0-0)		0 (0-0)		0 (0-0)	
2014	Poland	0 (0-0)		0 (0-0)		0 (0-0)		0 (0-0)		0 (0-0)		0 (0-0)	
2015	Poland	0 (0-0)	0 (0-0)	0 (0-0)	0 (0-0)	0 (0-0)	0 (0-0)	0 (0-0)	0 (0-0)	0 (0-0)	0 (0-0)	0 (0-0)	0 (0-0)
2000	Portugal	0 (0-1)		0 (0-0)		1 (0-1)		0 (0-0)		0 (0-0)		0 (0-0)	
2001	Portugal	0 (0-1)		0 (0-0)		0 (0-0)		0 (0-0)		0 (0-0)		0 (0-0)	
2002	Portugal	0 (0-1)		0 (0-0)		1 (0-1)		0 (0-0)		0 (0-0)		0 (0-0)	
2003	Portugal	0 (0-0)		0 (0-0)		0 (0-0)		0 (0-0)		0 (0-0)		0 (0-0)	
2004	Portugal	0 (0-0)		0 (0-0)		0 (0-0)		0 (0-0)		0 (0-0)		0 (0-0)	
2005	Portugal	0 (0-0)		0 (0-0)		0 (0-0)		0 (0-0)		0 (0-0)		0 (0-0)	
2006	Portugal	0 (0-0)		0 (0-0)		0 (0-0)		0 (0-0)		0 (0-0)		0 (0-0)	
2007	Portugal	0 (0-0)		0 (0-0)		0 (0-0)		0 (0-0)		0 (0-0)		0 (0-0)	
2008	Portugal	0 (0-0)		0 (0-0)		0 (0-0)		0 (0-0)		0 (0-0)		0 (0-0)	
2009	Portugal	0 (0-0)		0 (0-0)		0 (0-0)		0 (0-0)		0 (0-0)		0 (0-0)	
2010	Portugal	0 (0-0)		0 (0-0)		0 (0-0)		0 (0-0)		0 (0-0)		0 (0-0)	
2011	Portugal	0 (0-0)		0 (0-0)		0 (0-0)		0 (0-0)		0 (0-0)		0 (0-0)	
2012	Portugal	0 (0-0)		0 (0-0)		0 (0-0)		0 (0-0)		0 (0-0)		0 (0-0)	
2013	Portugal	0 (0-0)		0 (0-0)		0 (0-0)		0 (0-0)		0 (0-0)		0 (0-0)	
2014	Portugal	0 (0-0)		0 (0-0)		0 (0-0)		0 (0-0)		0 (0-0)		0 (0-0)	
2015	Portugal	0 (0-0)	0 (0-0)	0 (0-0)	0 (0-0)	0 (0-0)	0 (0-0)	0 (0-0)	0 (0-0)	0 (0-0)	0 (0-0)	0 (0-0)	0 (0-0)
2000	Qatar	0 (0-0)		0 (0-0)		0 (0-0)		0 (0-0)		0 (0-0)		0 (0-0)	
2001	Qatar	0 (0-0)		0 (0-0)		0 (0-0)		0 (0-0)		0 (0-0)		0 (0-0)	
2002	Qatar	0 (0-0)		0 (0-0)		0 (0-0)		0 (0-0)		0 (0-0)		0 (0-0)	
2003	Qatar	0 (0-0)		0 (0-0)		0 (0-0)		0 (0-0)		0 (0-0)		0 (0-0)	
2004	Qatar	0 (0-0)		0 (0-0)		0 (0-0)		0 (0-0)		0 (0-0)		0 (0-0)	
2005	Qatar	0 (0-0)		0 (0-0)		0 (0-0)		0 (0-0)		0 (0-0)		0 (0-0)	
2006	Qatar	0 (0-0)		0 (0-0)		0 (0-0)		0 (0-0)		0 (0-0)		0 (0-0)	

Hib = *Haemophilus influenzae* type b; npnm = non-pneumonia, non-meningitis; HIV = deaths only in HIV-infected children; Uncertainty range provided in parentheses.

Year	Country	Hib pneumonia deaths (UR)	Hib pneumonia deaths, HIV (UR)	Hib pneumonia mortality rate (UR)	Hib pneumonia mortality rate, HIV (UR)	Hib meningitis deaths (UR)	Hib meningitis deaths, HIV (UR)	Hib meningitis mortality rate (UR)	Hib meningitis mortality rate, HIV (UR)	Hib NPNM deaths (UR)	Hib NPNM deaths, HIV (UR)	Hib NPNM mortality rate (UR)	Hib NPNM mortality rate, HIV (UR)
2007	Qatar	0 (0-0)		0 (0-0)		0 (0-0)		0 (0-0)		0 (0-0)		0 (0-0)	
2008	Qatar	0 (0-0)		0 (0-0)		0 (0-0)		0 (0-0)		0 (0-0)		0 (0-0)	
2009	Qatar	0 (0-0)		0 (0-0)		0 (0-0)		0 (0-0)		0 (0-0)		0 (0-0)	
2010	Qatar	0 (0-0)		0 (0-0)		0 (0-0)		0 (0-0)		0 (0-0)		0 (0-0)	
2011	Qatar	0 (0-0)		0 (0-0)		0 (0-0)		0 (0-0)		0 (0-0)		0 (0-0)	
2012	Qatar	0 (0-0)		0 (0-0)		0 (0-0)		0 (0-0)		0 (0-0)		0 (0-0)	
2013	Qatar	0 (0-0)		0 (0-0)		0 (0-0)		0 (0-0)		0 (0-0)		0 (0-0)	
2014	Qatar	0 (0-0)		0 (0-0)		0 (0-0)		0 (0-0)		0 (0-0)		0 (0-0)	
2015	Qatar	0 (0-0)	0 (0-0)	0 (0-0)	0 (0-0)	0 (0-0)	0 (0-0)	0 (0-0)	0 (0-0)	0 (0-0)	0 (0-0)	0 (0-0)	0 (0-0)
2000	Republic of Korea	30 (21-39)		1 (1-1)		12 (6-20)		0 (0-1)		0 (0-0)		0 (0-0)	
2001	Republic of Korea	26 (18-34)		1 (1-1)		10 (5-17)		0 (0-1)		0 (0-0)		0 (0-0)	
2002	Republic of Korea	24 (17-31)		1 (1-1)		10 (5-17)		0 (0-1)		0 (0-0)		0 (0-0)	
2003	Republic of Korea	19 (13-25)		1 (0-1)		8 (4-13)		0 (0-0)		0 (0-0)		0 (0-0)	
2004	Republic of Korea	14 (10-18)		1 (0-1)		9 (4-14)		0 (0-1)		0 (0-0)		0 (0-0)	
2005	Republic of Korea	13 (9-16)		0 (0-1)		6 (3-11)		0 (0-0)		0 (0-0)		0 (0-0)	
2006	Republic of Korea	15 (10-19)		1 (0-1)		9 (4-14)		0 (0-1)		0 (0-0)		0 (0-0)	
2007	Republic of Korea	11 (8-15)		0 (0-1)		10 (5-17)		0 (0-1)		0 (0-0)		0 (0-0)	
2008	Republic of Korea	11 (8-15)		0 (0-1)		7 (3-11)		0 (0-0)		0 (0-0)		0 (0-0)	
2009	Republic of Korea	11 (8-15)		0 (0-1)		7 (4-12)		0 (0-1)		0 (0-0)		0 (0-0)	
2010	Republic of Korea	11 (7-14)		0 (0-1)		7 (4-12)		0 (0-1)		0 (0-0)		0 (0-0)	
2011	Republic of Korea	10 (7-14)		0 (0-1)		9 (5-15)		0 (0-1)		0 (0-0)		0 (0-0)	
2012	Republic of Korea	7 (5-9)		0 (0-0)		7 (3-11)		0 (0-0)		0 (0-0)		0 (0-0)	
2013	Republic of Korea	8 (6-10)		0 (0-0)		6 (3-11)		0 (0-0)		0 (0-0)		0 (0-0)	
2014	Republic of Korea	1 (1-2)		0 (0-0)		1 (1-2)		0 (0-0)		0 (0-0)		0 (0-0)	
2015	Republic of Korea	1 (0-1)	0 (0-0)	0 (0-0)	0 (0-0)	1 (0-1)	0 (0-0)	0 (0-0)	0 (0-0)	0 (0-0)	0 (0-0)	0 (0-0)	0 (0-0)
2000	Republic of Moldova	28 (19-36)		12 (8-15)		6 (2-12)		3 (1-5)		0 (0-0)		0 (0-0)	
2001	Republic of Moldova	23 (16-30)		10 (7-13)		2 (0-5)		1 (0-2)		0 (0-0)		0 (0-0)	
2002	Republic of Moldova	17 (12-22)		8 (5-10)		3 (0-7)		2 (0-3)		0 (0-0)		0 (0-0)	
2003	Republic of Moldova	15 (11-20)		7 (5-10)		3 (0-6)		1 (0-3)		0 (0-0)		0 (0-0)	
2004	Republic of Moldova	11 (8-15)		6 (4-7)		3 (0-6)		1 (0-3)		0 (0-0)		0 (0-0)	
2005	Republic of Moldova	11 (7-14)		5 (4-7)		4 (0-9)		2 (0-4)		0 (0-0)		0 (0-0)	
2006	Republic of Moldova	11 (8-15)		6 (4-7)		1 (0-2)		0 (0-1)		0 (0-0)		0 (0-0)	
2007	Republic of Moldova	8 (6-11)		4 (3-5)		2 (0-4)		1 (0-2)		0 (0-0)		0 (0-0)	
2008	Republic of Moldova	9 (6-11)		4 (3-5)		2 (0-5)		1 (0-2)		0 (0-0)		0 (0-0)	
2009	Republic of Moldova	5 (4-7)		2 (2-3)		2 (0-4)		1 (0-2)		0 (0-0)		0 (0-0)	
2010	Republic of Moldova	1 (1-2)		1 (0-1)		0 (0-1)		0 (0-0)		0 (0-0)		0 (0-0)	
2011	Republic of Moldova	1 (1-1)		0 (0-1)		1 (0-1)		0 (0-0)		0 (0-0)		0 (0-0)	
2012	Republic of Moldova	1 (0-1)		0 (0-0)		0 (0-0)		0 (0-0)		0 (0-0)		0 (0-0)	
2013	Republic of Moldova	1 (0-1)		0 (0-0)		0 (0-0)		0 (0-0)		0 (0-0)		0 (0-0)	
2014	Republic of Moldova	0 (0-0)		0 (0-0)		0 (0-0)		0 (0-0)		0 (0-0)		0 (0-0)	
2015	Republic of Moldova	0 (0-0)	0 (0-0)	0 (0-0)	0 (0-0)	0 (0-0)	0 (0-0)	0 (0-0)	0 (0-0)	0 (0-0)	0 (0-0)	0 (0-0)	0 (0-0)
2000	Romania	322 (226-423)		29 (21-38)		35 (15-64)		3 (1-6)		0 (0-1)		0 (0-0)	
2001	Romania	298 (210-391)		26 (18-34)		34 (15-63)		3 (1-6)		0 (0-1)		0 (0-0)	
2002	Romania	262 (185-345)		23 (16-30)		35 (16-65)		3 (1-6)		0 (0-1)		0 (0-0)	
2003	Romania	209 (147-275)		19 (13-25)		22 (10-41)		2 (1-4)		0 (0-0)		0 (0-0)	
2004	Romania	203 (143-267)		19 (13-25)		16 (7-30)		2 (1-3)		0 (0-0)		0 (0-0)	
2005	Romania	167 (118-220)		16 (11-21)		14 (6-27)		1 (1-3)		0 (0-0)		0 (0-0)	
2006	Romania	147 (103-193)		14 (10-19)		13 (6-24)		1 (1-2)		0 (0-0)		0 (0-0)	
2007	Romania	138 (97-181)		13 (9-18)		12 (5-23)		1 (1-2)		0 (0-0)		0 (0-0)	
2008	Romania	135 (95-177)		13 (9-17)		8 (4-15)		1 (0-1)		0 (0-0)		0 (0-0)	
2009	Romania	120 (84-157)		11 (8-15)		7 (3-14)		1 (0-1)		0 (0-0)		0 (0-0)	
2010	Romania	117 (83-154)		11 (8-15)		9 (4-16)		1 (0-2)		0 (0-0)		0 (0-0)	
2011	Romania	20 (14-26)		2 (1-3)		2 (1-3)		0 (0-0)		0 (0-0)		0 (0-0)	
2012	Romania	9 (6-11)		1 (1-1)		0 (0-1)		0 (0-0)		0 (0-0)		0 (0-0)	
2013	Romania	8 (6-11)		1 (1-1)		1 (0-1)		0 (0-0)		0 (0-0)		0 (0-0)	
2014	Romania	7 (5-10)		1 (1-1)		1 (0-1)		0 (0-0)		0 (0-0)		0 (0-0)	
2015	Romania	7 (5-9)	0 (0-0)	1 (1-1)	0 (0-0)	0 (0-1)	0 (0-0)	0 (0-0)	0 (0-0)	0 (0-0)	0 (0-0)	0 (0-0)	0 (0-0)
2000	Russian Federation	607 (427-797)		10 (7-13)		154 (68-286)		2 (1-5)		1 (1-2)		0 (0-0)	
2001	Russian Federation	580 (408-763)		9 (6-12)		147 (65-272)		2 (1-4)		1 (1-2)		0 (0-0)	
2002	Russian Federation	531 (374-698)		8 (6-10)		135 (60-249)		2 (1-4)		1 (0-2)		0 (0-0)	
2003	Russian Federation	469 (330-616)		7 (5-9)		120 (53-221)		2 (1-3)		1 (0-2)		0 (0-0)	
2004	Russian Federation	421 (296-553)		6 (4-8)		108 (48-200)		2 (1-3)		1 (0-2)		0 (0-0)	
2005	Russian Federation	378 (266-497)		6 (4-7)		98 (43-181)		1 (1-3)		1 (0-1)		0 (0-0)	
2006	Russian Federation	336 (236-441)		5 (3-6)		87 (39-162)		1 (1-2)		1 (0-1)		0 (0-0)	
2007	Russian Federation	299 (210-393)		4 (3-5)		78 (35-145)		1 (0-2)		1 (0-1)		0 (0-0)	
2008	Russian Federation	270 (190-354)		4 (3-5)		70 (31-130)		1 (0-2)		1 (0-1)		0 (0-0)	
2009	Russian Federation	253 (178-332)		3 (2-4)		66 (29-121)		1 (0-2)		1 (0-1)		0 (0-0)	
2010	Russian Federation	239 (168-314)		3 (2-4)		62 (27-114)		1 (0-1)		0 (0-1)		0 (0-0)	
2011	Russian Federation	226 (159-297)		3 (2-4)		57 (25-106)		1 (0-1)		0 (0-1)		0 (0-0)	
2012	Russian Federation	209 (147-275)		3 (2-3)		64 (28-118)		1 (0-1)		1 (0-1)		0 (0-0)	
2013	Russian Federation	210 (148-276)		2 (2-3)		63 (28-117)		1 (0-1)		1 (0-1)		0 (0-0)	
2014	Russian Federation	204 (143-268)		2 (2-3)		61 (27-112)		1 (0-1)		0 (0-1)		0 (0-0)	
2015	Russian Federation	198 (139-260)	0 (0-0)	2 (2-3)	0 (0-0)	58 (26-107)	0 (0-0)	1 (0-1)	0 (0-0)	0 (0-1)	0 (0-0)	0 (0-0)	0 (0-0)
2000	Rwanda	1851 (1302-2432)		141 (99-185)		919 (395-1392)		70 (30-106)		4 (2-6)		0 (0-0)	
2001	Rwanda	1894 (1332-2489)		133 (94-175)		931 (404-1408)		65 (28-99)		4 (2-6)		0 (0-0)	
2002	Rwanda	266 (187-350)		18 (13-24)		129 (57-195)		9 (4-13)		1 (0-1)		0 (0-0)	
2003	Rwanda	106 (75-139)		7 (5-9)		51 (15-85)		3 (1-6)		0 (0-0)		0 (0-0)	
2004	Rwanda	106 (74-139)		7 (5-10)		51 (15-84)		3 (1-6)		0 (0-0)		0 (0-0)	
2005	Rwanda	81 (57-106)		6 (4-7)		38 (11-63)		3 (1-4)		0 (0-0)		0 (0-0)	
2006	Rwanda	58 (41-76)		4 (3-5)		27 (8-45)		2 (1-3)		0 (0-0)		0 (0-0)	
2007	Rwanda	9 (6-11)		1 (0-1)		4 (1-7)		0 (0-0)		0 (0-0)		0 (0-0)	
2008	Rwanda	8 (6-10)		1 (0-1)		4 (1-6)		0 (0-0)		0 (0-0)		0 (0-0)	
2009	Rwanda	7 (5-9)		0 (0-1)		3 (1-5)		0 (0-0)		0 (0-0)		0 (0-0)	
2010	Rwanda	6 (5-9)		0 (0-1)		3 (1-5)		0 (0-0)		0 (0-0)		0 (0-0)	
2011	Rwanda	6 (4-8)		0 (0-0)		3 (1-4)		0 (0-0)		0 (0-0)		0 (0-0)	
2012	Rwanda	5 (4-7)		0 (0-0)		2 (1-4)		0 (0-0)		0 (0-0)		0 (0-0)	
2013	Rwanda	5 (3-6)		0 (0-0)		2 (1-3)		0 (0-0)		0 (0-0)		0 (0-0)	
2014	Rwanda	4 (3-5)		0 (0-0)		2 (1-3)		0 (0-0)		0 (0-0)		0 (0-0)	

Hib = *Haemophilus influenzae* type b; npnm = non-pneumonia, non-meningitis; HIV = deaths only in HIV-infected children; Uncertainty range provided in parentheses.

Year	Country	Hib pneumonia deaths (UR)	Hib pneumonia deaths, HIV (UR)	Hib pneumonia mortality rate (UR)	Hib pneumonia mortality rate, HIV (UR)	Hib meningitis deaths (UR)	Hib meningitis deaths, HIV (UR)	Hib meningitis mortality rate (UR)	Hib meningitis mortality rate, HIV (UR)	Hib NPNM deaths (UR)	Hib NPNM deaths, HIV (UR)	Hib NPNM mortality rate (UR)	Hib NPNM mortality rate, HIV (UR)
2015	Rwanda	4 (3-5)	0 (0-0)	0 (0-0)	0 (0-0)	2 (1-3)	0 (0-0)	0 (0-0)	0 (0-0)	0 (0-0)	0 (0-0)	0 (0-0)	0 (0-0)
2000	Saint Kitts and Nevis	0 (0-0)		1 (1-2)		0 (0-0)		0 (0-0)		0 (0-0)		0 (0-0)	
2001	Saint Kitts and Nevis	0 (0-0)		0 (0-0)		0 (0-0)		0 (0-0)		0 (0-0)		0 (0-0)	
2002	Saint Kitts and Nevis	0 (0-0)		0 (0-0)		0 (0-0)		0 (0-0)		0 (0-0)		0 (0-0)	
2003	Saint Kitts and Nevis	0 (0-0)		0 (0-0)		0 (0-0)		0 (0-0)		0 (0-0)		0 (0-0)	
2004	Saint Kitts and Nevis	0 (0-0)		0 (0-0)		0 (0-0)		0 (0-0)		0 (0-0)		0 (0-0)	
2005	Saint Kitts and Nevis	0 (0-0)		0 (0-0)		0 (0-0)		0 (0-0)		0 (0-0)		0 (0-0)	
2006	Saint Kitts and Nevis	0 (0-0)		0 (0-0)		0 (0-0)		0 (0-0)		0 (0-0)		0 (0-0)	
2007	Saint Kitts and Nevis	0 (0-0)		0 (0-0)		0 (0-0)		0 (0-0)		0 (0-0)		0 (0-0)	
2008	Saint Kitts and Nevis	0 (0-0)		0 (0-0)		0 (0-0)		0 (0-0)		0 (0-0)		0 (0-0)	
2009	Saint Kitts and Nevis	0 (0-0)		0 (0-0)		0 (0-0)		0 (0-0)		0 (0-0)		0 (0-0)	
2010	Saint Kitts and Nevis	0 (0-0)		0 (0-0)		0 (0-0)		0 (0-0)		0 (0-0)		0 (0-0)	
2011	Saint Kitts and Nevis	0 (0-0)		0 (0-0)		0 (0-0)		0 (0-0)		0 (0-0)		0 (0-0)	
2012	Saint Kitts and Nevis	0 (0-0)		0 (0-0)		0 (0-0)		0 (0-0)		0 (0-0)		0 (0-0)	
2013	Saint Kitts and Nevis	0 (0-0)		0 (0-0)		0 (0-0)		0 (0-0)		0 (0-0)		0 (0-0)	
2014	Saint Kitts and Nevis	0 (0-0)		0 (0-0)		0 (0-0)		0 (0-0)		0 (0-0)		0 (0-0)	
2015	Saint Kitts and Nevis	0 (0-0)	0 (0-0)	0 (0-0)	0 (0-0)	0 (0-0)	0 (0-0)	0 (0-0)	0 (0-0)	0 (0-0)	0 (0-0)	0 (0-0)	0 (0-0)
2000	Saint Lucia	0 (0-0)		2 (2-3)		0 (0-0)		1 (0-1)		0 (0-0)		0 (0-0)	
2001	Saint Lucia	0 (0-1)		3 (2-4)		0 (0-0)		0 (0-1)		0 (0-0)		0 (0-0)	
2002	Saint Lucia	0 (0-1)		3 (2-4)		0 (0-0)		1 (0-1)		0 (0-0)		0 (0-0)	
2003	Saint Lucia	0 (0-0)		0 (0-0)		0 (0-0)		0 (0-0)		0 (0-0)		0 (0-0)	
2004	Saint Lucia	0 (0-0)		0 (0-0)		0 (0-0)		0 (0-0)		0 (0-0)		0 (0-0)	
2005	Saint Lucia	0 (0-0)		0 (0-0)		0 (0-0)		0 (0-0)		0 (0-0)		0 (0-0)	
2006	Saint Lucia	0 (0-0)		1 (0-1)		0 (0-0)		0 (0-0)		0 (0-0)		0 (0-0)	
2007	Saint Lucia	0 (0-0)		0 (0-0)		0 (0-0)		0 (0-0)		0 (0-0)		0 (0-0)	
2008	Saint Lucia	0 (0-0)		0 (0-0)		0 (0-0)		0 (0-0)		0 (0-0)		0 (0-0)	
2009	Saint Lucia	0 (0-0)		0 (0-0)		0 (0-0)		0 (0-0)		0 (0-0)		0 (0-0)	
2010	Saint Lucia	0 (0-0)		0 (0-0)		0 (0-0)		0 (0-0)		0 (0-0)		0 (0-0)	
2011	Saint Lucia	0 (0-0)		0 (0-0)		0 (0-0)		0 (0-0)		0 (0-0)		0 (0-0)	
2012	Saint Lucia	0 (0-0)		0 (0-0)		0 (0-0)		0 (0-0)		0 (0-0)		0 (0-0)	
2013	Saint Lucia	0 (0-0)		0 (0-0)		0 (0-0)		0 (0-0)		0 (0-0)		0 (0-0)	
2014	Saint Lucia	0 (0-0)		0 (0-0)		0 (0-0)		0 (0-0)		0 (0-0)		0 (0-0)	
2015	Saint Lucia	0 (0-0)	0 (0-0)	0 (0-0)	0 (0-0)	0 (0-0)	0 (0-0)	0 (0-0)	0 (0-0)	0 (0-0)	0 (0-0)	0 (0-0)	0 (0-0)
2000	Saint Vincent and the Grenadines	0 (0-0)		3 (2-3)		0 (0-0)		1 (1-2)		0 (0-0)		0 (0-0)	
2001	Saint Vincent and the Grenadines	0 (0-0)		3 (2-3)		0 (0-0)		1 (0-1)		0 (0-0)		0 (0-0)	
2002	Saint Vincent and the Grenadines	0 (0-0)		1 (1-2)		0 (0-0)		0 (0-0)		0 (0-0)		0 (0-0)	
2003	Saint Vincent and the Grenadines	0 (0-0)		1 (1-1)		0 (0-0)		0 (0-0)		0 (0-0)		0 (0-0)	
2004	Saint Vincent and the Grenadines	0 (0-0)		0 (0-0)		0 (0-0)		0 (0-0)		0 (0-0)		0 (0-0)	
2005	Saint Vincent and the Grenadines	0 (0-0)		0 (0-0)		0 (0-0)		0 (0-0)		0 (0-0)		0 (0-0)	
2006	Saint Vincent and the Grenadines	0 (0-0)		0 (0-0)		0 (0-0)		0 (0-0)		0 (0-0)		0 (0-0)	
2007	Saint Vincent and the Grenadines	0 (0-0)		0 (0-0)		0 (0-0)		0 (0-0)		0 (0-0)		0 (0-0)	
2008	Saint Vincent and the Grenadines	0 (0-0)		0 (0-0)		0 (0-0)		0 (0-0)		0 (0-0)		0 (0-0)	
2009	Saint Vincent and the Grenadines	0 (0-0)		0 (0-0)		0 (0-0)		0 (0-0)		0 (0-0)		0 (0-0)	
2010	Saint Vincent and the Grenadines	0 (0-0)		0 (0-0)		0 (0-0)		0 (0-0)		0 (0-0)		0 (0-0)	
2011	Saint Vincent and the Grenadines	0 (0-0)		0 (0-0)		0 (0-0)		0 (0-0)		0 (0-0)		0 (0-0)	
2012	Saint Vincent and the Grenadines	0 (0-0)		0 (0-0)		0 (0-0)		0 (0-0)		0 (0-0)		0 (0-0)	
2013	Saint Vincent and the Grenadines	0 (0-0)		0 (0-0)		0 (0-0)		0 (0-0)		0 (0-0)		0 (0-0)	
2014	Saint Vincent and the Grenadines	0 (0-0)		0 (0-0)		0 (0-0)		0 (0-0)		0 (0-0)		0 (0-0)	
2015	Saint Vincent and the Grenadines	0 (0-0)	0 (0-0)	0 (0-0)	0 (0-0)	0 (0-0)	0 (0-0)	0 (0-0)	0 (0-0)	0 (0-0)	0 (0-0)	0 (0-0)	0 (0-0)
2000	Samoa	2 (1-3)		8 (6-10)		1 (0-1)		3 (2-4)		0 (0-0)		0 (0-0)	
2001	Samoa	2 (1-2)		7 (5-10)		1 (0-1)		3 (1-4)		0 (0-0)		0 (0-0)	
2002	Samoa	2 (1-2)		7 (5-9)		1 (0-1)		2 (1-3)		0 (0-0)		0 (0-0)	
2003	Samoa	2 (1-2)		7 (5-9)		1 (0-1)		2 (1-3)		0 (0-0)		0 (0-0)	
2004	Samoa	1 (1-2)		6 (4-8)		1 (0-1)		2 (1-3)		0 (0-0)		0 (0-0)	
2005	Samoa	1 (1-2)		6 (4-7)		0 (0-1)		2 (1-3)		0 (0-0)		0 (0-0)	
2006	Samoa	1 (1-2)		5 (4-7)		0 (0-1)		2 (1-2)		0 (0-0)		0 (0-0)	
2007	Samoa	1 (1-2)		5 (4-7)		0 (0-1)		2 (1-2)		0 (0-0)		0 (0-0)	
2008	Samoa	1 (1-2)		5 (3-6)		1 (0-1)		2 (1-3)		0 (0-0)		0 (0-0)	
2009	Samoa	1 (1-1)		3 (2-4)		0 (0-1)		2 (1-2)		0 (0-0)		0 (0-0)	
2010	Samoa	0 (0-0)		1 (1-1)		0 (0-0)		1 (0-1)		0 (0-0)		0 (0-0)	
2011	Samoa	0 (0-0)		1 (1-1)		0 (0-0)		1 (0-1)		0 (0-0)		0 (0-0)	
2012	Samoa	0 (0-0)		1 (1-1)		0 (0-0)		0 (0-1)		0 (0-0)		0 (0-0)	
2013	Samoa	0 (0-0)		1 (1-1)		0 (0-0)		0 (0-1)		0 (0-0)		0 (0-0)	
2014	Samoa	0 (0-0)		1 (1-1)		0 (0-0)		0 (0-1)		0 (0-0)		0 (0-0)	
2015	Samoa	0 (0-0)	0 (0-0)	1 (0-1)	0 (0-0)	0 (0-0)	0 (0-0)	0 (0-1)	0 (0-0)	0 (0-0)	0 (0-0)	0 (0-0)	0 (0-0)
2000	San Marino	0 (0-0)		0 (0-0)		0 (0-0)		0 (0-0)		0 (0-0)		0 (0-0)	
2001	San Marino	0 (0-0)		0 (0-0)		0 (0-0)		0 (0-0)		0 (0-0)		0 (0-0)	
2002	San Marino	0 (0-0)		0 (0-0)		0 (0-0)		0 (0-0)		0 (0-0)		0 (0-0)	
2003	San Marino	0 (0-0)		0 (0-0)		0 (0-0)		0 (0-0)		0 (0-0)		0 (0-0)	
2004	San Marino	0 (0-0)		0 (0-0)		0 (0-0)		0 (0-0)		0 (0-0)		0 (0-0)	
2005	San Marino	0 (0-0)		0 (0-0)		0 (0-0)		0 (0-0)		0 (0-0)		0 (0-0)	
2006	San Marino	0 (0-0)		0 (0-0)		0 (0-0)		0 (0-0)		0 (0-0)		0 (0-0)	
2007	San Marino	0 (0-0)		0 (0-0)		0 (0-0)		0 (0-0)		0 (0-0)		0 (0-0)	
2008	San Marino	0 (0-0)		0 (0-0)		0 (0-0)		0 (0-0)		0 (0-0)		0 (0-0)	
2009	San Marino	0 (0-0)		0 (0-0)		0 (0-0)		0 (0-0)		0 (0-0)		0 (0-0)	
2010	San Marino	0 (0-0)		0 (0-0)		0 (0-0)		0 (0-0)		0 (0-0)		0 (0-0)	
2011	San Marino	0 (0-0)		0 (0-0)		0 (0-0)		0 (0-0)		0 (0-0)		0 (0-0)	
2012	San Marino	0 (0-0)		0 (0-0)		0 (0-0)		0 (0-0)		0 (0-0)		0 (0-0)	
2013	San Marino	0 (0-0)		0 (0-0)		0 (0-0)		0 (0-0)		0 (0-0)		0 (0-0)	
2014	San Marino	0 (0-0)		0 (0-0)		0 (0-0)		0 (0-0)		0 (0-0)		0 (0-0)	
2015	San Marino	0 (0-0)	0 (0-0)	0 (0-0)	0 (0-0)	0 (0-0)	0 (0-0)	0 (0-0)	0 (0-0)	0 (0-0)	0 (0-0)	0 (0-0)	0 (0-0)
2000	Sao Tome and Principe	13 (9-18)		61 (43-80)		5 (2-8)		22 (7-35)		0 (0-0)		0 (0-0)	
2001	Sao Tome and Principe	13 (9-17)		58 (41-76)		5 (2-8)		21 (7-33)		0 (0-0)		0 (0-0)	
2002	Sao Tome and Principe	13 (9-17)		55 (38-72)		5 (2-7)		20 (7-31)		0 (0-0)		0 (0-0)	
2003	Sao Tome and Principe	13 (9-16)		52 (36-68)		4 (2-7)		18 (7-29)		0 (0-0)		0 (0-0)	
2004	Sao Tome and Principe	12 (8-16)		48 (34-63)		4 (1-7)		17 (6-28)		0 (0-0)		0 (0-0)	
2005	Sao Tome and Principe	12 (8-15)		45 (32-60)		4 (1-7)		16 (6-26)		0 (0-0)		0 (0-0)	
2006	Sao Tome and Principe	11 (8-15)		43 (30-56)		4 (1-6)		15 (6-24)		0 (0-0)		0 (0-0)	

Hib = *Haemophilus influenzae* type b; npnm = non-pneumonia, non-meningitis; HIV = deaths only in HIV-infected children; Uncertainty range provided in parentheses.

Year	Country	Hib pneumonia deaths (UR)	Hib pneumonia deaths, HIV (UR)	Hib pneumonia mortality rate (UR)	Hib pneumonia mortality rate, HIV (UR)	Hib meningitis deaths (UR)	Hib meningitis deaths, HIV (UR)	Hib meningitis mortality rate (UR)	Hib meningitis mortality rate, HIV (UR)	Hib NPNM deaths (UR)	Hib NPNM deaths, HIV (UR)	Hib NPNM mortality rate (UR)	Hib NPNM mortality rate, HIV (UR)
2007	Sao Tome and Principe	11 (7-14)		40 (28-53)		4 (1-6)		14 (5-22)		0 (0-0)		0 (0-0)	
2008	Sao Tome and Principe	11 (7-14)		40 (28-52)		4 (1-6)		13 (5-21)		0 (0-0)		0 (0-0)	
2009	Sao Tome and Principe	10 (7-13)		37 (26-48)		3 (1-5)		12 (5-19)		0 (0-0)		0 (0-0)	
2010	Sao Tome and Principe	1 (1-2)		5 (4-7)		0 (0-1)		2 (1-3)		0 (0-0)		0 (0-0)	
2011	Sao Tome and Principe	1 (0-1)		2 (2-3)		0 (0-0)		1 (0-1)		0 (0-0)		0 (0-0)	
2012	Sao Tome and Principe	1 (0-1)		2 (2-3)		0 (0-0)		1 (0-1)		0 (0-0)		0 (0-0)	
2013	Sao Tome and Principe	1 (0-1)		2 (2-3)		0 (0-0)		1 (0-1)		0 (0-0)		0 (0-0)	
2014	Sao Tome and Principe	1 (0-1)		2 (2-3)		0 (0-0)		1 (0-1)		0 (0-0)		0 (0-0)	
2015	Sao Tome and Principe	0 (0-0)	0 (0-0)	0 (0-0)	0 (0-0)	0 (0-0)	0 (0-0)	0 (0-0)	0 (0-0)	0 (0-0)	0 (0-0)	0 (0-0)	0 (0-0)
2000	Saudi Arabia	211 (148-277)		8 (5-10)		25 (13-42)		1 (0-2)		0 (0-0)		0 (0-0)	
2001	Saudi Arabia	194 (137-255)		7 (5-9)		23 (12-39)		1 (0-1)		0 (0-0)		0 (0-0)	
2002	Saudi Arabia	30 (21-40)		1 (1-1)		5 (3-9)		0 (0-0)		0 (0-0)		0 (0-0)	
2003	Saudi Arabia	14 (10-18)		1 (0-1)		2 (1-4)		0 (0-0)		0 (0-0)		0 (0-0)	
2004	Saudi Arabia	13 (9-17)		0 (0-1)		2 (1-4)		0 (0-0)		0 (0-0)		0 (0-0)	
2005	Saudi Arabia	12 (8-16)		0 (0-1)		2 (1-4)		0 (0-0)		0 (0-0)		0 (0-0)	
2006	Saudi Arabia	11 (8-15)		0 (0-1)		2 (1-3)		0 (0-0)		0 (0-0)		0 (0-0)	
2007	Saudi Arabia	2 (1-2)		0 (0-0)		0 (0-0)		0 (0-0)		0 (0-0)		0 (0-0)	
2008	Saudi Arabia	2 (1-2)		0 (0-0)		0 (0-0)		0 (0-0)		0 (0-0)		0 (0-0)	
2009	Saudi Arabia	2 (1-2)		0 (0-0)		0 (0-1)		0 (0-0)		0 (0-0)		0 (0-0)	
2010	Saudi Arabia	2 (1-2)		0 (0-0)		0 (0-1)		0 (0-0)		0 (0-0)		0 (0-0)	
2011	Saudi Arabia	2 (1-2)		0 (0-0)		0 (0-1)		0 (0-0)		0 (0-0)		0 (0-0)	
2012	Saudi Arabia	1 (1-2)		0 (0-0)		0 (0-1)		0 (0-0)		0 (0-0)		0 (0-0)	
2013	Saudi Arabia	1 (1-2)		0 (0-0)		0 (0-1)		0 (0-0)		0 (0-0)		0 (0-0)	
2014	Saudi Arabia	1 (1-2)		0 (0-0)		0 (0-1)		0 (0-0)		0 (0-0)		0 (0-0)	
2015	Saudi Arabia	1 (1-2)	0 (0-0)	0 (0-0)	0 (0-0)	0 (0-0)	0 (0-0)	0 (0-0)	0 (0-0)	0 (0-0)	0 (0-0)	0 (0-0)	0 (0-0)
2000	Senegal	1201 (845-1578)		73 (51-95)		453 (132-749)		27 (8-45)		2 (1-3)		0 (0-0)	
2001	Senegal	1223 (860-1607)		72 (51-95)		459 (137-754)		27 (8-44)		2 (1-3)		0 (0-0)	
2002	Senegal	1198 (843-1574)		69 (48-91)		447 (137-731)		26 (8-42)		2 (1-3)		0 (0-0)	
2003	Senegal	1168 (821-1534)		65 (46-86)		434 (137-705)		24 (8-40)		2 (1-3)		0 (0-0)	
2004	Senegal	1135 (798-1491)		62 (44-82)		420 (137-678)		23 (7-37)		2 (1-3)		0 (0-0)	
2005	Senegal	1002 (705-1317)		53 (38-70)		370 (125-593)		20 (7-32)		2 (1-2)		0 (0-0)	
2006	Senegal	88 (62-115)		5 (3-6)		32 (11-52)		2 (1-3)		0 (0-0)		0 (0-0)	
2007	Senegal	75 (53-99)		4 (3-5)		28 (9-44)		1 (0-2)		0 (0-0)		0 (0-0)	
2008	Senegal	83 (58-108)		4 (3-5)		30 (10-49)		1 (1-2)		0 (0-0)		0 (0-0)	
2009	Senegal	83 (58-109)		4 (3-5)		30 (9-50)		1 (0-2)		0 (0-0)		0 (0-0)	
2010	Senegal	9 (6-12)		0 (0-1)		3 (1-6)		0 (0-0)		0 (0-0)		0 (0-0)	
2011	Senegal	9 (6-12)		0 (0-1)		3 (1-5)		0 (0-0)		0 (0-0)		0 (0-0)	
2012	Senegal	9 (6-12)		0 (0-1)		3 (1-5)		0 (0-0)		0 (0-0)		0 (0-0)	
2013	Senegal	9 (6-12)		0 (0-0)		3 (1-5)		0 (0-0)		0 (0-0)		0 (0-0)	
2014	Senegal	9 (6-12)		0 (0-0)		3 (1-5)		0 (0-0)		0 (0-0)		0 (0-0)	
2015	Senegal	9 (6-12)	0 (0-0)	0 (0-0)	0 (0-0)	3 (1-5)	0 (0-0)	0 (0-0)	0 (0-0)	0 (0-0)	0 (0-0)	0 (0-0)	0 (0-0)
2000	Serbia	13 (9-17)		2 (1-3)		6 (2-10)		1 (0-2)		0 (0-0)		0 (0-0)	
2001	Serbia	9 (6-12)		1 (1-2)		5 (2-9)		1 (0-1)		0 (0-0)		0 (0-0)	
2002	Serbia	7 (5-9)		1 (1-2)		3 (1-5)		0 (0-1)		0 (0-0)		0 (0-0)	
2003	Serbia	10 (7-14)		2 (1-2)		4 (1-8)		1 (0-1)		0 (0-0)		0 (0-0)	
2004	Serbia	9 (7-12)		2 (1-2)		3 (1-6)		1 (0-1)		0 (0-0)		0 (0-0)	
2005	Serbia	7 (5-10)		1 (1-2)		5 (1-9)		1 (0-2)		0 (0-0)		0 (0-0)	
2006	Serbia	3 (2-4)		1 (0-1)		2 (1-4)		0 (0-1)		0 (0-0)		0 (0-0)	
2007	Serbia	1 (0-1)		0 (0-0)		0 (0-1)		0 (0-0)		0 (0-0)		0 (0-0)	
2008	Serbia	1 (0-1)		0 (0-0)		0 (0-0)		0 (0-0)		0 (0-0)		0 (0-0)	
2009	Serbia	0 (0-1)		0 (0-0)		0 (0-1)		0 (0-0)		0 (0-0)		0 (0-0)	
2010	Serbia	0 (0-1)		0 (0-0)		0 (0-0)		0 (0-0)		0 (0-0)		0 (0-0)	
2011	Serbia	0 (0-0)		0 (0-0)		0 (0-0)		0 (0-0)		0 (0-0)		0 (0-0)	
2012	Serbia	0 (0-0)		0 (0-0)		0 (0-0)		0 (0-0)		0 (0-0)		0 (0-0)	
2013	Serbia	0 (0-0)		0 (0-0)		0 (0-0)		0 (0-0)		0 (0-0)		0 (0-0)	
2014	Serbia	0 (0-0)		0 (0-0)		0 (0-0)		0 (0-0)		0 (0-0)		0 (0-0)	
2015	Serbia	0 (0-0)	0 (0-0)	0 (0-0)	0 (0-0)	0 (0-0)	0 (0-0)	0 (0-0)	0 (0-0)	0 (0-0)	0 (0-0)	0 (0-0)	0 (0-0)
2000	Seychelles	0 (0-0)		2 (2-3)		0 (0-0)		1 (0-1)		0 (0-0)		0 (0-0)	
2001	Seychelles	0 (0-0)		3 (2-4)		0 (0-0)		1 (0-1)		0 (0-0)		0 (0-0)	
2002	Seychelles	0 (0-0)		3 (2-4)		0 (0-0)		1 (0-1)		0 (0-0)		0 (0-0)	
2003	Seychelles	0 (0-0)		3 (2-3)		0 (0-0)		1 (0-1)		0 (0-0)		0 (0-0)	
2004	Seychelles	0 (0-0)		3 (2-3)		0 (0-0)		1 (0-1)		0 (0-0)		0 (0-0)	
2005	Seychelles	0 (0-0)		3 (2-4)		0 (0-0)		1 (0-1)		0 (0-0)		0 (0-0)	
2006	Seychelles	0 (0-0)		3 (2-4)		0 (0-0)		1 (0-1)		0 (0-0)		0 (0-0)	
2007	Seychelles	0 (0-0)		3 (2-3)		0 (0-0)		1 (0-1)		0 (0-0)		0 (0-0)	
2008	Seychelles	0 (0-0)		3 (2-3)		0 (0-0)		1 (0-1)		0 (0-0)		0 (0-0)	
2009	Seychelles	0 (0-0)		3 (2-4)		0 (0-0)		1 (0-1)		0 (0-0)		0 (0-0)	
2010	Seychelles	0 (0-0)		0 (0-1)		0 (0-0)		0 (0-0)		0 (0-0)		0 (0-0)	
2011	Seychelles	0 (0-0)		0 (0-0)		0 (0-0)		0 (0-0)		0 (0-0)		0 (0-0)	
2012	Seychelles	0 (0-0)		0 (0-0)		0 (0-0)		0 (0-0)		0 (0-0)		0 (0-0)	
2013	Seychelles	0 (0-0)		0 (0-0)		0 (0-0)		0 (0-0)		0 (0-0)		0 (0-0)	
2014	Seychelles	0 (0-0)		0 (0-0)		0 (0-0)		0 (0-0)		0 (0-0)		0 (0-0)	
2015	Seychelles	0 (0-0)	0 (0-0)	0 (0-0)	0 (0-0)	0 (0-0)	0 (0-0)	0 (0-0)	0 (0-0)	0 (0-0)	0 (0-0)	0 (0-0)	0 (0-0)
2000	Sierra Leone	844 (594-1109)		121 (85-159)		381 (191-555)		55 (27-80)		2 (1-2)		0 (0-0)	
2001	Sierra Leone	860 (605-1129)		118 (83-155)		387 (193-564)		53 (27-78)		2 (1-2)		0 (0-0)	
2002	Sierra Leone	886 (623-1165)		116 (82-153)		396 (197-577)		52 (26-76)		2 (1-2)		0 (0-0)	
2003	Sierra Leone	920 (647-1209)		115 (81-152)		408 (203-595)		51 (25-75)		2 (1-2)		0 (0-0)	
2004	Sierra Leone	957 (673-1257)		115 (81-151)		421 (209-614)		51 (25-74)		2 (1-3)		0 (0-0)	
2005	Sierra Leone	980 (689-1288)		114 (80-150)		425 (210-620)		49 (24-72)		2 (1-3)		0 (0-0)	
2006	Sierra Leone	997 (701-1310)		112 (79-147)		426 (210-623)		48 (24-70)		2 (1-3)		0 (0-0)	
2007	Sierra Leone	365 (257-480)		40 (28-53)		154 (76-225)		17 (8-25)		1 (0-1)		0 (0-0)	
2008	Sierra Leone	97 (68-127)		10 (7-14)		40 (20-59)		4 (2-6)		0 (0-0)		0 (0-0)	
2009	Sierra Leone	81 (57-106)		9 (6-11)		33 (18-48)		4 (2-5)		0 (0-0)		0 (0-0)	
2010	Sierra Leone	73 (52-96)		8 (5-10)		30 (17-41)		3 (2-4)		0 (0-0)		0 (0-0)	
2011	Sierra Leone	67 (47-88)		7 (5-9)		27 (15-38)		3 (2-4)		0 (0-0)		0 (0-0)	
2012	Sierra Leone	8 (6-11)		1 (1-1)		3 (1-5)		0 (0-0)		0 (0-0)		0 (0-0)	
2013	Sierra Leone	8 (6-10)		1 (1-1)		3 (1-5)		0 (0-0)		0 (0-0)		0 (0-0)	
2014	Sierra Leone	8 (6-10)		1 (1-1)		3 (1-5)		0 (0-0)		0 (0-0)		0 (0-0)	

Hib = *Haemophilus influenzae* type b; npnm = non-pneumonia, non-meningitis; HIV = deaths only in HIV-infected children; Uncertainty range provided in parentheses.

Year	Country	Hib pneumonia deaths (UR)	Hib pneumonia deaths, HIV (UR)	Hib pneumonia mortality rate (UR)	Hib pneumonia mortality rate, HIV (UR)	Hib meningitis deaths (UR)	Hib meningitis deaths, HIV (UR)	Hib meningitis mortality rate (UR)	Hib meningitis mortality rate, HIV (UR)	Hib NPNM deaths (UR)	Hib NPNM deaths, HIV (UR)	Hib NPNM mortality rate (UR)	Hib NPNM mortality rate, HIV (UR)
2015	Sierra Leone	8 (6-10)	0 (0-0)	1 (1-1)	0 (0-0)	3 (1-5)	0 (0-0)	0 (0-0)	0 (0-0)	0 (0-0)	0 (0-0)	0 (0-0)	0 (0-0)
2000	Singapore	2 (2-3)		1 (1-1)		1 (1-2)		1 (0-1)		0 (0-0)		0 (0-0)	
2001	Singapore	3 (2-4)		1 (1-2)		1 (0-1)		0 (0-0)		0 (0-0)		0 (0-0)	
2002	Singapore	3 (2-4)		1 (1-2)		1 (0-1)		0 (0-1)		0 (0-0)		0 (0-0)	
2003	Singapore	5 (3-6)		2 (1-2)		0 (0-0)		0 (0-0)		0 (0-0)		0 (0-0)	
2004	Singapore	3 (2-5)		1 (1-2)		0 (0-0)		0 (0-0)		0 (0-0)		0 (0-0)	
2005	Singapore	4 (3-5)		2 (1-2)		0 (0-1)		0 (0-0)		0 (0-0)		0 (0-0)	
2006	Singapore	4 (3-5)		2 (1-2)		0 (0-0)		0 (0-0)		0 (0-0)		0 (0-0)	
2007	Singapore	5 (3-6)		2 (1-2)		0 (0-1)		0 (0-0)		0 (0-0)		0 (0-0)	
2008	Singapore	3 (2-3)		1 (1-1)		0 (0-1)		0 (0-0)		0 (0-0)		0 (0-0)	
2009	Singapore	3 (2-4)		1 (1-1)		1 (0-1)		0 (0-0)		0 (0-0)		0 (0-0)	
2010	Singapore	2 (1-2)		1 (1-1)		0 (0-0)		0 (0-0)		0 (0-0)		0 (0-0)	
2011	Singapore	2 (2-3)		1 (1-1)		1 (0-1)		0 (0-0)		0 (0-0)		0 (0-0)	
2012	Singapore	4 (3-5)		2 (1-2)		0 (0-0)		0 (0-0)		0 (0-0)		0 (0-0)	
2013	Singapore	1 (0-1)		0 (0-0)		0 (0-0)		0 (0-0)		0 (0-0)		0 (0-0)	
2014	Singapore	0 (0-1)		0 (0-0)		0 (0-0)		0 (0-0)		0 (0-0)		0 (0-0)	
2015	Singapore	0 (0-1)	0 (0-0)	0 (0-0)	0 (0-0)	0 (0-0)	0 (0-0)	0 (0-0)	0 (0-0)	0 (0-0)	0 (0-0)	0 (0-0)	0 (0-0)
2000	Slovakia	2 (1-2)		1 (0-1)		0 (0-1)		0 (0-0)		0 (0-0)		0 (0-0)	
2001	Slovakia	0 (0-0)		0 (0-0)		0 (0-0)		0 (0-0)		0 (0-0)		0 (0-0)	
2002	Slovakia	0 (0-0)		0 (0-0)		0 (0-0)		0 (0-0)		0 (0-0)		0 (0-0)	
2003	Slovakia	0 (0-0)		0 (0-0)		0 (0-0)		0 (0-0)		0 (0-0)		0 (0-0)	
2004	Slovakia	0 (0-0)		0 (0-0)		0 (0-0)		0 (0-0)		0 (0-0)		0 (0-0)	
2005	Slovakia	0 (0-0)		0 (0-0)		0 (0-0)		0 (0-0)		0 (0-0)		0 (0-0)	
2006	Slovakia	0 (0-0)		0 (0-0)		0 (0-0)		0 (0-0)		0 (0-0)		0 (0-0)	
2007	Slovakia	0 (0-0)		0 (0-0)		0 (0-0)		0 (0-0)		0 (0-0)		0 (0-0)	
2008	Slovakia	0 (0-0)		0 (0-0)		0 (0-0)		0 (0-0)		0 (0-0)		0 (0-0)	
2009	Slovakia	0 (0-0)		0 (0-0)		0 (0-0)		0 (0-0)		0 (0-0)		0 (0-0)	
2010	Slovakia	0 (0-0)		0 (0-0)		0 (0-0)		0 (0-0)		0 (0-0)		0 (0-0)	
2011	Slovakia	0 (0-0)		0 (0-0)		0 (0-0)		0 (0-0)		0 (0-0)		0 (0-0)	
2012	Slovakia	0 (0-0)		0 (0-0)		0 (0-0)		0 (0-0)		0 (0-0)		0 (0-0)	
2013	Slovakia	0 (0-0)		0 (0-0)		0 (0-0)		0 (0-0)		0 (0-0)		0 (0-0)	
2014	Slovakia	0 (0-0)		0 (0-0)		0 (0-0)		0 (0-0)		0 (0-0)		0 (0-0)	
2015	Slovakia	0 (0-0)	0 (0-0)	0 (0-0)	0 (0-0)	0 (0-0)	0 (0-0)	0 (0-0)	0 (0-0)	0 (0-0)	0 (0-0)	0 (0-0)	0 (0-0)
2000	Slovenia	0 (0-0)		0 (0-0)		0 (0-0)		0 (0-0)		0 (0-0)		0 (0-0)	
2001	Slovenia	0 (0-0)		0 (0-0)		0 (0-0)		0 (0-0)		0 (0-0)		0 (0-0)	
2002	Slovenia	0 (0-0)		0 (0-0)		0 (0-0)		0 (0-0)		0 (0-0)		0 (0-0)	
2003	Slovenia	0 (0-0)		0 (0-0)		0 (0-0)		0 (0-0)		0 (0-0)		0 (0-0)	
2004	Slovenia	0 (0-0)		0 (0-0)		0 (0-0)		0 (0-0)		0 (0-0)		0 (0-0)	
2005	Slovenia	0 (0-0)		0 (0-0)		0 (0-0)		0 (0-0)		0 (0-0)		0 (0-0)	
2006	Slovenia	0 (0-0)		0 (0-0)		0 (0-0)		0 (0-0)		0 (0-0)		0 (0-0)	
2007	Slovenia	0 (0-0)		0 (0-0)		0 (0-0)		0 (0-0)		0 (0-0)		0 (0-0)	
2008	Slovenia	0 (0-0)		0 (0-0)		0 (0-0)		0 (0-0)		0 (0-0)		0 (0-0)	
2009	Slovenia	0 (0-0)		0 (0-0)		0 (0-0)		0 (0-0)		0 (0-0)		0 (0-0)	
2010	Slovenia	0 (0-0)		0 (0-0)		0 (0-0)		0 (0-0)		0 (0-0)		0 (0-0)	
2011	Slovenia	0 (0-0)		0 (0-0)		0 (0-0)		0 (0-0)		0 (0-0)		0 (0-0)	
2012	Slovenia	0 (0-0)		0 (0-0)		0 (0-0)		0 (0-0)		0 (0-0)		0 (0-0)	
2013	Slovenia	0 (0-0)		0 (0-0)		0 (0-0)		0 (0-0)		0 (0-0)		0 (0-0)	
2014	Slovenia	0 (0-0)		0 (0-0)		0 (0-0)		0 (0-0)		0 (0-0)		0 (0-0)	
2015	Slovenia	0 (0-0)	0 (0-0)	0 (0-0)	0 (0-0)	0 (0-0)	0 (0-0)	0 (0-0)	0 (0-0)	0 (0-0)	0 (0-0)	0 (0-0)	0 (0-0)
2000	Solomon Islands	12 (8-16)		19 (13-25)		7 (4-9)		11 (6-14)		0 (0-0)		0 (0-0)	
2001	Solomon Islands	12 (9-16)		19 (13-25)		7 (4-10)		11 (6-15)		0 (0-0)		0 (0-0)	
2002	Solomon Islands	13 (9-16)		19 (13-25)		8 (4-10)		12 (6-15)		0 (0-0)		0 (0-0)	
2003	Solomon Islands	13 (9-17)		19 (13-25)		8 (4-10)		12 (6-15)		0 (0-0)		0 (0-0)	
2004	Solomon Islands	13 (9-18)		19 (14-25)		8 (4-11)		12 (6-15)		0 (0-0)		0 (0-0)	
2005	Solomon Islands	14 (10-18)		19 (14-25)		8 (4-11)		12 (6-15)		0 (0-0)		0 (0-0)	
2006	Solomon Islands	14 (10-19)		19 (14-26)		9 (4-11)		12 (6-15)		0 (0-0)		0 (0-0)	
2007	Solomon Islands	14 (10-19)		19 (13-25)		9 (4-11)		11 (6-15)		0 (0-0)		0 (0-0)	
2008	Solomon Islands	2 (2-3)		3 (2-4)		1 (1-2)		2 (1-2)		0 (0-0)		0 (0-0)	
2009	Solomon Islands	1 (1-1)		1 (1-2)		1 (0-1)		1 (0-1)		0 (0-0)		0 (0-0)	
2010	Solomon Islands	1 (1-2)		1 (1-2)		1 (0-1)		1 (0-1)		0 (0-0)		0 (0-0)	
2011	Solomon Islands	1 (1-1)		1 (1-1)		0 (0-1)		1 (0-1)		0 (0-0)		0 (0-0)	
2012	Solomon Islands	1 (1-1)		1 (1-1)		0 (0-1)		1 (0-1)		0 (0-0)		0 (0-0)	
2013	Solomon Islands	0 (0-0)		0 (0-0)		0 (0-0)		0 (0-0)		0 (0-0)		0 (0-0)	
2014	Solomon Islands	0 (0-0)		0 (0-0)		0 (0-0)		0 (0-0)		0 (0-0)		0 (0-0)	
2015	Solomon Islands	0 (0-0)	0 (0-0)	0 (0-0)	0 (0-0)	0 (0-0)	0 (0-0)	0 (0-0)	0 (0-0)	0 (0-0)	0 (0-0)	0 (0-0)	0 (0-0)
2000	Somalia	1668 (1173-2192)		115 (81-152)		834 (351-1270)		58 (24-88)		3 (1-5)		0 (0-0)	
2001	Somalia	1689 (1188-2220)		113 (79-148)		846 (356-1288)		56 (24-86)		3 (1-5)		0 (0-0)	
2002	Somalia	1750 (1231-2300)		114 (80-150)		877 (369-1335)		57 (24-87)		4 (2-5)		0 (0-0)	
2003	Somalia	1868 (1314-2455)		120 (85-158)		936 (394-1425)		60 (25-92)		4 (2-6)		0 (0-0)	
2004	Somalia	1922 (1352-2525)		122 (86-160)		962 (405-1465)		61 (26-93)		4 (2-6)		0 (0-0)	
2005	Somalia	1994 (1402-2620)		125 (88-164)		996 (419-1516)		62 (26-95)		4 (2-6)		0 (0-0)	
2006	Somalia	2070 (1456-2720)		127 (89-167)		1032 (434-1572)		63 (27-97)		4 (2-6)		0 (0-0)	
2007	Somalia	2151 (1513-2826)		129 (91-170)		1071 (451-1631)		64 (27-98)		4 (2-7)		0 (0-0)	
2008	Somalia	2211 (1555-2906)		130 (91-170)		1100 (464-1675)		64 (27-98)		4 (2-7)		0 (0-0)	
2009	Somalia	2250 (1583-2956)		129 (91-169)		1120 (472-1704)		64 (27-97)		5 (2-7)		0 (0-0)	
2010	Somalia	2263 (1592-2974)		127 (89-167)		1126 (475-1713)		63 (27-96)		5 (2-7)		0 (0-0)	
2011	Somalia	2236 (1572-2937)		122 (86-160)		1111 (470-1689)		61 (26-92)		5 (2-7)		0 (0-0)	
2012	Somalia	2186 (1538-2873)		118 (83-155)		1084 (459-1648)		58 (25-89)		4 (2-7)		0 (0-0)	
2013	Somalia	1342 (944-1764)		71 (50-94)		664 (177-1109)		35 (9-59)		3 (1-5)		0 (0-0)	
2014	Somalia	339 (238-445)		18 (13-23)		167 (45-279)		9 (2-15)		1 (0-1)		0 (0-0)	
2015	Somalia	334 (235-439)	3 (2-3)	17 (12-23)	0 (0-0)	165 (44-275)	1 (0-2)	8 (2-14)	0 (0-0)	1 (0-1)	0 (0-0)	0 (0-0)	0 (0-0)
2000	South Africa	544 (383-715)		10 (7-13)		126 (51-196)		2 (1-4)		1 (0-1)		0 (0-0)	
2001	South Africa	243 (171-319)		5 (3-6)		56 (22-87)		1 (0-2)		0 (0-0)		0 (0-0)	
2002	South Africa	235 (165-308)		4 (3-6)		54 (21-83)		1 (0-2)		0 (0-0)		0 (0-0)	
2003	South Africa	221 (156-291)		4 (3-6)		50 (20-78)		1 (0-1)		0 (0-0)		0 (0-0)	
2004	South Africa	221 (156-291)		4 (3-6)		50 (20-77)		1 (0-2)		0 (0-0)		0 (0-0)	
2005	South Africa	17 (12-22)		0 (0-0)		4 (2-6)		0 (0-0)		0 (0-0)		0 (0-0)	
2006	South Africa	17 (12-22)		0 (0-0)		4 (1-6)		0 (0-0)		0 (0-0)		0 (0-0)	

Hib = *Haemophilus influenzae* type b; npnm = non-pneumonia, non-meningitis; HIV = deaths only in HIV-infected children; Uncertainty range provided in parentheses.

Year	Country	Hib pneumonia deaths (UR)	Hib pneumonia deaths, HIV (UR)	Hib pneumonia mortality rate (UR)	Hib pneumonia mortality rate, HIV (UR)	Hib meningitis deaths (UR)	Hib meningitis deaths, HIV (UR)	Hib meningitis mortality rate (UR)	Hib meningitis mortality rate, HIV (UR)	Hib NPNM deaths (UR)	Hib NPNM deaths, HIV (UR)	Hib NPNM mortality rate (UR)	Hib NPNM mortality rate, HIV (UR)
2007	South Africa	18 (12-23)		0 (0-0)		4 (1-6)		0 (0-0)		0 (0-0)		0 (0-0)	
2008	South Africa	19 (13-24)		0 (0-0)		4 (2-6)		0 (0-0)		0 (0-0)		0 (0-0)	
2009	South Africa	19 (13-25)		0 (0-0)		4 (2-6)		0 (0-0)		0 (0-0)		0 (0-0)	
2010	South Africa	19 (14-25)		0 (0-0)		4 (2-7)		0 (0-0)		0 (0-0)		0 (0-0)	
2011	South Africa	19 (13-25)		0 (0-0)		4 (2-6)		0 (0-0)		0 (0-0)		0 (0-0)	
2012	South Africa	18 (13-24)		0 (0-0)		4 (2-6)		0 (0-0)		0 (0-0)		0 (0-0)	
2013	South Africa	212 (149-278)		4 (3-5)		45 (18-71)		1 (0-1)		0 (0-1)		0 (0-0)	
2014	South Africa	16 (11-21)		0 (0-0)		3 (1-5)		0 (0-0)		0 (0-0)		0 (0-0)	
2015	South Africa	15 (11-20)	1 (1-2)	0 (0-0)	0 (0-0)	3 (1-5)	0 (0-0)	0 (0-0)	0 (0-0)	0 (0-0)	0 (0-0)	0 (0-0)	0 (0-0)
2000	South Sudan	1285 (904-1688)		110 (77-144)		453 (213-672)		39 (18-57)		2 (1-3)		0 (0-0)	
2001	South Sudan	1285 (904-1689)		106 (74-139)		450 (212-666)		37 (17-55)		2 (1-3)		0 (0-0)	
2002	South Sudan	1243 (874-1633)		99 (70-130)		431 (205-637)		34 (16-51)		2 (1-3)		0 (0-0)	
2003	South Sudan	1197 (842-1573)		93 (66-123)		411 (196-606)		32 (15-47)		2 (1-2)		0 (0-0)	
2004	South Sudan	1157 (814-1520)		88 (62-116)		393 (127-637)		30 (10-49)		2 (1-3)		0 (0-0)	
2005	South Sudan	1098 (772-1443)		81 (57-107)		370 (121-598)		27 (9-44)		2 (0-2)		0 (0-0)	
2006	South Sudan	1071 (753-1407)		77 (54-101)		356 (117-574)		25 (8-41)		1 (0-2)		0 (0-0)	
2007	South Sudan	1050 (739-1380)		72 (51-95)		346 (115-557)		24 (8-38)		1 (0-2)		0 (0-0)	
2008	South Sudan	1065 (749-1400)		71 (50-93)		350 (117-563)		23 (8-37)		1 (0-2)		0 (0-0)	
2009	South Sudan	1063 (748-1397)		68 (48-89)		351 (118-564)		22 (8-36)		1 (0-2)		0 (0-0)	
2010	South Sudan	1064 (748-1397)		65 (46-86)		357 (121-573)		22 (7-35)		1 (0-2)		0 (0-0)	
2011	South Sudan	1061 (746-1394)		63 (44-82)		365 (125-585)		21 (7-34)		1 (1-2)		0 (0-0)	
2012	South Sudan	1021 (718-1341)		58 (41-76)		359 (123-574)		20 (7-33)		1 (1-2)		0 (0-0)	
2013	South Sudan	1027 (723-1350)		56 (40-74)		368 (127-588)		20 (7-32)		1 (1-2)		0 (0-0)	
2014	South Sudan	1007 (708-1324)		54 (38-70)		366 (127-584)		19 (7-31)		1 (1-2)		0 (0-0)	
2015	South Sudan	755 (531-992)	26 (18-34)	39 (28-52)	1 (1-2)	276 (96-440)	9 (3-15)	14 (5-23)	0 (0-1)	1 (0-2)	0 (0-0)	0 (0-0)	0 (0-0)
2000	Spain	1 (0-1)		0 (0-0)		1 (1-3)		0 (0-0)		0 (0-0)		0 (0-0)	
2001	Spain	0 (0-0)		0 (0-0)		1 (0-1)		0 (0-0)		0 (0-0)		0 (0-0)	
2002	Spain	1 (0-1)		0 (0-0)		1 (0-1)		0 (0-0)		0 (0-0)		0 (0-0)	
2003	Spain	0 (0-0)		0 (0-0)		0 (0-0)		0 (0-0)		0 (0-0)		0 (0-0)	
2004	Spain	0 (0-0)		0 (0-0)		0 (0-0)		0 (0-0)		0 (0-0)		0 (0-0)	
2005	Spain	0 (0-0)		0 (0-0)		0 (0-0)		0 (0-0)		0 (0-0)		0 (0-0)	
2006	Spain	0 (0-0)		0 (0-0)		0 (0-0)		0 (0-0)		0 (0-0)		0 (0-0)	
2007	Spain	0 (0-0)		0 (0-0)		0 (0-0)		0 (0-0)		0 (0-0)		0 (0-0)	
2008	Spain	0 (0-0)		0 (0-0)		0 (0-0)		0 (0-0)		0 (0-0)		0 (0-0)	
2009	Spain	0 (0-0)		0 (0-0)		0 (0-0)		0 (0-0)		0 (0-0)		0 (0-0)	
2010	Spain	0 (0-0)		0 (0-0)		0 (0-0)		0 (0-0)		0 (0-0)		0 (0-0)	
2011	Spain	0 (0-0)		0 (0-0)		0 (0-0)		0 (0-0)		0 (0-0)		0 (0-0)	
2012	Spain	0 (0-0)		0 (0-0)		0 (0-0)		0 (0-0)		0 (0-0)		0 (0-0)	
2013	Spain	0 (0-0)		0 (0-0)		0 (0-0)		0 (0-0)		0 (0-0)		0 (0-0)	
2014	Spain	0 (0-0)		0 (0-0)		0 (0-0)		0 (0-0)		0 (0-0)		0 (0-0)	
2015	Spain	0 (0-0)	0 (0-0)	0 (0-0)	0 (0-0)	0 (0-0)	0 (0-0)	0 (0-0)	0 (0-0)	0 (0-0)	0 (0-0)	0 (0-0)	0 (0-0)
2000	Sri Lanka	64 (45-84)		4 (3-5)		18 (2-31)		1 (0-2)		0 (0-0)		0 (0-0)	
2001	Sri Lanka	60 (42-79)		4 (3-5)		17 (2-30)		1 (0-2)		0 (0-0)		0 (0-0)	
2002	Sri Lanka	57 (40-75)		3 (2-5)		16 (2-28)		1 (0-2)		0 (0-0)		0 (0-0)	
2003	Sri Lanka	56 (39-74)		3 (2-4)		16 (2-28)		1 (0-2)		0 (0-0)		0 (0-0)	
2004	Sri Lanka	41 (29-54)		2 (2-3)		12 (1-21)		1 (0-1)		0 (0-0)		0 (0-0)	
2005	Sri Lanka	51 (36-67)		3 (2-4)		15 (2-25)		1 (0-1)		0 (0-0)		0 (0-0)	
2006	Sri Lanka	46 (32-61)		3 (2-3)		14 (2-23)		1 (0-1)		0 (0-0)		0 (0-0)	
2007	Sri Lanka	42 (30-55)		2 (2-3)		12 (2-21)		1 (0-1)		0 (0-0)		0 (0-0)	
2008	Sri Lanka	39 (28-51)		2 (2-3)		11 (1-20)		1 (0-1)		0 (0-0)		0 (0-0)	
2009	Sri Lanka	36 (26-48)		2 (1-3)		11 (1-18)		1 (0-1)		0 (0-0)		0 (0-0)	
2010	Sri Lanka	6 (4-8)		0 (0-0)		3 (0-5)		0 (0-0)		0 (0-0)		0 (0-0)	
2011	Sri Lanka	2 (2-3)		0 (0-0)		1 (0-2)		0 (0-0)		0 (0-0)		0 (0-0)	
2012	Sri Lanka	2 (2-3)		0 (0-0)		1 (0-2)		0 (0-0)		0 (0-0)		0 (0-0)	
2013	Sri Lanka	2 (1-3)		0 (0-0)		1 (0-2)		0 (0-0)		0 (0-0)		0 (0-0)	
2014	Sri Lanka	2 (1-3)		0 (0-0)		1 (0-2)		0 (0-0)		0 (0-0)		0 (0-0)	
2015	Sri Lanka	0 (0-0)	0 (0-0)	0 (0-0)	0 (0-0)	0 (0-0)	0 (0-0)	0 (0-0)	0 (0-0)	0 (0-0)	0 (0-0)	0 (0-0)	0 (0-0)
2000	Sudan	3198 (2249-4202)		68 (48-89)		1091 (480-1651)		23 (10-35)		4 (2-7)		0 (0-0)	
2001	Sudan	3285 (2311-4317)		68 (48-90)		1115 (481-1697)		23 (10-35)		5 (2-7)		0 (0-0)	
2002	Sudan	3280 (2307-4310)		67 (47-88)		1107 (468-1694)		22 (10-34)		5 (2-7)		0 (0-0)	
2003	Sudan	3284 (2310-4315)		65 (46-86)		1101 (456-1694)		22 (9-34)		4 (2-7)		0 (0-0)	
2004	Sudan	3261 (2294-4285)		63 (45-83)		1086 (441-1678)		21 (9-33)		4 (2-7)		0 (0-0)	
2005	Sudan	3141 (2209-4127)		60 (42-79)		1038 (414-1611)		20 (8-31)		4 (2-7)		0 (0-0)	
2006	Sudan	3090 (2173-4059)		58 (41-76)		1013 (396-1579)		19 (7-30)		4 (2-6)		0 (0-0)	
2007	Sudan	3014 (2120-3961)		56 (39-73)		982 (378-1537)		18 (7-28)		4 (2-6)		0 (0-0)	
2008	Sudan	576 (405-757)		11 (7-14)		187 (71-294)		3 (1-5)		1 (0-1)		0 (0-0)	
2009	Sudan	328 (231-431)		6 (4-8)		106 (39-167)		2 (1-3)		0 (0-1)		0 (0-0)	
2010	Sudan	304 (214-400)		5 (4-7)		99 (36-156)		2 (1-3)		0 (0-1)		0 (0-0)	
2011	Sudan	214 (151-282)		4 (3-5)		70 (25-110)		1 (0-2)		0 (0-0)		0 (0-0)	
2012	Sudan	212 (149-279)		4 (3-5)		69 (24-110)		1 (0-2)		0 (0-0)		0 (0-0)	
2013	Sudan	27 (19-36)		0 (0-1)		9 (3-15)		0 (0-0)		0 (0-0)		0 (0-0)	
2014	Sudan	27 (19-35)		0 (0-1)		9 (3-15)		0 (0-0)		0 (0-0)		0 (0-0)	
2015	Sudan	26 (19-35)	0 (0-0)	0 (0-1)	0 (0-0)	9 (3-14)	0 (0-0)	0 (0-0)	0 (0-0)	0 (0-0)	0 (0-0)	0 (0-0)	0 (0-0)
2000	Suriname	4 (2-5)		7 (5-9)		3 (1-5)		6 (2-10)		0 (0-0)		0 (0-0)	
2001	Suriname	4 (3-5)		7 (5-9)		2 (1-4)		4 (1-7)		0 (0-0)		0 (0-0)	
2002	Suriname	2 (1-3)		4 (3-5)		5 (2-9)		11 (3-17)		0 (0-0)		0 (0-0)	
2003	Suriname	6 (4-7)		11 (8-14)		1 (0-2)		3 (1-4)		0 (0-0)		0 (0-0)	
2004	Suriname	2 (2-3)		4 (3-6)		2 (1-4)		5 (2-8)		0 (0-0)		0 (0-0)	
2005	Suriname	1 (1-1)		2 (1-2)		0 (0-0)		0 (0-1)		0 (0-0)		0 (0-0)	
2006	Suriname	0 (0-0)		1 (0-1)		0 (0-1)		1 (0-1)		0 (0-0)		0 (0-0)	
2007	Suriname	0 (0-1)		1 (1-1)		0 (0-0)		0 (0-0)		0 (0-0)		0 (0-0)	
2008	Suriname	0 (0-0)		1 (0-1)		0 (0-1)		1 (0-1)		0 (0-0)		0 (0-0)	
2009	Suriname	0 (0-1)		1 (1-1)		0 (0-1)		1 (0-1)		0 (0-0)		0 (0-0)	
2010	Suriname	0 (0-0)		0 (0-0)		0 (0-0)		0 (0-0)		0 (0-0)		0 (0-0)	
2011	Suriname	0 (0-0)		0 (0-0)		0 (0-0)		0 (0-0)		0 (0-0)		0 (0-0)	
2012	Suriname	0 (0-0)		0 (0-0)		0 (0-0)		0 (0-0)		0 (0-0)		0 (0-0)	
2013	Suriname	0 (0-0)		0 (0-0)		0 (0-0)		0 (0-0)		0 (0-0)		0 (0-0)	
2014	Suriname	0 (0-0)		0 (0-0)		0 (0-0)		0 (0-0)		0 (0-0)		0 (0-0)	

Hib = *Haemophilus influenzae* type b; npnm = non-pneumonia, non-meningitis; HIV = deaths only in HIV-infected children; Uncertainty range provided in parentheses.

Year	Country	Hib pneumonia deaths (UR)	Hib pneumonia deaths, HIV (UR)	Hib pneumonia mortality rate (UR)	Hib pneumonia mortality rate, HIV (UR)	Hib meningitis deaths (UR)	Hib meningitis deaths, HIV (UR)	Hib meningitis mortality rate (UR)	Hib meningitis mortality rate, HIV (UR)	Hib NPNM deaths (UR)	Hib NPNM deaths, HIV (UR)	Hib NPNM mortality rate (UR)	Hib NPNM mortality rate, HIV (UR)
2015	Suriname	0 (0-0)	0 (0-0)	0 (0-0)	0 (0-0)	0 (0-0)	0 (0-0)	0 (0-0)	0 (0-0)	0 (0-0)	0 (0-0)	0 (0-0)	0 (0-0)
2000	Swaziland	102 (72-134)		66 (46-87)		27 (10-43)		18 (7-28)		0 (0-0)		0 (0-0)	
2001	Swaziland	105 (74-138)		68 (48-90)		28 (11-44)		18 (7-29)		0 (0-0)		0 (0-0)	
2002	Swaziland	107 (75-140)		70 (49-92)		29 (11-45)		19 (7-29)		0 (0-0)		0 (0-0)	
2003	Swaziland	108 (76-142)		71 (50-93)		29 (12-45)		19 (7-29)		0 (0-0)		0 (0-0)	
2004	Swaziland	108 (76-142)		71 (50-93)		29 (12-44)		19 (8-29)		0 (0-0)		0 (0-0)	
2005	Swaziland	108 (76-141)		70 (50-93)		28 (12-43)		19 (8-28)		0 (0-0)		0 (0-0)	
2006	Swaziland	104 (73-137)		68 (48-89)		27 (12-41)		18 (8-27)		0 (0-0)		0 (0-0)	
2007	Swaziland	102 (72-134)		66 (46-87)		27 (12-41)		17 (7-26)		0 (0-0)		0 (0-0)	
2008	Swaziland	100 (70-131)		63 (45-83)		26 (11-41)		17 (7-26)		0 (0-0)		0 (0-0)	
2009	Swaziland	15 (10-20)		9 (7-12)		4 (2-6)		2 (1-4)		0 (0-0)		0 (0-0)	
2010	Swaziland	8 (5-10)		5 (3-6)		2 (1-3)		1 (0-2)		0 (0-0)		0 (0-0)	
2011	Swaziland	7 (5-9)		4 (3-5)		2 (1-3)		1 (0-2)		0 (0-0)		0 (0-0)	
2012	Swaziland	6 (4-8)		4 (3-5)		2 (1-3)		1 (0-2)		0 (0-0)		0 (0-0)	
2013	Swaziland	6 (4-7)		3 (2-4)		1 (1-2)		1 (0-1)		0 (0-0)		0 (0-0)	
2014	Swaziland	1 (1-1)		0 (0-1)		0 (0-0)		0 (0-0)		0 (0-0)		0 (0-0)	
2015	Swaziland	1 (1-1)	0 (0-0)	0 (0-1)	0 (0-0)	0 (0-0)	0 (0-0)	0 (0-0)	0 (0-0)	0 (0-0)	0 (0-0)	0 (0-0)	0 (0-0)
2000	Sweden	0 (0-0)		0 (0-0)		0 (0-0)		0 (0-0)		0 (0-0)		0 (0-0)	
2001	Sweden	0 (0-0)		0 (0-0)		0 (0-0)		0 (0-0)		0 (0-0)		0 (0-0)	
2002	Sweden	0 (0-0)		0 (0-0)		0 (0-0)		0 (0-0)		0 (0-0)		0 (0-0)	
2003	Sweden	0 (0-0)		0 (0-0)		0 (0-0)		0 (0-0)		0 (0-0)		0 (0-0)	
2004	Sweden	0 (0-0)		0 (0-0)		0 (0-0)		0 (0-0)		0 (0-0)		0 (0-0)	
2005	Sweden	0 (0-0)		0 (0-0)		0 (0-0)		0 (0-0)		0 (0-0)		0 (0-0)	
2006	Sweden	0 (0-0)		0 (0-0)		0 (0-0)		0 (0-0)		0 (0-0)		0 (0-0)	
2007	Sweden	0 (0-0)		0 (0-0)		0 (0-0)		0 (0-0)		0 (0-0)		0 (0-0)	
2008	Sweden	0 (0-0)		0 (0-0)		0 (0-0)		0 (0-0)		0 (0-0)		0 (0-0)	
2009	Sweden	0 (0-0)		0 (0-0)		0 (0-0)		0 (0-0)		0 (0-0)		0 (0-0)	
2010	Sweden	0 (0-0)		0 (0-0)		0 (0-0)		0 (0-0)		0 (0-0)		0 (0-0)	
2011	Sweden	0 (0-0)		0 (0-0)		0 (0-0)		0 (0-0)		0 (0-0)		0 (0-0)	
2012	Sweden	0 (0-0)		0 (0-0)		0 (0-0)		0 (0-0)		0 (0-0)		0 (0-0)	
2013	Sweden	0 (0-0)		0 (0-0)		0 (0-0)		0 (0-0)		0 (0-0)		0 (0-0)	
2014	Sweden	0 (0-0)		0 (0-0)		0 (0-0)		0 (0-0)		0 (0-0)		0 (0-0)	
2015	Sweden	0 (0-0)	0 (0-0)	0 (0-0)	0 (0-0)	0 (0-0)	0 (0-0)	0 (0-0)	0 (0-0)	0 (0-0)	0 (0-0)	0 (0-0)	0 (0-0)
2000	Switzerland	0 (0-0)		0 (0-0)		0 (0-0)		0 (0-0)		0 (0-0)		0 (0-0)	
2001	Switzerland	0 (0-0)		0 (0-0)		0 (0-0)		0 (0-0)		0 (0-0)		0 (0-0)	
2002	Switzerland	0 (0-0)		0 (0-0)		0 (0-0)		0 (0-0)		0 (0-0)		0 (0-0)	
2003	Switzerland	0 (0-0)		0 (0-0)		0 (0-0)		0 (0-0)		0 (0-0)		0 (0-0)	
2004	Switzerland	0 (0-0)		0 (0-0)		0 (0-0)		0 (0-0)		0 (0-0)		0 (0-0)	
2005	Switzerland	0 (0-0)		0 (0-0)		0 (0-0)		0 (0-0)		0 (0-0)		0 (0-0)	
2006	Switzerland	0 (0-0)		0 (0-0)		0 (0-0)		0 (0-0)		0 (0-0)		0 (0-0)	
2007	Switzerland	0 (0-0)		0 (0-0)		0 (0-0)		0 (0-0)		0 (0-0)		0 (0-0)	
2008	Switzerland	0 (0-0)		0 (0-0)		0 (0-0)		0 (0-0)		0 (0-0)		0 (0-0)	
2009	Switzerland	0 (0-0)		0 (0-0)		0 (0-0)		0 (0-0)		0 (0-0)		0 (0-0)	
2010	Switzerland	0 (0-0)		0 (0-0)		0 (0-0)		0 (0-0)		0 (0-0)		0 (0-0)	
2011	Switzerland	0 (0-0)		0 (0-0)		0 (0-0)		0 (0-0)		0 (0-0)		0 (0-0)	
2012	Switzerland	0 (0-0)		0 (0-0)		0 (0-0)		0 (0-0)		0 (0-0)		0 (0-0)	
2013	Switzerland	0 (0-0)		0 (0-0)		0 (0-0)		0 (0-0)		0 (0-0)		0 (0-0)	
2014	Switzerland	0 (0-0)		0 (0-0)		0 (0-0)		0 (0-0)		0 (0-0)		0 (0-0)	
2015	Switzerland	0 (0-0)	0 (0-0)	0 (0-0)	0 (0-0)	0 (0-0)	0 (0-0)	0 (0-0)	0 (0-0)	0 (0-0)	0 (0-0)	0 (0-0)	0 (0-0)
2000	Syrian Arab Republic	250 (176-329)		11 (7-14)		26 (4-46)		1 (0-2)		0 (0-0)		0 (0-0)	
2001	Syrian Arab Republic	41 (29-54)		2 (1-2)		8 (1-13)		0 (0-1)		0 (0-0)		0 (0-0)	
2002	Syrian Arab Republic	21 (15-28)		1 (1-1)		4 (1-7)		0 (0-0)		0 (0-0)		0 (0-0)	
2003	Syrian Arab Republic	19 (14-26)		1 (1-1)		4 (1-6)		0 (0-0)		0 (0-0)		0 (0-0)	
2004	Syrian Arab Republic	18 (13-24)		1 (1-1)		3 (1-6)		0 (0-0)		0 (0-0)		0 (0-0)	
2005	Syrian Arab Republic	17 (12-22)		1 (0-1)		3 (1-5)		0 (0-0)		0 (0-0)		0 (0-0)	
2006	Syrian Arab Republic	2 (1-2)		0 (0-0)		0 (0-1)		0 (0-0)		0 (0-0)		0 (0-0)	
2007	Syrian Arab Republic	2 (1-2)		0 (0-0)		0 (0-1)		0 (0-0)		0 (0-0)		0 (0-0)	
2008	Syrian Arab Republic	2 (1-2)		0 (0-0)		0 (0-0)		0 (0-0)		0 (0-0)		0 (0-0)	
2009	Syrian Arab Republic	2 (1-2)		0 (0-0)		0 (0-0)		0 (0-0)		0 (0-0)		0 (0-0)	
2010	Syrian Arab Republic	1 (1-2)		0 (0-0)		0 (0-0)		0 (0-0)		0 (0-0)		0 (0-0)	
2011	Syrian Arab Republic	1 (1-2)		0 (0-0)		0 (0-0)		0 (0-0)		0 (0-0)		0 (0-0)	
2012	Syrian Arab Republic	14 (10-19)		1 (0-1)		2 (1-3)		0 (0-0)		0 (0-0)		0 (0-0)	
2013	Syrian Arab Republic	12 (8-16)		1 (0-1)		1 (1-2)		0 (0-0)		0 (0-0)		0 (0-0)	
2014	Syrian Arab Republic	10 (7-14)		0 (0-1)		1 (1-2)		0 (0-0)		0 (0-0)		0 (0-0)	
2015	Syrian Arab Republic	11 (7-14)	0 (0-0)	0 (0-1)	0 (0-0)	1 (0-2)	0 (0-0)	0 (0-0)	0 (0-0)	0 (0-0)	0 (0-0)	0 (0-0)	0 (0-0)
2000	Tajikistan	658 (463-864)		75 (53-99)		301 (105-475)		35 (12-54)		1 (0-2)		0 (0-0)	
2001	Tajikistan	595 (419-782)		68 (48-90)		268 (95-420)		31 (11-48)		1 (0-2)		0 (0-0)	
2002	Tajikistan	531 (374-698)		62 (43-81)		234 (85-365)		27 (10-42)		1 (0-1)		0 (0-0)	
2003	Tajikistan	473 (333-621)		56 (39-73)		204 (69-326)		24 (8-38)		2 (1-3)		0 (0-0)	
2004	Tajikistan	431 (303-566)		51 (36-67)		181 (63-288)		22 (7-34)		1 (1-2)		0 (0-0)	
2005	Tajikistan	402 (283-528)		47 (33-62)		165 (59-261)		20 (7-31)		1 (0-2)		0 (0-0)	
2006	Tajikistan	384 (270-504)		45 (32-59)		155 (55-245)		18 (7-29)		1 (0-2)		0 (0-0)	
2007	Tajikistan	377 (265-495)		43 (31-57)		149 (53-237)		17 (6-27)		1 (0-2)		0 (0-0)	
2008	Tajikistan	218 (154-287)		24 (17-32)		86 (31-136)		10 (3-15)		1 (0-1)		0 (0-0)	
2009	Tajikistan	29 (21-38)		3 (2-4)		11 (4-18)		1 (0-2)		0 (0-0)		0 (0-0)	
2010	Tajikistan	30 (21-39)		3 (2-4)		11 (4-18)		1 (0-2)		0 (0-0)		0 (0-0)	
2011	Tajikistan	28 (20-37)		3 (2-4)		11 (4-17)		1 (0-2)		0 (0-0)		0 (0-0)	
2012	Tajikistan	30 (21-39)		3 (2-4)		11 (4-18)		1 (0-2)		0 (0-0)		0 (0-0)	
2013	Tajikistan	4 (3-5)		0 (0-0)		1 (1-2)		0 (0-0)		0 (0-0)		0 (0-0)	
2014	Tajikistan	4 (3-5)		0 (0-0)		1 (1-2)		0 (0-0)		0 (0-0)		0 (0-0)	
2015	Tajikistan	4 (3-5)	0 (0-0)	0 (0-0)	0 (0-0)	1 (1-2)	0 (0-0)	0 (0-0)	0 (0-0)	0 (0-0)	0 (0-0)	0 (0-0)	0 (0-0)
2000	Thailand	323 (227-424)		7 (5-9)		75 (17-123)		2 (0-3)		1 (0-1)		0 (0-0)	
2001	Thailand	297 (209-390)		7 (5-9)		70 (16-114)		2 (0-3)		1 (0-1)		0 (0-0)	
2002	Thailand	277 (195-363)		6 (4-8)		65 (15-107)		1 (0-2)		1 (0-1)		0 (0-0)	
2003	Thailand	258 (181-339)		6 (4-8)		61 (14-101)		1 (0-2)		0 (0-1)		0 (0-0)	
2004	Thailand	239 (168-314)		5 (4-7)		57 (13-94)		1 (0-2)		0 (0-1)		0 (0-0)	
2005	Thailand	218 (154-287)		5 (3-7)		53 (12-86)		1 (0-2)		0 (0-1)		0 (0-0)	
2006	Thailand	200 (141-263)		5 (3-6)		49 (11-80)		1 (0-2)		0 (0-1)		0 (0-0)	

Hib = *Haemophilus influenzae* type b; npnm = non-pneumonia, non-meningitis; HIV = deaths only in HIV-infected children; Uncertainty range provided in parentheses.

Year	Country	Hib pneumonia deaths (UR)	Hib pneumonia deaths, HIV (UR)	Hib pneumonia mortality rate (UR)	Hib pneumonia mortality rate, HIV (UR)	Hib meningitis deaths (UR)	Hib meningitis deaths, HIV (UR)	Hib meningitis mortality rate (UR)	Hib meningitis mortality rate, HIV (UR)	Hib NPNM deaths (UR)	Hib NPNM deaths, HIV (UR)	Hib NPNM mortality rate (UR)	Hib NPNM mortality rate, HIV (UR)
2007	Thailand	184 (129-242)		4 (3-6)		45 (11-74)		1 (0-2)		0 (0-1)		0 (0-0)	
2008	Thailand	166 (117-219)		4 (3-5)		41 (10-67)		1 (0-2)		0 (0-1)		0 (0-0)	
2009	Thailand	157 (110-206)		4 (3-5)		39 (9-63)		1 (0-2)		0 (0-1)		0 (0-0)	
2010	Thailand	149 (105-195)		4 (3-5)		37 (8-60)		1 (0-2)		0 (0-0)		0 (0-0)	
2011	Thailand	139 (98-183)		4 (3-5)		34 (8-56)		1 (0-1)		0 (0-0)		0 (0-0)	
2012	Thailand	131 (92-173)		3 (2-4)		32 (7-52)		1 (0-1)		0 (0-0)		0 (0-0)	
2013	Thailand	124 (87-162)		3 (2-4)		30 (7-49)		1 (0-1)		0 (0-0)		0 (0-0)	
2014	Thailand	118 (83-155)		3 (2-4)		28 (7-46)		1 (0-1)		0 (0-0)		0 (0-0)	
2015	Thailand	113 (80-149)	0 (0-0)	3 (2-4)	0 (0-0)	27 (6-43)	0 (0-0)	1 (0-1)	0 (0-0)	0 (0-0)	0 (0-0)	0 (0-0)	0 (0-0)
2000	The former Yugoslav Republic of Macedonia	6 (4-8)		4 (3-6)		1 (0-2)		1 (0-1)		0 (0-0)		0 (0-0)	
2001	The former Yugoslav Republic of Macedonia	7 (5-9)		5 (3-7)		2 (1-4)		2 (0-3)		0 (0-0)		0 (0-0)	
2002	The former Yugoslav Republic of Macedonia	5 (4-7)		4 (3-5)		2 (1-4)		1 (0-3)		0 (0-0)		0 (0-0)	
2003	The former Yugoslav Republic of Macedonia	5 (4-7)		4 (3-5)		2 (1-3)		1 (0-3)		0 (0-0)		0 (0-0)	
2004	The former Yugoslav Republic of Macedonia	4 (3-6)		4 (3-5)		2 (1-3)		1 (0-3)		0 (0-0)		0 (0-0)	
2005	The former Yugoslav Republic of Macedonia	4 (3-5)		3 (2-4)		1 (0-1)		0 (0-1)		0 (0-0)		0 (0-0)	
2006	The former Yugoslav Republic of Macedonia	4 (3-5)		3 (2-4)		1 (0-2)		1 (0-1)		0 (0-0)		0 (0-0)	
2007	The former Yugoslav Republic of Macedonia	3 (2-4)		2 (2-3)		3 (1-6)		3 (1-5)		0 (0-0)		0 (0-0)	
2008	The former Yugoslav Republic of Macedonia	1 (1-2)		1 (1-2)		1 (0-2)		1 (0-2)		0 (0-0)		0 (0-0)	
2009	The former Yugoslav Republic of Macedonia	0 (0-0)		0 (0-0)		0 (0-0)		0 (0-0)		0 (0-0)		0 (0-0)	
2010	The former Yugoslav Republic of Macedonia	0 (0-0)		0 (0-0)		0 (0-0)		0 (0-0)		0 (0-0)		0 (0-0)	
2011	The former Yugoslav Republic of Macedonia	0 (0-0)		0 (0-0)		0 (0-0)		0 (0-0)		0 (0-0)		0 (0-0)	
2012	The former Yugoslav Republic of Macedonia	0 (0-0)		0 (0-0)		0 (0-0)		0 (0-0)		0 (0-0)		0 (0-0)	
2013	The former Yugoslav Republic of Macedonia	0 (0-0)		0 (0-0)		0 (0-0)		0 (0-0)		0 (0-0)		0 (0-0)	
2014	The former Yugoslav Republic of Macedonia	0 (0-0)		0 (0-0)		0 (0-0)		0 (0-0)		0 (0-0)		0 (0-0)	
2015	The former Yugoslav Republic of Macedonia	0 (0-0)	0 (0-0)	0 (0-0)	0 (0-0)	0 (0-0)	0 (0-0)	0 (0-0)	0 (0-0)	0 (0-0)	0 (0-0)	0 (0-0)	0 (0-0)
2000	Timor-Leste	156 (110-205)		91 (64-120)		53 (19-83)		31 (11-48)		0 (0-0)		0 (0-0)	
2001	Timor-Leste	144 (101-189)		84 (59-110)		51 (19-80)		30 (11-47)		0 (0-0)		0 (0-0)	
2002	Timor-Leste	133 (93-174)		77 (54-102)		49 (18-76)		28 (10-44)		0 (0-0)		0 (0-0)	
2003	Timor-Leste	122 (86-160)		72 (50-94)		45 (17-70)		26 (10-41)		0 (0-0)		0 (0-0)	
2004	Timor-Leste	111 (78-146)		66 (46-87)		40 (15-61)		24 (9-37)		0 (0-0)		0 (0-0)	
2005	Timor-Leste	100 (70-132)		61 (43-80)		34 (13-52)		21 (8-32)		0 (0-0)		0 (0-0)	
2006	Timor-Leste	90 (63-118)		57 (40-75)		28 (11-44)		18 (7-28)		0 (0-0)		0 (0-0)	
2007	Timor-Leste	80 (56-105)		53 (37-70)		24 (9-37)		16 (6-25)		0 (0-0)		0 (0-0)	
2008	Timor-Leste	73 (51-96)		49 (35-65)		20 (8-32)		14 (5-22)		0 (0-0)		0 (0-0)	
2009	Timor-Leste	68 (48-90)		47 (33-61)		19 (7-29)		13 (5-20)		0 (0-0)		0 (0-0)	
2010	Timor-Leste	69 (49-91)		46 (32-61)		18 (7-29)		12 (5-19)		0 (0-0)		0 (0-0)	
2011	Timor-Leste	72 (51-95)		48 (34-63)		19 (7-29)		13 (5-20)		0 (0-0)		0 (0-0)	
2012	Timor-Leste	79 (56-104)		49 (35-65)		21 (8-32)		13 (5-20)		0 (0-0)		0 (0-0)	
2013	Timor-Leste	14 (10-19)		8 (6-11)		4 (1-6)		2 (1-3)		0 (0-0)		0 (0-0)	
2014	Timor-Leste	9 (7-12)		5 (3-6)		2 (1-4)		1 (1-2)		0 (0-0)		0 (0-0)	
2015	Timor-Leste	10 (7-13)	0 (0-0)	5 (3-6)	0 (0-0)	3 (1-4)	0 (0-0)	1 (1-2)	0 (0-0)	0 (0-0)	0 (0-0)	0 (0-0)	0 (0-0)
2000	Togo	364 (256-478)		45 (32-60)		161 (48-265)		20 (6-33)		1 (0-1)		0 (0-0)	
2001	Togo	370 (260-486)		45 (32-59)		165 (48-272)		20 (6-33)		1 (0-1)		0 (0-0)	
2002	Togo	373 (263-491)		44 (31-58)		168 (49-277)		20 (6-33)		1 (0-1)		0 (0-0)	
2003	Togo	374 (263-491)		43 (30-56)		169 (49-279)		19 (6-32)		1 (0-1)		0 (0-0)	
2004	Togo	373 (262-490)		42 (29-55)		170 (49-280)		19 (5-31)		1 (0-1)		0 (0-0)	
2005	Togo	370 (260-486)		40 (28-53)		169 (48-279)		18 (5-30)		1 (0-1)		0 (0-0)	
2006	Togo	367 (258-482)		39 (27-51)		167 (47-277)		18 (5-29)		1 (0-1)		0 (0-0)	
2007	Togo	367 (258-483)		38 (26-49)		167 (48-276)		17 (5-28)		1 (0-1)		0 (0-0)	
2008	Togo	318 (224-418)		32 (22-42)		144 (42-238)		14 (4-24)		1 (0-1)		0 (0-0)	
2009	Togo	40 (28-52)		4 (3-5)		18 (5-30)		2 (1-3)		0 (0-0)		0 (0-0)	
2010	Togo	39 (27-51)		4 (3-5)		17 (5-28)		2 (0-3)		0 (0-0)		0 (0-0)	
2011	Togo	40 (28-53)		4 (3-5)		18 (6-29)		2 (1-3)		0 (0-0)		0 (0-0)	
2012	Togo	44 (31-57)		4 (3-5)		19 (6-31)		2 (1-3)		0 (0-0)		0 (0-0)	
2013	Togo	5 (3-6)		0 (0-1)		2 (1-3)		0 (0-0)		0 (0-0)		0 (0-0)	
2014	Togo	5 (4-7)		0 (0-1)		2 (1-4)		0 (0-0)		0 (0-0)		0 (0-0)	
2015	Togo	5 (4-7)	0 (0-0)	0 (0-1)	0 (0-0)	2 (1-4)	0 (0-0)	0 (0-0)	0 (0-0)	0 (0-0)	0 (0-0)	0 (0-0)	0 (0-0)
2000	Tonga	1 (1-1)		7 (5-9)		0 (0-0)		3 (1-4)		0 (0-0)		0 (0-0)	
2001	Tonga	1 (1-1)		7 (5-9)		0 (0-0)		2 (1-4)		0 (0-0)		0 (0-0)	
2002	Tonga	1 (1-1)		7 (5-9)		0 (0-0)		2 (1-3)		0 (0-0)		0 (0-0)	
2003	Tonga	1 (1-1)		6 (5-8)		0 (0-0)		2 (1-3)		0 (0-0)		0 (0-0)	
2004	Tonga	1 (1-1)		7 (5-9)		0 (0-0)		2 (1-3)		0 (0-0)		0 (0-0)	
2005	Tonga	0 (0-0)		1 (1-2)		0 (0-0)		1 (0-1)		0 (0-0)		0 (0-0)	
2006	Tonga	0 (0-0)		1 (0-1)		0 (0-0)		0 (0-1)		0 (0-0)		0 (0-0)	
2007	Tonga	0 (0-0)		1 (0-1)		0 (0-0)		0 (0-1)		0 (0-0)		0 (0-0)	
2008	Tonga	0 (0-0)		1 (0-1)		0 (0-0)		0 (0-1)		0 (0-0)		0 (0-0)	
2009	Tonga	0 (0-0)		1 (1-1)		0 (0-0)		1 (0-1)		0 (0-0)		0 (0-0)	
2010	Tonga	0 (0-0)		0 (0-0)		0 (0-0)		0 (0-0)		0 (0-0)		0 (0-0)	
2011	Tonga	0 (0-0)		0 (0-0)		0 (0-0)		0 (0-0)		0 (0-0)		0 (0-0)	
2012	Tonga	0 (0-0)		0 (0-0)		0 (0-0)		0 (0-0)		0 (0-0)		0 (0-0)	
2013	Tonga	0 (0-0)		0 (0-0)		0 (0-0)		0 (0-0)		0 (0-0)		0 (0-0)	
2014	Tonga	0 (0-0)		0 (0-0)		0 (0-0)		0 (0-0)		0 (0-0)		0 (0-0)	
2015	Tonga	0 (0-0)	0 (0-0)	0 (0-0)	0 (0-0)	0 (0-0)	0 (0-0)	0 (0-0)	0 (0-0)	0 (0-0)	0 (0-0)	0 (0-0)	0 (0-0)
2000	Trinidad and Tobago	0 (0-0)		0 (0-0)		0 (0-1)		0 (0-1)		0 (0-0)		0 (0-0)	
2001	Trinidad and Tobago	0 (0-0)		0 (0-0)		0 (0-0)		0 (0-0)		0 (0-0)		0 (0-0)	
2002	Trinidad and Tobago	0 (0-1)		1 (0-1)		0 (0-1)		0 (0-1)		0 (0-0)		0 (0-0)	
2003	Trinidad and Tobago	0 (0-0)		0 (0-0)		0 (0-0)		0 (0-0)		0 (0-0)		0 (0-0)	
2004	Trinidad and Tobago	0 (0-0)		0 (0-0)		0 (0-0)		0 (0-0)		0 (0-0)		0 (0-0)	
2005	Trinidad and Tobago	0 (0-0)		0 (0-0)		0 (0-0)		0 (0-0)		0 (0-0)		0 (0-0)	
2006	Trinidad and Tobago	0 (0-0)		0 (0-0)		0 (0-0)		0 (0-0)		0 (0-0)		0 (0-0)	
2007	Trinidad and Tobago	0 (0-0)		0 (0-0)		0 (0-0)		0 (0-0)		0 (0-0)		0 (0-0)	
2008	Trinidad and Tobago	0 (0-0)		0 (0-0)		0 (0-0)		0 (0-0)		0 (0-0)		0 (0-0)	
2009	Trinidad and Tobago	0 (0-0)		0 (0-0)		0 (0-0)		0 (0-0)		0 (0-0)		0 (0-0)	
2010	Trinidad and Tobago	0 (0-0)		0 (0-0)		0 (0-0)		0 (0-0)		0 (0-0)		0 (0-0)	
2011	Trinidad and Tobago	0 (0-0)		0 (0-0)		0 (0-0)		0 (0-0)		0 (0-0)		0 (0-0)	
2012	Trinidad and Tobago	0 (0-0)		0 (0-0)		0 (0-0)		0 (0-0)		0 (0-0)		0 (0-0)	
2013	Trinidad and Tobago	0 (0-0)		0 (0-0)		0 (0-0)		0 (0-0)		0 (0-0)		0 (0-0)	
2014	Trinidad and Tobago	0 (0-0)		0 (0-0)		0 (0-0)		0 (0-0)		0 (0-0)		0 (0-0)	

Hib = *Haemophilus influenzae* type b; npnm = non-pneumonia, non-meningitis; HIV = deaths only in HIV-infected children; Uncertainty range provided in parentheses.

Year	Country	Hib pneumonia deaths (UR)	Hib pneumonia deaths, HIV (UR)	Hib pneumonia mortality rate (UR)	Hib pneumonia mortality rate, HIV (UR)	Hib meningitis deaths (UR)	Hib meningitis deaths, HIV (UR)	Hib meningitis mortality rate (UR)	Hib meningitis mortality rate, HIV (UR)	Hib NPNM deaths (UR)	Hib NPNM deaths, HIV (UR)	Hib NPNM mortality rate (UR)	Hib NPNM mortality rate, HIV (UR)
2015	Trinidad and Tobago	0 (0-0)	0 (0-0)	0 (0-0)	0 (0-0)	0 (0-0)	0 (0-0)	0 (0-0)	0 (0-0)	0 (0-0)	0 (0-0)	0 (0-0)	0 (0-0)
2000	Tunisia	101 (71-133)		12 (9-16)		13 (4-21)		2 (0-3)		0 (0-0)		0 (0-0)	
2001	Tunisia	90 (64-119)		11 (8-15)		11 (1-20)		1 (0-2)		0 (0-0)		0 (0-0)	
2002	Tunisia	82 (57-107)		10 (7-13)		10 (1-18)		1 (0-2)		0 (0-0)		0 (0-0)	
2003	Tunisia	12 (9-16)		2 (1-2)		3 (0-5)		0 (0-1)		0 (0-0)		0 (0-0)	
2004	Tunisia	5 (4-7)		1 (0-1)		1 (0-2)		0 (0-0)		0 (0-0)		0 (0-0)	
2005	Tunisia	7 (5-9)		1 (1-1)		2 (0-3)		0 (0-0)		0 (0-0)		0 (0-0)	
2006	Tunisia	57 (40-74)		7 (5-9)		7 (1-13)		1 (0-2)		0 (0-0)		0 (0-0)	
2007	Tunisia	52 (37-69)		7 (5-9)		7 (1-12)		1 (0-1)		0 (0-0)		0 (0-0)	
2008	Tunisia	49 (34-64)		6 (4-8)		6 (1-11)		1 (0-1)		0 (0-0)		0 (0-0)	
2009	Tunisia	46 (32-61)		6 (4-7)		6 (1-11)		1 (0-1)		0 (0-0)		0 (0-0)	
2010	Tunisia	43 (30-57)		5 (4-7)		6 (1-10)		1 (0-1)		0 (0-0)		0 (0-0)	
2011	Tunisia	26 (18-34)		3 (2-4)		5 (1-8)		1 (0-1)		0 (0-0)		0 (0-0)	
2012	Tunisia	3 (2-4)		0 (0-0)		1 (0-1)		0 (0-0)		0 (0-0)		0 (0-0)	
2013	Tunisia	3 (2-4)		0 (0-0)		1 (0-1)		0 (0-0)		0 (0-0)		0 (0-0)	
2014	Tunisia	3 (2-4)		0 (0-0)		1 (0-1)		0 (0-0)		0 (0-0)		0 (0-0)	
2015	Tunisia	3 (2-3)	0 (0-0)	0 (0-0)	0 (0-0)	1 (0-1)	0 (0-0)	0 (0-0)	0 (0-0)	0 (0-0)	0 (0-0)	0 (0-0)	0 (0-0)
2000	Turkey	678 (477-891)		10 (7-14)		126 (70-196)		2 (1-3)		1 (1-2)		0 (0-0)	
2001	Turkey	601 (423-790)		9 (6-12)		114 (64-179)		2 (1-3)		1 (1-1)		0 (0-0)	
2002	Turkey	525 (369-689)		8 (6-10)		101 (58-161)		2 (1-2)		1 (0-1)		0 (0-0)	
2003	Turkey	463 (326-608)		7 (5-9)		89 (53-146)		1 (1-2)		1 (0-1)		0 (0-0)	
2004	Turkey	410 (288-538)		6 (4-8)		75 (30-100)		1 (0-2)		1 (0-1)		0 (0-0)	
2005	Turkey	366 (257-480)		6 (4-7)		63 (28-92)		1 (0-1)		1 (0-1)		0 (0-0)	
2006	Turkey	325 (228-426)		5 (4-7)		53 (25-84)		1 (0-1)		0 (0-1)		0 (0-0)	
2007	Turkey	74 (52-98)		1 (1-2)		13 (6-21)		0 (0-0)		0 (0-0)		0 (0-0)	
2008	Turkey	23 (16-30)		0 (0-0)		4 (2-7)		0 (0-0)		0 (0-0)		0 (0-0)	
2009	Turkey	22 (16-29)		0 (0-0)		4 (2-7)		0 (0-0)		0 (0-0)		0 (0-0)	
2010	Turkey	15 (10-19)		0 (0-0)		4 (2-6)		0 (0-0)		0 (0-0)		0 (0-0)	
2011	Turkey	14 (10-18)		0 (0-0)		4 (2-6)		0 (0-0)		0 (0-0)		0 (0-0)	
2012	Turkey	2 (1-2)		0 (0-0)		1 (0-1)		0 (0-0)		0 (0-0)		0 (0-0)	
2013	Turkey	2 (2-3)		0 (0-0)		1 (0-1)		0 (0-0)		0 (0-0)		0 (0-0)	
2014	Turkey	2 (1-2)		0 (0-0)		1 (0-1)		0 (0-0)		0 (0-0)		0 (0-0)	
2015	Turkey	2 (1-2)	0 (0-0)	0 (0-0)	0 (0-0)	0 (0-1)	0 (0-0)	0 (0-0)	0 (0-0)	0 (0-0)	0 (0-0)	0 (0-0)	0 (0-0)
2000	Turkmenistan	256 (180-336)		53 (37-70)		79 (27-124)		16 (6-26)		0 (0-1)		0 (0-0)	
2001	Turkmenistan	252 (177-331)		54 (38-72)		77 (27-120)		17 (6-26)		0 (0-0)		0 (0-0)	
2002	Turkmenistan	258 (181-339)		56 (39-73)		78 (27-122)		17 (6-26)		0 (0-0)		0 (0-0)	
2003	Turkmenistan	269 (189-354)		57 (40-75)		80 (26-130)		17 (5-27)		1 (0-1)		0 (0-0)	
2004	Turkmenistan	276 (194-363)		57 (40-74)		81 (26-131)		17 (5-27)		1 (0-1)		0 (0-0)	
2005	Turkmenistan	275 (193-361)		55 (39-73)		79 (26-128)		16 (5-26)		1 (0-1)		0 (0-0)	
2006	Turkmenistan	265 (186-348)		51 (36-67)		74 (24-120)		14 (5-23)		1 (0-1)		0 (0-0)	
2007	Turkmenistan	249 (175-328)		48 (34-63)		68 (22-110)		13 (4-21)		1 (0-1)		0 (0-0)	
2008	Turkmenistan	230 (161-302)		45 (32-59)		61 (20-99)		12 (4-19)		0 (0-1)		0 (0-0)	
2009	Turkmenistan	211 (148-277)		42 (30-55)		55 (18-88)		11 (4-18)		0 (0-1)		0 (0-0)	
2010	Turkmenistan	87 (61-114)		17 (12-23)		22 (7-35)		4 (1-7)		0 (0-0)		0 (0-0)	
2011	Turkmenistan	22 (15-29)		4 (3-6)		5 (2-9)		1 (0-2)		0 (0-0)		0 (0-0)	
2012	Turkmenistan	13 (9-17)		3 (2-4)		3 (1-5)		1 (0-1)		0 (0-0)		0 (0-0)	
2013	Turkmenistan	13 (9-17)		3 (2-3)		3 (1-5)		1 (0-1)		0 (0-0)		0 (0-0)	
2014	Turkmenistan	13 (9-17)		3 (2-3)		3 (1-5)		1 (0-1)		0 (0-0)		0 (0-0)	
2015	Turkmenistan	2 (1-2)	0 (0-0)	0 (0-0)	0 (0-0)	0 (0-1)	0 (0-0)	0 (0-0)	0 (0-0)	0 (0-0)	0 (0-0)	0 (0-0)	0 (0-0)
2000	Tuvalu	0 (0-0)		19 (14-26)		0 (0-0)		6 (4-8)		0 (0-0)		0 (0-0)	
2001	Tuvalu	0 (0-0)		15 (11-20)		0 (0-0)		5 (3-6)		0 (0-0)		0 (0-0)	
2002	Tuvalu	0 (0-0)		15 (11-20)		0 (0-0)		5 (3-6)		0 (0-0)		0 (0-0)	
2003	Tuvalu	0 (0-0)		17 (12-22)		0 (0-0)		5 (3-7)		0 (0-0)		0 (0-0)	
2004	Tuvalu	0 (0-0)		12 (8-15)		0 (0-0)		4 (2-5)		0 (0-0)		0 (0-0)	
2005	Tuvalu	0 (0-0)		15 (11-20)		0 (0-0)		5 (3-6)		0 (0-0)		0 (0-0)	
2006	Tuvalu	0 (0-0)		13 (9-17)		0 (0-0)		4 (2-5)		0 (0-0)		0 (0-0)	
2007	Tuvalu	0 (0-0)		10 (7-13)		0 (0-0)		3 (2-4)		0 (0-0)		0 (0-0)	
2008	Tuvalu	0 (0-0)		9 (7-12)		0 (0-0)		3 (2-4)		0 (0-0)		0 (0-0)	
2009	Tuvalu	0 (0-0)		2 (2-3)		0 (0-0)		1 (1-2)		0 (0-0)		0 (0-0)	
2010	Tuvalu	0 (0-0)		1 (1-1)		0 (0-0)		1 (0-1)		0 (0-0)		0 (0-0)	
2011	Tuvalu	0 (0-0)		1 (1-1)		0 (0-0)		0 (0-1)		0 (0-0)		0 (0-0)	
2012	Tuvalu	0 (0-0)		1 (1-1)		0 (0-0)		0 (0-1)		0 (0-0)		0 (0-0)	
2013	Tuvalu	0 (0-0)		1 (1-1)		0 (0-0)		0 (0-1)		0 (0-0)		0 (0-0)	
2014	Tuvalu	0 (0-0)		0 (0-0)		0 (0-0)		0 (0-0)		0 (0-0)		0 (0-0)	
2015	Tuvalu	0 (0-0)	0 (0-0)	0 (0-0)	0 (0-0)	0 (0-0)	0 (0-0)	0 (0-0)	0 (0-0)	0 (0-0)	0 (0-0)	0 (0-0)	0 (0-0)
2000	Uganda	3106 (2184-4081)		67 (47-88)		1428 (581-2210)		31 (12-48)		6 (2-9)		0 (0-0)	
2001	Uganda	3052 (2147-4010)		64 (45-84)		1396 (568-2161)		29 (12-45)		6 (2-9)		0 (0-0)	
2002	Uganda	2373 (1669-3118)		48 (34-63)		1077 (443-1661)		22 (9-34)		4 (2-7)		0 (0-0)	
2003	Uganda	470 (330-617)		9 (6-12)		211 (88-325)		4 (2-6)		1 (0-1)		0 (0-0)	
2004	Uganda	390 (274-512)		7 (5-10)		174 (73-266)		3 (1-5)		1 (0-1)		0 (0-0)	
2005	Uganda	379 (266-498)		7 (5-9)		167 (71-254)		3 (1-5)		1 (0-1)		0 (0-0)	
2006	Uganda	361 (254-474)		6 (4-8)		157 (68-238)		3 (1-4)		1 (0-1)		0 (0-0)	
2007	Uganda	28 (19-36)		0 (0-1)		12 (5-18)		0 (0-0)		0 (0-0)		0 (0-0)	
2008	Uganda	27 (19-36)		0 (0-1)		11 (5-17)		0 (0-0)		0 (0-0)		0 (0-0)	
2009	Uganda	26 (18-34)		0 (0-1)		11 (5-16)		0 (0-0)		0 (0-0)		0 (0-0)	
2010	Uganda	26 (18-34)		0 (0-1)		11 (5-16)		0 (0-0)		0 (0-0)		0 (0-0)	
2011	Uganda	26 (18-34)		0 (0-1)		10 (5-16)		0 (0-0)		0 (0-0)		0 (0-0)	
2012	Uganda	25 (18-33)		0 (0-0)		10 (4-16)		0 (0-0)		0 (0-0)		0 (0-0)	
2013	Uganda	25 (18-33)		0 (0-0)		10 (5-16)		0 (0-0)		0 (0-0)		0 (0-0)	
2014	Uganda	25 (17-32)		0 (0-0)		10 (4-15)		0 (0-0)		0 (0-0)		0 (0-0)	
2015	Uganda	24 (17-32)	1 (1-1)	0 (0-0)	0 (0-0)	10 (4-15)	0 (0-1)	0 (0-0)	0 (0-0)	0 (0-0)	0 (0-0)	0 (0-0)	0 (0-0)
2000	Ukraine	117 (82-154)		6 (4-7)		30 (8-58)		1 (0-3)		0 (0-0)		0 (0-0)	
2001	Ukraine	104 (73-137)		5 (4-7)		26 (7-51)		1 (0-3)		0 (0-0)		0 (0-0)	
2002	Ukraine	90 (64-119)		5 (3-6)		23 (6-44)		1 (0-2)		0 (0-0)		0 (0-0)	
2003	Ukraine	81 (57-106)		4 (3-5)		21 (5-40)		1 (0-2)		0 (0-0)		0 (0-0)	
2004	Ukraine	74 (52-97)		4 (3-5)		19 (5-37)		1 (0-2)		0 (0-0)		0 (0-0)	
2005	Ukraine	70 (49-92)		4 (3-5)		18 (5-35)		1 (0-2)		0 (0-0)		0 (0-0)	
2006	Ukraine	64 (45-85)		3 (2-4)		20 (5-39)		1 (0-2)		0 (0-0)		0 (0-0)	

Hib = *Haemophilus influenzae* type b; npnm = non-pneumonia, non-meningitis; HIV = deaths only in HIV-infected children; Uncertainty range provided in parentheses.

Year	Country	Hib pneumonia deaths (UR)	Hib pneumonia deaths, HIV (UR)	Hib pneumonia mortality rate (UR)	Hib pneumonia mortality rate, HIV (UR)	Hib meningitis deaths (UR)	Hib meningitis deaths, HIV (UR)	Hib meningitis mortality rate (UR)	Hib meningitis mortality rate, HIV (UR)	Hib NPNM deaths (UR)	Hib NPNM deaths, HIV (UR)	Hib NPNM mortality rate (UR)	Hib NPNM mortality rate, HIV (UR)
2007	Ukraine	13 (9-17)		1 (0-1)		5 (1-9)		0 (0-0)		0 (0-0)		0 (0-0)	
2008	Ukraine	8 (6-11)		0 (0-0)		3 (1-6)		0 (0-0)		0 (0-0)		0 (0-0)	
2009	Ukraine	10 (7-13)		0 (0-1)		4 (1-7)		0 (0-0)		0 (0-0)		0 (0-0)	
2010	Ukraine	11 (8-15)		0 (0-1)		4 (1-8)		0 (0-0)		0 (0-0)		0 (0-0)	
2011	Ukraine	34 (24-44)		1 (1-2)		11 (3-21)		0 (0-1)		0 (0-0)		0 (0-0)	
2012	Ukraine	1 (0-1)		0 (0-0)		0 (0-0)		0 (0-0)		0 (0-0)		0 (0-0)	
2013	Ukraine	1 (0-1)		0 (0-0)		0 (0-0)		0 (0-0)		0 (0-0)		0 (0-0)	
2014	Ukraine	10 (7-14)		0 (0-1)		4 (1-7)		0 (0-0)		0 (0-0)		0 (0-0)	
2015	Ukraine	9 (6-12)	0 (0-0)	0 (0-0)	0 (0-0)	3 (1-6)	0 (0-0)	0 (0-0)	0 (0-0)	0 (0-0)	0 (0-0)	0 (0-0)	0 (0-0)
2000	United Arab Emirates	1 (0-1)		0 (0-0)		0 (0-0)		0 (0-0)		0 (0-0)		0 (0-0)	
2001	United Arab Emirates	0 (0-1)		0 (0-0)		0 (0-0)		0 (0-0)		0 (0-0)		0 (0-0)	
2002	United Arab Emirates	0 (0-1)		0 (0-0)		0 (0-0)		0 (0-0)		0 (0-0)		0 (0-0)	
2003	United Arab Emirates	0 (0-1)		0 (0-0)		0 (0-0)		0 (0-0)		0 (0-0)		0 (0-0)	
2004	United Arab Emirates	0 (0-0)		0 (0-0)		0 (0-0)		0 (0-0)		0 (0-0)		0 (0-0)	
2005	United Arab Emirates	0 (0-0)		0 (0-0)		0 (0-0)		0 (0-0)		0 (0-0)		0 (0-0)	
2006	United Arab Emirates	0 (0-0)		0 (0-0)		0 (0-0)		0 (0-0)		0 (0-0)		0 (0-0)	
2007	United Arab Emirates	0 (0-0)		0 (0-0)		0 (0-0)		0 (0-0)		0 (0-0)		0 (0-0)	
2008	United Arab Emirates	0 (0-0)		0 (0-0)		0 (0-0)		0 (0-0)		0 (0-0)		0 (0-0)	
2009	United Arab Emirates	0 (0-0)		0 (0-0)		0 (0-0)		0 (0-0)		0 (0-0)		0 (0-0)	
2010	United Arab Emirates	0 (0-0)		0 (0-0)		0 (0-0)		0 (0-0)		0 (0-0)		0 (0-0)	
2011	United Arab Emirates	0 (0-0)		0 (0-0)		0 (0-0)		0 (0-0)		0 (0-0)		0 (0-0)	
2012	United Arab Emirates	0 (0-0)		0 (0-0)		0 (0-0)		0 (0-0)		0 (0-0)		0 (0-0)	
2013	United Arab Emirates	0 (0-0)		0 (0-0)		0 (0-0)		0 (0-0)		0 (0-0)		0 (0-0)	
2014	United Arab Emirates	0 (0-0)		0 (0-0)		0 (0-0)		0 (0-0)		0 (0-0)		0 (0-0)	
2015	United Arab Emirates	0 (0-0)	0 (0-0)	0 (0-0)	0 (0-0)	0 (0-0)	0 (0-0)	0 (0-0)	0 (0-0)	0 (0-0)	0 (0-0)	0 (0-0)	0 (0-0)
2000	United Kingdom	0 (0-0)		0 (0-0)		0 (0-1)		0 (0-0)		0 (0-0)		0 (0-0)	
2001	United Kingdom	0 (0-0)		0 (0-0)		0 (0-1)		0 (0-0)		0 (0-0)		0 (0-0)	
2002	United Kingdom	0 (0-0)		0 (0-0)		0 (0-1)		0 (0-0)		0 (0-0)		0 (0-0)	
2003	United Kingdom	0 (0-0)		0 (0-0)		0 (0-1)		0 (0-0)		0 (0-0)		0 (0-0)	
2004	United Kingdom	0 (0-0)		0 (0-0)		0 (0-1)		0 (0-0)		0 (0-0)		0 (0-0)	
2005	United Kingdom	0 (0-0)		0 (0-0)		0 (0-1)		0 (0-0)		0 (0-0)		0 (0-0)	
2006	United Kingdom	0 (0-0)		0 (0-0)		0 (0-0)		0 (0-0)		0 (0-0)		0 (0-0)	
2007	United Kingdom	0 (0-0)		0 (0-0)		0 (0-0)		0 (0-0)		0 (0-0)		0 (0-0)	
2008	United Kingdom	0 (0-0)		0 (0-0)		0 (0-0)		0 (0-0)		0 (0-0)		0 (0-0)	
2009	United Kingdom	0 (0-0)		0 (0-0)		0 (0-1)		0 (0-0)		0 (0-0)		0 (0-0)	
2010	United Kingdom	0 (0-0)		0 (0-0)		0 (0-1)		0 (0-0)		0 (0-0)		0 (0-0)	
2011	United Kingdom	0 (0-1)		0 (0-0)		0 (0-0)		0 (0-0)		0 (0-0)		0 (0-0)	
2012	United Kingdom	0 (0-0)		0 (0-0)		0 (0-0)		0 (0-0)		0 (0-0)		0 (0-0)	
2013	United Kingdom	0 (0-0)		0 (0-0)		0 (0-0)		0 (0-0)		0 (0-0)		0 (0-0)	
2014	United Kingdom	0 (0-0)		0 (0-0)		0 (0-0)		0 (0-0)		0 (0-0)		0 (0-0)	
2015	United Kingdom	0 (0-0)	0 (0-0)	0 (0-0)	0 (0-0)	0 (0-0)	0 (0-0)	0 (0-0)	0 (0-0)	0 (0-0)	0 (0-0)	0 (0-0)	0 (0-0)
2000	United Republic of Tanzania	4382 (3082-5758)		75 (53-99)		1972 (706-3137)		34 (12-54)		8 (3-13)		0 (0-0)	
2001	United Republic of Tanzania	4244 (2985-5577)		71 (50-93)		1902 (689-3017)		32 (12-50)		8 (3-12)		0 (0-0)	
2002	United Republic of Tanzania	4063 (2858-5339)		66 (46-86)		1811 (663-2863)		29 (11-46)		7 (3-12)		0 (0-0)	
2003	United Republic of Tanzania	4108 (2889-5398)		64 (45-84)		1815 (673-2862)		28 (11-45)		7 (3-12)		0 (0-0)	
2004	United Republic of Tanzania	4005 (2817-5263)		60 (42-79)		1748 (656-2748)		26 (10-41)		7 (3-11)		0 (0-0)	
2005	United Republic of Tanzania	3885 (2733-5105)		56 (40-74)		1671 (628-2631)		24 (9-38)		7 (3-11)		0 (0-0)	
2006	United Republic of Tanzania	3748 (2636-4924)		52 (37-69)		1588 (610-2486)		22 (9-35)		6 (2-10)		0 (0-0)	
2007	United Republic of Tanzania	3521 (2477-4627)		48 (34-63)		1471 (577-2289)		20 (8-31)		6 (2-9)		0 (0-0)	
2008	United Republic of Tanzania	3339 (2348-4387)		44 (31-58)		1376 (506-2198)		18 (7-29)		11 (4-18)		0 (0-0)	
2009	United Republic of Tanzania	521 (366-684)		7 (5-9)		212 (80-336)		3 (1-4)		2 (1-3)		0 (0-0)	
2010	United Republic of Tanzania	245 (173-322)		3 (2-4)		99 (38-156)		1 (0-2)		1 (0-1)		0 (0-0)	
2011	United Republic of Tanzania	245 (172-322)		3 (2-4)		98 (38-153)		1 (0-2)		1 (0-1)		0 (0-0)	
2012	United Republic of Tanzania	233 (164-307)		3 (2-4)		93 (37-145)		1 (0-2)		1 (0-1)		0 (0-0)	
2013	United Republic of Tanzania	239 (168-314)		3 (2-4)		94 (38-147)		1 (0-2)		1 (0-1)		0 (0-0)	
2014	United Republic of Tanzania	30 (21-39)		0 (0-0)		12 (5-18)		0 (0-0)		0 (0-0)		0 (0-0)	
2015	United Republic of Tanzania	29 (21-39)	1 (0-1)	0 (0-0)	0 (0-0)	11 (5-18)	0 (0-0)	0 (0-0)	0 (0-0)	0 (0-0)	0 (0-0)	0 (0-0)	0 (0-0)
2000	United States of America	2 (1-3)		0 (0-0)		2 (1-3)		0 (0-0)		0 (0-0)		0 (0-0)	
2001	United States of America	2 (1-3)		0 (0-0)		1 (1-2)		0 (0-0)		0 (0-0)		0 (0-0)	
2002	United States of America	2 (1-3)		0 (0-0)		1 (1-2)		0 (0-0)		0 (0-0)		0 (0-0)	
2003	United States of America	3 (2-3)		0 (0-0)		1 (1-2)		0 (0-0)		0 (0-0)		0 (0-0)	
2004	United States of America	3 (2-3)		0 (0-0)		1 (1-2)		0 (0-0)		0 (0-0)		0 (0-0)	
2005	United States of America	2 (2-3)		0 (0-0)		2 (1-3)		0 (0-0)		0 (0-0)		0 (0-0)	
2006	United States of America	2 (2-3)		0 (0-0)		1 (1-2)		0 (0-0)		0 (0-0)		0 (0-0)	
2007	United States of America	2 (2-3)		0 (0-0)		1 (1-2)		0 (0-0)		0 (0-0)		0 (0-0)	
2008	United States of America	2 (2-3)		0 (0-0)		1 (0-2)		0 (0-0)		0 (0-0)		0 (0-0)	
2009	United States of America	2 (2-3)		0 (0-0)		1 (0-2)		0 (0-0)		0 (0-0)		0 (0-0)	
2010	United States of America	2 (1-3)		0 (0-0)		1 (1-3)		0 (0-0)		0 (0-0)		0 (0-0)	
2011	United States of America	2 (2-3)		0 (0-0)		1 (0-2)		0 (0-0)		0 (0-0)		0 (0-0)	
2012	United States of America	2 (1-3)		0 (0-0)		1 (0-1)		0 (0-0)		0 (0-0)		0 (0-0)	
2013	United States of America	2 (1-2)		0 (0-0)		1 (0-2)		0 (0-0)		0 (0-0)		0 (0-0)	
2014	United States of America	2 (1-3)		0 (0-0)		1 (0-2)		0 (0-0)		0 (0-0)		0 (0-0)	
2015	United States of America	2 (1-3)	0 (0-0)	0 (0-0)	0 (0-0)	1 (0-1)	0 (0-0)	0 (0-0)	0 (0-0)	0 (0-0)	0 (0-0)	0 (0-0)	0 (0-0)
2000	Uruguay	1 (1-2)		0 (0-1)		0 (0-1)		0 (0-0)		0 (0-0)		0 (0-0)	
2001	Uruguay	2 (1-2)		1 (0-1)		1 (0-1)		0 (0-0)		0 (0-0)		0 (0-0)	
2002	Uruguay	0 (0-0)		0 (0-0)		0 (0-0)		0 (0-0)		0 (0-0)		0 (0-0)	
2003	Uruguay	0 (0-0)		0 (0-0)		0 (0-0)		0 (0-0)		0 (0-0)		0 (0-0)	
2004	Uruguay	0 (0-0)		0 (0-0)		0 (0-0)		0 (0-0)		0 (0-0)		0 (0-0)	
2005	Uruguay	0 (0-0)		0 (0-0)		0 (0-0)		0 (0-0)		0 (0-0)		0 (0-0)	
2006	Uruguay	0 (0-0)		0 (0-0)		0 (0-0)		0 (0-0)		0 (0-0)		0 (0-0)	
2007	Uruguay	0 (0-0)		0 (0-0)		0 (0-0)		0 (0-0)		0 (0-0)		0 (0-0)	
2008	Uruguay	0 (0-0)		0 (0-0)		0 (0-0)		0 (0-0)		0 (0-0)		0 (0-0)	
2009	Uruguay	0 (0-0)		0 (0-0)		0 (0-0)		0 (0-0)		0 (0-0)		0 (0-0)	
2010	Uruguay	0 (0-0)		0 (0-0)		0 (0-0)		0 (0-0)		0 (0-0)		0 (0-0)	
2011	Uruguay	0 (0-0)		0 (0-0)		0 (0-0)		0 (0-0)		0 (0-0)		0 (0-0)	
2012	Uruguay	0 (0-0)		0 (0-0)		0 (0-0)		0 (0-0)		0 (0-0)		0 (0-0)	
2013	Uruguay	0 (0-0)		0 (0-0)		0 (0-0)		0 (0-0)		0 (0-0)		0 (0-0)	
2014	Uruguay	0 (0-0)		0 (0-0)		0 (0-0)		0 (0-0)		0 (0-0)		0 (0-0)	

Hib = *Haemophilus influenzae* type b; npnm = non-pneumonia, non-meningitis; HIV = deaths only in HIV-infected children; Uncertainty range provided in parentheses.

Year	Country	Hib pneumonia deaths (UR)	Hib pneumonia deaths, HIV (UR)	Hib pneumonia mortality rate (UR)	Hib pneumonia mortality rate, HIV (UR)	Hib meningitis deaths (UR)	Hib meningitis deaths, HIV (UR)	Hib meningitis mortality rate (UR)	Hib meningitis mortality rate, HIV (UR)	Hib NPNM deaths (UR)	Hib NPNM deaths, HIV (UR)	Hib NPNM mortality rate (UR)	Hib NPNM mortality rate, HIV (UR)
2015	Uruguay	0 (0-0)	0 (0-0)	0 (0-0)	0 (0-0)	0 (0-0)	0 (0-0)	0 (0-0)	0 (0-0)	0 (0-0)	0 (0-0)	0 (0-0)	0 (0-0)
2000	Uzbekistan	995 (700-1308)		37 (26-49)		369 (134-582)		14 (5-22)		3 (1-5)		0 (0-0)	
2001	Uzbekistan	945 (664-1241)		36 (25-47)		347 (127-548)		13 (5-21)		3 (1-4)		0 (0-0)	
2002	Uzbekistan	898 (631-1180)		35 (25-46)		326 (120-514)		13 (5-20)		3 (1-4)		0 (0-0)	
2003	Uzbekistan	855 (601-1123)		34 (24-45)		307 (113-483)		12 (4-19)		2 (1-4)		0 (0-0)	
2004	Uzbekistan	832 (585-1093)		33 (23-44)		294 (109-461)		12 (4-19)		2 (1-4)		0 (0-0)	
2005	Uzbekistan	811 (571-1066)		33 (23-43)		281 (105-440)		11 (4-18)		2 (1-4)		0 (0-0)	
2006	Uzbekistan	806 (567-1058)		32 (22-42)		272 (102-427)		11 (4-17)		2 (1-3)		0 (0-0)	
2007	Uzbekistan	805 (566-1058)		31 (22-41)		266 (100-416)		10 (4-16)		2 (1-3)		0 (0-0)	
2008	Uzbekistan	813 (572-1068)		31 (22-40)		263 (99-411)		10 (4-15)		2 (1-3)		0 (0-0)	
2009	Uzbekistan	124 (87-163)		5 (3-6)		39 (15-61)		1 (1-2)		0 (0-0)		0 (0-0)	
2010	Uzbekistan	49 (34-64)		2 (1-2)		15 (6-24)		1 (0-1)		0 (0-0)		0 (0-0)	
2011	Uzbekistan	48 (34-63)		2 (1-2)		15 (6-23)		0 (0-1)		0 (0-0)		0 (0-0)	
2012	Uzbekistan	47 (33-61)		2 (1-2)		14 (5-22)		0 (0-1)		0 (0-0)		0 (0-0)	
2013	Uzbekistan	45 (32-59)		1 (1-2)		14 (5-21)		0 (0-1)		0 (0-0)		0 (0-0)	
2014	Uzbekistan	7 (5-10)		0 (0-0)		2 (1-3)		0 (0-0)		0 (0-0)		0 (0-0)	
2015	Uzbekistan	7 (5-9)	0 (0-0)	0 (0-0)	0 (0-0)	2 (1-3)	0 (0-0)	0 (0-0)	0 (0-0)	0 (0-0)	0 (0-0)	0 (0-0)	0 (0-0)
2000	Vanuatu	4 (3-5)		14 (10-19)		1 (0-2)		4 (1-6)		0 (0-0)		0 (0-0)	
2001	Vanuatu	4 (3-5)		14 (10-18)		1 (0-2)		4 (1-6)		0 (0-0)		0 (0-0)	
2002	Vanuatu	3 (2-4)		11 (8-14)		1 (0-1)		3 (1-4)		0 (0-0)		0 (0-0)	
2003	Vanuatu	3 (2-3)		9 (6-12)		1 (0-1)		3 (1-4)		0 (0-0)		0 (0-0)	
2004	Vanuatu	3 (2-4)		10 (7-13)		1 (0-1)		3 (1-4)		0 (0-0)		0 (0-0)	
2005	Vanuatu	4 (3-5)		12 (9-16)		1 (0-1)		4 (1-5)		0 (0-0)		0 (0-0)	
2006	Vanuatu	4 (3-5)		13 (9-17)		1 (0-2)		4 (1-5)		0 (0-0)		0 (0-0)	
2007	Vanuatu	4 (3-6)		14 (10-18)		1 (0-2)		4 (1-6)		0 (0-0)		0 (0-0)	
2008	Vanuatu	4 (3-6)		14 (10-18)		1 (0-2)		4 (1-6)		0 (0-0)		0 (0-0)	
2009	Vanuatu	5 (3-6)		14 (10-19)		1 (0-2)		4 (1-6)		0 (0-0)		0 (0-0)	
2010	Vanuatu	5 (3-6)		14 (10-19)		1 (0-2)		4 (1-6)		0 (0-0)		0 (0-0)	
2011	Vanuatu	2 (1-2)		5 (4-7)		1 (0-1)		3 (1-4)		0 (0-0)		0 (0-0)	
2012	Vanuatu	1 (0-1)		2 (1-2)		0 (0-1)		1 (0-2)		0 (0-0)		0 (0-0)	
2013	Vanuatu	1 (0-1)		2 (1-2)		0 (0-1)		1 (0-2)		0 (0-0)		0 (0-0)	
2014	Vanuatu	1 (0-1)		2 (1-2)		0 (0-1)		1 (0-2)		0 (0-0)		0 (0-0)	
2015	Vanuatu	1 (0-1)	0 (0-0)	2 (1-2)	0 (0-0)	0 (0-0)	0 (0-0)	1 (0-1)	0 (0-0)	0 (0-0)	0 (0-0)	0 (0-0)	0 (0-0)
2000	Venezuela (Bolivarian Republic of)	143 (100-187)		5 (4-7)		84 (12-146)		3 (0-5)		1 (0-1)		0 (0-0)	
2001	Venezuela (Bolivarian Republic of)	59 (41-77)		2 (1-3)		30 (4-52)		1 (0-2)		0 (0-0)		0 (0-0)	
2002	Venezuela (Bolivarian Republic of)	22 (15-29)		1 (1-1)		13 (2-23)		0 (0-1)		0 (0-0)		0 (0-0)	
2003	Venezuela (Bolivarian Republic of)	27 (19-36)		1 (1-1)		10 (1-17)		0 (0-1)		0 (0-0)		0 (0-0)	
2004	Venezuela (Bolivarian Republic of)	27 (19-35)		1 (1-1)		13 (2-23)		0 (0-1)		0 (0-0)		0 (0-0)	
2005	Venezuela (Bolivarian Republic of)	2 (1-2)		0 (0-0)		1 (0-2)		0 (0-0)		0 (0-0)		0 (0-0)	
2006	Venezuela (Bolivarian Republic of)	2 (1-2)		0 (0-0)		1 (0-1)		0 (0-0)		0 (0-0)		0 (0-0)	
2007	Venezuela (Bolivarian Republic of)	21 (15-28)		1 (1-1)		9 (1-16)		0 (0-1)		0 (0-0)		0 (0-0)	
2008	Venezuela (Bolivarian Republic of)	23 (16-30)		1 (1-1)		11 (2-19)		0 (0-1)		0 (0-0)		0 (0-0)	
2009	Venezuela (Bolivarian Republic of)	1 (1-2)		0 (0-0)		1 (0-1)		0 (0-0)		0 (0-0)		0 (0-0)	
2010	Venezuela (Bolivarian Republic of)	1 (1-2)		0 (0-0)		1 (0-1)		0 (0-0)		0 (0-0)		0 (0-0)	
2011	Venezuela (Bolivarian Republic of)	1 (1-2)		0 (0-0)		1 (0-1)		0 (0-0)		0 (0-0)		0 (0-0)	
2012	Venezuela (Bolivarian Republic of)	1 (1-2)		0 (0-0)		1 (0-1)		0 (0-0)		0 (0-0)		0 (0-0)	
2013	Venezuela (Bolivarian Republic of)	1 (1-2)		0 (0-0)		1 (0-1)		0 (0-0)		0 (0-0)		0 (0-0)	
2014	Venezuela (Bolivarian Republic of)	1 (1-2)		0 (0-0)		1 (0-1)		0 (0-0)		0 (0-0)		0 (0-0)	
2015	Venezuela (Bolivarian Republic of)	1 (1-2)	0 (0-0)	0 (0-0)	0 (0-0)	1 (0-1)	0 (0-0)	0 (0-0)	0 (0-0)	0 (0-0)	0 (0-0)	0 (0-0)	0 (0-0)
2000	Viet Nam	1053 (741-1384)		15 (10-19)		225 (78-361)		3 (1-5)		2 (1-3)		0 (0-0)	
2001	Viet Nam	1004 (706-1319)		15 (11-20)		216 (79-343)		3 (1-5)		2 (1-3)		0 (0-0)	
2002	Viet Nam	1017 (716-1337)		16 (11-21)		220 (86-343)		3 (1-5)		2 (1-3)		0 (0-0)	
2003	Viet Nam	1038 (730-1364)		16 (11-21)		224 (91-349)		3 (1-5)		2 (1-3)		0 (0-0)	
2004	Viet Nam	1037 (729-1362)		16 (11-21)		222 (42-364)		3 (1-6)		2 (0-3)		0 (0-0)	
2005	Viet Nam	1014 (713-1333)		15 (11-20)		216 (44-352)		3 (1-5)		2 (0-3)		0 (0-0)	
2006	Viet Nam	967 (680-1271)		14 (10-19)		205 (46-329)		3 (1-5)		2 (0-3)		0 (0-0)	
2007	Viet Nam	902 (634-1185)		13 (9-17)		192 (41-315)		3 (1-5)		2 (0-3)		0 (0-0)	
2008	Viet Nam	822 (578-1079)		12 (8-15)		177 (36-293)		3 (1-4)		1 (0-2)		0 (0-0)	
2009	Viet Nam	757 (533-995)		11 (8-14)		164 (31-274)		2 (0-4)		1 (0-2)		0 (0-0)	
2010	Viet Nam	283 (199-372)		4 (3-5)		96 (18-162)		1 (0-2)		1 (0-1)		0 (0-0)	
2011	Viet Nam	55 (39-73)		1 (1-1)		22 (4-37)		0 (0-1)		0 (0-0)		0 (0-0)	
2012	Viet Nam	51 (36-67)		1 (0-1)		20 (4-33)		0 (0-0)		0 (0-0)		0 (0-0)	
2013	Viet Nam	97 (68-127)		1 (1-2)		35 (7-57)		0 (0-1)		0 (0-0)		0 (0-0)	
2014	Viet Nam	52 (37-68)		1 (0-1)		19 (4-30)		0 (0-0)		0 (0-0)		0 (0-0)	
2015	Viet Nam	7 (5-9)	0 (0-0)	0 (0-0)	0 (0-0)	3 (1-4)	0 (0-0)	0 (0-0)	0 (0-0)	0 (0-0)	0 (0-0)	0 (0-0)	0 (0-0)
2000	Yemen	1858 (1306-2441)		60 (42-79)		599 (207-943)		19 (7-31)		2 (1-4)		0 (0-0)	
2001	Yemen	1773 (1247-2329)		58 (41-76)		569 (195-898)		19 (6-29)		2 (1-4)		0 (0-0)	
2002	Yemen	1700 (1196-2234)		55 (39-72)		544 (185-859)		18 (6-28)		2 (1-4)		0 (0-0)	
2003	Yemen	1646 (1157-2162)		52 (37-69)		525 (177-831)		17 (6-26)		2 (1-3)		0 (0-0)	
2004	Yemen	1588 (1117-2087)		49 (35-65)		506 (169-802)		16 (5-25)		2 (1-3)		0 (0-0)	
2005	Yemen	996 (700-1309)		30 (21-40)		317 (94-518)		10 (3-16)		3 (1-4)		0 (0-0)	
2006	Yemen	168 (118-221)		5 (3-7)		53 (16-87)		2 (0-3)		0 (0-1)		0 (0-0)	
2007	Yemen	155 (109-204)		4 (3-6)		49 (14-80)		1 (0-2)		0 (0-1)		0 (0-0)	
2008	Yemen	146 (103-192)		4 (3-5)		46 (13-76)		1 (0-2)		0 (0-1)		0 (0-0)	
2009	Yemen	140 (99-184)		4 (3-5)		45 (13-73)		1 (0-2)		0 (0-1)		0 (0-0)	
2010	Yemen	12 (8-15)		0 (0-0)		4 (1-6)		0 (0-0)		0 (0-0)		0 (0-0)	
2011	Yemen	11 (8-14)		0 (0-0)		3 (1-6)		0 (0-0)		0 (0-0)		0 (0-0)	
2012	Yemen	10 (7-14)		0 (0-0)		3 (1-6)		0 (0-0)		0 (0-0)		0 (0-0)	
2013	Yemen	10 (7-13)		0 (0-0)		3 (1-5)		0 (0-0)		0 (0-0)		0 (0-0)	
2014	Yemen	9 (7-12)		0 (0-0)		3 (1-5)		0 (0-0)		0 (0-0)		0 (0-0)	
2015	Yemen	9 (6-12)	0 (0-0)	0 (0-0)	0 (0-0)	3 (1-5)	0 (0-0)	0 (0-0)	0 (0-0)	0 (0-0)	0 (0-0)	0 (0-0)	0 (0-0)
2000	Zambia	1781 (1253-2340)		94 (66-124)		644 (355-911)		34 (19-48)		3 (1-4)		0 (0-0)	
2001	Zambia	1731 (1217-2274)		89 (62-117)		621 (345-876)		32 (18-45)		3 (1-4)		0 (0-0)	
2002	Zambia	1649 (1160-2167)		82 (58-108)		587 (243-903)		29 (12-45)		2 (1-4)		0 (0-0)	
2003	Zambia	1565 (1101-2056)		76 (53-100)		552 (229-850)		27 (11-41)		2 (1-3)		0 (0-0)	
2004	Zambia	245 (173-322)		12 (8-15)		86 (36-132)		4 (2-6)		0 (0-1)		0 (0-0)	
2005	Zambia	137 (96-180)		6 (4-8)		48 (20-73)		2 (1-3)		0 (0-0)		0 (0-0)	
2006	Zambia	136 (96-179)		6 (4-8)		47 (19-72)		2 (1-3)		0 (0-0)		0 (0-0)	

Hib = *Haemophilus influenzae* type b; npnm = non-pneumonia, non-meningitis; HIV = deaths only in HIV-infected children; Uncertainty range provided in parentheses.

Year	Country	Hib pneumonia deaths (UR)	Hib pneumonia deaths, HIV (UR)	Hib pneumonia mortality rate (UR)	Hib pneumonia mortality rate, HIV (UR)	Hib meningitis deaths (UR)	Hib meningitis deaths, HIV (UR)	Hib meningitis mortality rate (UR)	Hib meningitis mortality rate, HIV (UR)	Hib NPNM deaths (UR)	Hib NPNM deaths, HIV (UR)	Hib NPNM mortality rate (UR)	Hib NPNM mortality rate, HIV (UR)
2007	Zambia	137 (96-180)		6 (4-8)		46 (19-72)		2 (1-3)		0 (0-0)		0 (0-0)	
2008	Zambia	119 (84-157)		5 (4-7)		40 (16-61)		2 (1-3)		0 (0-0)		0 (0-0)	
2009	Zambia	14 (10-18)		1 (0-1)		4 (2-7)		0 (0-0)		0 (0-0)		0 (0-0)	
2010	Zambia	13 (9-18)		1 (0-1)		4 (2-7)		0 (0-0)		0 (0-0)		0 (0-0)	
2011	Zambia	13 (9-17)		1 (0-1)		4 (2-6)		0 (0-0)		0 (0-0)		0 (0-0)	
2012	Zambia	13 (9-16)		0 (0-1)		4 (2-6)		0 (0-0)		0 (0-0)		0 (0-0)	
2013	Zambia	12 (8-16)		0 (0-1)		4 (1-6)		0 (0-0)		0 (0-0)		0 (0-0)	
2014	Zambia	12 (8-15)		0 (0-1)		4 (1-6)		0 (0-0)		0 (0-0)		0 (0-0)	
2015	Zambia	12 (8-15)	0 (0-1)	0 (0-1)	0 (0-0)	4 (1-6)	0 (0-0)	0 (0-0)	0 (0-0)	0 (0-0)	0 (0-0)	0 (0-0)	0 (0-0)
2000	Zimbabwe	745 (524-979)		41 (29-54)		267 (76-442)		15 (4-24)		1 (0-2)		0 (0-0)	
2001	Zimbabwe	767 (540-1008)		42 (30-55)		278 (79-460)		15 (4-25)		1 (0-2)		0 (0-0)	
2002	Zimbabwe	801 (563-1052)		44 (31-57)		296 (84-489)		16 (5-27)		1 (0-2)		0 (0-0)	
2003	Zimbabwe	841 (592-1105)		45 (32-60)		318 (91-526)		17 (5-28)		1 (0-2)		0 (0-0)	
2004	Zimbabwe	891 (627-1171)		48 (33-62)		345 (98-571)		18 (5-30)		1 (0-2)		0 (0-0)	
2005	Zimbabwe	946 (665-1243)		50 (35-65)		376 (107-622)		20 (6-33)		2 (0-3)		0 (0-0)	
2006	Zimbabwe	991 (697-1302)		51 (36-67)		402 (115-665)		21 (6-34)		2 (0-3)		0 (0-0)	
2007	Zimbabwe	1037 (729-1362)		52 (37-68)		426 (127-700)		21 (6-35)		2 (1-3)		0 (0-0)	
2008	Zimbabwe	244 (171-320)		12 (8-16)		101 (32-165)		5 (2-8)		0 (0-1)		0 (0-0)	
2009	Zimbabwe	121 (85-159)		6 (4-8)		50 (16-81)		2 (1-4)		0 (0-0)		0 (0-0)	
2010	Zimbabwe	90 (63-118)		4 (3-5)		37 (13-60)		2 (1-3)		0 (0-0)		0 (0-0)	
2011	Zimbabwe	83 (58-109)		4 (3-5)		34 (12-55)		2 (1-2)		0 (0-0)		0 (0-0)	
2012	Zimbabwe	75 (53-98)		3 (2-4)		30 (11-49)		1 (0-2)		0 (0-0)		0 (0-0)	
2013	Zimbabwe	11 (8-14)		0 (0-1)		4 (1-7)		0 (0-0)		0 (0-0)		0 (0-0)	
2014	Zimbabwe	11 (8-14)		0 (0-1)		4 (1-7)		0 (0-0)		0 (0-0)		0 (0-0)	
2015	Zimbabwe	11 (8-14)	1 (0-1)	0 (0-1)	0 (0-0)	4 (1-7)	0 (0-0)	0 (0-0)	0 (0-0)	0 (0-0)	0 (0-0)	0 (0-0)	0 (0-0)

Hib = *Haemophilus influenzae* type b; npnm = non-pneumonia, non-meningitis; HIV = deaths only in HIV-infected children; Uncertainty range provided in parentheses.

Table 9: *Streptococcus pneumoniae* morbidity by syndrome, country, and year

		Pneumonia cases				Severe pneumonia cases				Meningitis cases				NPNM cases				NPNM non-severe cases			
Year	Country	Spn pneumonia cases (UR)	Spn pneumonia cases, HIV (UR)	Spn pneumonia incidence rate (UR)	Spn pneumonia incidence rate, HIV (UR)	Spn severe pneumonia cases (UR)	Spn severe pneumonia cases, HIV (UR)	Spn severe pneumonia incidence rate (UR)	Spn severe pneumonia incidence rate, HIV (UR)	Spn meningitis cases (UR)	Spn meningitis cases, HIV (UR)	Spn meningitis incidence rate (UR)	Spn meningitis incidence rate, HIV (UR)	Spn NPNM cases (UR)	Spn NPNM cases, HIV (UR)	Spn NPNM incidence rate (UR)	Spn NPNM incidence rate, HIV (UR)	Spn NPNM non-severe cases (UR)	Spn NPNM non-severe cases, HIV (UR)	SPN NPNM non-severe incidence rate (UR)	SPN NPNM non-severe incidence rate, HIV (UR)
2000	Afghanistan	191556 (165279-227706)		4946 (4268-5880)		74930 (56137-85464)		1935 (1450-2207)		2832 (1137-6003)		73 (29-155)		1136 (456-2408)		29 (12-62)		8552 (3434-18127)		221 (89-468)	
2001	Afghanistan									2893 (1167-6120)		72 (29-152)		1160 (468-2455)		29 (12-61)		8734 (3523-18479)		217 (87-458)	
2002	Afghanistan									2913 (1181-6151)		70 (28-147)		1169 (474-2467)		28 (11-59)		8797 (3567-18573)		210 (85-443)	
2003	Afghanistan									2929 (1193-6168)		67 (27-142)		1175 (479-2474)		27 (11-57)		8843 (3604-18625)		204 (83-429)	
2004	Afghanistan									2921 (1196-6135)		65 (27-137)		1172 (480-2461)		26 (11-55)		8820 (3612-18525)		197 (81-414)	
2005	Afghanistan									2885 (1188-6042)		63 (26-132)		1157 (476-2423)		25 (10-53)		8710 (3586-18243)		190 (78-398)	
2006	Afghanistan									2855 (1181-5963)		61 (25-127)		1145 (474-2392)		24 (10-51)		8621 (3566-18006)		183 (76-383)	
2007	Afghanistan									2793 (1161-5819)		58 (24-122)		1120 (466-2334)		23 (10-49)		8435 (3505-17572)		176 (73-367)	
2008	Afghanistan									2719 (1135-5650)		56 (23-117)		1090 (455-2266)		23 (9-47)		8209 (3426-17060)		169 (71-352)	
2009	Afghanistan									2628 (1102-5448)		54 (23-111)		1054 (442-2185)		22 (9-45)		7934 (3326-16449)		162 (68-337)	
2010	Afghanistan									2530 (1065-5234)		51 (22-106)		1015 (427-2099)		21 (9-43)		7640 (3217-15805)		155 (65-321)	
2011	Afghanistan									2424 (1020-5009)		49 (21-101)		972 (409-2009)		20 (8-41)		7320 (3079-15126)		148 (62-306)	
2012	Afghanistan									2317 (979-4776)		47 (20-97)		929 (393-1915)		19 (8-39)		6995 (2956-14420)		142 (60-292)	
2013	Afghanistan									2214 (940-4554)		45 (19-93)		888 (377-1826)		18 (8-37)		6685 (2840-13750)		136 (58-280)	
2014	Afghanistan									1679 (717-3446)		34 (15-71)		673 (288-1382)		14 (6-28)		5070 (2165-10404)		104 (44-213)	
2015	Afghanistan	127148 (109706-151143)	224 (194-267)	2612 (2254-3105)	5 (4-5)	50138 (37563-57187)	89 (66-101)	1030 (772-1175)	2 (1-2)	1277 (549-2615)	2 (1-5)	26 (11-54)	0 (0-0)	512 (220-1049)	1 (0-2)	11 (5-22)	0 (0-0)	3856 (1656-7897)	7 (3-14)	79 (34-162)	0 (0-0)
2000	Albania	630 (544-749)		230 (198-273)		409 (306-466)		149 (112-170)		16 (6-60)		6 (2-22)		18 (6-68)		7 (2-25)		48 (17-180)		18 (6-66)	
2001	Albania									12 (3-50)		5 (1-19)		14 (4-57)		5 (1-22)		37 (10-150)		14 (4-57)	
2002	Albania									9 (2-40)		4 (1-16)		10 (2-46)		4 (1-18)		28 (7-121)		11 (3-49)	
2003	Albania									7 (1-33)		3 (1-14)		8 (2-38)		3 (1-16)		22 (4-100)		9 (2-42)	
2004	Albania									6 (1-28)		3 (0-12)		6 (1-31)		3 (1-14)		17 (3-83)		8 (1-37)	
2005	Albania									5 (1-24)		2 (0-11)		5 (1-27)		3 (0-13)		14 (2-72)		7 (1-34)	
2006	Albania									4 (1-21)		2 (0-11)		5 (1-24)		2 (0-12)		13 (2-63)		7 (1-32)	
2007	Albania									4 (1-19)		2 (0-10)		5 (1-21)		3 (1-12)		12 (3-57)		7 (1-31)	
2008	Albania									4 (1-17)		2 (1-10)		4 (1-19)		3 (1-11)		12 (3-52)		7 (2-30)	
2009	Albania									7 (2-29)		4 (1-18)		8 (2-32)		5 (1-20)		20 (5-86)		12 (3-54)	
2010	Albania									7 (2-29)		4 (1-19)		8 (2-33)		5 (1-21)		21 (5-88)		13 (3-56)	
2011	Albania									4 (1-17)		3 (1-11)		5 (1-19)		3 (1-12)		12 (3-51)		8 (2-33)	
2012	Albania									3 (1-13)		2 (0-8)		4 (1-15)		2 (1-10)		10 (2-41)		6 (1-25)	
2013	Albania									3 (1-13)		2 (0-8)		3 (1-14)		2 (1-9)		9 (2-38)		5 (1-23)	
2014	Albania									3 (1-13)		2 (0-7)		3 (1-14)		2 (0-8)		9 (2-38)		5 (1-22)	
2015	Albania	129 (112-154)	0 (0-1)	71 (61-84)	0 (0-0)	84 (63-96)	0 (0-0)	46 (34-52)	0 (0-0)	2 (1-9)	0 (0-0)	1 (0-5)	0 (0-0)	3 (1-10)	0 (0-0)	1 (0-6)	0 (0-0)	7 (2-28)	0 (0-0)	4 (1-15)	0 (0-0)
2000	Algeria	66030 (56972-78491)		2175 (1876-2585)		25976 (19461-29628)		855 (641-976)		193 (69-419)		6 (2-14)		219 (78-476)		7 (3-16)		583 (207-1266)		19 (7-42)	
2001	Algeria									183 (65-397)		6 (2-14)		208 (74-451)		7 (3-16)		553 (197-1200)		19 (7-41)	
2002	Algeria									174 (62-378)		6 (2-13)		198 (71-429)		7 (2-15)		526 (188-1140)		19 (7-40)	
2003	Algeria									167 (60-362)		6 (2-13)		190 (68-411)		7 (2-15)		505 (181-1094)		18 (6-39)	
2004	Algeria									163 (59-352)		6 (2-12)		185 (67-400)		7 (2-14)		492 (177-1064)		17 (6-37)	
2005	Algeria									162 (58-349)		6 (2-12)		184 (66-397)		6 (2-14)		488 (176-1054)		17 (6-36)	
2006	Algeria									167 (60-360)		6 (2-12)		190 (69-409)		6 (2-14)		505 (182-1088)		17 (6-36)	
2007	Algeria									176 (65-377)		6 (2-12)		200 (74-428)		6 (2-13)		532 (197-1137)		17 (6-36)	
2008	Algeria									192 (51-483)		6 (2-14)		218 (58-549)		6 (2-16)		579 (155-1459)		17 (5-43)	
2009	Algeria									206 (57-516)		6 (2-14)		235 (65-586)		7 (2-16)		623 (173-1558)		17 (5-43)	
2010	Algeria									221 (64-547)		6 (2-14)		251 (72-621)		7 (2-16)		668 (193-1652)		18 (5-44)	
2011	Algeria									233 (70-571)		6 (2-14)		265 (79-649)		7 (2-16)		704 (211-1725)		17 (5-43)	
2012	Algeria									243 (76-590)		6 (2-14)		276 (87-671)		7 (2-16)		735 (230-1783)		17 (5-42)	
2013	Algeria									250 (78-604)		6 (2-14)		284 (89-686)		6 (2-16)		754 (235-1824)		17 (5-42)	
2014	Algeria									251 (78-606)		6 (2-14)		285 (89-688)		6 (2-15)		758 (237-1829)		17 (5-41)	
2015	Algeria	74877 (64606-89008)	58 (50-69)	1659 (1431-1972)	1 (1-2)	29391 (22019-33523)	23 (17-26)	651 (488-743)	1 (0-1)	251 (78-606)	0 (0-0)	6 (2-13)	0 (0-0)	285 (89-688)	0 (0-1)	6 (2-15)	0 (0-0)	758 (237-1830)	1 (0-1)	17 (5-41)	0 (0-0)
2000	Andorra	4 (3-5)		115 (99-137)		3 (2-3)		75 (56-85)		0 (0-0)		2 (1-4)		0 (0-0)		3 (1-5)		0 (0-0)		7 (3-13)	
2001	Andorra									0 (0-0)		5 (2-8)		0 (0-0)		5 (2-9)		0 (0-1)		14 (6-25)	
2002	Andorra									0 (0-0)		4 (2-8)		0 (0-0)		5 (2-9)		0 (0-1)		14 (6-25)	
2003	Andorra									0 (0-0)		5 (2-8)		0 (0-0)		5 (2-9)		0 (0-1)		14 (6-25)	
2004	Andorra									0 (0-0)		4 (2-8)		0 (0-0)		5 (2-9)		0 (0-1)		13 (6-24)	
2005	Andorra									0 (0-0)		4 (2-8)		0 (0-0)		5 (2-9)		0 (0-1)		13 (6-24)	
2006	Andorra									0 (0-0)		2 (1-4)		0 (0-0)		2 (1-4)		0 (0-0)		6 (3-11)	
2007	Andorra									0 (0-0)		2 (1-3)		0 (0-0)		2 (1-4)		0 (0-0)		6 (3-10)	
2008	Andorra									0 (0-0)		1 (0-2)		0 (0-0)		1 (1-2)		0 (0-0)		3 (1-6)	
2009	Andorra									0 (0-0)		1 (0-1)		0 (0-0)		1 (0-2)		0 (0-0)		2 (1-5)	
2010	Andorra									0 (0-0)		1 (0-1)		0 (0-0)		1 (0-2)		0 (0-0)		2 (1-4)	
2011	Andorra									0 (0-0)		1 (0-1)		0 (0-0)		1 (0-1)		0 (0-0)		2 (1-4)	
2012	Andorra									0 (0-0)		0 (0-1)		0 (0-0)		0 (0-1)		0 (0-0)		1 (1-2)	
2013	Andorra									0 (0-0)		0 (0-1)		0 (0-0)		0 (0-1)		0 (0-0)		1 (0-2)	
2014	Andorra									0 (0-0)		0 (0-0)		0 (0-0)		0 (0-1)		0 (0-0)		1 (0-1)	
2015	Andorra	1 (1-1)	0 (0-0)	21 (18-25)	0 (0-0)	1 (0-1)	0 (0-0)	14 (10-16)	0 (0-0)	0 (0-0)	0 (0-0)	0 (0-0)	0 (0-0)	0 (0-0)	0 (0-0)	0 (0-1)	0 (0-0)	0 (0-0)	0 (0-0)	1 (0-1)	0 (0-0)
2000	Angola	67203 (57984-79885)		2289 (1975-2721)		26476 (19836-30199)		902 (676-1029)		1701 (896-3254)		58 (31-111)		682 (359-1305)		23 (12-44)		5136 (2706-9827)		175 (92-335)	
2001	Angola									1725 (910-3298)		57 (30-108)		692 (365-1323)		23 (12-43)		5208 (2747-9959)		171 (90-327)	
2002	Angola									1787 (944-3415)		57 (30-108)		717 (379-1370)		23 (12-43)		5397 (2850-10313)		171 (90-326)	
2003	Angola									1805 (954-3446)		55 (29-105)		724 (383-1382)		22 (12-42)		5450 (2881-10407)		167 (88-318)	
2004	Angola									1823 (965-3478)		54 (29-103)		731 (387-1395)		22 (11-41)		5505 (2913-10503)		163 (86-311)	
2005	Angola									1859 (985-3543)		53 (28-101)		746 (395-1421)		21 (11-41)		5614 (2973-10700)		160 (85-306)	
2006	Angola									1897 (1006-3612)		52 (28-99)		761 (404-1449)		21 (11-40)		5730 (3039-10908)		158 (84-300)	
2007	Angola									1928 (1024-3664)		51 (27-98)		773 (411-1470)		21 (11-39)		5821 (3092-11065)		155 (82-295)	
2008	Angola									1958 (1042-3717)		51 (27-96)		786 (418-1491)		20 (11-39)		5914 (3147-11225)		153 (81-290)	
2009	Angola									1968 (1049-3728)		49 (26-94)		789 (421-1495)		20 (11-38)		5942 (3169-11258)		149 (80-283)	
2010	Angola									1979 (1057-3742)		48 (26-91)		794 (424-1501)		19 (10-37)		5975 (3193-11299)		146 (78-276)	
2011	Angola									1976 (1058-3729)		47 (25-88)		792 (425-1496)		19 (10-35)		5966 (3196-11260)		141 (76-267)	
2012	Angola									1960 (1052-3691)		45 (24-85)		786 (422-1481)		18 (10-34)		5918 (3178-11146)		137 (73-257)	
2013	Angola									1858 (1001-3493)		42 (23-79)		745 (401-1401)		17 (9-32)		5611 (3021-10548)		127 (68-238)	
2014	Angola									1537 (830-2885)		34 (18-64)		617 (333-1157)		14 (7-26)		4642 (2506-8710)		102 (55-192)	
2015	Angola	40976 (35355-48709)	5467 (4717-6499)	883 (762-1050)	118 (102-140)	15971 (11966-18217)	2131 (1596-2430)	344 (258-393)	46 (34-52)	1324 (717-2481)	177 (96-331)	29 (15-53)	4 (2-7)	531 (288-995)	71 (38-133)	11 (6-21)	2 (1-3)	3998 (2167-7491)	533 (289-999)	86 (47-161)	11 (6-22)
2000	Antigua and Barbuda	111 (96-132)		1497 (1291-1779)		43 (32-49)		581 (436-663)		1 (1-2)		19 (9-29)		2 (1-2)		21 (11-33)		4 (2-7)		57 (28-88)	
2001	Antigua and Barbuda									4 (2-6)		48 (24-74)		4 (2-6)		55 (27-85)		11 (5-17)		145 (71-225)	
2002	Antigua and Barbuda									4 (2-6)		53 (26-82)		5 (2-7)		60 (30-93)		12 (6-19)		160 (79-248)	
2003	Antigua and Barbuda									3 (1-4)		34 (17-52)		3 (1-5)		38 (19-59)		8 (4-12)		102 (50-158)	
2004	Antigua and Barbuda									2 (1-3)		26 (13-41)		2 (1-4)		30 (15-46)		6 (3-9)		80 (39-123)	
2005	Antigua and Barbuda									2 (1-3)		28 (14-43)		2 (1-4)		32 (16-49)		6 (3-10)		84 (41-130)	
2006	Antigua and Barbuda									2 (1-3)		26 (13-41)		2 (1-3)		30 (15-46)		6 (3-9)		80 (39-123)	
2007	Antigua and Barbuda									0 (0-0)		0 (0-0)		0 (0-0)		0 (0-0)		0 (0-0)		0 (0-0)	
2008	Antigua and Barbuda									0 (0-0)		0 (0-0)		0 (0-0)		0 (0-0)		0 (0-0)		0 (0-0)	
2009	Antigua and Barbuda									0 (0-0)		0 (0-0)		0 (0-0)		0 (0-0)		0 (0-0)		0 (0-0)	
2010	Antigua and Barbuda									0 (0-0)		0 (0-0)		0 (0-0)		0 (0-0)		0 (0-0)		0 (0-0)	
2011	Antigua and Barbuda									0 (0-0)		0 (0-0)		0 (0-0)		0 (0-0)		0 (0-0)		0 (0-0)	
2012	Antigua and Barbuda									0 (0-0)		0 (0-0)		0 (0-0)		0 (0-0)		0 (0-0)		0 (0-0)	
2013	Antigua and Barbuda									0 (0-0)		0 (0-0)		0 (0-0)		0 (0-0)		0 (0-0)		0 (0-0)	
2014	Antigua and Barbuda									0 (0-0)		0 (0-0)		0 (0-0)		0 (0-0)		0 (0-0)		0 (0-0)	
2015	Antigua and Barbuda	66 (57-79)	0 (0-0)	929 (801-1104)	0 (0-0)	26 (19-30)	0 (0-0)	361 (271-412)	0 (0-0)	0 (0-0)	0 (0-0)	0 (0-0)	0 (0-0)	0 (0-0)	0 (0-0)	0 (0-0)	0 (0-0)	0 (0-0)	0 (0-0)	0 (0-0)	0 (0-0)
2000	Argentina	49467 (42681-58802)		1449 (1250-1722)		19239 (14413-21943)		564 (422-643)		546 (244-1119)		16 (7-33)		621 (278-1271)		18 (8-37)		1650 (738-3379)		48 (22-99)	
2001	Argentina									493 (221-1008)		14 (7-30)		560 (252-1145)		16 (7-34)		1490 (669-3045)		44 (20-89)	
2002	Argentina									535 (241-1092)		16 (7-32)		608 (274-1240)		18 (8-36)		1617 (728-3297)		47 (21-96)	
2003	Argentina									633 (286-1288)		18 (8-37)		719 (325-1463)		21 (9-42)		1912 (864-3889)		55 (25-112)	
2004	Argentina									643 (292-1302)		18 (8-37)		730 (331-1479)		21 (9-42)		1941 (881-3932)		55 (25-112)	
2005	Argentina									581 (265-1171)		16 (7-33)		660 (301-1330)		19 (9-38)		1753 (800-3537)		50 (23-100)	
2006	Argentina									558 (256-1122)		16 (7-31)		634 (291-1275)		18 (8-36)		1686 (772-3388)		47 (22-95)	
2007	Argentina									467 (215-936)		13 (6-26)		531 (244-1063)		15 (7-30)		1412 (649-2827)		39 (18-79)	
2008	Argentina									480 (221-959)		13 (6-27)		546 (251-1089)		15 (7-30)		1451 (668-2896)		40 (19-81)	
2009	Argentina									468 (216-931)		13 (6-26)		531 (245-1058)		15 (7-30)		1412 (652-2812)		39 (18-79)	
2010	Argentina									519 (240-1031)		14 (7-29)		589 (273-1171)		16 (8-33)		1567 (725-3113)		44 (20-87)	
2011	Argentina									382 (177-758)		11 (5-21)		434 (202-861)		12 (6-24)		1155 (536-2289)		32 (15-64)	
2012	Argentina									361 (167-713)		10 (5-20)		410 (190-810)		11 (5-23)		1089 (504-2154)		30 (14-60)	
2013	Argentina									242 (113-478)		7 (3-13)		275 (128-543)		8 (4-15)		731 (340-1443)		20 (9-40)	
2014	Argentina									219 (102-431)		6 (3-12)		249 (116-490)		7 (3-13)		661 (309-1302)		18 (8-36)	
2015	Argentina	16778 (14476-19944)	86 (74-103)	459 (396-545)	2 (2-3)	6543 (4902-7463)	34 (25-38)	179 (134-204)	1 (1-1)	213 (100-419)	1 (1-2)	6 (3-11)	0 (0-0)	242 (114-476)	1 (1-2)	7 (3-13)	0 (0-0)	644 (302-1267)	3 (2-7)	18 (8-35)	0 (0-0)
2000	Armenia	446 (385-530)		230 (198-273)		289 (217-330)		149 (112-170)		10 (3-23)		5 (1-12)		11 (3-26)		6 (1-14)		29 (8-70)		15 (4-36)	
2001	Armenia									9 (2-26)		5 (1-14)		10 (2-30)		6 (1-16)		28 (5-79)		15 (3-43)	
2002	Armenia									9 (2-26)		5 (1-14)		10 (2-29)		6 (1-16)		27 (5-77)		15 (3-42)	
2003	Armenia									9 (2-26)		5 (1-14)		10 (2-29)		6 (1-16)		27 (5-77)		15 (3-41)	
2004	Armenia									9 (2-26)		5 (1-13)		10 (2-29)		5 (1-15)		27 (5-77)		14 (3-41)	
2005	Armenia									9 (2-25)		5 (1-13)		10 (2-29)		5 (1-15)		27 (5-76)		14 (3-39)	
2006	Armenia									9 (2-25)		4 (1-12)		10 (2-28)		5 (1-14)		27 (5-75)		13 (3-37)	
2007	Armenia									9 (2-25)		4 (1-12)		10 (2-28)		5 (1-13)		27 (6-75)		13 (3-36)	
2008	Armenia									9 (2-24)		4 (1-11)		10 (2-27)		5 (1-13)		26 (6-71)		13 (3-34)	
2009	Armenia									9 (2-23)		4 (1-11)		10 (2-26)		5 (1-12)		26 (6-70)		12 (3-33)	
2010	Armenia									13 (3-34)		6 (1-16)		15 (4-38)		7 (2-18)		39 (10-102)		18 (5-48)	
2011	Armenia									17 (5-44)		8 (2-21)		20 (5-50)		9 (2-24)		52 (14-134)		25 (7-63)	
2012	Armenia									17 (5-42)		8 (2-20)		19 (5-48)		9 (3-23)		51 (14-128)		24 (7-61)	
2013	Armenia									16 (5-40)		8 (2-19)		18 (6-45)		9 (3-22)		49 (15-120)		23 (7-58)	
2014	Armenia									16 (5-37)		8 (3-18)		18 (6-43)		9 (3-21)		47 (16-113)		23 (8-55)	
2015	Armenia	399 (344-474)	1 (1-1)	196 (169-233)	0 (0-0)	259 (194-295)	0 (0-0)	127 (95-145)	0 (0-0)	13 (5-30)	0 (0-0)	6 (2-15)	0 (0-0)	15 (5-34)	0 (0-0)	7 (3-17)	0 (0-0)	39 (15-91)	0 (0-0)	19 (7-45)	0 (0-0)
2000	Australia	2421 (2089-2878)		192 (165-228)		1570 (1176-1790)		124 (93-142)		97 (43-171)		8 (3-14)		110 (49-194)		9 (4-15)		292 (130-516)		23 (10-41)	
2001	Australia									141 (63-250)		11 (5-20)		161 (72-284)		13 (6-23)		427 (191-756)		34 (15-61)	
2002	Australia									49 (22-87)		4 (2-7)		56 (25-99)		5 (2-8)		148 (66-262)		12 (5-21)	
2003	Australia									100 (45-177)		8 (4-14)		114 (51-201)		9 (4-16)		302 (135-534)		25 (11-43)	
2004	Australia									108 (48-191)		9 (4-15)		122 (55-217)		10 (4-18)		326 (146-576)		26 (12-47)	
2005	Australia									73 (33-129)		6 (3-10)		83 (37-146)		7 (3-12)		220 (98-389)		18 (8-31)	
2006	Australia									61 (27-108)		5 (2-9)		70 (31-123)		5 (2-10)		185 (83-327)		15 (6-26)	
2007	Australia									31 (14-55)		2 (1-4)		35 (16-62)		3 (1-5)		94 (42-166)		7 (3-13)	
2008	Australia									37 (17-66)		3 (1-5)		42 (19-75)		3 (1-6)		113 (50-200)		8 (4-15)	
2009	Australia									19 (8-34)		1 (1-2)		22 (10-38)		2 (1-3)		57 (26-101)		4 (2-7)	
2010	Australia									57 (25-100)		4 (2-7)		64 (29-114)		4 (2-8)		171 (76-303)		12 (5-21)	
2011	Australia									31 (14-56)		2 (1-4)		36 (16-63)		2 (1-4)		95 (42-168)		6 (3-11)	
2012	Australia									19 (8-33)		1 (1-2)		21 (9-38)		1 (1-3)		56 (25-100)		4 (2-7)	
2013	Australia									14 (6-25)		1 (0-2)		16 (7-29)		1 (0-2)		43 (19-77)		3 (1-5)	
2014	Australia									13 (6-22)		1 (0-1)		14 (6-25)		1 (0-2)		38 (17-68)		3 (1-4)	
2015	Australia	769 (663-914)	1 (1-1)	51 (44-60)	0 (0-0)	498 (373-568)	1 (0-1)	33 (25-37)	0 (0-0)	12 (5-21)	0 (0-0)	1 (0-1)	0 (0-0)	14 (6-24)	0 (0-0)	1 (0-2)	0 (0-0)	36 (16-64)	0 (0-0)	2 (1-4)	0 (0-0)
2000	Austria	470 (406-559)		115 (99-137)		305 (228-348)		75 (56-85)		18 (8-32)		4 (2-8)		20 (9-37)		5 (2-9)		54 (24-98)		13 (6-24)	
2001	Austria									19 (9-35)		5 (2-9)		22 (10-40)		6 (3-10)		58 (26-106)		15 (7-27)	
2002	Austria									12 (5-22)		3 (1-6)		14 (6-25)		4 (2-6)		36 (16-66)		9 (4-17)	
2003	Austria									20 (9-37)		5 (2-9)		23 (10-42)		6 (3-11)		61 (28-111)		16 (7-29)	
2004	Austria									14 (6-25)		4 (2-6)		16 (7-29)		4 (2-7)		42 (19-76)		11 (5-20)	
2005	Austria									18 (8-33)		5 (2-8)		21 (9-37)		5 (2-10)		55 (25-100)		14 (6-25)	
2006	Austria									16 (7-28)		4 (2-7)		18 (8-32)		5 (2-8)		47 (21-85)		12 (5-22)	
2007	Austria									4 (2-8)		1 (0-2)		5 (2-9)		1 (1-2)		13 (6-23)		3 (1-6)	
2008	Austria									22 (10-40)		6 (3-10)		25 (11-45)		6 (3-12)		66 (30-121)		17 (8-31)	
2009	Austria									26 (12-48)		7 (3-12)		30 (14-54)		8 (4-14)		79 (36-144)		21 (9-37)	
2010	Austria									23 (11-43)		6 (3-11)		27 (12-48)		7 (3-13)		71 (32-129)		18 (8-34)	
2011	Austria									10 (5-18)		3 (1-5)		12 (5-21)		3 (1-5)		31 (14-56)		8 (4-15)	
2012	Austria									20 (9-37)		5 (2-10)		23 (10-42)		6 (3-11)		61 (28-112)		16 (7-29)	
2013	Austria									23 (11-42)		6 (3-11)		26 (12-48)		7 (3-12)		70 (32-128)		18 (8-33)	
2014	Austria									7 (3-12)		2 (1-3)		8 (3-14)		2 (1-3)		20 (9-36)		5 (2-9)	
2015	Austria	457 (394-543)	0 (0-0)	115 (99-137)	0 (0-0)	296 (222-338)	0 (0-0)	75 (56-85)	0 (0-0)	15 (7-27)	0 (0-0)	4 (2-7)	0 (0-0)	17 (8-31)	0 (0-0)	4 (2-8)	0 (0-0)	46 (21-83)	0 (0-0)	12 (5-21)	0 (0-0)
2000	Azerbaijan	1581 (1364-1879)		230 (198-273)		1025 (768-1169)		149 (112-170)		103 (29-244)		15 (4-36)		117 (33-277)		17 (5-40)		310 (89-738)		45 (13-107)	
2001	Azerbaijan									88 (25-208)		14 (4-32)		100 (29-236)		15 (4-36)		265 (77-628)		41 (12-97)	
2002	Azerbaijan									77 (22-181)		12 (4-29)		87 (25-205)		14 (4-33)		231 (68-545)		37 (11-88)	
2003	Azerbaijan									68 (20-159)		11 (3-26)		77 (23-181)		13 (4-30)		205 (61-482)		34 (10-79)	
2004	Azerbaijan									61 (18-144)		10 (3-24)		70 (21-164)		12 (3-27)		186 (56-435)		31 (9-72)	
2005	Azerbaijan									56 (17-130)		9 (3-22)		63 (19-148)		11 (3-25)		168 (51-393)		28 (8-65)	
2006	Azerbaijan									51 (16-119)		8 (3-20)		58 (18-136)		10 (3-22)		155 (47-360)		25 (8-59)	
2007	Azerbaijan									47 (15-110)		8 (2-18)		54 (17-125)		9 (3-20)		143 (44-331)		23 (7-53)	

Spn = *Streptococcus pneumoniae*; npnm = non-pneumonia, non-meningitis; HIV = cases only in HIV-infected children; Uncertainty range provided in parentheses.

Year	Country	Spn pneumonia cases (UR)	Spn pneumonia cases, HIV (UR)	Spn pneumonia incidence rate (UR)	Spn pneumonia incidence rate, HIV (UR)	Spn severe pneumonia cases (UR)	Spn severe pneumonia cases, HIV (UR)	Spn severe pneumonia incidence rate (UR)	Spn severe pneumonia incidence rate, HIV (UR)	Spn meningitis cases (UR)	Spn meningitis cases, HIV (UR)	Spn meningitis incidence rate (UR)	Spn meningitis incidence rate, HIV (UR)	Spn NPNM cases (UR)	Spn NPNM cases, HIV (UR)	Spn NPNM incidence rate (UR)	Spn NPNM incidence rate, HIV (UR)	Spn NPNM non-severe cases (UR)	Spn NPNM non-severe cases, HIV (UR)	SPN NPNM non-severe incidence rate (UR)	SPN NPNM non-severe incidence rate, HIV (UR)
2008	Azerbaijan									45 (14-103)		7 (2-16)		51 (16-117)		8 (2-18)		135 (42-311)		21 (7-49)	
2009	Azerbaijan									43 (13-99)		6 (2-15)		49 (15-113)		7 (2-17)		130 (41-299)		19 (6-45)	
2010	Azerbaijan									43 (14-98)		6 (2-14)		49 (15-112)		7 (2-16)		129 (41-297)		18 (6-42)	
2011	Azerbaijan									44 (14-101)		6 (2-14)		50 (16-114)		7 (2-16)		133 (42-304)		18 (6-41)	
2012	Azerbaijan									45 (15-104)		6 (2-13)		52 (17-118)		7 (2-15)		137 (44-314)		17 (6-40)	
2013	Azerbaijan									47 (15-108)		6 (2-13)		54 (17-122)		6 (2-15)		142 (46-325)		17 (5-39)	
2014	Azerbaijan									33 (11-75)		4 (1-9)		38 (12-86)		4 (1-10)		100 (32-228)		11 (4-26)	
2015	Azerbaijan	1337 (1154-1590)	4 (4-5)	146 (126-174)	0 (0-1)	867 (649-989)	3 (2-3)	95 (71-108)	0 (0-0)	23 (8-53)	0 (0-0)	3 (1-6)	0 (0-0)	27 (9-61)	0 (0-0)	3 (1-7)	0 (0-0)	71 (23-161)	0 (0-1)	8 (3-18)	0 (0-0)
2000	Bahamas	404 (348-480)		1458 (1258-1734)		157 (118-179)		568 (426-648)		3 (2-5)		11 (6-17)		4 (2-5)		13 (6-20)		9 (5-15)		34 (17-53)	
2001	Bahamas									4 (2-6)		16 (8-24)		5 (2-7)		18 (9-27)		12 (6-19)		47 (23-73)	
2002	Bahamas									4 (2-6)		16 (8-24)		5 (2-7)		18 (9-28)		12 (6-19)		48 (23-74)	
2003	Bahamas									2 (1-3)		8 (4-13)		2 (1-4)		9 (5-14)		6 (3-10)		25 (12-38)	
2004	Bahamas									2 (1-3)		7 (3-10)		2 (1-3)		7 (4-12)		5 (3-8)		20 (10-31)	
2005	Bahamas									2 (1-3)		7 (3-10)		2 (1-3)		8 (4-12)		5 (3-8)		20 (10-31)	
2006	Bahamas									2 (1-3)		7 (3-10)		2 (1-3)		8 (4-12)		5 (3-8)		20 (10-31)	
2007	Bahamas									0 (0-0)		0 (0-0)		0 (0-0)		0 (0-0)		0 (0-0)		0 (0-0)	
2008	Bahamas									0 (0-0)		0 (0-0)		0 (0-0)		0 (0-0)		0 (0-0)		0 (0-0)	
2009	Bahamas									2 (1-4)		9 (4-14)		3 (1-4)		10 (5-16)		7 (3-11)		27 (13-42)	
2010	Bahamas									2 (1-4)		9 (4-14)		3 (1-4)		10 (5-16)		7 (4-11)		27 (13-42)	
2011	Bahamas									2 (1-4)		8 (4-13)		3 (1-4)		10 (5-15)		7 (3-11)		25 (12-39)	
2012	Bahamas									1 (0-1)		2 (1-4)		1 (0-1)		3 (1-4)		2 (1-3)		7 (3-11)	
2013	Bahamas									1 (0-1)		3 (1-4)		1 (0-1)		3 (2-5)		2 (1-4)		8 (4-13)	
2014	Bahamas									1 (0-1)		3 (2-5)		1 (1-2)		4 (2-6)		3 (1-4)		10 (5-15)	
2015	Bahamas	82 (71-97)	3 (3-4)	286 (247-340)	12 (10-14)	32 (24-36)	1 (1-1)	112 (84-127)	5 (3-5)	1 (0-1)	0 (0-0)	3 (1-4)	0 (0-0)	1 (0-1)	0 (0-0)	3 (2-5)	0 (0-0)	2 (1-4)	0 (0-0)	8 (4-13)	0 (0-1)
2000	Bahrain	1638 (1414-1947)		2299 (1984-2733)		644 (482-734)		903 (677-1031)		4 (2-5)		5 (3-7)		4 (2-6)		6 (3-8)		11 (7-15)		15 (9-21)	
2001	Bahrain									2 (1-3)		3 (2-5)		3 (2-4)		4 (2-5)		7 (4-10)		10 (6-14)	
2002	Bahrain									8 (5-11)		11 (7-16)		9 (5-12)		13 (8-18)		24 (14-33)		34 (20-47)	
2003	Bahrain									6 (3-8)		8 (5-11)		6 (4-9)		9 (5-12)		17 (10-23)		23 (14-32)	
2004	Bahrain									10 (6-14)		14 (8-19)		12 (7-16)		16 (9-22)		31 (19-43)		42 (25-57)	
2005	Bahrain									10 (6-14)		13 (8-18)		12 (7-16)		15 (9-21)		31 (19-43)		40 (24-55)	
2006	Bahrain									22 (13-31)		29 (17-40)		25 (15-35)		33 (20-45)		67 (40-93)		87 (52-119)	
2007	Bahrain									21 (13-29)		26 (16-36)		24 (14-33)		30 (18-41)		63 (38-87)		79 (48-110)	
2008	Bahrain									15 (9-21)		18 (11-25)		17 (10-23)		20 (12-28)		45 (27-62)		54 (33-75)	
2009	Bahrain									4 (3-6)		5 (3-7)		5 (3-7)		6 (4-8)		13 (8-18)		16 (9-22)	
2010	Bahrain									2 (1-3)		2 (1-3)		2 (1-3)		3 (2-4)		6 (4-9)		7 (4-10)	
2011	Bahrain									8 (5-11)		8 (5-11)		9 (5-12)		9 (6-13)		23 (14-32)		25 (15-35)	
2012	Bahrain									5 (3-6)		5 (3-6)		5 (3-7)		5 (3-7)		14 (8-19)		14 (8-19)	
2013	Bahrain									6 (3-8)		6 (3-8)		7 (4-9)		6 (4-9)		17 (10-24)		17 (10-23)	
2014	Bahrain									3 (2-4)		3 (2-3)		3 (2-4)		3 (2-4)		8 (5-11)		8 (5-11)	
2015	Bahrain	350 (302-416)	0 (0-0)	327 (283-389)	0 (0-0)	136 (102-156)	0 (0-0)	128 (96-145)	0 (0-0)	4 (2-5)	0 (0-0)	4 (2-5)	0 (0-0)	4 (3-6)	0 (0-0)	4 (2-6)	0 (0-0)	12 (7-16)	0 (0-0)	11 (7-15)	0 (0-0)
2000	Bangladesh	455932 (393388-541975)		2763 (2384-3285)		179129 (134202-204314)		1086 (813-1238)		3559 (1154-8411)		22 (7-51)		1428 (463-3374)		9 (3-20)		10747 (3484-25399)		65 (21-154)	
2001	Bangladesh									3315 (1061-7869)		20 (6-47)		1330 (426-3156)		8 (3-19)		10010 (3204-23762)		60 (19-143)	
2002	Bangladesh									2949 (932-7030)		18 (6-42)		1183 (374-2820)		7 (2-17)		8904 (2814-21228)		54 (17-128)	
2003	Bangladesh									2723 (689-6717)		16 (4-40)		3093 (782-7629)		19 (5-46)		8222 (2079-20282)		50 (13-122)	
2004	Bangladesh									2521 (627-6234)		15 (4-38)		2863 (713-7080)		17 (4-43)		7612 (1895-18824)		46 (11-114)	
2005	Bangladesh									2425 (631-5942)		15 (4-36)		2755 (716-6749)		17 (4-41)		7323 (1904-17942)		45 (12-109)	
2006	Bangladesh									2263 (615-5484)		14 (4-34)		2570 (699-6228)		16 (4-38)		6834 (1857-16558)		42 (11-101)	
2007	Bangladesh									2108 (613-5028)		13 (4-31)		2394 (696-5711)		15 (4-35)		6364 (1850-15183)		39 (11-94)	
2008	Bangladesh									1918 (554-4568)		12 (3-29)		2179 (629-5189)		14 (4-33)		5792 (1671-13794)		36 (10-87)	
2009	Bangladesh									1728 (496-4113)		11 (3-26)		1963 (563-4672)		13 (4-30)		5218 (1497-12420)		33 (10-79)	
2010	Bangladesh									1560 (445-3716)		10 (3-24)		1772 (506-4221)		11 (3-27)		4712 (1345-11222)		30 (9-72)	
2011	Bangladesh									1412 (401-3363)		9 (3-22)		1603 (455-3820)		10 (3-25)		4262 (1211-10156)		28 (8-66)	
2012	Bangladesh									1303 (372-3098)		9 (2-20)		1480 (422-3518)		10 (3-23)		3934 (1123-9354)		26 (7-62)	
2013	Bangladesh									1209 (345-2871)		8 (2-19)		1373 (392-3261)		9 (3-22)		3651 (1041-8669)		24 (7-57)	
2014	Bangladesh									1142 (347-2675)		8 (2-18)		1297 (394-3038)		9 (3-20)		3447 (1048-8078)		23 (7-53)	
2015	Bangladesh	259285 (223717-308217)	139 (120-165)	1720 (1484-2044)	1 (1-1)	101842 (76300-116161)	54 (41-62)	676 (506-771)	0 (0-0)	822 (251-1919)	0 (0-1)	5 (2-13)	0 (0-0)	933 (286-2180)	0 (0-1)	6 (2-14)	0 (0-0)	2481 (759-5795)	1 (0-3)	16 (5-38)	0 (0-0)
2000	Barbados	265 (229-315)		1440 (1243-1712)		103 (77-118)		561 (420-640)		6 (3-9)		31 (15-49)		7 (3-10)		36 (18-55)		17 (9-27)		95 (47-147)	
2001	Barbados									4 (2-6)		22 (11-35)		5 (2-7)		25 (12-39)		12 (6-19)		67 (33-104)	
2002	Barbados									3 (2-5)		18 (9-28)		4 (2-6)		21 (10-32)		10 (5-15)		55 (27-85)	
2003	Barbados									0 (0-0)		0 (0-0)		0 (0-0)		0 (0-0)		0 (0-0)		0 (0-0)	
2004	Barbados									0 (0-0)		0 (0-0)		0 (0-0)		0 (0-0)		0 (0-0)		0 (0-0)	
2005	Barbados									0 (0-0)		0 (0-0)		0 (0-0)		0 (0-0)		0 (0-0)		0 (0-0)	
2006	Barbados									0 (0-0)		0 (0-0)		0 (0-0)		0 (0-0)		0 (0-0)		0 (0-0)	
2007	Barbados									0 (0-0)		0 (0-0)		0 (0-0)		0 (0-0)		0 (0-0)		0 (0-0)	
2008	Barbados									0 (0-0)		0 (0-0)		0 (0-0)		0 (0-0)		0 (0-0)		0 (0-0)	
2009	Barbados									0 (0-0)		0 (0-0)		0 (0-0)		0 (0-0)		0 (0-0)		0 (0-0)	
2010	Barbados									0 (0-0)		0 (0-0)		0 (0-0)		0 (0-0)		0 (0-0)		0 (0-0)	
2011	Barbados									0 (0-0)		0 (0-0)		0 (0-0)		0 (0-0)		0 (0-0)		0 (0-0)	
2012	Barbados									0 (0-0)		0 (0-0)		0 (0-0)		0 (0-0)		0 (0-0)		0 (0-0)	
2013	Barbados									0 (0-0)		0 (0-0)		0 (0-0)		0 (0-0)		0 (0-0)		0 (0-0)	
2014	Barbados									0 (0-0)		0 (0-0)		0 (0-0)		0 (0-0)		0 (0-0)		0 (0-0)	
2015	Barbados	49 (42-58)	0 (0-0)	284 (245-338)	1 (1-1)	19 (14-22)	0 (0-0)	111 (83-127)	0 (0-0)	0 (0-0)	0 (0-0)	0 (0-0)	0 (0-0)	0 (0-0)	0 (0-0)	0 (0-0)	0 (0-0)	0 (0-0)	0 (0-0)	0 (0-0)	0 (0-0)
2000	Belarus	2085 (1799-2478)		460 (397-547)		1352 (1013-1542)		298 (223-340)		19 (7-60)		4 (2-13)		22 (8-68)		5 (2-15)		59 (21-182)		13 (5-40)	
2001	Belarus									17 (6-53)		4 (1-12)		20 (7-60)		4 (2-14)		52 (19-161)		12 (4-36)	
2002	Belarus									16 (6-48)		4 (1-11)		18 (7-54)		4 (2-12)		47 (18-144)		11 (4-33)	
2003	Belarus									14 (5-43)		3 (1-10)		16 (6-49)		4 (1-11)		43 (17-129)		10 (4-29)	
2004	Belarus									14 (5-41)		3 (1-9)		16 (6-47)		4 (1-11)		42 (16-124)		10 (4-28)	
2005	Belarus									13 (5-38)		3 (1-9)		15 (6-43)		3 (1-10)		40 (16-115)		9 (4-26)	
2006	Belarus									13 (5-36)		3 (1-8)		14 (6-41)		3 (1-9)		38 (15-109)		8 (3-24)	
2007	Belarus									12 (5-33)		3 (1-7)		13 (5-38)		3 (1-8)		36 (14-100)		8 (3-21)	
2008	Belarus									12 (5-34)		3 (1-7)		14 (6-39)		3 (1-8)		37 (15-103)		8 (3-22)	
2009	Belarus									14 (6-36)		3 (1-8)		15 (6-41)		3 (1-9)		41 (17-110)		8 (4-23)	
2010	Belarus									10 (4-26)		2 (1-5)		11 (5-29)		2 (1-6)		29 (12-77)		6 (2-15)	
2011	Belarus									11 (5-28)		2 (1-5)		12 (5-32)		2 (1-6)		32 (14-84)		6 (3-16)	
2012	Belarus									11 (5-29)		2 (1-5)		13 (6-33)		2 (1-6)		34 (15-87)		6 (3-16)	
2013	Belarus									11 (5-27)		2 (1-5)		12 (6-31)		2 (1-6)		33 (15-82)		6 (3-15)	
2014	Belarus									10 (4-24)		2 (1-4)		11 (5-28)		2 (1-5)		30 (14-73)		5 (2-13)	
2015	Belarus	2652 (2288-3153)	2 (2-2)	460 (397-547)	0 (0-0)	1719 (1288-1961)	1 (1-2)	298 (223-340)	0 (0-0)	9 (4-23)	0 (0-0)	2 (1-4)	0 (0-0)	11 (5-26)	0 (0-0)	2 (1-4)	0 (0-0)	29 (13-69)	0 (0-0)	5 (2-12)	0 (0-0)
2000	Belgium	649 (560-772)		115 (99-137)		421 (315-480)		75 (56-85)		75 (34-136)		13 (6-24)		85 (39-154)		15 (7-27)		226 (103-410)		40 (18-73)	
2001	Belgium									93 (42-168)		17 (8-30)		105 (48-191)		19 (9-34)		280 (127-509)		51 (23-92)	
2002	Belgium									64 (29-116)		12 (5-21)		72 (33-131)		13 (6-24)		192 (87-349)		35 (16-63)	
2003	Belgium									64 (29-117)		12 (5-21)		73 (33-133)		13 (6-24)		195 (88-353)		35 (16-63)	
2004	Belgium									82 (37-149)		15 (7-26)		93 (42-170)		17 (7-30)		248 (113-451)		44 (20-80)	
2005	Belgium									67 (31-122)		12 (5-21)		76 (35-139)		13 (6-24)		203 (92-369)		35 (16-64)	
2006	Belgium									76 (35-139)		13 (6-24)		87 (39-158)		15 (7-27)		231 (105-419)		39 (18-72)	
2007	Belgium									67 (30-122)		11 (5-20)		76 (35-138)		13 (6-23)		202 (92-367)		34 (15-62)	
2008	Belgium									47 (21-86)		8 (4-14)		54 (24-98)		9 (4-16)		143 (65-260)		24 (11-43)	
2009	Belgium									46 (21-84)		8 (3-14)		53 (24-96)		9 (4-16)		140 (64-255)		23 (10-41)	
2010	Belgium									20 (9-36)		3 (1-6)		22 (10-41)		4 (2-7)		60 (27-108)		10 (4-17)	
2011	Belgium									28 (12-50)		4 (2-8)		31 (14-57)		5 (2-9)		83 (38-151)		13 (6-24)	
2012	Belgium									24 (11-43)		4 (2-7)		27 (12-49)		4 (2-8)		72 (32-130)		11 (5-20)	
2013	Belgium									14 (6-26)		2 (1-4)		16 (7-29)		3 (1-5)		43 (20-78)		7 (3-12)	
2014	Belgium									13 (6-23)		2 (1-4)		14 (6-26)		2 (1-4)		38 (17-69)		6 (3-11)	
2015	Belgium	306 (264-364)	0 (0-0)	48 (41-57)	0 (0-0)	198 (149-226)	0 (0-0)	31 (23-35)	0 (0-0)	11 (5-20)	0 (0-0)	2 (1-3)	0 (0-0)	13 (6-23)	0 (0-0)	2 (1-4)	0 (0-0)	34 (15-61)	0 (0-0)	5 (2-10)	0 (0-0)
2000	Belize	686 (592-815)		1875 (1617-2228)		268 (201-306)		733 (549-836)		3 (1-10)		9 (2-29)		4 (1-12)		10 (2-33)		10 (2-32)		27 (5-86)	
2001	Belize									6 (1-18)		15 (3-48)		6 (1-20)		17 (4-54)		17 (3-54)		46 (9-144)	
2002	Belize									9 (2-27)		23 (5-71)		10 (2-30)		26 (6-81)		26 (6-81)		69 (15-216)	
2003	Belize									2 (0-6)		5 (1-17)		2 (1-7)		6 (1-19)		6 (1-19)		16 (4-50)	
2004	Belize									5 (1-15)		14 (3-41)		6 (1-18)		15 (4-47)		15 (4-47)		41 (9-124)	
2005	Belize									0 (0-0)		0 (0-0)		0 (0-0)		0 (0-0)		0 (0-0)		0 (0-0)	
2006	Belize									1 (0-4)		3 (1-10)		1 (0-4)		4 (1-11)		4 (1-11)		10 (3-30)	
2007	Belize									4 (1-10)		10 (3-28)		4 (1-12)		11 (3-32)		11 (3-31)		29 (8-84)	
2008	Belize									0 (0-0)		0 (0-0)		0 (0-0)		0 (0-0)		0 (0-0)		0 (0-0)	
2009	Belize									0 (0-0)		0 (0-0)		0 (0-0)		0 (0-0)		0 (0-0)		0 (0-0)	
2010	Belize									3 (1-7)		7 (2-19)		3 (1-8)		8 (2-21)		8 (2-21)		21 (6-56)	
2011	Belize									0 (0-0)		0 (0-0)		0 (0-0)		0 (0-0)		0 (0-0)		0 (0-0)	
2012	Belize									4 (1-9)		10 (3-24)		4 (1-10)		11 (4-28)		11 (4-27)		29 (10-74)	
2013	Belize									8 (3-19)		21 (8-50)		9 (3-21)		23 (9-57)		23 (9-57)		62 (23-151)	
2014	Belize									4 (2-10)		11 (5-27)		5 (2-12)		13 (5-30)		13 (5-31)		34 (14-81)	
2015	Belize	476 (410-565)	9 (7-10)	1226 (1058-1457)	22 (19-26)	186 (139-212)	3 (3-4)	479 (359-546)	9 (6-10)	5 (2-10)	0 (0-0)	12 (5-27)	0 (0-0)	5 (2-12)	0 (0-0)	13 (6-31)	0 (0-1)	14 (6-31)	0 (0-1)	36 (16-81)	1 (0-1)
2000	Benin	48793 (42099-58001)		3984 (3438-4736)		19006 (14239-21678)		1552 (1163-1770)		554 (202-1230)		45 (17-100)		222 (81-493)		18 (7-40)		1673 (611-3713)		137 (50-303)	
2001	Benin									540 (198-1198)		43 (16-95)		217 (79-481)		17 (6-38)		1632 (598-3618)		130 (48-288)	
2002	Benin									535 (196-1185)		41 (15-92)		215 (79-475)		17 (6-37)		1615 (593-3579)		125 (46-277)	
2003	Benin									528 (194-1169)		40 (15-88)		212 (78-469)		16 (6-35)		1594 (586-3529)		120 (44-266)	
2004	Benin									518 (191-1147)		38 (14-84)		208 (77-460)		15 (6-34)		1566 (577-3465)		115 (42-255)	
2005	Benin									511 (188-1130)		37 (14-81)		205 (76-453)		15 (5-33)		1542 (569-3411)		111 (41-245)	
2006	Benin									521 (193-1153)		36 (13-81)		209 (77-462)		15 (5-32)		1573 (582-3481)		110 (41-243)	
2007	Benin									525 (192-1164)		36 (13-79)		210 (77-467)		14 (5-32)		1585 (581-3515)		108 (40-240)	
2008	Benin									532 (194-1184)		36 (13-79)		214 (78-475)		14 (5-32)		1607 (585-3576)		108 (39-239)	
2009	Benin									541 (195-1207)		36 (13-79)		217 (78-484)		14 (5-32)		1633 (590-3644)		107 (39-240)	
2010	Benin									547 (196-1224)		35 (13-79)		219 (79-491)		14 (5-32)		1652 (592-3695)		107 (38-239)	
2011	Benin									466 (166-1046)		30 (11-66)		187 (67-419)		12 (4-27)		1408 (501-3157)		89 (32-200)	
2012	Benin									342 (122-768)		21 (8-48)		137 (49-308)		9 (3-19)		1034 (368-2319)		64 (23-145)	
2013	Benin									332 (118-744)		20 (7-46)		133 (47-298)		8 (3-18)		1001 (358-2245)		61 (22-138)	
2014	Benin									316 (113-708)		19 (7-43)		127 (45-284)		8 (3-17)		954 (342-2137)		58 (21-129)	
2015	Benin	16758 (14459-19920)	881 (760-1047)	998 (861-1186)	52 (45-62)	6559 (4914-7481)	345 (258-393)	391 (293-445)	21 (15-23)	195 (70-436)	10 (4-23)	12 (4-26)	1 (0-1)	78 (28-175)	4 (1-9)	5 (2-10)	0 (0-1)	588 (211-1316)	31 (11-69)	35 (13-78)	2 (1-4)
2000	Bhutan	1223 (1055-1454)		1652 (1426-1964)		476 (357-543)		643 (482-734)		22 (10-45)		30 (13-60)		9 (4-18)		12 (5-24)		67 (29-134)		90 (40-182)	
2001	Bhutan									20 (9-41)		28 (12-55)		8 (4-16)		11 (5-22)		61 (27-123)		83 (37-168)	
2002	Bhutan									19 (7-39)		26 (10-53)		21 (8-44)		29 (11-60)		56 (22-117)		77 (31-160)	
2003	Bhutan									17 (7-35)		24 (9-49)		19 (8-40)		27 (11-56)		51 (21-106)		72 (29-148)	
2004	Bhutan									15 (6-32)		22 (9-45)		18 (7-36)		25 (10-51)		47 (19-96)		66 (27-136)	
2005	Bhutan									14 (6-29)		20 (8-41)		16 (7-33)		23 (9-47)		43 (18-88)		61 (25-125)	
2006	Bhutan									13 (5-27)		19 (8-38)		15 (6-30)		21 (9-43)		39 (16-80)		56 (23-114)	
2007	Bhutan									12 (5-24)		17 (7-35)		14 (6-28)		19 (8-39)		36 (15-73)		52 (22-104)	
2008	Bhutan									11 (5-22)		16 (7-31)		13 (5-25)		18 (8-36)		34 (14-67)		47 (20-95)	
2009	Bhutan									10 (4-20)		14 (6-29)		12 (5-23)		16 (7-33)		31 (13-61)		43 (19-86)	
2010	Bhutan									9 (4-18)		13 (6-26)		11 (5-21)		15 (6-30)		28 (12-56)		40 (17-79)	
2011	Bhutan									8 (4-17)		12 (5-23)		10 (4-19)		13 (6-27)		25 (11-50)		36 (16-71)	
2012	Bhutan									8 (3-15)		11 (5-21)		9 (4-17)		12 (5-24)		23 (10-45)		33 (14-64)	
2013	Bhutan									7 (3-13)		10 (4-20)		8 (3-15)		11 (5-22)		20 (9-40)		30 (13-59)	
2014	Bhutan									6 (3-12)		9 (4-18)		7 (3-14)		11 (5-21)		19 (8-36)		28 (13-55)	
2015	Bhutan	834 (720-992)	31 (26-36)	1287 (1110-1529)	47 (41-56)	325 (244-371)	12 (9-14)	501 (376-572)	18 (14-21)	6 (3-11)	0 (0-0)	9 (4-17)	0 (0-1)	6 (3-13)	0 (0-0)	10 (5-19)	0 (0-1)	17 (8-34)	1 (0-1)	27 (12-52)	1 (0-2)
2000	Bolivia (Plurinational State of)	25340 (21864-30122)		2221 (1916-2640)		9889 (7409-11280)		867 (649-989)		218 (71-559)		19 (6-49)		87 (28-224)		8 (2-20)		657 (213-1689)		58 (19-148)	
2001	Bolivia (Plurinational State of)									206 (67-533)		18 (6-46)		83 (27-214)		7 (2-19)		623 (201-1609)		54 (17-140)	
2002	Bolivia (Plurinational State of)									193 (52-516)		17 (4-45)		219 (59-586)		19 (5-51)		582 (156-1559)		50 (13-135)	
2003	Bolivia (Plurinational State of)									178 (47-478)		15 (4-41)		202 (54-543)		17 (5-47)		536 (143-1443)		46 (12-124)	
2004	Bolivia (Plurinational State of)									161 (43-435)		14 (4-37)		183 (49-494)		16 (4-43)		486 (130-1314)		42 (11-113)	
2005	Bolivia (Plurinational State of)									147 (39-397)		13 (3-34)		166 (44-451)		14 (4-39)		443 (118-1198)		38 (10-103)	
2006	Bolivia (Plurinational State of)									136 (36-367)		12 (3-31)		154 (41-417)		13 (4-36)		410 (109-1108)		35 (9-95)	
2007	Bolivia (Plurinational State of)									128 (34-345)		11 (3-30)		145 (38-392)		12 (3-34)		386 (102-1041)		33 (9-89)	
2008	Bolivia (Plurinational State of)									124 (32-333)		11 (3-28)		141 (37-378)		12 (3-32)		375 (98-1005)		32 (8-86)	
2009	Bolivia (Plurinational State of)									122 (39-311)		10 (3-27)		138 (45-353)		12 (4-30)		368 (119-940)		31 (10-80)	
2010	Bolivia (Plurinational State of)									117 (36-299)		10 (3-26)		133 (41-340)		11 (4-29)		352 (110-903)		30 (9-77)	
2011	Bolivia (Plurinational State of)									109 (33-281)		9 (3-24)		124 (37-319)		11 (3-27)		329 (99-848)		28 (8-72)	
2012	Bolivia (Plurinational State of)									100 (29-261)		9 (2-22)		114 (33-297)		10 (3-25)		303 (88-789)		26 (8-67)	
2013	Bolivia (Plurinational State of)									91 (26-239)		8 (2-20)		103 (29-271)		9 (2-23)		274 (77-721)		23 (7-62)	
2014	Bolivia (Plurinational State of)									63 (17-168)		5 (1-14)		72 (20-190)		6 (2-16)		190 (52-506)		16 (4-43)	
2015	Bolivia (Plurinational State of)	6984 (6026-8302)	10 (9-12)	599 (517-712)	1 (1-1)	2724 (2041-3107)	4 (3-5)	234 (175-267)	0 (0-0)	43 (11-115)	0 (0-0)	4 (1-10)	0 (0-0)	49 (13-130)	0 (0-0)	4 (1-11)	0 (0-0)	129 (34-347)	0 (0-1)	11 (3-30)	0 (0-0)
2000	Bosnia and Herzegovina	493 (425-586)		230 (198-273)		319 (239-364)		149 (112-170)		2 (1-7)		1 (0-3)		2 (1-8)		1 (0-4)		6 (2-21)		3 (1-10)	

Spn = *Streptococcus pneumoniae*; npnm = non-pneumonia, non-meningitis; HIV = cases only in HIV-infected children; Uncertainty range provided in parentheses.

Year	Country	Spn pneumonia cases (UR)	Spn pneumonia cases, HIV (UR)	Spn pneumonia incidence rate (UR)	Spn pneumonia incidence rate, HIV (UR)	Spn severe pneumonia cases (UR)	Spn severe pneumonia cases, HIV (UR)	Spn severe pneumonia incidence rate (UR)	Spn severe pneumonia incidence rate, HIV (UR)	Spn meningitis cases (UR)	Spn meningitis cases, HIV (UR)	Spn meningitis incidence rate (UR)	Spn meningitis incidence rate, HIV (UR)	Spn NPNM cases (UR)	Spn NPNM cases, HIV (UR)	Spn NPNM incidence rate (UR)	Spn NPNM incidence rate, HIV (UR)	Spn NPNM non-severe cases (UR)	Spn NPNM non-severe cases, HIV (UR)	SPN NPNM non-severe incidence rate (UR)	SPN NPNM non-severe incidence rate, HIV (UR)
2001	Bosnia and Herzegovina									2 (1-6)		1 (0-3)		2 (1-7)		1 (0-3)		5 (2-19)		3 (1-9)	
2002	Bosnia and Herzegovina									2 (1-6)		1 (0-3)		2 (1-7)		1 (0-3)		5 (2-18)		3 (1-9)	
2003	Bosnia and Herzegovina									2 (1-6)		1 (0-3)		2 (1-6)		1 (0-3)		5 (2-17)		3 (1-9)	
2004	Bosnia and Herzegovina									3 (1-8)		1 (1-4)		3 (1-9)		2 (1-5)		8 (3-23)		4 (2-13)	
2005	Bosnia and Herzegovina									3 (1-9)		2 (1-5)		3 (1-10)		2 (1-6)		9 (4-27)		6 (2-16)	
2006	Bosnia and Herzegovina									3 (1-9)		2 (1-6)		3 (1-10)		2 (1-7)		9 (4-27)		6 (3-18)	
2007	Bosnia and Herzegovina									3 (1-9)		2 (1-6)		4 (1-11)		2 (1-7)		10 (4-29)		7 (3-19)	
2008	Bosnia and Herzegovina									3 (1-10)		2 (1-6)		4 (1-11)		2 (1-7)		10 (4-29)		6 (2-18)	
2009	Bosnia and Herzegovina									3 (1-9)		2 (1-5)		3 (1-10)		2 (1-6)		9 (3-27)		5 (2-16)	
2010	Bosnia and Herzegovina									3 (1-8)		1 (1-5)		3 (1-9)		2 (1-5)		8 (3-24)		4 (2-14)	
2011	Bosnia and Herzegovina									2 (1-6)		1 (0-3)		2 (1-7)		1 (0-4)		6 (2-19)		3 (1-10)	
2012	Bosnia and Herzegovina									2 (1-5)		1 (0-3)		2 (1-6)		1 (0-3)		5 (2-17)		3 (1-9)	
2013	Bosnia and Herzegovina									1 (1-4)		1 (0-2)		2 (1-5)		1 (0-3)		4 (2-13)		2 (1-7)	
2014	Bosnia and Herzegovina									1 (0-4)		1 (0-2)		1 (0-4)		1 (0-2)		3 (1-11)		2 (1-6)	
2015	Bosnia and Herzegovina	390 (337-464)	0 (0-0)	230 (198-273)	0 (0-0)	253 (189-288)	0 (0-0)	149 (112-170)	0 (0-0)	1 (0-3)	0 (0-0)	1 (0-2)	0 (0-0)	1 (0-3)	0 (0-0)	1 (0-2)	0 (0-0)	3 (1-9)	0 (0-0)	2 (1-5)	0 (0-0)
2000	Botswana	7464 (6440-8873)		3411 (2943-4054)		2934 (2198-3347)		1341 (1004-1529)		10 (3-25)		5 (2-11)		4 (1-10)		2 (1-5)		32 (10-74)		14 (5-34)	
2001	Botswana									11 (3-25)		5 (2-12)		4 (1-10)		2 (1-5)		32 (10-76)		15 (5-35)	
2002	Botswana									11 (4-26)		5 (2-12)		4 (1-10)		2 (1-5)		34 (11-79)		15 (5-36)	
2003	Botswana									12 (4-27)		5 (2-12)		5 (1-11)		2 (1-5)		35 (11-82)		16 (5-37)	
2004	Botswana									12 (4-28)		6 (2-13)		5 (2-11)		2 (1-5)		36 (12-85)		17 (5-39)	
2005	Botswana									12 (3-30)		6 (1-14)		14 (4-34)		6 (2-16)		38 (10-91)		17 (4-42)	
2006	Botswana									13 (3-31)		6 (2-14)		14 (4-35)		7 (2-16)		39 (10-93)		18 (5-42)	
2007	Botswana									13 (3-32)		6 (2-14)		15 (4-36)		7 (2-16)		40 (10-96)		18 (5-43)	
2008	Botswana									13 (4-32)		6 (2-14)		15 (4-37)		7 (2-16)		40 (11-98)		18 (5-43)	
2009	Botswana									13 (4-33)		6 (2-14)		15 (4-37)		7 (2-16)		41 (11-98)		18 (5-43)	
2010	Botswana									13 (4-32)		6 (2-14)		15 (4-37)		7 (2-16)		41 (11-98)		17 (5-42)	
2011	Botswana									14 (4-33)		6 (2-14)		15 (4-37)		6 (2-15)		41 (11-98)		17 (5-41)	
2012	Botswana									11 (3-26)		4 (1-10)		12 (3-29)		5 (1-12)		32 (9-77)		13 (3-31)	
2013	Botswana									9 (2-22)		4 (1-9)		10 (3-24)		4 (1-10)		27 (7-65)		11 (3-26)	
2014	Botswana									8 (2-18)		3 (1-7)		9 (2-21)		3 (1-8)		23 (6-55)		9 (2-21)	
2015	Botswana	2677 (2309-3182)	625 (539-743)	1022 (882-1215)	239 (206-284)	1047 (784-1194)	245 (183-279)	400 (300-456)	93 (70-107)	7 (2-17)	2 (0-4)	3 (1-6)	1 (0-1)	8 (2-19)	2 (0-4)	3 (1-7)	1 (0-2)	21 (6-50)	5 (1-12)	8 (2-19)	2 (1-4)
2000	Brazil	319238 (275446-379484)		1842 (1589-2190)		124349 (93162-141832)		718 (538-818)		1984 (487-5568)		11 (3-32)		2253 (554-6324)		13 (3-36)		5990 (1472-16813)		35 (8-97)	
2001	Brazil									1858 (303-6227)		11 (2-35)		2110 (344-7072)		12 (2-40)		5610 (916-18802)		32 (5-107)	
2002	Brazil									1477 (245-4940)		8 (1-28)		1678 (278-5610)		9 (2-32)		4460 (740-14915)		25 (4-84)	
2003	Brazil									1258 (212-4194)		7 (1-24)		1428 (240-4764)		8 (1-27)		3797 (639-12665)		22 (4-72)	
2004	Brazil									1205 (206-4007)		7 (1-23)		1369 (234-4551)		8 (1-26)		3639 (621-12099)		21 (4-69)	
2005	Brazil									1151 (199-3815)		7 (1-22)		1308 (226-4334)		8 (1-25)		3477 (602-11521)		20 (3-67)	
2006	Brazil									1102 (193-3640)		7 (1-22)		1252 (220-4134)		7 (1-24)		3328 (584-10990)		20 (3-65)	
2007	Brazil									912 (162-3002)		6 (1-18)		1035 (184-3409)		6 (1-21)		2753 (488-9064)		17 (3-55)	
2008	Brazil									795 (143-2610)		5 (1-16)		903 (162-2964)		6 (1-19)		2400 (431-7881)		15 (3-49)	
2009	Brazil									658 (119-2154)		4 (1-14)		747 (135-2447)		5 (1-16)		1986 (360-6505)		13 (2-42)	
2010	Brazil									520 (95-1699)		3 (1-11)		590 (108-1930)		4 (1-13)		1569 (286-5131)		10 (2-34)	
2011	Brazil									265 (49-866)		2 (0-6)		301 (55-984)		2 (0-7)		801 (147-2616)		5 (1-18)	
2012	Brazil									205 (38-669)		1 (0-5)		233 (43-760)		2 (0-5)		619 (114-2021)		4 (1-14)	
2013	Brazil									176 (32-574)		1 (0-4)		200 (37-652)		1 (0-4)		531 (97-1733)		4 (1-12)	
2014	Brazil									178 (33-582)		1 (0-4)		202 (37-661)		1 (0-4)		538 (98-1757)		4 (1-12)	
2015	Brazil	57716 (49799-68608)	89 (77-105)	390 (337-464)	1 (1-1)	22494 (16852-25657)	35 (26-39)	152 (114-174)	0 (0-0)	181 (33-590)	0 (0-1)	1 (0-4)	0 (0-0)	205 (37-670)	0 (0-1)	1 (0-5)	0 (0-0)	545 (100-1781)	1 (0-3)	4 (1-12)	0 (0-0)
2000	Brunei Darussalam	78 (67-92)		230 (198-273)		50 (38-57)		149 (112-170)		0 (0-0)		0 (0-0)		0 (0-0)		0 (0-0)		0 (0-0)		0 (0-0)	
2001	Brunei Darussalam									0 (0-0)		0 (0-0)		0 (0-0)		0 (0-0)		0 (0-0)		0 (0-0)	
2002	Brunei Darussalam									0 (0-0)		0 (0-0)		0 (0-0)		0 (0-0)		0 (0-0)		0 (0-0)	
2003	Brunei Darussalam									0 (0-0)		0 (0-0)		0 (0-0)		0 (0-0)		0 (0-0)		0 (0-0)	
2004	Brunei Darussalam									0 (0-0)		0 (0-0)		0 (0-0)		0 (0-0)		0 (0-0)		0 (0-0)	
2005	Brunei Darussalam									0 (0-0)		0 (0-0)		0 (0-0)		0 (0-0)		0 (0-0)		0 (0-0)	
2006	Brunei Darussalam									0 (0-0)		0 (0-0)		0 (0-0)		0 (0-0)		0 (0-0)		0 (0-0)	
2007	Brunei Darussalam									0 (0-0)		0 (0-0)		0 (0-0)		0 (0-0)		0 (0-0)		0 (0-0)	
2008	Brunei Darussalam									0 (0-0)		0 (0-0)		0 (0-0)		0 (0-0)		0 (0-0)		0 (0-0)	
2009	Brunei Darussalam									0 (0-0)		0 (0-0)		0 (0-0)		0 (0-0)		0 (0-0)		0 (0-0)	
2010	Brunei Darussalam									0 (0-0)		0 (0-0)		0 (0-0)		0 (0-0)		0 (0-0)		0 (0-0)	
2011	Brunei Darussalam									0 (0-0)		0 (0-0)		0 (0-0)		0 (0-0)		0 (0-0)		0 (0-0)	
2012	Brunei Darussalam									2 (1-2)		6 (4-8)		2 (1-3)		7 (4-9)		5 (3-7)		18 (11-24)	
2013	Brunei Darussalam									2 (1-2)		6 (3-8)		2 (1-3)		6 (4-9)		5 (3-7)		17 (10-23)	
2014	Brunei Darussalam									2 (1-3)		8 (5-11)		3 (2-4)		9 (5-12)		8 (5-10)		23 (14-32)	
2015	Brunei Darussalam	76 (66-91)	0 (0-0)	230 (198-273)	0 (0-0)	50 (37-57)	0 (0-0)	149 (112-170)	0 (0-0)	1 (1-1)	0 (0-0)	3 (2-4)	0 (0-0)	1 (1-2)	0 (0-0)	4 (2-5)	0 (0-0)	3 (2-4)	0 (0-0)	10 (6-13)	0 (0-0)
2000	Bulgaria	746 (644-887)		230 (198-273)		484 (362-552)		149 (112-170)		44 (20-80)		14 (6-25)		50 (23-91)		15 (7-28)		133 (60-241)		41 (19-74)	
2001	Bulgaria									37 (17-66)		11 (5-21)		41 (19-75)		13 (6-24)		110 (50-200)		34 (16-62)	
2002	Bulgaria									20 (9-36)		6 (3-11)		22 (10-41)		7 (3-13)		59 (27-108)		19 (9-34)	
2003	Bulgaria									52 (24-94)		17 (8-30)		59 (27-107)		19 (9-34)		157 (71-285)		50 (23-92)	
2004	Bulgaria									44 (20-79)		14 (6-26)		50 (23-90)		16 (7-29)		132 (60-240)		43 (19-77)	
2005	Bulgaria									38 (17-69)		12 (5-22)		43 (19-78)		14 (6-25)		114 (52-207)		37 (17-66)	
2006	Bulgaria									49 (22-90)		16 (7-28)		56 (25-102)		18 (8-32)		149 (68-271)		47 (21-85)	
2007	Bulgaria									40 (18-72)		12 (6-22)		45 (21-82)		14 (6-25)		121 (55-219)		37 (17-67)	
2008	Bulgaria									54 (24-98)		16 (7-29)		61 (28-111)		18 (8-33)		163 (74-295)		48 (22-87)	
2009	Bulgaria									37 (17-67)		11 (5-19)		42 (19-76)		12 (5-22)		111 (51-202)		32 (15-58)	
2010	Bulgaria									35 (16-63)		10 (4-18)		39 (18-72)		11 (5-20)		105 (48-190)		30 (13-54)	
2011	Bulgaria									20 (9-37)		6 (3-10)		23 (10-42)		6 (3-12)		61 (28-112)		17 (8-31)	
2012	Bulgaria									19 (9-35)		5 (2-10)		22 (10-40)		6 (3-11)		58 (26-106)		16 (7-30)	
2013	Bulgaria									17 (8-30)		5 (2-9)		19 (9-34)		5 (2-10)		50 (23-92)		15 (7-26)	
2014	Bulgaria									11 (5-19)		3 (1-6)		12 (5-22)		4 (2-6)		32 (15-58)		9 (4-17)	
2015	Bulgaria	220 (190-261)	0 (0-0)	66 (57-79)	0 (0-0)	142 (107-162)	0 (0-0)	43 (32-49)	0 (0-0)	9 (4-16)	0 (0-0)	3 (1-5)	0 (0-0)	10 (5-19)	0 (0-0)	3 (1-6)	0 (0-0)	27 (12-49)	0 (0-0)	8 (4-15)	0 (0-0)
2000	Burkina Faso	57355 (49487-68178)		2685 (2317-3192)		22429 (16804-25583)		1050 (787-1198)		715 (377-1361)		33 (18-64)		287 (151-546)		13 (7-26)		2158 (1139-4110)		101 (53-192)	
2001	Burkina Faso									759 (402-1441)		34 (18-65)		304 (161-578)		14 (7-26)		2291 (1215-4352)		104 (55-197)	
2002	Burkina Faso									807 (430-1528)		36 (19-68)		324 (172-613)		14 (8-27)		2436 (1298-4615)		108 (57-204)	
2003	Burkina Faso									861 (462-1629)		37 (20-70)		345 (185-653)		15 (8-28)		2601 (1394-4919)		112 (60-212)	
2004	Burkina Faso									916 (494-1732)		39 (21-73)		368 (198-695)		15 (8-29)		2767 (1493-5229)		116 (63-220)	
2005	Burkina Faso									952 (518-1799)		39 (21-74)		382 (208-722)		16 (8-30)		2876 (1563-5433)		118 (64-222)	
2006	Burkina Faso									959 (358-2071)		38 (14-82)		385 (143-831)		15 (6-33)		2897 (1080-6254)		115 (43-248)	
2007	Burkina Faso									927 (359-1985)		36 (14-76)		372 (144-796)		14 (6-31)		2800 (1085-5993)		108 (42-230)	
2008	Burkina Faso									880 (354-1861)		33 (13-70)		353 (142-746)		13 (5-28)		2657 (1069-5619)		99 (40-210)	
2009	Burkina Faso									873 (364-1818)		32 (13-66)		350 (146-729)		13 (5-27)		2637 (1100-5489)		96 (40-200)	
2010	Burkina Faso									909 (393-1857)		32 (14-66)		365 (158-745)		13 (6-27)		2746 (1186-5608)		98 (42-200)	
2011	Burkina Faso									1013 (454-2026)		35 (16-70)		406 (182-813)		14 (6-28)		3059 (1371-6118)		106 (48-212)	
2012	Burkina Faso									1131 (526-2210)		38 (18-75)		453 (211-886)		15 (7-30)		3414 (1587-6673)		116 (54-227)	
2013	Burkina Faso									1257 (609-2404)		42 (20-80)		504 (244-964)		17 (8-32)		3797 (1840-7260)		127 (62-243)	
2014	Burkina Faso									840 (426-1572)		28 (14-52)		337 (171-631)		11 (6-21)		2537 (1287-4747)		84 (42-156)	
2015	Burkina Faso	37869 (32675-45016)	1438 (1241-1709)	1225 (1057-1456)	47 (40-55)	14894 (11158-16988)	565 (424-645)	482 (361-549)	18 (14-21)	728 (388-1334)	28 (15-51)	24 (13-43)	1 (0-2)	292 (156-535)	11 (6-20)	9 (5-17)	0 (0-1)	2198 (1172-4027)	83 (44-153)	71 (38-130)	3 (1-5)
2000	Burundi	34264 (29564-40731)		2858 (2466-3398)		13467 (10089-15360)		1123 (842-1281)		720 (398-1362)		60 (33-114)		289 (159-546)		24 (13-46)		2174 (1200-4111)		181 (100-343)	
2001	Burundi									730 (275-1583)		60 (23-131)		293 (110-635)		24 (9-52)		2204 (831-4782)		182 (69-394)	
2002	Burundi									725 (273-1580)		59 (22-128)		291 (110-634)		24 (9-51)		2190 (825-4770)		177 (67-386)	
2003	Burundi									778 (293-1699)		61 (23-134)		312 (117-681)		25 (9-54)		2348 (884-5130)		185 (70-405)	
2004	Burundi									818 (307-1791)		63 (23-137)		328 (123-718)		25 (9-55)		2471 (927-5408)		189 (71-414)	
2005	Burundi									827 (309-1808)		61 (23-133)		332 (124-725)		24 (9-53)		2497 (932-5458)		184 (69-402)	
2006	Burundi									892 (342-1931)		63 (24-136)		358 (137-775)		25 (10-55)		2693 (1033-5832)		190 (73-411)	
2007	Burundi									922 (363-1974)		62 (25-133)		370 (146-792)		25 (10-53)		2784 (1097-5961)		188 (74-402)	
2008	Burundi									953 (386-2015)		61 (25-130)		382 (155-808)		25 (10-52)		2877 (1165-6083)		185 (75-392)	
2009	Burundi									995 (414-2075)		61 (25-128)		399 (166-832)		25 (10-51)		3005 (1250-6267)		185 (77-386)	
2010	Burundi									1036 (443-2129)		61 (26-126)		416 (178-854)		25 (10-50)		3129 (1337-6428)		185 (79-380)	
2011	Burundi									1028 (452-2080)		58 (25-117)		413 (181-834)		23 (10-47)		3105 (1365-6280)		175 (77-353)	
2012	Burundi									664 (300-1319)		36 (16-71)		266 (120-529)		14 (7-29)		2004 (906-3984)		108 (49-216)	
2013	Burundi									571 (267-1118)		30 (14-59)		229 (107-448)		12 (6-23)		1725 (805-3375)		90 (42-177)	
2014	Burundi									557 (269-1073)		28 (14-55)		224 (108-431)		11 (5-22)		1683 (813-3241)		86 (41-165)	
2015	Burundi	12721 (10976-15121)	556 (480-661)	627 (541-746)	27 (24-33)	4993 (3741-5695)	218 (163-249)	246 (184-281)	11 (8-12)	410 (205-777)	18 (9-34)	20 (10-38)	1 (0-2)	164 (82-312)	7 (4-14)	8 (4-15)	0 (0-1)	1237 (620-2347)	54 (27-103)	61 (31-116)	3 (1-5)
2000	Cambodia	52189 (45029-62038)		3357 (2897-3991)		20103 (15061-22930)		1293 (969-1475)		610 (211-1388)		39 (14-89)		245 (85-557)		16 (5-36)		1843 (638-4191)		119 (41-270)	
2001	Cambodia									534 (189-1209)		35 (12-80)		214 (76-485)		14 (5-32)		1613 (569-3650)		106 (37-240)	
2002	Cambodia									465 (167-1047)		31 (11-70)		187 (67-420)		12 (4-28)		1405 (506-3162)		94 (34-211)	
2003	Cambodia									410 (150-918)		27 (10-61)		164 (60-368)		11 (4-25)		1238 (454-2771)		83 (30-185)	
2004	Cambodia									376 (118-862)		25 (8-57)		427 (134-979)		28 (9-65)		1135 (355-2602)		76 (24-173)	
2005	Cambodia									352 (113-801)		23 (7-53)		400 (128-910)		26 (8-60)		1063 (340-2418)		70 (23-160)	
2006	Cambodia									336 (111-756)		22 (7-49)		382 (127-859)		25 (8-56)		1015 (336-2283)		66 (22-148)	
2007	Cambodia									322 (111-716)		20 (7-45)		366 (126-813)		23 (8-52)		973 (334-2162)		62 (21-137)	
2008	Cambodia									310 (110-679)		19 (7-42)		352 (125-771)		22 (8-48)		936 (333-2051)		59 (21-128)	
2009	Cambodia									294 (109-634)		18 (7-39)		334 (123-720)		21 (8-44)		887 (328-1914)		55 (20-118)	
2010	Cambodia									269 (104-573)		16 (6-35)		306 (118-650)		19 (7-39)		813 (313-1729)		49 (19-105)	
2011	Cambodia									246 (96-518)		14 (6-30)		279 (109-589)		16 (6-35)		743 (289-1566)		44 (17-92)	
2012	Cambodia									220 (87-460)		13 (5-27)		250 (98-523)		14 (6-30)		664 (262-1389)		38 (15-80)	
2013	Cambodia									203 (81-421)		12 (5-24)		230 (92-479)		13 (5-28)		613 (245-1272)		35 (14-73)	
2014	Cambodia									191 (78-395)		11 (4-23)		217 (88-448)		13 (5-26)		577 (234-1191)		33 (14-69)	
2015	Cambodia	25084 (21643-29818)	402 (347-478)	1440 (1242-1712)	23 (20-27)	9860 (7387-11246)	158 (119-180)	566 (424-646)	9 (7-10)	119 (37-293)	2 (1-5)	7 (2-17)	0 (0-0)	135 (42-333)	2 (1-5)	8 (2-19)	0 (0-0)	359 (112-886)	6 (2-14)	21 (6-51)	0 (0-1)
2000	Cameroon	50131 (43254-59591)		1849 (1595-2197)		19714 (14769-22485)		727 (545-829)		1001 (515-1967)		37 (19-73)		401 (207-789)		15 (8-29)		3021 (1556-5940)		111 (57-219)	
2001	Cameroon									992 (345-2226)		36 (12-80)		398 (139-893)		14 (5-32)		2994 (1043-6723)		108 (38-242)	
2002	Cameroon									1007 (361-2243)		35 (13-79)		404 (145-900)		14 (5-32)		3039 (1091-6774)		107 (38-238)	
2003	Cameroon									996 (369-2203)		34 (13-76)		400 (148-884)		14 (5-30)		3009 (1115-6653)		103 (38-229)	
2004	Cameroon									988 (378-2168)		33 (13-73)		396 (152-870)		13 (5-29)		2985 (1143-6548)		100 (38-220)	
2005	Cameroon									984 (368-2167)		32 (12-71)		395 (148-869)		13 (5-28)		2970 (1111-6543)		97 (36-215)	
2006	Cameroon									995 (364-2204)		32 (12-70)		399 (146-884)		13 (5-28)		3004 (1101-6656)		96 (35-212)	
2007	Cameroon									994 (362-2210)		31 (11-69)		399 (145-886)		12 (5-28)		3002 (1092-6673)		94 (34-208)	
2008	Cameroon									1017 (367-2268)		31 (11-70)		408 (147-910)		13 (5-28)		3070 (1109-6849)		94 (34-210)	
2009	Cameroon									1054 (378-2359)		32 (11-71)		423 (152-946)		13 (5-29)		3181 (1141-7123)		96 (34-215)	
2010	Cameroon									1053 (375-2365)		31 (11-70)		422 (150-949)		13 (4-28)		3179 (1132-7141)		94 (34-211)	
2011	Cameroon									944 (334-2126)		27 (10-62)		378 (134-853)		11 (4-25)		2849 (1007-6419)		83 (29-186)	
2012	Cameroon									637 (224-1440)		18 (6-41)		256 (90-578)		7 (3-16)		1924 (675-4349)		55 (19-124)	
2013	Cameroon									527 (183-1193)		15 (5-33)		211 (74-479)		6 (2-13)		1590 (554-3603)		45 (16-101)	
2014	Cameroon									488 (169-1108)		13 (5-31)		196 (68-444)		5 (2-12)		1473 (509-3345)		41 (14-92)	
2015	Cameroon	25684 (22161-30532)	4946 (4267-5879)	699 (603-831)	135 (116-160)	10042 (7524-11454)	1934 (1449-2206)	273 (205-312)	53 (39-60)	327 (112-743)	63 (22-143)	9 (3-20)	2 (1-4)	131 (45-298)	25 (9-57)	4 (1-8)	1 (0-2)	986 (338-2245)	190 (65-432)	27 (9-61)	5 (2-12)
2000	Canada	2027 (1749-2409)		115 (99-137)		1314 (984-1498)		75 (56-85)		131 (42-237)		7 (2-13)		149 (47-269)		8 (3-15)		395 (126-714)		22 (7-41)	
2001	Canada									100 (32-180)		6 (2-11)		113 (36-204)		7 (2-12)		301 (96-543)		18 (6-32)	
2002	Canada									93 (29-167)		6 (2-10)		105 (33-190)		6 (2-11)		279 (89-505)		17 (5-30)	
2003	Canada									94 (30-170)		6 (2-10)		107 (34-193)		6 (2-12)		284 (90-514)		17 (5-31)	
2004	Canada									81 (26-146)		5 (2-9)		92 (29-165)		5 (2-10)		243 (77-440)		15 (5-26)	
2005	Canada									77 (25-139)		5 (1-8)		88 (28-158)		5 (2-9)		233 (74-421)		14 (4-25)	
2006	Canada									90 (29-163)		5 (2-9)		102 (32-185)		6 (2-11)		272 (86-491)		16 (5-29)	
2007	Canada									84 (27-151)		5 (2-9)		95 (30-172)		5 (2-10)		253 (80-457)		14 (5-26)	
2008	Canada									35 (11-63)		2 (1-4)		40 (13-71)		2 (1-4)		105 (33-190)		6 (2-11)	
2009	Canada									94 (30-170)		5 (2-9)		107 (34-193)		6 (2-11)		284 (90-514)		16 (5-28)	
2010	Canada									76 (24-138)		4 (1-7)		87 (28-157)		5 (1-8)		230 (73-416)		12 (4-22)	
2011	Canada									32 (10-58)		2 (1-3)		37 (12-66)		2 (1-3)		97 (31-176)		5 (2-9)	
2012	Canada									30 (10-55)		2 (1-3)		35 (11-62)		2 (1-3)		92 (29-166)		5 (2-9)	
2013	Canada									27 (8-48)		1 (0-3)		30 (10-54)		2 (1-3)		80 (25-145)		4 (1-8)	
2014	Canada									22 (7-40)		1 (0-2)		25 (8-45)		1 (0-2)		67 (21-121)		4 (1-6)	
2015	Canada	360 (311-428)	0 (0-0)	19 (16-22)	0 (0-0)	234 (175-266)	0 (0-0)	12 (9-14)	0 (0-0)	21 (7-37)	0 (0-0)	1 (0-2)	0 (0-0)	23 (7-42)	0 (0-0)	1 (0-2)	0 (0-0)	62 (20-112)	0 (0-0)	3 (1-6)	0 (0-0)
2000	Cape Verde	1515 (1307-1801)		2465 (2127-2930)		596 (446-680)		969 (726-1106)		11 (6-16)		19 (9-27)		13 (6-19)		21 (10-30)		35 (17-50)		56 (27-81)	
2001	Cape Verde									11 (5-15)		18 (9-25)		12 (6-17)		21 (10-29)		33 (16-46)		55 (26-77)	
2002	Cape Verde									11 (8-17)		19 (14-29)		13 (9-20)		22 (16-33)		35 (25-53)		58 (42-89)	
2003	Cape Verde									12 (8-17)		21 (14-30)		13 (9-20)		23 (16-34)		36 (24-52)		62 (42-91)	
2004	Cape Verde									13 (8-18)		23 (15-32)		15 (10-20)		26 (17-36)		39 (25-54)		70 (45-97)	
2005	Cape Verde									14 (9-19)		25 (16-34)		16 (10-21)		29 (18-39)		41 (26-56)		76 (48-103)	
2006	Cape Verde									14 (9-19)		26 (17-36)		16 (10-21)		30 (19-40)		42 (27-57)		80 (50-107)	
2007	Cape Verde									14 (9-19)		27 (17-37)		16 (10-22)		31 (19-42)		43 (27-58)		82 (52-111)	
2008	Cape Verde									14 (9-19)		28 (17-37)		16 (10-22)		31 (20-42)		43 (27-58)		83 (53-112)	
2009	Cape Verde									14 (9-19)		27 (17-37)		16 (10-21)		31 (20-42)		42 (27-57)		83 (52-112)	

Spn = *Streptococcus pneumoniae*; npnm = non-pneumonia, non-meningitis; HIV = cases only in HIV-infected children; Uncertainty range provided in parentheses.

Year	Country	Spn pneumonia cases (UR)	Spn pneumonia cases, HIV (UR)	Spn pneumonia incidence rate (UR)	Spn pneumonia incidence rate, HIV (UR)	Spn severe pneumonia cases (UR)	Spn severe pneumonia cases, HIV (UR)	Spn severe pneumonia incidence rate (UR)	Spn severe pneumonia incidence rate, HIV (UR)	Spn meningitis cases (UR)	Spn meningitis cases, HIV (UR)	Spn meningitis incidence rate (UR)	Spn meningitis incidence rate, HIV (UR)	Spn NPNM cases (UR)	Spn NPNM cases, HIV (UR)	Spn NPNM incidence rate (UR)	Spn NPNM incidence rate, HIV (UR)	Spn NPNM non-severe cases (UR)	Spn NPNM non-severe cases, HIV (UR)	SPN NPNM non-severe incidence rate (UR)	SPN NPNM non-severe incidence rate, HIV (UR)
2010	Cape Verde									14 (9-18)		27 (17-36)		16 (10-21)		30 (19-41)		41 (26-56)		81 (51-109)	
2011	Cape Verde									21 (13-28)		40 (25-54)		24 (15-32)		46 (29-62)		64 (40-86)		122 (77-164)	
2012	Cape Verde									21 (13-28)		39 (25-53)		23 (15-32)		45 (28-60)		62 (39-84)		119 (75-160)	
2013	Cape Verde									20 (13-27)		38 (24-51)		23 (14-30)		43 (27-58)		60 (38-81)		114 (72-154)	
2014	Cape Verde									19 (12-25)		35 (22-48)		21 (13-29)		40 (25-54)		56 (35-76)		107 (68-144)	
2015	Cape Verde	856 (739-1018)	33 (29-40)	1625 (1402-1931)	63 (55-75)	335 (251-383)	13 (10-15)	637 (477-726)	25 (19-28)	17 (11-24)	1 (0-1)	33 (21-45)	1 (1-2)	20 (13-27)	1 (0-1)	38 (24-51)	1 (1-2)	53 (33-71)	2 (1-3)	100 (63-135)	4 (2-5)
2000	Central African Republic	23800 (20535-28291)		4002 (3453-4757)		9291 (6960-10597)		1562 (1170-1782)		249 (131-477)		42 (22-80)		100 (53-191)		17 (9-32)		753 (397-1442)		127 (67-242)	
2001	Central African Republic									252 (133-482)		41 (22-79)		101 (53-193)		17 (9-32)		760 (401-1456)		125 (66-239)	
2002	Central African Republic									256 (135-490)		41 (22-79)		103 (54-196)		17 (9-32)		772 (407-1478)		125 (66-239)	
2003	Central African Republic									251 (132-480)		40 (21-77)		101 (53-193)		16 (9-31)		757 (399-1450)		122 (64-233)	
2004	Central African Republic									244 (128-467)		39 (21-75)		98 (52-187)		16 (8-30)		736 (388-1411)		118 (62-226)	
2005	Central African Republic									236 (124-452)		38 (20-72)		95 (50-181)		15 (8-29)		713 (375-1366)		113 (60-217)	
2006	Central African Republic									244 (128-467)		38 (20-74)		98 (51-187)		15 (8-30)		736 (388-1411)		116 (61-223)	
2007	Central African Republic									251 (132-482)		40 (21-76)		101 (53-193)		16 (8-30)		758 (398-1455)		119 (63-229)	
2008	Central African Republic									256 (135-494)		40 (21-78)		103 (54-198)		16 (8-31)		774 (407-1491)		122 (64-234)	
2009	Central African Republic									275 (144-531)		43 (23-83)		110 (58-213)		17 (9-33)		831 (436-1605)		130 (68-251)	
2010	Central African Republic									290 (153-563)		45 (24-87)		116 (61-226)		18 (9-35)		876 (461-1700)		136 (71-263)	
2011	Central African Republic									296 (103-663)		45 (16-102)		119 (41-266)		18 (6-41)		894 (312-2002)		137 (48-307)	
2012	Central African Republic									248 (87-558)		37 (13-84)		99 (35-224)		15 (5-34)		749 (262-1686)		113 (40-255)	
2013	Central African Republic									283 (99-639)		42 (15-95)		113 (40-256)		17 (6-38)		854 (298-1931)		127 (44-287)	
2014	Central African Republic									259 (90-587)		38 (13-86)		104 (36-235)		15 (5-34)		782 (272-1771)		114 (40-259)	
2015	Central African Republic	8561 (7386-10176)	2128 (1836-2530)	1229 (1061-1461)	306 (264-363)	3357 (2515-3828)	834 (625-952)	482 (361-550)	120 (90-137)	251 (87-569)	62 (22-141)	36 (12-82)	9 (3-20)	101 (35-228)	25 (9-57)	14 (5-33)	4 (1-8)	758 (263-1719)	188 (65-427)	109 (38-247)	27 (9-61)
2000	Chad	67255 (58029-79948)		4053 (3497-4817)		25976 (19461-29629)		1565 (1173-1785)		1105 (563-2194)		67 (34-132)		443 (226-880)		27 (14-53)		3336 (1701-6626)		201 (102-399)	
2001	Chad									1104 (556-2203)		64 (32-128)		443 (223-884)		26 (13-51)		3333 (1679-6653)		193 (97-385)	
2002	Chad									1156 (576-2321)		65 (32-130)		464 (231-931)		26 (13-52)		3492 (1739-7007)		195 (97-391)	
2003	Chad									1122 (552-2261)		60 (30-122)		450 (221-907)		24 (12-49)		3387 (1667-6828)		183 (90-368)	
2004	Chad									1122 (545-2271)		59 (28-118)		450 (219-911)		23 (11-47)		3388 (1647-6857)		177 (86-358)	
2005	Chad									1138 (560-2295)		57 (28-116)		456 (225-921)		23 (11-47)		3436 (1690-6930)		174 (85-350)	
2006	Chad									1208 (600-2424)		59 (29-118)		485 (241-972)		24 (12-48)		3648 (1813-7320)		178 (89-358)	
2007	Chad									1204 (604-2402)		57 (29-114)		483 (242-963)		23 (12-46)		3637 (1825-7252)		173 (87-344)	
2008	Chad									1276 (646-2526)		59 (30-117)		512 (259-1013)		24 (12-47)		3852 (1950-7628)		178 (90-353)	
2009	Chad									1264 (645-2485)		57 (29-112)		507 (259-997)		23 (12-45)		3817 (1949-7505)		172 (88-339)	
2010	Chad									1296 (669-2533)		57 (29-111)		520 (268-1016)		23 (12-45)		3914 (2019-7648)		172 (89-336)	
2011	Chad									1332 (695-2591)		57 (30-111)		534 (279-1039)		23 (12-44)		4023 (2100-7824)		172 (90-334)	
2012	Chad									1368 (723-2651)		57 (30-110)		549 (290-1063)		23 (12-44)		4132 (2185-8006)		172 (91-333)	
2013	Chad									1411 (507-3125)		57 (21-127)		566 (203-1253)		23 (8-51)		4260 (1530-9435)		173 (62-383)	
2014	Chad									1431 (525-3160)		57 (21-125)		574 (211-1267)		23 (8-50)		4322 (1586-9542)		171 (63-378)	
2015	Chad	45472 (39234-54053)	7305 (6303-8684)	1757 (1516-2088)	282 (244-335)	17656 (13228-20139)	2837 (2125-3235)	682 (511-778)	110 (82-125)	1444 (542-3177)	232 (87-510)	56 (21-123)	9 (3-20)	579 (217-1274)	93 (35-205)	22 (8-49)	4 (1-8)	4361 (1636-9594)	701 (263-1541)	168 (63-371)	27 (10-60)
2000	Chile	19129 (16505-22739)		1512 (1305-1797)		7435 (5571-8481)		588 (440-670)		235 (115-364)		19 (9-29)		267 (131-414)		21 (10-33)		709 (348-1099)		56 (28-87)	
2001	Chile									323 (159-501)		26 (13-40)		367 (180-569)		29 (14-45)		977 (480-1514)		78 (38-121)	
2002	Chile									233 (114-361)		19 (9-29)		264 (130-410)		21 (10-33)		703 (345-1090)		57 (28-88)	
2003	Chile									241 (118-373)		20 (10-30)		273 (134-424)		22 (11-34)		727 (357-1127)		59 (29-92)	
2004	Chile									199 (98-309)		16 (8-25)		226 (111-351)		19 (9-29)		602 (296-933)		49 (24-77)	
2005	Chile									217 (106-336)		18 (9-28)		246 (121-381)		20 (10-32)		654 (321-1014)		54 (27-84)	
2006	Chile									133 (65-206)		11 (5-17)		151 (74-234)		13 (6-20)		402 (197-623)		33 (16-52)	
2007	Chile									107 (53-166)		9 (4-14)		122 (60-189)		10 (5-16)		324 (159-502)		27 (13-42)	
2008	Chile									117 (57-181)		10 (5-15)		132 (65-205)		11 (5-17)		352 (173-546)		30 (15-46)	
2009	Chile									88 (43-137)		7 (4-12)		100 (49-155)		9 (4-13)		267 (131-413)		23 (11-35)	
2010	Chile									149 (73-230)		13 (6-20)		169 (83-261)		14 (7-22)		448 (220-695)		38 (19-59)	
2011	Chile									74 (36-115)		6 (3-10)		84 (41-131)		7 (4-11)		224 (110-347)		19 (9-30)	
2012	Chile									74 (37-115)		6 (3-10)		84 (41-131)		7 (4-11)		225 (110-348)		19 (9-30)	
2013	Chile									36 (18-56)		3 (2-5)		41 (20-63)		4 (2-5)		109 (53-168)		9 (5-15)	
2014	Chile									48 (24-75)		4 (2-6)		55 (27-85)		5 (2-7)		146 (72-226)		13 (6-20)	
2015	Chile	4256 (3672-5059)	1 (1-1)	370 (319-440)	0 (0-0)	1657 (1242-1890)	0 (0-0)	144 (108-164)	0 (0-0)	37 (18-57)	0 (0-0)	3 (2-5)	0 (0-0)	41 (20-64)	0 (0-0)	4 (2-6)	0 (0-0)	110 (54-171)	0 (0-0)	10 (5-15)	0 (0-0)
2000	China	1634538 (1410315-1943006)		2082 (1796-2475)		638603 (478438-728388)		813 (609-928)		12867 (6021-19531)		16 (8-25)		14615 (6838-22183)		19 (9-28)		38854 (18181-58976)		49 (23-75)	
2001	China									11887 (5459-17709)		15 (7-23)		13501 (6201-20114)		18 (8-26)		35894 (16484-53474)		47 (21-70)	
2002	China									11436 (5102-16551)		15 (7-22)		12989 (5795-18799)		17 (8-25)		34531 (15407-49979)		46 (20-66)	
2003	China									11834 (8141-18708)		16 (11-25)		13441 (9247-21248)		18 (12-28)		35733 (24584-56490)		48 (33-75)	
2004	China									12202 (7931-18224)		16 (11-24)		13859 (9008-20699)		19 (12-28)		36844 (23948-55030)		50 (32-74)	
2005	China									12785 (7907-18168)		17 (11-24)		14522 (8980-20636)		20 (12-28)		38606 (23875-54861)		52 (32-74)	
2006	China									13503 (8104-18622)		18 (11-24)		15336 (9204-21150)		20 (12-28)		40772 (24471-56230)		53 (32-73)	
2007	China									13260 (7958-18287)		17 (10-24)		15061 (9039-20770)		19 (12-27)		40040 (24031-55219)		52 (31-71)	
2008	China									13586 (8154-18737)		18 (11-24)		15431 (9261-21281)		20 (12-28)		41025 (24622-56578)		53 (32-73)	
2009	China									13332 (8002-18386)		17 (10-24)		15142 (9088-20883)		20 (12-27)		40257 (24161-55519)		52 (31-72)	
2010	China									13100 (7863-18067)		17 (10-23)		14879 (8930-20521)		19 (12-26)		39558 (23742-54555)		51 (31-70)	
2011	China									12304 (7385-16969)		16 (9-22)		13975 (8388-19274)		18 (11-25)		37155 (22299-51241)		48 (29-66)	
2012	China									11123 (6676-15340)		14 (8-19)		12633 (7582-17423)		16 (10-22)		33586 (20158-46320)		43 (26-59)	
2013	China									10182 (6111-14042)		13 (8-17)		11564 (6941-15949)		14 (9-20)		30745 (18452-42401)		38 (23-53)	
2014	China									9162 (5499-12636)		11 (7-16)		10407 (6246-14352)		13 (8-18)		27667 (16605-38156)		34 (20-47)	
2015	China	506501 (437020-602087)	596 (514-708)	619 (534-736)	1 (1-1)	196288 (147058-223885)	231 (173-263)	240 (180-274)	0 (0-0)	8686 (5213-11980)	10 (6-14)	11 (6-15)	0 (0-0)	9866 (5921-13606)	12 (7-16)	12 (7-17)	0 (0-0)	26230 (15742-36174)	31 (19-43)	32 (19-44)	0 (0-0)
2000	Colombia	66634 (57493-79209)		1632 (1408-1940)		26006 (19484-29662)		637 (477-727)		296 (53-966)		7 (1-24)		336 (60-1098)		8 (1-27)		893 (159-2918)		22 (4-71)	
2001	Colombia									278 (51-901)		7 (1-22)		315 (58-1024)		8 (1-25)		838 (154-2722)		21 (4-67)	
2002	Colombia									292 (56-940)		7 (1-23)		331 (63-1068)		8 (2-27)		881 (168-2840)		22 (4-71)	
2003	Colombia									318 (63-1017)		8 (2-25)		361 (71-1155)		9 (2-29)		961 (190-3070)		24 (5-76)	
2004	Colombia									304 (62-963)		8 (2-24)		346 (71-1094)		9 (2-27)		919 (189-2907)		23 (5-72)	
2005	Colombia									347 (74-1089)		9 (2-27)		395 (84-1236)		10 (2-31)		1049 (224-3287)		26 (6-81)	
2006	Colombia									338 (72-1053)		8 (2-26)		384 (82-1196)		9 (2-29)		1020 (219-3181)		25 (5-78)	
2007	Colombia									251 (54-780)		6 (1-19)		285 (62-886)		7 (2-22)		758 (164-2356)		19 (4-58)	
2008	Colombia									235 (51-727)		6 (1-18)		266 (58-825)		7 (1-21)		708 (154-2194)		18 (4-55)	
2009	Colombia									236 (52-729)		6 (1-19)		268 (59-828)		7 (2-21)		712 (156-2201)		18 (4-56)	
2010	Colombia									180 (40-556)		5 (1-14)		205 (45-631)		5 (1-16)		545 (120-1678)		14 (3-43)	
2011	Colombia									145 (32-447)		4 (1-12)		165 (37-508)		4 (1-13)		439 (98-1350)		12 (3-35)	
2012	Colombia									87 (20-268)		2 (1-7)		99 (22-304)		3 (1-8)		264 (59-809)		7 (2-22)	
2013	Colombia									93 (21-284)		2 (1-8)		106 (24-323)		3 (1-9)		281 (64-859)		8 (2-23)	
2014	Colombia									66 (15-200)		2 (0-5)		75 (17-228)		2 (0-6)		198 (45-605)		5 (1-16)	
2015	Colombia	11394 (9831-13545)	27 (23-32)	310 (267-368)	1 (1-1)	4449 (3333-5075)	10 (8-12)	121 (91-138)	0 (0-0)	55 (13-168)	0 (0-0)	1 (0-5)	0 (0-0)	63 (14-191)	0 (0-0)	2 (0-5)	0 (0-0)	166 (38-507)	0 (0-1)	5 (1-14)	0 (0-0)
2000	Comoros	2996 (2585-3561)		3392 (2927-4032)		1167 (874-1331)		1322 (990-1507)		29 (12-59)		32 (14-66)		11 (5-23)		13 (6-27)		86 (37-177)		98 (42-200)	
2001	Comoros									29 (12-59)		32 (14-66)		12 (5-24)		13 (5-26)		87 (37-178)		96 (41-198)	
2002	Comoros									29 (12-60)		32 (13-65)		12 (5-24)		13 (5-26)		88 (37-182)		95 (40-198)	
2003	Comoros									29 (12-60)		31 (13-65)		12 (5-24)		12 (5-26)		88 (36-182)		94 (39-195)	
2004	Comoros									29 (12-60)		30 (12-63)		11 (5-24)		12 (5-25)		87 (36-182)		91 (38-191)	
2005	Comoros									28 (11-59)		29 (12-61)		11 (5-24)		12 (5-24)		84 (34-178)		87 (36-184)	
2006	Comoros									27 (11-58)		28 (11-59)		11 (4-23)		11 (4-24)		82 (33-175)		84 (34-177)	
2007	Comoros									27 (11-57)		26 (10-56)		11 (4-23)		11 (4-23)		80 (32-172)		80 (32-170)	
2008	Comoros									26 (10-56)		25 (10-54)		10 (4-22)		10 (4-22)		79 (31-169)		77 (30-164)	
2009	Comoros									25 (10-54)		24 (9-52)		10 (4-22)		10 (4-21)		76 (29-164)		73 (28-157)	
2010	Comoros									24 (9-53)		23 (9-49)		10 (4-21)		9 (3-20)		73 (28-159)		69 (26-149)	
2011	Comoros									23 (9-51)		21 (8-47)		9 (4-20)		9 (3-19)		71 (27-154)		65 (24-141)	
2012	Comoros									22 (8-49)		20 (8-44)		9 (3-20)		8 (3-18)		68 (25-148)		61 (23-133)	
2013	Comoros									22 (8-48)		19 (7-42)		9 (3-19)		8 (3-17)		65 (24-144)		57 (21-126)	
2014	Comoros									21 (8-46)		18 (7-40)		8 (3-19)		7 (3-16)		63 (23-140)		55 (20-121)	
2015	Comoros	2586 (2231-3074)	0 (0-0)	2204 (1902-2620)	0 (0-0)	1016 (762-1159)	0 (0-0)	867 (649-988)	0 (0-0)	20 (6-47)	0 (0-0)	17 (5-40)	0 (0-0)	23 (7-53)	0 (0-0)	20 (6-45)	0 (0-0)	61 (18-141)	0 (0-0)	52 (16-120)	0 (0-0)
2000	Congo	8154 (7036-9693)		1641 (1416-1951)		3192 (2392-3641)		643 (481-733)		130 (51-281)		26 (10-57)		52 (20-113)		11 (4-23)		394 (153-848)		79 (31-171)	
2001	Congo									137 (53-294)		27 (10-57)		55 (21-118)		11 (4-23)		413 (161-887)		80 (31-173)	
2002	Congo									139 (55-298)		26 (10-57)		56 (22-120)		11 (4-23)		420 (165-901)		80 (31-171)	
2003	Congo									139 (55-296)		26 (10-55)		56 (22-119)		10 (4-22)		419 (166-895)		78 (31-166)	
2004	Congo									139 (56-295)		25 (10-53)		56 (22-119)		10 (4-21)		419 (168-892)		76 (30-162)	
2005	Congo									138 (56-291)		24 (10-51)		55 (22-117)		10 (4-21)		415 (168-880)		73 (30-155)	
2006	Congo									134 (55-284)		23 (9-48)		54 (22-114)		9 (4-19)		405 (165-857)		69 (28-146)	
2007	Congo									130 (54-275)		22 (9-45)		52 (21-110)		9 (4-18)		393 (162-830)		65 (27-137)	
2008	Congo									124 (43-270)		20 (7-43)		141 (49-306)		23 (8-49)		374 (131-814)		60 (21-130)	
2009	Congo									116 (41-252)		18 (6-39)		132 (46-286)		20 (7-44)		350 (123-760)		54 (19-118)	
2010	Congo									107 (38-233)		16 (6-35)		122 (43-265)		18 (7-40)		324 (115-704)		49 (17-106)	
2011	Congo									101 (36-218)		15 (5-32)		114 (41-248)		17 (6-36)		304 (109-658)		44 (16-96)	
2012	Congo									91 (33-197)		13 (5-28)		104 (37-224)		15 (5-32)		275 (99-596)		39 (14-85)	
2013	Congo									57 (21-124)		8 (3-17)		65 (24-141)		9 (3-20)		173 (63-374)		24 (9-52)	
2014	Congo									48 (17-102)		6 (2-14)		54 (20-116)		7 (3-16)		144 (53-309)		20 (7-42)	
2015	Congo	7100 (6126-8440)	1506 (1300-1791)	951 (821-1131)	202 (174-240)	2788 (2089-3180)	592 (443-675)	374 (280-426)	79 (59-90)	45 (17-97)	10 (4-21)	6 (2-13)	1 (0-3)	51 (19-110)	11 (4-23)	7 (3-15)	1 (1-3)	136 (50-292)	29 (11-62)	18 (7-39)	4 (1-8)
2000	Cook Islands	43 (37-51)		2130 (1838-2532)		17 (12-19)		828 (620-944)		0 (0-0)		11 (5-19)		0 (0-0)		12 (5-21)		1 (0-1)		32 (14-57)	
2001	Cook Islands									0 (0-0)		11 (5-19)		0 (0-0)		12 (6-22)		1 (0-1)		33 (15-58)	
2002	Cook Islands									0 (0-0)		11 (5-20)		0 (0-0)		13 (6-22)		1 (0-1)		33 (15-59)	
2003	Cook Islands									0 (0-0)		7 (3-13)		0 (0-0)		8 (4-15)		0 (0-1)		23 (10-40)	
2004	Cook Islands									0 (0-0)		7 (3-13)		0 (0-0)		8 (4-15)		0 (0-1)		22 (10-40)	
2005	Cook Islands									0 (0-0)		11 (5-19)		0 (0-0)		12 (6-22)		1 (0-1)		33 (15-59)	
2006	Cook Islands									0 (0-0)		7 (3-12)		0 (0-0)		8 (4-14)		0 (0-1)		21 (9-37)	
2007	Cook Islands									0 (0-0)		7 (3-12)		0 (0-0)		7 (3-13)		0 (0-1)		20 (9-35)	
2008	Cook Islands									0 (0-0)		6 (3-11)		0 (0-0)		7 (3-12)		0 (0-1)		18 (8-32)	
2009	Cook Islands									0 (0-0)		12 (5-21)		0 (0-0)		13 (6-23)		1 (0-1)		35 (16-62)	
2010	Cook Islands									0 (0-0)		12 (5-22)		0 (0-0)		14 (6-24)		1 (0-1)		37 (16-65)	
2011	Cook Islands									0 (0-0)		12 (5-21)		0 (0-0)		13 (6-23)		1 (0-1)		35 (16-62)	
2012	Cook Islands									0 (0-0)		6 (3-10)		0 (0-0)		7 (3-12)		0 (0-1)		17 (8-31)	
2013	Cook Islands									0 (0-0)		6 (3-10)		0 (0-0)		6 (3-11)		0 (0-1)		17 (8-30)	
2014	Cook Islands									0 (0-0)		6 (3-10)		0 (0-0)		7 (3-12)		0 (0-1)		18 (8-32)	
2015	Cook Islands	43 (37-51)	0 (0-0)	2496 (2154-2967)	0 (0-0)	17 (13-19)	0 (0-0)	976 (731-1113)	0 (0-0)	0 (0-0)	0 (0-0)	6 (3-10)	0 (0-0)	0 (0-0)	0 (0-0)	7 (3-12)	0 (0-0)	0 (0-1)	0 (0-0)	18 (8-31)	0 (0-0)
2000	Costa Rica	5660 (4883-6728)		1461 (1260-1736)		2196 (1645-2505)		567 (425-647)		11 (3-32)		3 (1-8)		13 (4-37)		3 (1-10)		35 (9-98)		9 (2-25)	
2001	Costa Rica									10 (3-27)		2 (1-7)		11 (3-31)		3 (1-8)		29 (8-81)		8 (2-21)	
2002	Costa Rica									19 (5-54)		5 (1-14)		22 (6-62)		6 (2-16)		59 (16-164)		16 (4-44)	
2003	Costa Rica									6 (2-18)		2 (0-5)		7 (2-21)		2 (1-6)		20 (6-55)		5 (2-15)	
2004	Costa Rica									4 (1-12)		1 (0-3)		5 (1-13)		1 (0-4)		13 (4-35)		4 (1-10)	
2005	Costa Rica									10 (3-27)		3 (1-8)		11 (3-31)		3 (1-9)		29 (8-82)		8 (2-23)	
2006	Costa Rica									8 (2-23)		2 (1-7)		10 (3-26)		3 (1-8)		25 (7-70)		7 (2-20)	
2007	Costa Rica									9 (3-26)		3 (1-7)		11 (3-30)		3 (1-9)		28 (8-79)		8 (2-23)	
2008	Costa Rica									14 (4-39)		4 (1-11)		16 (5-44)		4 (1-12)		42 (12-116)		12 (3-33)	
2009	Costa Rica									10 (3-28)		3 (1-8)		11 (3-32)		3 (1-9)		30 (9-84)		9 (2-24)	
2010	Costa Rica									8 (2-23)		2 (1-7)		9 (3-26)		3 (1-7)		25 (7-70)		7 (2-20)	
2011	Costa Rica									9 (3-25)		2 (1-7)		10 (3-28)		3 (1-8)		27 (8-75)		7 (2-21)	
2012	Costa Rica									9 (2-24)		2 (1-7)		10 (3-27)		3 (1-7)		26 (7-71)		7 (2-20)	
2013	Costa Rica									3 (1-9)		1 (0-2)		4 (1-10)		1 (0-3)		10 (3-27)		3 (1-8)	
2014	Costa Rica									3 (1-9)		1 (0-3)		4 (1-10)		1 (0-3)		10 (3-28)		3 (1-8)	
2015	Costa Rica	938 (810-1115)	4 (3-4)	272 (235-324)	1 (1-1)	365 (273-416)	1 (1-2)	106 (79-121)	0 (0-0)	3 (1-8)	0 (0-0)	1 (0-2)	0 (0-0)	3 (1-9)	0 (0-0)	1 (0-3)	0 (0-0)	9 (3-24)	0 (0-0)	3 (1-7)	0 (0-0)
2000	Cote d'Ivoire	62495 (53922-74288)		2233 (1927-2654)		24639 (18459-28103)		880 (660-1004)		499 (187-1096)		18 (7-39)		200 (75-440)		7 (3-16)		1506 (565-3309)		54 (20-118)	
2001	Cote d'Ivoire									495 (185-1089)		17 (6-38)		199 (74-437)		7 (3-15)		1495 (559-3289)		52 (19-115)	
2002	Cote d'Ivoire									487 (181-1072)		17 (6-37)		195 (73-430)		7 (2-15)		1470 (547-3238)		50 (19-111)	
2003	Cote d'Ivoire									478 (177-1054)		16 (6-36)		192 (71-423)		6 (2-14)		1443 (535-3183)		49 (18-108)	
2004	Cote d'Ivoire									466 (172-1029)		16 (6-34)		187 (69-413)		6 (2-14)		1407 (520-3109)		47 (17-104)	
2005	Cote d'Ivoire									470 (173-1040)		16 (6-34)		189 (69-417)		6 (2-14)		1420 (522-3141)		47 (17-104)	
2006	Cote d'Ivoire									481 (176-1066)		16 (6-35)		193 (71-428)		6 (2-14)		1454 (532-3220)		48 (17-105)	
2007	Cote d'Ivoire									509 (187-1125)		16 (6-36)		204 (75-451)		7 (2-15)		1536 (566-3398)		50 (18-110)	
2008	Cote d'Ivoire									535 (198-1180)		17 (6-38)		214 (79-473)		7 (3-15)		1615 (598-3565)		52 (19-114)	
2009	Cote d'Ivoire									586 (218-1291)		18 (7-41)		235 (87-518)		7 (3-16)		1769 (658-3897)		56 (21-122)	
2010	Cote d'Ivoire									633 (236-1392)		20 (7-43)		254 (95-558)		8 (3-17)		1911 (714-4202)		59 (22-130)	
2011	Cote d'Ivoire									676 (254-1483)		20 (8-45)		271 (102-595)		8 (3-18)		2040 (766-4477)		62 (23-136)	
2012	Cote d'Ivoire									701 (263-1536)		21 (8-46)		281 (105-616)		8 (3-18)		2115 (794-4638)		63 (24-138)	
2013	Cote d'Ivoire									730 (275-1597)		21 (8-46)		293 (110-641)		8 (3-19)		2204 (831-4823)		64 (24-140)	
2014	Cote d'Ivoire									742 (281-1620)		21 (8-46)		297 (113-650)		8 (3-18)		2239 (849-4893)		64 (24-139)	
2015	Cote d'Ivoire	27641 (23850-32858)	4004 (3455-4760)	767 (661-911)	111 (96-132)	10768 (8068-12282)	1560 (1169-1779)	299 (224-341)	43 (32-49)	520 (198-1133)	75 (29-164)	14 (5-31)	2 (1-5)	208 (79-455)	30 (12-66)	6 (2-13)	1 (0-2)	1569 (598-3422)	227 (87-496)	44 (17-95)	6 (2-14)
2000	Croatia	274 (237-326)		115 (99-137)		178 (133-203)		75 (56-85)		13 (6-23)		5 (2-10)		15 (7-27)		6 (3-11)		39 (18-70)		16 (7-30)	
2001	Croatia									15 (7-27)		6 (3-11)		17 (8-30)		7 (3-13)		45 (20-81)		18 (8-34)	
2002	Croatia									16 (7-29)		7 (3-13)		18 (8-33)		8 (4-14)		49 (22-89)		21 (9-38)	

Spn = *Streptococcus pneumoniae*; npnm = non-pneumonia, non-meningitis; HIV = cases only in HIV-infected children; Uncertainty range provided in parentheses.

Year	Country	Spn pneumonia cases (UR)	Spn pneumonia cases, HIV (UR)	Spn pneumonia incidence rate (UR)	Spn pneumonia incidence rate, HIV (UR)	Spn severe pneumonia cases (UR)	Spn severe pneumonia cases, HIV (UR)	Spn severe pneumonia incidence rate (UR)	Spn severe pneumonia incidence rate, HIV (UR)	Spn meningitis cases (UR)	Spn meningitis cases, HIV (UR)	Spn meningitis incidence rate (UR)	Spn meningitis incidence rate, HIV (UR)	Spn NPNM cases (UR)	Spn NPNM cases, HIV (UR)	Spn NPNM incidence rate (UR)	Spn NPNM incidence rate, HIV (UR)	Spn NPNM non-severe cases (UR)	Spn NPNM non-severe cases, HIV (UR)	SPN NPNM non-severe incidence rate (UR)	SPN NPNM non-severe incidence rate, HIV (UR)
2003	Croatia									8 (4-15)		4 (2-7)		10 (4-17)		4 (2-8)		25 (12-46)		11 (5-21)	
2004	Croatia									3 (2-6)		2 (1-3)		4 (2-7)		2 (1-3)		10 (5-19)		5 (2-9)	
2005	Croatia									22 (10-40)		11 (5-20)		25 (11-46)		12 (6-23)		67 (30-121)		33 (15-60)	
2006	Croatia									4 (2-7)		2 (1-3)		4 (2-7)		2 (1-4)		11 (5-20)		6 (3-10)	
2007	Croatia									0 (0-0)		0 (0-0)		0 (0-0)		0 (0-0)		0 (0-0)		0 (0-0)	
2008	Croatia									11 (5-19)		5 (2-10)		12 (6-22)		6 (3-11)		32 (15-59)		16 (7-29)	
2009	Croatia									16 (7-29)		8 (3-14)		18 (8-33)		9 (4-16)		48 (22-87)		23 (10-41)	
2010	Croatia									16 (7-29)		8 (3-14)		18 (8-33)		9 (4-15)		49 (22-88)		23 (10-41)	
2011	Croatia									14 (6-25)		6 (3-11)		16 (7-29)		7 (3-13)		42 (19-77)		19 (9-35)	
2012	Croatia									23 (10-41)		10 (5-19)		26 (12-47)		12 (5-21)		69 (31-125)		31 (14-56)	
2013	Croatia									7 (3-14)		3 (2-6)		9 (4-15)		4 (2-7)		23 (10-41)		10 (5-19)	
2014	Croatia									13 (6-24)		6 (3-11)		15 (7-27)		7 (3-13)		39 (18-71)		19 (9-34)	
2015	Croatia	234 (202-279)	0 (0-0)	115 (99-137)	0 (0-0)	152 (114-173)	0 (0-0)	75 (56-85)	0 (0-0)	12 (5-21)	0 (0-0)	6 (3-10)	0 (0-0)	13 (6-24)	0 (0-0)	7 (3-12)	0 (0-0)	36 (16-65)	0 (0-0)	17 (8-32)	0 (0-0)
2000	Cuba	1695 (1462-2014)		230 (198-273)		1099 (823-1253)		149 (112-170)		154 (76-284)		21 (10-39)		175 (86-323)		24 (12-44)		466 (229-858)		63 (31-116)	
2001	Cuba									114 (56-210)		16 (8-29)		130 (64-239)		18 (9-32)		345 (170-634)		47 (23-86)	
2002	Cuba									58 (29-107)		8 (4-15)		66 (33-122)		9 (5-17)		176 (87-323)		24 (12-45)	
2003	Cuba									89 (44-163)		13 (6-23)		101 (50-185)		14 (7-26)		268 (133-491)		38 (19-69)	
2004	Cuba									71 (35-130)		10 (5-19)		81 (40-147)		12 (6-21)		214 (106-391)		31 (15-57)	
2005	Cuba									89 (44-162)		13 (7-24)		101 (50-184)		15 (7-27)		268 (133-488)		40 (20-73)	
2006	Cuba									73 (36-132)		11 (6-20)		83 (41-150)		13 (6-23)		220 (110-400)		34 (17-61)	
2007	Cuba									54 (27-98)		8 (4-15)		61 (31-111)		10 (5-17)		163 (81-296)		26 (13-46)	
2008	Cuba									72 (36-130)		11 (6-21)		81 (41-147)		13 (6-24)		216 (108-392)		35 (17-63)	
2009	Cuba									44 (22-80)		7 (4-13)		50 (25-91)		8 (4-15)		133 (67-242)		22 (11-39)	
2010	Cuba									49 (25-89)		8 (4-15)		55 (28-101)		9 (5-17)		147 (74-268)		24 (12-44)	
2011	Cuba									67 (35-125)		11 (6-21)		76 (39-142)		13 (7-24)		203 (104-378)		34 (17-63)	
2012	Cuba									69 (33-132)		12 (6-22)		78 (37-150)		13 (6-25)		207 (100-399)		35 (17-67)	
2013	Cuba									45 (20-89)		8 (3-15)		51 (23-101)		9 (4-17)		135 (61-269)		23 (10-46)	
2014	Cuba									52 (22-107)		9 (4-18)		59 (25-122)		10 (4-21)		158 (68-324)		27 (12-55)	
2015	Cuba	1329 (1147-1580)	0 (0-0)	230 (198-273)	0 (0-0)	862 (646-983)	0 (0-0)	149 (112-170)	0 (0-0)	50 (22-104)	0 (0-0)	9 (4-18)	0 (0-0)	57 (25-119)	0 (0-0)	10 (4-21)	0 (0-0)	152 (66-315)	0 (0-0)	26 (11-55)	0 (0-0)
2000	Cyprus	154 (133-183)		230 (198-273)		100 (75-114)		149 (112-170)		6 (3-8)		9 (5-12)		7 (4-9)		10 (6-13)		17 (10-24)		26 (16-36)	
2001	Cyprus									5 (3-8)		8 (5-12)		6 (4-9)		10 (6-13)		16 (10-23)		25 (15-35)	
2002	Cyprus									5 (3-7)		8 (5-11)		6 (3-8)		9 (5-12)		15 (9-21)		24 (14-32)	
2003	Cyprus									5 (3-7)		8 (5-11)		6 (3-8)		9 (5-12)		15 (9-20)		23 (14-32)	
2004	Cyprus									4 (3-6)		7 (4-9)		5 (3-7)		8 (5-11)		13 (8-18)		21 (12-29)	
2005	Cyprus									4 (2-5)		6 (4-8)		4 (2-6)		7 (4-9)		11 (7-15)		18 (11-25)	
2006	Cyprus									4 (2-5)		6 (3-8)		4 (2-5)		7 (4-9)		11 (6-15)		18 (11-24)	
2007	Cyprus									3 (2-4)		5 (3-7)		3 (2-5)		6 (3-8)		9 (6-13)		15 (9-21)	
2008	Cyprus									3 (2-4)		5 (3-6)		3 (2-4)		5 (3-7)		9 (5-12)		14 (8-19)	
2009	Cyprus									2 (1-3)		4 (2-5)		3 (2-4)		4 (3-6)		7 (4-10)		12 (7-16)	
2010	Cyprus									2 (1-3)		4 (2-5)		3 (2-3)		4 (2-6)		7 (4-9)		11 (6-15)	
2011	Cyprus									2 (1-3)		3 (2-4)		2 (1-3)		4 (2-5)		6 (4-8)		10 (6-13)	
2012	Cyprus									2 (1-3)		3 (2-4)		2 (1-3)		3 (2-4)		5 (3-8)		9 (5-12)	
2013	Cyprus									2 (1-2)		3 (2-3)		2 (1-3)		3 (2-4)		5 (3-7)		8 (5-11)	
2014	Cyprus									1 (1-2)		2 (1-3)		2 (1-2)		3 (2-4)		4 (3-6)		7 (4-10)	
2015	Cyprus	110 (95-131)	0 (0-0)	171 (147-203)	0 (0-0)	71 (53-81)	0 (0-0)	111 (83-126)	0 (0-0)	1 (1-1)	0 (0-0)	1 (1-2)	0 (0-0)	1 (1-1)	0 (0-0)	2 (1-2)	0 (0-0)	3 (2-4)	0 (0-0)	4 (3-6)	0 (0-0)
2000	Czech Republic	505 (436-600)		115 (99-137)		327 (245-373)		75 (56-85)		17 (8-32)		4 (2-7)		20 (9-36)		4 (2-8)		52 (24-95)		12 (5-22)	
2001	Czech Republic									19 (9-34)		5 (2-8)		21 (10-39)		5 (2-9)		57 (26-103)		14 (6-25)	
2002	Czech Republic									13 (6-23)		3 (1-6)		14 (7-26)		4 (2-6)		38 (17-69)		9 (4-17)	
2003	Czech Republic									14 (6-26)		3 (2-6)		16 (7-29)		4 (2-7)		43 (19-78)		10 (5-19)	
2004	Czech Republic									35 (16-64)		8 (4-14)		40 (18-72)		9 (4-16)		106 (48-192)		24 (11-44)	
2005	Czech Republic									20 (9-37)		4 (2-8)		23 (11-42)		5 (2-9)		62 (28-112)		13 (6-24)	
2006	Czech Republic									9 (4-16)		2 (1-3)		10 (4-18)		2 (1-4)		26 (12-47)		5 (2-9)	
2007	Czech Republic									37 (17-67)		7 (3-13)		42 (19-76)		8 (4-15)		112 (51-202)		21 (10-39)	
2008	Czech Republic									13 (6-24)		2 (1-4)		15 (7-27)		3 (1-5)		40 (18-73)		7 (3-14)	
2009	Czech Republic									35 (16-63)		6 (3-11)		39 (18-71)		7 (3-13)		105 (47-190)		19 (9-35)	
2010	Czech Republic									11 (5-20)		2 (1-4)		12 (6-22)		2 (1-4)		33 (15-59)		6 (3-11)	
2011	Czech Republic									21 (9-37)		4 (2-7)		23 (11-43)		4 (2-8)		62 (28-113)		11 (5-20)	
2012	Czech Republic									12 (6-22)		2 (1-4)		14 (6-26)		2 (1-5)		37 (17-68)		7 (3-12)	
2013	Czech Republic									15 (7-28)		3 (1-5)		17 (8-31)		3 (1-6)		46 (21-83)		8 (4-15)	
2014	Czech Republic									14 (6-26)		3 (1-5)		16 (7-29)		3 (1-5)		43 (19-77)		8 (4-14)	
2015	Czech Republic	608 (525-723)	0 (0-0)	115 (99-137)	0 (0-0)	394 (295-450)	0 (0-0)	75 (56-85)	0 (0-0)	14 (6-25)	0 (0-0)	3 (1-5)	0 (0-0)	16 (7-28)	0 (0-0)	3 (1-5)	0 (0-0)	42 (19-76)	0 (0-0)	8 (4-14)	0 (0-0)
2000	Democratic People's Republic of Korea	50970 (43978-60589)		2614 (2255-3107)		19977 (14966-22785)		1024 (767-1168)		1291 (619-2007)		66 (32-103)		1467 (703-2280)		75 (36-117)		3899 (1868-6061)		200 (96-311)	
2001	Democratic People's Republic of Korea									1030 (499-1619)		54 (26-85)		1170 (567-1839)		61 (30-96)		3110 (1507-4888)		163 (79-256)	
2002	Democratic People's Republic of Korea									802 (401-1246)		42 (21-66)		911 (456-1416)		48 (24-75)		2423 (1212-3763)		128 (64-199)	
2003	Democratic People's Republic of Korea									625 (325-993)		33 (17-53)		710 (370-1128)		38 (20-60)		1889 (983-2999)		100 (52-159)	
2004	Democratic People's Republic of Korea									499 (270-822)		27 (14-44)		567 (306-933)		30 (16-50)		1506 (815-2481)		80 (43-132)	
2005	Democratic People's Republic of Korea									428 (235-730)		23 (13-39)		486 (267-829)		26 (14-45)		1292 (709-2204)		70 (38-119)	
2006	Democratic People's Republic of Korea									387 (204-681)		21 (11-37)		439 (231-773)		24 (13-42)		1168 (615-2056)		64 (33-112)	
2007	Democratic People's Republic of Korea									358 (181-648)		20 (10-36)		406 (206-736)		23 (11-41)		1080 (547-1956)		60 (30-108)	
2008	Democratic People's Republic of Korea									324 (158-602)		18 (9-34)		368 (179-684)		21 (10-39)		979 (476-1818)		56 (27-103)	
2009	Democratic People's Republic of Korea									300 (140-570)		17 (8-33)		341 (159-647)		20 (9-38)		905 (424-1721)		53 (25-100)	
2010	Democratic People's Republic of Korea									279 (126-541)		16 (7-32)		317 (143-614)		19 (8-36)		842 (380-1633)		50 (22-97)	
2011	Democratic People's Republic of Korea									263 (114-520)		16 (7-31)		299 (130-590)		18 (8-36)		794 (345-1570)		48 (21-94)	
2012	Democratic People's Republic of Korea									250 (81-607)		15 (5-37)		284 (92-690)		17 (6-41)		756 (244-1834)		45 (15-110)	
2013	Democratic People's Republic of Korea									241 (74-594)		14 (4-35)		274 (84-675)		16 (5-40)		729 (223-1793)		43 (13-107)	
2014	Democratic People's Republic of Korea									231 (67-576)		14 (4-34)		262 (76-654)		15 (4-38)		698 (202-1739)		41 (12-102)	
2015	Democratic People's Republic of Korea	18438 (15909-21918)	8 (6-9)	1073 (926-1276)	0 (0-1)	7172 (5373-8180)	3 (2-3)	417 (313-476)	0 (0-0)	218 (60-550)	0 (0-0)	13 (3-32)	0 (0-0)	248 (68-625)	0 (0-0)	14 (4-36)	0 (0-0)	659 (182-1661)	0 (0-1)	38 (11-97)	0 (0-0)
2000	Democratic Republic of the Congo	274584 (236917-326404)		3078 (2656-3659)		107874 (80819-123041)		1209 (906-1379)		3803 (2032-7200)		43 (23-81)		1525 (815-2888)		17 (9-32)		11483 (6137-21741)		129 (69-244)	
2001	Democratic Republic of the Congo									3783 (2033-7172)		41 (22-78)		1517 (815-2877)		17 (9-31)		11423 (6138-21657)		124 (67-236)	
2002	Democratic Republic of the Congo									3828 (2075-7267)		40 (22-77)		1535 (832-2915)		16 (9-31)		11558 (6265-21944)		122 (66-232)	
2003	Democratic Republic of the Congo									3852 (1431-8378)		39 (15-86)		1545 (574-3360)		16 (6-34)		11632 (4320-25299)		119 (44-259)	
2004	Democratic Republic of the Congo									3942 (1482-8582)		39 (15-85)		1581 (594-3442)		16 (6-34)		11903 (4474-25914)		118 (44-257)	
2005	Democratic Republic of the Congo									3855 (1465-8391)		37 (14-81)		1546 (587-3366)		15 (6-32)		11641 (4423-25339)		112 (42-243)	
2006	Democratic Republic of the Congo									3975 (1525-8641)		37 (14-80)		1595 (612-3466)		15 (6-32)		12004 (4605-26091)		111 (43-242)	
2007	Democratic Republic of the Congo									3996 (1546-8664)		36 (14-78)		1603 (620-3475)		14 (6-31)		12068 (4670-26163)		108 (42-235)	
2008	Democratic Republic of the Congo									4209 (1620-9133)		37 (14-80)		1688 (650-3663)		15 (6-32)		12710 (4891-27579)		111 (43-241)	
2009	Democratic Republic of the Congo									4648 (1779-10097)		40 (15-86)		1864 (714-4050)		16 (6-35)		14034 (5373-30489)		120 (46-260)	
2010	Democratic Republic of the Congo									5068 (1930-11022)		42 (16-92)		2033 (774-4421)		17 (6-37)		15302 (5828-33283)		127 (48-277)	
2011	Democratic Republic of the Congo									5475 (2094-11898)		44 (17-96)		2196 (840-4772)		18 (7-39)		16532 (6322-35928)		134 (51-290)	
2012	Democratic Republic of the Congo									5499 (2111-11937)		43 (17-94)		2206 (847-4788)		17 (7-38)		16605 (6373-36044)		131 (50-284)	
2013	Democratic Republic of the Congo									5077 (1956-11008)		39 (15-85)		2037 (785-4415)		16 (6-34)		15332 (5906-33241)		118 (45-256)	
2014	Democratic Republic of the Congo									4252 (1637-9215)		32 (12-69)		1705 (657-3696)		13 (5-28)		12839 (4944-27826)		96 (37-209)	
2015	Democratic Republic of the Congo	186642 (161039-221865)	12312 (10623-14636)	1368 (1180-1626)	90 (78-107)	73474 (55046-83804)	4847 (3631-5528)	538 (403-614)	36 (27-41)	3678 (1424-7959)	243 (94-525)	27 (10-58)	2 (1-4)	1475 (571-3192)	97 (38-211)	11 (4-23)	1 (0-2)	11106 (4301-24033)	733 (284-1585)	81 (32-176)	5 (2-12)
2000	Denmark	383 (330-455)		115 (99-137)		248 (186-283)		75 (56-85)		18 (8-32)		5 (2-10)		20 (9-36)		6 (3-11)		53 (24-97)		16 (7-29)	
2001	Denmark									22 (10-40)		7 (3-12)		25 (11-45)		8 (3-14)		66 (30-119)		20 (9-37)	
2002	Denmark									17 (8-32)		5 (2-10)		20 (9-36)		6 (3-11)		53 (24-95)		16 (7-30)	
2003	Denmark									17 (8-31)		5 (2-10)		20 (9-36)		6 (3-11)		52 (24-94)		16 (7-29)	
2004	Denmark									20 (9-36)		6 (3-11)		22 (10-40)		7 (3-13)		59 (27-107)		18 (8-33)	
2005	Denmark									10 (4-18)		3 (1-6)		11 (5-20)		4 (2-6)		30 (14-54)		9 (4-17)	
2006	Denmark									4 (2-7)		1 (1-2)		5 (2-8)		1 (1-3)		12 (6-23)		4 (2-7)	
2007	Denmark									10 (5-18)		3 (1-6)		11 (5-21)		4 (2-6)		30 (14-55)		9 (4-17)	
2008	Denmark									6 (3-11)		2 (1-3)		7 (3-12)		2 (1-4)		18 (8-33)		6 (3-10)	
2009	Denmark									4 (2-7)		1 (1-2)		4 (2-8)		1 (1-2)		11 (5-21)		4 (2-6)	
2010	Denmark									4 (2-7)		1 (1-2)		4 (2-8)		1 (1-2)		11 (5-21)		4 (2-6)	
2011	Denmark									3 (1-6)		1 (0-2)		4 (2-7)		1 (1-2)		10 (4-18)		3 (1-6)	
2012	Denmark									2 (1-4)		1 (0-1)		3 (1-5)		1 (0-2)		7 (3-13)		2 (1-4)	
2013	Denmark									1 (1-3)		0 (0-1)		2 (1-3)		1 (0-1)		4 (2-8)		1 (1-2)	
2014	Denmark									1 (0-2)		0 (0-1)		1 (1-2)		0 (0-1)		3 (1-6)		1 (0-2)	
2015	Denmark	72 (62-85)	0 (0-0)	25 (21-29)	0 (0-0)	47 (35-53)	0 (0-0)	16 (12-18)	0 (0-0)	1 (0-2)	0 (0-0)	0 (0-1)	0 (0-0)	1 (0-2)	0 (0-0)	0 (0-1)	0 (0-0)	3 (1-5)	0 (0-0)	1 (0-2)	0 (0-0)
2000	Djibouti	1435 (1238-1706)		1444 (1246-1716)		557 (417-635)		560 (420-639)		31 (14-62)		31 (14-62)		12 (6-25)		13 (6-25)		94 (43-186)		94 (43-187)	
2001	Djibouti									30 (14-60)		31 (14-61)		12 (6-24)		12 (6-24)		91 (42-181)		92 (42-183)	
2002	Djibouti									30 (13-59)		30 (14-59)		12 (5-24)		12 (5-24)		89 (41-177)		91 (41-179)	
2003	Djibouti									29 (13-57)		29 (13-58)		12 (5-23)		12 (5-23)		87 (40-173)		88 (40-175)	
2004	Djibouti									28 (13-56)		29 (13-57)		11 (5-22)		11 (5-23)		85 (39-168)		86 (39-171)	
2005	Djibouti									27 (12-54)		28 (13-55)		11 (5-22)		11 (5-22)		83 (38-163)		84 (38-166)	
2006	Djibouti									27 (12-53)		28 (12-54)		11 (5-21)		11 (5-22)		82 (37-161)		83 (37-163)	
2007	Djibouti									27 (13-53)		28 (13-53)		11 (5-21)		11 (5-21)		83 (39-159)		84 (40-160)	
2008	Djibouti									28 (14-52)		29 (14-53)		11 (6-21)		12 (6-21)		85 (43-158)		87 (44-161)	
2009	Djibouti									30 (16-53)		31 (16-55)		12 (6-21)		12 (6-22)		90 (48-161)		92 (49-165)	
2010	Djibouti									33 (18-56)		33 (19-57)		13 (7-22)		13 (8-23)		98 (55-169)		101 (57-173)	
2011	Djibouti									37 (21-62)		38 (21-63)		42 (24-70)		43 (24-72)		111 (63-186)		114 (65-191)	
2012	Djibouti									43 (24-68)		43 (25-69)		49 (27-77)		49 (28-78)		129 (73-204)		131 (74-207)	
2013	Djibouti									31 (16-48)		31 (16-48)		35 (18-54)		35 (19-55)		92 (49-144)		93 (49-145)	
2014	Djibouti									29 (15-44)		29 (15-44)		33 (17-50)		33 (17-50)		87 (45-132)		87 (45-133)	
2015	Djibouti	849 (733-1010)	110 (95-131)	846 (730-1006)	110 (95-130)	333 (249-380)	43 (32-49)	332 (249-378)	43 (32-49)	27 (14-40)	3 (2-5)	27 (14-40)	3 (2-5)	31 (16-46)	4 (2-6)	30 (15-46)	4 (2-6)	81 (41-122)	11 (5-16)	81 (41-122)	10 (5-16)
2000	Dominica	100 (86-119)		1964 (1695-2335)		39 (29-44)		766 (574-874)		0 (0-0)		0 (0-0)		0 (0-0)		0 (0-0)		0 (0-0)		0 (0-0)	
2001	Dominica									1 (1-2)		25 (12-39)		1 (1-2)		29 (14-45)		4 (2-5)		76 (38-118)	
2002	Dominica									1 (0-1)		21 (10-33)		1 (1-2)		24 (12-37)		3 (1-4)		64 (31-99)	
2003	Dominica									1 (0-2)		23 (11-36)		1 (1-2)		26 (13-40)		3 (1-5)		69 (34-107)	
2004	Dominica									0 (0-0)		0 (0-0)		0 (0-0)		0 (0-0)		0 (0-0)		0 (0-0)	
2005	Dominica									0 (0-0)		0 (0-0)		0 (0-0)		0 (0-0)		0 (0-0)		0 (0-0)	
2006	Dominica									0 (0-0)		0 (0-0)		0 (0-0)		0 (0-0)		0 (0-0)		0 (0-0)	
2007	Dominica									0 (0-0)		0 (0-0)		0 (0-0)		0 (0-0)		0 (0-0)		0 (0-0)	
2008	Dominica									1 (0-1)		13 (7-21)		1 (0-1)		15 (7-24)		2 (1-3)		40 (20-63)	
2009	Dominica									1 (0-1)		12 (6-19)		1 (0-1)		14 (7-21)		2 (1-3)		37 (18-57)	
2010	Dominica									1 (0-1)		14 (7-21)		1 (0-1)		16 (8-24)		2 (1-3)		41 (20-64)	
2011	Dominica									0 (0-0)		0 (0-0)		0 (0-0)		0 (0-0)		0 (0-0)		0 (0-0)	
2012	Dominica									0 (0-0)		0 (0-0)		0 (0-0)		0 (0-0)		0 (0-0)		0 (0-0)	
2013	Dominica									0 (0-0)		0 (0-0)		0 (0-0)		0 (0-0)		0 (0-0)		0 (0-0)	
2014	Dominica									0 (0-0)		0 (0-0)		0 (0-0)		0 (0-0)		0 (0-0)		0 (0-0)	
2015	Dominica	56 (48-66)	0 (0-0)	1049 (905-1247)	0 (0-0)	22 (16-25)	0 (0-0)	407 (305-464)	0 (0-0)	0 (0-0)	0 (0-0)	0 (0-0)	0 (0-0)	0 (0-0)	0 (0-0)	0 (0-0)	0 (0-0)	0 (0-0)	0 (0-0)	0 (0-0)	0 (0-0)
2000	Dominican Republic	14464 (12480-17193)		1442 (1244-1714)		5634 (4221-6426)		562 (421-641)		57 (18-141)		6 (2-14)		64 (21-160)		6 (2-16)		171 (56-425)		17 (6-42)	
2001	Dominican Republic									52 (16-130)		5 (2-13)		59 (19-147)		6 (2-15)		157 (49-392)		16 (5-39)	
2002	Dominican Republic									49 (15-123)		5 (1-12)		55 (17-140)		6 (2-14)		147 (45-371)		15 (4-37)	
2003	Dominican Republic									47 (15-118)		5 (1-12)		53 (17-134)		5 (2-13)		142 (44-356)		14 (4-35)	
2004	Dominican Republic									46 (14-114)		4 (1-11)		52 (16-129)		5 (2-13)		138 (44-344)		13 (4-33)	
2005	Dominican Republic									44 (14-108)		4 (1-10)		49 (16-122)		5 (2-12)		131 (42-325)		13 (4-31)	
2006	Dominican Republic									42 (14-103)		4 (1-10)		47 (16-116)		5 (1-11)		126 (41-310)		12 (4-30)	
2007	Dominican Republic									39 (13-96)		4 (1-9)		45 (15-109)		4 (1-10)		119 (40-291)		11 (4-28)	
2008	Dominican Republic									38 (12-93)		4 (1-9)		43 (14-105)		4 (1-10)		114 (38-280)		11 (4-27)	
2009	Dominican Republic									36 (12-88)		3 (1-8)		41 (13-100)		4 (1-10)		108 (35-267)		10 (3-25)	
2010	Dominican Republic									35 (11-86)		3 (1-8)		40 (13-98)		4 (1-9)		106 (34-261)		10 (3-25)	
2011	Dominican Republic									34 (11-85)		3 (1-8)		39 (13-97)		4 (1-9)		104 (33-257)		10 (3-25)	
2012	Dominican Republic									34 (11-85)		3 (1-8)		39 (12-96)		4 (1-9)		103 (33-256)		10 (3-25)	
2013	Dominican Republic									35 (11-86)		3 (1-8)		40 (12-98)		4 (1-9)		105 (33-260)		10 (3-25)	
2014	Dominican Republic									31 (11-74)		3 (1-7)		35 (12-84)		3 (1-8)		93 (33-224)		9 (3-21)	
2015	Dominican Republic	10433 (9002-12402)	39 (34-46)	999 (862-1187)	4 (3-4)	4069 (3049-4641)	15 (11-17)	390 (292-444)	1 (1-2)	33 (12-80)	0 (0-0)	3 (1-8)	0 (0-0)	38 (13-91)	0 (0-0)	4 (1-9)	0 (0-0)	101 (36-242)	0 (0-1)	10 (3-23)	0 (0-0)
2000	Ecuador	21568 (18609-25638)		1444 (1246-1716)		8380 (6279-9559)		561 (420-640)		228 (87-400)		15 (6-27)		259 (99-454)		17 (7-30)		688 (263-1207)		46 (18-81)	
2001	Ecuador									216 (83-379)		14 (6-25)		245 (94-431)		16 (6-29)		653 (250-1145)		44 (17-76)	
2002	Ecuador									213 (81-370)		14 (5-25)		242 (92-420)		16 (6-28)		642 (244-1117)		43 (16-75)	
2003	Ecuador									340 (127-581)		23 (8-39)		387 (144-660)		26 (10-44)		1028 (383-1756)		69 (26-118)	
2004	Ecuador									468 (169-774)		31 (11-52)		531 (192-879)		36 (13-59)		1413 (510-2336)		95 (34-157)	
2005	Ecuador									541 (308-971)		36 (21-65)		614 (349-1102)		41 (23-74)		1634 (929-2931)		109 (62-196)	
2006	Ecuador									613 (328-1035)		41 (22-69)		696 (372-1175)		47 (25-79)		1851 (990-3125)		124 (66-209)	
2007	Ecuador									668 (339-1069)		44 (23-71)		759 (385-1214)		50 (26-81)		2017 (1023-3228)		134 (68-215)	
2008	Ecuador									718 (353-1113)		47 (23-73)		815 (401-1264)		54 (26-83)		2168 (1065-3360)		143 (70-221)	
2009	Ecuador									696 (342-1079)		45 (22-70)		791 (388-1225)		51 (25-80)		2102 (1032-3257)		137 (67-212)	
2010	Ecuador									654 (321-1013)		42 (21-65)		742 (365-1151)		48 (24-74)		1974 (969-3059)		127 (63-198)	
2011	Ecuador									540 (265-837)		34 (17-53)		613 (301-950)		39 (19-61)		1630 (801-2527)		104 (51-161)	

Spn = *Streptococcus pneumoniae*; npnm = non-pneumonia, non-meningitis; HIV = cases only in HIV-infected children; Uncertainty range provided in parentheses.

Year	Country	Spn pneumonia cases (UR)	Spn pneumonia cases, HIV (UR)	Spn pneumonia incidence rate (UR)	Spn pneumonia incidence rate, HIV (UR)	Spn severe pneumonia cases (UR)	Spn severe pneumonia cases, HIV (UR)	Spn severe pneumonia incidence rate (UR)	Spn severe pneumonia incidence rate, HIV (UR)	Spn meningitis cases (UR)	Spn meningitis cases, HIV (UR)	Spn meningitis incidence rate (UR)	Spn meningitis incidence rate, HIV (UR)	Spn NPNM cases (UR)	Spn NPNM cases, HIV (UR)	Spn NPNM incidence rate (UR)	Spn NPNM incidence rate, HIV (UR)	Spn NPNM non-severe cases (UR)	Spn NPNM non-severe cases, HIV (UR)	SPN NPNM non-severe incidence rate (UR)	SPN NPNM non-severe incidence rate, HIV (UR)
2012	Ecuador									404 (198-626)		26 (13-40)		459 (225-711)		29 (14-45)		1220 (599-1891)		77 (38-120)	
2013	Ecuador									359 (176-556)		23 (11-35)		408 (200-632)		26 (13-40)		1084 (532-1680)		69 (34-106)	
2014	Ecuador									258 (126-399)		16 (8-25)		293 (144-453)		19 (9-29)		778 (382-1205)		49 (24-76)	
2015	Ecuador	4822 (4161-5732)	5 (4-5)	305 (263-362)	0 (0-0)	1881 (1410-2146)	2 (1-2)	119 (89-136)	0 (0-0)	211 (104-327)	0 (0-0)	13 (7-21)	0 (0-0)	239 (118-371)	0 (0-0)	15 (7-23)	0 (0-0)	637 (313-987)	1 (0-1)	40 (20-62)	0 (0-0)
2000	Egypt	88759 (76584-105510)		1130 (975-1344)		34398 (25771-39234)		438 (328-500)		459 (191-925)		6 (2-12)		521 (217-1050)		7 (3-13)		1385 (577-2792)		18 (7-36)	
2001	Egypt									428 (182-859)		5 (2-11)		486 (206-975)		6 (3-12)		1292 (548-2593)		16 (7-33)	
2002	Egypt									395 (171-790)		5 (2-10)		449 (194-897)		6 (2-11)		1193 (517-2386)		15 (6-29)	
2003	Egypt									363 (161-724)		4 (2-9)		412 (182-822)		5 (2-10)		1096 (485-2185)		13 (6-26)	
2004	Egypt									337 (142-679)		4 (2-8)		383 (161-772)		5 (2-9)		1018 (427-2052)		12 (5-24)	
2005	Egypt									313 (125-637)		4 (1-7)		356 (142-724)		4 (2-8)		946 (377-1924)		11 (4-22)	
2006	Egypt									299 (125-602)		3 (1-7)		340 (142-683)		4 (2-8)		903 (377-1816)		10 (4-21)	
2007	Egypt									293 (128-581)		3 (1-6)		333 (146-660)		4 (2-7)		884 (387-1755)		10 (4-20)	
2008	Egypt									289 (133-566)		3 (1-6)		329 (151-643)		4 (2-7)		874 (402-1708)		10 (4-19)	
2009	Egypt									291 (132-568)		3 (1-6)		330 (149-646)		4 (2-7)		879 (397-1716)		10 (4-19)	
2010	Egypt									296 (102-695)		3 (1-7)		337 (115-789)		4 (1-8)		895 (307-2098)		9 (3-22)	
2011	Egypt									299 (101-704)		3 (1-7)		340 (114-800)		3 (1-8)		904 (304-2126)		9 (3-22)	
2012	Egypt									302 (100-714)		3 (1-7)		343 (114-811)		3 (1-8)		912 (302-2156)		9 (3-21)	
2013	Egypt									303 (99-719)		3 (1-7)		344 (112-816)		3 (1-7)		913 (298-2170)		8 (3-20)	
2014	Egypt									449 (144-1071)		4 (1-9)		510 (163-1216)		4 (1-11)		1355 (435-3233)		12 (4-28)	
2015	Egypt	102056 (88057-121316)	67 (58-79)	857 (739-1018)	1 (0-1)	39826 (29837-45425)	26 (19-30)	334 (250-381)	0 (0-0)	454 (143-1087)	0 (0-1)	4 (1-9)	0 (0-0)	515 (163-1234)	0 (0-1)	4 (1-10)	0 (0-0)	1370 (432-3281)	1 (0-2)	12 (4-28)	0 (0-0)
2000	El Salvador	15424 (13308-18335)		2191 (1890-2604)		6047 (4530-6897)		859 (644-980)		38 (11-99)		5 (2-14)		44 (13-112)		6 (2-16)		116 (34-299)		16 (5-42)	
2001	El Salvador									35 (10-90)		5 (2-13)		40 (12-102)		6 (2-15)		106 (31-272)		15 (5-40)	
2002	El Salvador									64 (14-200)		10 (2-30)		73 (15-227)		11 (2-34)		195 (41-604)		29 (6-91)	
2003	El Salvador									65 (14-199)		10 (2-31)		74 (16-227)		11 (3-35)		196 (43-602)		30 (7-93)	
2004	El Salvador									61 (14-185)		10 (2-29)		69 (16-210)		11 (3-33)		183 (43-558)		29 (7-89)	
2005	El Salvador									57 (13-173)		9 (2-28)		65 (15-196)		11 (2-32)		172 (40-522)		28 (7-85)	
2006	El Salvador									53 (12-160)		9 (2-27)		60 (14-182)		10 (2-31)		160 (37-484)		27 (6-82)	
2007	El Salvador									51 (12-155)		9 (2-27)		58 (14-176)		10 (2-30)		155 (36-467)		27 (6-81)	
2008	El Salvador									47 (11-142)		8 (2-25)		54 (12-161)		9 (2-28)		143 (33-429)		25 (6-75)	
2009	El Salvador									44 (11-132)		8 (2-24)		50 (12-150)		9 (2-27)		134 (32-398)		24 (6-71)	
2010	El Salvador									37 (9-108)		7 (2-20)		42 (11-123)		8 (2-22)		111 (28-326)		20 (5-59)	
2011	El Salvador									28 (7-80)		5 (1-15)		32 (8-91)		6 (2-17)		84 (22-241)		15 (4-44)	
2012	El Salvador									22 (6-61)		4 (1-11)		25 (7-70)		5 (1-13)		66 (18-185)		12 (3-35)	
2013	El Salvador									20 (6-55)		4 (1-10)		22 (7-62)		4 (1-12)		59 (17-165)		11 (3-31)	
2014	El Salvador									13 (4-36)		3 (1-7)		15 (5-41)		3 (1-8)		40 (12-109)		8 (2-21)	
2015	El Salvador	1723 (1487-2049)	15 (13-18)	337 (291-401)	3 (3-4)	672 (504-767)	6 (4-7)	132 (99-150)	1 (1-1)	11 (3-29)	0 (0-0)	2 (1-6)	0 (0-0)	12 (4-33)	0 (0-0)	2 (1-6)	0 (0-0)	33 (10-88)	0 (0-1)	6 (2-17)	0 (0-0)
2000	Equatorial Guinea	1840 (1588-2187)		2120 (1829-2519)		724 (542-825)		833 (624-951)		21 (12-39)		24 (14-44)		8 (5-15)		10 (6-18)		63 (36-117)		73 (41-134)	
2001	Equatorial Guinea									20 (8-41)		22 (9-46)		8 (3-16)		9 (4-19)		59 (24-124)		66 (27-139)	
2002	Equatorial Guinea									18 (7-38)		20 (8-41)		7 (3-15)		8 (3-17)		54 (22-113)		59 (24-125)	
2003	Equatorial Guinea									16 (7-34)		17 (7-36)		6 (3-13)		7 (3-15)		48 (20-102)		52 (21-110)	
2004	Equatorial Guinea									15 (6-31)		15 (6-32)		6 (2-12)		6 (3-13)		44 (18-92)		46 (19-97)	
2005	Equatorial Guinea									14 (6-29)		14 (6-30)		6 (2-12)		6 (2-12)		42 (17-88)		43 (18-91)	
2006	Equatorial Guinea									14 (6-30)		14 (6-30)		6 (2-12)		6 (2-12)		43 (18-90)		43 (18-91)	
2007	Equatorial Guinea									15 (6-32)		15 (6-31)		6 (3-13)		6 (2-12)		46 (19-96)		45 (19-94)	
2008	Equatorial Guinea									16 (7-34)		15 (6-32)		6 (3-13)		6 (3-13)		49 (20-101)		46 (19-96)	
2009	Equatorial Guinea									17 (7-35)		15 (6-32)		7 (3-14)		6 (3-13)		50 (21-104)		47 (20-96)	
2010	Equatorial Guinea									17 (7-35)		15 (7-32)		7 (3-14)		6 (3-13)		52 (22-107)		47 (20-96)	
2011	Equatorial Guinea									17 (7-35)		15 (6-31)		7 (3-14)		6 (3-12)		52 (22-107)		45 (19-93)	
2012	Equatorial Guinea									17 (7-35)		14 (6-29)		7 (3-14)		6 (2-12)		51 (22-105)		43 (19-89)	
2013	Equatorial Guinea									18 (8-36)		15 (6-30)		7 (3-14)		6 (3-12)		53 (23-108)		44 (19-90)	
2014	Equatorial Guinea									18 (8-36)		14 (6-30)		7 (3-15)		6 (3-12)		54 (23-110)		44 (19-89)	
2015	Equatorial Guinea	2161 (1864-2569)	876 (756-1041)	1714 (1479-2037)	695 (599-826)	847 (634-966)	343 (257-392)	672 (503-766)	272 (204-311)	17 (8-36)	7 (3-14)	14 (6-28)	6 (2-11)	7 (3-14)	3 (1-6)	6 (2-11)	2 (1-5)	53 (23-107)	21 (9-44)	42 (18-85)	17 (7-35)
2000	Eritrea	18997 (16391-22583)		3452 (2978-4103)		7458 (5587-8507)		1355 (1015-1546)		191 (75-411)		35 (14-75)		77 (30-165)		14 (5-30)		577 (226-1241)		105 (41-226)	
2001	Eritrea									192 (75-412)		34 (13-73)		77 (30-165)		14 (5-29)		579 (227-1244)		102 (40-220)	
2002	Eritrea									194 (76-417)		33 (13-71)		78 (31-167)		13 (5-28)		586 (230-1261)		100 (39-214)	
2003	Eritrea									198 (78-427)		32 (13-69)		79 (31-171)		13 (5-28)		598 (236-1288)		97 (38-210)	
2004	Eritrea									202 (67-449)		32 (10-70)		229 (76-510)		36 (12-80)		610 (202-1357)		95 (31-212)	
2005	Eritrea									205 (68-456)		31 (10-69)		232 (77-517)		35 (12-78)		618 (205-1376)		93 (31-207)	
2006	Eritrea									207 (69-462)		30 (10-66)		235 (78-524)		34 (11-75)		625 (208-1394)		90 (30-200)	
2007	Eritrea									207 (69-462)		29 (10-64)		235 (78-524)		32 (11-72)		625 (208-1394)		86 (29-193)	
2008	Eritrea									205 (68-456)		28 (9-62)		232 (77-518)		31 (10-70)		618 (206-1378)		83 (28-186)	
2009	Eritrea									200 (67-446)		27 (9-59)		227 (76-507)		30 (10-67)		604 (201-1347)		80 (27-178)	
2010	Eritrea									195 (65-434)		25 (8-57)		221 (74-493)		29 (10-64)		589 (196-1312)		77 (26-171)	
2011	Eritrea									188 (63-420)		24 (8-54)		214 (71-477)		27 (9-61)		569 (190-1267)		73 (24-163)	
2012	Eritrea									181 (60-402)		23 (8-51)		205 (69-457)		26 (9-58)		546 (182-1215)		69 (23-154)	
2013	Eritrea									173 (58-385)		22 (7-49)		197 (66-438)		25 (8-55)		523 (175-1163)		66 (22-147)	
2014	Eritrea									168 (56-373)		21 (7-47)		190 (64-423)		24 (8-53)		506 (169-1126)		64 (21-141)	
2015	Eritrea	14161 (12218-16834)	474 (409-564)	1768 (1525-2101)	59 (51-70)	5541 (4151-6319)	186 (139-212)	692 (518-789)	23 (17-26)	161 (54-359)	5 (2-12)	20 (7-45)	1 (0-2)	183 (61-408)	6 (2-14)	23 (8-51)	1 (0-2)	488 (163-1085)	16 (5-36)	61 (20-135)	2 (1-5)
2000	Estonia	277 (239-330)		460 (397-547)		180 (135-205)		298 (223-340)		3 (1-5)		4 (2-8)		3 (1-5)		5 (2-9)		8 (3-14)		13 (6-23)	
2001	Estonia									4 (2-8)		7 (3-13)		5 (2-9)		8 (4-15)		13 (6-24)		22 (10-41)	
2002	Estonia									5 (2-9)		8 (4-14)		5 (2-10)		9 (4-16)		15 (7-26)		24 (11-43)	
2003	Estonia									2 (1-4)		4 (2-7)		3 (1-5)		4 (2-8)		7 (3-13)		12 (5-22)	
2004	Estonia									3 (1-5)		4 (2-8)		3 (1-5)		5 (2-9)		8 (4-14)		13 (6-23)	
2005	Estonia									5 (2-10)		8 (4-15)		6 (3-11)		9 (4-17)		16 (7-29)		25 (11-45)	
2006	Estonia									3 (1-5)		4 (2-7)		3 (1-6)		5 (2-8)		8 (4-15)		12 (6-22)	
2007	Estonia									3 (1-6)		4 (2-8)		4 (2-7)		5 (2-9)		10 (4-18)		14 (6-25)	
2008	Estonia									9 (4-17)		13 (6-23)		11 (5-19)		14 (7-26)		28 (13-51)		38 (17-69)	
2009	Estonia									4 (2-8)		6 (3-11)		5 (2-9)		7 (3-12)		13 (6-24)		17 (8-32)	
2010	Estonia									6 (3-11)		8 (4-15)		7 (3-13)		9 (4-17)		19 (8-34)		24 (11-44)	
2011	Estonia									5 (2-10)		7 (3-13)		6 (3-11)		8 (4-14)		16 (7-29)		21 (10-38)	
2012	Estonia									0 (0-0)		0 (0-0)		0 (0-0)		0 (0-0)		0 (0-0)		0 (0-0)	
2013	Estonia									3 (1-5)		4 (2-7)		3 (2-6)		5 (2-8)		9 (4-16)		12 (6-22)	
2014	Estonia									3 (1-5)		3 (2-6)		3 (1-5)		4 (2-7)		8 (3-14)		11 (5-19)	
2015	Estonia	326 (281-387)	2 (2-2)	460 (397-547)	3 (2-3)	211 (158-241)	1 (1-1)	298 (223-340)	2 (1-2)	2 (1-4)	0 (0-0)	3 (1-6)	0 (0-0)	3 (1-5)	0 (0-0)	4 (2-7)	0 (0-0)	7 (3-13)	0 (0-0)	10 (4-18)	0 (0-0)
2000	Ethiopia	328121 (283110-390043)		2690 (2321-3198)		127208 (95304-145093)		1043 (781-1190)		6642 (2137-15534)		54 (18-127)		2664 (857-6231)		22 (7-51)		20057 (6452-46907)		164 (53-385)	
2001	Ethiopia									6485 (2095-15149)		52 (17-120)		2601 (840-6076)		21 (7-48)		19584 (6326-45745)		156 (50-364)	
2002	Ethiopia									6195 (2009-14453)		48 (16-113)		2485 (806-5797)		19 (6-45)		18707 (6068-43642)		146 (47-340)	
2003	Ethiopia									5821 (1896-13560)		45 (15-104)		2335 (760-5439)		18 (6-42)		17576 (5724-40945)		135 (44-315)	
2004	Ethiopia									5388 (1761-12529)		41 (13-96)		2161 (706-5026)		16 (5-38)		16268 (5318-37834)		124 (41-288)	
2005	Ethiopia									4947 (1623-11482)		37 (12-87)		1984 (651-4606)		15 (5-35)		14937 (4900-34672)		113 (37-262)	
2006	Ethiopia									4701 (1556-10877)		35 (12-82)		1885 (624-4363)		14 (5-33)		14194 (4699-32844)		106 (35-246)	
2007	Ethiopia									4251 (1420-9804)		32 (11-73)		1705 (570-3932)		13 (4-29)		12836 (4288-29603)		96 (32-221)	
2008	Ethiopia									3726 (1256-8565)		28 (9-63)		1494 (504-3435)		11 (4-25)		11251 (3794-25863)		83 (28-192)	
2009	Ethiopia									3554 (1210-8145)		26 (9-60)		1425 (485-3267)		10 (4-24)		10732 (3654-24596)		79 (27-181)	
2010	Ethiopia									3302 (1136-7547)		24 (8-55)		1324 (456-3027)		10 (3-22)		9969 (3429-22788)		73 (25-167)	
2011	Ethiopia									2931 (833-6885)		21 (6-50)		3329 (946-7820)		24 (7-57)		8850 (2514-20791)		64 (18-151)	
2012	Ethiopia									2435 (700-5705)		18 (5-41)		2765 (795-6480)		20 (6-47)		7352 (2114-17227)		53 (15-124)	
2013	Ethiopia									1962 (571-4584)		14 (4-33)		2228 (648-5207)		16 (5-37)		5924 (1724-13843)		42 (12-99)	
2014	Ethiopia									1675 (494-3904)		12 (3-27)		1903 (561-4434)		13 (4-31)		5059 (1490-11789)		36 (10-83)	
2015	Ethiopia	130620 (112701-155270)	5091 (4392-6051)	910 (785-1081)	35 (31-42)	51106 (38288-58291)	1992 (1492-2272)	356 (267-406)	14 (10-16)	1090 (325-2533)	42 (13-99)	8 (2-18)	0 (0-1)	1238 (369-2877)	48 (14-112)	9 (3-20)	0 (0-1)	3292 (982-7650)	128 (38-298)	23 (7-53)	1 (0-2)
2000	Fiji	1925 (1661-2288)		1992 (1719-2368)		753 (564-859)		780 (584-889)		37 (17-65)		38 (17-68)		42 (19-74)		43 (19-77)		112 (50-197)		115 (52-204)	
2001	Fiji									35 (16-63)		38 (17-67)		40 (18-71)		43 (19-76)		107 (48-189)		115 (51-203)	
2002	Fiji									36 (16-64)		40 (18-70)		41 (18-72)		45 (20-79)		109 (49-192)		119 (53-211)	
2003	Fiji									36 (16-63)		40 (18-70)		40 (18-71)		45 (20-80)		107 (48-190)		120 (54-212)	
2004	Fiji									36 (16-64)		41 (18-72)		41 (18-73)		46 (21-82)		109 (49-193)		123 (55-218)	
2005	Fiji									37 (17-66)		42 (19-75)		42 (19-75)		48 (22-85)		112 (50-199)		128 (57-226)	
2006	Fiji									40 (18-70)		45 (20-80)		45 (20-80)		51 (23-91)		120 (54-212)		136 (61-241)	
2007	Fiji									42 (19-75)		48 (21-84)		48 (21-85)		54 (24-96)		127 (57-225)		144 (64-255)	
2008	Fiji									43 (19-77)		49 (22-87)		49 (22-87)		56 (25-99)		131 (58-232)		148 (66-263)	
2009	Fiji									44 (19-77)		49 (22-87)		49 (22-87)		56 (25-99)		131 (59-233)		149 (67-264)	
2010	Fiji									43 (19-75)		48 (22-86)		48 (22-86)		55 (25-97)		129 (58-228)		146 (65-258)	
2011	Fiji									41 (18-73)		46 (21-81)		47 (21-82)		52 (23-92)		124 (55-219)		139 (62-245)	
2012	Fiji									39 (18-69)		44 (20-78)		45 (20-79)		50 (22-88)		119 (53-210)		133 (59-235)	
2013	Fiji									25 (11-45)		29 (13-51)		29 (13-51)		33 (15-58)		77 (34-136)		87 (39-154)	
2014	Fiji									19 (8-33)		22 (10-38)		21 (10-38)		24 (11-43)		57 (25-101)		65 (29-115)	
2015	Fiji	472 (407-561)	1 (1-2)	545 (470-648)	2 (1-2)	184 (138-210)	1 (0-1)	213 (159-243)	1 (0-1)	16 (7-29)	0 (0-0)	19 (8-33)	0 (0-0)	18 (8-33)	0 (0-0)	21 (10-38)	0 (0-0)	49 (22-87)	0 (0-0)	57 (25-100)	0 (0-0)
2000	Finland	333 (287-396)		115 (99-137)		216 (162-246)		75 (56-85)		4 (2-7)		1 (1-2)		4 (2-7)		1 (1-3)		11 (5-20)		4 (2-7)	
2001	Finland									4 (2-7)		1 (1-2)		4 (2-8)		1 (1-3)		11 (5-20)		4 (2-7)	
2002	Finland									0 (0-0)		0 (0-0)		0 (0-0)		0 (0-0)		0 (0-0)		0 (0-0)	
2003	Finland									15 (7-27)		5 (2-10)		17 (8-31)		6 (3-11)		45 (20-81)		16 (7-30)	
2004	Finland									13 (6-24)		5 (2-9)		15 (7-28)		5 (2-10)		41 (18-74)		15 (7-27)	
2005	Finland									7 (3-14)		3 (1-5)		9 (4-15)		3 (1-6)		23 (10-41)		8 (4-15)	
2006	Finland									4 (2-8)		1 (1-3)		5 (2-9)		2 (1-3)		13 (6-23)		4 (2-8)	
2007	Finland									7 (3-12)		2 (1-4)		8 (3-14)		3 (1-5)		20 (9-37)		7 (3-13)	
2008	Finland									7 (3-14)		3 (1-5)		9 (4-15)		3 (1-5)		23 (10-41)		8 (4-14)	
2009	Finland									0 (0-0)		0 (0-0)		0 (0-0)		0 (0-0)		0 (0-0)		0 (0-0)	
2010	Finland									7 (3-13)		2 (1-4)		8 (4-15)		3 (1-5)		21 (10-39)		7 (3-13)	
2011	Finland									4 (2-7)		1 (1-2)		4 (2-8)		1 (1-3)		12 (5-21)		4 (2-7)	
2012	Finland									0 (0-0)		0 (0-0)		0 (0-0)		0 (0-0)		0 (0-0)		0 (0-0)	
2013	Finland									4 (2-6)		1 (1-2)		4 (2-7)		1 (1-2)		11 (5-19)		4 (2-6)	
2014	Finland									2 (1-3)		1 (0-1)		2 (1-3)		1 (0-1)		5 (2-9)		2 (1-3)	
2015	Finland	166 (143-197)	0 (0-0)	55 (48-66)	0 (0-0)	108 (81-123)	0 (0-0)	36 (27-41)	0 (0-0)	1 (1-2)	0 (0-0)	0 (0-1)	0 (0-0)	2 (1-3)	0 (0-0)	1 (0-1)	0 (0-0)	4 (2-7)	0 (0-0)	1 (1-2)	0 (0-0)
2000	France	4154 (3585-4938)		115 (99-137)		2693 (2018-3072)		75 (56-85)		225 (102-409)		6 (3-11)		256 (116-464)		7 (3-13)		680 (308-1234)		19 (9-34)	
2001	France									206 (93-374)		6 (3-10)		234 (106-425)		6 (3-12)		622 (282-1129)		17 (8-31)	
2002	France									201 (91-364)		5 (2-10)		228 (103-413)		6 (3-11)		605 (275-1099)		16 (7-30)	
2003	France									180 (82-327)		5 (2-9)		205 (93-372)		5 (2-10)		545 (247-988)		15 (7-27)	
2004	France									169 (77-307)		5 (2-8)		192 (87-349)		5 (2-9)		511 (232-927)		14 (6-25)	
2005	France									120 (54-218)		3 (1-6)		136 (62-247)		4 (2-7)		363 (164-658)		10 (4-17)	
2006	France									157 (71-284)		4 (2-7)		178 (81-323)		5 (2-8)		473 (215-858)		12 (6-23)	
2007	France									99 (45-179)		3 (1-5)		112 (51-204)		3 (1-5)		299 (135-542)		8 (4-14)	
2008	France									122 (55-221)		3 (1-6)		138 (63-251)		4 (2-7)		367 (167-666)		10 (4-17)	
2009	France									73 (33-133)		2 (1-3)		83 (38-151)		2 (1-4)		221 (100-401)		6 (3-10)	
2010	France									107 (49-195)		3 (1-5)		122 (55-221)		3 (1-6)		324 (147-588)		8 (4-15)	
2011	France									54 (24-98)		1 (1-3)		61 (28-111)		2 (1-3)		163 (74-295)		4 (2-8)	
2012	France									40 (18-72)		1 (0-2)		45 (21-82)		1 (1-2)		120 (55-219)		3 (1-6)	
2013	France									37 (17-66)		1 (0-2)		42 (19-75)		1 (0-2)		111 (50-201)		3 (1-5)	
2014	France									35 (16-63)		1 (0-2)		40 (18-72)		1 (0-2)		105 (48-191)		3 (1-5)	
2015	France	728 (628-866)	0 (0-0)	19 (16-22)	0 (0-0)	472 (354-539)	0 (0-0)	12 (9-14)	0 (0-0)	33 (15-60)	0 (0-0)	1 (0-2)	0 (0-0)	38 (17-68)	0 (0-0)	1 (0-2)	0 (0-0)	100 (45-182)	0 (0-0)	3 (1-5)	0 (0-0)
2000	Gabon	4458 (3846-5299)		2454 (2117-2917)		1756 (1315-2003)		967 (724-1103)		15 (6-33)		8 (3-18)		6 (2-13)		3 (1-7)		47 (19-98)		26 (10-54)	
2001	Gabon									16 (7-34)		9 (4-19)		7 (3-14)		4 (1-7)		49 (20-104)		27 (11-56)	
2002	Gabon									17 (7-36)		9 (4-19)		7 (3-14)		4 (2-8)		52 (22-109)		28 (12-58)	
2003	Gabon									18 (8-38)		10 (4-20)		7 (3-15)		4 (2-8)		55 (23-114)		29 (12-61)	
2004	Gabon									19 (8-40)		10 (4-21)		8 (3-16)		4 (2-8)		58 (25-120)		31 (13-63)	
2005	Gabon									20 (9-41)		11 (5-22)		8 (4-17)		4 (2-9)		61 (27-125)		32 (14-65)	
2006	Gabon									21 (8-44)		11 (4-23)		24 (9-50)		12 (5-26)		64 (24-134)		33 (12-69)	
2007	Gabon									22 (8-46)		11 (4-23)		25 (10-52)		13 (5-26)		66 (26-139)		33 (13-70)	
2008	Gabon									22 (9-47)		11 (4-23)		26 (10-53)		13 (5-26)		68 (27-141)		33 (13-69)	
2009	Gabon									23 (9-47)		11 (4-23)		26 (11-54)		13 (5-26)		69 (28-143)		33 (13-69)	
2010	Gabon									23 (9-47)		11 (4-22)		26 (11-54)		12 (5-25)		70 (29-143)		33 (14-67)	
2011	Gabon									24 (10-48)		11 (5-22)		27 (11-54)		12 (5-25)		71 (30-144)		33 (14-66)	
2012	Gabon									24 (10-47)		11 (5-21)		27 (11-54)		12 (5-24)		71 (30-143)		32 (14-64)	
2013	Gabon									23 (10-47)		10 (4-21)		27 (12-53)		12 (5-23)		71 (31-141)		31 (14-62)	
2014	Gabon									23 (10-45)		10 (4-20)		26 (12-52)		11 (5-22)		70 (31-137)		30 (13-59)	
2015	Gabon	4268 (3683-5074)	163 (141-194)	1816 (1566-2158)	69 (60-82)	1672 (1253-1907)	64 (48-73)	711 (533-811)	27 (20-31)	23 (10-44)	1 (0-2)	10 (4-19)	0 (0-1)	26 (12-50)	1 (0-2)	11 (5-21)	0 (0-1)	69 (31-134)	3 (1-5)	29 (13-57)	1 (1-2)
2000	Gambia	6497 (5606-7724)		2861 (2469-3401)		2556 (1915-2915)		1125 (843-1284)		149 (77-274)		66 (34-121)		60 (31-110)		26 (14-48)		451 (232-828)		198 (102-365)	
2001	Gambia									144 (73-266)		61 (31-113)		58 (29-107)		25 (12-45)		435 (221-804)		185 (94-342)	
2002	Gambia									145 (73-271)		60 (30-111)		58 (29-109)		24 (12-45)		439 (220-818)		181 (91-336)	
2003	Gambia									142 (70-267)		57 (28-106)		57 (28-107)		23 (11-43)		430 (213-805)		172 (85-321)	
2004	Gambia									138 (68-261)		54 (26-101)		56 (27-105)		22 (11-41)		418 (205-789)		162 (79-306)	

Spn = *Streptococcus pneumoniae*; npnm = non-pneumonia, non-meningitis; HIV = cases only in HIV-infected children; Uncertainty range provided in parentheses.

Year	Country	Spn pneumonia cases (UR)	Spn pneumonia cases, HIV (UR)	Spn pneumonia incidence rate (UR)	Spn pneumonia incidence rate, HIV (UR)	Spn severe pneumonia cases (UR)	Spn severe pneumonia cases, HIV (UR)	Spn severe pneumonia incidence rate (UR)	Spn severe pneumonia incidence rate, HIV (UR)	Spn meningitis cases (UR)	Spn meningitis cases, HIV (UR)	Spn meningitis incidence rate (UR)	Spn meningitis incidence rate, HIV (UR)	Spn NPNM cases (UR)	Spn NPNM cases, HIV (UR)	Spn NPNM incidence rate (UR)	Spn NPNM incidence rate, HIV (UR)	Spn NPNM non-severe cases (UR)	Spn NPNM non-severe cases, HIV (UR)	SPN NPNM non-severe incidence rate (UR)	SPN NPNM non-severe incidence rate, HIV (UR)
2005	Gambia									136 (66-258)		51 (25-97)		55 (26-104)		20 (10-39)		411 (198-780)		154 (74-293)	
2006	Gambia									134 (65-255)		49 (24-93)		54 (26-102)		20 (9-37)		405 (196-770)		147 (71-280)	
2007	Gambia									132 (64-251)		46 (22-88)		53 (26-101)		19 (9-35)		398 (192-757)		140 (68-267)	
2008	Gambia									130 (63-247)		44 (22-85)		52 (25-99)		18 (9-34)		392 (190-747)		134 (65-256)	
2009	Gambia									128 (62-244)		43 (21-81)		51 (25-98)		17 (8-33)		387 (187-738)		129 (62-245)	
2010	Gambia									109 (53-207)		35 (17-67)		44 (21-83)		14 (7-27)		328 (159-626)		106 (51-202)	
2011	Gambia									87 (42-167)		27 (13-52)		35 (17-67)		11 (5-21)		263 (127-504)		82 (40-157)	
2012	Gambia									63 (30-121)		19 (9-37)		25 (12-49)		8 (4-15)		190 (92-366)		58 (28-111)	
2013	Gambia									59 (25-117)		17 (7-34)		67 (29-133)		20 (8-39)		178 (76-354)		52 (22-104)	
2014	Gambia									44 (19-87)		13 (5-25)		50 (21-99)		14 (6-28)		133 (57-264)		38 (16-75)	
2015	Gambia	2020 (1743-2402)	230 (198-273)	561 (484-667)	64 (55-76)	791 (592-902)	90 (67-103)	220 (164-250)	25 (19-28)	39 (17-77)	4 (2-9)	11 (5-21)	1 (1-2)	44 (19-88)	5 (2-10)	12 (5-24)	1 (1-3)	117 (51-233)	13 (6-27)	33 (14-65)	4 (2-7)
2000	Georgia	625 (539-743)		230 (198-273)		405 (303-462)		149 (112-170)		47 (24-76)		17 (9-28)		54 (27-87)		20 (10-32)		143 (73-231)		53 (27-85)	
2001	Georgia									39 (21-64)		15 (8-25)		44 (24-73)		17 (9-29)		117 (63-193)		46 (25-76)	
2002	Georgia									33 (17-56)		13 (7-23)		37 (20-64)		15 (8-26)		98 (52-169)		41 (22-70)	
2003	Georgia									28 (12-60)		12 (5-26)		32 (14-69)		13 (6-29)		84 (36-182)		36 (15-77)	
2004	Georgia									23 (9-52)		10 (4-23)		26 (10-60)		11 (4-26)		69 (26-158)		30 (11-68)	
2005	Georgia									19 (6-46)		8 (3-20)		22 (7-52)		9 (3-22)		58 (19-137)		25 (8-60)	
2006	Georgia									16 (5-39)		7 (2-16)		18 (5-44)		7 (2-18)		48 (15-118)		20 (6-49)	
2007	Georgia									13 (4-33)		5 (1-13)		15 (4-37)		6 (2-15)		39 (11-98)		16 (4-39)	
2008	Georgia									10 (3-27)		4 (1-11)		12 (3-31)		5 (1-12)		31 (8-82)		12 (3-32)	
2009	Georgia									8 (2-22)		3 (1-9)		10 (2-25)		4 (1-10)		25 (6-68)		10 (2-26)	
2010	Georgia									12 (3-33)		5 (1-13)		14 (3-38)		5 (1-14)		37 (8-100)		14 (3-38)	
2011	Georgia									12 (3-34)		5 (1-12)		14 (3-38)		5 (1-14)		37 (8-101)		14 (3-38)	
2012	Georgia									10 (2-27)		4 (1-10)		11 (2-31)		4 (1-11)		30 (6-83)		11 (2-30)	
2013	Georgia									8 (1-23)		3 (1-8)		9 (2-26)		3 (1-9)		24 (4-69)		9 (2-25)	
2014	Georgia									7 (1-20)		3 (0-7)		8 (1-22)		3 (0-8)		21 (4-59)		8 (1-22)	
2015	Georgia	590 (509-701)	1 (1-2)	218 (188-259)	1 (0-1)	382 (286-436)	1 (1-1)	141 (106-161)	0 (0-0)	6 (1-17)	0 (0-0)	2 (0-6)	0 (0-0)	7 (1-19)	0 (0-0)	2 (0-7)	0 (0-0)	18 (3-51)	0 (0-0)	7 (1-19)	0 (0-0)
2000	Germany	4401 (3798-5232)		115 (99-137)		2853 (2138-3255)		75 (56-85)		213 (97-387)		6 (3-10)		242 (110-440)		6 (3-11)		644 (292-1169)		17 (8-31)	
2001	Germany									149 (68-271)		4 (2-7)		170 (77-308)		4 (2-8)		451 (205-819)		12 (5-21)	
2002	Germany									215 (97-389)		6 (3-10)		244 (111-442)		6 (3-12)		648 (294-1176)		17 (8-31)	
2003	Germany									194 (88-351)		5 (2-9)		220 (100-399)		6 (3-11)		585 (265-1061)		16 (7-29)	
2004	Germany									194 (88-352)		5 (2-10)		220 (100-400)		6 (3-11)		586 (266-1063)		16 (7-30)	
2005	Germany									142 (64-257)		4 (2-7)		161 (73-292)		5 (2-8)		428 (194-777)		12 (6-22)	
2006	Germany									161 (73-293)		5 (2-9)		183 (83-332)		5 (2-10)		487 (221-884)		14 (6-26)	
2007	Germany									86 (39-157)		3 (1-5)		98 (44-178)		3 (1-5)		261 (118-473)		8 (4-14)	
2008	Germany									72 (33-130)		2 (1-4)		81 (37-148)		2 (1-4)		217 (98-393)		7 (3-12)	
2009	Germany									43 (19-77)		1 (1-2)		48 (22-88)		1 (1-3)		129 (58-234)		4 (2-7)	
2010	Germany									23 (11-42)		1 (0-1)		27 (12-48)		1 (0-1)		71 (32-128)		2 (1-4)	
2011	Germany									15 (7-27)		0 (0-1)		17 (8-31)		1 (0-1)		46 (21-83)		1 (1-3)	
2012	Germany									16 (7-29)		0 (0-1)		18 (8-33)		1 (0-1)		49 (22-89)		1 (1-3)	
2013	Germany									14 (6-26)		0 (0-1)		16 (7-29)		0 (0-1)		43 (20-78)		1 (1-2)	
2014	Germany									13 (6-23)		0 (0-1)		14 (7-26)		0 (0-1)		38 (17-69)		1 (1-2)	
2015	Germany	628 (542-746)	0 (0-0)	19 (16-22)	0 (0-0)	407 (305-464)	0 (0-0)	12 (9-14)	0 (0-0)	13 (6-23)	0 (0-0)	0 (0-1)	0 (0-0)	14 (7-26)	0 (0-0)	0 (0-1)	0 (0-0)	39 (17-70)	0 (0-0)	1 (1-2)	0 (0-0)
2000	Ghana	61964 (53464-73657)		2203 (1901-2619)		23924 (17924-27287)		851 (637-970)		579 (248-1203)		21 (9-43)		232 (99-482)		8 (4-17)		1749 (749-3632)		62 (27-129)	
2001	Ghana									578 (240-1214)		20 (8-43)		232 (96-487)		8 (3-17)		1746 (725-3666)		61 (25-128)	
2002	Ghana									582 (235-1235)		20 (8-42)		233 (94-496)		8 (3-17)		1757 (708-3730)		60 (24-128)	
2003	Ghana									571 (224-1226)		19 (7-41)		229 (90-492)		8 (3-16)		1724 (676-3703)		58 (23-124)	
2004	Ghana									575 (220-1248)		19 (7-41)		231 (88-501)		8 (3-16)		1736 (663-3770)		57 (22-123)	
2005	Ghana									583 (216-1275)		19 (7-41)		234 (87-512)		7 (3-16)		1760 (653-3851)		56 (21-122)	
2006	Ghana									605 (218-1331)		19 (7-41)		243 (87-534)		7 (3-16)		1827 (657-4018)		56 (20-124)	
2007	Ghana									617 (239-1334)		19 (7-40)		248 (96-535)		7 (3-16)		1864 (723-4028)		56 (22-122)	
2008	Ghana									604 (254-1282)		18 (8-38)		242 (102-514)		7 (3-15)		1825 (768-3870)		54 (23-115)	
2009	Ghana									602 (245-1286)		18 (7-37)		242 (98-516)		7 (3-15)		1819 (741-3884)		53 (22-113)	
2010	Ghana									588 (194-1303)		17 (6-37)		667 (221-1480)		19 (6-42)		1774 (587-3934)		50 (17-112)	
2011	Ghana									587 (188-1312)		16 (5-36)		667 (213-1490)		18 (6-41)		1772 (566-3960)		49 (16-110)	
2012	Ghana									487 (162-1079)		13 (4-29)		553 (184-1226)		15 (5-33)		1469 (489-3259)		40 (13-88)	
2013	Ghana									358 (124-787)		9 (3-21)		407 (141-894)		11 (4-23)		1082 (376-2376)		28 (10-62)	
2014	Ghana									314 (114-682)		8 (3-17)		357 (129-775)		9 (3-20)		949 (343-2060)		24 (9-53)	
2015	Ghana	28407 (24510-33768)	1629 (1405-1936)	712 (615-847)	41 (35-49)	11106 (8321-12668)	637 (477-726)	278 (209-318)	16 (12-18)	308 (117-661)	18 (7-38)	8 (3-17)	0 (0-1)	350 (132-751)	20 (8-43)	9 (3-19)	1 (0-1)	931 (352-1996)	53 (20-114)	23 (9-50)	1 (1-3)
2000	Greece	590 (509-702)		115 (99-137)		383 (287-436)		75 (56-85)		26 (12-47)		5 (2-9)		30 (13-54)		6 (3-10)		78 (36-142)		15 (7-28)	
2001	Greece									14 (6-25)		3 (1-5)		16 (7-28)		3 (1-6)		42 (19-75)		8 (4-15)	
2002	Greece									3 (1-5)		1 (0-1)		3 (2-6)		1 (0-1)		9 (4-16)		2 (1-3)	
2003	Greece									15 (7-28)		3 (1-5)		17 (8-32)		3 (2-6)		46 (21-84)		9 (4-16)	
2004	Greece									13 (6-23)		2 (1-5)		15 (7-26)		3 (1-5)		39 (18-70)		7 (3-14)	
2005	Greece									6 (3-11)		1 (1-2)		7 (3-13)		1 (1-2)		19 (8-34)		4 (2-7)	
2006	Greece									10 (4-17)		2 (1-3)		11 (5-20)		2 (1-4)		29 (13-53)		6 (3-10)	
2007	Greece									6 (3-10)		1 (0-2)		6 (3-12)		1 (1-2)		17 (8-31)		3 (1-6)	
2008	Greece									10 (4-17)		2 (1-3)		11 (5-20)		2 (1-4)		29 (13-52)		5 (2-10)	
2009	Greece									13 (6-23)		2 (1-4)		14 (7-26)		3 (1-5)		38 (17-70)		7 (3-13)	
2010	Greece									2 (1-4)		0 (0-1)		3 (1-5)		0 (0-1)		7 (3-13)		1 (1-2)	
2011	Greece									5 (2-9)		1 (0-2)		5 (2-10)		1 (0-2)		15 (7-26)		3 (1-5)	
2012	Greece									3 (1-5)		0 (0-1)		3 (1-6)		1 (0-1)		8 (4-15)		1 (1-3)	
2013	Greece									3 (1-6)		1 (0-1)		4 (2-7)		1 (0-1)		10 (4-17)		2 (1-3)	
2014	Greece									1 (1-3)		0 (0-1)		2 (1-3)		0 (0-1)		4 (2-8)		1 (0-2)	
2015	Greece	231 (200-275)	0 (0-0)	44 (38-52)	0 (0-0)	150 (112-171)	0 (0-0)	29 (21-33)	0 (0-0)	1 (0-2)	0 (0-0)	0 (0-0)	0 (0-0)	1 (1-2)	0 (0-0)	0 (0-0)	0 (0-0)	3 (1-6)	0 (0-0)	1 (0-1)	0 (0-0)
2000	Grenada	150 (129-178)		1457 (1257-1732)		58 (44-66)		567 (425-646)		0 (0-0)		0 (0-0)		0 (0-0)		0 (0-0)		0 (0-0)		0 (0-0)	
2001	Grenada									0 (0-0)		0 (0-0)		0 (0-0)		0 (0-0)		0 (0-0)		0 (0-0)	
2002	Grenada									0 (0-0)		0 (0-0)		0 (0-0)		0 (0-0)		0 (0-0)		0 (0-0)	
2003	Grenada									0 (0-0)		0 (0-0)		0 (0-0)		0 (0-0)		0 (0-0)		0 (0-0)	
2004	Grenada									0 (0-0)		0 (0-0)		0 (0-0)		0 (0-0)		0 (0-0)		0 (0-0)	
2005	Grenada									0 (0-0)		0 (0-0)		0 (0-0)		0 (0-0)		0 (0-0)		0 (0-0)	
2006	Grenada									0 (0-0)		0 (0-0)		0 (0-0)		0 (0-0)		0 (0-0)		0 (0-0)	
2007	Grenada									0 (0-0)		0 (0-0)		0 (0-0)		0 (0-0)		0 (0-0)		0 (0-0)	
2008	Grenada									0 (0-0)		0 (0-0)		0 (0-0)		0 (0-0)		0 (0-0)		0 (0-0)	
2009	Grenada									0 (0-0)		0 (0-0)		0 (0-0)		0 (0-0)		0 (0-0)		0 (0-0)	
2010	Grenada									0 (0-0)		0 (0-0)		0 (0-0)		0 (0-0)		0 (0-0)		0 (0-0)	
2011	Grenada									0 (0-0)		0 (0-0)		0 (0-0)		0 (0-0)		0 (0-0)		0 (0-0)	
2012	Grenada									0 (0-0)		0 (0-0)		0 (0-0)		0 (0-0)		0 (0-0)		0 (0-0)	
2013	Grenada									0 (0-0)		0 (0-0)		0 (0-0)		0 (0-0)		0 (0-0)		0 (0-0)	
2014	Grenada									0 (0-0)		0 (0-0)		0 (0-0)		0 (0-0)		0 (0-0)		0 (0-0)	
2015	Grenada	91 (78-108)	0 (0-0)	928 (800-1103)	0 (0-0)	35 (26-40)	0 (0-0)	362 (271-413)	0 (0-0)	0 (0-0)	0 (0-0)	0 (0-0)	0 (0-0)	0 (0-0)	0 (0-0)	0 (0-0)	0 (0-0)	0 (0-0)	0 (0-0)	0 (0-0)	0 (0-0)
2000	Guatemala	54166 (46736-64388)		2941 (2537-3496)		21288 (15949-24281)		1156 (866-1318)		247 (75-628)		13 (4-34)		280 (85-714)		15 (5-39)		745 (225-1897)		40 (12-103)	
2001	Guatemala									239 (73-608)		13 (4-33)		272 (83-691)		15 (4-37)		723 (220-1836)		39 (12-99)	
2002	Guatemala									232 (71-588)		12 (4-31)		264 (81-668)		14 (4-35)		701 (216-1775)		37 (11-94)	
2003	Guatemala									224 (70-566)		12 (4-30)		255 (79-643)		13 (4-34)		677 (210-1709)		36 (11-90)	
2004	Guatemala									218 (68-548)		11 (4-29)		248 (77-623)		13 (4-32)		658 (205-1655)		34 (11-86)	
2005	Guatemala									211 (66-528)		11 (3-27)		239 (75-599)		12 (4-31)		636 (200-1594)		33 (10-82)	
2006	Guatemala									202 (64-506)		10 (3-26)		230 (73-574)		12 (4-30)		611 (193-1527)		31 (10-79)	
2007	Guatemala									194 (62-483)		10 (3-25)		221 (70-549)		11 (4-28)		586 (187-1460)		30 (10-75)	
2008	Guatemala									185 (59-459)		9 (3-24)		210 (67-521)		11 (3-27)		558 (179-1386)		29 (9-71)	
2009	Guatemala									179 (58-444)		9 (3-23)		204 (66-504)		10 (3-26)		542 (175-1340)		28 (9-69)	
2010	Guatemala									175 (57-433)		9 (3-22)		199 (65-491)		10 (3-25)		530 (172-1306)		27 (9-67)	
2011	Guatemala									172 (56-424)		9 (3-22)		196 (64-481)		10 (3-24)		521 (170-1280)		26 (9-65)	
2012	Guatemala									170 (56-418)		9 (3-21)		194 (64-475)		10 (3-24)		515 (169-1262)		26 (9-64)	
2013	Guatemala									153 (50-373)		8 (3-19)		173 (57-424)		9 (3-21)		461 (152-1126)		23 (8-56)	
2014	Guatemala									126 (42-308)		6 (2-15)		144 (48-350)		7 (2-17)		382 (127-931)		19 (6-46)	
2015	Guatemala	16539 (14271-19661)	295 (255-351)	805 (695-957)	14 (12-17)	6465 (4844-7374)	115 (86-132)	315 (236-359)	6 (4-6)	96 (24-285)	2 (0-5)	5 (1-14)	0 (0-0)	109 (27-324)	2 (0-6)	5 (1-16)	0 (0-0)	291 (72-861)	5 (1-15)	14 (4-42)	0 (0-1)
2000	Guinea	35546 (30669-42254)		2349 (2027-2792)		14007 (10494-15976)		926 (694-1056)		594 (297-1176)		39 (20-78)		238 (119-472)		16 (8-31)		1794 (896-3550)		119 (59-235)	
2001	Guinea									607 (310-1192)		39 (20-77)		243 (124-478)		16 (8-31)		1833 (937-3600)		119 (61-234)	
2002	Guinea									622 (326-1214)		40 (21-78)		250 (131-487)		16 (8-31)		1879 (984-3665)		121 (63-235)	
2003	Guinea									645 (232-1431)		41 (15-91)		259 (93-574)		16 (6-36)		1948 (700-4322)		123 (44-274)	
2004	Guinea									676 (252-1485)		42 (16-93)		271 (101-596)		17 (6-37)		2042 (762-4485)		128 (48-280)	
2005	Guinea									702 (272-1526)		43 (17-94)		282 (109-612)		17 (7-38)		2120 (823-4609)		130 (50-283)	
2006	Guinea									717 (276-1559)		43 (17-94)		288 (111-625)		17 (7-38)		2165 (834-4708)		130 (50-283)	
2007	Guinea									718 (275-1563)		42 (16-92)		288 (110-627)		17 (7-37)		2169 (830-4721)		128 (49-279)	
2008	Guinea									688 (262-1501)		40 (15-87)		276 (105-602)		16 (6-35)		2079 (791-4531)		120 (46-263)	
2009	Guinea									687 (260-1502)		39 (15-85)		276 (104-602)		16 (6-34)		2075 (786-4535)		118 (45-258)	
2010	Guinea									666 (251-1458)		37 (14-81)		267 (101-585)		15 (6-32)		2010 (757-4403)		112 (42-245)	
2011	Guinea									646 (242-1417)		35 (13-77)		259 (97-568)		14 (5-31)		1949 (731-4279)		106 (40-232)	
2012	Guinea									631 (235-1389)		33 (12-74)		253 (94-557)		13 (5-30)		1907 (711-4196)		101 (38-222)	
2013	Guinea									628 (233-1384)		33 (12-72)		252 (93-555)		13 (5-29)		1895 (703-4178)		98 (36-217)	
2014	Guinea									624 (230-1378)		32 (12-70)		250 (92-553)		13 (5-28)		1883 (695-4160)		96 (35-211)	
2015	Guinea	36091 (31140-42902)	1486 (1282-1766)	1794 (1548-2133)	74 (64-88)	14083 (10551-16063)	580 (434-661)	700 (525-799)	29 (22-33)	614 (225-1359)	25 (9-56)	31 (11-68)	1 (0-3)	246 (90-545)	10 (4-22)	12 (4-27)	1 (0-1)	1854 (681-4103)	76 (28-169)	92 (34-204)	4 (1-8)
2000	Guinea-Bissau	6221 (5367-7395)		2874 (2480-3417)		2445 (1832-2789)		1130 (846-1289)		134 (80-226)		62 (37-104)		54 (32-91)		25 (15-42)		403 (242-682)		186 (112-315)	
2001	Guinea-Bissau									138 (83-234)		63 (38-107)		55 (33-94)		25 (15-43)		417 (249-708)		190 (113-322)	
2002	Guinea-Bissau									142 (84-243)		64 (38-109)		57 (34-97)		26 (15-44)		429 (255-732)		193 (115-329)	
2003	Guinea-Bissau									146 (87-252)		65 (38-111)		59 (35-101)		26 (15-45)		442 (262-760)		195 (116-336)	
2004	Guinea-Bissau									149 (89-260)		65 (39-113)		60 (36-104)		26 (15-45)		450 (267-784)		196 (117-341)	
2005	Guinea-Bissau									151 (65-300)		65 (28-129)		60 (26-120)		26 (11-52)		455 (197-906)		195 (84-388)	
2006	Guinea-Bissau									149 (64-300)		62 (27-126)		60 (26-121)		25 (11-51)		450 (194-907)		189 (82-380)	
2007	Guinea-Bissau									144 (62-293)		59 (26-121)		58 (25-118)		24 (10-48)		435 (187-886)		179 (77-365)	
2008	Guinea-Bissau									136 (58-280)		55 (24-113)		55 (23-112)		22 (9-45)		411 (175-845)		166 (71-342)	
2009	Guinea-Bissau									127 (54-262)		50 (21-104)		51 (21-105)		20 (9-42)		382 (162-792)		152 (64-315)	
2010	Guinea-Bissau									118 (49-246)		46 (19-96)		47 (20-99)		18 (8-38)		355 (149-742)		139 (58-290)	
2011	Guinea-Bissau									109 (44-230)		41 (17-88)		44 (18-92)		17 (7-35)		328 (133-696)		125 (51-265)	
2012	Guinea-Bissau									100 (39-217)		37 (15-81)		40 (16-87)		15 (6-32)		303 (118-654)		113 (44-244)	
2013	Guinea-Bissau									95 (36-207)		35 (13-76)		38 (14-83)		14 (5-30)		285 (107-624)		104 (39-228)	
2014	Guinea-Bissau									90 (33-199)		32 (12-71)		36 (13-80)		13 (5-29)		271 (98-600)		97 (35-215)	
2015	Guinea-Bissau	5033 (4342-5982)	1502 (1296-1786)	1772 (1529-2107)	529 (456-629)	1971 (1476-2248)	588 (441-671)	694 (520-792)	207 (155-236)	81 (28-182)	24 (9-54)	29 (10-64)	9 (3-19)	33 (11-73)	10 (3-22)	11 (4-26)	3 (1-8)	245 (86-549)	73 (26-164)	86 (30-193)	26 (9-58)
2000	Guyana	1736 (1498-2064)		1826 (1576-2171)		681 (510-777)		717 (537-817)		4 (2-10)		4 (2-10)		5 (2-11)		5 (2-11)		13 (5-29)		13 (5-30)	
2001	Guyana									11 (4-25)		11 (4-27)		12 (4-28)		13 (5-30)		32 (12-75)		34 (12-80)	
2002	Guyana									7 (3-18)		8 (3-19)		8 (3-20)		9 (3-22)		22 (8-53)		24 (8-58)	
2003	Guyana									8 (3-20)		9 (3-23)		9 (3-23)		11 (4-26)		25 (8-61)		28 (9-68)	
2004	Guyana									9 (3-21)		10 (3-24)		10 (3-24)		11 (4-28)		26 (8-63)		30 (10-73)	
2005	Guyana									9 (3-21)		10 (3-26)		10 (3-24)		12 (4-29)		26 (8-65)		31 (10-78)	
2006	Guyana									5 (1-12)		6 (2-15)		5 (2-14)		7 (2-17)		15 (4-37)		18 (6-46)	
2007	Guyana									6 (2-16)		9 (3-22)		7 (2-18)		10 (3-25)		19 (6-49)		26 (8-66)	
2008	Guyana									6 (2-16)		9 (3-22)		7 (2-18)		10 (3-25)		19 (6-47)		27 (8-68)	
2009	Guyana									13 (4-32)		19 (6-48)		14 (4-36)		22 (7-54)		39 (12-96)		58 (18-144)	
2010	Guyana									3 (1-8)		5 (2-13)		4 (1-9)		6 (2-15)		10 (3-25)		16 (5-39)	
2011	Guyana									7 (2-17)		12 (4-29)		8 (3-20)		14 (5-32)		22 (7-52)		36 (12-86)	
2012	Guyana									5 (2-12)		9 (3-21)		6 (2-14)		10 (4-24)		16 (6-38)		27 (10-63)	
2013	Guyana									5 (2-12)		9 (3-20)		6 (2-14)		10 (4-22)		16 (6-37)		27 (10-60)	
2014	Guyana									6 (2-12)		9 (4-19)		6 (3-14)		10 (4-21)		17 (7-37)		26 (11-57)	
2015	Guyana	363 (313-431)	14 (12-16)	550 (474-653)	21 (18-25)	142 (107-162)	5 (4-6)	216 (162-246)	8 (6-9)	4 (2-9)	0 (0-0)	7 (3-14)	0 (0-1)	5 (2-10)	0 (0-0)	7 (3-16)	0 (0-1)	13 (6-28)	0 (0-1)	20 (9-42)	1 (0-2)
2000	Haiti	29382 (25351-34927)		2452 (2116-2915)		11339 (8495-12933)		946 (709-1079)		323 (83-922)		27 (7-77)		130 (33-370)		11 (3-31)		975 (251-2785)		81 (21-232)	
2001	Haiti									313 (81-891)		26 (7-74)		125 (32-357)		10 (3-30)		944 (244-2689)		78 (20-223)	
2002	Haiti									302 (79-859)		25 (7-71)		121 (32-345)		10 (3-29)		913 (238-2594)		76 (20-215)	
2003	Haiti									292 (77-828)		24 (6-69)		117 (31-332)		10 (3-28)		883 (232-2500)		73 (19-207)	
2004	Haiti									285 (75-804)		24 (6-67)		114 (30-322)		9 (3-27)		859 (228-2426)		71 (19-201)	
2005	Haiti									280 (75-789)		23 (6-65)		112 (30-317)		9 (2-26)		847 (226-2384)		70 (19-198)	
2006	Haiti									278 (74-782)		23 (6-65)		112 (30-314)		9 (2-26)		840 (224-2361)		70 (19-196)	
2007	Haiti									276 (74-772)		23 (6-64)		111 (30-309)		9 (2-26)		833 (224-2330)		69 (18-192)	
2008	Haiti									274 (74-761)		22 (6-62)		110 (30-305)		9 (2-25)		826 (224-2298)		68 (18-189)	
2009	Haiti									271 (74-748)		22 (6-61)		109 (30-300)		9 (2-25)		818 (223-2260)		67 (18-185)	
2010	Haiti									1046 (442-2491)		85 (36-203)		419 (177-999)		34 (14-81)		3157 (1334-7523)		257 (109-613)	
2011	Haiti									265 (74-728)		21 (6-59)		106 (30-292)		9 (2-24)		800 (223-2198)		65 (18-178)	
2012	Haiti									263 (74-721)		21 (6-59)		105 (30-289)		9 (2-23)		794 (224-2179)		64 (18-177)	
2013	Haiti									257 (60-730)		21 (5-59)		292 (68-829)		24 (6-68)		775 (180-2203)		63 (15-180)	

Spn = *Streptococcus pneumoniae*; npnm = non-pneumonia, non-meningitis; HIV = cases only in HIV-infected children; Uncertainty range provided in parentheses.

Year	Country	Spn pneumonia cases (UR)	Spn pneumonia cases, HIV (UR)	Spn pneumonia incidence rate (UR)	Spn pneumonia incidence rate, HIV (UR)	Spn severe pneumonia cases (UR)	Spn severe pneumonia cases, HIV (UR)	Spn severe pneumonia incidence rate (UR)	Spn severe pneumonia incidence rate, HIV (UR)	Spn meningitis cases (UR)	Spn meningitis cases, HIV (UR)	Spn meningitis incidence rate (UR)	Spn meningitis incidence rate, HIV (UR)	Spn NPNM cases (UR)	Spn NPNM cases, HIV (UR)	Spn NPNM incidence rate (UR)	Spn NPNM incidence rate, HIV (UR)	Spn NPNM non-severe cases (UR)	Spn NPNM non-severe cases, HIV (UR)	SPN NPNM non-severe incidence rate (UR)	SPN NPNM non-severe incidence rate, HIV (UR)
2014	Haiti									252 (59-718)		21 (5-59)		286 (67-815)		23 (5-67)		761 (178-2167)		62 (15-178)	
2015	Haiti	20282 (17500-24109)	691 (596-821)	1667 (1438-1981)	57 (49-67)	7899 (5918-9009)	269 (201-307)	649 (486-740)	22 (17-25)	244 (58-696)	8 (2-24)	20 (5-57)	1 (0-2)	278 (65-790)	9 (2-27)	23 (5-65)	1 (0-2)	738 (174-2101)	25 (6-72)	61 (14-173)	2 (0-6)
2000	Honduras	14317 (12353-17019)		1531 (1321-1820)		5618 (4209-6408)		601 (450-685)		79 (21-212)		8 (2-23)		90 (24-241)		10 (3-26)		238 (64-641)		26 (7-69)	
2001	Honduras									77 (21-207)		8 (2-22)		88 (24-235)		10 (3-25)		233 (63-624)		25 (7-67)	
2002	Honduras									70 (19-187)		8 (2-20)		79 (22-213)		9 (2-23)		211 (58-565)		23 (6-61)	
2003	Honduras									65 (18-173)		7 (2-19)		73 (20-196)		8 (2-21)		195 (54-521)		21 (6-57)	
2004	Honduras									61 (17-163)		7 (2-18)		70 (19-185)		8 (2-20)		185 (52-493)		20 (6-54)	
2005	Honduras									62 (17-165)		7 (2-18)		71 (20-187)		8 (2-21)		188 (53-498)		21 (6-55)	
2006	Honduras									60 (11-191)		7 (1-21)		68 (13-217)		8 (1-24)		180 (35-577)		20 (4-65)	
2007	Honduras									58 (11-184)		7 (1-21)		66 (13-209)		7 (1-24)		175 (34-556)		20 (4-63)	
2008	Honduras									56 (11-178)		6 (1-20)		64 (13-202)		7 (1-23)		170 (34-537)		19 (4-61)	
2009	Honduras									55 (11-172)		6 (1-20)		62 (13-195)		7 (1-23)		165 (34-519)		19 (4-60)	
2010	Honduras									52 (11-163)		6 (1-19)		59 (13-185)		7 (1-22)		158 (34-492)		18 (4-57)	
2011	Honduras									35 (8-108)		4 (1-13)		39 (9-122)		5 (1-14)		105 (23-325)		12 (3-38)	
2012	Honduras									26 (6-80)		3 (1-10)		29 (6-90)		4 (1-11)		78 (17-240)		9 (2-29)	
2013	Honduras									22 (5-69)		3 (1-8)		25 (6-78)		3 (1-10)		67 (15-207)		8 (2-25)	
2014	Honduras									20 (4-62)		2 (1-8)		23 (5-70)		3 (1-9)		61 (14-187)		8 (2-23)	
2015	Honduras	3451 (2978-4102)	44 (38-52)	430 (371-511)	5 (5-7)	1349 (1011-1539)	17 (13-20)	168 (126-192)	2 (2-2)	13 (3-39)	0 (0-0)	2 (0-5)	0 (0-0)	14 (3-44)	0 (0-1)	2 (0-5)	0 (0-0)	38 (9-117)	0 (0-1)	5 (1-15)	0 (0-0)
2000	Hungary	2235 (1928-2656)		460 (397-547)		1449 (1085-1652)		298 (223-340)		31 (14-56)		6 (3-11)		35 (16-63)		7 (3-13)		92 (42-168)		19 (9-35)	
2001	Hungary									50 (23-92)		11 (5-20)		57 (26-104)		12 (6-22)		152 (69-276)		33 (15-60)	
2002	Hungary									31 (14-55)		7 (3-12)		35 (16-63)		8 (3-14)		92 (42-167)		20 (9-37)	
2003	Hungary									24 (11-43)		5 (2-9)		27 (12-49)		6 (3-11)		72 (33-131)		16 (7-29)	
2004	Hungary									47 (21-86)		10 (5-19)		54 (24-98)		12 (5-21)		143 (65-259)		31 (14-56)	
2005	Hungary									51 (23-92)		11 (5-20)		58 (26-105)		12 (6-22)		153 (69-278)		33 (15-60)	
2006	Hungary									36 (16-66)		8 (3-14)		41 (19-74)		9 (4-16)		109 (49-198)		23 (10-41)	
2007	Hungary									30 (14-55)		6 (3-11)		34 (15-62)		7 (3-13)		91 (41-165)		19 (9-34)	
2008	Hungary									29 (13-52)		6 (3-11)		33 (15-59)		7 (3-12)		87 (40-158)		18 (8-33)	
2009	Hungary									27 (12-49)		6 (3-10)		31 (14-56)		6 (3-12)		82 (37-148)		17 (8-31)	
2010	Hungary									22 (10-39)		4 (2-8)		25 (11-45)		5 (2-9)		65 (30-119)		14 (6-25)	
2011	Hungary									13 (6-24)		3 (1-5)		15 (7-27)		3 (1-6)		39 (18-71)		8 (4-15)	
2012	Hungary									6 (3-11)		1 (1-2)		7 (3-13)		1 (1-3)		19 (8-34)		4 (2-7)	
2013	Hungary									16 (7-30)		3 (2-6)		18 (8-34)		4 (2-7)		49 (22-89)		11 (5-19)	
2014	Hungary									10 (4-17)		2 (1-4)		11 (5-20)		2 (1-4)		29 (13-52)		6 (3-11)	
2015	Hungary	597 (515-709)	0 (0-0)	131 (113-156)	0 (0-0)	387 (290-441)	0 (0-0)	85 (64-97)	0 (0-0)	6 (3-10)	0 (0-0)	1 (1-2)	0 (0-0)	6 (3-12)	0 (0-0)	1 (1-3)	0 (0-0)	17 (8-31)	0 (0-0)	4 (2-7)	0 (0-0)
2000	Iceland	24 (21-29)		115 (99-137)		16 (12-18)		75 (56-85)		0 (0-0)		0 (0-0)		0 (0-0)		0 (0-0)		0 (0-0)		0 (0-0)	
2001	Iceland									0 (0-0)		0 (0-0)		0 (0-0)		0 (0-0)		0 (0-0)		0 (0-0)	
2002	Iceland									2 (1-3)		8 (4-15)		2 (1-3)		10 (4-17)		5 (2-9)		25 (11-46)	
2003	Iceland									2 (1-3)		8 (4-15)		2 (1-4)		10 (4-17)		5 (2-9)		26 (12-46)	
2004	Iceland									2 (1-3)		9 (4-16)		2 (1-4)		10 (5-18)		5 (2-10)		27 (12-49)	
2005	Iceland									1 (0-2)		5 (2-9)		1 (1-2)		6 (3-10)		3 (1-6)		15 (7-28)	
2006	Iceland									1 (1-2)		6 (3-11)		1 (1-3)		7 (3-12)		4 (2-7)		18 (8-32)	
2007	Iceland									1 (1-2)		5 (2-10)		1 (1-2)		6 (3-11)		4 (2-6)		16 (7-30)	
2008	Iceland									0 (0-0)		0 (0-0)		0 (0-0)		0 (0-0)		0 (0-0)		0 (0-0)	
2009	Iceland									0 (0-0)		0 (0-0)		0 (0-0)		0 (0-0)		0 (0-0)		0 (0-0)	
2010	Iceland									0 (0-0)		0 (0-0)		0 (0-0)		0 (0-0)		0 (0-0)		0 (0-0)	
2011	Iceland									0 (0-0)		0 (0-0)		0 (0-0)		0 (0-0)		0 (0-0)		0 (0-0)	
2012	Iceland									0 (0-0)		0 (0-0)		0 (0-0)		0 (0-0)		0 (0-0)		0 (0-0)	
2013	Iceland									0 (0-0)		0 (0-0)		0 (0-0)		0 (0-0)		0 (0-0)		0 (0-0)	
2014	Iceland									0 (0-0)		0 (0-0)		0 (0-0)		0 (0-0)		0 (0-0)		0 (0-0)	
2015	Iceland	13 (11-15)	0 (0-0)	56 (48-66)	0 (0-0)	8 (6-9)	0 (0-0)	36 (27-41)	0 (0-0)	0 (0-0)	0 (0-0)	0 (0-0)	0 (0-0)	0 (0-0)	0 (0-0)	0 (0-0)	0 (0-0)	0 (0-0)	0 (0-0)	0 (0-0)	0 (0-0)
2000	India	3391559 (2926311-4031609)		2702 (2331-3212)		1335371 (1000453-1523119)		1064 (797-1213)		48798 (23297-92634)		39 (19-74)		27031 (12803-51406)		22 (10-41)		147350 (70349-279719)		117 (56-223)	
2001	India									46758 (22182-88944)		37 (18-70)		28042 (13150-53536)		22 (10-42)		141189 (66979-268576)		112 (53-212)	
2002	India									44678 (21195-85017)		35 (17-67)		26688 (12512-50976)		21 (10-40)		134909 (63999-256716)		106 (50-202)	
2003	India									42690 (20144-81384)		34 (16-64)		26736 (12385-51339)		21 (10-40)		128906 (60825-245746)		101 (48-193)	
2004	India									40722 (19159-77695)		32 (15-61)		26171 (12080-50249)		21 (9-39)		122963 (57853-234607)		97 (45-184)	
2005	India									38866 (18438-73992)		30 (14-58)		24976 (11704-47734)		20 (9-37)		117360 (55676-223427)		92 (44-175)	
2006	India									37203 (17588-70878)		29 (14-56)		27605 (13006-52528)		22 (10-41)		112339 (53110-214024)		88 (42-168)	
2007	India									35385 (16880-67226)		28 (13-53)		26871 (12815-50959)		21 (10-40)		106848 (50972-202997)		84 (40-159)	
2008	India									33517 (15979-63664)		26 (13-50)		25461 (12128-48240)		20 (10-38)		101209 (48250-192239)		79 (38-151)	
2009	India									31490 (14882-59979)		25 (12-47)		25581 (12056-48618)		20 (9-38)		95088 (44938-181113)		75 (35-143)	
2010	India									29647 (14011-56547)		23 (11-45)		24024 (11315-45695)		19 (9-36)		89521 (42308-170750)		71 (33-135)	
2011	India									27949 (12738-54077)		22 (10-43)		28982 (13224-55929)		23 (11-45)		84395 (38464-163290)		67 (31-130)	
2012	India									25987 (11782-50796)		21 (9-41)		26857 (12184-52390)		22 (10-42)		78471 (35576-153384)		63 (29-123)	
2013	India									24438 (10959-47820)		20 (9-39)		26823 (12034-52550)		22 (10-43)		73794 (33092-144698)		60 (27-117)	
2014	India									22774 (10223-44800)		19 (8-37)		24934 (11198-49008)		20 (9-40)		68767 (30870-135279)		56 (25-111)	
2015	India	3133014 (2703233-3724272)	46741 (40329-55562)	2575 (2222-3061)	38 (33-46)	1229970 (921487-1402899)	17246 (12921-19671)	1011 (757-1153)	14 (11-16)	21368 (9528-42098)	319 (142-628)	18 (8-35)	0 (0-1)	24127 (10760-47529)	360 (161-709)	20 (9-39)	0 (0-1)	64522 (28771-127120)	963 (429-1896)	53 (24-104)	1 (0-2)
2000	Indonesia	769893 (664280-915186)		3675 (3170-4368)		301833 (226132-344270)		1441 (1079-1643)		3045 (1103-6597)		15 (5-31)		3459 (1253-7492)		17 (6-36)		9195 (3332-19919)		44 (16-95)	
2001	Indonesia									2885 (1056-6232)		14 (5-30)		3277 (1199-7078)		16 (6-34)		8711 (3188-18819)		41 (15-89)	
2002	Indonesia									2748 (1014-5919)		13 (5-28)		3121 (1152-6723)		15 (5-32)		8298 (3063-17873)		39 (14-84)	
2003	Indonesia									2601 (958-5593)		12 (4-26)		2954 (1089-6353)		14 (5-30)		7854 (2894-16889)		37 (13-79)	
2004	Indonesia									3185 (1192-6810)		15 (5-31)		3617 (1353-7735)		17 (6-36)		9616 (3598-20563)		44 (17-95)	
2005	Indonesia									2358 (896-5011)		11 (4-23)		2679 (1017-5692)		12 (5-26)		7121 (2705-15132)		33 (12-69)	
2006	Indonesia									2259 (871-4767)		10 (4-22)		2566 (989-5415)		12 (5-25)		6822 (2630-14395)		31 (12-66)	
2007	Indonesia									2156 (843-4515)		10 (4-20)		2449 (958-5128)		11 (4-23)		6512 (2546-13633)		29 (12-62)	
2008	Indonesia									2058 (824-4265)		9 (4-19)		2338 (936-4844)		11 (4-22)		6215 (2487-12878)		28 (11-58)	
2009	Indonesia									2001 (820-4100)		9 (4-18)		2273 (931-4657)		10 (4-21)		6042 (2476-12380)		27 (11-55)	
2010	Indonesia									1997 (838-4042)		9 (4-18)		2268 (952-4591)		10 (4-20)		6029 (2530-12205)		27 (11-54)	
2011	Indonesia									2057 (884-4112)		9 (4-18)		2336 (1004-4670)		10 (4-21)		6211 (2670-12417)		27 (12-55)	
2012	Indonesia									2175 (959-4293)		9 (4-19)		2471 (1089-4876)		11 (5-21)		6568 (2896-12963)		28 (13-56)	
2013	Indonesia									2343 (822-5532)		10 (3-23)		2662 (934-6283)		11 (4-27)		7076 (2482-16703)		30 (10-70)	
2014	Indonesia									2440 (861-5726)		10 (4-24)		2772 (978-6503)		11 (4-27)		7368 (2601-17289)		30 (11-71)	
2015	Indonesia	585770 (505415-696315)	12889 (11121-15322)	2396 (2067-2848)	53 (45-63)	230600 (172765-263022)	5074 (3801-5787)	943 (707-1076)	21 (16-24)	2480 (881-5791)	55 (19-127)	10 (4-24)	0 (0-1)	2816 (1001-6577)	62 (22-145)	12 (4-27)	0 (0-1)	7488 (2661-17487)	165 (59-385)	31 (11-72)	1 (0-2)
2000	Iran (Islamic Republic of)	142751 (123168-169690)		2276 (1963-2705)		56139 (42059-64031)		895 (670-1021)		794 (434-1321)		13 (7-21)		902 (493-1501)		14 (8-24)		2398 (1310-3989)		38 (21-64)	
2001	Iran (Islamic Republic of)									722 (398-1213)		12 (7-20)		820 (453-1377)		14 (7-23)		2180 (1203-3662)		36 (20-61)	
2002	Iran (Islamic Republic of)									663 (354-1126)		11 (6-19)		754 (403-1278)		13 (7-22)		2003 (1070-3399)		34 (18-58)	
2003	Iran (Islamic Republic of)									609 (292-1248)		11 (5-22)		692 (332-1417)		12 (6-25)		1838 (882-3768)		33 (16-67)	
2004	Iran (Islamic Republic of)									556 (251-1159)		10 (5-21)		632 (286-1317)		12 (5-24)		1679 (759-3500)		31 (14-64)	
2005	Iran (Islamic Republic of)									496 (213-1053)		9 (4-19)		563 (242-1196)		10 (4-22)		1497 (642-3180)		28 (12-59)	
2006	Iran (Islamic Republic of)									445 (182-966)		8 (3-17)		506 (207-1097)		9 (4-20)		1345 (551-2918)		24 (10-52)	
2007	Iran (Islamic Republic of)									395 (155-876)		7 (3-15)		449 (176-995)		8 (3-17)		1193 (469-2644)		21 (8-46)	
2008	Iran (Islamic Republic of)									356 (134-805)		6 (2-14)		405 (153-915)		7 (3-15)		1076 (406-2431)		18 (7-41)	
2009	Iran (Islamic Republic of)									327 (118-750)		5 (2-12)		371 (134-852)		6 (2-14)		987 (357-2265)		16 (6-37)	
2010	Iran (Islamic Republic of)									310 (108-719)		5 (2-11)		352 (122-817)		6 (2-13)		936 (325-2173)		15 (5-35)	
2011	Iran (Islamic Republic of)									293 (102-683)		5 (2-11)		333 (116-776)		5 (2-12)		886 (309-2064)		14 (5-32)	
2012	Iran (Islamic Republic of)									282 (100-657)		4 (2-10)		321 (113-746)		5 (2-11)		852 (300-1984)		13 (5-30)	
2013	Iran (Islamic Republic of)									275 (98-640)		4 (1-10)		313 (111-727)		5 (2-11)		832 (296-1932)		12 (4-29)	
2014	Iran (Islamic Republic of)									263 (95-610)		4 (1-9)		299 (107-693)		4 (2-10)		795 (286-1843)		12 (4-27)	
2015	Iran (Islamic Republic of)	72319 (62398-85967)	163 (140-193)	1073 (926-1275)	2 (2-3)	28173 (21107-32134)	63 (47-72)	418 (313-477)	1 (1-1)	250 (90-577)	1 (0-1)	4 (1-9)	0 (0-0)	284 (103-656)	1 (0-1)	4 (2-10)	0 (0-0)	754 (273-1744)	2 (1-4)	11 (4-26)	0 (0-0)
2000	Iraq	55593 (47967-66084)		1472 (1270-1750)		21628 (16203-24668)		573 (429-653)		438 (194-855)		12 (5-23)		497 (220-971)		13 (6-26)		1322 (585-2581)		35 (15-68)	
2001	Iraq									441 (198-856)		11 (5-22)		501 (225-972)		13 (6-25)		1331 (599-2585)		34 (15-67)	
2002	Iraq									447 (204-863)		11 (5-22)		508 (232-981)		13 (6-25)		1351 (617-2607)		34 (16-66)	
2003	Iraq									457 (212-875)		11 (5-22)		519 (240-994)		13 (6-25)		1379 (639-2642)		34 (16-66)	
2004	Iraq									460 (216-874)		11 (5-21)		522 (246-993)		13 (6-24)		1389 (653-2639)		34 (16-65)	
2005	Iraq									461 (221-871)		11 (5-21)		524 (251-989)		13 (6-24)		1393 (666-2630)		33 (16-63)	
2006	Iraq									456 (222-857)		11 (5-20)		517 (252-974)		12 (6-23)		1376 (671-2588)		32 (16-61)	
2007	Iraq									451 (214-856)		10 (5-20)		512 (243-972)		12 (6-22)		1362 (647-2584)		31 (15-59)	
2008	Iraq									446 (207-856)		10 (5-19)		507 (235-972)		11 (5-22)		1348 (625-2585)		30 (14-57)	
2009	Iraq									438 (198-849)		9 (4-18)		497 (225-964)		11 (5-21)		1321 (599-2563)		28 (13-55)	
2010	Iraq									422 (188-829)		9 (4-17)		480 (213-941)		10 (4-19)		1275 (566-2502)		26 (12-51)	
2011	Iraq									401 (174-795)		8 (3-16)		455 (198-903)		9 (4-18)		1209 (527-2401)		24 (10-48)	
2012	Iraq									384 (164-771)		7 (3-15)		436 (186-876)		8 (4-17)		1160 (495-2328)		22 (9-45)	
2013	Iraq									372 (155-753)		7 (3-14)		423 (177-856)		8 (3-16)		1123 (470-2275)		21 (9-42)	
2014	Iraq									355 (145-724)		6 (3-13)		403 (165-822)		7 (3-15)		1071 (438-2186)		19 (8-40)	
2015	Iraq	79601 (68682-94623)	0 (0-0)	1413 (1219-1680)	0 (0-0)	31294 (23445-35694)	0 (0-0)	556 (416-634)	0 (0-0)	347 (139-713)	0 (0-0)	6 (2-13)	0 (0-0)	394 (158-810)	0 (0-0)	7 (3-14)	0 (0-0)	1047 (419-2153)	0 (0-0)	19 (7-38)	0 (0-0)
2000	Ireland	304 (262-361)		115 (99-137)		197 (148-225)		75 (56-85)		60 (27-108)		23 (10-41)		68 (31-123)		26 (12-47)		180 (82-327)		68 (31-124)	
2001	Ireland									33 (15-61)		12 (6-22)		38 (17-69)		14 (6-25)		101 (46-183)		37 (17-67)	
2002	Ireland									38 (17-70)		14 (6-25)		44 (20-79)		16 (7-28)		116 (53-210)		41 (19-75)	
2003	Ireland									36 (16-66)		13 (6-23)		41 (19-75)		14 (7-26)		109 (50-198)		38 (17-69)	
2004	Ireland									36 (16-66)		12 (6-23)		41 (19-75)		14 (6-26)		109 (50-199)		37 (17-68)	
2005	Ireland									42 (19-75)		14 (6-25)		47 (21-86)		16 (7-28)		126 (57-228)		42 (19-76)	
2006	Ireland									16 (7-30)		5 (2-9)		18 (8-34)		6 (3-11)		49 (22-89)		16 (7-29)	
2007	Ireland									31 (14-56)		10 (4-17)		35 (16-64)		11 (5-20)		93 (42-169)		29 (13-52)	
2008	Ireland									28 (13-51)		8 (4-15)		32 (14-58)		9 (4-17)		85 (38-154)		25 (11-45)	
2009	Ireland									19 (9-34)		5 (2-10)		21 (10-39)		6 (3-11)		57 (26-103)		16 (7-30)	
2010	Ireland									13 (6-23)		4 (2-6)		14 (6-26)		4 (2-7)		38 (17-69)		11 (5-19)	
2011	Ireland									11 (5-19)		3 (1-5)		12 (5-22)		3 (2-6)		32 (15-58)		9 (4-16)	
2012	Ireland									8 (4-15)		2 (1-4)		9 (4-17)		3 (1-5)		25 (11-45)		7 (3-12)	
2013	Ireland									7 (3-13)		2 (1-4)		8 (4-15)		2 (1-4)		21 (10-39)		6 (3-11)	
2014	Ireland									4 (2-8)		1 (1-2)		5 (2-9)		1 (1-3)		13 (6-24)		4 (2-7)	
2015	Ireland	94 (81-112)	0 (0-0)	27 (23-32)	0 (0-0)	61 (46-69)	0 (0-0)	18 (13-20)	0 (0-0)	3 (1-6)	0 (0-0)	1 (0-2)	0 (0-0)	4 (2-7)	0 (0-0)	1 (0-2)	0 (0-0)	10 (4-17)	0 (0-0)	3 (1-5)	0 (0-0)
2000	Israel	694 (599-825)		115 (99-137)		450 (337-513)		75 (56-85)		7 (4-10)		1 (1-2)		8 (5-12)		1 (1-2)		22 (13-31)		4 (2-5)	
2001	Israel									32 (19-44)		5 (3-7)		37 (22-50)		6 (4-8)		97 (58-134)		16 (9-22)	
2002	Israel									79 (47-108)		12 (7-17)		89 (54-123)		14 (8-19)		237 (142-327)		38 (23-52)	
2003	Israel									61 (36-84)		9 (6-13)		69 (41-95)		11 (6-15)		183 (110-253)		29 (17-40)	
2004	Israel									35 (21-48)		5 (3-7)		40 (24-55)		6 (4-8)		105 (63-145)		16 (10-23)	
2005	Israel									25 (15-34)		4 (2-5)		28 (17-39)		4 (3-6)		75 (45-103)		11 (7-16)	
2006	Israel									72 (44-100)		11 (7-15)		82 (49-114)		12 (7-17)		219 (131-302)		33 (20-46)	
2007	Israel									27 (16-37)		4 (2-6)		31 (18-42)		5 (3-6)		81 (49-112)		12 (7-17)	
2008	Israel									49 (30-68)		7 (4-10)		56 (34-77)		8 (5-11)		149 (90-206)		22 (13-30)	
2009	Israel									40 (24-54)		6 (3-8)		45 (27-62)		6 (4-9)		119 (72-165)		17 (10-23)	
2010	Israel									37 (22-51)		5 (3-7)		42 (25-58)		6 (3-8)		112 (67-154)		15 (9-21)	
2011	Israel									32 (19-45)		4 (3-6)		37 (22-51)		5 (3-7)		98 (59-135)		13 (8-18)	
2012	Israel									18 (11-25)		2 (1-3)		20 (12-28)		3 (2-4)		54 (33-75)		7 (4-10)	
2013	Israel									23 (14-31)		3 (2-4)		26 (16-36)		3 (2-5)		69 (41-95)		9 (5-12)	
2014	Israel									23 (14-32)		3 (2-4)		27 (16-37)		3 (2-5)		71 (42-97)		9 (5-12)	
2015	Israel	341 (295-406)	0 (0-0)	42 (36-50)	0 (0-0)	221 (166-252)	0 (0-0)	27 (20-31)	0 (0-0)	18 (11-25)	0 (0-0)	2 (1-3)	0 (0-0)	21 (12-28)	0 (0-0)	3 (2-3)	0 (0-0)	55 (33-76)	0 (0-0)	7 (4-9)	0 (0-0)
2000	Italy	2978 (2569-3539)		115 (99-137)		1930 (1446-2202)		75 (56-85)		40 (18-72)		2 (1-3)		45 (21-82)		2 (1-3)		120 (55-218)		5 (2-8)	
2001	Italy									49 (22-89)		2 (1-3)		56 (25-101)		2 (1-4)		148 (67-268)		6 (3-10)	
2002	Italy									50 (23-91)		2 (1-4)		57 (26-104)		2 (1-4)		152 (69-276)		6 (3-11)	
2003	Italy									81 (37-146)		3 (1-6)		91 (41-166)		3 (2-6)		243 (110-441)		9 (4-17)	
2004	Italy									54 (25-98)		2 (1-4)		61 (28-111)		2 (1-4)		163 (74-296)		6 (3-11)	
2005	Italy									54 (25-99)		2 (1-4)		62 (28-112)		2 (1-4)		164 (75-298)		6 (3-11)	
2006	Italy									72 (33-131)		3 (1-5)		82 (37-149)		3 (1-5)		218 (99-395)		8 (4-14)	
2007	Italy									60 (27-109)		2 (1-4)		68 (31-124)		2 (1-4)		181 (82-328)		7 (3-12)	
2008	Italy									34 (15-62)		1 (1-2)		39 (18-70)		1 (1-3)		103 (47-187)		4 (2-7)	
2009	Italy									45 (20-81)		2 (1-3)		51 (23-92)		2 (1-3)		135 (61-245)		5 (2-9)	
2010	Italy									33 (15-59)		1 (1-2)		37 (17-67)		1 (1-2)		98 (45-178)		4 (2-6)	
2011	Italy									18 (8-32)		1 (0-1)		20 (9-37)		1 (0-1)		54 (24-97)		2 (1-4)	
2012	Italy									11 (5-20)		0 (0-1)		13 (6-23)		0 (0-1)		34 (15-61)		1 (1-2)	
2013	Italy									9 (4-16)		0 (0-1)		10 (4-18)		0 (0-1)		26 (12-48)		1 (0-2)	
2014	Italy									8 (3-14)		0 (0-1)		9 (4-16)		0 (0-1)		23 (11-42)		1 (0-2)	
2015	Italy	573 (494-681)	0 (0-0)	23 (20-27)	0 (0-0)	371 (278-424)	0 (0-0)	15 (11-17)	0 (0-0)	7 (3-12)	0 (0-0)	0 (0-0)	0 (0-0)	8 (3-14)	0 (0-0)	0 (0-1)	0 (0-0)	20 (8-36)	0 (0-0)	1 (0-1)	0 (0-0)
2000	Jamaica	4080 (3520-4849)		1452 (1253-1726)		1589 (1191-1812)		566 (424-645)		2 (0-7)		1 (0-3)		2 (0-8)		1 (0-3)		6 (1-22)		2 (0-8)	
2001	Jamaica									2 (0-7)		1 (0-3)		2 (0-8)		1 (0-3)		6 (1-21)		2 (0-8)	
2002	Jamaica									2 (0-7)		1 (0-3)		3 (0-8)		1 (0-3)		7 (1-22)		2 (0-8)	
2003	Jamaica									3 (1-10)		1 (0-4)		4 (1-12)		1 (0-4)		10 (2-31)		4 (1-12)	
2004	Jamaica									5 (1-16)		2 (1-6)		6 (1-19)		2 (1-7)		16 (4-50)		6 (2-19)	
2005	Jamaica									7 (2-20)		3 (1-8)		8 (2-22)		3 (1-9)		20 (6-59)		8 (2-24)	
2006	Jamaica									8 (2-22)		3 (1-9)		9 (3-25)		4 (1-10)		23 (7-66)		10 (3-28)	

Spn = *Streptococcus pneumoniae*; npnm = non-pneumonia, non-meningitis; HIV = cases only in HIV-infected children; Uncertainty range provided in parentheses.

Year	Country	Spn pneumonia cases (UR)	Spn pneumonia cases, HIV (UR)	Spn pneumonia incidence rate (UR)	Spn pneumonia incidence rate, HIV (UR)	Spn severe pneumonia cases (UR)	Spn severe pneumonia cases, HIV (UR)	Spn severe pneumonia incidence rate (UR)	Spn severe pneumonia incidence rate, HIV (UR)	Spn meningitis cases (UR)	Spn meningitis cases, HIV (UR)	Spn meningitis incidence rate (UR)	Spn meningitis incidence rate, HIV (UR)	Spn NPNM cases (UR)	Spn NPNM cases, HIV (UR)	Spn NPNM incidence rate (UR)	Spn NPNM incidence rate, HIV (UR)	Spn NPNM non-severe cases (UR)	Spn NPNM non-severe cases, HIV (UR)	SPN NPNM non-severe incidence rate (UR)	SPN NPNM non-severe incidence rate, HIV (UR)
2007	Jamaica									9 (3-24)		4 (1-11)		10 (3-28)		4 (1-12)		27 (8-74)		11 (3-32)	
2008	Jamaica									10 (3-28)		5 (1-12)		12 (4-32)		5 (2-14)		31 (9-86)		14 (4-37)	
2009	Jamaica									10 (3-26)		4 (1-12)		11 (3-30)		5 (2-13)		30 (9-80)		13 (4-35)	
2010	Jamaica									10 (3-25)		4 (1-11)		11 (3-29)		5 (2-13)		29 (9-76)		13 (4-35)	
2011	Jamaica									9 (3-24)		4 (1-11)		10 (3-27)		5 (2-12)		28 (9-73)		13 (4-33)	
2012	Jamaica									9 (3-24)		4 (1-11)		11 (4-27)		5 (2-13)		28 (9-72)		13 (4-34)	
2013	Jamaica									8 (3-21)		4 (1-10)		10 (3-24)		5 (2-12)		25 (9-64)		12 (4-31)	
2014	Jamaica									8 (3-20)		4 (1-10)		9 (3-23)		5 (2-11)		25 (9-61)		12 (4-30)	
2015	Jamaica	1861 (1606-2213)	42 (36-49)	929 (802-1105)	21 (18-25)	725 (543-827)	16 (12-18)	362 (271-413)	8 (6-9)	8 (3-19)	0 (0-0)	4 (1-10)	0 (0-0)	9 (3-22)	0 (0-0)	4 (2-11)	0 (0-0)	24 (9-58)	1 (0-1)	12 (4-29)	0 (0-1)
2000	Japan	11041 (9527-13125)		192 (165-228)		7158 (5363-8164)		124 (93-142)		222 (133-306)		4 (2-5)		252 (151-348)		4 (3-6)		670 (402-924)		12 (7-16)	
2001	Japan									200 (120-275)		3 (2-5)		227 (136-313)		4 (2-5)		603 (362-832)		10 (6-14)	
2002	Japan									152 (91-210)		3 (2-4)		173 (104-239)		3 (2-4)		460 (276-635)		8 (5-11)	
2003	Japan									182 (109-252)		3 (2-4)		207 (124-286)		4 (2-5)		551 (331-760)		10 (6-13)	
2004	Japan									137 (82-189)		2 (1-3)		155 (93-214)		3 (2-4)		413 (248-570)		7 (4-10)	
2005	Japan									153 (92-211)		3 (2-4)		174 (104-239)		3 (2-4)		461 (277-636)		8 (5-11)	
2006	Japan									115 (69-159)		2 (1-3)		131 (78-180)		2 (1-3)		348 (209-479)		6 (4-9)	
2007	Japan									150 (90-207)		3 (2-4)		170 (102-235)		3 (2-4)		453 (272-625)		8 (5-12)	
2008	Japan									86 (52-119)		2 (1-2)		98 (59-135)		2 (1-3)		260 (156-359)		5 (3-7)	
2009	Japan									116 (70-160)		2 (1-3)		132 (79-182)		2 (1-3)		351 (211-484)		7 (4-9)	
2010	Japan									155 (93-214)		3 (2-4)		176 (106-243)		3 (2-5)		468 (281-646)		9 (5-12)	
2011	Japan									69 (41-95)		1 (1-2)		78 (47-108)		1 (1-2)		209 (125-288)		4 (2-5)	
2012	Japan									52 (31-72)		1 (1-1)		59 (35-81)		1 (1-2)		157 (94-217)		3 (2-4)	
2013	Japan									58 (35-81)		1 (1-2)		66 (40-92)		1 (1-2)		176 (106-243)		3 (2-5)	
2014	Japan									53 (32-73)		1 (1-1)		60 (36-83)		1 (1-2)		160 (96-221)		3 (2-4)	
2015	Japan	6714 (5793-7981)	1 (0-1)	130 (112-154)	0 (0-0)	4353 (3261-4965)	0 (0-0)	84 (63-96)	0 (0-0)	45 (27-63)	0 (0-0)	1 (1-1)	0 (0-0)	52 (31-71)	0 (0-0)	1 (1-1)	0 (0-0)	137 (82-189)	0 (0-0)	3 (2-4)	0 (0-0)
2000	Jordan	14753 (12730-17538)		2162 (1866-2570)		5720 (4285-6524)		838 (628-956)		31 (12-71)		5 (2-10)		35 (13-80)		5 (2-12)		94 (35-213)		14 (5-31)	
2001	Jordan									47 (17-107)		7 (3-16)		53 (20-122)		8 (3-18)		142 (53-324)		21 (8-47)	
2002	Jordan									55 (20-126)		8 (3-18)		62 (23-143)		9 (3-21)		166 (61-381)		24 (9-55)	
2003	Jordan									52 (19-121)		8 (3-17)		59 (21-137)		9 (3-20)		158 (57-364)		23 (8-53)	
2004	Jordan									49 (18-114)		7 (3-16)		56 (20-130)		8 (3-18)		149 (53-344)		21 (8-49)	
2005	Jordan									48 (17-111)		7 (2-15)		54 (19-126)		8 (3-18)		144 (51-336)		20 (7-47)	
2006	Jordan									48 (17-112)		7 (2-15)		54 (19-128)		7 (3-17)		145 (50-339)		20 (7-46)	
2007	Jordan									47 (16-111)		6 (2-15)		54 (18-126)		7 (2-17)		143 (49-335)		19 (6-44)	
2008	Jordan									47 (16-109)		6 (2-14)		53 (18-124)		7 (2-16)		141 (49-330)		18 (6-42)	
2009	Jordan									46 (16-109)		6 (2-13)		53 (18-123)		6 (2-15)		140 (49-328)		17 (6-40)	
2010	Jordan									46 (16-106)		5 (2-12)		52 (18-121)		6 (2-14)		138 (49-321)		16 (6-38)	
2011	Jordan									45 (16-105)		5 (2-12)		52 (18-120)		6 (2-14)		137 (49-318)		16 (6-36)	
2012	Jordan									45 (16-104)		5 (2-11)		51 (18-118)		6 (2-13)		136 (49-314)		15 (5-34)	
2013	Jordan									44 (16-102)		5 (2-11)		50 (18-115)		5 (2-12)		133 (48-307)		14 (5-33)	
2014	Jordan									43 (16-98)		4 (2-10)		49 (18-112)		5 (2-12)		129 (48-297)		14 (5-31)	
2015	Jordan	10345 (8926-12297)	0 (0-0)	1073 (926-1276)	0 (0-0)	4022 (3013-4588)	0 (0-0)	417 (313-476)	0 (0-0)	41 (15-93)	0 (0-0)	4 (2-10)	0 (0-0)	46 (17-106)	0 (0-0)	5 (2-11)	0 (0-0)	123 (46-282)	0 (0-0)	13 (5-29)	0 (0-0)
2000	Kazakhstan	5055 (4361-6009)		460 (397-547)		3277 (2455-3738)		298 (223-340)		114 (45-239)		10 (4-22)		130 (51-271)		12 (5-25)		345 (135-721)		31 (12-66)	
2001	Kazakhstan									106 (42-220)		10 (4-21)		120 (48-250)		11 (4-23)		319 (126-664)		30 (12-62)	
2002	Kazakhstan									102 (41-211)		9 (4-20)		116 (46-240)		11 (4-22)		308 (123-638)		29 (11-59)	
2003	Kazakhstan									99 (40-205)		9 (4-18)		113 (46-233)		10 (4-21)		300 (121-620)		27 (11-56)	
2004	Kazakhstan									99 (40-203)		9 (3-17)		113 (46-231)		10 (4-20)		299 (122-614)		26 (10-53)	
2005	Kazakhstan									102 (42-208)		8 (3-17)		116 (48-236)		9 (4-19)		307 (126-627)		25 (10-51)	
2006	Kazakhstan									106 (44-215)		8 (3-17)		121 (50-244)		9 (4-19)		321 (133-649)		25 (10-50)	
2007	Kazakhstan									114 (38-272)		8 (3-20)		129 (43-309)		9 (3-23)		343 (114-820)		25 (8-60)	
2008	Kazakhstan									121 (43-284)		8 (3-20)		138 (49-323)		10 (3-23)		366 (129-858)		26 (9-60)	
2009	Kazakhstan									126 (47-289)		8 (3-19)		143 (54-328)		10 (4-22)		381 (143-873)		25 (10-58)	
2010	Kazakhstan									129 (51-288)		8 (3-18)		146 (58-327)		9 (4-21)		389 (155-870)		25 (10-55)	
2011	Kazakhstan									124 (49-276)		7 (3-16)		141 (56-313)		8 (3-19)		376 (148-832)		22 (9-50)	
2012	Kazakhstan									97 (39-212)		5 (2-12)		110 (44-241)		6 (3-14)		292 (117-642)		17 (7-36)	
2013	Kazakhstan									80 (33-175)		4 (2-10)		91 (37-198)		5 (2-11)		242 (99-528)		13 (5-29)	
2014	Kazakhstan									70 (29-152)		4 (2-8)		80 (33-173)		4 (2-9)		212 (88-459)		11 (5-24)	
2015	Kazakhstan	5635 (4862-6698)	9 (8-10)	294 (254-350)	0 (0-1)	3653 (2737-4167)	6 (4-6)	191 (143-217)	0 (0-0)	57 (24-123)	0 (0-0)	3 (1-6)	0 (0-0)	65 (27-139)	0 (0-0)	3 (1-7)	0 (0-0)	172 (72-370)	0 (0-1)	9 (4-19)	0 (0-0)
2000	Kenya	154482 (133290-183635)		3101 (2676-3687)		60216 (45114-68683)		1209 (906-1379)		1656 (662-3515)		33 (13-71)		664 (265-1410)		13 (5-28)		4999 (1998-10615)		100 (40-213)	
2001	Kenya									1649 (663-3493)		32 (13-68)		661 (266-1401)		13 (5-27)		4979 (2002-10548)		97 (39-204)	
2002	Kenya									1643 (665-3471)		31 (12-65)		659 (267-1392)		12 (5-26)		4962 (2008-10480)		93 (38-197)	
2003	Kenya									1622 (661-3413)		30 (12-62)		650 (265-1369)		12 (5-25)		4896 (1996-10305)		89 (36-188)	
2004	Kenya									1597 (659-3343)		28 (12-59)		641 (264-1341)		11 (5-24)		4822 (1989-10094)		86 (35-179)	
2005	Kenya									1528 (638-3183)		26 (11-55)		613 (256-1277)		11 (4-22)		4614 (1926-9611)		80 (33-166)	
2006	Kenya									1516 (641-3144)		25 (11-53)		608 (257-1261)		10 (4-21)		4577 (1936-9495)		77 (32-159)	
2007	Kenya									1534 (658-3171)		25 (11-52)		615 (264-1272)		10 (4-21)		4632 (1987-9574)		75 (32-156)	
2008	Kenya									1513 (562-3211)		24 (9-51)		1719 (639-3647)		27 (10-58)		4569 (1698-9696)		73 (27-155)	
2009	Kenya									1482 (550-3143)		23 (9-49)		1683 (625-3570)		26 (10-56)		4475 (1662-9491)		70 (26-148)	
2010	Kenya									1442 (549-3038)		22 (8-47)		1637 (623-3451)		25 (10-53)		4353 (1657-9174)		67 (25-140)	
2011	Kenya									938 (365-1960)		14 (5-29)		1065 (415-2227)		16 (6-33)		2832 (1103-5920)		43 (17-89)	
2012	Kenya									812 (324-1681)		12 (5-25)		922 (368-1909)		14 (5-28)		2451 (977-5074)		36 (14-75)	
2013	Kenya									792 (324-1625)		12 (5-24)		899 (368-1845)		13 (5-27)		2391 (977-4906)		35 (14-72)	
2014	Kenya									729 (306-1483)		10 (4-21)		828 (347-1685)		12 (5-24)		2202 (923-4478)		32 (13-64)	
2015	Kenya	53799 (46419-63952)	7520 (6488-8939)	763 (659-907)	107 (92-127)	21076 (15790-24039)	2946 (2207-3360)	299 (224-341)	42 (31-48)	502 (212-1017)	70 (30-142)	7 (3-14)	1 (0-2)	570 (240-1155)	80 (34-161)	8 (3-16)	1 (0-2)	1515 (639-3070)	212 (89-429)	22 (9-44)	3 (1-6)
2000	Kiribati	194 (167-230)		1647 (1421-1957)		76 (57-86)		644 (482-734)		2 (1-6)		19 (7-52)		3 (1-7)		22 (8-59)		7 (2-18)		58 (21-157)	
2001	Kiribati									2 (1-6)		18 (6-48)		2 (1-6)		20 (7-55)		6 (2-17)		54 (19-146)	
2002	Kiribati									2 (1-5)		17 (6-46)		2 (1-6)		19 (7-52)		6 (2-16)		51 (19-139)	
2003	Kiribati									2 (1-5)		16 (6-43)		2 (1-5)		18 (7-48)		5 (2-14)		48 (17-129)	
2004	Kiribati									2 (1-4)		15 (6-41)		2 (1-5)		17 (6-47)		5 (2-13)		47 (17-125)	
2005	Kiribati									2 (1-5)		16 (6-43)		2 (1-5)		18 (7-49)		5 (2-14)		48 (18-130)	
2006	Kiribati									2 (1-5)		17 (6-47)		2 (1-5)		20 (7-53)		5 (2-14)		52 (19-141)	
2007	Kiribati									2 (1-5)		19 (7-51)		2 (1-6)		22 (8-58)		6 (2-17)		57 (21-154)	
2008	Kiribati									2 (1-6)		20 (7-54)		3 (1-7)		23 (8-61)		7 (3-19)		60 (22-162)	
2009	Kiribati									3 (1-7)		20 (7-54)		3 (1-8)		23 (8-62)		8 (3-21)		61 (22-164)	
2010	Kiribati									3 (1-7)		20 (7-54)		3 (1-8)		23 (8-61)		8 (3-22)		61 (22-163)	
2011	Kiribati									3 (1-8)		19 (7-51)		3 (1-9)		22 (8-58)		9 (3-23)		58 (21-155)	
2012	Kiribati									3 (1-7)		18 (7-49)		3 (1-8)		21 (8-55)		8 (3-22)		55 (20-147)	
2013	Kiribati									2 (1-6)		14 (5-38)		2 (1-6)		16 (6-43)		6 (2-17)		43 (16-113)	
2014	Kiribati									2 (1-4)		11 (4-30)		2 (1-5)		13 (5-34)		5 (2-13)		34 (13-91)	
2015	Kiribati	109 (94-130)	0 (0-0)	752 (649-894)	0 (0-0)	43 (32-49)	0 (0-0)	293 (220-335)	0 (0-0)	1 (0-3)	0 (0-0)	8 (3-21)	0 (0-0)	1 (0-3)	0 (0-0)	9 (3-23)	0 (0-0)	3 (1-9)	0 (0-0)	23 (9-62)	0 (0-0)
2000	Kuwait	4215 (3637-5011)		2107 (1818-2505)		1658 (1242-1891)		829 (621-945)		21 (12-29)		10 (6-14)		23 (14-32)		12 (7-16)		62 (37-86)		31 (19-43)	
2001	Kuwait									41 (25-56)		20 (12-27)		46 (28-64)		22 (13-31)		123 (74-170)		59 (36-82)	
2002	Kuwait									8 (5-10)		4 (2-5)		9 (5-12)		4 (2-6)		23 (14-31)		11 (7-15)	
2003	Kuwait									33 (20-45)		16 (9-22)		37 (22-51)		18 (11-25)		98 (59-136)		47 (29-65)	
2004	Kuwait									21 (12-28)		10 (6-14)		23 (14-32)		11 (7-16)		62 (37-86)		30 (18-41)	
2005	Kuwait									32 (19-45)		15 (9-21)		37 (22-51)		17 (10-23)		98 (59-135)		45 (27-62)	
2006	Kuwait									7 (4-10)		3 (2-4)		8 (5-11)		4 (2-5)		21 (13-29)		10 (6-13)	
2007	Kuwait									14 (9-20)		6 (4-9)		16 (10-23)		7 (4-10)		44 (26-60)		19 (11-26)	
2008	Kuwait									0 (0-0)		0 (0-0)		0 (0-0)		0 (0-0)		0 (0-0)		0 (0-0)	
2009	Kuwait									20 (12-28)		7 (4-10)		23 (14-32)		8 (5-12)		62 (37-85)		22 (13-31)	
2010	Kuwait									18 (11-25)		6 (4-8)		20 (12-28)		7 (4-10)		54 (33-75)		18 (11-25)	
2011	Kuwait									33 (20-46)		10 (6-14)		38 (23-52)		12 (7-16)		101 (60-139)		32 (19-44)	
2012	Kuwait									20 (12-28)		6 (4-8)		23 (14-32)		7 (4-10)		62 (37-85)		19 (11-26)	
2013	Kuwait									17 (10-23)		5 (3-7)		19 (11-26)		6 (3-8)		50 (30-69)		15 (9-21)	
2014	Kuwait									19 (11-26)		5 (3-8)		21 (13-29)		6 (4-9)		56 (34-77)		16 (10-23)	
2015	Kuwait	1120 (966-1331)	0 (0-0)	327 (282-389)	0 (0-0)	436 (327-497)	0 (0-0)	127 (95-145)	0 (0-0)	18 (11-24)	0 (0-0)	5 (3-7)	0 (0-0)	20 (12-28)	0 (0-0)	6 (4-8)	0 (0-0)	53 (32-74)	0 (0-0)	16 (9-22)	0 (0-0)
2000	Kyrgyzstan	1235 (1066-1468)		230 (198-273)		801 (600-913)		149 (112-170)		102 (39-216)		19 (7-40)		115 (44-245)		21 (8-46)		307 (117-652)		57 (22-121)	
2001	Kyrgyzstan									92 (35-196)		18 (7-37)		105 (40-223)		20 (8-43)		278 (106-592)		53 (20-113)	
2002	Kyrgyzstan									84 (32-178)		16 (6-35)		95 (36-202)		19 (7-40)		252 (96-538)		50 (19-106)	
2003	Kyrgyzstan									76 (28-161)		15 (6-33)		86 (32-183)		17 (7-37)		228 (86-487)		46 (17-99)	
2004	Kyrgyzstan									69 (26-148)		14 (5-31)		78 (29-168)		16 (6-35)		209 (78-446)		43 (16-92)	
2005	Kyrgyzstan									65 (24-139)		13 (5-29)		74 (28-157)		15 (6-33)		196 (73-418)		41 (15-87)	
2006	Kyrgyzstan									62 (23-134)		13 (5-27)		71 (26-152)		14 (5-31)		189 (70-404)		38 (14-82)	
2007	Kyrgyzstan									61 (23-131)		12 (4-26)		69 (26-149)		14 (5-29)		184 (68-395)		36 (13-77)	
2008	Kyrgyzstan									60 (22-128)		11 (4-24)		68 (25-145)		13 (5-27)		180 (67-387)		34 (12-72)	
2009	Kyrgyzstan									60 (22-130)		11 (4-23)		68 (25-147)		12 (4-26)		182 (67-391)		32 (12-69)	
2010	Kyrgyzstan									61 (22-130)		10 (4-22)		69 (25-148)		11 (4-25)		183 (67-394)		31 (11-66)	
2011	Kyrgyzstan									62 (16-160)		10 (3-25)		70 (18-181)		11 (3-28)		187 (49-482)		29 (8-75)	
2012	Kyrgyzstan									62 (16-159)		9 (2-23)		70 (18-181)		10 (3-27)		186 (48-481)		27 (7-71)	
2013	Kyrgyzstan									61 (16-157)		8 (2-22)		69 (18-179)		10 (2-25)		183 (48-475)		25 (7-66)	
2014	Kyrgyzstan									59 (15-152)		8 (2-20)		66 (17-173)		9 (2-23)		177 (46-459)		24 (6-61)	
2015	Kyrgyzstan	1765 (1523-2098)	25 (22-30)	230 (198-273)	3 (3-4)	1144 (857-1305)	16 (12-19)	149 (112-170)	2 (2-2)	55 (14-142)	1 (0-2)	7 (2-18)	0 (0-0)	62 (16-161)	1 (0-2)	8 (2-21)	0 (0-0)	165 (42-428)	2 (1-6)	21 (6-56)	0 (0-1)
2000	Lao People's Democratic Republic	28492 (24583-33869)		3617 (3121-4299)		10963 (8213-12504)		1392 (1043-1587)		257 (89-590)		33 (11-75)		103 (36-237)		13 (5-30)		776 (268-1781)		99 (34-226)	
2001	Lao People's Democratic Republic									243 (83-558)		32 (11-73)		97 (33-224)		13 (4-29)		734 (252-1686)		96 (33-220)	
2002	Lao People's Democratic Republic									230 (79-529)		30 (10-70)		92 (32-212)		12 (4-28)		694 (237-1598)		92 (31-212)	
2003	Lao People's Democratic Republic									218 (74-504)		29 (10-68)		88 (30-202)		12 (4-27)		660 (224-1521)		88 (30-204)	
2004	Lao People's Democratic Republic									208 (70-480)		28 (9-65)		83 (28-193)		11 (4-26)		628 (212-1451)		84 (29-195)	
2005	Lao People's Democratic Republic									200 (67-462)		27 (9-62)		80 (27-185)		11 (4-25)		603 (203-1394)		81 (27-187)	
2006	Lao People's Democratic Republic									195 (65-450)		26 (9-60)		78 (26-181)		10 (3-24)		589 (196-1359)		79 (26-182)	
2007	Lao People's Democratic Republic									192 (66-439)		25 (9-58)		77 (27-176)		10 (4-23)		581 (201-1325)		77 (26-175)	
2008	Lao People's Democratic Republic									193 (69-435)		25 (9-56)		77 (28-174)		10 (4-23)		583 (209-1312)		76 (27-170)	
2009	Lao People's Democratic Republic									194 (72-430)		25 (9-55)		78 (29-173)		10 (4-22)		586 (218-1300)		75 (28-165)	
2010	Lao People's Democratic Republic									196 (76-427)		25 (9-54)		79 (30-171)		10 (4-22)		591 (228-1291)		74 (29-162)	
2011	Lao People's Democratic Republic									196 (79-423)		24 (10-52)		79 (32-169)		10 (4-21)		592 (239-1276)		73 (29-156)	
2012	Lao People's Democratic Republic									192 (66-426)		23 (8-52)		218 (75-484)		26 (9-59)		581 (199-1287)		70 (24-156)	
2013	Lao People's Democratic Republic									185 (64-409)		22 (8-50)		210 (72-464)		25 (9-56)		558 (192-1234)		68 (23-150)	
2014	Lao People's Democratic Republic									111 (38-245)		13 (5-30)		126 (44-278)		15 (5-34)		335 (116-739)		41 (14-90)	
2015	Lao People's Democratic Republic	14542 (12547-17286)	277 (239-329)	1763 (1521-2096)	34 (29-40)	5709 (4277-6512)	109 (81-124)	692 (519-790)	13 (10-15)	93 (32-204)	2 (1-4)	11 (4-25)	0 (0-0)	105 (37-232)	2 (1-4)	13 (4-28)	0 (0-1)	280 (98-617)	5 (2-12)	34 (12-75)	1 (0-1)
2000	Latvia	444 (383-528)		460 (397-547)		288 (216-328)		298 (223-340)		13 (6-23)		13 (6-24)		15 (7-27)		15 (7-28)		39 (18-71)		40 (18-73)	
2001	Latvia									15 (7-26)		16 (7-28)		16 (7-30)		18 (8-32)		44 (20-80)		47 (21-85)	
2002	Latvia									3 (1-6)		3 (2-6)		4 (2-6)		4 (2-7)		9 (4-17)		10 (5-18)	
2003	Latvia									14 (6-26)		15 (7-27)		16 (7-29)		17 (8-31)		43 (19-78)		46 (21-83)	
2004	Latvia									9 (4-16)		9 (4-16)		10 (4-18)		10 (5-18)		26 (12-47)		27 (12-49)	
2005	Latvia									11 (5-21)		12 (5-21)		13 (6-23)		13 (6-24)		34 (16-62)		35 (16-63)	
2006	Latvia									11 (5-21)		11 (5-20)		13 (6-23)		12 (6-23)		34 (16-62)		33 (15-60)	
2007	Latvia									4 (2-7)		4 (2-7)		5 (2-8)		4 (2-8)		12 (6-22)		11 (5-21)	
2008	Latvia									10 (5-19)		10 (4-17)		12 (5-21)		11 (5-20)		31 (14-56)		29 (13-52)	
2009	Latvia									7 (3-13)		6 (3-12)		8 (4-14)		7 (3-13)		21 (10-38)		19 (9-35)	
2010	Latvia									0 (0-0)		0 (0-0)		0 (0-0)		0 (0-0)		0 (0-0)		0 (0-0)	
2011	Latvia									9 (4-16)		8 (4-15)		10 (5-18)		10 (4-17)		27 (12-49)		25 (11-46)	
2012	Latvia									0 (0-0)		0 (0-0)		0 (0-0)		0 (0-0)		0 (0-0)		0 (0-0)	
2013	Latvia									2 (1-3)		2 (1-3)		2 (1-3)		2 (1-3)		5 (2-9)		5 (2-9)	
2014	Latvia									2 (1-3)		2 (1-3)		2 (1-3)		2 (1-3)		5 (2-8)		5 (2-9)	
2015	Latvia	167 (144-199)	0 (0-0)	179 (154-213)	0 (0-0)	108 (81-124)	0 (0-0)	116 (87-132)	0 (0-0)	1 (0-2)	0 (0-0)	1 (0-2)	0 (0-0)	1 (1-2)	0 (0-0)	1 (1-2)	0 (0-0)	3 (1-5)	0 (0-0)	3 (1-6)	0 (0-0)
2000	Lebanon	6897 (5951-8199)		2298 (1983-2732)		2715 (2034-3097)		905 (678-1032)		8 (3-19)		3 (1-6)		9 (3-21)		3 (1-7)		24 (8-56)		8 (3-19)	
2001	Lebanon									7 (2-16)		2 (1-5)		8 (3-19)		3 (1-6)		21 (7-50)		7 (2-16)	
2002	Lebanon									6 (2-15)		2 (1-5)		7 (3-17)		2 (1-6)		19 (7-46)		6 (2-15)	
2003	Lebanon									11 (4-25)		3 (1-8)		12 (4-29)		4 (1-9)		32 (11-76)		10 (4-24)	
2004	Lebanon									11 (4-25)		3 (1-8)		12 (4-28)		4 (1-9)		32 (11-74)		10 (3-23)	
2005	Lebanon									9 (3-22)		3 (1-7)		11 (4-25)		3 (1-8)		29 (10-66)		9 (3-21)	
2006	Lebanon									8 (3-19)		3 (1-6)		9 (3-22)		3 (1-7)		25 (9-57)		8 (3-18)	
2007	Lebanon									7 (3-16)		2 (1-5)		8 (3-18)		3 (1-6)		21 (8-47)		7 (3-16)	
2008	Lebanon									6 (2-14)		2 (1-5)		7 (3-16)		3 (1-6)		18 (7-41)		7 (2-15)	
2009	Lebanon									5 (2-12)		2 (1-5)		6 (2-14)		2 (1-5)		16 (6-36)		6 (2-14)	
2010	Lebanon									5 (2-11)		2 (1-4)		6 (2-13)		2 (1-5)		15 (6-34)		6 (2-13)	
2011	Lebanon									5 (2-12)		2 (1-4)		6 (2-14)		2 (1-5)		16 (6-36)		6 (2-12)	
2012	Lebanon									6 (2-13)		2 (1-4)		7 (3-15)		2 (1-5)		18 (7-39)		5 (2-12)	
2013	Lebanon									6 (2-14)		2 (1-4)		7 (3-16)		2 (1-4)		19 (7-43)		5 (2-11)	
2014	Lebanon									7 (3-15)		2 (1-4)		8 (3-17)		2 (1-4)		21 (8-46)		5 (2-11)	
2015	Lebanon	4868 (4200-5787)	3 (2-3)	1074 (926-1276)	1 (0-1)	1895 (1420-2161)	1 (1-1)	418 (313-477)	0 (0-0)	7 (3-15)	0 (0-0)	1 (1-3)	0 (0-0)	8 (3-17)	0 (0-0)	2 (1-4)	0 (0-0)	20 (8-46)	0 (0-0)	5 (2-10)	0 (0-0)

Spn = *Streptococcus pneumoniae*; npnm = non-pneumonia, non-meningitis; HIV = cases only in HIV-infected children; Uncertainty range provided in parentheses.

Year	Country	Spn pneumonia cases (UR)	Spn pneumonia cases, HIV (UR)	Spn pneumonia incidence rate (UR)	Spn pneumonia incidence rate, HIV (UR)	Spn severe pneumonia cases (UR)	Spn severe pneumonia cases, HIV (UR)	Spn severe pneumonia incidence rate (UR)	Spn severe pneumonia incidence rate, HIV (UR)	Spn meningitis cases (UR)	Spn meningitis cases, HIV (UR)	Spn meningitis incidence rate (UR)	Spn meningitis incidence rate, HIV (UR)	Spn NPNM cases (UR)	Spn NPNM cases, HIV (UR)	Spn NPNM incidence rate (UR)	Spn NPNM incidence rate, HIV (UR)	Spn NPNM non-severe cases (UR)	Spn NPNM non-severe cases, HIV (UR)	SPN NPNM non-severe incidence rate (UR)	SPN NPNM non-severe incidence rate, HIV (UR)
2000	Lesotho	7727 (6667-9186)		2912 (2513-3462)		3029 (2269-3454)		1141 (855-1302)		84 (34-175)		32 (13-66)		34 (14-70)		13 (5-27)		253 (103-529)		95 (39-200)	
2001	Lesotho									86 (36-179)		32 (13-67)		35 (14-72)		13 (5-27)		260 (108-540)		98 (41-204)	
2002	Lesotho									88 (38-182)		34 (14-69)		35 (15-73)		14 (6-28)		267 (113-549)		102 (43-210)	
2003	Lesotho									90 (39-184)		35 (15-72)		36 (16-74)		14 (6-29)		273 (119-557)		106 (46-216)	
2004	Lesotho									93 (41-187)		37 (16-74)		37 (17-75)		15 (7-30)		280 (124-565)		111 (49-224)	
2005	Lesotho									95 (43-191)		38 (17-77)		38 (17-77)		15 (7-31)		288 (129-576)		115 (52-231)	
2006	Lesotho									98 (44-195)		40 (18-80)		39 (18-78)		16 (7-32)		296 (134-588)		121 (55-240)	
2007	Lesotho									100 (46-197)		41 (19-81)		40 (18-79)		16 (8-33)		302 (138-595)		124 (57-245)	
2008	Lesotho									102 (47-200)		42 (19-82)		41 (19-80)		17 (8-33)		308 (143-604)		126 (58-247)	
2009	Lesotho									102 (48-199)		41 (19-80)		41 (19-80)		17 (8-32)		308 (145-601)		125 (59-243)	
2010	Lesotho									101 (47-198)		40 (19-79)		41 (19-79)		16 (8-32)		306 (143-597)		122 (57-238)	
2011	Lesotho									100 (47-196)		39 (18-77)		40 (19-79)		16 (7-31)		303 (141-592)		119 (55-232)	
2012	Lesotho									100 (46-195)		38 (18-75)		40 (18-78)		15 (7-30)		301 (139-589)		115 (53-226)	
2013	Lesotho									98 (45-193)		37 (17-73)		39 (18-78)		15 (7-29)		297 (137-584)		112 (52-220)	
2014	Lesotho									97 (44-191)		36 (16-71)		39 (18-76)		14 (7-28)		293 (134-576)		108 (50-213)	
2015	Lesotho	4865 (4198-5784)	2446 (2110-2907)	1777 (1533-2113)	893 (771-1062)	1906 (1428-2174)	958 (718-1093)	696 (522-794)	350 (262-399)	84 (38-165)	42 (19-83)	31 (14-60)	15 (7-30)	34 (15-66)	17 (8-33)	12 (6-24)	6 (3-12)	253 (115-498)	127 (58-250)	92 (42-182)	46 (21-91)
2000	Liberia	9045 (7805-10752)		1853 (1599-2203)		3513 (2632-4007)		720 (539-821)		431 (273-691)		88 (56-142)		173 (109-277)		35 (22-57)		1301 (823-2086)		267 (169-427)	
2001	Liberia									436 (274-707)		85 (53-138)		175 (110-284)		34 (21-55)		1317 (827-2135)		256 (161-416)	
2002	Liberia									433 (271-712)		82 (51-135)		174 (109-286)		33 (21-54)		1309 (819-2151)		247 (155-407)	
2003	Liberia									435 (208-810)		81 (39-151)		174 (83-325)		33 (16-61)		1313 (628-2447)		245 (117-457)	
2004	Liberia									422 (200-802)		78 (37-148)		169 (80-322)		31 (15-59)		1273 (603-2420)		235 (111-446)	
2005	Liberia									407 (191-787)		73 (34-142)		163 (77-316)		29 (14-57)		1229 (576-2377)		221 (104-428)	
2006	Liberia									387 (179-759)		68 (31-133)		155 (72-305)		27 (13-53)		1169 (541-2293)		204 (95-401)	
2007	Liberia									366 (166-725)		62 (28-123)		147 (67-291)		25 (11-49)		1105 (502-2189)		187 (85-371)	
2008	Liberia									350 (156-700)		57 (26-114)		140 (63-281)		23 (10-46)		1056 (472-2112)		173 (77-346)	
2009	Liberia									331 (146-668)		52 (23-106)		133 (58-268)		21 (9-43)		999 (440-2018)		159 (70-320)	
2010	Liberia									308 (133-627)		48 (21-97)		123 (53-252)		19 (8-39)		929 (403-1895)		144 (63-294)	
2011	Liberia									286 (122-589)		44 (19-90)		115 (49-236)		17 (7-36)		864 (369-1777)		132 (56-271)	
2012	Liberia									268 (113-557)		40 (17-84)		107 (45-223)		16 (7-34)		809 (341-1682)		121 (51-252)	
2013	Liberia									251 (104-527)		37 (15-78)		101 (42-211)		15 (6-31)		759 (315-1592)		113 (47-236)	
2014	Liberia									195 (67-424)		29 (10-62)		221 (76-482)		32 (11-71)		588 (202-1281)		86 (30-188)	
2015	Liberia	8155 (7036-9694)	452 (390-537)	1183 (1021-1406)	66 (57-78)	3154 (2363-3597)	175 (131-199)	457 (343-522)	25 (19-29)	167 (56-366)	9 (3-20)	24 (8-53)	1 (0-3)	189 (64-415)	10 (4-23)	27 (9-60)	2 (1-3)	503 (170-1104)	28 (9-61)	73 (25-160)	4 (1-9)
2000	Libya	12401 (10700-14741)		2271 (1960-2700)		4879 (3656-5565)		894 (670-1019)		107 (68-144)		20 (12-26)		122 (77-164)		22 (14-30)		323 (204-436)		59 (37-80)	
2001	Libya									103 (65-139)		18 (12-25)		117 (74-158)		21 (13-28)		310 (196-419)		56 (35-75)	
2002	Libya									99 (62-133)		18 (11-24)		112 (71-151)		20 (13-27)		298 (188-402)		53 (33-71)	
2003	Libya									93 (58-125)		16 (10-22)		105 (66-142)		19 (12-25)		280 (176-377)		49 (31-67)	
2004	Libya									87 (55-117)		15 (10-21)		99 (62-133)		17 (11-23)		262 (166-354)		46 (29-62)	
2005	Libya									81 (51-109)		14 (9-19)		92 (58-124)		16 (10-21)		245 (155-330)		42 (27-57)	
2006	Libya									75 (48-102)		13 (8-17)		85 (54-115)		14 (9-19)		227 (143-307)		38 (24-51)	
2007	Libya									103 (65-139)		17 (11-23)		117 (74-158)		19 (12-26)		311 (196-420)		50 (32-68)	
2008	Libya									110 (69-148)		17 (11-23)		125 (79-168)		20 (12-26)		331 (209-447)		52 (33-70)	
2009	Libya									101 (63-136)		15 (10-21)		114 (72-154)		17 (11-24)		304 (192-410)		46 (29-63)	
2010	Libya									93 (58-125)		14 (9-19)		105 (66-142)		16 (10-21)		279 (176-377)		42 (27-57)	
2011	Libya									86 (55-117)		13 (8-17)		98 (62-132)		15 (9-20)		261 (165-352)		39 (24-52)	
2012	Libya									79 (50-106)		12 (7-16)		89 (56-120)		13 (8-18)		237 (150-320)		35 (22-48)	
2013	Libya									70 (44-95)		11 (7-14)		80 (51-108)		12 (8-16)		213 (134-287)		32 (20-44)	
2014	Libya									57 (36-77)		9 (6-12)		65 (41-88)		10 (6-14)		173 (109-233)		27 (17-36)	
2015	Libya	5697 (4915-6772)	0 (0-0)	893 (770-1061)	0 (0-0)	2218 (1662-2530)	0 (0-0)	348 (260-396)	0 (0-0)	51 (32-69)	0 (0-0)	8 (5-11)	0 (0-0)	58 (36-78)	0 (0-0)	9 (6-12)	0 (0-0)	153 (97-207)	0 (0-0)	24 (15-32)	0 (0-0)
2000	Lithuania	844 (728-1003)		460 (397-547)		547 (410-624)		298 (223-340)		21 (9-37)		11 (5-20)		23 (11-42)		13 (6-23)		62 (28-113)		34 (15-61)	
2001	Lithuania									22 (10-40)		13 (6-23)		25 (11-46)		14 (7-26)		67 (31-122)		38 (17-70)	
2002	Lithuania									22 (10-40)		13 (6-24)		25 (11-45)		15 (7-27)		66 (30-120)		40 (18-72)	
2003	Lithuania									27 (12-49)		17 (8-31)		31 (14-56)		19 (9-35)		82 (37-148)		52 (23-94)	
2004	Lithuania									7 (3-13)		5 (2-9)		8 (4-15)		5 (2-10)		21 (10-39)		14 (6-26)	
2005	Lithuania									18 (8-34)		13 (6-23)		21 (10-38)		14 (7-26)		56 (25-101)		39 (17-70)	
2006	Lithuania									21 (10-39)		15 (7-27)		24 (11-44)		17 (8-31)		65 (29-117)		45 (20-82)	
2007	Lithuania									14 (6-25)		10 (4-18)		16 (7-29)		11 (5-20)		42 (19-76)		29 (13-53)	
2008	Lithuania									12 (5-22)		8 (4-15)		14 (6-25)		9 (4-17)		36 (16-66)		25 (11-46)	
2009	Lithuania									7 (3-13)		5 (2-9)		8 (4-15)		6 (3-10)		22 (10-39)		15 (7-27)	
2010	Lithuania									25 (11-46)		17 (8-32)		29 (13-52)		20 (9-36)		76 (35-139)		53 (24-96)	
2011	Lithuania									16 (7-29)		11 (5-20)		18 (8-33)		12 (6-22)		48 (22-87)		33 (15-59)	
2012	Lithuania									17 (8-31)		11 (5-21)		19 (9-35)		13 (6-23)		51 (23-93)		34 (16-62)	
2013	Lithuania									17 (7-30)		11 (5-20)		19 (9-34)		13 (6-23)		50 (23-91)		33 (15-61)	
2014	Lithuania									16 (7-30)		11 (5-20)		19 (8-34)		12 (6-23)		49 (22-89)		33 (15-60)	
2015	Lithuania	687 (593-817)	0 (0-0)	460 (397-547)	0 (0-0)	445 (334-508)	0 (0-0)	298 (223-340)	0 (0-0)	17 (8-30)	0 (0-0)	11 (5-20)	0 (0-0)	19 (9-34)	0 (0-0)	13 (6-23)	0 (0-0)	50 (23-92)	0 (0-0)	34 (15-61)	0 (0-0)
2000	Luxembourg	32 (28-38)		115 (99-137)		21 (16-24)		75 (56-85)		5 (2-9)		18 (8-33)		6 (3-11)		21 (9-38)		16 (7-28)		56 (25-101)	
2001	Luxembourg									4 (2-7)		13 (6-24)		4 (2-7)		15 (7-27)		11 (5-20)		40 (18-72)	
2002	Luxembourg									1 (1-2)		5 (2-9)		2 (1-3)		6 (3-10)		4 (2-7)		15 (7-27)	
2003	Luxembourg									0 (0-0)		0 (0-0)		0 (0-0)		0 (0-0)		0 (0-0)		0 (0-0)	
2004	Luxembourg									0 (0-0)		0 (0-0)		0 (0-0)		0 (0-0)		0 (0-0)		0 (0-0)	
2005	Luxembourg									0 (0-0)		0 (0-0)		0 (0-0)		0 (0-0)		0 (0-0)		0 (0-0)	
2006	Luxembourg									0 (0-0)		0 (0-0)		0 (0-0)		0 (0-0)		0 (0-0)		0 (0-0)	
2007	Luxembourg									0 (0-0)		0 (0-0)		0 (0-0)		0 (0-0)		0 (0-0)		0 (0-0)	
2008	Luxembourg									1 (0-1)		3 (1-5)		1 (0-1)		3 (1-5)		2 (1-4)		8 (4-14)	
2009	Luxembourg									1 (1-2)		4 (2-7)		1 (1-2)		5 (2-8)		3 (2-6)		12 (6-22)	
2010	Luxembourg									1 (0-1)		3 (1-5)		1 (0-2)		3 (1-6)		2 (1-4)		8 (4-15)	
2011	Luxembourg									0 (0-0)		1 (0-1)		0 (0-0)		1 (0-2)		1 (0-1)		2 (1-4)	
2012	Luxembourg									0 (0-0)		1 (0-1)		0 (0-0)		1 (0-1)		0 (0-1)		2 (1-3)	
2013	Luxembourg									0 (0-0)		0 (0-0)		0 (0-0)		0 (0-0)		0 (0-0)		1 (0-1)	
2014	Luxembourg									0 (0-0)		0 (0-1)		0 (0-0)		0 (0-1)		0 (0-0)		1 (0-2)	
2015	Luxembourg	6 (5-7)	0 (0-0)	19 (16-22)	0 (0-0)	4 (3-4)	0 (0-0)	12 (9-14)	0 (0-0)	0 (0-0)	0 (0-0)	1 (0-1)	0 (0-0)	0 (0-0)	0 (0-0)	1 (0-1)	0 (0-0)	0 (0-1)	0 (0-0)	2 (1-3)	0 (0-0)
2000	Madagascar	97359 (84004-115733)		3484 (3006-4142)		38261 (28665-43640)		1369 (1026-1562)		1310 (526-2779)		47 (19-99)		525 (211-1115)		19 (8-40)		3955 (1587-8391)		142 (57-300)	
2001	Madagascar									1269 (511-2690)		44 (18-94)		509 (205-1079)		18 (7-38)		3832 (1542-8122)		134 (54-283)	
2002	Madagascar									1221 (493-2586)		42 (17-88)		490 (198-1037)		17 (7-35)		3686 (1488-7808)		126 (51-267)	
2003	Madagascar									1162 (471-2460)		39 (16-83)		466 (189-987)		16 (6-33)		3509 (1422-7429)		118 (48-250)	
2004	Madagascar									1095 (445-2323)		36 (15-77)		439 (178-932)		15 (6-31)		3308 (1342-7014)		110 (45-233)	
2005	Madagascar									1031 (413-2199)		34 (14-72)		414 (166-882)		14 (5-29)		3114 (1249-6642)		102 (41-218)	
2006	Madagascar									964 (382-2070)		31 (12-67)		387 (153-830)		12 (5-27)		2911 (1155-6251)		94 (37-202)	
2007	Madagascar									912 (299-2031)		29 (10-65)		1035 (339-2307)		33 (11-73)		2752 (903-6134)		88 (29-195)	
2008	Madagascar									868 (281-1948)		27 (9-61)		986 (319-2212)		31 (10-69)		2622 (849-5882)		82 (27-184)	
2009	Madagascar									841 (273-1890)		26 (8-58)		955 (310-2147)		29 (10-66)		2538 (824-5708)		78 (25-175)	
2010	Madagascar									814 (263-1835)		24 (8-55)		925 (299-2084)		28 (9-63)		2458 (795-5541)		74 (24-167)	
2011	Madagascar									797 (256-1799)		23 (8-53)		905 (291-2043)		27 (9-60)		2406 (774-5432)		71 (23-160)	
2012	Madagascar									782 (250-1767)		23 (7-51)		888 (284-2007)		26 (8-58)		2361 (756-5336)		68 (22-154)	
2013	Madagascar									524 (168-1185)		15 (5-33)		595 (191-1346)		17 (5-38)		1583 (507-3578)		45 (14-101)	
2014	Madagascar									468 (151-1057)		13 (4-29)		532 (171-1200)		15 (5-33)		1414 (455-3192)		39 (13-88)	
2015	Madagascar	33711 (29087-40073)	538 (464-640)	909 (785-1081)	15 (13-17)	13192 (9883-15047)	211 (158-240)	356 (267-406)	6 (4-6)	450 (146-1014)	7 (2-16)	12 (4-27)	0 (0-0)	511 (166-1152)	8 (3-18)	14 (4-31)	0 (0-0)	1359 (440-3063)	22 (7-49)	37 (12-83)	1 (0-1)
2000	Malawi	61122 (52738-72657)		3043 (2626-3617)		23891 (17899-27250)		1189 (891-1357)		1249 (646-2433)		62 (32-121)		501 (259-976)		25 (13-49)		3772 (1951-7346)		188 (97-366)	
2001	Malawi									1210 (636-2352)		59 (31-114)		485 (255-943)		24 (12-46)		3654 (1920-7102)		177 (93-344)	
2002	Malawi									1151 (410-2557)		55 (19-121)		462 (164-1026)		22 (8-49)		3476 (1238-7722)		165 (59-366)	
2003	Malawi									1095 (398-2417)		51 (18-112)		439 (159-969)		20 (7-45)		3305 (1201-7298)		154 (56-339)	
2004	Malawi									1064 (394-2330)		48 (18-106)		427 (158-934)		19 (7-43)		3214 (1188-7035)		146 (54-320)	
2005	Malawi									1055 (415-2259)		47 (18-100)		423 (166-906)		19 (7-40)		3185 (1253-6821)		141 (56-303)	
2006	Malawi									1090 (457-2275)		47 (20-98)		437 (183-913)		19 (8-39)		3292 (1381-6870)		142 (60-297)	
2007	Malawi									1114 (484-2273)		47 (20-95)		447 (194-912)		19 (8-38)		3363 (1463-6862)		141 (61-288)	
2008	Malawi									1144 (517-2281)		46 (21-93)		459 (207-915)		19 (8-37)		3455 (1562-6887)		140 (63-280)	
2009	Malawi									1193 (563-2326)		47 (22-92)		479 (226-933)		19 (9-37)		3603 (1701-7024)		142 (67-277)	
2010	Malawi									1181 (586-2258)		45 (22-86)		474 (235-906)		18 (9-35)		3566 (1770-6817)		137 (68-261)	
2011	Malawi									1249 (613-2386)		46 (23-89)		501 (246-957)		19 (9-36)		3771 (1850-7205)		140 (69-268)	
2012	Malawi									726 (353-1391)		26 (13-51)		291 (142-558)		11 (5-20)		2192 (1067-4201)		80 (39-153)	
2013	Malawi									632 (269-1251)		23 (10-45)		717 (305-1421)		26 (11-51)		1907 (811-3778)		68 (29-135)	
2014	Malawi									596 (254-1184)		21 (9-42)		677 (289-1345)		24 (10-47)		1799 (768-3576)		63 (27-126)	
2015	Malawi	22218 (19171-26411)	4807 (4148-5715)	765 (660-909)	166 (143-197)	8724 (6536-9950)	1888 (1414-2153)	300 (225-343)	65 (49-74)	550 (238-1094)	119 (51-237)	19 (8-38)	4 (2-8)	625 (270-1243)	135 (58-269)	22 (9-43)	5 (2-9)	1661 (718-3304)	359 (155-715)	57 (25-114)	12 (5-25)
2000	Malaysia	95188 (82130-113151)		3558 (3070-4229)		36621 (27436-41769)		1369 (1025-1561)		304 (182-419)		11 (7-16)		345 (207-476)		13 (8-18)		917 (551-1265)		34 (21-47)	
2001	Malaysia									264 (159-364)		10 (6-14)		300 (180-414)		11 (7-16)		798 (479-1100)		30 (18-41)	
2002	Malaysia									240 (144-331)		9 (5-13)		273 (164-376)		10 (6-14)		725 (435-1000)		28 (17-38)	
2003	Malaysia									217 (130-299)		8 (5-12)		246 (148-340)		10 (6-13)		655 (393-903)		25 (15-35)	
2004	Malaysia									309 (185-426)		12 (7-17)		351 (210-484)		14 (8-19)		932 (559-1286)		37 (22-51)	
2005	Malaysia									313 (188-432)		13 (8-17)		356 (214-491)		14 (9-20)		946 (568-1305)		38 (23-53)	
2006	Malaysia									290 (174-400)		12 (7-16)		330 (198-455)		13 (8-19)		876 (526-1209)		36 (22-49)	
2007	Malaysia									259 (156-358)		11 (7-15)		295 (177-406)		12 (7-17)		783 (470-1080)		33 (20-45)	
2008	Malaysia									242 (145-333)		10 (6-14)		274 (165-378)		12 (7-16)		729 (438-1006)		31 (19-43)	
2009	Malaysia									245 (147-338)		11 (6-15)		278 (167-384)		12 (7-17)		740 (444-1020)		32 (19-44)	
2010	Malaysia									242 (145-334)		11 (6-15)		275 (165-379)		12 (7-17)		731 (439-1008)		32 (19-44)	
2011	Malaysia									241 (145-333)		11 (6-15)		274 (164-378)		12 (7-17)		728 (437-1005)		33 (20-45)	
2012	Malaysia									237 (142-327)		11 (6-14)		270 (162-372)		12 (7-16)		717 (430-988)		32 (19-44)	
2013	Malaysia									229 (137-316)		10 (6-14)		260 (156-359)		11 (7-15)		691 (415-953)		30 (18-41)	
2014	Malaysia									217 (130-299)		9 (5-13)		246 (148-340)		10 (6-14)		655 (393-903)		27 (16-38)	
2015	Malaysia	28350 (24461-33700)	16 (14-19)	1164 (1004-1384)	1 (1-1)	11070 (8293-12626)	6 (5-7)	455 (341-518)	0 (0-0)	209 (126-288)	0 (0-0)	9 (5-12)	0 (0-0)	238 (143-328)	0 (0-0)	10 (6-13)	0 (0-0)	632 (379-871)	0 (0-0)	26 (16-36)	0 (0-0)
2000	Maldives	537 (464-639)		1656 (1429-1969)		210 (158-240)		648 (486-739)		1 (0-3)		4 (1-10)		1 (0-4)		4 (1-11)		4 (1-10)		12 (3-30)	
2001	Maldives									1 (0-3)		3 (1-8)		1 (0-3)		4 (1-10)		3 (1-8)		10 (3-25)	
2002	Maldives									1 (0-2)		3 (1-7)		1 (0-2)		3 (1-8)		3 (1-6)		9 (2-21)	
2003	Maldives									1 (0-2)		3 (0-7)		1 (0-2)		3 (0-8)		2 (0-6)		8 (1-22)	
2004	Maldives									1 (0-2)		2 (0-6)		1 (0-2)		2 (0-7)		2 (0-6)		7 (1-19)	
2005	Maldives									1 (0-2)		2 (0-5)		1 (0-2)		2 (0-6)		2 (0-5)		6 (1-17)	
2006	Maldives									1 (0-1)		2 (0-5)		1 (0-2)		2 (0-6)		2 (0-4)		5 (1-15)	
2007	Maldives									0 (0-1)		1 (0-4)		1 (0-1)		2 (0-5)		1 (0-4)		4 (1-13)	
2008	Maldives									0 (0-1)		1 (0-4)		0 (0-1)		1 (0-4)		1 (0-3)		4 (1-11)	
2009	Maldives									0 (0-1)		1 (0-3)		0 (0-1)		1 (0-4)		1 (0-3)		4 (1-10)	
2010	Maldives									0 (0-1)		1 (0-3)		0 (0-1)		1 (0-3)		1 (0-3)		3 (1-9)	
2011	Maldives									0 (0-1)		1 (0-2)		0 (0-1)		1 (0-3)		1 (0-2)		3 (0-7)	
2012	Maldives									0 (0-1)		1 (0-2)		0 (0-1)		1 (0-2)		1 (0-2)		2 (0-6)	
2013	Maldives									0 (0-1)		1 (0-3)		0 (0-1)		1 (0-4)		1 (0-3)		3 (1-9)	
2014	Maldives									0 (0-1)		1 (0-4)		1 (0-1)		1 (0-4)		1 (0-4)		4 (1-11)	
2015	Maldives	453 (391-539)	0 (0-0)	1233 (1064-1466)	1 (0-1)	178 (133-203)	0 (0-0)	484 (363-552)	0 (0-0)	0 (0-1)	0 (0-0)	1 (0-3)	0 (0-0)	0 (0-1)	0 (0-0)	1 (0-4)	0 (0-0)	1 (0-4)	0 (0-0)	3 (1-10)	0 (0-0)
2000	Mali	52538 (45331-62453)		2565 (2213-3049)		20599 (15433-23495)		1006 (753-1147)		1324 (707-2503)		65 (35-122)		531 (284-1004)		26 (14-49)		3999 (2135-7557)		195 (104-369)	
2001	Mali									1314 (703-2481)		62 (33-117)		527 (282-995)		25 (13-47)		3968 (2123-7491)		187 (100-354)	
2002	Mali									1284 (688-2422)		59 (31-111)		515 (276-971)		24 (13-44)		3878 (2079-7313)		177 (95-334)	
2003	Mali									1219 (654-2295)		54 (29-102)		489 (262-921)		22 (12-41)		3680 (1976-6931)		163 (88-307)	
2004	Mali									1189 (639-2236)		51 (27-96)		477 (256-897)		20 (11-39)		3590 (1931-6752)		154 (83-290)	
2005	Mali									1190 (641-2237)		49 (27-93)		477 (257-897)		20 (11-37)		3592 (1937-6754)		149 (80-280)	
2006	Mali									1230 (666-2316)		49 (27-92)		493 (267-929)		20 (11-37)		3715 (2011-6993)		148 (80-278)	
2007	Mali									1325 (724-2498)		51 (28-96)		531 (290-1002)		20 (11-38)		4000 (2185-7543)		154 (84-289)	
2008	Mali									1359 (512-2935)		50 (19-109)		545 (205-1177)		20 (8-44)		4105 (1545-8864)		152 (57-329)	
2009	Mali									1432 (545-3096)		51 (20-111)		574 (219-1242)		21 (8-45)		4323 (1647-9348)		155 (59-336)	
2010	Mali									1433 (552-3097)		50 (19-108)		575 (221-1242)		20 (8-43)		4326 (1666-9353)		151 (58-327)	
2011	Mali									1078 (419-2328)		36 (14-79)		433 (168-934)		15 (6-32)		3256 (1265-7028)		110 (43-237)	
2012	Mali									860 (336-1849)		28 (11-61)		345 (135-742)		11 (4-24)		2596 (1016-5584)		86 (33-184)	
2013	Mali									763 (301-1636)		25 (10-53)		306 (121-656)		10 (4-21)		2304 (909-4939)		74 (29-160)	
2014	Mali									676 (269-1445)		21 (9-46)		271 (108-580)		9 (3-18)		2042 (812-4365)		65 (26-138)	
2015	Mali	40950 (35332-48678)	3476 (2999-4132)	1273 (1098-1513)	108 (93-128)	16003 (11990-18253)	1358 (1018-1549)	498 (373-567)	42 (32-48)	737 (296-1571)	63 (25-133)	23 (9-49)	2 (1-4)	296 (119-630)	25 (10-53)	9 (4-20)	1 (0-2)	2226 (892-4744)	189 (76-403)	69 (28-147)	6 (2-13)
2000	Malta	28 (24-33)		115 (99-137)		18 (13-20)		75 (56-85)		5 (2-10)		23 (10-41)		6 (3-11)		26 (12-47)		16 (7-30)		68 (31-124)	
2001	Malta									4 (2-6)		15 (7-28)		4 (2-7)		17 (8-31)		11 (5-20)		46 (21-84)	
2002	Malta									0 (0-0)		0 (0-0)		0 (0-0)		0 (0-0)		0 (0-0)		0 (0-0)	
2003	Malta									0 (0-0)		0 (0-0)		0 (0-0)		0 (0-0)		0 (0-0)		0 (0-0)	
2004	Malta									1 (0-2)		4 (2-8)		1 (0-2)		5 (2-9)		3 (1-5)		13 (6-23)	
2005	Malta									1 (0-1)		4 (2-8)		1 (0-2)		5 (2-9)		2 (1-4)		13 (6-23)	
2006	Malta									1 (0-2)		5 (2-8)		1 (0-2)		5 (2-9)		3 (1-5)		14 (6-25)	
2007	Malta									0 (0-0)		0 (0-0)		0 (0-0)		0 (0-0)		0 (0-0)		0 (0-0)	
2008	Malta									1 (1-2)		6 (3-12)		1 (1-2)		7 (3-13)		4 (2-6)		19 (9-35)	

Spn = *Streptococcus pneumoniae*; npnm = non-pneumonia, non-meningitis; HIV = cases only in HIV-infected children; Uncertainty range provided in parentheses.

Year	Country	Spn pneumonia cases (UR)	Spn pneumonia cases, HIV (UR)	Spn pneumonia incidence rate (UR)	Spn pneumonia incidence rate, HIV (UR)	Spn severe pneumonia cases (UR)	Spn severe pneumonia cases, HIV (UR)	Spn severe pneumonia incidence rate (UR)	Spn severe pneumonia incidence rate, HIV (UR)	Spn meningitis cases (UR)	Spn meningitis cases, HIV (UR)	Spn meningitis incidence rate (UR)	Spn meningitis incidence rate, HIV (UR)	Spn NPNM cases (UR)	Spn NPNM cases, HIV (UR)	Spn NPNM incidence rate (UR)	Spn NPNM incidence rate, HIV (UR)	Spn NPNM non-severe cases (UR)	Spn NPNM non-severe cases, HIV (UR)	SPN NPNM non-severe incidence rate (UR)	SPN NPNM non-severe incidence rate, HIV (UR)
2009	Malta									2 (1-3)		9 (4-16)		2 (1-3)		10 (5-18)		5 (2-9)		27 (12-48)	
2010	Malta									4 (2-7)		21 (9-37)		4 (2-8)		23 (11-43)		12 (5-22)		62 (28-113)	
2011	Malta									3 (1-5)		15 (7-27)		3 (1-6)		17 (8-31)		9 (4-16)		45 (21-82)	
2012	Malta									4 (2-6)		19 (9-34)		4 (2-7)		21 (10-39)		11 (5-20)		57 (26-103)	
2013	Malta									2 (1-4)		11 (5-20)		2 (1-4)		12 (6-22)		6 (3-11)		33 (15-59)	
2014	Malta									3 (1-6)		16 (7-30)		3 (2-6)		19 (8-34)		9 (4-17)		50 (23-90)	
2015	Malta	21 (18-25)	0 (0-0)	115 (99-137)	0 (0-0)	14 (10-16)	0 (0-0)	75 (56-85)	0 (0-0)	3 (1-6)	0 (0-0)	17 (8-30)	0 (0-0)	3 (2-6)	0 (0-0)	19 (9-34)	0 (0-0)	9 (4-17)	0 (0-0)	50 (23-91)	0 (0-0)
2000	Marshall Islands	122 (105-145)		1110 (958-1320)		48 (36-55)		435 (326-496)		1 (0-2)		6 (2-18)		1 (0-2)		6 (2-21)		2 (1-6)		17 (5-55)	
2001	Marshall Islands									1 (0-2)		6 (2-18)		1 (0-2)		6 (2-20)		2 (1-6)		17 (5-54)	
2002	Marshall Islands									1 (0-2)		6 (2-18)		1 (0-2)		6 (2-21)		2 (1-6)		17 (5-55)	
2003	Marshall Islands									1 (0-2)		6 (2-18)		1 (0-2)		6 (2-20)		2 (0-6)		17 (5-54)	
2004	Marshall Islands									1 (0-2)		6 (2-18)		1 (0-2)		6 (2-21)		2 (0-5)		17 (5-56)	
2005	Marshall Islands									1 (0-2)		6 (2-18)		1 (0-2)		6 (2-21)		2 (0-5)		17 (5-55)	
2006	Marshall Islands									1 (0-2)		6 (2-19)		1 (0-2)		7 (2-21)		2 (0-5)		18 (5-57)	
2007	Marshall Islands									1 (0-2)		6 (2-19)		1 (0-2)		7 (2-22)		2 (0-5)		18 (5-58)	
2008	Marshall Islands									0 (0-2)		6 (2-19)		1 (0-2)		7 (2-21)		1 (0-5)		18 (5-57)	
2009	Marshall Islands									0 (0-1)		5 (1-16)		0 (0-1)		6 (2-18)		1 (0-4)		15 (4-49)	
2010	Marshall Islands									0 (0-1)		5 (1-16)		0 (0-1)		6 (2-19)		1 (0-4)		15 (4-50)	
2011	Marshall Islands									0 (0-1)		5 (1-15)		0 (0-1)		5 (2-17)		1 (0-3)		14 (4-46)	
2012	Marshall Islands									0 (0-1)		4 (1-14)		0 (0-1)		5 (1-16)		1 (0-3)		13 (4-42)	
2013	Marshall Islands									0 (0-1)		4 (1-14)		0 (0-1)		5 (1-16)		1 (0-3)		13 (4-42)	
2014	Marshall Islands									0 (0-1)		4 (1-14)		0 (0-1)		5 (1-16)		1 (0-3)		13 (4-43)	
2015	Marshall Islands	53 (46-63)	0 (0-0)	801 (691-952)	0 (0-0)	21 (16-24)	0 (0-0)	315 (236-359)	0 (0-0)	0 (0-0)	0 (0-0)	2 (1-7)	0 (0-0)	0 (0-1)	0 (0-0)	2 (1-8)	0 (0-0)	0 (0-1)	0 (0-0)	6 (2-20)	0 (0-0)
2000	Mauritania	10812 (9329-12852)		2468 (2130-2934)		4247 (3182-4844)		970 (726-1106)		145 (55-314)		33 (13-72)		58 (22-126)		13 (5-29)		437 (167-949)		100 (38-217)	
2001	Mauritania									151 (58-327)		34 (13-73)		61 (23-131)		13 (5-29)		456 (175-989)		101 (39-220)	
2002	Mauritania									158 (61-341)		34 (13-74)		63 (24-137)		14 (5-30)		476 (183-1030)		103 (40-223)	
2003	Mauritania									163 (63-352)		34 (13-75)		65 (25-141)		14 (5-30)		492 (191-1063)		104 (40-225)	
2004	Mauritania									166 (65-358)		34 (13-74)		67 (26-143)		14 (5-30)		501 (195-1080)		104 (40-224)	
2005	Mauritania									167 (65-360)		34 (13-73)		67 (26-144)		14 (5-29)		505 (198-1087)		102 (40-220)	
2006	Mauritania									169 (66-362)		33 (13-72)		68 (27-145)		13 (5-29)		509 (200-1092)		101 (40-216)	
2007	Mauritania									169 (67-362)		33 (13-70)		68 (27-145)		13 (5-28)		510 (202-1093)		99 (39-211)	
2008	Mauritania									168 (66-360)		32 (13-68)		67 (27-145)		13 (5-27)		507 (200-1088)		96 (38-207)	
2009	Mauritania									166 (65-357)		31 (12-67)		67 (26-143)		12 (5-27)		502 (197-1079)		94 (37-201)	
2010	Mauritania									165 (65-355)		30 (12-65)		66 (26-143)		12 (5-26)		499 (195-1073)		92 (36-197)	
2011	Mauritania									164 (64-354)		30 (12-64)		66 (26-142)		12 (5-26)		496 (193-1069)		89 (35-192)	
2012	Mauritania									161 (63-347)		29 (11-61)		65 (25-139)		11 (4-25)		487 (190-1046)		86 (34-185)	
2013	Mauritania									158 (62-339)		28 (11-59)		63 (25-136)		11 (4-24)		477 (187-1024)		83 (33-178)	
2014	Mauritania									108 (43-232)		19 (7-40)		43 (17-93)		7 (3-16)		327 (129-701)		56 (22-120)	
2015	Mauritania	6654 (5741-7910)	334 (288-396)	1126 (971-1338)	56 (49-67)	2611 (1956-2978)	131 (98-149)	442 (331-504)	22 (17-25)	93 (37-199)	5 (2-10)	16 (6-34)	1 (0-2)	37 (15-80)	2 (1-4)	6 (3-14)	0 (0-1)	281 (112-602)	14 (6-30)	48 (19-102)	2 (1-5)
2000	Mauritius	2095 (1808-2490)		2161 (1865-2569)		825 (618-941)		851 (637-970)		20 (12-27)		20 (13-28)		22 (14-30)		23 (15-31)		60 (38-81)		62 (39-83)	
2001	Mauritius									21 (13-29)		23 (15-31)		24 (15-33)		26 (17-35)		64 (41-87)		70 (44-94)	
2002	Mauritius									8 (5-11)		9 (6-12)		9 (6-13)		10 (6-14)		25 (16-33)		27 (17-37)	
2003	Mauritius									13 (8-17)		14 (9-18)		14 (9-19)		15 (10-21)		38 (24-51)		41 (26-55)	
2004	Mauritius									15 (9-20)		16 (10-21)		17 (11-23)		18 (11-24)		45 (28-61)		48 (30-65)	
2005	Mauritius									15 (9-20)		16 (10-21)		17 (11-22)		18 (11-24)		44 (28-60)		47 (30-64)	
2006	Mauritius									46 (29-61)		48 (31-65)		52 (33-70)		55 (35-74)		137 (87-185)		146 (92-197)	
2007	Mauritius									7 (4-10)		8 (5-10)		8 (5-11)		9 (6-12)		21 (14-29)		23 (15-31)	
2008	Mauritius									22 (14-30)		25 (16-34)		25 (16-34)		29 (18-39)		67 (42-90)		77 (48-103)	
2009	Mauritius									19 (12-26)		23 (14-31)		22 (14-29)		26 (16-35)		57 (36-77)		69 (44-93)	
2010	Mauritius									18 (11-24)		23 (14-31)		21 (13-28)		26 (16-35)		55 (34-74)		69 (43-93)	
2011	Mauritius									12 (8-16)		16 (10-22)		14 (9-18)		18 (11-24)		36 (23-49)		48 (30-65)	
2012	Mauritius									0 (0-0)		0 (0-0)		0 (0-0)		0 (0-0)		0 (0-0)		0 (0-0)	
2013	Mauritius									7 (4-10)		10 (6-13)		8 (5-11)		11 (7-15)		21 (13-29)		30 (19-40)	
2014	Mauritius									6 (4-8)		8 (5-11)		7 (4-9)		10 (6-13)		18 (11-24)		25 (16-34)	
2015	Mauritius	1152 (994-1370)	0 (0-0)	1659 (1431-1972)	0 (0-0)	452 (339-516)	0 (0-0)	651 (488-742)	0 (0-0)	6 (4-8)	0 (0-0)	9 (5-12)	0 (0-0)	7 (4-9)	0 (0-0)	10 (6-13)	0 (0-0)	18 (11-24)	0 (0-0)	26 (16-35)	0 (0-0)
2000	Mexico	176451 (152246-209750)		1469 (1267-1746)		68577 (51377-78218)		571 (428-651)		4372 (2147-6775)		36 (18-56)		4966 (2439-7695)		41 (20-64)		13201 (6484-20459)		110 (54-170)	
2001	Mexico									3516 (1727-5449)		29 (14-45)		3994 (1962-6189)		33 (16-52)		10618 (5215-16455)		88 (43-137)	
2002	Mexico									3119 (1532-4833)		26 (13-40)		3542 (1740-5490)		30 (15-46)		9417 (4625-14595)		79 (39-122)	
2003	Mexico									3156 (1550-4892)		27 (13-41)		3585 (1761-5556)		30 (15-47)		9531 (4681-14771)		80 (39-124)	
2004	Mexico									2917 (1433-4521)		25 (12-38)		3313 (1627-5135)		28 (14-43)		8809 (4327-13652)		75 (37-116)	
2005	Mexico									2443 (1200-3786)		21 (10-32)		2774 (1363-4300)		24 (12-37)		7376 (3623-11431)		63 (31-97)	
2006	Mexico									2636 (1295-4085)		23 (11-35)		2994 (1470-4639)		26 (13-40)		7959 (3909-12334)		68 (34-106)	
2007	Mexico									2527 (1241-3916)		22 (11-34)		2870 (1410-4448)		25 (12-38)		7630 (3748-11825)		66 (32-102)	
2008	Mexico									2337 (1148-3622)		20 (10-31)		2655 (1304-4114)		23 (11-36)		7058 (3467-10938)		61 (30-95)	
2009	Mexico									1611 (791-2496)		14 (7-22)		1829 (898-2835)		16 (8-25)		4863 (2388-7537)		42 (21-66)	
2010	Mexico									1277 (627-1979)		11 (5-17)		1451 (713-2248)		13 (6-20)		3857 (1894-5977)		34 (17-52)	
2011	Mexico									928 (456-1439)		8 (4-13)		1054 (518-1634)		9 (5-14)		2803 (1377-4344)		24 (12-38)	
2012	Mexico									676 (332-1047)		6 (3-9)		767 (377-1189)		7 (3-10)		2040 (1002-3162)		18 (9-28)	
2013	Mexico									675 (332-1046)		6 (3-9)		767 (377-1188)		7 (3-10)		2038 (1001-3159)		18 (9-28)	
2014	Mexico									633 (311-980)		6 (3-9)		718 (353-1113)		6 (3-10)		1910 (938-2960)		17 (8-26)	
2015	Mexico	43214 (37286-51369)	0 (0-0)	378 (326-450)	0 (0-0)	16837 (12614-19205)	0 (0-0)	147 (110-168)	0 (0-0)	601 (295-932)	0 (0-0)	5 (3-8)	0 (0-0)	683 (335-1058)	0 (0-0)	6 (3-9)	0 (0-0)	1815 (891-2813)	0 (0-0)	16 (8-25)	0 (0-0)
2000	Micronesia (Federated States of)	293 (253-348)		1984 (1712-2359)		114 (86-130)		775 (581-884)		1 (0-4)		8 (2-26)		1 (0-4)		9 (2-30)		3 (1-12)		24 (6-79)	
2001	Micronesia (Federated States of)									1 (0-4)		8 (2-25)		1 (0-4)		9 (2-29)		3 (1-11)		23 (6-76)	
2002	Micronesia (Federated States of)									1 (0-3)		7 (2-24)		1 (0-4)		8 (2-27)		3 (1-10)		22 (6-73)	
2003	Micronesia (Federated States of)									1 (0-3)		7 (2-24)		1 (0-4)		8 (2-27)		3 (1-10)		22 (6-72)	
2004	Micronesia (Federated States of)									1 (0-3)		7 (2-23)		1 (0-4)		8 (2-26)		3 (1-9)		21 (6-69)	
2005	Micronesia (Federated States of)									1 (0-3)		7 (2-22)		1 (0-3)		8 (2-25)		3 (1-9)		20 (6-68)	
2006	Micronesia (Federated States of)									1 (0-3)		6 (2-21)		1 (0-3)		7 (2-24)		3 (1-8)		20 (5-64)	
2007	Micronesia (Federated States of)									1 (0-3)		6 (2-21)		1 (0-3)		7 (2-23)		2 (1-8)		19 (5-62)	
2008	Micronesia (Federated States of)									1 (0-2)		6 (2-20)		1 (0-3)		7 (2-23)		2 (1-7)		19 (5-61)	
2009	Micronesia (Federated States of)									1 (0-2)		6 (2-19)		1 (0-3)		7 (2-22)		2 (1-7)		18 (5-59)	
2010	Micronesia (Federated States of)									0 (0-2)		4 (1-13)		1 (0-2)		5 (1-15)		1 (0-5)		12 (4-40)	
2011	Micronesia (Federated States of)									0 (0-1)		4 (1-12)		0 (0-2)		4 (1-13)		1 (0-4)		11 (3-35)	
2012	Micronesia (Federated States of)									0 (0-1)		4 (1-11)		0 (0-2)		4 (1-13)		1 (0-4)		11 (3-35)	
2013	Micronesia (Federated States of)									0 (0-1)		3 (1-11)		0 (0-1)		4 (1-12)		1 (0-4)		10 (3-33)	
2014	Micronesia (Federated States of)									0 (0-1)		3 (1-10)		0 (0-1)		4 (1-11)		1 (0-3)		10 (3-30)	
2015	Micronesia (Federated States of)	92 (80-110)	0 (0-0)	812 (701-965)	0 (0-0)	36 (27-41)	0 (0-0)	319 (239-364)	0 (0-0)	0 (0-1)	0 (0-0)	2 (1-6)	0 (0-0)	0 (0-1)	0 (0-0)	2 (1-7)	0 (0-0)	1 (0-2)	0 (0-0)	5 (2-17)	0 (0-0)
2000	Monaco	1 (1-2)		115 (99-137)		1 (1-1)		75 (56-85)		0 (0-0)		0 (0-0)		0 (0-0)		0 (0-0)		0 (0-0)		0 (0-0)	
2001	Monaco									0 (0-0)		0 (0-0)		0 (0-0)		0 (0-0)		0 (0-0)		0 (0-0)	
2002	Monaco									0 (0-0)		0 (0-0)		0 (0-0)		0 (0-0)		0 (0-0)		0 (0-0)	
2003	Monaco									0 (0-0)		4 (2-8)		0 (0-0)		5 (2-9)		0 (0-0)		14 (6-25)	
2004	Monaco									0 (0-0)		4 (2-8)		0 (0-0)		5 (2-9)		0 (0-0)		13 (6-24)	
2005	Monaco									0 (0-0)		0 (0-0)		0 (0-0)		0 (0-0)		0 (0-0)		0 (0-0)	
2006	Monaco									0 (0-0)		0 (0-0)		0 (0-0)		0 (0-0)		0 (0-0)		0 (0-0)	
2007	Monaco									0 (0-0)		0 (0-0)		0 (0-0)		0 (0-0)		0 (0-0)		0 (0-0)	
2008	Monaco									0 (0-0)		8 (4-14)		0 (0-0)		9 (4-16)		0 (0-0)		24 (11-43)	
2009	Monaco									0 (0-0)		10 (5-18)		0 (0-0)		12 (5-21)		0 (0-0)		31 (14-55)	
2010	Monaco									0 (0-0)		9 (4-17)		0 (0-0)		11 (5-19)		0 (0-0)		28 (13-52)	
2011	Monaco									0 (0-0)		6 (3-11)		0 (0-0)		7 (3-12)		0 (0-0)		18 (8-33)	
2012	Monaco									0 (0-0)		0 (0-0)		0 (0-0)		0 (0-0)		0 (0-0)		0 (0-0)	
2013	Monaco									0 (0-0)		0 (0-0)		0 (0-0)		0 (0-0)		0 (0-0)		0 (0-0)	
2014	Monaco									0 (0-0)		2 (1-4)		0 (0-0)		2 (1-4)		0 (0-0)		6 (3-11)	
2015	Monaco	3 (3-4)	0 (0-0)	115 (99-137)	0 (0-0)	2 (2-2)	0 (0-0)	75 (56-85)	0 (0-0)	0 (0-0)	0 (0-0)	2 (1-3)	0 (0-0)	0 (0-0)	0 (0-0)	2 (1-4)	0 (0-0)	0 (0-0)	0 (0-0)	5 (2-10)	0 (0-0)
2000	Mongolia	4564 (3938-5425)		1984 (1712-2358)		1788 (1340-2039)		777 (582-887)		63 (29-123)		27 (13-54)		71 (33-140)		31 (14-61)		190 (88-372)		83 (38-162)	
2001	Mongolia									54 (24-108)		24 (11-47)		61 (27-122)		27 (12-54)		163 (72-325)		72 (32-143)	
2002	Mongolia									46 (19-93)		21 (9-42)		52 (22-105)		23 (10-47)		138 (58-279)		62 (26-126)	
2003	Mongolia									40 (16-81)		18 (7-37)		45 (18-92)		21 (8-43)		120 (48-246)		55 (22-113)	
2004	Mongolia									34 (13-72)		16 (6-33)		39 (15-82)		18 (7-38)		104 (40-217)		48 (19-101)	
2005	Mongolia									31 (12-66)		14 (5-30)		36 (13-75)		16 (6-34)		95 (35-199)		43 (16-91)	
2006	Mongolia									30 (12-62)		13 (5-28)		34 (14-70)		15 (6-31)		91 (36-187)		41 (16-84)	
2007	Mongolia									31 (13-61)		13 (6-26)		35 (15-70)		15 (6-30)		92 (39-185)		39 (17-79)	
2008	Mongolia									33 (15-64)		13 (6-26)		37 (17-72)		15 (7-29)		99 (45-193)		40 (18-78)	
2009	Mongolia									37 (18-70)		14 (7-27)		43 (21-79)		16 (8-30)		113 (55-210)		43 (21-80)	
2010	Mongolia									44 (23-78)		16 (8-28)		50 (26-88)		18 (9-32)		134 (68-234)		48 (25-85)	
2011	Mongolia									56 (30-107)		19 (10-36)		63 (34-122)		22 (12-41)		168 (91-324)		57 (31-110)	
2012	Mongolia									71 (46-122)		23 (15-39)		81 (52-138)		26 (17-45)		215 (139-367)		70 (45-119)	
2013	Mongolia									87 (60-138)		27 (19-43)		99 (68-157)		31 (21-49)		263 (181-417)		82 (57-130)	
2014	Mongolia									101 (64-147)		31 (20-45)		114 (73-167)		35 (22-51)		304 (193-444)		93 (59-136)	
2015	Mongolia	3202 (2763-3806)	0 (0-0)	964 (832-1146)	0 (0-0)	1245 (933-1420)	0 (0-0)	375 (281-427)	0 (0-0)	104 (63-146)	0 (0-0)	31 (19-44)	0 (0-0)	118 (72-165)	0 (0-0)	35 (22-50)	0 (0-0)	313 (191-440)	0 (0-0)	94 (58-132)	0 (0-0)
2000	Montenegro	94 (81-112)		230 (198-273)		61 (46-70)		149 (112-170)		0 (0-0)		0 (0-0)		0 (0-0)		0 (0-0)		0 (0-0)		0 (0-0)	
2001	Montenegro									0 (0-0)		0 (0-0)		0 (0-0)		0 (0-0)		0 (0-0)		0 (0-0)	
2002	Montenegro									1 (0-2)		2 (1-6)		1 (0-3)		2 (1-7)		2 (1-7)		6 (2-19)	
2003	Montenegro									1 (0-3)		2 (1-6)		1 (0-3)		2 (1-7)		3 (1-8)		6 (2-19)	
2004	Montenegro									2 (1-5)		4 (2-12)		2 (1-5)		5 (2-14)		5 (2-15)		12 (5-37)	
2005	Montenegro									1 (0-3)		2 (1-7)		1 (0-3)		3 (1-8)		3 (1-8)		7 (3-20)	
2006	Montenegro									1 (0-3)		3 (1-8)		1 (1-4)		3 (1-9)		3 (1-10)		9 (3-25)	
2007	Montenegro									1 (0-2)		2 (1-6)		1 (0-3)		2 (1-7)		2 (1-7)		6 (2-18)	
2008	Montenegro									1 (0-2)		2 (1-6)		1 (0-3)		2 (1-7)		2 (1-7)		6 (2-18)	
2009	Montenegro									1 (0-3)		3 (1-7)		1 (0-3)		3 (1-8)		3 (1-9)		8 (3-22)	
2010	Montenegro									0 (0-1)		1 (1-4)		1 (0-2)		1 (1-4)		1 (1-4)		4 (2-11)	
2011	Montenegro									1 (0-2)		2 (1-5)		1 (0-2)		2 (1-6)		2 (1-6)		5 (2-15)	
2012	Montenegro									1 (0-2)		2 (1-5)		1 (0-2)		2 (1-5)		2 (1-5)		5 (2-14)	
2013	Montenegro									1 (0-2)		1 (1-4)		1 (0-2)		2 (1-5)		2 (1-5)		4 (2-12)	
2014	Montenegro									1 (0-2)		2 (1-4)		1 (0-2)		2 (1-5)		2 (1-5)		5 (2-13)	
2015	Montenegro	83 (72-99)	0 (0-0)	230 (198-273)	0 (0-0)	54 (40-62)	0 (0-0)	149 (112-170)	0 (0-0)	1 (0-1)	0 (0-0)	1 (1-4)	0 (0-0)	1 (0-2)	0 (0-0)	2 (1-4)	0 (0-0)	2 (1-4)	0 (0-0)	4 (2-12)	0 (0-0)
2000	Morocco	61705 (53241-73350)		2028 (1750-2411)		24085 (18044-27471)		792 (593-903)		289 (89-662)		9 (3-22)		328 (101-752)		11 (3-25)		872 (268-1999)		29 (9-66)	
2001	Morocco									264 (82-603)		9 (3-20)		300 (93-685)		10 (3-23)		796 (246-1822)		27 (8-61)	
2002	Morocco									242 (75-554)		8 (3-19)		275 (86-629)		9 (3-22)		732 (228-1672)		25 (8-57)	
2003	Morocco									227 (71-516)		8 (2-18)		257 (80-586)		9 (3-20)		684 (214-1558)		24 (7-54)	
2004	Morocco									216 (67-491)		8 (2-17)		246 (77-558)		9 (3-19)		654 (204-1483)		23 (7-52)	
2005	Morocco									211 (69-473)		7 (2-17)		240 (79-537)		8 (3-19)		639 (210-1427)		22 (7-50)	
2006	Morocco									210 (73-461)		7 (3-16)		239 (83-523)		8 (3-18)		634 (220-1392)		22 (8-48)	
2007	Morocco									214 (78-459)		7 (3-16)		243 (89-522)		8 (3-18)		646 (236-1387)		22 (8-48)	
2008	Morocco									219 (85-460)		7 (3-16)		249 (97-522)		8 (3-18)		662 (257-1388)		22 (9-47)	
2009	Morocco									229 (95-469)		8 (3-16)		260 (108-532)		9 (4-18)		692 (286-1416)		23 (10-47)	
2010	Morocco									237 (105-474)		8 (3-15)		270 (120-538)		9 (4-18)		717 (318-1431)		23 (10-47)	
2011	Morocco									232 (102-459)		7 (3-15)		263 (116-521)		8 (4-17)		699 (308-1386)		22 (10-44)	
2012	Morocco									178 (79-351)		6 (2-11)		203 (89-399)		6 (3-12)		538 (238-1060)		17 (7-33)	
2013	Morocco									159 (54-374)		5 (2-11)		181 (61-425)		5 (2-13)		481 (163-1130)		15 (5-34)	
2014	Morocco									106 (36-248)		3 (1-7)		120 (41-282)		4 (1-8)		320 (110-750)		10 (3-22)	
2015	Morocco	17180 (14823-20422)	52 (44-61)	511 (441-607)	2 (1-2)	6743 (5052-7691)	20 (15-23)	200 (150-229)	1 (0-1)	87 (30-204)	0 (0-1)	3 (1-6)	0 (0-0)	99 (34-231)	0 (0-1)	3 (1-7)	0 (0-0)	263 (91-615)	1 (0-2)	8 (3-18)	0 (0-0)
2000	Mozambique	96827 (83544-115100)		2955 (2550-3513)		38049 (28506-43399)		1161 (870-1324)		1815 (1020-3256)		55 (31-99)		728 (409-1306)		22 (12-40)		5480 (3080-9833)		167 (94-300)	
2001	Mozambique									1821 (1038-3262)		54 (31-96)		730 (417-1308)		22 (12-39)		5498 (3136-9850)		162 (93-291)	
2002	Mozambique									1866 (1081-3340)		53 (31-96)		748 (434-1340)		21 (12-38)		5634 (3264-10086)		161 (93-289)	
2003	Mozambique									1952 (825-3952)		54 (23-110)		783 (331-1585)		22 (9-44)		5895 (2492-11933)		164 (69-332)	
2004	Mozambique									2067 (894-4172)		56 (24-113)		829 (359-1673)		22 (10-45)		6242 (2701-12597)		169 (73-341)	
2005	Mozambique									2179 (964-4376)		57 (25-115)		874 (386-1755)		23 (10-46)		6581 (2910-13213)		174 (77-348)	
2006	Mozambique									2245 (1014-4478)		58 (26-115)		901 (407-1796)		23 (10-46)		6780 (3061-13522)		174 (78-346)	
2007	Mozambique									2233 (1030-4425)		56 (26-111)		896 (413-1775)		22 (10-44)		6744 (3110-13360)		169 (78-334)	
2008	Mozambique									2157 (1021-4259)		53 (25-104)		865 (409-1708)		21 (10-42)		6513 (3082-12861)		159 (75-314)	
2009	Mozambique									2010 (903-4042)		48 (22-97)		806 (362-1621)		19 (9-39)		6068 (2727-12205)		145 (65-292)	
2010	Mozambique									1844 (792-3784)		43 (19-89)		740 (318-1518)		17 (7-36)		5567 (2391-11427)		130 (56-267)	
2011	Mozambique									1675 (690-3510)		38 (16-80)		672 (277-1408)		15 (6-32)		5058 (2083-10599)		116 (48-242)	
2012	Mozambique									1521 (602-3251)		34 (13-73)		610 (242-1304)		14 (5-29)		4592 (1819-9816)		103 (41-220)	
2013	Mozambique									1170 (445-2543)		26 (10-56)		469 (179-1020)		10 (4-22)		3533 (1345-7678)		78 (30-169)	
2014	Mozambique									894 (327-1971)		19 (7-42)		359 (131-791)		8 (3-17)		2700 (988-5952)		58 (21-128)	
2015	Mozambique	62930 (54297-74806)	23240 (20052-27625)	1329 (1147-1580)	491 (423-583)	24789 (18571-28274)	9154 (6858-10441)	523 (392-597)	193 (145-220)	768 (271-1714)	284 (100-633)	16 (6-36)	6 (2-13)	308 (109-688)	114 (40-254)	7 (2-15)	2 (1-5)	2319 (817-5176)	856 (302-1912)	49 (17-109)	18 (6-40)
2000	Myanmar	129023 (111324-153373)		2522 (2176-2998)		50125 (37554-57173)		980 (734-1117)		1687 (630-3647)		33 (12-71)		677 (253-1463)		13 (5-29)		5095 (1903-11013)		100 (37-215)	
2001	Myanmar									1676 (663-3556)		32 (13-68)		672 (266-1426)		13 (5-27)		5061 (2002-10739)		97 (38-206)	

Spn = *Streptococcus pneumoniae*; npnm = non-pneumonia, non-meningitis; HIV = cases only in HIV-infected children; Uncertainty range provided in parentheses.

Year	Country	Spn pneumonia cases (UR)	Spn pneumonia cases, HIV (UR)	Spn pneumonia incidence rate (UR)	Spn pneumonia incidence rate, HIV (UR)	Spn severe pneumonia cases (UR)	Spn severe pneumonia cases, HIV (UR)	Spn severe pneumonia incidence rate (UR)	Spn severe pneumonia incidence rate, HIV (UR)	Spn meningitis cases (UR)	Spn meningitis cases, HIV (UR)	Spn meningitis incidence rate (UR)	Spn meningitis incidence rate, HIV (UR)	Spn NPNM cases (UR)	Spn NPNM cases, HIV (UR)	Spn NPNM incidence rate (UR)	Spn NPNM incidence rate, HIV (UR)	Spn NPNM non-severe cases (UR)	Spn NPNM non-severe cases, HIV (UR)	SPN NPNM non-severe incidence rate (UR)	SPN NPNM non-severe incidence rate, HIV (UR)
2002	Myanmar									1671 (703-3477)		32 (13-66)		670 (282-1395)		13 (5-26)		5045 (2122-10498)		95 (40-199)	
2003	Myanmar									1657 (745-3379)		31 (14-63)		665 (299-1356)		12 (6-25)		5005 (2250-10205)		94 (42-192)	
2004	Myanmar									1632 (642-3404)		31 (12-64)		1854 (729-3867)		35 (14-72)		4928 (1939-10279)		92 (36-193)	
2005	Myanmar									1563 (618-3244)		29 (12-61)		1775 (702-3685)		33 (13-69)		4718 (1866-9796)		89 (35-184)	
2006	Myanmar									1466 (584-3034)		27 (11-57)		1666 (663-3446)		31 (12-65)		4428 (1763-9161)		83 (33-172)	
2007	Myanmar									1355 (543-2794)		26 (10-53)		1539 (617-3174)		29 (12-60)		4092 (1640-8438)		77 (31-160)	
2008	Myanmar									2269 (1042-4532)		44 (20-87)		910 (418-1818)		18 (8-35)		6851 (3147-13684)		132 (61-264)	
2009	Myanmar									1166 (474-2390)		23 (9-47)		1324 (539-2714)		26 (11-54)		3521 (1432-7216)		69 (28-142)	
2010	Myanmar									1091 (443-2235)		22 (9-45)		1239 (504-2539)		25 (10-51)		3294 (1339-6749)		66 (27-136)	
2011	Myanmar									1009 (413-2063)		21 (9-43)		1146 (470-2343)		24 (10-49)		3046 (1248-6230)		63 (26-129)	
2012	Myanmar									942 (389-1924)		20 (8-41)		1070 (442-2185)		23 (9-46)		2846 (1175-5810)		61 (25-124)	
2013	Myanmar									890 (370-1812)		19 (8-39)		1011 (420-2058)		22 (9-45)		2687 (1116-5471)		58 (24-119)	
2014	Myanmar									845 (353-1717)		19 (8-38)		960 (401-1951)		21 (9-43)		2553 (1067-5186)		56 (23-114)	
2015	Myanmar	94384 (81437-112196)	3388 (2924-4028)	2103 (1814-2499)	75 (65-90)	36962 (27692-42159)	1327 (994-1514)	823 (617-939)	30 (22-34)	802 (338-1626)	29 (12-58)	18 (8-36)	1 (0-1)	911 (383-1847)	33 (14-66)	20 (9-41)	1 (0-1)	2423 (1019-4910)	87 (37-176)	54 (23-109)	2 (1-4)
2000	Namibia	7734 (6673-9194)		2848 (2458-3386)		3040 (2278-3468)		1120 (839-1277)		39 (17-81)		15 (6-30)		16 (7-33)		6 (2-12)		119 (50-246)		44 (18-91)	
2001	Namibia									40 (17-82)		15 (6-30)		16 (7-33)		6 (2-12)		120 (51-247)		44 (19-91)	
2002	Namibia									40 (17-82)		15 (6-30)		16 (7-33)		6 (3-12)		121 (52-248)		44 (19-91)	
2003	Namibia									40 (18-83)		15 (6-30)		16 (7-33)		6 (3-12)		122 (53-250)		45 (20-92)	
2004	Namibia									41 (15-86)		15 (6-32)		47 (18-98)		17 (6-36)		124 (47-260)		45 (17-95)	
2005	Namibia									42 (16-87)		15 (6-32)		47 (18-99)		17 (7-36)		126 (48-264)		46 (18-97)	
2006	Namibia									42 (16-88)		15 (6-32)		48 (19-100)		17 (7-36)		127 (50-266)		46 (18-97)	
2007	Namibia									43 (17-88)		15 (6-32)		48 (19-100)		18 (7-36)		128 (51-267)		47 (18-97)	
2008	Namibia									43 (17-89)		16 (6-32)		49 (20-102)		18 (7-36)		131 (52-270)		47 (19-97)	
2009	Namibia									44 (18-91)		16 (6-32)		50 (20-103)		18 (7-37)		134 (54-275)		47 (19-97)	
2010	Namibia									46 (19-93)		16 (6-32)		52 (21-106)		18 (7-36)		138 (57-281)		48 (20-97)	
2011	Namibia									47 (20-95)		16 (7-32)		53 (22-108)		18 (7-36)		141 (59-287)		48 (20-97)	
2012	Namibia									49 (20-98)		16 (7-32)		55 (23-111)		18 (8-36)		147 (62-295)		48 (20-97)	
2013	Namibia									50 (21-100)		16 (7-32)		56 (24-113)		18 (8-36)		150 (64-300)		48 (20-95)	
2014	Namibia									51 (22-101)		16 (7-31)		57 (25-114)		18 (8-35)		153 (66-304)		47 (20-93)	
2015	Namibia	4676 (4034-5558)	1157 (999-1376)	1406 (1213-1671)	348 (300-414)	1837 (1376-2095)	455 (341-519)	552 (414-630)	137 (102-156)	33 (15-66)	8 (4-16)	10 (4-20)	2 (1-5)	38 (17-75)	9 (4-19)	11 (5-23)	3 (1-6)	101 (45-200)	25 (11-49)	30 (13-60)	8 (3-15)
2000	Nauru	24 (20-28)		2816 (2430-3348)		9 (7-10)		1091 (818-1245)		0 (0-0)		4 (1-13)		0 (0-0)		5 (1-15)		0 (0-0)		13 (4-40)	
2001	Nauru									0 (0-0)		4 (1-14)		0 (0-0)		5 (2-16)		0 (0-0)		13 (4-41)	
2002	Nauru									0 (0-0)		5 (1-14)		0 (0-0)		5 (2-16)		0 (0-0)		14 (4-42)	
2003	Nauru									0 (0-0)		5 (1-14)		0 (0-0)		5 (2-16)		0 (0-0)		14 (4-42)	
2004	Nauru									0 (0-0)		5 (1-15)		0 (0-0)		5 (2-17)		0 (0-0)		14 (4-44)	
2005	Nauru									0 (0-0)		5 (2-15)		0 (0-0)		6 (2-17)		0 (0-0)		15 (5-45)	
2006	Nauru									0 (0-0)		5 (2-16)		0 (0-0)		6 (2-18)		0 (0-0)		16 (5-48)	
2007	Nauru									0 (0-0)		5 (2-16)		0 (0-0)		6 (2-19)		0 (0-0)		16 (5-49)	
2008	Nauru									0 (0-0)		5 (2-17)		0 (0-0)		6 (2-19)		0 (0-0)		16 (5-50)	
2009	Nauru									0 (0-0)		4 (1-11)		0 (0-0)		4 (1-13)		0 (0-0)		11 (3-34)	
2010	Nauru									0 (0-0)		4 (1-12)		0 (0-0)		4 (1-13)		0 (0-0)		11 (3-35)	
2011	Nauru									0 (0-0)		4 (1-12)		0 (0-0)		4 (1-13)		0 (0-0)		11 (4-35)	
2012	Nauru									0 (0-0)		4 (1-12)		0 (0-0)		4 (1-13)		0 (0-0)		11 (4-35)	
2013	Nauru									0 (0-0)		4 (1-12)		0 (0-0)		4 (1-13)		0 (0-0)		12 (4-35)	
2014	Nauru									0 (0-0)		4 (1-12)		0 (0-0)		4 (1-13)		0 (0-0)		12 (4-35)	
2015	Nauru	10 (8-12)	0 (0-0)	1338 (1155-1591)	0 (0-0)	4 (3-4)	0 (0-0)	512 (383-584)	0 (0-0)	0 (0-0)	0 (0-0)	4 (1-12)	0 (0-0)	0 (0-0)	0 (0-0)	4 (1-13)	0 (0-0)	0 (0-0)	0 (0-0)	12 (4-36)	0 (0-0)
2000	Nepal	87768 (75728-104332)		2499 (2156-2971)		34370 (25750-39203)		979 (733-1116)		665 (208-1583)		19 (6-45)		267 (83-635)		8 (2-18)		2009 (628-4780)		57 (18-136)	
2001	Nepal									633 (203-1496)		18 (6-42)		254 (81-600)		7 (2-17)		1910 (614-4517)		54 (17-128)	
2002	Nepal									595 (160-1440)		17 (5-41)		676 (182-1635)		19 (5-46)		1796 (484-4347)		51 (14-124)	
2003	Nepal									561 (156-1346)		16 (4-39)		637 (177-1529)		18 (5-44)		1693 (471-4064)		49 (14-117)	
2004	Nepal									527 (151-1254)		15 (4-37)		599 (172-1424)		17 (5-42)		1593 (457-3787)		46 (13-110)	
2005	Nepal									497 (147-1171)		15 (4-35)		565 (167-1330)		17 (5-39)		1502 (445-3536)		44 (13-105)	
2006	Nepal									464 (142-1081)		14 (4-33)		527 (161-1227)		16 (5-37)		1401 (429-3263)		42 (13-98)	
2007	Nepal									434 (135-1005)		13 (4-31)		493 (153-1141)		15 (5-35)		1310 (406-3033)		40 (12-93)	
2008	Nepal									402 (126-925)		13 (4-29)		456 (143-1051)		14 (4-33)		1214 (381-2794)		38 (12-87)	
2009	Nepal									374 (119-857)		12 (4-27)		425 (135-974)		13 (4-31)		1130 (360-2589)		36 (11-82)	
2010	Nepal									342 (111-780)		11 (4-25)		389 (126-886)		13 (4-28)		1033 (334-2354)		33 (11-76)	
2011	Nepal									314 (103-713)		10 (3-23)		357 (117-809)		12 (4-27)		949 (311-2152)		31 (10-71)	
2012	Nepal									281 (92-637)		9 (3-22)		319 (105-723)		11 (4-24)		849 (278-1923)		29 (9-65)	
2013	Nepal									256 (84-580)		9 (3-20)		291 (95-658)		10 (3-23)		773 (253-1750)		27 (9-61)	
2014	Nepal									236 (77-534)		8 (3-19)		268 (88-606)		10 (3-22)		712 (233-1611)		25 (8-57)	
2015	Nepal	46732 (40321-55551)	407 (351-484)	1693 (1461-2012)	15 (13-18)	18419 (13799-21009)	160 (120-183)	667 (500-761)	6 (4-7)	183 (60-414)	2 (1-4)	7 (2-15)	0 (0-0)	208 (68-471)	2 (1-4)	8 (2-17)	0 (0-0)	553 (181-1251)	5 (2-11)	20 (7-45)	0 (0-0)
2000	Netherlands	1106 (954-1315)		115 (99-137)		717 (537-818)		75 (56-85)		97 (44-176)		10 (5-18)		110 (50-200)		11 (5-21)		292 (133-531)		30 (14-55)	
2001	Netherlands									96 (44-175)		10 (5-18)		109 (50-199)		11 (5-21)		291 (132-528)		30 (14-55)	
2002	Netherlands									96 (44-175)		10 (5-18)		110 (50-199)		11 (5-21)		291 (132-529)		30 (14-55)	
2003	Netherlands									85 (39-154)		9 (4-16)		96 (44-175)		10 (4-18)		257 (116-466)		26 (12-48)	
2004	Netherlands									35 (16-63)		4 (2-6)		39 (18-71)		4 (2-7)		104 (47-189)		11 (5-19)	
2005	Netherlands									67 (30-121)		7 (3-12)		76 (34-138)		8 (4-14)		202 (92-366)		21 (9-37)	
2006	Netherlands									30 (13-54)		3 (1-6)		34 (15-61)		3 (2-6)		90 (41-163)		9 (4-17)	
2007	Netherlands									43 (20-78)		4 (2-8)		49 (22-89)		5 (2-9)		130 (59-237)		14 (6-25)	
2008	Netherlands									32 (14-58)		3 (2-6)		36 (16-65)		4 (2-7)		96 (43-174)		10 (5-18)	
2009	Netherlands									31 (14-56)		3 (2-6)		35 (16-63)		4 (2-7)		93 (42-168)		10 (5-18)	
2010	Netherlands									27 (12-48)		3 (1-5)		30 (14-55)		3 (2-6)		80 (36-146)		9 (4-16)	
2011	Netherlands									20 (9-36)		2 (1-4)		23 (10-41)		3 (1-5)		60 (27-109)		7 (3-12)	
2012	Netherlands									11 (5-20)		1 (1-2)		13 (6-23)		1 (1-3)		34 (15-62)		4 (2-7)	
2013	Netherlands									1 (1-2)		0 (0-0)		2 (1-3)		0 (0-0)		4 (2-8)		0 (0-1)	
2014	Netherlands									7 (3-12)		1 (0-1)		7 (3-14)		1 (0-2)		20 (9-36)		2 (1-4)	
2015	Netherlands	262 (226-311)	0 (0-0)	30 (26-36)	0 (0-0)	170 (127-193)	0 (0-0)	19 (15-22)	0 (0-0)	6 (3-11)	0 (0-0)	1 (0-1)	0 (0-0)	7 (3-12)	0 (0-0)	1 (0-1)	0 (0-0)	18 (8-33)	0 (0-0)	2 (1-4)	0 (0-0)
2000	New Zealand	535 (461-636)		192 (165-228)		347 (260-395)		124 (93-142)		64 (29-114)		23 (10-41)		73 (33-129)		26 (12-46)		194 (87-343)		70 (31-123)	
2001	New Zealand									81 (36-143)		29 (13-52)		92 (41-163)		34 (15-59)		244 (109-432)		89 (40-158)	
2002	New Zealand									62 (28-110)		23 (10-40)		71 (32-125)		26 (12-46)		188 (84-332)		69 (31-122)	
2003	New Zealand									44 (20-78)		16 (7-28)		50 (22-88)		18 (8-32)		133 (59-235)		48 (22-86)	
2004	New Zealand									47 (21-84)		17 (8-30)		54 (24-95)		19 (9-34)		143 (64-252)		52 (23-91)	
2005	New Zealand									55 (25-98)		20 (9-35)		63 (28-111)		22 (10-40)		167 (75-296)		60 (27-106)	
2006	New Zealand									12 (5-21)		4 (2-7)		13 (6-23)		5 (2-8)		35 (16-62)		12 (6-22)	
2007	New Zealand									31 (14-56)		11 (5-19)		36 (16-63)		12 (5-22)		95 (42-168)		33 (15-58)	
2008	New Zealand									43 (19-76)		14 (6-26)		49 (22-87)		16 (7-29)		130 (58-231)		44 (20-77)	
2009	New Zealand									11 (5-19)		3 (2-6)		12 (5-21)		4 (2-7)		32 (14-56)		10 (5-19)	
2010	New Zealand									20 (9-35)		6 (3-11)		22 (10-39)		7 (3-13)		59 (26-105)		19 (9-34)	
2011	New Zealand									31 (14-56)		10 (4-18)		36 (16-63)		11 (5-20)		95 (42-168)		30 (14-54)	
2012	New Zealand									16 (7-28)		5 (2-9)		18 (8-32)		6 (3-10)		48 (21-85)		15 (7-27)	
2013	New Zealand									11 (5-19)		4 (2-6)		12 (6-22)		4 (2-7)		33 (15-59)		11 (5-19)	
2014	New Zealand									10 (4-17)		3 (1-6)		11 (5-19)		4 (2-6)		29 (13-52)		10 (4-17)	
2015	New Zealand	181 (157-216)	0 (0-0)	60 (52-71)	0 (0-0)	118 (88-134)	0 (0-0)	39 (29-44)	0 (0-0)	9 (4-16)	0 (0-0)	3 (1-5)	0 (0-0)	10 (5-18)	0 (0-0)	3 (2-6)	0 (0-0)	28 (12-49)	0 (0-0)	9 (4-16)	0 (0-0)
2000	Nicaragua	12491 (10777-14848)		1907 (1645-2267)		4891 (3664-5578)		747 (559-852)		71 (20-188)		11 (3-29)		81 (23-213)		12 (4-33)		216 (61-567)		33 (9-87)	
2001	Nicaragua									68 (20-179)		11 (3-28)		78 (22-203)		12 (3-31)		206 (59-540)		32 (9-83)	
2002	Nicaragua									63 (18-164)		10 (3-25)		71 (21-186)		11 (3-29)		190 (55-495)		29 (9-77)	
2003	Nicaragua									58 (17-151)		9 (3-24)		66 (19-172)		10 (3-27)		175 (51-456)		28 (8-72)	
2004	Nicaragua									59 (17-152)		9 (3-24)		66 (20-172)		11 (3-27)		177 (52-458)		28 (8-73)	
2005	Nicaragua									57 (17-148)		9 (3-24)		65 (19-168)		10 (3-27)		173 (51-447)		28 (8-71)	
2006	Nicaragua									57 (17-148)		9 (3-24)		65 (19-168)		10 (3-27)		173 (52-447)		28 (8-72)	
2007	Nicaragua									59 (12-182)		9 (2-29)		66 (14-207)		11 (2-33)		177 (37-551)		28 (6-89)	
2008	Nicaragua									59 (13-184)		10 (2-30)		67 (14-209)		11 (2-34)		179 (38-557)		29 (6-90)	
2009	Nicaragua									58 (13-181)		9 (2-29)		66 (14-206)		11 (2-33)		176 (38-547)		28 (6-88)	
2010	Nicaragua									56 (12-174)		9 (2-28)		64 (14-198)		10 (2-32)		170 (37-527)		27 (6-85)	
2011	Nicaragua									42 (9-129)		7 (1-21)		47 (10-146)		8 (2-24)		126 (28-388)		20 (4-63)	
2012	Nicaragua									28 (6-87)		5 (1-14)		32 (7-98)		5 (1-16)		85 (19-261)		14 (3-42)	
2013	Nicaragua									23 (5-71)		4 (1-12)		26 (6-80)		4 (1-13)		69 (15-213)		11 (3-35)	
2014	Nicaragua									20 (4-61)		3 (1-10)		23 (5-70)		4 (1-12)		60 (14-186)		10 (2-31)	
2015	Nicaragua	2298 (1982-2731)	5 (4-6)	386 (333-459)	1 (1-1)	898 (673-1024)	2 (1-2)	151 (113-172)	0 (0-0)	14 (3-42)	0 (0-0)	2 (1-7)	0 (0-0)	16 (4-48)	0 (0-0)	3 (1-8)	0 (0-0)	42 (9-127)	0 (0-0)	7 (2-21)	0 (0-0)
2000	Niger	88818 (76635-105580)		3912 (3375-4650)		34636 (25949-39506)		1525 (1143-1740)		2184 (1121-4222)		96 (49-186)		876 (450-1693)		39 (20-75)		6595 (3386-12748)		290 (149-561)	
2001	Niger									2269 (1184-4352)		95 (50-183)		910 (475-1746)		38 (20-73)		6851 (3574-13141)		288 (150-552)	
2002	Niger									2315 (1226-4402)		93 (49-178)		929 (492-1765)		37 (20-71)		6991 (3703-13291)		282 (149-536)	
2003	Niger									2336 (1255-4396)		91 (49-171)		937 (503-1763)		37 (20-69)		7053 (3790-13275)		275 (148-517)	
2004	Niger									2349 (1279-4370)		88 (48-165)		942 (513-1753)		35 (19-66)		7094 (3863-13197)		267 (145-497)	
2005	Niger									2363 (1307-4353)		86 (47-158)		948 (524-1746)		34 (19-63)		7134 (3948-13145)		259 (143-477)	
2006	Niger									2386 (1346-4365)		83 (47-152)		957 (540-1751)		33 (19-61)		7204 (4064-13182)		251 (142-459)	
2007	Niger									2476 (1407-4527)		83 (47-152)		993 (564-1816)		33 (19-61)		7477 (4249-13671)		251 (143-459)	
2008	Niger									2384 (961-4961)		77 (31-161)		956 (386-1990)		31 (12-64)		7199 (2903-14981)		233 (94-485)	
2009	Niger									2361 (965-4919)		74 (30-154)		947 (387-1973)		30 (12-62)		7129 (2914-14854)		222 (91-464)	
2010	Niger									2420 (1002-5042)		73 (30-151)		971 (402-2022)		29 (12-61)		7308 (3025-15224)		219 (91-457)	
2011	Niger									2412 (1009-5013)		69 (29-144)		967 (405-2011)		28 (12-58)		7282 (3046-15137)		209 (87-435)	
2012	Niger									2403 (1013-4972)		66 (28-137)		964 (406-1994)		27 (11-55)		7255 (3060-15014)		200 (84-414)	
2013	Niger									2359 (1004-4862)		63 (27-129)		946 (403-1950)		25 (11-52)		7123 (3031-14680)		189 (80-389)	
2014	Niger									2257 (969-4634)		58 (25-118)		905 (389-1859)		23 (10-47)		6815 (2927-13992)		174 (75-357)	
2015	Niger	70460 (60794-83757)	1231 (1062-1463)	1729 (1492-2055)	30 (26-36)	27477 (20586-31340)	480 (360-547)	674 (505-769)	12 (9-13)	1614 (700-3302)	28 (12-58)	40 (17-81)	1 (0-1)	647 (281-1324)	11 (5-23)	16 (7-32)	0 (0-1)	4873 (2114-9970)	85 (37-174)	120 (52-245)	2 (1-4)
2000	Nigeria	641024 (553090-761997)		3092 (2668-3675)		248760 (186369-283734)		1200 (899-1369)		8034 (4110-15630)		39 (20-75)		3223 (1649-6269)		16 (8-30)		24261 (12412-47196)		117 (60-228)	
2001	Nigeria									8173 (4230-15810)		38 (20-74)		3278 (1697-6341)		15 (8-30)		24678 (12774-47739)		115 (60-223)	
2002	Nigeria									8227 (4307-15814)		37 (20-72)		3300 (1727-6343)		15 (8-29)		24843 (13005-47753)		113 (59-217)	
2003	Nigeria									8057 (4263-15375)		36 (19-68)		3232 (1710-6167)		14 (8-27)		24328 (12871-46426)		107 (57-205)	
2004	Nigeria									8070 (4318-15294)		35 (19-66)		3237 (1732-6134)		14 (7-26)		24368 (13037-46181)		105 (56-198)	
2005	Nigeria									8133 (4408-15329)		34 (18-64)		3262 (1768-6149)		14 (7-26)		24559 (13311-46288)		103 (56-194)	
2006	Nigeria									8455 (4653-15882)		34 (19-64)		3391 (1866-6370)		14 (8-26)		25529 (14051-47958)		103 (57-194)	
2007	Nigeria									8706 (3360-18630)		34 (13-74)		3492 (1348-7472)		14 (5-29)		26288 (10146-56255)		104 (40-222)	
2008	Nigeria									8781 (3482-18764)		34 (13-72)		3522 (1397-7526)		14 (5-29)		26515 (10515-56660)		102 (41-218)	
2009	Nigeria									8873 (3468-19102)		33 (13-72)		3559 (1391-7662)		13 (5-29)		26792 (10472-57682)		101 (39-217)	
2010	Nigeria									8935 (3444-19382)		33 (13-71)		3584 (1381-7774)		13 (5-29)		26981 (10399-58525)		99 (38-215)	
2011	Nigeria									8679 (3297-18952)		31 (12-68)		3481 (1322-7602)		12 (5-27)		26206 (9955-57228)		94 (36-205)	
2012	Nigeria									8213 (3059-18087)		29 (11-63)		3294 (1227-7255)		12 (4-25)		24799 (9236-54617)		87 (32-191)	
2013	Nigeria									8030 (2931-17819)		27 (10-61)		3221 (1176-7147)		11 (4-24)		24247 (8851-53807)		83 (30-184)	
2014	Nigeria									7749 (2772-17315)		26 (9-58)		3108 (1112-6945)		10 (4-23)		23399 (8372-52283)		78 (28-174)	
2015	Nigeria	664724 (573539-790170)	130683 (112756-155346)	2173 (1875-2583)	427 (369-508)	254709 (190827-290521)	50075 (37516-57116)	833 (624-950)	164 (123-187)	7119 (2496-16006)	1400 (491-3147)	23 (8-52)	5 (2-10)	2855 (1001-6420)	561 (197-1262)	9 (3-21)	2 (1-4)	21497 (7538-48333)	4226 (1482-9502)	70 (25-158)	14 (5-31)
2000	Niue	8 (7-10)		5263 (4541-6256)		3 (2-4)		2011 (1507-2294)		0 (0-0)		95 (43-168)		0 (0-0)		108 (48-191)		0 (0-1)		287 (128-509)	
2001	Niue									0 (0-0)		103 (46-183)		0 (0-0)		117 (52-207)		0 (0-1)		312 (139-551)	
2002	Niue									0 (0-0)		100 (45-177)		0 (0-0)		114 (51-201)		0 (0-1)		302 (135-535)	
2003	Niue									0 (0-0)		97 (43-171)		0 (0-0)		110 (49-194)		0 (0-1)		292 (130-517)	
2004	Niue									0 (0-0)		0 (0-0)		0 (0-0)		0 (0-0)		0 (0-0)		0 (0-0)	
2005	Niue									0 (0-0)		0 (0-0)		0 (0-0)		0 (0-0)		0 (0-0)		0 (0-0)	
2006	Niue									0 (0-0)		102 (45-180)		0 (0-0)		115 (52-204)		0 (0-1)		307 (137-543)	
2007	Niue									0 (0-0)		103 (46-183)		0 (0-0)		118 (53-208)		0 (0-1)		313 (140-553)	
2008	Niue									0 (0-0)		109 (49-193)		0 (0-0)		124 (55-219)		0 (0-1)		329 (147-583)	
2009	Niue									0 (0-0)		84 (37-148)		0 (0-0)		95 (43-168)		0 (0-1)		253 (113-448)	
2010	Niue									0 (0-0)		80 (36-142)		0 (0-0)		91 (41-161)		0 (0-1)		242 (108-429)	
2011	Niue									0 (0-0)		62 (28-110)		0 (0-0)		70 (31-125)		0 (0-0)		187 (84-331)	
2012	Niue									0 (0-0)		53 (24-94)		0 (0-0)		60 (27-107)		0 (0-0)		160 (72-284)	
2013	Niue									0 (0-0)		38 (17-67)		0 (0-0)		43 (19-77)		0 (0-0)		115 (51-204)	
2014	Niue									0 (0-0)		33 (15-58)		0 (0-0)		37 (17-65)		0 (0-0)		98 (44-174)	
2015	Niue	0 (0-0)	0 (0-0)	283 (244-337)	0 (0-0)	0 (0-0)	0 (0-0)	103 (77-118)	0 (0-0)	0 (0-0)	0 (0-0)	31 (14-54)	0 (0-0)	0 (0-0)	0 (0-0)	35 (16-62)	0 (0-0)	0 (0-0)	0 (0-0)	93 (41-164)	0 (0-0)
2000	Norway	341 (294-406)		115 (99-137)		221 (166-252)		75 (56-85)		40 (18-73)		14 (6-25)		46 (21-83)		15 (7-28)		121 (55-220)		41 (19-74)	
2001	Norway									0 (0-0)		0 (0-0)		0 (0-0)		0 (0-0)		0 (0-0)		0 (0-0)	
2002	Norway									7 (3-13)		2 (1-4)		8 (4-14)		3 (1-5)		21 (10-39)		7 (3-13)	
2003	Norway									16 (7-30)		6 (3-10)		19 (8-34)		6 (3-12)		49 (22-89)		17 (8-31)	
2004	Norway									7 (3-13)		3 (1-5)		8 (4-15)		3 (1-5)		22 (10-41)		8 (4-14)	
2005	Norway									0 (0-0)		0 (0-0)		0 (0-0)		0 (0-0)		0 (0-0)		0 (0-0)	
2006	Norway									15 (7-27)		5 (2-10)		17 (8-31)		6 (3-11)		45 (20-82)		16 (7-29)	
2007	Norway									11 (5-20)		4 (2-7)		13 (6-23)		4 (2-8)		34 (15-62)		12 (5-22)	
2008	Norway									0 (0-0)		0 (0-0)		0 (0-0)		0 (0-0)		0 (0-0)		0 (0-0)	
2009	Norway									9 (4-17)		3 (1-6)		10 (5-19)		3 (2-6)		28 (13-50)		9 (4-17)	
2010	Norway									2 (1-4)		1 (0-1)		3 (1-5)		1 (0-2)		7 (3-14)		2 (1-4)	

Spn = *Streptococcus pneumoniae*; npnm = non-pneumonia, non-meningitis; HIV = cases only in HIV-infected children; Uncertainty range provided in parentheses.

Year	Country	Spn pneumonia cases (UR)	Spn pneumonia cases, HIV (UR)	Spn pneumonia incidence rate (UR)	Spn pneumonia incidence rate, HIV (UR)	Spn severe pneumonia cases (UR)	Spn severe pneumonia cases, HIV (UR)	Spn severe pneumonia incidence rate (UR)	Spn severe pneumonia incidence rate, HIV (UR)	Spn meningitis cases (UR)	Spn meningitis cases, HIV (UR)	Spn meningitis incidence rate (UR)	Spn meningitis incidence rate, HIV (UR)	Spn NPNM cases (UR)	Spn NPNM cases, HIV (UR)	Spn NPNM incidence rate (UR)	Spn NPNM incidence rate, HIV (UR)	Spn NPNM non-severe cases (UR)	Spn NPNM non-severe cases, HIV (UR)	SPN NPNM non-severe incidence rate (UR)	SPN NPNM non-severe incidence rate, HIV (UR)
2011	Norway									6 (3-12)		2 (1-4)		7 (3-13)		2 (1-4)		20 (9-35)		6 (3-12)	
2012	Norway									1 (1-2)		0 (0-1)		2 (1-3)		0 (0-1)		4 (2-7)		1 (1-2)	
2013	Norway									1 (0-1)		0 (0-0)		1 (0-2)		0 (0-1)		2 (1-5)		1 (0-1)	
2014	Norway									1 (1-2)		0 (0-1)		1 (1-3)		0 (0-1)		4 (2-7)		1 (1-2)	
2015	Norway	68 (59-81)	0 (0-0)	22 (19-26)	0 (0-0)	44 (33-50)	0 (0-0)	14 (11-16)	0 (0-0)	1 (1-2)	0 (0-0)	0 (0-1)	0 (0-0)	1 (1-3)	0 (0-0)	0 (0-1)	0 (0-0)	4 (2-7)	0 (0-0)	1 (1-2)	0 (0-0)
2000	Oman	6102 (5265-7254)		2222 (1917-2642)		2398 (1797-2736)		873 (654-996)		30 (18-41)		11 (6-15)		34 (20-46)		12 (7-17)		89 (53-123)		32 (19-45)	
2001	Oman									26 (15-35)		9 (6-13)		29 (17-40)		11 (6-15)		77 (46-106)		29 (17-39)	
2002	Oman									36 (22-50)		14 (8-19)		41 (25-57)		16 (9-22)		110 (66-152)		41 (25-57)	
2003	Oman									34 (20-47)		13 (8-18)		38 (23-53)		15 (9-20)		102 (61-141)		39 (23-54)	
2004	Oman									31 (18-42)		12 (7-16)		35 (21-48)		13 (8-18)		93 (56-128)		36 (21-49)	
2005	Oman									29 (17-40)		11 (7-15)		33 (20-45)		13 (8-17)		87 (52-120)		33 (20-46)	
2006	Oman									27 (16-37)		11 (6-15)		31 (19-43)		12 (7-17)		82 (49-113)		32 (19-45)	
2007	Oman									27 (16-38)		11 (6-15)		31 (19-43)		12 (7-17)		83 (50-114)		33 (20-45)	
2008	Oman									28 (17-38)		11 (6-15)		31 (19-43)		12 (7-17)		83 (50-115)		32 (19-44)	
2009	Oman									25 (15-34)		9 (5-12)		28 (17-39)		10 (6-14)		75 (45-103)		27 (16-38)	
2010	Oman									22 (13-31)		7 (4-10)		25 (15-35)		8 (5-12)		67 (40-92)		23 (14-31)	
2011	Oman									18 (11-25)		6 (3-8)		20 (12-28)		7 (4-9)		54 (33-75)		17 (10-24)	
2012	Oman									18 (11-25)		5 (3-7)		20 (12-28)		6 (4-8)		54 (33-75)		16 (10-22)	
2013	Oman									15 (9-21)		4 (3-6)		17 (10-24)		5 (3-7)		46 (28-64)		13 (8-18)	
2014	Oman									15 (9-20)		4 (2-5)		17 (10-23)		4 (3-6)		44 (26-61)		12 (7-16)	
2015	Oman	1275 (1100-1515)	6 (5-7)	336 (290-400)	2 (1-2)	496 (372-566)	2 (2-3)	131 (98-149)	1 (0-1)	15 (9-20)	0 (0-0)	4 (2-5)	0 (0-0)	17 (10-23)	0 (0-0)	4 (3-6)	0 (0-0)	44 (27-61)	0 (0-0)	12 (7-16)	0 (0-0)
2000	Pakistan	308958 (266575-367264)		1563 (1349-1858)		120599 (90352-137554)		610 (457-696)		6794 (3025-13537)		34 (15-68)		2725 (1214-5430)		14 (6-27)		20516 (9136-40877)		104 (46-207)	
2001	Pakistan									6675 (2985-13277)		34 (15-67)		2677 (1197-5325)		13 (6-27)		20157 (9014-40091)		101 (45-202)	
2002	Pakistan									6485 (2912-12876)		33 (15-65)		2601 (1168-5164)		13 (6-26)		19583 (8792-38880)		99 (44-196)	
2003	Pakistan									6324 (2849-12531)		32 (14-64)		2537 (1143-5026)		13 (6-26)		19096 (8604-37840)		97 (44-192)	
2004	Pakistan									6212 (2808-12284)		32 (14-63)		2492 (1126-4927)		13 (6-25)		18758 (8479-37094)		96 (43-189)	
2005	Pakistan									6172 (2800-12186)		31 (14-62)		2476 (1123-4888)		13 (6-25)		18638 (8454-36796)		95 (43-187)	
2006	Pakistan									6112 (2784-12056)		31 (14-61)		2452 (1116-4835)		12 (6-24)		18456 (8405-36403)		93 (42-183)	
2007	Pakistan									6076 (2769-11993)		30 (14-59)		2437 (1111-4810)		12 (5-24)		18346 (8362-36215)		91 (41-179)	
2008	Pakistan									6040 (2725-11973)		29 (13-57)		2423 (1093-4802)		12 (5-23)		18238 (8229-36154)		88 (40-174)	
2009	Pakistan									6000 (2682-11952)		28 (13-56)		2406 (1076-4794)		11 (5-22)		18117 (8099-36091)		84 (38-168)	
2010	Pakistan									5903 (2616-11820)		27 (12-54)		2368 (1049-4741)		11 (5-22)		17825 (7898-35692)		81 (36-162)	
2011	Pakistan									5819 (2555-11708)		26 (11-51)		2334 (1025-4696)		10 (4-21)		17572 (7716-35352)		77 (34-155)	
2012	Pakistan									5665 (2463-11442)		24 (11-49)		2272 (988-4589)		10 (4-20)		17105 (7438-34549)		73 (32-148)	
2013	Pakistan									3715 (1617-7509)		16 (7-32)		1490 (648-3012)		6 (3-13)		11218 (4882-22675)		47 (21-96)	
2014	Pakistan									3192 (1397-6444)		13 (6-27)		1280 (560-2585)		5 (2-11)		9637 (4218-19459)		40 (18-81)	
2015	Pakistan	339428 (292866-403485)	1099 (948-1307)	1400 (1208-1664)	5 (4-5)	132256 (99085-150850)	428 (321-488)	545 (409-622)	2 (1-2)	3098 (1363-6246)	10 (4-20)	13 (6-26)	0 (0-0)	1243 (547-2505)	4 (2-8)	5 (2-10)	0 (0-0)	9355 (4116-18862)	30 (13-61)	39 (17-78)	0 (0-0)
2000	Palau	81 (69-96)		6273 (5413-7457)		31 (23-35)		2408 (1804-2747)		1 (0-1)		47 (21-84)		1 (0-1)		54 (24-95)		2 (1-3)		143 (64-253)	
2001	Palau									1 (0-1)		44 (20-78)		1 (0-1)		50 (22-88)		2 (1-3)		132 (59-234)	
2002	Palau									1 (0-1)		51 (23-91)		1 (0-2)		58 (26-103)		2 (1-4)		155 (69-274)	
2003	Palau									1 (0-1)		35 (16-63)		1 (0-1)		40 (18-71)		2 (1-3)		107 (48-189)	
2004	Palau									1 (0-1)		42 (19-75)		1 (0-2)		48 (22-85)		2 (1-4)		128 (57-227)	
2005	Palau									1 (0-1)		40 (18-71)		1 (0-2)		46 (20-81)		2 (1-4)		121 (54-215)	
2006	Palau									1 (0-1)		41 (18-72)		1 (0-1)		46 (21-82)		2 (1-4)		123 (55-217)	
2007	Palau									1 (0-1)		32 (14-57)		1 (0-1)		36 (16-65)		2 (1-3)		97 (43-172)	
2008	Palau									0 (0-1)		34 (15-60)		1 (0-1)		39 (17-68)		1 (1-3)		103 (46-182)	
2009	Palau									0 (0-0)		20 (9-36)		0 (0-1)		23 (10-41)		1 (0-1)		61 (27-108)	
2010	Palau									0 (0-1)		34 (15-61)		0 (0-1)		39 (18-69)		1 (1-2)		104 (47-184)	
2011	Palau									0 (0-1)		30 (13-52)		0 (0-1)		34 (15-60)		1 (0-2)		89 (40-158)	
2012	Palau									0 (0-0)		17 (8-31)		0 (0-0)		20 (9-35)		1 (0-1)		52 (23-92)	
2013	Palau									0 (0-0)		8 (4-15)		0 (0-0)		10 (4-17)		0 (0-1)		25 (11-45)	
2014	Palau									0 (0-0)		7 (3-12)		0 (0-0)		8 (4-14)		0 (0-1)		21 (9-37)	
2015	Palau	6 (6-8)	0 (0-0)	420 (362-499)	0 (0-0)	3 (2-3)	0 (0-0)	164 (123-187)	0 (0-0)	0 (0-0)	0 (0-0)	6 (3-11)	0 (0-0)	0 (0-0)	0 (0-0)	7 (3-12)	0 (0-0)	0 (0-0)	0 (0-0)	18 (8-32)	0 (0-0)
2000	Panama	4995 (4310-5937)		1486 (1282-1766)		1944 (1456-2217)		578 (433-660)		25 (7-69)		7 (2-20)		29 (8-78)		9 (3-23)		76 (22-208)		23 (7-62)	
2001	Panama									37 (11-100)		11 (3-29)		42 (12-114)		12 (4-33)		111 (33-303)		33 (10-89)	
2002	Panama									35 (11-96)		10 (3-28)		40 (12-109)		12 (3-32)		106 (32-289)		31 (9-85)	
2003	Panama									16 (5-45)		5 (1-13)		19 (6-51)		6 (2-15)		50 (15-135)		15 (4-40)	
2004	Panama									34 (10-91)		10 (3-27)		38 (12-103)		11 (3-31)		101 (31-274)		30 (9-81)	
2005	Panama									23 (7-61)		7 (2-18)		26 (8-69)		8 (2-20)		68 (21-184)		20 (6-54)	
2006	Panama									27 (8-72)		8 (2-21)		30 (9-82)		9 (3-24)		81 (25-217)		24 (7-65)	
2007	Panama									26 (8-71)		8 (2-21)		30 (9-80)		9 (3-24)		80 (25-214)		24 (7-63)	
2008	Panama									17 (5-45)		5 (2-13)		19 (6-51)		6 (2-15)		51 (16-137)		15 (5-40)	
2009	Panama									37 (11-98)		11 (3-28)		42 (13-111)		12 (4-32)		111 (35-296)		32 (10-85)	
2010	Panama									31 (10-82)		9 (3-23)		35 (11-93)		10 (3-27)		93 (29-248)		27 (8-71)	
2011	Panama									29 (9-78)		8 (3-22)		33 (11-89)		9 (3-25)		89 (28-235)		25 (8-66)	
2012	Panama									17 (5-44)		5 (1-12)		19 (6-50)		5 (2-14)		51 (16-134)		14 (4-37)	
2013	Panama									18 (6-47)		5 (2-13)		20 (6-53)		6 (2-15)		54 (17-142)		15 (5-39)	
2014	Panama									11 (4-30)		3 (1-8)		13 (4-34)		4 (1-9)		35 (11-91)		10 (3-25)	
2015	Panama	1107 (955-1316)	2 (1-2)	306 (264-363)	0 (0-0)	432 (324-493)	1 (0-1)	119 (89-136)	0 (0-0)	9 (3-23)	0 (0-0)	2 (1-6)	0 (0-0)	10 (3-26)	0 (0-0)	3 (1-7)	0 (0-0)	27 (9-70)	0 (0-0)	7 (2-19)	0 (0-0)
2000	Papua New Guinea	16462 (14203-19568)		2005 (1730-2384)		6442 (4826-7347)		785 (588-895)		123 (40-375)		15 (5-46)		49 (16-150)		6 (2-18)		371 (119-1133)		45 (15-138)	
2001	Papua New Guinea									127 (41-385)		15 (5-46)		51 (16-155)		6 (2-18)		382 (123-1164)		45 (15-138)	
2002	Papua New Guinea									129 (42-393)		15 (5-46)		52 (17-158)		6 (2-18)		390 (126-1188)		46 (15-139)	
2003	Papua New Guinea									131 (43-399)		15 (5-46)		53 (17-160)		6 (2-18)		396 (128-1206)		46 (15-139)	
2004	Papua New Guinea									133 (44-407)		15 (5-46)		54 (17-163)		6 (2-19)		403 (131-1230)		46 (15-140)	
2005	Papua New Guinea									136 (38-438)		15 (4-49)		154 (43-497)		17 (5-56)		410 (115-1322)		46 (13-149)	
2006	Papua New Guinea									137 (39-442)		15 (4-49)		155 (44-502)		17 (5-56)		413 (117-1334)		46 (13-149)	
2007	Papua New Guinea									137 (39-443)		15 (4-49)		156 (44-503)		17 (5-56)		415 (118-1338)		46 (13-148)	
2008	Papua New Guinea									136 (39-438)		15 (4-48)		154 (44-497)		17 (5-54)		411 (117-1322)		45 (13-144)	
2009	Papua New Guinea									134 (39-431)		14 (4-46)		153 (44-490)		16 (5-52)		406 (117-1303)		43 (13-140)	
2010	Papua New Guinea									132 (38-420)		14 (4-45)		149 (43-478)		16 (5-51)		397 (115-1270)		42 (12-134)	
2011	Papua New Guinea									129 (37-410)		13 (4-43)		146 (43-466)		15 (4-49)		389 (113-1239)		41 (12-130)	
2012	Papua New Guinea									126 (37-399)		13 (4-41)		143 (42-453)		15 (4-47)		380 (111-1205)		39 (12-125)	
2013	Papua New Guinea									124 (36-391)		13 (4-40)		140 (41-444)		14 (4-46)		373 (110-1180)		39 (11-122)	
2014	Papua New Guinea									120 (36-378)		12 (4-39)		136 (40-430)		14 (4-44)		362 (107-1142)		37 (11-117)	
2015	Papua New Guinea	14709 (12691-17484)	769 (663-914)	1502 (1296-1786)	79 (68-93)	5778 (4329-6590)	302 (226-345)	590 (442-673)	31 (23-35)	106 (31-332)	6 (2-17)	11 (3-34)	1 (0-2)	120 (36-377)	6 (2-20)	12 (4-39)	1 (0-2)	319 (95-1002)	17 (5-52)	33 (10-102)	2 (1-5)
2000	Paraguay	14104 (12169-16765)		2036 (1756-2420)		5517 (4133-6293)		796 (597-908)		89 (34-157)		13 (5-23)		102 (39-178)		15 (6-26)		270 (103-473)		39 (15-68)	
2001	Paraguay									89 (34-155)		13 (5-22)		101 (38-176)		15 (6-25)		269 (102-467)		39 (15-67)	
2002	Paraguay									90 (34-154)		13 (5-22)		103 (38-175)		15 (6-25)		273 (101-465)		40 (15-68)	
2003	Paraguay									176 (63-290)		26 (9-43)		200 (72-330)		30 (11-49)		530 (191-877)		78 (28-130)	
2004	Paraguay									207 (118-372)		31 (18-56)		235 (134-422)		35 (20-63)		625 (356-1122)		94 (53-169)	
2005	Paraguay									227 (121-383)		34 (18-58)		258 (138-435)		39 (21-66)		685 (366-1156)		104 (56-176)	
2006	Paraguay									243 (123-388)		37 (19-60)		276 (140-441)		43 (22-68)		733 (372-1173)		113 (57-181)	
2007	Paraguay									245 (120-380)		38 (19-59)		279 (137-432)		43 (21-67)		741 (364-1148)		114 (56-177)	
2008	Paraguay									240 (118-372)		37 (18-57)		273 (134-423)		42 (20-65)		725 (356-1124)		111 (54-172)	
2009	Paraguay									228 (112-353)		34 (17-53)		259 (127-401)		39 (19-61)		688 (338-1066)		104 (51-161)	
2010	Paraguay									218 (107-339)		33 (16-51)		248 (122-385)		37 (18-58)		660 (324-1022)		99 (49-154)	
2011	Paraguay									206 (101-319)		31 (15-47)		234 (115-362)		35 (17-54)		621 (305-963)		92 (45-143)	
2012	Paraguay									148 (72-229)		22 (11-34)		168 (82-260)		25 (12-39)		446 (219-691)		66 (33-103)	
2013	Paraguay									117 (58-181)		18 (9-27)		133 (65-206)		20 (10-31)		354 (174-548)		53 (26-82)	
2014	Paraguay									104 (51-162)		16 (8-24)		119 (58-184)		18 (9-28)		315 (155-488)		47 (23-74)	
2015	Paraguay	3857 (3328-4585)	39 (33-46)	582 (502-692)	6 (5-7)	1506 (1129-1718)	15 (11-17)	227 (170-259)	2 (2-3)	92 (45-143)	1 (0-1)	14 (7-22)	0 (0-0)	105 (51-162)	1 (1-2)	16 (8-25)	0 (0-0)	279 (137-432)	3 (1-4)	42 (21-65)	0 (0-1)
2000	Peru	44816 (38668-53273)		1514 (1307-1800)		17577 (13169-20049)		594 (445-677)		469 (133-1220)		16 (5-41)		532 (151-1386)		18 (5-47)		1415 (403-3685)		48 (14-125)	
2001	Peru									404 (118-1041)		14 (4-35)		459 (135-1183)		16 (5-40)		1219 (358-3144)		42 (12-107)	
2002	Peru									382 (116-976)		13 (4-33)		434 (132-1109)		15 (5-38)		1155 (350-2948)		40 (12-101)	
2003	Peru									354 (111-893)		12 (4-31)		402 (126-1014)		14 (4-35)		1068 (335-2696)		37 (11-92)	
2004	Peru									383 (90-1162)		13 (3-40)		435 (103-1319)		15 (4-45)		1157 (273-3508)		40 (9-120)	
2005	Peru									359 (83-1086)		12 (3-37)		408 (95-1234)		14 (3-42)		1084 (252-3280)		37 (9-113)	
2006	Peru									346 (80-1042)		12 (3-36)		393 (91-1184)		13 (3-41)		1044 (242-3148)		36 (8-108)	
2007	Peru									326 (78-976)		11 (3-34)		371 (89-1108)		13 (3-38)		985 (236-2947)		34 (8-101)	
2008	Peru									301 (75-897)		10 (3-31)		342 (85-1018)		12 (3-35)		910 (227-2707)		31 (8-93)	
2009	Peru									268 (70-794)		9 (2-27)		304 (79-902)		10 (3-31)		808 (211-2399)		28 (7-83)	
2010	Peru									202 (49-610)		7 (2-21)		229 (55-692)		8 (2-24)		609 (147-1841)		21 (5-63)	
2011	Peru									159 (33-494)		5 (1-17)		181 (38-561)		6 (1-19)		481 (101-1492)		17 (3-51)	
2012	Peru									115 (23-362)		4 (1-12)		131 (26-411)		4 (1-14)		348 (70-1092)		12 (2-37)	
2013	Peru									75 (15-235)		3 (1-8)		85 (17-267)		3 (1-9)		225 (46-710)		8 (2-24)	
2014	Peru									59 (12-185)		2 (0-6)		67 (14-210)		2 (0-7)		177 (37-559)		6 (1-19)	
2015	Peru	8362 (7215-9940)	33 (29-40)	282 (243-335)	1 (1-1)	3247 (2432-3703)	13 (10-15)	109 (82-125)	0 (0-0)	52 (11-165)	0 (0-1)	2 (0-6)	0 (0-0)	59 (12-187)	0 (0-1)	2 (0-6)	0 (0-0)	158 (33-497)	1 (0-2)	5 (1-17)	0 (0-0)
2000	Philippines	419497 (361951-498663)		3973 (3428-4722)		164392 (123162-187505)		1557 (1166-1776)		1177 (421-2580)		11 (4-24)		1337 (478-2931)		13 (5-28)		3555 (1272-7791)		34 (12-74)	
2001	Philippines									1144 (404-2516)		11 (4-23)		1300 (459-2858)		12 (4-27)		3455 (1221-7599)		32 (11-71)	
2002	Philippines									1106 (386-2443)		10 (4-22)		1257 (439-2774)		12 (4-25)		3341 (1167-7375)		31 (11-68)	
2003	Philippines									1071 (370-2373)		10 (3-21)		1216 (420-2696)		11 (4-24)		3233 (1116-7167)		29 (10-65)	
2004	Philippines									1027 (351-2288)		9 (3-20)		1167 (399-2598)		10 (4-23)		3102 (1060-6907)		28 (9-62)	
2005	Philippines									955 (323-2135)		9 (3-19)		1084 (367-2425)		10 (3-22)		2883 (974-6446)		26 (9-57)	
2006	Philippines									924 (309-2074)		8 (3-19)		1050 (351-2356)		9 (3-21)		2791 (933-6264)		25 (8-56)	
2007	Philippines									880 (291-1981)		8 (3-18)		1000 (330-2250)		9 (3-20)		2658 (878-5983)		24 (8-54)	
2008	Philippines									845 (276-1905)		8 (3-18)		960 (313-2163)		9 (3-20)		2552 (832-5751)		24 (8-53)	
2009	Philippines									821 (276-1834)		8 (3-17)		933 (314-2083)		9 (3-20)		2479 (835-5538)		23 (8-52)	
2010	Philippines									818 (284-1808)		8 (3-17)		929 (323-2053)		9 (3-19)		2470 (858-5459)		23 (8-52)	
2011	Philippines									847 (304-1848)		8 (3-17)		962 (345-2099)		9 (3-20)		2556 (917-5580)		24 (9-52)	
2012	Philippines									890 (330-1917)		8 (3-18)		1011 (374-2177)		9 (3-20)		2689 (995-5788)		25 (9-54)	
2013	Philippines									952 (265-2425)		9 (2-22)		1081 (301-2754)		10 (3-25)		2874 (801-7322)		26 (7-67)	
2014	Philippines									819 (241-2061)		7 (2-19)		930 (273-2341)		8 (2-21)		2473 (727-6225)		23 (7-57)	
2015	Philippines	211269 (182287-251139)	202 (175-241)	1909 (1647-2269)	2 (2-2)	83555 (62599-95302)	80 (60-91)	755 (566-861)	1 (1-1)	913 (284-2270)	1 (0-2)	8 (3-21)	0 (0-0)	1037 (323-2578)	1 (0-2)	9 (3-23)	0 (0-0)	2757 (859-6854)	3 (1-7)	25 (8-62)	0 (0-0)
2000	Poland	4519 (3899-5372)		230 (198-273)		2930 (2195-3342)		149 (112-170)		82 (37-148)		4 (2-8)		93 (42-168)		5 (2-9)		247 (112-448)		13 (6-23)	
2001	Poland									74 (33-134)		4 (2-7)		84 (38-152)		4 (2-8)		222 (101-404)		12 (5-22)	
2002	Poland									52 (24-95)		3 (1-5)		59 (27-108)		3 (2-6)		158 (71-286)		9 (4-16)	
2003	Poland									57 (26-104)		3 (1-6)		65 (29-118)		4 (2-7)		173 (78-313)		10 (4-18)	
2004	Poland									48 (22-87)		3 (1-5)		55 (25-99)		3 (1-6)		145 (66-263)		8 (4-15)	
2005	Poland									50 (23-90)		3 (1-5)		57 (26-103)		3 (1-6)		150 (68-273)		9 (4-16)	
2006	Poland									81 (37-146)		5 (2-8)		92 (42-166)		5 (2-9)		244 (111-442)		14 (6-25)	
2007	Poland									64 (29-116)		4 (2-6)		73 (33-132)		4 (2-7)		194 (88-352)		11 (5-19)	
2008	Poland									76 (35-138)		4 (2-7)		87 (39-157)		5 (2-8)		230 (104-418)		12 (6-22)	
2009	Poland									65 (30-119)		3 (2-6)		74 (34-135)		4 (2-7)		198 (90-359)		10 (5-19)	
2010	Poland									59 (27-107)		3 (1-5)		67 (30-121)		3 (2-6)		178 (81-323)		9 (4-16)	
2011	Poland									55 (25-100)		3 (1-5)		63 (28-114)		3 (1-6)		167 (76-302)		8 (4-15)	
2012	Poland									49 (22-88)		2 (1-4)		55 (25-100)		3 (1-5)		147 (67-267)		7 (3-13)	
2013	Poland									43 (20-79)		2 (1-4)		49 (22-89)		2 (1-4)		131 (59-238)		6 (3-12)	
2014	Poland									45 (20-81)		2 (1-4)		51 (23-92)		3 (1-5)		135 (61-245)		7 (3-12)	
2015	Poland	4511 (3892-5362)	5 (4-5)	230 (198-273)	0 (0-0)	2925 (2191-3336)	3 (2-3)	149 (112-170)	0 (0-0)	45 (21-82)	0 (0-0)	2 (1-4)	0 (0-0)	51 (23-93)	0 (0-0)	3 (1-5)	0 (0-0)	137 (62-248)	0 (0-0)	7 (3-13)	0 (0-0)
2000	Portugal	601 (518-714)		115 (99-137)		389 (292-444)		75 (56-85)		96 (44-174)		18 (8-33)		109 (49-198)		21 (9-38)		290 (131-526)		56 (25-101)	
2001	Portugal									27 (12-49)		5 (2-9)		31 (14-56)		6 (3-11)		82 (37-149)		16 (7-28)	
2002	Portugal									74 (33-134)		14 (6-25)		84 (38-152)		16 (7-29)		222 (101-403)		42 (19-76)	
2003	Portugal									48 (22-86)		9 (4-16)		54 (25-98)		10 (5-18)		144 (65-261)		27 (12-49)	
2004	Portugal									26 (12-46)		5 (2-9)		29 (13-53)		5 (2-10)		77 (35-140)		14 (6-26)	
2005	Portugal									21 (10-39)		4 (2-7)		24 (11-44)		4 (2-8)		64 (29-117)		12 (5-22)	
2006	Portugal									20 (9-36)		4 (2-7)		23 (10-41)		4 (2-8)		60 (27-109)		11 (5-20)	
2007	Portugal									11 (5-19)		2 (1-4)		12 (5-22)		2 (1-4)		32 (15-58)		6 (3-11)	
2008	Portugal									7 (3-12)		1 (1-2)		7 (3-13)		1 (1-3)		20 (9-36)		4 (2-7)	
2009	Portugal									6 (3-12)		1 (1-2)		7 (3-13)		1 (1-3)		20 (9-35)		4 (2-7)	
2010	Portugal									12 (5-22)		3 (1-5)		14 (6-25)		3 (1-5)		36 (17-66)		8 (3-14)	
2011	Portugal									7 (3-13)		2 (1-3)		8 (4-15)		2 (1-3)		22 (10-40)		5 (2-8)	
2012	Portugal									2 (1-4)		1 (0-1)		3 (1-5)		1 (0-1)		7 (3-13)		2 (1-3)	
2013	Portugal									6 (3-11)		1 (1-2)		7 (3-13)		2 (1-3)		19 (8-34)		4 (2-7)	
2014	Portugal									3 (1-6)		1 (0-1)		4 (2-7)		1 (0-2)		10 (4-18)		2 (1-4)	
2015	Portugal	149 (129-177)	4 (4-5)	35 (30-41)	1 (1-1)	97 (72-110)	3 (2-3)	22 (17-26)	1 (0-1)	2 (1-4)	0 (0-0)	1 (0-1)	0 (0-0)	3 (1-5)	0 (0-0)	1 (0-1)	0 (0-0)	7 (3-12)	0 (0-0)	2 (1-3)	0 (0-0)
2000	Qatar	1254 (1082-1491)		2296 (1981-2729)		493 (369-562)		903 (676-1030)		6 (4-8)		11 (7-15)		7 (4-9)		13 (8-17)		18 (11-25)		33 (20-46)	
2001	Qatar									6 (3-8)		10 (6-14)		6 (4-9)		12 (7-16)		17 (10-24)		31 (18-42)	
2002	Qatar									5 (3-7)		9 (6-13)		6 (4-8)		11 (6-15)		16 (10-22)		28 (17-39)	
2003	Qatar									5 (3-7)		9 (5-12)		6 (4-8)		10 (6-14)		16 (9-21)		27 (16-37)	

Spn = *Streptococcus pneumoniae*; npnm = non-pneumonia, non-meningitis; HIV = cases only in HIV-infected children; Uncertainty range provided in parentheses.

Year	Country	Spn pneumonia cases (UR)	Spn pneumonia cases, HIV (UR)	Spn pneumonia incidence rate (UR)	Spn pneumonia incidence rate, HIV (UR)	Spn severe pneumonia cases (UR)	Spn severe pneumonia cases, HIV (UR)	Spn severe pneumonia incidence rate (UR)	Spn severe pneumonia incidence rate, HIV (UR)	Spn meningitis cases (UR)	Spn meningitis cases, HIV (UR)	Spn meningitis incidence rate (UR)	Spn meningitis incidence rate, HIV (UR)	Spn NPNM cases (UR)	Spn NPNM cases, HIV (UR)	Spn NPNM incidence rate (UR)	Spn NPNM incidence rate, HIV (UR)	Spn NPNM non-severe cases (UR)	Spn NPNM non-severe cases, HIV (UR)	SPN NPNM non-severe incidence rate (UR)	SPN NPNM non-severe incidence rate, HIV (UR)
2004	Qatar									5 (3-7)		8 (5-11)		6 (4-8)		9 (6-13)		16 (9-21)		25 (15-34)	
2005	Qatar									5 (3-7)		8 (5-10)		6 (4-8)		9 (5-12)		16 (10-22)		23 (14-32)	
2006	Qatar									5 (3-7)		7 (4-10)		6 (3-8)		8 (5-11)		15 (9-21)		22 (13-30)	
2007	Qatar									5 (3-7)		7 (4-9)		6 (3-8)		8 (5-11)		15 (9-20)		20 (12-28)	
2008	Qatar									5 (3-7)		6 (4-9)		6 (3-8)		7 (4-10)		15 (9-21)		20 (12-27)	
2009	Qatar									4 (3-6)		5 (3-7)		5 (3-7)		6 (4-8)		13 (8-18)		16 (10-22)	
2010	Qatar									4 (3-6)		5 (3-7)		5 (3-7)		6 (3-8)		13 (8-18)		15 (9-20)	
2011	Qatar									3 (2-5)		4 (2-5)		4 (2-5)		4 (2-6)		10 (6-14)		11 (6-15)	
2012	Qatar									4 (2-5)		3 (2-5)		4 (2-6)		4 (2-5)		11 (6-15)		10 (6-14)	
2013	Qatar									3 (2-4)		3 (2-4)		3 (2-5)		3 (2-4)		9 (5-12)		8 (5-11)	
2014	Qatar									3 (2-4)		2 (1-3)		3 (2-4)		3 (2-4)		9 (5-12)		7 (4-10)	
2015	Qatar	442 (382-526)	0 (0-0)	340 (293-404)	0 (0-0)	172 (129-196)	0 (0-0)	132 (99-151)	0 (0-0)	3 (2-4)	0 (0-0)	2 (1-3)	0 (0-0)	3 (2-5)	0 (0-0)	3 (2-3)	0 (0-0)	9 (5-12)	0 (0-0)	7 (4-9)	0 (0-0)
2000	Republic of Korea	61015 (52645-72530)		1973 (1703-2346)		23843 (17863-27196)		771 (578-880)		188 (113-260)		6 (4-8)		214 (128-295)		7 (4-10)		569 (341-784)		18 (11-25)	
2001	Republic of Korea									158 (95-219)		6 (3-8)		180 (108-248)		6 (4-9)		479 (287-660)		17 (10-23)	
2002	Republic of Korea									161 (96-222)		6 (4-8)		183 (110-252)		7 (4-9)		485 (291-669)		18 (11-24)	
2003	Republic of Korea									126 (76-174)		5 (3-6)		143 (86-197)		5 (3-7)		380 (228-525)		14 (8-19)	
2004	Republic of Korea									134 (80-185)		5 (3-7)		152 (91-210)		6 (3-8)		405 (243-558)		15 (9-21)	
2005	Republic of Korea									100 (60-137)		4 (2-5)		113 (68-156)		4 (3-6)		301 (181-415)		11 (7-16)	
2006	Republic of Korea									132 (79-182)		5 (3-7)		150 (90-207)		6 (4-8)		399 (239-550)		16 (9-22)	
2007	Republic of Korea									157 (94-216)		6 (4-9)		178 (107-246)		7 (4-10)		474 (284-653)		19 (12-26)	
2008	Republic of Korea									103 (62-143)		4 (3-6)		118 (71-162)		5 (3-7)		313 (188-431)		13 (8-18)	
2009	Republic of Korea									109 (65-150)		5 (3-7)		123 (74-170)		5 (3-7)		328 (197-452)		14 (9-20)	
2010	Republic of Korea									110 (66-151)		5 (3-7)		125 (75-172)		6 (3-8)		332 (199-457)		15 (9-21)	
2011	Republic of Korea									139 (83-191)		6 (4-8)		157 (95-217)		7 (4-10)		419 (251-577)		18 (11-25)	
2012	Republic of Korea									101 (61-139)		4 (3-6)		115 (69-158)		5 (3-7)		305 (183-420)		13 (8-18)	
2013	Republic of Korea									100 (60-138)		4 (3-6)		113 (68-156)		5 (3-7)		301 (181-415)		13 (8-18)	
2014	Republic of Korea									122 (73-168)		5 (3-7)		138 (83-191)		6 (4-8)		368 (221-507)		16 (10-23)	
2015	Republic of Korea	15398 (13286-18304)	1 (0-1)	685 (591-814)	0 (0-0)	6002 (4496-6846)	0 (0-0)	267 (200-304)	0 (0-0)	79 (47-108)	0 (0-0)	3 (2-5)	0 (0-0)	89 (54-123)	0 (0-0)	4 (2-5)	0 (0-0)	237 (142-327)	0 (0-0)	11 (6-15)	0 (0-0)
2000	Republic of Moldova	1091 (941-1297)		460 (397-547)		707 (530-807)		298 (223-340)		11 (4-32)		4 (2-13)		12 (5-36)		5 (2-15)		32 (12-96)		13 (5-41)	
2001	Republic of Moldova									4 (1-16)		2 (0-7)		5 (1-18)		2 (1-8)		12 (3-48)		5 (1-21)	
2002	Republic of Moldova									5 (1-23)		2 (1-10)		6 (2-26)		3 (1-12)		16 (4-68)		7 (2-31)	
2003	Republic of Moldova									4 (1-19)		2 (0-9)		5 (1-22)		2 (1-10)		13 (3-57)		6 (1-27)	
2004	Republic of Moldova									4 (1-18)		2 (0-9)		5 (1-21)		2 (0-10)		12 (3-55)		6 (1-27)	
2005	Republic of Moldova									5 (1-25)		3 (1-12)		6 (1-28)		3 (1-14)		17 (3-76)		8 (2-37)	
2006	Republic of Moldova									1 (0-6)		1 (0-3)		1 (0-7)		1 (0-3)		4 (1-17)		2 (0-9)	
2007	Republic of Moldova									2 (1-11)		1 (0-5)		3 (1-12)		1 (0-6)		7 (2-32)		3 (1-15)	
2008	Republic of Moldova									3 (1-15)		2 (0-7)		4 (1-17)		2 (0-8)		10 (2-44)		5 (1-21)	
2009	Republic of Moldova									6 (1-24)		3 (1-11)		6 (2-27)		3 (1-13)		17 (4-71)		8 (2-34)	
2010	Republic of Moldova									6 (2-26)		3 (1-12)		7 (2-29)		3 (1-14)		19 (5-78)		9 (2-36)	
2011	Republic of Moldova									10 (3-41)		5 (1-19)		12 (3-46)		5 (1-21)		31 (9-123)		14 (4-56)	
2012	Republic of Moldova									6 (2-23)		3 (1-11)		7 (2-27)		3 (1-12)		19 (6-71)		9 (3-32)	
2013	Republic of Moldova									4 (1-13)		2 (1-6)		4 (1-15)		2 (1-7)		11 (4-41)		5 (2-18)	
2014	Republic of Moldova									7 (2-25)		3 (1-11)		8 (3-28)		4 (1-13)		22 (8-74)		10 (3-34)	
2015	Republic of Moldova	761 (657-905)	9 (7-10)	347 (300-413)	4 (3-5)	493 (370-563)	6 (4-6)	225 (169-257)	3 (2-3)	5 (2-18)	0 (0-0)	2 (1-8)	0 (0-0)	6 (2-20)	0 (0-0)	3 (1-9)	0 (0-0)	16 (6-54)	0 (0-1)	7 (3-24)	0 (0-0)
2000	Romania	2533 (2186-3011)		230 (198-273)		1642 (1230-1873)		149 (112-170)		326 (148-592)		30 (13-54)		371 (168-672)		34 (15-61)		985 (447-1788)		89 (41-162)	
2001	Romania									323 (147-587)		28 (13-51)		367 (167-667)		32 (15-58)		977 (443-1773)		85 (39-154)	
2002	Romania									338 (153-613)		29 (13-53)		383 (174-696)		33 (15-61)		1019 (462-1850)		89 (40-162)	
2003	Romania									215 (98-391)		19 (9-35)		244 (111-444)		22 (10-40)		650 (295-1179)		59 (27-107)	
2004	Romania									157 (71-285)		15 (7-27)		178 (81-323)		17 (8-30)		473 (215-859)		44 (20-81)	
2005	Romania									141 (64-256)		13 (6-24)		160 (73-291)		15 (7-28)		427 (194-774)		41 (18-74)	
2006	Romania									128 (58-232)		12 (6-23)		145 (66-264)		14 (6-26)		386 (175-701)		37 (17-68)	
2007	Romania									122 (55-221)		12 (5-22)		139 (63-251)		13 (6-24)		368 (167-668)		36 (16-65)	
2008	Romania									80 (36-145)		8 (4-14)		91 (41-165)		9 (4-16)		242 (110-439)		23 (11-42)	
2009	Romania									72 (33-131)		7 (3-13)		82 (37-149)		8 (4-14)		219 (99-397)		21 (10-38)	
2010	Romania									84 (38-152)		8 (4-15)		95 (43-173)		9 (4-17)		253 (115-459)		24 (11-44)	
2011	Romania									106 (48-193)		10 (5-19)		121 (55-219)		12 (5-21)		321 (145-582)		31 (14-56)	
2012	Romania									55 (25-101)		5 (2-10)		63 (29-114)		6 (3-11)		167 (76-304)		17 (8-30)	
2013	Romania									74 (34-135)		8 (3-14)		84 (38-153)		9 (4-16)		224 (102-407)		23 (10-42)	
2014	Romania									68 (31-123)		7 (3-13)		77 (35-139)		8 (4-15)		204 (93-370)		22 (10-40)	
2015	Romania	2090 (1804-2485)	0 (0-0)	230 (198-273)	0 (0-0)	1355 (1015-1546)	0 (0-0)	149 (112-170)	0 (0-0)	63 (28-114)	0 (0-0)	7 (3-12)	0 (0-0)	71 (32-129)	0 (0-0)	8 (4-14)	0 (0-0)	189 (86-343)	0 (0-0)	21 (9-38)	0 (0-0)
2000	Russian Federation	29109 (25116-34602)		460 (397-547)		18871 (14138-21524)		298 (223-340)		1504 (682-2730)		24 (11-43)		1709 (775-3100)		27 (12-49)		4542 (2061-8243)		72 (33-130)	
2001	Russian Federation									1424 (646-2584)		22 (10-39)		1617 (734-2935)		25 (11-45)		4299 (1950-7802)		65 (30-119)	
2002	Russian Federation									1304 (591-2366)		20 (9-35)		1481 (672-2687)		22 (10-40)		3937 (1786-7145)		59 (27-107)	
2003	Russian Federation									1153 (523-2092)		17 (8-31)		1309 (594-2376)		20 (9-36)		3481 (1579-6316)		52 (24-94)	
2004	Russian Federation									1048 (475-1901)		16 (7-28)		1190 (540-2159)		18 (8-32)		3163 (1435-5740)		47 (21-86)	
2005	Russian Federation									943 (428-1711)		14 (6-25)		1071 (486-1943)		16 (7-28)		2847 (1292-5167)		42 (19-76)	
2006	Russian Federation									849 (385-1541)		12 (5-22)		964 (438-1750)		14 (6-25)		2564 (1163-4653)		36 (16-66)	
2007	Russian Federation									758 (344-1375)		10 (5-19)		861 (390-1562)		12 (5-22)		2288 (1038-4152)		32 (14-57)	
2008	Russian Federation									686 (311-1244)		9 (4-17)		779 (353-1413)		11 (5-19)		2071 (939-3758)		28 (13-51)	
2009	Russian Federation									639 (290-1160)		8 (4-15)		726 (329-1317)		10 (4-17)		1930 (876-3502)		25 (12-46)	
2010	Russian Federation									599 (272-1087)		8 (3-14)		680 (309-1235)		9 (4-16)		1809 (821-3283)		23 (10-42)	
2011	Russian Federation									558 (253-1012)		7 (3-13)		633 (287-1150)		8 (4-14)		1684 (764-3056)		21 (10-38)	
2012	Russian Federation									618 (280-1121)		7 (3-14)		702 (318-1274)		8 (4-15)		1866 (846-3386)		23 (10-41)	
2013	Russian Federation									614 (279-1114)		7 (3-13)		698 (316-1266)		8 (4-15)		1854 (841-3365)		22 (10-39)	
2014	Russian Federation									586 (266-1063)		7 (3-12)		665 (302-1207)		8 (3-14)		1769 (802-3210)		20 (9-36)	
2015	Russian Federation	41461 (35774-49286)	371 (320-441)	460 (397-547)	4 (4-5)	26879 (20138-30658)	241 (180-275)	298 (223-340)	3 (2-3)	562 (255-1019)	5 (2-9)	6 (3-11)	0 (0-0)	638 (289-1158)	6 (3-10)	7 (3-13)	0 (0-0)	1696 (770-3078)	15 (7-28)	19 (9-34)	0 (0-0)
2000	Rwanda	30099 (25970-35779)		2294 (1979-2727)		11745 (8799-13396)		895 (671-1021)		1008 (502-2011)		77 (38-153)		404 (202-807)		31 (15-61)		3043 (1517-6073)		232 (116-463)	
2001	Rwanda									1039 (524-2069)		73 (37-145)		417 (210-830)		29 (15-58)		3137 (1581-6246)		220 (111-439)	
2002	Rwanda									925 (472-1838)		63 (32-125)		371 (189-737)		25 (13-50)		2792 (1424-5549)		190 (97-377)	
2003	Rwanda									847 (286-1933)		58 (19-132)		340 (115-775)		23 (8-53)		2559 (864-5836)		174 (59-397)	
2004	Rwanda									736 (253-1674)		51 (17-115)		295 (101-672)		20 (7-46)		2223 (763-5056)		153 (53-348)	
2005	Rwanda									645 (225-1463)		44 (15-100)		259 (90-587)		18 (6-40)		1949 (680-4419)		134 (47-303)	
2006	Rwanda									572 (199-1295)		39 (13-88)		229 (80-520)		16 (5-35)		1726 (602-3911)		117 (41-265)	
2007	Rwanda									518 (180-1170)		34 (12-77)		208 (72-469)		14 (5-31)		1563 (544-3534)		103 (36-233)	
2008	Rwanda									480 (166-1081)		31 (11-69)		192 (67-434)		12 (4-28)		1449 (503-3264)		92 (32-208)	
2009	Rwanda									456 (144-1034)		28 (9-64)		518 (163-1174)		32 (10-72)		1378 (434-3122)		85 (27-193)	
2010	Rwanda									367 (130-809)		22 (8-49)		417 (148-919)		25 (9-56)		1109 (394-2443)		67 (24-148)	
2011	Rwanda									274 (98-598)		16 (6-35)		311 (111-679)		18 (7-40)		826 (295-1805)		49 (17-107)	
2012	Rwanda									186 (67-403)		11 (4-24)		211 (76-457)		12 (4-27)		560 (201-1216)		33 (12-72)	
2013	Rwanda									149 (54-321)		9 (3-19)		169 (61-365)		10 (4-22)		449 (163-970)		27 (10-58)	
2014	Rwanda									99 (36-212)		6 (2-13)		112 (41-241)		7 (2-14)		298 (109-641)		18 (7-38)	
2015	Rwanda	5908 (5098-7023)	93 (80-111)	354 (306-421)	6 (5-7)	2307 (1729-2632)	36 (27-41)	138 (104-158)	2 (2-2)	80 (29-172)	1 (0-3)	5 (2-10)	0 (0-0)	91 (33-196)	1 (1-3)	5 (2-12)	0 (0-0)	242 (88-520)	4 (1-8)	15 (5-31)	0 (0-0)
2000	Saint Kitts and Nevis	65 (56-77)		1318 (1137-1566)		25 (19-29)		512 (383-584)		0 (0-0)		0 (0-0)		0 (0-0)		0 (0-0)		0 (0-0)		0 (0-0)	
2001	Saint Kitts and Nevis									0 (0-0)		0 (0-0)		0 (0-0)		0 (0-0)		0 (0-0)		0 (0-0)	
2002	Saint Kitts and Nevis									0 (0-0)		0 (0-0)		0 (0-0)		0 (0-0)		0 (0-0)		0 (0-0)	
2003	Saint Kitts and Nevis									0 (0-0)		0 (0-0)		0 (0-0)		0 (0-0)		0 (0-0)		0 (0-0)	
2004	Saint Kitts and Nevis									0 (0-0)		0 (0-0)		0 (0-0)		0 (0-0)		0 (0-0)		0 (0-0)	
2005	Saint Kitts and Nevis									0 (0-0)		0 (0-0)		0 (0-0)		0 (0-0)		0 (0-0)		0 (0-0)	
2006	Saint Kitts and Nevis									0 (0-0)		0 (0-0)		0 (0-0)		0 (0-0)		0 (0-0)		0 (0-0)	
2007	Saint Kitts and Nevis									0 (0-0)		0 (0-0)		0 (0-0)		0 (0-0)		0 (0-0)		0 (0-0)	
2008	Saint Kitts and Nevis									0 (0-0)		0 (0-0)		0 (0-0)		0 (0-0)		0 (0-0)		0 (0-0)	
2009	Saint Kitts and Nevis									0 (0-0)		0 (0-0)		0 (0-0)		0 (0-0)		0 (0-0)		0 (0-0)	
2010	Saint Kitts and Nevis									0 (0-0)		0 (0-0)		0 (0-0)		0 (0-0)		0 (0-0)		0 (0-0)	
2011	Saint Kitts and Nevis									2 (1-3)		37 (18-57)		2 (1-3)		42 (20-65)		5 (3-8)		111 (54-172)	
2012	Saint Kitts and Nevis									2 (1-3)		40 (19-61)		2 (1-3)		45 (22-70)		6 (3-9)		119 (59-185)	
2013	Saint Kitts and Nevis									3 (1-4)		54 (27-84)		3 (1-5)		62 (30-96)		8 (4-12)		164 (80-254)	
2014	Saint Kitts and Nevis									2 (1-2)		33 (16-52)		2 (1-3)		38 (19-59)		5 (2-7)		100 (49-156)	
2015	Saint Kitts and Nevis	42 (36-50)	0 (0-0)	917 (791-1089)	0 (0-0)	16 (12-19)	0 (0-0)	359 (269-409)	0 (0-0)	2 (1-2)	0 (0-0)	34 (17-52)	0 (0-0)	2 (1-3)	0 (0-0)	38 (19-59)	0 (0-0)	5 (2-7)	0 (0-0)	102 (50-158)	0 (0-0)
2000	Saint Lucia	227 (196-270)		1459 (1259-1734)		88 (66-101)		568 (425-647)		1 (1-2)		9 (4-14)		2 (1-2)		10 (5-16)		4 (2-7)		28 (14-43)	
2001	Saint Lucia									1 (0-1)		6 (3-10)		1 (1-2)		7 (3-11)		3 (1-4)		19 (9-29)	
2002	Saint Lucia									2 (1-2)		11 (5-16)		2 (1-3)		12 (6-19)		5 (2-7)		32 (16-50)	
2003	Saint Lucia									2 (1-3)		11 (6-17)		2 (1-3)		13 (6-20)		5 (2-8)		34 (17-52)	
2004	Saint Lucia									2 (1-2)		11 (5-17)		2 (1-3)		13 (6-19)		5 (2-7)		33 (16-52)	
2005	Saint Lucia									0 (0-0)		0 (0-0)		0 (0-0)		0 (0-0)		0 (0-0)		0 (0-0)	
2006	Saint Lucia									1 (0-1)		4 (2-6)		1 (0-1)		5 (2-7)		2 (1-3)		13 (6-20)	
2007	Saint Lucia									1 (0-1)		4 (2-7)		1 (0-1)		5 (2-8)		2 (1-3)		13 (7-21)	
2008	Saint Lucia									1 (0-1)		4 (2-6)		1 (0-1)		5 (2-7)		2 (1-3)		13 (6-19)	
2009	Saint Lucia									3 (1-4)		18 (9-28)		3 (1-4)		20 (10-32)		8 (4-12)		54 (27-84)	
2010	Saint Lucia									2 (1-4)		17 (8-26)		3 (1-4)		19 (9-30)		7 (4-11)		51 (25-80)	
2011	Saint Lucia									2 (1-4)		18 (9-28)		3 (1-4)		20 (10-31)		7 (4-12)		54 (26-83)	
2012	Saint Lucia									1 (0-1)		5 (3-8)		1 (0-1)		6 (3-9)		2 (1-3)		16 (8-25)	
2013	Saint Lucia									2 (1-2)		11 (5-17)		2 (1-3)		12 (6-19)		5 (2-7)		33 (16-51)	
2014	Saint Lucia									2 (1-4)		17 (8-26)		3 (1-4)		19 (9-29)		7 (3-11)		50 (25-78)	
2015	Saint Lucia	126 (108-149)	0 (0-0)	929 (801-1104)	0 (0-0)	49 (37-56)	0 (0-0)	362 (271-412)	0 (0-0)	2 (1-3)	0 (0-0)	16 (8-24)	0 (0-0)	2 (1-4)	0 (0-0)	18 (9-28)	0 (0-0)	6 (3-10)	0 (0-0)	47 (23-73)	0 (0-0)
2000	Saint Vincent and the Grenadines	157 (135-186)		1458 (1258-1733)		61 (46-70)		567 (425-647)		2 (1-3)		15 (8-24)		2 (1-3)		17 (9-27)		5 (2-8)		46 (23-72)	
2001	Saint Vincent and the Grenadines									1 (1-2)		10 (5-16)		1 (1-2)		12 (6-18)		3 (2-5)		31 (15-48)	
2002	Saint Vincent and the Grenadines									0 (0-0)		0 (0-0)		0 (0-0)		0 (0-0)		0 (0-0)		0 (0-0)	
2003	Saint Vincent and the Grenadines									0 (0-0)		0 (0-0)		0 (0-0)		0 (0-0)		0 (0-0)		0 (0-0)	
2004	Saint Vincent and the Grenadines									0 (0-0)		0 (0-0)		0 (0-0)		0 (0-0)		0 (0-0)		0 (0-0)	
2005	Saint Vincent and the Grenadines									0 (0-0)		0 (0-0)		0 (0-0)		0 (0-0)		0 (0-0)		0 (0-0)	
2006	Saint Vincent and the Grenadines									0 (0-0)		0 (0-0)		0 (0-0)		0 (0-0)		0 (0-0)		0 (0-0)	
2007	Saint Vincent and the Grenadines									0 (0-0)		0 (0-0)		0 (0-0)		0 (0-0)		0 (0-0)		0 (0-0)	
2008	Saint Vincent and the Grenadines									0 (0-0)		0 (0-0)		0 (0-0)		0 (0-0)		0 (0-0)		0 (0-0)	
2009	Saint Vincent and the Grenadines									0 (0-0)		0 (0-0)		0 (0-0)		0 (0-0)		0 (0-0)		0 (0-0)	
2010	Saint Vincent and the Grenadines									0 (0-0)		0 (0-0)		0 (0-0)		0 (0-0)		0 (0-0)		0 (0-0)	
2011	Saint Vincent and the Grenadines									0 (0-0)		0 (0-0)		0 (0-0)		0 (0-0)		0 (0-0)		0 (0-0)	
2012	Saint Vincent and the Grenadines									0 (0-0)		0 (0-0)		0 (0-0)		0 (0-0)		0 (0-0)		0 (0-0)	
2013	Saint Vincent and the Grenadines									0 (0-0)		0 (0-0)		0 (0-0)		0 (0-0)		0 (0-0)		0 (0-0)	
2014	Saint Vincent and the Grenadines									0 (0-0)		0 (0-0)		0 (0-0)		0 (0-0)		0 (0-0)		0 (0-0)	
2015	Saint Vincent and the Grenadines	78 (67-92)	0 (0-0)	928 (801-1103)	0 (0-0)	30 (23-35)	0 (0-0)	363 (272-413)	0 (0-0)	0 (0-0)	0 (0-0)	0 (0-0)	0 (0-0)	0 (0-0)	0 (0-0)	0 (0-0)	0 (0-0)	0 (0-0)	0 (0-0)	0 (0-0)	0 (0-0)
2000	Samoa	499 (430-593)		1982 (1710-2356)		196 (147-223)		777 (582-887)		4 (2-6)		14 (6-26)		4 (2-7)		16 (7-29)		11 (5-19)		44 (19-77)	
2001	Samoa									3 (1-6)		13 (6-24)		4 (2-7)		15 (7-27)		10 (4-18)		41 (18-72)	
2002	Samoa									3 (1-5)		12 (6-22)		3 (2-6)		14 (6-25)		9 (4-16)		38 (17-66)	
2003	Samoa									3 (1-5)		12 (5-21)		3 (1-6)		13 (6-24)		9 (4-15)		35 (16-63)	
2004	Samoa									3 (1-5)		11 (5-19)		3 (1-5)		12 (5-22)		8 (4-14)		32 (14-57)	
2005	Samoa									2 (1-4)		10 (4-18)		3 (1-5)		11 (5-20)		7 (3-13)		30 (13-53)	
2006	Samoa									2 (1-4)		9 (4-16)		3 (1-5)		10 (5-18)		7 (3-12)		27 (12-48)	
2007	Samoa									2 (1-4)		9 (4-16)		3 (1-4)		10 (4-18)		7 (3-12)		27 (12-47)	
2008	Samoa									3 (1-5)		11 (5-19)		3 (1-5)		12 (5-21)		8 (4-14)		32 (14-56)	
2009	Samoa									3 (2-6)		13 (6-24)		4 (2-7)		15 (7-27)		10 (5-18)		41 (18-72)	
2010	Samoa									5 (2-9)		19 (9-34)		6 (2-10)		22 (10-39)		15 (7-26)		58 (26-103)	
2011	Samoa									5 (2-9)		19 (9-34)		6 (2-10)		22 (10-38)		15 (7-26)		58 (26-102)	
2012	Samoa									5 (2-9)		19 (8-34)		5 (2-10)		22 (10-38)		15 (7-26)		57 (26-101)	
2013	Samoa									4 (2-8)		18 (8-31)		5 (2-9)		20 (9-35)		13 (6-23)		53 (24-94)	
2014	Samoa									4 (2-7)		17 (8-30)		5 (2-8)		19 (9-34)		12 (6-22)		52 (23-91)	
2015	Samoa	267 (230-317)	0 (0-0)	1122 (968-1333)	0 (0-0)	103 (77-118)	0 (0-0)	435 (326-496)	0 (0-0)	4 (2-7)	0 (0-0)	16 (7-28)	0 (0-0)	4 (2-8)	0 (0-0)	18 (8-32)	0 (0-0)	11 (5-20)	0 (0-0)	48 (21-84)	0 (0-0)
2000	San Marino	1 (1-1)		115 (99-137)		1 (1-1)		75 (56-85)		0 (0-0)		9 (4-16)		0 (0-0)		10 (5-18)		0 (0-0)		27 (12-48)	
2001	San Marino									0 (0-0)		8 (4-15)		0 (0-0)		9 (4-17)		0 (0-0)		24 (11-44)	
2002	San Marino									0 (0-0)		7 (3-12)		0 (0-0)		8 (3-14)		0 (0-0)		20 (9-37)	
2003	San Marino									0 (0-0)		6 (3-10)		0 (0-0)		6 (3-11)		0 (0-0)		17 (8-30)	
2004	San Marino									0 (0-0)		5 (2-9)		0 (0-0)		5 (2-10)		0 (0-0)		14 (7-26)	
2005	San Marino									0 (0-0)		4 (2-8)		0 (0-0)		5 (2-9)		0 (0-0)		13 (6-23)	
2006	San Marino									0 (0-0)		4 (2-7)		0 (0-0)		5 (2-8)		0 (0-0)		12 (6-22)	
2007	San Marino									0 (0-0)		4 (2-7)		0 (0-0)		4 (2-8)		0 (0-0)		12 (5-22)	
2008	San Marino									0 (0-0)		4 (2-7)		0 (0-0)		5 (2-8)		0 (0-0)		12 (5-22)	
2009	San Marino									0 (0-0)		4 (2-7)		0 (0-0)		5 (2-8)		0 (0-0)		12 (6-22)	
2010	San Marino									0 (0-0)		4 (2-8)		0 (0-0)		5 (2-9)		0 (0-0)		12 (6-23)	
2011	San Marino									0 (0-0)		4 (2-8)		0 (0-0)		5 (2-9)		0 (0-0)		13 (6-23)	
2012	San Marino									0 (0-0)		4 (2-8)		0 (0-0)		5 (2-9)		0 (0-0)		13 (6-23)	

Spn = *Streptococcus pneumoniae*; npnm = non-pneumonia, non-meningitis; HIV = cases only in HIV-infected children; Uncertainty range provided in parentheses.

Year	Country	Spn pneumonia cases (UR)	Spn pneumonia cases, HIV (UR)	Spn pneumonia incidence rate (UR)	Spn pneumonia incidence rate, HIV (UR)	Spn severe pneumonia cases (UR)	Spn severe pneumonia cases, HIV (UR)	Spn severe pneumonia incidence rate (UR)	Spn severe pneumonia incidence rate, HIV (UR)	Spn meningitis cases (UR)	Spn meningitis cases, HIV (UR)	Spn meningitis incidence rate (UR)	Spn meningitis incidence rate, HIV (UR)	Spn NPNM cases (UR)	Spn NPNM cases, HIV (UR)	Spn NPNM incidence rate (UR)	Spn NPNM incidence rate, HIV (UR)	Spn NPNM non-severe cases (UR)	Spn NPNM non-severe cases, HIV (UR)	SPN NPNM non-severe incidence rate (UR)	SPN NPNM non-severe incidence rate, HIV (UR)
2013	San Marino									0 (0-0)		4 (2-8)		0 (0-0)		5 (2-9)		0 (0-0)		13 (6-23)	
2014	San Marino									0 (0-0)		4 (2-7)		0 (0-0)		5 (2-8)		0 (0-0)		12 (6-22)	
2015	San Marino	1 (1-2)	0 (0-0)	115 (99-137)	0 (0-0)	1 (1-1)	0 (0-0)	75 (56-85)	0 (0-0)	0 (0-0)	0 (0-0)	4 (2-7)	0 (0-0)	0 (0-0)	0 (0-0)	4 (2-8)	0 (0-0)	0 (0-0)	0 (0-0)	12 (5-22)	0 (0-0)
2000	Sao Tome and Principe	539 (465-641)		2441 (2107-2902)		212 (159-242)		960 (719-1095)		8 (3-16)		34 (14-72)		3 (1-6)		14 (5-29)		23 (9-48)		103 (41-217)	
2001	Sao Tome and Principe									8 (3-16)		33 (14-69)		3 (1-6)		13 (5-28)		23 (9-48)		100 (41-210)	
2002	Sao Tome and Principe									8 (3-16)		33 (14-67)		3 (1-6)		13 (6-27)		23 (10-48)		99 (42-204)	
2003	Sao Tome and Principe									8 (3-16)		32 (14-66)		3 (1-6)		13 (6-26)		24 (10-48)		97 (43-199)	
2004	Sao Tome and Principe									8 (3-16)		32 (13-66)		9 (4-19)		36 (14-75)		24 (9-50)		97 (38-200)	
2005	Sao Tome and Principe									8 (3-17)		32 (13-65)		9 (4-19)		36 (15-74)		25 (10-50)		96 (39-196)	
2006	Sao Tome and Principe									8 (4-17)		32 (14-64)		9 (4-19)		36 (15-72)		25 (11-50)		96 (41-192)	
2007	Sao Tome and Principe									8 (4-16)		31 (14-62)		9 (4-18)		36 (16-70)		25 (11-49)		95 (42-186)	
2008	Sao Tome and Principe									9 (4-17)		32 (15-62)		10 (5-19)		36 (17-70)		26 (12-50)		97 (45-187)	
2009	Sao Tome and Principe									8 (4-16)		30 (14-58)		9 (4-18)		34 (16-66)		25 (11-47)		91 (43-175)	
2010	Sao Tome and Principe									8 (4-15)		30 (14-57)		9 (4-18)		34 (16-65)		24 (11-47)		90 (42-172)	
2011	Sao Tome and Principe									8 (4-15)		29 (14-55)		9 (4-17)		33 (16-63)		24 (11-46)		88 (41-166)	
2012	Sao Tome and Principe									8 (4-15)		28 (13-53)		9 (4-17)		32 (15-60)		23 (11-44)		85 (40-161)	
2013	Sao Tome and Principe									5 (2-9)		16 (8-31)		5 (2-10)		19 (9-35)		14 (7-26)		49 (24-93)	
2014	Sao Tome and Principe									4 (2-7)		13 (6-25)		4 (2-8)		15 (7-28)		11 (5-22)		40 (19-75)	
2015	Sao Tome and Principe	227 (196-270)	3 (2-3)	782 (674-929)	9 (8-11)	89 (67-102)	1 (1-1)	307 (230-350)	4 (3-4)	3 (2-6)	0 (0-0)	12 (6-22)	0 (0-0)	4 (2-7)	0 (0-0)	13 (6-25)	0 (0-0)	10 (5-19)	0 (0-0)	35 (17-66)	0 (0-1)
2000	Saudi Arabia	63385 (54690-75347)		2298 (1983-2732)		24877 (18638-28374)		902 (676-1029)		406 (244-660)		15 (9-20)		462 (277-637)		17 (10-23)		1227 (737-1692)		44 (27-61)	
2001	Saudi Arabia									377 (226-520)		14 (8-19)		428 (257-590)		16 (9-21)		1138 (683-1569)		41 (25-57)	
2002	Saudi Arabia									562 (337-775)		21 (12-28)		638 (383-880)		23 (14-32)		1696 (1018-2339)		62 (37-85)	
2003	Saudi Arabia									547 (328-754)		20 (12-28)		621 (373-857)		23 (14-31)		1652 (991-2278)		61 (36-84)	
2004	Saudi Arabia									513 (308-708)		19 (11-26)		583 (350-804)		21 (13-30)		1550 (930-2138)		57 (34-79)	
2005	Saudi Arabia									492 (296-679)		18 (11-25)		559 (336-771)		21 (12-28)		1487 (892-2050)		55 (33-75)	
2006	Saudi Arabia									470 (282-648)		17 (10-24)		534 (320-736)		19 (12-27)		1419 (851-1957)		52 (31-71)	
2007	Saudi Arabia									468 (281-646)		17 (10-23)		532 (319-734)		19 (12-26)		1415 (849-1951)		51 (31-70)	
2008	Saudi Arabia									451 (271-622)		16 (10-22)		512 (308-707)		18 (11-25)		1362 (818-1879)		48 (29-66)	
2009	Saudi Arabia									383 (230-528)		13 (8-18)		434 (261-599)		15 (9-21)		1155 (693-1593)		40 (24-55)	
2010	Saudi Arabia									307 (184-424)		10 (6-14)		349 (209-481)		12 (7-16)		928 (557-1279)		32 (19-44)	
2011	Saudi Arabia									239 (144-330)		8 (5-11)		272 (163-375)		9 (5-12)		723 (434-997)		24 (14-33)	
2012	Saudi Arabia									220 (132-304)		7 (4-10)		250 (150-345)		8 (5-11)		665 (399-917)		22 (13-30)	
2013	Saudi Arabia									164 (98-226)		5 (3-7)		186 (111-256)		6 (4-8)		494 (296-681)		16 (10-22)	
2014	Saudi Arabia									147 (88-203)		5 (3-7)		167 (100-231)		5 (3-7)		445 (267-613)		14 (9-20)	
2015	Saudi Arabia	10474 (9037-12451)	0 (0-0)	337 (291-401)	0 (0-0)	4085 (3060-4659)	0 (0-0)	131 (98-150)	0 (0-0)	137 (82-189)	0 (0-0)	4 (3-6)	0 (0-0)	156 (94-215)	0 (0-0)	5 (3-7)	0 (0-0)	414 (249-571)	0 (0-0)	13 (8-18)	0 (0-0)
2000	Senegal	35282 (30442-41941)		2131 (1839-2533)		13878 (10398-15830)		838 (628-956)		662 (228-1496)		40 (14-90)		266 (91-600)		16 (6-36)		1999 (688-4516)		121 (42-273)	
2001	Senegal									681 (242-1525)		40 (14-90)		273 (97-612)		16 (6-36)		2058 (731-4605)		121 (43-271)	
2002	Senegal									678 (248-1502)		39 (14-86)		272 (100-602)		16 (6-35)		2048 (750-4536)		118 (43-261)	
2003	Senegal									673 (254-1472)		38 (14-83)		270 (102-591)		15 (6-33)		2032 (767-4446)		114 (43-249)	
2004	Senegal									667 (260-1440)		36 (14-79)		268 (104-578)		15 (6-32)		2014 (785-4349)		110 (43-238)	
2005	Senegal									654 (264-1394)		35 (14-74)		262 (106-559)		14 (6-30)		1974 (796-4210)		105 (42-224)	
2006	Senegal									636 (257-1350)		33 (13-70)		255 (103-542)		13 (5-28)		1920 (777-4078)		99 (40-210)	
2007	Senegal									609 (248-1291)		31 (12-65)		244 (99-518)		12 (5-26)		1840 (748-3899)		92 (38-196)	
2008	Senegal									581 (237-1229)		28 (12-60)		233 (95-493)		11 (5-24)		1753 (717-3712)		86 (35-182)	
2009	Senegal									554 (192-1209)		26 (9-58)		630 (219-1373)		30 (10-66)		1674 (581-3651)		80 (28-174)	
2010	Senegal									533 (186-1161)		25 (9-54)		605 (212-1319)		28 (10-61)		1609 (563-3507)		74 (26-162)	
2011	Senegal									509 (178-1110)		23 (8-50)		578 (202-1261)		26 (9-57)		1536 (538-3352)		69 (24-150)	
2012	Senegal									486 (177-1056)		21 (8-46)		553 (201-1200)		24 (9-52)		1469 (535-3190)		63 (23-138)	
2013	Senegal									468 (171-1019)		19 (7-42)		532 (194-1157)		22 (8-48)		1414 (516-3077)		59 (21-128)	
2014	Senegal									293 (95-653)		12 (4-26)		333 (107-742)		13 (4-30)		884 (285-1972)		36 (11-79)	
2015	Senegal	29710 (25635-35317)	402 (347-478)	1161 (1002-1381)	16 (14-19)	11614 (8701-13247)	157 (118-179)	454 (340-518)	6 (5-7)	226 (74-504)	3 (1-7)	9 (3-20)	0 (0-0)	256 (84-572)	3 (1-8)	10 (3-22)	0 (0-0)	682 (223-1522)	9 (3-21)	27 (9-59)	0 (0-1)
2000	Serbia	1403 (1210-1667)		230 (198-273)		909 (681-1037)		149 (112-170)		31 (15-68)		5 (2-11)		35 (17-78)		6 (3-13)		92 (45-207)		15 (7-34)	
2001	Serbia									25 (12-57)		4 (2-9)		28 (13-65)		5 (2-11)		75 (36-172)		12 (6-29)	
2002	Serbia									14 (6-32)		2 (1-6)		15 (7-37)		3 (1-6)		41 (19-97)		7 (3-17)	
2003	Serbia									20 (9-49)		4 (2-9)		23 (10-56)		4 (2-10)		61 (27-148)		11 (5-26)	
2004	Serbia									16 (7-40)		3 (1-7)		18 (8-45)		3 (1-8)		48 (21-120)		9 (4-22)	
2005	Serbia									22 (9-56)		4 (2-11)		24 (11-63)		5 (2-12)		65 (28-168)		12 (5-32)	
2006	Serbia									12 (5-33)		2 (1-6)		14 (6-37)		3 (1-7)		37 (16-98)		7 (3-19)	
2007	Serbia									16 (7-44)		3 (1-9)		18 (8-50)		4 (2-10)		49 (20-134)		10 (4-27)	
2008	Serbia									5 (2-13)		1 (0-3)		5 (2-15)		1 (0-3)		15 (6-41)		3 (1-8)	
2009	Serbia									12 (5-35)		3 (1-7)		14 (6-40)		3 (1-8)		37 (15-106)		8 (3-22)	
2010	Serbia									4 (1-11)		1 (0-2)		4 (2-13)		1 (0-3)		11 (4-33)		2 (1-7)	
2011	Serbia									9 (4-28)		2 (1-6)		10 (4-31)		2 (1-7)		28 (11-83)		6 (2-18)	
2012	Serbia									2 (1-6)		0 (0-1)		2 (1-7)		1 (0-2)		6 (2-19)		1 (1-4)	
2013	Serbia									4 (2-13)		1 (0-3)		5 (2-15)		1 (0-3)		13 (5-39)		3 (1-9)	
2014	Serbia									5 (2-15)		1 (0-3)		5 (2-17)		1 (0-4)		14 (5-45)		3 (1-10)	
2015	Serbia	1020 (880-1212)	0 (0-0)	230 (198-273)	0 (0-0)	661 (495-754)	0 (0-0)	149 (112-170)	0 (0-0)	5 (2-15)	0 (0-0)	1 (0-3)	0 (0-0)	5 (2-17)	0 (0-0)	1 (0-4)	0 (0-0)	14 (5-45)	0 (0-0)	3 (1-10)	0 (0-0)
2000	Seychelles	155 (133-184)		2146 (1852-2551)		61 (46-69)		845 (633-964)		1 (0-1)		10 (6-14)		1 (1-1)		12 (7-16)		2 (1-3)		31 (19-41)	
2001	Seychelles									1 (1-1)		12 (7-16)		1 (1-1)		13 (8-18)		2 (2-3)		35 (22-47)	
2002	Seychelles									1 (0-1)		11 (7-15)		1 (1-1)		13 (8-17)		2 (2-3)		33 (21-45)	
2003	Seychelles									1 (0-1)		10 (7-14)		1 (1-1)		12 (7-16)		2 (1-3)		32 (20-43)	
2004	Seychelles									1 (0-1)		10 (6-13)		1 (1-1)		11 (7-15)		2 (1-3)		30 (19-40)	
2005	Seychelles									1 (1-1)		10 (7-14)		1 (1-1)		12 (8-16)		2 (2-3)		32 (20-43)	
2006	Seychelles									1 (0-1)		10 (6-13)		1 (1-1)		11 (7-15)		2 (1-3)		30 (19-41)	
2007	Seychelles									1 (0-1)		10 (6-13)		1 (1-1)		11 (7-15)		2 (1-3)		29 (18-39)	
2008	Seychelles									1 (0-1)		10 (6-13)		1 (1-1)		11 (7-15)		2 (1-3)		29 (18-39)	
2009	Seychelles									1 (0-1)		9 (6-13)		1 (1-1)		11 (7-15)		2 (1-3)		29 (18-39)	
2010	Seychelles									1 (1-1)		14 (9-18)		1 (1-2)		15 (10-21)		3 (2-4)		41 (26-55)	
2011	Seychelles									1 (1-2)		15 (9-20)		1 (1-2)		17 (11-23)		4 (2-5)		45 (28-60)	
2012	Seychelles									1 (1-1)		13 (8-17)		1 (1-2)		14 (9-19)		3 (2-4)		38 (24-52)	
2013	Seychelles									1 (1-1)		14 (9-19)		1 (1-2)		16 (10-21)		3 (2-5)		42 (26-56)	
2014	Seychelles									1 (1-1)		13 (8-18)		1 (1-2)		15 (10-21)		3 (2-4)		40 (26-55)	
2015	Seychelles	137 (118-163)	0 (0-0)	1660 (1432-1973)	0 (0-0)	54 (40-61)	0 (0-0)	651 (488-742)	0 (0-0)	1 (1-1)	0 (0-0)	13 (8-18)	0 (0-0)	1 (1-2)	0 (0-0)	15 (10-20)	0 (0-0)	3 (2-4)	0 (0-0)	40 (25-54)	0 (0-0)
2000	Sierra Leone	20359 (17566-24201)		2923 (2522-3475)		7974 (5974-9096)		1145 (858-1306)		535 (303-960)		77 (44-138)		215 (122-385)		31 (17-55)		1617 (916-2900)		232 (131-416)	
2001	Sierra Leone									543 (307-975)		75 (42-134)		218 (123-391)		30 (17-54)		1640 (927-2945)		225 (127-405)	
2002	Sierra Leone									553 (312-994)		73 (41-131)		222 (125-399)		29 (16-52)		1669 (941-3001)		219 (124-394)	
2003	Sierra Leone									567 (319-1021)		71 (40-128)		227 (128-409)		29 (16-51)		1712 (964-3083)		215 (121-386)	
2004	Sierra Leone									580 (326-1047)		70 (39-126)		233 (131-420)		28 (16-50)		1753 (985-3161)		211 (118-380)	
2005	Sierra Leone									582 (327-1051)		68 (38-122)		233 (131-422)		27 (15-49)		1757 (987-3175)		204 (115-369)	
2006	Sierra Leone									583 (325-1053)		66 (37-118)		234 (131-422)		26 (15-47)		1760 (983-3180)		198 (110-357)	
2007	Sierra Leone									572 (316-1029)		63 (35-113)		230 (127-413)		25 (14-45)		1728 (955-3108)		190 (105-341)	
2008	Sierra Leone									558 (304-993)		60 (33-107)		224 (122-398)		24 (13-43)		1686 (917-3000)		182 (99-323)	
2009	Sierra Leone									556 (328-957)		59 (35-102)		223 (132-384)		24 (14-41)		1679 (990-2888)		179 (105-308)	
2010	Sierra Leone									563 (364-935)		59 (38-99)		226 (146-375)		24 (15-40)		1700 (1098-2824)		179 (116-298)	
2011	Sierra Leone									399 (255-659)		41 (26-68)		160 (102-264)		17 (11-27)		1205 (769-1989)		125 (80-206)	
2012	Sierra Leone									326 (160-598)		33 (17-62)		131 (64-240)		13 (7-25)		983 (485-1806)		101 (50-186)	
2013	Sierra Leone									301 (148-557)		31 (15-57)		121 (59-223)		12 (6-23)		908 (446-1682)		93 (46-172)	
2014	Sierra Leone									308 (151-575)		31 (15-59)		123 (61-231)		13 (6-24)		930 (456-1738)		95 (47-177)	
2015	Sierra Leone	6617 (5710-7866)	268 (232-319)	670 (578-797)	27 (23-32)	2590 (1940-2954)	105 (79-120)	262 (196-299)	11 (8-12)	207 (101-389)	8 (4-16)	21 (10-39)	1 (0-2)	83 (41-156)	3 (2-6)	8 (4-16)	0 (0-1)	624 (305-1175)	25 (12-48)	63 (31-119)	3 (1-5)
2000	Singapore	579 (499-688)		230 (198-273)		375 (281-428)		149 (112-170)		23 (14-32)		9 (5-13)		26 (16-37)		11 (6-15)		70 (42-97)		28 (17-39)	
2001	Singapore									9 (6-13)		4 (2-5)		11 (6-15)		4 (3-6)		28 (17-39)		12 (7-16)	
2002	Singapore									14 (8-19)		6 (3-8)		15 (9-21)		6 (4-9)		41 (24-56)		17 (10-23)	
2003	Singapore									0 (0-0)		0 (0-0)		0 (0-0)		0 (0-0)		0 (0-0)		0 (0-0)	
2004	Singapore									5 (3-7)		2 (1-3)		6 (3-8)		2 (1-3)		15 (9-20)		6 (4-8)	
2005	Singapore									6 (3-8)		2 (1-3)		6 (4-9)		3 (2-4)		17 (10-24)		7 (4-9)	
2006	Singapore									0 (0-0)		0 (0-0)		0 (0-0)		0 (0-0)		0 (0-0)		0 (0-0)	
2007	Singapore									6 (4-9)		2 (1-3)		7 (4-10)		3 (2-4)		19 (11-26)		8 (5-10)	
2008	Singapore									6 (4-9)		2 (1-3)		7 (4-10)		3 (2-4)		19 (11-26)		7 (4-10)	
2009	Singapore									11 (7-15)		4 (3-6)		12 (7-17)		5 (3-7)		33 (20-46)		13 (8-18)	
2010	Singapore									0 (0-0)		0 (0-0)		0 (0-0)		0 (0-0)		0 (0-0)		0 (0-0)	
2011	Singapore									8 (5-11)		3 (2-4)		9 (6-13)		4 (2-5)		25 (15-34)		10 (6-13)	
2012	Singapore									0 (0-0)		0 (0-0)		0 (0-0)		0 (0-0)		0 (0-0)		0 (0-0)	
2013	Singapore									0 (0-0)		0 (0-0)		0 (0-0)		0 (0-0)		0 (0-0)		0 (0-0)	
2014	Singapore									3 (2-4)		1 (1-1)		3 (2-4)		1 (1-2)		8 (5-11)		3 (2-4)	
2015	Singapore	398 (343-473)	1 (1-1)	151 (130-179)	0 (0-0)	258 (193-294)	1 (0-1)	98 (73-111)	0 (0-0)	3 (2-4)	0 (0-0)	1 (1-1)	0 (0-0)	3 (2-4)	0 (0-0)	1 (1-2)	0 (0-0)	8 (5-11)	0 (0-0)	3 (2-4)	0 (0-0)
2000	Slovakia	647 (558-769)		230 (198-273)		419 (314-478)		149 (112-170)		24 (11-44)		9 (4-16)		28 (13-50)		10 (4-18)		74 (33-134)		26 (12-48)	
2001	Slovakia									41 (19-74)		15 (7-28)		46 (21-84)		17 (8-31)		123 (56-223)		46 (21-84)	
2002	Slovakia									16 (7-28)		6 (3-11)		18 (8-32)		7 (3-12)		47 (21-85)		18 (8-33)	
2003	Slovakia									26 (12-47)		10 (5-18)		29 (13-53)		11 (5-21)		78 (35-141)		30 (14-55)	
2004	Slovakia									15 (7-27)		6 (3-11)		17 (8-31)		7 (3-12)		45 (20-82)		18 (8-32)	
2005	Slovakia									18 (8-32)		7 (3-13)		20 (9-36)		8 (4-14)		53 (24-96)		21 (9-38)	
2006	Slovakia									16 (7-29)		6 (3-11)		18 (8-33)		7 (3-13)		49 (22-88)		19 (9-34)	
2007	Slovakia									10 (5-18)		4 (2-7)		11 (5-21)		4 (2-8)		30 (14-55)		12 (5-21)	
2008	Slovakia									29 (13-53)		11 (5-20)		33 (15-60)		12 (6-23)		89 (40-161)		33 (15-60)	
2009	Slovakia									13 (6-24)		5 (2-9)		15 (7-27)		6 (3-10)		40 (18-73)		15 (7-27)	
2010	Slovakia									8 (4-15)		3 (1-6)		10 (4-17)		3 (2-6)		25 (12-46)		9 (4-17)	
2011	Slovakia									7 (3-12)		2 (1-4)		8 (3-14)		3 (1-5)		20 (9-36)		7 (3-13)	
2012	Slovakia									8 (4-15)		3 (1-5)		9 (4-17)		3 (1-6)		25 (11-45)		9 (4-16)	
2013	Slovakia									1 (1-2)		0 (0-1)		1 (1-2)		0 (0-1)		4 (2-7)		1 (1-2)	
2014	Slovakia									6 (3-10)		2 (1-4)		6 (3-12)		2 (1-4)		17 (8-31)		6 (3-11)	
2015	Slovakia	221 (190-262)	0 (0-0)	79 (68-94)	0 (0-0)	143 (107-163)	0 (0-0)	51 (39-59)	0 (0-0)	5 (2-9)	0 (0-0)	2 (1-3)	0 (0-0)	5 (2-10)	0 (0-0)	2 (1-4)	0 (0-0)	14 (7-26)	0 (0-0)	5 (2-9)	0 (0-0)
2000	Slovenia	102 (88-122)		115 (99-137)		66 (50-76)		75 (56-85)		0 (0-0)		0 (0-0)		0 (0-0)		0 (0-0)		0 (0-0)		0 (0-0)	
2001	Slovenia									0 (0-0)		0 (0-0)		0 (0-0)		0 (0-0)		0 (0-0)		0 (0-0)	
2002	Slovenia									7 (3-13)		8 (4-15)		8 (4-15)		9 (4-17)		22 (10-40)		25 (11-45)	
2003	Slovenia									0 (0-0)		0 (0-0)		0 (0-0)		0 (0-0)		0 (0-0)		0 (0-0)	
2004	Slovenia									0 (0-0)		0 (0-0)		0 (0-0)		0 (0-0)		0 (0-0)		0 (0-0)	
2005	Slovenia									3 (1-5)		3 (1-5)		3 (1-5)		3 (2-6)		8 (4-14)		9 (4-17)	
2006	Slovenia									0 (0-0)		0 (0-0)		0 (0-0)		0 (0-0)		0 (0-0)		0 (0-0)	
2007	Slovenia									0 (0-0)		0 (0-0)		0 (0-0)		0 (0-0)		0 (0-0)		0 (0-0)	
2008	Slovenia									0 (0-0)		0 (0-0)		0 (0-0)		0 (0-0)		0 (0-0)		0 (0-0)	
2009	Slovenia									0 (0-0)		0 (0-0)		0 (0-0)		0 (0-0)		0 (0-0)		0 (0-0)	
2010	Slovenia									0 (0-0)		0 (0-0)		0 (0-0)		0 (0-0)		0 (0-0)		0 (0-0)	
2011	Slovenia									0 (0-0)		0 (0-0)		0 (0-0)		0 (0-0)		0 (0-0)		0 (0-0)	
2012	Slovenia									0 (0-0)		0 (0-0)		0 (0-0)		0 (0-0)		0 (0-0)		0 (0-0)	
2013	Slovenia									0 (0-0)		0 (0-0)		0 (0-0)		0 (0-0)		0 (0-0)		0 (0-0)	
2014	Slovenia									0 (0-0)		0 (0-0)		0 (0-0)		0 (0-0)		0 (0-0)		0 (0-0)	
2015	Slovenia	109 (94-130)	0 (0-0)	100 (86-119)	0 (0-0)	71 (53-81)	0 (0-0)	65 (49-74)	0 (0-0)	0 (0-0)	0 (0-0)	0 (0-0)	0 (0-0)	0 (0-0)	0 (0-0)	0 (0-0)	0 (0-0)	0 (0-0)	0 (0-0)	0 (0-0)	0 (0-0)
2000	Solomon Islands	1264 (1091-1503)		1987 (1714-2362)		493 (370-563)		775 (581-884)		5 (1-14)		7 (2-21)		5 (2-15)		8 (3-24)		14 (4-41)		22 (7-64)	
2001	Solomon Islands									5 (2-14)		7 (2-22)		5 (2-16)		8 (3-25)		14 (5-43)		22 (7-66)	
2002	Solomon Islands									5 (2-15)		7 (2-22)		6 (2-17)		8 (3-25)		15 (5-44)		23 (7-66)	
2003	Solomon Islands									5 (2-15)		7 (2-22)		6 (2-17)		8 (3-25)		15 (5-45)		23 (7-67)	
2004	Solomon Islands									5 (2-16)		8 (2-23)		6 (2-18)		9 (3-26)		16 (5-47)		23 (7-68)	
2005	Solomon Islands									5 (2-16)		8 (2-22)		6 (2-18)		9 (3-26)		16 (5-48)		23 (7-68)	
2006	Solomon Islands									6 (2-16)		8 (2-22)		6 (2-19)		9 (3-26)		17 (5-49)		23 (7-68)	
2007	Solomon Islands									6 (2-16)		7 (2-22)		6 (2-19)		8 (3-25)		17 (5-49)		22 (7-66)	
2008	Solomon Islands									6 (2-16)		7 (2-21)		6 (2-18)		8 (3-24)		17 (5-49)		22 (7-65)	
2009	Solomon Islands									6 (2-16)		7 (2-21)		6 (2-18)		8 (3-24)		17 (5-49)		22 (7-64)	
2010	Solomon Islands									6 (2-16)		7 (2-21)		6 (2-18)		8 (3-24)		17 (5-49)		21 (7-63)	
2011	Solomon Islands									6 (2-16)		7 (2-21)		6 (2-19)		8 (3-24)		17 (6-50)		22 (7-63)	
2012	Solomon Islands									6 (2-17)		7 (2-21)		7 (2-19)		8 (3-24)		17 (6-51)		22 (7-64)	
2013	Solomon Islands									6 (1-25)		7 (2-31)		7 (2-28)		8 (2-35)		18 (4-75)		22 (6-93)	
2014	Solomon Islands									6 (1-25)		7 (2-31)		7 (2-28)		8 (2-35)		18 (5-75)		22 (6-93)	
2015	Solomon Islands	1006 (868-1195)	0 (0-0)	1251 (1080-1487)	0 (0-0)	393 (295-449)	0 (0-0)	490 (367-558)	0 (0-0)	5 (1-20)	0 (0-0)	6 (2-25)	0 (0-0)	5 (1-23)	0 (0-0)	7 (2-28)	0 (0-0)	15 (4-60)	0 (0-0)	18 (5-75)	0 (0-0)
2000	Somalia	63696 (54958-75716)		4410 (3805-5243)		24751 (18544-28231)		1714 (1284-1955)		885 (434-1795)		61 (30-124)		355 (174-720)		25 (12-50)		2672 (1310-5421)		185 (91-375)	
2001	Somalia									902 (442-1830)		60 (30-122)		362 (177-734)		24 (12-49)		2723 (1336-5526)		182 (89-369)	
2002	Somalia									939 (461-1906)		61 (30-124)		377 (185-765)		25 (12-50)		2837 (1391-5756)		185 (91-376)	
2003	Somalia									1006 (493-2042)		65 (32-131)		404 (198-819)		26 (13-53)		3038 (1490-6165)		195 (96-397)	
2004	Somalia									1036 (508-2102)		66 (32-134)		415 (204-843)		26 (13-54)		3128 (1534-6347)		199 (97-403)	
2005	Somalia									1073 (526-2177)		67 (33-136)		430 (211-873)		27 (13-55)		3239 (1589-6573)		202 (99-411)	

Spn = *Streptococcus pneumoniae*; npnm = non-pneumonia, non-meningitis; HIV = cases only in HIV-infected children; Uncertainty range provided in parentheses.

Year	Country	Spn pneumonia cases (UR)	Spn pneumonia cases, HIV (UR)	Spn pneumonia incidence rate (UR)	Spn pneumonia incidence rate, HIV (UR)	Spn severe pneumonia cases (UR)	Spn severe pneumonia cases, HIV (UR)	Spn severe pneumonia incidence rate (UR)	Spn severe pneumonia incidence rate, HIV (UR)	Spn meningitis cases (UR)	Spn meningitis cases, HIV (UR)	Spn meningitis incidence rate (UR)	Spn meningitis incidence rate, HIV (UR)	Spn NPNM cases (UR)	Spn NPNM cases, HIV (UR)	Spn NPNM incidence rate (UR)	Spn NPNM incidence rate, HIV (UR)	Spn NPNM non-severe cases (UR)	Spn NPNM non-severe cases, HIV (UR)	SPN NPNM non-severe incidence rate (UR)	SPN NPNM non-severe incidence rate, HIV (UR)
2006	Somalia									1110 (545-2253)		68 (33-138)		445 (218-904)		27 (13-56)		3353 (1644-6802)		206 (101-418)	
2007	Somalia									1149 (564-2331)		69 (34-140)		461 (226-935)		28 (14-56)		3471 (1703-7039)		209 (102-423)	
2008	Somalia									1178 (579-2389)		69 (34-140)		473 (232-958)		28 (14-56)		3558 (1747-7213)		209 (102-423)	
2009	Somalia									1198 (589-2427)		69 (34-139)		481 (236-974)		27 (14-56)		3618 (1778-7329)		207 (102-419)	
2010	Somalia									1207 (594-2445)		68 (33-137)		484 (238-981)		27 (13-55)		3646 (1795-7384)		204 (101-414)	
2011	Somalia									1202 (593-2435)		66 (32-133)		482 (238-977)		26 (13-53)		3629 (1791-7353)		198 (98-402)	
2012	Somalia									1184 (586-2401)		64 (32-129)		475 (235-963)		26 (13-52)		3575 (1769-7249)		192 (95-390)	
2013	Somalia									1165 (371-2725)		62 (20-145)		467 (149-1093)		25 (8-58)		3517 (1120-8227)		187 (60-438)	
2014	Somalia									1137 (363-2661)		60 (19-140)		456 (146-1067)		24 (8-56)		3433 (1097-8036)		181 (58-423)	
2015	Somalia	66153 (57078-78637)	2820 (2433-3352)	3413 (2945-4057)	146 (126-173)	25887 (19394-29526)	1104 (827-1259)	1336 (1001-1523)	57 (43-65)	1124 (360-2631)	48 (15-112)	58 (19-136)	2 (1-6)	451 (145-1055)	19 (6-45)	23 (7-54)	1 (0-2)	3393 (1088-7945)	145 (46-339)	175 (56-410)	7 (2-17)
2000	South Africa	172266 (148635-204776)		3235 (2792-3846)		67188 (50337-76635)		1262 (945-1439)		913 (429-1776)		17 (8-33)		366 (172-712)		7 (3-13)		2758 (1296-5363)		52 (24-101)	
2001	South Africa									904 (423-1760)		17 (8-33)		363 (170-706)		7 (3-13)		2730 (1278-5315)		51 (24-99)	
2002	South Africa									860 (401-1675)		16 (8-31)		345 (161-672)		6 (3-13)		2596 (1212-5057)		49 (23-95)	
2003	South Africa									802 (375-1563)		15 (7-30)		322 (150-627)		6 (3-12)		2422 (1131-4721)		46 (22-90)	
2004	South Africa									752 (353-1467)		15 (7-29)		302 (142-588)		6 (3-11)		2271 (1066-4430)		44 (21-86)	
2005	South Africa									734 (347-1434)		14 (7-28)		295 (139-575)		6 (3-11)		2217 (1047-4330)		43 (20-85)	
2006	South Africa									740 (308-1488)		15 (6-29)		841 (350-1691)		17 (7-33)		2235 (929-4494)		44 (18-89)	
2007	South Africa									775 (324-1558)		15 (6-30)		880 (368-1770)		17 (7-34)		2340 (980-4705)		45 (19-91)	
2008	South Africa									831 (351-1667)		16 (7-32)		944 (399-1893)		18 (8-36)		2510 (1060-5033)		47 (20-95)	
2009	South Africa									862 (370-1720)		16 (7-32)		979 (420-1953)		18 (8-36)		2604 (1117-5193)		48 (21-96)	
2010	South Africa									788 (349-1554)		14 (6-28)		895 (397-1765)		16 (7-32)		2379 (1054-4692)		43 (19-86)	
2011	South Africa									691 (310-1353)		12 (6-24)		785 (352-1536)		14 (6-27)		2086 (936-4084)		37 (17-73)	
2012	South Africa									508 (229-988)		9 (4-18)		576 (260-1123)		10 (5-20)		1533 (692-2985)		27 (12-53)	
2013	South Africa									488 (224-944)		9 (4-17)		554 (254-1073)		10 (5-20)		1474 (676-2852)		27 (12-52)	
2014	South Africa									443 (205-854)		8 (4-16)		503 (233-969)		9 (4-18)		1337 (618-2577)		25 (12-48)	
2015	South Africa	40805 (35207-48505)	21175 (18270-25171)	773 (667-919)	401 (346-477)	15994 (11982-18242)	8299 (6218-9466)	303 (227-345)	157 (118-179)	259 (120-497)	134 (62-258)	5 (2-9)	3 (1-5)	294 (136-565)	152 (71-293)	6 (3-11)	3 (1-6)	781 (362-1502)	405 (188-779)	15 (7-28)	8 (4-15)
2000	South Sudan	0 (0-0)		0 (0-0)		0 (0-0)		0 (0-0)		612 (329-1148)		52 (28-98)		246 (132-460)		21 (11-39)		1849 (992-3466)		158 (85-296)	
2001	South Sudan									616 (333-1155)		51 (27-95)		247 (134-463)		20 (11-38)		1862 (1006-3487)		153 (83-286)	
2002	South Sudan									602 (328-1128)		48 (26-90)		242 (132-452)		19 (11-36)		1818 (991-3406)		145 (79-272)	
2003	South Sudan									590 (325-1106)		46 (25-86)		237 (130-444)		18 (10-35)		1782 (981-3340)		139 (77-261)	
2004	South Sudan									584 (223-1252)		45 (17-95)		234 (90-502)		18 (7-38)		1764 (674-3782)		134 (51-288)	
2005	South Sudan									566 (219-1214)		42 (16-90)		227 (88-487)		17 (6-36)		1709 (662-3666)		126 (49-271)	
2006	South Sudan									559 (219-1198)		40 (16-86)		224 (88-481)		16 (6-34)		1687 (661-3618)		121 (47-259)	
2007	South Sudan									552 (219-1182)		38 (15-82)		222 (88-474)		15 (6-33)		1668 (660-3570)		115 (46-246)	
2008	South Sudan									566 (225-1206)		38 (15-80)		227 (90-484)		15 (6-32)		1708 (681-3642)		113 (45-242)	
2009	South Sudan									573 (230-1217)		37 (15-78)		230 (92-488)		15 (6-31)		1729 (693-3674)		110 (44-235)	
2010	South Sudan									589 (237-1247)		36 (15-77)		236 (95-500)		15 (6-31)		1777 (717-3765)		109 (44-231)	
2011	South Sudan									608 (247-1284)		36 (15-76)		244 (99-515)		14 (6-30)		1836 (745-3878)		108 (44-228)	
2012	South Sudan									605 (247-1273)		34 (14-72)		242 (99-511)		14 (6-29)		1826 (745-3844)		104 (42-218)	
2013	South Sudan									626 (257-1315)		34 (14-72)		251 (103-527)		14 (6-29)		1890 (776-3969)		104 (43-218)	
2014	South Sudan									627 (259-1313)		33 (14-70)		251 (104-527)		13 (6-28)		1893 (782-3965)		101 (42-211)	
2015	South Sudan	26689 (23028-31726)	5021 (4332-5968)	1388 (1197-1650)	261 (225-310)	10383 (7779-11843)	1953 (1463-2228)	540 (404-616)	102 (76-116)	624 (259-1304)	117 (49-245)	32 (13-68)	6 (3-13)	250 (104-523)	47 (20-98)	13 (5-27)	2 (1-5)	1884 (783-3939)	354 (147-741)	98 (41-205)	18 (8-39)
2000	Spain	2133 (1840-2536)		115 (99-137)		1383 (1036-1577)		75 (56-85)		197 (89-358)		11 (5-19)		224 (102-406)		12 (5-22)		595 (270-1080)		32 (15-58)	
2001	Spain									119 (54-217)		6 (3-12)		136 (62-246)		7 (3-13)		361 (164-654)		19 (9-35)	
2002	Spain									116 (53-211)		6 (3-11)		132 (60-240)		7 (3-12)		351 (159-638)		18 (8-33)	
2003	Spain									159 (72-288)		8 (4-14)		180 (82-327)		9 (4-16)		479 (217-869)		24 (11-44)	
2004	Spain									124 (56-225)		6 (3-11)		141 (64-256)		7 (3-12)		375 (170-680)		18 (8-33)	
2005	Spain									171 (78-311)		8 (4-14)		195 (88-353)		9 (4-16)		517 (235-938)		24 (11-44)	
2006	Spain									141 (64-255)		6 (3-11)		160 (73-290)		7 (3-13)		425 (193-771)		19 (9-34)	
2007	Spain									135 (61-245)		6 (3-11)		153 (70-279)		7 (3-12)		408 (185-740)		18 (8-32)	
2008	Spain									114 (52-207)		5 (2-9)		130 (59-235)		5 (2-10)		345 (156-625)		14 (7-26)	
2009	Spain									133 (60-242)		6 (3-10)		151 (69-275)		6 (3-11)		402 (183-730)		17 (8-30)	
2010	Spain									73 (33-133)		3 (1-6)		83 (38-151)		3 (2-6)		222 (101-402)		9 (4-17)	
2011	Spain									79 (36-144)		3 (2-6)		90 (41-164)		4 (2-7)		240 (109-435)		10 (5-18)	
2012	Spain									57 (26-103)		2 (1-4)		64 (29-117)		3 (1-5)		171 (78-311)		7 (3-13)	
2013	Spain									79 (36-143)		3 (2-6)		89 (41-162)		4 (2-7)		238 (108-432)		11 (5-19)	
2014	Spain									63 (29-114)		3 (1-5)		72 (32-130)		3 (1-6)		190 (86-345)		9 (4-16)	
2015	Spain	2425 (2092-2883)	1 (1-1)	115 (99-137)	0 (0-0)	1572 (1178-1793)	1 (0-1)	75 (56-85)	0 (0-0)	57 (26-103)	0 (0-0)	3 (1-5)	0 (0-0)	65 (29-117)	0 (0-0)	3 (1-6)	0 (0-0)	172 (78-312)	0 (0-0)	8 (4-15)	0 (0-0)
2000	Sri Lanka	29625 (25561-35216)		1835 (1583-2182)		11567 (8666-13193)		717 (537-817)		32 (8-84)		2 (0-5)		36 (9-95)		2 (1-6)		96 (23-252)		6 (1-16)	
2001	Sri Lanka									30 (7-78)		2 (0-5)		34 (8-89)		2 (1-6)		90 (22-237)		6 (1-15)	
2002	Sri Lanka									28 (7-75)		2 (0-5)		32 (8-85)		2 (0-5)		85 (21-225)		5 (1-14)	
2003	Sri Lanka									28 (7-73)		2 (0-4)		31 (8-83)		2 (0-5)		83 (21-220)		5 (1-13)	
2004	Sri Lanka									25 (5-68)		2 (0-4)		29 (6-77)		2 (0-5)		77 (17-206)		5 (1-12)	
2005	Sri Lanka									25 (6-67)		1 (0-4)		29 (7-76)		2 (0-4)		76 (19-202)		4 (1-12)	
2006	Sri Lanka									24 (6-62)		1 (0-4)		27 (7-71)		2 (0-4)		71 (18-187)		4 (1-11)	
2007	Sri Lanka									22 (6-58)		1 (0-3)		25 (6-66)		1 (0-4)		67 (17-176)		4 (1-10)	
2008	Sri Lanka									21 (5-54)		1 (0-3)		24 (6-62)		1 (0-3)		63 (16-164)		4 (1-9)	
2009	Sri Lanka									20 (5-51)		1 (0-3)		22 (6-58)		1 (0-3)		59 (15-154)		3 (1-9)	
2010	Sri Lanka									38 (10-98)		2 (1-6)		43 (11-112)		2 (1-6)		114 (30-297)		6 (2-17)	
2011	Sri Lanka									39 (10-100)		2 (1-6)		44 (11-114)		3 (1-7)		116 (30-302)		7 (2-17)	
2012	Sri Lanka									37 (10-95)		2 (1-6)		42 (11-108)		2 (1-6)		111 (29-287)		6 (2-17)	
2013	Sri Lanka									35 (9-90)		2 (1-5)		39 (10-102)		2 (1-6)		105 (27-271)		6 (2-16)	
2014	Sri Lanka									32 (8-83)		2 (1-5)		36 (10-94)		2 (1-6)		97 (25-250)		6 (2-15)	
2015	Sri Lanka	22742 (19623-27034)	27 (23-32)	1408 (1215-1673)	2 (1-2)	8987 (6733-10250)	11 (8-12)	556 (417-634)	1 (0-1)	32 (8-82)	0 (0-0)	2 (1-5)	0 (0-0)	36 (10-93)	0 (0-0)	2 (1-6)	0 (0-0)	96 (25-248)	0 (0-0)	6 (2-15)	0 (0-0)
2000	Sudan	68038 (58705-80878)		1444 (1246-1716)		26437 (19807-30154)		561 (420-640)		2539 (1305-4693)		54 (28-100)		1018 (523-1882)		22 (11-40)		7666 (3939-14172)		163 (84-301)	
2001	Sudan									2547 (1281-4752)		53 (27-99)		1021 (514-1906)		21 (11-40)		7690 (3868-14349)		160 (80-298)	
2002	Sudan									2470 (1218-4654)		50 (25-95)		991 (488-1867)		20 (10-38)		7458 (3677-14055)		152 (75-286)	
2003	Sudan									2389 (1156-4550)		47 (23-90)		958 (464-1825)		19 (9-36)		7214 (3491-13739)		143 (69-273)	
2004	Sudan									2283 (1086-4398)		44 (21-86)		916 (436-1764)		18 (8-34)		6895 (3280-13281)		134 (64-258)	
2005	Sudan									2118 (991-4125)		40 (19-79)		850 (397-1655)		16 (8-32)		6397 (2992-12456)		122 (57-238)	
2006	Sudan									2011 (925-3958)		38 (17-74)		807 (371-1588)		15 (7-30)		6074 (2794-11952)		114 (52-224)	
2007	Sudan									1905 (862-3786)		35 (16-70)		764 (346-1519)		14 (6-28)		5752 (2603-11433)		106 (48-211)	
2008	Sudan									1853 (825-3719)		34 (15-68)		743 (331-1492)		14 (6-27)		5596 (2492-11230)		102 (46-205)	
2009	Sudan									1750 (767-3546)		32 (14-64)		702 (308-1422)		13 (6-26)		5286 (2316-10707)		96 (42-194)	
2010	Sudan									1666 (719-3407)		30 (13-61)		668 (288-1367)		12 (5-25)		5030 (2171-10289)		90 (39-185)	
2011	Sudan									1613 (687-3333)		29 (12-59)		647 (275-1337)		12 (5-24)		4872 (2073-10065)		87 (37-180)	
2012	Sudan									1533 (643-3199)		27 (11-57)		615 (258-1283)		11 (5-23)		4628 (1943-9661)		82 (34-171)	
2013	Sudan									1328 (464-2883)		23 (8-50)		1508 (527-3274)		26 (9-57)		4010 (1402-8704)		70 (25-152)	
2014	Sudan									829 (285-1815)		14 (5-31)		942 (323-2061)		16 (6-36)		2503 (860-5479)		43 (15-95)	
2015	Sudan	47186 (40713-56091)	723 (624-860)	806 (696-958)	12 (11-15)	18532 (13884-21138)	284 (213-324)	317 (237-361)	5 (4-6)	709 (239-1563)	11 (4-24)	12 (4-27)	0 (0-0)	805 (272-1776)	12 (4-27)	14 (5-30)	0 (0-0)	2141 (722-4721)	33 (11-72)	37 (12-81)	1 (0-1)
2000	Suriname	724 (625-861)		1377 (1188-1637)		282 (211-321)		536 (401-611)		4 (1-10)		7 (2-19)		4 (1-11)		8 (2-21)		11 (3-30)		22 (6-57)	
2001	Suriname									3 (1-8)		6 (2-15)		3 (1-9)		6 (2-17)		9 (3-23)		17 (5-44)	
2002	Suriname									7 (2-18)		14 (4-36)		8 (3-21)		16 (5-41)		22 (7-56)		43 (13-109)	
2003	Suriname									2 (1-5)		4 (1-9)		2 (1-5)		4 (1-10)		5 (2-14)		11 (3-27)	
2004	Suriname									4 (1-9)		7 (2-18)		4 (1-10)		8 (3-20)		11 (4-27)		22 (7-54)	
2005	Suriname									2 (0-5)		3 (1-10)		2 (0-5)		4 (1-11)		5 (1-14)		10 (3-29)	
2006	Suriname									8 (2-24)		17 (5-50)		10 (3-28)		20 (5-57)		25 (7-73)		52 (14-151)	
2007	Suriname									2 (1-6)		5 (1-13)		3 (1-7)		5 (1-15)		7 (2-19)		14 (4-40)	
2008	Suriname									7 (2-19)		14 (4-39)		8 (2-21)		16 (4-44)		20 (5-57)		41 (11-118)	
2009	Suriname									8 (2-22)		16 (4-44)		9 (2-24)		18 (5-50)		23 (6-65)		47 (13-134)	
2010	Suriname									14 (4-39)		29 (8-81)		16 (4-44)		32 (9-91)		42 (12-118)		86 (24-243)	
2011	Suriname									7 (2-20)		15 (4-42)		8 (2-23)		17 (5-47)		22 (6-61)		45 (13-126)	
2012	Suriname									0 (0-0)		0 (0-0)		0 (0-0)		0 (0-0)		0 (0-0)		0 (0-0)	
2013	Suriname									6 (2-18)		13 (4-37)		7 (2-20)		15 (4-42)		19 (6-53)		40 (12-112)	
2014	Suriname									6 (2-17)		13 (4-36)		7 (2-19)		15 (4-41)		19 (5-52)		39 (11-109)	
2015	Suriname	440 (379-523)	3 (3-4)	928 (800-1103)	7 (6-8)	171 (128-196)	1 (1-1)	362 (271-413)	3 (2-3)	6 (2-17)	0 (0-0)	13 (4-35)	0 (0-0)	7 (2-19)	0 (0-0)	15 (4-40)	0 (0-0)	18 (5-50)	0 (0-0)	39 (11-106)	0 (0-1)
2000	Swaziland	5203 (4489-6185)		3372 (2909-4008)		2043 (1531-2330)		1324 (992-1510)		50 (22-99)		32 (14-64)		20 (9-40)		13 (6-26)		151 (67-299)		98 (43-194)	
2001	Swaziland									52 (24-103)		34 (15-67)		21 (10-41)		14 (6-27)		158 (72-311)		103 (47-202)	
2002	Swaziland									54 (25-106)		36 (16-69)		22 (10-43)		14 (7-28)		164 (76-320)		107 (50-209)	
2003	Swaziland									56 (27-109)		37 (18-71)		23 (11-44)		15 (7-29)		170 (81-328)		112 (53-216)	
2004	Swaziland									58 (28-110)		38 (18-73)		23 (11-44)		15 (7-29)		175 (85-333)		115 (56-219)	
2005	Swaziland									59 (29-110)		38 (19-72)		24 (12-44)		15 (8-29)		177 (88-334)		116 (58-219)	
2006	Swaziland									57 (29-107)		37 (19-70)		23 (12-43)		15 (8-28)		173 (88-324)		113 (58-211)	
2007	Swaziland									55 (28-103)		35 (18-66)		22 (11-41)		14 (7-27)		166 (85-311)		107 (55-200)	
2008	Swaziland									51 (25-98)		33 (16-63)		21 (10-39)		13 (6-25)		155 (75-297)		98 (47-189)	
2009	Swaziland									47 (21-92)		30 (13-58)		19 (9-37)		12 (5-23)		142 (65-279)		89 (41-175)	
2010	Swaziland									43 (19-87)		27 (12-54)		17 (8-35)		11 (5-22)		131 (57-262)		81 (35-163)	
2011	Swaziland									41 (18-82)		25 (11-50)		16 (7-33)		10 (4-20)		123 (55-248)		75 (33-150)	
2012	Swaziland									40 (16-82)		24 (9-49)		45 (18-93)		27 (11-56)		120 (47-247)		72 (28-148)	
2013	Swaziland									39 (15-80)		23 (9-47)		44 (18-91)		26 (10-54)		117 (47-241)		69 (28-143)	
2014	Swaziland									27 (11-56)		16 (7-33)		31 (13-63)		18 (7-37)		82 (33-168)		48 (20-99)	
2015	Swaziland	1448 (1249-1721)	835 (720-993)	849 (733-1009)	490 (423-582)	564 (422-643)	325 (244-371)	331 (248-377)	191 (143-218)	21 (8-42)	12 (5-24)	12 (5-25)	7 (3-14)	23 (10-48)	14 (6-28)	14 (6-28)	8 (3-16)	62 (25-128)	36 (15-74)	37 (15-75)	21 (9-43)
2000	Sweden	524 (452-623)		115 (99-137)		340 (254-387)		75 (56-85)		11 (5-20)		2 (1-4)		13 (6-23)		3 (1-5)		34 (15-62)		7 (3-14)	
2001	Sweden									10 (4-18)		2 (1-4)		11 (5-20)		3 (1-5)		30 (13-54)		7 (3-13)	
2002	Sweden									18 (8-32)		4 (2-8)		20 (9-37)		5 (2-9)		54 (24-97)		13 (6-23)	
2003	Sweden									15 (7-27)		3 (2-6)		17 (8-31)		4 (2-7)		45 (21-82)		10 (5-19)	
2004	Sweden									12 (5-22)		3 (1-5)		14 (6-25)		3 (1-5)		36 (16-66)		8 (4-14)	
2005	Sweden									15 (7-27)		3 (1-6)		17 (8-31)		4 (2-6)		45 (20-82)		9 (4-17)	
2006	Sweden									11 (5-20)		2 (1-4)		12 (6-22)		2 (1-4)		33 (15-60)		6 (3-12)	
2007	Sweden									22 (10-39)		4 (2-7)		25 (11-45)		5 (2-8)		65 (30-119)		12 (6-22)	
2008	Sweden									4 (2-7)		1 (0-1)		4 (2-8)		1 (0-1)		11 (5-21)		2 (1-4)	
2009	Sweden									2 (1-4)		0 (0-1)		2 (1-4)		0 (0-1)		6 (3-11)		1 (1-2)	
2010	Sweden									6 (3-11)		1 (0-2)		7 (3-12)		1 (1-2)		18 (8-32)		3 (1-6)	
2011	Sweden									0 (0-0)		0 (0-0)		0 (0-0)		0 (0-0)		0 (0-0)		0 (0-0)	
2012	Sweden									3 (1-6)		1 (0-1)		4 (2-7)		1 (0-1)		10 (4-18)		2 (1-3)	
2013	Sweden									1 (1-2)		0 (0-0)		2 (1-3)		0 (0-0)		4 (2-7)		1 (0-1)	
2014	Sweden									2 (1-3)		0 (0-0)		2 (1-3)		0 (0-1)		5 (2-8)		1 (0-1)	
2015	Sweden	229 (198-273)	1 (1-1)	40 (34-47)	0 (0-0)	149 (111-170)	0 (0-0)	26 (19-29)	0 (0-0)	2 (1-3)	0 (0-0)	0 (0-0)	0 (0-0)	2 (1-3)	0 (0-0)	0 (0-1)	0 (0-0)	5 (2-8)	0 (0-0)	1 (0-1)	0 (0-0)
2000	Switzerland	454 (391-539)		115 (99-137)		294 (220-335)		75 (56-85)		24 (11-44)		6 (3-11)		27 (12-50)		7 (3-13)		73 (33-132)		18 (8-33)	
2001	Switzerland									32 (14-58)		8 (4-15)		36 (16-66)		9 (4-17)		96 (44-175)		25 (11-46)	
2002	Switzerland									32 (15-59)		9 (4-16)		37 (17-67)		10 (4-18)		98 (44-177)		26 (12-47)	
2003	Switzerland									19 (8-34)		5 (2-9)		21 (10-38)		6 (3-10)		56 (26-102)		15 (7-28)	
2004	Switzerland									4 (2-7)		1 (0-2)		4 (2-8)		1 (1-2)		12 (5-21)		3 (1-6)	
2005	Switzerland									16 (7-28)		4 (2-8)		18 (8-32)		5 (2-9)		47 (21-86)		13 (6-24)	
2006	Switzerland									15 (7-26)		4 (2-7)		17 (8-30)		5 (2-8)		44 (20-80)		12 (6-22)	
2007	Switzerland									8 (4-15)		2 (1-4)		9 (4-17)		3 (1-5)		25 (11-45)		7 (3-12)	
2008	Switzerland									5 (2-10)		1 (1-3)		6 (3-11)		2 (1-3)		16 (7-29)		4 (2-8)	
2009	Switzerland									15 (7-27)		4 (2-7)		17 (8-31)		5 (2-8)		46 (21-83)		12 (6-22)	
2010	Switzerland									5 (2-9)		1 (1-2)		6 (3-11)		2 (1-3)		16 (7-28)		4 (2-7)	
2011	Switzerland									3 (1-5)		1 (0-1)		3 (2-6)		1 (0-2)		9 (4-16)		2 (1-4)	
2012	Switzerland									0 (0-0)		0 (0-0)		0 (0-0)		0 (0-0)		0 (0-0)		0 (0-0)	
2013	Switzerland									1 (1-2)		0 (0-1)		1 (1-2)		0 (0-1)		3 (2-6)		1 (0-2)	
2014	Switzerland									1 (0-1)		0 (0-0)		1 (0-2)		0 (0-0)		2 (1-4)		1 (0-1)	
2015	Switzerland	125 (108-148)	0 (0-0)	30 (26-36)	0 (0-0)	81 (61-92)	0 (0-0)	19 (15-22)	0 (0-0)	1 (0-1)	0 (0-0)	0 (0-0)	0 (0-0)	1 (0-1)	0 (0-0)	0 (0-0)	0 (0-0)	2 (1-4)	0 (0-0)	1 (0-1)	0 (0-0)
2000	Syrian Arab Republic	34490 (29759-40999)		1462 (1262-1738)		13412 (10049-15298)		569 (426-649)		52 (14-134)		2 (1-6)		59 (15-152)		3 (1-6)		157 (41-404)		7 (2-17)	
2001	Syrian Arab Republic									95 (26-242)		4 (1-10)		108 (30-275)		4 (1-11)		288 (79-731)		12 (3-30)	
2002	Syrian Arab Republic									96 (28-241)		4 (1-10)		109 (31-273)		5 (1-11)		290 (83-727)		12 (3-30)	
2003	Syrian Arab Republic									91 (28-226)		4 (1-9)		104 (31-257)		4 (1-11)		276 (84-683)		12 (3-29)	
2004	Syrian Arab Republic									90 (29-217)		4 (1-9)		102 (33-247)		4 (1-10)		271 (86-656)		11 (4-28)	
2005	Syrian Arab Republic									89 (30-212)		4 (1-9)		102 (34-241)		4 (1-10)		270 (91-641)		11 (4-27)	
2006	Syrian Arab Republic									96 (34-223)		4 (1-9)		109 (39-253)		5 (2-11)		291 (104-673)		12 (4-28)	
2007	Syrian Arab Republic									101 (39-228)		4 (2-9)		115 (44-259)		5 (2-11)		306 (117-688)		13 (5-28)	
2008	Syrian Arab Republic									106 (43-230)		4 (2-9)		120 (49-261)		5 (2-10)		319 (130-694)		13 (5-28)	
2009	Syrian Arab Republic									112 (49-234)		4 (2-9)		127 (55-266)		5 (2-11)		337 (147-706)		13 (6-28)	
2010	Syrian Arab Republic									111 (52-221)		4 (2-9)		126 (59-251)		5 (2-10)		334 (157-666)		13 (6-27)	
2011	Syrian Arab Republic									107 (54-202)		4 (2-8)		122 (62-229)		5 (2-9)		323 (164-609)		13 (7-24)	
2012	Syrian Arab Republic									89 (49-156)		4 (2-6)		101 (56-177)		4 (2-7)		268 (149-471)		11 (6-19)	
2013	Syrian Arab Republic									78 (48-128)		3 (2-6)		89 (55-145)		4 (2-6)		237 (146-386)		10 (6-17)	
2014	Syrian Arab Republic									74 (49-111)		3 (2-5)		84 (55-127)		4 (3-6)		223 (147-336)		10 (7-15)	

Spn = *Streptococcus pneumoniae*; npnm = non-pneumonia, non-meningitis; HIV = cases only in HIV-infected children; Uncertainty range provided in parentheses.

Year	Country	Spn pneumonia cases (UR)	Spn pneumonia cases, HIV (UR)	Spn pneumonia incidence rate (UR)	Spn pneumonia incidence rate, HIV (UR)	Spn severe pneumonia cases (UR)	Spn severe pneumonia cases, HIV (UR)	Spn severe pneumonia incidence rate (UR)	Spn severe pneumonia incidence rate, HIV (UR)	Spn meningitis cases (UR)	Spn meningitis cases, HIV (UR)	Spn meningitis incidence rate (UR)	Spn meningitis incidence rate, HIV (UR)	Spn NPNM cases (UR)	Spn NPNM cases, HIV (UR)	Spn NPNM incidence rate (UR)	Spn NPNM incidence rate, HIV (UR)	Spn NPNM non-severe cases (UR)	Spn NPNM non-severe cases, HIV (UR)	SPN NPNM non-severe incidence rate (UR)	SPN NPNM non-severe incidence rate, HIV (UR)
2015	Syrian Arab Republic	23133 (19959-27498)	6 (5-7)	1073 (926-1276)	0 (0-0)	9015 (6754-10283)	2 (2-3)	418 (313-477)	0 (0-0)	77 (50-114)	0 (0-0)	4 (2-5)	0 (0-0)	87 (57-130)	0 (0-0)	4 (3-6)	0 (0-0)	231 (150-345)	0 (0-0)	11 (7-16)	0 (0-0)
2000	Tajikistan	2008 (1732-2386)		230 (198-273)		1301 (975-1484)		149 (112-170)		411 (159-884)		47 (18-101)		165 (64-355)		19 (7-41)		1240 (479-2671)		142 (55-306)	
2001	Tajikistan									375 (148-801)		43 (17-92)		150 (60-321)		17 (7-37)		1131 (448-2418)		130 (52-278)	
2002	Tajikistan									338 (138-717)		39 (16-83)		136 (55-288)		16 (6-33)		1021 (416-2166)		119 (48-252)	
2003	Tajikistan									306 (110-664)		36 (13-78)		348 (125-754)		41 (15-89)		925 (331-2004)		109 (39-236)	
2004	Tajikistan									283 (105-609)		34 (12-72)		322 (119-691)		38 (14-82)		856 (317-1838)		102 (38-218)	
2005	Tajikistan									266 (102-566)		31 (12-67)		302 (116-643)		36 (14-76)		803 (308-1709)		95 (36-202)	
2006	Tajikistan									252 (96-536)		30 (11-63)		286 (109-608)		34 (13-72)		761 (291-1617)		90 (34-191)	
2007	Tajikistan									244 (93-518)		28 (11-60)		277 (105-588)		32 (12-68)		736 (280-1563)		85 (32-180)	
2008	Tajikistan									240 (91-510)		27 (10-57)		273 (104-579)		30 (12-65)		725 (276-1540)		81 (31-172)	
2009	Tajikistan									241 (92-513)		26 (10-55)		274 (104-582)		29 (11-63)		728 (276-1549)		78 (30-167)	
2010	Tajikistan									242 (92-514)		25 (9-53)		275 (104-584)		28 (11-61)		730 (277-1553)		76 (29-161)	
2011	Tajikistan									241 (91-514)		24 (9-51)		274 (104-583)		27 (10-58)		728 (276-1551)		72 (27-153)	
2012	Tajikistan									239 (90-510)		23 (9-48)		272 (103-580)		26 (10-55)		723 (273-1541)		68 (26-146)	
2013	Tajikistan									236 (89-504)		22 (8-46)		268 (101-572)		25 (9-52)		713 (269-1521)		65 (25-139)	
2014	Tajikistan									232 (87-495)		21 (8-44)		263 (99-562)		23 (9-50)		700 (264-1495)		62 (23-133)	
2015	Tajikistan	2659 (2294-3161)	50 (43-59)	230 (198-273)	4 (4-5)	1724 (1291-1966)	32 (24-37)	149 (112-170)	3 (2-3)	224 (84-479)	4 (2-9)	19 (7-41)	0 (0-1)	255 (96-544)	5 (2-10)	22 (8-47)	0 (0-1)	678 (255-1447)	13 (5-27)	59 (22-125)	1 (0-2)
2000	Thailand	72507 (62560-86190)		1584 (1367-1883)		28037 (21005-31979)		613 (459-699)		283 (114-617)		6 (3-13)		322 (130-700)		7 (3-15)		856 (346-1862)		19 (8-41)	
2001	Thailand									264 (108-573)		6 (2-13)		300 (123-651)		7 (3-14)		798 (326-1731)		18 (7-38)	
2002	Thailand									250 (103-540)		6 (2-12)		284 (117-613)		6 (3-14)		755 (311-1631)		17 (7-36)	
2003	Thailand									238 (99-512)		5 (2-11)		270 (112-582)		6 (3-13)		719 (299-1547)		16 (7-35)	
2004	Thailand									224 (94-480)		5 (2-11)		254 (107-545)		6 (2-12)		676 (284-1449)		15 (6-33)	
2005	Thailand									207 (88-442)		5 (2-10)		235 (99-502)		5 (2-11)		624 (265-1334)		14 (6-30)	
2006	Thailand									191 (81-408)		4 (2-10)		217 (92-464)		5 (2-11)		577 (245-1233)		14 (6-29)	
2007	Thailand									176 (74-377)		4 (2-9)		200 (84-428)		5 (2-10)		533 (225-1138)		13 (5-27)	
2008	Thailand									160 (67-342)		4 (2-8)		182 (76-389)		4 (2-10)		483 (203-1034)		12 (5-25)	
2009	Thailand									150 (63-321)		4 (2-8)		170 (71-365)		4 (2-9)		452 (190-969)		11 (5-24)	
2010	Thailand									141 (59-302)		4 (1-8)		160 (67-344)		4 (2-9)		426 (178-913)		11 (5-23)	
2011	Thailand									132 (55-283)		3 (1-7)		149 (62-321)		4 (2-8)		397 (165-853)		10 (4-22)	
2012	Thailand									123 (51-264)		3 (1-7)		140 (58-300)		4 (2-8)		371 (154-798)		10 (4-21)	
2013	Thailand									115 (48-248)		3 (1-6)		131 (55-281)		3 (1-7)		348 (145-747)		9 (4-20)	
2014	Thailand									107 (45-230)		3 (1-6)		122 (51-261)		3 (1-7)		324 (136-694)		9 (4-18)	
2015	Thailand	45873 (39580-54530)	0 (0-0)	1228 (1060-1460)	0 (0-0)	17853 (13375-20363)	0 (0-0)	478 (358-545)	0 (0-0)	103 (44-221)	0 (0-0)	3 (1-6)	0 (0-0)	117 (50-251)	0 (0-0)	3 (1-7)	0 (0-0)	312 (132-668)	0 (0-0)	8 (4-18)	0 (0-0)
2000	The former Yugoslav Republic of Macedonia	312 (269-371)		230 (198-273)		202 (152-231)		149 (112-170)		4 (2-10)		3 (1-8)		4 (2-12)		3 (1-9)		12 (5-31)		9 (4-23)	
2001	The former Yugoslav Republic of Macedonia									8 (3-21)		6 (3-16)		9 (4-24)		7 (3-18)		24 (10-63)		18 (8-48)	
2002	The former Yugoslav Republic of Macedonia									8 (3-20)		6 (3-15)		9 (4-23)		7 (3-18)		23 (10-60)		18 (8-47)	
2003	The former Yugoslav Republic of Macedonia									7 (3-18)		6 (2-14)		8 (3-21)		6 (3-16)		21 (9-55)		17 (7-44)	
2004	The former Yugoslav Republic of Macedonia									7 (3-19)		6 (2-15)		8 (4-21)		7 (3-17)		22 (9-57)		18 (8-45)	
2005	The former Yugoslav Republic of Macedonia									3 (1-7)		2 (1-5)		3 (1-8)		2 (1-6)		8 (3-20)		6 (3-16)	
2006	The former Yugoslav Republic of Macedonia									4 (2-11)		3 (1-9)		5 (2-12)		4 (2-10)		13 (5-33)		10 (4-27)	
2007	The former Yugoslav Republic of Macedonia									13 (6-34)		11 (5-29)		15 (6-38)		13 (5-32)		40 (17-102)		34 (15-86)	
2008	The former Yugoslav Republic of Macedonia									6 (2-14)		5 (2-12)		6 (3-16)		5 (2-14)		17 (7-43)		14 (6-37)	
2009	The former Yugoslav Republic of Macedonia									7 (3-18)		6 (3-16)		8 (3-20)		7 (3-18)		21 (9-53)		19 (8-48)	
2010	The former Yugoslav Republic of Macedonia									14 (6-34)		12 (5-31)		16 (7-39)		14 (6-35)		41 (18-103)		37 (17-93)	
2011	The former Yugoslav Republic of Macedonia									8 (4-19)		7 (3-18)		9 (4-22)		8 (4-20)		24 (11-59)		22 (10-54)	
2012	The former Yugoslav Republic of Macedonia									7 (3-17)		7 (3-16)		8 (4-20)		8 (3-18)		22 (10-53)		20 (9-48)	
2013	The former Yugoslav Republic of Macedonia									7 (3-17)		6 (3-15)		8 (4-19)		7 (3-17)		21 (10-51)		19 (9-46)	
2014	The former Yugoslav Republic of Macedonia									7 (3-16)		6 (3-14)		8 (4-18)		7 (3-16)		21 (9-49)		18 (8-44)	
2015	The former Yugoslav Republic of Macedonia	261 (225-310)	0 (0-0)	230 (198-273)	0 (0-0)	169 (127-193)	0 (0-0)	149 (112-170)	0 (0-0)	6 (3-15)	0 (0-0)	6 (3-13)	0 (0-0)	7 (3-17)	0 (0-0)	6 (3-15)	0 (0-0)	19 (9-45)	0 (0-0)	17 (8-39)	0 (0-0)
2000	Timor-Leste	4332 (3737-5149)		2530 (2183-3008)		1675 (1255-1910)		978 (733-1116)		75 (30-160)		44 (18-93)		30 (12-64)		18 (7-37)		228 (91-483)		133 (53-282)	
2001	Timor-Leste									74 (30-156)		43 (17-91)		30 (12-63)		17 (7-36)		223 (90-472)		130 (53-275)	
2002	Timor-Leste									71 (29-150)		42 (17-87)		29 (12-60)		17 (7-35)		216 (88-453)		126 (51-264)	
2003	Timor-Leste									67 (28-140)		39 (16-82)		27 (11-56)		16 (7-33)		203 (84-422)		119 (49-248)	
2004	Timor-Leste									61 (26-127)		37 (15-76)		25 (10-51)		15 (6-30)		185 (78-383)		111 (47-228)	
2005	Timor-Leste									54 (23-111)		33 (14-68)		22 (9-44)		13 (6-27)		164 (71-335)		100 (43-204)	
2006	Timor-Leste									47 (21-96)		30 (13-61)		19 (8-39)		12 (5-25)		143 (63-291)		91 (40-185)	
2007	Timor-Leste									42 (16-86)		28 (11-57)		47 (19-98)		31 (12-65)		126 (50-261)		83 (33-173)	
2008	Timor-Leste									37 (15-77)		25 (10-52)		42 (17-87)		29 (12-59)		113 (46-232)		77 (31-158)	
2009	Timor-Leste									35 (15-71)		24 (10-49)		40 (17-81)		27 (11-55)		106 (44-215)		72 (30-147)	
2010	Timor-Leste									35 (15-71)		23 (10-48)		40 (17-81)		27 (11-54)		106 (44-215)		71 (29-144)	
2011	Timor-Leste									36 (15-73)		24 (10-48)		41 (17-83)		27 (11-55)		109 (46-220)		72 (30-146)	
2012	Timor-Leste									40 (17-80)		25 (10-49)		45 (19-91)		28 (12-56)		119 (50-241)		74 (31-149)	
2013	Timor-Leste									43 (18-87)		25 (10-49)		49 (21-99)		28 (12-56)		131 (56-263)		74 (32-149)	
2014	Timor-Leste									46 (20-92)		24 (10-48)		52 (22-105)		27 (12-55)		139 (59-278)		73 (31-146)	
2015	Timor-Leste	3942 (3401-4685)	34 (30-41)	1967 (1698-2339)	17 (15-20)	1524 (1142-1739)	13 (10-15)	761 (570-868)	7 (5-8)	47 (20-94)	0 (0-1)	23 (10-47)	0 (0-0)	53 (23-106)	0 (0-1)	27 (11-53)	0 (0-0)	141 (61-283)	1 (1-2)	71 (30-141)	1 (0-1)
2000	Togo	31131 (26861-37006)		3877 (3345-4608)		12149 (9102-13858)		1513 (1133-1726)		203 (72-459)		25 (9-57)		82 (29-184)		10 (4-23)		614 (218-1386)		77 (27-173)	
2001	Togo									207 (73-467)		25 (9-57)		83 (29-188)		10 (4-23)		624 (219-1412)		76 (27-171)	
2002	Togo									208 (72-472)		25 (9-56)		83 (29-189)		10 (3-22)		628 (219-1425)		74 (26-168)	
2003	Togo									207 (72-472)		24 (8-54)		83 (29-189)		10 (3-22)		626 (216-1424)		72 (25-163)	
2004	Togo									206 (70-470)		23 (8-52)		83 (28-188)		9 (3-21)		621 (213-1418)		69 (24-158)	
2005	Togo									203 (69-465)		22 (7-51)		82 (28-187)		9 (3-20)		614 (208-1405)		67 (23-153)	
2006	Togo									202 (68-463)		21 (7-49)		81 (27-186)		9 (3-20)		610 (205-1398)		64 (22-147)	
2007	Togo									204 (70-464)		21 (7-48)		82 (28-186)		8 (3-19)		615 (210-1403)		63 (22-143)	
2008	Togo									211 (73-479)		21 (7-48)		85 (29-192)		8 (3-19)		638 (222-1446)		64 (22-144)	
2009	Togo									225 (79-506)		22 (8-49)		90 (32-203)		9 (3-20)		679 (240-1529)		66 (23-149)	
2010	Togo									246 (88-549)		24 (8-52)		99 (35-220)		9 (3-21)		743 (266-1657)		71 (25-158)	
2011	Togo									276 (103-605)		26 (10-57)		111 (41-243)		10 (4-23)		832 (311-1828)		78 (29-171)	
2012	Togo									305 (119-659)		28 (11-61)		122 (48-264)		11 (4-24)		921 (359-1991)		85 (33-183)	
2013	Togo									325 (133-690)		29 (12-62)		130 (53-277)		12 (5-25)		980 (401-2083)		89 (36-188)	
2014	Togo									291 (119-617)		26 (11-55)		117 (48-247)		10 (4-22)		880 (359-1862)		78 (32-166)	
2015	Togo	14667 (12655-17435)	812 (700-965)	1286 (1109-1528)	71 (61-85)	5742 (4302-6549)	318 (238-362)	503 (377-574)	28 (21-32)	207 (85-437)	11 (5-24)	18 (7-38)	1 (0-2)	83 (34-175)	5 (2-10)	7 (3-15)	0 (0-1)	625 (256-1319)	35 (14-73)	55 (22-116)	3 (1-6)
2000	Tonga	260 (224-309)		1988 (1715-2363)		101 (76-116)		777 (582-886)		2 (1-3)		13 (6-22)		2 (1-3)		14 (6-25)		5 (2-9)		38 (17-68)	
2001	Tonga									2 (1-3)		13 (6-22)		2 (1-3)		14 (6-25)		5 (2-9)		38 (17-67)	
2002	Tonga									2 (1-3)		12 (5-22)		2 (1-3)		14 (6-25)		5 (2-8)		37 (17-66)	
2003	Tonga									2 (1-3)		12 (5-21)		2 (1-3)		13 (6-24)		5 (2-8)		36 (16-63)	
2004	Tonga									2 (1-3)		12 (5-21)		2 (1-3)		13 (6-24)		5 (2-8)		36 (16-64)	
2005	Tonga									3 (1-6)		24 (11-43)		4 (2-7)		27 (12-48)		10 (4-17)		73 (33-129)	
2006	Tonga									4 (2-6)		26 (12-46)		4 (2-7)		29 (13-52)		11 (5-19)		78 (35-138)	
2007	Tonga									3 (2-6)		25 (11-45)		4 (2-7)		29 (13-51)		10 (5-19)		76 (34-135)	
2008	Tonga									3 (2-6)		25 (11-44)		4 (2-7)		28 (13-50)		10 (5-18)		75 (34-133)	
2009	Tonga									3 (2-6)		26 (11-45)		4 (2-7)		29 (13-51)		11 (5-19)		77 (35-137)	
2010	Tonga									4 (2-7)		27 (12-49)		4 (2-7)		31 (14-55)		11 (5-20)		83 (37-147)	
2011	Tonga									4 (2-6)		27 (12-48)		4 (2-7)		31 (14-54)		11 (5-19)		82 (37-145)	
2012	Tonga									3 (1-6)		25 (11-44)		4 (2-7)		28 (13-50)		10 (4-18)		75 (34-134)	
2013	Tonga									3 (1-6)		25 (11-44)		4 (2-7)		28 (13-50)		10 (4-17)		75 (34-133)	
2014	Tonga									3 (1-5)		24 (11-42)		3 (2-6)		27 (12-48)		9 (4-16)		72 (32-127)	
2015	Tonga	139 (120-165)	0 (0-0)	1095 (945-1302)	0 (0-0)	54 (40-62)	0 (0-0)	425 (319-485)	0 (0-0)	3 (1-5)	0 (0-0)	24 (11-42)	0 (0-0)	3 (2-6)	0 (0-0)	27 (12-47)	0 (0-0)	9 (4-16)	0 (0-0)	71 (32-126)	0 (0-0)
2000	Trinidad and Tobago	1288 (1111-1531)		1462 (1261-1738)		501 (375-571)		568 (426-648)		8 (2-23)		9 (2-26)		9 (2-26)		10 (3-30)		24 (6-69)		27 (7-79)	
2001	Trinidad and Tobago									3 (1-9)		4 (1-10)		4 (1-10)		4 (1-12)		10 (3-27)		11 (3-31)	
2002	Trinidad and Tobago									5 (1-14)		6 (1-16)		6 (1-16)		6 (2-18)		15 (4-42)		17 (4-48)	
2003	Trinidad and Tobago									12 (3-33)		13 (4-38)		13 (4-38)		15 (4-43)		35 (9-101)		40 (11-114)	
2004	Trinidad and Tobago									3 (1-9)		4 (1-11)		4 (1-11)		4 (1-12)		10 (3-28)		11 (3-32)	
2005	Trinidad and Tobago									5 (1-15)		6 (2-17)		6 (2-17)		7 (2-19)		16 (4-45)		17 (5-50)	
2006	Trinidad and Tobago									6 (2-17)		7 (2-19)		7 (2-20)		7 (2-21)		18 (5-52)		20 (5-57)	
2007	Trinidad and Tobago									4 (1-10)		4 (1-11)		4 (1-11)		4 (1-12)		11 (3-30)		11 (3-32)	
2008	Trinidad and Tobago									6 (1-16)		6 (2-17)		6 (2-18)		7 (2-19)		17 (4-48)		18 (5-51)	
2009	Trinidad and Tobago									4 (1-13)		5 (1-14)		5 (1-15)		5 (1-15)		14 (4-39)		14 (4-41)	
2010	Trinidad and Tobago									4 (1-12)		4 (1-13)		5 (1-14)		5 (1-15)		13 (3-37)		14 (4-39)	
2011	Trinidad and Tobago									4 (1-12)		4 (1-12)		5 (1-13)		5 (1-14)		12 (3-36)		13 (3-37)	
2012	Trinidad and Tobago									3 (1-10)		4 (1-10)		4 (1-11)		4 (1-12)		10 (3-30)		11 (3-31)	
2013	Trinidad and Tobago									3 (1-7)		3 (1-8)		3 (1-8)		3 (1-9)		8 (2-23)		8 (2-24)	
2014	Trinidad and Tobago									2 (1-6)		2 (1-6)		2 (1-7)		2 (1-7)		6 (2-18)		7 (2-19)	
2015	Trinidad and Tobago	429 (370-510)	2 (2-3)	454 (391-539)	2 (2-3)	167 (125-191)	1 (1-1)	177 (132-202)	1 (1-1)	2 (1-5)	0 (0-0)	2 (1-6)	0 (0-0)	2 (1-6)	0 (0-0)	2 (1-6)	0 (0-0)	6 (2-16)	0 (0-0)	6 (2-17)	0 (0-0)
2000	Tunisia	16916 (14596-20109)		2047 (1766-2434)		6653 (4985-7589)		805 (603-918)		20 (6-44)		2 (1-5)		22 (7-50)		3 (1-6)		59 (19-133)		7 (2-16)	
2001	Tunisia									18 (4-48)		2 (1-6)		21 (5-54)		3 (1-7)		55 (12-144)		7 (2-18)	
2002	Tunisia									17 (4-45)		2 (1-6)		19 (5-51)		2 (1-6)		52 (12-135)		6 (2-17)	
2003	Tunisia									31 (7-79)		4 (1-10)		35 (8-90)		4 (1-11)		92 (23-239)		12 (3-30)	
2004	Tunisia									31 (8-79)		4 (1-10)		35 (9-90)		4 (1-11)		93 (24-238)		12 (3-30)	
2005	Tunisia									29 (8-73)		4 (1-9)		33 (9-83)		4 (1-11)		87 (23-221)		11 (3-28)	
2006	Tunisia									14 (4-35)		2 (0-4)		16 (4-40)		2 (1-5)		42 (12-107)		5 (1-13)	
2007	Tunisia									13 (4-33)		2 (0-4)		15 (4-38)		2 (1-5)		40 (11-101)		5 (1-13)	
2008	Tunisia									13 (3-31)		2 (0-4)		14 (4-36)		2 (0-4)		38 (10-95)		5 (1-12)	
2009	Tunisia									12 (3-30)		1 (0-4)		13 (4-34)		2 (0-4)		36 (10-89)		4 (1-11)	
2010	Tunisia									11 (3-28)		1 (0-3)		13 (3-32)		1 (0-4)		33 (9-84)		4 (1-10)	
2011	Tunisia									15 (4-38)		2 (0-4)		17 (5-43)		2 (1-5)		45 (13-114)		5 (1-13)	
2012	Tunisia									20 (5-49)		2 (1-5)		22 (6-56)		2 (1-6)		59 (16-148)		7 (2-16)	
2013	Tunisia									19 (5-47)		2 (1-5)		22 (6-54)		2 (1-6)		57 (16-143)		6 (2-15)	
2014	Tunisia									18 (5-44)		2 (1-5)		20 (6-51)		2 (1-5)		54 (15-134)		6 (2-14)	
2015	Tunisia	10365 (8943-12321)	6 (5-7)	1073 (926-1276)	1 (0-1)	4035 (3023-4602)	2 (2-2)	418 (313-477)	0 (0-0)	17 (5-41)	0 (0-0)	2 (0-4)	0 (0-0)	19 (5-47)	0 (0-0)	2 (1-5)	0 (0-0)	50 (14-124)	0 (0-0)	5 (1-13)	0 (0-0)
2000	Turkey	15010 (12951-17843)		230 (198-273)		9731 (7290-11099)		149 (112-170)		626 (296-962)		10 (5-15)		711 (337-1092)		11 (5-17)		1889 (895-2904)		29 (14-44)	
2001	Turkey									596 (280-908)		9 (4-14)		677 (318-1031)		10 (5-16)		1801 (845-2742)		27 (13-42)	
2002	Turkey									580 (268-868)		9 (4-13)		659 (304-986)		10 (5-15)		1751 (808-2621)		27 (12-40)	
2003	Turkey									592 (265-861)		9 (4-13)		672 (301-978)		10 (5-15)		1788 (801-2599)		27 (12-40)	
2004	Turkey									655 (451-1035)		10 (7-16)		744 (512-1176)		12 (8-18)		1978 (1361-3126)		31 (21-48)	
2005	Turkey									720 (468-1076)		11 (7-17)		818 (532-1222)		13 (8-19)		2174 (1413-3248)		34 (22-51)	
2006	Turkey									752 (465-1069)		12 (7-17)		855 (529-1214)		13 (8-19)		2272 (1405-3229)		36 (22-51)	
2007	Turkey									950 (570-1310)		15 (9-21)		1079 (647-1488)		17 (10-24)		2868 (1721-3955)		46 (27-63)	
2008	Turkey									918 (551-1266)		15 (9-20)		1043 (626-1438)		17 (10-23)		2772 (1664-3823)		44 (27-61)	
2009	Turkey									743 (446-1025)		12 (7-16)		844 (506-1164)		13 (8-19)		2243 (1346-3094)		36 (21-49)	
2010	Turkey									630 (378-869)		10 (6-14)		716 (430-987)		11 (7-16)		1903 (1142-2625)		30 (18-42)	
2011	Turkey									410 (246-565)		6 (4-9)		465 (279-642)		7 (4-10)		1237 (742-1706)		19 (12-27)	
2012	Turkey									448 (269-617)		7 (4-10)		508 (305-701)		8 (5-11)		1351 (811-1864)		21 (13-29)	
2013	Turkey									360 (216-496)		5 (3-8)		408 (245-563)		6 (4-9)		1086 (652-1498)		16 (10-23)	
2014	Turkey									361 (216-497)		5 (3-7)		410 (246-565)		6 (4-8)		1089 (654-1502)		16 (10-22)	
2015	Turkey	6881 (5937-8179)	5 (4-6)	103 (89-122)	0 (0-0)	4461 (3342-5088)	3 (2-3)	67 (50-76)	0 (0-0)	339 (203-467)	0 (0-0)	5 (3-7)	0 (0-0)	385 (231-531)	0 (0-0)	6 (3-8)	0 (0-0)	1024 (614-1412)	1 (0-1)	15 (9-21)	0 (0-0)
2000	Turkmenistan	1108 (956-1317)		230 (198-273)		718 (538-819)		149 (112-170)		107 (42-232)		22 (9-48)		43 (17-93)		9 (3-19)		323 (125-699)		67 (26-145)	
2001	Turkmenistan									104 (40-224)		22 (9-48)		42 (16-90)		9 (4-19)		313 (122-676)		68 (26-146)	
2002	Turkmenistan									105 (41-227)		23 (9-49)		42 (17-91)		9 (4-20)		316 (124-684)		69 (27-148)	
2003	Turkmenistan									109 (36-243)		23 (8-51)		124 (41-276)		26 (9-58)		329 (110-733)		69 (23-155)	
2004	Turkmenistan									111 (38-248)		23 (8-51)		126 (43-281)		26 (9-58)		335 (113-748)		69 (23-153)	
2005	Turkmenistan									109 (37-243)		22 (7-49)		124 (42-276)		25 (8-56)		330 (112-734)		66 (23-148)	
2006	Turkmenistan									104 (36-232)		20 (7-45)		119 (41-264)		23 (8-51)		315 (108-701)		61 (21-136)	
2007	Turkmenistan									97 (33-215)		19 (6-42)		110 (38-245)		21 (7-47)		293 (101-650)		57 (19-126)	
2008	Turkmenistan									88 (31-195)		17 (6-38)		100 (35-221)		20 (7-43)		266 (92-588)		52 (18-115)	
2009	Turkmenistan									80 (28-176)		16 (6-35)		91 (32-200)		18 (6-40)		241 (84-533)		48 (17-107)	
2010	Turkmenistan									75 (26-164)		15 (5-33)		85 (30-187)		17 (6-38)		225 (79-496)		45 (16-100)	
2011	Turkmenistan									70 (25-155)		14 (5-31)		80 (28-176)		16 (6-36)		213 (75-467)		43 (15-95)	
2012	Turkmenistan									68 (24-150)		14 (5-30)		78 (28-170)		16 (6-34)		206 (73-452)		42 (15-91)	
2013	Turkmenistan									67 (24-147)		13 (5-29)		76 (27-167)		15 (5-33)		203 (72-444)		40 (14-88)	
2014	Turkmenistan									66 (24-145)		13 (5-28)		75 (27-164)		15 (5-32)		200 (72-437)		39 (14-85)	
2015	Turkmenistan	1194 (1030-1419)	0 (0-0)	230 (198-273)	0 (0-0)	774 (580-883)	0 (0-0)	149 (112-170)	0 (0-0)	65 (23-141)	0 (0-0)	12 (4-27)	0 (0-0)	73 (26-160)	0 (0-0)	14 (5-31)	0 (0-0)	195 (70-425)	0 (0-0)	38 (14-82)	0 (0-0)
2000	Tuvalu	23 (19-27)		1614 (1393-1919)		9 (7-10)		627 (469-715)		0 (0-0)		10 (4-20)		0 (0-0)		11 (4-23)		0 (0-1)		30 (11-61)	
2001	Tuvalu									0 (0-0)		8 (3-17)		0 (0-0)		9 (3-19)		0 (0-1)		25 (9-50)	
2002	Tuvalu									0 (0-0)		8 (3-17)		0 (0-0)		10 (3-20)		0 (0-1)		26 (9-52)	
2003	Tuvalu									0 (0-0)		9 (3-18)		0 (0-0)		10 (4-20)		0 (0-1)		27 (9-54)	
2004	Tuvalu									0 (0-0)		7 (2-14)		0 (0-0)		8 (3-16)		0 (0-0)		21 (7-43)	
2005	Tuvalu									0 (0-0)		7 (3-15)		0 (0-0)		8 (3-17)		0 (0-0)		22 (8-44)	
2006	Tuvalu									0 (0-0)		7 (3-15)		0 (0-0)		8 (3-17)		0 (0-0)		22 (8-45)	
2007	Tuvalu									0 (0-0)		5 (2-10)		0 (0-0)		6 (2-11)		0 (0-0)		15 (5-30)	

Spn = *Streptococcus pneumoniae*; npnm = non-pneumonia, non-meningitis; HIV = cases only in HIV-infected children; Uncertainty range provided in parentheses.

Year	Country	Spn pneumonia cases (UR)	Spn pneumonia cases, HIV (UR)	Spn pneumonia incidence rate (UR)	Spn pneumonia incidence rate, HIV (UR)	Spn severe pneumonia cases (UR)	Spn severe pneumonia cases, HIV (UR)	Spn severe pneumonia incidence rate (UR)	Spn severe pneumonia incidence rate, HIV (UR)	Spn meningitis cases (UR)	Spn meningitis cases, HIV (UR)	Spn meningitis incidence rate (UR)	Spn meningitis incidence rate, HIV (UR)	Spn NPNM cases (UR)	Spn NPNM cases, HIV (UR)	Spn NPNM incidence rate (UR)	Spn NPNM incidence rate, HIV (UR)	Spn NPNM non-severe cases (UR)	Spn NPNM non-severe cases, HIV (UR)	SPN NPNM non-severe incidence rate (UR)	SPN NPNM non-severe incidence rate, HIV (UR)
2008	Tuvalu									0 (0-0)		5 (2-10)		0 (0-0)		6 (2-11)		0 (0-0)		15 (5-30)	
2009	Tuvalu									0 (0-0)		19 (6-37)		0 (0-0)		21 (7-42)		1 (0-1)		56 (19-112)	
2010	Tuvalu									0 (0-0)		15 (5-28)		0 (0-0)		17 (6-32)		0 (0-1)		44 (15-86)	
2011	Tuvalu									0 (0-0)		16 (5-31)		0 (0-0)		19 (6-35)		0 (0-1)		50 (16-92)	
2012	Tuvalu									0 (0-0)		20 (11-43)		0 (0-0)		22 (12-49)		1 (0-1)		59 (33-131)	
2013	Tuvalu									0 (0-0)		22 (11-44)		0 (0-1)		25 (13-50)		1 (0-1)		66 (34-134)	
2014	Tuvalu									0 (0-0)		25 (12-48)		0 (0-1)		29 (14-54)		1 (0-1)		77 (36-144)	
2015	Tuvalu	10 (8-11)	0 (0-0)	936 (807-1112)	0 (0-0)	4 (3-4)	0 (0-0)	361 (270-411)	0 (0-0)	0 (0-0)	0 (0-0)	26 (12-47)	0 (0-0)	0 (0-1)	0 (0-0)	30 (13-53)	0 (0-0)	1 (0-1)	0 (0-0)	80 (36-141)	0 (0-0)
2000	Uganda	104455 (90126-124168)		2248 (1940-2672)		41013 (30727-46780)		883 (661-1007)		2948 (1400-5671)		63 (30-122)		1183 (561-2275)		25 (12-49)		8902 (4226-17125)		192 (91-369)	
2001	Uganda									2899 (1374-5568)		60 (29-116)		1163 (551-2233)		24 (11-47)		8754 (4150-16814)		182 (86-350)	
2002	Uganda									2872 (1379-5486)		58 (28-111)		1152 (553-2201)		23 (11-44)		8672 (4163-16566)		175 (84-334)	
2003	Uganda									2807 (1364-5328)		55 (27-104)		1126 (547-2137)		22 (11-42)		8476 (4119-16089)		165 (80-314)	
2004	Uganda									2905 (1428-5474)		55 (27-103)		1165 (573-2195)		22 (11-41)		8771 (4311-16528)		165 (81-311)	
2005	Uganda									2924 (1453-5467)		53 (26-100)		1173 (583-2193)		21 (11-40)		8829 (4388-16508)		161 (80-301)	
2006	Uganda									2808 (1411-5208)		49 (25-92)		1126 (566-2089)		20 (10-37)		8478 (4261-15725)		149 (75-277)	
2007	Uganda									2724 (1384-5012)		47 (24-86)		1093 (555-2010)		19 (9-34)		8225 (4179-15136)		141 (71-259)	
2008	Uganda									2705 (1391-4942)		45 (23-82)		1085 (558-1982)		18 (9-33)		8168 (4199-14923)		136 (70-248)	
2009	Uganda									2657 (1384-4825)		43 (22-78)		1066 (555-1935)		17 (9-31)		8023 (4179-14569)		130 (68-236)	
2010	Uganda									2696 (1425-4870)		43 (22-77)		1081 (571-1953)		17 (9-31)		8140 (4302-14705)		128 (68-232)	
2011	Uganda									2779 (1356-5128)		43 (21-79)		3157 (1540-5825)		49 (24-90)		8392 (4095-15486)		129 (63-238)	
2012	Uganda									2857 (1419-5250)		43 (21-79)		3245 (1611-5963)		49 (24-89)		8628 (4284-15854)		129 (64-238)	
2013	Uganda									2867 (1440-5251)		42 (21-77)		3256 (1636-5964)		48 (24-87)		8657 (4349-15855)		127 (64-232)	
2014	Uganda									2288 (1162-4177)		33 (17-60)		2598 (1319-4744)		37 (19-68)		6908 (3507-12611)		99 (50-181)	
2015	Uganda	92487 (79799-109940)	19317 (16667-22963)	1292 (1115-1536)	270 (233-321)	36220 (27136-41312)	7565 (5668-8629)	506 (379-577)	106 (79-121)	1939 (991-3528)	405 (207-737)	27 (14-49)	6 (3-10)	2202 (1125-4007)	460 (235-837)	31 (16-56)	6 (3-12)	5854 (2991-10652)	1223 (625-2225)	82 (42-149)	17 (9-31)
2000	Ukraine	9773 (8433-11618)		460 (397-547)		6336 (4747-7227)		298 (223-340)		107 (42-308)		5 (2-15)		122 (48-350)		6 (2-16)		325 (128-930)		15 (6-44)	
2001	Ukraine									95 (38-271)		5 (2-13)		108 (43-308)		5 (2-15)		287 (114-818)		14 (6-40)	
2002	Ukraine									84 (34-238)		4 (2-12)		95 (38-270)		5 (2-14)		253 (102-718)		13 (5-36)	
2003	Ukraine									76 (31-215)		4 (2-11)		87 (35-244)		4 (2-13)		231 (93-650)		12 (5-34)	
2004	Ukraine									70 (29-197)		4 (1-10)		80 (33-224)		4 (2-12)		213 (86-595)		11 (4-31)	
2005	Ukraine									68 (28-190)		4 (1-10)		78 (32-216)		4 (2-11)		206 (84-573)		11 (4-29)	
2006	Ukraine									79 (32-219)		4 (2-11)		90 (37-249)		5 (2-13)		239 (98-661)		12 (5-34)	
2007	Ukraine									113 (47-310)		6 (2-15)		128 (53-353)		6 (3-17)		341 (141-937)		17 (7-46)	
2008	Ukraine									125 (52-341)		6 (2-16)		142 (59-388)		7 (3-18)		377 (156-1031)		17 (7-48)	
2009	Ukraine									116 (48-314)		5 (2-14)		131 (55-357)		6 (2-16)		350 (145-949)		16 (6-42)	
2010	Ukraine									109 (46-296)		5 (2-13)		124 (52-336)		5 (2-14)		330 (138-892)		14 (6-38)	
2011	Ukraine									87 (36-233)		3 (1-9)		98 (41-264)		4 (2-11)		262 (110-702)		11 (4-28)	
2012	Ukraine									101 (43-269)		4 (2-11)		115 (48-306)		5 (2-12)		305 (129-813)		12 (5-32)	
2013	Ukraine									88 (37-233)		4 (2-9)		100 (43-265)		4 (2-11)		266 (113-705)		11 (5-28)	
2014	Ukraine									73 (31-193)		3 (1-8)		83 (36-220)		3 (1-9)		222 (95-584)		9 (4-24)	
2015	Ukraine	11132 (9605-13233)	331 (285-393)	460 (397-547)	14 (12-16)	7217 (5407-8231)	214 (161-245)	298 (223-340)	9 (7-10)	63 (27-166)	2 (1-5)	3 (1-7)	0 (0-0)	72 (31-188)	2 (1-6)	3 (1-8)	0 (0-0)	191 (82-500)	6 (2-15)	8 (3-21)	0 (0-1)
2000	United Arab Emirates	5863 (5058-6969)		2291 (1977-2724)		2303 (1726-2627)		900 (674-1027)		23 (14-31)		9 (5-12)		26 (16-36)		10 (6-14)		69 (41-95)		27 (16-37)	
2001	United Arab Emirates									22 (13-30)		9 (5-12)		24 (15-34)		10 (6-14)		65 (39-90)		26 (16-36)	
2002	United Arab Emirates									20 (12-27)		8 (5-11)		23 (14-31)		9 (6-13)		60 (36-83)		25 (15-34)	
2003	United Arab Emirates									19 (11-26)		8 (5-11)		22 (13-30)		9 (5-12)		57 (34-79)		24 (14-33)	
2004	United Arab Emirates									20 (12-27)		8 (5-11)		22 (13-31)		9 (5-12)		60 (36-82)		23 (14-32)	
2005	United Arab Emirates									21 (12-28)		7 (4-10)		23 (14-32)		8 (5-11)		62 (37-86)		22 (13-30)	
2006	United Arab Emirates									21 (13-29)		7 (4-10)		24 (14-33)		8 (5-11)		63 (38-87)		21 (13-29)	
2007	United Arab Emirates									23 (14-31)		7 (4-10)		26 (16-36)		8 (5-11)		69 (41-95)		21 (13-29)	
2008	United Arab Emirates									24 (14-33)		6 (4-9)		27 (16-37)		7 (4-10)		71 (43-98)		20 (12-27)	
2009	United Arab Emirates									24 (14-33)		6 (4-8)		27 (16-37)		7 (4-9)		72 (43-99)		18 (11-25)	
2010	United Arab Emirates									18 (11-25)		4 (3-6)		21 (12-28)		5 (3-7)		55 (33-76)		13 (8-18)	
2011	United Arab Emirates									15 (9-20)		3 (2-4)		17 (10-23)		4 (2-5)		44 (26-61)		10 (6-14)	
2012	United Arab Emirates									13 (8-18)		3 (2-4)		15 (9-20)		3 (2-4)		39 (24-54)		8 (5-12)	
2013	United Arab Emirates									12 (7-16)		2 (1-3)		13 (8-18)		3 (2-4)		36 (21-49)		7 (4-10)	
2014	United Arab Emirates									8 (5-12)		2 (1-2)		10 (6-13)		2 (1-3)		26 (15-35)		5 (3-7)	
2015	United Arab Emirates	1729 (1492-2056)	0 (0-0)	358 (309-426)	0 (0-0)	673 (504-768)	0 (0-0)	139 (104-159)	0 (0-0)	7 (4-10)	0 (0-0)	1 (1-2)	0 (0-0)	8 (5-11)	0 (0-0)	2 (1-2)	0 (0-0)	22 (13-30)	0 (0-0)	5 (3-6)	0 (0-0)
2000	United Kingdom	4016 (3465-4774)		115 (99-137)		2604 (1951-2970)		75 (56-85)		378 (172-686)		11 (5-20)		429 (195-779)		12 (6-22)		1142 (518-2072)		33 (15-59)	
2001	United Kingdom									419 (190-760)		12 (6-22)		476 (216-863)		14 (6-25)		1265 (574-2295)		37 (17-67)	
2002	United Kingdom									334 (151-606)		10 (4-18)		379 (172-688)		11 (5-20)		1008 (457-1829)		30 (14-54)	
2003	United Kingdom									378 (172-687)		11 (5-20)		430 (195-780)		13 (6-23)		1142 (518-2073)		34 (15-62)	
2004	United Kingdom									325 (147-590)		10 (4-18)		369 (167-670)		11 (5-20)		981 (445-1781)		29 (13-53)	
2005	United Kingdom									324 (147-588)		10 (4-17)		368 (167-668)		11 (5-20)		979 (444-1776)		29 (13-52)	
2006	United Kingdom									231 (105-420)		7 (3-12)		263 (119-477)		8 (3-14)		699 (317-1268)		20 (9-37)	
2007	United Kingdom									194 (88-352)		5 (2-10)		221 (100-400)		6 (3-11)		586 (266-1064)		17 (8-30)	
2008	United Kingdom									157 (71-284)		4 (2-8)		178 (81-323)		5 (2-9)		473 (215-859)		13 (6-23)	
2009	United Kingdom									168 (76-305)		4 (2-8)		191 (87-347)		5 (2-9)		508 (231-922)		13 (6-24)	
2010	United Kingdom									126 (57-229)		3 (1-6)		143 (65-260)		4 (2-7)		381 (173-692)		10 (4-18)	
2011	United Kingdom									64 (29-117)		2 (1-3)		73 (33-133)		2 (1-3)		195 (88-353)		5 (2-9)	
2012	United Kingdom									40 (18-72)		1 (0-2)		45 (20-82)		1 (0-2)		120 (54-217)		3 (1-5)	
2013	United Kingdom									23 (10-41)		1 (0-1)		26 (12-47)		1 (0-1)		68 (31-124)		2 (1-3)	
2014	United Kingdom									30 (14-54)		1 (0-1)		34 (15-62)		1 (0-2)		90 (41-164)		2 (1-4)	
2015	United Kingdom	753 (649-895)	4 (3-4)	19 (16-22)	0 (0-0)	488 (366-556)	2 (2-3)	12 (9-14)	0 (0-0)	27 (12-50)	0 (0-0)	1 (0-1)	0 (0-0)	31 (14-56)	0 (0-0)	1 (0-1)	0 (0-0)	82 (37-149)	0 (0-1)	2 (1-4)	0 (0-0)
2000	United Republic of Tanzania	124873 (107743-148439)		2150 (1855-2555)		49156 (36828-56067)		846 (634-965)		3373 (1424-6941)		58 (25-119)		1353 (571-2784)		23 (10-48)		10185 (4300-20960)		175 (74-361)	
2001	United Republic of Tanzania									3275 (1400-6709)		55 (23-112)		1314 (561-2691)		22 (9-45)		9889 (4226-20260)		165 (71-339)	
2002	United Republic of Tanzania									3146 (1361-6413)		51 (22-104)		1262 (546-2572)		20 (9-42)		9498 (4110-19363)		154 (67-313)	
2003	United Republic of Tanzania									3190 (1398-6468)		50 (22-101)		1280 (561-2594)		20 (9-40)		9634 (4222-19530)		150 (66-305)	
2004	United Republic of Tanzania									3127 (1387-6298)		47 (21-95)		1254 (556-2526)		19 (8-38)		9442 (4188-19017)		142 (63-286)	
2005	United Republic of Tanzania									3061 (1354-6148)		44 (20-89)		1228 (543-2466)		18 (8-36)		9244 (4088-18565)		134 (59-269)	
2006	United Republic of Tanzania									3005 (1361-5985)		42 (19-84)		1205 (546-2401)		17 (8-34)		9074 (4109-18072)		127 (57-253)	
2007	United Republic of Tanzania									2909 (1350-5744)		39 (18-78)		1167 (541-2304)		16 (7-31)		8784 (4075-17346)		119 (55-235)	
2008	United Republic of Tanzania									2861 (1189-5762)		38 (16-76)		3250 (1350-6544)		43 (18-86)		8640 (3590-17398)		114 (47-230)	
2009	United Republic of Tanzania									2842 (1219-5666)		37 (16-73)		3228 (1384-6435)		42 (18-83)		8581 (3680-17109)		110 (47-220)	
2010	United Republic of Tanzania									2836 (1256-5595)		35 (16-70)		3221 (1427-6355)		40 (18-79)		8563 (3793-16895)		107 (47-211)	
2011	United Republic of Tanzania									2807 (1260-5501)		34 (15-67)		3189 (1431-6248)		39 (17-76)		8477 (3803-16611)		103 (46-202)	
2012	United Republic of Tanzania									2815 (1274-5483)		33 (15-65)		3198 (1447-6227)		38 (17-73)		8501 (3847-16556)		100 (45-195)	
2013	United Republic of Tanzania									1882 (858-3650)		22 (10-42)		2138 (974-4146)		24 (11-47)		5684 (2590-11022)		65 (30-126)	
2014	United Republic of Tanzania									1456 (670-2813)		16 (7-31)		1654 (761-3195)		18 (8-35)		4397 (2024-8495)		49 (22-94)	
2015	United Republic of Tanzania	92663 (79952-110151)	8963 (7734-10655)	1003 (865-1192)	97 (84-115)	36429 (27293-41551)	3524 (2640-4019)	394 (295-450)	38 (29-43)	1292 (598-2489)	125 (58-241)	14 (6-27)	1 (1-3)	1467 (680-2827)	142 (66-273)	16 (7-31)	2 (1-3)	3900 (1807-7515)	377 (175-727)	42 (20-81)	4 (2-8)
2000	United States of America	21767 (18781-25874)		115 (99-137)		14111 (10572-16095)		75 (56-85)		1355 (431-2449)		7 (2-13)		1539 (489-2782)		8 (3-15)		4093 (1301-7395)		22 (7-39)	
2001	United States of America									1088 (346-1966)		6 (2-10)		1236 (393-2233)		7 (2-12)		3286 (1044-5937)		17 (6-32)	
2002	United States of America									891 (283-1610)		5 (1-9)		1012 (322-1829)		5 (2-10)		2691 (855-4861)		14 (5-26)	
2003	United States of America									757 (240-1367)		4 (1-7)		859 (273-1553)		4 (1-8)		2284 (726-4128)		12 (4-22)	
2004	United States of America									673 (214-1215)		3 (1-6)		764 (243-1380)		4 (1-7)		2031 (646-3670)		10 (3-19)	
2005	United States of America									616 (196-1114)		3 (1-6)		700 (223-1265)		4 (1-6)		1861 (592-3363)		10 (3-17)	
2006	United States of America									630 (200-1138)		3 (1-6)		716 (227-1293)		4 (1-6)		1903 (605-3438)		10 (3-17)	
2007	United States of America									577 (183-1043)		3 (1-5)		656 (208-1185)		3 (1-6)		1743 (554-3149)		9 (3-16)	
2008	United States of America									525 (167-950)		3 (1-5)		597 (190-1078)		3 (1-5)		1587 (504-2867)		8 (3-14)	
2009	United States of America									532 (169-961)		3 (1-5)		604 (192-1092)		3 (1-5)		1607 (511-2903)		8 (3-15)	
2010	United States of America									598 (190-1080)		3 (1-5)		679 (216-1226)		3 (1-6)		1804 (574-3260)		9 (3-16)	
2011	United States of America									209 (67-378)		1 (0-2)		238 (76-430)		1 (0-2)		633 (201-1143)		3 (1-6)	
2012	United States of America									136 (43-246)		1 (0-1)		155 (49-279)		1 (0-1)		411 (131-742)		2 (1-4)	
2013	United States of America									166 (53-299)		1 (0-2)		188 (60-340)		1 (0-2)		500 (159-904)		3 (1-5)	
2014	United States of America									126 (40-228)		1 (0-1)		143 (45-259)		1 (0-1)		380 (121-687)		2 (1-4)	
2015	United States of America	3654 (3153-4344)	2 (2-2)	19 (16-22)	0 (0-0)	2369 (1775-2702)	1 (1-1)	12 (9-14)	0 (0-0)	116 (37-210)	0 (0-0)	1 (0-1)	0 (0-0)	132 (42-238)	0 (0-0)	1 (0-1)	0 (0-0)	351 (112-634)	0 (0-0)	2 (1-3)	0 (0-0)
2000	Uruguay	3887 (3354-4621)		1458 (1258-1733)		1513 (1133-1725)		567 (425-647)		50 (24-77)		19 (9-29)		56 (28-87)		21 (10-33)		150 (74-232)		56 (28-87)	
2001	Uruguay									94 (46-146)		36 (18-56)		107 (53-166)		41 (20-63)		285 (140-441)		109 (53-169)	
2002	Uruguay									76 (37-118)		29 (14-46)		86 (42-134)		33 (16-52)		229 (113-355)		89 (44-138)	
2003	Uruguay									75 (37-117)		29 (14-46)		85 (42-132)		33 (16-52)		227 (112-352)		89 (44-138)	
2004	Uruguay									69 (34-107)		27 (13-42)		78 (38-121)		31 (15-48)		208 (102-322)		82 (40-127)	
2005	Uruguay									75 (37-117)		30 (15-46)		86 (42-133)		34 (17-53)		227 (112-352)		91 (44-140)	
2006	Uruguay									66 (33-103)		27 (13-41)		75 (37-117)		30 (15-47)		200 (98-310)		80 (39-124)	
2007	Uruguay									101 (50-156)		41 (20-63)		115 (56-178)		46 (23-72)		305 (150-473)		123 (60-191)	
2008	Uruguay									62 (30-96)		25 (12-39)		70 (35-109)		29 (14-44)		187 (92-290)		76 (37-118)	
2009	Uruguay									74 (36-114)		30 (15-47)		84 (41-129)		34 (17-53)		222 (109-344)		91 (45-141)	
2010	Uruguay									37 (18-58)		15 (8-24)		42 (21-66)		17 (9-27)		113 (55-174)		46 (23-72)	
2011	Uruguay									26 (13-41)		11 (5-17)		30 (15-47)		12 (6-19)		80 (39-124)		33 (16-51)	
2012	Uruguay									15 (7-23)		6 (3-10)		17 (8-26)		7 (3-11)		45 (22-70)		19 (9-29)	
2013	Uruguay									19 (9-30)		8 (4-12)		22 (11-34)		9 (4-14)		58 (28-90)		24 (12-38)	
2014	Uruguay									12 (6-19)		5 (3-8)		14 (7-22)		6 (3-9)		37 (18-58)		16 (8-24)	
2015	Uruguay	594 (513-706)	1 (1-1)	251 (217-298)	0 (0-0)	231 (173-264)	0 (0-0)	98 (73-111)	0 (0-0)	10 (5-16)	0 (0-0)	4 (2-7)	0 (0-0)	12 (6-18)	0 (0-0)	5 (2-8)	0 (0-0)	31 (15-48)	0 (0-0)	13 (6-20)	0 (0-0)
2000	Uzbekistan	6199 (5349-7369)		230 (198-273)		4019 (3011-4584)		149 (112-170)		646 (250-1359)		24 (9-50)		734 (284-1544)		27 (11-57)		1952 (756-4104)		72 (28-152)	
2001	Uzbekistan									609 (237-1278)		23 (9-49)		691 (269-1451)		26 (10-55)		1838 (716-3858)		70 (27-147)	
2002	Uzbekistan									574 (225-1202)		22 (9-47)		652 (256-1365)		25 (10-53)		1734 (679-3630)		68 (26-141)	
2003	Uzbekistan									542 (214-1132)		22 (8-45)		616 (243-1286)		24 (10-51)		1637 (645-3419)		65 (26-136)	
2004	Uzbekistan									520 (206-1083)		21 (8-43)		590 (234-1230)		24 (9-49)		1570 (622-3269)		63 (25-131)	
2005	Uzbekistan									499 (199-1037)		20 (8-42)		567 (226-1178)		23 (9-47)		1507 (601-3131)		60 (24-126)	
2006	Uzbekistan									486 (195-1008)		19 (8-40)		553 (222-1144)		22 (9-45)		1469 (589-3043)		58 (23-121)	
2007	Uzbekistan									477 (192-985)		18 (7-38)		541 (218-1118)		21 (8-43)		1439 (581-2973)		56 (23-115)	
2008	Uzbekistan									474 (192-975)		18 (7-37)		538 (218-1108)		20 (8-42)		1430 (580-2945)		54 (22-111)	
2009	Uzbekistan									470 (192-965)		17 (7-35)		533 (218-1096)		19 (8-40)		1418 (579-2913)		52 (21-106)	
2010	Uzbekistan									465 (191-952)		16 (7-34)		528 (217-1081)		19 (8-38)		1403 (576-2874)		50 (20-102)	
2011	Uzbekistan									455 (188-930)		15 (6-32)		517 (213-1056)		18 (7-36)		1375 (567-2808)		47 (19-95)	
2012	Uzbekistan									443 (184-903)		15 (6-30)		503 (209-1025)		17 (7-34)		1338 (555-2726)		44 (18-90)	
2013	Uzbekistan									429 (179-871)		14 (6-28)		487 (203-989)		16 (7-32)		1294 (540-2629)		42 (18-85)	
2014	Uzbekistan									415 (174-842)		13 (6-27)		472 (198-956)		15 (6-31)		1254 (526-2541)		40 (17-81)	
2015	Uzbekistan	7226 (6235-8590)	31 (27-37)	230 (198-273)	1 (1-1)	4685 (3510-5343)	20 (15-23)	149 (112-170)	1 (0-1)	395 (167-799)	2 (1-3)	13 (5-25)	0 (0-0)	449 (190-908)	2 (1-4)	14 (6-29)	0 (0-0)	1193 (504-2414)	5 (2-10)	38 (16-77)	0 (0-0)
2000	Vanuatu	552 (476-656)		1981 (1710-2355)		216 (162-247)		776 (582-886)		1 (0-4)		4 (1-15)		1 (0-5)		4 (1-17)		3 (1-13)		11 (3-45)	
2001	Vanuatu									1 (0-4)		3 (1-15)		1 (0-5)		4 (1-17)		3 (1-12)		10 (2-44)	
2002	Vanuatu									1 (0-3)		3 (1-11)		1 (0-4)		3 (1-13)		2 (1-10)		8 (2-34)	
2003	Vanuatu									1 (0-3)		2 (1-9)		1 (0-3)		3 (1-11)		2 (0-8)		7 (2-28)	
2004	Vanuatu									1 (0-3)		2 (1-10)		1 (0-3)		3 (1-11)		2 (0-9)		7 (2-30)	
2005	Vanuatu									1 (0-4)		3 (1-13)		1 (0-4)		3 (1-14)		3 (1-11)		9 (2-39)	
2006	Vanuatu									1 (0-4)		3 (1-14)		1 (0-5)		4 (1-15)		3 (1-12)		10 (2-41)	
2007	Vanuatu									1 (0-4)		3 (1-14)		1 (0-5)		4 (1-16)		3 (1-13)		10 (2-42)	
2008	Vanuatu									1 (0-4)		3 (1-14)		1 (0-5)		4 (1-16)		3 (1-14)		10 (2-43)	
2009	Vanuatu									1 (0-5)		3 (1-14)		1 (0-5)		4 (1-16)		3 (1-14)		10 (2-43)	
2010	Vanuatu									1 (0-5)		3 (1-14)		1 (0-5)		4 (1-16)		3 (1-14)		10 (2-43)	
2011	Vanuatu									2 (1-9)		6 (2-27)		2 (1-11)		7 (2-31)		7 (2-28)		19 (5-82)	
2012	Vanuatu									3 (1-12)		8 (2-34)		3 (1-13)		9 (2-38)		8 (2-36)		24 (6-102)	
2013	Vanuatu									3 (1-11)		8 (2-32)		3 (1-13)		9 (2-37)		8 (2-34)		23 (5-98)	
2014	Vanuatu									2 (1-11)		7 (2-31)		3 (1-12)		8 (2-35)		7 (2-32)		22 (5-93)	
2015	Vanuatu	584 (504-694)	0 (0-0)	1707 (1473-2029)	0 (0-0)	228 (171-260)	0 (0-0)	667 (500-761)	0 (0-0)	2 (1-10)	0 (0-0)	7 (2-29)	0 (0-0)	3 (1-11)	0 (0-0)	8 (2-33)	0 (0-0)	7 (2-30)	0 (0-0)	21 (5-88)	0 (0-0)
2000	Venezuela (Bolivarian Republic of)	39912 (34437-47445)		1441 (1243-1713)		15528 (11633-17711)		561 (420-639)		173 (44-504)		6 (2-18)		196 (50-572)		7 (2-21)		522 (134-1520)		19 (5-55)	
2001	Venezuela (Bolivarian Republic of)									195 (50-566)		7 (2-20)		221 (57-643)		8 (2-23)		589 (152-1710)		21 (5-61)	
2002	Venezuela (Bolivarian Republic of)									199 (52-577)		7 (2-21)		226 (59-655)		8 (2-23)		601 (156-1742)		21 (6-62)	
2003	Venezuela (Bolivarian Republic of)									140 (37-405)		5 (1-14)		159 (42-460)		6 (1-16)		423 (111-1222)		15 (4-44)	
2004	Venezuela (Bolivarian Republic of)									209 (55-604)		7 (2-21)		238 (63-686)		8 (2-24)		632 (167-1823)		22 (6-65)	
2005	Venezuela (Bolivarian Republic of)									186 (49-535)		7 (2-19)		212 (56-608)		7 (2-21)		562 (149-1617)		20 (5-57)	
2006	Venezuela (Bolivarian Republic of)									171 (46-491)		6 (2-17)		194 (52-557)		7 (2-20)		517 (138-1482)		18 (5-52)	
2007	Venezuela (Bolivarian Republic of)									152 (41-435)		5 (1-15)		173 (46-494)		6 (2-17)		459 (124-1313)		16 (4-46)	
2008	Venezuela (Bolivarian Republic of)									159 (43-453)		6 (1-16)		180 (49-514)		6 (2-18)		479 (129-1367)		17 (5-48)	
2009	Venezuela (Bolivarian Republic of)									145 (39-414)		5 (1-14)		165 (45-470)		6 (2-16)		439 (119-1250)		15 (4-43)	
2010	Venezuela (Bolivarian Republic of)									138 (38-394)		5 (1-14)		157 (43-447)		5 (1-16)		418 (113-1190)		14 (4-41)	
2011	Venezuela (Bolivarian Republic of)									177 (48-502)		6 (2-17)		201 (55-570)		7 (2-20)		533 (145-1516)		18 (5-52)	
2012	Venezuela (Bolivarian Republic of)									160 (44-454)		6 (2-16)		182 (50-516)		6 (2-18)		483 (132-1371)		17 (5-47)	
2013	Venezuela (Bolivarian Republic of)									153 (42-433)		5 (1-15)		174 (48-492)		6 (2-17)		461 (127-1307)		16 (4-45)	
2014	Venezuela (Bolivarian Republic of)									151 (42-428)		5 (1-15)		172 (47-486)		6 (2-17)		456 (126-1291)		16 (4-44)	
2015	Venezuela (Bolivarian Republic of)	25362 (21883-30148)	349 (301-415)	871 (752-1036)	12 (10-14)	9890 (7409-11280)	136 (102-155)	340 (255-388)	5 (4-5)	137 (38-386)	2 (1-5)	5 (1-13)	0 (0-0)	155 (43-439)	2 (1-6)	5 (1-15)	0 (0-0)	413 (115-1166)	6 (2-16)	14 (4-40)	0 (0-1)
2000	Viet Nam	139172 (120081-165436)		1954 (1686-2322)		54442 (40788-62097)		764 (573-872)		436 (157-931)		6 (2-13)		496 (178-1057)		7 (3-15)		1317 (474-2811)		18 (7-39)	

Spn = *Streptococcus pneumoniae*; npnm = non-pneumonia, non-meningitis; HIV = cases only in HIV-infected children; Uncertainty range provided in parentheses.

Year	Country	Spn pneumonia cases (UR)	Spn pneumonia cases, HIV (UR)	Spn pneumonia incidence rate (UR)	Spn pneumonia incidence rate, HIV (UR)	Spn severe pneumonia cases (UR)	Spn severe pneumonia cases, HIV (UR)	Spn severe pneumonia incidence rate (UR)	Spn severe pneumonia incidence rate, HIV (UR)	Spn meningitis cases (UR)	Spn meningitis cases, HIV (UR)	Spn meningitis incidence rate (UR)	Spn meningitis incidence rate, HIV (UR)	Spn NPNM cases (UR)	Spn NPNM cases, HIV (UR)	Spn NPNM incidence rate (UR)	Spn NPNM incidence rate, HIV (UR)	Spn NPNM non-severe cases (UR)	Spn NPNM non-severe cases, HIV (UR)	SPN NPNM non-severe incidence rate (UR)	SPN NPNM non-severe incidence rate, HIV (UR)
2001	Viet Nam									433 (167-905)		7 (3-14)		492 (190-1028)		7 (3-16)		1309 (506-2732)		20 (8-41)	
2002	Viet Nam									466 (194-947)		7 (3-15)		529 (221-1076)		8 (3-17)		1406 (586-2861)		22 (9-44)	
2003	Viet Nam									520 (225-1034)		8 (3-16)		590 (256-1175)		9 (4-18)		1569 (679-3123)		24 (10-48)	
2004	Viet Nam									591 (214-1380)		9 (3-21)		672 (243-1567)		10 (4-24)		1786 (645-4167)		27 (10-63)	
2005	Viet Nam									646 (251-1468)		10 (4-22)		734 (285-1668)		11 (4-25)		1951 (758-4433)		29 (11-67)	
2006	Viet Nam									669 (284-1487)		10 (4-22)		760 (322-1689)		11 (5-25)		2021 (856-4490)		29 (12-65)	
2007	Viet Nam									641 (254-1436)		9 (4-21)		728 (289-1631)		10 (4-23)		1936 (768-4336)		28 (11-62)	
2008	Viet Nam									574 (215-1305)		8 (3-19)		652 (245-1482)		9 (3-21)		1733 (651-3939)		25 (9-56)	
2009	Viet Nam									511 (183-1181)		7 (3-17)		581 (208-1342)		8 (3-19)		1544 (552-3567)		22 (8-51)	
2010	Viet Nam									776 (265-1818)		11 (4-25)		882 (300-2064)		12 (4-29)		2344 (799-5489)		33 (11-77)	
2011	Viet Nam									897 (291-2115)		12 (4-29)		1019 (330-2403)		14 (5-33)		2708 (877-6388)		37 (12-88)	
2012	Viet Nam									887 (307-2064)		12 (4-28)		1007 (348-2344)		14 (5-32)		2678 (926-6231)		36 (13-84)	
2013	Viet Nam									843 (312-1928)		11 (4-26)		958 (355-2189)		13 (5-29)		2546 (943-5821)		34 (13-78)	
2014	Viet Nam									889 (354-1991)		12 (5-26)		1010 (402-2261)		13 (5-30)		2684 (1068-6012)		35 (14-79)	
2015	Viet Nam	134841 (116344-160288)	1663 (1435-1977)	1771 (1528-2106)	22 (19-26)	52475 (39314-59852)	647 (485-738)	689 (516-786)	9 (6-10)	922 (397-2020)	11 (5-25)	12 (5-27)	0 (0-0)	1048 (451-2294)	13 (6-28)	14 (6-30)	0 (0-0)	2785 (1200-6099)	34 (15-75)	37 (16-80)	0 (0-1)
2000	Yemen	44753 (38614-53199)		1451 (1252-1724)		17426 (13055-19876)		565 (423-644)		855 (331-1860)		28 (11-60)		343 (133-746)		11 (4-24)		2582 (999-5617)		84 (32-182)	
2001	Yemen									801 (306-1748)		26 (10-57)		321 (123-701)		10 (4-23)		2418 (925-5277)		79 (30-172)	
2002	Yemen									755 (286-1655)		24 (9-53)		303 (115-664)		10 (4-21)		2280 (865-4998)		74 (28-161)	
2003	Yemen									719 (271-1585)		23 (9-50)		288 (109-636)		9 (3-20)		2171 (817-4786)		69 (26-152)	
2004	Yemen									684 (256-1517)		21 (8-47)		274 (103-608)		9 (3-19)		2066 (772-4581)		64 (24-142)	
2005	Yemen									649 (201-1493)		20 (6-45)		737 (228-1696)		22 (7-52)		1959 (607-4508)		60 (18-137)	
2006	Yemen									610 (187-1412)		18 (6-42)		693 (213-1604)		20 (6-47)		1841 (566-4263)		54 (17-126)	
2007	Yemen									570 (173-1327)		17 (5-38)		647 (196-1508)		19 (6-44)		1721 (522-4008)		50 (15-116)	
2008	Yemen									528 (158-1237)		15 (5-35)		600 (180-1405)		17 (5-40)		1595 (478-3736)		45 (14-107)	
2009	Yemen									491 (145-1155)		14 (4-32)		557 (165-1312)		16 (5-37)		1482 (439-3487)		42 (12-98)	
2010	Yemen									455 (133-1075)		13 (4-30)		517 (151-1221)		14 (4-34)		1374 (401-3247)		38 (11-90)	
2011	Yemen									333 (96-790)		9 (3-21)		378 (109-897)		10 (3-24)		1005 (290-2385)		27 (8-65)	
2012	Yemen									248 (71-592)		7 (2-16)		282 (80-673)		8 (2-18)		750 (214-1788)		20 (6-48)	
2013	Yemen									218 (61-521)		6 (2-14)		247 (70-592)		7 (2-16)		657 (185-1573)		17 (5-42)	
2014	Yemen									203 (56-487)		5 (1-13)		230 (64-553)		6 (2-14)		612 (170-1470)		16 (4-38)	
2015	Yemen	23439 (20223-27862)	52 (45-62)	607 (524-722)	1 (1-2)	9197 (6891-10490)	20 (15-23)	238 (179-272)	1 (0-1)	111 (30-267)	0 (0-1)	3 (1-7)	0 (0-0)	126 (35-303)	0 (0-1)	3 (1-8)	0 (0-0)	335 (92-806)	1 (0-2)	9 (2-21)	0 (0-0)
2000	Zambia	61470 (53037-73070)		3260 (2813-3875)		24037 (18008-27417)		1275 (955-1454)		1170 (717-1946)		62 (38-103)		469 (288-781)		25 (15-41)		3534 (2166-5877)		187 (115-312)	
2001	Zambia									1169 (726-1951)		60 (37-100)		469 (291-782)		24 (15-40)		3529 (2192-5891)		181 (113-302)	
2002	Zambia									1153 (550-2163)		57 (27-108)		462 (221-868)		23 (11-43)		3481 (1662-6532)		173 (83-326)	
2003	Zambia									1117 (536-2112)		54 (26-102)		448 (215-847)		22 (10-41)		3374 (1617-6376)		164 (78-309)	
2004	Zambia									1089 (524-2071)		51 (25-98)		437 (210-831)		21 (10-39)		3288 (1581-6254)		155 (75-295)	
2005	Zambia									1056 (509-2017)		48 (23-92)		424 (204-809)		19 (9-37)		3189 (1536-6092)		146 (70-279)	
2006	Zambia									1019 (490-1948)		45 (22-86)		409 (196-781)		18 (9-35)		3077 (1478-5883)		136 (66-261)	
2007	Zambia									992 (475-1898)		43 (20-82)		398 (191-761)		17 (8-33)		2995 (1436-5730)		129 (62-247)	
2008	Zambia									970 (467-1854)		41 (20-78)		389 (187-744)		16 (8-31)		2929 (1409-5599)		123 (59-235)	
2009	Zambia									945 (456-1805)		39 (19-74)		379 (183-724)		15 (7-30)		2853 (1377-5449)		116 (56-222)	
2010	Zambia									921 (447-1759)		37 (18-70)		369 (179-706)		15 (7-28)		2780 (1348-5312)		111 (54-211)	
2011	Zambia									896 (437-1715)		35 (17-67)		360 (175-688)		14 (7-27)		2707 (1320-5180)		105 (51-201)	
2012	Zambia									870 (376-1714)		33 (14-65)		988 (428-1947)		38 (16-74)		2627 (1137-5177)		100 (43-197)	
2013	Zambia									840 (366-1659)		31 (14-62)		955 (416-1884)		36 (15-70)		2538 (1106-5010)		94 (41-186)	
2014	Zambia									583 (255-1153)		21 (9-42)		662 (289-1310)		24 (11-48)		1761 (769-3482)		64 (28-127)	
2015	Zambia	39873 (34404-47398)	9461 (8163-11246)	1422 (1227-1691)	337 (291-401)	15597 (11685-17790)	3701 (2772-4221)	556 (417-635)	132 (99-151)	481 (212-950)	114 (50-225)	17 (8-34)	4 (2-8)	546 (241-1079)	130 (57-256)	19 (9-38)	5 (2-9)	1452 (640-2869)	345 (152-681)	52 (23-102)	12 (5-24)
2000	Zimbabwe	46175 (39841-54889)		2555 (2205-3037)		18068 (13536-20608)		1000 (749-1140)		320 (109-732)		18 (6-40)		128 (44-293)		7 (2-16)		965 (329-2209)		53 (18-122)	
2001	Zimbabwe									334 (114-764)		18 (6-42)		134 (46-306)		7 (3-17)		1008 (343-2307)		55 (19-127)	
2002	Zimbabwe									356 (121-814)		19 (7-44)		143 (49-327)		8 (3-18)		1075 (366-2459)		59 (20-134)	
2003	Zimbabwe									384 (131-878)		21 (7-47)		154 (53-352)		8 (3-19)		1159 (395-2651)		63 (21-143)	
2004	Zimbabwe									418 (143-955)		22 (8-51)		168 (57-383)		9 (3-20)		1262 (431-2884)		67 (23-154)	
2005	Zimbabwe									460 (157-1050)		24 (8-55)		185 (63-421)		10 (3-22)		1390 (474-3171)		73 (25-166)	
2006	Zimbabwe									503 (171-1145)		26 (9-59)		202 (69-459)		10 (4-24)		1520 (517-3456)		78 (27-177)	
2007	Zimbabwe									553 (196-1239)		28 (10-62)		222 (79-497)		11 (4-25)		1670 (593-3742)		84 (30-187)	
2008	Zimbabwe									606 (225-1335)		30 (11-65)		243 (90-536)		12 (4-26)		1829 (679-4032)		89 (33-197)	
2009	Zimbabwe									654 (254-1415)		31 (12-67)		262 (102-568)		12 (5-27)		1973 (768-4272)		94 (36-203)	
2010	Zimbabwe									696 (283-1480)		32 (13-68)		279 (114-594)		13 (5-27)		2100 (855-4469)		97 (39-206)	
2011	Zimbabwe									731 (296-1546)		33 (13-69)		293 (119-620)		13 (5-28)		2206 (893-4668)		98 (40-208)	
2012	Zimbabwe									679 (284-1421)		29 (12-62)		272 (114-570)		12 (5-25)		2051 (857-4290)		89 (37-186)	
2013	Zimbabwe									444 (164-946)		19 (7-40)		504 (186-1074)		21 (8-45)		1339 (494-2856)		57 (21-121)	
2014	Zimbabwe									400 (154-844)		17 (6-35)		454 (175-959)		19 (7-40)		1208 (466-2550)		50 (19-105)	
2015	Zimbabwe	23202 (20019-27580)	6702 (5783-7967)	942 (813-1120)	272 (235-323)	9092 (6812-10371)	2627 (1968-2996)	369 (277-421)	107 (80-122)	395 (153-832)	114 (44-240)	16 (6-34)	5 (2-10)	449 (174-945)	130 (50-273)	18 (7-38)	5 (2-11)	1193 (462-2513)	345 (133-726)	48 (19-102)	14 (5-29)

Spn = *Streptococcus pneumoniae*; npnm = non-pneumonia, non-meningitis; HIV = cases only in HIV-infected children; Uncertainty range provided in parentheses.

Table 10: *Haemophilus influenzae* type b morbidity by syndrome, country, and year

		Pneumonia cases				Severe pneumonia cases				Meningitis cases				NPNM cases			
Year	Country	Hib pneumonia cases (UR)	Hib pneumonia cases, HIV (UR)	Hib pneumonia incidence rate (UR)	Hib pneumonia incidence rate, HIV (UR)	Hib severe pneumonia cases (UR)	Hib severe pneumonia cases, HIV (UR)	Hib severe pneumonia incidence rate (UR)	Hib severe pneumonia incidence rate, HIV (UR)	Hib meningitis cases (UR)	Hib meningitis cases, HIV (UR)	Hib meningitis incidence rate (UR)	Hib meningitis incidence rate, HIV (UR)	Hib NPNM cases (UR)	Hib NPNM cases, HIV (UR)	Hib NPNM incidence rate (UR)	Hib NPNM incidence rate, HIV (UR)
2000	Afghanistan	123938 (113158-202503)		3200 (2922-5229)		23227 (13907-46882)		600 (359-1211)		3707 (1341-5799)		96 (35-150)		638 (231-999)		16 (6-26)	
2001	Afghanistan									3778 (1372-5905)		94 (34-146)		650 (236-1017)		16 (6-25)	
2002	Afghanistan									3799 (1387-5932)		91 (33-142)		654 (239-1021)		16 (6-24)	
2003	Afghanistan									3815 (1399-5951)		88 (32-137)		657 (241-1025)		15 (6-24)	
2004	Afghanistan									3800 (1401-5922)		85 (31-132)		654 (241-1020)		15 (5-23)	
2005	Afghanistan									3749 (1390-5837)		82 (30-127)		646 (239-1005)		14 (5-22)	
2006	Afghanistan									3707 (1381-5766)		79 (29-123)		638 (238-993)		14 (5-21)	
2007	Afghanistan									3626 (1358-5636)		76 (28-118)		624 (234-970)		13 (5-20)	
2008	Afghanistan									3527 (1326-5476)		73 (27-113)		607 (228-943)		13 (5-19)	
2009	Afghanistan									1271 (480-1972)		26 (10-40)		219 (83-340)		4 (2-7)	
2010	Afghanistan									400 (152-620)		8 (3-13)		69 (26-107)		1 (1-2)	
2011	Afghanistan									372 (141-576)		8 (3-12)		64 (24-99)		1 (0-2)	
2012	Afghanistan									360 (137-557)		7 (3-11)		62 (24-96)		1 (0-2)	
2013	Afghanistan									329 (126-508)		7 (3-10)		57 (22-88)		1 (0-2)	
2014	Afghanistan									27 (10-42)		1 (0-1)		5 (2-7)		0 (0-0)	
2015	Afghanistan	1120 (1022-1830)	0 (0-1)	23 (21-38)	0 (0-0)	212 (127-427)	0 (0-0)	4 (3-9)	0 (0-0)	26 (10-40)	0 (0-0)	1 (0-1)	0 (0-0)	5 (2-7)	0 (0-0)	0 (0-0)	0 (0-0)
2000	Albania	408 (372-667)		149 (136-243)		127 (76-256)		46 (28-93)		38 (7-78)		14 (3-28)		13 (2-26)		5 (1-10)	
2001	Albania									30 (4-63)		11 (2-24)		10 (1-21)		4 (1-8)	
2002	Albania									22 (3-49)		9 (1-20)		7 (1-16)		3 (0-7)	
2003	Albania									17 (2-38)		7 (1-16)		6 (1-13)		2 (0-5)	
2004	Albania									14 (1-31)		6 (1-14)		5 (0-11)		2 (0-5)	
2005	Albania									12 (1-27)		6 (0-13)		4 (0-9)		2 (0-4)	
2006	Albania									10 (1-24)		5 (0-12)		4 (0-8)		2 (0-4)	
2007	Albania									10 (1-22)		5 (1-12)		3 (0-7)		2 (0-4)	
2008	Albania									10 (1-21)		6 (1-12)		3 (0-7)		2 (0-4)	
2009	Albania									2 (0-5)		2 (0-3)		1 (0-2)		1 (0-1)	
2010	Albania									1 (0-2)		1 (0-1)		0 (0-1)		0 (0-0)	
2011	Albania									1 (0-2)		1 (0-2)		0 (0-1)		0 (0-1)	
2012	Albania									1 (0-2)		1 (0-2)		0 (0-1)		0 (0-1)	
2013	Albania									1 (0-2)		1 (0-1)		0 (0-1)		0 (0-1)	
2014	Albania									0 (0-0)		0 (0-0)		0 (0-0)		0 (0-0)	
2015	Albania	3 (2-4)	0 (0-0)	1 (1-2)	0 (0-0)	1 (1-2)	0 (0-0)	0 (0-1)	0 (0-0)	0 (0-0)	0 (0-0)	0 (0-0)	0 (0-0)	0 (0-0)	0 (0-0)	0 (0-0)	0 (0-0)
2000	Algeria	42722 (39006-69804)		1407 (1285-2299)		8052 (4821-16252)		265 (159-535)		246 (78-406)		8 (3-13)		83 (26-137)		3 (1-5)	
2001	Algeria									235 (74-387)		8 (3-13)		79 (25-131)		3 (1-4)	
2002	Algeria									224 (71-369)		8 (3-13)		76 (24-124)		3 (1-4)	
2003	Algeria									216 (69-355)		8 (2-13)		73 (23-120)		3 (1-4)	
2004	Algeria									211 (67-346)		7 (2-12)		71 (23-117)		2 (1-4)	
2005	Algeria									209 (67-344)		7 (2-12)		71 (23-116)		2 (1-4)	
2006	Algeria									218 (70-359)		7 (2-12)		73 (24-121)		2 (1-4)	
2007	Algeria									230 (76-378)		7 (2-12)		78 (26-127)		2 (1-4)	
2008	Algeria									39 (5-69)		1 (0-2)		13 (2-23)		0 (0-1)	
2009	Algeria									20 (3-34)		1 (0-1)		7 (1-12)		0 (0-0)	
2010	Algeria									21 (3-37)		1 (0-1)		7 (1-12)		0 (0-0)	
2011	Algeria									22 (3-38)		1 (0-1)		7 (1-13)		0 (0-0)	
2012	Algeria									23 (3-39)		1 (0-1)		8 (1-13)		0 (0-0)	
2013	Algeria									3 (0-6)		0 (0-0)		1 (0-2)		0 (0-0)	
2014	Algeria									3 (0-6)		0 (0-0)		1 (0-2)		0 (0-0)	
2015	Algeria	484 (442-792)	0 (0-0)	11 (10-18)	0 (0-0)	91 (55-184)	0 (0-0)	2 (1-4)	0 (0-0)	3 (0-6)	0 (0-0)	0 (0-0)	0 (0-0)	1 (0-2)	0 (0-0)	0 (0-0)	0 (0-0)
2000	Andorra	0 (0-0)		6 (6-10)		0 (0-0)		2 (1-4)		0 (0-0)		1 (0-1)		0 (0-0)		0 (0-0)	
2001	Andorra									0 (0-0)		1 (0-1)		0 (0-0)		0 (0-0)	
2002	Andorra									0 (0-0)		1 (0-1)		0 (0-0)		0 (0-0)	
2003	Andorra									0 (0-0)		0 (0-0)		0 (0-0)		0 (0-0)	
2004	Andorra									0 (0-0)		0 (0-0)		0 (0-0)		0 (0-0)	
2005	Andorra									0 (0-0)		0 (0-0)		0 (0-0)		0 (0-0)	
2006	Andorra									0 (0-0)		0 (0-0)		0 (0-0)		0 (0-0)	
2007	Andorra									0 (0-0)		0 (0-0)		0 (0-0)		0 (0-0)	
2008	Andorra									0 (0-0)		0 (0-0)		0 (0-0)		0 (0-0)	
2009	Andorra									0 (0-0)		0 (0-0)		0 (0-0)		0 (0-0)	
2010	Andorra									0 (0-0)		0 (0-0)		0 (0-0)		0 (0-0)	
2011	Andorra									0 (0-0)		0 (0-0)		0 (0-0)		0 (0-0)	
2012	Andorra									0 (0-0)		0 (0-0)		0 (0-0)		0 (0-0)	
2013	Andorra									0 (0-0)		0 (0-0)		0 (0-0)		0 (0-0)	
2014	Andorra									0 (0-0)		0 (0-0)		0 (0-0)		0 (0-0)	
2015	Andorra	0 (0-0)	0 (0-0)	1 (1-1)	0 (0-0)	0 (0-0)	0 (0-0)	0 (0-0)	0 (0-0)	0 (0-0)	0 (0-0)	0 (0-0)	0 (0-0)	0 (0-0)	0 (0-0)	0 (0-0)	0 (0-0)
2000	Angola	43481 (39699-71043)		1481 (1352-2420)		8207 (4914-16566)		280 (167-564)		1898 (869-2831)		65 (30-96)		327 (150-487)		11 (5-17)	
2001	Angola									1921 (880-2863)		63 (29-94)		331 (152-493)		11 (5-16)	
2002	Angola									1990 (913-2966)		63 (29-94)		343 (157-511)		11 (5-16)	
2003	Angola									2009 (923-2993)		61 (28-92)		346 (159-515)		11 (5-16)	
2004	Angola									2031 (934-3025)		60 (28-89)		350 (161-521)		10 (5-15)	
2005	Angola									2071 (954-3083)		59 (27-88)		357 (164-531)		10 (5-15)	
2006	Angola									2112 (974-3143)		58 (27-87)		364 (168-541)		10 (5-15)	
2007	Angola									539 (249-802)		14 (7-21)		93 (43-138)		2 (1-4)	
2008	Angola									255 (118-379)		7 (3-10)		44 (20-65)		1 (1-2)	
2009	Angola									286 (133-425)		7 (3-11)		49 (23-73)		1 (1-2)	
2010	Angola									227 (106-338)		6 (3-8)		39 (18-58)		1 (0-1)	
2011	Angola									245 (115-364)		6 (3-9)		42 (20-63)		1 (0-1)	
2012	Angola									22 (10-32)		1 (0-1)		4 (2-6)		0 (0-0)	
2013	Angola									21 (10-32)		0 (0-1)		4 (2-5)		0 (0-0)	
2014	Angola									267 (126-395)		6 (3-9)		46 (22-68)		1 (0-2)	
2015	Angola	4356 (3977-7117)	105 (96-171)	94 (86-153)	2 (2-0)	813 (487-1642)	20 (12-39)	18 (10-35)	0 (0-0)	262 (124-387)	6 (3-9)	6 (3-8)	0 (0-0)	45 (21-67)	1 (1-2)	1 (0-1)	0 (0-0)
2000	Antigua and Barbuda	72 (66-118)		968 (884-1582)		13 (8-27)		180 (108-364)		2 (1-4)		33 (15-59)		1 (0-1)		11 (5-20)	
2001	Antigua and Barbuda									1 (0-2)		13 (6-22)		0 (0-1)		4 (2-8)	
2002	Antigua and Barbuda									0 (0-1)		6 (3-11)		0 (0-0)		2 (1-4)	
2003	Antigua and Barbuda									0 (0-1)		4 (2-8)		0 (0-0)		1 (1-3)	
2004	Antigua and Barbuda									0 (0-0)		3 (1-5)		0 (0-0)		1 (0-2)	
2005	Antigua and Barbuda									0 (0-0)		3 (1-5)		0 (0-0)		1 (0-2)	
2006	Antigua and Barbuda									0 (0-0)		0 (0-1)		0 (0-0)		0 (0-0)	
2007	Antigua and Barbuda									0 (0-0)		0 (0-0)		0 (0-0)		0 (0-0)	
2008	Antigua and Barbuda									0 (0-0)		0 (0-0)		0 (0-0)		0 (0-0)	
2009	Antigua and Barbuda									0 (0-0)		0 (0-0)		0 (0-0)		0 (0-0)	
2010	Antigua and Barbuda									0 (0-0)		0 (0-0)		0 (0-0)		0 (0-0)	
2011	Antigua and Barbuda									0 (0-0)		0 (0-0)		0 (0-0)		0 (0-0)	
2012	Antigua and Barbuda									0 (0-0)		0 (0-0)		0 (0-0)		0 (0-0)	
2013	Antigua and Barbuda									0 (0-0)		0 (0-0)		0 (0-0)		0 (0-0)	
2014	Antigua and Barbuda									0 (0-0)		0 (0-0)		0 (0-0)		0 (0-0)	
2015	Antigua and Barbuda	0 (0-1)	0 (0-0)	6 (5-10)	0 (0-0)	0 (0-0)	0 (0-0)	1 (1-2)	0 (0-0)	0 (0-0)	0 (0-0)	0 (0-0)	0 (0-0)	0 (0-0)	0 (0-0)	0 (0-0)	0 (0-0)
2000	Argentina	2982 (2723-4872)		87 (80-143)		556 (333-1121)		16 (10-33)		93 (28-154)		3 (1-4)		31 (9-52)		1 (0-2)	
2001	Argentina									84 (25-139)		2 (1-4)		28 (9-47)		1 (0-1)	
2002	Argentina									75 (23-123)		2 (1-4)		25 (8-42)		1 (0-1)	
2003	Argentina									12 (4-19)		0 (0-1)		4 (1-7)		0 (0-0)	
2004	Argentina									12 (4-20)		0 (0-1)		4 (1-7)		0 (0-0)	
2005	Argentina									11 (3-18)		0 (0-1)		4 (1-6)		0 (0-0)	
2006	Argentina									10 (3-17)		0 (0-0)		3 (1-6)		0 (0-0)	
2007	Argentina									9 (3-14)		0 (0-0)		3 (1-5)		0 (0-0)	
2008	Argentina									9 (3-15)		0 (0-0)		3 (1-5)		0 (0-0)	
2009	Argentina									9 (3-14)		0 (0-0)		3 (1-5)		0 (0-0)	
2010	Argentina									10 (3-16)		0 (0-0)		3 (1-5)		0 (0-0)	
2011	Argentina									7 (2-12)		0 (0-0)		2 (1-4)		0 (0-0)	
2012	Argentina									8 (3-13)		0 (0-0)		3 (1-5)		0 (0-0)	
2013	Argentina									8 (3-13)		0 (0-0)		3 (1-4)		0 (0-0)	
2014	Argentina									8 (3-14)		0 (0-0)		3 (1-5)		0 (0-0)	
2015	Argentina	220 (200-359)	0 (0-0)	6 (5-10)	0 (0-0)	41 (25-83)	0 (0-0)	1 (1-2)	0 (0-0)	8 (3-13)	0 (0-0)	0 (0-0)	0 (0-0)	3 (1-5)	0 (0-0)	0 (0-0)	0 (0-0)
2000	Armenia	289 (263-471)		149 (136-243)		90 (54-181)		46 (28-93)		14 (4-23)		7 (2-12)		5 (1-8)		2 (1-4)	
2001	Armenia									13 (1-23)		7 (1-13)		4 (0-8)		2 (0-4)	
2002	Armenia									13 (1-23)		7 (1-13)		4 (0-8)		2 (0-4)	
2003	Armenia									13 (1-24)		7 (1-13)		5 (0-8)		2 (0-4)	
2004	Armenia									14 (1-24)		7 (1-13)		5 (0-8)		2 (0-4)	
2005	Armenia									14 (1-24)		7 (1-12)		5 (0-8)		2 (0-4)	
2006	Armenia									13 (1-24)		7 (1-12)		5 (0-8)		2 (0-4)	
2007	Armenia									13 (1-23)		6 (1-11)		5 (0-8)		2 (0-4)	
2008	Armenia									13 (1-23)		6 (1-11)		4 (0-8)		2 (0-4)	
2009	Armenia									13 (2-22)		6 (1-11)		4 (1-8)		2 (0-4)	
2010	Armenia									11 (1-18)		5 (1-9)		4 (0-6)		2 (0-3)	
2011	Armenia									2 (0-3)		1 (0-2)		1 (0-1)		0 (0-1)	
2012	Armenia									2 (0-3)		1 (0-1)		1 (0-1)		0 (0-0)	
2013	Armenia									2 (0-3)		1 (0-1)		1 (0-1)		0 (0-0)	
2014	Armenia									2 (0-3)		1 (0-1)		1 (0-1)		0 (0-0)	
2015	Armenia	3 (3-5)	0 (0-0)	1 (1-2)	0 (0-0)	1 (1-2)	0 (0-0)	0 (0-1)	0 (0-0)	0 (0-0)	0 (0-0)	0 (0-0)	0 (0-0)	0 (0-0)	0 (0-0)	0 (0-0)	0 (0-0)
2000	Australia	127 (116-208)		10 (9-16)		40 (24-80)		3 (2-6)		32 (18-45)		3 (1-4)		11 (6-15)		1 (0-1)	
2001	Australia									41 (24-58)		3 (2-5)		14 (8-20)		1 (1-2)	
2002	Australia									2 (1-3)		0 (0-0)		1 (0-1)		0 (0-0)	
2003	Australia									4 (2-6)		0 (0-0)		1 (1-2)		0 (0-0)	
2004	Australia									4 (3-6)		0 (0-1)		2 (1-2)		0 (0-0)	
2005	Australia									3 (2-4)		0 (0-0)		1 (1-1)		0 (0-0)	
2006	Australia									3 (2-5)		0 (0-0)		1 (1-2)		0 (0-0)	
2007	Australia									2 (1-3)		0 (0-0)		1 (0-1)		0 (0-0)	
2008	Australia									3 (1-4)		0 (0-0)		1 (0-1)		0 (0-0)	
2009	Australia									1 (1-2)		0 (0-0)		0 (0-1)		0 (0-0)	
2010	Australia									3 (2-4)		0 (0-0)		1 (1-1)		0 (0-0)	
2011	Australia									2 (1-3)		0 (0-0)		1 (0-1)		0 (0-0)	
2012	Australia									2 (1-3)		0 (0-0)		1 (0-1)		0 (0-0)	
2013	Australia									2 (1-3)		0 (0-0)		1 (0-1)		0 (0-0)	
2014	Australia									2 (1-3)		0 (0-0)		1 (0-1)		0 (0-0)	
2015	Australia	19 (17-31)	0 (0-0)	1 (1-2)	0 (0-0)	6 (4-12)	0 (0-0)	0 (0-1)	0 (0-0)	2 (1-3)	0 (0-0)	0 (0-0)	0 (0-0)	1 (0-1)	0 (0-0)	0 (0-0)	0 (0-0)
2000	Austria	34 (31-56)		8 (8-14)		11 (6-21)		3 (2-5)		4 (2-8)		1 (0-2)		1 (1-3)		0 (0-1)	
2001	Austria									4 (2-8)		1 (1-2)		2 (1-3)		0 (0-1)	
2002	Austria									2 (1-5)		1 (0-1)		1 (0-2)		0 (0-0)	
2003	Austria									4 (2-7)		1 (0-2)		1 (1-2)		0 (0-1)	
2004	Austria									0 (0-1)		0 (0-0)		0 (0-0)		0 (0-0)	
2005	Austria									0 (0-1)		0 (0-0)		0 (0-0)		0 (0-0)	
2006	Austria									0 (0-1)		0 (0-0)		0 (0-0)		0 (0-0)	
2007	Austria									0 (0-0)		0 (0-0)		0 (0-0)		0 (0-0)	
2008	Austria									0 (0-1)		0 (0-0)		0 (0-0)		0 (0-0)	
2009	Austria									1 (0-1)		0 (0-0)		0 (0-0)		0 (0-0)	
2010	Austria									1 (0-1)		0 (0-0)		0 (0-0)		0 (0-0)	
2011	Austria									0 (0-0)		0 (0-0)		0 (0-0)		0 (0-0)	
2012	Austria									0 (0-1)		0 (0-0)		0 (0-0)		0 (0-0)	
2013	Austria									0 (0-1)		0 (0-0)		0 (0-0)		0 (0-0)	
2014	Austria									0 (0-0)		0 (0-0)		0 (0-0)		0 (0-0)	
2015	Austria	3 (3-5)	0 (0-0)	1 (1-1)	0 (0-0)	1 (1-2)	0 (0-0)	0 (0-0)	0 (0-0)	0 (0-1)	0 (0-0)	0 (0-0)	0 (0-0)	0 (0-0)	0 (0-0)	0 (0-0)	0 (0-0)
2000	Azerbaijan	1023 (934-1671)		149 (136-243)		318 (190-641)		46 (28-93)		139 (38-230)		20 (6-33)		47 (13-78)		7 (2-11)	
2001	Azerbaijan									120 (33-198)		18 (5-31)		40 (11-67)		6 (2-10)	
2002	Azerbaijan									105 (30-174)		17 (5-28)		36 (10-59)		6 (2-9)	
2003	Azerbaijan									94 (27-155)		15 (4-25)		32 (9-52)		5 (1-9)	
2004	Azerbaijan									87 (25-142)		14 (4-24)		29 (8-48)		5 (1-8)	
2005	Azerbaijan									79 (23-130)		13 (4-22)		27 (8-44)		4 (1-7)	
2006	Azerbaijan									73 (21-120)		12 (3-20)		25 (7-40)		4 (1-7)	
2007	Azerbaijan									68 (20-111)		11 (3-18)		23 (7-37)		4 (1-6)	

Hib = *Haemophilus influenzae* type b; npnm = non-pneumonia, non-meningitis; HIV = cases only in HIV-infected children; Uncertainty range provided in parentheses.

Year	Country	Hib pneumonia cases (UR)	Hib pneumonia cases, HIV (UR)	Hib pneumonia incidence rate (UR)	Hib pneumonia incidence rate, HIV (UR)	Hib severe pneumonia cases (UR)	Hib severe pneumonia cases, HIV (UR)	Hib severe pneumonia incidence rate (UR)	Hib severe pneumonia incidence rate, HIV (UR)	Hib meningitis cases (UR)	Hib meningitis cases, HIV (UR)	Hib meningitis incidence rate (UR)	Hib meningitis incidence rate, HIV (UR)	Hib NPNM cases (UR)	Hib NPNM cases, HIV (UR)	Hib NPNM incidence rate (UR)	Hib NPNM incidence rate, HIV (UR)
2008	Azerbaijan									64 (19-105)		10 (3-16)		22 (6-35)		3 (1-6)	
2009	Azerbaijan									62 (19-101)		9 (3-15)		21 (6-34)		3 (1-5)	
2010	Azerbaijan									62 (19-101)		9 (3-14)		21 (6-34)		3 (1-5)	
2011	Azerbaijan									45 (14-73)		6 (2-10)		15 (5-25)		2 (1-3)	
2012	Azerbaijan									6 (2-9)		1 (0-1)		2 (1-3)		0 (0-0)	
2013	Azerbaijan									5 (2-9)		1 (0-1)		2 (1-3)		0 (0-0)	
2014	Azerbaijan									5 (2-9)		1 (0-1)		2 (1-3)		0 (0-0)	
2015	Azerbaijan	97 (88-158)	0 (0-0)	11 (10-17)	0 (0-0)	30 (18-61)	0 (0-0)	3 (2-7)	0 (0-0)	5 (2-8)	0 (0-0)	1 (0-1)	0 (0-0)	2 (1-3)	0 (0-0)	0 (0-0)	0 (0-0)
2000	Bahamas	21 (19-35)		77 (70-125)		4 (2-8)		14 (9-29)		0 (0-1)		2 (1-3)		0 (0-0)		1 (0-1)	
2001	Bahamas									0 (0-1)		2 (1-3)		0 (0-0)		1 (0-1)	
2002	Bahamas									1 (0-1)		2 (1-3)		0 (0-0)		1 (0-1)	
2003	Bahamas									0 (0-0)		1 (0-2)		0 (0-0)		0 (0-1)	
2004	Bahamas									0 (0-0)		0 (0-0)		0 (0-0)		0 (0-0)	
2005	Bahamas									0 (0-0)		0 (0-0)		0 (0-0)		0 (0-0)	
2006	Bahamas									0 (0-0)		0 (0-0)		0 (0-0)		0 (0-0)	
2007	Bahamas									0 (0-0)		0 (0-0)		0 (0-0)		0 (0-0)	
2008	Bahamas									0 (0-0)		0 (0-0)		0 (0-0)		0 (0-0)	
2009	Bahamas									0 (0-0)		0 (0-0)		0 (0-0)		0 (0-0)	
2010	Bahamas									0 (0-0)		0 (0-0)		0 (0-0)		0 (0-0)	
2011	Bahamas									0 (0-0)		0 (0-0)		0 (0-0)		0 (0-0)	
2012	Bahamas									0 (0-0)		0 (0-0)		0 (0-0)		0 (0-0)	
2013	Bahamas									0 (0-0)		0 (0-0)		0 (0-0)		0 (0-0)	
2014	Bahamas									0 (0-0)		0 (0-0)		0 (0-0)		0 (0-0)	
2015	Bahamas	2 (2-3)	0 (0-0)	6 (5-10)	0 (0-0)	0 (0-1)	0 (0-0)	1 (1-2)	0 (0-0)	0 (0-0)	0 (0-0)	0 (0-0)	0 (0-0)	0 (0-0)	0 (0-0)	0 (0-0)	0 (0-0)
2000	Bahrain	73 (67-120)		103 (94-168)		14 (8-28)		19 (12-39)		0 (0-1)		1 (0-1)		0 (0-0)		0 (0-0)	
2001	Bahrain									0 (0-0)		0 (0-1)		0 (0-0)		0 (0-0)	
2002	Bahrain									1 (0-1)		1 (1-2)		0 (0-0)		0 (0-1)	
2003	Bahrain									0 (0-0)		0 (0-0)		0 (0-0)		0 (0-0)	
2004	Bahrain									0 (0-0)		0 (0-0)		0 (0-0)		0 (0-0)	
2005	Bahrain									0 (0-0)		0 (0-0)		0 (0-0)		0 (0-0)	
2006	Bahrain									0 (0-1)		0 (0-1)		0 (0-0)		0 (0-0)	
2007	Bahrain									0 (0-1)		0 (0-1)		0 (0-0)		0 (0-0)	
2008	Bahrain									0 (0-0)		0 (0-0)		0 (0-0)		0 (0-0)	
2009	Bahrain									0 (0-0)		0 (0-0)		0 (0-0)		0 (0-0)	
2010	Bahrain									0 (0-0)		0 (0-0)		0 (0-0)		0 (0-0)	
2011	Bahrain									0 (0-0)		0 (0-1)		0 (0-0)		0 (0-0)	
2012	Bahrain									0 (0-0)		0 (0-0)		0 (0-0)		0 (0-0)	
2013	Bahrain									0 (0-0)		0 (0-0)		0 (0-0)		0 (0-0)	
2014	Bahrain									0 (0-0)		0 (0-0)		0 (0-0)		0 (0-0)	
2015	Bahrain	7 (7-12)	0 (0-0)	7 (6-11)	0 (0-0)	1 (1-3)	0 (0-0)	1 (1-3)	0 (0-0)	0 (0-0)	0 (0-0)	0 (0-0)	0 (0-0)	0 (0-0)	0 (0-0)	0 (0-0)	0 (0-0)
2000	Bangladesh	294992 (269334-481988)		1788 (1632-2921)		55528 (33247-112077)		337 (202-679)		4390 (1271-7104)		27 (8-43)		756 (219-1223)		5 (1-7)	
2001	Bangladesh									4085 (1168-6623)		25 (7-40)		703 (201-1141)		4 (1-7)	
2002	Bangladesh									3637 (1028-5909)		22 (6-36)		626 (177-1017)		4 (1-6)	
2003	Bangladesh									3361 (832-5635)		20 (5-34)		1133 (281-1900)		7 (2-11)	
2004	Bangladesh									3123 (764-5247)		19 (5-32)		1053 (258-1769)		6 (2-11)	
2005	Bangladesh									2999 (761-5014)		18 (5-31)		1011 (256-1690)		6 (2-10)	
2006	Bangladesh									2801 (737-4656)		17 (5-29)		944 (248-1570)		6 (2-10)	
2007	Bangladesh									2602 (723-4287)		16 (4-27)		877 (244-1445)		5 (2-9)	
2008	Bangladesh									2371 (657-3912)		15 (4-25)		799 (221-1319)		5 (1-8)	
2009	Bangladesh									325 (90-536)		2 (1-3)		109 (30-181)		1 (0-1)	
2010	Bangladesh									144 (40-237)		1 (0-2)		48 (13-80)		0 (0-1)	
2011	Bangladesh									152 (42-251)		1 (0-2)		51 (14-85)		0 (0-1)	
2012	Bangladesh									121 (33-201)		1 (0-1)		41 (11-68)		0 (0-0)	
2013	Bangladesh									113 (31-186)		1 (0-1)		38 (10-63)		0 (0-0)	
2014	Bangladesh									14 (4-24)		0 (0-0)		5 (1-8)		0 (0-0)	
2015	Bangladesh	1981 (1809-3237)	0 (0-0)	13 (12-21)	0 (0-0)	373 (223-752)	0 (0-0)	2 (1-5)	0 (0-0)	14 (4-22)	0 (0-0)	0 (0-0)	0 (0-0)	5 (1-8)	0 (0-0)	0 (0-0)	0 (0-0)
2000	Barbados	171 (156-280)		932 (851-1523)		32 (19-65)		174 (104-351)		11 (5-19)		59 (26-104)		4 (2-6)		20 (9-35)	
2001	Barbados									7 (3-12)		38 (17-67)		2 (1-4)		13 (6-23)	
2002	Barbados									1 (0-1)		4 (2-7)		0 (0-0)		1 (1-2)	
2003	Barbados									0 (0-0)		0 (0-0)		0 (0-0)		0 (0-0)	
2004	Barbados									0 (0-0)		0 (0-0)		0 (0-0)		0 (0-0)	
2005	Barbados									0 (0-0)		0 (0-0)		0 (0-0)		0 (0-0)	
2006	Barbados									0 (0-0)		0 (0-0)		0 (0-0)		0 (0-0)	
2007	Barbados									0 (0-0)		0 (0-0)		0 (0-0)		0 (0-0)	
2008	Barbados									0 (0-0)		0 (0-0)		0 (0-0)		0 (0-0)	
2009	Barbados									0 (0-0)		0 (0-0)		0 (0-0)		0 (0-0)	
2010	Barbados									0 (0-0)		0 (0-0)		0 (0-0)		0 (0-0)	
2011	Barbados									0 (0-0)		0 (0-0)		0 (0-0)		0 (0-0)	
2012	Barbados									0 (0-0)		0 (0-0)		0 (0-0)		0 (0-0)	
2013	Barbados									0 (0-0)		0 (0-0)		0 (0-0)		0 (0-0)	
2014	Barbados									0 (0-0)		0 (0-0)		0 (0-0)		0 (0-0)	
2015	Barbados	1 (1-2)	0 (0-0)	6 (5-10)	0 (0-0)	0 (0-0)	0 (0-0)	1 (1-2)	0 (0-0)	0 (0-0)	0 (0-0)	0 (0-0)	0 (0-0)	0 (0-0)	0 (0-0)	0 (0-0)	0 (0-0)
2000	Belarus	1349 (1232-2204)		298 (272-486)		419 (251-846)		92 (55-187)		42 (9-83)		9 (2-18)		14 (3-28)		3 (1-6)	
2001	Belarus									37 (9-74)		8 (2-17)		13 (3-25)		3 (1-6)	
2002	Belarus									34 (8-67)		8 (2-15)		11 (3-22)		3 (1-5)	
2003	Belarus									31 (8-61)		7 (2-14)		11 (3-21)		2 (1-5)	
2004	Belarus									30 (8-60)		7 (2-14)		10 (3-20)		2 (1-5)	
2005	Belarus									29 (7-57)		7 (2-13)		10 (2-19)		2 (1-4)	
2006	Belarus									28 (7-55)		6 (2-12)		10 (2-19)		2 (1-4)	
2007	Belarus									27 (7-51)		6 (2-11)		9 (2-17)		2 (1-4)	
2008	Belarus									27 (7-52)		6 (2-11)		9 (2-17)		2 (1-4)	
2009	Belarus									21 (6-39)		4 (1-8)		7 (2-13)		1 (0-3)	
2010	Belarus									22 (6-41)		4 (1-8)		7 (2-14)		1 (0-3)	
2011	Belarus									21 (6-40)		4 (1-8)		7 (2-13)		1 (0-3)	
2012	Belarus									15 (5-28)		3 (1-5)		5 (2-10)		1 (0-2)	
2013	Belarus									14 (4-26)		3 (1-5)		5 (1-9)		1 (0-2)	
2014	Belarus									14 (4-26)		2 (1-5)		5 (2-9)		1 (0-2)	
2015	Belarus	1119 (1022-1829)	0 (0-0)	194 (177-317)	0 (0-0)	348 (208-702)	0 (0-0)	60 (36-122)	0 (0-0)	13 (4-24)	0 (0-0)	2 (1-4)	0 (0-0)	4 (1-8)	0 (0-0)	1 (0-1)	0 (0-0)
2000	Belgium	4 (4-7)		1 (1-1)		1 (1-3)		0 (0-0)		2 (1-3)		0 (0-1)		1 (0-1)		0 (0-0)	
2001	Belgium									2 (1-4)		0 (0-1)		1 (0-1)		0 (0-0)	
2002	Belgium									1 (1-3)		0 (0-0)		0 (0-1)		0 (0-0)	
2003	Belgium									1 (1-3)		0 (0-0)		0 (0-1)		0 (0-0)	
2004	Belgium									2 (1-3)		0 (0-1)		1 (0-1)		0 (0-0)	
2005	Belgium									1 (1-3)		0 (0-0)		1 (0-1)		0 (0-0)	
2006	Belgium									2 (1-3)		0 (0-1)		1 (0-1)		0 (0-0)	
2007	Belgium									1 (1-3)		0 (0-0)		0 (0-1)		0 (0-0)	
2008	Belgium									1 (0-2)		0 (0-0)		0 (0-1)		0 (0-0)	
2009	Belgium									1 (0-2)		0 (0-0)		0 (0-1)		0 (0-0)	
2010	Belgium									0 (0-1)		0 (0-0)		0 (0-0)		0 (0-0)	
2011	Belgium									1 (0-1)		0 (0-0)		0 (0-0)		0 (0-0)	
2012	Belgium									1 (0-2)		0 (0-0)		0 (0-1)		0 (0-0)	
2013	Belgium									1 (0-1)		0 (0-0)		0 (0-0)		0 (0-0)	
2014	Belgium									1 (0-1)		0 (0-0)		0 (0-0)		0 (0-0)	
2015	Belgium	5 (4-8)	0 (0-0)	1 (1-1)	0 (0-0)	1 (1-3)	0 (0-0)	0 (0-0)	0 (0-0)	1 (0-1)	0 (0-0)	0 (0-0)	0 (0-0)	0 (0-0)	0 (0-0)	0 (0-0)	0 (0-0)
2000	Belize	444 (405-725)		1213 (1107-1982)		83 (50-168)		227 (136-459)		6 (1-10)		16 (2-28)		2 (0-3)		5 (1-10)	
2001	Belize									2 (0-3)		4 (0-7)		1 (0-1)		1 (0-2)	
2002	Belize									1 (0-2)		3 (0-6)		0 (0-1)		1 (0-2)	
2003	Belize									0 (0-0)		1 (0-1)		0 (0-0)		0 (0-0)	
2004	Belize									1 (0-1)		2 (0-3)		0 (0-0)		1 (0-1)	
2005	Belize									0 (0-0)		0 (0-0)		0 (0-0)		0 (0-0)	
2006	Belize									0 (0-0)		0 (0-0)		0 (0-0)		0 (0-0)	
2007	Belize									0 (0-0)		0 (0-0)		0 (0-0)		0 (0-0)	
2008	Belize									0 (0-0)		0 (0-0)		0 (0-0)		0 (0-0)	
2009	Belize									0 (0-0)		0 (0-0)		0 (0-0)		0 (0-0)	
2010	Belize									0 (0-0)		0 (0-0)		0 (0-0)		0 (0-0)	
2011	Belize									0 (0-0)		0 (0-0)		0 (0-0)		0 (0-0)	
2012	Belize									0 (0-0)		0 (0-0)		0 (0-0)		0 (0-0)	
2013	Belize									0 (0-0)		0 (0-1)		0 (0-0)		0 (0-0)	
2014	Belize									0 (0-0)		0 (0-0)		0 (0-0)		0 (0-0)	
2015	Belize	3 (3-5)	0 (0-0)	8 (7-13)	0 (0-0)	1 (0-1)	0 (0-0)	1 (1-3)	0 (0-0)	0 (0-0)	0 (0-0)	0 (0-0)	0 (0-0)	0 (0-0)	0 (0-0)	0 (0-0)	0 (0-0)
2000	Benin	31569 (28823-51581)		2578 (2354-4212)		5892 (3528-11892)		481 (288-971)		630 (193-1031)		51 (16-84)		109 (33-178)		9 (3-14)	
2001	Benin									616 (189-1007)		49 (15-80)		106 (33-173)		8 (3-14)	
2002	Benin									611 (188-998)		47 (15-77)		105 (32-172)		8 (3-13)	
2003	Benin									604 (186-986)		46 (14-74)		104 (32-170)		8 (2-13)	
2004	Benin									594 (183-969)		44 (13-71)		102 (32-167)		8 (2-12)	
2005	Benin									417 (129-681)		30 (9-49)		72 (22-117)		5 (2-8)	
2006	Benin									65 (20-106)		5 (1-7)		11 (3-18)		1 (0-1)	
2007	Benin									57 (17-93)		4 (1-6)		10 (3-16)		1 (0-1)	
2008	Benin									65 (20-106)		4 (1-7)		11 (3-18)		1 (0-1)	
2009	Benin									62 (19-101)		4 (1-7)		11 (3-17)		1 (0-1)	
2010	Benin									6 (2-10)		0 (0-1)		1 (0-2)		0 (0-0)	
2011	Benin									6 (2-10)		0 (0-1)		1 (0-2)		0 (0-0)	
2012	Benin									6 (2-10)		0 (0-1)		1 (0-2)		0 (0-0)	
2013	Benin									6 (2-10)		0 (0-1)		1 (0-2)		0 (0-0)	
2014	Benin									6 (2-10)		0 (0-1)		1 (0-2)		0 (0-0)	
2015	Benin	244 (223-399)	2 (2-4)	15 (13-24)	0 (0-0)	46 (27-93)	0 (0-1)	3 (2-6)	0 (0-0)	6 (2-10)	0 (0-0)	0 (0-1)	0 (0-0)	1 (0-2)	0 (0-0)	0 (0-0)	0 (0-0)
2000	Bhutan	791 (722-1293)		1069 (976-1747)		148 (88-298)		199 (119-403)		29 (11-44)		39 (15-60)		5 (2-8)		7 (3-10)	
2001	Bhutan									26 (11-40)		36 (14-55)		5 (2-7)		6 (2-9)	
2002	Bhutan									24 (9-38)		33 (12-53)		8 (3-13)		11 (4-18)	
2003	Bhutan									22 (8-35)		31 (12-48)		7 (3-12)		10 (4-16)	
2004	Bhutan									20 (8-32)		29 (11-45)		7 (3-11)		10 (4-15)	
2005	Bhutan									19 (7-29)		26 (10-41)		6 (2-10)		9 (3-14)	
2006	Bhutan									17 (7-27)		24 (9-38)		6 (2-9)		8 (3-13)	
2007	Bhutan									16 (6-25)		22 (9-35)		5 (2-8)		8 (3-12)	
2008	Bhutan									15 (6-23)		21 (8-32)		5 (2-8)		7 (3-11)	
2009	Bhutan									14 (5-21)		19 (8-29)		5 (2-7)		6 (3-10)	
2010	Bhutan									12 (5-19)		17 (7-27)		4 (2-6)		6 (2-9)	
2011	Bhutan									11 (5-17)		16 (6-24)		4 (2-6)		5 (2-8)	
2012	Bhutan									2 (1-2)		2 (1-3)		1 (0-1)		1 (0-1)	
2013	Bhutan									1 (0-1)		1 (0-1)		0 (0-0)		0 (0-0)	
2014	Bhutan									0 (0-1)		1 (0-1)		0 (0-0)		0 (0-0)	
2015	Bhutan	32 (29-52)	0 (0-0)	50 (45-81)	0 (0-0)	6 (4-12)	0 (0-0)	9 (6-19)	0 (0-0)	0 (0-1)	0 (0-0)	1 (0-1)	0 (0-0)	0 (0-0)	0 (0-0)	0 (0-0)	0 (0-0)
2000	Bolivia (Plurinational State of)	3330 (3041-5441)		292 (266-477)		623 (373-1257)		55 (33-110)		73 (23-118)		6 (2-10)		13 (4-20)		1 (0-2)	
2001	Bolivia (Plurinational State of)									35 (11-57)		3 (1-5)		6 (2-10)		1 (0-1)	
2002	Bolivia (Plurinational State of)									33 (9-55)		3 (1-5)		11 (3-18)		1 (0-2)	
2003	Bolivia (Plurinational State of)									29 (8-48)		2 (1-4)		10 (3-16)		1 (0-1)	
2004	Bolivia (Plurinational State of)									24 (7-40)		2 (1-3)		8 (2-14)		1 (0-1)	
2005	Bolivia (Plurinational State of)									2 (1-4)		0 (0-0)		1 (0-1)		0 (0-0)	
2006	Bolivia (Plurinational State of)									2 (1-4)		0 (0-0)		1 (0-1)		0 (0-0)	
2007	Bolivia (Plurinational State of)									2 (1-4)		0 (0-0)		1 (0-1)		0 (0-0)	
2008	Bolivia (Plurinational State of)									2 (1-3)		0 (0-0)		1 (0-1)		0 (0-0)	
2009	Bolivia (Plurinational State of)									2 (1-3)		0 (0-0)		1 (0-1)		0 (0-0)	
2010	Bolivia (Plurinational State of)									2 (1-3)		0 (0-0)		1 (0-1)		0 (0-0)	
2011	Bolivia (Plurinational State of)									2 (1-3)		0 (0-0)		1 (0-1)		0 (0-0)	
2012	Bolivia (Plurinational State of)									2 (0-3)		0 (0-0)		1 (0-1)		0 (0-0)	
2013	Bolivia (Plurinational State of)									1 (0-2)		0 (0-0)		1 (0-1)		0 (0-0)	
2014	Bolivia (Plurinational State of)									1 (0-2)		0 (0-0)		0 (0-1)		0 (0-0)	
2015	Bolivia (Plurinational State of)	68 (62-111)	0 (0-0)	6 (5-10)	0 (0-0)	13 (8-26)	0 (0-0)	1 (1-2)	0 (0-0)	1 (0-2)	0 (0-0)	0 (0-0)	0 (0-0)	0 (0-1)	0 (0-0)	0 (0-0)	0 (0-0)
2000	Bosnia and Herzegovina	319 (291-521)		149 (136-243)		99 (59-200)		46 (28-93)		4 (1-9)		2 (0-4)		1 (0-3)		1 (0-1)	

Hib = *Haemophilus influenzae* type b; npnm = non-pneumonia, non-meningitis; HIV = cases only in HIV-infected children; Uncertainty range provided in parentheses.

Year	Country	Hib pneumonia cases (UR)	Hib pneumonia cases, HIV (UR)	Hib pneumonia incidence rate (UR)	Hib pneumonia incidence rate, HIV (UR)	Hib severe pneumonia cases (UR)	Hib severe pneumonia cases, HIV (UR)	Hib severe pneumonia incidence rate (UR)	Hib severe pneumonia incidence rate, HIV (UR)	Hib meningitis cases (UR)	Hib meningitis cases, HIV (UR)	Hib meningitis incidence rate (UR)	Hib meningitis incidence rate, HIV (UR)	Hib NPNM cases (UR)	Hib NPNM cases, HIV (UR)	Hib NPNM incidence rate (UR)	Hib NPNM incidence rate, HIV (UR)
2001	Bosnia and Herzegovina									4 (1-8)		2 (0-4)		1 (0-3)		1 (0-1)	
2002	Bosnia and Herzegovina									4 (1-8)		2 (0-4)		1 (0-3)		1 (0-1)	
2003	Bosnia and Herzegovina									4 (1-7)		2 (0-4)		1 (0-2)		1 (0-1)	
2004	Bosnia and Herzegovina									3 (1-6)		2 (0-3)		1 (0-2)		1 (0-1)	
2005	Bosnia and Herzegovina									1 (0-2)		1 (0-1)		0 (0-1)		0 (0-0)	
2006	Bosnia and Herzegovina									1 (0-1)		0 (0-1)		0 (0-0)		0 (0-0)	
2007	Bosnia and Herzegovina									1 (0-1)		0 (0-1)		0 (0-0)		0 (0-0)	
2008	Bosnia and Herzegovina									1 (0-1)		0 (0-1)		0 (0-0)		0 (0-0)	
2009	Bosnia and Herzegovina									0 (0-0)		0 (0-0)		0 (0-0)		0 (0-0)	
2010	Bosnia and Herzegovina									0 (0-0)		0 (0-0)		0 (0-0)		0 (0-0)	
2011	Bosnia and Herzegovina									0 (0-0)		0 (0-0)		0 (0-0)		0 (0-0)	
2012	Bosnia and Herzegovina									0 (0-0)		0 (0-0)		0 (0-0)		0 (0-0)	
2013	Bosnia and Herzegovina									0 (0-0)		0 (0-0)		0 (0-0)		0 (0-0)	
2014	Bosnia and Herzegovina									0 (0-0)		0 (0-0)		0 (0-0)		0 (0-0)	
2015	Bosnia and Herzegovina	3 (2-4)	0 (0-0)	1 (1-2)	0 (0-0)	1 (0-2)	0 (0-0)	0 (0-1)	0 (0-0)	0 (0-0)	0 (0-0)	0 (0-0)	0 (0-0)	0 (0-0)	0 (0-0)	0 (0-0)	0 (0-0)
2000	Botswana	4829 (4409-7891)		2207 (2015-3605)		910 (545-1836)		416 (249-839)		12 (3-20)		5 (1-9)		2 (1-3)		1 (0-2)	
2001	Botswana									12 (3-20)		6 (1-9)		2 (1-3)		1 (0-2)	
2002	Botswana									12 (3-21)		6 (2-10)		2 (1-4)		1 (0-2)	
2003	Botswana									13 (3-22)		6 (2-10)		2 (1-4)		1 (0-2)	
2004	Botswana									14 (4-23)		6 (2-10)		2 (1-4)		1 (0-2)	
2005	Botswana									14 (3-24)		6 (1-11)		5 (1-8)		2 (1-4)	
2006	Botswana									15 (3-25)		7 (2-11)		5 (1-8)		2 (1-4)	
2007	Botswana									16 (4-27)		7 (2-12)		5 (1-9)		2 (1-4)	
2008	Botswana									16 (4-28)		7 (2-12)		5 (1-9)		2 (1-4)	
2009	Botswana									16 (4-28)		7 (2-12)		5 (1-9)		2 (1-4)	
2010	Botswana									16 (4-27)		7 (2-12)		5 (1-9)		2 (1-4)	
2011	Botswana									3 (1-4)		1 (0-2)		1 (0-1)		0 (0-1)	
2012	Botswana									1 (0-2)		0 (0-1)		0 (0-1)		0 (0-0)	
2013	Botswana									1 (0-2)		0 (0-1)		0 (0-1)		0 (0-0)	
2014	Botswana									1 (0-2)		0 (0-1)		0 (0-1)		0 (0-0)	
2015	Botswana	226 (206-369)	9 (9-16)	86 (79-141)	4 (3-1)	42 (25-85)	2 (1-4)	16 (10-33)	1 (0-0)	1 (0-2)	0 (0-0)	0 (0-1)	0 (0-0)	0 (0-1)	0 (0-0)	0 (0-0)	0 (0-0)
2000	Brazil	32666 (29825-53373)		188 (172-308)		6096 (3650-12304)		35 (21-71)		579 (148-983)		3 (1-6)		195 (50-332)		1 (0-2)	
2001	Brazil									261 (23-463)		1 (0-3)		88 (8-156)		0 (0-1)	
2002	Brazil									208 (19-369)		1 (0-2)		70 (6-124)		0 (0-1)	
2003	Brazil									172 (16-306)		1 (0-2)		58 (5-103)		0 (0-1)	
2004	Brazil									161 (15-287)		1 (0-2)		54 (5-97)		0 (0-1)	
2005	Brazil									21 (2-38)		0 (0-0)		7 (1-13)		0 (0-0)	
2006	Brazil									20 (2-36)		0 (0-0)		7 (1-12)		0 (0-0)	
2007	Brazil									17 (2-29)		0 (0-0)		6 (1-10)		0 (0-0)	
2008	Brazil									14 (1-25)		0 (0-0)		5 (0-9)		0 (0-0)	
2009	Brazil									12 (1-21)		0 (0-0)		4 (0-7)		0 (0-0)	
2010	Brazil									10 (1-18)		0 (0-0)		3 (0-6)		0 (0-0)	
2011	Brazil									7 (1-13)		0 (0-0)		2 (0-4)		0 (0-0)	
2012	Brazil									7 (1-12)		0 (0-0)		2 (0-4)		0 (0-0)	
2013	Brazil									7 (1-12)		0 (0-0)		2 (0-4)		0 (0-0)	
2014	Brazil									10 (1-17)		0 (0-0)		3 (0-6)		0 (0-0)	
2015	Brazil	1168 (1066-1908)	0 (0-1)	8 (7-13)	0 (0-0)	218 (131-440)	0 (0-0)	1 (1-3)	0 (0-0)	11 (1-20)	0 (0-0)	0 (0-0)	0 (0-0)	4 (0-7)	0 (0-0)	0 (0-0)	0 (0-0)
2000	Brunei Darussalam	50 (46-82)		149 (136-243)		16 (9-32)		46 (28-93)		0 (0-0)		0 (0-0)		0 (0-0)		0 (0-0)	
2001	Brunei Darussalam									0 (0-0)		0 (0-0)		0 (0-0)		0 (0-0)	
2002	Brunei Darussalam									0 (0-0)		0 (0-0)		0 (0-0)		0 (0-0)	
2003	Brunei Darussalam									0 (0-0)		0 (0-0)		0 (0-0)		0 (0-0)	
2004	Brunei Darussalam									0 (0-0)		0 (0-0)		0 (0-0)		0 (0-0)	
2005	Brunei Darussalam									0 (0-0)		0 (0-0)		0 (0-0)		0 (0-0)	
2006	Brunei Darussalam									0 (0-0)		0 (0-0)		0 (0-0)		0 (0-0)	
2007	Brunei Darussalam									0 (0-0)		0 (0-0)		0 (0-0)		0 (0-0)	
2008	Brunei Darussalam									0 (0-0)		0 (0-0)		0 (0-0)		0 (0-0)	
2009	Brunei Darussalam									0 (0-0)		0 (0-0)		0 (0-0)		0 (0-0)	
2010	Brunei Darussalam									0 (0-0)		0 (0-0)		0 (0-0)		0 (0-0)	
2011	Brunei Darussalam									0 (0-0)		0 (0-0)		0 (0-0)		0 (0-0)	
2012	Brunei Darussalam									0 (0-0)		0 (0-0)		0 (0-0)		0 (0-0)	
2013	Brunei Darussalam									0 (0-0)		0 (0-0)		0 (0-0)		0 (0-0)	
2014	Brunei Darussalam									0 (0-0)		0 (0-0)		0 (0-0)		0 (0-0)	
2015	Brunei Darussalam	0 (0-1)	0 (0-0)	1 (1-2)	0 (0-0)	0 (0-0)	0 (0-0)	0 (0-1)	0 (0-0)	0 (0-0)	0 (0-0)	0 (0-0)	0 (0-0)	0 (0-0)	0 (0-0)	0 (0-0)	0 (0-0)
2000	Bulgaria	483 (441-789)		149 (136-243)		150 (90-303)		46 (28-93)		100 (44-185)		31 (14-57)		34 (15-62)		10 (5-19)	
2001	Bulgaria									83 (37-154)		26 (12-48)		28 (12-52)		9 (4-16)	
2002	Bulgaria									45 (20-83)		14 (6-26)		15 (7-28)		5 (2-9)	
2003	Bulgaria									119 (53-221)		38 (17-71)		40 (18-74)		13 (6-24)	
2004	Bulgaria									101 (45-187)		33 (14-60)		34 (15-63)		11 (5-20)	
2005	Bulgaria									87 (39-161)		28 (12-52)		29 (13-54)		9 (4-17)	
2006	Bulgaria									115 (51-212)		36 (16-67)		39 (17-72)		12 (5-22)	
2007	Bulgaria									94 (41-173)		29 (13-53)		32 (14-58)		10 (4-18)	
2008	Bulgaria									126 (56-233)		37 (16-69)		42 (19-79)		13 (6-23)	
2009	Bulgaria									86 (38-160)		25 (11-46)		29 (13-54)		8 (4-16)	
2010	Bulgaria									19 (8-34)		5 (2-10)		6 (3-12)		2 (1-3)	
2011	Bulgaria									7 (3-13)		2 (1-4)		2 (1-4)		1 (0-1)	
2012	Bulgaria									8 (3-14)		2 (1-4)		3 (1-5)		1 (0-1)	
2013	Bulgaria									7 (3-13)		2 (1-4)		2 (1-4)		1 (0-1)	
2014	Bulgaria									8 (4-15)		2 (1-5)		3 (1-5)		1 (0-2)	
2015	Bulgaria	5 (5-8)	0 (0-0)	1 (1-2)	0 (0-0)	2 (1-3)	0 (0-0)	0 (0-1)	0 (0-0)	1 (0-2)	0 (0-0)	0 (0-0)	0 (0-0)	0 (0-1)	0 (0-0)	0 (0-0)	0 (0-0)
2000	Burkina Faso	37109 (33881-60632)		1737 (1586-2839)		6953 (4163-14033)		325 (195-657)		760 (350-1133)		36 (16-53)		131 (60-195)		6 (3-9)	
2001	Burkina Faso									808 (373-1202)		37 (17-55)		139 (64-207)		6 (3-9)	
2002	Burkina Faso									861 (400-1280)		38 (18-57)		148 (69-220)		7 (3-10)	
2003	Burkina Faso									924 (431-1370)		40 (19-59)		159 (74-236)		7 (3-10)	
2004	Burkina Faso									989 (463-1465)		42 (19-62)		170 (80-252)		7 (3-11)	
2005	Burkina Faso									1036 (488-1532)		42 (20-63)		178 (84-264)		7 (3-11)	
2006	Burkina Faso									226 (72-368)		9 (3-15)		39 (12-63)		2 (0-3)	
2007	Burkina Faso									85 (28-137)		3 (1-5)		15 (5-24)		1 (0-1)	
2008	Burkina Faso									75 (25-120)		3 (1-4)		13 (4-21)		0 (0-1)	
2009	Burkina Faso									77 (27-122)		3 (1-4)		13 (5-21)		0 (0-1)	
2010	Burkina Faso									82 (30-130)		3 (1-5)		14 (5-22)		1 (0-1)	
2011	Burkina Faso									12 (4-18)		0 (0-1)		2 (1-3)		0 (0-0)	
2012	Burkina Faso									13 (5-20)		0 (0-1)		2 (1-4)		0 (0-0)	
2013	Burkina Faso									15 (6-23)		0 (0-1)		3 (1-4)		0 (0-0)	
2014	Burkina Faso									16 (7-24)		1 (0-1)		3 (1-4)		0 (0-0)	
2015	Burkina Faso	403 (368-659)	3 (3-5)	13 (12-21)	0 (0-0)	76 (45-153)	1 (0-1)	2 (1-5)	0 (0-0)	17 (8-25)	0 (0-0)	1 (0-1)	0 (0-0)	3 (1-4)	0 (0-0)	0 (0-0)	0 (0-0)
2000	Burundi	22169 (20241-36222)		1849 (1688-3022)		4175 (2500-8426)		348 (209-703)		819 (389-1208)		68 (32-101)		141 (67-208)		12 (6-17)	
2001	Burundi									832 (265-1351)		69 (22-111)		143 (46-233)		12 (4-19)	
2002	Burundi									829 (263-1348)		67 (21-109)		143 (45-232)		12 (4-19)	
2003	Burundi									891 (282-1449)		70 (22-114)		153 (49-249)		12 (4-20)	
2004	Burundi									154 (49-251)		12 (4-19)		27 (8-43)		2 (1-3)	
2005	Burundi									82 (26-134)		6 (2-10)		14 (4-23)		1 (0-2)	
2006	Burundi									80 (26-130)		6 (2-9)		14 (4-22)		1 (0-2)	
2007	Burundi									63 (21-102)		4 (1-7)		11 (4-18)		1 (0-1)	
2008	Burundi									86 (29-138)		6 (2-9)		15 (5-24)		1 (0-2)	
2009	Burundi									12 (4-18)		1 (0-1)		2 (1-3)		0 (0-0)	
2010	Burundi									12 (4-19)		1 (0-1)		2 (1-3)		0 (0-0)	
2011	Burundi									13 (5-20)		1 (0-1)		2 (1-3)		0 (0-0)	
2012	Burundi									13 (5-21)		1 (0-1)		2 (1-4)		0 (0-0)	
2013	Burundi									14 (5-21)		1 (0-1)		2 (1-4)		0 (0-0)	
2014	Burundi									14 (6-22)		1 (0-1)		2 (1-4)		0 (0-0)	
2015	Burundi	222 (203-363)	2 (2-3)	11 (10-18)	0 (0-0)	42 (25-84)	0 (0-1)	2 (1-4)	0 (0-0)	14 (6-22)	0 (0-0)	1 (0-1)	0 (0-0)	2 (1-4)	0 (0-0)	0 (0-0)	0 (0-0)
2000	Cambodia	33766 (30829-55171)		2172 (1983-3549)		6232 (3731-12578)		401 (240-809)		806 (252-1294)		52 (16-83)		139 (43-223)		9 (3-14)	
2001	Cambodia									716 (227-1145)		47 (15-75)		123 (39-197)		8 (3-13)	
2002	Cambodia									634 (204-1011)		42 (14-67)		109 (35-174)		7 (2-12)	
2003	Cambodia									568 (186-903)		38 (12-60)		98 (32-155)		7 (2-10)	
2004	Cambodia									529 (159-864)		35 (11-57)		178 (53-291)		12 (4-19)	
2005	Cambodia									500 (153-814)		33 (10-54)		169 (52-275)		11 (3-18)	
2006	Cambodia									481 (152-779)		31 (10-50)		162 (51-263)		11 (3-17)	
2007	Cambodia									465 (151-749)		30 (10-48)		157 (51-252)		10 (3-16)	
2008	Cambodia									449 (151-720)		28 (9-45)		152 (51-243)		9 (3-15)	
2009	Cambodia									427 (148-679)		26 (9-42)		144 (50-229)		9 (3-14)	
2010	Cambodia									64 (23-101)		4 (1-6)		22 (8-34)		1 (0-2)	
2011	Cambodia									32 (12-50)		2 (1-3)		11 (4-17)		1 (0-1)	
2012	Cambodia									28 (10-44)		2 (1-3)		9 (3-15)		1 (0-1)	
2013	Cambodia									27 (10-43)		2 (1-2)		9 (3-14)		1 (0-1)	
2014	Cambodia									23 (9-36)		1 (1-2)		8 (3-12)		0 (0-1)	
2015	Cambodia	211 (192-344)	1 (1-1)	12 (11-20)	0 (0-0)	40 (24-80)	0 (0-0)	2 (1-5)	0 (0-0)	3 (0-4)	0 (0-0)	0 (0-0)	0 (0-0)	1 (0-1)	0 (0-0)	0 (0-0)	0 (0-0)
2000	Cameroon	32435 (29614-52995)		1196 (1092-1954)		6111 (3659-12334)		225 (135-455)		1154 (512-1736)		43 (19-64)		199 (88-299)		7 (3-11)	
2001	Cameroon									1147 (337-1891)		41 (12-68)		197 (58-326)		7 (2-12)	
2002	Cameroon									1167 (352-1913)		41 (12-67)		201 (61-329)		7 (2-12)	
2003	Cameroon									1157 (358-1886)		40 (12-65)		199 (62-325)		7 (2-11)	
2004	Cameroon									1149 (366-1862)		39 (12-62)		198 (63-321)		7 (2-11)	
2005	Cameroon									1144 (358-1862)		37 (12-61)		197 (62-321)		6 (2-11)	
2006	Cameroon									1155 (355-1888)		37 (11-60)		199 (61-325)		6 (2-10)	
2007	Cameroon									1154 (352-1889)		36 (11-59)		199 (61-325)		6 (2-10)	
2008	Cameroon									1179 (356-1931)		36 (11-59)		203 (61-333)		6 (2-10)	
2009	Cameroon									204 (61-334)		6 (2-10)		35 (11-58)		1 (0-2)	
2010	Cameroon									111 (33-183)		3 (1-5)		19 (6-32)		1 (0-1)	
2011	Cameroon									115 (34-189)		3 (1-5)		20 (6-33)		1 (0-1)	
2012	Cameroon									107 (31-176)		3 (1-5)		18 (5-30)		1 (0-1)	
2013	Cameroon									96 (28-158)		3 (1-4)		16 (5-27)		0 (0-1)	
2014	Cameroon									11 (3-19)		0 (0-1)		2 (1-3)		0 (0-0)	
2015	Cameroon	418 (382-683)	15 (13-24)	11 (10-19)	0 (0-0)	78 (47-158)	3 (2-5)	2 (1-4)	0 (0-0)	11 (3-18)	0 (0-1)	0 (0-0)	0 (0-0)	2 (1-3)	0 (0-0)	0 (0-0)	0 (0-0)
2000	Canada	111 (101-181)		6 (6-10)		34 (21-70)		2 (1-4)		29 (12-49)		2 (1-3)		10 (4-17)		1 (0-1)	
2001	Canada									3 (1-5)		0 (0-0)		1 (0-2)		0 (0-0)	
2002	Canada									3 (1-4)		0 (0-0)		1 (0-1)		0 (0-0)	
2003	Canada									3 (1-4)		0 (0-0)		1 (0-1)		0 (0-0)	
2004	Canada									2 (1-4)		0 (0-0)		1 (0-1)		0 (0-0)	
2005	Canada									2 (1-4)		0 (0-0)		1 (0-1)		0 (0-0)	
2006	Canada									3 (1-5)		0 (0-0)		1 (0-2)		0 (0-0)	
2007	Canada									3 (1-5)		0 (0-0)		1 (0-2)		0 (0-0)	
2008	Canada									1 (0-2)		0 (0-0)		0 (0-1)		0 (0-0)	
2009	Canada									4 (1-6)		0 (0-0)		1 (0-2)		0 (0-0)	
2010	Canada									3 (1-6)		0 (0-0)		1 (0-2)		0 (0-0)	
2011	Canada									3 (1-5)		0 (0-0)		1 (0-2)		0 (0-0)	
2012	Canada									4 (1-6)		0 (0-0)		1 (0-2)		0 (0-0)	
2013	Canada									4 (1-6)		0 (0-0)		1 (0-2)		0 (0-0)	
2014	Canada									3 (1-6)		0 (0-0)		1 (0-2)		0 (0-0)	
2015	Canada	14 (13-23)	0 (0-0)	1 (1-1)	0 (0-0)	4 (3-9)	0 (0-0)	0 (0-0)	0 (0-0)	3 (1-6)	0 (0-0)	0 (0-0)	0 (0-0)	1 (0-2)	0 (0-0)	0 (0-0)	0 (0-0)
2000	Cape Verde	980 (895-1602)		1595 (1456-2606)		185 (111-373)		301 (180-607)		14 (9-24)		24 (14-39)		5 (3-8)		8 (5-13)	
2001	Cape Verde									14 (9-24)		23 (14-39)		5 (3-8)		8 (5-13)	
2002	Cape Verde									15 (6-19)		25 (10-33)		5 (2-7)		8 (3-11)	
2003	Cape Verde									15 (7-22)		26 (12-38)		5 (2-7)		9 (4-13)	
2004	Cape Verde									17 (8-26)		30 (14-46)		6 (3-9)		10 (5-16)	
2005	Cape Verde									18 (9-29)		32 (17-53)		6 (3-10)		11 (6-18)	
2006	Cape Verde									18 (9-30)		34 (18-56)		6 (3-10)		11 (6-19)	
2007	Cape Verde									18 (9-30)		35 (18-58)		6 (3-10)		12 (6-20)	
2008	Cape Verde									18 (10-31)		36 (19-59)		6 (3-10)		12 (6-20)	
2009	Cape Verde									18 (9-30)		36 (18-59)		6 (3-10)		12 (6-20)	

Hib = *Haemophilus influenzae* type b; npnm = non-pneumonia, non-meningitis; HIV = cases only in HIV-infected children; Uncertainty range provided in parentheses.

Year	Country	Hib pneumonia cases (UR)	Hib pneumonia cases, HIV (UR)	Hib pneumonia incidence rate (UR)	Hib pneumonia incidence rate, HIV (UR)	Hib severe pneumonia cases (UR)	Hib severe pneumonia cases, HIV (UR)	Hib severe pneumonia incidence rate (UR)	Hib severe pneumonia incidence rate, HIV (UR)	Hib meningitis cases (UR)	Hib meningitis cases, HIV (UR)	Hib meningitis incidence rate (UR)	Hib meningitis incidence rate, HIV (UR)	Hib NPNM cases (UR)	Hib NPNM cases, HIV (UR)	Hib NPNM incidence rate (UR)	Hib NPNM incidence rate, HIV (UR)
2010	Cape Verde									18 (9-30)		35 (18-58)		6 (3-10)		12 (6-20)	
2011	Cape Verde									4 (2-7)		8 (4-14)		1 (1-2)		3 (1-5)	
2012	Cape Verde									2 (1-3)		4 (2-6)		1 (0-1)		1 (1-2)	
2013	Cape Verde									2 (1-3)		4 (2-6)		1 (0-1)		1 (1-2)	
2014	Cape Verde									2 (1-3)		3 (2-6)		1 (0-1)		1 (1-2)	
2015	Cape Verde	42 (38-69)	0 (0-0)	80 (73-131)	1 (1-0)	8 (5-16)	0 (0-0)	15 (9-30)	0 (0-0)	2 (1-3)	0 (0-0)	3 (2-5)	0 (0-0)	1 (0-1)	0 (0-0)	1 (1-2)	0 (0-0)
2000	Central African Republic	15399 (14059-25160)		2589 (2364-4231)		2880 (1724-5813)		484 (290-977)		292 (134-436)		49 (22-73)		50 (23-75)		8 (4-13)	
2001	Central African Republic									294 (135-439)		48 (22-72)		51 (23-76)		8 (4-12)	
2002	Central African Republic									298 (136-445)		48 (22-72)		51 (23-77)		8 (4-12)	
2003	Central African Republic									292 (134-436)		47 (21-70)		50 (23-75)		8 (4-12)	
2004	Central African Republic									284 (130-424)		45 (21-68)		49 (22-73)		8 (4-12)	
2005	Central African Republic									275 (126-410)		44 (20-65)		47 (22-71)		8 (3-11)	
2006	Central African Republic									283 (130-423)		45 (20-67)		49 (22-73)		8 (4-11)	
2007	Central African Republic									292 (133-436)		46 (21-69)		50 (23-75)		8 (4-12)	
2008	Central African Republic									299 (136-447)		47 (21-70)		52 (23-77)		8 (4-12)	
2009	Central African Republic									202 (92-302)		32 (14-47)		35 (16-52)		5 (2-8)	
2010	Central African Republic									54 (24-81)		8 (4-12)		9 (4-14)		1 (1-2)	
2011	Central African Republic									56 (16-92)		9 (3-14)		10 (3-16)		1 (0-2)	
2012	Central African Republic									57 (17-95)		9 (3-14)		10 (3-16)		1 (0-2)	
2013	Central African Republic									231 (68-381)		34 (10-57)		40 (12-66)		6 (2-10)	
2014	Central African Republic									64 (19-105)		9 (3-15)		11 (3-18)		2 (0-3)	
2015	Central African Republic	1162 (1060-1898)	52 (47-85)	167 (152-273)	7 (7-1)	218 (131-440)	10 (6-20)	31 (19-63)	1 (1-0)	64 (19-106)	3 (1-5)	9 (3-15)	0 (0-0)	11 (3-18)	0 (0-1)	2 (0-3)	0 (0-0)
2000	Chad	43515 (39730-71099)		2622 (2394-4284)		8052 (4821-16253)		485 (291-979)		1207 (527-1819)		73 (32-110)		208 (91-313)		13 (5-19)	
2001	Chad									1207 (522-1824)		70 (30-106)		208 (90-314)		12 (5-18)	
2002	Chad									1265 (541-1918)		71 (30-107)		218 (93-330)		12 (5-18)	
2003	Chad									1229 (520-1869)		66 (28-101)		212 (90-322)		11 (5-17)	
2004	Chad									1231 (516-1878)		64 (27-98)		212 (89-323)		11 (5-17)	
2005	Chad									1250 (529-1902)		63 (27-96)		215 (91-328)		11 (5-17)	
2006	Chad									1328 (568-2014)		65 (28-98)		229 (98-347)		11 (5-17)	
2007	Chad									1325 (572-2005)		63 (27-95)		228 (99-345)		11 (5-16)	
2008	Chad									1293 (564-1951)		60 (26-90)		223 (97-336)		10 (4-16)	
2009	Chad									776 (342-1168)		35 (15-53)		134 (59-201)		6 (3-9)	
2010	Chad									275 (122-412)		12 (5-18)		47 (21-71)		2 (1-3)	
2011	Chad									499 (224-746)		21 (10-32)		86 (39-129)		4 (2-5)	
2012	Chad									241 (110-360)		10 (5-15)		42 (19-62)		2 (1-3)	
2013	Chad									242 (73-396)		10 (3-16)		42 (13-68)		2 (1-3)	
2014	Chad									252 (78-411)		10 (3-16)		43 (13-71)		2 (1-3)	
2015	Chad	4150 (3789-6780)	120 (110-196)	160 (146-262)	5 (4-1)	772 (462-1558)	22 (13-45)	30 (18-60)	1 (1-0)	230 (72-374)	7 (2-11)	9 (3-14)	0 (0-0)	40 (12-64)	1 (0-2)	2 (0-2)	0 (0-0)
2000	Chile	984 (898-1607)		78 (71-127)		183 (110-370)		14 (9-29)		32 (14-57)		3 (1-5)		11 (5-19)		1 (0-2)	
2001	Chile									6 (2-10)		0 (0-1)		2 (1-3)		0 (0-0)	
2002	Chile									4 (2-7)		0 (0-1)		1 (1-2)		0 (0-0)	
2003	Chile									4 (2-7)		0 (0-1)		1 (1-2)		0 (0-0)	
2004	Chile									3 (1-6)		0 (0-0)		1 (1-2)		0 (0-0)	
2005	Chile									4 (2-6)		0 (0-1)		1 (1-2)		0 (0-0)	
2006	Chile									2 (1-4)		0 (0-0)		1 (0-1)		0 (0-0)	
2007	Chile									2 (1-3)		0 (0-0)		1 (0-1)		0 (0-0)	
2008	Chile									2 (1-3)		0 (0-0)		1 (0-1)		0 (0-0)	
2009	Chile									1 (1-3)		0 (0-0)		0 (0-1)		0 (0-0)	
2010	Chile									3 (1-5)		0 (0-0)		1 (0-2)		0 (0-0)	
2011	Chile									2 (1-3)		0 (0-0)		1 (0-1)		0 (0-0)	
2012	Chile									2 (1-4)		0 (0-0)		1 (0-1)		0 (0-0)	
2013	Chile									1 (0-2)		0 (0-0)		0 (0-1)		0 (0-0)	
2014	Chile									2 (1-3)		0 (0-0)		1 (0-1)		0 (0-0)	
2015	Chile	69 (63-113)	0 (0-0)	6 (5-10)	0 (0-0)	13 (8-26)	0 (0-0)	1 (1-2)	0 (0-0)	2 (1-3)	0 (0-0)	0 (0-0)	0 (0-0)	1 (0-1)	0 (0-0)	0 (0-0)	0 (0-0)
2000	China	1057559 (965574-1727949)		1347 (1230-2201)		197961 (118529-399559)		252 (151-509)		16775 (9490-26409)		21 (12-34)		5655 (3199-8902)		7 (4-11)	
2001	China									15714 (9079-25266)		20 (12-33)		5297 (3061-8517)		7 (4-11)	
2002	China									15312 (9156-25480)		20 (12-34)		5162 (3086-8589)		7 (4-11)	
2003	China									16110 (6520-21483)		22 (9-29)		5431 (2198-7242)		7 (3-10)	
2004	China									16858 (7419-24447)		23 (10-33)		5683 (2501-8241)		8 (3-11)	
2005	China									17782 (8514-28056)		24 (11-38)		5994 (2870-9458)		8 (4-13)	
2006	China									18944 (9599-31630)		25 (12-41)		6386 (3236-10662)		8 (4-14)	
2007	China									18683 (9467-31194)		24 (12-40)		6298 (3191-10515)		8 (4-14)	
2008	China									19176 (9717-32018)		25 (13-41)		6464 (3275-10793)		8 (4-14)	
2009	China									18969 (9612-31671)		25 (12-41)		6394 (3240-10676)		8 (4-14)	
2010	China									18717 (9484-31250)		24 (12-40)		6309 (3197-10535)		8 (4-14)	
2011	China									17663 (8950-29492)		23 (11-38)		5954 (3017-9942)		8 (4-13)	
2012	China									15919 (8066-26579)		20 (10-34)		5366 (2719-8960)		7 (3-11)	
2013	China									14657 (7427-24473)		18 (9-30)		4941 (2504-8250)		6 (3-10)	
2014	China									13135 (6655-21931)		16 (8-27)		4428 (2244-7393)		5 (3-9)	
2015	China	327710 (299206-535446)	69 (63-113)	401 (366-655)	0 (0-0)	60847 (36432-122813)	13 (8-26)	74 (45-150)	0 (0-0)	12464 (6315-20810)	3 (1-4)	15 (8-25)	0 (0-0)	4202 (2129-7015)	1 (0-1)	5 (3-9)	0 (0-0)
2000	Colombia	5344 (4879-8731)		131 (120-214)		999 (598-2017)		24 (15-49)		63 (6-112)		2 (0-3)		21 (2-38)		1 (0-1)	
2001	Colombia									68 (7-120)		2 (0-3)		23 (2-41)		1 (0-1)	
2002	Colombia									59 (6-105)		1 (0-3)		20 (2-35)		0 (0-1)	
2003	Colombia									6 (1-10)		0 (0-0)		2 (0-3)		0 (0-0)	
2004	Colombia									5 (1-9)		0 (0-0)		2 (0-3)		0 (0-0)	
2005	Colombia									6 (1-11)		0 (0-0)		2 (0-4)		0 (0-0)	
2006	Colombia									6 (1-10)		0 (0-0)		2 (0-3)		0 (0-0)	
2007	Colombia									4 (1-8)		0 (0-0)		1 (0-3)		0 (0-0)	
2008	Colombia									4 (0-7)		0 (0-0)		1 (0-2)		0 (0-0)	
2009	Colombia									4 (0-7)		0 (0-0)		1 (0-2)		0 (0-0)	
2010	Colombia									3 (0-6)		0 (0-0)		1 (0-2)		0 (0-0)	
2011	Colombia									3 (0-6)		0 (0-0)		1 (0-2)		0 (0-0)	
2012	Colombia									3 (0-5)		0 (0-0)		1 (0-2)		0 (0-0)	
2013	Colombia									3 (0-6)		0 (0-0)		1 (0-2)		0 (0-0)	
2014	Colombia									3 (0-6)		0 (0-0)		1 (0-2)		0 (0-0)	
2015	Colombia	217 (198-355)	0 (0-0)	6 (5-10)	0 (0-0)	41 (24-82)	0 (0-0)	1 (1-2)	0 (0-0)	3 (0-6)	0 (0-0)	0 (0-0)	0 (0-0)	1 (0-2)	0 (0-0)	0 (0-0)	0 (0-0)
2000	Comoros	1938 (1770-3167)		2195 (2004-3586)		362 (217-730)		410 (245-827)		34 (12-53)		38 (14-60)		6 (2-9)		7 (2-10)	
2001	Comoros									34 (12-53)		37 (13-59)		6 (2-9)		6 (2-10)	
2002	Comoros									34 (12-54)		37 (13-59)		6 (2-9)		6 (2-10)	
2003	Comoros									34 (12-54)		37 (13-58)		6 (2-9)		6 (2-10)	
2004	Comoros									34 (12-54)		36 (12-57)		6 (2-9)		6 (2-10)	
2005	Comoros									33 (11-53)		34 (12-55)		6 (2-9)		6 (2-9)	
2006	Comoros									32 (11-52)		33 (11-52)		6 (2-9)		6 (2-9)	
2007	Comoros									31 (10-50)		31 (10-50)		5 (2-9)		5 (2-9)	
2008	Comoros									31 (10-50)		30 (10-48)		5 (2-9)		5 (2-8)	
2009	Comoros									20 (7-32)		19 (6-31)		3 (1-6)		3 (1-5)	
2010	Comoros									3 (1-5)		3 (1-5)		1 (0-1)		1 (0-1)	
2011	Comoros									3 (1-4)		2 (1-4)		0 (0-1)		0 (0-1)	
2012	Comoros									2 (1-4)		2 (1-3)		0 (0-1)		0 (0-1)	
2013	Comoros									2 (1-4)		2 (1-3)		0 (0-1)		0 (0-1)	
2014	Comoros									0 (0-0)		0 (0-0)		0 (0-0)		0 (0-0)	
2015	Comoros	17 (15-27)	0 (0-0)	14 (13-23)	0 (0-0)	3 (2-6)	0 (0-0)	3 (2-5)	0 (0-0)	0 (0-0)	0 (0-0)	0 (0-0)	0 (0-0)	0 (0-0)	0 (0-0)	0 (0-0)	0 (0-0)
2000	Congo	5276 (4817-8620)		1062 (970-1735)		990 (593-1997)		199 (119-402)		151 (50-244)		30 (10-49)		26 (9-42)		5 (2-8)	
2001	Congo									158 (52-256)		31 (10-50)		27 (9-44)		5 (2-9)	
2002	Congo									161 (53-260)		31 (10-49)		28 (9-45)		5 (2-9)	
2003	Congo									161 (54-259)		30 (10-48)		28 (9-45)		5 (2-8)	
2004	Congo									161 (54-259)		29 (10-47)		28 (9-45)		5 (2-8)	
2005	Congo									160 (54-257)		28 (10-45)		28 (9-44)		5 (2-8)	
2006	Congo									156 (54-251)		27 (9-43)		27 (9-43)		5 (2-7)	
2007	Congo									152 (52-243)		25 (9-40)		26 (9-42)		4 (1-7)	
2008	Congo									146 (45-241)		23 (7-39)		49 (15-81)		8 (2-13)	
2009	Congo									36 (11-59)		6 (2-9)		12 (4-20)		2 (1-3)	
2010	Congo									14 (4-23)		2 (1-3)		5 (1-8)		1 (0-1)	
2011	Congo									12 (4-19)		2 (1-3)		4 (1-7)		1 (0-1)	
2012	Congo									11 (4-19)		2 (1-3)		4 (1-6)		1 (0-1)	
2013	Congo									10 (3-16)		1 (0-2)		3 (1-5)		0 (0-1)	
2014	Congo									1 (0-2)		0 (0-0)		0 (0-1)		0 (0-0)	
2015	Congo	80 (73-130)	3 (3-5)	11 (10-17)	0 (0-0)	15 (9-30)	1 (0-1)	2 (1-4)	0 (0-0)	1 (0-2)	0 (0-0)	0 (0-0)	0 (0-0)	0 (0-1)	0 (0-0)	0 (0-0)	0 (0-0)
2000	Cook Islands	28 (25-45)		1378 (1258-2252)		5 (3-10)		257 (154-518)		1 (1-1)		46 (27-65)		0 (0-0)		15 (9-22)	
2001	Cook Islands									1 (1-1)		47 (27-66)		0 (0-0)		16 (9-22)	
2002	Cook Islands									1 (1-1)		48 (27-67)		0 (0-0)		16 (9-23)	
2003	Cook Islands									1 (0-1)		29 (17-41)		0 (0-0)		10 (6-14)	
2004	Cook Islands									0 (0-1)		29 (17-41)		0 (0-0)		10 (6-14)	
2005	Cook Islands									1 (0-1)		47 (27-66)		0 (0-0)		16 (9-22)	
2006	Cook Islands									1 (0-1)		36 (21-51)		0 (0-0)		12 (7-17)	
2007	Cook Islands									1 (0-1)		34 (20-48)		0 (0-0)		11 (7-16)	
2008	Cook Islands									0 (0-1)		24 (14-33)		0 (0-0)		8 (5-11)	
2009	Cook Islands									0 (0-0)		7 (4-10)		0 (0-0)		3 (1-4)	
2010	Cook Islands									0 (0-0)		3 (2-4)		0 (0-0)		1 (1-1)	
2011	Cook Islands									0 (0-0)		3 (2-5)		0 (0-0)		1 (1-2)	
2012	Cook Islands									0 (0-0)		2 (1-3)		0 (0-0)		1 (0-1)	
2013	Cook Islands									0 (0-0)		2 (1-3)		0 (0-0)		1 (0-1)	
2014	Cook Islands									0 (0-0)		0 (0-0)		0 (0-0)		0 (0-0)	
2015	Cook Islands	0 (0-0)	0 (0-0)	16 (15-26)	0 (0-0)	0 (0-0)	0 (0-0)	3 (2-6)	0 (0-0)	0 (0-0)	0 (0-0)	0 (0-0)	0 (0-0)	0 (0-0)	0 (0-0)	0 (0-0)	0 (0-0)
2000	Costa Rica	285 (260-465)		74 (67-120)		53 (32-107)		14 (8-28)		2 (0-3)		0 (0-1)		1 (0-1)		0 (0-0)	
2001	Costa Rica									1 (0-2)		0 (0-1)		0 (0-1)		0 (0-0)	
2002	Costa Rica									2 (0-4)		1 (0-1)		1 (0-1)		0 (0-0)	
2003	Costa Rica									0 (0-0)		0 (0-0)		0 (0-0)		0 (0-0)	
2004	Costa Rica									0 (0-0)		0 (0-0)		0 (0-0)		0 (0-0)	
2005	Costa Rica									0 (0-0)		0 (0-0)		0 (0-0)		0 (0-0)	
2006	Costa Rica									0 (0-0)		0 (0-0)		0 (0-0)		0 (0-0)	
2007	Costa Rica									0 (0-0)		0 (0-0)		0 (0-0)		0 (0-0)	
2008	Costa Rica									0 (0-0)		0 (0-0)		0 (0-0)		0 (0-0)	
2009	Costa Rica									0 (0-0)		0 (0-0)		0 (0-0)		0 (0-0)	
2010	Costa Rica									0 (0-0)		0 (0-0)		0 (0-0)		0 (0-0)	
2011	Costa Rica									0 (0-0)		0 (0-0)		0 (0-0)		0 (0-0)	
2012	Costa Rica									0 (0-0)		0 (0-0)		0 (0-0)		0 (0-0)	
2013	Costa Rica									0 (0-0)		0 (0-0)		0 (0-0)		0 (0-0)	
2014	Costa Rica									0 (0-0)		0 (0-0)		0 (0-0)		0 (0-0)	
2015	Costa Rica	22 (20-36)	0 (0-0)	6 (6-10)	0 (0-0)	4 (2-8)	0 (0-0)	1 (1-2)	0 (0-0)	0 (0-0)	0 (0-0)	0 (0-0)	0 (0-0)	0 (0-0)	0 (0-0)	0 (0-0)	0 (0-0)
2000	Cote d'Ivoire	40434 (36918-66066)		1445 (1319-2360)		7638 (4573-15416)		273 (163-551)		585 (184-953)		21 (7-34)		101 (32-164)		4 (1-6)	
2001	Cote d'Ivoire									580 (181-944)		20 (6-33)		100 (31-163)		3 (1-6)	
2002	Cote d'Ivoire									569 (177-927)		19 (6-32)		98 (31-160)		3 (1-5)	
2003	Cote d'Ivoire									558 (173-909)		19 (6-31)		96 (30-157)		3 (1-5)	
2004	Cote d'Ivoire									543 (168-887)		18 (6-30)		94 (29-153)		3 (1-5)	
2005	Cote d'Ivoire									548 (169-895)		18 (6-30)		94 (29-154)		3 (1-5)	
2006	Cote d'Ivoire									562 (173-919)		18 (6-30)		97 (30-158)		3 (1-5)	
2007	Cote d'Ivoire									594 (183-970)		19 (6-31)		102 (32-167)		3 (1-5)	
2008	Cote d'Ivoire									625 (194-1019)		20 (6-33)		108 (33-175)		3 (1-6)	
2009	Cote d'Ivoire									114 (35-185)		4 (1-6)		20 (6-32)		1 (0-1)	
2010	Cote d'Ivoire									67 (21-109)		2 (1-3)		11 (4-19)		0 (0-1)	
2011	Cote d'Ivoire									103 (32-167)		3 (1-5)		18 (6-29)		1 (0-1)	
2012	Cote d'Ivoire									78 (25-127)		2 (1-4)		13 (4-22)		0 (0-1)	
2013	Cote d'Ivoire									85 (27-138)		2 (1-4)		15 (5-24)		0 (0-1)	
2014	Cote d'Ivoire									9 (3-14)		0 (0-0)		2 (0-2)		0 (0-0)	
2015	Cote d'Ivoire	221 (202-361)	6 (5-9)	6 (6-10)	0 (0-0)	41 (25-83)	1 (1-2)	1 (1-2)	0 (0-0)	9 (3-14)	0 (0-0)	0 (0-0)	0 (0-0)	2 (0-2)	0 (0-0)	0 (0-0)	0 (0-0)
2000	Croatia	177 (162-290)		74 (68-122)		55 (33-111)		23 (14-47)		28 (12-52)		12 (5-22)		9 (4-17)		4 (2-7)	
2001	Croatia									32 (14-59)		13 (6-24)		11 (5-20)		4 (2-8)	
2002	Croatia									6 (3-11)		3 (1-5)		2 (1-4)		1 (0-2)	

Hib = *Haemophilus influenzae* type b; npnm = non-pneumonia, non-meningitis; HIV = cases only in HIV-infected children; Uncertainty range provided in parentheses.

Year	Country	Hib pneumonia cases (UR)	Hib pneumonia cases, HIV (UR)	Hib pneumonia incidence rate (UR)	Hib pneumonia incidence rate, HIV (UR)	Hib severe pneumonia cases (UR)	Hib severe pneumonia cases, HIV (UR)	Hib severe pneumonia incidence rate (UR)	Hib severe pneumonia incidence rate, HIV (UR)	Hib meningitis cases (UR)	Hib meningitis cases, HIV (UR)	Hib meningitis incidence rate (UR)	Hib meningitis incidence rate, HIV (UR)	Hib NPNM cases (UR)	Hib NPNM cases, HIV (UR)	Hib NPNM incidence rate (UR)	Hib NPNM incidence rate, HIV (UR)
2003	Croatia									1 (1-2)		1 (0-1)		0 (0-1)		0 (0-0)	
2004	Croatia									1 (0-1)		0 (0-0)		0 (0-0)		0 (0-0)	
2005	Croatia									3 (1-6)		2 (1-3)		1 (0-2)		1 (0-1)	
2006	Croatia									1 (0-1)		0 (0-1)		0 (0-0)		0 (0-0)	
2007	Croatia									0 (0-0)		0 (0-0)		0 (0-0)		0 (0-0)	
2008	Croatia									0 (0-0)		0 (0-0)		0 (0-0)		0 (0-0)	
2009	Croatia									0 (0-1)		0 (0-0)		0 (0-0)		0 (0-0)	
2010	Croatia									0 (0-1)		0 (0-0)		0 (0-0)		0 (0-0)	
2011	Croatia									0 (0-1)		0 (0-0)		0 (0-0)		0 (0-0)	
2012	Croatia									0 (0-1)		0 (0-0)		0 (0-0)		0 (0-0)	
2013	Croatia									0 (0-0)		0 (0-0)		0 (0-0)		0 (0-0)	
2014	Croatia									0 (0-1)		0 (0-0)		0 (0-0)		0 (0-0)	
2015	Croatia	2 (1-2)	0 (0-0)	1 (1-1)	0 (0-0)	0 (0-1)	0 (0-0)	0 (0-0)	0 (0-0)	0 (0-0)	0 (0-0)	0 (0-0)	0 (0-0)	0 (0-0)	0 (0-0)	0 (0-0)	0 (0-0)
2000	Cuba	65 (60-107)		9 (8-14)		20 (12-41)		3 (2-6)		15 (5-24)		2 (1-3)		5 (2-8)		1 (0-1)	
2001	Cuba									11 (4-18)		1 (1-2)		4 (1-6)		0 (0-1)	
2002	Cuba									5 (2-9)		1 (0-1)		2 (1-3)		0 (0-0)	
2003	Cuba									8 (3-13)		1 (0-2)		3 (1-5)		0 (0-1)	
2004	Cuba									1 (0-2)		0 (0-0)		0 (0-1)		0 (0-0)	
2005	Cuba									1 (0-2)		0 (0-0)		0 (0-1)		0 (0-0)	
2006	Cuba									1 (0-2)		0 (0-0)		0 (0-1)		0 (0-0)	
2007	Cuba									1 (0-1)		0 (0-0)		0 (0-0)		0 (0-0)	
2008	Cuba									1 (0-2)		0 (0-0)		0 (0-1)		0 (0-0)	
2009	Cuba									1 (0-1)		0 (0-0)		0 (0-0)		0 (0-0)	
2010	Cuba									1 (0-1)		0 (0-0)		0 (0-0)		0 (0-0)	
2011	Cuba									1 (0-2)		0 (0-0)		0 (0-1)		0 (0-0)	
2012	Cuba									1 (0-2)		0 (0-0)		0 (0-1)		0 (0-0)	
2013	Cuba									1 (0-1)		0 (0-0)		0 (0-0)		0 (0-0)	
2014	Cuba									1 (0-1)		0 (0-0)		0 (0-0)		0 (0-0)	
2015	Cuba	9 (8-14)	0 (0-0)	1 (1-2)	0 (0-0)	3 (2-5)	0 (0-0)	0 (0-1)	0 (0-0)	1 (0-1)	0 (0-0)	0 (0-0)	0 (0-0)	0 (0-0)	0 (0-0)	0 (0-0)	0 (0-0)
2000	Cyprus	75 (68-122)		112 (102-182)		23 (14-47)		35 (21-70)		6 (3-10)		9 (4-15)		2 (1-3)		3 (2-5)	
2001	Cyprus									3 (1-5)		4 (2-7)		1 (0-2)		1 (1-2)	
2002	Cyprus									2 (1-4)		4 (2-6)		1 (0-1)		1 (1-2)	
2003	Cyprus									1 (0-1)		1 (1-2)		0 (0-1)		0 (0-1)	
2004	Cyprus									1 (0-1)		1 (1-2)		0 (0-0)		0 (0-1)	
2005	Cyprus									1 (0-1)		1 (1-2)		0 (0-0)		0 (0-1)	
2006	Cyprus									0 (0-0)		0 (0-0)		0 (0-0)		0 (0-0)	
2007	Cyprus									0 (0-0)		0 (0-0)		0 (0-0)		0 (0-0)	
2008	Cyprus									0 (0-0)		0 (0-0)		0 (0-0)		0 (0-0)	
2009	Cyprus									0 (0-0)		0 (0-0)		0 (0-0)		0 (0-0)	
2010	Cyprus									0 (0-0)		0 (0-0)		0 (0-0)		0 (0-0)	
2011	Cyprus									0 (0-0)		0 (0-0)		0 (0-0)		0 (0-0)	
2012	Cyprus									0 (0-0)		0 (0-0)		0 (0-0)		0 (0-0)	
2013	Cyprus									0 (0-0)		0 (0-0)		0 (0-0)		0 (0-0)	
2014	Cyprus									0 (0-0)		0 (0-0)		0 (0-0)		0 (0-0)	
2015	Cyprus	1 (1-2)	0 (0-0)	1 (1-2)	0 (0-0)	0 (0-1)	0 (0-0)	0 (0-1)	0 (0-0)	0 (0-0)	0 (0-0)	0 (0-0)	0 (0-0)	0 (0-0)	0 (0-0)	0 (0-0)	0 (0-0)
2000	Czech Republic	327 (298-534)		74 (68-122)		101 (61-205)		23 (14-47)		37 (16-68)		8 (4-15)		12 (5-23)		3 (1-5)	
2001	Czech Republic									41 (18-76)		10 (4-19)		14 (6-26)		3 (1-6)	
2002	Czech Republic									4 (2-8)		1 (0-2)		1 (1-3)		0 (0-1)	
2003	Czech Republic									2 (1-4)		1 (0-1)		1 (0-1)		0 (0-0)	
2004	Czech Republic									5 (2-10)		1 (1-2)		2 (1-3)		0 (0-1)	
2005	Czech Republic									3 (1-6)		1 (0-1)		1 (0-2)		0 (0-0)	
2006	Czech Republic									1 (1-2)		0 (0-0)		0 (0-1)		0 (0-0)	
2007	Czech Republic									1 (0-2)		0 (0-0)		0 (0-1)		0 (0-0)	
2008	Czech Republic									0 (0-1)		0 (0-0)		0 (0-0)		0 (0-0)	
2009	Czech Republic									1 (0-1)		0 (0-0)		0 (0-0)		0 (0-0)	
2010	Czech Republic									0 (0-0)		0 (0-0)		0 (0-0)		0 (0-0)	
2011	Czech Republic									0 (0-1)		0 (0-0)		0 (0-0)		0 (0-0)	
2012	Czech Republic									0 (0-1)		0 (0-0)		0 (0-0)		0 (0-0)	
2013	Czech Republic									0 (0-1)		0 (0-0)		0 (0-0)		0 (0-0)	
2014	Czech Republic									0 (0-1)		0 (0-0)		0 (0-0)		0 (0-0)	
2015	Czech Republic	4 (4-6)	0 (0-0)	1 (1-1)	0 (0-0)	1 (1-2)	0 (0-0)	0 (0-0)	0 (0-0)	0 (0-1)	0 (0-0)	0 (0-0)	0 (0-0)	0 (0-0)	0 (0-0)	0 (0-0)	0 (0-0)
2000	Democratic People's Republic of Korea	32978 (30110-53883)		1691 (1544-2763)		6193 (3708-12499)		318 (190-641)		1628 (903-2514)		83 (46-129)		549 (305-847)		28 (16-43)	
2001	Democratic People's Republic of Korea									1300 (718-1997)		68 (38-105)		438 (242-673)		23 (13-35)	
2002	Democratic People's Republic of Korea									1019 (584-1534)		54 (31-81)		343 (197-517)		18 (10-27)	
2003	Democratic People's Republic of Korea									802 (447-1186)		43 (24-63)		270 (151-400)		14 (8-21)	
2004	Democratic People's Republic of Korea									645 (343-939)		34 (18-50)		218 (116-317)		12 (6-17)	
2005	Democratic People's Republic of Korea									560 (284-811)		30 (15-44)		189 (96-273)		10 (5-15)	
2006	Democratic People's Republic of Korea									505 (246-741)		27 (13-40)		170 (83-250)		9 (5-14)	
2007	Democratic People's Republic of Korea									464 (217-688)		26 (12-38)		156 (73-232)		9 (4-13)	
2008	Democratic People's Republic of Korea									418 (188-627)		24 (11-36)		141 (63-211)		8 (4-12)	
2009	Democratic People's Republic of Korea									384 (167-582)		22 (10-34)		129 (56-196)		8 (3-11)	
2010	Democratic People's Republic of Korea									357 (150-549)		21 (9-32)		120 (51-185)		7 (3-11)	
2011	Democratic People's Republic of Korea									338 (138-526)		20 (8-32)		114 (47-177)		7 (3-11)	
2012	Democratic People's Republic of Korea									243 (40-405)		15 (2-24)		82 (14-136)		5 (1-8)	
2013	Democratic People's Republic of Korea									24 (4-40)		1 (0-2)		8 (1-14)		0 (0-1)	
2014	Democratic People's Republic of Korea									23 (3-39)		1 (0-2)		8 (1-13)		0 (0-1)	
2015	Democratic People's Republic of Korea	846 (773-1383)	0 (0-0)	49 (45-80)	0 (0-0)	158 (94-318)	0 (0-0)	9 (5-19)	0 (0-0)	20 (3-35)	0 (0-0)	1 (0-2)	0 (0-0)	7 (1-12)	0 (0-0)	0 (0-1)	0 (0-0)
2000	Democratic Republic of the Congo	177658 (162206-290276)		1992 (1818-3254)		33440 (20022-67495)		375 (224-757)		4604 (2142-6838)		52 (24-77)		793 (369-1177)		9 (4-13)	
2001	Democratic Republic of the Congo									4589 (2140-6808)		50 (23-74)		790 (369-1172)		9 (4-13)	
2002	Democratic Republic of the Congo									4659 (2182-6899)		49 (23-73)		802 (376-1188)		8 (4-13)	
2003	Democratic Republic of the Congo									4709 (1481-7672)		48 (15-78)		811 (255-1321)		8 (3-14)	
2004	Democratic Republic of the Congo									4842 (1534-7874)		48 (15-78)		834 (264-1356)		8 (3-13)	
2005	Democratic Republic of the Congo									4755 (1518-7720)		46 (15-74)		819 (261-1329)		8 (3-13)	
2006	Democratic Republic of the Congo									4921 (1582-7975)		46 (15-74)		847 (272-1373)		8 (3-13)	
2007	Democratic Republic of the Congo									4963 (1608-8029)		45 (14-72)		855 (277-1383)		8 (2-12)	
2008	Democratic Republic of the Congo									5247 (1693-8499)		46 (15-74)		904 (292-1463)		8 (3-13)	
2009	Democratic Republic of the Congo									1535 (493-2489)		13 (4-21)		264 (85-429)		2 (1-4)	
2010	Democratic Republic of the Congo									844 (270-1371)		7 (2-11)		145 (47-236)		1 (0-2)	
2011	Democratic Republic of the Congo									750 (241-1216)		6 (2-10)		129 (41-209)		1 (0-2)	
2012	Democratic Republic of the Congo									786 (253-1274)		6 (2-10)		135 (44-219)		1 (0-2)	
2013	Democratic Republic of the Congo									818 (264-1324)		6 (2-10)		141 (45-228)		1 (0-2)	
2014	Democratic Republic of the Congo									77 (25-124)		1 (0-1)		13 (4-21)		0 (0-0)	
2015	Democratic Republic of the Congo	1800 (1643-2940)	21 (20-35)	13 (12-22)	0 (0-0)	339 (203-685)	4 (2-8)	2 (1-5)	0 (0-0)	77 (25-124)	1 (0-1)	1 (0-1)	0 (0-0)	13 (4-21)	0 (0-0)	0 (0-0)	0 (0-0)
2000	Denmark	2 (2-4)		1 (1-1)		1 (0-2)		0 (0-0)		0 (0-1)		0 (0-0)		0 (0-0)		0 (0-0)	
2001	Denmark									0 (0-1)		0 (0-0)		0 (0-0)		0 (0-0)	
2002	Denmark									0 (0-1)		0 (0-0)		0 (0-0)		0 (0-0)	
2003	Denmark									0 (0-1)		0 (0-0)		0 (0-0)		0 (0-0)	
2004	Denmark									0 (0-1)		0 (0-0)		0 (0-0)		0 (0-0)	
2005	Denmark									0 (0-0)		0 (0-0)		0 (0-0)		0 (0-0)	
2006	Denmark									0 (0-0)		0 (0-0)		0 (0-0)		0 (0-0)	
2007	Denmark									0 (0-0)		0 (0-0)		0 (0-0)		0 (0-0)	
2008	Denmark									0 (0-0)		0 (0-0)		0 (0-0)		0 (0-0)	
2009	Denmark									0 (0-0)		0 (0-0)		0 (0-0)		0 (0-0)	
2010	Denmark									0 (0-0)		0 (0-0)		0 (0-0)		0 (0-0)	
2011	Denmark									0 (0-0)		0 (0-0)		0 (0-0)		0 (0-0)	
2012	Denmark									0 (0-0)		0 (0-0)		0 (0-0)		0 (0-0)	
2013	Denmark									0 (0-0)		0 (0-0)		0 (0-0)		0 (0-0)	
2014	Denmark									0 (0-0)		0 (0-0)		0 (0-0)		0 (0-0)	
2015	Denmark	2 (2-4)	0 (0-0)	1 (1-1)	0 (0-0)	1 (0-1)	0 (0-0)	0 (0-0)	0 (0-0)	0 (0-0)	0 (0-0)	0 (0-0)	0 (0-0)	0 (0-0)	0 (0-0)	0 (0-0)	0 (0-0)
2000	Djibouti	929 (848-1517)		934 (853-1526)		173 (103-348)		174 (104-350)		39 (15-61)		39 (15-61)		7 (3-10)		7 (3-10)	
2001	Djibouti									38 (15-59)		38 (15-60)		7 (3-10)		7 (3-10)	
2002	Djibouti									37 (14-58)		38 (15-59)		6 (2-10)		6 (3-10)	
2003	Djibouti									36 (14-57)		37 (14-58)		6 (2-10)		6 (2-10)	
2004	Djibouti									35 (14-55)		36 (14-56)		6 (2-10)		6 (2-10)	
2005	Djibouti									35 (13-54)		35 (14-55)		6 (2-9)		6 (2-9)	
2006	Djibouti									34 (13-54)		35 (13-54)		6 (2-9)		6 (2-9)	
2007	Djibouti									29 (12-45)		29 (12-45)		5 (2-8)		5 (2-8)	
2008	Djibouti									3 (1-4)		3 (1-5)		1 (0-1)		1 (0-1)	
2009	Djibouti									3 (1-5)		3 (1-5)		1 (0-1)		1 (0-1)	
2010	Djibouti									3 (2-5)		4 (2-5)		1 (0-1)		1 (0-1)	
2011	Djibouti									4 (2-6)		4 (2-6)		1 (1-2)		1 (1-2)	
2012	Djibouti									1 (0-1)		1 (0-1)		0 (0-0)		0 (0-0)	
2013	Djibouti									1 (0-1)		1 (0-1)		0 (0-0)		0 (0-0)	
2014	Djibouti									1 (0-1)		1 (0-1)		0 (0-0)		0 (0-0)	
2015	Djibouti	9 (8-15)	0 (0-0)	9 (8-15)	0 (0-0)	2 (1-4)	0 (0-0)	2 (1-4)	0 (0-0)	1 (0-1)	0 (0-0)	1 (0-1)	0 (0-0)	0 (0-0)	0 (0-0)	0 (0-0)	0 (0-0)
2000	Dominica	65 (59-105)		1271 (1160-2076)		12 (7-24)		237 (142-479)		0 (0-0)		0 (0-0)		0 (0-0)		0 (0-0)	
2001	Dominica									2 (1-3)		42 (18-75)		1 (0-1)		14 (6-25)	
2002	Dominica									1 (1-3)		33 (14-59)		0 (0-1)		11 (5-20)	
2003	Dominica									2 (1-3)		36 (16-63)		1 (0-1)		12 (5-21)	
2004	Dominica									0 (0-0)		0 (0-0)		0 (0-0)		0 (0-0)	
2005	Dominica									0 (0-0)		0 (0-0)		0 (0-0)		0 (0-0)	
2006	Dominica									0 (0-0)		0 (0-0)		0 (0-0)		0 (0-0)	
2007	Dominica									0 (0-0)		0 (0-0)		0 (0-0)		0 (0-0)	
2008	Dominica									0 (0-0)		2 (1-3)		0 (0-0)		1 (0-1)	
2009	Dominica									0 (0-0)		1 (1-2)		0 (0-0)		0 (0-1)	
2010	Dominica									0 (0-0)		2 (1-3)		0 (0-0)		1 (0-1)	
2011	Dominica									0 (0-0)		0 (0-0)		0 (0-0)		0 (0-0)	
2012	Dominica									0 (0-0)		0 (0-0)		0 (0-0)		0 (0-0)	
2013	Dominica									0 (0-0)		0 (0-0)		0 (0-0)		0 (0-0)	
2014	Dominica									0 (0-0)		0 (0-0)		0 (0-0)		0 (0-0)	
2015	Dominica	0 (0-1)	0 (0-0)	7 (6-11)	0 (0-0)	0 (0-0)	0 (0-0)	1 (1-3)	0 (0-0)	0 (0-0)	0 (0-0)	0 (0-0)	0 (0-0)	0 (0-0)	0 (0-0)	0 (0-0)	0 (0-0)
2000	Dominican Republic	9358 (8544-15290)		933 (852-1525)		1746 (1046-3525)		174 (104-351)		90 (30-147)		9 (3-15)		30 (10-49)		3 (1-5)	
2001	Dominican Republic									74 (24-121)		7 (2-12)		25 (8-41)		2 (1-4)	
2002	Dominican Republic									13 (4-21)		1 (0-2)		4 (1-7)		0 (0-1)	
2003	Dominican Republic									8 (3-13)		1 (0-1)		3 (1-4)		0 (0-0)	
2004	Dominican Republic									7 (2-12)		1 (0-1)		2 (1-4)		0 (0-0)	
2005	Dominican Republic									6 (2-10)		1 (0-1)		2 (1-3)		0 (0-0)	
2006	Dominican Republic									1 (0-1)		0 (0-0)		0 (0-0)		0 (0-0)	
2007	Dominican Republic									1 (0-1)		0 (0-0)		0 (0-0)		0 (0-0)	
2008	Dominican Republic									1 (0-1)		0 (0-0)		0 (0-0)		0 (0-0)	
2009	Dominican Republic									1 (0-1)		0 (0-0)		0 (0-0)		0 (0-0)	
2010	Dominican Republic									1 (0-1)		0 (0-0)		0 (0-0)		0 (0-0)	
2011	Dominican Republic									1 (0-1)		0 (0-0)		0 (0-0)		0 (0-0)	
2012	Dominican Republic									1 (0-1)		0 (0-0)		0 (0-0)		0 (0-0)	
2013	Dominican Republic									1 (0-1)		0 (0-0)		0 (0-0)		0 (0-0)	
2014	Dominican Republic									1 (0-1)		0 (0-0)		0 (0-0)		0 (0-0)	
2015	Dominican Republic	72 (66-117)	0 (0-0)	7 (6-11)	0 (0-0)	13 (8-27)	0 (0-0)	1 (1-3)	0 (0-0)	1 (0-1)	0 (0-0)	0 (0-0)	0 (0-0)	0 (0-0)	0 (0-0)	0 (0-0)	0 (0-0)
2000	Ecuador	13954 (12741-22800)		934 (853-1526)		2598 (1555-5243)		174 (104-351)		393 (197-620)		26 (13-42)		132 (66-209)		9 (4-14)	
2001	Ecuador									373 (187-589)		25 (12-39)		126 (63-198)		8 (4-13)	
2002	Ecuador									368 (186-586)		25 (12-39)		124 (63-197)		8 (4-13)	
2003	Ecuador									284 (146-460)		19 (10-31)		96 (49-155)		6 (3-10)	
2004	Ecuador									71 (38-120)		5 (3-8)		24 (13-40)		2 (1-3)	
2005	Ecuador									76 (27-109)		5 (2-7)		26 (9-37)		2 (1-2)	
2006	Ecuador									80 (30-123)		5 (2-8)		27 (10-41)		2 (1-3)	
2007	Ecuador									85 (35-143)		6 (2-9)		29 (12-48)		2 (1-3)	
2008	Ecuador									13 (6-22)		1 (0-1)		4 (2-8)		0 (0-0)	
2009	Ecuador									12 (5-22)		1 (0-1)		4 (2-7)		0 (0-0)	
2010	Ecuador									12 (5-21)		1 (0-1)		4 (2-7)		0 (0-0)	
2011	Ecuador									13 (6-22)		1 (0-1)		4 (2-8)		0 (0-0)	

Hib = *Haemophilus influenzae* type b; npnm = non-pneumonia, non-meningitis; HIV = cases only in HIV-infected children; Uncertainty range provided in parentheses.

Year	Country	Hib pneumonia cases (UR)	Hib pneumonia cases, HIV (UR)	Hib pneumonia incidence rate (UR)	Hib pneumonia incidence rate, HIV (UR)	Hib severe pneumonia cases (UR)	Hib severe pneumonia cases, HIV (UR)	Hib severe pneumonia incidence rate (UR)	Hib severe pneumonia incidence rate, HIV (UR)	Hib meningitis cases (UR)	Hib meningitis cases, HIV (UR)	Hib meningitis incidence rate (UR)	Hib meningitis incidence rate, HIV (UR)	Hib NPNM cases (UR)	Hib NPNM cases, HIV (UR)	Hib NPNM incidence rate (UR)	Hib NPNM incidence rate, HIV (UR)
2012	Ecuador									13 (6-24)		1 (0-1)		4 (2-8)		0 (0-1)	
2013	Ecuador									13 (6-23)		1 (0-1)		4 (2-8)		0 (0-0)	
2014	Ecuador									13 (6-23)		1 (0-1)		4 (2-8)		0 (0-0)	
2015	Ecuador	95 (87-155)	0 (0-0)	6 (5-10)	0 (0-0)	18 (11-36)	0 (0-0)	1 (1-2)	0 (0-0)	13 (6-22)	0 (0-0)	1 (0-1)	0 (0-0)	4 (2-8)	0 (0-0)	0 (0-0)	0 (0-0)
2000	Egypt	57428 (52433-93832)		731 (668-1195)		10663 (6384-21522)		136 (81-274)		571 (210-913)		7 (3-12)		192 (71-308)		2 (1-4)	
2001	Egypt									535 (200-851)		7 (3-11)		180 (67-287)		2 (1-4)	
2002	Egypt									496 (188-784)		6 (2-10)		167 (63-264)		2 (1-3)	
2003	Egypt									457 (176-719)		6 (2-9)		154 (59-242)		2 (1-3)	
2004	Egypt									425 (157-679)		5 (2-8)		143 (53-229)		2 (1-3)	
2005	Egypt									394 (141-637)		5 (2-7)		133 (47-215)		2 (1-2)	
2006	Egypt									376 (139-600)		4 (2-7)		127 (47-202)		1 (1-2)	
2007	Egypt									369 (142-584)		4 (2-7)		124 (48-197)		1 (1-2)	
2008	Egypt									365 (146-571)		4 (2-6)		123 (49-193)		1 (1-2)	
2009	Egypt									367 (146-580)		4 (2-6)		124 (49-196)		1 (1-2)	
2010	Egypt									378 (62-643)		4 (1-7)		128 (21-217)		1 (0-2)	
2011	Egypt									385 (63-661)		4 (1-7)		130 (21-223)		1 (0-2)	
2012	Egypt									393 (63-678)		4 (1-7)		133 (21-229)		1 (0-2)	
2013	Egypt									396 (63-686)		4 (1-6)		134 (21-231)		1 (0-2)	
2014	Egypt									91 (14-158)		1 (0-1)		31 (5-53)		0 (0-0)	
2015	Egypt	5023 (4586-8207)	1 (1-1)	42 (38-69)	0 (0-0)	939 (562-1896)	0 (0-0)	8 (5-16)	0 (0-0)	45 (7-79)	0 (0-0)	0 (0-1)	0 (0-0)	15 (2-27)	0 (0-0)	0 (0-0)	0 (0-0)
2000	El Salvador	9979 (9111-16305)		1418 (1294-2316)		1874 (1122-3783)		266 (159-537)		67 (20-111)		10 (3-16)		23 (7-37)		3 (1-5)	
2001	El Salvador									61 (19-102)		9 (3-15)		21 (6-34)		3 (1-5)	
2002	El Salvador									19 (2-33)		3 (0-5)		6 (1-11)		1 (0-2)	
2003	El Salvador									9 (1-15)		1 (0-2)		3 (0-5)		0 (0-1)	
2004	El Salvador									9 (1-15)		1 (0-2)		3 (0-5)		0 (0-1)	
2005	El Salvador									8 (1-15)		1 (0-2)		3 (0-5)		0 (0-1)	
2006	El Salvador									7 (1-12)		1 (0-2)		2 (0-4)		0 (0-1)	
2007	El Salvador									1 (0-2)		0 (0-0)		0 (0-1)		0 (0-0)	
2008	El Salvador									1 (0-1)		0 (0-0)		0 (0-0)		0 (0-0)	
2009	El Salvador									1 (0-1)		0 (0-0)		0 (0-0)		0 (0-0)	
2010	El Salvador									1 (0-1)		0 (0-0)		0 (0-0)		0 (0-0)	
2011	El Salvador									1 (0-1)		0 (0-0)		0 (0-0)		0 (0-0)	
2012	El Salvador									1 (0-1)		0 (0-0)		0 (0-0)		0 (0-0)	
2013	El Salvador									1 (0-1)		0 (0-0)		0 (0-0)		0 (0-0)	
2014	El Salvador									1 (0-1)		0 (0-0)		0 (0-0)		0 (0-0)	
2015	El Salvador	40 (37-66)	0 (0-0)	8 (7-13)	0 (0-0)	8 (5-15)	0 (0-0)	1 (1-3)	0 (0-0)	1 (0-1)	0 (0-0)	0 (0-0)	0 (0-0)	0 (0-0)	0 (0-0)	0 (0-0)	0 (0-0)
2000	Equatorial Guinea	1191 (1087-1945)		1371 (1252-2241)		224 (134-453)		258 (155-521)		25 (12-37)		29 (14-42)		4 (2-6)		5 (2-7)	
2001	Equatorial Guinea									24 (8-38)		27 (9-43)		4 (1-7)		5 (2-7)	
2002	Equatorial Guinea									22 (7-35)		24 (8-38)		4 (1-6)		4 (1-7)	
2003	Equatorial Guinea									19 (7-31)		21 (7-33)		3 (1-5)		4 (1-6)	
2004	Equatorial Guinea									18 (6-28)		19 (6-30)		3 (1-5)		3 (1-5)	
2005	Equatorial Guinea									17 (6-27)		17 (6-28)		3 (1-5)		3 (1-5)	
2006	Equatorial Guinea									17 (6-28)		17 (6-28)		3 (1-5)		3 (1-5)	
2007	Equatorial Guinea									18 (6-29)		18 (6-29)		3 (1-5)		3 (1-5)	
2008	Equatorial Guinea									20 (7-31)		19 (7-30)		3 (1-5)		3 (1-5)	
2009	Equatorial Guinea									20 (7-32)		19 (7-30)		3 (1-6)		3 (1-5)	
2010	Equatorial Guinea									21 (7-33)		19 (7-30)		4 (1-6)		3 (1-5)	
2011	Equatorial Guinea									21 (8-33)		18 (7-29)		4 (1-6)		3 (1-5)	
2012	Equatorial Guinea									21 (7-33)		17 (6-28)		4 (1-6)		3 (1-5)	
2013	Equatorial Guinea									21 (8-34)		18 (6-28)		4 (1-6)		3 (1-5)	
2014	Equatorial Guinea									19 (7-31)		16 (6-25)		3 (1-5)		3 (1-4)	
2015	Equatorial Guinea	1048 (957-1712)	77 (70-125)	831 (759-1358)	61 (55-10)	197 (118-397)	14 (9-29)	156 (93-315)	11 (7-2)	16 (6-25)	1 (0-2)	13 (5-20)	1 (0-0)	3 (1-4)	0 (0-0)	2 (1-3)	0 (0-0)
2000	Eritrea	12291 (11222-20083)		2233 (2039-3649)		2312 (1384-4666)		420 (252-848)		223 (74-361)		41 (13-66)		38 (13-62)		7 (2-11)	
2001	Eritrea									225 (74-363)		40 (13-64)		39 (13-63)		7 (2-11)	
2002	Eritrea									229 (75-369)		39 (13-63)		39 (13-64)		7 (2-11)	
2003	Eritrea									234 (77-378)		38 (13-62)		40 (13-65)		7 (2-11)	
2004	Eritrea									240 (71-401)		37 (11-63)		81 (24-135)		13 (4-21)	
2005	Eritrea									243 (72-406)		37 (11-61)		82 (24-137)		12 (4-21)	
2006	Eritrea									246 (73-410)		35 (10-59)		83 (25-138)		12 (4-20)	
2007	Eritrea									246 (73-410)		34 (10-57)		83 (25-138)		11 (3-19)	
2008	Eritrea									37 (11-61)		5 (1-8)		12 (4-21)		2 (0-3)	
2009	Eritrea									14 (4-24)		2 (1-3)		5 (1-8)		1 (0-1)	
2010	Eritrea									14 (4-23)		2 (1-3)		5 (1-8)		1 (0-1)	
2011	Eritrea									13 (4-22)		2 (1-3)		4 (1-7)		1 (0-1)	
2012	Eritrea									16 (5-27)		2 (1-3)		5 (2-9)		1 (0-1)	
2013	Eritrea									2 (1-3)		0 (0-0)		1 (0-1)		0 (0-0)	
2014	Eritrea									2 (1-3)		0 (0-0)		1 (0-1)		0 (0-0)	
2015	Eritrea	92 (84-150)	1 (1-1)	11 (10-19)	0 (0-0)	17 (10-35)	0 (0-0)	2 (1-4)	0 (0-0)	2 (1-3)	0 (0-0)	0 (0-0)	0 (0-0)	1 (0-1)	0 (0-0)	0 (0-0)	0 (0-0)
2000	Estonia	179 (164-293)		298 (272-486)		56 (33-112)		92 (55-187)		5 (2-10)		9 (4-16)		2 (1-3)		3 (1-5)	
2001	Estonia									10 (4-18)		16 (7-29)		3 (1-6)		5 (2-10)	
2002	Estonia									10 (5-19)		17 (7-31)		3 (2-6)		6 (3-11)	
2003	Estonia									5 (2-10)		9 (4-16)		2 (1-3)		3 (1-5)	
2004	Estonia									5 (2-9)		7 (3-14)		2 (1-3)		2 (1-5)	
2005	Estonia									3 (1-5)		4 (2-8)		1 (0-2)		1 (1-3)	
2006	Estonia									1 (0-1)		1 (0-1)		0 (0-0)		0 (0-0)	
2007	Estonia									0 (0-1)		1 (0-1)		0 (0-0)		0 (0-0)	
2008	Estonia									1 (1-3)		2 (1-4)		0 (0-1)		1 (0-1)	
2009	Estonia									0 (0-0)		0 (0-0)		0 (0-0)		0 (0-0)	
2010	Estonia									0 (0-0)		0 (0-0)		0 (0-0)		0 (0-0)	
2011	Estonia									0 (0-0)		0 (0-0)		0 (0-0)		0 (0-0)	
2012	Estonia									0 (0-0)		0 (0-0)		0 (0-0)		0 (0-0)	
2013	Estonia									0 (0-0)		0 (0-0)		0 (0-0)		0 (0-0)	
2014	Estonia									0 (0-0)		0 (0-0)		0 (0-0)		0 (0-0)	
2015	Estonia	2 (2-3)	0 (0-0)	3 (3-5)	0 (0-0)	1 (0-1)	0 (0-0)	1 (1-2)	0 (0-0)	0 (0-0)	0 (0-0)	0 (0-0)	0 (0-0)	0 (0-0)	0 (0-0)	0 (0-0)	0 (0-0)
2000	Ethiopia	212297 (193831-346872)		1740 (1589-2844)		39433 (23611-79591)		323 (194-653)		7336 (1972-12248)		60 (16-100)		1263 (339-2109)		10 (3-17)	
2001	Ethiopia									7132 (1924-11899)		57 (15-95)		1228 (331-2049)		10 (3-16)	
2002	Ethiopia									6789 (1839-11319)		53 (14-88)		1169 (317-1949)		9 (2-15)	
2003	Ethiopia									6359 (1729-10596)		49 (13-82)		1095 (298-1825)		8 (2-14)	
2004	Ethiopia									5875 (1604-9784)		45 (12-75)		1012 (276-1685)		8 (2-13)	
2005	Ethiopia									5380 (1475-8956)		41 (11-68)		927 (254-1542)		7 (2-12)	
2006	Ethiopia									5115 (1415-8502)		38 (11-64)		881 (244-1464)		7 (2-11)	
2007	Ethiopia									2620 (731-4348)		20 (5-32)		451 (126-749)		3 (1-6)	
2008	Ethiopia									592 (167-981)		4 (1-7)		102 (29-169)		1 (0-1)	
2009	Ethiopia									539 (153-892)		4 (1-7)		93 (26-154)		1 (0-1)	
2010	Ethiopia									465 (133-769)		3 (1-6)		80 (23-132)		1 (0-1)	
2011	Ethiopia									417 (107-711)		3 (1-5)		141 (36-240)		1 (0-2)	
2012	Ethiopia									32 (8-54)		0 (0-0)		11 (3-18)		0 (0-0)	
2013	Ethiopia									30 (8-52)		0 (0-0)		10 (3-17)		0 (0-0)	
2014	Ethiopia									30 (8-51)		0 (0-0)		10 (3-17)		0 (0-0)	
2015	Ethiopia	1655 (1511-2704)	12 (11-19)	12 (11-19)	0 (0-0)	310 (186-626)	2 (1-4)	2 (1-4)	0 (0-0)	29 (8-49)	0 (0-0)	0 (0-0)	0 (0-0)	10 (3-16)	0 (0-0)	0 (0-0)	0 (0-0)
2000	Fiji	86 (78-140)		89 (81-145)		16 (10-33)		17 (10-34)		11 (6-15)		11 (6-16)		4 (2-5)		4 (2-5)	
2001	Fiji									10 (6-15)		11 (6-16)		3 (2-5)		4 (2-5)	
2002	Fiji									2 (1-2)		2 (1-2)		1 (0-1)		1 (0-1)	
2003	Fiji									2 (1-2)		2 (1-2)		1 (0-1)		1 (0-1)	
2004	Fiji									2 (1-2)		2 (1-2)		1 (0-1)		1 (0-1)	
2005	Fiji									2 (1-2)		2 (1-3)		1 (0-1)		1 (0-1)	
2006	Fiji									2 (1-2)		2 (1-3)		1 (0-1)		1 (0-1)	
2007	Fiji									2 (1-3)		2 (1-3)		1 (0-1)		1 (0-1)	
2008	Fiji									2 (1-3)		2 (1-3)		1 (0-1)		1 (0-1)	
2009	Fiji									2 (1-3)		2 (1-3)		1 (0-1)		1 (0-1)	
2010	Fiji									2 (1-3)		2 (1-3)		1 (0-1)		1 (0-1)	
2011	Fiji									2 (1-3)		2 (1-3)		1 (0-1)		1 (0-1)	
2012	Fiji									2 (1-2)		2 (1-3)		1 (0-1)		1 (0-1)	
2013	Fiji									2 (1-3)		2 (1-3)		1 (0-1)		1 (0-1)	
2014	Fiji									2 (1-3)		2 (1-3)		1 (0-1)		1 (0-1)	
2015	Fiji	7 (6-11)	0 (0-0)	8 (7-13)	0 (0-0)	1 (1-3)	0 (0-0)	1 (1-3)	0 (0-0)	2 (1-2)	0 (0-0)	2 (1-3)	0 (0-0)	1 (0-1)	0 (0-0)	1 (0-1)	0 (0-0)
2000	Finland	2 (2-4)		1 (1-1)		1 (0-1)		0 (0-0)		0 (0-0)		0 (0-0)		0 (0-0)		0 (0-0)	
2001	Finland									0 (0-0)		0 (0-0)		0 (0-0)		0 (0-0)	
2002	Finland									0 (0-0)		0 (0-0)		0 (0-0)		0 (0-0)	
2003	Finland									0 (0-1)		0 (0-0)		0 (0-0)		0 (0-0)	
2004	Finland									0 (0-1)		0 (0-0)		0 (0-0)		0 (0-0)	
2005	Finland									0 (0-0)		0 (0-0)		0 (0-0)		0 (0-0)	
2006	Finland									0 (0-0)		0 (0-0)		0 (0-0)		0 (0-0)	
2007	Finland									0 (0-0)		0 (0-0)		0 (0-0)		0 (0-0)	
2008	Finland									0 (0-0)		0 (0-0)		0 (0-0)		0 (0-0)	
2009	Finland									0 (0-0)		0 (0-0)		0 (0-0)		0 (0-0)	
2010	Finland									0 (0-0)		0 (0-0)		0 (0-0)		0 (0-0)	
2011	Finland									0 (0-0)		0 (0-0)		0 (0-0)		0 (0-0)	
2012	Finland									0 (0-0)		0 (0-0)		0 (0-0)		0 (0-0)	
2013	Finland									0 (0-0)		0 (0-0)		0 (0-0)		0 (0-0)	
2014	Finland									0 (0-0)		0 (0-0)		0 (0-0)		0 (0-0)	
2015	Finland	2 (2-4)	0 (0-0)	1 (1-1)	0 (0-0)	1 (0-1)	0 (0-0)	0 (0-0)	0 (0-0)	0 (0-0)	0 (0-0)	0 (0-0)	0 (0-0)	0 (0-0)	0 (0-0)	0 (0-0)	0 (0-0)
2000	France	237 (216-387)		7 (6-11)		74 (44-148)		2 (1-4)		44 (19-81)		1 (1-2)		15 (7-27)		0 (0-1)	
2001	France									41 (18-76)		1 (0-2)		14 (6-26)		0 (0-1)	
2002	France									38 (17-71)		1 (0-2)		13 (6-24)		0 (0-1)	
2003	France									4 (2-7)		0 (0-0)		1 (1-2)		0 (0-0)	
2004	France									4 (2-7)		0 (0-0)		1 (1-2)		0 (0-0)	
2005	France									3 (1-5)		0 (0-0)		1 (0-2)		0 (0-0)	
2006	France									5 (2-9)		0 (0-0)		2 (1-3)		0 (0-0)	
2007	France									4 (2-7)		0 (0-0)		1 (1-2)		0 (0-0)	
2008	France									4 (2-8)		0 (0-0)		2 (1-3)		0 (0-0)	
2009	France									3 (1-6)		0 (0-0)		1 (0-2)		0 (0-0)	
2010	France									4 (2-7)		0 (0-0)		1 (1-3)		0 (0-0)	
2011	France									4 (2-8)		0 (0-0)		1 (1-3)		0 (0-0)	
2012	France									4 (2-8)		0 (0-0)		1 (1-3)		0 (0-0)	
2013	France									4 (2-8)		0 (0-0)		1 (1-3)		0 (0-0)	
2014	France									4 (2-8)		0 (0-0)		1 (1-3)		0 (0-0)	
2015	France	29 (26-47)	0 (0-0)	1 (1-1)	0 (0-0)	9 (5-18)	0 (0-0)	0 (0-0)	0 (0-0)	4 (2-8)	0 (0-0)	0 (0-0)	0 (0-0)	1 (1-3)	0 (0-0)	0 (0-0)	0 (0-0)
2000	Gabon	2884 (2633-4712)		1588 (1450-2594)		544 (326-1099)		300 (179-605)		17 (6-28)		10 (3-15)		3 (1-5)		2 (1-3)	
2001	Gabon									18 (6-29)		10 (3-16)		3 (1-5)		2 (1-3)	
2002	Gabon									19 (7-31)		10 (4-17)		3 (1-5)		2 (1-3)	
2003	Gabon									20 (7-33)		11 (4-17)		4 (1-6)		2 (1-3)	
2004	Gabon									22 (8-34)		11 (4-18)		4 (1-6)		2 (1-3)	
2005	Gabon									23 (8-36)		12 (4-19)		4 (1-6)		2 (1-3)	
2006	Gabon									24 (8-39)		12 (4-20)		8 (3-13)		4 (1-7)	
2007	Gabon									25 (8-40)		12 (4-20)		8 (3-13)		4 (1-7)	
2008	Gabon									25 (9-41)		12 (4-20)		8 (3-14)		4 (1-7)	
2009	Gabon									26 (9-41)		12 (4-20)		9 (3-14)		4 (1-7)	
2010	Gabon									8 (3-14)		4 (1-6)		3 (1-5)		1 (0-2)	
2011	Gabon									3 (1-5)		1 (0-2)		1 (0-2)		0 (0-1)	
2012	Gabon									3 (1-4)		1 (0-2)		1 (0-1)		0 (0-1)	
2013	Gabon									3 (1-4)		1 (0-2)		1 (0-1)		0 (0-1)	
2014	Gabon									3 (1-5)		1 (1-2)		1 (0-2)		0 (0-1)	
2015	Gabon	28 (25-45)	0 (0-0)	12 (11-19)	0 (0-0)	5 (3-10)	0 (0-0)	2 (1-4)	0 (0-0)	0 (0-0)	0 (0-0)	0 (0-0)	0 (0-0)	0 (0-0)	0 (0-0)	0 (0-0)	0 (0-0)
2000	Gambia	341 (312-558)		150 (137-246)		64 (39-130)		28 (17-57)		12 (5-18)		5 (2-8)		2 (1-3)		1 (0-1)	
2001	Gambia									12 (5-19)		5 (2-8)		2 (1-3)		1 (0-1)	
2002	Gambia									12 (5-18)		5 (2-8)		2 (1-3)		1 (0-1)	
2003	Gambia									1 (1-2)		1 (0-1)		0 (0-0)		0 (0-0)	
2004	Gambia									1 (1-2)		1 (0-1)		0 (0-0)		0 (0-0)	

Hib = *Haemophilus influenzae* type b; npnm = non-pneumonia, non-meningitis; HIV = cases only in HIV-infected children; Uncertainty range provided in parentheses.

Year	Country	Hib pneumonia cases (UR)	Hib pneumonia cases, HIV (UR)	Hib pneumonia incidence rate (UR)	Hib pneumonia incidence rate, HIV (UR)	Hib severe pneumonia cases (UR)	Hib severe pneumonia cases, HIV (UR)	Hib severe pneumonia incidence rate (UR)	Hib severe pneumonia incidence rate, HIV (UR)	Hib meningitis cases (UR)	Hib meningitis cases, HIV (UR)	Hib meningitis incidence rate (UR)	Hib meningitis incidence rate, HIV (UR)	Hib NPNM cases (UR)	Hib NPNM cases, HIV (UR)	Hib NPNM incidence rate (UR)	Hib NPNM incidence rate, HIV (UR)
2005	Gambia									1 (1-2)		1 (0-1)		0 (0-0)		0 (0-0)	
2006	Gambia									1 (1-2)		1 (0-1)		0 (0-0)		0 (0-0)	
2007	Gambia									1 (1-2)		0 (0-1)		0 (0-0)		0 (0-0)	
2008	Gambia									1 (1-2)		0 (0-1)		0 (0-0)		0 (0-0)	
2009	Gambia									1 (1-2)		0 (0-1)		0 (0-0)		0 (0-0)	
2010	Gambia									1 (1-2)		0 (0-1)		0 (0-0)		0 (0-0)	
2011	Gambia									1 (1-2)		0 (0-1)		0 (0-0)		0 (0-0)	
2012	Gambia									1 (1-2)		0 (0-1)		0 (0-0)		0 (0-0)	
2013	Gambia									1 (1-2)		0 (0-1)		0 (0-1)		0 (0-0)	
2014	Gambia									1 (1-2)		0 (0-1)		1 (0-1)		0 (0-0)	
2015	Gambia	41 (38-68)	1 (1-1)	11 (10-19)	0 (0-0)	8 (5-16)	0 (0-0)	2 (1-4)	0 (0-0)	2 (1-2)	0 (0-0)	0 (0-1)	0 (0-0)	1 (0-1)	0 (0-0)	0 (0-0)	0 (0-0)
2000	Georgia	404 (369-660)		149 (136-243)		126 (75-253)		46 (28-93)		66 (37-99)		24 (14-36)		22 (12-33)		8 (5-12)	
2001	Georgia									55 (30-80)		22 (12-32)		18 (10-27)		7 (4-11)	
2002	Georgia									47 (24-69)		19 (10-29)		16 (8-23)		6 (3-10)	
2003	Georgia									40 (9-64)		17 (4-27)		14 (3-22)		6 (1-9)	
2004	Georgia									34 (7-55)		14 (3-24)		11 (2-19)		5 (1-8)	
2005	Georgia									28 (5-47)		12 (2-21)		10 (2-16)		4 (1-7)	
2006	Georgia									23 (4-40)		10 (2-16)		8 (1-13)		3 (1-5)	
2007	Georgia									19 (3-32)		8 (1-13)		6 (1-11)		3 (0-4)	
2008	Georgia									15 (2-26)		6 (1-10)		5 (1-9)		2 (0-4)	
2009	Georgia									12 (1-22)		5 (1-8)		4 (1-7)		2 (0-3)	
2010	Georgia									6 (1-10)		2 (0-4)		2 (0-3)		1 (0-1)	
2011	Georgia									1 (0-2)		0 (0-1)		0 (0-1)		0 (0-0)	
2012	Georgia									1 (0-2)		0 (0-1)		0 (0-1)		0 (0-0)	
2013	Georgia									1 (0-1)		0 (0-1)		0 (0-0)		0 (0-0)	
2014	Georgia									1 (0-1)		0 (0-1)		0 (0-0)		0 (0-0)	
2015	Georgia	4 (4-7)	0 (0-0)	1 (1-2)	0 (0-0)	1 (1-3)	0 (0-0)	0 (0-1)	0 (0-0)	0 (0-0)	0 (0-0)	0 (0-0)	0 (0-0)	0 (0-0)	0 (0-0)	0 (0-0)	0 (0-0)
2000	Germany	28 (26-47)		1 (1-1)		9 (5-18)		0 (0-0)		5 (2-9)		0 (0-0)		2 (1-3)		0 (0-0)	
2001	Germany									3 (1-6)		0 (0-0)		1 (0-2)		0 (0-0)	
2002	Germany									5 (2-9)		0 (0-0)		2 (1-3)		0 (0-0)	
2003	Germany									4 (2-8)		0 (0-0)		1 (1-3)		0 (0-0)	
2004	Germany									4 (2-8)		0 (0-0)		1 (1-3)		0 (0-0)	
2005	Germany									3 (1-6)		0 (0-0)		1 (0-2)		0 (0-0)	
2006	Germany									4 (2-7)		0 (0-0)		1 (1-2)		0 (0-0)	
2007	Germany									3 (1-5)		0 (0-0)		1 (0-2)		0 (0-0)	
2008	Germany									2 (1-4)		0 (0-0)		1 (0-1)		0 (0-0)	
2009	Germany									2 (1-3)		0 (0-0)		1 (0-1)		0 (0-0)	
2010	Germany									2 (1-3)		0 (0-0)		1 (0-1)		0 (0-0)	
2011	Germany									2 (1-3)		0 (0-0)		1 (0-1)		0 (0-0)	
2012	Germany									2 (1-4)		0 (0-0)		1 (0-1)		0 (0-0)	
2013	Germany									2 (1-3)		0 (0-0)		1 (0-1)		0 (0-0)	
2014	Germany									2 (1-3)		0 (0-0)		1 (0-1)		0 (0-0)	
2015	Germany	25 (23-40)	0 (0-0)	1 (1-1)	0 (0-0)	8 (5-16)	0 (0-0)	0 (0-0)	0 (0-0)	2 (1-3)	0 (0-0)	0 (0-0)	0 (0-0)	1 (0-1)	0 (0-0)	0 (0-0)	0 (0-0)
2000	Ghana	40091 (36604-65505)		1426 (1302-2329)		7416 (4440-14969)		264 (158-532)		642 (230-1017)		23 (8-36)		111 (40-175)		4 (1-6)	
2001	Ghana									643 (224-1025)		23 (8-36)		111 (39-177)		4 (1-6)	
2002	Ghana									108 (37-174)		4 (1-6)		19 (6-30)		1 (0-1)	
2003	Ghana									63 (21-101)		2 (1-3)		11 (4-17)		0 (0-1)	
2004	Ghana									63 (20-102)		2 (1-3)		11 (3-18)		0 (0-1)	
2005	Ghana									60 (19-97)		2 (1-3)		10 (3-17)		0 (0-1)	
2006	Ghana									62 (19-101)		2 (1-3)		11 (3-17)		0 (0-1)	
2007	Ghana									7 (2-11)		0 (0-0)		1 (0-2)		0 (0-0)	
2008	Ghana									7 (2-11)		0 (0-0)		1 (0-2)		0 (0-0)	
2009	Ghana									7 (2-11)		0 (0-0)		1 (0-2)		0 (0-0)	
2010	Ghana									6 (2-11)		0 (0-0)		2 (1-4)		0 (0-0)	
2011	Ghana									6 (2-11)		0 (0-0)		2 (1-4)		0 (0-0)	
2012	Ghana									7 (2-11)		0 (0-0)		2 (1-4)		0 (0-0)	
2013	Ghana									7 (2-11)		0 (0-0)		2 (1-4)		0 (0-0)	
2014	Ghana									7 (2-12)		0 (0-0)		2 (1-4)		0 (0-0)	
2015	Ghana	361 (329-590)	4 (3-6)	9 (8-15)	0 (0-0)	68 (40-136)	1 (0-1)	2 (1-3)	0 (0-0)	7 (2-12)	0 (0-0)	0 (0-0)	0 (0-0)	2 (1-4)	0 (0-0)	0 (0-0)	0 (0-0)
2000	Greece	4 (3-6)		1 (1-1)		1 (1-2)		0 (0-0)		1 (0-1)		0 (0-0)		0 (0-0)		0 (0-0)	
2001	Greece									0 (0-1)		0 (0-0)		0 (0-0)		0 (0-0)	
2002	Greece									0 (0-0)		0 (0-0)		0 (0-0)		0 (0-0)	
2003	Greece									0 (0-1)		0 (0-0)		0 (0-0)		0 (0-0)	
2004	Greece									0 (0-1)		0 (0-0)		0 (0-0)		0 (0-0)	
2005	Greece									0 (0-0)		0 (0-0)		0 (0-0)		0 (0-0)	
2006	Greece									0 (0-0)		0 (0-0)		0 (0-0)		0 (0-0)	
2007	Greece									0 (0-0)		0 (0-0)		0 (0-0)		0 (0-0)	
2008	Greece									0 (0-0)		0 (0-0)		0 (0-0)		0 (0-0)	
2009	Greece									0 (0-1)		0 (0-0)		0 (0-0)		0 (0-0)	
2010	Greece									0 (0-0)		0 (0-0)		0 (0-0)		0 (0-0)	
2011	Greece									0 (0-0)		0 (0-0)		0 (0-0)		0 (0-0)	
2012	Greece									0 (0-0)		0 (0-0)		0 (0-0)		0 (0-0)	
2013	Greece									0 (0-0)		0 (0-0)		0 (0-0)		0 (0-0)	
2014	Greece									0 (0-0)		0 (0-0)		0 (0-0)		0 (0-0)	
2015	Greece	4 (4-6)	0 (0-0)	1 (1-1)	0 (0-0)	1 (1-2)	0 (0-0)	0 (0-0)	0 (0-0)	0 (0-0)	0 (0-0)	0 (0-0)	0 (0-0)	0 (0-0)	0 (0-0)	0 (0-0)	0 (0-0)
2000	Grenada	71 (65-117)		695 (635-1136)		13 (8-27)		130 (78-261)		0 (0-0)		0 (0-0)		0 (0-0)		0 (0-0)	
2001	Grenada									0 (0-0)		0 (0-0)		0 (0-0)		0 (0-0)	
2002	Grenada									0 (0-0)		0 (0-0)		0 (0-0)		0 (0-0)	
2003	Grenada									0 (0-0)		0 (0-0)		0 (0-0)		0 (0-0)	
2004	Grenada									0 (0-0)		0 (0-0)		0 (0-0)		0 (0-0)	
2005	Grenada									0 (0-0)		0 (0-0)		0 (0-0)		0 (0-0)	
2006	Grenada									0 (0-0)		0 (0-0)		0 (0-0)		0 (0-0)	
2007	Grenada									0 (0-0)		0 (0-0)		0 (0-0)		0 (0-0)	
2008	Grenada									0 (0-0)		0 (0-0)		0 (0-0)		0 (0-0)	
2009	Grenada									0 (0-0)		0 (0-0)		0 (0-0)		0 (0-0)	
2010	Grenada									0 (0-0)		0 (0-0)		0 (0-0)		0 (0-0)	
2011	Grenada									0 (0-0)		0 (0-0)		0 (0-0)		0 (0-0)	
2012	Grenada									0 (0-0)		0 (0-0)		0 (0-0)		0 (0-0)	
2013	Grenada									0 (0-0)		0 (0-0)		0 (0-0)		0 (0-0)	
2014	Grenada									0 (0-0)		0 (0-0)		0 (0-0)		0 (0-0)	
2015	Grenada	1 (1-1)	0 (0-0)	6 (5-10)	0 (0-0)	0 (0-0)	0 (0-0)	1 (1-2)	0 (0-0)	0 (0-0)	0 (0-0)	0 (0-0)	0 (0-0)	0 (0-0)	0 (0-0)	0 (0-0)	0 (0-0)
2000	Guatemala	35046 (31998-57262)		1903 (1737-3109)		6599 (3951-13320)		358 (215-723)		423 (131-696)		23 (7-38)		143 (44-235)		8 (2-13)	
2001	Guatemala									411 (128-675)		22 (7-36)		139 (43-228)		7 (2-12)	
2002	Guatemala									400 (126-656)		21 (7-35)		135 (42-221)		7 (2-12)	
2003	Guatemala									388 (122-635)		20 (6-33)		131 (41-214)		7 (2-11)	
2004	Guatemala									377 (120-616)		20 (6-32)		127 (40-208)		7 (2-11)	
2005	Guatemala									59 (19-96)		3 (1-5)		20 (6-32)		1 (0-2)	
2006	Guatemala									29 (9-47)		1 (0-2)		10 (3-16)		1 (0-1)	
2007	Guatemala									30 (10-49)		2 (1-3)		10 (3-17)		1 (0-1)	
2008	Guatemala									23 (8-38)		1 (0-2)		8 (3-13)		0 (0-1)	
2009	Guatemala									24 (8-40)		1 (0-2)		8 (3-13)		0 (0-1)	
2010	Guatemala									3 (1-5)		0 (0-0)		1 (0-2)		0 (0-0)	
2011	Guatemala									3 (1-5)		0 (0-0)		1 (0-2)		0 (0-0)	
2012	Guatemala									3 (1-5)		0 (0-0)		1 (0-2)		0 (0-0)	
2013	Guatemala									3 (1-5)		0 (0-0)		1 (0-2)		0 (0-0)	
2014	Guatemala									3 (1-5)		0 (0-0)		1 (0-2)		0 (0-0)	
2015	Guatemala	162 (148-265)	1 (0-1)	8 (7-13)	0 (0-0)	30 (18-61)	0 (0-0)	1 (1-3)	0 (0-0)	3 (0-5)	0 (0-0)	0 (0-0)	0 (0-0)	1 (0-2)	0 (0-0)	0 (0-0)	0 (0-0)
2000	Guinea	22998 (20998-37577)		1520 (1388-2483)		4342 (2600-8764)		287 (172-579)		665 (288-1007)		44 (19-67)		114 (50-173)		8 (3-11)	
2001	Guinea									681 (301-1025)		44 (20-67)		117 (52-177)		8 (3-11)	
2002	Guinea									700 (315-1047)		45 (20-67)		120 (54-180)		8 (3-12)	
2003	Guinea									726 (220-1190)		46 (14-75)		125 (38-205)		8 (2-13)	
2004	Guinea									762 (238-1241)		48 (15-78)		131 (41-214)		8 (3-13)	
2005	Guinea									792 (256-1280)		49 (16-79)		136 (44-220)		8 (3-14)	
2006	Guinea									810 (261-1311)		49 (16-79)		139 (45-226)		8 (3-14)	
2007	Guinea									814 (261-1320)		48 (15-78)		140 (45-227)		8 (3-13)	
2008	Guinea									782 (250-1270)		45 (14-74)		135 (43-219)		8 (2-13)	
2009	Guinea									349 (111-567)		20 (6-32)		60 (19-98)		3 (1-6)	
2010	Guinea									95 (30-155)		5 (2-9)		16 (5-27)		1 (0-1)	
2011	Guinea									94 (29-153)		5 (2-8)		16 (5-26)		1 (0-1)	
2012	Guinea									93 (29-151)		5 (2-8)		16 (5-26)		1 (0-1)	
2013	Guinea									91 (28-149)		5 (1-8)		16 (5-26)		1 (0-1)	
2014	Guinea									105 (33-172)		5 (2-9)		18 (6-30)		1 (0-2)	
2015	Guinea	3453 (3153-5642)	26 (23-42)	172 (157-281)	1 (1-0)	646 (387-1303)	5 (3-10)	32 (19-65)	0 (0-0)	104 (32-170)	1 (0-1)	5 (2-8)	0 (0-0)	18 (5-29)	0 (0-0)	1 (0-1)	0 (0-0)
2000	Guinea-Bissau	4025 (3675-6576)		1860 (1698-3039)		758 (454-1530)		350 (210-707)		146 (79-207)		67 (36-96)		25 (14-36)		12 (6-17)	
2001	Guinea-Bissau									151 (81-215)		69 (37-98)		26 (14-37)		12 (6-17)	
2002	Guinea-Bissau									155 (83-222)		70 (37-100)		27 (14-38)		12 (6-17)	
2003	Guinea-Bissau									160 (85-229)		71 (37-101)		28 (15-39)		12 (6-17)	
2004	Guinea-Bissau									163 (85-234)		71 (37-102)		28 (15-40)		12 (6-18)	
2005	Guinea-Bissau									164 (61-259)		70 (26-111)		28 (11-45)		12 (5-19)	
2006	Guinea-Bissau									162 (60-255)		68 (25-107)		28 (10-44)		12 (4-18)	
2007	Guinea-Bissau									156 (57-247)		64 (23-102)		27 (10-42)		11 (4-18)	
2008	Guinea-Bissau									147 (53-233)		60 (21-95)		25 (9-40)		10 (4-16)	
2009	Guinea-Bissau									23 (8-36)		9 (3-14)		4 (1-6)		2 (1-2)	
2010	Guinea-Bissau									12 (4-20)		5 (2-8)		2 (1-3)		1 (0-1)	
2011	Guinea-Bissau									12 (4-18)		4 (1-7)		2 (1-3)		1 (0-1)	
2012	Guinea-Bissau									11 (3-17)		4 (1-6)		2 (1-3)		1 (0-1)	
2013	Guinea-Bissau									10 (3-16)		4 (1-6)		2 (1-3)		1 (0-1)	
2014	Guinea-Bissau									1 (0-2)		0 (0-1)		0 (0-0)		0 (0-0)	
2015	Guinea-Bissau	33 (31-55)	2 (2-3)	12 (11-19)	1 (1-0)	6 (4-13)	0 (0-1)	2 (1-4)	0 (0-0)	1 (0-2)	0 (0-0)	0 (0-1)	0 (0-0)	0 (0-0)	0 (0-0)	0 (0-0)	0 (0-0)
2000	Guyana	1123 (1026-1836)		1181 (1079-1930)		211 (126-426)		222 (133-448)		7 (2-10)		7 (3-11)		2 (1-3)		2 (1-4)	
2001	Guyana									3 (1-4)		3 (1-5)		1 (0-1)		1 (0-2)	
2002	Guyana									1 (0-1)		1 (0-2)		0 (0-0)		0 (0-1)	
2003	Guyana									1 (0-2)		1 (0-2)		0 (0-1)		0 (0-1)	
2004	Guyana									1 (0-2)		1 (0-2)		0 (0-1)		0 (0-1)	
2005	Guyana									1 (0-2)		1 (0-2)		0 (0-1)		0 (0-1)	
2006	Guyana									0 (0-0)		0 (0-0)		0 (0-0)		0 (0-0)	
2007	Guyana									0 (0-0)		0 (0-0)		0 (0-0)		0 (0-0)	
2008	Guyana									0 (0-0)		0 (0-0)		0 (0-0)		0 (0-0)	
2009	Guyana									0 (0-0)		0 (0-0)		0 (0-0)		0 (0-0)	
2010	Guyana									0 (0-0)		0 (0-0)		0 (0-0)		0 (0-0)	
2011	Guyana									0 (0-0)		0 (0-0)		0 (0-0)		0 (0-0)	
2012	Guyana									0 (0-0)		0 (0-0)		0 (0-0)		0 (0-0)	
2013	Guyana									0 (0-0)		0 (0-1)		0 (0-0)		0 (0-0)	
2014	Guyana									0 (0-0)		0 (0-1)		0 (0-0)		0 (0-0)	
2015	Guyana	7 (7-12)	0 (0-0)	11 (10-18)	0 (0-0)	1 (1-3)	0 (0-0)	2 (1-4)	0 (0-0)	0 (0-0)	0 (0-0)	0 (0-1)	0 (0-0)	0 (0-0)	0 (0-0)	0 (0-0)	0 (0-0)
2000	Haiti	19010 (17357-31061)		1586 (1448-2592)		3515 (2105-7094)		293 (176-592)		538 (133-903)		45 (11-75)		93 (23-156)		8 (2-13)	
2001	Haiti									520 (129-872)		43 (11-72)		90 (22-150)		7 (2-12)	
2002	Haiti									502 (126-841)		42 (10-70)		86 (22-145)		7 (2-12)	
2003	Haiti									485 (122-811)		40 (10-67)		83 (21-140)		7 (2-12)	
2004	Haiti									472 (120-789)		39 (10-65)		81 (21-136)		7 (2-11)	
2005	Haiti									465 (119-776)		39 (10-64)		80 (21-134)		7 (2-11)	
2006	Haiti									461 (118-771)		38 (10-64)		79 (20-133)		7 (2-11)	
2007	Haiti									459 (119-765)		38 (10-63)		79 (20-132)		7 (2-11)	
2008	Haiti									456 (119-760)		37 (10-62)		79 (20-131)		6 (2-11)	
2009	Haiti									452 (119-753)		37 (10-62)		78 (20-130)		6 (2-11)	
2010	Haiti									1412 (574-2202)		115 (47-179)		243 (99-379)		20 (8-31)	
2011	Haiti									443 (119-736)		36 (10-60)		76 (20-127)		6 (2-10)	
2012	Haiti									439 (119-729)		36 (10-59)		76 (20-125)		6 (2-10)	
2013	Haiti									134 (32-227)		11 (3-19)		45 (11-77)		4 (1-6)	

Hib = *Haemophilus influenzae* type b; npnm = non-pneumonia, non-meningitis; HIV = cases only in HIV-infected children; Uncertainty range provided in parentheses.

Year	Country	Hib pneumonia cases (UR)	Hib pneumonia cases, HIV (UR)	Hib pneumonia incidence rate (UR)	Hib pneumonia incidence rate, HIV (UR)	Hib severe pneumonia cases (UR)	Hib severe pneumonia cases, HIV (UR)	Hib severe pneumonia incidence rate (UR)	Hib severe pneumonia incidence rate, HIV (UR)	Hib meningitis cases (UR)	Hib meningitis cases, HIV (UR)	Hib meningitis incidence rate (UR)	Hib meningitis incidence rate, HIV (UR)	Hib NPNM cases (UR)	Hib NPNM cases, HIV (UR)	Hib NPNM incidence rate (UR)	Hib NPNM incidence rate, HIV (UR)
2014	Haiti									65 (16-109)		5 (1-9)		22 (5-37)		2 (0-3)	
2015	Haiti	1739 (1588-2841)	11 (10-17)	143 (130-233)	1 (1-0)	324 (194-655)	2 (1-4)	27 (16-54)	0 (0-0)	54 (13-92)	0 (0-1)	4 (1-8)	0 (0-0)	18 (4-31)	0 (0-0)	2 (0-3)	0 (0-0)
2000	Honduras	1433 (1309-2342)		153 (140-250)		269 (161-544)		29 (17-58)		21 (6-35)		2 (1-4)		7 (2-12)		1 (0-1)	
2001	Honduras									9 (3-16)		1 (0-2)		3 (1-5)		0 (0-1)	
2002	Honduras									9 (2-15)		1 (0-2)		3 (1-5)		0 (0-1)	
2003	Honduras									9 (2-14)		1 (0-2)		3 (1-5)		0 (0-1)	
2004	Honduras									8 (2-13)		1 (0-1)		3 (1-4)		0 (0-0)	
2005	Honduras									1 (0-2)		0 (0-0)		0 (0-1)		0 (0-0)	
2006	Honduras									1 (0-2)		0 (0-0)		0 (0-1)		0 (0-0)	
2007	Honduras									1 (0-2)		0 (0-0)		0 (0-1)		0 (0-0)	
2008	Honduras									1 (0-2)		0 (0-0)		0 (0-1)		0 (0-0)	
2009	Honduras									1 (0-2)		0 (0-0)		0 (0-1)		0 (0-0)	
2010	Honduras									1 (0-2)		0 (0-0)		0 (0-1)		0 (0-0)	
2011	Honduras									1 (0-2)		0 (0-0)		0 (0-1)		0 (0-0)	
2012	Honduras									1 (0-2)		0 (0-0)		0 (0-1)		0 (0-0)	
2013	Honduras									1 (0-1)		0 (0-0)		0 (0-0)		0 (0-0)	
2014	Honduras									1 (0-1)		0 (0-0)		0 (0-0)		0 (0-0)	
2015	Honduras	63 (57-103)	0 (0-0)	8 (7-13)	0 (0-0)	12 (7-24)	0 (0-0)	1 (1-3)	0 (0-0)	1 (0-1)	0 (0-0)	0 (0-0)	0 (0-0)	0 (0-0)	0 (0-0)	0 (0-0)	0 (0-0)
2000	Hungary	86 (79-141)		18 (16-29)		27 (16-54)		6 (3-11)		4 (2-7)		1 (0-2)		1 (1-2)		0 (0-1)	
2001	Hungary									7 (3-12)		1 (1-3)		2 (1-4)		0 (0-1)	
2002	Hungary									4 (2-8)		1 (0-2)		1 (1-3)		0 (0-1)	
2003	Hungary									3 (1-6)		1 (0-1)		1 (0-2)		0 (0-0)	
2004	Hungary									1 (0-2)		0 (0-0)		0 (0-1)		0 (0-0)	
2005	Hungary									1 (1-2)		0 (0-0)		0 (0-1)		0 (0-0)	
2006	Hungary									1 (0-2)		0 (0-0)		0 (0-1)		0 (0-0)	
2007	Hungary									1 (0-1)		0 (0-0)		0 (0-0)		0 (0-0)	
2008	Hungary									1 (0-1)		0 (0-0)		0 (0-0)		0 (0-0)	
2009	Hungary									1 (0-1)		0 (0-0)		0 (0-0)		0 (0-0)	
2010	Hungary									0 (0-1)		0 (0-0)		0 (0-0)		0 (0-0)	
2011	Hungary									0 (0-1)		0 (0-0)		0 (0-0)		0 (0-0)	
2012	Hungary									0 (0-1)		0 (0-0)		0 (0-0)		0 (0-0)	
2013	Hungary									1 (0-2)		0 (0-0)		0 (0-1)		0 (0-0)	
2014	Hungary									1 (0-1)		0 (0-0)		0 (0-0)		0 (0-0)	
2015	Hungary	14 (12-22)	0 (0-0)	3 (3-5)	0 (0-0)	4 (3-9)	0 (0-0)	1 (1-2)	0 (0-0)	1 (0-1)	0 (0-0)	0 (0-0)	0 (0-0)	0 (0-0)	0 (0-0)	0 (0-0)	0 (0-0)
2000	Iceland	0 (0-0)		1 (1-1)		0 (0-0)		0 (0-0)		0 (0-0)		0 (0-0)		0 (0-0)		0 (0-0)	
2001	Iceland									0 (0-0)		0 (0-0)		0 (0-0)		0 (0-0)	
2002	Iceland									0 (0-0)		0 (0-0)		0 (0-0)		0 (0-0)	
2003	Iceland									0 (0-0)		0 (0-0)		0 (0-0)		0 (0-0)	
2004	Iceland									0 (0-0)		0 (0-0)		0 (0-0)		0 (0-0)	
2005	Iceland									0 (0-0)		0 (0-0)		0 (0-0)		0 (0-0)	
2006	Iceland									0 (0-0)		0 (0-0)		0 (0-0)		0 (0-0)	
2007	Iceland									0 (0-0)		0 (0-0)		0 (0-0)		0 (0-0)	
2008	Iceland									0 (0-0)		0 (0-0)		0 (0-0)		0 (0-0)	
2009	Iceland									0 (0-0)		0 (0-0)		0 (0-0)		0 (0-0)	
2010	Iceland									0 (0-0)		0 (0-0)		0 (0-0)		0 (0-0)	
2011	Iceland									0 (0-0)		0 (0-0)		0 (0-0)		0 (0-0)	
2012	Iceland									0 (0-0)		0 (0-0)		0 (0-0)		0 (0-0)	
2013	Iceland									0 (0-0)		0 (0-0)		0 (0-0)		0 (0-0)	
2014	Iceland									0 (0-0)		0 (0-0)		0 (0-0)		0 (0-0)	
2015	Iceland	0 (0-0)	0 (0-0)	1 (1-1)	0 (0-0)	0 (0-0)	0 (0-0)	0 (0-0)	0 (0-0)	0 (0-0)	0 (0-0)	0 (0-0)	0 (0-0)	0 (0-0)	0 (0-0)	0 (0-0)	0 (0-0)
2000	India	2194365 (2003502-3585380)		1748 (1596-2856)		413952 (247853-835511)		330 (197-666)		59618 (26086-89933)		47 (21-72)		12380 (5399-18701)		10 (4-15)	
2001	India									57238 (24918-86475)		45 (20-68)		12515 (5420-18943)		10 (4-15)	
2002	India									54791 (23846-82783)		43 (19-65)		11955 (5175-18098)		9 (4-14)	
2003	India									52518 (22764-79462)		41 (18-62)		11796 (5066-17900)		9 (4-14)	
2004	India									50278 (21738-76147)		39 (17-60)		11494 (4924-17463)		9 (4-14)	
2005	India									48079 (20891-72660)		38 (16-57)		10991 (4746-16644)		9 (4-13)	
2006	India									46197 (20007-69838)		36 (16-55)		11605 (5023-17553)		9 (4-14)	
2007	India									43963 (19171-66292)		34 (15-52)		11215 (4894-16910)		9 (4-13)	
2008	India									41714 (18191-62945)		33 (14-49)		10645 (4646-16065)		8 (4-13)	
2009	India									39353 (17055-59543)		31 (13-47)		10510 (4556-15910)		8 (4-13)	
2010	India									37076 (16059-56138)		29 (13-44)		9880 (4281-14972)		8 (3-12)	
2011	India									34914 (14588-53338)		28 (12-42)		11022 (4613-16837)		9 (4-13)	
2012	India									31591 (12956-48298)		25 (10-39)		9928 (4074-15178)		8 (3-12)	
2013	India									27280 (11040-41933)		22 (9-34)		8938 (3618-13740)		7 (3-11)	
2014	India									24482 (9760-37652)		20 (8-31)		7995 (3187-12296)		7 (3-10)	
2015	India	320583 (292699-523802)	862 (787-1408)	264 (241-431)	1 (1-0)	180176 (107880-363664)	455 (273-919)	148 (89-299)	0 (0-0)	12503 (4919-19308)	34 (13-52)	10 (4-16)	0 (0-0)	4184 (1646-6462)	11 (4-17)	3 (1-5)	0 (0-0)
2000	Indonesia	498126 (454800-813890)		2377 (2171-3885)		93565 (56022-188850)		447 (267-901)		4133 (1414-6604)		20 (7-32)		1393 (477-2226)		7 (2-11)	
2001	Indonesia									3922 (1352-6255)		19 (6-30)		1322 (456-2109)		6 (2-10)	
2002	Indonesia									3740 (1298-5955)		18 (6-28)		1261 (438-2007)		6 (2-9)	
2003	Indonesia									3551 (1233-5656)		17 (6-26)		1197 (416-1907)		6 (2-9)	
2004	Indonesia									4110 (1446-6529)		19 (7-30)		1385 (487-2201)		6 (2-10)	
2005	Indonesia									3223 (1149-5107)		15 (5-23)		1087 (387-1721)		5 (2-8)	
2006	Indonesia									3093 (1118-4888)		14 (5-22)		1043 (377-1648)		5 (2-7)	
2007	Indonesia									2957 (1083-4660)		13 (5-21)		997 (365-1571)		5 (2-7)	
2008	Indonesia									2827 (1057-4435)		13 (5-20)		953 (356-1495)		4 (2-7)	
2009	Indonesia									2744 (1048-4284)		12 (5-19)		925 (353-1444)		4 (2-6)	
2010	Indonesia									2761 (1079-4295)		12 (5-19)		931 (364-1448)		4 (2-6)	
2011	Indonesia									2869 (1150-4453)		13 (5-20)		967 (388-1501)		4 (2-7)	
2012	Indonesia									3057 (1260-4741)		13 (5-21)		1030 (425-1598)		4 (2-7)	
2013	Indonesia									3246 (590-5341)		14 (2-23)		1094 (199-1800)		5 (1-8)	
2014	Indonesia									1419 (262-2347)		6 (1-10)		478 (88-791)		2 (0-3)	
2015	Indonesia	36607 (33423-59813)	145 (133-237)	150 (137-245)	1 (1-0)	6905 (4134-13936)	27 (16-55)	28 (17-57)	0 (0-0)	341 (64-566)	1 (0-2)	1 (0-2)	0 (0-0)	115 (22-191)	0 (0-1)	0 (0-1)	0 (0-0)
2000	Iran (Islamic Republic of)	92361 (84327-150909)		1472 (1344-2406)		17402 (10420-35125)		277 (166-560)		1063 (565-1548)		17 (9-25)		358 (190-522)		6 (3-8)	
2001	Iran (Islamic Republic of)									973 (507-1420)		16 (8-23)		328 (171-479)		5 (3-8)	
2002	Iran (Islamic Republic of)									898 (461-1337)		15 (8-23)		303 (155-451)		5 (3-8)	
2003	Iran (Islamic Republic of)									830 (215-1283)		15 (4-23)		280 (73-433)		5 (1-8)	
2004	Iran (Islamic Republic of)									764 (190-1215)		14 (3-22)		257 (64-409)		5 (1-7)	
2005	Iran (Islamic Republic of)									681 (162-1105)		13 (3-20)		230 (54-372)		4 (1-7)	
2006	Iran (Islamic Republic of)									610 (138-1002)		11 (2-18)		206 (46-338)		4 (1-6)	
2007	Iran (Islamic Republic of)									543 (116-895)		9 (2-16)		183 (39-302)		3 (1-5)	
2008	Iran (Islamic Republic of)									493 (100-817)		8 (2-14)		166 (34-276)		3 (1-5)	
2009	Iran (Islamic Republic of)									452 (88-755)		7 (1-12)		152 (30-254)		2 (0-4)	
2010	Iran (Islamic Republic of)									428 (80-718)		7 (1-11)		144 (27-242)		2 (0-4)	
2011	Iran (Islamic Republic of)									405 (75-679)		6 (1-10)		136 (25-229)		2 (0-4)	
2012	Iran (Islamic Republic of)									391 (74-654)		6 (1-10)		132 (25-220)		2 (0-3)	
2013	Iran (Islamic Republic of)									381 (72-637)		6 (1-9)		129 (24-215)		2 (0-3)	
2014	Iran (Islamic Republic of)									55 (11-92)		1 (0-1)		19 (4-31)		0 (0-0)	
2015	Iran (Islamic Republic of)	3229 (2948-5275)	1 (1-2)	48 (44-78)	0 (0-0)	603 (361-1216)	0 (0-0)	9 (5-18)	0 (0-0)	24 (5-39)	0 (0-0)	0 (0-1)	0 (0-0)	8 (2-13)	0 (0-0)	0 (0-0)	0 (0-0)
2000	Iraq	35969 (32841-58770)		952 (870-1556)		6704 (4014-13532)		178 (106-358)		578 (239-888)		15 (6-24)		195 (80-299)		5 (2-8)	
2001	Iraq									581 (243-890)		15 (6-23)		196 (82-300)		5 (2-8)	
2002	Iraq									588 (249-897)		15 (6-23)		198 (84-302)		5 (2-8)	
2003	Iraq									600 (257-911)		15 (6-23)		202 (87-307)		5 (2-8)	
2004	Iraq									604 (262-914)		15 (6-22)		204 (88-308)		5 (2-8)	
2005	Iraq									609 (268-917)		15 (6-22)		205 (90-309)		5 (2-7)	
2006	Iraq									603 (269-903)		14 (6-21)		203 (91-305)		5 (2-7)	
2007	Iraq									600 (262-905)		14 (6-21)		202 (88-305)		5 (2-7)	
2008	Iraq									596 (255-905)		13 (6-20)		201 (86-305)		4 (2-7)	
2009	Iraq									586 (246-894)		12 (5-19)		197 (83-301)		4 (2-6)	
2010	Iraq									567 (234-871)		12 (5-18)		191 (79-294)		4 (2-6)	
2011	Iraq									536 (217-826)		11 (4-16)		181 (73-279)		4 (1-6)	
2012	Iraq									361 (143-559)		7 (3-11)		122 (48-188)		2 (1-4)	
2013	Iraq									59 (23-92)		1 (0-2)		20 (8-31)		0 (0-1)	
2014	Iraq									60 (23-93)		1 (0-2)		20 (8-31)		0 (0-1)	
2015	Iraq	7000 (6391-11438)	0 (0-0)	124 (113-203)	0 (0-0)	1319 (789-2661)	0 (0-0)	23 (14-47)	0 (0-0)	63 (24-99)	0 (0-0)	1 (0-2)	0 (0-0)	21 (8-33)	0 (0-0)	0 (0-1)	0 (0-0)
2000	Ireland	18 (16-29)		7 (6-11)		5 (3-11)		2 (1-4)		12 (5-22)		5 (2-8)		4 (2-7)		2 (1-3)	
2001	Ireland									7 (3-13)		3 (1-5)		2 (1-4)		1 (0-2)	
2002	Ireland									1 (0-2)		0 (0-1)		0 (0-1)		0 (0-0)	
2003	Ireland									1 (0-1)		0 (0-1)		0 (0-1)		0 (0-0)	
2004	Ireland									1 (0-1)		0 (0-1)		0 (0-1)		0 (0-0)	
2005	Ireland									1 (0-2)		0 (0-1)		0 (0-1)		0 (0-0)	
2006	Ireland									0 (0-1)		0 (0-0)		0 (0-0)		0 (0-0)	
2007	Ireland									1 (0-1)		0 (0-0)		0 (0-0)		0 (0-0)	
2008	Ireland									1 (0-1)		0 (0-0)		0 (0-0)		0 (0-0)	
2009	Ireland									0 (0-1)		0 (0-0)		0 (0-0)		0 (0-0)	
2010	Ireland									0 (0-1)		0 (0-0)		0 (0-0)		0 (0-0)	
2011	Ireland									0 (0-1)		0 (0-0)		0 (0-0)		0 (0-0)	
2012	Ireland									0 (0-1)		0 (0-0)		0 (0-0)		0 (0-0)	
2013	Ireland									0 (0-1)		0 (0-0)		0 (0-0)		0 (0-0)	
2014	Ireland									0 (0-1)		0 (0-0)		0 (0-0)		0 (0-0)	
2015	Ireland	3 (2-4)	0 (0-0)	1 (1-1)	0 (0-0)	1 (0-2)	0 (0-0)	0 (0-0)	0 (0-0)	0 (0-1)	0 (0-0)	0 (0-0)	0 (0-0)	0 (0-0)	0 (0-0)	0 (0-0)	0 (0-0)
2000	Israel	4 (4-7)		1 (1-1)		1 (1-3)		0 (0-0)		0 (0-0)		0 (0-0)		0 (0-0)		0 (0-0)	
2001	Israel									0 (0-1)		0 (0-0)		0 (0-0)		0 (0-0)	
2002	Israel									1 (1-2)		0 (0-0)		0 (0-1)		0 (0-0)	
2003	Israel									1 (0-1)		0 (0-0)		0 (0-0)		0 (0-0)	
2004	Israel									0 (0-1)		0 (0-0)		0 (0-0)		0 (0-0)	
2005	Israel									0 (0-1)		0 (0-0)		0 (0-0)		0 (0-0)	
2006	Israel									1 (1-2)		0 (0-0)		0 (0-1)		0 (0-0)	
2007	Israel									0 (0-1)		0 (0-0)		0 (0-0)		0 (0-0)	
2008	Israel									1 (0-1)		0 (0-0)		0 (0-0)		0 (0-0)	
2009	Israel									1 (0-1)		0 (0-0)		0 (0-0)		0 (0-0)	
2010	Israel									0 (0-1)		0 (0-0)		0 (0-0)		0 (0-0)	
2011	Israel									1 (0-1)		0 (0-0)		0 (0-0)		0 (0-0)	
2012	Israel									1 (0-1)		0 (0-0)		0 (0-0)		0 (0-0)	
2013	Israel									1 (0-1)		0 (0-0)		0 (0-0)		0 (0-0)	
2014	Israel									1 (0-1)		0 (0-0)		0 (0-0)		0 (0-0)	
2015	Israel	6 (6-10)	0 (0-0)	1 (1-1)	0 (0-0)	2 (1-4)	0 (0-0)	0 (0-0)	0 (0-0)	1 (0-1)	0 (0-0)	0 (0-0)	0 (0-0)	0 (0-0)	0 (0-0)	0 (0-0)	0 (0-0)
2000	Italy	906 (827-1480)		35 (32-57)		281 (168-568)		11 (7-22)		41 (18-76)		2 (1-3)		14 (6-26)		1 (0-1)	
2001	Italy									12 (5-23)		0 (0-1)		4 (2-8)		0 (0-0)	
2002	Italy									10 (5-19)		0 (0-1)		4 (2-6)		0 (0-0)	
2003	Italy									14 (6-27)		1 (0-1)		5 (2-9)		0 (0-0)	
2004	Italy									9 (4-16)		0 (0-1)		3 (1-5)		0 (0-0)	
2005	Italy									1 (1-2)		0 (0-0)		0 (0-1)		0 (0-0)	
2006	Italy									2 (1-3)		0 (0-0)		1 (0-1)		0 (0-0)	
2007	Italy									1 (1-2)		0 (0-0)		0 (0-1)		0 (0-0)	
2008	Italy									1 (0-2)		0 (0-0)		0 (0-1)		0 (0-0)	
2009	Italy									1 (1-3)		0 (0-0)		0 (0-1)		0 (0-0)	
2010	Italy									1 (1-2)		0 (0-0)		0 (0-1)		0 (0-0)	
2011	Italy									1 (0-1)		0 (0-0)		0 (0-0)		0 (0-0)	
2012	Italy									1 (0-2)		0 (0-0)		0 (0-1)		0 (0-0)	
2013	Italy									1 (0-2)		0 (0-0)		0 (0-1)		0 (0-0)	
2014	Italy									1 (0-2)		0 (0-0)		0 (0-1)		0 (0-0)	
2015	Italy	19 (17-31)	0 (0-0)	1 (1-1)	0 (0-0)	6 (3-12)	0 (0-0)	0 (0-0)	0 (0-0)	1 (0-2)	0 (0-0)	0 (0-0)	0 (0-0)	0 (0-1)	0 (0-0)	0 (0-0)	0 (0-0)
2000	Jamaica	2640 (2410-4313)		940 (858-1535)		493 (295-994)		175 (105-354)		3 (0-5)		1 (0-2)		1 (0-2)		0 (0-1)	
2001	Jamaica									3 (0-5)		1 (0-2)		1 (0-2)		0 (0-1)	
2002	Jamaica									3 (0-5)		1 (0-2)		1 (0-2)		0 (0-1)	
2003	Jamaica									3 (0-6)		1 (0-2)		1 (0-2)		0 (0-1)	
2004	Jamaica									1 (0-1)		0 (0-0)		0 (0-0)		0 (0-0)	
2005	Jamaica									1 (0-1)		0 (0-0)		0 (0-0)		0 (0-0)	
2006	Jamaica									1 (0-1)		0 (0-1)		0 (0-0)		0 (0-0)	

Hib = *Haemophilus influenzae* type b; npnm = non-pneumonia, non-meningitis; HIV = cases only in HIV-infected children; Uncertainty range provided in parentheses.

Year	Country	Hib pneumonia cases (UR)	Hib pneumonia cases, HIV (UR)	Hib pneumonia incidence rate (UR)	Hib pneumonia incidence rate, HIV (UR)	Hib severe pneumonia cases (UR)	Hib severe pneumonia cases, HIV (UR)	Hib severe pneumonia incidence rate (UR)	Hib severe pneumonia incidence rate, HIV (UR)	Hib meningitis cases (UR)	Hib meningitis cases, HIV (UR)	Hib meningitis incidence rate (UR)	Hib meningitis incidence rate, HIV (UR)	Hib NPNM cases (UR)	Hib NPNM cases, HIV (UR)	Hib NPNM incidence rate (UR)	Hib NPNM incidence rate, HIV (UR)
2007	Jamaica									1 (0-2)		0 (0-1)		0 (0-1)		0 (0-0)	
2008	Jamaica									0 (0-0)		0 (0-0)		0 (0-0)		0 (0-0)	
2009	Jamaica									0 (0-0)		0 (0-0)		0 (0-0)		0 (0-0)	
2010	Jamaica									0 (0-0)		0 (0-0)		0 (0-0)		0 (0-0)	
2011	Jamaica									0 (0-0)		0 (0-0)		0 (0-0)		0 (0-0)	
2012	Jamaica									0 (0-0)		0 (0-0)		0 (0-0)		0 (0-0)	
2013	Jamaica									0 (0-0)		0 (0-0)		0 (0-0)		0 (0-0)	
2014	Jamaica									0 (0-0)		0 (0-0)		0 (0-0)		0 (0-0)	
2015	Jamaica	12 (11-20)	0 (0-0)	6 (5-10)	0 (0-0)	2 (1-5)	0 (0-0)	1 (1-2)	0 (0-0)	0 (0-0)	0 (0-0)	0 (0-0)	0 (0-0)	0 (0-0)	0 (0-0)	0 (0-0)	0 (0-0)
2000	Japan	7144 (6523-11672)		124 (113-203)		2219 (1329-4479)		39 (23-78)		289 (147-483)		5 (3-8)		97 (49-163)		2 (1-3)	
2001	Japan									257 (130-429)		4 (2-7)		87 (44-145)		2 (1-3)	
2002	Japan									196 (99-327)		3 (2-6)		66 (33-110)		1 (1-2)	
2003	Japan									234 (119-391)		4 (2-7)		79 (40-132)		1 (1-2)	
2004	Japan									180 (91-301)		3 (2-5)		61 (31-101)		1 (1-2)	
2005	Japan									199 (101-332)		4 (2-6)		67 (34-112)		1 (1-2)	
2006	Japan									154 (78-257)		3 (1-5)		52 (26-87)		1 (0-2)	
2007	Japan									199 (101-332)		4 (2-6)		67 (34-112)		1 (1-2)	
2008	Japan									114 (58-191)		2 (1-4)		39 (20-64)		1 (0-1)	
2009	Japan									153 (77-255)		3 (1-5)		51 (26-86)		1 (0-2)	
2010	Japan									210 (107-351)		4 (2-7)		71 (36-118)		1 (1-2)	
2011	Japan									89 (45-149)		2 (1-3)		30 (15-50)		1 (0-1)	
2012	Japan									70 (35-116)		1 (1-2)		23 (12-39)		0 (0-1)	
2013	Japan									77 (39-129)		1 (1-2)		26 (13-43)		0 (0-1)	
2014	Japan									72 (36-119)		1 (1-2)		24 (12-40)		0 (0-1)	
2015	Japan	983 (898-1607)	0 (0-0)	19 (17-31)	0 (0-0)	305 (183-616)	0 (0-0)	6 (4-12)	0 (0-0)	19 (9-31)	0 (0-0)	0 (0-1)	0 (0-0)	6 (3-11)	0 (0-0)	0 (0-0)	0 (0-0)
2000	Jordan	9546 (8715-15597)		1399 (1277-2286)		1773 (1062-3579)		260 (156-524)		42 (8-69)		6 (1-10)		14 (3-23)		2 (0-3)	
2001	Jordan									27 (5-44)		4 (1-6)		9 (2-15)		1 (0-2)	
2002	Jordan									5 (1-9)		1 (0-1)		2 (0-3)		0 (0-0)	
2003	Jordan									5 (1-8)		1 (0-1)		2 (0-3)		0 (0-0)	
2004	Jordan									5 (1-8)		1 (0-1)		2 (0-3)		0 (0-0)	
2005	Jordan									5 (1-8)		1 (0-1)		2 (0-3)		0 (0-0)	
2006	Jordan									1 (0-1)		0 (0-0)		0 (0-0)		0 (0-0)	
2007	Jordan									1 (0-1)		0 (0-0)		0 (0-0)		0 (0-0)	
2008	Jordan									1 (0-1)		0 (0-0)		0 (0-0)		0 (0-0)	
2009	Jordan									1 (0-1)		0 (0-0)		0 (0-0)		0 (0-0)	
2010	Jordan									1 (0-1)		0 (0-0)		0 (0-0)		0 (0-0)	
2011	Jordan									1 (0-1)		0 (0-0)		0 (0-0)		0 (0-0)	
2012	Jordan									1 (0-1)		0 (0-0)		0 (0-0)		0 (0-0)	
2013	Jordan									1 (0-1)		0 (0-0)		0 (0-0)		0 (0-0)	
2014	Jordan									1 (0-1)		0 (0-0)		0 (0-0)		0 (0-0)	
2015	Jordan	67 (61-109)	0 (0-0)	7 (6-11)	0 (0-0)	12 (7-25)	0 (0-0)	1 (1-3)	0 (0-0)	1 (0-1)	0 (0-0)	0 (0-0)	0 (0-0)	0 (0-0)	0 (0-0)	0 (0-0)	0 (0-0)
2000	Kazakhstan	3270 (2986-5343)		298 (272-486)		1016 (608-2050)		92 (55-187)		164 (60-259)		15 (5-24)		55 (20-87)		5 (2-8)	
2001	Kazakhstan									154 (57-242)		14 (5-23)		52 (19-82)		5 (2-8)	
2002	Kazakhstan									150 (56-235)		14 (5-22)		50 (19-79)		5 (2-7)	
2003	Kazakhstan									147 (55-230)		13 (5-21)		50 (19-78)		4 (2-7)	
2004	Kazakhstan									147 (56-230)		13 (5-20)		50 (19-78)		4 (2-7)	
2005	Kazakhstan									152 (59-239)		12 (5-20)		51 (20-81)		4 (2-7)	
2006	Kazakhstan									160 (63-252)		12 (5-19)		54 (21-85)		4 (2-7)	
2007	Kazakhstan									171 (30-284)		13 (2-21)		58 (10-96)		4 (1-7)	
2008	Kazakhstan									100 (18-165)		7 (1-12)		34 (6-56)		2 (0-4)	
2009	Kazakhstan									13 (3-22)		1 (0-1)		4 (1-7)		0 (0-0)	
2010	Kazakhstan									14 (3-23)		1 (0-1)		5 (1-8)		0 (0-0)	
2011	Kazakhstan									14 (3-22)		1 (0-1)		5 (1-8)		0 (0-0)	
2012	Kazakhstan									13 (3-21)		1 (0-1)		4 (1-7)		0 (0-0)	
2013	Kazakhstan									2 (0-3)		0 (0-0)		1 (0-1)		0 (0-0)	
2014	Kazakhstan									2 (0-3)		0 (0-0)		1 (0-1)		0 (0-0)	
2015	Kazakhstan	57 (52-93)	0 (0-0)	3 (3-5)	0 (0-0)	18 (11-36)	0 (0-0)	1 (1-2)	0 (0-0)	1 (0-2)	0 (0-0)	0 (0-0)	0 (0-0)	0 (0-1)	0 (0-0)	0 (0-0)	0 (0-0)
2000	Kenya	99951 (91257-163310)		2007 (1832-3278)		18667 (11177-37676)		375 (224-756)		1900 (640-3056)		38 (13-61)		327 (110-526)		7 (2-11)	
2001	Kenya									1887 (639-3031)		37 (12-59)		325 (110-522)		6 (2-10)	
2002	Kenya									306 (104-491)		6 (2-9)		53 (18-85)		1 (0-2)	
2003	Kenya									203 (70-326)		4 (1-6)		35 (12-56)		1 (0-1)	
2004	Kenya									200 (69-319)		4 (1-6)		34 (12-55)		1 (0-1)	
2005	Kenya									181 (64-288)		3 (1-5)		31 (11-50)		1 (0-1)	
2006	Kenya									167 (60-266)		3 (1-4)		29 (10-46)		0 (0-1)	
2007	Kenya									17 (6-27)		0 (0-0)		3 (1-5)		0 (0-0)	
2008	Kenya									17 (6-28)		0 (0-0)		6 (2-9)		0 (0-0)	
2009	Kenya									17 (6-27)		0 (0-0)		6 (2-9)		0 (0-0)	
2010	Kenya									16 (5-26)		0 (0-0)		5 (2-9)		0 (0-0)	
2011	Kenya									16 (5-26)		0 (0-0)		5 (2-9)		0 (0-0)	
2012	Kenya									16 (6-25)		0 (0-0)		5 (2-9)		0 (0-0)	
2013	Kenya									15 (5-25)		0 (0-0)		5 (2-8)		0 (0-0)	
2014	Kenya									15 (6-24)		0 (0-0)		5 (2-8)		0 (0-0)	
2015	Kenya	787 (718-1286)	20 (18-32)	11 (10-18)	0 (0-0)	148 (88-298)	4 (2-8)	2 (1-4)	0 (0-0)	15 (5-24)	0 (0-1)	0 (0-0)	0 (0-0)	5 (2-8)	0 (0-0)	0 (0-0)	0 (0-0)
2000	Kiribati	125 (114-205)		1065 (973-1741)		23 (14-47)		200 (119-403)		9 (5-12)		78 (44-100)		3 (2-4)		26 (15-34)	
2001	Kiribati									9 (5-11)		72 (41-92)		3 (2-4)		24 (14-31)	
2002	Kiribati									8 (4-10)		68 (38-87)		3 (1-3)		23 (13-29)	
2003	Kiribati									7 (4-9)		62 (35-80)		2 (1-3)		21 (12-27)	
2004	Kiribati									6 (4-8)		60 (34-77)		2 (1-3)		20 (11-26)	
2005	Kiribati									7 (4-9)		63 (36-81)		2 (1-3)		21 (12-27)	
2006	Kiribati									7 (4-9)		71 (40-91)		2 (1-3)		24 (14-31)	
2007	Kiribati									9 (5-11)		80 (45-101)		3 (2-4)		27 (15-34)	
2008	Kiribati									2 (1-2)		14 (8-18)		1 (0-1)		5 (3-6)	
2009	Kiribati									1 (1-1)		8 (4-10)		0 (0-0)		3 (1-3)	
2010	Kiribati									1 (1-1)		7 (4-9)		0 (0-0)		2 (1-3)	
2011	Kiribati									1 (0-1)		6 (3-8)		0 (0-0)		2 (1-3)	
2012	Kiribati									1 (0-1)		6 (3-7)		0 (0-0)		2 (1-2)	
2013	Kiribati									0 (0-0)		1 (0-1)		0 (0-0)		0 (0-0)	
2014	Kiribati									0 (0-0)		1 (0-1)		0 (0-0)		0 (0-0)	
2015	Kiribati	1 (1-2)	0 (0-0)	8 (7-13)	0 (0-0)	0 (0-0)	0 (0-0)	1 (1-3)	0 (0-0)	0 (0-0)	0 (0-0)	1 (0-1)	0 (0-0)	0 (0-0)	0 (0-0)	0 (0-0)	0 (0-0)
2000	Kuwait	188 (172-307)		94 (86-154)		35 (21-72)		18 (11-36)		2 (1-3)		1 (1-2)		1 (0-1)		0 (0-1)	
2001	Kuwait									4 (2-6)		2 (1-3)		1 (1-2)		1 (0-1)	
2002	Kuwait									1 (0-1)		0 (0-1)		0 (0-0)		0 (0-0)	
2003	Kuwait									0 (0-1)		0 (0-0)		0 (0-0)		0 (0-0)	
2004	Kuwait									0 (0-0)		0 (0-0)		0 (0-0)		0 (0-0)	
2005	Kuwait									0 (0-1)		0 (0-0)		0 (0-0)		0 (0-0)	
2006	Kuwait									0 (0-0)		0 (0-0)		0 (0-0)		0 (0-0)	
2007	Kuwait									0 (0-0)		0 (0-0)		0 (0-0)		0 (0-0)	
2008	Kuwait									0 (0-0)		0 (0-0)		0 (0-0)		0 (0-0)	
2009	Kuwait									0 (0-1)		0 (0-0)		0 (0-0)		0 (0-0)	
2010	Kuwait									0 (0-1)		0 (0-0)		0 (0-0)		0 (0-0)	
2011	Kuwait									1 (1-2)		0 (0-1)		0 (0-1)		0 (0-0)	
2012	Kuwait									1 (0-2)		0 (0-0)		0 (0-1)		0 (0-0)	
2013	Kuwait									1 (0-1)		0 (0-0)		0 (0-0)		0 (0-0)	
2014	Kuwait									1 (0-2)		0 (0-0)		0 (0-1)		0 (0-0)	
2015	Kuwait	24 (22-39)	0 (0-0)	7 (6-11)	0 (0-0)	4 (3-9)	0 (0-0)	1 (1-3)	0 (0-0)	1 (0-1)	0 (0-0)	0 (0-0)	0 (0-0)	0 (0-0)	0 (0-0)	0 (0-0)	0 (0-0)
2000	Kyrgyzstan	799 (730-1305)		149 (136-243)		248 (149-501)		46 (28-93)		147 (52-232)		27 (10-43)		50 (18-78)		9 (3-15)	
2001	Kyrgyzstan									134 (48-212)		26 (9-40)		45 (16-71)		9 (3-14)	
2002	Kyrgyzstan									121 (43-192)		24 (8-38)		41 (15-65)		8 (3-13)	
2003	Kyrgyzstan									110 (39-174)		22 (8-35)		37 (13-59)		8 (3-12)	
2004	Kyrgyzstan									101 (35-160)		21 (7-33)		34 (12-54)		7 (2-11)	
2005	Kyrgyzstan									95 (33-150)		20 (7-31)		32 (11-51)		7 (2-11)	
2006	Kyrgyzstan									91 (32-145)		19 (7-29)		31 (11-49)		6 (2-10)	
2007	Kyrgyzstan									90 (31-142)		18 (6-28)		30 (11-48)		6 (2-9)	
2008	Kyrgyzstan									88 (31-140)		16 (6-26)		30 (10-47)		6 (2-9)	
2009	Kyrgyzstan									89 (31-142)		16 (6-25)		30 (10-48)		5 (2-8)	
2010	Kyrgyzstan									14 (5-22)		2 (1-4)		5 (2-7)		1 (0-1)	
2011	Kyrgyzstan									7 (1-11)		1 (0-2)		2 (0-4)		0 (0-1)	
2012	Kyrgyzstan									7 (1-11)		1 (0-2)		2 (0-4)		0 (0-1)	
2013	Kyrgyzstan									6 (1-11)		1 (0-2)		2 (0-4)		0 (0-1)	
2014	Kyrgyzstan									6 (1-11)		1 (0-1)		2 (0-4)		0 (0-0)	
2015	Kyrgyzstan	11 (10-19)	0 (0-0)	1 (1-2)	0 (0-0)	4 (2-7)	0 (0-0)	0 (0-1)	0 (0-0)	1 (0-1)	0 (0-0)	0 (0-0)	0 (0-0)	0 (0-0)	0 (0-0)	0 (0-0)	0 (0-0)
2000	Lao People's Democratic Republic	18434 (16831-30120)		2340 (2137-3823)		3398 (2035-6859)		431 (258-871)		324 (100-519)		41 (13-66)		56 (17-89)		7 (2-11)	
2001	Lao People's Democratic Republic									307 (94-493)		40 (12-64)		53 (16-85)		7 (2-11)	
2002	Lao People's Democratic Republic									291 (89-468)		39 (12-62)		50 (15-81)		7 (2-11)	
2003	Lao People's Democratic Republic									278 (85-447)		37 (11-60)		48 (15-77)		6 (2-10)	
2004	Lao People's Democratic Republic									266 (81-428)		36 (11-58)		46 (14-74)		6 (2-10)	
2005	Lao People's Democratic Republic									257 (77-414)		34 (10-56)		44 (13-71)		6 (2-10)	
2006	Lao People's Democratic Republic									252 (76-407)		34 (10-54)		43 (13-70)		6 (2-9)	
2007	Lao People's Democratic Republic									250 (78-401)		33 (10-53)		43 (13-69)		6 (2-9)	
2008	Lao People's Democratic Republic									252 (81-403)		33 (10-52)		43 (14-69)		6 (2-9)	
2009	Lao People's Democratic Republic									254 (85-403)		32 (11-51)		44 (15-69)		6 (2-9)	
2010	Lao People's Democratic Republic									62 (21-97)		8 (3-12)		11 (4-17)		1 (0-2)	
2011	Lao People's Democratic Republic									26 (9-41)		3 (1-5)		4 (2-7)		1 (0-1)	
2012	Lao People's Democratic Republic									25 (8-40)		3 (1-5)		8 (3-14)		1 (0-2)	
2013	Lao People's Democratic Republic									21 (7-33)		3 (1-4)		7 (2-11)		1 (0-1)	
2014	Lao People's Democratic Republic									20 (6-31)		2 (1-4)		7 (2-11)		1 (0-1)	
2015	Lao People's Democratic Republic	142 (129-232)	0 (0-1)	17 (16-28)	0 (0-0)	27 (16-54)	0 (0-0)	3 (2-7)	0 (0-0)	2 (1-4)	0 (0-0)	0 (0-0)	0 (0-0)	1 (0-1)	0 (0-0)	0 (0-0)	0 (0-0)
2000	Latvia	3 (3-5)		3 (3-5)		1 (1-2)		1 (1-2)		0 (0-1)		0 (0-1)		0 (0-0)		0 (0-0)	
2001	Latvia									0 (0-1)		0 (0-1)		0 (0-0)		0 (0-0)	
2002	Latvia									0 (0-0)		0 (0-0)		0 (0-0)		0 (0-0)	
2003	Latvia									0 (0-1)		0 (0-1)		0 (0-0)		0 (0-0)	
2004	Latvia									0 (0-0)		0 (0-0)		0 (0-0)		0 (0-0)	
2005	Latvia									0 (0-0)		0 (0-0)		0 (0-0)		0 (0-0)	
2006	Latvia									0 (0-0)		0 (0-0)		0 (0-0)		0 (0-0)	
2007	Latvia									0 (0-0)		0 (0-0)		0 (0-0)		0 (0-0)	
2008	Latvia									0 (0-0)		0 (0-0)		0 (0-0)		0 (0-0)	
2009	Latvia									0 (0-0)		0 (0-0)		0 (0-0)		0 (0-0)	
2010	Latvia									0 (0-0)		0 (0-0)		0 (0-0)		0 (0-0)	
2011	Latvia									0 (0-0)		0 (0-0)		0 (0-0)		0 (0-0)	
2012	Latvia									0 (0-0)		0 (0-0)		0 (0-0)		0 (0-0)	
2013	Latvia									0 (0-0)		0 (0-0)		0 (0-0)		0 (0-0)	
2014	Latvia									0 (0-0)		0 (0-0)		0 (0-0)		0 (0-0)	
2015	Latvia	3 (3-5)	0 (0-0)	3 (3-5)	0 (0-0)	1 (1-2)	0 (0-0)	1 (1-2)	0 (0-0)	0 (0-0)	0 (0-0)	0 (0-0)	0 (0-0)	0 (0-0)	0 (0-0)	0 (0-0)	0 (0-0)
2000	Lebanon	4462 (4074-7291)		1487 (1358-2430)		842 (504-1699)		280 (168-566)		11 (2-18)		4 (1-6)		4 (1-6)		1 (0-2)	
2001	Lebanon									9 (2-16)		3 (1-5)		3 (1-5)		1 (0-2)	
2002	Lebanon									9 (2-15)		3 (1-5)		3 (1-5)		1 (0-2)	
2003	Lebanon									4 (1-6)		1 (0-2)		1 (0-2)		0 (0-1)	
2004	Lebanon									2 (0-3)		0 (0-1)		1 (0-1)		0 (0-0)	
2005	Lebanon									1 (0-2)		0 (0-1)		0 (0-1)		0 (0-0)	
2006	Lebanon									1 (0-2)		0 (0-1)		0 (0-1)		0 (0-0)	
2007	Lebanon									1 (0-2)		0 (0-1)		0 (0-1)		0 (0-0)	
2008	Lebanon									0 (0-0)		0 (0-0)		0 (0-0)		0 (0-0)	
2009	Lebanon									0 (0-0)		0 (0-0)		0 (0-0)		0 (0-0)	
2010	Lebanon									0 (0-0)		0 (0-0)		0 (0-0)		0 (0-0)	
2011	Lebanon									0 (0-0)		0 (0-0)		0 (0-0)		0 (0-0)	
2012	Lebanon									0 (0-0)		0 (0-0)		0 (0-0)		0 (0-0)	
2013	Lebanon									0 (0-0)		0 (0-0)		0 (0-0)		0 (0-0)	
2014	Lebanon									0 (0-0)		0 (0-0)		0 (0-0)		0 (0-0)	
2015	Lebanon	31 (29-51)	0 (0-0)	7 (6-11)	0 (0-0)	6 (4-12)	0 (0-0)	1 (1-3)	0 (0-0)	0 (0-0)	0 (0-0)	0 (0-0)	0 (0-0)	0 (0-0)	0 (0-0)	0 (0-0)	0 (0-0)

Hib = *Haemophilus influenzae* type b; npnm = non-pneumonia, non-meningitis; HIV = cases only in HIV-infected children; Uncertainty range provided in parentheses.

Year	Country	Hib pneumonia cases (UR)	Hib pneumonia cases, HIV (UR)	Hib pneumonia incidence rate (UR)	Hib pneumonia incidence rate, HIV (UR)	Hib severe pneumonia cases (UR)	Hib severe pneumonia cases, HIV (UR)	Hib severe pneumonia incidence rate (UR)	Hib severe pneumonia incidence rate, HIV (UR)	Hib meningitis cases (UR)	Hib meningitis cases, HIV (UR)	Hib meningitis incidence rate (UR)	Hib meningitis incidence rate, HIV (UR)	Hib NPNM cases (UR)	Hib NPNM cases, HIV (UR)	Hib NPNM incidence rate (UR)	Hib NPNM incidence rate, HIV (UR)
2000	Lesotho	5000 (4565-8169)		1884 (1720-3079)		939 (562-1895)		354 (212-714)		102 (35-163)		38 (13-62)		18 (6-28)		7 (2-11)	
2001	Lesotho									105 (37-167)		39 (14-63)		18 (6-29)		7 (2-11)	
2002	Lesotho									107 (38-170)		41 (15-65)		18 (7-29)		7 (3-11)	
2003	Lesotho									110 (40-173)		43 (16-67)		19 (7-30)		7 (3-12)	
2004	Lesotho									112 (42-177)		44 (17-70)		19 (7-30)		8 (3-12)	
2005	Lesotho									115 (44-181)		46 (18-73)		20 (8-31)		8 (3-12)	
2006	Lesotho									119 (46-186)		49 (19-76)		20 (8-32)		8 (3-13)	
2007	Lesotho									121 (47-189)		50 (19-78)		21 (8-32)		9 (3-13)	
2008	Lesotho									126 (50-196)		51 (20-80)		22 (9-34)		9 (3-14)	
2009	Lesotho									20 (8-31)		8 (3-12)		3 (1-5)		1 (1-2)	
2010	Lesotho									9 (4-15)		4 (1-6)		2 (1-3)		1 (0-1)	
2011	Lesotho									9 (3-14)		3 (1-5)		2 (1-2)		1 (0-1)	
2012	Lesotho									9 (4-14)		4 (1-5)		2 (1-2)		1 (0-1)	
2013	Lesotho									9 (4-15)		4 (1-6)		2 (1-3)		1 (0-1)	
2014	Lesotho									1 (0-2)		0 (0-1)		0 (0-0)		0 (0-0)	
2015	Lesotho	34 (31-56)	3 (3-5)	12 (11-20)	1 (1-0)	6 (4-13)	1 (0-1)	2 (1-5)	0 (0-0)	1 (0-2)	0 (0-0)	0 (0-1)	0 (0-0)	0 (0-0)	0 (0-0)	0 (0-0)	0 (0-0)
2000	Liberia	5852 (5343-9562)		1199 (1095-1959)		1089 (652-2198)		223 (134-450)		527 (303-734)		108 (62-150)		91 (52-126)		19 (11-26)	
2001	Liberia									534 (304-748)		104 (59-146)		92 (52-129)		18 (10-25)	
2002	Liberia									531 (299-747)		100 (57-141)		91 (52-129)		17 (10-24)	
2003	Liberia									533 (222-819)		99 (42-153)		92 (38-141)		17 (7-26)	
2004	Liberia									517 (212-798)		95 (39-147)		89 (36-137)		16 (7-25)	
2005	Liberia									499 (200-774)		90 (36-140)		86 (35-133)		15 (6-24)	
2006	Liberia									475 (187-740)		83 (33-129)		82 (32-127)		14 (6-22)	
2007	Liberia									450 (174-703)		76 (29-119)		77 (30-121)		13 (5-21)	
2008	Liberia									156 (59-244)		25 (10-40)		27 (10-42)		4 (2-7)	
2009	Liberia									51 (19-81)		8 (3-13)		9 (3-14)		1 (1-2)	
2010	Liberia									59 (22-93)		9 (3-14)		10 (4-16)		2 (1-2)	
2011	Liberia									37 (13-58)		6 (2-9)		6 (2-10)		1 (0-2)	
2012	Liberia									33 (12-52)		5 (2-8)		6 (2-9)		1 (0-1)	
2013	Liberia									3 (1-5)		0 (0-1)		1 (0-1)		0 (0-0)	
2014	Liberia									45 (14-74)		7 (2-11)		15 (5-25)		2 (1-4)	
2015	Liberia	993 (907-1622)	10 (9-16)	144 (131-235)	1 (1-0)	184 (110-371)	2 (1-4)	27 (16-54)	0 (0-0)	43 (13-71)	0 (0-1)	6 (2-10)	0 (0-0)	14 (4-24)	0 (0-0)	2 (1-3)	0 (0-0)
2000	Libya	8023 (7326-13110)		1470 (1342-2401)		1513 (906-3053)		277 (166-559)		141 (73-233)		26 (13-43)		48 (25-79)		9 (5-14)	
2001	Libya									135 (70-223)		24 (13-40)		45 (24-75)		8 (4-14)	
2002	Libya									129 (67-214)		23 (12-38)		44 (23-72)		8 (4-13)	
2003	Libya									122 (63-201)		21 (11-36)		41 (21-68)		7 (4-12)	
2004	Libya									114 (59-188)		20 (10-33)		38 (20-63)		7 (3-11)	
2005	Libya									106 (55-176)		18 (9-30)		36 (19-59)		6 (3-10)	
2006	Libya									99 (51-163)		17 (9-27)		33 (17-55)		6 (3-9)	
2007	Libya									62 (32-103)		10 (5-17)		21 (11-35)		3 (2-6)	
2008	Libya									10 (5-16)		2 (1-3)		3 (2-6)		1 (0-1)	
2009	Libya									9 (5-15)		1 (1-2)		3 (2-5)		0 (0-1)	
2010	Libya									8 (4-14)		1 (1-2)		3 (1-5)		0 (0-1)	
2011	Libya									7 (4-12)		1 (1-2)		2 (1-4)		0 (0-1)	
2012	Libya									1 (1-2)		0 (0-0)		0 (0-1)		0 (0-0)	
2013	Libya									1 (0-2)		0 (0-0)		0 (0-1)		0 (0-0)	
2014	Libya									1 (0-1)		0 (0-0)		0 (0-0)		0 (0-0)	
2015	Libya	44 (40-72)	0 (0-0)	7 (6-11)	0 (0-0)	8 (5-17)	0 (0-0)	1 (1-3)	0 (0-0)	1 (0-1)	0 (0-0)	0 (0-0)	0 (0-0)	0 (0-0)	0 (0-0)	0 (0-0)	0 (0-0)
2000	Lithuania	535 (489-875)		292 (267-477)		166 (100-336)		91 (54-183)		45 (20-83)		24 (11-45)		15 (7-28)		8 (4-15)	
2001	Lithuania									48 (21-88)		27 (12-50)		16 (7-30)		9 (4-17)	
2002	Lithuania									46 (21-86)		28 (12-52)		16 (7-29)		9 (4-17)	
2003	Lithuania									54 (24-101)		34 (15-64)		18 (8-34)		12 (5-22)	
2004	Lithuania									5 (2-8)		3 (1-6)		2 (1-3)		1 (0-2)	
2005	Lithuania									5 (2-10)		4 (2-7)		2 (1-3)		1 (1-2)	
2006	Lithuania									0 (0-1)		0 (0-1)		0 (0-0)		0 (0-0)	
2007	Lithuania									0 (0-1)		0 (0-0)		0 (0-0)		0 (0-0)	
2008	Lithuania									0 (0-1)		0 (0-0)		0 (0-0)		0 (0-0)	
2009	Lithuania									0 (0-0)		0 (0-0)		0 (0-0)		0 (0-0)	
2010	Lithuania									1 (0-1)		0 (0-1)		0 (0-0)		0 (0-0)	
2011	Lithuania									0 (0-1)		0 (0-0)		0 (0-0)		0 (0-0)	
2012	Lithuania									0 (0-1)		0 (0-0)		0 (0-0)		0 (0-0)	
2013	Lithuania									0 (0-1)		0 (0-0)		0 (0-0)		0 (0-0)	
2014	Lithuania									0 (0-1)		0 (0-0)		0 (0-0)		0 (0-0)	
2015	Lithuania	4 (4-7)	0 (0-0)	3 (3-5)	0 (0-0)	1 (1-3)	0 (0-0)	1 (1-2)	0 (0-0)	0 (0-1)	0 (0-0)	0 (0-0)	0 (0-0)	0 (0-0)	0 (0-0)	0 (0-0)	0 (0-0)
2000	Luxembourg	2 (1-3)		6 (5-9)		0 (0-1)		2 (1-4)		1 (0-2)		3 (1-6)		0 (0-1)		1 (0-2)	
2001	Luxembourg									1 (0-1)		2 (1-4)		0 (0-0)		1 (0-1)	
2002	Luxembourg									0 (0-0)		0 (0-0)		0 (0-0)		0 (0-0)	
2003	Luxembourg									0 (0-0)		0 (0-0)		0 (0-0)		0 (0-0)	
2004	Luxembourg									0 (0-0)		0 (0-0)		0 (0-0)		0 (0-0)	
2005	Luxembourg									0 (0-0)		0 (0-0)		0 (0-0)		0 (0-0)	
2006	Luxembourg									0 (0-0)		0 (0-0)		0 (0-0)		0 (0-0)	
2007	Luxembourg									0 (0-0)		0 (0-0)		0 (0-0)		0 (0-0)	
2008	Luxembourg									0 (0-0)		0 (0-0)		0 (0-0)		0 (0-0)	
2009	Luxembourg									0 (0-0)		0 (0-0)		0 (0-0)		0 (0-0)	
2010	Luxembourg									0 (0-0)		0 (0-0)		0 (0-0)		0 (0-0)	
2011	Luxembourg									0 (0-0)		0 (0-0)		0 (0-0)		0 (0-0)	
2012	Luxembourg									0 (0-0)		0 (0-0)		0 (0-0)		0 (0-0)	
2013	Luxembourg									0 (0-0)		0 (0-0)		0 (0-0)		0 (0-0)	
2014	Luxembourg									0 (0-0)		0 (0-0)		0 (0-0)		0 (0-0)	
2015	Luxembourg	0 (0-0)	0 (0-0)	1 (1-1)	0 (0-0)	0 (0-0)	0 (0-0)	0 (0-0)	0 (0-0)	0 (0-0)	0 (0-0)	0 (0-0)	0 (0-0)	0 (0-0)	0 (0-0)	0 (0-0)	0 (0-0)
2000	Madagascar	62992 (57513-102923)		2254 (2058-3684)		11861 (7101-23939)		424 (254-857)		1513 (511-2431)		54 (18-87)		260 (88-419)		9 (3-15)	
2001	Madagascar									1463 (496-2349)		51 (17-82)		252 (85-405)		9 (3-14)	
2002	Madagascar									1407 (478-2258)		48 (16-77)		242 (82-389)		8 (3-13)	
2003	Madagascar									1340 (456-2148)		45 (15-72)		231 (79-370)		8 (3-12)	
2004	Madagascar									1263 (430-2024)		42 (14-67)		218 (74-349)		7 (2-12)	
2005	Madagascar									1190 (400-1913)		39 (13-63)		205 (69-329)		7 (2-11)	
2006	Madagascar									1116 (371-1797)		36 (12-58)		192 (64-310)		6 (2-10)	
2007	Madagascar									1057 (311-1767)		34 (10-56)		356 (105-596)		11 (3-19)	
2008	Madagascar									513 (149-860)		16 (5-27)		173 (50-290)		5 (2-9)	
2009	Madagascar									102 (30-171)		3 (1-5)		34 (10-58)		1 (0-2)	
2010	Madagascar									109 (31-182)		3 (1-5)		37 (11-61)		1 (0-2)	
2011	Madagascar									104 (30-174)		3 (1-5)		35 (10-59)		1 (0-2)	
2012	Madagascar									107 (31-179)		3 (1-5)		36 (10-60)		1 (0-2)	
2013	Madagascar									9 (3-15)		0 (0-0)		3 (1-5)		0 (0-0)	
2014	Madagascar									9 (3-15)		0 (0-0)		3 (1-5)		0 (0-0)	
2015	Madagascar	340 (311-556)	1 (1-2)	9 (8-15)	0 (0-0)	64 (38-129)	0 (0-0)	2 (1-3)	0 (0-0)	9 (3-15)	0 (0-0)	0 (0-0)	0 (0-0)	3 (1-5)	0 (0-0)	0 (0-0)	0 (0-0)
2000	Malawi	39547 (36107-64615)		1969 (1798-3217)		7406 (4434-14948)		369 (221-744)		1434 (642-2153)		71 (32-107)		247 (111-371)		12 (6-18)	
2001	Malawi									1391 (629-2080)		67 (30-101)		240 (108-358)		12 (5-17)	
2002	Malawi									478 (144-785)		23 (7-37)		82 (25-135)		4 (1-6)	
2003	Malawi									115 (35-188)		5 (2-9)		20 (6-32)		1 (0-2)	
2004	Malawi									101 (32-166)		5 (1-8)		17 (5-29)		1 (0-1)	
2005	Malawi									92 (30-149)		4 (1-7)		16 (5-26)		1 (0-1)	
2006	Malawi									74 (26-119)		3 (1-5)		13 (5-20)		1 (0-1)	
2007	Malawi									13 (5-20)		1 (0-1)		2 (1-3)		0 (0-0)	
2008	Malawi									13 (5-21)		1 (0-1)		2 (1-4)		0 (0-0)	
2009	Malawi									14 (6-21)		1 (0-1)		2 (1-4)		0 (0-0)	
2010	Malawi									14 (6-21)		1 (0-1)		2 (1-4)		0 (0-0)	
2011	Malawi									14 (6-22)		1 (0-1)		2 (1-4)		0 (0-0)	
2012	Malawi									14 (6-22)		1 (0-1)		2 (1-4)		0 (0-0)	
2013	Malawi									14 (5-22)		1 (0-1)		5 (2-8)		0 (0-0)	
2014	Malawi									14 (5-22)		0 (0-1)		5 (2-7)		0 (0-0)	
2015	Malawi	305 (279-499)	12 (11-19)	11 (10-17)	0 (0-0)	57 (34-116)	2 (1-5)	2 (1-4)	0 (0-0)	14 (5-22)	1 (0-1)	0 (0-1)	0 (0-0)	5 (2-7)	0 (0-0)	0 (0-0)	0 (0-0)
2000	Malaysia	61587 (56230-100627)		2302 (2102-3761)		11352 (6797-22913)		424 (254-856)		431 (219-720)		16 (8-27)		145 (74-243)		5 (3-9)	
2001	Malaysia									378 (192-631)		14 (7-24)		127 (65-213)		5 (2-8)	
2002	Malaysia									340 (172-568)		13 (7-22)		115 (58-192)		4 (2-7)	
2003	Malaysia									309 (156-515)		12 (6-20)		104 (53-174)		4 (2-7)	
2004	Malaysia									121 (61-201)		5 (2-8)		41 (21-68)		2 (1-3)	
2005	Malaysia									40 (20-66)		2 (1-3)		13 (7-22)		1 (0-1)	
2006	Malaysia									34 (17-56)		1 (1-2)		11 (6-19)		0 (0-1)	
2007	Malaysia									30 (15-49)		1 (1-2)		10 (5-17)		0 (0-1)	
2008	Malaysia									24 (12-41)		1 (1-2)		8 (4-14)		0 (0-1)	
2009	Malaysia									3 (2-6)		0 (0-0)		1 (1-2)		0 (0-0)	
2010	Malaysia									3 (2-6)		0 (0-0)		1 (1-2)		0 (0-0)	
2011	Malaysia									3 (2-6)		0 (0-0)		1 (1-2)		0 (0-0)	
2012	Malaysia									3 (2-6)		0 (0-0)		1 (1-2)		0 (0-0)	
2013	Malaysia									3 (2-5)		0 (0-0)		1 (1-2)		0 (0-0)	
2014	Malaysia									3 (2-5)		0 (0-0)		1 (1-2)		0 (0-0)	
2015	Malaysia	183 (167-300)	0 (0-0)	8 (7-12)	0 (0-0)	34 (21-69)	0 (0-0)	1 (1-3)	0 (0-0)	3 (2-5)	0 (0-0)	0 (0-0)	0 (0-0)	1 (1-2)	0 (0-0)	0 (0-0)	0 (0-0)
2000	Maldives	348 (317-568)		1072 (978-1751)		65 (39-132)		201 (120-406)		2 (0-3)		5 (1-8)		1 (0-1)		2 (0-3)	
2001	Maldives									1 (0-2)		4 (1-7)		0 (0-1)		1 (0-2)	
2002	Maldives									1 (0-2)		4 (1-6)		0 (0-1)		1 (0-2)	
2003	Maldives									1 (0-2)		3 (0-6)		0 (0-1)		1 (0-2)	
2004	Maldives									1 (0-1)		3 (0-5)		0 (0-0)		1 (0-2)	
2005	Maldives									1 (0-1)		3 (0-5)		0 (0-0)		1 (0-2)	
2006	Maldives									1 (0-1)		2 (0-4)		0 (0-0)		1 (0-1)	
2007	Maldives									1 (0-1)		2 (0-4)		0 (0-0)		1 (0-1)	
2008	Maldives									1 (0-1)		2 (0-3)		0 (0-0)		1 (0-1)	
2009	Maldives									1 (0-1)		2 (0-3)		0 (0-0)		1 (0-1)	
2010	Maldives									0 (0-1)		1 (0-3)		0 (0-0)		0 (0-1)	
2011	Maldives									0 (0-1)		1 (0-2)		0 (0-0)		0 (0-1)	
2012	Maldives									0 (0-1)		1 (0-2)		0 (0-0)		0 (0-1)	
2013	Maldives									0 (0-0)		1 (0-1)		0 (0-0)		0 (0-0)	
2014	Maldives									0 (0-0)		0 (0-0)		0 (0-0)		0 (0-0)	
2015	Maldives	17 (16-29)	0 (0-0)	47 (43-78)	0 (0-0)	3 (2-7)	0 (0-0)	9 (5-18)	0 (0-0)	0 (0-0)	0 (0-0)	0 (0-0)	0 (0-0)	0 (0-0)	0 (0-0)	0 (0-0)	0 (0-0)
2000	Mali	33993 (31036-55541)		1660 (1515-2712)		6385 (3823-12888)		312 (187-629)		1394 (649-2070)		68 (32-101)		240 (112-356)		12 (5-17)	
2001	Mali									1391 (649-2064)		66 (31-97)		239 (112-355)		11 (5-17)	
2002	Mali									1367 (639-2028)		63 (29-93)		235 (110-349)		11 (5-16)	
2003	Mali									1304 (611-1934)		58 (27-86)		225 (105-333)		10 (5-15)	
2004	Mali									1279 (600-1896)		55 (26-81)		220 (103-326)		9 (4-14)	
2005	Mali									1270 (597-1880)		53 (25-78)		219 (103-324)		9 (4-13)	
2006	Mali									1002 (472-1483)		40 (19-59)		173 (81-255)		7 (3-10)	
2007	Mali									232 (109-342)		9 (4-13)		40 (19-59)		2 (1-2)	
2008	Mali									162 (52-264)		6 (2-10)		28 (9-45)		1 (0-2)	
2009	Mali									175 (56-283)		6 (2-10)		30 (10-49)		1 (0-2)	
2010	Mali									16 (5-26)		1 (0-1)		3 (1-4)		0 (0-0)	
2011	Mali									16 (5-25)		1 (0-1)		3 (1-4)		0 (0-0)	
2012	Mali									15 (5-24)		0 (0-1)		3 (1-4)		0 (0-0)	
2013	Mali									14 (5-23)		0 (0-1)		2 (1-4)		0 (0-0)	
2014	Mali									14 (5-22)		0 (0-1)		2 (1-4)		0 (0-0)	
2015	Mali	449 (410-733)	7 (6-11)	14 (13-23)	0 (0-0)	84 (50-170)	1 (1-3)	3 (2-5)	0 (0-0)	14 (5-22)	0 (0-0)	0 (0-1)	0 (0-0)	2 (1-4)	0 (0-0)	0 (0-0)	0 (0-0)
2000	Malta	1 (1-2)		6 (5-9)		0 (0-1)		2 (1-4)		1 (0-2)		4 (2-7)		0 (0-1)		1 (1-2)	
2001	Malta									0 (0-0)		0 (0-1)		0 (0-0)		0 (0-0)	
2002	Malta									0 (0-0)		0 (0-0)		0 (0-0)		0 (0-0)	
2003	Malta									0 (0-0)		0 (0-0)		0 (0-0)		0 (0-0)	
2004	Malta									0 (0-0)		0 (0-0)		0 (0-0)		0 (0-0)	
2005	Malta									0 (0-0)		0 (0-0)		0 (0-0)		0 (0-0)	
2006	Malta									0 (0-0)		0 (0-0)		0 (0-0)		0 (0-0)	
2007	Malta									0 (0-0)		0 (0-0)		0 (0-0)		0 (0-0)	
2008	Malta									0 (0-0)		0 (0-0)		0 (0-0)		0 (0-0)	

Hib = *Haemophilus influenzae* type b; npnm = non-pneumonia, non-meningitis; HIV = cases only in HIV-infected children; Uncertainty range provided in parentheses.

Year	Country	Hib pneumonia cases (UR)	Hib pneumonia cases, HIV (UR)	Hib pneumonia incidence rate (UR)	Hib pneumonia incidence rate, HIV (UR)	Hib severe pneumonia cases (UR)	Hib severe pneumonia cases, HIV (UR)	Hib severe pneumonia incidence rate (UR)	Hib severe pneumonia incidence rate, HIV (UR)	Hib meningitis cases (UR)	Hib meningitis cases, HIV (UR)	Hib meningitis incidence rate (UR)	Hib meningitis incidence rate, HIV (UR)	Hib NPNM cases (UR)	Hib NPNM cases, HIV (UR)	Hib NPNM incidence rate (UR)	Hib NPNM incidence rate, HIV (UR)
2009	Malta									0 (0-0)		0 (0-0)		0 (0-0)		0 (0-0)	
2010	Malta									0 (0-0)		0 (0-1)		0 (0-0)		0 (0-0)	
2011	Malta									0 (0-0)		0 (0-1)		0 (0-0)		0 (0-0)	
2012	Malta									0 (0-0)		0 (0-1)		0 (0-0)		0 (0-0)	
2013	Malta									0 (0-0)		0 (0-0)		0 (0-0)		0 (0-0)	
2014	Malta									0 (0-0)		0 (0-1)		0 (0-0)		0 (0-0)	
2015	Malta	0 (0-0)	0 (0-0)	1 (1-1)	0 (0-0)	0 (0-0)	0 (0-0)	0 (0-0)	0 (0-0)	0 (0-0)	0 (0-0)	0 (0-1)	0 (0-0)	0 (0-0)	0 (0-0)	0 (0-0)	0 (0-0)
2000	Marshall Islands	47 (43-76)		425 (388-695)		9 (5-18)		80 (48-161)		2 (1-2)		15 (7-19)		1 (0-1)		5 (2-6)	
2001	Marshall Islands									0 (0-1)		4 (2-5)		0 (0-0)		1 (1-2)	
2002	Marshall Islands									0 (0-0)		2 (1-3)		0 (0-0)		1 (0-1)	
2003	Marshall Islands									0 (0-0)		3 (1-4)		0 (0-0)		1 (0-1)	
2004	Marshall Islands									0 (0-0)		4 (2-5)		0 (0-0)		1 (1-2)	
2005	Marshall Islands									0 (0-0)		0 (0-0)		0 (0-0)		0 (0-0)	
2006	Marshall Islands									0 (0-0)		3 (2-4)		0 (0-0)		1 (1-1)	
2007	Marshall Islands									0 (0-0)		0 (0-0)		0 (0-0)		0 (0-0)	
2008	Marshall Islands									0 (0-0)		0 (0-0)		0 (0-0)		0 (0-0)	
2009	Marshall Islands									0 (0-0)		0 (0-0)		0 (0-0)		0 (0-0)	
2010	Marshall Islands									0 (0-0)		0 (0-0)		0 (0-0)		0 (0-0)	
2011	Marshall Islands									0 (0-0)		0 (0-0)		0 (0-0)		0 (0-0)	
2012	Marshall Islands									0 (0-0)		0 (0-0)		0 (0-0)		0 (0-0)	
2013	Marshall Islands									0 (0-0)		0 (0-0)		0 (0-0)		0 (0-0)	
2014	Marshall Islands									0 (0-0)		3 (1-4)		0 (0-0)		1 (0-1)	
2015	Marshall Islands	1 (1-1)	0 (0-0)	8 (8-14)	0 (0-0)	0 (0-0)	0 (0-0)	2 (1-3)	0 (0-0)	0 (0-0)	0 (0-0)	0 (0-0)	0 (0-0)	0 (0-0)	0 (0-0)	0 (0-0)	0 (0-0)
2000	Mauritania	6995 (6387-11430)		1597 (1458-2609)		1316 (788-2657)		301 (180-607)		165 (53-267)		38 (12-61)		28 (9-46)		6 (2-10)	
2001	Mauritania									172 (55-278)		38 (12-62)		30 (10-48)		7 (2-11)	
2002	Mauritania									179 (58-290)		39 (13-63)		31 (10-50)		7 (2-11)	
2003	Mauritania									186 (60-300)		39 (13-64)		32 (10-52)		7 (2-11)	
2004	Mauritania									189 (62-306)		39 (13-63)		33 (11-53)		7 (2-11)	
2005	Mauritania									191 (63-309)		39 (13-63)		33 (11-53)		7 (2-11)	
2006	Mauritania									193 (64-312)		38 (13-62)		33 (11-54)		7 (2-11)	
2007	Mauritania									195 (65-314)		38 (13-61)		34 (11-54)		6 (2-10)	
2008	Mauritania									195 (65-315)		37 (12-60)		34 (11-54)		6 (2-10)	
2009	Mauritania									70 (23-113)		13 (4-21)		12 (4-20)		2 (1-4)	
2010	Mauritania									24 (8-40)		4 (1-7)		4 (1-7)		1 (0-1)	
2011	Mauritania									21 (7-34)		4 (1-6)		4 (1-6)		1 (0-1)	
2012	Mauritania									19 (6-31)		3 (1-5)		3 (1-5)		1 (0-1)	
2013	Mauritania									19 (6-30)		3 (1-5)		3 (1-5)		1 (0-1)	
2014	Mauritania									2 (1-3)		0 (0-1)		0 (0-1)		0 (0-0)	
2015	Mauritania	62 (57-101)	1 (1-1)	11 (10-17)	0 (0-0)	12 (7-24)	0 (0-0)	2 (1-4)	0 (0-0)	2 (1-3)	0 (0-0)	0 (0-1)	0 (0-0)	0 (0-1)	0 (0-0)	0 (0-0)	0 (0-0)
2000	Mauritius	1356 (1238-2215)		1398 (1277-2285)		256 (153-516)		264 (158-532)		24 (13-40)		25 (13-42)		8 (4-14)		8 (4-14)	
2001	Mauritius									27 (14-44)		29 (15-48)		9 (5-15)		10 (5-16)	
2002	Mauritius									10 (5-17)		12 (6-19)		4 (2-6)		4 (2-6)	
2003	Mauritius									16 (8-27)		18 (9-29)		5 (3-9)		6 (3-10)	
2004	Mauritius									19 (10-32)		20 (11-34)		6 (3-11)		7 (4-11)	
2005	Mauritius									19 (10-32)		20 (11-34)		6 (3-11)		7 (4-11)	
2006	Mauritius									9 (5-15)		10 (5-16)		3 (2-5)		3 (2-5)	
2007	Mauritius									1 (0-1)		1 (0-1)		0 (0-0)		0 (0-0)	
2008	Mauritius									2 (1-3)		2 (1-3)		1 (0-1)		1 (0-1)	
2009	Mauritius									1 (1-2)		2 (1-3)		0 (0-1)		1 (0-1)	
2010	Mauritius									1 (1-2)		2 (1-3)		0 (0-1)		1 (0-1)	
2011	Mauritius									0 (0-0)		0 (0-0)		0 (0-0)		0 (0-0)	
2012	Mauritius									0 (0-0)		0 (0-0)		0 (0-0)		0 (0-0)	
2013	Mauritius									0 (0-0)		0 (0-0)		0 (0-0)		0 (0-0)	
2014	Mauritius									0 (0-0)		0 (0-0)		0 (0-0)		0 (0-0)	
2015	Mauritius	7 (7-12)	0 (0-0)	11 (10-18)	0 (0-0)	1 (1-3)	0 (0-0)	2 (1-4)	0 (0-0)	0 (0-0)	0 (0-0)	0 (0-0)	0 (0-0)	0 (0-0)	0 (0-0)	0 (0-0)	0 (0-0)
2000	Mexico	17372 (15861-28384)		145 (132-236)		3235 (1937-6529)		27 (16-54)		1208 (530-2139)		10 (4-18)		407 (178-721)		3 (1-6)	
2001	Mexico									444 (195-786)		4 (2-7)		150 (66-265)		1 (1-2)	
2002	Mexico									397 (174-702)		3 (1-6)		134 (59-237)		1 (0-2)	
2003	Mexico									399 (175-706)		3 (1-6)		134 (59-238)		1 (0-2)	
2004	Mexico									368 (161-652)		3 (1-6)		124 (54-220)		1 (0-2)	
2005	Mexico									45 (20-79)		0 (0-1)		15 (7-27)		0 (0-0)	
2006	Mexico									48 (21-86)		0 (0-1)		16 (7-29)		0 (0-0)	
2007	Mexico									47 (20-83)		0 (0-1)		16 (7-28)		0 (0-0)	
2008	Mexico									45 (20-79)		0 (0-1)		15 (7-27)		0 (0-0)	
2009	Mexico									34 (15-60)		0 (0-1)		11 (5-20)		0 (0-0)	
2010	Mexico									41 (18-72)		0 (0-1)		14 (6-24)		0 (0-0)	
2011	Mexico									37 (16-65)		0 (0-1)		12 (5-22)		0 (0-0)	
2012	Mexico									29 (13-51)		0 (0-0)		10 (4-17)		0 (0-0)	
2013	Mexico									27 (12-47)		0 (0-0)		9 (4-16)		0 (0-0)	
2014	Mexico									27 (12-48)		0 (0-0)		9 (4-16)		0 (0-0)	
2015	Mexico	686 (626-1121)	0 (0-0)	6 (5-10)	0 (0-0)	128 (77-258)	0 (0-0)	1 (1-2)	0 (0-0)	26 (12-47)	0 (0-0)	0 (0-0)	0 (0-0)	9 (4-16)	0 (0-0)	0 (0-0)	0 (0-0)
2000	Micronesia (Federated States of)	20 (18-33)		137 (125-224)		4 (2-8)		26 (15-52)		1 (0-1)		3 (2-5)		0 (0-0)		1 (1-2)	
2001	Micronesia (Federated States of)									1 (0-1)		3 (2-4)		0 (0-0)		1 (1-2)	
2002	Micronesia (Federated States of)									0 (0-1)		3 (2-4)		0 (0-0)		1 (1-2)	
2003	Micronesia (Federated States of)									0 (0-1)		4 (2-5)		0 (0-0)		1 (1-2)	
2004	Micronesia (Federated States of)									0 (0-1)		3 (2-5)		0 (0-0)		1 (1-2)	
2005	Micronesia (Federated States of)									0 (0-0)		0 (0-0)		0 (0-0)		0 (0-0)	
2006	Micronesia (Federated States of)									0 (0-1)		4 (2-5)		0 (0-0)		1 (1-2)	
2007	Micronesia (Federated States of)									0 (0-0)		0 (0-0)		0 (0-0)		0 (0-0)	
2008	Micronesia (Federated States of)									0 (0-0)		0 (0-0)		0 (0-0)		0 (0-0)	
2009	Micronesia (Federated States of)									0 (0-0)		0 (0-0)		0 (0-0)		0 (0-0)	
2010	Micronesia (Federated States of)									0 (0-0)		0 (0-0)		0 (0-0)		0 (0-0)	
2011	Micronesia (Federated States of)									0 (0-0)		0 (0-0)		0 (0-0)		0 (0-0)	
2012	Micronesia (Federated States of)									0 (0-0)		0 (0-0)		0 (0-0)		0 (0-0)	
2013	Micronesia (Federated States of)									0 (0-0)		0 (0-0)		0 (0-0)		0 (0-0)	
2014	Micronesia (Federated States of)									0 (0-0)		3 (1-4)		0 (0-0)		1 (0-1)	
2015	Micronesia (Federated States of)	16 (14-26)	0 (0-0)	139 (127-227)	0 (0-0)	3 (2-6)	0 (0-0)	26 (16-53)	0 (0-0)	0 (0-0)	0 (0-0)	3 (1-4)	0 (0-0)	0 (0-0)	0 (0-0)	1 (0-1)	0 (0-0)
2000	Monaco	0 (0-0)		4 (4-7)		0 (0-0)		1 (1-3)		0 (0-0)		0 (0-0)		0 (0-0)		0 (0-0)	
2001	Monaco									0 (0-0)		0 (0-0)		0 (0-0)		0 (0-0)	
2002	Monaco									0 (0-0)		0 (0-0)		0 (0-0)		0 (0-0)	
2003	Monaco									0 (0-0)		0 (0-1)		0 (0-0)		0 (0-0)	
2004	Monaco									0 (0-0)		0 (0-0)		0 (0-0)		0 (0-0)	
2005	Monaco									0 (0-0)		0 (0-0)		0 (0-0)		0 (0-0)	
2006	Monaco									0 (0-0)		0 (0-0)		0 (0-0)		0 (0-0)	
2007	Monaco									0 (0-0)		0 (0-0)		0 (0-0)		0 (0-0)	
2008	Monaco									0 (0-0)		0 (0-0)		0 (0-0)		0 (0-0)	
2009	Monaco									0 (0-0)		0 (0-0)		0 (0-0)		0 (0-0)	
2010	Monaco									0 (0-0)		0 (0-0)		0 (0-0)		0 (0-0)	
2011	Monaco									0 (0-0)		0 (0-0)		0 (0-0)		0 (0-0)	
2012	Monaco									0 (0-0)		0 (0-0)		0 (0-0)		0 (0-0)	
2013	Monaco									0 (0-0)		0 (0-0)		0 (0-0)		0 (0-0)	
2014	Monaco									0 (0-0)		0 (0-0)		0 (0-0)		0 (0-0)	
2015	Monaco	0 (0-0)	0 (0-0)	1 (1-1)	0 (0-0)	0 (0-0)	0 (0-0)	0 (0-0)	0 (0-0)	0 (0-0)	0 (0-0)	0 (0-0)	0 (0-0)	0 (0-0)	0 (0-0)	0 (0-0)	0 (0-0)
2000	Mongolia	2953 (2696-4825)		1284 (1172-2097)		554 (332-1119)		241 (144-486)		84 (35-128)		37 (15-55)		28 (12-43)		12 (5-19)	
2001	Mongolia									73 (29-111)		32 (13-49)		24 (10-38)		11 (4-17)	
2002	Mongolia									62 (24-96)		28 (11-43)		21 (8-32)		9 (4-15)	
2003	Mongolia									54 (20-84)		25 (9-39)		18 (7-28)		8 (3-13)	
2004	Mongolia									47 (17-74)		22 (8-35)		16 (6-25)		7 (3-12)	
2005	Mongolia									34 (12-55)		16 (6-25)		12 (4-19)		5 (2-8)	
2006	Mongolia									6 (2-9)		3 (1-4)		2 (1-3)		1 (0-1)	
2007	Mongolia									4 (2-7)		2 (1-3)		1 (1-2)		1 (0-1)	
2008	Mongolia									3 (1-5)		1 (1-2)		1 (0-2)		0 (0-1)	
2009	Mongolia									4 (2-6)		1 (1-2)		1 (1-2)		0 (0-1)	
2010	Mongolia									1 (0-1)		0 (0-0)		0 (0-0)		0 (0-0)	
2011	Mongolia									1 (0-1)		0 (0-0)		0 (0-0)		0 (0-0)	
2012	Mongolia									1 (0-1)		0 (0-0)		0 (0-0)		0 (0-0)	
2013	Mongolia									1 (1-2)		0 (0-1)		0 (0-1)		0 (0-0)	
2014	Mongolia									1 (1-2)		0 (0-1)		0 (0-1)		0 (0-0)	
2015	Mongolia	21 (19-34)	0 (0-0)	6 (6-10)	0 (0-0)	4 (2-8)	0 (0-0)	1 (1-2)	0 (0-0)	1 (1-2)	0 (0-0)	0 (0-1)	0 (0-0)	0 (0-1)	0 (0-0)	0 (0-0)	0 (0-0)
2000	Montenegro	61 (56-99)		149 (136-243)		19 (11-38)		46 (28-93)		0 (0-0)		0 (0-0)		0 (0-0)		0 (0-0)	
2001	Montenegro									0 (0-0)		0 (0-0)		0 (0-0)		0 (0-0)	
2002	Montenegro									2 (0-4)		5 (1-10)		1 (0-1)		2 (0-3)	
2003	Montenegro									2 (0-4)		5 (1-10)		1 (0-1)		2 (0-3)	
2004	Montenegro									4 (1-8)		10 (2-19)		1 (0-3)		3 (1-6)	
2005	Montenegro									2 (1-4)		5 (1-10)		1 (0-1)		2 (0-4)	
2006	Montenegro									0 (0-1)		1 (0-2)		0 (0-0)		0 (0-1)	
2007	Montenegro									0 (0-0)		0 (0-1)		0 (0-0)		0 (0-0)	
2008	Montenegro									0 (0-0)		0 (0-1)		0 (0-0)		0 (0-0)	
2009	Montenegro									0 (0-0)		1 (0-1)		0 (0-0)		0 (0-0)	
2010	Montenegro									0 (0-0)		0 (0-0)		0 (0-0)		0 (0-0)	
2011	Montenegro									0 (0-0)		0 (0-0)		0 (0-0)		0 (0-0)	
2012	Montenegro									0 (0-0)		0 (0-0)		0 (0-0)		0 (0-0)	
2013	Montenegro									0 (0-0)		0 (0-0)		0 (0-0)		0 (0-0)	
2014	Montenegro									0 (0-0)		0 (0-0)		0 (0-0)		0 (0-0)	
2015	Montenegro	1 (0-1)	0 (0-0)	1 (1-2)	0 (0-0)	0 (0-0)	0 (0-0)	0 (0-1)	0 (0-0)	0 (0-0)	0 (0-0)	0 (0-0)	0 (0-0)	0 (0-0)	0 (0-0)	0 (0-0)	0 (0-0)
2000	Morocco	39924 (36451-65231)		1312 (1198-2144)		7466 (4470-15069)		245 (147-495)		363 (100-612)		12 (3-20)		122 (34-206)		4 (1-7)	
2001	Morocco									330 (92-557)		11 (3-19)		111 (31-188)		4 (1-6)	
2002	Morocco									304 (85-513)		10 (3-18)		103 (29-173)		4 (1-6)	
2003	Morocco									285 (80-480)		10 (3-17)		96 (27-162)		3 (1-6)	
2004	Morocco									272 (76-458)		9 (3-16)		92 (26-154)		3 (1-5)	
2005	Morocco									265 (78-444)		9 (3-15)		89 (26-150)		3 (1-5)	
2006	Morocco									263 (81-436)		9 (3-15)		89 (27-147)		3 (1-5)	
2007	Morocco									42 (14-69)		1 (0-2)		14 (5-23)		0 (0-1)	
2008	Morocco									16 (6-26)		1 (0-1)		5 (2-9)		0 (0-0)	
2009	Morocco									17 (6-27)		1 (0-1)		6 (2-9)		0 (0-0)	
2010	Morocco									18 (7-28)		1 (0-1)		6 (2-9)		0 (0-0)	
2011	Morocco									19 (7-30)		1 (0-1)		6 (2-10)		0 (0-0)	
2012	Morocco									3 (1-5)		0 (0-0)		1 (0-2)		0 (0-0)	
2013	Morocco									3 (1-6)		0 (0-0)		1 (0-2)		0 (0-0)	
2014	Morocco									3 (1-6)		0 (0-0)		1 (0-2)		0 (0-0)	
2015	Morocco	308 (281-503)	0 (0-0)	9 (8-15)	0 (0-0)	58 (35-117)	0 (0-0)	2 (1-3)	0 (0-0)	3 (1-6)	0 (0-0)	0 (0-0)	0 (0-0)	1 (0-2)	0 (0-0)	0 (0-0)	0 (0-0)
2000	Mozambique	62648 (57199-102360)		1912 (1746-3124)		11795 (7062-23806)		360 (216-727)		2191 (1091-3199)		67 (33-98)		377 (188-551)		12 (6-17)	
2001	Mozambique									2197 (1104-3195)		65 (33-94)		378 (190-550)		11 (6-16)	
2002	Mozambique									2252 (1143-3263)		65 (33-93)		388 (197-562)		11 (6-16)	
2003	Mozambique									2357 (854-3738)		66 (24-104)		406 (147-644)		11 (4-18)	
2004	Mozambique									2497 (921-3944)		68 (25-107)		430 (159-679)		12 (4-18)	
2005	Mozambique									2633 (989-4141)		69 (26-109)		453 (170-713)		12 (4-19)	
2006	Mozambique									2710 (1037-4243)		69 (27-109)		467 (179-731)		12 (5-19)	
2007	Mozambique									2688 (1047-4185)		67 (26-105)		463 (180-721)		12 (5-18)	
2008	Mozambique									2605 (1033-4028)		64 (25-98)		449 (178-694)		11 (4-17)	
2009	Mozambique									582 (220-912)		14 (5-22)		100 (38-157)		2 (1-4)	
2010	Mozambique									243 (88-385)		6 (2-9)		42 (15-66)		1 (0-2)	
2011	Mozambique									213 (74-341)		5 (2-8)		37 (13-59)		1 (0-1)	
2012	Mozambique									193 (64-310)		4 (1-7)		33 (11-53)		1 (0-1)	
2013	Mozambique									175 (56-285)		4 (1-6)		30 (10-49)		1 (0-1)	
2014	Mozambique									16 (5-27)		0 (0-1)		3 (1-5)		0 (0-0)	
2015	Mozambique	620 (566-1012)	41 (38-67)	13 (12-21)	1 (1-0)	117 (70-236)	8 (5-16)	2 (1-5)	0 (0-0)	16 (5-26)	1 (0-2)	0 (0-1)	0 (0-0)	3 (1-4)	0 (0-0)	0 (0-0)	0 (0-0)
2000	Myanmar	83479 (76218-136397)		1632 (1490-2666)		15538 (9304-31362)		304 (182-613)		2156 (736-3424)		42 (14-67)		371 (127-590)		7 (2-12)	
2001	Myanmar									2143 (768-3365)		41 (15-65)		369 (132-579)		7 (3-11)	

Hib = *Haemophilus influenzae* type b; npnm = non-pneumonia, non-meningitis; HIV = cases only in HIV-infected children; Uncertainty range provided in parentheses.

Year	Country	Hib pneumonia cases (UR)	Hib pneumonia cases, HIV (UR)	Hib pneumonia incidence rate (UR)	Hib pneumonia incidence rate, HIV (UR)	Hib severe pneumonia cases (UR)	Hib severe pneumonia cases, HIV (UR)	Hib severe pneumonia incidence rate (UR)	Hib severe pneumonia incidence rate, HIV (UR)	Hib meningitis cases (UR)	Hib meningitis cases, HIV (UR)	Hib meningitis incidence rate (UR)	Hib meningitis incidence rate, HIV (UR)	Hib NPNM cases (UR)	Hib NPNM cases, HIV (UR)	Hib NPNM incidence rate (UR)	Hib NPNM incidence rate, HIV (UR)
2002	Myanmar									2141 (808-3320)		41 (15-63)		369 (139-572)		7 (3-11)	
2003	Myanmar									2124 (846-3245)		40 (16-61)		366 (146-559)		7 (3-10)	
2004	Myanmar									2093 (771-3291)		39 (14-62)		706 (260-1109)		13 (5-21)	
2005	Myanmar									2001 (742-3146)		38 (14-59)		675 (250-1060)		13 (5-20)	
2006	Myanmar									1873 (699-2940)		35 (13-55)		631 (236-991)		12 (4-19)	
2007	Myanmar									1725 (648-2704)		33 (12-51)		581 (218-912)		11 (4-17)	
2008	Myanmar									2597 (1066-3956)		50 (21-76)		447 (184-681)		9 (4-13)	
2009	Myanmar									1481 (563-2315)		29 (11-46)		499 (190-780)		10 (4-15)	
2010	Myanmar									1382 (526-2162)		28 (11-44)		466 (177-729)		9 (4-15)	
2011	Myanmar									1285 (491-2006)		27 (10-42)		433 (166-676)		9 (3-14)	
2012	Myanmar									1202 (462-1874)		26 (10-40)		405 (156-632)		9 (3-13)	
2013	Myanmar									259 (100-403)		6 (2-9)		87 (34-136)		2 (1-3)	
2014	Myanmar									116 (45-180)		3 (1-4)		39 (15-61)		1 (0-1)	
2015	Myanmar	6525 (5957-10661)	42 (39-69)	145 (133-238)	1 (1-0)	1224 (733-2471)	8 (5-16)	27 (16-55)	0 (0-0)	110 (43-172)	1 (0-1)	2 (1-4)	0 (0-0)	37 (15-58)	0 (0-0)	1 (0-1)	0 (0-0)
2000	Namibia	5004 (4569-8177)		1843 (1683-3011)		942 (564-1902)		347 (208-700)		46 (16-74)		17 (6-27)		8 (3-13)		3 (1-5)	
2001	Namibia									47 (17-74)		17 (6-27)		8 (3-13)		3 (1-5)	
2002	Namibia									47 (17-74)		17 (6-27)		8 (3-13)		3 (1-5)	
2003	Namibia									47 (17-75)		17 (6-27)		8 (3-13)		3 (1-5)	
2004	Namibia									48 (16-78)		18 (6-29)		16 (5-26)		6 (2-10)	
2005	Namibia									49 (17-79)		18 (6-29)		16 (6-27)		6 (2-10)	
2006	Namibia									50 (17-80)		18 (6-29)		17 (6-27)		6 (2-10)	
2007	Namibia									51 (18-82)		18 (6-30)		17 (6-28)		6 (2-10)	
2008	Namibia									52 (18-84)		19 (7-30)		18 (6-28)		6 (2-10)	
2009	Namibia									53 (19-86)		19 (7-30)		18 (6-29)		6 (2-10)	
2010	Namibia									9 (3-14)		3 (1-5)		3 (1-5)		1 (0-2)	
2011	Namibia									5 (2-9)		2 (1-3)		2 (1-3)		1 (0-1)	
2012	Namibia									5 (2-9)		2 (1-3)		2 (1-3)		1 (0-1)	
2013	Namibia									5 (2-8)		2 (1-3)		2 (1-3)		1 (0-1)	
2014	Namibia									5 (2-8)		2 (1-3)		2 (1-3)		1 (0-1)	
2015	Namibia	39 (35-63)	2 (2-3)	12 (11-19)	1 (0-0)	7 (4-15)	0 (0-1)	2 (1-4)	0 (0-0)	1 (0-1)	0 (0-0)	0 (0-0)	0 (0-0)	0 (0-0)	0 (0-0)	0 (0-0)	0 (0-0)
2000	Nauru	15 (14-25)		1822 (1664-2977)		3 (2-6)		338 (203-683)		0 (0-0)		19 (9-24)		0 (0-0)		6 (3-8)	
2001	Nauru									0 (0-0)		19 (10-25)		0 (0-0)		6 (3-8)	
2002	Nauru									0 (0-0)		16 (8-20)		0 (0-0)		5 (3-7)	
2003	Nauru									0 (0-0)		20 (10-25)		0 (0-0)		7 (3-9)	
2004	Nauru									0 (0-0)		20 (10-26)		0 (0-0)		7 (3-9)	
2005	Nauru									0 (0-0)		21 (11-27)		0 (0-0)		7 (4-9)	
2006	Nauru									0 (0-0)		22 (11-29)		0 (0-0)		7 (4-10)	
2007	Nauru									0 (0-0)		18 (9-24)		0 (0-0)		6 (3-8)	
2008	Nauru									0 (0-0)		19 (9-24)		0 (0-0)		6 (3-8)	
2009	Nauru									0 (0-0)		2 (1-3)		0 (0-0)		1 (0-1)	
2010	Nauru									0 (0-0)		1 (0-1)		0 (0-0)		0 (0-0)	
2011	Nauru									0 (0-0)		1 (0-1)		0 (0-0)		0 (0-0)	
2012	Nauru									0 (0-0)		1 (1-2)		0 (0-0)		0 (0-1)	
2013	Nauru									0 (0-0)		1 (1-2)		0 (0-0)		0 (0-1)	
2014	Nauru									0 (0-0)		0 (0-0)		0 (0-0)		0 (0-0)	
2015	Nauru	0 (0-0)	0 (0-0)	9 (8-14)	0 (0-0)	0 (0-0)	0 (0-0)	2 (1-3)	0 (0-0)	0 (0-0)	0 (0-0)	0 (0-0)	0 (0-0)	0 (0-0)	0 (0-0)	0 (0-0)	0 (0-0)
2000	Nepal	56787 (51847-92784)		1617 (1476-2642)		10655 (6379-21505)		303 (182-612)		827 (233-1347)		24 (7-38)		142 (40-232)		4 (1-7)	
2001	Nepal									787 (226-1276)		22 (6-36)		135 (39-220)		4 (1-6)	
2002	Nepal									737 (193-1226)		21 (5-35)		248 (65-413)		7 (2-12)	
2003	Nepal									694 (187-1151)		20 (5-33)		234 (63-388)		7 (2-11)	
2004	Nepal									653 (181-1079)		19 (5-31)		220 (61-364)		6 (2-11)	
2005	Nepal									615 (175-1012)		18 (5-30)		207 (59-341)		6 (2-10)	
2006	Nepal									576 (168-943)		17 (5-28)		194 (57-318)		6 (2-10)	
2007	Nepal									539 (160-881)		17 (5-27)		182 (54-297)		6 (2-9)	
2008	Nepal									500 (150-816)		16 (5-25)		169 (51-275)		5 (2-9)	
2009	Nepal									467 (142-761)		15 (4-24)		157 (48-256)		5 (2-8)	
2010	Nepal									428 (132-696)		14 (4-22)		144 (44-235)		5 (1-8)	
2011	Nepal									61 (19-100)		2 (1-3)		21 (6-34)		1 (0-1)	
2012	Nepal									29 (9-46)		1 (0-2)		10 (3-16)		0 (0-1)	
2013	Nepal									25 (8-40)		1 (0-1)		8 (3-14)		0 (0-0)	
2014	Nepal									23 (7-37)		1 (0-1)		8 (2-13)		0 (0-0)	
2015	Nepal	2686 (2453-4389)	4 (4-7)	97 (89-159)	0 (0-0)	507 (304-1024)	1 (0-2)	18 (11-37)	0 (0-0)	22 (7-36)	0 (0-0)	1 (0-1)	0 (0-0)	7 (2-12)	0 (0-0)	0 (0-0)	0 (0-0)
2000	Netherlands	51 (46-83)		5 (5-9)		16 (9-32)		2 (1-3)		14 (6-26)		1 (1-3)		5 (2-9)		1 (0-1)	
2001	Netherlands									14 (6-26)		1 (1-3)		5 (2-9)		0 (0-1)	
2002	Netherlands									2 (1-4)		0 (0-0)		1 (0-1)		0 (0-0)	
2003	Netherlands									2 (1-3)		0 (0-0)		1 (0-1)		0 (0-0)	
2004	Netherlands									1 (0-1)		0 (0-0)		0 (0-0)		0 (0-0)	
2005	Netherlands									1 (1-3)		0 (0-0)		0 (0-1)		0 (0-0)	
2006	Netherlands									1 (0-1)		0 (0-0)		0 (0-0)		0 (0-0)	
2007	Netherlands									1 (0-2)		0 (0-0)		0 (0-1)		0 (0-0)	
2008	Netherlands									1 (0-1)		0 (0-0)		0 (0-0)		0 (0-0)	
2009	Netherlands									1 (0-2)		0 (0-0)		0 (0-1)		0 (0-0)	
2010	Netherlands									1 (0-2)		0 (0-0)		0 (0-1)		0 (0-0)	
2011	Netherlands									1 (0-2)		0 (0-0)		0 (0-1)		0 (0-0)	
2012	Netherlands									1 (0-1)		0 (0-0)		0 (0-0)		0 (0-0)	
2013	Netherlands									0 (0-0)		0 (0-0)		0 (0-0)		0 (0-0)	
2014	Netherlands									1 (0-1)		0 (0-0)		0 (0-0)		0 (0-0)	
2015	Netherlands	6 (6-11)	0 (0-0)	1 (1-1)	0 (0-0)	2 (1-4)	0 (0-0)	0 (0-0)	0 (0-0)	1 (0-1)	0 (0-0)	0 (0-0)	0 (0-0)	0 (0-0)	0 (0-0)	0 (0-0)	0 (0-0)
2000	New Zealand	28 (26-46)		10 (9-16)		9 (5-18)		3 (2-6)		22 (13-31)		8 (5-11)		7 (4-11)		3 (2-4)	
2001	New Zealand									28 (16-39)		10 (6-14)		9 (5-13)		3 (2-5)	
2002	New Zealand									3 (2-4)		1 (1-1)		1 (1-1)		0 (0-0)	
2003	New Zealand									2 (1-3)		1 (0-1)		1 (0-1)		0 (0-0)	
2004	New Zealand									2 (1-3)		1 (0-1)		1 (0-1)		0 (0-0)	
2005	New Zealand									2 (1-3)		1 (0-1)		1 (0-1)		0 (0-0)	
2006	New Zealand									1 (0-1)		0 (0-0)		0 (0-0)		0 (0-0)	
2007	New Zealand									1 (1-2)		0 (0-1)		0 (0-1)		0 (0-0)	
2008	New Zealand									2 (1-3)		1 (0-1)		1 (0-1)		0 (0-0)	
2009	New Zealand									1 (0-1)		0 (0-0)		0 (0-0)		0 (0-0)	
2010	New Zealand									1 (1-2)		0 (0-1)		0 (0-1)		0 (0-0)	
2011	New Zealand									2 (1-3)		1 (0-1)		1 (0-1)		0 (0-0)	
2012	New Zealand									1 (1-2)		0 (0-1)		0 (0-1)		0 (0-0)	
2013	New Zealand									1 (1-2)		0 (0-1)		0 (0-1)		0 (0-0)	
2014	New Zealand									1 (1-2)		0 (0-1)		0 (0-1)		0 (0-0)	
2015	New Zealand	4 (3-6)	0 (0-0)	1 (1-2)	0 (0-0)	1 (1-2)	0 (0-0)	0 (0-1)	0 (0-0)	1 (1-2)	0 (0-0)	0 (0-1)	0 (0-0)	0 (0-1)	0 (0-0)	0 (0-0)	0 (0-0)
2000	Nicaragua	753 (687-1230)		115 (105-188)		141 (85-285)		22 (13-44)		12 (3-19)		2 (1-3)		4 (1-6)		1 (0-1)	
2001	Nicaragua									10 (3-17)		2 (0-3)		3 (1-6)		1 (0-1)	
2002	Nicaragua									10 (3-16)		2 (0-3)		3 (1-5)		1 (0-1)	
2003	Nicaragua									9 (3-15)		1 (0-2)		3 (1-5)		0 (0-1)	
2004	Nicaragua									1 (0-2)		0 (0-0)		0 (0-1)		0 (0-0)	
2005	Nicaragua									1 (0-2)		0 (0-0)		0 (0-1)		0 (0-0)	
2006	Nicaragua									1 (0-2)		0 (0-0)		0 (0-1)		0 (0-0)	
2007	Nicaragua									1 (0-2)		0 (0-0)		0 (0-1)		0 (0-0)	
2008	Nicaragua									1 (0-2)		0 (0-0)		0 (0-1)		0 (0-0)	
2009	Nicaragua									1 (0-2)		0 (0-0)		0 (0-1)		0 (0-0)	
2010	Nicaragua									1 (0-2)		0 (0-0)		0 (0-1)		0 (0-0)	
2011	Nicaragua									1 (0-2)		0 (0-0)		0 (0-1)		0 (0-0)	
2012	Nicaragua									1 (0-2)		0 (0-0)		0 (0-1)		0 (0-0)	
2013	Nicaragua									1 (0-2)		0 (0-0)		0 (0-1)		0 (0-0)	
2014	Nicaragua									1 (0-2)		0 (0-0)		0 (0-1)		0 (0-0)	
2015	Nicaragua	47 (43-77)	0 (0-0)	8 (7-13)	0 (0-0)	9 (5-18)	0 (0-0)	1 (1-3)	0 (0-0)	1 (0-2)	0 (0-0)	0 (0-0)	0 (0-0)	0 (0-1)	0 (0-0)	0 (0-0)	0 (0-0)
2000	Niger	57466 (52468-93894)		2531 (2311-4135)		10737 (6429-21671)		473 (283-954)		2228 (998-3348)		98 (44-147)		384 (172-577)		17 (8-25)	
2001	Niger									2315 (1052-3462)		97 (44-145)		399 (181-596)		17 (8-25)	
2002	Niger									2369 (1093-3527)		96 (44-142)		408 (188-607)		16 (8-25)	
2003	Niger									2400 (1124-3556)		93 (44-139)		413 (194-612)		16 (8-24)	
2004	Niger									2425 (1155-3578)		91 (43-135)		418 (199-616)		16 (7-23)	
2005	Niger									2450 (1186-3598)		89 (43-131)		422 (204-620)		15 (7-22)	
2006	Niger									2486 (1222-3630)		87 (43-126)		428 (210-625)		15 (7-22)	
2007	Niger									2596 (1283-3786)		87 (43-127)		447 (221-652)		15 (7-22)	
2008	Niger									2519 (866-4037)		82 (28-131)		434 (149-695)		14 (5-22)	
2009	Niger									695 (241-1111)		22 (8-35)		120 (41-191)		4 (1-6)	
2010	Niger									300 (105-480)		9 (3-14)		52 (18-83)		2 (1-2)	
2011	Niger									279 (99-445)		8 (3-13)		48 (17-77)		1 (0-2)	
2012	Niger									299 (106-475)		8 (3-13)		51 (18-82)		1 (1-2)	
2013	Niger									314 (113-498)		8 (3-13)		54 (19-86)		1 (1-2)	
2014	Niger									27 (10-42)		1 (0-1)		5 (2-7)		0 (0-0)	
2015	Niger	7222 (6594-11801)	23 (21-37)	177 (162-290)	1 (1-0)	1349 (808-2724)	4 (3-9)	33 (20-67)	0 (0-0)	333 (122-525)	1 (0-2)	8 (3-13)	0 (0-0)	57 (21-90)	0 (0-0)	1 (1-2)	0 (0-0)
2000	Nigeria	414748 (378673-677658)		2001 (1827-3269)		77113 (46171-155643)		372 (223-751)		9075 (4038-13650)		44 (19-66)		1563 (695-2351)		8 (3-11)	
2001	Nigeria									9231 (4152-13839)		43 (19-65)		1590 (715-2383)		7 (3-11)	
2002	Nigeria									9301 (4229-13898)		42 (19-63)		1602 (728-2393)		7 (3-11)	
2003	Nigeria									9126 (4197-13591)		40 (19-60)		1571 (723-2340)		7 (3-10)	
2004	Nigeria									9159 (4260-13597)		39 (18-58)		1577 (734-2341)		7 (3-10)	
2005	Nigeria									9242 (4349-13673)		39 (18-57)		1591 (749-2355)		7 (3-10)	
2006	Nigeria									9601 (4570-14152)		39 (19-57)		1653 (787-2437)		7 (3-10)	
2007	Nigeria									9886 (3230-15984)		39 (13-63)		1702 (556-2752)		7 (2-11)	
2008	Nigeria									9974 (3320-16042)		38 (13-62)		1717 (572-2762)		7 (2-11)	
2009	Nigeria									10075 (3305-16264)		38 (12-61)		1735 (569-2801)		7 (2-11)	
2010	Nigeria									10152 (3280-16437)		37 (12-60)		1748 (565-2831)		6 (2-10)	
2011	Nigeria									9858 (3138-16005)		35 (11-57)		1698 (540-2756)		6 (2-10)	
2012	Nigeria									8892 (2774-14490)		31 (10-51)		1531 (478-2495)		5 (2-9)	
2013	Nigeria									1444 (442-2362)		5 (2-8)		249 (76-407)		1 (0-1)	
2014	Nigeria									1334 (401-2190)		4 (1-7)		230 (69-377)		1 (0-1)	
2015	Nigeria	61921 (56536-101174)	2193 (2003-3584)	202 (185-331)	7 (7-1)	11368 (6807-22945)	403 (241-813)	37 (22-75)	1 (1-0)	1189 (351-1959)	42 (12-69)	4 (1-6)	0 (0-0)	205 (60-337)	7 (2-12)	1 (0-1)	0 (0-0)
2000	Niue	1 (1-1)		512 (468-837)		0 (0-0)		94 (56-189)		0 (0-0)		74 (43-104)		0 (0-0)		25 (14-35)	
2001	Niue									0 (0-0)		32 (18-45)		0 (0-0)		11 (6-15)	
2002	Niue									0 (0-0)		31 (18-43)		0 (0-0)		10 (6-15)	
2003	Niue									0 (0-0)		30 (17-42)		0 (0-0)		10 (6-14)	
2004	Niue									0 (0-0)		0 (0-0)		0 (0-0)		0 (0-0)	
2005	Niue									0 (0-0)		0 (0-0)		0 (0-0)		0 (0-0)	
2006	Niue									0 (0-0)		5 (3-7)		0 (0-0)		2 (1-2)	
2007	Niue									0 (0-0)		5 (3-8)		0 (0-0)		2 (1-3)	
2008	Niue									0 (0-0)		6 (3-8)		0 (0-0)		2 (1-3)	
2009	Niue									0 (0-0)		6 (3-8)		0 (0-0)		2 (1-3)	
2010	Niue									0 (0-0)		7 (4-10)		0 (0-0)		2 (1-3)	
2011	Niue									0 (0-0)		7 (4-10)		0 (0-0)		2 (1-3)	
2012	Niue									0 (0-0)		7 (4-10)		0 (0-0)		2 (1-3)	
2013	Niue									0 (0-0)		7 (4-9)		0 (0-0)		2 (1-3)	
2014	Niue									0 (0-0)		6 (4-9)		0 (0-0)		2 (1-3)	
2015	Niue	0 (0-0)	0 (0-0)	7 (6-11)	0 (0-0)	0 (0-0)	0 (0-0)	1 (1-2)	0 (0-0)	0 (0-0)	0 (0-0)	6 (4-9)	0 (0-0)	0 (0-0)	0 (0-0)	2 (1-3)	0 (0-0)
2000	Norway	17 (15-27)		6 (5-9)		5 (3-11)		2 (1-4)		7 (3-12)		2 (1-4)		2 (1-4)		1 (0-1)	
2001	Norway									0 (0-0)		0 (0-0)		0 (0-0)		0 (0-0)	
2002	Norway									0 (0-0)		0 (0-0)		0 (0-0)		0 (0-0)	
2003	Norway									0 (0-1)		0 (0-0)		0 (0-0)		0 (0-0)	
2004	Norway									0 (0-0)		0 (0-0)		0 (0-0)		0 (0-0)	
2005	Norway									0 (0-0)		0 (0-0)		0 (0-0)		0 (0-0)	
2006	Norway									0 (0-1)		0 (0-0)		0 (0-0)		0 (0-0)	
2007	Norway									0 (0-0)		0 (0-0)		0 (0-0)		0 (0-0)	
2008	Norway									0 (0-0)		0 (0-0)		0 (0-0)		0 (0-0)	
2009	Norway									0 (0-1)		0 (0-0)		0 (0-0)		0 (0-0)	
2010	Norway									0 (0-0)		0 (0-0)		0 (0-0)		0 (0-0)	

Hib = *Haemophilus influenzae* type b; npnm = non-pneumonia, non-meningitis; HIV = cases only in HIV-infected children; Uncertainty range provided in parentheses.

Year	Country	Hib pneumonia cases (UR)	Hib pneumonia cases, HIV (UR)	Hib pneumonia incidence rate (UR)	Hib pneumonia incidence rate, HIV (UR)	Hib severe pneumonia cases (UR)	Hib severe pneumonia cases, HIV (UR)	Hib severe pneumonia incidence rate (UR)	Hib severe pneumonia incidence rate, HIV (UR)	Hib meningitis cases (UR)	Hib meningitis cases, HIV (UR)	Hib meningitis incidence rate (UR)	Hib meningitis incidence rate, HIV (UR)	Hib NPNM cases (UR)	Hib NPNM cases, HIV (UR)	Hib NPNM incidence rate (UR)	Hib NPNM incidence rate, HIV (UR)
2011	Norway									0 (0-1)		0 (0-0)		0 (0-0)		0 (0-0)	
2012	Norway									0 (0-0)		0 (0-0)		0 (0-0)		0 (0-0)	
2013	Norway									0 (0-0)		0 (0-0)		0 (0-0)		0 (0-0)	
2014	Norway									0 (0-0)		0 (0-0)		0 (0-0)		0 (0-0)	
2015	Norway	2 (2-4)	0 (0-0)	1 (1-1)	0 (0-0)	1 (0-1)	0 (0-0)	0 (0-0)	0 (0-0)	0 (0-0)	0 (0-0)	0 (0-0)	0 (0-0)	0 (0-0)	0 (0-0)	0 (0-0)	0 (0-0)
2000	Oman	3948 (3605-6451)		1438 (1313-2349)		744 (445-1501)		271 (162-547)		43 (22-72)		16 (8-26)		14 (7-24)		5 (3-9)	
2001	Oman									37 (19-62)		14 (7-23)		12 (6-21)		5 (2-8)	
2002	Oman									8 (4-13)		3 (2-5)		3 (1-5)		1 (1-2)	
2003	Oman									3 (1-5)		1 (1-2)		1 (0-2)		0 (0-1)	
2004	Oman									3 (1-4)		1 (1-2)		1 (0-1)		0 (0-1)	
2005	Oman									2 (1-4)		1 (0-2)		1 (0-1)		0 (0-1)	
2006	Oman									3 (1-5)		1 (1-2)		1 (0-2)		0 (0-1)	
2007	Oman									0 (0-1)		0 (0-0)		0 (0-0)		0 (0-0)	
2008	Oman									0 (0-1)		0 (0-0)		0 (0-0)		0 (0-0)	
2009	Oman									0 (0-1)		0 (0-0)		0 (0-0)		0 (0-0)	
2010	Oman									1 (0-1)		0 (0-0)		0 (0-0)		0 (0-0)	
2011	Oman									1 (0-1)		0 (0-0)		0 (0-0)		0 (0-0)	
2012	Oman									1 (0-1)		0 (0-0)		0 (0-0)		0 (0-0)	
2013	Oman									1 (0-1)		0 (0-0)		0 (0-0)		0 (0-0)	
2014	Oman									1 (0-1)		0 (0-0)		0 (0-0)		0 (0-0)	
2015	Oman	26 (24-43)	0 (0-0)	7 (6-11)	0 (0-0)	5 (3-10)	0 (0-0)	1 (1-3)	0 (0-0)	1 (0-1)	0 (0-0)	0 (0-0)	0 (0-0)	0 (0-0)	0 (0-0)	0 (0-0)	0 (0-0)
2000	Pakistan	199898 (182511-326614)		1011 (923-1652)		37384 (22384-75456)		189 (113-382)		8627 (3484-13221)		44 (18-67)		1485 (600-2277)		8 (3-12)	
2001	Pakistan									8488 (3441-12995)		43 (17-65)		1462 (593-2238)		7 (3-11)	
2002	Pakistan									8250 (3356-12620)		42 (17-64)		1421 (578-2173)		7 (3-11)	
2003	Pakistan									8077 (3298-12344)		41 (17-63)		1391 (568-2126)		7 (3-11)	
2004	Pakistan									7970 (3265-12173)		41 (17-62)		1372 (562-2096)		7 (3-11)	
2005	Pakistan									7968 (3275-12160)		40 (17-62)		1372 (564-2094)		7 (3-11)	
2006	Pakistan									7950 (3277-12120)		40 (17-61)		1369 (564-2087)		7 (3-11)	
2007	Pakistan									7952 (3277-12119)		39 (16-60)		1369 (564-2087)		7 (3-10)	
2008	Pakistan									7942 (3244-12133)		38 (16-58)		1368 (559-2089)		7 (3-10)	
2009	Pakistan									1703 (689-2607)		8 (3-12)		293 (119-449)		1 (1-2)	
2010	Pakistan									740 (297-1135)		3 (1-5)		127 (51-195)		1 (0-1)	
2011	Pakistan									832 (331-1279)		4 (1-6)		143 (57-220)		1 (0-1)	
2012	Pakistan									833 (328-1284)		4 (1-5)		144 (57-221)		1 (0-1)	
2013	Pakistan									811 (319-1248)		3 (1-5)		140 (55-215)		1 (0-1)	
2014	Pakistan									70 (28-107)		0 (0-0)		12 (5-18)		0 (0-0)	
2015	Pakistan	3343 (3052-5462)	2 (2-3)	14 (13-23)	0 (0-0)	624 (374-1260)	0 (0-1)	3 (2-5)	0 (0-0)	68 (27-105)	0 (0-0)	0 (0-0)	0 (0-0)	12 (5-18)	0 (0-0)	0 (0-0)	0 (0-0)
2000	Palau	4 (3-6)		288 (263-470)		1 (0-1)		53 (32-107)		0 (0-0)		14 (8-20)		0 (0-0)		5 (3-7)	
2001	Palau									0 (0-0)		14 (8-20)		0 (0-0)		5 (3-7)	
2002	Palau									0 (0-0)		16 (9-23)		0 (0-0)		5 (3-8)	
2003	Palau									0 (0-0)		2 (1-2)		0 (0-0)		1 (0-1)	
2004	Palau									0 (0-0)		2 (1-3)		0 (0-0)		1 (0-1)	
2005	Palau									0 (0-0)		2 (1-2)		0 (0-0)		1 (0-1)	
2006	Palau									0 (0-0)		2 (1-3)		0 (0-0)		1 (0-1)	
2007	Palau									0 (0-0)		1 (1-2)		0 (0-0)		0 (0-1)	
2008	Palau									0 (0-0)		1 (1-2)		0 (0-0)		1 (0-1)	
2009	Palau									0 (0-0)		13 (7-18)		0 (0-0)		4 (2-6)	
2010	Palau									0 (0-0)		2 (1-2)		0 (0-0)		1 (0-1)	
2011	Palau									0 (0-0)		2 (1-3)		0 (0-0)		1 (0-1)	
2012	Palau									0 (0-0)		2 (1-3)		0 (0-0)		1 (0-1)	
2013	Palau									0 (0-0)		1 (1-2)		0 (0-0)		0 (0-1)	
2014	Palau									0 (0-0)		1 (1-1)		0 (0-0)		0 (0-0)	
2015	Palau	0 (0-0)	0 (0-0)	10 (9-17)	0 (0-0)	0 (0-0)	0 (0-0)	2 (1-4)	0 (0-0)	0 (0-0)	0 (0-0)	1 (1-1)	0 (0-0)	0 (0-0)	0 (0-0)	0 (0-0)	0 (0-0)
2000	Panama	3232 (2951-5280)		961 (878-1571)		603 (361-1216)		179 (107-362)		43 (7-75)		13 (2-22)		15 (2-25)		4 (1-7)	
2001	Panama									9 (2-16)		3 (0-5)		3 (1-6)		1 (0-2)	
2002	Panama									4 (1-6)		1 (0-2)		1 (0-2)		0 (0-1)	
2003	Panama									2 (0-3)		1 (0-1)		1 (0-1)		0 (0-0)	
2004	Panama									4 (1-7)		1 (0-2)		1 (0-2)		0 (0-1)	
2005	Panama									3 (1-5)		1 (0-2)		1 (0-2)		0 (0-1)	
2006	Panama									0 (0-1)		0 (0-0)		0 (0-0)		0 (0-0)	
2007	Panama									0 (0-1)		0 (0-0)		0 (0-0)		0 (0-0)	
2008	Panama									0 (0-1)		0 (0-0)		0 (0-0)		0 (0-0)	
2009	Panama									1 (0-1)		0 (0-0)		0 (0-0)		0 (0-0)	
2010	Panama									1 (0-1)		0 (0-0)		0 (0-0)		0 (0-0)	
2011	Panama									1 (0-1)		0 (0-0)		0 (0-0)		0 (0-0)	
2012	Panama									0 (0-1)		0 (0-0)		0 (0-0)		0 (0-0)	
2013	Panama									1 (0-1)		0 (0-0)		0 (0-0)		0 (0-0)	
2014	Panama									1 (0-1)		0 (0-0)		0 (0-0)		0 (0-0)	
2015	Panama	22 (20-36)	0 (0-0)	6 (5-10)	0 (0-0)	4 (2-8)	0 (0-0)	1 (1-2)	0 (0-0)	1 (0-1)	0 (0-0)	0 (0-0)	0 (0-0)	0 (0-0)	0 (0-0)	0 (0-0)	0 (0-0)
2000	Papua New Guinea	10651 (9724-17402)		1297 (1185-2120)		1997 (1196-4030)		243 (146-491)		507 (258-653)		62 (31-80)		87 (44-112)		11 (5-14)	
2001	Papua New Guinea									520 (265-670)		62 (32-80)		90 (46-115)		11 (5-14)	
2002	Papua New Guinea									529 (270-682)		62 (32-80)		91 (47-117)		11 (5-14)	
2003	Papua New Guinea									536 (274-690)		62 (32-80)		92 (47-119)		11 (5-14)	
2004	Papua New Guinea									545 (279-702)		62 (32-80)		94 (48-121)		11 (5-14)	
2005	Papua New Guinea									555 (265-722)		63 (30-81)		187 (89-243)		21 (10-27)	
2006	Papua New Guinea									560 (268-729)		63 (30-81)		189 (90-246)		21 (10-27)	
2007	Papua New Guinea									564 (271-733)		62 (30-81)		190 (91-247)		21 (10-27)	
2008	Papua New Guinea									560 (270-728)		61 (29-79)		189 (91-245)		20 (10-27)	
2009	Papua New Guinea									214 (103-278)		23 (11-30)		72 (35-94)		8 (4-10)	
2010	Papua New Guinea									76 (37-98)		8 (4-10)		26 (12-33)		3 (1-4)	
2011	Papua New Guinea									70 (34-91)		7 (4-9)		24 (11-31)		2 (1-3)	
2012	Papua New Guinea									66 (32-86)		7 (3-9)		22 (11-29)		2 (1-3)	
2013	Papua New Guinea									61 (30-79)		6 (3-8)		21 (10-27)		2 (1-3)	
2014	Papua New Guinea									64 (32-83)		7 (3-9)		22 (11-28)		2 (1-3)	
2015	Papua New Guinea	1278 (1167-2089)	12 (11-20)	131 (119-213)	1 (1-0)	241 (144-486)	2 (1-5)	25 (15-50)	0 (0-0)	62 (31-81)	1 (0-1)	6 (3-8)	0 (0-0)	21 (10-27)	0 (0-0)	2 (1-3)	0 (0-0)
2000	Paraguay	9125 (8331-14910)		1317 (1203-2152)		1710 (1024-3452)		247 (148-498)		153 (77-241)		22 (11-35)		51 (26-81)		7 (4-12)	
2001	Paraguay									152 (77-242)		22 (11-35)		51 (26-82)		7 (4-12)	
2002	Paraguay									153 (79-249)		22 (12-36)		52 (27-84)		8 (4-12)	
2003	Paraguay									48 (26-81)		7 (4-12)		16 (9-27)		2 (1-4)	
2004	Paraguay									27 (10-39)		4 (1-6)		9 (3-13)		1 (0-2)	
2005	Paraguay									29 (11-45)		4 (2-7)		10 (4-15)		2 (1-2)	
2006	Paraguay									32 (13-54)		5 (2-8)		11 (5-18)		2 (1-3)	
2007	Paraguay									36 (16-63)		6 (2-10)		12 (5-21)		2 (1-3)	
2008	Paraguay									4 (2-7)		1 (0-1)		1 (1-2)		0 (0-0)	
2009	Paraguay									4 (2-7)		1 (0-1)		1 (1-2)		0 (0-0)	
2010	Paraguay									4 (2-7)		1 (0-1)		1 (1-2)		0 (0-0)	
2011	Paraguay									4 (2-6)		1 (0-1)		1 (1-2)		0 (0-0)	
2012	Paraguay									4 (2-7)		1 (0-1)		1 (1-2)		0 (0-0)	
2013	Paraguay									4 (2-7)		1 (0-1)		1 (1-2)		0 (0-0)	
2014	Paraguay									4 (2-6)		1 (0-1)		1 (1-2)		0 (0-0)	
2015	Paraguay	52 (48-86)	0 (0-0)	8 (7-13)	0 (0-0)	10 (6-20)	0 (0-0)	1 (1-3)	0 (0-0)	4 (2-6)	0 (0-0)	1 (0-1)	0 (0-0)	1 (1-2)	0 (0-0)	0 (0-0)	0 (0-0)
2000	Peru	4685 (4277-7655)		158 (145-259)		880 (527-1777)		30 (18-60)		126 (37-210)		4 (1-7)		43 (13-71)		1 (0-2)	
2001	Peru									145 (44-240)		5 (1-8)		49 (15-81)		2 (1-3)	
2002	Peru									106 (33-176)		4 (1-6)		36 (11-59)		1 (0-2)	
2003	Peru									98 (31-161)		3 (1-6)		33 (11-54)		1 (0-2)	
2004	Peru									6 (1-11)		0 (0-0)		2 (0-4)		0 (0-0)	
2005	Peru									6 (1-10)		0 (0-0)		2 (0-4)		0 (0-0)	
2006	Peru									6 (1-10)		0 (0-0)		2 (0-3)		0 (0-0)	
2007	Peru									5 (1-9)		0 (0-0)		2 (0-3)		0 (0-0)	
2008	Peru									5 (1-9)		0 (0-0)		2 (0-3)		0 (0-0)	
2009	Peru									4 (1-8)		0 (0-0)		1 (0-3)		0 (0-0)	
2010	Peru									4 (1-7)		0 (0-0)		1 (0-2)		0 (0-0)	
2011	Peru									4 (0-7)		0 (0-0)		1 (0-2)		0 (0-0)	
2012	Peru									3 (0-6)		0 (0-0)		1 (0-2)		0 (0-0)	
2013	Peru									3 (0-6)		0 (0-0)		1 (0-2)		0 (0-0)	
2014	Peru									3 (0-5)		0 (0-0)		1 (0-2)		0 (0-0)	
2015	Peru	178 (163-291)	0 (0-0)	6 (5-10)	0 (0-0)	33 (20-67)	0 (0-0)	1 (1-2)	0 (0-0)	3 (0-5)	0 (0-0)	0 (0-0)	0 (0-0)	1 (0-2)	0 (0-0)	0 (0-0)	0 (0-0)
2000	Philippines	271418 (247810-443470)		2570 (2347-4200)		50960 (30512-102857)		483 (289-974)		1559 (524-2494)		15 (5-24)		526 (177-841)		5 (2-8)	
2001	Philippines									1515 (505-2430)		14 (5-23)		511 (170-819)		5 (2-8)	
2002	Philippines									1465 (483-2354)		13 (4-22)		494 (163-794)		5 (1-7)	
2003	Philippines									1419 (464-2284)		13 (4-21)		478 (156-770)		4 (1-7)	
2004	Philippines									1362 (441-2197)		12 (4-20)		459 (149-741)		4 (1-7)	
2005	Philippines									1267 (406-2047)		11 (4-18)		427 (137-690)		4 (1-6)	
2006	Philippines									1230 (391-1991)		11 (4-18)		415 (132-671)		4 (1-6)	
2007	Philippines									1173 (369-1902)		11 (3-17)		395 (124-641)		4 (1-6)	
2008	Philippines									1131 (353-1839)		10 (3-17)		381 (119-620)		4 (1-6)	
2009	Philippines									1105 (354-1787)		10 (3-17)		373 (119-602)		4 (1-6)	
2010	Philippines									1098 (361-1768)		10 (3-17)		370 (122-596)		4 (1-6)	
2011	Philippines									911 (309-1461)		9 (3-14)		307 (104-493)		3 (1-5)	
2012	Philippines									698 (244-1118)		6 (2-10)		235 (82-377)		2 (1-4)	
2013	Philippines									96 (14-163)		1 (0-1)		32 (5-55)		0 (0-1)	
2014	Philippines									136 (20-229)		1 (0-2)		46 (7-77)		0 (0-1)	
2015	Philippines	19949 (18214-32595)	3 (3-6)	180 (165-295)	0 (0-0)	3780 (2263-7630)	1 (0-1)	34 (20-69)	0 (0-0)	185 (29-311)	0 (0-0)	2 (0-3)	0 (0-0)	63 (10-105)	0 (0-0)	1 (0-1)	0 (0-0)
2000	Poland	2924 (2670-4778)		149 (136-243)		908 (544-1833)		46 (28-93)		180 (80-333)		9 (4-17)		61 (27-112)		3 (1-6)	
2001	Poland									162 (72-299)		9 (4-16)		54 (24-101)		3 (1-5)	
2002	Poland									116 (51-214)		6 (3-12)		39 (17-72)		2 (1-4)	
2003	Poland									127 (56-234)		7 (3-13)		43 (19-79)		2 (1-5)	
2004	Poland									106 (47-197)		6 (3-11)		36 (16-66)		2 (1-4)	
2005	Poland									99 (44-182)		6 (3-10)		33 (15-61)		2 (1-4)	
2006	Poland									71 (31-131)		4 (2-7)		24 (11-44)		1 (1-2)	
2007	Poland									12 (5-23)		1 (0-1)		4 (2-8)		0 (0-0)	
2008	Poland									12 (5-22)		1 (0-1)		4 (2-8)		0 (0-0)	
2009	Poland									9 (4-16)		0 (0-1)		3 (1-5)		0 (0-0)	
2010	Poland									1 (1-2)		0 (0-0)		0 (0-1)		0 (0-0)	
2011	Poland									1 (1-2)		0 (0-0)		0 (0-1)		0 (0-0)	
2012	Poland									1 (0-2)		0 (0-0)		0 (0-1)		0 (0-0)	
2013	Poland									1 (0-2)		0 (0-0)		0 (0-1)		0 (0-0)	
2014	Poland									1 (0-2)		0 (0-0)		0 (0-1)		0 (0-0)	
2015	Poland	29 (27-48)	0 (0-0)	1 (1-2)	0 (0-0)	9 (5-18)	0 (0-0)	0 (0-1)	0 (0-0)	1 (0-2)	0 (0-0)	0 (0-0)	0 (0-0)	0 (0-1)	0 (0-0)	0 (0-0)	0 (0-0)
2000	Portugal	34 (31-56)		7 (6-11)		11 (6-21)		2 (1-4)		18 (8-33)		3 (2-6)		6 (3-11)		1 (1-2)	
2001	Portugal									5 (2-9)		1 (0-2)		2 (1-3)		0 (0-1)	
2002	Portugal									13 (6-24)		2 (1-4)		4 (2-8)		1 (0-2)	
2003	Portugal									1 (1-2)		0 (0-0)		0 (0-1)		0 (0-0)	
2004	Portugal									1 (0-1)		0 (0-0)		0 (0-0)		0 (0-0)	
2005	Portugal									1 (0-1)		0 (0-0)		0 (0-0)		0 (0-0)	
2006	Portugal									1 (0-1)		0 (0-0)		0 (0-0)		0 (0-0)	
2007	Portugal									0 (0-1)		0 (0-0)		0 (0-0)		0 (0-0)	
2008	Portugal									0 (0-0)		0 (0-0)		0 (0-0)		0 (0-0)	
2009	Portugal									0 (0-0)		0 (0-0)		0 (0-0)		0 (0-0)	
2010	Portugal									0 (0-1)		0 (0-0)		0 (0-0)		0 (0-0)	
2011	Portugal									0 (0-1)		0 (0-0)		0 (0-0)		0 (0-0)	
2012	Portugal									0 (0-0)		0 (0-0)		0 (0-0)		0 (0-0)	
2013	Portugal									0 (0-0)		0 (0-0)		0 (0-0)		0 (0-0)	
2014	Portugal									0 (0-0)		0 (0-0)		0 (0-0)		0 (0-0)	
2015	Portugal	3 (3-5)	0 (0-0)	1 (1-1)	0 (0-0)	1 (1-2)	0 (0-0)	0 (0-0)	0 (0-0)	0 (0-0)	0 (0-0)	0 (0-0)	0 (0-0)	0 (0-0)	0 (0-0)	0 (0-0)	0 (0-0)
2000	Qatar	80 (73-130)		146 (133-239)		15 (9-30)		28 (16-56)		1 (0-1)		2 (1-3)		0 (0-0)		1 (0-1)	
2001	Qatar									1 (0-1)		1 (1-2)		0 (0-0)		0 (0-1)	
2002	Qatar									0 (0-0)		0 (0-0)		0 (0-0)		0 (0-0)	
2003	Qatar									0 (0-0)		0 (0-0)		0 (0-0)		0 (0-0)	

Hib = *Haemophilus influenzae* type b; npnm = non-pneumonia, non-meningitis; HIV = cases only in HIV-infected children; Uncertainty range provided in parentheses.

Year	Country	Hib pneumonia cases (UR)	Hib pneumonia cases, HIV (UR)	Hib pneumonia incidence rate (UR)	Hib pneumonia incidence rate, HIV (UR)	Hib severe pneumonia cases (UR)	Hib severe pneumonia cases, HIV (UR)	Hib severe pneumonia incidence rate (UR)	Hib severe pneumonia incidence rate, HIV (UR)	Hib meningitis cases (UR)	Hib meningitis cases, HIV (UR)	Hib meningitis incidence rate (UR)	Hib meningitis incidence rate, HIV (UR)	Hib NPNM cases (UR)	Hib NPNM cases, HIV (UR)	Hib NPNM incidence rate (UR)	Hib NPNM incidence rate, HIV (UR)
2004	Qatar									0 (0-0)		0 (0-0)		0 (0-0)		0 (0-0)	
2005	Qatar									0 (0-0)		0 (0-0)		0 (0-0)		0 (0-0)	
2006	Qatar									0 (0-0)		0 (0-0)		0 (0-0)		0 (0-0)	
2007	Qatar									0 (0-0)		0 (0-0)		0 (0-0)		0 (0-0)	
2008	Qatar									0 (0-0)		0 (0-0)		0 (0-0)		0 (0-0)	
2009	Qatar									0 (0-0)		0 (0-0)		0 (0-0)		0 (0-0)	
2010	Qatar									0 (0-0)		0 (0-0)		0 (0-0)		0 (0-0)	
2011	Qatar									0 (0-0)		0 (0-0)		0 (0-0)		0 (0-0)	
2012	Qatar									0 (0-0)		0 (0-0)		0 (0-0)		0 (0-0)	
2013	Qatar									0 (0-0)		0 (0-0)		0 (0-0)		0 (0-0)	
2014	Qatar									0 (0-0)		0 (0-0)		0 (0-0)		0 (0-0)	
2015	Qatar	9 (8-15)	0 (0-0)	7 (6-11)	0 (0-0)	2 (1-3)	0 (0-0)	1 (1-3)	0 (0-0)	0 (0-0)	0 (0-0)	0 (0-0)	0 (0-0)	0 (0-0)	0 (0-0)	0 (0-0)	0 (0-0)
2000	Republic of Korea	39477 (36044-64502)		1277 (1166-2086)		7391 (4425-14918)		239 (143-482)		269 (136-449)		9 (4-15)		91 (46-152)		3 (1-5)	
2001	Republic of Korea									227 (115-380)		8 (4-13)		77 (39-128)		3 (1-4)	
2002	Republic of Korea									230 (117-385)		8 (4-14)		78 (39-130)		3 (1-5)	
2003	Republic of Korea									177 (90-296)		7 (3-11)		60 (30-100)		2 (1-4)	
2004	Republic of Korea									194 (99-325)		7 (4-12)		66 (33-109)		2 (1-4)	
2005	Republic of Korea									144 (73-240)		5 (3-9)		48 (25-81)		2 (1-3)	
2006	Republic of Korea									195 (99-325)		8 (4-13)		66 (33-110)		3 (1-4)	
2007	Republic of Korea									230 (117-384)		9 (5-16)		78 (39-130)		3 (2-5)	
2008	Republic of Korea									154 (78-257)		6 (3-11)		52 (26-87)		2 (1-4)	
2009	Republic of Korea									162 (82-270)		7 (4-12)		54 (28-91)		2 (1-4)	
2010	Republic of Korea									163 (82-272)		7 (4-12)		55 (28-92)		2 (1-4)	
2011	Republic of Korea									203 (103-340)		9 (5-15)		69 (35-114)		3 (2-5)	
2012	Republic of Korea									149 (76-249)		7 (3-11)		50 (26-84)		2 (1-4)	
2013	Republic of Korea									147 (74-245)		6 (3-11)		49 (25-83)		2 (1-4)	
2014	Republic of Korea									26 (13-44)		1 (1-2)		9 (4-15)		0 (0-1)	
2015	Republic of Korea	1009 (921-1648)	0 (0-0)	45 (41-73)	0 (0-0)	188 (113-380)	0 (0-0)	8 (5-17)	0 (0-0)	15 (8-26)	0 (0-0)	1 (0-1)	0 (0-0)	5 (3-9)	0 (0-0)	0 (0-0)	0 (0-0)
2000	Republic of Moldova	706 (644-1153)		298 (272-486)		219 (131-442)		92 (55-187)		21 (8-41)		9 (4-17)		7 (3-14)		3 (1-6)	
2001	Republic of Moldova									8 (1-17)		4 (1-7)		3 (0-6)		1 (0-2)	
2002	Republic of Moldova									11 (1-24)		5 (1-11)		4 (0-8)		2 (0-4)	
2003	Republic of Moldova									9 (1-20)		4 (1-9)		3 (0-7)		1 (0-3)	
2004	Republic of Moldova									8 (1-19)		4 (0-9)		3 (0-6)		1 (0-3)	
2005	Republic of Moldova									11 (1-26)		6 (1-13)		4 (0-9)		2 (0-4)	
2006	Republic of Moldova									3 (0-6)		1 (0-3)		1 (0-2)		0 (0-1)	
2007	Republic of Moldova									5 (1-11)		2 (0-5)		2 (0-4)		1 (0-2)	
2008	Republic of Moldova									7 (1-15)		3 (0-7)		2 (0-5)		1 (0-2)	
2009	Republic of Moldova									6 (1-14)		3 (0-6)		2 (0-5)		1 (0-2)	
2010	Republic of Moldova									2 (0-3)		1 (0-2)		1 (0-1)		0 (0-1)	
2011	Republic of Moldova									2 (0-4)		1 (0-2)		1 (0-1)		0 (0-1)	
2012	Republic of Moldova									1 (0-2)		0 (0-1)		0 (0-1)		0 (0-0)	
2013	Republic of Moldova									1 (0-1)		0 (0-1)		0 (0-0)		0 (0-0)	
2014	Republic of Moldova									0 (0-0)		0 (0-0)		0 (0-0)		0 (0-0)	
2015	Republic of Moldova	7 (6-11)	0 (0-0)	3 (3-5)	0 (0-0)	2 (1-4)	0 (0-0)	1 (1-2)	0 (0-0)	0 (0-0)	0 (0-0)	0 (0-0)	0 (0-0)	0 (0-0)	0 (0-0)	0 (0-0)	0 (0-0)
2000	Romania	1639 (1497-2678)		149 (136-243)		509 (305-1028)		46 (28-93)		784 (347-1451)		71 (32-132)		264 (117-489)		24 (11-44)	
2001	Romania									771 (342-1428)		67 (30-124)		260 (115-481)		23 (10-42)	
2002	Romania									798 (353-1477)		70 (31-129)		269 (119-498)		23 (10-43)	
2003	Romania									504 (223-933)		45 (20-84)		170 (75-314)		15 (7-28)	
2004	Romania									364 (161-674)		34 (15-63)		123 (54-227)		12 (5-21)	
2005	Romania									325 (144-602)		31 (14-57)		110 (49-203)		10 (5-19)	
2006	Romania									293 (130-543)		28 (13-53)		99 (44-183)		10 (4-18)	
2007	Romania									280 (124-519)		27 (12-50)		94 (42-175)		9 (4-17)	
2008	Romania									185 (82-342)		18 (8-33)		62 (28-115)		6 (3-11)	
2009	Romania									168 (74-311)		16 (7-30)		57 (25-105)		5 (2-10)	
2010	Romania									194 (86-359)		19 (8-35)		65 (29-121)		6 (3-12)	
2011	Romania									39 (17-72)		4 (2-7)		13 (6-24)		1 (1-2)	
2012	Romania									10 (4-18)		1 (0-2)		3 (1-6)		0 (0-1)	
2013	Romania									13 (6-25)		1 (1-3)		4 (2-8)		0 (0-1)	
2014	Romania									12 (5-22)		1 (1-2)		4 (2-8)		0 (0-1)	
2015	Romania	105 (96-172)	0 (0-0)	12 (11-19)	0 (0-0)	33 (20-66)	0 (0-0)	4 (2-7)	0 (0-0)	11 (5-21)	0 (0-0)	1 (1-2)	0 (0-0)	4 (2-7)	0 (0-0)	0 (0-1)	0 (0-0)
2000	Russian Federation	18834 (17195-30772)		298 (272-486)		5850 (3503-11807)		92 (55-187)		3485 (1544-6451)		55 (24-102)		1175 (520-2175)		19 (8-34)	
2001	Russian Federation									3317 (1470-6141)		50 (22-93)		1118 (495-2070)		17 (8-32)	
2002	Russian Federation									3040 (1347-5627)		46 (20-84)		1025 (454-1897)		15 (7-28)	
2003	Russian Federation									2700 (1196-4999)		40 (18-75)		910 (403-1685)		14 (6-25)	
2004	Russian Federation									2443 (1082-4523)		36 (16-67)		824 (365-1525)		12 (5-23)	
2005	Russian Federation									2211 (980-4094)		32 (14-60)		745 (330-1380)		11 (5-20)	
2006	Russian Federation									1975 (875-3655)		28 (12-52)		666 (295-1232)		9 (4-17)	
2007	Russian Federation									1763 (781-3264)		24 (11-45)		594 (263-1100)		8 (4-15)	
2008	Russian Federation									1589 (704-2942)		21 (9-40)		536 (237-992)		7 (3-13)	
2009	Russian Federation									1481 (656-2741)		19 (9-36)		499 (221-924)		7 (3-12)	
2010	Russian Federation									1389 (615-2571)		18 (8-33)		468 (207-867)		6 (3-11)	
2011	Russian Federation									1298 (575-2403)		16 (7-30)		438 (194-810)		5 (2-10)	
2012	Russian Federation									1443 (639-2671)		17 (8-32)		486 (216-901)		6 (3-11)	
2013	Russian Federation									1431 (634-2649)		17 (7-31)		482 (214-893)		6 (2-10)	
2014	Russian Federation									1367 (605-2530)		15 (7-29)		461 (204-853)		5 (2-10)	
2015	Russian Federation	26826 (24493-43831)	43 (40-71)	298 (272-486)	0 (0-0)	8332 (4989-16818)	13 (8-27)	92 (55-187)	0 (0-0)	1304 (578-2415)	2 (1-4)	14 (6-27)	0 (0-0)	440 (195-814)	1 (0-1)	5 (2-9)	0 (0-0)
2000	Rwanda	19474 (17780-31819)		1484 (1355-2425)		3641 (2180-7348)		277 (166-560)		1175 (506-1781)		90 (39-136)		202 (87-307)		15 (7-23)	
2001	Rwanda									1208 (524-1827)		85 (37-128)		208 (90-315)		15 (6-22)	
2002	Rwanda									171 (75-258)		12 (5-18)		29 (13-44)		2 (1-3)	
2003	Rwanda									69 (20-115)		5 (1-8)		12 (3-20)		1 (0-1)	
2004	Rwanda									70 (20-116)		5 (1-8)		12 (3-20)		1 (0-1)	
2005	Rwanda									54 (16-88)		4 (1-6)		9 (3-15)		1 (0-1)	
2006	Rwanda									39 (11-64)		3 (1-4)		7 (2-11)		0 (0-1)	
2007	Rwanda									6 (2-10)		0 (0-1)		1 (0-2)		0 (0-0)	
2008	Rwanda									6 (2-9)		0 (0-1)		1 (0-2)		0 (0-0)	
2009	Rwanda									5 (1-9)		0 (0-1)		2 (1-3)		0 (0-0)	
2010	Rwanda									5 (2-8)		0 (0-0)		2 (1-3)		0 (0-0)	
2011	Rwanda									5 (1-8)		0 (0-0)		2 (0-3)		0 (0-0)	
2012	Rwanda									4 (1-7)		0 (0-0)		1 (0-2)		0 (0-0)	
2013	Rwanda									4 (1-6)		0 (0-0)		1 (0-2)		0 (0-0)	
2014	Rwanda									3 (1-6)		0 (0-0)		1 (0-2)		0 (0-0)	
2015	Rwanda	122 (111-199)	0 (0-1)	7 (7-12)	0 (0-0)	23 (14-46)	0 (0-0)	1 (1-3)	0 (0-0)	3 (1-5)	0 (0-0)	0 (0-0)	0 (0-0)	1 (0-2)	0 (0-0)	0 (0-0)	0 (0-0)
2000	Saint Kitts and Nevis	40 (36-65)		804 (734-1313)		7 (4-15)		150 (90-302)		0 (0-0)		0 (0-0)		0 (0-0)		0 (0-0)	
2001	Saint Kitts and Nevis									0 (0-0)		0 (0-0)		0 (0-0)		0 (0-0)	
2002	Saint Kitts and Nevis									0 (0-0)		0 (0-0)		0 (0-0)		0 (0-0)	
2003	Saint Kitts and Nevis									0 (0-0)		0 (0-0)		0 (0-0)		0 (0-0)	
2004	Saint Kitts and Nevis									0 (0-0)		0 (0-0)		0 (0-0)		0 (0-0)	
2005	Saint Kitts and Nevis									0 (0-0)		0 (0-0)		0 (0-0)		0 (0-0)	
2006	Saint Kitts and Nevis									0 (0-0)		0 (0-0)		0 (0-0)		0 (0-0)	
2007	Saint Kitts and Nevis									0 (0-0)		0 (0-0)		0 (0-0)		0 (0-0)	
2008	Saint Kitts and Nevis									0 (0-0)		0 (0-0)		0 (0-0)		0 (0-0)	
2009	Saint Kitts and Nevis									0 (0-0)		0 (0-0)		0 (0-0)		0 (0-0)	
2010	Saint Kitts and Nevis									0 (0-0)		0 (0-0)		0 (0-0)		0 (0-0)	
2011	Saint Kitts and Nevis									0 (0-0)		1 (0-1)		0 (0-0)		0 (0-0)	
2012	Saint Kitts and Nevis									0 (0-0)		1 (0-1)		0 (0-0)		0 (0-0)	
2013	Saint Kitts and Nevis									0 (0-0)		1 (0-2)		0 (0-0)		0 (0-1)	
2014	Saint Kitts and Nevis									0 (0-0)		1 (0-1)		0 (0-0)		0 (0-0)	
2015	Saint Kitts and Nevis	0 (0-0)	0 (0-0)	6 (5-10)	0 (0-0)	0 (0-0)	0 (0-0)	1 (1-2)	0 (0-0)	0 (0-0)	0 (0-0)	1 (0-1)	0 (0-0)	0 (0-0)	0 (0-0)	0 (0-0)	0 (0-0)
2000	Saint Lucia	147 (134-240)		944 (862-1543)		27 (16-55)		176 (105-355)		2 (1-4)		15 (7-26)		1 (0-1)		5 (2-9)	
2001	Saint Lucia									2 (1-3)		10 (5-18)		1 (0-1)		3 (2-6)	
2002	Saint Lucia									2 (1-4)		16 (7-28)		1 (0-1)		5 (2-10)	
2003	Saint Lucia									0 (0-0)		2 (1-3)		0 (0-0)		1 (0-1)	
2004	Saint Lucia									0 (0-0)		1 (1-3)		0 (0-0)		0 (0-1)	
2005	Saint Lucia									0 (0-0)		0 (0-0)		0 (0-0)		0 (0-0)	
2006	Saint Lucia									0 (0-0)		1 (0-1)		0 (0-0)		0 (0-0)	
2007	Saint Lucia									0 (0-0)		0 (0-0)		0 (0-0)		0 (0-0)	
2008	Saint Lucia									0 (0-0)		0 (0-0)		0 (0-0)		0 (0-0)	
2009	Saint Lucia									0 (0-0)		0 (0-1)		0 (0-0)		0 (0-0)	
2010	Saint Lucia									0 (0-0)		0 (0-1)		0 (0-0)		0 (0-0)	
2011	Saint Lucia									0 (0-0)		0 (0-1)		0 (0-0)		0 (0-0)	
2012	Saint Lucia									0 (0-0)		0 (0-0)		0 (0-0)		0 (0-0)	
2013	Saint Lucia									0 (0-0)		0 (0-0)		0 (0-0)		0 (0-0)	
2014	Saint Lucia									0 (0-0)		0 (0-1)		0 (0-0)		0 (0-0)	
2015	Saint Lucia	1 (1-1)	0 (0-0)	6 (5-10)	0 (0-0)	0 (0-0)	0 (0-0)	1 (1-2)	0 (0-0)	0 (0-0)	0 (0-0)	0 (0-0)	0 (0-0)	0 (0-0)	0 (0-0)	0 (0-0)	0 (0-0)
2000	Saint Vincent and the Grenadines	101 (93-166)		943 (861-1541)		19 (11-38)		176 (105-355)		3 (1-5)		26 (11-46)		1 (0-2)		9 (4-16)	
2001	Saint Vincent and the Grenadines									2 (1-3)		18 (8-32)		1 (0-1)		6 (3-11)	
2002	Saint Vincent and the Grenadines									0 (0-0)		0 (0-0)		0 (0-0)		0 (0-0)	
2003	Saint Vincent and the Grenadines									0 (0-0)		0 (0-0)		0 (0-0)		0 (0-0)	
2004	Saint Vincent and the Grenadines									0 (0-0)		0 (0-0)		0 (0-0)		0 (0-0)	
2005	Saint Vincent and the Grenadines									0 (0-0)		0 (0-0)		0 (0-0)		0 (0-0)	
2006	Saint Vincent and the Grenadines									0 (0-0)		0 (0-0)		0 (0-0)		0 (0-0)	
2007	Saint Vincent and the Grenadines									0 (0-0)		0 (0-0)		0 (0-0)		0 (0-0)	
2008	Saint Vincent and the Grenadines									0 (0-0)		0 (0-0)		0 (0-0)		0 (0-0)	
2009	Saint Vincent and the Grenadines									0 (0-0)		0 (0-0)		0 (0-0)		0 (0-0)	
2010	Saint Vincent and the Grenadines									0 (0-0)		0 (0-0)		0 (0-0)		0 (0-0)	
2011	Saint Vincent and the Grenadines									0 (0-0)		0 (0-0)		0 (0-0)		0 (0-0)	
2012	Saint Vincent and the Grenadines									0 (0-0)		0 (0-0)		0 (0-0)		0 (0-0)	
2013	Saint Vincent and the Grenadines									0 (0-0)		0 (0-0)		0 (0-0)		0 (0-0)	
2014	Saint Vincent and the Grenadines									0 (0-0)		0 (0-0)		0 (0-0)		0 (0-0)	
2015	Saint Vincent and the Grenadines	1 (0-1)	0 (0-0)	6 (5-10)	0 (0-0)	0 (0-0)	0 (0-0)	1 (1-2)	0 (0-0)	0 (0-0)	0 (0-0)	0 (0-0)	0 (0-0)	0 (0-0)	0 (0-0)	0 (0-0)	0 (0-0)
2000	Samoa	323 (295-527)		1282 (1171-2095)		61 (36-122)		241 (144-486)		16 (9-22)		62 (36-88)		5 (3-7)		21 (12-30)	
2001	Samoa									14 (8-20)		58 (34-82)		5 (3-7)		20 (11-28)	
2002	Samoa									13 (8-19)		55 (32-77)		5 (3-6)		18 (11-26)	
2003	Samoa									13 (7-18)		52 (30-73)		4 (2-6)		17 (10-24)	
2004	Samoa									12 (7-16)		47 (27-66)		4 (2-5)		16 (9-22)	
2005	Samoa									11 (6-15)		43 (25-61)		4 (2-5)		15 (8-21)	
2006	Samoa									10 (6-14)		40 (23-56)		3 (2-5)		13 (8-19)	
2007	Samoa									10 (6-14)		39 (23-55)		3 (2-5)		13 (8-19)	
2008	Samoa									12 (7-16)		46 (27-65)		4 (2-6)		16 (9-22)	
2009	Samoa									9 (5-13)		36 (21-51)		3 (2-4)		12 (7-17)	
2010	Samoa									3 (2-4)		12 (7-17)		1 (1-1)		4 (2-6)	
2011	Samoa									3 (2-4)		12 (7-17)		1 (1-1)		4 (2-6)	
2012	Samoa									3 (2-4)		10 (6-15)		1 (1-1)		3 (2-5)	
2013	Samoa									3 (1-4)		10 (6-14)		1 (0-1)		3 (2-5)	
2014	Samoa									2 (1-4)		10 (6-14)		1 (0-1)		3 (2-5)	
2015	Samoa	23 (21-38)	0 (0-0)	97 (89-159)	0 (0-0)	4 (3-9)	0 (0-0)	18 (11-36)	0 (0-0)	2 (1-3)	0 (0-0)	9 (5-13)	0 (0-0)	1 (0-1)	0 (0-0)	3 (2-4)	0 (0-0)
2000	San Marino	0 (0-0)		1 (1-1)		0 (0-0)		0 (0-0)		0 (0-0)		0 (0-0)		0 (0-0)		0 (0-0)	
2001	San Marino									0 (0-0)		0 (0-0)		0 (0-0)		0 (0-0)	
2002	San Marino									0 (0-0)		0 (0-0)		0 (0-0)		0 (0-0)	
2003	San Marino									0 (0-0)		0 (0-0)		0 (0-0)		0 (0-0)	
2004	San Marino									0 (0-0)		0 (0-0)		0 (0-0)		0 (0-0)	
2005	San Marino									0 (0-0)		0 (0-0)		0 (0-0)		0 (0-0)	
2006	San Marino									0 (0-0)		0 (0-0)		0 (0-0)		0 (0-0)	
2007	San Marino									0 (0-0)		0 (0-0)		0 (0-0)		0 (0-0)	
2008	San Marino									0 (0-0)		0 (0-0)		0 (0-0)		0 (0-0)	
2009	San Marino									0 (0-0)		0 (0-0)		0 (0-0)		0 (0-0)	
2010	San Marino									0 (0-0)		0 (0-0)		0 (0-0)		0 (0-0)	
2011	San Marino									0 (0-0)		0 (0-0)		0 (0-0)		0 (0-0)	
2012	San Marino									0 (0-0)		0 (0-0)		0 (0-0)		0 (0-0)	

Hib = *Haemophilus influenzae* type b; npnm = non-pneumonia, non-meningitis; HIV = cases only in HIV-infected children; Uncertainty range provided in parentheses.

Year	Country	Hib pneumonia cases (UR)	Hib pneumonia cases, HIV (UR)	Hib pneumonia incidence rate (UR)	Hib pneumonia incidence rate, HIV (UR)	Hib severe pneumonia cases (UR)	Hib severe pneumonia cases, HIV (UR)	Hib severe pneumonia incidence rate (UR)	Hib severe pneumonia incidence rate, HIV (UR)	Hib meningitis cases (UR)	Hib meningitis cases, HIV (UR)	Hib meningitis incidence rate (UR)	Hib meningitis incidence rate, HIV (UR)	Hib NPNM cases (UR)	Hib NPNM cases, HIV (UR)	Hib NPNM incidence rate (UR)	Hib NPNM incidence rate, HIV (UR)
2013	San Marino									0 (0-0)		0 (0-0)		0 (0-0)		0 (0-0)	
2014	San Marino									0 (0-0)		0 (0-0)		0 (0-0)		0 (0-0)	
2015	San Marino	0 (0-0)	0 (0-0)	1 (1-1)	0 (0-0)	0 (0-0)	0 (0-0)	0 (0-0)	0 (0-0)	0 (0-0)	0 (0-0)	0 (0-0)	0 (0-0)	0 (0-0)	0 (0-0)	0 (0-0)	0 (0-0)
2000	Sao Tome and Principe	349 (318-570)		1580 (1442-2581)		66 (39-133)		298 (178-601)		9 (3-14)		41 (14-65)		2 (1-2)		7 (2-11)	
2001	Sao Tome and Principe									9 (3-15)		40 (14-63)		2 (1-3)		7 (2-11)	
2002	Sao Tome and Principe									9 (3-15)		39 (14-62)		2 (1-3)		7 (2-11)	
2003	Sao Tome and Principe									9 (3-15)		38 (14-61)		2 (1-3)		7 (2-10)	
2004	Sao Tome and Principe									9 (3-15)		38 (13-62)		3 (1-5)		13 (4-21)	
2005	Sao Tome and Principe									10 (3-16)		38 (14-61)		3 (1-5)		13 (5-21)	
2006	Sao Tome and Principe									10 (4-16)		38 (14-60)		3 (1-5)		13 (5-20)	
2007	Sao Tome and Principe									10 (4-15)		37 (15-58)		3 (1-5)		13 (5-20)	
2008	Sao Tome and Principe									10 (4-16)		38 (16-59)		3 (1-5)		13 (5-20)	
2009	Sao Tome and Principe									10 (4-15)		36 (15-56)		3 (1-5)		12 (5-19)	
2010	Sao Tome and Principe									1 (1-2)		5 (2-8)		0 (0-1)		2 (1-3)	
2011	Sao Tome and Principe									1 (0-1)		2 (1-4)		0 (0-0)		1 (0-1)	
2012	Sao Tome and Principe									1 (0-1)		2 (1-4)		0 (0-0)		1 (0-1)	
2013	Sao Tome and Principe									1 (0-1)		2 (1-4)		0 (0-0)		1 (0-1)	
2014	Sao Tome and Principe									1 (0-1)		2 (1-4)		0 (0-0)		1 (0-1)	
2015	Sao Tome and Principe	3 (3-5)	0 (0-0)	10 (9-16)	0 (0-0)	1 (0-1)	0 (0-0)	2 (1-4)	0 (0-0)	0 (0-0)	0 (0-0)	0 (0-0)	0 (0-0)	0 (0-0)	0 (0-0)	0 (0-0)	0 (0-0)
2000	Saudi Arabia	41010 (37443-67007)		1487 (1358-2430)		7712 (4617-15565)		280 (167-564)		567 (287-946)		21 (10-34)		191 (97-319)		7 (4-12)	
2001	Saudi Arabia									527 (267-880)		19 (10-32)		178 (90-297)		6 (3-11)	
2002	Saudi Arabia									121 (61-202)		4 (2-7)		41 (21-68)		1 (1-2)	
2003	Saudi Arabia									55 (28-93)		2 (1-3)		19 (9-31)		1 (0-1)	
2004	Saudi Arabia									51 (26-85)		2 (1-3)		17 (9-29)		1 (0-1)	
2005	Saudi Arabia									49 (25-82)		2 (1-3)		16 (8-28)		1 (0-1)	
2006	Saudi Arabia									47 (24-78)		2 (1-3)		16 (8-26)		1 (0-1)	
2007	Saudi Arabia									7 (3-11)		0 (0-0)		2 (1-4)		0 (0-0)	
2008	Saudi Arabia									6 (3-11)		0 (0-0)		2 (1-4)		0 (0-0)	
2009	Saudi Arabia									7 (4-12)		0 (0-0)		2 (1-4)		0 (0-0)	
2010	Saudi Arabia									7 (4-12)		0 (0-0)		3 (1-4)		0 (0-0)	
2011	Saudi Arabia									8 (4-13)		0 (0-0)		3 (1-4)		0 (0-0)	
2012	Saudi Arabia									7 (4-12)		0 (0-0)		3 (1-4)		0 (0-0)	
2013	Saudi Arabia									7 (4-12)		0 (0-0)		2 (1-4)		0 (0-0)	
2014	Saudi Arabia									7 (4-12)		0 (0-0)		2 (1-4)		0 (0-0)	
2015	Saudi Arabia	216 (197-352)	0 (0-0)	7 (6-11)	0 (0-0)	40 (24-81)	0 (0-0)	1 (1-3)	0 (0-0)	7 (3-11)	0 (0-0)	0 (0-0)	0 (0-0)	2 (1-4)	0 (0-0)	0 (0-0)	0 (0-0)
2000	Senegal	22828 (20842-37299)		1379 (1259-2253)		4302 (2576-8683)		260 (156-524)		668 (194-1104)		40 (12-67)		115 (33-190)		7 (2-11)	
2001	Senegal									692 (206-1137)		41 (12-67)		119 (36-196)		7 (2-12)	
2002	Senegal									694 (213-1135)		40 (12-65)		120 (37-195)		7 (2-11)	
2003	Senegal									696 (220-1131)		39 (12-63)		120 (38-195)		7 (2-11)	
2004	Senegal									700 (229-1131)		38 (13-62)		121 (39-195)		7 (2-11)	
2005	Senegal									638 (215-1023)		34 (11-54)		110 (37-176)		6 (2-9)	
2006	Senegal									57 (19-92)		3 (1-5)		10 (3-16)		1 (0-1)	
2007	Senegal									50 (17-80)		3 (1-4)		9 (3-14)		0 (0-1)	
2008	Senegal									56 (19-89)		3 (1-4)		10 (3-15)		0 (0-1)	
2009	Senegal									57 (18-94)		3 (1-4)		19 (6-32)		1 (0-2)	
2010	Senegal									6 (2-11)		0 (0-0)		2 (1-4)		0 (0-0)	
2011	Senegal									6 (2-10)		0 (0-0)		2 (1-3)		0 (0-0)	
2012	Senegal									6 (2-10)		0 (0-0)		2 (1-3)		0 (0-0)	
2013	Senegal									6 (2-10)		0 (0-0)		2 (1-3)		0 (0-0)	
2014	Senegal									6 (2-10)		0 (0-0)		2 (1-3)		0 (0-0)	
2015	Senegal	305 (279-499)	1 (1-1)	12 (11-20)	0 (0-0)	57 (34-115)	0 (0-0)	2 (1-5)	0 (0-0)	6 (2-10)	0 (0-0)	0 (0-0)	0 (0-0)	2 (1-3)	0 (0-0)	0 (0-0)	0 (0-0)
2000	Serbia	908 (829-1483)		149 (136-243)		282 (169-569)		46 (28-93)		69 (25-121)		11 (4-20)		23 (8-41)		4 (1-7)	
2001	Serbia									56 (19-100)		9 (3-17)		19 (7-34)		3 (1-6)	
2002	Serbia									31 (10-56)		5 (2-10)		10 (3-19)		2 (1-3)	
2003	Serbia									45 (14-83)		8 (3-15)		15 (5-28)		3 (1-5)	
2004	Serbia									35 (11-65)		6 (2-12)		12 (4-22)		2 (1-4)	
2005	Serbia									48 (14-89)		9 (3-17)		16 (5-30)		3 (1-6)	
2006	Serbia									17 (5-32)		3 (1-6)		6 (2-11)		1 (0-2)	
2007	Serbia									3 (1-6)		1 (0-1)		1 (0-2)		0 (0-0)	
2008	Serbia									1 (0-1)		0 (0-0)		0 (0-0)		0 (0-0)	
2009	Serbia									2 (1-4)		0 (0-1)		1 (0-1)		0 (0-0)	
2010	Serbia									1 (0-1)		0 (0-0)		0 (0-0)		0 (0-0)	
2011	Serbia									0 (0-0)		0 (0-0)		0 (0-0)		0 (0-0)	
2012	Serbia									0 (0-0)		0 (0-0)		0 (0-0)		0 (0-0)	
2013	Serbia									0 (0-0)		0 (0-0)		0 (0-0)		0 (0-0)	
2014	Serbia									0 (0-0)		0 (0-0)		0 (0-0)		0 (0-0)	
2015	Serbia	7 (6-11)	0 (0-0)	1 (1-2)	0 (0-0)	2 (1-4)	0 (0-0)	0 (0-1)	0 (0-0)	0 (0-0)	0 (0-0)	0 (0-0)	0 (0-0)	0 (0-0)	0 (0-0)	0 (0-0)	0 (0-0)
2000	Seychelles	100 (91-163)		1389 (1268-2269)		19 (11-38)		262 (157-529)		1 (0-1)		12 (6-20)		0 (0-0)		4 (2-7)	
2001	Seychelles									1 (1-2)		14 (7-24)		0 (0-1)		5 (3-8)	
2002	Seychelles									1 (1-2)		14 (7-23)		0 (0-1)		5 (2-8)	
2003	Seychelles									1 (0-2)		13 (7-22)		0 (0-1)		4 (2-7)	
2004	Seychelles									1 (0-2)		12 (6-20)		0 (0-1)		4 (2-7)	
2005	Seychelles									1 (1-2)		13 (7-22)		0 (0-1)		5 (2-7)	
2006	Seychelles									1 (1-2)		13 (7-21)		0 (0-1)		4 (2-7)	
2007	Seychelles									1 (1-2)		12 (6-21)		0 (0-1)		4 (2-7)	
2008	Seychelles									1 (0-2)		12 (6-20)		0 (0-1)		4 (2-7)	
2009	Seychelles									1 (0-2)		12 (6-20)		0 (0-1)		4 (2-7)	
2010	Seychelles									0 (0-0)		3 (1-4)		0 (0-0)		1 (0-1)	
2011	Seychelles									0 (0-0)		1 (1-2)		0 (0-0)		0 (0-1)	
2012	Seychelles									0 (0-0)		1 (1-2)		0 (0-0)		0 (0-1)	
2013	Seychelles									0 (0-0)		1 (1-2)		0 (0-0)		0 (0-1)	
2014	Seychelles									0 (0-0)		1 (0-2)		0 (0-0)		0 (0-1)	
2015	Seychelles	1 (1-1)	0 (0-0)	11 (10-18)	0 (0-0)	0 (0-0)	0 (0-0)	2 (1-4)	0 (0-0)	0 (0-0)	0 (0-0)	0 (0-0)	0 (0-0)	0 (0-0)	0 (0-0)	0 (0-0)	0 (0-0)
2000	Sierra Leone	13172 (12027-21522)		1891 (1727-3090)		2472 (1480-4989)		355 (213-716)		620 (310-903)		89 (45-130)		107 (53-156)		15 (8-22)	
2001	Sierra Leone									629 (314-916)		86 (43-126)		108 (54-158)		15 (7-22)	
2002	Sierra Leone									641 (319-935)		84 (42-123)		110 (55-161)		15 (7-21)	
2003	Sierra Leone									659 (328-962)		83 (41-121)		114 (56-166)		14 (7-21)	
2004	Sierra Leone									676 (335-987)		81 (40-119)		116 (58-170)		14 (7-20)	
2005	Sierra Leone									680 (337-993)		79 (39-115)		117 (58-171)		14 (7-20)	
2006	Sierra Leone									684 (337-1000)		77 (38-112)		118 (58-172)		13 (7-19)	
2007	Sierra Leone									251 (124-369)		28 (14-40)		43 (21-64)		5 (2-7)	
2008	Sierra Leone									68 (34-101)		7 (4-11)		12 (6-17)		1 (1-2)	
2009	Sierra Leone									60 (32-87)		6 (3-9)		10 (5-15)		1 (1-2)	
2010	Sierra Leone									59 (34-82)		6 (4-9)		10 (6-14)		1 (1-1)	
2011	Sierra Leone									58 (33-81)		6 (3-8)		10 (6-14)		1 (1-1)	
2012	Sierra Leone									7 (3-11)		1 (0-1)		1 (1-2)		0 (0-0)	
2013	Sierra Leone									8 (3-12)		1 (0-1)		1 (1-2)		0 (0-0)	
2014	Sierra Leone									8 (3-12)		1 (0-1)		1 (1-2)		0 (0-0)	
2015	Sierra Leone	112 (102-183)	1 (1-1)	11 (10-19)	0 (0-0)	21 (13-42)	0 (0-0)	2 (1-4)	0 (0-0)	8 (3-12)	0 (0-0)	1 (0-1)	0 (0-0)	1 (1-2)	0 (0-0)	0 (0-0)	0 (0-0)
2000	Singapore	375 (342-612)		149 (136-243)		116 (70-235)		46 (28-93)		31 (16-51)		12 (6-20)		10 (5-17)		4 (2-7)	
2001	Singapore									12 (6-21)		5 (3-8)		4 (2-7)		2 (1-3)	
2002	Singapore									17 (9-29)		7 (4-12)		6 (3-10)		2 (1-4)	
2003	Singapore									0 (0-0)		0 (0-0)		0 (0-0)		0 (0-0)	
2004	Singapore									6 (3-11)		3 (1-4)		2 (1-4)		1 (0-1)	
2005	Singapore									8 (4-13)		3 (2-5)		3 (1-4)		1 (1-2)	
2006	Singapore									0 (0-0)		0 (0-0)		0 (0-0)		0 (0-0)	
2007	Singapore									9 (4-14)		3 (2-6)		3 (1-5)		1 (1-2)	
2008	Singapore									8 (4-14)		3 (2-6)		3 (1-5)		1 (1-2)	
2009	Singapore									15 (8-25)		6 (3-10)		5 (3-8)		2 (1-3)	
2010	Singapore									0 (0-0)		0 (0-0)		0 (0-0)		0 (0-0)	
2011	Singapore									11 (6-19)		4 (2-7)		4 (2-6)		1 (1-2)	
2012	Singapore									0 (0-0)		0 (0-0)		0 (0-0)		0 (0-0)	
2013	Singapore									0 (0-0)		0 (0-0)		0 (0-0)		0 (0-0)	
2014	Singapore									1 (0-1)		0 (0-0)		0 (0-0)		0 (0-0)	
2015	Singapore	35 (32-58)	0 (0-0)	13 (12-22)	0 (0-0)	11 (7-22)	0 (0-0)	4 (2-8)	0 (0-0)	1 (0-1)	0 (0-0)	0 (0-0)	0 (0-0)	0 (0-0)	0 (0-0)	0 (0-0)	0 (0-0)
2000	Slovakia	56 (51-92)		20 (18-33)		17 (10-35)		6 (4-13)		7 (3-13)		3 (1-5)		2 (1-4)		1 (0-2)	
2001	Slovakia									1 (0-2)		0 (0-1)		0 (0-1)		0 (0-0)	
2002	Slovakia									0 (0-1)		0 (0-0)		0 (0-0)		0 (0-0)	
2003	Slovakia									1 (0-1)		0 (0-0)		0 (0-0)		0 (0-0)	
2004	Slovakia									0 (0-1)		0 (0-0)		0 (0-0)		0 (0-0)	
2005	Slovakia									0 (0-1)		0 (0-0)		0 (0-0)		0 (0-0)	
2006	Slovakia									0 (0-1)		0 (0-0)		0 (0-0)		0 (0-0)	
2007	Slovakia									0 (0-0)		0 (0-0)		0 (0-0)		0 (0-0)	
2008	Slovakia									1 (0-1)		0 (0-0)		0 (0-0)		0 (0-0)	
2009	Slovakia									0 (0-1)		0 (0-0)		0 (0-0)		0 (0-0)	
2010	Slovakia									0 (0-1)		0 (0-0)		0 (0-0)		0 (0-0)	
2011	Slovakia									0 (0-1)		0 (0-0)		0 (0-0)		0 (0-0)	
2012	Slovakia									0 (0-1)		0 (0-0)		0 (0-0)		0 (0-0)	
2013	Slovakia									0 (0-0)		0 (0-0)		0 (0-0)		0 (0-0)	
2014	Slovakia									0 (0-1)		0 (0-0)		0 (0-0)		0 (0-0)	
2015	Slovakia	4 (4-7)	0 (0-0)	1 (1-2)	0 (0-0)	1 (1-3)	0 (0-0)	0 (0-1)	0 (0-0)	0 (0-1)	0 (0-0)	0 (0-0)	0 (0-0)	0 (0-0)	0 (0-0)	0 (0-0)	0 (0-0)
2000	Slovenia	66 (60-108)		74 (68-122)		21 (12-41)		23 (14-47)		0 (0-0)		0 (0-0)		0 (0-0)		0 (0-0)	
2001	Slovenia									0 (0-0)		0 (0-0)		0 (0-0)		0 (0-0)	
2002	Slovenia									1 (1-2)		1 (1-2)		0 (0-1)		0 (0-1)	
2003	Slovenia									0 (0-0)		0 (0-0)		0 (0-0)		0 (0-0)	
2004	Slovenia									0 (0-0)		0 (0-0)		0 (0-0)		0 (0-0)	
2005	Slovenia									0 (0-1)		0 (0-1)		0 (0-0)		0 (0-0)	
2006	Slovenia									0 (0-0)		0 (0-0)		0 (0-0)		0 (0-0)	
2007	Slovenia									0 (0-0)		0 (0-0)		0 (0-0)		0 (0-0)	
2008	Slovenia									0 (0-0)		0 (0-0)		0 (0-0)		0 (0-0)	
2009	Slovenia									0 (0-0)		0 (0-0)		0 (0-0)		0 (0-0)	
2010	Slovenia									0 (0-0)		0 (0-0)		0 (0-0)		0 (0-0)	
2011	Slovenia									0 (0-0)		0 (0-0)		0 (0-0)		0 (0-0)	
2012	Slovenia									0 (0-0)		0 (0-0)		0 (0-0)		0 (0-0)	
2013	Slovenia									0 (0-0)		0 (0-0)		0 (0-0)		0 (0-0)	
2014	Slovenia									0 (0-0)		0 (0-0)		0 (0-0)		0 (0-0)	
2015	Slovenia	1 (1-1)	0 (0-0)	1 (1-1)	0 (0-0)	0 (0-1)	0 (0-0)	0 (0-0)	0 (0-0)	0 (0-0)	0 (0-0)	0 (0-0)	0 (0-0)	0 (0-0)	0 (0-0)	0 (0-0)	0 (0-0)
2000	Solomon Islands	818 (747-1336)		1286 (1174-2100)		153 (92-309)		240 (144-485)		20 (11-26)		32 (17-41)		7 (4-9)		11 (6-14)	
2001	Solomon Islands									21 (11-27)		32 (17-42)		7 (4-9)		11 (6-14)	
2002	Solomon Islands									22 (11-28)		33 (17-43)		7 (4-10)		11 (6-14)	
2003	Solomon Islands									22 (12-29)		33 (17-43)		8 (4-10)		11 (6-14)	
2004	Solomon Islands									23 (12-30)		34 (18-43)		8 (4-10)		11 (6-15)	
2005	Solomon Islands									24 (13-31)		34 (18-43)		8 (4-10)		11 (6-15)	
2006	Solomon Islands									24 (13-32)		34 (18-43)		8 (4-11)		11 (6-15)	
2007	Solomon Islands									24 (13-31)		33 (17-42)		8 (4-11)		11 (6-14)	
2008	Solomon Islands									4 (2-5)		5 (3-7)		1 (1-2)		2 (1-2)	
2009	Solomon Islands									2 (1-2)		2 (1-3)		1 (0-1)		1 (0-1)	
2010	Solomon Islands									2 (1-3)		3 (1-3)		1 (0-1)		1 (0-1)	
2011	Solomon Islands									1 (1-2)		2 (1-2)		1 (0-1)		1 (0-1)	
2012	Solomon Islands									2 (1-2)		2 (1-2)		1 (0-1)		1 (0-1)	
2013	Solomon Islands									0 (0-0)		0 (0-0)		0 (0-0)		0 (0-0)	
2014	Solomon Islands									0 (0-0)		0 (0-0)		0 (0-0)		0 (0-0)	
2015	Solomon Islands	7 (6-12)	0 (0-0)	9 (8-14)	0 (0-0)	1 (1-3)	0 (0-0)	2 (1-3)	0 (0-0)	0 (0-0)	0 (0-0)	0 (0-0)	0 (0-0)	0 (0-0)	0 (0-0)	0 (0-0)	0 (0-0)
2000	Somalia	41212 (37627-67336)		2853 (2605-4662)		7673 (4594-15486)		531 (318-1072)		1011 (425-1539)		70 (29-107)		174 (73-265)		12 (5-18)	
2001	Somalia									1025 (431-1560)		68 (29-104)		176 (74-269)		12 (5-18)	
2002	Somalia									1062 (447-1617)		69 (29-106)		183 (77-279)		12 (5-18)	
2003	Somalia									1134 (477-1727)		73 (31-111)		195 (82-297)		13 (5-19)	
2004	Somalia									1166 (490-1775)		74 (31-113)		201 (84-306)		13 (5-19)	
2005	Somalia									1207 (508-1838)		75 (32-115)		208 (87-316)		13 (5-20)	

Hib = *Haemophilus influenzae* type b; npnm = non-pneumonia, non-meningitis; HIV = cases only in HIV-infected children; Uncertainty range provided in parentheses.

Year	Country	Hib pneumonia cases (UR)	Hib pneumonia cases, HIV (UR)	Hib pneumonia incidence rate (UR)	Hib pneumonia incidence rate, HIV (UR)	Hib severe pneumonia cases (UR)	Hib severe pneumonia cases, HIV (UR)	Hib severe pneumonia incidence rate (UR)	Hib severe pneumonia incidence rate, HIV (UR)	Hib meningitis cases (UR)	Hib meningitis cases, HIV (UR)	Hib meningitis incidence rate (UR)	Hib meningitis incidence rate, HIV (UR)	Hib NPNM cases (UR)	Hib NPNM cases, HIV (UR)	Hib NPNM incidence rate (UR)	Hib NPNM incidence rate, HIV (UR)
2006	Somalia									1252 (527-1906)		77 (32-117)		216 (91-328)		13 (6-20)	
2007	Somalia									1300 (547-1979)		78 (33-119)		224 (94-341)		13 (6-20)	
2008	Somalia									1337 (563-2035)		78 (33-119)		230 (97-350)		13 (6-21)	
2009	Somalia									1363 (575-2073)		78 (33-119)		235 (99-357)		13 (6-20)	
2010	Somalia									1375 (581-2092)		77 (33-117)		237 (100-360)		13 (6-20)	
2011	Somalia									1364 (577-2074)		75 (32-113)		235 (99-357)		13 (5-20)	
2012	Somalia									1340 (567-2037)		72 (31-110)		231 (98-351)		12 (5-19)	
2013	Somalia									827 (221-1382)		44 (12-74)		142 (38-238)		8 (2-13)	
2014	Somalia									210 (56-350)		11 (3-18)		36 (10-60)		2 (1-3)	
2015	Somalia	6989 (6381-11419)	54 (49-88)	361 (329-589)	3 (3-0)	1310 (785-2645)	10 (6-20)	68 (40-136)	1 (0-0)	207 (56-346)	2 (0-3)	11 (3-18)	0 (0-0)	36 (10-60)	0 (0-0)	2 (0-3)	0 (0-0)
2000	South Africa	28052 (25612-45834)		527 (481-861)		5242 (3139-10580)		98 (59-199)		291 (117-451)		5 (2-8)		50 (20-78)		1 (0-1)	
2001	South Africa									129 (51-200)		2 (1-4)		22 (9-34)		0 (0-1)	
2002	South Africa									124 (49-192)		2 (1-4)		21 (8-33)		0 (0-1)	
2003	South Africa									116 (46-180)		2 (1-3)		20 (8-31)		0 (0-1)	
2004	South Africa									116 (46-180)		2 (1-4)		20 (8-31)		0 (0-1)	
2005	South Africa									9 (4-14)		0 (0-0)		2 (1-2)		0 (0-0)	
2006	South Africa									9 (3-15)		0 (0-0)		3 (1-5)		0 (0-0)	
2007	South Africa									10 (4-15)		0 (0-0)		3 (1-5)		0 (0-0)	
2008	South Africa									10 (4-17)		0 (0-0)		4 (1-6)		0 (0-0)	
2009	South Africa									11 (4-17)		0 (0-0)		4 (1-6)		0 (0-0)	
2010	South Africa									11 (4-18)		0 (0-0)		4 (1-6)		0 (0-0)	
2011	South Africa									11 (4-18)		0 (0-0)		4 (1-6)		0 (0-0)	
2012	South Africa									11 (4-17)		0 (0-0)		4 (1-6)		0 (0-0)	
2013	South Africa									129 (52-202)		2 (1-4)		44 (17-68)		1 (0-1)	
2014	South Africa									10 (4-15)		0 (0-0)		3 (1-5)		0 (0-0)	
2015	South Africa	585 (535-957)	55 (50-89)	11 (10-18)	1 (1-0)	110 (66-222)	10 (6-21)	2 (1-4)	0 (0-0)	9 (4-14)	1 (0-1)	0 (0-0)	0 (0-0)	3 (1-5)	0 (0-0)	0 (0-0)	0 (0-0)
2000	South Sudan	0 (0-0)		0 (0-0)		0 (0-0)		0 (0-0)		668 (314-991)		57 (27-84)		115 (54-171)		10 (5-15)	
2001	South Sudan									671 (317-993)		55 (26-82)		116 (55-171)		9 (4-14)	
2002	South Sudan									654 (310-966)		52 (25-77)		113 (53-166)		9 (4-13)	
2003	South Sudan									639 (305-942)		50 (24-74)		110 (52-162)		9 (4-13)	
2004	South Sudan									632 (205-1025)		48 (16-78)		109 (35-176)		8 (3-13)	
2005	South Sudan									612 (200-989)		45 (15-73)		105 (34-170)		8 (3-13)	
2006	South Sudan									604 (199-974)		43 (14-70)		104 (34-168)		7 (2-12)	
2007	South Sudan									597 (198-962)		41 (14-66)		103 (34-166)		7 (2-11)	
2008	South Sudan									612 (205-984)		41 (14-65)		105 (35-169)		7 (2-11)	
2009	South Sudan									620 (209-996)		40 (13-64)		107 (36-172)		7 (2-11)	
2010	South Sudan									637 (216-1022)		39 (13-63)		110 (37-176)		7 (2-11)	
2011	South Sudan									658 (225-1054)		39 (13-62)		113 (39-181)		7 (2-11)	
2012	South Sudan									653 (224-1045)		37 (13-59)		112 (39-180)		6 (2-10)	
2013	South Sudan									674 (233-1077)		37 (13-59)		116 (40-186)		6 (2-10)	
2014	South Sudan									674 (235-1077)		36 (12-57)		116 (40-185)		6 (2-10)	
2015	South Sudan	13151 (12007-21487)	446 (407-728)	684 (624-1117)	23 (21-4)	2451 (1468-4947)	83 (50-168)	127 (76-257)	4 (3-1)	511 (179-814)	17 (6-28)	27 (9-42)	1 (0-0)	88 (31-140)	3 (1-5)	5 (2-7)	0 (0-0)
2000	Spain	107 (98-175)		6 (5-9)		33 (20-67)		2 (1-4)		32 (14-60)		2 (1-3)		11 (5-20)		1 (0-1)	
2001	Spain									18 (8-33)		1 (0-2)		6 (3-11)		0 (0-1)	
2002	Spain									17 (8-32)		1 (0-2)		6 (3-11)		0 (0-1)	
2003	Spain									3 (2-6)		0 (0-0)		1 (1-2)		0 (0-0)	
2004	Spain									3 (1-5)		0 (0-0)		1 (0-2)		0 (0-0)	
2005	Spain									4 (2-7)		0 (0-0)		1 (1-2)		0 (0-0)	
2006	Spain									3 (1-6)		0 (0-0)		1 (0-2)		0 (0-0)	
2007	Spain									3 (1-5)		0 (0-0)		1 (0-2)		0 (0-0)	
2008	Spain									2 (1-5)		0 (0-0)		1 (0-2)		0 (0-0)	
2009	Spain									3 (1-5)		0 (0-0)		1 (0-2)		0 (0-0)	
2010	Spain									2 (1-3)		0 (0-0)		1 (0-1)		0 (0-0)	
2011	Spain									2 (1-3)		0 (0-0)		1 (0-1)		0 (0-0)	
2012	Spain									1 (1-2)		0 (0-0)		0 (0-1)		0 (0-0)	
2013	Spain									2 (1-3)		0 (0-0)		1 (0-1)		0 (0-0)	
2014	Spain									1 (1-2)		0 (0-0)		0 (0-1)		0 (0-0)	
2015	Spain	16 (14-26)	0 (0-0)	1 (1-1)	0 (0-0)	5 (3-10)	0 (0-0)	0 (0-0)	0 (0-0)	1 (1-2)	0 (0-0)	0 (0-0)	0 (0-0)	0 (0-1)	0 (0-0)	0 (0-0)	0 (0-0)
2000	Sri Lanka	19168 (17500-31318)		1187 (1084-1940)		3586 (2147-7237)		222 (133-448)		43 (5-75)		3 (0-5)		15 (2-25)		1 (0-2)	
2001	Sri Lanka									41 (5-70)		3 (0-4)		14 (2-24)		1 (0-1)	
2002	Sri Lanka									38 (5-66)		2 (0-4)		13 (2-22)		1 (0-1)	
2003	Sri Lanka									38 (5-65)		2 (0-4)		13 (2-22)		1 (0-1)	
2004	Sri Lanka									28 (3-49)		2 (0-3)		9 (1-17)		1 (0-1)	
2005	Sri Lanka									35 (4-60)		2 (0-4)		12 (2-20)		1 (0-1)	
2006	Sri Lanka									33 (4-56)		2 (0-3)		11 (1-19)		1 (0-1)	
2007	Sri Lanka									30 (4-52)		2 (0-3)		10 (1-18)		1 (0-1)	
2008	Sri Lanka									29 (4-50)		2 (0-3)		10 (1-17)		1 (0-1)	
2009	Sri Lanka									27 (4-47)		2 (0-3)		9 (1-16)		1 (0-1)	
2010	Sri Lanka									8 (1-14)		0 (0-1)		3 (0-5)		0 (0-0)	
2011	Sri Lanka									3 (0-5)		0 (0-0)		1 (0-2)		0 (0-0)	
2012	Sri Lanka									3 (0-5)		0 (0-0)		1 (0-2)		0 (0-0)	
2013	Sri Lanka									3 (0-5)		0 (0-0)		1 (0-2)		0 (0-0)	
2014	Sri Lanka									3 (0-5)		0 (0-0)		1 (0-2)		0 (0-0)	
2015	Sri Lanka	147 (134-240)	0 (0-0)	9 (8-15)	0 (0-0)	28 (17-56)	0 (0-0)	2 (1-3)	0 (0-0)	0 (0-1)	0 (0-0)	0 (0-0)	0 (0-0)	0 (0-0)	0 (0-0)	0 (0-0)	0 (0-0)
2000	Sudan	44021 (40192-71926)		934 (853-1526)		8195 (4907-16541)		174 (104-351)		2862 (1258-4329)		61 (27-92)		493 (217-745)		10 (5-16)	
2001	Sudan									2870 (1237-4368)		60 (26-91)		494 (213-752)		10 (4-16)	
2002	Sudan									2785 (1177-4264)		57 (24-87)		480 (203-734)		10 (4-15)	
2003	Sudan									2697 (1118-4151)		54 (22-82)		464 (193-715)		9 (4-14)	
2004	Sudan									2576 (1047-3982)		50 (20-77)		444 (180-686)		9 (4-13)	
2005	Sudan									2386 (952-3705)		46 (18-71)		411 (164-638)		8 (3-12)	
2006	Sudan									2259 (885-3522)		42 (17-66)		389 (152-607)		7 (3-11)	
2007	Sudan									2128 (818-3330)		39 (15-61)		366 (141-573)		7 (3-11)	
2008	Sudan									394 (149-619)		7 (3-11)		68 (26-107)		1 (0-2)	
2009	Sudan									217 (81-343)		4 (1-6)		37 (14-59)		1 (0-1)	
2010	Sudan									197 (72-312)		4 (1-6)		34 (12-54)		1 (0-1)	
2011	Sudan									136 (49-216)		2 (1-4)		23 (8-37)		0 (0-1)	
2012	Sudan									132 (47-210)		2 (1-4)		23 (8-36)		0 (0-1)	
2013	Sudan									17 (5-28)		0 (0-0)		6 (2-9)		0 (0-0)	
2014	Sudan									16 (5-27)		0 (0-0)		6 (2-9)		0 (0-0)	
2015	Sudan	535 (489-875)	1 (1-2)	9 (8-15)	0 (0-0)	101 (60-203)	0 (0-1)	2 (1-3)	0 (0-0)	16 (5-27)	0 (0-0)	0 (0-0)	0 (0-0)	5 (2-9)	0 (0-0)	0 (0-0)	0 (0-0)
2000	Suriname	469 (428-766)		891 (814-1456)		87 (52-176)		166 (99-335)		7 (2-11)		13 (4-22)		2 (1-4)		4 (1-7)	
2001	Suriname									5 (2-9)		10 (3-17)		2 (1-3)		3 (1-6)	
2002	Suriname									13 (4-22)		26 (8-42)		4 (1-7)		9 (3-14)	
2003	Suriname									3 (1-5)		6 (2-11)		1 (0-2)		2 (1-4)	
2004	Suriname									7 (2-11)		13 (4-21)		2 (1-4)		4 (1-7)	
2005	Suriname									0 (0-1)		1 (0-2)		0 (0-0)		0 (0-1)	
2006	Suriname									1 (0-2)		3 (0-5)		0 (0-1)		1 (0-2)	
2007	Suriname									0 (0-1)		1 (0-1)		0 (0-0)		0 (0-0)	
2008	Suriname									1 (0-2)		2 (0-4)		0 (0-1)		1 (0-1)	
2009	Suriname									1 (0-2)		2 (0-4)		0 (0-1)		1 (0-1)	
2010	Suriname									0 (0-0)		1 (0-1)		0 (0-0)		0 (0-0)	
2011	Suriname									0 (0-0)		0 (0-0)		0 (0-0)		0 (0-0)	
2012	Suriname									0 (0-0)		0 (0-0)		0 (0-0)		0 (0-0)	
2013	Suriname									0 (0-0)		0 (0-0)		0 (0-0)		0 (0-0)	
2014	Suriname									0 (0-0)		0 (0-0)		0 (0-0)		0 (0-0)	
2015	Suriname	3 (3-5)	0 (0-0)	6 (5-10)	0 (0-0)	1 (0-1)	0 (0-0)	1 (1-2)	0 (0-0)	0 (0-0)	0 (0-0)	0 (0-0)	0 (0-0)	0 (0-0)	0 (0-0)	0 (0-0)	0 (0-0)
2000	Swaziland	3366 (3073-5500)		2182 (1992-3565)		633 (379-1278)		410 (246-828)		60 (23-95)		39 (15-62)		10 (4-16)		7 (3-11)	
2001	Swaziland									63 (25-99)		41 (16-64)		11 (4-17)		7 (3-11)	
2002	Swaziland									66 (26-102)		43 (17-67)		11 (4-18)		7 (3-12)	
2003	Swaziland									68 (28-106)		45 (18-69)		12 (5-18)		8 (3-12)	
2004	Swaziland									70 (29-108)		46 (19-71)		12 (5-19)		8 (3-12)	
2005	Swaziland									71 (30-109)		47 (20-71)		12 (5-19)		8 (3-12)	
2006	Swaziland									69 (30-105)		45 (20-68)		12 (5-18)		8 (3-12)	
2007	Swaziland									67 (29-101)		43 (19-65)		11 (5-17)		7 (3-11)	
2008	Swaziland									63 (26-96)		40 (16-61)		11 (4-17)		7 (3-11)	
2009	Swaziland									9 (4-14)		6 (2-9)		2 (1-2)		1 (0-2)	
2010	Swaziland									4 (2-7)		3 (1-4)		1 (0-1)		0 (0-1)	
2011	Swaziland									4 (2-6)		2 (1-4)		1 (0-1)		0 (0-1)	
2012	Swaziland									4 (1-6)		2 (1-4)		1 (0-2)		1 (0-1)	
2013	Swaziland									3 (1-5)		2 (1-3)		1 (0-2)		1 (0-1)	
2014	Swaziland									0 (0-1)		0 (0-0)		0 (0-0)		0 (0-0)	
2015	Swaziland	14 (13-23)	1 (1-2)	8 (8-14)	1 (1-0)	3 (2-5)	0 (0-1)	2 (1-3)	0 (0-0)	0 (0-1)	0 (0-0)	0 (0-0)	0 (0-0)	0 (0-0)	0 (0-0)	0 (0-0)	0 (0-0)
2000	Sweden	3 (3-6)		1 (1-1)		1 (1-2)		0 (0-0)		0 (0-0)		0 (0-0)		0 (0-0)		0 (0-0)	
2001	Sweden									0 (0-0)		0 (0-0)		0 (0-0)		0 (0-0)	
2002	Sweden									0 (0-1)		0 (0-0)		0 (0-0)		0 (0-0)	
2003	Sweden									0 (0-1)		0 (0-0)		0 (0-0)		0 (0-0)	
2004	Sweden									0 (0-1)		0 (0-0)		0 (0-0)		0 (0-0)	
2005	Sweden									0 (0-1)		0 (0-0)		0 (0-0)		0 (0-0)	
2006	Sweden									0 (0-0)		0 (0-0)		0 (0-0)		0 (0-0)	
2007	Sweden									0 (0-1)		0 (0-0)		0 (0-0)		0 (0-0)	
2008	Sweden									0 (0-0)		0 (0-0)		0 (0-0)		0 (0-0)	
2009	Sweden									0 (0-0)		0 (0-0)		0 (0-0)		0 (0-0)	
2010	Sweden									0 (0-1)		0 (0-0)		0 (0-0)		0 (0-0)	
2011	Sweden									0 (0-0)		0 (0-0)		0 (0-0)		0 (0-0)	
2012	Sweden									0 (0-0)		0 (0-0)		0 (0-0)		0 (0-0)	
2013	Sweden									0 (0-0)		0 (0-0)		0 (0-0)		0 (0-0)	
2014	Sweden									0 (0-0)		0 (0-0)		0 (0-0)		0 (0-0)	
2015	Sweden	4 (4-7)	0 (0-0)	1 (1-1)	0 (0-0)	1 (1-3)	0 (0-0)	0 (0-0)	0 (0-0)	0 (0-0)	0 (0-0)	0 (0-0)	0 (0-0)	0 (0-0)	0 (0-0)	0 (0-0)	0 (0-0)
2000	Switzerland	23 (21-38)		6 (5-10)		7 (4-15)		2 (1-4)		4 (2-7)		1 (0-2)		1 (1-2)		0 (0-1)	
2001	Switzerland									5 (2-10)		1 (1-3)		2 (1-3)		0 (0-1)	
2002	Switzerland									5 (2-10)		1 (1-3)		2 (1-3)		0 (0-1)	
2003	Switzerland									0 (0-1)		0 (0-0)		0 (0-0)		0 (0-0)	
2004	Switzerland									0 (0-0)		0 (0-0)		0 (0-0)		0 (0-0)	
2005	Switzerland									0 (0-1)		0 (0-0)		0 (0-0)		0 (0-0)	
2006	Switzerland									0 (0-1)		0 (0-0)		0 (0-0)		0 (0-0)	
2007	Switzerland									0 (0-0)		0 (0-0)		0 (0-0)		0 (0-0)	
2008	Switzerland									0 (0-0)		0 (0-0)		0 (0-0)		0 (0-0)	
2009	Switzerland									0 (0-1)		0 (0-0)		0 (0-0)		0 (0-0)	
2010	Switzerland									0 (0-0)		0 (0-0)		0 (0-0)		0 (0-0)	
2011	Switzerland									0 (0-0)		0 (0-0)		0 (0-0)		0 (0-0)	
2012	Switzerland									0 (0-0)		0 (0-0)		0 (0-0)		0 (0-0)	
2013	Switzerland									0 (0-0)		0 (0-0)		0 (0-0)		0 (0-0)	
2014	Switzerland									0 (0-0)		0 (0-0)		0 (0-0)		0 (0-0)	
2015	Switzerland	3 (3-5)	0 (0-0)	1 (1-1)	0 (0-0)	1 (1-2)	0 (0-0)	0 (0-0)	0 (0-0)	0 (0-0)	0 (0-0)	0 (0-0)	0 (0-0)	0 (0-0)	0 (0-0)	0 (0-0)	0 (0-0)
2000	Syrian Arab Republic	22315 (20374-36461)		946 (864-1546)		4158 (2489-8392)		176 (106-356)		74 (10-127)		3 (0-5)		25 (3-43)		1 (0-2)	
2001	Syrian Arab Republic									22 (3-38)		1 (0-2)		7 (1-13)		0 (0-1)	
2002	Syrian Arab Republic									13 (2-21)		1 (0-1)		4 (1-7)		0 (0-0)	
2003	Syrian Arab Republic									12 (2-21)		1 (0-1)		4 (1-7)		0 (0-0)	
2004	Syrian Arab Republic									12 (2-20)		1 (0-1)		4 (1-7)		0 (0-0)	
2005	Syrian Arab Republic									12 (2-21)		1 (0-1)		4 (1-7)		0 (0-0)	
2006	Syrian Arab Republic									1 (0-2)		0 (0-0)		0 (0-1)		0 (0-0)	
2007	Syrian Arab Republic									1 (0-2)		0 (0-0)		0 (0-1)		0 (0-0)	
2008	Syrian Arab Republic									1 (0-2)		0 (0-0)		1 (0-1)		0 (0-0)	
2009	Syrian Arab Republic									2 (0-3)		0 (0-0)		1 (0-1)		0 (0-0)	
2010	Syrian Arab Republic									2 (0-3)		0 (0-0)		1 (0-1)		0 (0-0)	
2011	Syrian Arab Republic									2 (0-2)		0 (0-0)		1 (0-1)		0 (0-0)	
2012	Syrian Arab Republic									19 (7-29)		1 (0-1)		6 (2-10)		0 (0-0)	
2013	Syrian Arab Republic									17 (7-26)		1 (0-1)		6 (2-9)		0 (0-0)	
2014	Syrian Arab Republic									16 (8-24)		1 (0-1)		5 (3-8)		0 (0-0)	

Hib = *Haemophilus influenzae* type b; npnm = non-pneumonia, non-meningitis; HIV = cases only in HIV-infected children; Uncertainty range provided in parentheses.

Year	Country	Hib pneumonia cases (UR)	Hib pneumonia cases, HIV (UR)	Hib pneumonia incidence rate (UR)	Hib pneumonia incidence rate, HIV (UR)	Hib severe pneumonia cases (UR)	Hib severe pneumonia cases, HIV (UR)	Hib severe pneumonia incidence rate (UR)	Hib severe pneumonia incidence rate, HIV (UR)	Hib meningitis cases (UR)	Hib meningitis cases, HIV (UR)	Hib meningitis incidence rate (UR)	Hib meningitis incidence rate, HIV (UR)	Hib NPNM cases (UR)	Hib NPNM cases, HIV (UR)	Hib NPNM incidence rate (UR)	Hib NPNM incidence rate, HIV (UR)
2015	Syrian Arab Republic	2469 (2255-4035)	0 (0-0)	115 (105-187)	0 (0-0)	461 (276-931)	0 (0-0)	21 (13-43)	0 (0-0)	18 (8-27)	0 (0-0)	1 (0-1)	0 (0-0)	6 (3-9)	0 (0-0)	0 (0-0)	0 (0-0)
2000	Tajikistan	1299 (1186-2122)		149 (136-243)		403 (242-814)		46 (28-93)		585 (204-922)		67 (23-106)		101 (35-159)		12 (4-18)	
2001	Tajikistan									533 (190-836)		61 (22-96)		92 (33-144)		11 (4-17)	
2002	Tajikistan									481 (176-751)		56 (20-87)		83 (30-129)		10 (4-15)	
2003	Tajikistan									436 (148-698)		51 (17-82)		147 (50-235)		17 (6-28)	
2004	Tajikistan									405 (141-644)		48 (17-76)		137 (48-217)		16 (6-26)	
2005	Tajikistan									381 (136-602)		45 (16-71)		128 (46-203)		15 (5-24)	
2006	Tajikistan									364 (130-576)		43 (15-68)		123 (44-194)		14 (5-23)	
2007	Tajikistan									354 (126-560)		41 (15-65)		119 (43-189)		14 (5-22)	
2008	Tajikistan									203 (72-321)		23 (8-36)		68 (24-108)		8 (3-12)	
2009	Tajikistan									27 (10-43)		3 (1-5)		9 (3-14)		1 (0-2)	
2010	Tajikistan									27 (10-43)		3 (1-4)		9 (3-14)		1 (0-1)	
2011	Tajikistan									25 (9-40)		2 (1-4)		8 (3-13)		1 (0-1)	
2012	Tajikistan									26 (9-42)		2 (1-4)		9 (3-14)		1 (0-1)	
2013	Tajikistan									3 (1-6)		0 (0-1)		1 (0-2)		0 (0-0)	
2014	Tajikistan									3 (1-5)		0 (0-0)		1 (0-2)		0 (0-0)	
2015	Tajikistan	17 (16-28)	0 (0-0)	1 (1-2)	0 (0-0)	5 (3-11)	0 (0-0)	0 (0-1)	0 (0-0)	3 (1-5)	0 (0-0)	0 (0-0)	0 (0-0)	1 (0-2)	0 (0-0)	0 (0-0)	0 (0-0)
2000	Thailand	46912 (42832-76650)		1025 (936-1675)		8691 (5204-17542)		190 (114-383)		388 (87-639)		8 (2-14)		131 (29-216)		3 (1-5)	
2001	Thailand									364 (82-597)		8 (2-13)		123 (28-201)		3 (1-4)	
2002	Thailand									345 (79-566)		8 (2-13)		116 (26-191)		3 (1-4)	
2003	Thailand									328 (75-537)		7 (2-12)		111 (25-181)		2 (1-4)	
2004	Thailand									310 (72-507)		7 (2-11)		105 (24-171)		2 (1-4)	
2005	Thailand									288 (68-470)		7 (2-11)		97 (23-158)		2 (1-4)	
2006	Thailand									267 (63-436)		6 (1-10)		90 (21-147)		2 (0-3)	
2007	Thailand									247 (58-403)		6 (1-10)		83 (19-136)		2 (0-3)	
2008	Thailand									223 (52-365)		5 (1-9)		75 (18-123)		2 (0-3)	
2009	Thailand									210 (49-343)		5 (1-9)		71 (16-116)		2 (0-3)	
2010	Thailand									198 (46-324)		5 (1-8)		67 (15-109)		2 (0-3)	
2011	Thailand									184 (42-301)		5 (1-8)		62 (14-101)		2 (0-3)	
2012	Thailand									172 (40-283)		4 (1-7)		58 (13-95)		2 (0-2)	
2013	Thailand									161 (37-263)		4 (1-7)		54 (13-89)		1 (0-2)	
2014	Thailand									152 (35-248)		4 (1-7)		51 (12-83)		1 (0-2)	
2015	Thailand	29680 (27099-48495)	0 (0-0)	795 (725-1298)	0 (0-0)	5534 (3314-11170)	0 (0-0)	148 (89-299)	0 (0-0)	144 (34-236)	0 (0-0)	4 (1-6)	0 (0-0)	49 (11-79)	0 (0-0)	1 (0-2)	0 (0-0)
2000	The former Yugoslav Republic of Macedonia	202 (184-330)		149 (136-243)		63 (38-127)		46 (28-93)		9 (3-17)		7 (2-13)		3 (1-6)		2 (1-4)	
2001	The former Yugoslav Republic of Macedonia									19 (5-35)		14 (4-27)		6 (2-12)		5 (1-9)	
2002	The former Yugoslav Republic of Macedonia									18 (5-33)		14 (4-26)		6 (2-11)		5 (1-9)	
2003	The former Yugoslav Republic of Macedonia									16 (5-31)		13 (4-24)		6 (2-10)		4 (1-8)	
2004	The former Yugoslav Republic of Macedonia									17 (5-32)		13 (4-25)		6 (2-11)		4 (1-8)	
2005	The former Yugoslav Republic of Macedonia									6 (2-11)		5 (1-9)		2 (1-4)		2 (0-3)	
2006	The former Yugoslav Republic of Macedonia									9 (3-17)		8 (2-14)		3 (1-6)		3 (1-5)	
2007	The former Yugoslav Republic of Macedonia									29 (9-55)		25 (7-46)		10 (3-18)		8 (2-16)	
2008	The former Yugoslav Republic of Macedonia									12 (3-22)		10 (3-19)		4 (1-7)		3 (1-6)	
2009	The former Yugoslav Republic of Macedonia									1 (0-3)		1 (0-2)		0 (0-1)		0 (0-1)	
2010	The former Yugoslav Republic of Macedonia									3 (1-5)		2 (1-4)		1 (0-2)		1 (0-1)	
2011	The former Yugoslav Republic of Macedonia									1 (0-2)		1 (0-2)		0 (0-1)		0 (0-1)	
2012	The former Yugoslav Republic of Macedonia									1 (0-2)		1 (0-2)		0 (0-1)		0 (0-1)	
2013	The former Yugoslav Republic of Macedonia									0 (0-0)		0 (0-0)		0 (0-0)		0 (0-0)	
2014	The former Yugoslav Republic of Macedonia									0 (0-0)		0 (0-0)		0 (0-0)		0 (0-0)	
2015	The former Yugoslav Republic of Macedonia	2 (2-3)	0 (0-0)	1 (1-2)	0 (0-0)	1 (0-1)	0 (0-0)	0 (0-1)	0 (0-0)	0 (0-0)	0 (0-0)	0 (0-0)	0 (0-0)	0 (0-0)	0 (0-0)	0 (0-0)	0 (0-0)
2000	Timor-Leste	2803 (2559-4579)		1637 (1495-2675)		519 (311-1048)		303 (182-612)		106 (38-166)		62 (22-97)		18 (7-29)		11 (4-17)	
2001	Timor-Leste									104 (38-162)		60 (22-94)		18 (7-28)		10 (4-16)	
2002	Timor-Leste									100 (37-156)		58 (21-91)		17 (6-27)		10 (4-16)	
2003	Timor-Leste									94 (35-146)		55 (21-86)		16 (6-25)		9 (4-15)	
2004	Timor-Leste									86 (33-133)		51 (19-79)		15 (6-23)		9 (3-14)	
2005	Timor-Leste									76 (29-117)		46 (18-71)		13 (5-20)		8 (3-12)	
2006	Timor-Leste									67 (26-102)		42 (17-65)		11 (5-18)		7 (3-11)	
2007	Timor-Leste									59 (22-92)		39 (14-61)		20 (7-31)		13 (5-21)	
2008	Timor-Leste									53 (20-82)		36 (14-56)		18 (7-28)		12 (5-19)	
2009	Timor-Leste									49 (19-77)		34 (13-52)		17 (6-26)		11 (4-18)	
2010	Timor-Leste									50 (19-78)		33 (13-52)		17 (7-26)		11 (4-18)	
2011	Timor-Leste									52 (20-81)		35 (14-54)		18 (7-27)		12 (5-18)	
2012	Timor-Leste									58 (23-90)		36 (14-56)		20 (8-30)		12 (5-19)	
2013	Timor-Leste									11 (4-16)		6 (2-9)		4 (1-6)		2 (1-3)	
2014	Timor-Leste									7 (3-11)		4 (1-6)		2 (1-4)		1 (0-2)	
2015	Timor-Leste	268 (245-438)	0 (0-1)	134 (122-219)	0 (0-0)	50 (30-100)	0 (0-0)	25 (15-50)	0 (0-0)	7 (3-11)	0 (0-0)	4 (1-6)	0 (0-0)	2 (1-4)	0 (0-0)	1 (0-2)	0 (0-0)
2000	Togo	20142 (18390-32910)		2508 (2290-4098)		3766 (2255-7602)		469 (281-947)		232 (69-382)		29 (9-48)		40 (12-66)		5 (1-8)	
2001	Togo									236 (69-389)		29 (8-47)		41 (12-67)		5 (1-8)	
2002	Togo									238 (69-393)		28 (8-46)		41 (12-68)		5 (1-8)	
2003	Togo									238 (69-392)		27 (8-45)		41 (12-68)		5 (1-8)	
2004	Togo									236 (68-391)		26 (8-44)		41 (12-67)		5 (1-8)	
2005	Togo									234 (66-387)		25 (7-42)		40 (11-67)		4 (1-7)	
2006	Togo									232 (65-385)		24 (7-40)		40 (11-66)		4 (1-7)	
2007	Togo									234 (67-387)		24 (7-40)		40 (12-67)		4 (1-7)	
2008	Togo									206 (60-339)		21 (6-34)		35 (10-58)		4 (1-6)	
2009	Togo									26 (8-43)		3 (1-4)		5 (1-7)		0 (0-1)	
2010	Togo									26 (8-43)		3 (1-4)		5 (1-7)		0 (0-1)	
2011	Togo									28 (9-46)		3 (1-4)		5 (2-8)		0 (0-1)	
2012	Togo									32 (10-52)		3 (1-5)		6 (2-9)		1 (0-1)	
2013	Togo									4 (1-6)		0 (0-1)		1 (0-1)		0 (0-0)	
2014	Togo									4 (1-6)		0 (0-1)		1 (0-1)		0 (0-0)	
2015	Togo	133 (121-217)	1 (1-2)	12 (11-19)	0 (0-0)	25 (15-50)	0 (0-1)	2 (1-4)	0 (0-0)	4 (1-6)	0 (0-0)	0 (0-1)	0 (0-0)	1 (0-1)	0 (0-0)	0 (0-0)	0 (0-0)
2000	Tonga	168 (153-274)		1286 (1174-2102)		31 (19-63)		241 (144-486)		8 (4-11)		58 (33-82)		3 (1-4)		20 (11-28)	
2001	Tonga									7 (4-10)		56 (32-79)		2 (1-3)		19 (11-27)	
2002	Tonga									7 (4-10)		56 (32-79)		2 (1-3)		19 (11-27)	
2003	Tonga									7 (4-10)		54 (31-76)		2 (1-3)		18 (11-26)	
2004	Tonga									7 (4-10)		55 (32-77)		2 (1-3)		18 (11-26)	
2005	Tonga									2 (1-3)		18 (11-26)		1 (0-1)		6 (4-9)	
2006	Tonga									2 (1-2)		11 (6-16)		1 (0-1)		4 (2-5)	
2007	Tonga									2 (1-2)		11 (6-16)		1 (0-1)		4 (2-5)	
2008	Tonga									2 (1-2)		11 (6-16)		1 (0-1)		4 (2-5)	
2009	Tonga									2 (1-2)		11 (7-16)		1 (0-1)		4 (2-5)	
2010	Tonga									0 (0-0)		1 (1-2)		0 (0-0)		0 (0-1)	
2011	Tonga									0 (0-0)		1 (1-2)		0 (0-0)		0 (0-1)	
2012	Tonga									0 (0-0)		1 (1-2)		0 (0-0)		0 (0-1)	
2013	Tonga									0 (0-0)		1 (1-2)		0 (0-0)		0 (0-1)	
2014	Tonga									0 (0-0)		1 (1-2)		0 (0-0)		0 (0-1)	
2015	Tonga	1 (1-1)	0 (0-0)	7 (6-12)	0 (0-0)	0 (0-0)	0 (0-0)	1 (1-3)	0 (0-0)	0 (0-0)	0 (0-0)	1 (1-2)	0 (0-0)	0 (0-0)	0 (0-0)	0 (0-1)	0 (0-0)
2000	Trinidad and Tobago	90 (83-148)		103 (94-168)		17 (10-34)		19 (11-39)		2 (0-3)		2 (0-3)		1 (0-1)		1 (0-1)	
2001	Trinidad and Tobago									0 (0-1)		1 (0-1)		0 (0-0)		0 (0-0)	
2002	Trinidad and Tobago									1 (0-2)		1 (0-2)		0 (0-1)		0 (0-1)	
2003	Trinidad and Tobago									0 (0-0)		0 (0-0)		0 (0-0)		0 (0-0)	
2004	Trinidad and Tobago									0 (0-0)		0 (0-0)		0 (0-0)		0 (0-0)	
2005	Trinidad and Tobago									0 (0-0)		0 (0-0)		0 (0-0)		0 (0-0)	
2006	Trinidad and Tobago									0 (0-0)		0 (0-0)		0 (0-0)		0 (0-0)	
2007	Trinidad and Tobago									0 (0-0)		0 (0-0)		0 (0-0)		0 (0-0)	
2008	Trinidad and Tobago									0 (0-0)		0 (0-0)		0 (0-0)		0 (0-0)	
2009	Trinidad and Tobago									0 (0-0)		0 (0-0)		0 (0-0)		0 (0-0)	
2010	Trinidad and Tobago									0 (0-0)		0 (0-0)		0 (0-0)		0 (0-0)	
2011	Trinidad and Tobago									0 (0-0)		0 (0-0)		0 (0-0)		0 (0-0)	
2012	Trinidad and Tobago									0 (0-0)		0 (0-0)		0 (0-0)		0 (0-0)	
2013	Trinidad and Tobago									0 (0-0)		0 (0-0)		0 (0-0)		0 (0-0)	
2014	Trinidad and Tobago									0 (0-0)		0 (0-0)		0 (0-0)		0 (0-0)	
2015	Trinidad and Tobago	6 (5-9)	0 (0-0)	6 (5-10)	0 (0-0)	1 (1-2)	0 (0-0)	1 (1-2)	0 (0-0)	0 (0-0)	0 (0-0)	0 (0-0)	0 (0-0)	0 (0-0)	0 (0-0)	0 (0-0)	0 (0-0)
2000	Tunisia	10945 (9993-17883)		1325 (1209-2164)		2062 (1235-4163)		250 (149-504)		24 (7-40)		3 (1-5)		8 (2-14)		1 (0-2)	
2001	Tunisia									22 (2-40)		3 (0-5)		8 (1-14)		1 (0-2)	
2002	Tunisia									21 (2-38)		3 (0-5)		7 (1-13)		1 (0-2)	
2003	Tunisia									6 (1-10)		1 (0-1)		2 (0-4)		0 (0-0)	
2004	Tunisia									3 (0-5)		0 (0-1)		1 (0-2)		0 (0-0)	
2005	Tunisia									4 (0-6)		0 (0-1)		1 (0-2)		0 (0-0)	
2006	Tunisia									18 (2-31)		2 (0-4)		6 (1-11)		1 (0-1)	
2007	Tunisia									17 (2-30)		2 (0-4)		6 (1-10)		1 (0-1)	
2008	Tunisia									16 (2-28)		2 (0-3)		5 (1-10)		1 (0-1)	
2009	Tunisia									15 (2-27)		2 (0-3)		5 (1-9)		1 (0-1)	
2010	Tunisia									14 (2-25)		2 (0-3)		5 (1-8)		1 (0-1)	
2011	Tunisia									12 (2-21)		1 (0-2)		4 (1-7)		0 (0-1)	
2012	Tunisia									2 (0-3)		0 (0-0)		1 (0-1)		0 (0-0)	
2013	Tunisia									2 (0-3)		0 (0-0)		1 (0-1)		0 (0-0)	
2014	Tunisia									2 (0-3)		0 (0-0)		1 (0-1)		0 (0-0)	
2015	Tunisia	463 (422-756)	0 (0-0)	48 (44-78)	0 (0-0)	86 (52-174)	0 (0-0)	9 (5-18)	0 (0-0)	1 (0-3)	0 (0-0)	0 (0-0)	0 (0-0)	0 (0-1)	0 (0-0)	0 (0-0)	0 (0-0)
2000	Turkey	9712 (8867-15868)		149 (136-243)		3016 (1806-6088)		46 (28-93)		851 (475-1321)		13 (7-20)		287 (160-445)		4 (2-7)	
2001	Turkey									814 (459-1277)		12 (7-19)		274 (155-430)		4 (2-7)	
2002	Turkey									790 (454-1264)		12 (7-19)		266 (153-426)		4 (2-6)	
2003	Turkey									810 (481-1339)		12 (7-21)		273 (162-452)		4 (2-7)	
2004	Turkey									902 (365-1202)		14 (6-19)		304 (123-405)		5 (2-6)	
2005	Turkey									994 (438-1442)		16 (7-22)		335 (148-486)		5 (2-8)	
2006	Turkey									1048 (502-1654)		17 (8-26)		353 (169-557)		6 (3-9)	
2007	Turkey									285 (144-476)		5 (2-8)		96 (49-160)		2 (1-3)	
2008	Turkey									92 (46-153)		1 (1-2)		31 (16-52)		0 (0-1)	
2009	Turkey									96 (48-160)		2 (1-3)		32 (16-54)		1 (0-1)	
2010	Turkey									84 (43-141)		1 (1-2)		28 (14-47)		0 (0-1)	
2011	Turkey									84 (43-141)		1 (1-2)		28 (14-47)		0 (0-1)	
2012	Turkey									14 (7-24)		0 (0-0)		5 (2-8)		0 (0-0)	
2013	Turkey									12 (6-19)		0 (0-0)		4 (2-7)		0 (0-0)	
2014	Turkey									12 (6-19)		0 (0-0)		4 (2-7)		0 (0-0)	
2015	Turkey	100 (91-163)	0 (0-0)	1 (1-2)	0 (0-0)	31 (19-63)	0 (0-0)	0 (0-1)	0 (0-0)	11 (5-18)	0 (0-0)	0 (0-0)	0 (0-0)	4 (2-6)	0 (0-0)	0 (0-0)	0 (0-0)
2000	Turkmenistan	717 (655-1171)		149 (136-243)		223 (133-449)		46 (28-93)		148 (52-234)		31 (11-49)		26 (9-40)		5 (2-8)	
2001	Turkmenistan									146 (51-229)		32 (11-50)		25 (9-39)		5 (2-9)	
2002	Turkmenistan									149 (52-234)		32 (11-51)		26 (9-40)		6 (2-9)	
2003	Turkmenistan									157 (50-254)		33 (11-53)		53 (17-86)		11 (4-18)	
2004	Turkmenistan									161 (52-260)		33 (11-53)		54 (17-88)		11 (4-18)	
2005	Turkmenistan									159 (51-257)		32 (10-52)		54 (17-87)		11 (3-17)	
2006	Turkmenistan									151 (49-244)		29 (10-47)		51 (17-82)		10 (3-16)	
2007	Turkmenistan									140 (46-225)		27 (9-43)		47 (15-76)		9 (3-15)	
2008	Turkmenistan									127 (41-204)		25 (8-40)		43 (14-69)		8 (3-13)	
2009	Turkmenistan									114 (38-184)		23 (8-37)		39 (13-62)		8 (3-12)	
2010	Turkmenistan									46 (15-74)		9 (3-15)		16 (5-25)		3 (1-5)	
2011	Turkmenistan									11 (4-18)		2 (1-4)		4 (1-6)		1 (0-1)	
2012	Turkmenistan									7 (2-11)		1 (0-2)		2 (1-4)		0 (0-1)	
2013	Turkmenistan									7 (2-11)		1 (0-2)		2 (1-4)		0 (0-1)	
2014	Turkmenistan									7 (2-11)		1 (0-2)		2 (1-4)		0 (0-1)	
2015	Turkmenistan	8 (7-13)	0 (0-0)	1 (1-2)	0 (0-0)	2 (1-5)	0 (0-0)	0 (0-1)	0 (0-0)	1 (0-2)	0 (0-0)	0 (0-0)	0 (0-0)	0 (0-1)	0 (0-0)	0 (0-0)	0 (0-0)
2000	Tuvalu	15 (13-24)		1044 (954-1707)		3 (2-5)		194 (116-392)		1 (0-1)		41 (27-53)		0 (0-0)		14 (9-18)	
2001	Tuvalu									0 (0-1)		32 (21-41)		0 (0-0)		11 (7-14)	
2002	Tuvalu									0 (0-1)		33 (22-42)		0 (0-0)		11 (7-14)	
2003	Tuvalu									0 (0-1)		34 (23-44)		0 (0-0)		12 (8-15)	
2004	Tuvalu									0 (0-0)		24 (16-31)		0 (0-0)		8 (5-10)	
2005	Tuvalu									0 (0-0)		31 (20-40)		0 (0-0)		10 (7-13)	
2006	Tuvalu									0 (0-0)		25 (17-33)		0 (0-0)		9 (6-11)	
2007	Tuvalu									0 (0-0)		19 (13-25)		0 (0-0)		7 (4-8)	

Hib = *Haemophilus influenzae* type b; npnm = non-pneumonia, non-meningitis; HIV = cases only in HIV-infected children; Uncertainty range provided in parentheses.

Year	Country	Hib pneumonia cases (UR)	Hib pneumonia cases, HIV (UR)	Hib pneumonia incidence rate (UR)	Hib pneumonia incidence rate, HIV (UR)	Hib severe pneumonia cases (UR)	Hib severe pneumonia cases, HIV (UR)	Hib severe pneumonia incidence rate (UR)	Hib severe pneumonia incidence rate, HIV (UR)	Hib meningitis cases (UR)	Hib meningitis cases, HIV (UR)	Hib meningitis incidence rate (UR)	Hib meningitis incidence rate, HIV (UR)	Hib NPNM cases (UR)	Hib NPNM cases, HIV (UR)	Hib NPNM incidence rate (UR)	Hib NPNM incidence rate, HIV (UR)
2008	Tuvalu									0 (0-0)		19 (13-25)		0 (0-0)		6 (4-8)	
2009	Tuvalu									0 (0-0)		10 (7-13)		0 (0-0)		4 (2-5)	
2010	Tuvalu									0 (0-0)		5 (3-6)		0 (0-0)		2 (1-2)	
2011	Tuvalu									0 (0-0)		5 (3-6)		0 (0-0)		2 (1-2)	
2012	Tuvalu									0 (0-0)		5 (3-6)		0 (0-0)		2 (1-2)	
2013	Tuvalu									0 (0-0)		7 (4-9)		0 (0-0)		2 (1-3)	
2014	Tuvalu									0 (0-0)		1 (1-1)		0 (0-0)		0 (0-0)	
2015	Tuvalu	0 (0-0)	0 (0-0)	6 (6-10)	0 (0-0)	0 (0-0)	0 (0-0)	1 (1-2)	0 (0-0)	0 (0-0)	0 (0-0)	1 (1-1)	0 (0-0)	0 (0-0)	0 (0-0)	0 (0-0)	0 (0-0)
2000	Uganda	67583 (61705-110425)		1454 (1328-2376)		12714 (7612-25661)		274 (164-552)		3414 (1388-5281)		73 (30-114)		588 (239-909)		13 (5-20)	
2001	Uganda									3362 (1368-5203)		70 (29-108)		579 (235-896)		12 (5-19)	
2002	Uganda									2625 (1080-4048)		53 (22-82)		452 (186-697)		9 (4-14)	
2003	Uganda									523 (218-804)		10 (4-16)		90 (38-138)		2 (1-3)	
2004	Uganda									438 (185-671)		8 (3-13)		75 (32-116)		1 (1-2)	
2005	Uganda									430 (184-656)		8 (3-12)		74 (32-113)		1 (1-2)	
2006	Uganda									413 (179-629)		7 (3-11)		71 (31-108)		1 (1-2)	
2007	Uganda									32 (14-48)		1 (0-1)		5 (2-8)		0 (0-0)	
2008	Uganda									32 (14-48)		1 (0-1)		5 (2-8)		0 (0-0)	
2009	Uganda									31 (14-47)		1 (0-1)		5 (2-8)		0 (0-0)	
2010	Uganda									32 (14-48)		1 (0-1)		5 (2-8)		0 (0-0)	
2011	Uganda									33 (14-50)		1 (0-1)		11 (5-17)		0 (0-0)	
2012	Uganda									33 (15-51)		1 (0-1)		11 (5-17)		0 (0-0)	
2013	Uganda									34 (15-52)		1 (0-1)		12 (5-18)		0 (0-0)	
2014	Uganda									34 (15-51)		0 (0-1)		11 (5-17)		0 (0-0)	
2015	Uganda	789 (720-1289)	30 (27-48)	11 (10-18)	0 (0-0)	148 (89-299)	6 (3-11)	2 (1-4)	0 (0-0)	33 (15-50)	1 (1-2)	0 (0-1)	0 (0-0)	11 (5-17)	0 (0-1)	0 (0-0)	0 (0-0)
2000	Ukraine	6324 (5773-10332)		298 (272-486)		1964 (1176-3964)		92 (55-187)		234 (60-457)		11 (3-21)		79 (20-154)		4 (1-7)	
2001	Ukraine									209 (54-407)		10 (3-20)		71 (18-137)		3 (1-7)	
2002	Ukraine									184 (48-358)		9 (2-18)		62 (16-121)		3 (1-6)	
2003	Ukraine									169 (45-326)		9 (2-17)		57 (15-110)		3 (1-6)	
2004	Ukraine									158 (42-304)		8 (2-16)		53 (14-103)		3 (1-5)	
2005	Ukraine									154 (41-296)		8 (2-15)		52 (14-100)		3 (1-5)	
2006	Ukraine									170 (46-326)		9 (2-17)		57 (16-110)		3 (1-6)	
2007	Ukraine									41 (11-78)		2 (1-4)		14 (4-26)		1 (0-1)	
2008	Ukraine									28 (8-53)		1 (0-2)		9 (3-18)		0 (0-1)	
2009	Ukraine									33 (9-62)		1 (0-3)		11 (3-21)		0 (0-1)	
2010	Ukraine									37 (10-71)		2 (0-3)		13 (4-24)		1 (0-1)	
2011	Ukraine									99 (28-189)		4 (1-8)		33 (9-64)		1 (0-3)	
2012	Ukraine									2 (1-4)		0 (0-0)		1 (0-1)		0 (0-0)	
2013	Ukraine									2 (1-4)		0 (0-0)		1 (0-1)		0 (0-0)	
2014	Ukraine									34 (10-65)		1 (0-3)		12 (3-22)		0 (0-1)	
2015	Ukraine	1550 (1416-2533)	8 (6-14)	64 (58-105)	0 (0-0)	482 (288-972)	3 (2-5)	20 (12-40)	0 (0-0)	29 (9-55)	0 (0-0)	1 (0-2)	0 (0-0)	10 (3-19)	0 (0-0)	0 (0-1)	0 (0-0)
2000	United Arab Emirates	295 (269-482)		115 (105-188)		56 (33-112)		22 (13-44)		3 (1-4)		1 (1-2)		1 (0-1)		0 (0-1)	
2001	United Arab Emirates									2 (1-4)		1 (0-2)		1 (0-1)		0 (0-1)	
2002	United Arab Emirates									2 (1-4)		1 (0-1)		1 (0-1)		0 (0-0)	
2003	United Arab Emirates									2 (1-3)		1 (0-1)		1 (0-1)		0 (0-0)	
2004	United Arab Emirates									0 (0-0)		0 (0-0)		0 (0-0)		0 (0-0)	
2005	United Arab Emirates									0 (0-0)		0 (0-0)		0 (0-0)		0 (0-0)	
2006	United Arab Emirates									0 (0-1)		0 (0-0)		0 (0-0)		0 (0-0)	
2007	United Arab Emirates									0 (0-1)		0 (0-0)		0 (0-0)		0 (0-0)	
2008	United Arab Emirates									0 (0-1)		0 (0-0)		0 (0-0)		0 (0-0)	
2009	United Arab Emirates									0 (0-1)		0 (0-0)		0 (0-0)		0 (0-0)	
2010	United Arab Emirates									0 (0-1)		0 (0-0)		0 (0-0)		0 (0-0)	
2011	United Arab Emirates									0 (0-1)		0 (0-0)		0 (0-0)		0 (0-0)	
2012	United Arab Emirates									0 (0-1)		0 (0-0)		0 (0-0)		0 (0-0)	
2013	United Arab Emirates									0 (0-1)		0 (0-0)		0 (0-0)		0 (0-0)	
2014	United Arab Emirates									0 (0-1)		0 (0-0)		0 (0-0)		0 (0-0)	
2015	United Arab Emirates	34 (31-55)	0 (0-0)	7 (6-11)	0 (0-0)	6 (4-13)	0 (0-0)	1 (1-3)	0 (0-0)	0 (0-1)	0 (0-0)	0 (0-0)	0 (0-0)	0 (0-0)	0 (0-0)	0 (0-0)	0 (0-0)
2000	United Kingdom	26 (24-42)		1 (1-1)		8 (5-16)		0 (0-0)		8 (4-15)		0 (0-0)		3 (1-5)		0 (0-0)	
2001	United Kingdom									9 (4-17)		0 (0-0)		3 (1-6)		0 (0-0)	
2002	United Kingdom									7 (3-14)		0 (0-0)		2 (1-5)		0 (0-0)	
2003	United Kingdom									8 (4-15)		0 (0-0)		3 (1-5)		0 (0-0)	
2004	United Kingdom									7 (3-13)		0 (0-0)		2 (1-5)		0 (0-0)	
2005	United Kingdom									7 (3-13)		0 (0-0)		2 (1-5)		0 (0-0)	
2006	United Kingdom									5 (2-10)		0 (0-0)		2 (1-3)		0 (0-0)	
2007	United Kingdom									6 (3-11)		0 (0-0)		2 (1-4)		0 (0-0)	
2008	United Kingdom									6 (3-11)		0 (0-0)		2 (1-4)		0 (0-0)	
2009	United Kingdom									6 (3-12)		0 (0-0)		2 (1-4)		0 (0-0)	
2010	United Kingdom									6 (3-12)		0 (0-0)		2 (1-4)		0 (0-0)	
2011	United Kingdom									6 (3-11)		0 (0-0)		2 (1-4)		0 (0-0)	
2012	United Kingdom									5 (2-8)		0 (0-0)		2 (1-3)		0 (0-0)	
2013	United Kingdom									3 (1-5)		0 (0-0)		1 (0-2)		0 (0-0)	
2014	United Kingdom									4 (2-7)		0 (0-0)		1 (1-2)		0 (0-0)	
2015	United Kingdom	30 (27-49)	0 (0-0)	1 (1-1)	0 (0-0)	9 (6-19)	0 (0-0)	0 (0-0)	0 (0-0)	4 (2-7)	0 (0-0)	0 (0-0)	0 (0-0)	1 (1-2)	0 (0-0)	0 (0-0)	0 (0-0)
2000	United Republic of Tanzania	80794 (73767-132010)		1391 (1270-2272)		15238 (9124-30756)		262 (157-529)		3883 (1390-6176)		67 (24-106)		669 (239-1064)		12 (4-18)	
2001	United Republic of Tanzania									3783 (1369-6000)		63 (23-100)		651 (236-1033)		11 (4-17)	
2002	United Republic of Tanzania									3642 (1334-5760)		59 (22-93)		627 (230-992)		10 (4-16)	
2003	United Republic of Tanzania									3704 (1373-5841)		58 (21-91)		638 (236-1006)		10 (4-16)	
2004	United Republic of Tanzania									3634 (1365-5715)		55 (21-86)		626 (235-984)		9 (4-15)	
2005	United Republic of Tanzania									3560 (1339-5605)		52 (19-81)		613 (231-965)		9 (3-14)	
2006	United Republic of Tanzania									3497 (1343-5475)		49 (19-77)		602 (231-943)		8 (3-13)	
2007	United Republic of Tanzania									3379 (1327-5261)		46 (18-71)		582 (228-906)		8 (3-12)	
2008	United Republic of Tanzania									3326 (1224-5314)		44 (16-70)		1121 (413-1791)		15 (5-24)	
2009	United Republic of Tanzania									538 (203-854)		7 (3-11)		182 (69-288)		2 (1-4)	
2010	United Republic of Tanzania									262 (102-412)		3 (1-5)		88 (34-139)		1 (0-2)	
2011	United Republic of Tanzania									268 (105-420)		3 (1-5)		90 (35-142)		1 (0-2)	
2012	United Republic of Tanzania									259 (103-405)		3 (1-5)		87 (35-137)		1 (0-2)	
2013	United Republic of Tanzania									267 (107-418)		3 (1-5)		90 (36-141)		1 (0-2)	
2014	United Republic of Tanzania									33 (13-52)		0 (0-1)		11 (5-18)		0 (0-0)	
2015	United Republic of Tanzania	1122 (1024-1833)	20 (18-32)	12 (11-20)	0 (0-0)	211 (126-426)	4 (2-7)	2 (1-5)	0 (0-0)	33 (13-51)	1 (0-1)	0 (0-1)	0 (0-0)	11 (5-17)	0 (0-0)	0 (0-0)	0 (0-0)
2000	United States of America	141 (129-230)		1 (1-1)		44 (26-88)		0 (0-0)		37 (15-63)		0 (0-0)		12 (5-21)		0 (0-0)	
2001	United States of America									30 (12-51)		0 (0-0)		10 (4-17)		0 (0-0)	
2002	United States of America									28 (11-48)		0 (0-0)		10 (4-16)		0 (0-0)	
2003	United States of America									31 (13-53)		0 (0-0)		10 (4-18)		0 (0-0)	
2004	United States of America									32 (13-54)		0 (0-0)		11 (4-18)		0 (0-0)	
2005	United States of America									36 (14-61)		0 (0-0)		12 (5-21)		0 (0-0)	
2006	United States of America									31 (12-52)		0 (0-0)		10 (4-18)		0 (0-0)	
2007	United States of America									30 (12-52)		0 (0-0)		10 (4-17)		0 (0-0)	
2008	United States of America									26 (11-45)		0 (0-0)		9 (4-15)		0 (0-0)	
2009	United States of America									26 (11-45)		0 (0-0)		9 (4-15)		0 (0-0)	
2010	United States of America									34 (14-58)		0 (0-0)		11 (5-19)		0 (0-0)	
2011	United States of America									21 (9-36)		0 (0-0)		7 (3-12)		0 (0-0)	
2012	United States of America									18 (7-31)		0 (0-0)		6 (2-10)		0 (0-0)	
2013	United States of America									24 (10-41)		0 (0-0)		8 (3-14)		0 (0-0)	
2014	United States of America									20 (8-34)		0 (0-0)		7 (3-12)		0 (0-0)	
2015	United States of America	144 (132-236)	0 (0-0)	1 (1-1)	0 (0-0)	45 (27-90)	0 (0-0)	0 (0-0)	0 (0-0)	20 (8-34)	0 (0-0)	0 (0-0)	0 (0-0)	7 (3-11)	0 (0-0)	0 (0-0)	0 (0-0)
2000	Uruguay	213 (194-348)		80 (73-130)		40 (24-80)		15 (9-30)		8 (3-13)		3 (1-5)		3 (1-5)		1 (0-2)	
2001	Uruguay									14 (6-24)		5 (2-9)		5 (2-8)		2 (1-3)	
2002	Uruguay									1 (1-2)		1 (0-1)		0 (0-1)		0 (0-0)	
2003	Uruguay									1 (1-2)		1 (0-1)		0 (0-1)		0 (0-0)	
2004	Uruguay									1 (1-2)		0 (0-1)		0 (0-1)		0 (0-0)	
2005	Uruguay									1 (1-2)		1 (0-1)		0 (0-1)		0 (0-0)	
2006	Uruguay									1 (1-2)		0 (0-1)		0 (0-1)		0 (0-0)	
2007	Uruguay									2 (1-3)		1 (0-1)		1 (0-1)		0 (0-0)	
2008	Uruguay									1 (0-2)		0 (0-1)		0 (0-1)		0 (0-0)	
2009	Uruguay									1 (1-2)		1 (0-1)		0 (0-1)		0 (0-0)	
2010	Uruguay									1 (0-2)		0 (0-1)		0 (0-1)		0 (0-0)	
2011	Uruguay									1 (0-2)		0 (0-1)		0 (0-1)		0 (0-0)	
2012	Uruguay									1 (0-1)		0 (0-0)		0 (0-0)		0 (0-0)	
2013	Uruguay									1 (0-1)		0 (0-1)		0 (0-0)		0 (0-0)	
2014	Uruguay									1 (0-1)		0 (0-1)		0 (0-0)		0 (0-0)	
2015	Uruguay	14 (13-23)	0 (0-0)	6 (5-10)	0 (0-0)	3 (2-5)	0 (0-0)	1 (1-2)	0 (0-0)	1 (0-1)	0 (0-0)	0 (0-1)	0 (0-0)	0 (0-0)	0 (0-0)	0 (0-0)	0 (0-0)
2000	Uzbekistan	4011 (3662-6553)		149 (136-243)		1246 (746-2514)		46 (28-93)		908 (330-1433)		34 (12-53)		306 (111-483)		11 (4-18)	
2001	Uzbekistan									858 (313-1353)		33 (12-52)		289 (106-456)		11 (4-17)	
2002	Uzbekistan									811 (298-1278)		32 (12-50)		274 (100-431)		11 (4-17)	
2003	Uzbekistan									768 (283-1208)		30 (11-48)		259 (95-407)		10 (4-16)	
2004	Uzbekistan									741 (275-1165)		30 (11-47)		250 (93-393)		10 (4-16)	
2005	Uzbekistan									715 (266-1122)		29 (11-45)		241 (90-378)		10 (4-15)	
2006	Uzbekistan									700 (262-1098)		28 (10-44)		236 (88-370)		9 (4-15)	
2007	Uzbekistan									690 (260-1080)		27 (10-42)		232 (88-364)		9 (3-14)	
2008	Uzbekistan									687 (260-1075)		26 (10-41)		232 (88-362)		9 (3-14)	
2009	Uzbekistan									104 (39-162)		4 (1-6)		35 (13-55)		1 (0-2)	
2010	Uzbekistan									40 (15-63)		1 (1-2)		14 (5-21)		0 (0-1)	
2011	Uzbekistan									39 (15-61)		1 (1-2)		13 (5-21)		0 (0-1)	
2012	Uzbekistan									38 (15-60)		1 (0-2)		13 (5-20)		0 (0-1)	
2013	Uzbekistan									37 (14-58)		1 (0-2)		12 (5-19)		0 (0-1)	
2014	Uzbekistan									6 (2-9)		0 (0-0)		2 (1-3)		0 (0-0)	
2015	Uzbekistan	47 (43-76)	0 (0-0)	1 (1-2)	0 (0-0)	15 (9-29)	0 (0-0)	0 (0-1)	0 (0-0)	6 (2-9)	0 (0-0)	0 (0-0)	0 (0-0)	2 (1-3)	0 (0-0)	0 (0-0)	0 (0-0)
2000	Vanuatu	357 (326-584)		1282 (1171-2095)		67 (40-135)		241 (144-486)		4 (1-6)		16 (4-21)		1 (0-2)		5 (1-7)	
2001	Vanuatu									4 (1-6)		15 (4-20)		1 (0-2)		5 (1-7)	
2002	Vanuatu									3 (1-5)		12 (3-16)		1 (0-2)		4 (1-5)	
2003	Vanuatu									3 (1-4)		10 (3-13)		1 (0-1)		3 (1-4)	
2004	Vanuatu									3 (1-4)		10 (3-14)		1 (0-1)		4 (1-5)	
2005	Vanuatu									4 (1-5)		13 (3-18)		1 (0-2)		4 (1-6)	
2006	Vanuatu									4 (1-6)		14 (4-19)		1 (0-2)		5 (1-6)	
2007	Vanuatu									4 (1-6)		15 (4-20)		2 (0-2)		5 (1-7)	
2008	Vanuatu									5 (1-6)		15 (4-20)		2 (0-2)		5 (1-7)	
2009	Vanuatu									5 (1-7)		15 (4-20)		2 (0-2)		5 (1-7)	
2010	Vanuatu									5 (1-7)		15 (4-20)		2 (0-2)		5 (1-7)	
2011	Vanuatu									4 (1-5)		10 (3-14)		1 (0-2)		3 (1-5)	
2012	Vanuatu									2 (0-2)		4 (1-6)		1 (0-1)		1 (0-2)	
2013	Vanuatu									1 (0-2)		4 (1-6)		0 (0-1)		1 (0-2)	
2014	Vanuatu									1 (0-2)		4 (1-5)		0 (0-1)		1 (0-2)	
2015	Vanuatu	47 (43-78)	0 (0-0)	139 (127-227)	0 (0-0)	9 (5-18)	0 (0-0)	26 (16-52)	0 (0-0)	1 (0-2)	0 (0-0)	4 (1-5)	0 (0-0)	0 (0-1)	0 (0-0)	1 (0-2)	0 (0-0)
2000	Venezuela (Bolivarian Republic of)	25578 (23354-41792)		924 (843-1509)		4768 (2855-9623)		172 (103-347)		299 (43-521)		11 (2-19)		101 (15-176)		4 (1-6)	
2001	Venezuela (Bolivarian Republic of)									106 (15-185)		4 (1-7)		36 (5-62)		1 (0-2)	
2002	Venezuela (Bolivarian Republic of)									47 (7-81)		2 (0-3)		16 (2-27)		1 (0-1)	
2003	Venezuela (Bolivarian Republic of)									35 (5-61)		1 (0-2)		12 (2-21)		0 (0-1)	
2004	Venezuela (Bolivarian Republic of)									49 (7-86)		2 (0-3)		17 (2-29)		1 (0-1)	
2005	Venezuela (Bolivarian Republic of)									3 (1-6)		0 (0-0)		1 (0-2)		0 (0-0)	
2006	Venezuela (Bolivarian Republic of)									3 (0-5)		0 (0-0)		1 (0-2)		0 (0-0)	
2007	Venezuela (Bolivarian Republic of)									35 (5-60)		1 (0-2)		12 (2-20)		0 (0-1)	
2008	Venezuela (Bolivarian Republic of)									41 (6-70)		1 (0-2)		14 (2-24)		0 (0-1)	
2009	Venezuela (Bolivarian Republic of)									3 (0-4)		0 (0-0)		1 (0-1)		0 (0-0)	
2010	Venezuela (Bolivarian Republic of)									2 (0-4)		0 (0-0)		1 (0-1)		0 (0-0)	
2011	Venezuela (Bolivarian Republic of)									3 (0-5)		0 (0-0)		1 (0-2)		0 (0-0)	
2012	Venezuela (Bolivarian Republic of)									3 (0-5)		0 (0-0)		1 (0-2)		0 (0-0)	
2013	Venezuela (Bolivarian Republic of)									3 (0-5)		0 (0-0)		1 (0-2)		0 (0-0)	
2014	Venezuela (Bolivarian Republic of)									3 (0-5)		0 (0-0)		1 (0-2)		0 (0-0)	
2015	Venezuela (Bolivarian Republic of)	175 (160-286)	0 (0-1)	6 (5-10)	0 (0-0)	33 (20-66)	0 (0-0)	1 (1-2)	0 (0-0)	3 (0-5)	0 (0-0)	0 (0-0)	0 (0-0)	1 (0-2)	0 (0-0)	0 (0-0)	0 (0-0)
2000	Viet Nam	90045 (82213-147125)		1264 (1154-2065)		16877 (10105-34063)		237 (142-478)		550 (190-882)		8 (3-12)		185 (64-297)		3 (1-4)	

Hib = *Haemophilus influenzae* type b; npnm = non-pneumonia, non-meningitis; HIV = cases only in HIV-infected children; Uncertainty range provided in parentheses.

Year	Country	Hib pneumonia cases (UR)	Hib pneumonia cases, HIV (UR)	Hib pneumonia incidence rate (UR)	Hib pneumonia incidence rate, HIV (UR)	Hib severe pneumonia cases (UR)	Hib severe pneumonia cases, HIV (UR)	Hib severe pneumonia incidence rate (UR)	Hib severe pneumonia incidence rate, HIV (UR)
2001	Viet Nam								
2002	Viet Nam								
2003	Viet Nam								
2004	Viet Nam								
2005	Viet Nam								
2006	Viet Nam								
2007	Viet Nam								
2008	Viet Nam								
2009	Viet Nam								
2010	Viet Nam								
2011	Viet Nam								
2012	Viet Nam								
2013	Viet Nam								
2014	Viet Nam								
2015	Viet Nam	872 (797-1425)	2 (2-3)	11 (10-19)	0 (0-0)	163 (97-328)	0 (0-1)	2 (1-4)	0 (0-0)
2000	Yemen	28956 (26437-47311)		939 (857-1533)		5402 (3234-10903)		175 (105-353)	
2001	Yemen								
2002	Yemen								
2003	Yemen								
2004	Yemen								
2005	Yemen								
2006	Yemen								
2007	Yemen								
2008	Yemen								
2009	Yemen								
2010	Yemen								
2011	Yemen								
2012	Yemen								
2013	Yemen								
2014	Yemen								
2015	Yemen	353 (323-577)	0 (0-0)	9 (8-15)	0 (0-0)	66 (40-134)	0 (0-0)	2 (1-3)	0 (0-0)
2000	Zambia	39771 (36312-64983)		2109 (1926-3446)		7451 (4461-15039)		395 (237-798)	
2001	Zambia								
2002	Zambia								
2003	Zambia								
2004	Zambia								
2005	Zambia								
2006	Zambia								
2007	Zambia								
2008	Zambia								
2009	Zambia								
2010	Zambia								
2011	Zambia								
2012	Zambia								
2013	Zambia								
2014	Zambia								
2015	Zambia	387 (353-632)	17 (15-27)	14 (13-23)	1 (1-0)	72 (43-146)	3 (2-6)	3 (2-5)	0 (0-0)
2000	Zimbabwe	29875 (27277-48814)		1653 (1509-2701)		5601 (3353-11304)		310 (186-626)	
2001	Zimbabwe								
2002	Zimbabwe								
2003	Zimbabwe								
2004	Zimbabwe								
2005	Zimbabwe								
2006	Zimbabwe								
2007	Zimbabwe								
2008	Zimbabwe								
2009	Zimbabwe								
2010	Zimbabwe								
2011	Zimbabwe								
2012	Zimbabwe								
2013	Zimbabwe								
2014	Zimbabwe								
2015	Zimbabwe	283 (259-463)	15 (13-24)	12 (11-19)	1 (1-0)	53 (32-107)	3 (2-6)	2 (1-4)	0 (0-0)

Year	Country	Hib meningitis cases (UR)	Hib meningitis cases, HIV (UR)	Hib meningitis incidence rate (UR)	Hib meningitis incidence rate, HIV (UR)	Hib NPNM cases (UR)	Hib NPNM cases, HIV (UR)	Hib NPNM incidence rate (UR)	Hib NPNM incidence rate, HIV (UR)
2001	Viet Nam	562 (205-889)		8 (3-13)		189 (69-300)		3 (1-5)	
2002	Viet Nam	620 (242-969)		10 (4-15)		209 (81-327)		3 (1-5)	
2003	Viet Nam	705 (286-1099)		11 (4-17)		238 (97-370)		4 (1-6)	
2004	Viet Nam	813 (153-1334)		12 (2-20)		274 (52-450)		4 (1-7)	
2005	Viet Nam	897 (184-1463)		14 (3-22)		302 (62-493)		5 (1-7)	
2006	Viet Nam	926 (206-1487)		13 (3-22)		312 (70-501)		5 (1-7)	
2007	Viet Nam	882 (187-1446)		13 (3-21)		297 (63-487)		4 (1-7)	
2008	Viet Nam	779 (157-1291)		11 (2-18)		263 (53-435)		4 (1-6)	
2009	Viet Nam	689 (132-1150)		10 (2-16)		232 (44-388)		3 (1-5)	
2010	Viet Nam	387 (71-652)		5 (1-9)		131 (24-220)		2 (0-3)	
2011	Viet Nam	86 (15-147)		1 (0-2)		29 (5-49)		0 (0-1)	
2012	Viet Nam	81 (15-137)		1 (0-2)		27 (5-46)		0 (0-1)	
2013	Viet Nam	149 (30-248)		2 (0-3)		50 (10-84)		1 (0-1)	
2014	Viet Nam	85 (18-140)		1 (0-2)		29 (6-47)		0 (0-1)	
2015	Viet Nam	12 (3-20)	0 (0-0)	0 (0-0)	0 (0-0)	4 (1-7)	0 (0-0)	0 (0-0)	0 (0-0)
2000	Yemen	1092 (378-1719)		35 (12-56)		188 (65-296)		6 (2-10)	
2001	Yemen	1029 (353-1624)		34 (12-53)		177 (61-280)		6 (2-9)	
2002	Yemen	976 (332-1542)		32 (11-50)		168 (57-266)		5 (2-9)	
2003	Yemen	935 (315-1480)		30 (10-47)		161 (54-255)		5 (2-8)	
2004	Yemen	894 (298-1416)		28 (9-44)		154 (51-244)		5 (2-8)	
2005	Yemen	556 (165-909)		17 (5-28)		187 (56-306)		6 (2-9)	
2006	Yemen	93 (27-152)		3 (1-4)		31 (9-51)		1 (0-2)	
2007	Yemen	84 (24-138)		2 (1-4)		28 (8-46)		1 (0-1)	
2008	Yemen	78 (22-128)		2 (1-4)		26 (8-43)		1 (0-1)	
2009	Yemen	74 (21-122)		2 (1-3)		25 (7-41)		1 (0-1)	
2010	Yemen	6 (2-10)		0 (0-0)		2 (1-3)		0 (0-0)	
2011	Yemen	6 (2-9)		0 (0-0)		2 (1-3)		0 (0-0)	
2012	Yemen	5 (1-9)		0 (0-0)		2 (0-3)		0 (0-0)	
2013	Yemen	5 (1-8)		0 (0-0)		2 (0-3)		0 (0-0)	
2014	Yemen	5 (1-8)		0 (0-0)		2 (0-3)		0 (0-0)	
2015	Yemen	4 (1-7)	0 (0-0)	0 (0-0)	0 (0-0)	2 (0-3)	0 (0-0)	0 (0-0)	0 (0-0)
2000	Zambia	1350 (746-1911)		72 (40-101)		233 (128-329)		12 (7-17)	
2001	Zambia	1348 (748-1903)		69 (38-98)		232 (129-328)		12 (7-17)	
2002	Zambia	1331 (552-2047)		66 (28-102)		229 (95-353)		11 (5-18)	
2003	Zambia	1292 (535-1988)		63 (26-96)		222 (92-342)		11 (4-17)	
2004	Zambia	206 (85-318)		10 (4-15)		36 (15-55)		2 (1-3)	
2005	Zambia	116 (48-178)		5 (2-8)		20 (8-31)		1 (0-1)	
2006	Zambia	114 (47-175)		5 (2-8)		20 (8-30)		1 (0-1)	
2007	Zambia	112 (46-174)		5 (2-7)		19 (8-30)		1 (0-1)	
2008	Zambia	97 (40-149)		4 (2-6)		17 (7-26)		1 (0-1)	
2009	Zambia	11 (4-17)		0 (0-1)		2 (1-3)		0 (0-0)	
2010	Zambia	11 (4-16)		0 (0-1)		2 (1-3)		0 (0-0)	
2011	Zambia	10 (4-16)		0 (0-1)		2 (1-3)		0 (0-0)	
2012	Zambia	10 (4-16)		0 (0-1)		3 (1-5)		0 (0-0)	
2013	Zambia	10 (4-15)		0 (0-1)		3 (1-5)		0 (0-0)	
2014	Zambia	10 (4-15)		0 (0-1)		3 (1-5)		0 (0-0)	
2015	Zambia	10 (4-15)	0 (0-1)	0 (0-1)	0 (0-0)	3 (1-5)	0 (0-0)	0 (0-0)	0 (0-0)
2000	Zimbabwe	368 (105-609)		20 (6-34)		63 (18-105)		4 (1-6)	
2001	Zimbabwe	384 (109-635)		21 (6-35)		66 (19-109)		4 (1-6)	
2002	Zimbabwe	408 (116-676)		22 (6-37)		70 (20-116)		4 (1-6)	
2003	Zimbabwe	440 (125-727)		24 (7-39)		76 (22-125)		4 (1-7)	
2004	Zimbabwe	479 (137-793)		26 (7-42)		83 (24-137)		4 (1-7)	
2005	Zimbabwe	528 (151-873)		28 (8-46)		91 (26-150)		5 (1-8)	
2006	Zimbabwe	577 (165-956)		30 (8-49)		99 (28-165)		5 (1-8)	
2007	Zimbabwe	636 (190-1046)		32 (9-52)		110 (33-180)		5 (2-9)	
2008	Zimbabwe	159 (49-259)		8 (2-13)		27 (9-45)		1 (0-2)	
2009	Zimbabwe	83 (27-134)		4 (1-6)		14 (5-23)		1 (0-1)	
2010	Zimbabwe	65 (22-104)		3 (1-5)		11 (4-18)		1 (0-1)	
2011	Zimbabwe	62 (21-100)		3 (1-4)		11 (4-17)		0 (0-1)	
2012	Zimbabwe	58 (20-93)		3 (1-4)		10 (4-16)		0 (0-1)	
2013	Zimbabwe	9 (3-14)		0 (0-1)		3 (1-5)		0 (0-0)	
2014	Zimbabwe	9 (3-15)		0 (0-1)		3 (1-5)		0 (0-0)	
2015	Zimbabwe	9 (3-15)	0 (0-1)	0 (0-1)	0 (0-0)	3 (1-5)	0 (0-0)	0 (0-0)	0 (0-0)

Hib = *Haemophilus influenzae* type b; npnm = non-pneumonia, non-meningitis; HIV = cases only in HIV-infected children; Uncertainty range provided in parentheses.

Webappendix 12

Table 11: Comparison of pneumococcal pneumonia mortality estimates from sensitivity analyses on vaccine-type pneumococcal pneumonia efficacy

		Base Case	Vaccine-type AOM efficacy adjustment	VT pneumococcal pneumonia efficacy adjustment (from Dutch elderly trial)
Year	Country	Spn pneumonia deaths	Spn pneumonia deaths	Spn pneumonia deaths
2000	Afghanistan	8257 (5855-8605)	12316 (8306-12971)	15851 (9643-16977)
2001	Afghanistan	8415 (5967-8769)	12551 (8464-13219)	16154 (9827-17302)
2002	Afghanistan	8520 (6041-8879)	12708 (8570-13384)	16356 (9950-17517)
2003	Afghanistan	8598 (6097-8960)	12825 (8649-13507)	16506 (10042-17678)
2004	Afghanistan	8603 (6101-8966)	12833 (8654-13515)	16516 (10048-17689)
2005	Afghanistan	8534 (6051-8894)	12729 (8584-13406)	16383 (9967-17547)
2006	Afghanistan	8493 (6022-8851)	12668 (8543-13342)	16304 (9919-17462)
2007	Afghanistan	8394 (5952-8747)	12520 (8443-13185)	16113 (9803-17258)
2008	Afghanistan	8267 (5862-8616)	12331 (8316-12987)	15871 (9655-16998)
2009	Afghanistan	8113 (5753-8455)	12102 (8161-12745)	15575 (9475-16682)
2010	Afghanistan	7920 (5616-8254)	11814 (7967-12442)	15205 (9250-16284)
2011	Afghanistan	7663 (5434-7986)	11429 (7708-12037)	14710 (8949-15755)
2012	Afghanistan	7369 (5225-7679)	10991 (7412-11576)	14146 (8606-15151)
2013	Afghanistan	7046 (4996-7343)	10510 (7088-11069)	13526 (8229-14487)
2014	Afghanistan	5496 (3897-5728)	8198 (5528-8634)	10551 (6419-11300)
2015	Afghanistan	4199 (2978-4376)	6263 (4224-6596)	8061 (4904-8634)
2000	Albania	72 (51-75)	107 (72-113)	138 (84-148)
2001	Albania	62 (44-65)	93 (62-98)	119 (73-128)
2002	Albania	53 (38-55)	79 (53-83)	102 (62-109)
2003	Albania	46 (33-48)	69 (46-72)	88 (54-95)
2004	Albania	40 (28-42)	60 (40-63)	77 (47-82)
2005	Albania	35 (25-36)	52 (35-54)	66 (40-71)
2006	Albania	30 (21-31)	45 (30-47)	58 (35-62)
2007	Albania	26 (19-28)	39 (27-42)	51 (31-54)
2008	Albania	23 (16-24)	34 (23-36)	44 (27-48)
2009	Albania	23 (17-24)	35 (23-37)	45 (27-48)
2010	Albania	23 (16-24)	34 (23-36)	44 (27-47)
2011	Albania	14 (10-15)	23 (16-25)	33 (20-35)
2012	Albania	12 (8-12)	20 (13-21)	29 (18-31)
2013	Albania	11 (8-11)	19 (13-20)	28 (17-30)
2014	Albania	11 (8-12)	19 (13-20)	29 (17-31)
2015	Albania	9 (6-9)	15 (10-16)	24 (15-26)
2000	Algeria	1054 (747-1098)	1572 (1060-1656)	2023 (1231-2167)
2001	Algeria	1007 (714-1049)	1501 (1012-1581)	1932 (1176-2070)
2002	Algeria	964 (684-1005)	1438 (970-1515)	1851 (1126-1983)
2003	Algeria	932 (661-972)	1391 (938-1465)	1790 (1089-1917)
2004	Algeria	911 (646-949)	1358 (916-1430)	1748 (1063-1872)
2005	Algeria	894 (634-932)	1334 (900-1405)	1717 (1044-1839)
2006	Algeria	901 (639-939)	1343 (906-1415)	1729 (1052-1852)
2007	Algeria	908 (644-947)	1355 (914-1427)	1744 (1061-1868)
2008	Algeria	930 (659-969)	1387 (935-1461)	1785 (1086-1912)
2009	Algeria	941 (667-980)	1403 (946-1478)	1806 (1098-1934)
2010	Algeria	950 (674-990)	1417 (956-1493)	1824 (1110-1954)
2011	Algeria	956 (678-997)	1427 (962-1502)	1836 (1117-1966)
2012	Algeria	969 (687-1010)	1446 (975-1523)	1861 (1132-1993)
2013	Algeria	978 (694-1019)	1459 (984-1537)	1878 (1142-2011)
2014	Algeria	974 (691-1015)	1453 (980-1530)	1870 (1138-2003)
2015	Algeria	972 (689-1013)	1449 (977-1526)	1865 (1135-1998)
2000	Andorra	0 (0-0)	0 (0-0)	0 (0-0)
2001	Andorra	0 (0-0)	0 (0-0)	0 (0-0)
2002	Andorra	0 (0-0)	0 (0-0)	0 (0-0)
2003	Andorra	0 (0-0)	0 (0-0)	0 (0-0)
2004	Andorra	0 (0-0)	0 (0-0)	0 (0-0)
2005	Andorra	0 (0-0)	0 (0-0)	0 (0-0)
2006	Andorra	0 (0-0)	0 (0-0)	0 (0-0)
2007	Andorra	0 (0-0)	0 (0-0)	0 (0-0)
2008	Andorra	0 (0-0)	0 (0-0)	0 (0-0)
2009	Andorra	0 (0-0)	0 (0-0)	0 (0-0)
2010	Andorra	0 (0-0)	0 (0-0)	0 (0-0)
2011	Andorra	0 (0-0)	0 (0-0)	0 (0-0)
2012	Andorra	0 (0-0)	0 (0-0)	0 (0-0)
2013	Andorra	0 (0-0)	0 (0-0)	0 (0-0)
2014	Andorra	0 (0-0)	0 (0-0)	0 (0-0)
2015	Andorra	0 (0-0)	0 (0-0)	0 (0-0)
2000	Angola	9355 (6633-9749)	13953 (9410-14695)	17958 (10925-19234)
2001	Angola	9584 (6796-9988)	14295 (9640-15055)	18398 (11193-19705)
2002	Angola	10074 (7143-10499)	15026 (10133-15825)	19339 (11765-20713)
2003	Angola	10368 (7352-10806)	15466 (10430-16288)	19905 (12109-21319)
2004	Angola	10743 (7618-11196)	16024 (10806-16876)	20624 (12547-22089)
2005	Angola	11195 (7938-11667)	16698 (11261-17586)	21491 (13074-23017)
2006	Angola	11687 (8287-12179)	17432 (11756-18359)	22435 (13649-24029)
2007	Angola	12096 (8577-12606)	18042 (12167-19002)	23221 (14127-24871)
2008	Angola	12432 (8815-12956)	18543 (12505-19529)	23866 (14519-25561)
2009	Angola	12606 (8939-13137)	18802 (12680-19802)	24199 (14722-25918)
2010	Angola	12681 (8992-13216)	18915 (12756-19921)	24345 (14810-26074)
2011	Angola	12611 (8943-13143)	18811 (12686-19811)	24210 (14728-25930)
2012	Angola	12462 (8837-12988)	18589 (12536-19577)	23924 (14554-25624)
2013	Angola	11867 (8415-12368)	17701 (11937-18642)	22782 (13860-24400)
2014	Angola	10033 (7115-10456)	14966 (10093-15762)	19262 (11718-20630)
2015	Angola	8704 (6172-9071)	12984 (8756-13674)	16710 (10166-17897)
2000	Antigua and Barbuda	0 (0-0)	0 (0-0)	0 (0-0)
2001	Antigua and Barbuda	0 (0-0)	0 (0-0)	0 (0-0)
2002	Antigua and Barbuda	0 (0-0)	0 (0-0)	1 (0-1)
2003	Antigua and Barbuda	0 (0-0)	1 (0-1)	1 (0-1)
2004	Antigua and Barbuda	0 (0-0)	1 (0-1)	1 (0-1)
2005	Antigua and Barbuda	0 (0-0)	1 (0-1)	1 (0-1)
2006	Antigua and Barbuda	0 (0-0)	0 (0-0)	1 (0-1)
2007	Antigua and Barbuda	0 (0-0)	0 (0-0)	1 (0-1)
2008	Antigua and Barbuda	0 (0-0)	0 (0-0)	0 (0-1)
2009	Antigua and Barbuda	0 (0-0)	0 (0-0)	1 (0-1)
2010	Antigua and Barbuda	0 (0-0)	0 (0-0)	0 (0-0)
2011	Antigua and Barbuda	0 (0-0)	0 (0-0)	0 (0-0)
2012	Antigua and Barbuda	0 (0-0)	0 (0-0)	0 (0-0)
2013	Antigua and Barbuda	0 (0-0)	0 (0-0)	0 (0-0)
2014	Antigua and Barbuda	0 (0-0)	0 (0-0)	0 (0-0)
2015	Antigua and Barbuda	0 (0-0)	0 (0-0)	0 (0-0)
2000	Argentina	350 (248-365)	522 (352-550)	672 (409-719)
2001	Argentina	363 (258-378)	542 (365-570)	697 (424-747)
2002	Argentina	349 (248-364)	521 (351-548)	670 (408-718)
2003	Argentina	549 (390-572)	819 (553-863)	1055 (642-1129)
2004	Argentina	398 (282-414)	593 (400-625)	763 (464-818)
2005	Argentina	320 (227-334)	478 (322-503)	615 (374-659)
2006	Argentina	340 (241-355)	508 (343-535)	654 (398-700)
2007	Argentina	398 (283-415)	594 (401-626)	765 (465-819)
2008	Argentina	342 (243-356)	510 (344-537)	657 (399-703)
2009	Argentina	418 (296-435)	623 (420-656)	802 (488-859)
2010	Argentina	309 (219-322)	461 (311-485)	593 (361-635)

Spn = *Streptococcus pneumoniae*; Uncertainty range provided in parentheses.

Year	Country	Spn pneumonia deaths (UR)	Spn pneumonia deaths (UR)	Spn pneumonia deaths (UR)
2011	Argentina	314 (223-328)	469 (316-494)	604 (367-646)
2012	Argentina	238 (168-248)	366 (247-385)	484 (294-518)
2013	Argentina	190 (135-198)	309 (208-325)	432 (263-462)
2014	Argentina	163 (115-169)	271 (183-286)	390 (237-418)
2015	Argentina	154 (109-161)	259 (174-272)	373 (227-400)
2000	Armenia	52 (37-54)	78 (52-82)	100 (61-107)
2001	Armenia	49 (35-51)	73 (49-77)	94 (57-100)
2002	Armenia	46 (33-48)	69 (47-73)	89 (54-95)
2003	Armenia	45 (32-46)	66 (45-70)	85 (52-92)
2004	Armenia	43 (30-44)	64 (43-67)	82 (50-88)
2005	Armenia	40 (28-42)	60 (40-63)	77 (47-82)
2006	Armenia	37 (26-39)	56 (37-58)	71 (43-77)
2007	Armenia	35 (25-36)	52 (35-55)	67 (41-72)
2008	Armenia	32 (22-33)	47 (32-49)	60 (37-65)
2009	Armenia	29 (21-30)	43 (29-46)	56 (34-60)
2010	Armenia	28 (20-29)	41 (28-43)	53 (32-57)
2011	Armenia	27 (19-29)	41 (28-43)	53 (32-56)
2012	Armenia	25 (18-26)	37 (25-39)	48 (29-51)
2013	Armenia	22 (16-23)	33 (23-35)	43 (26-46)
2014	Armenia	20 (14-21)	30 (21-32)	39 (24-42)
2015	Armenia	16 (12-17)	25 (17-26)	33 (20-36)
2000	Australia	15 (10-15)	22 (15-23)	28 (17-30)
2001	Australia	21 (15-21)	31 (21-32)	39 (24-42)
2002	Australia	16 (11-16)	23 (16-24)	30 (18-32)
2003	Australia	22 (16-23)	33 (22-35)	43 (26-46)
2004	Australia	28 (20-29)	42 (28-44)	54 (33-57)
2005	Australia	18 (12-18)	26 (18-28)	34 (20-36)
2006	Australia	14 (10-14)	22 (15-23)	30 (18-32)
2007	Australia	13 (9-14)	22 (15-23)	30 (18-32)
2008	Australia	10 (7-10)	16 (11-17)	22 (13-23)
2009	Australia	11 (8-11)	18 (12-19)	26 (16-27)
2010	Australia	17 (12-18)	26 (18-28)	34 (21-36)
2011	Australia	9 (6-9)	15 (10-15)	20 (12-22)
2012	Australia	5 (4-5)	9 (6-9)	14 (9-15)
2013	Australia	5 (3-5)	8 (6-9)	13 (8-14)
2014	Australia	4 (3-5)	8 (5-8)	12 (8-13)
2015	Australia	4 (3-5)	8 (5-8)	12 (8-13)
2000	Austria	2 (2-2)	3 (2-3)	4 (2-4)
2001	Austria	4 (3-4)	6 (4-6)	7 (5-8)
2002	Austria	5 (3-5)	7 (5-7)	9 (6-10)
2003	Austria	2 (1-2)	3 (2-3)	3 (2-4)
2004	Austria	5 (3-5)	7 (5-7)	9 (6-10)
2005	Austria	1 (0-1)	1 (1-1)	1 (1-1)
2006	Austria	2 (1-2)	3 (2-3)	3 (2-4)
2007	Austria	2 (1-2)	3 (2-3)	4 (2-4)
2008	Austria	2 (1-2)	3 (2-3)	4 (2-4)
2009	Austria	2 (2-3)	4 (2-4)	5 (3-5)
2010	Austria	2 (1-2)	3 (2-3)	4 (2-4)
2011	Austria	4 (3-4)	6 (4-6)	8 (5-8)
2012	Austria	3 (2-3)	5 (3-5)	6 (4-6)
2013	Austria	1 (1-1)	2 (1-2)	2 (1-2)
2014	Austria	2 (1-2)	3 (2-3)	4 (2-4)
2015	Austria	2 (1-2)	3 (2-3)	4 (2-4)
2000	Azerbaijan	559 (397-583)	835 (563-879)	1074 (653-1150)
2001	Azerbaijan	485 (344-506)	724 (488-762)	931 (567-998)
2002	Azerbaijan	429 (305-448)	641 (432-675)	824 (502-883)
2003	Azerbaijan	388 (275-405)	579 (391-610)	745 (454-798)
2004	Azerbaijan	361 (256-376)	538 (363-567)	692 (421-742)
2005	Azerbaijan	337 (239-351)	502 (339-529)	646 (393-692)
2006	Azerbaijan	321 (228-335)	479 (323-504)	616 (375-660)
2007	Azerbaijan	305 (217-318)	455 (307-480)	586 (357-628)
2008	Azerbaijan	295 (209-307)	439 (296-463)	565 (344-606)
2009	Azerbaijan	288 (204-300)	430 (290-452)	553 (336-592)
2010	Azerbaijan	288 (204-300)	430 (290-453)	553 (337-592)
2011	Azerbaijan	294 (209-307)	439 (296-463)	565 (344-605)
2012	Azerbaijan	303 (215-316)	452 (305-476)	582 (354-623)
2013	Azerbaijan	313 (222-326)	467 (315-491)	601 (365-643)
2014	Azerbaijan	230 (163-240)	343 (231-361)	442 (269-473)
2015	Azerbaijan	163 (116-170)	243 (164-256)	313 (191-336)
2000	Bahamas	4 (3-4)	6 (4-7)	8 (5-9)
2001	Bahamas	3 (2-4)	5 (4-5)	7 (4-7)
2002	Bahamas	3 (2-3)	4 (3-4)	5 (3-6)
2003	Bahamas	2 (1-2)	2 (2-2)	3 (2-3)
2004	Bahamas	1 (1-1)	2 (1-2)	3 (2-3)
2005	Bahamas	2 (1-2)	3 (2-3)	3 (2-3)
2006	Bahamas	2 (1-2)	3 (2-3)	4 (2-4)
2007	Bahamas	3 (2-3)	4 (3-5)	6 (3-6)
2008	Bahamas	4 (3-4)	6 (4-6)	7 (4-8)
2009	Bahamas	4 (3-4)	6 (4-6)	8 (5-8)
2010	Bahamas	3 (2-3)	5 (3-5)	6 (4-7)
2011	Bahamas	3 (2-3)	5 (3-5)	6 (4-7)
2012	Bahamas	3 (2-3)	4 (3-5)	6 (4-6)
2013	Bahamas	2 (1-2)	3 (2-3)	5 (3-5)
2014	Bahamas	1 (1-1)	2 (1-2)	3 (2-3)
2015	Bahamas	1 (1-1)	2 (1-2)	3 (2-3)
2000	Bahrain	1 (1-1)	2 (1-2)	2 (1-3)
2001	Bahrain	3 (2-3)	4 (3-4)	5 (3-5)
2002	Bahrain	3 (2-3)	4 (3-5)	6 (3-6)
2003	Bahrain	3 (2-3)	5 (3-5)	6 (4-7)
2004	Bahrain	2 (2-3)	4 (2-4)	5 (3-5)
2005	Bahrain	2 (2-3)	4 (3-4)	5 (3-5)
2006	Bahrain	3 (2-3)	4 (3-4)	5 (3-5)
2007	Bahrain	3 (2-3)	5 (3-5)	6 (4-7)
2008	Bahrain	3 (2-3)	5 (3-5)	6 (4-7)
2009	Bahrain	3 (2-3)	4 (3-5)	6 (4-7)
2010	Bahrain	2 (1-2)	3 (2-3)	4 (3-5)
2011	Bahrain	1 (1-1)	2 (2-2)	3 (2-4)
2012	Bahrain	1 (0-1)	1 (1-1)	2 (1-2)
2013	Bahrain	0 (0-1)	1 (1-1)	1 (1-1)
2014	Bahrain	1 (0-1)	1 (1-1)	2 (1-2)
2015	Bahrain	1 (0-1)	1 (1-1)	1 (1-1)
2000	Bangladesh	16671 (11822-17374)	24867 (16770-26189)	32005 (19470-34278)
2001	Bangladesh	15795 (11200-16461)	23560 (15888-24813)	30323 (18447-32476)
2002	Bangladesh	14261 (10113-14863)	21272 (14346-22403)	27378 (16656-29323)
2003	Bangladesh	13286 (9421-13846)	19818 (13365-20872)	25506 (15517-27318)
2004	Bangladesh	12289 (8714-12807)	18330 (12361-19305)	23591 (14352-25267)
2005	Bangladesh	11650 (8261-12141)	17377 (11719-18301)	22365 (13606-23953)
2006	Bangladesh	10654 (7555-11103)	15891 (10717-16736)	20452 (12442-21905)
2007	Bangladesh	9724 (6895-10134)	14504 (9782-15276)	18668 (11357-19994)

Spn = Streptococcus pneumoniae; Uncertainty range provided in parentheses.

Year	Country	Spn pneumonia deaths (UR)	Spn pneumonia deaths (UR)	Spn pneumonia deaths (UR)
2008	Bangladesh	8777 (6224-9147)	13091 (8829-13788)	16849 (10250-18046)
2009	Bangladesh	7935 (5627-8270)	11836 (7982-12466)	15234 (9267-16316)
2010	Bangladesh	7258 (5147-7564)	10826 (7301-11401)	13933 (8476-14923)
2011	Bangladesh	6629 (4700-6908)	9887 (6668-10413)	12726 (7742-13629)
2012	Bangladesh	6123 (4342-6381)	9132 (6159-9618)	11754 (7151-12589)
2013	Bangladesh	5655 (4010-5893)	8435 (5688-8883)	10856 (6604-11627)
2014	Bangladesh	5273 (3739-5495)	7865 (5304-8283)	10123 (6158-10842)
2015	Bangladesh	3886 (2755-4049)	5796 (3908-6104)	7459 (4538-7989)
2000	Barbados	0 (0-0)	0 (0-0)	0 (0-0)
2001	Barbados	0 (0-0)	1 (0-1)	1 (0-1)
2002	Barbados	1 (0-1)	1 (1-1)	1 (1-1)
2003	Barbados	1 (0-1)	1 (1-1)	1 (1-1)
2004	Barbados	1 (1-1)	1 (1-1)	2 (1-2)
2005	Barbados	1 (1-1)	2 (1-2)	2 (1-2)
2006	Barbados	2 (1-2)	2 (2-3)	3 (2-3)
2007	Barbados	1 (1-1)	2 (1-2)	3 (2-3)
2008	Barbados	1 (1-1)	1 (1-1)	2 (1-2)
2009	Barbados	0 (0-0)	0 (0-1)	1 (0-1)
2010	Barbados	0 (0-0)	1 (0-1)	1 (1-1)
2011	Barbados	0 (0-0)	1 (0-1)	1 (1-1)
2012	Barbados	0 (0-0)	1 (1-1)	1 (1-1)
2013	Barbados	0 (0-0)	0 (0-0)	0 (0-0)
2014	Barbados	0 (0-0)	0 (0-0)	1 (0-1)
2015	Barbados	0 (0-0)	0 (0-0)	1 (0-1)
2000	Belarus	45 (32-47)	67 (45-70)	86 (52-92)
2001	Belarus	39 (28-41)	59 (40-62)	76 (46-81)
2002	Belarus	35 (25-36)	52 (35-55)	67 (41-72)
2003	Belarus	31 (22-32)	46 (31-48)	59 (36-63)
2004	Belarus	29 (21-30)	43 (29-46)	56 (34-60)
2005	Belarus	26 (18-27)	39 (26-41)	50 (30-54)
2006	Belarus	24 (17-25)	36 (24-38)	46 (28-49)
2007	Belarus	21 (15-22)	31 (21-33)	40 (25-43)
2008	Belarus	18 (13-19)	28 (19-29)	35 (22-38)
2009	Belarus	17 (12-18)	26 (17-27)	33 (20-35)
2010	Belarus	15 (11-16)	22 (15-23)	29 (17-31)
2011	Belarus	13 (9-13)	19 (13-20)	25 (15-27)
2012	Belarus	13 (9-13)	19 (13-20)	25 (15-26)
2013	Belarus	12 (9-13)	18 (12-19)	23 (14-25)
2014	Belarus	11 (8-11)	16 (11-17)	21 (13-23)
2015	Belarus	10 (7-11)	16 (11-16)	20 (12-21)
2000	Belgium	3 (2-3)	4 (3-4)	5 (3-5)
2001	Belgium	2 (2-2)	4 (2-4)	5 (3-5)
2002	Belgium	8 (5-8)	12 (8-12)	15 (9-16)
2003	Belgium	4 (3-4)	6 (4-7)	8 (5-9)
2004	Belgium	2 (2-3)	4 (2-4)	5 (3-5)
2005	Belgium	8 (5-8)	11 (8-12)	15 (9-16)
2006	Belgium	3 (2-3)	4 (3-5)	6 (3-6)
2007	Belgium	7 (5-8)	11 (7-12)	14 (9-15)
2008	Belgium	4 (3-4)	6 (4-6)	7 (4-8)
2009	Belgium	6 (4-6)	9 (6-9)	12 (7-12)
2010	Belgium	6 (4-6)	9 (6-10)	12 (7-13)
2011	Belgium	4 (3-4)	6 (4-7)	8 (5-9)
2012	Belgium	0 (0-0)	1 (0-1)	1 (1-1)
2013	Belgium	2 (1-2)	3 (2-3)	4 (3-5)
2014	Belgium	2 (1-2)	3 (2-3)	4 (2-4)
2015	Belgium	1 (1-1)	2 (2-3)	4 (2-4)
2000	Belize	8 (6-8)	12 (8-12)	15 (9-16)
2001	Belize	7 (5-7)	11 (7-11)	14 (8-14)
2002	Belize	11 (8-12)	17 (11-18)	22 (13-23)
2003	Belize	9 (6-9)	13 (9-14)	17 (11-19)
2004	Belize	9 (6-9)	14 (9-14)	17 (11-19)
2005	Belize	5 (3-5)	7 (5-8)	9 (6-10)
2006	Belize	9 (7-10)	14 (9-15)	18 (11-19)
2007	Belize	2 (1-2)	3 (2-3)	3 (2-4)
2008	Belize	3 (2-3)	4 (3-4)	5 (3-5)
2009	Belize	3 (2-3)	4 (3-5)	6 (3-6)
2010	Belize	4 (3-4)	6 (4-6)	8 (5-8)
2011	Belize	1 (1-1)	1 (1-1)	1 (1-2)
2012	Belize	8 (6-9)	12 (8-13)	16 (10-17)
2013	Belize	3 (2-3)	4 (3-4)	5 (3-6)
2014	Belize	4 (3-4)	6 (4-6)	8 (5-8)
2015	Belize	4 (3-4)	6 (4-6)	8 (5-8)
2000	Benin	2211 (1568-2304)	3297 (2224-3473)	4244 (2582-4545)
2001	Benin	2164 (1535-2256)	3229 (2177-3400)	4155 (2528-4450)
2002	Benin	2148 (1523-2239)	3204 (2161-3375)	4124 (2509-4417)
2003	Benin	2122 (1505-2211)	3165 (2134-3333)	4073 (2478-4363)
2004	Benin	2086 (1479-2174)	3111 (2098-3276)	4004 (2436-4288)
2005	Benin	2057 (1459-2144)	3068 (2069-3232)	3949 (2403-4230)
2006	Benin	2107 (1494-2196)	3143 (2120-3310)	4045 (2461-4333)
2007	Benin	2136 (1515-2226)	3186 (2149-3356)	4101 (2495-4392)
2008	Benin	2188 (1552-2280)	3264 (2201-3437)	4201 (2555-4499)
2009	Benin	2245 (1592-2340)	3349 (2259-3527)	4311 (2622-4617)
2010	Benin	2294 (1627-2390)	3421 (2307-3603)	4404 (2679-4716)
2011	Benin	2014 (1428-2099)	3004 (2026-3163)	3866 (2352-4140)
2012	Benin	1539 (1091-1604)	2296 (1548-2418)	2955 (1798-3165)
2013	Benin	1464 (1038-1525)	2183 (1472-2299)	2810 (1709-3010)
2014	Benin	1369 (971-1427)	2042 (1377-2151)	2629 (1599-2815)
2015	Benin	898 (637-936)	1339 (903-1410)	1724 (1049-1846)
2000	Bhutan	78 (55-81)	116 (78-122)	149 (91-160)
2001	Bhutan	71 (50-74)	106 (71-111)	136 (83-145)
2002	Bhutan	64 (46-67)	96 (65-101)	124 (75-133)
2003	Bhutan	58 (41-60)	86 (58-91)	111 (68-119)
2004	Bhutan	52 (37-54)	78 (52-82)	100 (61-107)
2005	Bhutan	47 (34-49)	70 (48-74)	91 (55-97)
2006	Bhutan	43 (31-45)	65 (44-68)	83 (51-89)
2007	Bhutan	40 (28-42)	60 (40-63)	77 (47-82)
2008	Bhutan	37 (26-38)	55 (37-58)	71 (43-76)
2009	Bhutan	34 (24-35)	50 (34-53)	65 (39-69)
2010	Bhutan	31 (22-32)	46 (31-48)	59 (36-63)
2011	Bhutan	28 (20-29)	42 (28-44)	53 (32-57)
2012	Bhutan	25 (18-26)	37 (25-39)	48 (29-51)
2013	Bhutan	22 (16-23)	33 (22-35)	43 (26-46)
2014	Bhutan	20 (14-21)	30 (20-32)	39 (24-42)
2015	Bhutan	19 (13-20)	28 (19-30)	36 (22-39)
2000	Bolivia (Plurinational State of)	1187 (841-1237)	1770 (1194-1864)	2278 (1386-2440)
2001	Bolivia (Plurinational State of)	1116 (791-1163)	1665 (1123-1753)	2143 (1304-2295)
2002	Bolivia (Plurinational State of)	1036 (734-1079)	1545 (1042-1627)	1988 (1210-2129)
2003	Bolivia (Plurinational State of)	955 (677-995)	1424 (960-1500)	1833 (1115-1963)
2004	Bolivia (Plurinational State of)	872 (618-908)	1300 (877-1369)	1673 (1018-1792)

Spn = Streptococcus pneumoniae; Uncertainty range provided in parentheses.

Year	Country	Spn pneumonia deaths (UR)	Spn pneumonia deaths (UR)	Spn pneumonia deaths (UR)
2005	Bolivia (Plurinational State of)	801 (568-835)	1195 (806-1259)	1539 (936-1648)
2006	Bolivia (Plurinational State of)	741 (525-772)	1105 (745-1164)	1422 (865-1523)
2007	Bolivia (Plurinational State of)	688 (488-717)	1027 (692-1081)	1321 (804-1415)
2008	Bolivia (Plurinational State of)	650 (461-677)	969 (654-1021)	1247 (759-1336)
2009	Bolivia (Plurinational State of)	613 (434-638)	914 (616-962)	1176 (715-1259)
2010	Bolivia (Plurinational State of)	577 (410-602)	861 (581-907)	1109 (674-1187)
2011	Bolivia (Plurinational State of)	542 (384-565)	809 (545-852)	1041 (633-1115)
2012	Bolivia (Plurinational State of)	513 (364-534)	765 (516-806)	984 (599-1054)
2013	Bolivia (Plurinational State of)	481 (341-501)	717 (484-755)	923 (562-989)
2014	Bolivia (Plurinational State of)	351 (249-366)	524 (353-552)	675 (410-723)
2015	Bolivia (Plurinational State of)	245 (174-255)	365 (246-385)	470 (286-504)
2000	Bosnia and Herzegovina	6 (4-6)	9 (6-9)	11 (7-12)
2001	Bosnia and Herzegovina	5 (4-5)	8 (5-8)	10 (6-11)
2002	Bosnia and Herzegovina	5 (3-5)	7 (5-7)	9 (6-10)
2003	Bosnia and Herzegovina	4 (3-5)	7 (4-7)	8 (5-9)
2004	Bosnia and Herzegovina	4 (3-4)	6 (4-7)	8 (5-9)
2005	Bosnia and Herzegovina	5 (3-5)	7 (5-7)	9 (5-9)
2006	Bosnia and Herzegovina	4 (3-4)	6 (4-7)	8 (5-9)
2007	Bosnia and Herzegovina	5 (3-5)	7 (5-7)	9 (5-9)
2008	Bosnia and Herzegovina	5 (3-5)	7 (5-8)	9 (6-10)
2009	Bosnia and Herzegovina	5 (3-5)	7 (5-7)	9 (5-9)
2010	Bosnia and Herzegovina	4 (3-4)	6 (4-6)	8 (5-8)
2011	Bosnia and Herzegovina	3 (2-3)	5 (3-5)	6 (4-7)
2012	Bosnia and Herzegovina	3 (2-3)	4 (3-5)	6 (3-6)
2013	Bosnia and Herzegovina	2 (2-2)	4 (2-4)	5 (3-5)
2014	Bosnia and Herzegovina	2 (1-2)	3 (2-3)	4 (2-4)
2015	Bosnia and Herzegovina	2 (1-2)	2 (2-3)	3 (2-3)
2000	Botswana	86 (61-89)	128 (86-135)	164 (100-176)
2001	Botswana	88 (62-92)	131 (89-138)	169 (103-181)
2002	Botswana	91 (64-95)	136 (91-143)	175 (106-187)
2003	Botswana	94 (67-98)	140 (95-148)	181 (110-194)
2004	Botswana	99 (70-103)	147 (99-155)	190 (115-203)
2005	Botswana	103 (73-108)	154 (104-162)	198 (121-212)
2006	Botswana	106 (76-111)	159 (107-167)	204 (124-219)
2007	Botswana	110 (78-115)	165 (111-174)	212 (129-227)
2008	Botswana	114 (81-118)	169 (114-178)	218 (133-233)
2009	Botswana	115 (82-120)	171 (116-181)	221 (134-236)
2010	Botswana	115 (82-120)	172 (116-181)	221 (134-237)
2011	Botswana	117 (83-122)	174 (118-184)	224 (136-240)
2012	Botswana	95 (68-99)	142 (96-150)	183 (111-196)
2013	Botswana	82 (58-85)	122 (82-128)	157 (95-168)
2014	Botswana	68 (48-71)	102 (69-107)	131 (80-140)
2015	Botswana	61 (44-64)	92 (62-97)	118 (72-126)
2000	Brazil	5337 (3784-5562)	7960 (5368-8383)	10245 (6233-10972)
2001	Brazil	4715 (3344-4914)	7033 (4743-7407)	9052 (5507-9695)
2002	Brazil	4095 (2904-4268)	6108 (4119-6433)	7862 (4783-8420)
2003	Brazil	3735 (2648-3892)	5571 (3757-5867)	7170 (4362-7679)
2004	Brazil	3412 (2419-3555)	5089 (3432-5359)	6549 (3984-7014)
2005	Brazil	2877 (2040-2999)	4292 (2894-4520)	5524 (3361-5916)
2006	Brazil	2612 (1852-2722)	3896 (2627-4103)	5014 (3050-5370)
2007	Brazil	2193 (1555-2286)	3271 (2206-3445)	4210 (2561-4509)
2008	Brazil	1791 (1270-1866)	2671 (1801-2813)	3437 (2091-3682)
2009	Brazil	1587 (1125-1654)	2367 (1596-2493)	3046 (1853-3263)
2010	Brazil	1146 (813-1195)	1739 (1172-1831)	2271 (1381-2432)
2011	Brazil	900 (638-938)	1441 (972-1518)	1981 (1205-2122)
2012	Brazil	752 (533-784)	1239 (835-1305)	1754 (1067-1878)
2013	Brazil	753 (534-784)	1263 (852-1330)	1825 (1110-1955)
2014	Brazil	678 (481-706)	1184 (799-1247)	1792 (1090-1919)
2015	Brazil	701 (497-730)	1241 (837-1307)	1907 (1160-2043)
2000	Brunei Darussalam	1 (1-1)	2 (1-2)	2 (1-2)
2001	Brunei Darussalam	1 (1-1)	2 (1-2)	2 (1-2)
2002	Brunei Darussalam	1 (1-1)	2 (1-2)	2 (1-2)
2003	Brunei Darussalam	1 (1-1)	2 (1-2)	2 (1-2)
2004	Brunei Darussalam	1 (1-1)	2 (1-2)	2 (1-2)
2005	Brunei Darussalam	1 (1-1)	2 (1-2)	2 (1-2)
2006	Brunei Darussalam	1 (1-1)	2 (1-2)	2 (1-2)
2007	Brunei Darussalam	1 (1-1)	2 (1-2)	2 (1-2)
2008	Brunei Darussalam	1 (1-1)	2 (1-2)	2 (1-2)
2009	Brunei Darussalam	1 (1-1)	1 (1-2)	2 (1-2)
2010	Brunei Darussalam	1 (1-1)	2 (1-2)	2 (1-2)
2011	Brunei Darussalam	1 (1-1)	1 (1-2)	2 (1-2)
2012	Brunei Darussalam	1 (1-1)	2 (1-2)	2 (1-2)
2013	Brunei Darussalam	1 (1-1)	1 (1-1)	2 (1-2)
2014	Brunei Darussalam	1 (1-1)	1 (1-1)	2 (1-2)
2015	Brunei Darussalam	1 (1-1)	1 (1-1)	1 (1-2)
2000	Bulgaria	84 (60-88)	125 (85-132)	161 (98-173)
2001	Bulgaria	78 (56-82)	117 (79-123)	150 (91-161)
2002	Bulgaria	63 (45-65)	94 (63-99)	121 (73-129)
2003	Bulgaria	73 (52-77)	110 (74-115)	141 (86-151)
2004	Bulgaria	52 (37-54)	77 (52-81)	99 (60-106)
2005	Bulgaria	58 (41-61)	87 (58-91)	112 (68-119)
2006	Bulgaria	56 (40-58)	83 (56-88)	107 (65-115)
2007	Bulgaria	51 (36-53)	76 (51-80)	98 (59-105)
2008	Bulgaria	45 (32-47)	67 (45-71)	87 (53-93)
2009	Bulgaria	67 (47-69)	99 (67-105)	128 (78-137)
2010	Bulgaria	48 (34-50)	76 (52-81)	104 (63-112)
2011	Bulgaria	29 (21-31)	49 (33-52)	71 (43-76)
2012	Bulgaria	25 (17-26)	42 (28-44)	62 (38-66)
2013	Bulgaria	23 (16-24)	39 (26-41)	58 (35-62)
2014	Bulgaria	15 (11-16)	27 (18-28)	41 (25-44)
2015	Bulgaria	13 (9-13)	23 (15-24)	36 (22-39)
2000	Burkina Faso	2822 (2001-2941)	4210 (2839-4434)	5418 (3296-5803)
2001	Burkina Faso	2991 (2121-3117)	4461 (3008-4698)	5741 (3493-6149)
2002	Burkina Faso	3173 (2250-3307)	4733 (3192-4985)	6092 (3706-6525)
2003	Burkina Faso	3363 (2385-3505)	5017 (3383-5283)	6457 (3928-6915)
2004	Burkina Faso	3525 (2499-3673)	5257 (3545-5537)	6766 (4116-7247)
2005	Burkina Faso	3569 (2531-3719)	5323 (3590-5606)	6851 (4168-7338)
2006	Burkina Faso	3454 (2449-3600)	5152 (3474-5426)	6631 (4034-7102)
2007	Burkina Faso	3186 (2259-3320)	4752 (3204-5004)	6115 (3720-6550)
2008	Burkina Faso	2878 (2041-2999)	4293 (2895-4521)	5525 (3361-5917)
2009	Burkina Faso	2719 (1928-2834)	4056 (2735-4271)	5220 (3175-5590)
2010	Burkina Faso	2707 (1919-2821)	4037 (2722-4252)	5196 (3161-5565)
2011	Burkina Faso	2877 (2040-2998)	4291 (2894-4519)	5523 (3360-5915)
2012	Burkina Faso	3054 (2165-3182)	4555 (3072-4797)	5862 (3566-6278)
2013	Burkina Faso	3253 (2307-3390)	4852 (3272-5110)	6244 (3799-6688)
2014	Burkina Faso	2241 (1589-2335)	3342 (2254-3520)	4302 (2617-4607)
2015	Burkina Faso	1882 (1335-1961)	2807 (1893-2956)	3613 (2198-3870)
2000	Burundi	2274 (1613-2370)	3392 (2287-3572)	4366 (2656-4676)
2001	Burundi	2264 (1605-2359)	3376 (2277-3556)	4345 (2644-4654)

Spn = Streptococcus pneumoniae; Uncertainty range provided in parentheses.

Year	Country	Spn pneumonia deaths (UR)	Spn pneumonia deaths (UR)	Spn pneumonia deaths (UR)
2002	Burundi	2224 (1577-2318)	3317 (2237-3494)	4269 (2597-4573)
2003	Burundi	2358 (1672-2457)	3517 (2372-3704)	4527 (2754-4848)
2004	Burundi	2445 (1734-2548)	3647 (2460-3841)	4694 (2856-5028)
2005	Burundi	2433 (1725-2535)	3629 (2447-3822)	4670 (2841-5002)
2006	Burundi	2565 (1819-2673)	3826 (2580-4029)	4924 (2996-5274)
2007	Burundi	2578 (1828-2686)	3845 (2593-4049)	4948 (3010-5300)
2008	Burundi	2574 (1826-2683)	3840 (2590-4044)	4942 (3007-5293)
2009	Burundi	2582 (1831-2691)	3851 (2597-4056)	4956 (3015-5309)
2010	Burundi	2574 (1826-2683)	3840 (2590-4044)	4942 (3007-5293)
2011	Burundi	2459 (1744-2563)	3668 (2474-3863)	4721 (2872-5057)
2012	Burundi	1623 (1151-1691)	2421 (1632-2549)	3115 (1895-3337)
2013	Burundi	1338 (949-1394)	1996 (1346-2102)	2568 (1562-2751)
2014	Burundi	1199 (850-1249)	1788 (1206-1883)	2301 (1400-2465)
2015	Burundi	879 (623-916)	1311 (884-1380)	1687 (1026-1807)
2000	Cambodia	2341 (1660-2440)	3492 (2355-3678)	4494 (2734-4814)
2001	Cambodia	2053 (1456-2139)	3062 (2065-3225)	3941 (2397-4221)
2002	Cambodia	1783 (1264-1858)	2660 (1794-2801)	3423 (2083-3666)
2003	Cambodia	1557 (1104-1622)	2322 (1566-2445)	2988 (1818-3200)
2004	Cambodia	1400 (993-1459)	2088 (1408-2199)	2687 (1635-2878)
2005	Cambodia	1286 (912-1340)	1918 (1293-2020)	2468 (1502-2644)
2006	Cambodia	1199 (851-1250)	1789 (1207-1884)	2303 (1401-2466)
2007	Cambodia	1117 (792-1164)	1665 (1123-1754)	2143 (1304-2296)
2008	Cambodia	1037 (735-1080)	1546 (1043-1628)	1990 (1211-2131)
2009	Cambodia	946 (671-986)	1411 (952-1486)	1816 (1105-1945)
2010	Cambodia	841 (596-876)	1254 (846-1321)	1614 (982-1728)
2011	Cambodia	752 (533-784)	1122 (757-1182)	1444 (878-1546)
2012	Cambodia	657 (466-685)	980 (661-1032)	1261 (767-1351)
2013	Cambodia	594 (422-620)	887 (598-934)	1141 (694-1222)
2014	Cambodia	548 (388-571)	817 (551-860)	1052 (640-1126)
2015	Cambodia	349 (247-364)	520 (351-548)	670 (407-717)
2000	Cameroon	4934 (3499-5142)	7360 (4963-7751)	9472 (5762-10145)
2001	Cameroon	4771 (3383-4973)	7117 (4800-7495)	9160 (5572-9810)
2002	Cameroon	4738 (3360-4938)	7067 (4766-7443)	9096 (5534-9742)
2003	Cameroon	4600 (3262-4794)	6862 (4628-7227)	8832 (5373-9459)
2004	Cameroon	4488 (3182-4677)	6694 (4514-7050)	8615 (5241-9227)
2005	Cameroon	4464 (3165-4652)	6658 (4490-7012)	8569 (5213-9178)
2006	Cameroon	4553 (3228-4745)	6791 (4580-7152)	8740 (5317-9361)
2007	Cameroon	4603 (3264-4797)	6866 (4630-7231)	8837 (5376-9465)
2008	Cameroon	4770 (3383-4971)	7115 (4799-7494)	9158 (5571-9808)
2009	Cameroon	5010 (3553-5221)	7473 (5040-7870)	9618 (5851-10301)
2010	Cameroon	5066 (3593-5280)	7557 (5096-7959)	9726 (5917-10417)
2011	Cameroon	4654 (3300-4850)	6941 (4681-7310)	8934 (5435-9568)
2012	Cameroon	3341 (2369-3482)	4983 (3360-5248)	6413 (3902-6869)
2013	Cameroon	2771 (1965-2888)	4133 (2787-4353)	5319 (3236-5697)
2014	Cameroon	2492 (1767-2597)	3716 (2506-3914)	4783 (2910-5123)
2015	Cameroon	1739 (1233-1812)	2594 (1749-2732)	3338 (2031-3575)
2000	Canada	18 (13-18)	26 (18-28)	34 (21-36)
2001	Canada	18 (12-18)	26 (18-28)	34 (21-36)
2002	Canada	14 (10-14)	21 (14-22)	27 (16-29)
2003	Canada	15 (10-15)	22 (15-23)	29 (17-31)
2004	Canada	12 (8-12)	17 (12-18)	23 (14-24)
2005	Canada	12 (8-12)	18 (12-19)	23 (14-25)
2006	Canada	14 (10-14)	21 (14-22)	28 (17-30)
2007	Canada	18 (13-19)	27 (18-29)	36 (22-39)
2008	Canada	9 (6-9)	14 (9-14)	18 (11-19)
2009	Canada	14 (10-15)	23 (15-24)	31 (19-33)
2010	Canada	10 (7-11)	16 (11-17)	23 (14-24)
2011	Canada	6 (4-6)	10 (7-11)	15 (9-16)
2012	Canada	5 (3-5)	8 (6-9)	13 (8-14)
2013	Canada	4 (3-4)	7 (5-7)	11 (7-12)
2014	Canada	3 (2-3)	6 (4-6)	9 (6-10)
2015	Canada	3 (2-3)	6 (4-6)	9 (6-10)
2000	Cape Verde	22 (16-23)	33 (22-35)	43 (26-46)
2001	Cape Verde	19 (14-20)	29 (19-30)	37 (22-39)
2002	Cape Verde	17 (12-18)	25 (17-26)	32 (20-35)
2003	Cape Verde	15 (11-16)	23 (15-24)	29 (18-31)
2004	Cape Verde	14 (10-15)	21 (14-23)	28 (17-30)
2005	Cape Verde	14 (10-15)	21 (14-22)	27 (16-29)
2006	Cape Verde	14 (10-15)	21 (14-22)	27 (17-29)
2007	Cape Verde	14 (10-15)	21 (14-23)	28 (17-30)
2008	Cape Verde	14 (10-15)	21 (14-23)	28 (17-29)
2009	Cape Verde	14 (10-15)	21 (14-22)	27 (16-29)
2010	Cape Verde	14 (10-14)	20 (14-22)	26 (16-28)
2011	Cape Verde	15 (10-15)	22 (15-23)	28 (17-30)
2012	Cape Verde	14 (10-15)	21 (14-22)	27 (17-29)
2013	Cape Verde	14 (10-14)	21 (14-22)	27 (16-29)
2014	Cape Verde	13 (9-14)	20 (13-21)	25 (15-27)
2015	Cape Verde	13 (9-13)	19 (13-20)	24 (15-26)
2000	Central African Republic	891 (632-929)	1330 (897-1400)	1711 (1041-1833)
2001	Central African Republic	899 (638-937)	1342 (905-1413)	1727 (1050-1849)
2002	Central African Republic	911 (646-950)	1360 (917-1432)	1750 (1064-1874)
2003	Central African Republic	891 (632-929)	1330 (897-1400)	1711 (1041-1833)
2004	Central African Republic	868 (616-905)	1295 (873-1364)	1666 (1014-1785)
2005	Central African Republic	843 (598-879)	1258 (848-1324)	1619 (985-1734)
2006	Central African Republic	879 (623-916)	1311 (884-1381)	1688 (1027-1807)
2007	Central African Republic	919 (652-958)	1370 (924-1443)	1764 (1073-1889)
2008	Central African Republic	950 (674-990)	1418 (956-1493)	1825 (1110-1954)
2009	Central African Republic	1029 (730-1072)	1535 (1035-1616)	1975 (1202-2116)
2010	Central African Republic	1081 (767-1127)	1613 (1088-1699)	2076 (1263-2223)
2011	Central African Republic	1091 (773-1137)	1627 (1097-1713)	2094 (1274-2242)
2012	Central African Republic	916 (650-955)	1367 (922-1439)	1759 (1070-1884)
2013	Central African Republic	995 (706-1037)	1485 (1001-1564)	1911 (1162-2046)
2014	Central African Republic	893 (633-930)	1332 (898-1402)	1714 (1043-1836)
2015	Central African Republic	856 (607-892)	1276 (861-1344)	1643 (999-1759)
2000	Chad	4851 (3440-5055)	7235 (4879-7620)	9312 (5665-9974)
2001	Chad	4956 (3515-5165)	7393 (4986-7786)	9515 (5789-10191)
2002	Chad	5366 (3805-5592)	8004 (5398-8429)	10301 (6267-11033)
2003	Chad	5402 (3831-5630)	8058 (5434-8486)	10371 (6309-11107)
2004	Chad	5598 (3970-5834)	8350 (5631-8795)	10747 (6538-11511)
2005	Chad	5788 (4104-6032)	8634 (5822-9093)	11112 (6760-11901)
2006	Chad	6167 (4373-6427)	9199 (6204-9688)	11840 (7203-12681)
2007	Chad	6115 (4336-6373)	9121 (6151-9606)	11739 (7142-12573)
2008	Chad	6402 (4539-6671)	9549 (6439-10056)	12289 (7476-13162)
2009	Chad	6268 (4444-6532)	9349 (6305-9846)	12033 (7320-12887)
2010	Chad	6331 (4490-6598)	9444 (6369-9946)	12155 (7394-13018)
2011	Chad	6376 (4521-6644)	9510 (6413-10016)	12240 (7446-13109)
2012	Chad	6389 (4530-6658)	9530 (6427-10037)	12265 (7462-13136)
2013	Chad	6426 (4557-6697)	9585 (6464-10094)	12336 (7505-13212)
2014	Chad	6391 (4532-6661)	9533 (6429-10040)	12270 (7464-13141)

Spn = Streptococcus pneumoniae; Uncertainty range provided in parentheses.

Year	Country	Spn pneumonia deaths (UR)	Spn pneumonia deaths (UR)	Spn pneumonia deaths (UR)
2015	Chad	6347 (4500-6614)	9467 (6384-9970)	12184 (7412-13049)
2000	Chile	102 (72-106)	152 (103-160)	196 (119-210)
2001	Chile	113 (80-118)	169 (114-178)	217 (132-233)
2002	Chile	55 (39-57)	81 (55-86)	105 (64-112)
2003	Chile	54 (38-56)	81 (54-85)	104 (63-111)
2004	Chile	52 (37-54)	78 (52-82)	100 (61-107)
2005	Chile	45 (32-47)	67 (46-71)	87 (53-93)
2006	Chile	34 (24-35)	50 (34-53)	64 (39-69)
2007	Chile	41 (29-42)	61 (41-64)	78 (47-84)
2008	Chile	46 (32-47)	68 (46-72)	87 (53-94)
2009	Chile	44 (31-46)	65 (44-69)	84 (51-90)
2010	Chile	33 (24-35)	50 (33-52)	64 (39-68)
2011	Chile	18 (13-18)	27 (19-29)	37 (22-39)
2012	Chile	15 (10-15)	24 (16-25)	33 (20-36)
2013	Chile	16 (11-17)	26 (18-28)	37 (23-40)
2014	Chile	12 (9-13)	21 (14-22)	30 (18-32)
2015	Chile	9 (7-10)	16 (11-17)	25 (15-27)
2000	China	22713 (16106-23670)	33878 (22847-35679)	43602 (26526-46699)
2001	China	19466 (13803-20286)	29035 (19580-30578)	37369 (22733-40023)
2002	China	16759 (11884-17465)	24997 (16858-26327)	32173 (19572-34458)
2003	China	14281 (10126-14883)	21301 (14365-22434)	27415 (16678-29363)
2004	China	12283 (8710-12801)	18321 (12355-19295)	23580 (14345-25255)
2005	China	10736 (7613-11189)	16014 (10799-16865)	20610 (12538-22074)
2006	China	9824 (6966-10238)	14653 (9882-15433)	18859 (11473-20199)
2007	China	9131 (6475-9516)	13620 (9185-14344)	17529 (10664-18774)
2008	China	8604 (6101-8966)	12833 (8655-13516)	16517 (10048-17690)
2009	China	8067 (5720-8407)	12033 (8115-12673)	15487 (9422-16587)
2010	China	7614 (5399-7934)	11356 (7658-11960)	14616 (8892-15654)
2011	China	7019 (4977-7315)	10469 (7060-11026)	13474 (8197-14431)
2012	China	6490 (4602-6763)	9680 (6528-10194)	12458 (7579-13343)
2013	China	6012 (4263-6265)	8967 (6047-9443)	11540 (7021-12360)
2014	China	5545 (3932-5779)	8272 (5578-8711)	10646 (6476-11402)
2015	China	5257 (3728-5479)	7842 (5288-8259)	10093 (6140-10810)
2000	Colombia	906 (643-945)	1352 (912-1424)	1740 (1058-1863)
2001	Colombia	785 (556-818)	1170 (789-1233)	1506 (916-1613)
2002	Colombia	623 (442-649)	929 (627-979)	1196 (727-1281)
2003	Colombia	691 (490-720)	1030 (695-1085)	1326 (807-1420)
2004	Colombia	700 (496-729)	1044 (704-1099)	1343 (817-1439)
2005	Colombia	681 (483-709)	1015 (685-1069)	1307 (795-1400)
2006	Colombia	711 (504-741)	1061 (715-1117)	1365 (830-1462)
2007	Colombia	659 (468-687)	984 (663-1036)	1266 (770-1356)
2008	Colombia	568 (403-592)	848 (572-893)	1091 (664-1169)
2009	Colombia	534 (379-557)	797 (537-839)	1026 (624-1099)
2010	Colombia	456 (323-475)	691 (466-727)	901 (548-965)
2011	Colombia	424 (301-442)	658 (444-693)	877 (534-939)
2012	Colombia	325 (231-339)	527 (355-555)	733 (446-785)
2013	Colombia	278 (197-289)	459 (310-484)	653 (397-700)
2014	Colombia	200 (142-208)	346 (234-365)	519 (316-556)
2015	Colombia	166 (118-173)	293 (198-309)	450 (274-482)
2000	Comoros	89 (63-93)	133 (89-140)	171 (104-183)
2001	Comoros	90 (64-94)	135 (91-142)	174 (106-186)
2002	Comoros	93 (66-97)	138 (93-146)	178 (108-191)
2003	Comoros	94 (67-98)	140 (95-148)	181 (110-194)
2004	Comoros	95 (67-99)	141 (95-149)	182 (111-195)
2005	Comoros	94 (67-98)	140 (95-148)	181 (110-194)
2006	Comoros	93 (66-97)	139 (94-147)	179 (109-192)
2007	Comoros	92 (66-96)	138 (93-145)	177 (108-190)
2008	Comoros	92 (65-96)	137 (92-144)	176 (107-189)
2009	Comoros	90 (64-94)	134 (90-141)	173 (105-185)
2010	Comoros	88 (62-92)	131 (89-138)	169 (103-181)
2011	Comoros	86 (61-89)	128 (86-135)	165 (100-176)
2012	Comoros	83 (59-87)	124 (84-131)	160 (97-171)
2013	Comoros	81 (57-84)	121 (81-127)	155 (95-167)
2014	Comoros	79 (56-83)	118 (80-124)	152 (93-163)
2015	Comoros	77 (55-80)	115 (77-121)	148 (90-158)
2000	Congo	650 (461-677)	969 (654-1021)	1247 (759-1336)
2001	Congo	673 (477-702)	1004 (677-1058)	1293 (786-1385)
2002	Congo	676 (479-704)	1008 (680-1061)	1297 (789-1389)
2003	Congo	663 (470-691)	989 (667-1041)	1272 (774-1363)
2004	Congo	646 (458-673)	963 (650-1015)	1240 (754-1328)
2005	Congo	623 (442-649)	929 (627-979)	1196 (728-1281)
2006	Congo	595 (422-620)	887 (598-934)	1142 (695-1223)
2007	Congo	567 (402-591)	845 (570-890)	1088 (662-1165)
2008	Congo	535 (379-557)	797 (538-840)	1026 (624-1099)
2009	Congo	498 (353-519)	743 (501-783)	956 (582-1024)
2010	Congo	462 (328-482)	690 (465-726)	888 (540-951)
2011	Congo	437 (310-455)	651 (439-686)	838 (510-898)
2012	Congo	403 (285-420)	600 (405-632)	773 (470-828)
2013	Congo	270 (192-282)	403 (272-424)	519 (316-556)
2014	Congo	224 (159-234)	335 (226-352)	431 (262-461)
2015	Congo	203 (144-212)	303 (204-319)	390 (237-418)
2000	Cook Islands	0 (0-0)	0 (0-0)	0 (0-0)
2001	Cook Islands	0 (0-0)	0 (0-0)	0 (0-0)
2002	Cook Islands	0 (0-0)	0 (0-0)	0 (0-0)
2003	Cook Islands	0 (0-0)	0 (0-0)	0 (0-0)
2004	Cook Islands	0 (0-0)	0 (0-0)	0 (0-0)
2005	Cook Islands	0 (0-0)	0 (0-0)	0 (0-0)
2006	Cook Islands	0 (0-0)	0 (0-0)	0 (0-0)
2007	Cook Islands	0 (0-0)	0 (0-0)	0 (0-0)
2008	Cook Islands	0 (0-0)	0 (0-0)	0 (0-0)
2009	Cook Islands	0 (0-0)	0 (0-0)	0 (0-0)
2010	Cook Islands	0 (0-0)	0 (0-0)	0 (0-0)
2011	Cook Islands	0 (0-0)	0 (0-0)	0 (0-0)
2012	Cook Islands	0 (0-0)	0 (0-0)	0 (0-0)
2013	Cook Islands	0 (0-0)	0 (0-0)	0 (0-0)
2014	Cook Islands	0 (0-0)	0 (0-0)	0 (0-0)
2015	Cook Islands	0 (0-0)	0 (0-0)	0 (0-0)
2000	Costa Rica	24 (17-25)	35 (24-37)	45 (28-49)
2001	Costa Rica	19 (14-20)	29 (19-30)	37 (22-40)
2002	Costa Rica	17 (12-18)	25 (17-26)	32 (20-35)
2003	Costa Rica	15 (10-15)	22 (15-23)	28 (17-30)
2004	Costa Rica	14 (10-14)	21 (14-22)	26 (16-28)
2005	Costa Rica	17 (12-18)	26 (17-27)	33 (20-35)
2006	Costa Rica	10 (7-10)	14 (10-15)	18 (11-20)
2007	Costa Rica	13 (9-14)	20 (13-21)	25 (15-27)
2008	Costa Rica	8 (6-9)	12 (8-13)	16 (10-17)
2009	Costa Rica	8 (5-8)	11 (8-12)	15 (9-16)
2010	Costa Rica	8 (6-9)	13 (9-14)	18 (11-19)
2011	Costa Rica	8 (6-8)	13 (8-13)	17 (10-18)

Spn = Streptococcus pneumoniae; Uncertainty range provided in parentheses.

Year	Country	Spn pneumonia deaths (UR)	Spn pneumonia deaths (UR)	Spn pneumonia deaths (UR)
2012	Costa Rica	5 (3-5)	7 (5-8)	10 (6-11)
2013	Costa Rica	6 (4-6)	10 (7-10)	15 (9-16)
2014	Costa Rica	3 (2-4)	6 (4-6)	9 (6-10)
2015	Costa Rica	3 (2-3)	5 (3-5)	8 (5-8)
2000	Cote d'Ivoire	2369 (1680-2468)	3533 (2383-3721)	4547 (2766-4870)
2001	Cote d'Ivoire	2338 (1658-2436)	3487 (2352-3673)	4488 (2730-4807)
2002	Cote d'Ivoire	2286 (1621-2383)	3410 (2300-3592)	4389 (2670-4701)
2003	Cote d'Ivoire	2237 (1586-2331)	3336 (2250-3514)	4294 (2612-4599)
2004	Cote d'Ivoire	2179 (1545-2271)	3250 (2192-3423)	4183 (2545-4480)
2005	Cote d'Ivoire	2200 (1560-2293)	3281 (2213-3456)	4223 (2569-4523)
2006	Cote d'Ivoire	2260 (1602-2355)	3371 (2273-3550)	4338 (2639-4646)
2007	Cote d'Ivoire	2392 (1696-2493)	3568 (2406-3757)	4592 (2793-4918)
2008	Cote d'Ivoire	2506 (1777-2611)	3738 (2521-3936)	4811 (2927-5152)
2009	Cote d'Ivoire	2734 (1938-2849)	4077 (2750-4294)	5248 (3193-5620)
2010	Cote d'Ivoire	2940 (2085-3064)	4386 (2958-4619)	5645 (3434-6046)
2011	Cote d'Ivoire	3131 (2220-3263)	4670 (3149-4919)	6011 (3657-6438)
2012	Cote d'Ivoire	3241 (2298-3378)	4834 (3260-5091)	6222 (3785-6664)
2013	Cote d'Ivoire	3375 (2393-3517)	5033 (3394-5301)	6478 (3941-6938)
2014	Cote d'Ivoire	3426 (2429-3571)	5110 (3446-5382)	6577 (4001-7044)
2015	Cote d'Ivoire	2525 (1791-2632)	3767 (2540-3967)	4848 (2949-5192)
2000	Croatia	4 (3-4)	5 (4-6)	7 (4-7)
2001	Croatia	2 (2-3)	4 (2-4)	5 (3-5)
2002	Croatia	1 (1-2)	2 (1-2)	3 (2-3)
2003	Croatia	3 (2-3)	4 (3-4)	5 (3-5)
2004	Croatia	1 (0-1)	1 (1-1)	1 (1-1)
2005	Croatia	3 (2-4)	5 (3-5)	7 (4-7)
2006	Croatia	2 (2-2)	3 (2-3)	4 (3-4)
2007	Croatia	3 (2-3)	5 (3-5)	6 (4-6)
2008	Croatia	2 (2-2)	3 (2-3)	4 (2-4)
2009	Croatia	2 (1-2)	3 (2-3)	4 (2-4)
2010	Croatia	2 (1-2)	3 (2-3)	3 (2-4)
2011	Croatia	0 (0-0)	0 (0-0)	0 (0-0)
2012	Croatia	1 (0-1)	1 (1-1)	1 (1-1)
2013	Croatia	3 (2-3)	4 (3-4)	5 (3-6)
2014	Croatia	1 (1-1)	2 (1-2)	2 (1-2)
2015	Croatia	1 (1-1)	1 (1-2)	2 (1-2)
2000	Cuba	26 (19-27)	39 (26-41)	50 (31-54)
2001	Cuba	26 (19-27)	39 (26-41)	50 (31-54)
2002	Cuba	22 (16-23)	33 (22-35)	43 (26-46)
2003	Cuba	20 (14-21)	30 (21-32)	39 (24-42)
2004	Cuba	18 (13-19)	27 (18-28)	35 (21-37)
2005	Cuba	23 (17-24)	35 (23-37)	45 (27-48)
2006	Cuba	25 (17-26)	37 (25-39)	47 (29-51)
2007	Cuba	21 (15-21)	31 (21-32)	40 (24-42)
2008	Cuba	17 (12-18)	25 (17-27)	33 (20-35)
2009	Cuba	29 (20-30)	43 (29-45)	55 (33-59)
2010	Cuba	20 (14-21)	30 (20-32)	39 (24-42)
2011	Cuba	22 (15-23)	32 (22-34)	42 (25-45)
2012	Cuba	24 (17-25)	36 (25-38)	47 (29-50)
2013	Cuba	24 (17-25)	36 (24-38)	46 (28-50)
2014	Cuba	22 (16-23)	33 (22-35)	43 (26-46)
2015	Cuba	22 (16-23)	33 (22-35)	42 (26-45)
2000	Cyprus	1 (1-1)	2 (1-2)	3 (2-3)
2001	Cyprus	1 (1-1)	2 (1-2)	2 (1-3)
2002	Cyprus	1 (1-1)	2 (1-2)	2 (1-2)
2003	Cyprus	1 (1-1)	2 (1-2)	2 (1-2)
2004	Cyprus	1 (1-1)	1 (1-1)	2 (1-2)
2005	Cyprus	1 (1-1)	1 (1-1)	2 (1-2)
2006	Cyprus	1 (1-1)	1 (1-1)	1 (1-2)
2007	Cyprus	1 (0-1)	1 (1-1)	1 (1-1)
2008	Cyprus	1 (0-1)	1 (1-1)	1 (1-1)
2009	Cyprus	1 (0-1)	1 (1-1)	1 (1-1)
2010	Cyprus	0 (0-0)	1 (0-1)	1 (1-1)
2011	Cyprus	0 (0-0)	1 (0-1)	1 (1-1)
2012	Cyprus	0 (0-0)	1 (0-1)	1 (0-1)
2013	Cyprus	0 (0-0)	1 (0-1)	1 (0-1)
2014	Cyprus	0 (0-0)	1 (0-1)	1 (0-1)
2015	Cyprus	0 (0-0)	0 (0-0)	0 (0-1)
2000	Czech Republic	6 (4-6)	9 (6-9)	11 (7-12)
2001	Czech Republic	7 (5-7)	10 (7-11)	13 (8-14)
2002	Czech Republic	8 (6-9)	12 (8-13)	16 (10-17)
2003	Czech Republic	8 (6-8)	12 (8-13)	15 (9-17)
2004	Czech Republic	5 (4-6)	8 (5-8)	10 (6-11)
2005	Czech Republic	7 (5-8)	11 (8-12)	14 (9-15)
2006	Czech Republic	10 (7-11)	15 (10-16)	20 (12-21)
2007	Czech Republic	9 (7-10)	14 (9-15)	18 (11-19)
2008	Czech Republic	8 (6-8)	12 (8-12)	15 (9-16)
2009	Czech Republic	6 (4-6)	9 (6-10)	12 (7-12)
2010	Czech Republic	11 (8-12)	17 (11-17)	21 (13-23)
2011	Czech Republic	7 (5-8)	11 (7-12)	14 (9-15)
2012	Czech Republic	8 (6-8)	12 (8-13)	15 (9-17)
2013	Czech Republic	10 (7-11)	16 (11-16)	20 (12-22)
2014	Czech Republic	8 (5-8)	11 (8-12)	15 (9-16)
2015	Czech Republic	7 (5-8)	11 (8-12)	14 (9-15)
2000	Democratic People's Republic of Korea	1380 (978-1438)	2058 (1388-2167)	2649 (1611-2837)
2001	Democratic People's Republic of Korea	1126 (799-1174)	1680 (1133-1770)	2162 (1316-2316)
2002	Democratic People's Republic of Korea	917 (650-956)	1368 (922-1441)	1760 (1071-1886)
2003	Democratic People's Republic of Korea	762 (540-794)	1136 (766-1197)	1463 (890-1566)
2004	Democratic People's Republic of Korea	659 (467-687)	983 (663-1035)	1265 (770-1355)
2005	Democratic People's Republic of Korea	613 (435-639)	915 (617-963)	1177 (716-1261)
2006	Democratic People's Republic of Korea	598 (424-623)	892 (601-939)	1148 (698-1229)
2007	Democratic People's Republic of Korea	593 (420-618)	884 (596-931)	1138 (692-1219)
2008	Democratic People's Republic of Korea	574 (407-598)	856 (577-901)	1101 (670-1180)
2009	Democratic People's Republic of Korea	557 (395-581)	831 (560-875)	1069 (651-1145)
2010	Democratic People's Republic of Korea	536 (380-559)	800 (539-842)	1029 (626-1102)
2011	Democratic People's Republic of Korea	517 (366-538)	771 (520-812)	992 (603-1062)
2012	Democratic People's Republic of Korea	498 (353-519)	742 (501-782)	955 (581-1023)
2013	Democratic People's Republic of Korea	487 (345-508)	727 (490-765)	935 (569-1001)
2014	Democratic People's Republic of Korea	475 (337-495)	708 (478-746)	912 (555-976)
2015	Democratic People's Republic of Korea	458 (325-477)	683 (460-719)	879 (535-941)
2000	Democratic Republic of the Congo	12202 (8652-12716)	18200 (12274-19167)	23424 (14250-25087)
2001	Democratic Republic of the Congo	11857 (8408-12357)	17686 (11927-18626)	22762 (13848-24379)
2002	Democratic Republic of the Congo	11727 (8316-12222)	17492 (11796-18422)	22513 (13696-24112)
2003	Democratic Republic of the Congo	11524 (8172-12010)	17189 (11592-18103)	22123 (13459-23694)
2004	Democratic Republic of the Congo	11538 (8181-12024)	17209 (11606-18125)	22149 (13475-23722)
2005	Democratic Republic of the Congo	11112 (7879-11580)	16574 (11177-17456)	21332 (12977-22847)
2006	Democratic Republic of the Congo	11369 (8062-11848)	16958 (11436-17859)	21825 (13278-23375)
2007	Democratic Republic of the Congo	11434 (8107-11915)	17054 (11501-17961)	21949 (13353-23508)
2008	Democratic Republic of the Congo	12091 (8574-12601)	18035 (12163-18994)	23212 (14121-24861)

Spn = Streptococcus pneumoniae; Uncertainty range provided in parentheses.

Year	Country	Spn pneumonia deaths (UR)	Spn pneumonia deaths (UR)	Spn pneumonia deaths (UR)
2009	Democratic Republic of the Congo	13436 (9527-14002)	20041 (13515-21106)	25793 (15691-27625)
2010	Democratic Republic of the Congo	14760 (10466-15382)	22015 (14847-23186)	28335 (17238-30347)
2011	Democratic Republic of the Congo	16072 (11396-16749)	23972 (16167-25247)	30854 (18770-33045)
2012	Democratic Republic of the Congo	16377 (11613-17068)	24428 (16474-25727)	31440 (19127-33673)
2013	Democratic Republic of the Congo	15357 (10890-16005)	22907 (15448-24125)	29482 (17936-31576)
2014	Democratic Republic of the Congo	13144 (9320-13698)	19605 (13221-20648)	25233 (15350-27025)
2015	Democratic Republic of the Congo	11423 (8100-11904)	17038 (11490-17944)	21929 (13340-23486)
2000	Denmark	3 (2-3)	5 (3-5)	6 (4-7)
2001	Denmark	3 (2-3)	5 (3-5)	6 (4-7)
2002	Denmark	4 (3-4)	6 (4-6)	7 (4-8)
2003	Denmark	4 (3-4)	6 (4-6)	8 (5-9)
2004	Denmark	2 (1-2)	3 (2-3)	4 (2-4)
2005	Denmark	3 (2-3)	4 (3-5)	6 (4-6)
2006	Denmark	1 (1-1)	2 (1-2)	2 (2-3)
2007	Denmark	0 (0-1)	1 (0-1)	1 (1-1)
2008	Denmark	1 (1-1)	1 (1-1)	2 (1-2)
2009	Denmark	2 (2-2)	4 (2-4)	5 (3-5)
2010	Denmark	1 (1-1)	1 (1-1)	2 (1-2)
2011	Denmark	1 (0-1)	1 (1-1)	2 (1-2)
2012	Denmark	0 (0-1)	1 (1-1)	1 (1-1)
2013	Denmark	0 (0-0)	1 (0-1)	1 (1-1)
2014	Denmark	0 (0-0)	0 (0-0)	1 (0-1)
2015	Denmark	0 (0-0)	0 (0-0)	1 (0-1)
2000	Djibouti	100 (71-104)	149 (100-157)	192 (117-205)
2001	Djibouti	97 (69-101)	145 (98-153)	186 (113-200)
2002	Djibouti	95 (67-99)	141 (95-149)	182 (111-195)
2003	Djibouti	93 (66-97)	138 (93-146)	178 (108-191)
2004	Djibouti	90 (64-94)	135 (91-142)	173 (105-185)
2005	Djibouti	87 (62-91)	130 (88-137)	168 (102-179)
2006	Djibouti	85 (60-88)	126 (85-133)	162 (99-174)
2007	Djibouti	81 (58-85)	121 (82-128)	156 (95-167)
2008	Djibouti	78 (55-81)	116 (78-122)	149 (91-160)
2009	Djibouti	74 (53-77)	110 (74-116)	142 (86-152)
2010	Djibouti	71 (51-74)	106 (72-112)	137 (83-147)
2011	Djibouti	70 (49-73)	104 (70-109)	134 (81-143)
2012	Djibouti	70 (49-73)	104 (70-110)	134 (81-143)
2013	Djibouti	47 (33-49)	70 (47-74)	90 (55-97)
2014	Djibouti	40 (29-42)	60 (41-63)	78 (47-83)
2015	Djibouti	35 (25-36)	52 (35-55)	67 (41-72)
2000	Dominica	0 (0-0)	0 (0-0)	0 (0-0)
2001	Dominica	0 (0-0)	0 (0-0)	0 (0-0)
2002	Dominica	0 (0-0)	0 (0-0)	0 (0-0)
2003	Dominica	0 (0-0)	0 (0-0)	0 (0-0)
2004	Dominica	0 (0-0)	0 (0-0)	0 (0-0)
2005	Dominica	0 (0-0)	0 (0-0)	0 (0-1)
2006	Dominica	0 (0-0)	0 (0-0)	1 (0-1)
2007	Dominica	1 (0-1)	1 (1-1)	1 (1-1)
2008	Dominica	0 (0-0)	1 (0-1)	1 (0-1)
2009	Dominica	0 (0-0)	0 (0-1)	1 (0-1)
2010	Dominica	0 (0-0)	0 (0-0)	0 (0-0)
2011	Dominica	0 (0-0)	0 (0-0)	0 (0-0)
2012	Dominica	0 (0-0)	0 (0-1)	1 (0-1)
2013	Dominica	0 (0-0)	1 (0-1)	1 (0-1)
2014	Dominica	0 (0-0)	1 (0-1)	1 (0-1)
2015	Dominica	0 (0-0)	0 (0-0)	1 (0-1)
2000	Dominican Republic	286 (203-298)	427 (288-450)	550 (334-589)
2001	Dominican Republic	265 (188-277)	396 (267-417)	510 (310-546)
2002	Dominican Republic	252 (178-262)	375 (253-395)	483 (294-517)
2003	Dominican Republic	243 (173-254)	363 (245-382)	467 (284-500)
2004	Dominican Republic	236 (167-246)	352 (237-371)	453 (276-485)
2005	Dominican Republic	225 (159-234)	335 (226-353)	432 (263-462)
2006	Dominican Republic	216 (153-225)	322 (217-339)	414 (252-444)
2007	Dominican Republic	205 (146-214)	306 (206-322)	394 (240-422)
2008	Dominican Republic	199 (141-208)	297 (200-313)	383 (233-410)
2009	Dominican Republic	192 (136-201)	287 (194-302)	369 (225-396)
2010	Dominican Republic	191 (136-199)	285 (192-300)	367 (223-393)
2011	Dominican Republic	190 (135-198)	283 (191-298)	364 (222-390)
2012	Dominican Republic	189 (134-197)	282 (190-297)	363 (221-388)
2013	Dominican Republic	191 (135-199)	285 (192-300)	367 (223-393)
2014	Dominican Republic	167 (119-174)	250 (168-263)	321 (195-344)
2015	Dominican Republic	176 (125-184)	263 (177-277)	338 (206-362)
2000	Ecuador	447 (317-465)	666 (449-701)	857 (522-918)
2001	Ecuador	425 (301-443)	633 (427-667)	815 (496-873)
2002	Ecuador	402 (285-419)	599 (404-631)	771 (469-826)
2003	Ecuador	399 (283-416)	595 (401-626)	765 (466-820)
2004	Ecuador	417 (296-435)	622 (419-655)	800 (487-857)
2005	Ecuador	405 (287-422)	605 (408-637)	778 (473-833)
2006	Ecuador	396 (281-412)	590 (398-622)	760 (462-813)
2007	Ecuador	387 (275-404)	578 (390-609)	744 (452-797)
2008	Ecuador	391 (277-407)	583 (393-614)	750 (456-804)
2009	Ecuador	382 (271-398)	570 (384-600)	734 (446-786)
2010	Ecuador	361 (256-377)	542 (366-571)	701 (427-751)
2011	Ecuador	299 (212-312)	466 (314-490)	624 (379-668)
2012	Ecuador	222 (157-231)	364 (245-383)	512 (311-548)
2013	Ecuador	193 (137-201)	322 (217-339)	462 (281-495)
2014	Ecuador	137 (97-143)	239 (161-252)	360 (219-385)
2015	Ecuador	113 (80-118)	201 (136-212)	309 (188-331)
2000	Egypt	4163 (2952-4338)	6209 (4187-6539)	7992 (4862-8559)
2001	Egypt	3844 (2726-4007)	5734 (3867-6039)	7380 (4490-7905)
2002	Egypt	3547 (2515-3696)	5290 (3568-5572)	6809 (4142-7292)
2003	Egypt	3269 (2318-3407)	4877 (3289-5136)	6276 (3818-6722)
2004	Egypt	3053 (2165-3182)	4554 (3071-4796)	5861 (3566-6277)
2005	Egypt	2828 (2005-2947)	4218 (2845-4442)	5429 (3303-5814)
2006	Egypt	2646 (1877-2758)	3947 (2662-4157)	5081 (3091-5441)
2007	Egypt	2488 (1764-2593)	3711 (2503-3908)	4776 (2906-5115)
2008	Egypt	2329 (1651-2427)	3473 (2342-3658)	4470 (2719-4788)
2009	Egypt	2221 (1575-2315)	3313 (2235-3490)	4265 (2594-4567)
2010	Egypt	2161 (1533-2253)	3224 (2174-3395)	4149 (2524-4444)
2011	Egypt	2145 (1521-2235)	3199 (2158-3370)	4118 (2505-4410)
2012	Egypt	2174 (1542-2266)	3243 (2187-3416)	4174 (2539-4471)
2013	Egypt	2216 (1571-2310)	3306 (2229-3481)	4254 (2588-4557)
2014	Egypt	2696 (1912-2810)	4021 (2712-4235)	5175 (3148-5543)
2015	Egypt	2715 (1925-2829)	4049 (2731-4264)	5211 (3170-5581)
2000	El Salvador	253 (179-264)	378 (255-398)	486 (296-520)
2001	El Salvador	221 (156-230)	329 (222-347)	424 (258-454)
2002	El Salvador	219 (155-228)	326 (220-343)	420 (255-449)
2003	El Salvador	198 (141-207)	296 (200-312)	381 (232-408)
2004	El Salvador	178 (126-186)	266 (179-280)	342 (208-366)
2005	El Salvador	163 (115-170)	243 (164-256)	312 (190-335)

Spn = Streptococcus pneumoniae; Uncertainty range provided in parentheses.

Year	Country	Spn pneumonia deaths (UR)	Spn pneumonia deaths (UR)	Spn pneumonia deaths (UR)
2006	El Salvador	148 (105-154)	220 (149-232)	284 (173-304)
2007	El Salvador	138 (98-144)	206 (139-216)	265 (161-283)
2008	El Salvador	126 (89-131)	187 (126-197)	241 (147-258)
2009	El Salvador	116 (82-121)	173 (117-182)	222 (135-238)
2010	El Salvador	95 (67-99)	145 (98-153)	191 (116-205)
2011	El Salvador	71 (50-74)	114 (77-120)	156 (95-168)
2012	El Salvador	53 (38-55)	89 (60-93)	127 (77-136)
2013	El Salvador	45 (32-47)	77 (52-81)	112 (68-120)
2014	El Salvador	31 (22-32)	54 (37-57)	83 (51-89)
2015	El Salvador	25 (18-26)	45 (30-47)	70 (43-75)
2000	Equatorial Guinea	143 (101-149)	213 (144-224)	274 (167-293)
2001	Equatorial Guinea	137 (97-143)	204 (138-215)	263 (160-281)
2002	Equatorial Guinea	128 (91-133)	191 (129-201)	245 (149-263)
2003	Equatorial Guinea	117 (83-122)	175 (118-184)	225 (137-241)
2004	Equatorial Guinea	109 (77-114)	163 (110-171)	209 (127-224)
2005	Equatorial Guinea	107 (76-111)	159 (108-168)	205 (125-220)
2006	Equatorial Guinea	113 (80-118)	168 (114-177)	217 (132-232)
2007	Equatorial Guinea	122 (87-127)	182 (123-192)	234 (143-251)
2008	Equatorial Guinea	130 (92-135)	193 (130-203)	249 (151-266)
2009	Equatorial Guinea	133 (94-139)	198 (134-209)	255 (155-273)
2010	Equatorial Guinea	134 (95-139)	199 (134-210)	256 (156-275)
2011	Equatorial Guinea	130 (92-136)	194 (131-205)	250 (152-268)
2012	Equatorial Guinea	125 (88-130)	186 (125-196)	239 (146-256)
2013	Equatorial Guinea	126 (90-132)	188 (127-198)	242 (147-260)
2014	Equatorial Guinea	128 (90-133)	190 (128-200)	245 (149-262)
2015	Equatorial Guinea	125 (88-130)	186 (125-196)	239 (146-256)
2000	Eritrea	682 (484-711)	1017 (686-1071)	1309 (796-1402)
2001	Eritrea	675 (479-704)	1007 (679-1061)	1297 (789-1389)
2002	Eritrea	673 (477-702)	1004 (677-1058)	1292 (786-1384)
2003	Eritrea	673 (477-701)	1004 (677-1057)	1292 (786-1383)
2004	Eritrea	672 (476-700)	1002 (676-1056)	1290 (785-1382)
2005	Eritrea	666 (472-694)	994 (670-1047)	1279 (778-1370)
2006	Eritrea	662 (470-690)	988 (666-1041)	1272 (774-1362)
2007	Eritrea	651 (462-679)	972 (655-1023)	1251 (761-1339)
2008	Eritrea	634 (450-661)	946 (638-997)	1218 (741-1304)
2009	Eritrea	614 (436-640)	916 (618-965)	1179 (718-1263)
2010	Eritrea	596 (423-621)	889 (600-937)	1145 (696-1226)
2011	Eritrea	577 (409-602)	861 (581-907)	1108 (674-1187)
2012	Eritrea	556 (394-580)	830 (560-874)	1068 (650-1144)
2013	Eritrea	535 (379-557)	797 (538-840)	1026 (624-1099)
2014	Eritrea	519 (368-541)	774 (522-815)	996 (606-1066)
2015	Eritrea	500 (355-521)	746 (503-786)	961 (584-1029)
2000	Estonia	2 (1-2)	2 (2-3)	3 (2-3)
2001	Estonia	1 (0-1)	1 (1-1)	1 (1-1)
2002	Estonia	2 (1-2)	3 (2-3)	4 (2-4)
2003	Estonia	1 (1-1)	2 (1-2)	2 (1-2)
2004	Estonia	2 (1-2)	2 (2-3)	3 (2-3)
2005	Estonia	2 (1-2)	2 (2-3)	3 (2-3)
2006	Estonia	0 (0-0)	0 (0-0)	0 (0-0)
2007	Estonia	1 (1-1)	1 (1-1)	2 (1-2)
2008	Estonia	0 (0-1)	1 (0-1)	1 (1-1)
2009	Estonia	1 (1-1)	1 (1-2)	2 (1-2)
2010	Estonia	2 (1-2)	3 (2-3)	3 (2-4)
2011	Estonia	1 (1-1)	1 (1-1)	1 (1-2)
2012	Estonia	1 (1-1)	1 (1-1)	2 (1-2)
2013	Estonia	1 (1-1)	1 (1-1)	2 (1-2)
2014	Estonia	1 (1-1)	1 (1-1)	2 (1-2)
2015	Estonia	1 (1-1)	1 (1-1)	1 (1-1)
2000	Ethiopia	24802 (17587-25847)	36994 (24948-38961)	47613 (28966-50995)
2001	Ethiopia	24253 (17198-25275)	36175 (24396-38099)	46559 (28325-49866)
2002	Ethiopia	23217 (16463-24196)	34631 (23354-36472)	44571 (27115-47737)
2003	Ethiopia	21886 (15519-22808)	32645 (22015-34381)	42015 (25560-44999)
2004	Ethiopia	20400 (14465-21260)	30428 (20520-32046)	39162 (23825-41944)
2005	Ethiopia	18908 (13407-19705)	28203 (19019-29702)	36298 (22082-38876)
2006	Ethiopia	18146 (12868-18911)	27067 (18253-28506)	34836 (21193-37311)
2007	Ethiopia	16567 (11748-17266)	24712 (16665-26026)	31805 (19349-34064)
2008	Ethiopia	14618 (10366-15235)	21805 (14705-22964)	28064 (17073-30057)
2009	Ethiopia	14003 (9929-14593)	20887 (14086-21997)	26882 (16354-28791)
2010	Ethiopia	13052 (9255-13602)	19468 (13129-20503)	25056 (15243-26836)
2011	Ethiopia	11671 (8276-12163)	17408 (11740-18333)	22405 (13630-23996)
2012	Ethiopia	9846 (6982-10261)	14687 (9905-15468)	18903 (11500-20245)
2013	Ethiopia	8042 (5702-8381)	11995 (8089-12633)	15438 (9392-16534)
2014	Ethiopia	6856 (4862-7145)	10226 (6896-10770)	13162 (8007-14096)
2015	Ethiopia	4622 (3277-4817)	6894 (4649-7261)	8873 (5398-9503)
2000	Fiji	18 (13-19)	27 (18-28)	35 (21-37)
2001	Fiji	17 (12-18)	26 (17-27)	33 (20-36)
2002	Fiji	17 (12-18)	25 (17-27)	33 (20-35)
2003	Fiji	17 (12-18)	25 (17-27)	32 (20-35)
2004	Fiji	17 (12-18)	26 (17-27)	33 (20-35)
2005	Fiji	18 (13-18)	26 (18-28)	34 (21-36)
2006	Fiji	19 (13-20)	28 (19-30)	36 (22-39)
2007	Fiji	20 (14-21)	30 (20-31)	38 (23-41)
2008	Fiji	21 (15-21)	31 (21-32)	40 (24-42)
2009	Fiji	21 (15-22)	31 (21-33)	40 (24-43)
2010	Fiji	20 (14-21)	30 (21-32)	39 (24-42)
2011	Fiji	20 (14-21)	30 (20-31)	38 (23-41)
2012	Fiji	19 (14-20)	29 (19-30)	37 (22-40)
2013	Fiji	10 (7-11)	17 (12-18)	25 (15-27)
2014	Fiji	9 (6-9)	15 (10-16)	22 (13-23)
2015	Fiji	8 (6-8)	14 (9-15)	21 (13-22)
2000	Finland	2 (1-2)	2 (2-3)	3 (2-3)
2001	Finland	3 (2-3)	5 (3-5)	6 (4-7)
2002	Finland	0 (0-0)	0 (0-0)	0 (0-0)
2003	Finland	5 (3-5)	7 (5-7)	9 (5-9)
2004	Finland	1 (1-1)	2 (1-2)	3 (2-3)
2005	Finland	2 (1-2)	3 (2-3)	3 (2-4)
2006	Finland	0 (0-0)	0 (0-0)	0 (0-0)
2007	Finland	2 (1-2)	3 (2-3)	4 (2-4)
2008	Finland	2 (1-2)	3 (2-3)	3 (2-4)
2009	Finland	2 (1-2)	2 (2-2)	3 (2-3)
2010	Finland	1 (1-1)	2 (1-2)	2 (1-2)
2011	Finland	3 (2-3)	4 (3-4)	5 (3-5)
2012	Finland	1 (0-1)	1 (1-1)	1 (1-1)
2013	Finland	1 (1-1)	1 (1-1)	2 (1-2)
2014	Finland	1 (1-1)	1 (1-1)	2 (1-2)
2015	Finland	1 (0-1)	1 (1-1)	2 (1-2)
2000	France	23 (16-24)	34 (23-35)	43 (26-46)
2001	France	20 (14-21)	30 (21-32)	39 (24-42)
2002	France	20 (14-21)	30 (20-32)	39 (23-41)

Spn = Streptococcus pneumoniae; Uncertainty range provided in parentheses.

Year	Country	Spn pneumonia deaths (UR)	Spn pneumonia deaths (UR)	Spn pneumonia deaths (UR)
2003	France	24 (17-25)	35 (24-37)	45 (28-49)
2004	France	14 (10-14)	21 (14-22)	27 (16-28)
2005	France	25 (18-26)	38 (25-40)	48 (29-52)
2006	France	13 (9-13)	20 (14-21)	27 (16-29)
2007	France	20 (14-21)	32 (22-34)	44 (27-48)
2008	France	12 (8-12)	19 (13-20)	27 (16-29)
2009	France	14 (10-15)	23 (16-24)	33 (20-35)
2010	France	16 (11-17)	26 (18-28)	37 (22-39)
2011	France	9 (6-9)	16 (11-17)	25 (15-26)
2012	France	7 (5-7)	12 (8-13)	20 (12-21)
2013	France	6 (4-6)	11 (7-11)	18 (11-19)
2014	France	6 (4-6)	10 (7-11)	17 (10-18)
2015	France	6 (4-6)	10 (7-11)	17 (10-18)
2000	Gabon	106 (75-111)	158 (107-167)	204 (124-218)
2001	Gabon	110 (78-115)	164 (111-173)	212 (129-227)
2002	Gabon	114 (81-119)	170 (115-179)	219 (133-234)
2003	Gabon	118 (83-123)	176 (118-185)	226 (137-242)
2004	Gabon	120 (85-125)	179 (121-189)	231 (140-247)
2005	Gabon	122 (86-127)	182 (122-191)	234 (142-250)
2006	Gabon	122 (87-128)	183 (123-192)	235 (143-252)
2007	Gabon	123 (87-128)	184 (124-193)	236 (144-253)
2008	Gabon	122 (86-127)	182 (123-192)	234 (142-251)
2009	Gabon	121 (86-126)	181 (122-191)	233 (142-249)
2010	Gabon	119 (85-124)	178 (120-187)	229 (139-245)
2011	Gabon	119 (84-124)	177 (119-186)	228 (139-244)
2012	Gabon	116 (83-121)	174 (117-183)	223 (136-239)
2013	Gabon	114 (81-119)	170 (115-179)	219 (133-234)
2014	Gabon	110 (78-115)	164 (111-173)	211 (129-226)
2015	Gabon	107 (76-112)	160 (108-169)	206 (125-221)
2000	Gambia	343 (243-357)	511 (345-538)	658 (400-705)
2001	Gambia	335 (238-349)	500 (337-527)	643 (391-689)
2002	Gambia	343 (243-358)	512 (345-539)	659 (401-706)
2003	Gambia	341 (242-355)	508 (343-535)	654 (398-701)
2004	Gambia	337 (239-352)	503 (339-530)	648 (394-694)
2005	Gambia	336 (238-350)	501 (338-528)	645 (392-691)
2006	Gambia	334 (237-348)	499 (336-525)	642 (390-687)
2007	Gambia	330 (234-344)	492 (332-519)	634 (386-679)
2008	Gambia	327 (232-340)	487 (329-513)	627 (381-672)
2009	Gambia	324 (229-337)	483 (325-508)	621 (378-665)
2010	Gambia	280 (198-292)	417 (281-440)	537 (327-575)
2011	Gambia	228 (162-238)	340 (230-359)	438 (267-469)
2012	Gambia	166 (118-173)	248 (167-261)	319 (194-342)
2013	Gambia	150 (106-156)	224 (151-236)	288 (175-309)
2014	Gambia	111 (79-116)	166 (112-175)	213 (130-228)
2015	Gambia	96 (68-100)	144 (97-151)	185 (113-198)
2000	Georgia	69 (49-72)	104 (70-109)	133 (81-143)
2001	Georgia	59 (42-62)	89 (60-93)	114 (69-122)
2002	Georgia	52 (37-55)	78 (53-82)	101 (61-108)
2003	Georgia	47 (33-49)	70 (47-74)	90 (55-97)
2004	Georgia	43 (30-44)	64 (43-67)	82 (50-88)
2005	Georgia	39 (28-41)	59 (39-62)	75 (46-81)
2006	Georgia	36 (26-38)	54 (36-57)	70 (42-74)
2007	Georgia	33 (23-34)	49 (33-52)	63 (39-68)
2008	Georgia	30 (21-31)	44 (30-47)	57 (35-61)
2009	Georgia	27 (19-28)	40 (27-42)	51 (31-55)
2010	Georgia	26 (18-27)	39 (26-41)	50 (30-54)
2011	Georgia	25 (18-26)	37 (25-39)	48 (29-51)
2012	Georgia	22 (15-23)	32 (22-34)	42 (25-45)
2013	Georgia	19 (13-20)	28 (19-30)	36 (22-39)
2014	Georgia	17 (12-18)	26 (17-27)	33 (20-35)
2015	Georgia	15 (11-15)	22 (15-24)	29 (18-31)
2000	Germany	29 (21-30)	43 (29-46)	56 (34-60)
2001	Germany	43 (31-45)	64 (43-68)	83 (50-89)
2002	Germany	37 (26-38)	55 (37-57)	70 (43-75)
2003	Germany	36 (26-38)	54 (37-57)	70 (43-75)
2004	Germany	34 (24-35)	51 (34-54)	65 (40-70)
2005	Germany	25 (18-26)	37 (25-39)	48 (29-52)
2006	Germany	19 (14-20)	29 (20-31)	38 (23-41)
2007	Germany	19 (13-20)	30 (20-31)	40 (24-43)
2008	Germany	20 (14-21)	32 (22-34)	44 (27-47)
2009	Germany	17 (12-18)	28 (19-30)	40 (24-43)
2010	Germany	5 (4-6)	10 (6-10)	15 (9-16)
2011	Germany	6 (4-6)	11 (7-12)	18 (11-19)
2012	Germany	4 (3-4)	7 (5-8)	12 (7-13)
2013	Germany	4 (3-4)	7 (5-8)	12 (7-13)
2014	Germany	4 (3-4)	8 (5-8)	12 (7-13)
2015	Germany	4 (3-4)	8 (5-8)	12 (8-13)
2000	Ghana	2135 (1514-2225)	3185 (2148-3355)	4100 (2494-4391)
2001	Ghana	2195 (1557-2288)	3274 (2208-3448)	4214 (2564-4513)
2002	Ghana	2283 (1619-2380)	3406 (2297-3587)	4384 (2667-4695)
2003	Ghana	2328 (1651-2426)	3473 (2342-3657)	4469 (2719-4787)
2004	Ghana	2430 (1723-2533)	3625 (2444-3817)	4665 (2838-4997)
2005	Ghana	2512 (1781-2618)	3747 (2527-3946)	4823 (2934-5165)
2006	Ghana	2619 (1857-2729)	3906 (2634-4113)	5027 (3058-5384)
2007	Ghana	2633 (1867-2744)	3927 (2648-4135)	5054 (3074-5413)
2008	Ghana	2542 (1802-2649)	3791 (2557-3993)	4879 (2968-5226)
2009	Ghana	2522 (1789-2629)	3762 (2537-3962)	4842 (2946-5186)
2010	Ghana	2470 (1752-2575)	3685 (2485-3881)	4743 (2885-5079)
2011	Ghana	2490 (1766-2595)	3714 (2505-3911)	4780 (2908-5119)
2012	Ghana	2113 (1498-2202)	3151 (2125-3319)	4056 (2467-4344)
2013	Ghana	1605 (1138-1673)	2394 (1615-2522)	3082 (1875-3301)
2014	Ghana	1360 (964-1417)	2028 (1368-2136)	2611 (1588-2796)
2015	Ghana	1257 (891-1310)	1875 (1264-1974)	2413 (1468-2584)
2000	Greece	8 (6-9)	12 (8-13)	16 (10-17)
2001	Greece	7 (5-8)	11 (7-11)	14 (8-15)
2002	Greece	6 (4-6)	9 (6-9)	11 (7-12)
2003	Greece	8 (6-9)	13 (8-13)	16 (10-17)
2004	Greece	9 (7-10)	14 (9-15)	18 (11-19)
2005	Greece	11 (8-12)	17 (11-18)	21 (13-23)
2006	Greece	13 (9-14)	20 (13-21)	25 (15-27)
2007	Greece	12 (8-12)	18 (12-19)	23 (14-25)
2008	Greece	12 (8-12)	18 (12-19)	24 (14-25)
2009	Greece	7 (5-7)	10 (7-11)	14 (8-15)
2010	Greece	5 (4-6)	8 (6-9)	11 (7-12)
2011	Greece	5 (4-5)	8 (5-8)	11 (6-11)
2012	Greece	8 (6-8)	12 (8-13)	16 (10-17)
2013	Greece	6 (4-6)	9 (6-10)	12 (7-13)
2014	Greece	3 (2-3)	5 (3-5)	7 (5-8)
2015	Greece	2 (1-2)	4 (2-4)	6 (3-6)

Spn = Streptococcus pneumoniae; Uncertainty range provided in parentheses.

Year	Country	Spn pneumonia deaths (UR)	Spn pneumonia deaths (UR)	Spn pneumonia deaths (UR)
2000	Grenada	1 (1-1)	2 (1-2)	2 (1-2)
2001	Grenada	1 (1-1)	1 (1-2)	2 (1-2)
2002	Grenada	1 (1-1)	1 (1-1)	1 (1-1)
2003	Grenada	0 (0-0)	0 (0-0)	1 (0-1)
2004	Grenada	1 (0-1)	1 (1-1)	1 (1-1)
2005	Grenada	1 (1-1)	1 (1-2)	2 (1-2)
2006	Grenada	1 (1-2)	2 (1-2)	3 (2-3)
2007	Grenada	1 (1-1)	2 (1-2)	2 (1-2)
2008	Grenada	1 (0-1)	1 (1-1)	1 (1-1)
2009	Grenada	0 (0-0)	0 (0-0)	0 (0-0)
2010	Grenada	0 (0-0)	0 (0-0)	0 (0-0)
2011	Grenada	0 (0-0)	0 (0-0)	0 (0-0)
2012	Grenada	0 (0-0)	1 (0-1)	1 (0-1)
2013	Grenada	0 (0-0)	0 (0-1)	1 (0-1)
2014	Grenada	0 (0-0)	1 (0-1)	1 (0-1)
2015	Grenada	0 (0-0)	0 (0-1)	1 (0-1)
2000	Guatemala	1242 (881-1295)	1853 (1250-1952)	2385 (1451-2555)
2001	Guatemala	1197 (849-1247)	1785 (1204-1880)	2298 (1398-2461)
2002	Guatemala	1151 (816-1199)	1717 (1158-1808)	2210 (1344-2366)
2003	Guatemala	1102 (782-1149)	1644 (1109-1732)	2116 (1288-2267)
2004	Guatemala	1062 (753-1107)	1585 (1069-1669)	2039 (1241-2184)
2005	Guatemala	1021 (724-1064)	1523 (1027-1604)	1960 (1192-2099)
2006	Guatemala	977 (693-1018)	1457 (983-1535)	1876 (1141-2009)
2007	Guatemala	933 (662-972)	1392 (939-1466)	1791 (1090-1918)
2008	Guatemala	882 (625-919)	1315 (887-1385)	1693 (1030-1813)
2009	Guatemala	849 (602-885)	1266 (854-1334)	1630 (992-1746)
2010	Guatemala	824 (584-859)	1229 (829-1294)	1582 (962-1694)
2011	Guatemala	806 (572-840)	1202 (811-1266)	1547 (941-1657)
2012	Guatemala	785 (557-818)	1171 (790-1234)	1507 (917-1615)
2013	Guatemala	698 (495-727)	1041 (702-1097)	1340 (815-1435)
2014	Guatemala	570 (404-594)	850 (573-896)	1095 (666-1172)
2015	Guatemala	421 (298-439)	628 (423-661)	808 (492-865)
2000	Guinea	2545 (1805-2652)	3796 (2560-3998)	4885 (2972-5232)
2001	Guinea	2538 (1800-2645)	3786 (2553-3988)	4873 (2965-5219)
2002	Guinea	2515 (1783-2621)	3751 (2530-3950)	4828 (2937-5171)
2003	Guinea	2487 (1764-2592)	3710 (2502-3907)	4775 (2905-5114)
2004	Guinea	2480 (1759-2585)	3700 (2495-3896)	4762 (2897-5100)
2005	Guinea	2476 (1756-2580)	3693 (2491-3890)	4753 (2892-5091)
2006	Guinea	2473 (1754-2577)	3689 (2488-3885)	4748 (2888-5085)
2007	Guinea	2465 (1748-2569)	3677 (2480-3873)	4733 (2879-5069)
2008	Guinea	2368 (1679-2468)	3532 (2382-3720)	4546 (2765-4869)
2009	Guinea	2382 (1689-2483)	3554 (2396-3743)	4574 (2782-4898)
2010	Guinea	2328 (1651-2426)	3473 (2342-3657)	4469 (2719-4787)
2011	Guinea	2280 (1617-2376)	3401 (2294-3582)	4378 (2663-4689)
2012	Guinea	2256 (1600-2351)	3366 (2270-3545)	4332 (2635-4639)
2013	Guinea	2264 (1605-2359)	3377 (2277-3556)	4346 (2644-4655)
2014	Guinea	2268 (1608-2364)	3383 (2282-3563)	4354 (2649-4664)
2015	Guinea	2246 (1593-2341)	3351 (2260-3529)	4313 (2624-4619)
2000	Guinea-Bissau	446 (317-465)	666 (449-701)	857 (521-918)
2001	Guinea-Bissau	461 (327-480)	687 (464-724)	885 (538-948)
2002	Guinea-Bissau	471 (334-491)	703 (474-741)	905 (551-969)
2003	Guinea-Bissau	478 (339-498)	713 (481-751)	918 (558-983)
2004	Guinea-Bissau	477 (338-497)	711 (480-749)	916 (557-981)
2005	Guinea-Bissau	469 (332-488)	699 (471-736)	899 (547-963)
2006	Guinea-Bissau	456 (323-475)	680 (458-716)	875 (532-937)
2007	Guinea-Bissau	439 (311-458)	655 (442-690)	843 (513-903)
2008	Guinea-Bissau	420 (298-438)	627 (423-660)	807 (491-864)
2009	Guinea-Bissau	402 (285-419)	600 (404-632)	772 (470-827)
2010	Guinea-Bissau	386 (274-403)	576 (389-607)	741 (451-794)
2011	Guinea-Bissau	371 (263-387)	554 (373-583)	713 (434-763)
2012	Guinea-Bissau	359 (255-374)	535 (361-564)	689 (419-738)
2013	Guinea-Bissau	350 (248-365)	523 (352-550)	673 (409-720)
2014	Guinea-Bissau	343 (243-358)	512 (345-539)	659 (401-706)
2015	Guinea-Bissau	320 (227-333)	477 (322-503)	614 (374-658)
2000	Guyana	21 (15-22)	32 (21-33)	41 (25-44)
2001	Guyana	19 (13-19)	28 (19-29)	36 (22-38)
2002	Guyana	26 (18-27)	38 (26-40)	49 (30-53)
2003	Guyana	18 (13-19)	27 (18-29)	35 (21-38)
2004	Guyana	21 (15-22)	31 (21-33)	40 (24-43)
2005	Guyana	17 (12-18)	25 (17-27)	33 (20-35)
2006	Guyana	17 (12-18)	25 (17-27)	33 (20-35)
2007	Guyana	15 (10-15)	22 (15-23)	28 (17-30)
2008	Guyana	12 (9-13)	18 (12-19)	24 (14-25)
2009	Guyana	13 (9-14)	19 (13-20)	25 (15-27)
2010	Guyana	12 (8-12)	17 (12-18)	22 (14-24)
2011	Guyana	10 (7-11)	16 (11-17)	21 (13-23)
2012	Guyana	8 (5-8)	12 (8-13)	17 (11-19)
2013	Guyana	7 (5-7)	12 (8-13)	17 (11-19)
2014	Guyana	7 (5-7)	11 (8-12)	17 (10-18)
2015	Guyana	5 (4-5)	9 (6-10)	14 (9-15)
2000	Haiti	1883 (1335-1962)	2809 (1894-2958)	3615 (2199-3872)
2001	Haiti	1806 (1280-1882)	2693 (1816-2836)	3466 (2109-3713)
2002	Haiti	1726 (1224-1799)	2575 (1737-2712)	3314 (2016-3550)
2003	Haiti	1648 (1169-1718)	2458 (1658-2589)	3164 (1925-3389)
2004	Haiti	1583 (1122-1650)	2361 (1592-2487)	3039 (1849-3255)
2005	Haiti	1542 (1093-1607)	2300 (1551-2422)	2960 (1801-3170)
2006	Haiti	1514 (1074-1578)	2259 (1523-2379)	2907 (1768-3113)
2007	Haiti	1492 (1058-1555)	2226 (1501-2344)	2865 (1743-3068)
2008	Haiti	1469 (1042-1531)	2191 (1478-2308)	2820 (1716-3021)
2009	Haiti	1442 (1023-1503)	2151 (1451-2266)	2769 (1684-2965)
2010	Haiti	5516 (3911-5748)	8227 (5548-8665)	10589 (6442-11341)
2011	Haiti	1376 (976-1434)	2052 (1384-2161)	2641 (1607-2829)
2012	Haiti	1344 (953-1401)	2005 (1352-2111)	2580 (1570-2763)
2013	Haiti	1297 (920-1351)	1934 (1304-2037)	2490 (1515-2666)
2014	Haiti	1260 (894-1313)	1880 (1268-1980)	2420 (1472-2591)
2015	Haiti	1217 (863-1269)	1816 (1224-1912)	2337 (1422-2503)
2000	Honduras	417 (296-435)	623 (420-656)	801 (487-858)
2001	Honduras	388 (275-404)	579 (390-609)	745 (453-798)
2002	Honduras	350 (248-365)	522 (352-550)	672 (409-720)
2003	Honduras	320 (227-334)	478 (322-503)	615 (374-659)
2004	Honduras	296 (210-308)	441 (297-464)	568 (345-608)
2005	Honduras	280 (198-291)	417 (281-439)	537 (327-575)
2006	Honduras	258 (183-268)	384 (259-405)	494 (301-530)
2007	Honduras	239 (169-249)	356 (240-375)	458 (279-491)
2008	Honduras	223 (158-232)	332 (224-350)	428 (260-458)
2009	Honduras	209 (148-218)	312 (210-328)	401 (244-430)
2010	Honduras	196 (139-204)	292 (197-307)	376 (229-402)
2011	Honduras	137 (97-143)	218 (147-230)	298 (181-319)
2012	Honduras	102 (72-106)	168 (113-177)	239 (145-256)

Spn = Streptococcus pneumoniae; Uncertainty range provided in parentheses.

Year	Country	Spn pneumonia deaths (UR)	Spn pneumonia deaths (UR)	Spn pneumonia deaths (UR)
2013	Honduras	86 (61-90)	145 (98-152)	209 (127-224)
2014	Honduras	78 (55-81)	131 (88-138)	191 (116-204)
2015	Honduras	53 (38-55)	94 (63-99)	144 (88-154)
2000	Hungary	23 (16-24)	35 (23-36)	44 (27-48)
2001	Hungary	16 (11-16)	23 (16-25)	30 (18-32)
2002	Hungary	10 (7-10)	15 (10-15)	19 (11-20)
2003	Hungary	16 (12-17)	24 (16-25)	31 (19-33)
2004	Hungary	13 (9-14)	20 (13-21)	25 (16-27)
2005	Hungary	16 (12-17)	24 (16-26)	31 (19-33)
2006	Hungary	10 (7-11)	16 (11-16)	20 (12-22)
2007	Hungary	15 (11-16)	22 (15-24)	29 (18-31)
2008	Hungary	8 (6-8)	12 (8-13)	16 (9-17)
2009	Hungary	8 (6-9)	12 (8-13)	16 (10-17)
2010	Hungary	9 (6-9)	13 (9-14)	17 (10-18)
2011	Hungary	7 (5-8)	12 (8-13)	17 (10-18)
2012	Hungary	6 (4-6)	9 (6-10)	14 (8-15)
2013	Hungary	4 (3-4)	7 (5-7)	10 (6-11)
2014	Hungary	4 (3-4)	7 (5-8)	11 (7-12)
2015	Hungary	3 (2-3)	5 (3-5)	8 (5-8)
2000	Iceland	0 (0-0)	0 (0-0)	0 (0-0)
2001	Iceland	0 (0-0)	0 (0-0)	0 (0-0)
2002	Iceland	0 (0-0)	0 (0-0)	0 (0-0)
2003	Iceland	0 (0-0)	0 (0-0)	0 (0-0)
2004	Iceland	0 (0-0)	0 (0-0)	0 (0-0)
2005	Iceland	0 (0-0)	0 (0-0)	0 (0-0)
2006	Iceland	0 (0-0)	0 (0-0)	0 (0-0)
2007	Iceland	0 (0-0)	0 (0-0)	0 (0-0)
2008	Iceland	0 (0-0)	0 (0-0)	0 (0-0)
2009	Iceland	0 (0-0)	0 (0-0)	0 (0-0)
2010	Iceland	0 (0-0)	0 (0-0)	0 (0-0)
2011	Iceland	0 (0-0)	0 (0-0)	0 (0-0)
2012	Iceland	0 (0-0)	0 (0-0)	0 (0-0)
2013	Iceland	0 (0-0)	0 (0-0)	0 (0-0)
2014	Iceland	0 (0-0)	0 (0-0)	0 (0-0)
2015	Iceland	0 (0-0)	0 (0-0)	0 (0-0)
2000	India	135807 (96300-141532)	202569 (136608-213340)	260714 (158607-279231)
2001	India	130030 (92204-135511)	193951 (130797-204264)	249623 (151860-267353)
2002	India	124116 (88010-129348)	185131 (124848-194974)	238270 (144953-255194)
2003	India	118216 (83827-123199)	176330 (118914-185706)	226944 (138063-243063)
2004	India	112017 (79431-116739)	167083 (112678-175968)	215043 (130823-230317)
2005	India	105898 (75092-110362)	157956 (106523-166355)	203296 (123676-217735)
2006	India	100185 (71041-104408)	149434 (100776-157380)	192328 (117004-205989)
2007	India	94169 (66775-98139)	140462 (94725-147931)	180780 (109979-193621)
2008	India	88270 (62592-91991)	131663 (88791-138664)	169455 (103089-181491)
2009	India	82173 (58269-85637)	122568 (82658-129086)	157750 (95969-168955)
2010	India	76701 (54388-79934)	114406 (77153-120489)	147245 (89577-157703)
2011	India	71503 (50702-74517)	106653 (71924-112324)	137266 (83507-147016)
2012	India	65782 (46646-68555)	98120 (66170-103337)	126284 (76826-135254)
2013	India	61383 (43527-63971)	91559 (61745-96427)	117839 (71688-126209)
2014	India	56921 (40362-59320)	84902 (57257-89417)	109273 (66477-117034)
2015	India	53296 (37792-55542)	79495 (53610-83722)	102314 (62243-109581)
2000	Indonesia	12608 (8941-13140)	18806 (12683-19806)	24205 (14725-25924)
2001	Indonesia	11924 (8455-12426)	17785 (11994-18731)	22890 (13925-24516)
2002	Indonesia	11400 (8084-11881)	17005 (11468-17909)	21886 (13314-23440)
2003	Indonesia	10833 (7682-11289)	16158 (10897-17017)	20796 (12652-22273)
2004	Indonesia	13236 (9386-13794)	19743 (13314-20793)	25410 (15458-27215)
2005	Indonesia	9752 (6915-10163)	14546 (9809-15319)	18721 (11389-20051)
2006	Indonesia	9263 (6568-9653)	13816 (9317-14551)	17782 (10818-19045)
2007	Indonesia	8736 (6195-9104)	13031 (8788-13724)	16771 (10203-17962)
2008	Indonesia	8213 (5823-8559)	12250 (8261-12901)	15766 (9591-16886)
2009	Indonesia	7836 (5556-8166)	11687 (7882-12309)	15042 (9151-16111)
2010	Indonesia	7564 (5363-7883)	11282 (7608-11882)	14521 (8834-15552)
2011	Indonesia	7434 (5272-7748)	11089 (7478-11679)	14272 (8682-15286)
2012	Indonesia	7402 (5249-7714)	11041 (7446-11628)	14210 (8645-15220)
2013	Indonesia	7472 (5299-7787)	11146 (7516-11738)	14345 (8727-15364)
2014	Indonesia	7392 (5242-7704)	11026 (7436-11612)	14191 (8633-15199)
2015	Indonesia	7221 (5120-7525)	10771 (7264-11343)	13862 (8433-14847)
2000	Iran (Islamic Republic of)	1943 (1378-2025)	2898 (1955-3052)	3730 (2269-3995)
2001	Iran (Islamic Republic of)	1750 (1241-1824)	2610 (1760-2749)	3360 (2044-3598)
2002	Iran (Islamic Republic of)	1584 (1123-1651)	2363 (1594-2489)	3041 (1850-3257)
2003	Iran (Islamic Republic of)	1414 (1003-1474)	2109 (1423-2222)	2715 (1652-2908)
2004	Iran (Islamic Republic of)	1294 (918-1348)	1930 (1302-2033)	2484 (1511-2660)
2005	Iran (Islamic Republic of)	1186 (841-1236)	1769 (1193-1863)	2277 (1385-2438)
2006	Iran (Islamic Republic of)	1122 (795-1169)	1673 (1128-1762)	2154 (1310-2307)
2007	Iran (Islamic Republic of)	1070 (759-1115)	1596 (1077-1681)	2055 (1250-2200)
2008	Iran (Islamic Republic of)	1029 (730-1073)	1535 (1035-1617)	1976 (1202-2116)
2009	Iran (Islamic Republic of)	995 (705-1037)	1484 (1001-1563)	1910 (1162-2045)
2010	Iran (Islamic Republic of)	978 (694-1020)	1459 (984-1537)	1878 (1143-2012)
2011	Iran (Islamic Republic of)	944 (669-984)	1408 (949-1483)	1812 (1102-1941)
2012	Iran (Islamic Republic of)	912 (646-950)	1360 (917-1432)	1750 (1065-1875)
2013	Iran (Islamic Republic of)	885 (628-922)	1320 (890-1390)	1699 (1034-1820)
2014	Iran (Islamic Republic of)	839 (595-875)	1252 (844-1319)	1612 (980-1726)
2015	Iran (Islamic Republic of)	790 (560-824)	1179 (795-1242)	1517 (923-1625)
2000	Iraq	1648 (1169-1717)	2458 (1658-2589)	3164 (1925-3388)
2001	Iraq	1638 (1161-1707)	2443 (1648-2573)	3144 (1913-3368)
2002	Iraq	1622 (1150-1691)	2420 (1632-2548)	3114 (1895-3335)
2003	Iraq	1610 (1142-1678)	2401 (1619-2529)	3091 (1880-3310)
2004	Iraq	1601 (1136-1669)	2389 (1611-2516)	3074 (1870-3293)
2005	Iraq	1604 (1137-1672)	2392 (1613-2520)	3079 (1873-3298)
2006	Iraq	1605 (1138-1672)	2393 (1614-2521)	3080 (1874-3299)
2007	Iraq	1628 (1155-1697)	2429 (1638-2558)	3126 (1902-3348)
2008	Iraq	1662 (1178-1732)	2478 (1671-2610)	3190 (1940-3416)
2009	Iraq	1692 (1200-1763)	2524 (1702-2658)	3248 (1976-3479)
2010	Iraq	1707 (1211-1779)	2546 (1717-2682)	3277 (1994-3510)
2011	Iraq	1697 (1203-1769)	2531 (1707-2666)	3258 (1982-3489)
2012	Iraq	1705 (1209-1776)	2543 (1715-2678)	3272 (1991-3505)
2013	Iraq	1708 (1211-1781)	2548 (1719-2684)	3280 (1995-3513)
2014	Iraq	1672 (1186-1743)	2494 (1682-2627)	3210 (1953-3438)
2015	Iraq	1669 (1183-1739)	2489 (1678-2621)	3203 (1949-3431)
2000	Ireland	5 (3-5)	7 (5-8)	9 (6-10)
2001	Ireland	6 (4-7)	9 (6-10)	12 (7-13)
2002	Ireland	1 (1-1)	2 (1-2)	3 (2-3)
2003	Ireland	5 (4-6)	8 (5-8)	10 (6-11)
2004	Ireland	2 (2-2)	3 (2-4)	4 (3-5)
2005	Ireland	5 (3-5)	7 (5-7)	9 (5-10)
2006	Ireland	4 (3-4)	6 (4-6)	7 (4-8)
2007	Ireland	0 (0-0)	0 (0-0)	0 (0-0)
2008	Ireland	1 (1-1)	2 (1-2)	3 (2-3)
2009	Ireland	1 (1-1)	2 (1-2)	2 (1-2)

Spn = Streptococcus pneumoniae; Uncertainty range provided in parentheses.

Year	Country	Spn pneumonia deaths (UR)	Spn pneumonia deaths (UR)	Spn pneumonia deaths (UR)
2010	Ireland	4 (3-4)	6 (4-6)	7 (4-8)
2011	Ireland	1 (1-1)	2 (1-2)	3 (2-3)
2012	Ireland	1 (1-1)	2 (1-2)	2 (1-2)
2013	Ireland	1 (1-1)	1 (1-1)	2 (1-2)
2014	Ireland	0 (0-1)	1 (1-1)	1 (1-2)
2015	Ireland	0 (0-0)	1 (0-1)	1 (1-1)
2000	Israel	4 (3-4)	6 (4-7)	8 (5-9)
2001	Israel	5 (4-6)	8 (5-9)	10 (6-11)
2002	Israel	7 (5-8)	11 (7-11)	14 (8-15)
2003	Israel	2 (2-2)	3 (2-4)	4 (3-5)
2004	Israel	10 (7-11)	15 (10-16)	20 (12-21)
2005	Israel	9 (6-9)	13 (9-14)	17 (11-19)
2006	Israel	3 (2-3)	4 (3-5)	6 (4-6)
2007	Israel	5 (3-5)	7 (5-7)	9 (5-9)
2008	Israel	8 (6-9)	13 (8-13)	16 (10-17)
2009	Israel	5 (4-6)	8 (5-8)	10 (6-11)
2010	Israel	4 (3-4)	6 (4-6)	7 (4-8)
2011	Israel	3 (2-3)	5 (3-5)	7 (4-7)
2012	Israel	4 (3-4)	7 (5-7)	10 (6-11)
2013	Israel	3 (2-3)	5 (3-5)	7 (4-7)
2014	Israel	3 (2-3)	5 (3-5)	7 (4-8)
2015	Israel	2 (2-2)	4 (3-4)	6 (4-6)
2000	Italy	17 (12-17)	25 (17-26)	32 (20-34)
2001	Italy	18 (13-19)	27 (18-29)	35 (21-38)
2002	Italy	24 (17-25)	36 (24-38)	46 (28-49)
2003	Italy	11 (8-11)	16 (11-17)	21 (13-22)
2004	Italy	13 (10-14)	20 (14-21)	26 (16-28)
2005	Italy	14 (10-14)	21 (14-22)	27 (16-28)
2006	Italy	12 (8-12)	18 (12-19)	23 (14-25)
2007	Italy	10 (7-10)	14 (10-15)	19 (11-20)
2008	Italy	11 (8-11)	17 (11-18)	22 (14-24)
2009	Italy	6 (4-6)	9 (6-10)	13 (8-14)
2010	Italy	7 (5-7)	11 (8-12)	16 (10-17)
2011	Italy	8 (6-9)	14 (9-14)	20 (12-21)
2012	Italy	4 (3-4)	7 (5-7)	11 (7-12)
2013	Italy	3 (2-3)	5 (4-5)	8 (5-9)
2014	Italy	2 (2-3)	5 (3-5)	8 (5-8)
2015	Italy	2 (2-2)	4 (3-4)	7 (4-7)
2000	Jamaica	13 (9-13)	19 (13-20)	25 (15-26)
2001	Jamaica	12 (8-12)	18 (12-19)	23 (14-24)
2002	Jamaica	11 (8-12)	17 (11-18)	22 (13-23)
2003	Jamaica	11 (8-11)	16 (11-17)	21 (13-22)
2004	Jamaica	12 (8-12)	18 (12-19)	23 (14-24)
2005	Jamaica	13 (9-14)	19 (13-20)	25 (15-27)
2006	Jamaica	14 (10-14)	21 (14-22)	26 (16-28)
2007	Jamaica	15 (10-15)	22 (15-23)	28 (17-30)
2008	Jamaica	16 (12-17)	24 (16-26)	31 (19-34)
2009	Jamaica	15 (11-16)	22 (15-24)	29 (18-31)
2010	Jamaica	14 (10-15)	21 (14-22)	27 (17-29)
2011	Jamaica	13 (9-14)	20 (13-21)	26 (16-28)
2012	Jamaica	13 (9-14)	19 (13-21)	25 (15-27)
2013	Jamaica	12 (8-12)	17 (12-18)	22 (13-24)
2014	Jamaica	11 (8-11)	16 (11-17)	21 (13-23)
2015	Jamaica	11 (7-11)	16 (11-17)	20 (12-22)
2000	Japan	113 (80-118)	169 (114-178)	218 (132-233)
2001	Japan	89 (63-93)	133 (90-140)	172 (104-184)
2002	Japan	98 (69-102)	146 (98-153)	187 (114-201)
2003	Japan	99 (70-103)	147 (99-155)	190 (115-203)
2004	Japan	80 (57-84)	120 (81-126)	154 (94-165)
2005	Japan	88 (63-92)	132 (89-139)	170 (103-182)
2006	Japan	76 (54-79)	113 (76-119)	145 (88-155)
2007	Japan	80 (57-83)	119 (80-125)	153 (93-164)
2008	Japan	83 (59-87)	124 (84-131)	160 (97-171)
2009	Japan	63 (44-65)	94 (63-99)	120 (73-129)
2010	Japan	71 (50-74)	106 (71-111)	136 (83-146)
2011	Japan	69 (49-72)	102 (69-108)	132 (80-141)
2012	Japan	65 (46-68)	97 (66-102)	125 (76-134)
2013	Japan	60 (42-62)	89 (60-94)	115 (70-123)
2014	Japan	57 (41-60)	86 (58-90)	110 (67-118)
2015	Japan	46 (32-47)	75 (50-78)	105 (64-112)
2000	Jordan	126 (89-131)	188 (127-198)	242 (147-259)
2001	Jordan	125 (89-131)	187 (126-197)	241 (146-258)
2002	Jordan	127 (90-132)	189 (127-199)	243 (148-260)
2003	Jordan	121 (86-127)	181 (122-191)	233 (142-250)
2004	Jordan	116 (82-121)	173 (117-183)	223 (136-239)
2005	Jordan	114 (81-119)	171 (115-180)	220 (134-235)
2006	Jordan	113 (80-118)	169 (114-178)	217 (132-233)
2007	Jordan	113 (80-118)	168 (113-177)	217 (132-232)
2008	Jordan	112 (80-117)	167 (113-176)	215 (131-231)
2009	Jordan	113 (80-118)	168 (114-177)	217 (132-232)
2010	Jordan	112 (79-116)	166 (112-175)	214 (130-229)
2011	Jordan	112 (79-117)	167 (113-176)	215 (131-230)
2012	Jordan	112 (79-116)	166 (112-175)	214 (130-229)
2013	Jordan	110 (78-115)	164 (111-173)	212 (129-227)
2014	Jordan	108 (76-112)	161 (108-169)	207 (126-222)
2015	Jordan	104 (74-108)	155 (104-163)	199 (121-214)
2000	Kazakhstan	499 (354-520)	744 (502-784)	958 (583-1026)
2001	Kazakhstan	470 (333-490)	701 (473-739)	903 (549-967)
2002	Kazakhstan	461 (327-480)	688 (464-724)	885 (538-948)
2003	Kazakhstan	455 (323-474)	679 (458-715)	873 (531-936)
2004	Kazakhstan	450 (319-469)	671 (452-707)	864 (525-925)
2005	Kazakhstan	449 (318-468)	669 (451-705)	862 (524-923)
2006	Kazakhstan	444 (315-463)	663 (447-698)	853 (519-914)
2007	Kazakhstan	435 (308-453)	649 (437-683)	835 (508-894)
2008	Kazakhstan	424 (300-442)	632 (426-666)	813 (495-871)
2009	Kazakhstan	405 (287-422)	604 (408-637)	778 (473-833)
2010	Kazakhstan	388 (275-404)	578 (390-609)	744 (453-797)
2011	Kazakhstan	363 (257-378)	541 (365-570)	696 (424-746)
2012	Kazakhstan	285 (202-297)	425 (287-448)	547 (333-586)
2013	Kazakhstan	233 (165-243)	347 (234-366)	447 (272-479)
2014	Kazakhstan	200 (142-209)	299 (201-315)	384 (234-412)
2015	Kazakhstan	163 (115-170)	243 (164-256)	312 (190-335)
2000	Kenya	6300 (4467-6566)	9397 (6337-9897)	12095 (7358-12954)
2001	Kenya	6245 (4428-6508)	9315 (6282-9810)	11989 (7294-12840)
2002	Kenya	6180 (4382-6440)	9218 (6216-9708)	11864 (7217-12706)
2003	Kenya	6061 (4298-6316)	9040 (6096-9521)	11635 (7078-12461)
2004	Kenya	5919 (4197-6169)	8829 (5954-9299)	11363 (6913-12170)
2005	Kenya	5595 (3967-5831)	8345 (5628-8789)	10740 (6534-11503)
2006	Kenya	5451 (3866-5681)	8131 (5483-8563)	10465 (6367-11208)

Spn = Streptococcus pneumoniae; Uncertainty range provided in parentheses.

Year	Country	Spn pneumonia deaths (UR)	Spn pneumonia deaths (UR)	Spn pneumonia deaths (UR)
2007	Kenya	5393 (3824-5621)	8045 (5425-8472)	10354 (6299-11089)
2008	Kenya	5176 (3670-5394)	7720 (5206-8131)	9936 (6045-10642)
2009	Kenya	4954 (3513-5163)	7390 (4984-7783)	9511 (5786-10186)
2010	Kenya	4713 (3342-4911)	7029 (4741-7403)	9047 (5504-9690)
2011	Kenya	3168 (2246-3302)	4725 (3187-4977)	6082 (3700-6514)
2012	Kenya	2665 (1890-2777)	3975 (2681-4187)	5116 (3112-5480)
2013	Kenya	2430 (1723-2532)	3624 (2444-3817)	4664 (2837-4995)
2014	Kenya	2192 (1554-2284)	3269 (2205-3443)	4208 (2560-4507)
2015	Kenya	1548 (1098-1613)	2309 (1557-2432)	2972 (1808-3183)
2000	Kiribati	11 (8-11)	16 (11-17)	21 (13-23)
2001	Kiribati	10 (7-11)	15 (10-16)	20 (12-21)
2002	Kiribati	9 (7-10)	14 (10-15)	18 (11-19)
2003	Kiribati	8 (6-9)	13 (9-13)	16 (10-17)
2004	Kiribati	8 (6-8)	12 (8-13)	15 (9-16)
2005	Kiribati	8 (6-9)	12 (8-13)	16 (10-17)
2006	Kiribati	9 (6-9)	13 (9-13)	16 (10-18)
2007	Kiribati	10 (7-10)	15 (10-15)	19 (11-20)
2008	Kiribati	11 (8-12)	17 (11-18)	22 (13-23)
2009	Kiribati	12 (9-13)	19 (13-20)	24 (15-26)
2010	Kiribati	13 (9-14)	19 (13-20)	25 (15-27)
2011	Kiribati	13 (9-14)	20 (13-21)	26 (16-27)
2012	Kiribati	13 (9-13)	19 (13-20)	25 (15-26)
2013	Kiribati	9 (6-9)	13 (9-14)	17 (10-18)
2014	Kiribati	7 (5-7)	11 (7-11)	14 (8-15)
2015	Kiribati	4 (3-4)	6 (4-7)	8 (5-9)
2000	Kuwait	13 (9-14)	19 (13-20)	25 (15-27)
2001	Kuwait	12 (9-13)	18 (12-19)	23 (14-25)
2002	Kuwait	12 (9-13)	18 (12-19)	23 (14-25)
2003	Kuwait	10 (7-11)	16 (10-16)	20 (12-21)
2004	Kuwait	9 (7-10)	14 (9-15)	18 (11-19)
2005	Kuwait	9 (7-10)	14 (9-15)	18 (11-19)
2006	Kuwait	8 (6-8)	12 (8-13)	15 (9-16)
2007	Kuwait	14 (10-15)	21 (14-22)	27 (16-29)
2008	Kuwait	9 (6-9)	14 (9-14)	18 (11-19)
2009	Kuwait	12 (9-13)	19 (13-20)	26 (16-28)
2010	Kuwait	13 (9-13)	20 (14-21)	28 (17-30)
2011	Kuwait	16 (11-17)	27 (18-29)	39 (24-42)
2012	Kuwait	11 (8-12)	20 (13-21)	30 (18-32)
2013	Kuwait	10 (7-11)	18 (12-19)	28 (17-30)
2014	Kuwait	10 (7-10)	17 (11-18)	26 (16-28)
2015	Kuwait	9 (6-10)	16 (11-17)	25 (15-26)
2000	Kyrgyzstan	158 (112-165)	236 (159-248)	304 (185-325)
2001	Kyrgyzstan	117 (83-122)	174 (117-183)	224 (136-240)
2002	Kyrgyzstan	102 (72-106)	152 (103-160)	196 (119-210)
2003	Kyrgyzstan	93 (66-97)	139 (94-146)	179 (109-192)
2004	Kyrgyzstan	86 (61-90)	129 (87-136)	166 (101-178)
2005	Kyrgyzstan	87 (62-91)	130 (87-137)	167 (102-179)
2006	Kyrgyzstan	82 (58-85)	122 (82-128)	157 (96-168)
2007	Kyrgyzstan	169 (119-176)	251 (170-265)	323 (197-346)
2008	Kyrgyzstan	186 (132-194)	278 (188-293)	358 (218-383)
2009	Kyrgyzstan	191 (135-199)	285 (192-300)	366 (223-392)
2010	Kyrgyzstan	189 (134-197)	282 (190-297)	363 (221-388)
2011	Kyrgyzstan	188 (134-196)	281 (190-296)	362 (220-387)
2012	Kyrgyzstan	185 (131-193)	276 (186-291)	355 (216-381)
2013	Kyrgyzstan	181 (129-189)	271 (182-285)	348 (212-373)
2014	Kyrgyzstan	175 (124-182)	261 (176-275)	336 (204-359)
2015	Kyrgyzstan	164 (116-171)	245 (165-258)	315 (191-337)
2000	Lao People's Democratic Republic	1206 (855-1257)	1799 (1213-1895)	2316 (1409-2480)
2001	Lao People's Democratic Republic	1160 (822-1209)	1730 (1167-1822)	2227 (1355-2385)
2002	Lao People's Democratic Republic	1118 (792-1165)	1667 (1124-1756)	2145 (1305-2298)
2003	Lao People's Democratic Republic	1083 (768-1129)	1616 (1090-1702)	2080 (1265-2227)
2004	Lao People's Democratic Republic	1051 (746-1096)	1568 (1058-1652)	2019 (1228-2162)
2005	Lao People's Democratic Republic	1023 (725-1066)	1525 (1029-1606)	1963 (1194-2103)
2006	Lao People's Democratic Republic	1000 (709-1043)	1492 (1006-1572)	1921 (1168-2057)
2007	Lao People's Democratic Republic	976 (692-1017)	1455 (981-1533)	1873 (1139-2006)
2008	Lao People's Democratic Republic	955 (677-996)	1425 (961-1501)	1834 (1116-1964)
2009	Lao People's Democratic Republic	928 (658-967)	1384 (934-1458)	1782 (1084-1908)
2010	Lao People's Democratic Republic	901 (639-939)	1344 (907-1416)	1730 (1052-1853)
2011	Lao People's Democratic Republic	874 (619-910)	1303 (879-1372)	1677 (1020-1796)
2012	Lao People's Democratic Republic	835 (592-871)	1246 (840-1312)	1604 (976-1718)
2013	Lao People's Democratic Republic	790 (560-823)	1178 (795-1241)	1517 (923-1624)
2014	Lao People's Democratic Republic	502 (356-523)	749 (505-789)	964 (587-1033)
2015	Lao People's Democratic Republic	403 (286-420)	601 (406-633)	774 (471-829)
2000	Latvia	3 (2-3)	4 (3-4)	5 (3-5)
2001	Latvia	4 (3-4)	6 (4-6)	8 (5-8)
2002	Latvia	2 (1-2)	3 (2-3)	4 (2-4)
2003	Latvia	2 (2-2)	3 (2-3)	4 (3-4)
2004	Latvia	2 (1-2)	3 (2-3)	3 (2-4)
2005	Latvia	4 (3-4)	6 (4-6)	8 (5-8)
2006	Latvia	2 (1-2)	3 (2-3)	3 (2-4)
2007	Latvia	3 (2-3)	4 (3-5)	6 (3-6)
2008	Latvia	0 (0-0)	0 (0-0)	0 (0-0)
2009	Latvia	1 (1-1)	2 (1-2)	3 (2-3)
2010	Latvia	2 (2-2)	3 (2-3)	4 (3-4)
2011	Latvia	1 (1-1)	1 (1-2)	2 (1-2)
2012	Latvia	1 (1-1)	2 (1-2)	2 (1-3)
2013	Latvia	1 (1-1)	1 (1-1)	2 (1-2)
2014	Latvia	1 (0-1)	1 (1-1)	2 (1-2)
2015	Latvia	0 (0-0)	1 (1-1)	1 (1-1)
2000	Lebanon	32 (23-34)	48 (33-51)	62 (38-67)
2001	Lebanon	29 (20-30)	43 (29-45)	55 (34-59)
2002	Lebanon	27 (19-28)	40 (27-42)	52 (31-55)
2003	Lebanon	27 (19-28)	40 (27-42)	52 (32-55)
2004	Lebanon	25 (18-26)	37 (25-39)	48 (29-52)
2005	Lebanon	22 (16-23)	33 (22-35)	42 (26-45)
2006	Lebanon	19 (13-20)	28 (19-30)	36 (22-39)
2007	Lebanon	16 (11-16)	23 (16-24)	30 (18-32)
2008	Lebanon	13 (9-14)	19 (13-20)	25 (15-27)
2009	Lebanon	11 (8-12)	17 (11-18)	22 (13-23)
2010	Lebanon	11 (8-11)	16 (11-17)	21 (13-22)
2011	Lebanon	11 (8-12)	17 (11-18)	22 (13-23)
2012	Lebanon	12 (9-13)	19 (12-20)	24 (15-26)
2013	Lebanon	14 (10-14)	20 (14-21)	26 (16-28)
2014	Lebanon	15 (11-15)	22 (15-23)	28 (17-30)
2015	Lebanon	15 (11-16)	22 (15-23)	29 (17-31)
2000	Lesotho	309 (219-322)	461 (311-486)	594 (361-636)
2001	Lesotho	317 (225-331)	473 (319-498)	609 (371-652)
2002	Lesotho	321 (228-335)	479 (323-505)	617 (375-661)
2003	Lesotho	322 (228-335)	480 (323-505)	617 (376-661)

Spn = Streptococcus pneumoniae; Uncertainty range provided in parentheses.

Year	Country	Spn pneumonia deaths (UR)	Spn pneumonia deaths (UR)	Spn pneumonia deaths (UR)
2004	Lesotho	320 (227-334)	478 (322-503)	615 (374-659)
2005	Lesotho	320 (227-334)	478 (322-503)	615 (374-659)
2006	Lesotho	322 (228-336)	481 (324-506)	619 (376-662)
2007	Lesotho	323 (229-337)	482 (325-508)	620 (377-664)
2008	Lesotho	326 (231-340)	487 (328-513)	627 (381-671)
2009	Lesotho	325 (231-339)	485 (327-511)	625 (380-669)
2010	Lesotho	325 (230-338)	484 (327-510)	624 (379-668)
2011	Lesotho	326 (231-340)	487 (328-513)	627 (381-671)
2012	Lesotho	331 (235-345)	493 (333-520)	635 (386-680)
2013	Lesotho	334 (237-348)	498 (336-525)	641 (390-687)
2014	Lesotho	334 (237-348)	498 (336-524)	640 (390-686)
2015	Lesotho	296 (210-309)	442 (298-465)	568 (346-609)
2000	Liberia	842 (597-878)	1256 (847-1323)	1616 (983-1731)
2001	Liberia	838 (594-873)	1250 (843-1316)	1609 (979-1723)
2002	Liberia	812 (576-846)	1211 (817-1275)	1558 (948-1669)
2003	Liberia	776 (550-809)	1158 (781-1219)	1490 (906-1596)
2004	Liberia	749 (531-781)	1117 (753-1177)	1438 (875-1540)
2005	Liberia	740 (525-772)	1104 (745-1163)	1421 (865-1522)
2006	Liberia	736 (522-767)	1098 (741-1157)	1413 (860-1514)
2007	Liberia	740 (525-771)	1104 (745-1163)	1421 (864-1522)
2008	Liberia	746 (529-778)	1113 (751-1172)	1432 (871-1534)
2009	Liberia	743 (527-775)	1109 (748-1168)	1427 (868-1529)
2010	Liberia	726 (515-757)	1083 (730-1140)	1394 (848-1493)
2011	Liberia	701 (497-731)	1046 (706-1102)	1347 (819-1442)
2012	Liberia	677 (480-706)	1010 (681-1064)	1300 (791-1392)
2013	Liberia	646 (458-674)	964 (650-1015)	1241 (755-1329)
2014	Liberia	520 (369-542)	776 (523-817)	998 (607-1069)
2015	Liberia	452 (320-471)	674 (454-709)	867 (527-928)
2000	Libya	104 (74-108)	155 (104-163)	199 (121-213)
2001	Libya	99 (70-103)	148 (100-156)	191 (116-204)
2002	Libya	95 (67-99)	142 (96-149)	182 (111-195)
2003	Libya	89 (63-93)	133 (89-140)	171 (104-183)
2004	Libya	83 (59-87)	124 (84-131)	160 (97-171)
2005	Libya	77 (55-81)	115 (78-122)	149 (90-159)
2006	Libya	72 (51-75)	107 (72-112)	137 (84-147)
2007	Libya	72 (51-75)	107 (72-113)	138 (84-148)
2008	Libya	73 (51-76)	108 (73-114)	139 (85-149)
2009	Libya	68 (48-70)	101 (68-106)	130 (79-139)
2010	Libya	64 (45-67)	95 (64-100)	123 (75-131)
2011	Libya	62 (44-64)	92 (62-97)	118 (72-127)
2012	Libya	57 (40-59)	85 (57-89)	109 (66-117)
2013	Libya	52 (37-54)	78 (52-82)	100 (61-107)
2014	Libya	43 (30-44)	65 (44-69)	86 (52-92)
2015	Libya	38 (27-40)	59 (39-62)	78 (47-83)
2000	Lithuania	10 (7-11)	16 (11-16)	20 (12-22)
2001	Lithuania	7 (5-7)	10 (7-11)	13 (8-14)
2002	Lithuania	10 (7-10)	14 (10-15)	19 (11-20)
2003	Lithuania	7 (5-7)	10 (7-11)	13 (8-14)
2004	Lithuania	8 (6-9)	12 (8-13)	16 (10-17)
2005	Lithuania	9 (7-10)	14 (9-15)	18 (11-19)
2006	Lithuania	7 (5-7)	11 (7-11)	14 (8-15)
2007	Lithuania	4 (3-5)	7 (4-7)	9 (5-9)
2008	Lithuania	4 (3-4)	5 (4-6)	7 (4-7)
2009	Lithuania	8 (6-9)	12 (8-13)	16 (10-17)
2010	Lithuania	2 (1-2)	3 (2-3)	4 (2-4)
2011	Lithuania	1 (1-1)	2 (1-2)	2 (1-2)
2012	Lithuania	5 (4-5)	8 (5-8)	10 (6-11)
2013	Lithuania	3 (2-3)	4 (3-4)	5 (3-5)
2014	Lithuania	3 (2-3)	4 (3-4)	5 (3-5)
2015	Lithuania	3 (2-3)	4 (3-4)	5 (3-5)
2000	Luxembourg	0 (0-0)	0 (0-0)	0 (0-0)
2001	Luxembourg	0 (0-0)	0 (0-0)	0 (0-0)
2002	Luxembourg	0 (0-0)	0 (0-0)	0 (0-0)
2003	Luxembourg	0 (0-0)	0 (0-0)	0 (0-0)
2004	Luxembourg	0 (0-0)	0 (0-0)	0 (0-0)
2005	Luxembourg	0 (0-0)	0 (0-0)	0 (0-0)
2006	Luxembourg	0 (0-0)	0 (0-0)	0 (0-0)
2007	Luxembourg	0 (0-0)	0 (0-0)	0 (0-0)
2008	Luxembourg	0 (0-0)	0 (0-0)	0 (0-0)
2009	Luxembourg	0 (0-0)	0 (0-0)	0 (0-0)
2010	Luxembourg	0 (0-0)	0 (0-0)	0 (0-0)
2011	Luxembourg	0 (0-0)	0 (0-0)	0 (0-0)
2012	Luxembourg	0 (0-0)	0 (0-0)	0 (0-0)
2013	Luxembourg	0 (0-0)	0 (0-0)	0 (0-0)
2014	Luxembourg	0 (0-0)	0 (0-0)	0 (0-0)
2015	Luxembourg	0 (0-0)	0 (0-0)	0 (0-0)
2000	Madagascar	4461 (3163-4649)	6654 (4487-7008)	8564 (5210-9172)
2001	Madagascar	4297 (3047-4479)	6410 (4323-6751)	8250 (5019-8836)
2002	Madagascar	4100 (2907-4273)	6116 (4124-6441)	7871 (4789-8431)
2003	Madagascar	3892 (2760-4056)	5805 (3915-6114)	7472 (4545-8002)
2004	Madagascar	3675 (2606-3830)	5482 (3697-5774)	7056 (4292-7557)
2005	Madagascar	3494 (2477-3641)	5211 (3514-5488)	6707 (4080-7184)
2006	Madagascar	3312 (2348-3451)	4940 (3331-5202)	6358 (3868-6809)
2007	Madagascar	3167 (2246-3301)	4725 (3186-4976)	6081 (3699-6513)
2008	Madagascar	3049 (2162-3178)	4548 (3067-4790)	5853 (3561-6269)
2009	Madagascar	2964 (2102-3089)	4421 (2982-4656)	5690 (3462-6094)
2010	Madagascar	2875 (2039-2996)	4288 (2892-4516)	5519 (3358-5911)
2011	Madagascar	2813 (1995-2932)	4196 (2830-4419)	5401 (3286-5784)
2012	Madagascar	2754 (1953-2870)	4108 (2770-4326)	5287 (3216-5663)
2013	Madagascar	1945 (1379-2027)	2901 (1956-3055)	3734 (2271-3999)
2014	Madagascar	1725 (1223-1797)	2573 (1735-2709)	3311 (2014-3546)
2015	Madagascar	1590 (1128-1657)	2372 (1600-2498)	3053 (1857-3270)
2000	Malawi	4149 (2942-4324)	6188 (4173-6517)	7964 (4845-8530)
2001	Malawi	3921 (2780-4086)	5848 (3944-6159)	7527 (4579-8062)
2002	Malawi	3606 (2557-3758)	5378 (3627-5664)	6922 (4211-7414)
2003	Malawi	3291 (2334-3430)	4909 (3310-5170)	6318 (3843-6766)
2004	Malawi	3036 (2153-3164)	4529 (3054-4770)	5829 (3546-6243)
2005	Malawi	2835 (2010-2954)	4229 (2852-4453)	5442 (3311-5829)
2006	Malawi	2759 (1956-2875)	4115 (2775-4334)	5296 (3222-5672)
2007	Malawi	2651 (1880-2763)	3955 (2667-4165)	5090 (3097-5452)
2008	Malawi	2569 (1822-2678)	3833 (2585-4036)	4933 (3001-5283)
2009	Malawi	2552 (1810-2660)	3807 (2567-4009)	4899 (2980-5247)
2010	Malawi	2430 (1723-2532)	3624 (2444-3817)	4664 (2838-4996)
2011	Malawi	2508 (1778-2613)	3740 (2522-3939)	4814 (2929-5156)
2012	Malawi	1556 (1103-1622)	2321 (1565-2444)	2987 (1817-3199)
2013	Malawi	1327 (941-1383)	1980 (1335-2085)	2548 (1550-2729)
2014	Malawi	1177 (835-1227)	1756 (1184-1849)	2260 (1375-2420)
2015	Malawi	1076 (763-1121)	1604 (1082-1690)	2065 (1256-2212)
2000	Malaysia	141 (100-147)	210 (142-221)	270 (165-290)

Spn = Streptococcus pneumoniae; Uncertainty range provided in parentheses.

Year	Country	Spn pneumonia deaths (UR)	Spn pneumonia deaths (UR)	Spn pneumonia deaths (UR)
2001	Malaysia	122 (86-127)	181 (122-191)	233 (142-250)
2002	Malaysia	110 (78-114)	164 (110-172)	211 (128-226)
2003	Malaysia	99 (70-103)	147 (99-155)	189 (115-203)
2004	Malaysia	99 (70-103)	147 (99-155)	189 (115-203)
2005	Malaysia	97 (69-101)	145 (97-152)	186 (113-199)
2006	Malaysia	89 (63-93)	133 (90-140)	172 (104-184)
2007	Malaysia	80 (57-83)	119 (80-126)	154 (93-164)
2008	Malaysia	74 (53-78)	111 (75-117)	143 (87-153)
2009	Malaysia	76 (54-79)	113 (76-119)	145 (88-155)
2010	Malaysia	75 (53-78)	112 (76-118)	144 (88-155)
2011	Malaysia	76 (54-79)	113 (76-119)	145 (88-156)
2012	Malaysia	75 (53-78)	112 (75-118)	144 (88-154)
2013	Malaysia	73 (52-76)	109 (74-115)	141 (86-151)
2014	Malaysia	70 (50-73)	105 (71-111)	135 (82-145)
2015	Malaysia	69 (49-72)	103 (69-108)	132 (81-142)
2000	Maldives	12 (8-12)	18 (12-19)	23 (14-24)
2001	Maldives	9 (7-10)	14 (10-15)	18 (11-19)
2002	Maldives	7 (5-8)	11 (7-12)	14 (9-15)
2003	Maldives	6 (4-6)	9 (6-10)	12 (7-13)
2004	Maldives	5 (4-5)	8 (5-8)	10 (6-10)
2005	Maldives	4 (3-4)	6 (4-7)	8 (5-9)
2006	Maldives	4 (3-4)	6 (4-6)	7 (4-8)
2007	Maldives	3 (2-3)	5 (3-5)	6 (4-7)
2008	Maldives	3 (2-3)	4 (3-4)	5 (3-6)
2009	Maldives	3 (2-3)	4 (3-4)	5 (3-5)
2010	Maldives	2 (2-2)	3 (2-3)	4 (3-5)
2011	Maldives	2 (1-2)	3 (2-3)	4 (2-4)
2012	Maldives	2 (1-2)	3 (2-3)	3 (2-4)
2013	Maldives	2 (1-2)	2 (2-3)	3 (2-3)
2014	Maldives	2 (1-2)	2 (2-3)	3 (2-3)
2015	Maldives	2 (1-2)	2 (2-2)	3 (2-3)
2000	Mali	5390 (3822-5617)	8040 (5422-8467)	10347 (6295-11082)
2001	Mali	5389 (3822-5616)	8039 (5421-8466)	10346 (6294-11081)
2002	Mali	5294 (3754-5517)	7896 (5325-8316)	10163 (6183-10885)
2003	Mali	5040 (3574-5253)	7518 (5070-7918)	9676 (5887-10363)
2004	Mali	4922 (3490-5130)	7342 (4951-7732)	9449 (5748-10120)
2005	Mali	4900 (3474-5106)	7309 (4929-7697)	9406 (5722-10075)
2006	Mali	5019 (3559-5231)	7487 (5049-7885)	9636 (5862-10320)
2007	Mali	5314 (3768-5538)	7927 (5346-8348)	10202 (6207-10927)
2008	Mali	5316 (3770-5540)	7929 (5347-8351)	10206 (6209-10930)
2009	Mali	5468 (3877-5698)	8156 (5500-8589)	10497 (6386-11242)
2010	Mali	5360 (3801-5586)	7995 (5392-8420)	10290 (6260-11021)
2011	Mali	4115 (2918-4289)	6138 (4140-6465)	7900 (4806-8462)
2012	Mali	3274 (2322-3412)	4884 (3294-5144)	6286 (3824-6732)
2013	Mali	2819 (1999-2938)	4205 (2836-4428)	5412 (3292-5796)
2014	Mali	2463 (1746-2566)	3673 (2477-3868)	4727 (2876-5063)
2015	Mali	2597 (1841-2706)	3873 (2612-4079)	4985 (3033-5339)
2000	Malta	0 (0-0)	0 (0-0)	0 (0-0)
2001	Malta	0 (0-0)	0 (0-0)	0 (0-0)
2002	Malta	0 (0-0)	0 (0-0)	0 (0-0)
2003	Malta	0 (0-0)	0 (0-0)	0 (0-0)
2004	Malta	0 (0-0)	1 (0-1)	1 (0-1)
2005	Malta	0 (0-0)	1 (0-1)	1 (0-1)
2006	Malta	0 (0-0)	1 (0-1)	1 (0-1)
2007	Malta	0 (0-0)	0 (0-0)	0 (0-0)
2008	Malta	0 (0-0)	0 (0-0)	0 (0-0)
2009	Malta	0 (0-0)	0 (0-0)	0 (0-0)
2010	Malta	0 (0-0)	0 (0-0)	0 (0-0)
2011	Malta	0 (0-0)	0 (0-0)	0 (0-0)
2012	Malta	0 (0-0)	0 (0-0)	1 (0-1)
2013	Malta	0 (0-0)	1 (0-1)	1 (0-1)
2014	Malta	0 (0-0)	0 (0-0)	0 (0-0)
2015	Malta	0 (0-0)	0 (0-0)	0 (0-0)
2000	Marshall Islands	5 (3-5)	7 (5-8)	9 (6-10)
2001	Marshall Islands	5 (3-5)	7 (5-7)	9 (6-10)
2002	Marshall Islands	5 (3-5)	7 (5-7)	9 (6-10)
2003	Marshall Islands	4 (3-5)	7 (5-7)	9 (5-9)
2004	Marshall Islands	4 (3-5)	7 (4-7)	9 (5-9)
2005	Marshall Islands	4 (3-4)	6 (4-7)	8 (5-9)
2006	Marshall Islands	4 (3-4)	6 (4-7)	8 (5-9)
2007	Marshall Islands	4 (3-4)	6 (4-6)	8 (5-8)
2008	Marshall Islands	4 (3-4)	6 (4-6)	7 (5-8)
2009	Marshall Islands	3 (2-3)	5 (3-5)	6 (4-6)
2010	Marshall Islands	3 (2-3)	5 (3-5)	6 (4-7)
2011	Marshall Islands	3 (2-3)	4 (3-4)	5 (3-6)
2012	Marshall Islands	2 (2-3)	4 (2-4)	5 (3-5)
2013	Marshall Islands	3 (2-3)	4 (3-4)	5 (3-5)
2014	Marshall Islands	3 (2-3)	4 (3-4)	5 (3-5)
2015	Marshall Islands	1 (1-1)	1 (1-1)	1 (1-1)
2000	Mauritania	631 (447-657)	941 (634-991)	1211 (737-1297)
2001	Mauritania	655 (464-682)	976 (658-1028)	1257 (764-1346)
2002	Mauritania	679 (481-707)	1012 (683-1066)	1303 (793-1396)
2003	Mauritania	698 (495-728)	1041 (702-1097)	1340 (815-1436)
2004	Mauritania	711 (504-741)	1061 (715-1117)	1365 (830-1462)
2005	Mauritania	719 (510-749)	1072 (723-1129)	1380 (839-1478)
2006	Mauritania	723 (513-754)	1079 (727-1136)	1388 (845-1487)
2007	Mauritania	724 (513-755)	1080 (728-1138)	1390 (846-1489)
2008	Mauritania	720 (510-750)	1074 (724-1131)	1382 (841-1480)
2009	Mauritania	712 (505-742)	1063 (717-1119)	1368 (832-1465)
2010	Mauritania	707 (502-737)	1055 (711-1111)	1358 (826-1454)
2011	Mauritania	704 (500-734)	1051 (709-1107)	1352 (823-1448)
2012	Mauritania	693 (491-722)	1033 (697-1088)	1330 (809-1424)
2013	Mauritania	682 (483-710)	1017 (686-1071)	1309 (796-1402)
2014	Mauritania	494 (351-515)	737 (497-777)	949 (577-1016)
2015	Mauritania	427 (303-445)	637 (429-670)	819 (498-877)
2000	Mauritius	4 (3-4)	6 (4-6)	8 (5-8)
2001	Mauritius	6 (4-6)	8 (6-9)	11 (7-12)
2002	Mauritius	4 (3-4)	6 (4-7)	8 (5-9)
2003	Mauritius	7 (5-7)	10 (7-10)	13 (8-14)
2004	Mauritius	7 (5-7)	11 (7-11)	14 (8-15)
2005	Mauritius	5 (3-5)	7 (5-8)	9 (6-10)
2006	Mauritius	6 (5-7)	10 (6-10)	12 (7-13)
2007	Mauritius	8 (6-9)	12 (8-13)	16 (10-17)
2008	Mauritius	8 (5-8)	11 (8-12)	14 (9-15)
2009	Mauritius	6 (4-6)	9 (6-9)	11 (7-12)
2010	Mauritius	6 (4-6)	8 (6-9)	11 (6-11)
2011	Mauritius	6 (5-7)	10 (7-10)	12 (8-13)
2012	Mauritius	7 (5-7)	10 (7-10)	13 (8-14)
2013	Mauritius	8 (6-8)	12 (8-12)	15 (9-16)

Spn = Streptococcus pneumoniae; Uncertainty range provided in parentheses.

Year	Country	Spn pneumonia deaths (UR)	Spn pneumonia deaths (UR)	Spn pneumonia deaths (UR)
2014	Mauritius	6 (5-7)	10 (6-10)	12 (7-13)
2015	Mauritius	6 (5-7)	10 (6-10)	12 (7-13)
2000	Mexico	3758 (2664-3916)	5605 (3780-5903)	7213 (4388-7726)
2001	Mexico	3428 (2431-3572)	5113 (3448-5385)	6581 (4003-7048)
2002	Mexico	3486 (2472-3633)	5200 (3507-5476)	6692 (4071-7168)
2003	Mexico	2933 (2080-3057)	4375 (2951-4608)	5631 (3426-6031)
2004	Mexico	2953 (2094-3077)	4404 (2970-4638)	5668 (3448-6071)
2005	Mexico	2557 (1813-2665)	3814 (2572-4016)	4908 (2986-5257)
2006	Mexico	2428 (1722-2531)	3622 (2443-3815)	4662 (2836-4993)
2007	Mexico	2217 (1572-2310)	3307 (2230-3482)	4256 (2589-4558)
2008	Mexico	2065 (1464-2152)	3094 (2087-3259)	3999 (2433-4283)
2009	Mexico	1859 (1318-1937)	2829 (1908-2980)	3707 (2255-3971)
2010	Mexico	1180 (837-1230)	1916 (1292-2018)	2672 (1626-2862)
2011	Mexico	867 (615-904)	1457 (983-1535)	2108 (1283-2258)
2012	Mexico	652 (462-680)	1114 (751-1173)	1640 (998-1756)
2013	Mexico	651 (461-678)	1101 (742-1159)	1604 (976-1718)
2014	Mexico	611 (433-637)	1041 (702-1096)	1528 (930-1637)
2015	Mexico	583 (413-607)	994 (670-1047)	1463 (890-1567)
2000	Micronesia (Federated States of)	9 (7-10)	14 (9-15)	18 (11-19)
2001	Micronesia (Federated States of)	9 (6-9)	13 (9-14)	17 (10-18)
2002	Micronesia (Federated States of)	8 (6-9)	12 (8-13)	16 (10-17)
2003	Micronesia (Federated States of)	8 (6-8)	12 (8-13)	15 (9-16)
2004	Micronesia (Federated States of)	7 (5-8)	11 (7-12)	14 (9-15)
2005	Micronesia (Federated States of)	7 (5-7)	10 (7-11)	13 (8-14)
2006	Micronesia (Federated States of)	7 (5-7)	10 (7-10)	12 (8-13)
2007	Micronesia (Federated States of)	6 (4-6)	9 (6-10)	12 (7-13)
2008	Micronesia (Federated States of)	6 (4-6)	9 (6-9)	11 (7-12)
2009	Micronesia (Federated States of)	6 (4-6)	8 (6-9)	11 (6-11)
2010	Micronesia (Federated States of)	3 (2-3)	4 (3-5)	6 (4-6)
2011	Micronesia (Federated States of)	3 (2-3)	4 (3-5)	6 (4-6)
2012	Micronesia (Federated States of)	3 (2-3)	5 (3-5)	6 (4-6)
2013	Micronesia (Federated States of)	3 (2-3)	4 (3-5)	6 (3-6)
2014	Micronesia (Federated States of)	3 (2-3)	4 (3-4)	5 (3-5)
2015	Micronesia (Federated States of)	1 (1-1)	2 (1-2)	2 (1-2)
2000	Monaco	0 (0-0)	0 (0-0)	0 (0-0)
2001	Monaco	0 (0-0)	0 (0-0)	0 (0-0)
2002	Monaco	0 (0-0)	0 (0-0)	0 (0-0)
2003	Monaco	0 (0-0)	0 (0-0)	0 (0-0)
2004	Monaco	0 (0-0)	0 (0-0)	0 (0-0)
2005	Monaco	0 (0-0)	0 (0-0)	0 (0-0)
2006	Monaco	0 (0-0)	0 (0-0)	0 (0-0)
2007	Monaco	0 (0-0)	0 (0-0)	0 (0-0)
2008	Monaco	0 (0-0)	0 (0-0)	0 (0-0)
2009	Monaco	0 (0-0)	0 (0-0)	0 (0-0)
2010	Monaco	0 (0-0)	0 (0-0)	0 (0-0)
2011	Monaco	0 (0-0)	0 (0-0)	0 (0-0)
2012	Monaco	0 (0-0)	0 (0-0)	0 (0-0)
2013	Monaco	0 (0-0)	0 (0-0)	0 (0-0)
2014	Monaco	0 (0-0)	0 (0-0)	0 (0-0)
2015	Monaco	0 (0-0)	0 (0-0)	0 (0-0)
2000	Mongolia	179 (127-186)	267 (180-281)	343 (209-367)
2001	Mongolia	160 (113-166)	238 (161-251)	307 (187-328)
2002	Mongolia	142 (101-148)	212 (143-223)	273 (166-292)
2003	Mongolia	129 (92-135)	193 (130-203)	249 (151-266)
2004	Mongolia	118 (84-123)	176 (119-186)	227 (138-243)
2005	Mongolia	110 (78-115)	164 (111-173)	212 (129-227)
2006	Mongolia	104 (74-109)	156 (105-164)	200 (122-214)
2007	Mongolia	101 (72-105)	151 (102-159)	194 (118-208)
2008	Mongolia	99 (70-103)	148 (100-156)	190 (116-204)
2009	Mongolia	98 (70-103)	147 (99-155)	189 (115-202)
2010	Mongolia	96 (68-100)	144 (97-151)	185 (113-198)
2011	Mongolia	94 (67-98)	141 (95-148)	181 (110-194)
2012	Mongolia	92 (65-96)	137 (92-144)	176 (107-189)
2013	Mongolia	88 (63-92)	132 (89-139)	169 (103-181)
2014	Mongolia	85 (60-88)	126 (85-133)	163 (99-174)
2015	Mongolia	81 (57-84)	121 (81-127)	155 (95-166)
2000	Montenegro	0 (0-0)	0 (0-1)	1 (0-1)
2001	Montenegro	0 (0-0)	0 (0-0)	0 (0-0)
2002	Montenegro	0 (0-0)	0 (0-0)	0 (0-0)
2003	Montenegro	0 (0-0)	0 (0-0)	0 (0-0)
2004	Montenegro	0 (0-0)	0 (0-0)	1 (0-1)
2005	Montenegro	1 (0-1)	1 (1-1)	1 (1-1)
2006	Montenegro	1 (1-1)	2 (1-2)	3 (2-3)
2007	Montenegro	1 (1-1)	2 (1-2)	2 (1-2)
2008	Montenegro	1 (0-1)	1 (1-1)	1 (1-1)
2009	Montenegro	0 (0-0)	0 (0-0)	0 (0-0)
2010	Montenegro	0 (0-0)	0 (0-0)	1 (0-1)
2011	Montenegro	0 (0-0)	1 (0-1)	1 (1-1)
2012	Montenegro	0 (0-0)	1 (0-1)	1 (0-1)
2013	Montenegro	0 (0-0)	0 (0-1)	1 (0-1)
2014	Montenegro	0 (0-0)	0 (0-1)	1 (0-1)
2015	Montenegro	0 (0-0)	0 (0-0)	1 (0-1)
2000	Morocco	1477 (1047-1539)	2202 (1485-2320)	2835 (1724-3036)
2001	Morocco	1356 (961-1413)	2022 (1364-2129)	2602 (1583-2787)
2002	Morocco	1249 (886-1302)	1863 (1257-1962)	2398 (1459-2569)
2003	Morocco	1164 (826-1213)	1737 (1171-1829)	2235 (1360-2394)
2004	Morocco	1095 (776-1141)	1633 (1101-1720)	2101 (1278-2251)
2005	Morocco	1035 (734-1078)	1544 (1041-1626)	1987 (1209-2128)
2006	Morocco	981 (696-1022)	1463 (987-1541)	1883 (1146-2017)
2007	Morocco	940 (667-980)	1403 (946-1477)	1805 (1098-1934)
2008	Morocco	902 (640-940)	1345 (907-1417)	1732 (1053-1855)
2009	Morocco	885 (628-922)	1320 (890-1391)	1699 (1034-1820)
2010	Morocco	862 (611-898)	1286 (867-1354)	1655 (1007-1773)
2011	Morocco	805 (571-839)	1200 (810-1264)	1545 (940-1655)
2012	Morocco	609 (432-635)	908 (612-957)	1169 (711-1252)
2013	Morocco	515 (365-537)	769 (518-810)	989 (602-1060)
2014	Morocco	340 (241-354)	507 (342-534)	652 (397-699)
2015	Morocco	268 (190-279)	399 (269-421)	514 (313-550)
2000	Mozambique	5040 (3574-5252)	7517 (5070-7917)	9675 (5886-10362)
2001	Mozambique	4979 (3531-5189)	7427 (5009-7822)	9559 (5815-10238)
2002	Mozambique	4986 (3535-5196)	7436 (5015-7832)	9571 (5823-10251)
2003	Mozambique	5027 (3565-5239)	7498 (5057-7897)	9651 (5871-10336)
2004	Mozambique	5124 (3633-5340)	7643 (5154-8049)	9837 (5984-10536)
2005	Mozambique	5231 (3709-5451)	7802 (5262-8217)	10042 (6109-10755)
2006	Mozambique	5287 (3749-5509)	7885 (5318-8305)	10149 (6174-10870)
2007	Mozambique	5277 (3742-5499)	7871 (5308-8289)	10130 (6163-10849)
2008	Mozambique	5207 (3693-5427)	7767 (5238-8180)	9997 (6082-10707)
2009	Mozambique	5082 (3604-5297)	7581 (5112-7984)	9757 (5936-10450)
2010	Mozambique	4967 (3522-5176)	7408 (4996-7802)	9535 (5800-10212)

Spn = Streptococcus pneumoniae; Uncertainty range provided in parentheses.

Year	Country	Spn pneumonia deaths (UR)	Spn pneumonia deaths (UR)	Spn pneumonia deaths (UR)
2011	Mozambique	4844 (3435-5048)	7225 (4872-7609)	9298 (5657-9959)
2012	Mozambique	4722 (3348-4921)	7043 (4750-7418)	9065 (5515-9709)
2013	Mozambique	3927 (2784-4092)	5857 (3950-6168)	7538 (4586-8074)
2014	Mozambique	3190 (2262-3325)	4759 (3209-5012)	6125 (3726-6560)
2015	Mozambique	2793 (1980-2910)	4165 (2809-4387)	5361 (3261-5742)
2000	Myanmar	4718 (3346-4917)	7038 (4746-7412)	9058 (5510-9701)
2001	Myanmar	4648 (3296-4844)	6933 (4676-7302)	8923 (5429-9557)
2002	Myanmar	4563 (3236-4756)	6807 (4590-7169)	8761 (5330-9383)
2003	Myanmar	4436 (3146-4623)	6617 (4462-6969)	8516 (5181-9121)
2004	Myanmar	4265 (3024-4445)	6362 (4290-6700)	8188 (4981-8769)
2005	Myanmar	4075 (2890-4247)	6078 (4099-6401)	7823 (4759-8379)
2006	Myanmar	3862 (2738-4025)	5760 (3885-6066)	7414 (4510-7940)
2007	Myanmar	3619 (2566-3771)	5398 (3640-5685)	6947 (4226-7440)
2008	Myanmar	6132 (4348-6390)	9146 (6168-9632)	11771 (7161-12607)
2009	Myanmar	3151 (2234-3284)	4700 (3170-4950)	6049 (3680-6479)
2010	Myanmar	2949 (2091-3073)	4399 (2966-4633)	5661 (3444-6064)
2011	Myanmar	2729 (1935-2844)	4070 (2745-4287)	5239 (3187-5611)
2012	Myanmar	2553 (1810-2660)	3808 (2568-4010)	4901 (2981-5249)
2013	Myanmar	2419 (1716-2521)	3609 (2434-3801)	4645 (2826-4974)
2014	Myanmar	2304 (1634-2401)	3437 (2318-3619)	4423 (2691-4737)
2015	Myanmar	2189 (1552-2282)	3265 (2202-3439)	4203 (2557-4501)
2000	Namibia	191 (135-199)	284 (192-299)	366 (223-392)
2001	Namibia	191 (136-199)	285 (192-300)	367 (223-393)
2002	Namibia	191 (135-199)	285 (192-300)	366 (223-392)
2003	Namibia	190 (135-198)	284 (191-299)	365 (222-391)
2004	Namibia	190 (135-198)	283 (191-299)	365 (222-391)
2005	Namibia	191 (135-199)	284 (192-299)	366 (223-392)
2006	Namibia	190 (135-198)	284 (191-299)	365 (222-391)
2007	Namibia	189 (134-197)	282 (190-297)	363 (221-389)
2008	Namibia	190 (134-197)	283 (191-298)	364 (221-390)
2009	Namibia	191 (136-199)	285 (192-300)	367 (223-393)
2010	Namibia	194 (138-202)	289 (195-305)	372 (227-399)
2011	Namibia	197 (140-205)	294 (198-309)	378 (230-405)
2012	Namibia	201 (143-210)	300 (203-316)	387 (235-414)
2013	Namibia	204 (145-213)	304 (205-320)	392 (238-419)
2014	Namibia	206 (146-214)	307 (207-323)	395 (240-423)
2015	Namibia	143 (102-149)	214 (144-225)	275 (167-294)
2000	Nauru	0 (0-0)	0 (0-0)	1 (0-1)
2001	Nauru	0 (0-0)	0 (0-0)	1 (0-1)
2002	Nauru	0 (0-0)	0 (0-0)	1 (0-1)
2003	Nauru	0 (0-0)	0 (0-1)	1 (0-1)
2004	Nauru	0 (0-0)	0 (0-0)	1 (0-1)
2005	Nauru	0 (0-0)	0 (0-1)	1 (0-1)
2006	Nauru	0 (0-0)	0 (0-1)	1 (0-1)
2007	Nauru	0 (0-0)	0 (0-1)	1 (0-1)
2008	Nauru	0 (0-0)	0 (0-1)	1 (0-1)
2009	Nauru	0 (0-0)	0 (0-0)	0 (0-0)
2010	Nauru	0 (0-0)	0 (0-0)	0 (0-0)
2011	Nauru	0 (0-0)	0 (0-0)	0 (0-0)
2012	Nauru	0 (0-0)	0 (0-0)	0 (0-0)
2013	Nauru	0 (0-0)	0 (0-0)	0 (0-0)
2014	Nauru	0 (0-0)	0 (0-0)	0 (0-0)
2015	Nauru	0 (0-0)	0 (0-0)	0 (0-0)
2000	Nepal	3031 (2149-3159)	4521 (3049-4761)	5819 (3540-6232)
2001	Nepal	2835 (2011-2955)	4229 (2852-4454)	5443 (3312-5830)
2002	Nepal	2607 (1849-2717)	3888 (2622-4095)	5004 (3044-5360)
2003	Nepal	2390 (1694-2490)	3564 (2404-3754)	4587 (2791-4913)
2004	Nepal	2171 (1540-2263)	3239 (2184-3411)	4168 (2536-4465)
2005	Nepal	1984 (1407-2068)	2959 (1996-3117)	3809 (2317-4079)
2006	Nepal	1802 (1278-1878)	2688 (1813-2831)	3460 (2105-3706)
2007	Nepal	1653 (1172-1723)	2466 (1663-2597)	3173 (1930-3399)
2008	Nepal	1509 (1070-1572)	2250 (1518-2370)	2896 (1762-3102)
2009	Nepal	1390 (985-1448)	2073 (1398-2183)	2668 (1623-2857)
2010	Nepal	1261 (894-1314)	1881 (1269-1981)	2421 (1473-2593)
2011	Nepal	1153 (818-1202)	1720 (1160-1812)	2214 (1347-2371)
2012	Nepal	1032 (732-1076)	1540 (1038-1621)	1981 (1205-2122)
2013	Nepal	941 (668-981)	1404 (947-1479)	1807 (1099-1936)
2014	Nepal	869 (616-906)	1296 (874-1365)	1668 (1015-1786)
2015	Nepal	691 (490-721)	1031 (696-1086)	1327 (808-1422)
2000	Netherlands	9 (7-10)	14 (9-15)	18 (11-19)
2001	Netherlands	11 (7-11)	16 (11-17)	20 (12-22)
2002	Netherlands	8 (5-8)	11 (8-12)	15 (9-16)
2003	Netherlands	8 (6-8)	12 (8-13)	15 (9-17)
2004	Netherlands	8 (6-9)	12 (8-13)	16 (10-17)
2005	Netherlands	10 (7-10)	14 (10-15)	19 (11-20)
2006	Netherlands	7 (5-7)	10 (7-11)	13 (8-14)
2007	Netherlands	6 (4-6)	8 (6-9)	11 (7-11)
2008	Netherlands	6 (4-6)	8 (6-9)	11 (7-12)
2009	Netherlands	5 (4-5)	8 (5-8)	11 (7-12)
2010	Netherlands	4 (3-4)	6 (4-7)	9 (6-10)
2011	Netherlands	3 (2-3)	5 (3-5)	7 (4-8)
2012	Netherlands	3 (2-3)	6 (4-6)	8 (5-9)
2013	Netherlands	2 (1-2)	3 (2-3)	5 (3-5)
2014	Netherlands	2 (1-2)	3 (2-3)	5 (3-5)
2015	Netherlands	2 (1-2)	3 (2-3)	5 (3-5)
2000	New Zealand	9 (6-9)	13 (9-13)	16 (10-18)
2001	New Zealand	4 (3-4)	5 (4-6)	7 (4-8)
2002	New Zealand	5 (4-6)	8 (5-8)	10 (6-11)
2003	New Zealand	4 (3-5)	7 (4-7)	8 (5-9)
2004	New Zealand	13 (9-14)	20 (13-21)	25 (15-27)
2005	New Zealand	8 (6-9)	12 (8-13)	16 (10-17)
2006	New Zealand	10 (7-10)	14 (10-15)	18 (11-20)
2007	New Zealand	10 (7-11)	15 (10-16)	20 (12-21)
2008	New Zealand	8 (6-9)	12 (8-13)	16 (10-17)
2009	New Zealand	6 (4-6)	9 (6-10)	13 (8-14)
2010	New Zealand	5 (4-5)	8 (5-9)	11 (7-12)
2011	New Zealand	4 (3-4)	6 (4-6)	8 (5-9)
2012	New Zealand	3 (2-4)	6 (4-6)	8 (5-9)
2013	New Zealand	2 (2-2)	4 (3-4)	6 (4-7)
2014	New Zealand	2 (2-2)	4 (3-4)	6 (4-6)
2015	New Zealand	2 (2-2)	4 (3-4)	6 (4-6)
2000	Nicaragua	351 (249-365)	523 (353-551)	673 (410-721)
2001	Nicaragua	329 (233-343)	491 (331-517)	632 (384-677)
2002	Nicaragua	300 (213-312)	447 (302-471)	575 (350-616)
2003	Nicaragua	273 (194-285)	407 (275-429)	524 (319-562)
2004	Nicaragua	260 (185-271)	388 (262-409)	500 (304-535)
2005	Nicaragua	246 (175-257)	367 (248-387)	473 (288-506)
2006	Nicaragua	239 (169-249)	356 (240-375)	458 (279-491)
2007	Nicaragua	233 (165-243)	347 (234-366)	447 (272-479)

Spn = Streptococcus pneumoniae; Uncertainty range provided in parentheses.

Year	Country	Spn pneumonia deaths (UR)	Spn pneumonia deaths (UR)	Spn pneumonia deaths (UR)
2008	Nicaragua	228 (162-238)	340 (229-358)	438 (266-469)
2009	Nicaragua	220 (156-229)	328 (221-345)	422 (256-452)
2010	Nicaragua	210 (149-218)	313 (211-329)	402 (245-431)
2011	Nicaragua	162 (115-169)	253 (171-267)	341 (207-365)
2012	Nicaragua	113 (80-118)	188 (127-198)	268 (163-287)
2013	Nicaragua	91 (64-95)	154 (104-163)	226 (138-242)
2014	Nicaragua	78 (55-81)	134 (91-142)	200 (122-214)
2015	Nicaragua	57 (40-59)	101 (68-107)	158 (96-169)
2000	Niger	8155 (5782-8498)	12163 (8203-12810)	15655 (9524-16767)
2001	Niger	8344 (5917-8696)	12446 (8394-13108)	16019 (9745-17157)
2002	Niger	8355 (5924-8707)	12462 (8404-13124)	16039 (9757-17178)
2003	Niger	8250 (5850-8598)	12305 (8299-12960)	15838 (9635-16962)
2004	Niger	8081 (5730-8422)	12054 (8129-12695)	15514 (9438-16615)
2005	Niger	7884 (5590-8216)	11759 (7930-12384)	15135 (9207-16210)
2006	Niger	7689 (5452-8013)	11469 (7734-12079)	14761 (8980-15809)
2007	Niger	7680 (5446-8004)	11455 (7725-12064)	14743 (8969-15790)
2008	Niger	7083 (5023-7382)	10565 (7125-11127)	13598 (8272-14564)
2009	Niger	6775 (4804-7061)	10106 (6815-10643)	13007 (7913-13931)
2010	Niger	6777 (4806-7063)	10109 (6817-10646)	13010 (7915-13934)
2011	Niger	6643 (4710-6923)	9908 (6682-10435)	12753 (7758-13658)
2012	Niger	6570 (4658-6847)	9799 (6608-10320)	12612 (7673-13508)
2013	Niger	6409 (4545-6679)	9559 (6447-10068)	12303 (7485-13177)
2014	Niger	6141 (4355-6400)	9160 (6178-9647)	11790 (7172-12627)
2015	Niger	4571 (3241-4763)	6817 (4598-7180)	8774 (5338-9398)
2000	Nigeria	42934 (30444-44744)	64040 (43187-67445)	82422 (50142-88276)
2001	Nigeria	43717 (31000-45560)	65208 (43975-68675)	83925 (51056-89886)
2002	Nigeria	44229 (31363-46093)	65972 (44490-69479)	84908 (51654-90939)
2003	Nigeria	43777 (31042-45622)	65297 (44035-68769)	84040 (51126-90009)
2004	Nigeria	44065 (31247-45923)	65727 (44325-69222)	84594 (51463-90602)
2005	Nigeria	44093 (31266-45952)	65769 (44353-69266)	84647 (51496-90659)
2006	Nigeria	45173 (32032-47077)	67380 (45440-70962)	86720 (52757-92880)
2007	Nigeria	45444 (32224-47360)	67784 (45712-71388)	87241 (53074-93437)
2008	Nigeria	45148 (32014-47051)	67342 (45414-70923)	86672 (52727-92828)
2009	Nigeria	45719 (32419-47646)	68194 (45989-71820)	87768 (53394-94002)
2010	Nigeria	46948 (33291-48927)	70027 (47225-73751)	90128 (54830-96530)
2011	Nigeria	47046 (33360-49029)	70174 (47324-73905)	90316 (54944-96731)
2012	Nigeria	46006 (32623-47946)	68623 (46278-72271)	88320 (53730-94593)
2013	Nigeria	46045 (32651-47986)	68681 (46317-72333)	88395 (53776-94674)
2014	Nigeria	45213 (32060-47118)	67439 (45479-71025)	86796 (52803-92961)
2015	Nigeria	42386 (30055-44172)	63222 (42636-66584)	81369 (49501-87148)
2000	Niue	0 (0-0)	0 (0-0)	0 (0-0)
2001	Niue	0 (0-0)	0 (0-0)	0 (0-0)
2002	Niue	0 (0-0)	0 (0-0)	0 (0-0)
2003	Niue	0 (0-0)	0 (0-0)	0 (0-0)
2004	Niue	0 (0-0)	0 (0-0)	0 (0-0)
2005	Niue	0 (0-0)	0 (0-0)	0 (0-0)
2006	Niue	0 (0-0)	0 (0-0)	0 (0-0)
2007	Niue	0 (0-0)	0 (0-0)	0 (0-0)
2008	Niue	0 (0-0)	0 (0-0)	0 (0-0)
2009	Niue	0 (0-0)	0 (0-0)	0 (0-0)
2010	Niue	0 (0-0)	0 (0-0)	0 (0-0)
2011	Niue	0 (0-0)	0 (0-0)	0 (0-0)
2012	Niue	0 (0-0)	0 (0-0)	0 (0-0)
2013	Niue	0 (0-0)	0 (0-0)	0 (0-0)
2014	Niue	0 (0-0)	0 (0-0)	0 (0-0)
2015	Niue	0 (0-0)	0 (0-0)	0 (0-0)
2000	Norway	3 (2-3)	4 (3-4)	5 (3-6)
2001	Norway	2 (1-2)	3 (2-3)	3 (2-4)
2002	Norway	2 (2-2)	3 (2-3)	4 (3-4)
2003	Norway	2 (2-3)	4 (2-4)	5 (3-5)
2004	Norway	1 (0-1)	1 (1-1)	1 (1-1)
2005	Norway	2 (2-3)	4 (2-4)	5 (3-5)
2006	Norway	0 (0-0)	0 (0-0)	0 (0-0)
2007	Norway	5 (3-5)	7 (5-7)	9 (5-9)
2008	Norway	1 (0-1)	1 (1-1)	1 (1-1)
2009	Norway	3 (2-3)	5 (3-5)	6 (4-7)
2010	Norway	1 (1-1)	1 (1-1)	2 (1-2)
2011	Norway	1 (1-1)	1 (1-1)	2 (1-2)
2012	Norway	1 (1-1)	2 (1-2)	2 (1-3)
2013	Norway	0 (0-0)	0 (0-0)	0 (0-0)
2014	Norway	0 (0-0)	1 (0-1)	1 (1-1)
2015	Norway	0 (0-0)	1 (0-1)	1 (1-1)
2000	Oman	33 (24-35)	50 (34-52)	64 (39-68)
2001	Oman	29 (20-30)	43 (29-45)	55 (34-59)
2002	Oman	28 (20-29)	42 (28-44)	54 (33-58)
2003	Oman	26 (18-27)	39 (26-41)	50 (30-54)
2004	Oman	24 (17-25)	36 (24-38)	46 (28-49)
2005	Oman	23 (16-24)	34 (23-36)	44 (27-47)
2006	Oman	22 (16-23)	33 (22-35)	43 (26-46)
2007	Oman	22 (16-23)	34 (23-35)	43 (26-46)
2008	Oman	22 (16-23)	33 (23-35)	43 (26-46)
2009	Oman	19 (14-20)	30 (20-31)	40 (24-42)
2010	Oman	16 (12-17)	26 (18-28)	36 (22-39)
2011	Oman	13 (9-13)	21 (14-22)	30 (19-33)
2012	Oman	12 (8-12)	20 (14-21)	30 (18-32)
2013	Oman	10 (7-11)	18 (12-19)	27 (17-29)
2014	Oman	10 (7-10)	17 (12-18)	26 (16-28)
2015	Oman	10 (7-10)	17 (12-18)	27 (16-28)
2000	Pakistan	23715 (16817-24715)	35374 (23855-37255)	45527 (27697-48761)
2001	Pakistan	23339 (16550-24323)	34812 (23477-36664)	44805 (27257-47987)
2002	Pakistan	22769 (16146-23729)	33962 (22904-35768)	43711 (26592-46815)
2003	Pakistan	22329 (15833-23270)	33305 (22461-35076)	42865 (26077-45910)
2004	Pakistan	22049 (15635-22979)	32889 (22180-34638)	42329 (25751-45336)
2005	Pakistan	22061 (15643-22991)	32906 (22191-34656)	42351 (25765-45359)
2006	Pakistan	22049 (15635-22978)	32888 (22179-34637)	42328 (25751-45335)
2007	Pakistan	22201 (15743-23137)	33115 (22332-34875)	42620 (25928-45647)
2008	Pakistan	22404 (15887-23349)	33418 (22536-35195)	43010 (26165-46065)
2009	Pakistan	22641 (16055-23595)	33771 (22775-35567)	43465 (26442-46552)
2010	Pakistan	22655 (16064-23610)	33792 (22788-35588)	43491 (26458-46580)
2011	Pakistan	22691 (16090-23647)	33845 (22825-35645)	43560 (26500-46654)
2012	Pakistan	22356 (15853-23298)	33346 (22488-35119)	42918 (26109-45966)
2013	Pakistan	15577 (11045-16233)	23234 (15668-24469)	29903 (18192-32027)
2014	Pakistan	13020 (9232-13568)	19420 (13096-20452)	24994 (15205-26769)
2015	Pakistan	12361 (8765-12882)	18437 (12434-19418)	23730 (14436-25415)
2000	Palau	0 (0-0)	0 (0-0)	0 (0-0)
2001	Palau	0 (0-0)	0 (0-0)	0 (0-0)
2002	Palau	0 (0-0)	0 (0-0)	1 (0-1)
2003	Palau	0 (0-0)	0 (0-0)	0 (0-0)
2004	Palau	0 (0-0)	0 (0-0)	1 (0-1)

Spn = Streptococcus pneumoniae; Uncertainty range provided in parentheses.

Year	Country	Spn pneumonia deaths (UR)	Spn pneumonia deaths (UR)	Spn pneumonia deaths (UR)
2005	Palau	0 (0-0)	0 (0-0)	1 (0-1)
2006	Palau	0 (0-0)	0 (0-0)	1 (0-1)
2007	Palau	0 (0-0)	0 (0-0)	0 (0-0)
2008	Palau	0 (0-0)	0 (0-0)	0 (0-0)
2009	Palau	0 (0-0)	0 (0-0)	0 (0-0)
2010	Palau	0 (0-0)	0 (0-0)	0 (0-0)
2011	Palau	0 (0-0)	0 (0-0)	0 (0-0)
2012	Palau	0 (0-0)	0 (0-0)	0 (0-0)
2013	Palau	0 (0-0)	0 (0-0)	0 (0-0)
2014	Palau	0 (0-0)	0 (0-0)	0 (0-0)
2015	Palau	0 (0-0)	0 (0-0)	0 (0-0)
2000	Panama	38 (27-40)	57 (38-60)	73 (44-78)
2001	Panama	43 (31-45)	65 (44-68)	83 (51-89)
2002	Panama	40 (28-41)	59 (40-62)	76 (46-82)
2003	Panama	43 (31-45)	65 (44-68)	83 (51-89)
2004	Panama	45 (32-46)	66 (45-70)	86 (52-92)
2005	Panama	55 (39-58)	83 (56-87)	107 (65-114)
2006	Panama	53 (38-55)	79 (53-84)	102 (62-109)
2007	Panama	63 (45-66)	94 (64-100)	122 (74-130)
2008	Panama	68 (48-71)	101 (68-107)	131 (79-140)
2009	Panama	44 (31-46)	65 (44-69)	84 (51-90)
2010	Panama	73 (52-76)	111 (75-117)	145 (88-155)
2011	Panama	59 (42-62)	92 (62-96)	122 (74-131)
2012	Panama	45 (32-47)	71 (48-75)	97 (59-104)
2013	Panama	43 (31-45)	70 (47-74)	98 (59-105)
2014	Panama	28 (20-29)	48 (32-50)	72 (44-77)
2015	Panama	21 (15-22)	37 (25-39)	58 (35-62)
2000	Papua New Guinea	859 (609-895)	1281 (864-1349)	1648 (1003-1765)
2001	Papua New Guinea	875 (621-912)	1306 (880-1375)	1680 (1022-1800)
2002	Papua New Guinea	884 (627-921)	1318 (889-1388)	1697 (1032-1817)
2003	Papua New Guinea	886 (628-923)	1321 (891-1392)	1701 (1035-1821)
2004	Papua New Guinea	889 (631-927)	1326 (894-1397)	1707 (1039-1828)
2005	Papua New Guinea	891 (632-929)	1329 (896-1400)	1711 (1041-1832)
2006	Papua New Guinea	890 (631-928)	1328 (895-1398)	1709 (1039-1830)
2007	Papua New Guinea	891 (631-928)	1328 (896-1399)	1710 (1040-1831)
2008	Papua New Guinea	883 (626-920)	1317 (888-1388)	1696 (1032-1816)
2009	Papua New Guinea	878 (623-915)	1310 (883-1379)	1686 (1026-1806)
2010	Papua New Guinea	864 (613-900)	1288 (869-1357)	1658 (1009-1776)
2011	Papua New Guinea	849 (602-885)	1267 (854-1334)	1631 (992-1746)
2012	Papua New Guinea	832 (590-867)	1240 (836-1306)	1596 (971-1710)
2013	Papua New Guinea	817 (579-852)	1219 (822-1284)	1569 (954-1680)
2014	Papua New Guinea	794 (563-828)	1184 (799-1247)	1524 (927-1633)
2015	Papua New Guinea	671 (475-699)	1000 (674-1053)	1287 (783-1379)
2000	Paraguay	204 (145-212)	304 (205-320)	391 (238-419)
2001	Paraguay	195 (138-203)	290 (196-306)	373 (227-400)
2002	Paraguay	182 (129-190)	272 (184-287)	350 (213-375)
2003	Paraguay	188 (134-196)	281 (190-296)	362 (220-387)
2004	Paraguay	182 (129-189)	271 (183-285)	349 (212-374)
2005	Paraguay	172 (122-179)	256 (173-270)	330 (201-353)
2006	Paraguay	165 (117-172)	246 (166-259)	316 (192-339)
2007	Paraguay	158 (112-164)	235 (159-248)	303 (184-324)
2008	Paraguay	153 (109-160)	229 (154-241)	294 (179-315)
2009	Paraguay	146 (104-153)	218 (147-230)	281 (171-301)
2010	Paraguay	141 (100-147)	210 (142-221)	270 (165-290)
2011	Paraguay	134 (95-140)	200 (135-210)	257 (156-275)
2012	Paraguay	97 (68-101)	153 (103-162)	209 (127-224)
2013	Paraguay	76 (54-79)	125 (84-131)	176 (107-189)
2014	Paraguay	66 (47-69)	111 (75-116)	158 (96-170)
2015	Paraguay	59 (42-61)	99 (67-104)	144 (88-154)
2000	Peru	1417 (1005-1476)	2113 (1425-2226)	2720 (1655-2913)
2001	Peru	1227 (870-1279)	1830 (1234-1927)	2355 (1433-2523)
2002	Peru	1097 (778-1143)	1637 (1104-1724)	2106 (1281-2256)
2003	Peru	972 (689-1013)	1450 (978-1527)	1866 (1135-1998)
2004	Peru	873 (619-910)	1303 (878-1372)	1677 (1020-1796)
2005	Peru	786 (557-819)	1172 (790-1234)	1508 (917-1615)
2006	Peru	729 (517-760)	1088 (734-1146)	1400 (852-1500)
2007	Peru	670 (475-698)	999 (674-1052)	1286 (782-1377)
2008	Peru	615 (436-641)	918 (619-967)	1181 (719-1265)
2009	Peru	554 (393-578)	829 (559-874)	1070 (651-1146)
2010	Peru	446 (316-465)	690 (465-726)	917 (558-982)
2011	Peru	373 (265-389)	590 (398-621)	801 (487-858)
2012	Peru	286 (203-298)	469 (316-494)	661 (402-708)
2013	Peru	196 (139-205)	339 (229-357)	507 (308-543)
2014	Peru	157 (112-164)	278 (187-292)	424 (258-454)
2015	Peru	140 (99-146)	249 (168-262)	385 (234-412)
2000	Philippines	5040 (3574-5253)	7518 (5070-7917)	9676 (5886-10363)
2001	Philippines	4978 (3530-5188)	7425 (5007-7820)	9556 (5813-10235)
2002	Philippines	4899 (3474-5106)	7308 (4928-7697)	9406 (5722-10074)
2003	Philippines	4838 (3431-5042)	7217 (4867-7600)	9288 (5650-9948)
2004	Philippines	4753 (3371-4954)	7090 (4781-7467)	9125 (5551-9773)
2005	Philippines	4528 (3211-4718)	6753 (4554-7112)	8692 (5288-9309)
2006	Philippines	4482 (3178-4671)	6685 (4508-7040)	8604 (5234-9215)
2007	Philippines	4341 (3078-4524)	6475 (4367-6819)	8333 (5070-8925)
2008	Philippines	4193 (2973-4370)	6254 (4218-6587)	8050 (4897-8621)
2009	Philippines	4043 (2867-4214)	6031 (4067-6352)	7762 (4722-8313)
2010	Philippines	3917 (2778-4082)	5843 (3940-6153)	7520 (4575-8054)
2011	Philippines	3848 (2729-4011)	5740 (3871-6046)	7388 (4495-7913)
2012	Philippines	3771 (2674-3930)	5625 (3793-5924)	7240 (4404-7754)
2013	Philippines	3743 (2654-3901)	5583 (3765-5880)	7186 (4371-7696)
2014	Philippines	3096 (2195-3226)	4618 (3114-4863)	5943 (3616-6365)
2015	Philippines	3182 (2256-3316)	4746 (3200-4998)	6108 (3716-6541)
2000	Poland	47 (33-49)	70 (47-74)	91 (55-97)
2001	Poland	39 (27-40)	58 (39-61)	74 (45-80)
2002	Poland	29 (21-30)	44 (29-46)	56 (34-60)
2003	Poland	34 (24-35)	50 (34-53)	65 (39-69)
2004	Poland	35 (25-37)	53 (36-56)	68 (41-73)
2005	Poland	36 (26-38)	54 (36-57)	69 (42-74)
2006	Poland	39 (27-40)	57 (39-60)	74 (45-79)
2007	Poland	43 (31-45)	65 (44-68)	83 (51-89)
2008	Poland	40 (28-41)	59 (40-62)	76 (46-81)
2009	Poland	45 (32-47)	68 (46-71)	87 (53-93)
2010	Poland	38 (27-40)	57 (39-60)	74 (45-79)
2011	Poland	30 (21-31)	45 (30-47)	58 (35-62)
2012	Poland	30 (21-32)	45 (30-48)	58 (35-62)
2013	Poland	40 (28-41)	59 (40-63)	76 (46-82)
2014	Poland	31 (22-32)	46 (31-48)	59 (36-63)
2015	Poland	31 (22-32)	46 (31-49)	60 (36-64)
2000	Portugal	10 (7-11)	15 (10-16)	19 (12-21)
2001	Portugal	10 (7-11)	15 (10-16)	20 (12-21)

Spn = Streptococcus pneumoniae; Uncertainty range provided in parentheses.

Year	Country	Spn pneumonia deaths (UR)	Spn pneumonia deaths (UR)	Spn pneumonia deaths (UR)
2002	Portugal	11 (7-11)	16 (11-17)	21 (13-22)
2003	Portugal	9 (7-10)	14 (10-15)	19 (12-20)
2004	Portugal	6 (4-7)	10 (7-10)	13 (8-14)
2005	Portugal	5 (4-6)	8 (6-9)	12 (7-12)
2006	Portugal	5 (4-5)	8 (5-8)	10 (6-11)
2007	Portugal	3 (2-3)	5 (3-5)	6 (4-7)
2008	Portugal	4 (3-4)	6 (4-6)	8 (5-8)
2009	Portugal	7 (5-7)	10 (7-11)	13 (8-14)
2010	Portugal	1 (1-1)	2 (1-2)	3 (2-3)
2011	Portugal	3 (2-3)	4 (3-4)	6 (4-6)
2012	Portugal	1 (0-1)	1 (1-1)	2 (1-2)
2013	Portugal	1 (1-1)	2 (1-2)	3 (2-3)
2014	Portugal	1 (1-1)	1 (1-1)	2 (1-2)
2015	Portugal	0 (0-1)	1 (1-1)	1 (1-2)
2000	Qatar	3 (2-3)	5 (3-5)	6 (4-6)
2001	Qatar	3 (2-3)	4 (3-5)	6 (3-6)
2002	Qatar	3 (2-3)	4 (3-4)	5 (3-5)
2003	Qatar	3 (2-3)	4 (3-4)	5 (3-5)
2004	Qatar	3 (2-3)	4 (3-4)	5 (3-5)
2005	Qatar	3 (2-3)	4 (3-4)	5 (3-5)
2006	Qatar	2 (2-3)	4 (3-4)	5 (3-5)
2007	Qatar	2 (2-3)	4 (2-4)	5 (3-5)
2008	Qatar	3 (2-3)	4 (3-4)	5 (3-5)
2009	Qatar	2 (2-2)	3 (2-4)	5 (3-5)
2010	Qatar	2 (2-2)	3 (2-4)	5 (3-5)
2011	Qatar	2 (1-2)	3 (2-3)	4 (3-5)
2012	Qatar	2 (1-2)	3 (2-3)	4 (3-5)
2013	Qatar	2 (1-2)	3 (2-3)	4 (2-4)
2014	Qatar	1 (1-1)	3 (2-3)	4 (2-4)
2015	Qatar	1 (1-2)	3 (2-3)	4 (2-4)
2000	Republic of Korea	56 (40-58)	84 (56-88)	108 (66-115)
2001	Republic of Korea	49 (35-51)	73 (49-77)	94 (57-101)
2002	Republic of Korea	44 (32-46)	66 (45-70)	85 (52-91)
2003	Republic of Korea	36 (26-37)	54 (36-57)	69 (42-74)
2004	Republic of Korea	25 (18-27)	38 (26-40)	49 (30-52)
2005	Republic of Korea	23 (17-24)	35 (24-37)	45 (27-48)
2006	Republic of Korea	27 (19-28)	40 (27-42)	51 (31-55)
2007	Republic of Korea	20 (14-21)	30 (21-32)	39 (24-42)
2008	Republic of Korea	20 (14-21)	30 (20-32)	39 (23-41)
2009	Republic of Korea	21 (15-21)	31 (21-32)	39 (24-42)
2010	Republic of Korea	19 (14-20)	29 (19-30)	37 (22-39)
2011	Republic of Korea	19 (13-20)	28 (19-30)	36 (22-39)
2012	Republic of Korea	13 (9-13)	19 (13-20)	24 (15-26)
2013	Republic of Korea	14 (10-15)	22 (15-23)	28 (17-30)
2014	Republic of Korea	16 (12-17)	25 (17-26)	32 (19-34)
2015	Republic of Korea	10 (7-11)	17 (12-18)	24 (15-26)
2000	Republic of Moldova	60 (43-63)	90 (60-94)	115 (70-124)
2001	Republic of Moldova	49 (35-51)	73 (49-77)	94 (57-101)
2002	Republic of Moldova	36 (25-37)	54 (36-56)	69 (42-74)
2003	Republic of Moldova	33 (23-34)	49 (33-51)	63 (38-67)
2004	Republic of Moldova	24 (17-25)	36 (24-38)	46 (28-50)
2005	Republic of Moldova	22 (16-23)	33 (23-35)	43 (26-46)
2006	Republic of Moldova	24 (17-25)	36 (24-38)	47 (28-50)
2007	Republic of Moldova	18 (13-19)	27 (18-29)	35 (21-37)
2008	Republic of Moldova	19 (13-20)	28 (19-30)	37 (22-39)
2009	Republic of Moldova	20 (14-20)	29 (20-31)	38 (23-40)
2010	Republic of Moldova	20 (14-21)	30 (20-32)	39 (24-42)
2011	Republic of Moldova	20 (14-21)	30 (20-31)	38 (23-41)
2012	Republic of Moldova	18 (13-18)	26 (18-28)	34 (21-36)
2013	Republic of Moldova	19 (13-20)	28 (19-30)	36 (22-39)
2014	Republic of Moldova	17 (12-17)	26 (17-27)	34 (21-36)
2015	Republic of Moldova	12 (9-13)	20 (14-21)	28 (17-30)
2000	Romania	587 (416-611)	875 (590-921)	1126 (685-1206)
2001	Romania	547 (388-570)	816 (550-859)	1050 (639-1124)
2002	Romania	486 (345-506)	725 (489-763)	933 (568-999)
2003	Romania	392 (278-408)	584 (394-615)	752 (457-805)
2004	Romania	383 (271-399)	571 (385-601)	735 (447-787)
2005	Romania	318 (226-332)	475 (320-500)	611 (372-655)
2006	Romania	280 (199-292)	418 (282-440)	538 (327-576)
2007	Romania	263 (187-274)	392 (265-413)	505 (307-541)
2008	Romania	257 (182-268)	383 (258-403)	493 (300-528)
2009	Romania	226 (160-236)	338 (228-356)	434 (264-465)
2010	Romania	222 (158-231)	331 (223-349)	426 (259-457)
2011	Romania	239 (170-249)	357 (241-376)	459 (279-492)
2012	Romania	214 (151-223)	319 (215-336)	410 (249-439)
2013	Romania	201 (143-210)	300 (202-316)	386 (235-413)
2014	Romania	183 (130-191)	273 (184-288)	351 (214-376)
2015	Romania	169 (120-177)	253 (170-266)	325 (198-348)
2000	Russian Federation	1147 (813-1195)	1710 (1153-1801)	2201 (1339-2358)
2001	Russian Federation	1090 (773-1136)	1626 (1097-1713)	2093 (1273-2242)
2002	Russian Federation	997 (707-1039)	1487 (1003-1566)	1914 (1164-2050)
2003	Russian Federation	876 (621-913)	1307 (882-1377)	1682 (1023-1802)
2004	Russian Federation	790 (560-824)	1179 (795-1241)	1517 (923-1625)
2005	Russian Federation	706 (501-736)	1053 (710-1109)	1355 (824-1451)
2006	Russian Federation	632 (448-658)	942 (636-993)	1213 (738-1299)
2007	Russian Federation	562 (399-586)	838 (565-883)	1079 (656-1156)
2008	Russian Federation	509 (361-530)	759 (512-800)	977 (594-1046)
2009	Russian Federation	477 (338-497)	712 (480-749)	916 (557-981)
2010	Russian Federation	452 (320-471)	674 (454-709)	867 (527-928)
2011	Russian Federation	425 (302-443)	634 (428-668)	816 (497-874)
2012	Russian Federation	392 (278-408)	585 (394-616)	752 (458-806)
2013	Russian Federation	395 (280-412)	589 (397-621)	758 (461-812)
2014	Russian Federation	382 (271-399)	570 (385-601)	734 (447-786)
2015	Russian Federation	373 (264-388)	556 (375-585)	715 (435-766)
2000	Rwanda	3935 (2791-4101)	5870 (3959-6182)	7555 (4596-8092)
2001	Rwanda	4037 (2863-4207)	6022 (4061-6342)	7750 (4715-8301)
2002	Rwanda	3567 (2530-3718)	5321 (3589-5604)	6849 (4166-7335)
2003	Rwanda	3222 (2285-3358)	4806 (3241-5062)	6186 (3763-6625)
2004	Rwanda	2763 (1959-2879)	4121 (2779-4340)	5304 (3227-5681)
2005	Rwanda	2407 (1707-2508)	3590 (2421-3781)	4620 (2811-4948)
2006	Rwanda	2121 (1504-2210)	3164 (2134-3332)	4072 (2477-4361)
2007	Rwanda	1895 (1344-1975)	2827 (1907-2977)	3639 (2214-3897)
2008	Rwanda	1709 (1212-1781)	2549 (1719-2684)	3280 (1996-3513)
2009	Rwanda	1548 (1098-1613)	2309 (1557-2432)	2972 (1808-3183)
2010	Rwanda	1204 (854-1255)	1797 (1212-1892)	2312 (1407-2477)
2011	Rwanda	880 (624-917)	1312 (885-1382)	1689 (1027-1809)
2012	Rwanda	590 (418-615)	880 (593-926)	1132 (689-1213)
2013	Rwanda	457 (324-476)	682 (460-718)	878 (534-940)
2014	Rwanda	304 (215-317)	453 (306-477)	583 (355-625)

Spn = Streptococcus pneumoniae; Uncertainty range provided in parentheses.

Year	Country	Spn pneumonia deaths (UR)	Spn pneumonia deaths (UR)	Spn pneumonia deaths (UR)
2015	Rwanda	244 (173-254)	364 (245-383)	468 (285-502)
2000	Saint Kitts and Nevis	0 (0-0)	0 (0-0)	0 (0-0)
2001	Saint Kitts and Nevis	0 (0-0)	0 (0-0)	1 (0-1)
2002	Saint Kitts and Nevis	0 (0-0)	0 (0-0)	1 (0-1)
2003	Saint Kitts and Nevis	0 (0-0)	0 (0-0)	1 (0-1)
2004	Saint Kitts and Nevis	0 (0-0)	0 (0-0)	0 (0-0)
2005	Saint Kitts and Nevis	0 (0-0)	0 (0-0)	0 (0-0)
2006	Saint Kitts and Nevis	0 (0-0)	0 (0-0)	0 (0-0)
2007	Saint Kitts and Nevis	0 (0-0)	0 (0-0)	0 (0-0)
2008	Saint Kitts and Nevis	0 (0-0)	0 (0-0)	0 (0-0)
2009	Saint Kitts and Nevis	0 (0-0)	0 (0-0)	0 (0-0)
2010	Saint Kitts and Nevis	0 (0-0)	0 (0-0)	0 (0-0)
2011	Saint Kitts and Nevis	0 (0-0)	0 (0-0)	0 (0-0)
2012	Saint Kitts and Nevis	0 (0-0)	0 (0-0)	0 (0-0)
2013	Saint Kitts and Nevis	0 (0-0)	0 (0-0)	0 (0-0)
2014	Saint Kitts and Nevis	0 (0-0)	0 (0-0)	0 (0-0)
2015	Saint Kitts and Nevis	0 (0-0)	0 (0-0)	0 (0-0)
2000	Saint Lucia	1 (1-1)	1 (1-1)	1 (1-2)
2001	Saint Lucia	1 (1-1)	1 (1-1)	2 (1-2)
2002	Saint Lucia	1 (1-1)	1 (1-1)	2 (1-2)
2003	Saint Lucia	1 (1-1)	1 (1-1)	2 (1-2)
2004	Saint Lucia	1 (1-1)	2 (1-2)	2 (1-2)
2005	Saint Lucia	1 (1-1)	2 (1-2)	3 (2-3)
2006	Saint Lucia	2 (1-2)	3 (2-3)	3 (2-4)
2007	Saint Lucia	1 (1-2)	2 (1-2)	3 (2-3)
2008	Saint Lucia	1 (1-1)	2 (1-2)	2 (1-2)
2009	Saint Lucia	1 (1-1)	2 (1-2)	2 (1-3)
2010	Saint Lucia	1 (1-1)	2 (1-2)	2 (1-2)
2011	Saint Lucia	1 (1-1)	1 (1-1)	1 (1-2)
2012	Saint Lucia	0 (0-0)	1 (0-1)	1 (0-1)
2013	Saint Lucia	0 (0-0)	1 (0-1)	1 (1-1)
2014	Saint Lucia	1 (0-1)	1 (1-1)	1 (1-1)
2015	Saint Lucia	1 (0-1)	1 (1-1)	1 (1-1)
2000	Saint Vincent and the Grenadines	1 (0-1)	1 (1-1)	1 (1-1)
2001	Saint Vincent and the Grenadines	1 (0-1)	1 (1-1)	1 (1-1)
2002	Saint Vincent and the Grenadines	0 (0-0)	0 (0-0)	1 (0-1)
2003	Saint Vincent and the Grenadines	0 (0-0)	0 (0-0)	0 (0-0)
2004	Saint Vincent and the Grenadines	0 (0-0)	1 (0-1)	1 (0-1)
2005	Saint Vincent and the Grenadines	0 (0-0)	1 (0-1)	1 (0-1)
2006	Saint Vincent and the Grenadines	0 (0-0)	1 (0-1)	1 (1-1)
2007	Saint Vincent and the Grenadines	0 (0-0)	0 (0-0)	0 (0-0)
2008	Saint Vincent and the Grenadines	0 (0-0)	0 (0-0)	0 (0-0)
2009	Saint Vincent and the Grenadines	0 (0-0)	1 (0-1)	1 (1-1)
2010	Saint Vincent and the Grenadines	0 (0-0)	1 (0-1)	1 (1-1)
2011	Saint Vincent and the Grenadines	1 (1-1)	1 (1-1)	2 (1-2)
2012	Saint Vincent and the Grenadines	1 (1-1)	1 (1-1)	1 (1-1)
2013	Saint Vincent and the Grenadines	1 (1-1)	1 (1-1)	2 (1-2)
2014	Saint Vincent and the Grenadines	1 (0-1)	1 (1-1)	1 (1-1)
2015	Saint Vincent and the Grenadines	1 (0-1)	1 (1-1)	1 (1-1)
2000	Samoa	4 (3-4)	6 (4-6)	7 (5-8)
2001	Samoa	4 (3-4)	5 (4-6)	7 (4-7)
2002	Samoa	3 (2-3)	5 (3-5)	6 (4-7)
2003	Samoa	3 (2-3)	5 (3-5)	6 (4-6)
2004	Samoa	3 (2-3)	4 (3-4)	5 (3-6)
2005	Samoa	3 (2-3)	4 (3-4)	5 (3-5)
2006	Samoa	2 (2-3)	4 (2-4)	5 (3-5)
2007	Samoa	2 (2-3)	4 (2-4)	5 (3-5)
2008	Samoa	2 (2-2)	3 (2-4)	4 (3-5)
2009	Samoa	3 (2-3)	4 (3-4)	5 (3-5)
2010	Samoa	3 (2-3)	4 (3-5)	6 (3-6)
2011	Samoa	3 (2-3)	4 (3-5)	6 (3-6)
2012	Samoa	3 (2-3)	4 (3-5)	6 (3-6)
2013	Samoa	3 (2-3)	4 (3-4)	5 (3-5)
2014	Samoa	3 (2-3)	4 (3-4)	5 (3-5)
2015	Samoa	2 (2-2)	4 (2-4)	5 (3-5)
2000	San Marino	0 (0-0)	0 (0-0)	0 (0-0)
2001	San Marino	0 (0-0)	0 (0-0)	0 (0-0)
2002	San Marino	0 (0-0)	0 (0-0)	0 (0-0)
2003	San Marino	0 (0-0)	0 (0-0)	0 (0-0)
2004	San Marino	0 (0-0)	0 (0-0)	0 (0-0)
2005	San Marino	0 (0-0)	0 (0-0)	0 (0-0)
2006	San Marino	0 (0-0)	0 (0-0)	0 (0-0)
2007	San Marino	0 (0-0)	0 (0-0)	0 (0-0)
2008	San Marino	0 (0-0)	0 (0-0)	0 (0-0)
2009	San Marino	0 (0-0)	0 (0-0)	0 (0-0)
2010	San Marino	0 (0-0)	0 (0-0)	0 (0-0)
2011	San Marino	0 (0-0)	0 (0-0)	0 (0-0)
2012	San Marino	0 (0-0)	0 (0-0)	0 (0-0)
2013	San Marino	0 (0-0)	0 (0-0)	0 (0-0)
2014	San Marino	0 (0-0)	0 (0-0)	0 (0-0)
2015	San Marino	0 (0-0)	0 (0-0)	0 (0-0)
2000	Sao Tome and Principe	28 (20-29)	42 (28-44)	54 (33-58)
2001	Sao Tome and Principe	28 (20-29)	41 (28-43)	53 (32-57)
2002	Sao Tome and Principe	27 (19-28)	40 (27-42)	52 (31-55)
2003	Sao Tome and Principe	26 (18-27)	39 (26-41)	50 (30-54)
2004	Sao Tome and Principe	25 (18-26)	37 (25-39)	48 (29-51)
2005	Sao Tome and Principe	24 (17-25)	36 (24-38)	46 (28-49)
2006	Sao Tome and Principe	23 (16-24)	35 (23-36)	44 (27-48)
2007	Sao Tome and Principe	22 (16-23)	33 (22-35)	42 (26-45)
2008	Sao Tome and Principe	22 (16-23)	33 (22-35)	42 (26-45)
2009	Sao Tome and Principe	20 (14-21)	30 (21-32)	39 (24-42)
2010	Sao Tome and Principe	20 (14-21)	30 (20-32)	39 (23-41)
2011	Sao Tome and Principe	20 (14-20)	29 (20-31)	37 (23-40)
2012	Sao Tome and Principe	19 (14-20)	28 (19-30)	37 (22-39)
2013	Sao Tome and Principe	12 (9-13)	18 (12-19)	23 (14-25)
2014	Sao Tome and Principe	10 (7-10)	15 (10-16)	19 (12-20)
2015	Sao Tome and Principe	8 (6-9)	13 (9-13)	16 (10-17)
2000	Saudi Arabia	407 (289-424)	607 (409-639)	781 (475-837)
2001	Saudi Arabia	375 (266-390)	559 (377-588)	719 (437-770)
2002	Saudi Arabia	380 (269-396)	566 (382-596)	729 (443-780)
2003	Saudi Arabia	365 (259-380)	544 (367-573)	700 (426-750)
2004	Saudi Arabia	341 (242-355)	508 (343-535)	654 (398-700)
2005	Saudi Arabia	325 (231-339)	485 (327-511)	625 (380-669)
2006	Saudi Arabia	309 (219-322)	460 (310-485)	592 (360-635)
2007	Saudi Arabia	303 (215-316)	452 (305-476)	581 (354-623)
2008	Saudi Arabia	287 (204-299)	428 (289-451)	551 (335-591)
2009	Saudi Arabia	236 (168-246)	365 (246-385)	486 (296-520)
2010	Saudi Arabia	182 (129-189)	293 (197-308)	405 (246-433)
2011	Saudi Arabia	135 (96-141)	227 (153-240)	329 (200-352)

Spn = Streptococcus pneumoniae; Uncertainty range provided in parentheses.

Year	Country	Spn pneumonia deaths (UR)	Spn pneumonia deaths (UR)	Spn pneumonia deaths (UR)
2012	Saudi Arabia	119 (85-125)	203 (137-213)	296 (180-317)
2013	Saudi Arabia	89 (63-92)	155 (105-163)	235 (143-252)
2014	Saudi Arabia	78 (56-82)	138 (93-146)	212 (129-227)
2015	Saudi Arabia	73 (52-76)	130 (87-136)	199 (121-213)
2000	Senegal	2951 (2093-3076)	4402 (2969-4636)	5666 (3447-6068)
2001	Senegal	2988 (2119-3114)	4456 (3005-4693)	5735 (3489-6143)
2002	Senegal	2904 (2059-3026)	4331 (2921-4562)	5575 (3391-5970)
2003	Senegal	2799 (1985-2917)	4174 (2815-4396)	5373 (3268-5754)
2004	Senegal	2681 (1901-2794)	3998 (2696-4211)	5146 (3131-5512)
2005	Senegal	2548 (1807-2655)	3800 (2563-4003)	4891 (2976-5239)
2006	Senegal	2419 (1716-2521)	3609 (2434-3801)	4645 (2826-4975)
2007	Senegal	2276 (1614-2372)	3395 (2289-3575)	4369 (2658-4679)
2008	Senegal	2139 (1517-2229)	3190 (2151-3360)	4106 (2498-4398)
2009	Senegal	2009 (1424-2093)	2996 (2021-3155)	3856 (2346-4130)
2010	Senegal	1898 (1346-1978)	2831 (1909-2981)	3643 (2216-3902)
2011	Senegal	1798 (1275-1874)	2682 (1809-2825)	3452 (2100-3698)
2012	Senegal	1726 (1224-1799)	2574 (1736-2711)	3313 (2016-3549)
2013	Senegal	1684 (1194-1755)	2512 (1694-2645)	3232 (1966-3462)
2014	Senegal	1144 (811-1192)	1707 (1151-1797)	2196 (1336-2353)
2015	Senegal	909 (645-947)	1356 (914-1428)	1745 (1062-1869)
2000	Serbia	25 (18-26)	38 (25-40)	48 (29-52)
2001	Serbia	17 (12-18)	26 (17-27)	33 (20-36)
2002	Serbia	14 (10-14)	21 (14-22)	26 (16-28)
2003	Serbia	20 (14-21)	30 (20-32)	39 (24-42)
2004	Serbia	19 (13-20)	28 (19-30)	36 (22-39)
2005	Serbia	14 (10-15)	21 (14-23)	28 (17-30)
2006	Serbia	9 (7-10)	14 (9-15)	18 (11-19)
2007	Serbia	17 (12-17)	25 (17-26)	32 (20-34)
2008	Serbia	15 (11-16)	23 (15-24)	29 (18-31)
2009	Serbia	13 (9-14)	20 (13-21)	25 (15-27)
2010	Serbia	10 (7-11)	15 (10-16)	20 (12-21)
2011	Serbia	12 (9-13)	18 (12-19)	24 (14-25)
2012	Serbia	10 (7-11)	15 (10-16)	20 (12-21)
2013	Serbia	7 (5-7)	10 (7-11)	13 (8-14)
2014	Serbia	10 (7-10)	14 (10-15)	18 (11-20)
2015	Serbia	10 (7-10)	14 (10-15)	18 (11-20)
2000	Seychelles	0 (0-0)	1 (0-1)	1 (0-1)
2001	Seychelles	0 (0-0)	1 (0-1)	1 (0-1)
2002	Seychelles	0 (0-0)	1 (0-1)	1 (0-1)
2003	Seychelles	0 (0-0)	1 (0-1)	1 (0-1)
2004	Seychelles	0 (0-0)	1 (0-1)	1 (0-1)
2005	Seychelles	0 (0-0)	1 (0-1)	1 (0-1)
2006	Seychelles	0 (0-0)	1 (0-1)	1 (0-1)
2007	Seychelles	0 (0-0)	1 (0-1)	1 (0-1)
2008	Seychelles	0 (0-0)	1 (0-1)	1 (0-1)
2009	Seychelles	0 (0-0)	1 (0-1)	1 (0-1)
2010	Seychelles	0 (0-0)	1 (0-1)	1 (0-1)
2011	Seychelles	0 (0-0)	1 (0-1)	1 (1-1)
2012	Seychelles	0 (0-0)	1 (0-1)	1 (0-1)
2013	Seychelles	0 (0-0)	1 (0-1)	1 (1-1)
2014	Seychelles	0 (0-0)	1 (0-1)	1 (1-1)
2015	Seychelles	0 (0-0)	1 (0-1)	1 (1-1)
2000	Sierra Leone	1807 (1281-1883)	2695 (1817-2838)	3468 (2110-3714)
2001	Sierra Leone	1841 (1305-1919)	2746 (1852-2892)	3534 (2150-3785)
2002	Sierra Leone	1895 (1344-1975)	2826 (1906-2976)	3637 (2213-3896)
2003	Sierra Leone	1962 (1391-2045)	2927 (1974-3083)	3767 (2292-4035)
2004	Sierra Leone	2036 (1444-2122)	3037 (2048-3198)	3908 (2378-4186)
2005	Sierra Leone	2080 (1475-2167)	3102 (2092-3267)	3992 (2429-4276)
2006	Sierra Leone	2108 (1495-2197)	3144 (2120-3311)	4046 (2462-4334)
2007	Sierra Leone	2061 (1462-2148)	3074 (2073-3238)	3957 (2407-4238)
2008	Sierra Leone	1960 (1390-2043)	2924 (1972-3079)	3763 (2289-4030)
2009	Sierra Leone	1839 (1304-1917)	2743 (1850-2889)	3531 (2148-3782)
2010	Sierra Leone	1725 (1223-1798)	2573 (1735-2710)	3312 (2015-3547)
2011	Sierra Leone	1201 (851-1251)	1791 (1208-1886)	2305 (1402-2469)
2012	Sierra Leone	937 (664-977)	1398 (943-1472)	1799 (1094-1927)
2013	Sierra Leone	807 (573-842)	1204 (812-1268)	1550 (943-1660)
2014	Sierra Leone	796 (565-830)	1188 (801-1251)	1529 (930-1637)
2015	Sierra Leone	554 (393-578)	827 (558-871)	1064 (647-1140)
2000	Singapore	5 (4-5)	8 (5-8)	10 (6-10)
2001	Singapore	6 (4-7)	9 (6-10)	12 (7-13)
2002	Singapore	6 (5-7)	10 (6-10)	12 (8-13)
2003	Singapore	9 (6-10)	14 (9-14)	18 (11-19)
2004	Singapore	7 (5-8)	11 (7-11)	14 (8-15)
2005	Singapore	8 (6-8)	12 (8-13)	15 (9-16)
2006	Singapore	8 (5-8)	11 (8-12)	15 (9-16)
2007	Singapore	9 (7-10)	14 (9-15)	18 (11-19)
2008	Singapore	5 (4-5)	8 (5-8)	10 (6-10)
2009	Singapore	6 (4-6)	8 (6-9)	11 (7-12)
2010	Singapore	4 (3-4)	5 (4-6)	7 (4-7)
2011	Singapore	5 (3-5)	7 (5-7)	9 (5-9)
2012	Singapore	8 (6-8)	12 (8-12)	15 (9-16)
2013	Singapore	5 (4-5)	8 (6-9)	11 (7-12)
2014	Singapore	5 (4-6)	9 (6-9)	12 (7-13)
2015	Singapore	5 (4-5)	8 (6-9)	12 (7-13)
2000	Slovakia	22 (16-23)	33 (22-35)	43 (26-46)
2001	Slovakia	19 (13-20)	28 (19-30)	36 (22-39)
2002	Slovakia	19 (13-20)	28 (19-29)	36 (22-39)
2003	Slovakia	17 (12-17)	25 (17-26)	32 (20-34)
2004	Slovakia	18 (13-18)	26 (18-28)	34 (21-36)
2005	Slovakia	19 (14-20)	29 (20-31)	37 (23-40)
2006	Slovakia	16 (11-16)	23 (16-24)	30 (18-32)
2007	Slovakia	21 (15-21)	31 (21-32)	39 (24-42)
2008	Slovakia	21 (15-22)	31 (21-33)	40 (24-43)
2009	Slovakia	11 (8-12)	18 (12-19)	25 (15-26)
2010	Slovakia	8 (5-8)	13 (8-13)	18 (11-19)
2011	Slovakia	7 (5-7)	11 (8-12)	17 (10-18)
2012	Slovakia	6 (4-6)	10 (7-11)	15 (9-17)
2013	Slovakia	6 (4-6)	10 (7-11)	15 (9-17)
2014	Slovakia	5 (4-5)	9 (6-9)	13 (8-14)
2015	Slovakia	6 (4-6)	10 (6-10)	14 (9-15)
2000	Slovenia	0 (0-0)	0 (0-0)	0 (0-0)
2001	Slovenia	1 (0-1)	1 (1-1)	1 (1-1)
2002	Slovenia	1 (1-1)	1 (1-1)	2 (1-2)
2003	Slovenia	2 (1-2)	2 (2-2)	3 (2-3)
2004	Slovenia	0 (0-1)	1 (0-1)	1 (1-1)
2005	Slovenia	0 (0-0)	1 (0-1)	1 (0-1)
2006	Slovenia	0 (0-0)	0 (0-0)	0 (0-0)
2007	Slovenia	1 (1-1)	2 (1-2)	2 (1-2)
2008	Slovenia	0 (0-0)	0 (0-0)	0 (0-0)

Spn = Streptococcus pneumoniae; Uncertainty range provided in parentheses.

Year	Country	Spn pneumonia deaths (UR)	Spn pneumonia deaths (UR)	Spn pneumonia deaths (UR)
2009	Slovenia	1 (1-1)	1 (1-1)	2 (1-2)
2010	Slovenia	1 (1-1)	1 (1-1)	2 (1-2)
2011	Slovenia	1 (0-1)	1 (1-1)	1 (1-1)
2012	Slovenia	1 (0-1)	1 (1-1)	1 (1-1)
2013	Slovenia	1 (0-1)	1 (1-1)	1 (1-1)
2014	Slovenia	0 (0-0)	1 (0-1)	1 (1-1)
2015	Slovenia	0 (0-0)	1 (0-1)	1 (0-1)
2000	Solomon Islands	22 (16-23)	34 (23-35)	43 (26-46)
2001	Solomon Islands	23 (16-24)	34 (23-36)	44 (27-47)
2002	Solomon Islands	24 (17-25)	35 (24-37)	45 (28-49)
2003	Solomon Islands	24 (17-25)	36 (24-38)	46 (28-50)
2004	Solomon Islands	25 (18-26)	38 (25-40)	49 (30-52)
2005	Solomon Islands	26 (18-27)	39 (26-41)	50 (30-53)
2006	Solomon Islands	27 (19-28)	40 (27-42)	51 (31-55)
2007	Solomon Islands	27 (19-28)	40 (27-42)	52 (31-55)
2008	Solomon Islands	27 (19-28)	40 (27-43)	52 (32-56)
2009	Solomon Islands	27 (19-28)	41 (27-43)	52 (32-56)
2010	Solomon Islands	27 (19-28)	40 (27-43)	52 (32-56)
2011	Solomon Islands	27 (19-28)	40 (27-42)	52 (31-55)
2012	Solomon Islands	26 (19-27)	39 (26-41)	51 (31-54)
2013	Solomon Islands	26 (18-27)	38 (26-40)	49 (30-53)
2014	Solomon Islands	25 (17-26)	37 (25-39)	47 (29-51)
2015	Solomon Islands	18 (12-18)	26 (18-28)	34 (20-36)
2000	Somalia	3620 (2567-3772)	5399 (3641-5686)	6949 (4228-7443)
2001	Somalia	3687 (2615-3843)	5500 (3709-5792)	7079 (4306-7581)
2002	Somalia	3838 (2722-4000)	5725 (3861-6029)	7368 (4483-7892)
2003	Somalia	4111 (2915-4284)	6131 (4135-6457)	7891 (4801-8452)
2004	Somalia	4235 (3003-4414)	6318 (4260-6654)	8131 (4947-8709)
2005	Somalia	4394 (3116-4579)	6554 (4420-6902)	8435 (5131-9034)
2006	Somalia	4554 (3229-4745)	6792 (4580-7153)	8742 (5318-9363)
2007	Somalia	4716 (3344-4915)	7035 (4744-7409)	9054 (5508-9697)
2008	Somalia	4833 (3427-5037)	7209 (4862-7593)	9279 (5645-9938)
2009	Somalia	4906 (3479-5113)	7318 (4935-7707)	9419 (5730-10088)
2010	Somalia	4928 (3494-5135)	7350 (4957-7741)	9460 (5755-10132)
2011	Somalia	4885 (3464-5091)	7286 (4914-7674)	9378 (5705-10044)
2012	Somalia	4789 (3396-4991)	7143 (4817-7523)	9193 (5593-9846)
2013	Somalia	4688 (3324-4886)	6993 (4716-7365)	9000 (5475-9639)
2014	Somalia	4558 (3232-4750)	6799 (4585-7161)	8751 (5324-9372)
2015	Somalia	4493 (3186-4682)	6701 (4519-7057)	8624 (5247-9237)
2000	South Africa	4235 (3003-4413)	6316 (4260-6652)	8130 (4946-8707)
2001	South Africa	4220 (2993-4398)	6295 (4245-6630)	8102 (4929-8677)
2002	South Africa	4045 (2869-4216)	6034 (4069-6355)	7766 (4725-8318)
2003	South Africa	3789 (2686-3948)	5651 (3811-5951)	7273 (4425-7790)
2004	South Africa	3550 (2518-3700)	5296 (3571-5577)	6816 (4146-7300)
2005	South Africa	3443 (2441-3588)	5135 (3463-5408)	6609 (4021-7079)
2006	South Africa	3420 (2425-3564)	5101 (3440-5373)	6566 (3994-7032)
2007	South Africa	3518 (2495-3666)	5248 (3539-5527)	6754 (4109-7234)
2008	South Africa	3696 (2621-3852)	5513 (3718-5807)	7096 (4317-7600)
2009	South Africa	3751 (2660-3910)	5596 (3774-5893)	7202 (4381-7713)
2010	South Africa	3392 (2405-3535)	5059 (3412-5328)	6511 (3961-6974)
2011	South Africa	2943 (2087-3067)	4390 (2960-4623)	5650 (3437-6051)
2012	South Africa	2182 (1547-2274)	3255 (2195-3428)	4189 (2548-4486)
2013	South Africa	2016 (1430-2101)	3008 (2028-3168)	3871 (2355-4146)
2014	South Africa	1786 (1267-1862)	2665 (1797-2806)	3429 (2086-3673)
2015	South Africa	1118 (793-1165)	1668 (1125-1756)	2146 (1306-2299)
2000	South Sudan	2919 (2070-3042)	4354 (2936-4585)	5603 (3409-6001)
2001	South Sudan	2928 (2076-3052)	4368 (2946-4600)	5622 (3420-6021)
2002	South Sudan	2836 (2011-2956)	4230 (2853-4455)	5444 (3312-5831)
2003	South Sudan	2739 (1942-2855)	4086 (2756-4303)	5259 (3199-5632)
2004	South Sudan	2651 (1880-2763)	3954 (2667-4164)	5089 (3096-5450)
2005	South Sudan	2518 (1786-2624)	3756 (2533-3956)	4834 (2941-5178)
2006	South Sudan	2457 (1743-2561)	3665 (2472-3860)	4718 (2870-5053)
2007	South Sudan	2410 (1709-2512)	3595 (2424-3786)	4627 (2815-4956)
2008	South Sudan	2444 (1733-2547)	3645 (2458-3839)	4691 (2854-5024)
2009	South Sudan	2434 (1726-2537)	3631 (2449-3824)	4674 (2843-5005)
2010	South Sudan	2437 (1728-2539)	3634 (2451-3828)	4678 (2846-5010)
2011	South Sudan	2434 (1726-2536)	3630 (2448-3823)	4672 (2842-5004)
2012	South Sudan	2345 (1663-2444)	3498 (2359-3684)	4502 (2739-4821)
2013	South Sudan	2367 (1678-2467)	3530 (2381-3718)	4544 (2764-4867)
2014	South Sudan	2323 (1647-2421)	3465 (2336-3649)	4459 (2713-4776)
2015	South Sudan	2287 (1622-2384)	3412 (2301-3593)	4391 (2671-4703)
2000	Spain	18 (13-18)	26 (18-28)	34 (21-36)
2001	Spain	6 (4-6)	9 (6-10)	12 (7-13)
2002	Spain	16 (12-17)	24 (16-25)	31 (19-33)
2003	Spain	18 (13-19)	27 (18-28)	35 (21-37)
2004	Spain	14 (10-15)	21 (14-22)	27 (16-29)
2005	Spain	13 (9-14)	20 (13-21)	25 (15-27)
2006	Spain	18 (12-18)	26 (18-28)	34 (20-36)
2007	Spain	22 (16-23)	33 (22-34)	42 (26-45)
2008	Spain	13 (9-14)	19 (13-20)	25 (15-27)
2009	Spain	17 (12-17)	25 (17-26)	32 (19-34)
2010	Spain	14 (10-15)	21 (14-23)	28 (17-30)
2011	Spain	12 (9-13)	18 (12-19)	23 (14-25)
2012	Spain	9 (6-9)	13 (9-14)	17 (10-18)
2013	Spain	7 (5-7)	10 (7-11)	13 (8-14)
2014	Spain	8 (6-8)	12 (8-13)	15 (9-16)
2015	Spain	7 (5-8)	11 (7-11)	14 (8-15)
2000	Sri Lanka	126 (89-131)	188 (127-198)	242 (147-259)
2001	Sri Lanka	119 (84-124)	177 (119-186)	228 (138-244)
2002	Sri Lanka	113 (80-117)	168 (113-177)	216 (132-232)
2003	Sri Lanka	110 (78-115)	164 (111-173)	211 (128-226)
2004	Sri Lanka	101 (72-105)	151 (102-159)	194 (118-208)
2005	Sri Lanka	99 (70-103)	147 (99-155)	189 (115-203)
2006	Sri Lanka	90 (64-94)	134 (91-141)	173 (105-185)
2007	Sri Lanka	83 (59-87)	124 (84-131)	160 (97-171)
2008	Sri Lanka	76 (54-79)	114 (77-120)	146 (89-157)
2009	Sri Lanka	71 (50-74)	106 (72-112)	137 (83-146)
2010	Sri Lanka	76 (54-80)	114 (77-120)	146 (89-157)
2011	Sri Lanka	74 (53-77)	111 (75-117)	142 (87-153)
2012	Sri Lanka	70 (50-73)	105 (71-110)	135 (82-145)
2013	Sri Lanka	67 (47-69)	99 (67-105)	128 (78-137)
2014	Sri Lanka	62 (44-65)	92 (62-97)	119 (72-127)
2015	Sri Lanka	61 (43-63)	90 (61-95)	116 (71-124)
2000	Sudan	7034 (4988-7330)	10492 (7075-11049)	13503 (8215-14462)
2001	Sudan	7230 (5127-7534)	10784 (7272-11357)	13879 (8443-14865)
2002	Sudan	7214 (5116-7519)	10761 (7257-11333)	13850 (8426-14834)
2003	Sudan	7213 (5115-7517)	10759 (7255-11331)	13847 (8424-14830)
2004	Sudan	7168 (5083-7470)	10692 (7211-11261)	13761 (8372-14739)
2005	Sudan	6914 (4903-7206)	10313 (6955-10862)	13273 (8075-14216)

Spn = Streptococcus pneumoniae; Uncertainty range provided in parentheses.

Year	Country	Spn pneumonia deaths (UR)	Spn pneumonia deaths (UR)	Spn pneumonia deaths (UR)
2006	Sudan	6820 (4836-7108)	10173 (6861-10714)	13093 (7965-14023)
2007	Sudan	6692 (4745-6974)	9982 (6732-10513)	12847 (7816-13760)
2008	Sudan	6723 (4768-7007)	10029 (6763-10562)	12907 (7852-13824)
2009	Sudan	6541 (4638-6817)	9757 (6580-10275)	12557 (7639-13449)
2010	Sudan	6377 (4522-6645)	9511 (6414-10017)	12241 (7447-13111)
2011	Sudan	6311 (4475-6577)	9413 (6348-9914)	12115 (7370-12975)
2012	Sudan	6103 (4328-6360)	9103 (6139-9587)	11716 (7128-12548)
2013	Sudan	5430 (3850-5659)	8099 (5462-8530)	10424 (6342-11165)
2014	Sudan	3637 (2579-3790)	5425 (3658-5713)	6982 (4247-7478)
2015	Sudan	3072 (2178-3201)	4582 (3090-4826)	5897 (3588-6316)
2000	Suriname	7 (5-7)	10 (7-10)	13 (8-14)
2001	Suriname	7 (5-7)	10 (7-11)	13 (8-14)
2002	Suriname	4 (3-4)	5 (4-6)	7 (4-7)
2003	Suriname	10 (7-11)	15 (10-16)	20 (12-21)
2004	Suriname	4 (3-4)	6 (4-6)	8 (5-8)
2005	Suriname	10 (7-10)	15 (10-16)	19 (12-20)
2006	Suriname	7 (5-7)	10 (7-11)	13 (8-14)
2007	Suriname	9 (7-10)	14 (9-15)	18 (11-19)
2008	Suriname	6 (4-6)	9 (6-9)	11 (7-12)
2009	Suriname	8 (6-9)	12 (8-13)	16 (10-17)
2010	Suriname	9 (6-9)	13 (9-14)	17 (10-18)
2011	Suriname	6 (4-6)	9 (6-9)	11 (7-12)
2012	Suriname	8 (6-8)	12 (8-12)	15 (9-16)
2013	Suriname	7 (5-7)	10 (7-11)	13 (8-14)
2014	Suriname	7 (5-7)	10 (7-10)	13 (8-14)
2015	Suriname	6 (5-7)	10 (6-10)	12 (8-13)
2000	Swaziland	209 (148-218)	311 (210-328)	401 (244-429)
2001	Swaziland	215 (152-224)	320 (216-337)	412 (251-441)
2002	Swaziland	218 (155-228)	326 (220-343)	419 (255-449)
2003	Swaziland	221 (157-230)	329 (222-347)	424 (258-454)
2004	Swaziland	221 (156-230)	329 (222-347)	424 (258-454)
2005	Swaziland	219 (156-229)	327 (221-345)	421 (256-451)
2006	Swaziland	214 (152-223)	319 (215-336)	411 (250-440)
2007	Swaziland	209 (148-218)	311 (210-328)	401 (244-429)
2008	Swaziland	202 (143-211)	302 (203-318)	388 (236-416)
2009	Swaziland	193 (137-201)	287 (194-303)	370 (225-396)
2010	Swaziland	182 (129-190)	272 (183-286)	350 (213-375)
2011	Swaziland	173 (123-180)	258 (174-272)	332 (202-355)
2012	Swaziland	166 (118-173)	247 (167-261)	318 (194-341)
2013	Swaziland	159 (113-166)	238 (160-250)	306 (186-328)
2014	Swaziland	115 (81-120)	171 (115-180)	220 (134-236)
2015	Swaziland	88 (63-92)	132 (89-139)	169 (103-181)
2000	Sweden	2 (1-2)	3 (2-3)	3 (2-4)
2001	Sweden	2 (2-2)	3 (2-3)	4 (3-4)
2002	Sweden	2 (1-2)	3 (2-3)	4 (2-4)
2003	Sweden	4 (3-4)	6 (4-6)	8 (5-8)
2004	Sweden	5 (4-5)	7 (5-8)	9 (6-10)
2005	Sweden	5 (3-5)	7 (5-7)	9 (5-9)
2006	Sweden	3 (2-3)	4 (3-4)	5 (3-6)
2007	Sweden	4 (3-4)	6 (4-6)	8 (5-8)
2008	Sweden	7 (5-7)	11 (7-12)	15 (9-16)
2009	Sweden	2 (2-2)	4 (3-4)	5 (3-6)
2010	Sweden	2 (2-3)	4 (3-4)	6 (4-7)
2011	Sweden	3 (2-3)	4 (3-5)	7 (4-7)
2012	Sweden	1 (1-1)	2 (1-2)	3 (2-3)
2013	Sweden	2 (1-2)	3 (2-4)	5 (3-6)
2014	Sweden	2 (1-2)	3 (2-4)	5 (3-5)
2015	Sweden	2 (1-2)	3 (2-4)	5 (3-5)
2000	Switzerland	2 (1-2)	3 (2-3)	4 (2-4)
2001	Switzerland	3 (2-4)	5 (3-5)	7 (4-7)
2002	Switzerland	3 (2-3)	4 (3-4)	5 (3-6)
2003	Switzerland	2 (1-2)	3 (2-3)	4 (2-4)
2004	Switzerland	1 (1-1)	2 (1-2)	2 (1-2)
2005	Switzerland	3 (2-3)	4 (3-5)	6 (3-6)
2006	Switzerland	1 (1-1)	2 (1-2)	2 (1-2)
2007	Switzerland	1 (1-1)	2 (1-2)	2 (1-2)
2008	Switzerland	2 (2-2)	3 (2-4)	4 (3-5)
2009	Switzerland	2 (1-2)	3 (2-3)	3 (2-4)
2010	Switzerland	2 (1-2)	3 (2-3)	4 (2-4)
2011	Switzerland	1 (1-1)	1 (1-2)	2 (1-2)
2012	Switzerland	0 (0-0)	1 (0-1)	1 (1-1)
2013	Switzerland	0 (0-0)	1 (1-1)	1 (1-1)
2014	Switzerland	0 (0-0)	1 (0-1)	1 (1-1)
2015	Switzerland	0 (0-0)	1 (0-1)	1 (1-1)
2000	Syrian Arab Republic	476 (338-496)	710 (479-748)	914 (556-979)
2001	Syrian Arab Republic	479 (340-499)	714 (482-752)	919 (559-985)
2002	Syrian Arab Republic	443 (314-462)	661 (445-696)	850 (517-911)
2003	Syrian Arab Republic	396 (281-412)	590 (398-622)	760 (462-814)
2004	Syrian Arab Republic	358 (254-373)	534 (360-563)	688 (418-737)
2005	Syrian Arab Republic	331 (235-345)	494 (333-520)	635 (387-681)
2006	Syrian Arab Republic	318 (225-331)	474 (320-499)	610 (371-654)
2007	Syrian Arab Republic	310 (220-323)	462 (312-487)	595 (362-637)
2008	Syrian Arab Republic	299 (212-312)	447 (301-470)	575 (350-616)
2009	Syrian Arab Republic	291 (206-303)	434 (293-457)	558 (340-598)
2010	Syrian Arab Republic	262 (186-273)	391 (264-412)	503 (306-539)
2011	Syrian Arab Republic	231 (164-240)	344 (232-362)	443 (269-474)
2012	Syrian Arab Republic	184 (130-192)	274 (185-289)	353 (215-378)
2013	Syrian Arab Republic	148 (105-154)	220 (149-232)	283 (172-304)
2014	Syrian Arab Republic	127 (90-132)	189 (128-199)	244 (148-261)
2015	Syrian Arab Republic	123 (87-128)	183 (123-193)	236 (143-252)
2000	Tajikistan	1247 (884-1299)	1860 (1254-1958)	2393 (1456-2563)
2001	Tajikistan	1128 (800-1176)	1683 (1135-1772)	2166 (1318-2320)
2002	Tajikistan	1006 (713-1048)	1500 (1012-1580)	1930 (1174-2068)
2003	Tajikistan	895 (635-933)	1336 (901-1407)	1719 (1046-1841)
2004	Tajikistan	813 (577-848)	1213 (818-1278)	1562 (950-1673)
2005	Tajikistan	756 (536-788)	1128 (760-1188)	1451 (883-1554)
2006	Tajikistan	717 (508-747)	1070 (721-1126)	1376 (837-1474)
2007	Tajikistan	700 (496-729)	1044 (704-1099)	1343 (817-1438)
2008	Tajikistan	697 (494-726)	1039 (701-1095)	1338 (814-1433)
2009	Tajikistan	708 (502-738)	1056 (712-1112)	1359 (827-1455)
2010	Tajikistan	717 (509-748)	1070 (722-1127)	1377 (838-1475)
2011	Tajikistan	724 (514-755)	1080 (728-1138)	1390 (846-1489)
2012	Tajikistan	729 (517-760)	1087 (733-1145)	1399 (851-1499)
2013	Tajikistan	727 (516-758)	1085 (731-1142)	1396 (849-1495)
2014	Tajikistan	720 (510-750)	1073 (724-1130)	1381 (840-1479)
2015	Tajikistan	700 (496-729)	1044 (704-1099)	1343 (817-1439)
2000	Thailand	635 (450-662)	947 (639-998)	1219 (742-1306)
2001	Thailand	582 (413-607)	868 (585-914)	1117 (680-1197)
2002	Thailand	540 (383-563)	805 (543-848)	1036 (631-1110)

Spn = Streptococcus pneumoniae; Uncertainty range provided in parentheses.

Year	Country	Spn pneumonia deaths (UR)	Spn pneumonia deaths (UR)	Spn pneumonia deaths (UR)
2003	Thailand	504 (357-525)	752 (507-792)	968 (589-1036)
2004	Thailand	465 (330-484)	693 (468-730)	892 (543-956)
2005	Thailand	423 (300-440)	630 (425-664)	811 (493-869)
2006	Thailand	387 (274-403)	577 (389-608)	743 (452-795)
2007	Thailand	355 (251-370)	529 (357-557)	681 (414-729)
2008	Thailand	322 (228-335)	480 (323-505)	617 (375-661)
2009	Thailand	302 (214-314)	450 (303-474)	579 (352-620)
2010	Thailand	285 (202-297)	426 (287-448)	548 (333-587)
2011	Thailand	268 (190-280)	400 (270-422)	515 (313-552)
2012	Thailand	252 (179-263)	377 (254-397)	485 (295-519)
2013	Thailand	239 (170-249)	357 (241-376)	459 (279-492)
2014	Thailand	225 (159-234)	335 (226-353)	431 (262-462)
2015	Thailand	219 (155-228)	327 (220-344)	420 (256-450)
2000	The former Yugoslav Republic of Macedonia	11 (8-12)	17 (11-18)	22 (13-23)
2001	The former Yugoslav Republic of Macedonia	12 (9-13)	18 (12-19)	24 (14-25)
2002	The former Yugoslav Republic of Macedonia	10 (7-11)	15 (10-16)	19 (12-21)
2003	The former Yugoslav Republic of Macedonia	9 (7-10)	14 (10-15)	18 (11-20)
2004	The former Yugoslav Republic of Macedonia	9 (6-9)	13 (9-13)	16 (10-18)
2005	The former Yugoslav Republic of Macedonia	8 (6-8)	12 (8-12)	15 (9-16)
2006	The former Yugoslav Republic of Macedonia	7 (5-8)	11 (7-12)	14 (9-15)
2007	The former Yugoslav Republic of Macedonia	6 (4-6)	9 (6-9)	11 (7-12)
2008	The former Yugoslav Republic of Macedonia	3 (2-3)	4 (3-4)	5 (3-6)
2009	The former Yugoslav Republic of Macedonia	7 (5-7)	10 (7-11)	13 (8-14)
2010	The former Yugoslav Republic of Macedonia	6 (4-6)	8 (6-9)	11 (6-11)
2011	The former Yugoslav Republic of Macedonia	4 (3-5)	6 (4-7)	8 (5-9)
2012	The former Yugoslav Republic of Macedonia	4 (3-4)	6 (4-6)	7 (5-8)
2013	The former Yugoslav Republic of Macedonia	4 (3-4)	6 (4-6)	7 (4-8)
2014	The former Yugoslav Republic of Macedonia	4 (3-4)	5 (4-6)	7 (4-7)
2015	The former Yugoslav Republic of Macedonia	3 (2-3)	5 (3-5)	6 (4-7)
2000	Timor-Leste	299 (212-312)	446 (301-470)	575 (350-615)
2001	Timor-Leste	278 (197-289)	414 (279-436)	533 (324-571)
2002	Timor-Leste	256 (181-267)	382 (257-402)	491 (299-526)
2003	Timor-Leste	235 (167-245)	351 (236-369)	451 (275-483)
2004	Timor-Leste	214 (152-223)	320 (216-337)	411 (250-441)
2005	Timor-Leste	193 (137-202)	289 (195-304)	371 (226-398)
2006	Timor-Leste	173 (123-180)	258 (174-272)	332 (202-356)
2007	Timor-Leste	154 (109-160)	230 (155-242)	296 (180-317)
2008	Timor-Leste	140 (99-145)	208 (140-219)	268 (163-287)
2009	Timor-Leste	130 (93-136)	195 (131-205)	250 (152-268)
2010	Timor-Leste	130 (93-136)	195 (131-205)	250 (152-268)
2011	Timor-Leste	134 (95-140)	200 (135-211)	258 (157-276)
2012	Timor-Leste	145 (103-151)	217 (146-228)	279 (170-299)
2013	Timor-Leste	157 (111-163)	234 (158-246)	301 (183-322)
2014	Timor-Leste	164 (116-171)	245 (165-258)	315 (192-338)
2015	Timor-Leste	165 (117-172)	247 (166-260)	318 (193-340)
2000	Togo	791 (561-824)	1180 (796-1243)	1519 (924-1626)
2001	Togo	803 (569-836)	1197 (807-1261)	1541 (937-1650)
2002	Togo	809 (574-843)	1207 (814-1271)	1553 (945-1663)
2003	Togo	808 (573-842)	1205 (813-1269)	1551 (944-1661)
2004	Togo	805 (571-839)	1200 (809-1264)	1545 (940-1654)
2005	Togo	797 (565-831)	1189 (802-1252)	1530 (931-1639)
2006	Togo	791 (561-824)	1180 (796-1243)	1519 (924-1627)
2007	Togo	792 (562-825)	1181 (797-1244)	1520 (925-1628)
2008	Togo	810 (574-844)	1208 (815-1272)	1555 (946-1665)
2009	Togo	843 (598-878)	1257 (848-1324)	1618 (984-1733)
2010	Togo	893 (633-930)	1331 (898-1402)	1713 (1042-1835)
2011	Togo	962 (682-1003)	1436 (968-1512)	1848 (1124-1979)
2012	Togo	1026 (727-1069)	1530 (1032-1611)	1969 (1198-2109)
2013	Togo	1059 (751-1103)	1579 (1065-1663)	2032 (1236-2177)
2014	Togo	950 (674-990)	1417 (956-1492)	1824 (1109-1953)
2015	Togo	699 (495-728)	1042 (703-1098)	1341 (816-1437)
2000	Tonga	2 (1-2)	2 (2-3)	3 (2-3)
2001	Tonga	2 (1-2)	2 (2-3)	3 (2-3)
2002	Tonga	2 (1-2)	2 (2-2)	3 (2-3)
2003	Tonga	2 (1-2)	2 (2-2)	3 (2-3)
2004	Tonga	2 (1-2)	2 (2-2)	3 (2-3)
2005	Tonga	2 (1-2)	3 (2-3)	3 (2-4)
2006	Tonga	2 (1-2)	3 (2-3)	3 (2-4)
2007	Tonga	2 (1-2)	3 (2-3)	3 (2-4)
2008	Tonga	2 (1-2)	3 (2-3)	3 (2-4)
2009	Tonga	2 (1-2)	3 (2-3)	4 (2-4)
2010	Tonga	2 (1-2)	3 (2-3)	4 (2-4)
2011	Tonga	2 (1-2)	3 (2-3)	4 (2-4)
2012	Tonga	2 (1-2)	3 (2-3)	3 (2-4)
2013	Tonga	2 (1-2)	3 (2-3)	3 (2-4)
2014	Tonga	2 (1-2)	2 (2-3)	3 (2-3)
2015	Tonga	2 (1-2)	2 (2-3)	3 (2-3)
2000	Trinidad and Tobago	4 (3-4)	5 (4-6)	7 (4-8)
2001	Trinidad and Tobago	5 (4-5)	8 (5-8)	10 (6-10)
2002	Trinidad and Tobago	7 (5-7)	10 (7-10)	13 (8-13)
2003	Trinidad and Tobago	14 (10-14)	20 (14-21)	26 (16-28)
2004	Trinidad and Tobago	11 (8-11)	16 (11-17)	21 (13-22)
2005	Trinidad and Tobago	10 (7-10)	14 (10-15)	18 (11-20)
2006	Trinidad and Tobago	7 (5-7)	10 (7-11)	13 (8-14)
2007	Trinidad and Tobago	10 (7-10)	15 (10-16)	19 (12-20)
2008	Trinidad and Tobago	6 (4-6)	9 (6-9)	11 (7-12)
2009	Trinidad and Tobago	6 (5-7)	10 (6-10)	12 (7-13)
2010	Trinidad and Tobago	7 (5-7)	10 (7-11)	13 (8-14)
2011	Trinidad and Tobago	7 (5-7)	10 (7-11)	13 (8-14)
2012	Trinidad and Tobago	6 (4-6)	9 (6-9)	12 (7-13)
2013	Trinidad and Tobago	5 (3-5)	7 (5-8)	10 (6-11)
2014	Trinidad and Tobago	4 (3-4)	6 (4-6)	9 (5-9)
2015	Trinidad and Tobago	3 (2-3)	5 (4-6)	8 (5-8)
2000	Tunisia	205 (146-214)	306 (206-322)	394 (240-422)
2001	Tunisia	182 (129-190)	271 (183-286)	349 (213-374)
2002	Tunisia	163 (116-170)	243 (164-256)	313 (191-336)
2003	Tunisia	159 (113-166)	237 (160-250)	305 (186-327)
2004	Tunisia	146 (104-152)	218 (147-229)	280 (171-300)
2005	Tunisia	134 (95-139)	200 (135-210)	257 (156-275)
2006	Tunisia	110 (78-115)	164 (111-173)	211 (128-226)
2007	Tunisia	102 (72-106)	152 (102-160)	195 (119-209)
2008	Tunisia	94 (67-98)	141 (95-148)	181 (110-194)
2009	Tunisia	89 (63-92)	132 (89-139)	170 (103-182)
2010	Tunisia	83 (59-87)	124 (84-131)	160 (97-171)
2011	Tunisia	80 (57-84)	120 (81-126)	154 (94-165)
2012	Tunisia	83 (59-87)	124 (84-131)	160 (97-171)
2013	Tunisia	81 (57-84)	121 (82-127)	156 (95-167)
2014	Tunisia	77 (54-80)	115 (77-121)	147 (90-158)
2015	Tunisia	72 (51-75)	107 (72-113)	138 (84-148)

Spn = Streptococcus pneumoniae; Uncertainty range provided in parentheses.

Year	Country	Spn pneumonia deaths (UR)	Spn pneumonia deaths (UR)	Spn pneumonia deaths (UR)
2000	Turkey	1345 (954-1402)	2007 (1353-2113)	2583 (1571-2766)
2001	Turkey	1189 (843-1239)	1773 (1196-1867)	2282 (1388-2444)
2002	Turkey	1039 (736-1082)	1549 (1045-1632)	1994 (1213-2136)
2003	Turkey	913 (647-951)	1361 (918-1434)	1752 (1066-1877)
2004	Turkey	802 (569-836)	1196 (807-1260)	1540 (937-1649)
2005	Turkey	714 (506-744)	1065 (718-1121)	1370 (834-1468)
2006	Turkey	628 (446-655)	937 (632-987)	1206 (734-1292)
2007	Turkey	668 (474-697)	997 (672-1050)	1283 (781-1374)
2008	Turkey	620 (440-646)	925 (623-974)	1190 (724-1274)
2009	Turkey	483 (343-504)	747 (504-787)	993 (604-1064)
2010	Turkey	299 (212-312)	468 (316-493)	629 (383-674)
2011	Turkey	193 (137-201)	320 (216-337)	455 (277-488)
2012	Turkey	135 (95-140)	227 (153-239)	329 (200-352)
2013	Turkey	183 (129-190)	308 (208-325)	448 (273-480)
2014	Turkey	147 (105-154)	249 (168-262)	361 (220-386)
2015	Turkey	139 (98-144)	234 (158-246)	339 (206-363)
2000	Turkmenistan	497 (353-518)	742 (500-781)	955 (581-1022)
2001	Turkmenistan	482 (342-503)	719 (485-758)	926 (563-992)
2002	Turkmenistan	488 (346-509)	728 (491-767)	937 (570-1003)
2003	Turkmenistan	504 (358-525)	752 (507-792)	968 (589-1037)
2004	Turkmenistan	514 (364-535)	766 (517-807)	986 (600-1056)
2005	Turkmenistan	508 (360-530)	758 (511-798)	975 (593-1045)
2006	Turkmenistan	493 (350-514)	735 (496-774)	946 (576-1014)
2007	Turkmenistan	466 (331-486)	695 (469-732)	895 (545-959)
2008	Turkmenistan	431 (305-449)	642 (433-676)	827 (503-885)
2009	Turkmenistan	398 (282-414)	593 (400-625)	763 (464-818)
2010	Turkmenistan	378 (268-394)	564 (380-594)	726 (441-777)
2011	Turkmenistan	363 (258-379)	542 (365-571)	697 (424-747)
2012	Turkmenistan	358 (254-373)	534 (360-562)	687 (418-736)
2013	Turkmenistan	356 (253-371)	532 (359-560)	684 (416-733)
2014	Turkmenistan	354 (251-369)	529 (356-557)	680 (414-729)
2015	Turkmenistan	347 (246-362)	518 (349-546)	667 (406-714)
2000	Tuvalu	1 (0-1)	1 (1-1)	1 (1-1)
2001	Tuvalu	0 (0-0)	1 (0-1)	1 (1-1)
2002	Tuvalu	0 (0-0)	1 (0-1)	1 (1-1)
2003	Tuvalu	0 (0-0)	1 (0-1)	1 (1-1)
2004	Tuvalu	0 (0-0)	0 (0-1)	1 (0-1)
2005	Tuvalu	0 (0-0)	0 (0-1)	1 (0-1)
2006	Tuvalu	0 (0-0)	0 (0-1)	1 (0-1)
2007	Tuvalu	0 (0-0)	0 (0-0)	0 (0-0)
2008	Tuvalu	0 (0-0)	0 (0-0)	0 (0-0)
2009	Tuvalu	0 (0-0)	1 (0-1)	1 (0-1)
2010	Tuvalu	0 (0-0)	0 (0-0)	0 (0-0)
2011	Tuvalu	0 (0-0)	0 (0-0)	0 (0-0)
2012	Tuvalu	0 (0-0)	0 (0-0)	0 (0-0)
2013	Tuvalu	0 (0-0)	0 (0-0)	0 (0-0)
2014	Tuvalu	0 (0-0)	0 (0-0)	0 (0-0)
2015	Tuvalu	0 (0-0)	0 (0-0)	0 (0-0)
2000	Uganda	6651 (4716-6931)	9921 (6690-10448)	12768 (7768-13675)
2001	Uganda	6527 (4628-6802)	9735 (6565-10253)	12529 (7622-13419)
2002	Uganda	6439 (4566-6710)	9604 (6476-10114)	12360 (7519-13238)
2003	Uganda	6252 (4433-6516)	9325 (6289-9821)	12002 (7302-12855)
2004	Uganda	6410 (4545-6680)	9561 (6448-10070)	12306 (7486-13180)
2005	Uganda	6391 (4532-6660)	9533 (6429-10040)	12269 (7464-13141)
2006	Uganda	6086 (4315-6342)	9078 (6122-9560)	11683 (7108-12513)
2007	Uganda	5859 (4155-6106)	8739 (5894-9204)	11248 (6843-12047)
2008	Uganda	5743 (4072-5985)	8566 (5777-9021)	11025 (6707-11808)
2009	Uganda	5521 (3915-5754)	8235 (5554-8673)	10599 (6448-11352)
2010	Uganda	5441 (3858-5670)	8116 (5473-8547)	10445 (6355-11187)
2011	Uganda	5401 (3830-5629)	8056 (5433-8485)	10369 (6308-11105)
2012	Uganda	5371 (3809-5598)	8012 (5403-8438)	10312 (6273-11044)
2013	Uganda	5268 (3736-5490)	7858 (5299-8275)	10113 (6152-10831)
2014	Uganda	4256 (3018-4436)	6349 (4282-6686)	8171 (4971-8752)
2015	Uganda	3626 (2571-3779)	5409 (3648-5696)	6961 (4235-7456)
2000	Ukraine	235 (167-245)	351 (237-370)	452 (275-484)
2001	Ukraine	207 (147-216)	309 (208-325)	398 (242-426)
2002	Ukraine	180 (128-187)	268 (181-283)	345 (210-370)
2003	Ukraine	160 (114-167)	239 (161-252)	307 (187-329)
2004	Ukraine	144 (102-150)	214 (145-226)	276 (168-296)
2005	Ukraine	136 (97-142)	203 (137-214)	262 (159-280)
2006	Ukraine	131 (93-137)	196 (132-206)	252 (153-270)
2007	Ukraine	153 (108-159)	228 (154-240)	293 (178-314)
2008	Ukraine	160 (114-167)	239 (161-252)	308 (187-330)
2009	Ukraine	152 (108-158)	226 (153-238)	291 (177-312)
2010	Ukraine	147 (104-153)	220 (148-231)	283 (172-303)
2011	Ukraine	129 (91-134)	192 (129-202)	247 (150-265)
2012	Ukraine	129 (92-135)	193 (130-203)	248 (151-266)
2013	Ukraine	113 (80-118)	169 (114-178)	217 (132-233)
2014	Ukraine	98 (69-102)	146 (99-154)	188 (114-201)
2015	Ukraine	85 (60-89)	127 (86-134)	164 (99-175)
2000	United Arab Emirates	12 (9-13)	18 (12-19)	24 (14-25)
2001	United Arab Emirates	12 (8-12)	17 (12-18)	22 (14-24)
2002	United Arab Emirates	11 (8-11)	16 (11-17)	20 (12-22)
2003	United Arab Emirates	10 (7-11)	15 (10-16)	20 (12-21)
2004	United Arab Emirates	11 (7-11)	16 (11-17)	20 (12-22)
2005	United Arab Emirates	11 (8-11)	16 (11-17)	21 (13-23)
2006	United Arab Emirates	11 (8-12)	17 (11-18)	22 (13-23)
2007	United Arab Emirates	12 (9-13)	19 (13-20)	24 (15-26)
2008	United Arab Emirates	13 (9-14)	20 (13-21)	25 (15-27)
2009	United Arab Emirates	13 (10-14)	20 (14-21)	26 (16-28)
2010	United Arab Emirates	10 (7-11)	17 (11-17)	22 (14-24)
2011	United Arab Emirates	8 (6-9)	14 (9-15)	20 (12-21)
2012	United Arab Emirates	7 (5-7)	12 (8-13)	18 (11-19)
2013	United Arab Emirates	7 (5-7)	11 (7-12)	16 (10-17)
2014	United Arab Emirates	5 (3-5)	8 (6-9)	13 (8-14)
2015	United Arab Emirates	4 (3-4)	7 (5-8)	11 (7-12)
2000	United Kingdom	66 (47-69)	99 (67-104)	127 (77-136)
2001	United Kingdom	65 (46-68)	97 (65-102)	124 (76-133)
2002	United Kingdom	52 (37-55)	78 (53-82)	101 (61-108)
2003	United Kingdom	51 (36-53)	76 (51-80)	97 (59-104)
2004	United Kingdom	53 (38-55)	79 (53-83)	102 (62-109)
2005	United Kingdom	57 (41-60)	85 (57-90)	110 (67-118)
2006	United Kingdom	54 (38-56)	80 (54-85)	104 (63-111)
2007	United Kingdom	43 (31-45)	68 (46-71)	91 (55-98)
2008	United Kingdom	43 (31-45)	70 (47-74)	98 (59-105)
2009	United Kingdom	44 (31-45)	70 (48-74)	98 (60-105)
2010	United Kingdom	31 (22-32)	53 (35-55)	77 (47-82)
2011	United Kingdom	21 (15-22)	38 (25-40)	59 (36-63)
2012	United Kingdom	14 (10-14)	25 (17-26)	40 (25-43)

Spn = Streptococcus pneumoniae; Uncertainty range provided in parentheses.

Year	Country	Spn pneumonia deaths (UR)	Spn pneumonia deaths (UR)	Spn pneumonia deaths (UR)
2013	United Kingdom	9 (7-10)	18 (12-19)	29 (17-31)
2014	United Kingdom	11 (8-11)	20 (14-21)	33 (20-35)
2015	United Kingdom	10 (7-11)	19 (13-20)	31 (19-33)
2000	United Republic of Tanzania	9438 (6692-9836)	14077 (9493-14826)	18118 (11022-19405)
2001	United Republic of Tanzania	9111 (6461-9495)	13590 (9165-14313)	17491 (10641-18734)
2002	United Republic of Tanzania	8701 (6170-9068)	12979 (8752-13669)	16704 (10162-17890)
2003	United Republic of Tanzania	8774 (6222-9144)	13088 (8826-13784)	16845 (10247-18041)
2004	United Republic of Tanzania	8545 (6060-8906)	12746 (8596-13424)	16405 (9980-17570)
2005	United Republic of Tanzania	8284 (5874-8634)	12357 (8333-13014)	15904 (9675-17033)
2006	United Republic of Tanzania	7985 (5662-8322)	11910 (8032-12544)	15329 (9326-16418)
2007	United Republic of Tanzania	7516 (5330-7833)	11211 (7561-11807)	14429 (8778-15454)
2008	United Republic of Tanzania	7122 (5050-7422)	10623 (7164-11188)	13672 (8317-14643)
2009	United Republic of Tanzania	6813 (4831-7100)	10162 (6853-10703)	13079 (7957-14008)
2010	United Republic of Tanzania	6586 (4670-6863)	9823 (6624-10345)	12643 (7691-13541)
2011	United Republic of Tanzania	6369 (4516-6637)	9500 (6406-10005)	12227 (7438-13095)
2012	United Republic of Tanzania	6297 (4465-6562)	9392 (6334-9892)	12088 (7354-12947)
2013	United Republic of Tanzania	4423 (3136-4610)	6597 (4449-6948)	8491 (5166-9094)
2014	United Republic of Tanzania	3466 (2458-3612)	5170 (3486-5445)	6654 (4048-7126)
2015	United Republic of Tanzania	2942 (2086-3066)	4388 (2959-4622)	5648 (3436-6049)
2000	United States of America	375 (266-391)	559 (377-589)	719 (438-771)
2001	United States of America	385 (273-401)	574 (387-605)	739 (450-792)
2002	United States of America	339 (240-353)	518 (349-545)	681 (414-729)
2003	United States of America	338 (240-352)	537 (362-566)	734 (447-786)
2004	United States of America	285 (202-297)	462 (312-487)	644 (392-689)
2005	United States of America	227 (161-237)	380 (256-400)	546 (332-585)
2006	United States of America	266 (188-277)	435 (293-458)	611 (372-654)
2007	United States of America	224 (159-233)	369 (249-388)	523 (318-560)
2008	United States of America	246 (174-256)	404 (272-425)	569 (346-609)
2009	United States of America	268 (190-279)	437 (295-461)	614 (373-657)
2010	United States of America	192 (136-200)	320 (216-337)	458 (279-490)
2011	United States of America	133 (94-139)	236 (159-249)	365 (222-391)
2012	United States of America	81 (57-84)	148 (100-156)	236 (144-253)
2013	United States of America	65 (46-67)	119 (80-125)	193 (118-207)
2014	United States of America	63 (45-66)	117 (79-124)	192 (117-206)
2015	United States of America	61 (43-64)	113 (77-119)	186 (113-199)
2000	Uruguay	29 (20-30)	43 (29-45)	55 (34-59)
2001	Uruguay	35 (25-37)	52 (35-55)	67 (41-72)
2002	Uruguay	32 (23-33)	48 (32-50)	61 (37-66)
2003	Uruguay	31 (22-32)	46 (31-48)	59 (36-63)
2004	Uruguay	23 (16-24)	34 (23-36)	44 (27-47)
2005	Uruguay	29 (20-30)	43 (29-45)	55 (34-59)
2006	Uruguay	27 (19-28)	40 (27-42)	51 (31-55)
2007	Uruguay	31 (22-33)	47 (32-49)	60 (37-64)
2008	Uruguay	28 (20-30)	42 (29-45)	55 (33-58)
2009	Uruguay	27 (19-28)	41 (27-43)	52 (32-56)
2010	Uruguay	15 (10-15)	23 (16-25)	32 (20-35)
2011	Uruguay	10 (7-11)	17 (12-18)	25 (15-27)
2012	Uruguay	9 (6-9)	15 (10-15)	21 (13-23)
2013	Uruguay	5 (4-5)	9 (6-9)	13 (8-14)
2014	Uruguay	5 (3-5)	9 (6-9)	13 (8-14)
2015	Uruguay	4 (3-4)	7 (5-8)	11 (7-12)
2000	Uzbekistan	1912 (1356-1993)	2852 (1923-3004)	3670 (2233-3931)
2001	Uzbekistan	1807 (1281-1883)	2696 (1818-2839)	3469 (2111-3716)
2002	Uzbekistan	1713 (1215-1785)	2555 (1723-2691)	3289 (2001-3522)
2003	Uzbekistan	1627 (1154-1695)	2427 (1636-2556)	3123 (1900-3345)
2004	Uzbekistan	1573 (1115-1639)	2346 (1582-2471)	3020 (1837-3234)
2005	Uzbekistan	1527 (1083-1592)	2278 (1536-2400)	2932 (1784-3141)
2006	Uzbekistan	1509 (1070-1572)	2250 (1517-2370)	2896 (1762-3102)
2007	Uzbekistan	1500 (1064-1563)	2238 (1509-2357)	2880 (1752-3085)
2008	Uzbekistan	1510 (1071-1574)	2253 (1519-2372)	2899 (1764-3105)
2009	Uzbekistan	1514 (1073-1578)	2258 (1523-2378)	2906 (1768-3112)
2010	Uzbekistan	1510 (1071-1574)	2253 (1519-2373)	2899 (1764-3105)
2011	Uzbekistan	1489 (1056-1552)	2221 (1498-2339)	2858 (1739-3061)
2012	Uzbekistan	1457 (1033-1518)	2173 (1466-2289)	2797 (1702-2996)
2013	Uzbekistan	1415 (1004-1475)	2111 (1424-2223)	2717 (1653-2910)
2014	Uzbekistan	1374 (974-1432)	2049 (1382-2158)	2638 (1605-2825)
2015	Uzbekistan	1309 (928-1364)	1952 (1317-2056)	2513 (1529-2691)
2000	Vanuatu	7 (5-8)	11 (7-12)	14 (9-15)
2001	Vanuatu	7 (5-8)	11 (7-12)	14 (9-15)
2002	Vanuatu	6 (4-6)	9 (6-9)	11 (7-12)
2003	Vanuatu	5 (3-5)	7 (5-8)	9 (6-10)
2004	Vanuatu	5 (4-5)	8 (5-8)	10 (6-11)
2005	Vanuatu	7 (5-7)	10 (7-11)	13 (8-14)
2006	Vanuatu	7 (5-8)	11 (7-11)	14 (9-15)
2007	Vanuatu	8 (6-8)	12 (8-12)	15 (9-16)
2008	Vanuatu	8 (6-9)	12 (8-13)	16 (10-17)
2009	Vanuatu	9 (6-9)	13 (9-13)	16 (10-18)
2010	Vanuatu	9 (6-9)	13 (9-14)	17 (10-18)
2011	Vanuatu	9 (7-10)	14 (9-15)	18 (11-19)
2012	Vanuatu	10 (7-10)	15 (10-15)	19 (11-20)
2013	Vanuatu	9 (7-10)	14 (9-15)	18 (11-19)
2014	Vanuatu	9 (6-9)	13 (9-14)	17 (11-19)
2015	Vanuatu	9 (6-9)	13 (9-14)	17 (10-18)
2000	Venezuela (Bolivarian Republic of)	277 (197-289)	413 (279-435)	532 (324-570)
2001	Venezuela (Bolivarian Republic of)	362 (256-377)	539 (364-568)	694 (422-743)
2002	Venezuela (Bolivarian Republic of)	313 (222-327)	467 (315-492)	601 (366-644)
2003	Venezuela (Bolivarian Republic of)	367 (260-382)	547 (369-576)	704 (428-754)
2004	Venezuela (Bolivarian Republic of)	382 (271-399)	570 (385-601)	734 (447-786)
2005	Venezuela (Bolivarian Republic of)	342 (243-357)	511 (344-538)	657 (400-704)
2006	Venezuela (Bolivarian Republic of)	346 (245-360)	516 (348-543)	664 (404-711)
2007	Venezuela (Bolivarian Republic of)	317 (225-330)	473 (319-498)	608 (370-652)
2008	Venezuela (Bolivarian Republic of)	298 (212-311)	445 (300-469)	573 (348-613)
2009	Venezuela (Bolivarian Republic of)	282 (200-294)	421 (284-443)	542 (329-580)
2010	Venezuela (Bolivarian Republic of)	272 (193-284)	406 (274-428)	523 (318-560)
2011	Venezuela (Bolivarian Republic of)	287 (203-299)	428 (289-451)	551 (335-590)
2012	Venezuela (Bolivarian Republic of)	273 (193-284)	407 (274-428)	523 (318-560)
2013	Venezuela (Bolivarian Republic of)	265 (188-277)	396 (267-417)	509 (310-546)
2014	Venezuela (Bolivarian Republic of)	262 (185-273)	390 (263-411)	502 (305-538)
2015	Venezuela (Bolivarian Republic of)	239 (170-249)	363 (245-383)	475 (289-509)
2000	Viet Nam	2253 (1597-2348)	3360 (2266-3539)	4325 (2631-4632)
2001	Viet Nam	2090 (1482-2178)	3117 (2102-3283)	4012 (2440-4296)
2002	Viet Nam	2059 (1460-2146)	3072 (2072-3235)	3954 (2405-4234)
2003	Viet Nam	2063 (1463-2150)	3077 (2075-3240)	3960 (2409-4241)
2004	Viet Nam	2033 (1442-2119)	3033 (2045-3194)	3903 (2375-4181)
2005	Viet Nam	1970 (1397-2053)	2938 (1981-3094)	3782 (2301-4050)
2006	Viet Nam	1885 (1337-1965)	2812 (1896-2962)	3619 (2202-3876)
2007	Viet Nam	1768 (1254-1843)	2638 (1779-2778)	3395 (2065-3636)
2008	Viet Nam	1631 (1157-1700)	2433 (1641-2563)	3132 (1905-3354)
2009	Viet Nam	1515 (1075-1579)	2260 (1524-2381)	2909 (1770-3116)

Spn = Streptococcus pneumoniae; Uncertainty range provided in parentheses.

Year	Country	Spn pneumonia deaths (UR)	Spn pneumonia deaths (UR)	Spn pneumonia deaths (UR)
2010	Viet Nam	1528 (1084-1593)	2279 (1537-2401)	2934 (1785-3142)
2011	Viet Nam	1552 (1101-1618)	2315 (1561-2438)	2980 (1813-3191)
2012	Viet Nam	1499 (1063-1562)	2235 (1507-2354)	2877 (1750-3081)
2013	Viet Nam	1469 (1042-1531)	2192 (1478-2308)	2821 (1716-3021)
2014	Viet Nam	1462 (1037-1524)	2181 (1471-2297)	2807 (1707-3006)
2015	Viet Nam	1430 (1014-1490)	2133 (1439-2247)	2745 (1670-2940)
2000	Yemen	3924 (2782-4089)	5853 (3947-6164)	7533 (4583-8068)
2001	Yemen	3719 (2637-3876)	5547 (3741-5842)	7139 (4343-7646)
2002	Yemen	3547 (2515-3697)	5291 (3568-5572)	6810 (4143-7293)
2003	Yemen	3412 (2419-3555)	5089 (3432-5359)	6549 (3984-7015)
2004	Yemen	3277 (2324-3415)	4888 (3296-5148)	6291 (3827-6738)
2005	Yemen	3136 (2224-3268)	4678 (3154-4926)	6020 (3662-6448)
2006	Yemen	2986 (2117-3112)	4453 (3003-4690)	5732 (3487-6139)
2007	Yemen	2835 (2010-2954)	4228 (2851-4453)	5442 (3311-5828)
2008	Yemen	2667 (1891-2779)	3978 (2683-4190)	5120 (3115-5484)
2009	Yemen	2511 (1781-2617)	3746 (2526-3945)	4821 (2933-5163)
2010	Yemen	2349 (1666-2448)	3504 (2363-3691)	4510 (2744-4831)
2011	Yemen	1780 (1262-1855)	2655 (1790-2796)	3417 (2079-3660)
2012	Yemen	1345 (954-1401)	2006 (1353-2113)	2582 (1571-2765)
2013	Yemen	1160 (823-1209)	1731 (1167-1823)	2227 (1355-2386)
2014	Yemen	1075 (762-1120)	1604 (1081-1689)	2064 (1256-2210)
2015	Yemen	641 (454-668)	956 (645-1007)	1230 (749-1318)
2000	Zambia	3828 (2714-3989)	5710 (3850-6013)	7348 (4470-7870)
2001	Zambia	3720 (2638-3877)	5549 (3742-5844)	7142 (4345-7650)
2002	Zambia	3543 (2512-3692)	5284 (3564-5565)	6801 (4138-7284)
2003	Zambia	3356 (2380-3498)	5006 (3376-5272)	6443 (3920-6901)
2004	Zambia	3209 (2275-3344)	4786 (3228-5041)	6160 (3748-6598)
2005	Zambia	3101 (2199-3231)	4625 (3119-4871)	5952 (3621-6375)
2006	Zambia	3029 (2148-3157)	4519 (3047-4759)	5816 (3538-6229)
2007	Zambia	2988 (2119-3114)	4457 (3005-4694)	5736 (3489-6143)
2008	Zambia	2966 (2103-3091)	4424 (2983-4659)	5694 (3464-6098)
2009	Zambia	2921 (2072-3044)	4357 (2939-4589)	5608 (3412-6006)
2010	Zambia	2863 (2030-2984)	4271 (2880-4498)	5497 (3344-5888)
2011	Zambia	2786 (1975-2903)	4155 (2802-4376)	5348 (3253-5727)
2012	Zambia	2672 (1895-2785)	3986 (2688-4198)	5130 (3121-5494)
2013	Zambia	2551 (1809-2659)	3806 (2566-4008)	4898 (2980-5246)
2014	Zambia	1834 (1300-1911)	2735 (1845-2881)	3521 (2142-3771)
2015	Zambia	1509 (1070-1572)	2251 (1518-2370)	2897 (1762-3102)
2000	Zimbabwe	1604 (1137-1671)	2392 (1613-2519)	3079 (1873-3297)
2001	Zimbabwe	1655 (1174-1725)	2469 (1665-2600)	3177 (1933-3403)
2002	Zimbabwe	1731 (1227-1803)	2581 (1741-2718)	3322 (2021-3558)
2003	Zimbabwe	1821 (1291-1898)	2716 (1832-2860)	3496 (2127-3744)
2004	Zimbabwe	1927 (1366-2008)	2874 (1938-3027)	3699 (2251-3962)
2005	Zimbabwe	2045 (1450-2132)	3051 (2057-3213)	3927 (2389-4205)
2006	Zimbabwe	2143 (1519-2233)	3196 (2155-3366)	4114 (2503-4406)
2007	Zimbabwe	2234 (1584-2328)	3332 (2247-3509)	4288 (2608-4592)
2008	Zimbabwe	2308 (1636-2405)	3442 (2321-3625)	4430 (2695-4745)
2009	Zimbabwe	2364 (1676-2464)	3526 (2378-3714)	4538 (2761-4861)
2010	Zimbabwe	2392 (1696-2493)	3568 (2406-3757)	4592 (2793-4918)
2011	Zimbabwe	2409 (1708-2510)	3593 (2423-3784)	4624 (2813-4952)
2012	Zimbabwe	2179 (1545-2271)	3250 (2192-3423)	4183 (2545-4480)
2013	Zimbabwe	1467 (1040-1529)	2188 (1476-2305)	2817 (1713-3017)
2014	Zimbabwe	1269 (900-1323)	1893 (1277-1994)	2437 (1482-2610)
2015	Zimbabwe	1168 (828-1217)	1742 (1175-1835)	2243 (1364-2402)

Spn = Streptococcus pneumoniae; Uncertainty range provided in parentheses.

Webappendix 13: Comparison of meningitis mortality and morbidity estimates, proportional approach versus incidence approach

Figure 10: Pneumococcal meningitis cases by approach for 2000-2015 (bars indicate uncertainty ranges)

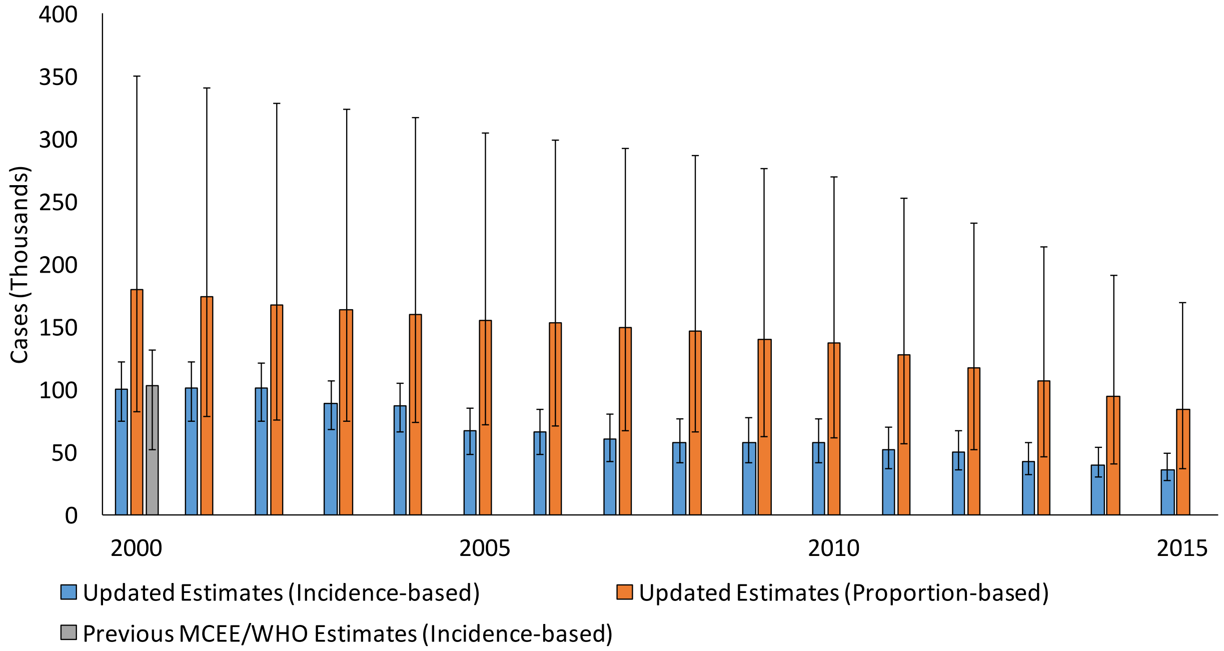

Figure 11: Pneumococcal meningitis deaths by approach for 2000-2015 (bars indicate uncertainty ranges)

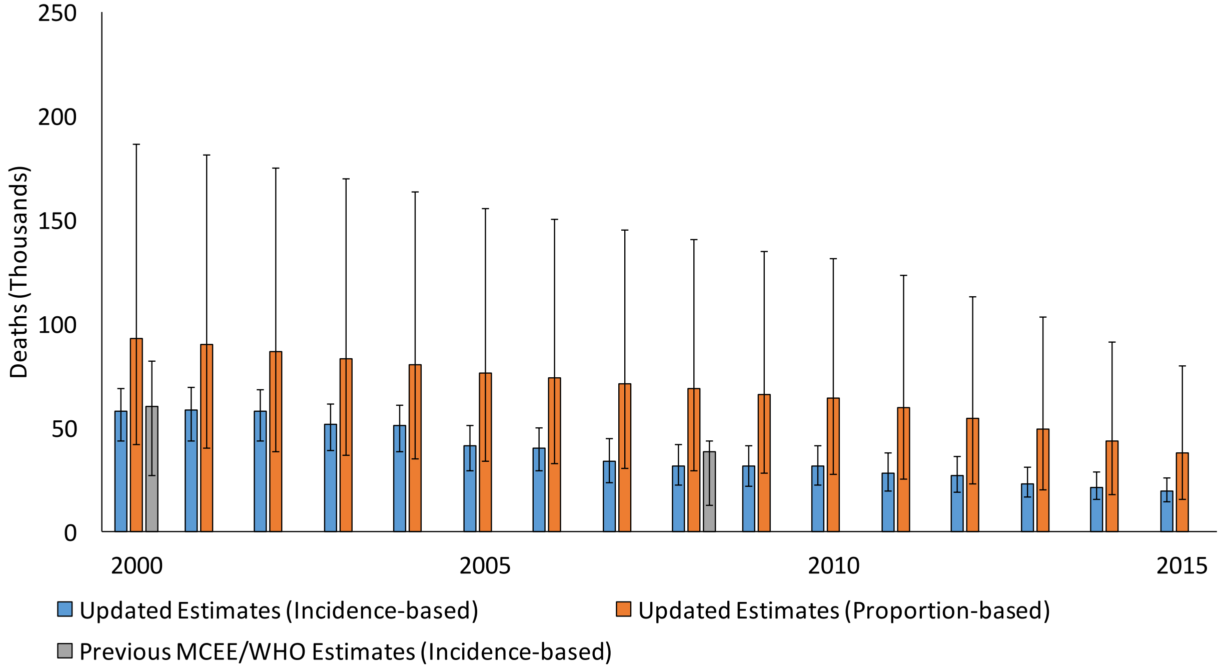

Figure 12: Hib meningitis cases by approach for 2000-2015 (bars indicate uncertainty ranges)

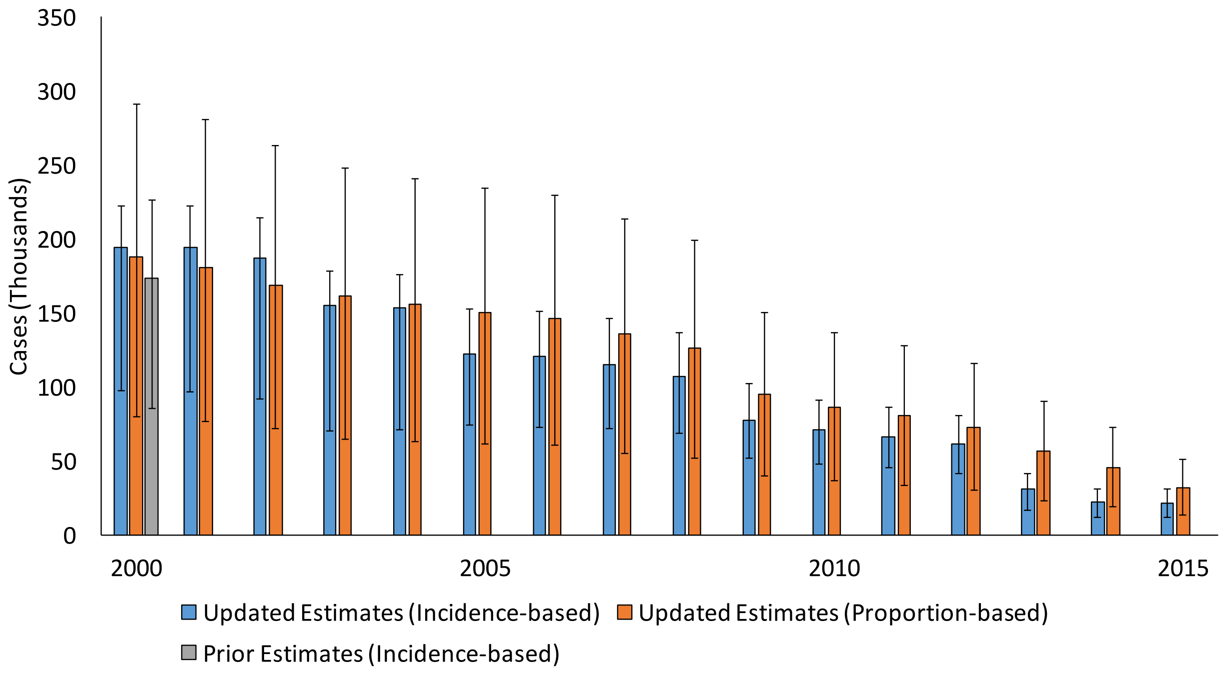

Figure 13: Hib meningitis deaths by approach for 2000-2015 (bars indicate uncertainty ranges)

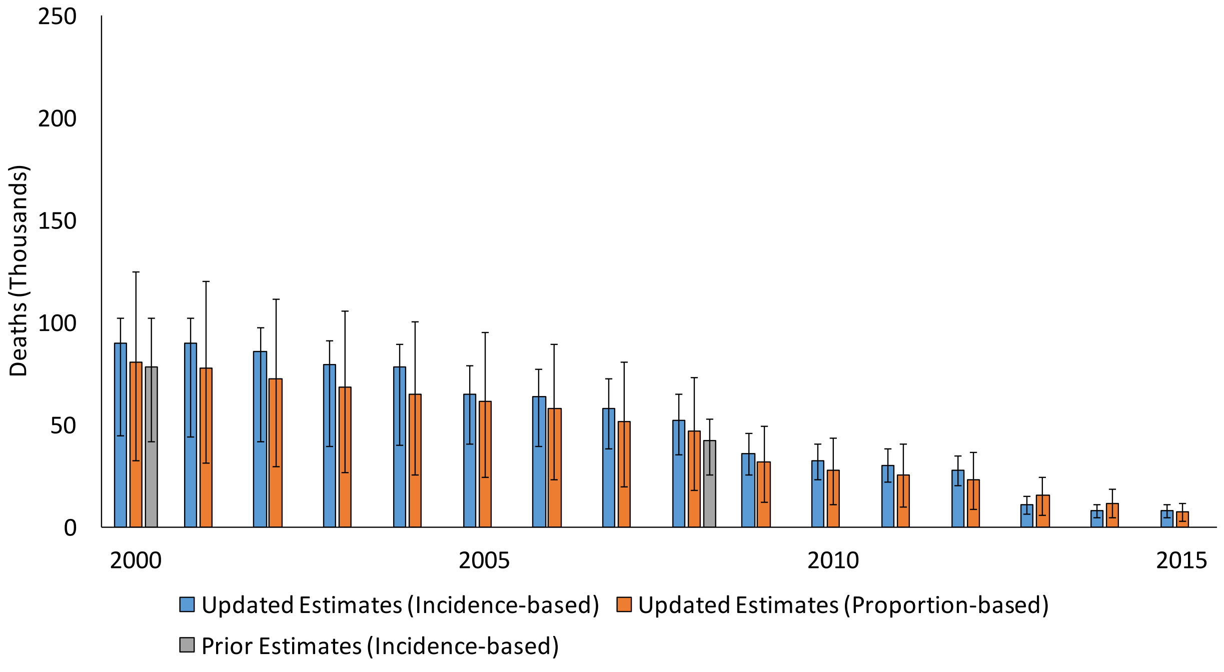

Webappendix 14: Plausibility check for pneumococcal morbidity and mortality

We checked the plausibility of our pneumococcal morbidity estimates for Bangladesh and Mozambique. A population-based, active-surveillance, active-case finding study conducted in Bangladesh[10] measured an IPD incidence of 447 cases 100,000 children less than 5 years. A clinic-based, passive-surveillance study conducted in Mozambique[11] found an IPD incidence of 416 cases per 100,000 children less than 5 years. We adjusted these estimates using the ratio of clinical pneumonia cases prevented per 1,000 children (i.e., 17) to IPD prevented per 1,000 children (i.e., 1·9) in a PCV trial from The Gambia to account for the lack of diagnostic sensitivity of IPD for all pneumococcal disease.[12] Our modeled values underestimated the pneumococcal disease burden by 39% in Bangladesh and 24% in Mozambique in the same years the studies were conducted, however the uncertainty ranges for the modeled estimates include the values observed in each of the two studies.

An RCT of PCV conducted in The Gambia was used to validate the output of our pneumococcal mortality models. The trial measured a 16% (95% CI: 3-28%) reduction in all-cause mortality in children 3-29 months and a 50% (95% CI: 21-69%) reduction in all-serotype IPD.[12] These results together imply that 32% (95% CI: 10-54%) of all deaths in the control group were attributable to pneumococcus, assuming that the serotype distribution of IPD and PCV efficacy against VT IPD was similar to that of pneumococcal deaths. We disaggregated our modeled 1-59 month old mortality estimates for The Gambia using regional data from a review of the age distribution of pneumococcal deaths[13] to determine the number of pneumococcal deaths estimated in children 3-23 months. We also used observational data from a study conducted in The Gambia[14] to estimate all-cause child deaths from the United Nations (UN) Inter-agency Group for Child Mortality Estimation (IGME) occurring in children 3-23 months.[15] The modeled estimate of the proportion of all-cause mortality accounted for by pneumococcus in this 3-23 month age group in The Gambia (17%) was lower than the inferred point estimate from the trial but was within the confidence intervals, indicating that our point estimate of disease burden may be an underestimate.

We were unable to validate the plausibility of our Hib cases and deaths because there was no other study that independently quantified the burden accounting for the poor diagnostic sensitivity.

References

1. Lucero MG, Dulalia VE, Nillos LT, et al. Pneumococcal conjugate vaccines for preventing vaccine-type invasive pneumococcal disease and X-ray defined pneumonia in children less than two years of age. *The Cochrane database of systematic reviews* 2009; (4): CD004977.
2. Knoll M, O'Brien K, Henkle E, et al. Global literature review of Haemophilus influenzae type b and Streptococcus pneumoniae invasive disease among children less than five years of age, 1980–2005. *Geneva, Switzerland: World Health Organization* 2009.
3. Feikin DR, Nelson CB, Watt JP, Mohsni E, Wenger JD, Levine OS. Rapid assessment tool for Haemophilus influenzae type b disease in developing countries. *Emerging infectious diseases* 2004; **10**(7): 1270-6.
4. Griffin MR, Zhu Y, Moore MR, Whitney CG, Grijalva CG. U.S. hospitalizations for pneumonia after a decade of pneumococcal vaccination. *The New England journal of medicine* 2013; **369**(2): 155-63.
5. Schuck-Paim C, Taylor RJ, Simonsen L, et al. Challenges to estimating vaccine impact using hospitalization data. *Vaccine* 2017; **35**(1): 118-24.
6. Bonten MJ, Huijts SM, Bolkenbaas M, et al. Polysaccharide conjugate vaccine against pneumococcal pneumonia in adults. *The New England journal of medicine* 2015; **372**(12): 1114-25.
7. Pavia M, Bianco A, Nobile CG, Marinelli P, Angelillo IF. Efficacy of pneumococcal vaccination in children younger than 24 months: a meta-analysis. *Pediatrics* 2009; **123**(6): e1103-10.
8. Le Polain De Waroux O, Flasche S, Prieto-Merino D, Goldblatt D, Edmunds WJ. The Efficacy and Duration of Protection of Pneumococcal Conjugate Vaccines Against Nasopharyngeal Carriage: A Meta-regression Model. *The Pediatric infectious disease journal* 2015; **34**(8): 858-64.
9. You D, Hug L, Ejdemyr S, et al. Global, regional, and national levels and trends in under-5 mortality between 1990 and 2015, with scenario-based projections to 2030: a systematic analysis by the UN Inter-agency Group for Child Mortality Estimation. *Lancet* 2015; **386**(10010): 2275-86.
10. Wolfson L, O'Brien K, Watt J, Henkle E, Deloria-Knoll M, McCall N. Methods to estimate the global burden of disease due to Haemophilus influenzae type b and Streptococcus pneumoniae in children less than 5 years of age. *Lancet Web annex* 2009: 893-902.
11. O'Brien KL, Wolfson LJ, Watt JP, et al. Burden of disease caused by Streptococcus pneumoniae in children younger than 5 years: global estimates. *Lancet* 2009; **374**(9693): 893-902.
12. Watt JP, Wolfson LJ, O'Brien KL, et al. Burden of disease caused by Haemophilus influenzae type b in children younger than 5 years: global estimates. *Lancet* 2009; **374**(9693): 903-11.
